# Dissecting Metabolism of Leaf Nodules in *Ardisia crenata* and *Psychotria punctata*

**DOI:** 10.3389/fmolb.2021.683671

**Published:** 2021-07-30

**Authors:** Florian Schindler, Lena Fragner, Johannes B. Herpell, Andreas Berger, Martin Brenner, Sonja Tischler, Anke Bellaire, Jürg Schönenberger, Weimin Li, Xiaoliang Sun, Johann Schinnerl, Lothar Brecker, Wolfram Weckwerth

**Affiliations:** ^1^ Molecular Systems Biology (MOSYS), Department of Functional and Evolutionary Ecology, University of Vienna, Vienna, Austria; ^2^ Vienna Metabolomics Center (VIME), University of Vienna, Vienna, Austria; ^3^ Department of Botany and Biodiversity Research, University of Vienna, Vienna, Austria; ^4^ Department of Pharmaceutical Sciences/Pharmacognosy, Faculty of Life Sciences, University of Vienna, Vienna, Austria; ^5^ Department of Organic Chemistry, University of Vienna, Vienna, Austria

**Keywords:** metabolomics, plant-microbe interaction, nitrogen assimilation, natural products, GC-MS, LC-MS, NMR, SEM

## Abstract

Root-microbe interaction and its specialized root nodule structures and functions are well studied. In contrast, leaf nodules harboring microbial endophytes in special glandular leaf structures have only recently gained increased interest as plant-microbe phyllosphere interactions. Here, we applied a comprehensive metabolomics platform in combination with natural product isolation and characterization to dissect leaf and leaf nodule metabolism and functions in *Ardisia crenata* (Primulaceae) and *Psychotria punctata* (Rubiaceae). The results indicate that abiotic stress resilience plays an important part within the leaf nodule symbiosis of both species. Both species showed metabolic signatures of enhanced nitrogen assimilation/dissimilation pattern and increased polyamine levels in nodules compared to leaf lamina tissue potentially involved in senescence processes and photosynthesis. Multiple links to cytokinin and REDOX-active pathways were found. Our results further demonstrate that secondary metabolite production by endophytes is a key feature of this symbiotic system. Multiple anhydromuropeptides (AhMP) and their derivatives were identified as highly characteristic biomarkers for nodulation within both species. A novel epicatechin derivative was structurally elucidated with NMR and shown to be enriched within the leaf nodules of *A. crenata*. This enrichment within nodulated tissues was also observed for catechin and other flavonoids indicating that flavonoid metabolism may play an important role for leaf nodule symbiosis of *A. crenata.* In contrast, pavettamine was only detected in *P. punctata* and showed no nodule specific enrichment but a developmental effect. Further natural products were detected, including three putative unknown depsipeptide structures in *A. crenata* leaf nodules. The analysis presents a first metabolomics reference data set for the intimate interaction of microbes and plants in leaf nodules, reveals novel metabolic processes of plant-microbe interaction as well as the potential of natural product discovery in these systems.

## Introduction

The leaf nodule symbiosis is an intimate kind of mutualism between plants and bacteria, in which the plants harbor endophytic bacteria in specialized glandular tissue inside their leaves (Horner and Lersten, 1972). Leaf nodule symbiosis has been reported in various species belonging to different genera of the three non-related dicot families Primulaceae subfam. Myrsinoideae (*Amblyanthopsis*, *Amblyanthus*, and *Ardisia*), Rubiaceae (*Pavetta*, *Psychotria*, and *Sericanthe*) and Styracaceae (*Styrax*), as well as in the monocot family Dioscoreaceae (*Dioscorea*). The identified bacterial endophytes belong to the order *Burkholderiales* within the Betaproteobacteria (Yang and Hu, 2018). Certain forms of associations which might present early stages of the development of leaf nodule symbiosis or divergence from early forms of leaf nodule symbiosis, have also been reported (Lemaire et al., 2012; Herpell et al., 2020). Most endophytic bacteria have undergone evolutionary genome reduction as result of this symbiosis to an extend that they are no longer capable of freely living outside their host (Carlier and Eberl, 2012; Carlier et al., 2016). Likewise, most host plants are no longer capable of surviving without their endophytes. When the symbiotic bacteria are experimentally removed, the plants lose vigor and may ultimately die (Yamada, 1955; Gordon, 1963). Because of this codependence of host and symbiont, the bacteria are transmitted vertically between host generations by infecting the developing ovule of the developing host-plant seeds (Pinto-Carbó et al., 2018).

Recent studies point towards production of specialized metabolites as the primary benefit of this symbiosis. Interestingly, it was discovered that certain natural products previously isolated from plants are actually produced by plant-associated microbes. Therefore, mutualistic systems of plants and microorganisms should be thoroughly investigated as source for novel biologically active natural products (Ludwig-Müller, 2015; Atanasov et al., 2021). Great potential can already be seen in the substances FR900359, a cyclic-depsipeptide isolated from the leaf nodulated plant species *Ardisia crenata* Sims and kirkamide, an aminocyclitol isolated from *Psychotria punctata* Vatke, a species better known under its synonym *Psychotria kirkii* Hiern (Sieber et al., 2015; Crüsemann et al., 2018; Lachenaud, 2019; Hermes et al., 2021). *P. punctata* was also found to contain the cardiotoxic substance pavettamine, a symmetrical hydroxylated polyamine, which is suspected to be the causing agent of gousiekte, a fatal poisoning of ruminants (Van Elst et al., 2013a).

Despite the enormous diversity and vast number of unique metabolic capabilities of bacteria only few genera have been studied for their natural products so far. To date, most isolated bioactive and antibiotic compounds from bacterial origin were isolated from environmental (soil, marine) bacteria or bacteria that are commensals or pathogens. Recently, bacteria living in symbioses with insects and nematodes have also been investigated as sources for novel bioactive natural products (Challinor and Bode, 2015). Biosynthetically, the non-ribosomal peptide synthetase (NRPS), polyketide synthase (PKS) and NRPS/PKS hybrid metabolic pathways account for a vast amount of biologically active substances with high chemodiversity (Challinor and Bode, 2015). Putative NRPS and PKS gene clusters have already been found in e.g. *Candidatus* Caballeronia crenata, the symbiont of *A. crenata* (Carlier et al., 2016) and a novel bacterial ectosymbiont *Paraburkholderia* sp. Msb3 from leaf acumens of *Dioscorea bulbifera* (Herpell et al., 2020). Considering the metabolic capabilities of both, plants and bacteria, there is evidence that leaf nodulated species might be a great resource for novel biologically active natural products. Potentially, even substances produced by both symbiotic partners could be discovered. The vast number of antimicrobial and other biologically active compounds produced by bacteria suggests that, by harboring symbiotic endophytic bacteria, plants could get access to their metabolic capabilities. This could lead to an expansion of the plants’ arsenal of defensive agents against pathogens and herbivores providing increased protection and evolutionary fitness.

**FIGURE 1:**
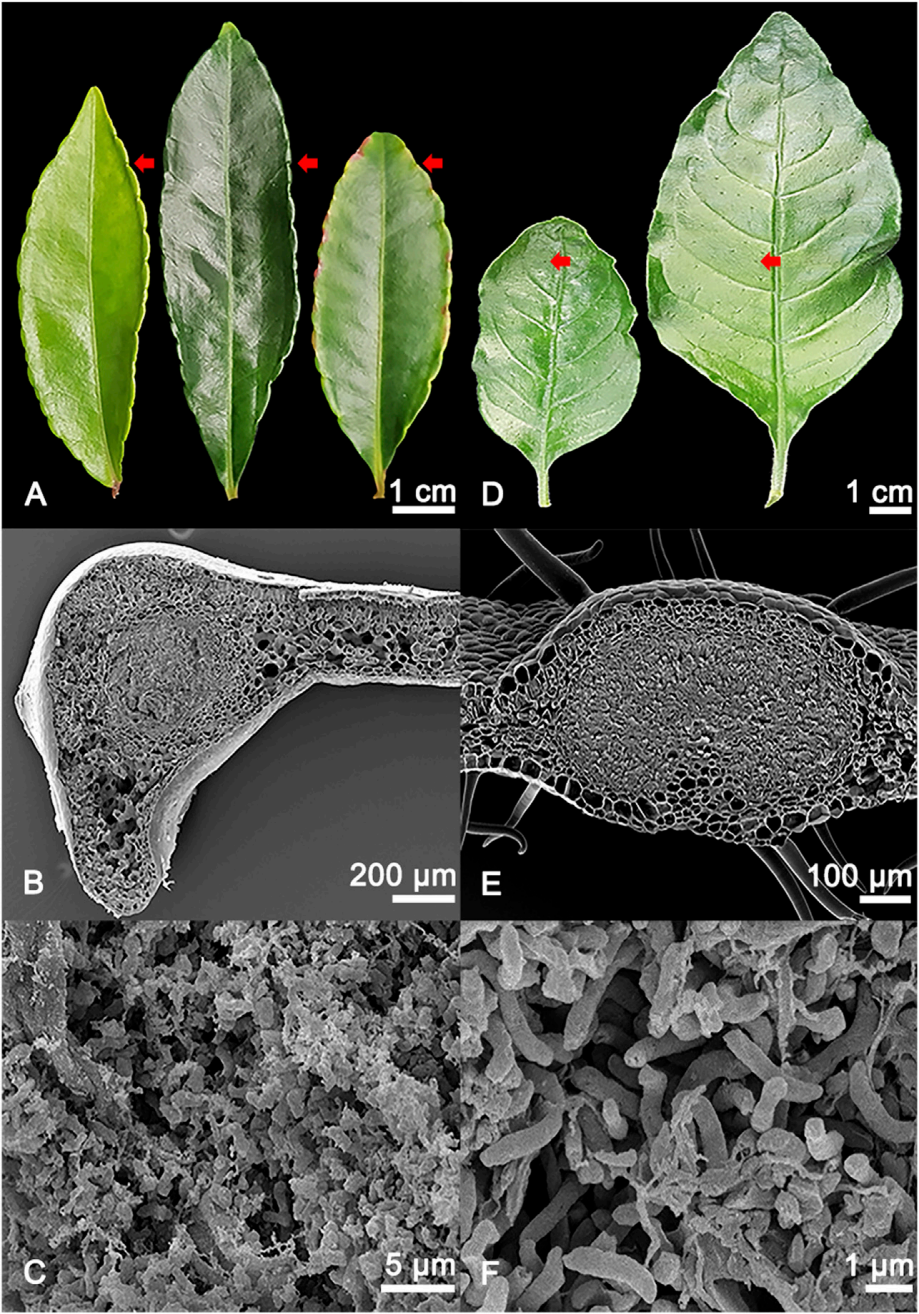
**(A)** Adaxial view of different developmental stages of *Ardisia crenata* (Primulaceae) leaves, from left to right: Stage I (developing leaf), stage II (mature leaf), stage III (oldest mature non-senescent leaf, distinguished by its position as one of the basalmost leaves still attached to the shoot of the plant). The symbiont-harbouring glands can be seen on the leaf margins and are indicated by red arrows. **(B)** SEM image of a cross section of an *A. crenata* leaf nodule, the symbiont harbouring tissue in the centre of the gland is morphologically distinct compared to the leaf parenchyma. **(C)** Magnified view of the individual endophytic bacteria of *A. crenata*. **(D)** Different developmental stages of *Psychotria punctata* (Rubiaceae) leaves, from left to right: Stage I (developing leaf), stage II (mature leaf). The symbiont harbouring glands can be seen distributed over the leaf and are indicated by red arrows. **(E)** SEM image of a cross section of a *P. punctata* leaf nodule, the symbiont harbouring tissue in the centre of the gland is morphologically distinct compared to the leaf parenchyma. **(F)** Magnified view of the individual endophytic bacteria of *P. punctata*.

Here, we investigated the leaf-nodulated species *A. crenata* and *P. punctata* (Figure 1). *A. crenata* is an evergreen shrub native to India, East and Southeast Asia, which is widely cultivated for medicinal and ornamental purposes. The nodules inhabited by its species-specific endophyte *Ca.* C. crenata are restricted to the leaf margin (Kobayashi and De Mejía, 2005; Kitajima et al., 2006; Carlier et al., 2016). The rubiaceous species *P. punctata* is an understory shrub distributed throughout the subtropical regions of continental Africa and Madagascar. Nodules harbouring its species-specific endophyte *Candidatus* Caballeronia kirkii are found dispersed over the leaf lamina (Carlier et al., 2013; Lachenaud, 2019).

To understand the metabolic interaction of bacterial symbionts and the plant host we applied a combined GC-MS/LC-MS metabolomics platform, which we use regularly for phytochemical analysis (Scherling et al., 2010; Doerfler et al., 2013; Mari et al., 2013; Wang et al., 2016; Wang et al., 2017). Using this platform our aim was to 1) detect metabolic differences between leaf nodule and lamina tissue, 2) reveal differences and common traits between the two species as indicators of specific symbiotic mechanisms and 3) investigate the interface of primary and secondary metabolism in leaf nodule and leaf lamina tissue. We also aimed to investigate the metabolic differences of leaf tissues with and without endophytic bacteria at different developmental stages to further reveal the potential of natural product discovery in these systems. Utilizing this metabolomics platform, we provide a first holistic view into the metabolome of leaf nodulated species highlighting specific metabolic features of leaf nodule interaction. We further isolated and structurally elucidated a novel epicatechin derivative potentially involved in plant bacteria interaction and annotated 3 novel putative cyclic-depsipeptides within *A. crenata*.

## Materials and Methods

### Plant Material and Cultivation Conditions

*Ardisia crenata* was obtained from the commercial supplier Bellaflora (Bellaflora Gartencenter, GmbH, Leonding, Austria), while samples from *Psychotria punctata* were obtained from University of Vienna greenhouse (Althanstraße, Vienna, Austria). Voucher specimens were deposited at the Herbarium of the University of Vienna (*Ardisia crenata*: WU 0120874; *Psychotria punctata*: WU 0120875), and collection data and scans are available at the herbarium database system JACQ (http://jacq.org/).

The plants were cultivated in a climate and light controlled greenhouse at the Department of Functional and Evolutionary Ecology at the University of Vienna. In the present study, four different leaf conditions for *P. punctata*, as well as six conditions for *A. crenata* were sampled. The conditions were chosen based on the developmental stage of the leaf and whether the leaf tissue was nodulated or not.

### Sampling and Extraction of Plant Material for Metabolomics Analysis

Five biological replicates were taken for each condition. Additionally, nine blanks starting from solvent extraction of empty tubes were included into the experiment. To account for biological variability and to get enough material from nodulated tissues, samples from four different individuals of the same species were pooled to create one biological replicate, as previously described (Doerfler et al., 2013). The different conditions were developing nodulated leaf tissue and developing non-nodulated leaf tissue, mature nodulated leaf tissue and mature non-nodulated leaf tissue. For the species *A. crenata*, the two additional conditions were oldest mature (non-senescent leaves distinguished from mature by their position as the basalmost leaves still attached to the shoot of the plant) nodulated leaf tissue and oldest mature non-nodulated leaf tissue (Figure 1).

For *A. crenata* the nodules on the leaf margin were harvested as the nodulated leaf tissue (dedicated as nodulated tissue) and the tissue near the primary leaf nerve as the non-nodulated leaf tissue (dedicated as lamina tissue). For the developmental stages, the first fully unfolded leaves were chosen as the developing leaf tissue (stage I). The basalmost non-senescent leaves on the lateral branches were chosen as the mature leaf tissue (stage II) and the lowest non-senescent leaves on the shoots were chosen as the oldest mature leaf tissue (stage III).

For *P. punctata* the nodules on the leaves were harvested as the nodulated leaf tissue by using a glass tube and a section where the nodules had been removed as the non-nodulated leaf tissue. For the developmental stages, the first fully unfolded leaves were chosen as the developing leaf tissue (stage I) and the lowest non-senescent leaves on the lateral branches or main shoots were chosen as the mature leaf tissue (stage II).

Care was taken not to include secondary nerves into the samples of both species. In addition, pooled samples of all conditions were collected for each species to be used in method development and method evaluation. The harvested plant material was put into 2 ml Safe-Lock tubes (Eppendorf®, Vienna, Austria) and immediately flash-frozen in liquid nitrogen and stored at −80°C. Homogenisation was carried out using a Retsch® mill MM 400 (RETSCH GmbH, Haan, Germany). All materials were prechilled in liquid nitrogen and 30–50 mg of frozen plant powder was weighed in 2 ml Safe-Lock tubes (Eppendorf®, Vienna, Austria). The metabolites were extracted using a modified extraction procedure reported by Dettmer et al. (2011). One millilitre ice cold methanol (MeOH) (HPLC grade, VWR, Vienna, Austria) was added to frozen plant powder, the mixture was vortexed and sonicated in an ice bath for 20 min. After centrifugation at 14,800 rpm and 4°C for 4 min using a Fresco™ 21 Microcentrifuge (Thermo Fisher Scientific, Waltham, MA, United States) the supernatant was collected in 2 ml amber glass vials (Agilent Technologies, CA, United States). The extract was dried under a gentle stream of nitrogen 5.0 (≥99.999%, Air Liquide, Vienna, Austria) using a REACTI-VAP III #TS-18826 Evaporation Unit (Thermo Fisher Scientific, Waltham, MA, United States). The remaining cell pellet was re-extracted with 1 ml MeOH following the same procedure and the supernatant was again added to the glass vial. Extraction was carried out four times in total and extracts were dried until weight consistency was achieved. To generate two equal aliquots for GC- and LC-MS analysis, extracts were redissolved in 1 ml MeOH, separated into two 1.5 ml Safe-Lock Eppendorf® tubes and dried in a vacuum concentrator ScanSpeed 40 (SCANVAC, LaboGene, Allerød, Denmark) connected to a CoolSafe SCANVAC and VACUUM PUMP RZ 2.5 vacuubrand® (VACUUBRAND GMBH + CO KG, Wertheim, Germany). Extract weights were determined and samples stored at −20°C until analyses.

### GC-MS Analyses of Metabolites

Internal standards were prepared as follows: stock solutions of 5 mM pentaerythritol (PE) and 50 mM phenyl-β-d-glucopyranoside (PGP) were dissolved in milliQ water and combined 1:1 (v/v) as internal standard mix. Ten microlitres were added to respective sample aliquots for GC-MS analyses and dried in a vacuum concentrator. Dried extracts were derivatized batch-wise directly before analyses by GC-MS. One batch consisted of 14–19 randomly selected samples, one blank and a standard solution of even numbered alkanes at 50 mg/ml in hexane (C_10_–C_40_, Sigma-Aldrich, Vienna, Austria) for determination of retention indices. A mix of metabolite standards was also included in different concentration in order to confirm metabolite identifications, resulting in MSI level I identifications (Supplementary Tables 1, 2) (Goodacre et al., 2007; Sumner et al., 2007). As the dynamic range of analytes within a sample was too high to quantify all peaks in one run, each sample and standard mix were injected in two split rates, 1:5 and 1:50, respectively. Low abundant analytes were quantified in 1:5. Analytes with too high abundances resulting in overloading the column and the detector were only quantified in analyses performed with a higher split rate of injection of 1:50 as indicated in Supplementary Table 2. Derivatization was performed as described earlier (Doerfler et al., 2013) with just slightly changed volumes of reagents. Fourty microlitres of methoxyamine hydrochloride solution in pyridine were used to dissolve the metabolite pellet. Including 80 µl of *N*-methyl-*N*-trimethylsilyl-trifluoroacetamid (Macherey-Nagel, Düren, Germany) the total derivatization volume was 120 µl. GC-MS analyses were performed using an Agilent 6890 gas chromatograph (Agilent Technologies, Santa Clara, United States) coupled to a LECO Pegasus 4D GC × GC-TOF mass spectrometer (LECO Corporation, MI, United States). Materials and instrument settings were used as described earlier (Doerfler et al., 2013; Nägele et al., 2014) with following changes: the Split/Splitless injector was equipped with an Ultra Inert single tapered glass liner with deactivated glass wool (Agilent Technologies Sales & Services GmbH & Co.KG, Waldbronn, Germany) and the temperature was set to 230°C. Oven temperature gradient was used as follows: initial temperature was set to 70°C, held for 1 min, ramped to 330°C and held for 8 min. Data were acquired using an acquisition rate of 20 spectra/second and a detector voltage of 1,625 V. Acquisition delay was set to 260 s.

### LC-MS Analyses of Metabolites

For the RP-HPLC-HRESIMS measurements a method similar to the one previously described by Doerfler et al. (2013) was used. The plant extracts were dissolved in a concentration of 10 mg/l in a solvent consisting of (98% water, 2% acetonitrile (ACN)) + 0.1% formic acid (FA) containing the internal standards ampicillin, chloramphenicol and reserpine each in a concentration of 1 × 10^−5^ mol/l. After the solvent was added the samples were vortexed and sonicated to aid solvation. The samples were centrifuged at 14,800 rpm at 4°C for 10 min and 100 µl of the supernatants were transferred into glass inserts for analysis. The samples were placed into an autosampler at 4°C. Extracts were separated on an Accucore™ Vanquish™ C-18+ UHPLC column (100·2.1 mm; 1.5 µm particle size), equipped with an Accucore™ Defender guards pk4 guard column (150 –C18 10·2.1 mm, 2.5 µm particle size (Thermo Fisher Scientific, Waltham, MA, United States). The mobile phase system consisted of a mixture of solvent A, an aqueous solution of 0.1% FA and solvent B, ACN containing 0.1% FA. A gradient elution method was used for the analysis, 0–5 min 99% A, 5–60 min linear gradient to 1% A, 60–89 min 1% A, 89–110 min linear gradient to 99% A and 110–120 min 99% A. Ten microlitres of sample were injected with a flowrate of 0.1 ml/min and a 25 µl loop was used. The column compartment was kept at 30°C. Between each measurement a column washing step was performed by injecting a solution composed of (99% water, 1% ACN) + 0.1% FA using the following gradient, 0–1 min 99% A, 1–2 min linear gradient to 1% A, 2–47 min 1% A, 47–48 min linear gradient to 99% A and 48–68 min 99% A.

MS-analysis was performed in positive ion mode using an Orbitrap Elite Instrument (Thermo Fisher Scientific, Waltham, MA, United States) with the following parameters: Resolution, 120,000; spray voltage, 3.8 kV; capillary temperature, 350°C; sheath gas, 5; auxiliary gas, 0. The mass scanning range of the MS1 fullscan was set at 100–1,800 *m/z*. Decamethylcyclopentasiloxane was used as lock mass with a *m/z* value of 371.101230. Each MS1 fullscan was followed up by a maximum of 10 data dependent MS2 fragmentation spectra, of the most abundant ion species. The time for dynamic exclusion duration of previously measured analytes was set at 30 s. The collision energy for collision induced dissociation (CID) was set at 35 eV. To reduce bias from measurement conditions, the samples were measured in sets that contained samples from each species and a blank arranged in a randomized order. Before the samples were measured, two measurements, each followed by a column washing step were performed to ensure system equilibrium. A solution composed of (99% water, 1% ACN) + 0.1% FA was injected for all non-sample-type measurements.

The internal standards were measured individually with the same HPLC and MS settings as the samples measured in the experiment, to gain accurate retention times, mass spectra and fragmentation spectra. The absence of internal standards or similar compounds within the original metabolite extracts was confirmed by measuring pooled samples of all tissue types of each species.

For metabolite identification standard compounds were dissolved in a solution consisting of 2% ACN in water with 0.1% FA added. The standards were dissolved at a concentration of 1 mg/ml, if the standards were not completely soluble at this concentration, the solution was centrifuged and the supernatant taken for measurement. The standard solutions were measured with the same HPLC and MS settings as the samples measured in the experiment. The standards were measured individually, to gain accurate retention times, mass spectra and fragmentation spectra.

### Data Processing of GC-MS Data

GC-MS data were processed using the LECO ChromaTOF® software (LECO Corporation, MI, United States) as described earlier (Doerfler et al., 2013) with slight modifications. Peak areas of analytes were divided by extract weight and peak areas of internal standards. For analytes relatively quantified in runs injected with split rate 1:50, areas of phenyl-β-d-glucopyranoside were used and analytes quantified in runs with split rate 1:5 were normalized to pentaerythritol in respective runs. Peak annotation were done according to Metabolomics Standard Initiative (Goodacre et al., 2007; Sumner et al., 2007) and stated in Supplementary Table 2.

### Data Processing of LC-MS Data

Initial untargeted LC-MS data analyses resulted in distinct metabolic profiles of each tissue type tested for each species consisting of about 2,500 *m/z* features detected per species (not shown). The datasets obtained for each species were curated based on *m/z* features that were detected in four out of five replicates in at least one condition.

In order to annotate the unknown *m/z* features all measured spectra were screened for over 800 known metabolites, as well as characteristic unknown *m/z* features. When a *m/z* feature was found within ±5 ppm of the exact mass of a known metabolite, the MS2 spectrum was extracted, if recorded, utilizing the Xcalibur software package (Thermo Fisher Scientific, Waltham, MA, United States) and compared with a suitable reference entry within the databases NIST, MASSBANK and mzCloud if available (Horai et al., 2010; Mikaia et al., 2014; HighChem, 2019). Additionally, we isolated and structurally elucidated three natural products as described in section Natural Product Isolation and NMR analysis. The isolated natural products were measured by LC-MS and results were used to confirm the identity of respective annotations.

The *m/z* feature was annotated as the corresponding metabolite when the MS2 spectrum matched with the database reference spectrum. For unknown *m/z* features or *m/z* features without a suitable MS2 reference spectrum a manual annotation of their corresponding MS2 spectra were performed. The MS1, MS2 and reference spectra of the annotated metabolites were then summarized in Supplementary Table 3. The manual annotation of fragmentation spectra of *m/z* features is provided in Supplementary Data Sheet 1. The level of identification was determined for each LC-MS *m/z* feature according to Metabolomics Standard Initiative (Goodacre et al., 2007; Sumner et al., 2007) and stated in Table 1.

**TABLE 1:** Table of annotated *m/z* features detected within leaf extracts of nodulated and lamina tissues of *Ardisia crenata* and *Psychotria punctata* measured by LC-MS/MS.

Name	RT [min]	[M+H]^+^ [*m/z*]	Exact mass [*m/z*]	Mass accuracy [ppm]	Sum formula	MS/MS [*m/z*]	Annotation level
Epigallocatechin	14.70	307.0811	307.0812	−0.3	C_15_H_14_O_7_	139, 151, 169, 181	2
Catechin^a^ ^,^ ^b^	17.81	291.0864	291.0863	0.3	C_15_H_14_O_6_	123, 139, 151, 165	1
Epicatechin^a^ ^,^ ^b^	19.47	291.0863	291.0863	0.0	C_15_H_14_O_6_	123, 139, 151, 165	1
Epicatechin 3'*O*-3-hydroxy-2-methyl-propanoate^b^	22.23	377.1227	377.1230	-0.8	C_19_H_20_O_8_	123, 139, 165, 273	1
Epigallocatechin gallate^a^	19.75	459.0915	459.0922	−1.5	C_22_H_18_O_11_	139, 151, 289, 307	1
Quercetin	20.24	303.0500	303.0499	0.3	C_15_H_10_O_7_	165, 229, 247, 257	2
Isoquercetin	20.20	465.1023	465.1028	−1.1	C_21_H_20_O_12_	303	2
Quercetin-dirhamnopyranoside-hexoside	20.20	757.2178	757.2186	−1.1	C_33_H_40_O_20_	303, 449, 465, 611	2
Quercetin	20.72	303.0501	303.0499	0.7	C_15_H_10_O_7_	165, 229, 247, 257	2
Isoquercetin	20.71	465.1028	465.1028	0.0	C_21_H_20_O_12_	303	2
Quercetin rha hex pent	20.71	743.2021	743.2029	−1.1	C_32_H_38_O_20_	303, 435, 449, 465, 597, 611	2
Kaempferol	21.11	287.0549	287.0550	−0.3	C_15_H_10_O_6_	165, 213, 231, 241, 269	2
Kaempferol-*O*-glucoside	21.11	449.1081	449.1078	0.7	C_21_H_20_O_11_	287	2
Kaempferol-*O*-rutinoside	21.02	595.1643	595.1658	−2.5	C_27_H_30_O_15_	287, 449	2
Robinin or isomer	21.11	741.2236	741.2237	−0.1	C_33_H_40_O_19_	287, 433, 449, 595	2
Kaempferol-*O*-glucoside	21.65	449.1074	449.1078	−0.9	C_21_H_20_O_11_	287	2
Kaempferol-*O*-rutinoside	21.65	595.1645	595.1658	−2.2	C_27_H_30_O_15_	287, 449	2
Kaempferol dihexoside	22.01	611.1608	611.1607	0.2	C_27_H_30_O_16_	287, 449	2
Quercetin	22.08	303.0493	303.0499	−2.0	C_15_H_10_O_7_	165, 229, 247, 257	2
Rutin	22.08	611.1599	611.1607	−1.3	C_27_H_30_O_16_	303, 465	2
Kaempferol-*O*-glucoside	22.84	449.1072	449.1078	−1.3	C_21_H_20_O_11_	287	2
Kaempferol-*O*-rutinoside	22.84	595.1644	595.1658	−2.4	C_27_H_30_O_15_	287, 449	2
Kaempferol-*O*-glucoside	23.82	449.1074	449.1078	−0.9	C_21_H_20_O_11_	287	2
Caffeoylquinic acid	15.97	355.1014	355.1024	−2.8	C_16_H_18_O_9_	163	2
Caffeoylquinic acid^a^	17.67	355.1022	355.1024	−0.6	C_16_H_18_O_9_	163	1
Caffeoylquinic acid	18.34	355.1013	355.1024	−3.1	C_16_H_18_O_9_	163	2
Ascorbic acid^a^	3.03	177.0390	177.0394	−2.3	C_6_H_8_O_6_	95, 121, 129, 141, 149	1
Dehydroascorbic acid hydrate	4.05	193.0342	193.0343	−0.5	C_6_H_8_O_7_	95, 147, 157, 165	4
Dehydroascorbic acid	4.05	175.0237	175.0237	0.0	C_6_H_6_O_6_	129, 139, 147	3
GSH^a^	3.62	308.0908	308.0911	−1.0	C_10_H_17_N_3_O_6_S	144, 162, 179, 215, 233	1
GSSG^a^	5.78	613.1585	613.1592	−1.1	C_20_H_32_N_6_O_12_S_2_	355, 409, 466, 484, 538	1
Ophthalmic acid	4.05	290.1345	290.1347	−0.7	C_11_H_19_N_3_O_6_	161, 197, 215, 227	2
Glutamic acid^a^	2.38	148.0603	148.0604	−0.7	C_5_H_9_NO_4_	84, 102	1
Pyroglutamic acid	2.33	130.0495	130.0499	−3.1	C_5_H_7_NO_3_	84	2
Pyroglutamic acid	4.16	130.0499	130.0499	0.0	C_5_H_7_NO_3_	84	2
Asparagine^a^	2.34	133.0609	133.0608	0.8	C_4_H_8_N_2_O_3_	87, 97	1
Proline^a^	2.47	116.0702	116.0706	−3.4	C_5_H_9_NO_2_	70	1
Methionine^a^	3.43	150.0583	150.0583	0.0	C_5_H_11_NO_2_S	104	1
Trimethyllysine	2.18	189.1595	189.1598	−1.6	C_9_H_21_N_2_O_2_^+^	60, 84, 130	2
Phenylalanine^a^	11.36	166.0859	166.0863	−2.4	C_9_H_11_NO_2_	120, 131	1
Tyrosine^a^	5.43	182.0809	182.0812	−1.6	C_9_H_11_NO_3_	136, 147	1
Tryptophan^a^	16.29	205.0971	205.0972	−0.5	C_11_H_12_N_2_O_2_	146, 160, 170	1
Kynurenin^a^	11.23	209.0919	209.0921	−1.0	C_10_H_12_N_2_O_3_	94, 150, 174	1
Cholin	2.34	104.1068	104.1070	−1.9	C_5_H_14_NO^+^	60	2
Creatine	2.64	132.0770	132.0768	1.5	C_4_H_9_N_3_O_2_	90	2
*N*-Acetylserotonin	20.76	219.1125	219.1128	−1.4	C_12_H_14_N_2_O_2_	160	2
Spermidine^a^	2.24	146.1652	146.1652	0.0	C_7_H_19_N_3_	72, 75	1
Pavettamine	2.03	252.1915	252.1918	−1.2	C_10_H_25_N_3_O_4_	82, 100, 118, 135, 210, 217	2
Pantothenic acid^a^	14.18	220.1178	220.1180	−0.9	C_9_H_17_NO_5_	90, 116, 131, 174, 184	1
NAD^+^ ^a^	3.89	664.1150	664.1164	−2.1	C_21_H_27_N_7_O_14_P_2_	232, 348, 428, 524, 542	1
Nicotinate d-ribonucleoside	2.74	256.0810	256.0816	−2.3	C_11_H_14_NO_6_^+^	124	2
Riboflavin-5'-phosphate	17.84	457.1113	457.1119	−1.3	C_17_H_21_N_4_O_9_P	243, 359, 421, 439	2
Leucopterin	4.48	196.0463	196.0465	−1.0	C_6_H_5_N_5_O_3_	140, 168	2
Adenosine	8.09	268.1035	268.1040	−1.9	C_10_H_13_N_5_O_4_	136	2
Adenosine monophosphate^a^	3.30	348.0696	348.0704	−2.3	C_10_H_14_N_5_O_7_P	136	1
Guanosine	9.92	284.0992	284.0989	1.1	C_10_H_13_N_5_O_5_	152	2
Guanosine monophosphate	3.43	364.0656	364.0653	0.8	C_10_H_14_N_5_O_8_P	NA	4
Cyclic adenosine monophosphate	4.90	330.0598	330.0598	0.0	C_10_H_12_N_5_O_6_P	NA	4
Cyclic ADP Ribose	4.07	542.0666	542.0680	−2.6	C_15_H_21_N_5_O_13_P_2_	232, 428	2
*S*-methyl-5'-thioadenosine	15.96	298.0973	298.0968	1.7	C_11_H_15_N_5_O_3_S	136, 145, 163	2
*S*-methyl-5'-thioadenosine	3.14	298.0964	298.0968	−1.3	C_11_H_15_N_5_O_3_S	136, 145, 163	2
Zeatin glucoside	14.96	382.1703	382.1721	−4.7	C_16_H_23_N_5_O_6_	220	2
Disaccharide^a^	2.63	343.1234	343.1235	−0.3	C_12_H_22_O_11_	145, 163, 181	1
*N*-Acetylmuramic acid	3.95	294.1185	294.1183	0.7	C_11_H_19_NO_8_	186	2
*N*-Acetylmuramic acid	6.49	294.1179	294.1183	−1.4	C_11_H_19_NO_8_	186	2
Putative aminosugar	13.96	647.2878	647.2883	−0.7	C_26_H_42_N_6_O_13_	190, 319, 329, 462	3
Putative aminosugar	14.31	648.2719	648.2723	−0.6	C_26_H_41_N_5_O_14_	191, 320, 329, 462	2
Putative aminosugar	14.38	850.3661	850.3676	−1.8	C_34_H_55_N_7_O_18_	319, 462, 532, 647, 661	3
Putative aminosugar	14.47	851.3517	851.3517	0.1	C_34_H_54_N_6_O_19_	320, 463, 532, 648, 661	3
Putative aminosugar	15.24	689.2983	689.2988	−0.8	C_28_H_44_N_6_O_14_	232, 258, 329, 361, 458, 504	3
Putative aminosugar	15.31	719.3095	719.3094	0.1	C_29_H_46_N_6_O_15_	262, 329, 391, 458, 534, 630	3
Putative aminosugar	15.31	922.3883	922.3888	−0.5	C_37_H_59_N_7_O_20_	262, 391, 534, 701, 719, 833	2
Putative aminosugar	15.56	892.3788	892.3782	0.7	C_36_H_57_NO_19_	262, 361, 504, 532, 671, 689	3
Putative aminosugar	15.74	790.3464	790.3465	−0.1	C_32_H_51_N_7_O_16_	333, 462, 605, 630, 701	3
Putative aminosugar	15.83	993.4261	993.4259	0.2	C_40_H_64_N_8_O_21_	333, 462, 532, 605, 790, 833	3
Putative aminosugar	16.28	680.2769	NA	NA	NA	291, 458, 476	3
Putative aminosugar	16.29	476.1871	476.1875	−0.8	C_19_H_29_N_3_O_11_	258, 291, 329	3
Putative aminosugar	16.36	679.2664	679.2669	−0.7	C_27_H_42_N_4_O_16_	291,458, 476, 532	3
FR900359	48.38	1,002.5403	1,002.5394	0.9	C_49_H_75_N_7_O_15_	385, 456, 603, 799, 974	2
AC-1	44.25	1,032.5525	1,032.5500	2.4	C_50_H_77_N_7_O_16_	415, 486, 633, 829, 1,004	2
AC-SC	39.74	817.4335	817.4342	−0.9	C_40_H_60_N_6_O_12_	385, 456, 603, 688, 789	2
Putative cyclic-depsipeptide	31.76	1,123.5748	NA	NA	NA	385, 456, 506, 662, 773, 976	4
Putative cyclic-depsipeptide	35.12	1,123.5715	NA	NA	NA	385, 456, 506, 920, 1,002, 1,087	4
Putative cyclic-depsipeptide	37.36	1,123.5723	NA	NA	NA	385, 456, 506, 789, 931, 1,002	4
Putative cyclic-depsipeptide	42.33	988.5260	988.5237	2.3	C_48_H_73_N_7_O_15_	385, 456, 589, 674, 785, 960	2
Putative cyclic-depsipeptide	45.46	988.5248	988.5237	1.1	C_48_H_73_N_7_O_15_	385, 456, 603, 688, 799, 960	2

a Confirmed with corresponding standard.

b Confirmed by isolation and structure elucidation.

rha, deoxy-hexose; pent, pentose; hex, hexose; GSH, glutathione; GSSG, glutathione disulfide; NAD^+^, nicotinamide adenine dinucleotide; ADP, adenosine diphosphate. The m/z features denoted as putative aminosugar correspond to anhydromuropeptides and their derivates. Annotation levels were labelled according to the metabolite identification standard published by the metabolomics society (Goodacre et al., 2007; Sumner et al., 2007). Level 1: the compound is identified with at least two independent parameters compared with an authentic compound. Level 2: the candidate was putatively annotated based on spectral similarity with public or commercial spectral libraries. Level 3: the candidate was assigned to a compound class based on spectral similarity to a known compound of a chemical class. Level 4: unknown compounds quantified based on spectral data but without further identification and classification.

The annotated *m/z* features were then integrated using Xcalibur Quanbrowser utilizing the following parameters: Exact mass and retention time of the annotated *m/z* feature, Mass tolerance: 15 ppm, Decimals: 4, Expected time: 600 s, Smoothing points: 3, Baseline window: 40, Area noise factor: 5 Peak noise factor: 10, ICIS Peak Detection: Highest Peak, Minimum peak height (S/N): 3.0. Since the mass accuracy of some *m/z* features in some of the measurements was higher than 5 ppm, the mass accuracy during peak integration was set at ±15 ppm. This was done in order to not lose parts of some peaks that albeit not having a mass accuracy within ±5 ppm were consistent in RT and MS2 spectra with the *m/z* features that were originally selected by having a mass accuracy within ±5 ppm. The integration borders of the peaks were corrected manually if necessary.

### Natural Product Isolation

For natural product isolation an adapted method described by Seidel (2012) was used. Nodulated *A. crenata* leaf margins (123 g) were cut from fresh leaves and immediately preserved in liquid nitrogen. The frozen plant material was homogenized and extracted with 1,000 ml MeOH (2 × 2 days) in darkness and then filtered. The extract was dried under vacuum to yield 10.21 g of crude extract. The dried crude extract was suspended in water and then subsequently extracted with petroleum ether (PE; 1.9 g), chloroform (CHCl_3_; 10 mg), ethyl acetate (EtOAc; 300 mg), 1-butanol (1-BuOH, 2.23 g) and water (5.22 g). Crude extract and obtained fractions were measured by LC-MS as described above prior to further separation. The CHCl_3_, EtOAc, and 1-BuOH-phases were pooled for further separation by silica gel column chromatography. The silica gel was equilibrated in a mixture of EtOAc/PE 50/50. Prior to elution, 0.8 g of the combined extracts were adsorbed on 2.0 g silica gel and loaded onto the column. Then the column was stepwise eluted with mixtures of PE/EtOAc/MeOH: 300 ml 50/50/0 followed by 100 ml each of 40/60/0, 30/70/0, 20/80/0, 10/90/0, 0/100/0, 0/90/10, 0/80/20, 0/70/30, and 0/60/40. The collected fractions were combined based on their thin layer chromatography (TLC) profiles using silica gel coated plates and EtOAc/MeOH 9/1 as mobile phase. The fractions eluting with PE/EtOAc 20/80 were combined yielding 17.8 mg. Further separation by size exclusion chromatography was performed following the method described by Buathong et al. (2019). The fraction was separated by Sephadex LH-20 with MeOH as eluent to yield 1.4 mg of (**1**), and 7.7 mg of a mixture of (**2**) and (**3**) in proportions of 13:10.

The purity of the fractions was determined by LC-MS as described above and HPLC-UV-Vis analyses as follows: On an Agilent 1100 series with photodiode array detector (Agilent Technologies, Inc., Santa Clara, CA, United States) the wavelength of detection was set at 230 nm (reference wavelength 360 nm). A Hypersil BDS C-18 column (250 4.6 mm; 5 µm particle size) (Thermo Fisher Scientific, Waltham, MA, United States) was used as the stationary phase. The mobile phase system consisted of a mixture of solvent A, an aqueous solution containing 10 mM ammonium acetate and solvent B being MeOH. A gradient elution method was used for the analysis, 0 min 90% A, 0 to 15 min linear gradient to 0% A, 15 to 22 min 0% A. Ten microlitres of sample were injected with a flowrate of 1.0 ml/min.

### NMR Analysis

Structure elucidation of the isolated compounds was performed via 1D and 2D NMR analysis according to Berger et al. (2017). For the NMR measurements ∼2.0 mg of isolated analyte was dissolved in 0.7 ml CD_3_OD and transferred into 5 mm high precision NMR sample tubes. The ^1^H, ^13^C and 2D spectra were recorded on an AVANCE III 600 NMR Spectrometer 600.13 MHz (^1^H), 150.61 MHz (^13^C) (Bruker Corporation, Billerica, MA, United States) and performed using Topspin 3.1 software (Bruker Corporation, Billerica, MA, United States). The measurement temperature was set at 298 ± 0.05 K. Residual CD_2_HOD was used as internal standard for ^1^H (δH 3.34) and CD_3_OD for ^13^C (δC 49.0) measurements. The NMR data can be found in Supplementary Table 4 NMR Spectroscopic data.

### Combination of GC- and LC-MS Data and Statistical Analyses

The data matrices obtained by manual *m/z* feature annotation were reduced by only considering *m/z* features that were detected in at least four out of five sample replicates in at least one condition per species for further analyses. Missing values were substituted by half of the minimum value over the whole dataset, this was done separately for the GC-MS and LC-MS data and individually for each species. The GC-MS and LC-MS data were subsequently combined. Data were scaled by normalizing each *m/z* feature to the percentage of the highest value of said *m/z* feature. Statistical analyses were carried out using Excel (Microsoft Corporation, Redmond, WA, United States) and R. The following R packages were used: car, factoextra, ggfortify, ggplot2, gplots, multcompView, plotly (Fox et al., 2012; Graves et al., 2015; Tang et al., 2016; Warnes et al., 2016; Wickham et al., 2016; Kassambara and Mundt, 2017; Galili et al., 2018).

One-way ANOVA was used to determine statistically significant differences in the datasets. The validity of applying the one-way ANOVA was determined by applying Levene- and Shapiro tests to the datasets (Levene, 1961; Shapiro and Wilk, 1965). Although, the requirements for equal variances and normal distribution seemed mostly fulfilled by the datasets a Kruskal-Wallis test was also employed on the datasets to compare the results with the one-way ANOVA (Kruskal and Wallis, 1952). The resulting significant *m/z* features of both tests were compared. The overlap of statistically significant *m/z* features were 85.8% for the *A. crenata*- and 87.6% for the *P. punctata* dataset. Based on studies by Vinaixa et al. (2012) these results were deemed sufficient to use the one-way ANOVA approach for further analyses.

The statistically significant *m/z* features obtained by the one-way ANOVA were visualized as heatmaps employing hierarchical bicluster analyses. (Supplementary Images 1, 2) In order to find statistically significant differences for each *m/z* feature between the tissues a Tukey's range test (within a 95% confidence interval) was used on the statistically significant *m/z* features (Tukey, 1949). Additionally, boxplots of each *m/z* feature were created with the results of the Tukey’s range test included (Supplementary Data Sheets 2, 3).

A principal component analysis (PCA) (Lopes-Santos et al., 2017) was also performed on the datasets. The data used contained all annotated metabolites, including those not found to be statistically significant. For PCA analysis the datasets were centered and scaled. The eigenvalues of the principal components were plotted in Scree plots (Cattell, 1966). In order to determine whether the differences between groups were statistically significant, the scores for each PCA were extracted and normalized to 100%. A one-way ANOVA was performed on the normalized principal components, followed by a Tukey's range test (within a 95% confidence interval) to determine the differences between the tissues.

### Chlorophyll Fluorescence Measurements

Chlorophyll Fluorescence Measurements of *P. punctata* leaves were performed with IMAGING-PAM M-Series (mini/B2) Chlorophyll Fluorometer (Heinz Walz, Germany).

### Scanning Electron Microscopy

Fresh leaf cross sections containing leaf nodules were fixed in FAA (40% formaldehyde (Merck KGaA, Darmstadt, Germany), acetic acid (Fisher Scientific (Austria), Vienna, Austria), 70% ethanol (AustrAlco, Spillern, Austria) 5v/5v/90v) and washed in 70% ethanol. After dehydration in a series of ethanol (85%, 96%) and acetone (Sigma-Aldrich Handels Gmbh, Vienna, Austria), the samples were critical point dried, mounted on aluminium stubs and sputter coated with gold using a sputter coater SCD 050. The samples were observed with a scanning electron microscope JSM-IT300 (JEOL, Freising, Germany) under high vacuum with 10.0 kV. Photos were taken with a magnification of ×85 and ×4,000.

## Results

### Metabolic Signature of Leaf Nodules is Age Dependent and Species Specific

**FIGURE 2:**
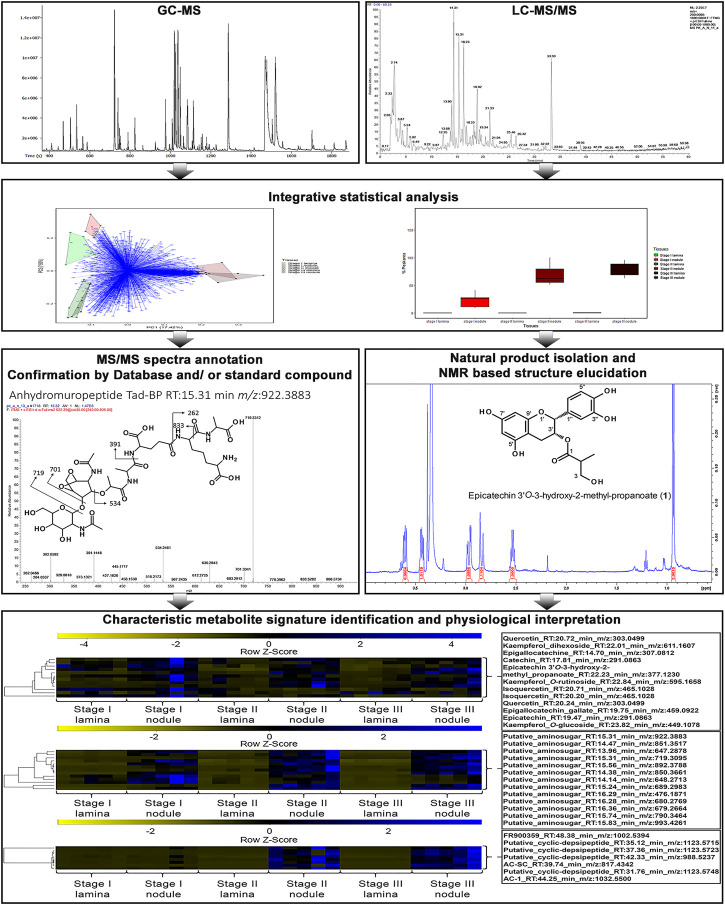
Overview for the workflow utilizing a combined GC-MS and LC-MS platform in addition with natural product isolation and characterisation to dissect the leaf and leaf nodule metabolism and function in the nodulated species *Ardisia crenata* and *Psychotria punctata*.

To investigate the differences in metabolic signatures of leaf tissue and nodulated (leaf) tissue we applied a combined GC-MS and LC-MS platform (Figure 2). Both platforms cover complementary parts of the metabolism, namely, primary and secondary metabolites, respectively (Weckwerth, 2011; Doerfler et al., 2013; Mari et al., 2013; Wang et al., 2016a). Through an untargeted GC-MS analysis we were able detect 203 distinguishable compounds that were manually curated (Supplementary Table 2) and through an untargeted LC-MS analysis we extended this dataset by more than 2,500 *m/z* features per species. After *m/z* feature selection and annotation the combined LC-MS and GC-MS datasets contained 276 and 255 metabolites for *A. crenata* and *P. punctata*, respectively. Of these *m/z* features 193 (57.1%) were detected in both species. These *m/z* features belonged to a wide variety of compound classes, here categorized as follows: amino acids, organic acids, carbohydrates, phenolics, polyamines, flavonoids, nucleotides, peptides and cofactors. The curated LC-MS *m/z* features are listed within (Supplementary Table 3).

**FIGURE 3:**
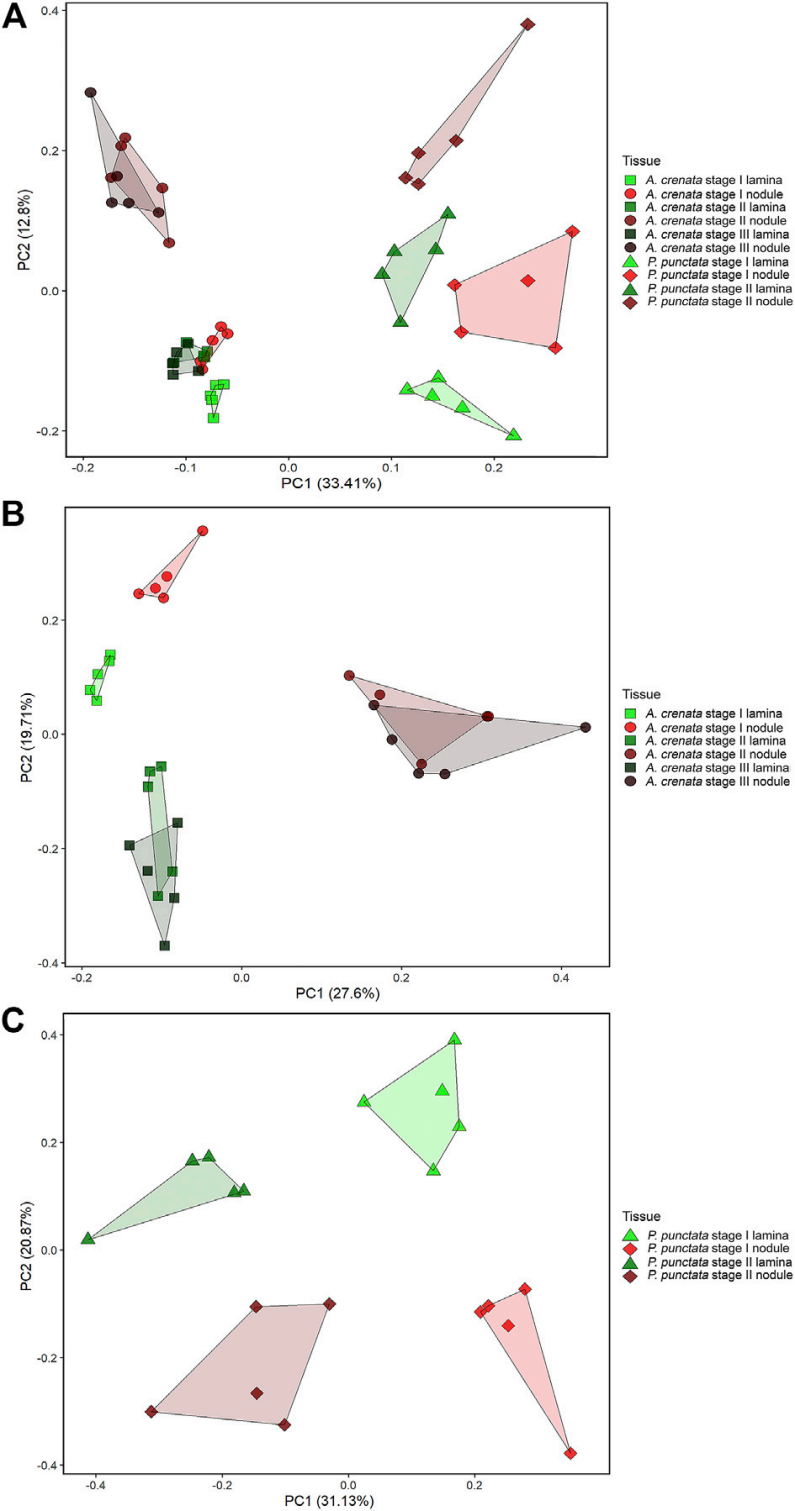
**(A)** PCA analysis of 338 centered and scaled *m/z* features from LC-MS and GC-MS analysis of *Ardisia crenata* and *Psychotria punctata* lamina tissue and nodule extracts. **(B)** PCA analysis of 276 centered and scaled *m/z* features from LC-MS and GC-MS analysis of *Ardisia crenata* lamina tissue and nodule extracts. **(C)** PCA analysis of 255 centered and scaled *m/z* features from LC-MS and GC-MS analysis of *Psychotria punctata* lamina tissue and nodule extracts.

To explore the relationship between the samples we first performed a principal component analysis (PCA) on the combined LC-MS and GC-MS datasets. Within the PCA, species and tissue specific separations can be observed (Figure 3). According to leaf maturity the samples were subdivided into two (*P. punctata*) or three (*A. crenata*) age-stages, ranging from developing (stage I) to mature (stage II) and oldest mature (stage III) leaves (Figure 1). All tissues have a unique and distinguishable metabolic composition, except for the stage II tissues from *A. crenata,* which largely overlap with the respective stage III tissues.

In individual PCA plots for each species (Figures 3B,C) separations can be observed more clearly. In *A. crenata* (Figure 3B) a significant separation between stage II and stage III nodulated tissues and all other tissue types occurred along principal component (PC) 1. On PC2 the lamina samples cluster according to their age. Stage I nodulated tissue samples align with laminar samples on PC1 but also separate from these along PC2.

For *P. punctata* (Figure 3C) a very similar pattern to *A. crenata* can be observed. Separation by age can be seen on PC1 and nodulated and lamina tissues separate along PC2.

**FIGURE 4:**
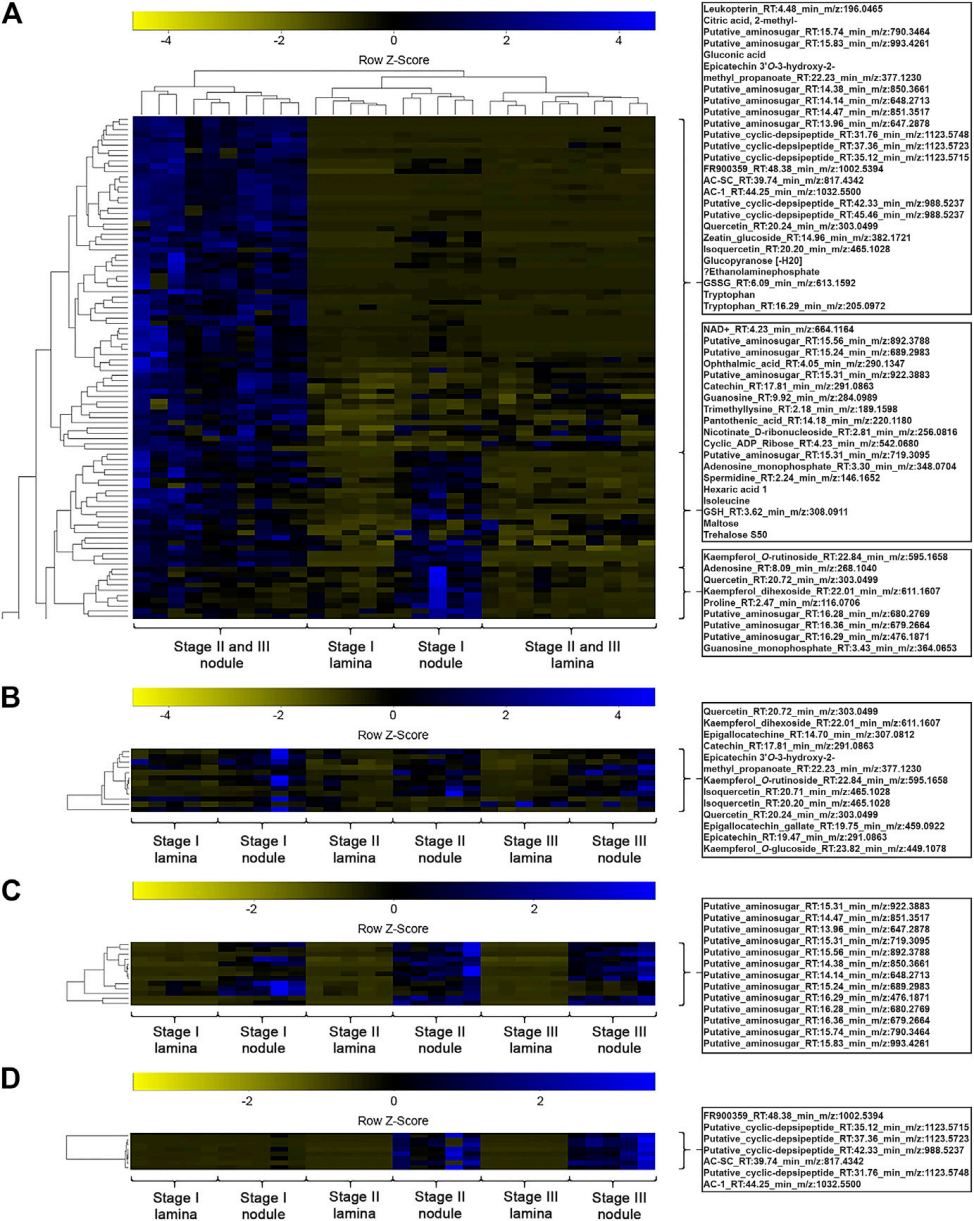
**(A)** Heatmap of a hierarchical bicluster analysis of statistically significant changed nodule specific metabolites of *Ardisia crenata* tissue extracts (selected metabolite names do not correspond with rows). The columns correspond to the five biological replicates of each condition. **(B)** Heatmap of a hierarchical cluster analysis of metabolites annotated as flavonoids of *A. crenata* tissue extracts. The columns correspond to the five biological replicates of each condition. **(C)** Heatmap of a hierarchical cluster analysis of metabolites annotated as anhydromuropeptdides of *A. crenata* tissue extracts. The columns correspond to the five biological replicates of each condition. **(D)** Heatmap of a hierarchical cluster analysis of metabolites annotated as cyclic-depsipeptides of *A. crenata* tissue extracts. The columns correspond to the five biological replicates of each condition.

**FIGURE 5:**
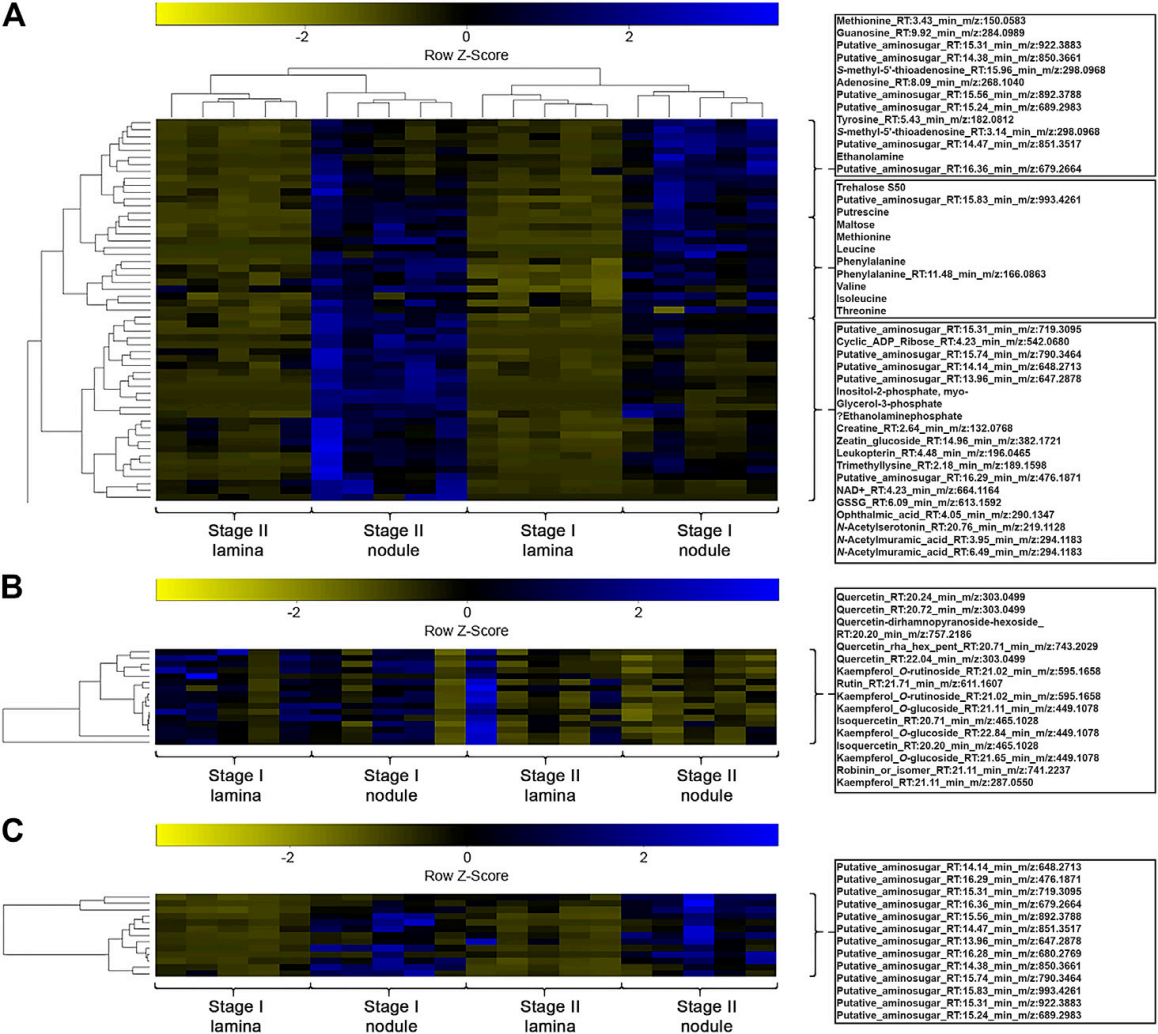
**(A)** Heatmap of a hierarchical bicluster analysis of statistically significant changed nodule specific metabolites of *Psychotria punctata* tissue extracts (selected metabolite names do not corresponds with rows). The columns correspond to the five biological replicates of each condition. **(B)** Heatmap of a hierarchical cluster analysis of *m/z* features annotated as flavonoids of *P. punctata* tissue extracts. The columns correspond to the five biological replicates of each condition. **(C)** Heatmap of a hierarchical cluster analysis of *m/z* features annotated as anhydromuropeptdides of *P. punctata* tissue extracts. The columns correspond to the five biological replicates of each condition.

A one-way ANOVA was performed on both datasets to dissect statistically significant changes between relative abundances of all detected *m/z* features (Figures 4, 5). It resulted in 198 (71.7% out of 276 initial *m/z* features) and 167 (65.5% out of 255 initial *m/z* features) statistically significant *m/z* features for *A. crenata* and *P. punctata*, respectively. These *m/z features* were further subjected to hierarchical bicluster and hierarchical cluster analyses and the results were visualized in the form of heatmaps (Figures 4, 5). The results of the hierarchical bicluster analysis show a similar pattern to the PCAs: Biological replicates of the same tissue cluster together and different tissue types are well distinguishable. Again, the exceptions are stage III tissue samples from *A. crenata* that are very similar to their respective younger stages (stage II). The PCA and hierarchical bicluster analysis suggest that in both species initial differences within and between nodulated and lamina tissues gradually increase with age. For *A. crenata* this effect subsides at a certain tissue maturity.

In the following we analyze the common metabolic signatures of leaf nodulation as well as species- and age-specific differences in these datasets according to their biological relevance, compound class and pathway contributions.

### Anhydromuropeptides are Characteristic for Leaf Nodule Tissue but Compounds Assigned Putative Symbiotic Functions are Species Specific

**FIGURE 6:**
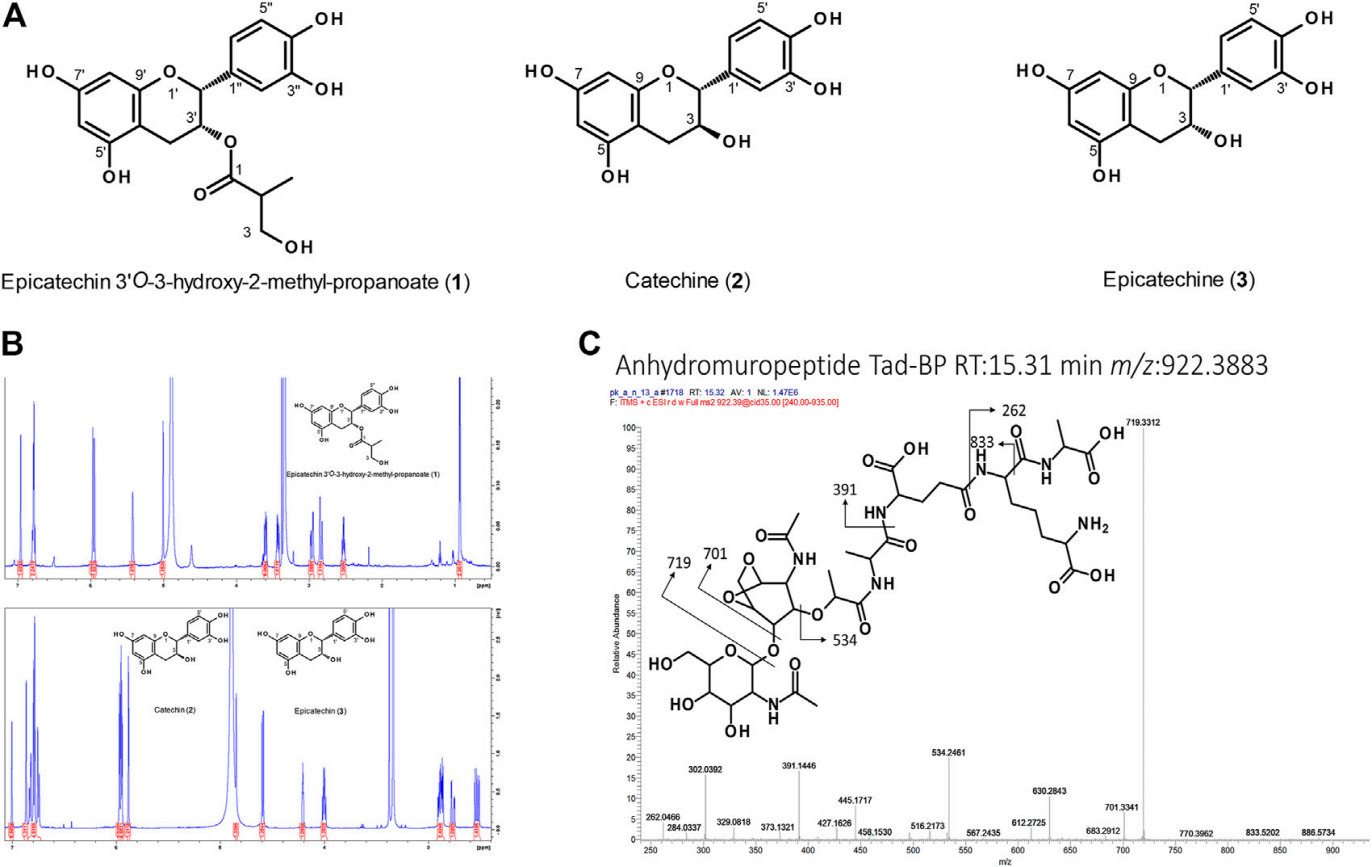
**(A)** Structures of flavan-3-ol derivatives isolated from nodulated *Ardisia crenata* leaf tissues. **(B)** NMR analysis of these flavan-3-ol derivatives. **(C)** Annotated ESI-MS2 spectrum (pos. mode, CID fragmentation) of anhydromuropeptide with a precursor *m/z* 922.39 in LC-MS analyses of *Psychotria punctata* leaf nodule sample.

We explored the datasets with respect to common nodule specific features: 13 *m/z* features, initially annotated as putative aminosugars, were detected in samples from both species and displayed higher relative abundances in nodulated tissues (Figures 4C, 5C). These metabolites ranged from 476 to 993 *m/z* and based on their MS2 fragmentation patterns (Packiam et al., 2015; Lee et al., 2016) we identified them as anhydromuropeptides (AhMPs) (Figure 6C). Within both species these putative AhMPs are highly characteristic for nodulated tissues (Figures 4, 5). We detected a broad variety of these compounds ranging from native AhMP to diaminopimelic acid amidated and *N*-acetylated diaminopimelic acid amide AhMPs. Interestingly, a distinct change in AhMP composition within the tissues, by age, was observed in both species. Three AhMPs accumulated with progressing age in both species (putative aminosugars with *m/z* values 647, 648, and 790) (Figures 4C, 5C). Differences in relative AhMP abundance between lamina and nodulated tissues are higher in *A. crenata*, this effect could be related to bacterial activity (see *Discussion*).

**FIGURE 7:**
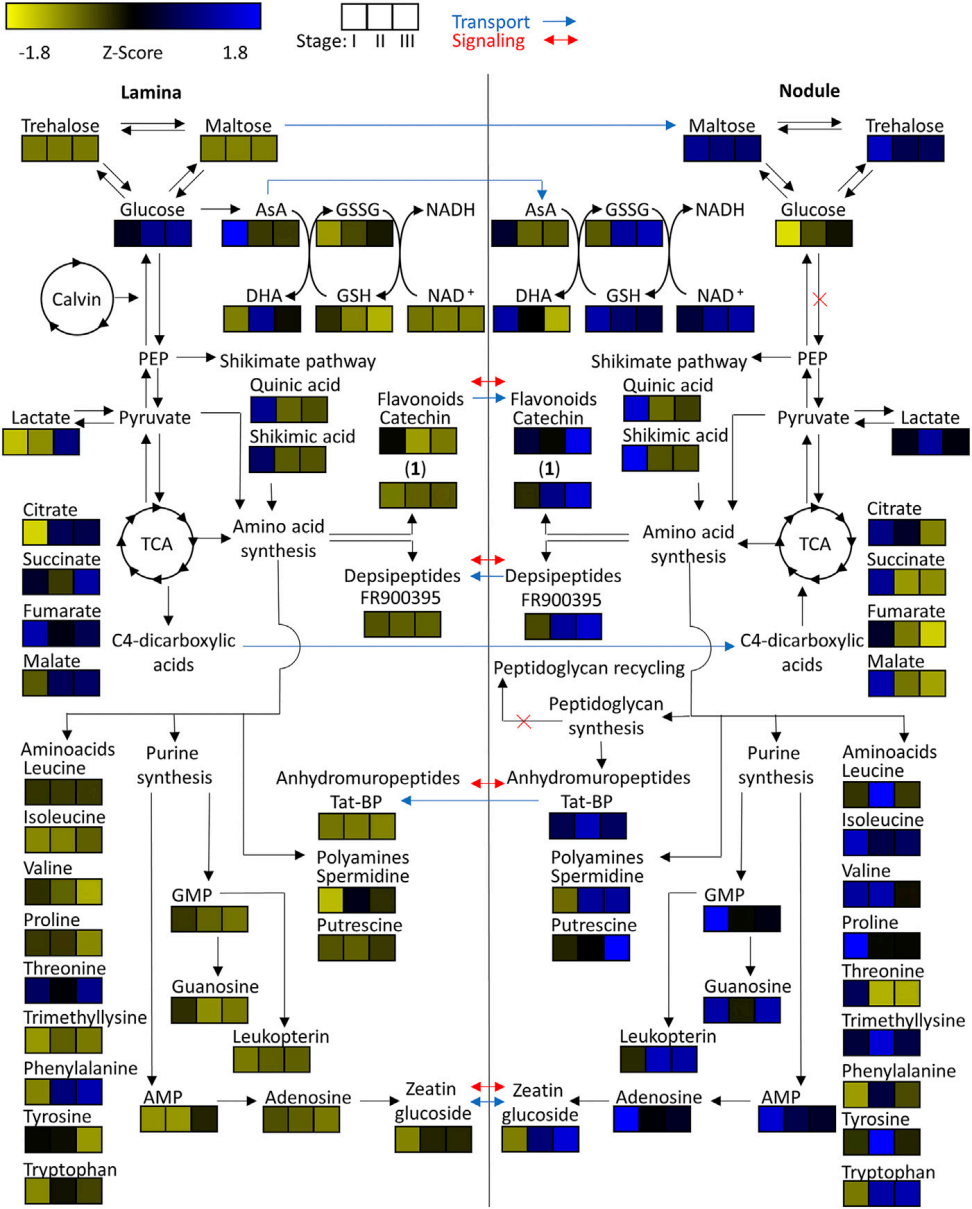
Heatmap of z-scored means of selected *m/z* features of *Ardisia crenata* tissue extracts illustrated within their connected metabolic pathways. Possible metabolite transport is denoted by a blue arrow and possible signaling is denoted by a red arrow.

With respect to species-specific compounds we identified putative cyclic-depsipeptides related to FR900359 based on their MS2 fragmentation patterns (Reher et al., 2018) (Figure 4D). This biologically active compound is produced by the *A. crenata* endophyte *Candidatus* Caballeronia crenata (Schrage et al., 2015; Carlier et al., 2016; Crüsemann et al., 2018). These peptides were only detected within samples of *A. crenata* and were generally more abundant in nodulated tissue and their abundance there strongly increased with progressing age (Figures 4D, 7).

**FIGURE 8:**
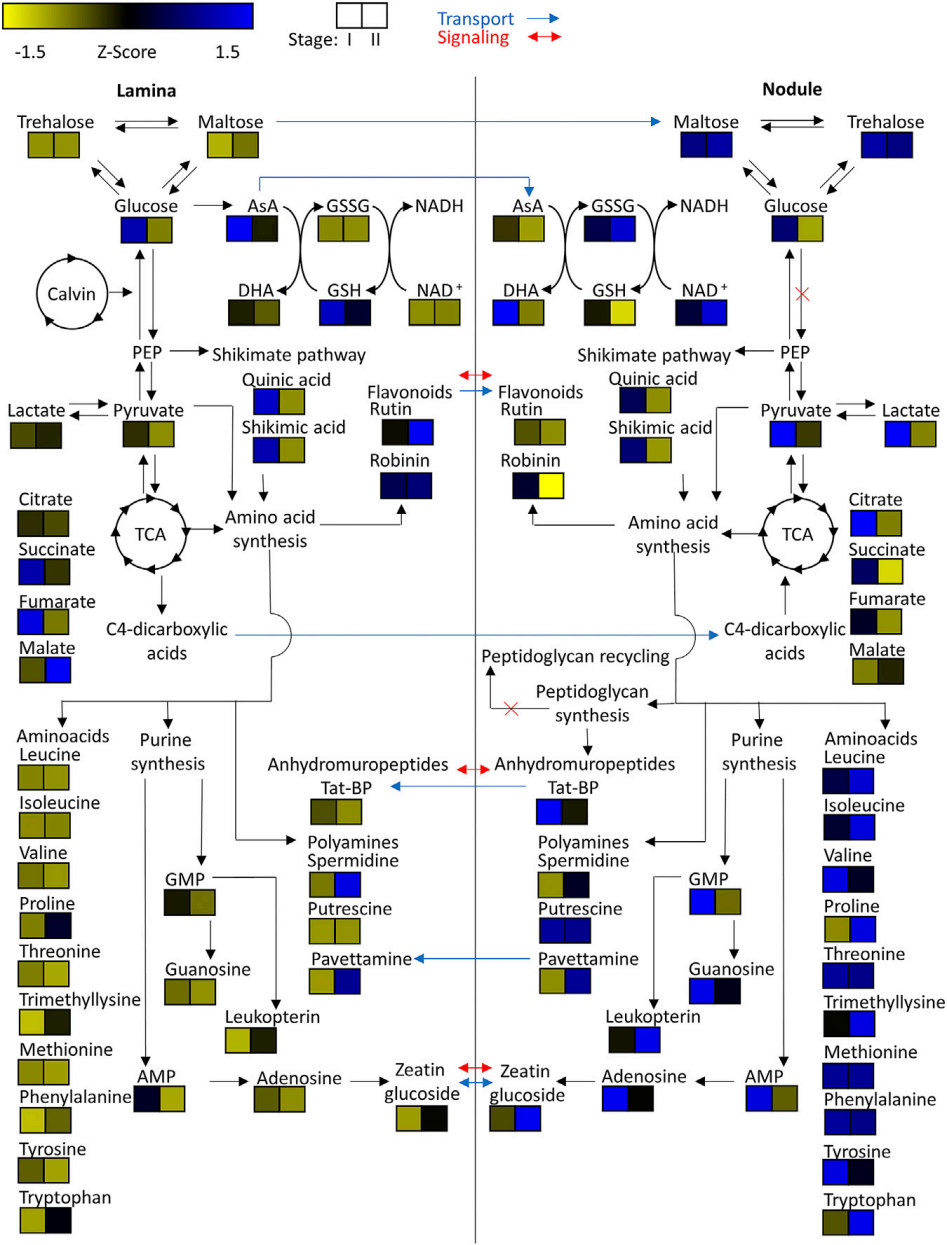
Heatmap of z-scored means of selected *m/z* features of *Psychotria punctata* tissue extracts illustrated within their connected metabolic pathways. Possible metabolite transport is denoted by a blue arrow and possible signaling is denoted by a red arrow.

Similarly, pavettamine, also annotated based on its MS2 fragmentation pattern (Bode et al., 2010), could only be detected in samples originating from *P. punctata* (Figure 8; Supplementary Image 2). Pavettamine is a polyamine and causative agent of Gousiekte, a disease of ruminants, and has been brought into connection with leaf endophytes in *Rubiaceae* (Verstraete et al., 2011; Van Elst et al., 2013b; Brader et al., 2014). It was, however, not nodule-specific and increased with age in both tissue types. Despite our efforts to detect kirkamide, a C_7_N aminocyclitol from the leaf nodule symbiont of *P. punctata* (formerly *P. kirkii*) (Sieber et al., 2015), we were not able to identify it with certainty.

### Flavonoid Signatures Are Highly Species Specific but Only Nodule Associated in *A. crenata*

Because of their diverse functions in regulating plant development and their possible roles in defense and signaling between plants and microorganisms (Mathesius, 2018) we directed our efforts at investigating and identifying flavonoids in the symbiotic plants. We observed drastically different flavonoid compositions in both plants (Calixto et al., 2016; Wong et al., 2020) as well as variations between tissue types.

In *A. crenata* we were able to identify several flavonoids that belong to both flavonols and flavan-3-ols. Catechin, epigallocatechin, and isoquercetin were all enriched in nodulated tissues (Figure 4A). Two kaempferol derivates, kaempferol-dihexoside and kaempferol-*O*-rutinoside, were also nodule specific, although they appear to only be enriched in the younger (stage I and some stage II) nodulated tissues.

We also found an unknown flavan-3-ol that was consistently differentially abundant throughout the *A. crenata* dataset with elevated relative levels in colonized tissues of all age stages (Figures 4A,B). The compound was purified and, along with other flavonoids, identified via NMR and HRMS (Figure 6). In total, three natural products were obtained from nodulated leaf tissues of *A. crenata* (Figures 6A,B). The structure of the novel flavan-3-ol was elucidated as epicatechin 3'*O*-3-hydroxy-2-methyl-propanoate (henceforth labeled as (**1**) in Figures 2, 4, 6, see also Supplementary Table 4 NMR Spectroscopic data) and the other two were found to be catechin (**2**) and epicatechin (**3**) (Figures 6A,B). For clarity, the epicatechin moiety of (**1**) and (**3**) are illustrated as (-)-epicatechin and (**2**) is illustrated as (+)-catechin, however their absolute configuration was not determined within this study (Figures 6A,B). Spectroscopic data are consistent with data reported for catechin and epicatechin described in the literature (Davis et al., 1996; El-Razek, 2007). The cis conformation of (**1**) can be inferred through the small coupling constant between the protons of carbon positions 2 and 3 (Figure 6A). This could be caused by the near 90° dihedral angle between the protons, which occurs in the cis conformation of the molecule and, following the Karplus equation, should yield a ^3^ *J*_H,H_ of near 0. In contrast, the trans conformation of (**2**) yields a higher ^3^ *J*_H,H_ (Karplus, 1959), a result of the dihedral angle of these protons being near 180°. For epicatechin 3'*O*-3-hydroxy-2-methyl-propanoate (**1**) the conformation at the stereocenter at 2'' could not be resolved. All necessary spectral information can be found within Supplementary Table 4 NMR Spectroscopic data. In *P. punctata* the detected flavonoid levels were similar in stage I tissues, irrespective of the tissue type. Flavonoid abundance generally decreased with age in nodulated tissues. We only identified flavonols within this species. Flavan-3-ols including the novel epicatechin 3'*O*-3-hydroxy-2-methyl-propanoate (**1**) isolated from *A. crenata* were not detected in *P. punctata* (for further information see Discussion).

### Citric Acid Accumulates in Developing Nodules and Trehalose and Maltose are Age-independent Nodule Markers

To investigate central carbohydrate and energy metabolisms we took a look at various common carbon substrates. Increased relative abundances of trehalose and maltose were observed in nodulated tissues of both plant species (Figures 7, 8). The relative abundances of glucose, fructose, galactose, and sucrose on the other hand are higher within the lamina tissue types of both species throughout their development.

Tricarboxylic acid (TCA) cycle related organic acids are mostly characteristic for young developing tissues. Their relative abundance is generally higher within the laminar tissues. There are, however, some exceptions. In both species the highest citric acid levels were detected within the still developing nodulated tissues (Figures 7, 8). In *A. crenata* this pattern was also observed for malate and succinate (Figure 7). Furthermore, 2-methylcitric acid was also found to be highly characteristic for nodulated *A. crenata* tissues, with increasing relative abundance by age (Figure 4).

### Amino Acid and Polyamines Are Abundant in Both Plants’ Nodule Tissues

As obligate leaf symbionts have been reported to carry largely eroded genomes and, as a consequence, are likely deficient in the biosynthesis of various amino acids (AAs) (Carlier and Eberl., 2012; Carlier et al., 2016) we examined their respective distributions across our dataset. At first glance the amino acid and polyamine signatures in both species showed a clear pattern: most of the detected amino acids and polyamines accumulated in nodulated tissues. Especially the branched chain amino acids (BCAA) leucine, isoleucine and valine were highly characteristic for nodulated tissues of both species (Figures 7, 8). Proline and trimethyllysine (a betaine) were also generally abundant in nodule tissue.

There were, however, some subtle differences between the two leaf nodulated species that are worth mentioning: Threonine occurred at elevated levels only within the lamina tissues of *A. crenata* and, similarly, phenylalanine was more abundant in laminar samples in this species than in its nodules. Generally, AAs were more abundant in nodular than in laminar tissue with a very clear pattern in *P. punctata* (Figure 8) and a more complex tendency in *A. crenata* (Figure 7).

Naturally, AAs could function as nitrogen as well as carbon sources for obligate endophytes (Moe, 2013). Polyamines could constitute another form of nitrogen source but could also act as signal or regulatory molecules. Putrescine, for example, was highly characteristic for colonized tissues (Figures 7, 8). In *A. crenata* that signature strongly increased with maturity. Additionally, spermidine levels within mature (stage II and III) nodulated tissues in *A. crenata* (Figure 7) were also elevated (see also *Discussion*).

### Nucleotides, Cofactors and Cytokinin Phytohormone Derivative are Common Nodule Markers

Adenosine, adenosine monophosphate, guanosine, and guanosine monophosphate were detected in tissues of both species. The relative abundances of these metabolites were higher within the nodulated tissues and decreased with age (Figures 7, 8). Cyclic adenosine monophosphate, which can be linked to bacterial signaling (Gomelsky, 2011), was only found within tissues of *P. punctata*. The highest relative abundance of this metabolite was found within the nodulated tissues. NAD^+^, nicotinate-d-ribonucleoside, leucopterin and riboflavin-5'-phosphate were detected in tissues of both species (Figures 4, 5, 7, 8). These metabolites were all present at elevated levels within the nodulated tissues and their relative abundances increased with age. The levels of these nucleotide derived cofactors are inversely correlated to the ones we observed for their biosynthetic precursors.

The only phytohormone derivative found in both species was annotated as zeatin glucoside, a cytokinin derivative. Its abundance increased with age and it was generally more abundant within colonized tissues (Figures 7, 8).

## Discussion

The PCAs (Figure 3) suggest that in both species initial differences within and between nodulated and lamina tissues gradually increase with age. In *A. crenata* this effect subsides at a certain age until a metabolic equilibrium is reached. We also demonstrate that there is a vast difference between metabolite composition of matured nodulated and lamina tissues. Although, both species show a very similar pattern in the trajectories of the developmental stages the differences between tissues are stronger in *A. crenata* (Figure 3B) compared to *P. punctata* (Figure 3C).

Within the hierarchical bicluster analyses (Figures 4, 5) of statistically significant *m/z* features a similar pattern was observed. The findings further highlight the divergence of metabolic composition of different tissues through increasing tissue maturity. Additionally, we clearly illustrate that bacteria that colonize leaf galls profoundly alter the overall metabolic signature of the host plant in individual ways. This will likely hold true for phylogenetically diverse host species and underpins the individuality and diversity of functions of different leaf nodule symbioses (Lemaire et al., 2011; Carlier and Eberl, 2012; Sieber et al., 2015; Carlier et al., 2016; Pinto-Carbo et al., 2018; De Meyer et al., 2019).

### Metabolites Assigned Putative Symbiotic Functions

#### Developmental Metabolic Gradients From Leaf to Nodule Tissue

Our findings suggest that the full metabolic capacity of the studied endophytes is only achieved once the colonies within the leaf nodules have fully matured. The overlap of stage II and stage III tissues in *A. crenata* (Figure 3B) can be explained by a metabolic equilibrium that is maintained within the matured leaves until the onset of senescence.

Similar patterns (Figures 3A–C) in both species investigated suggest that leaf nodule development follows a similar mechanism. The stronger differences between matured nodulated tissues and lamina in *A. crenata* compared to *P. punctata* (Figure 3A) could be explained by a later onset of full metabolic activity in *A. crenata*. Additionally, the production of cyclic-depsipeptides and nodule specific flavonoids only detected in *A. crenata* contribute to the strong separation.

#### Novel Putative Cyclic-Depsipeptides Indicate High Biologically Active Compound Diversity in *A. crenata* Leaf Nodules

Within the nodulated tissues of *A. crenata* 8 *m/z* features were annotated as cyclic-depsipeptides (cDPs). These *m/z* features were highly characteristic for stage II and stage III nodulated tissues (Figure 4D). Three of those have already been characterized and another two have been putatively described as cyclic-depsipeptides (Reher et al., 2018). Here, we managed to identify three putatively novel cyclic-depsipeptides. The increase in relative abundance from stage II to stage III (Figures 4D, 7) suggests that the production of these specialized metabolites is still ongoing while some of the endophytic bacteria are already degrading.

No such *m/z* features were detected within samples of *P. punctata,* which is in line with no such observations in *Ca*. C. kirkii (Carlier and Eberl, 2012; Carlier et al., 2013). This could suggest that the common ancestor of both endophytes had the capabilities for production of these non-ribosomal peptides. Subsequently, the descendants of the lineage that later engaged in symbiosis with *Psychotria* lost it before or during the symbiosis. Alternatively, the descendants of the lineage that later engaged in symbiosis with *Ardisia* acquired the means of producing these compounds before reaching the obligate form of symbiosis. While the first hypothesis remains speculative, the later can be inferred by the detection of FR900359 within other nodulated *Ardisia* species (Reher et al., 2018). MS3 spectra (Supplementary Data Sheet 1) suggest that at least two of the three novel putative cyclic-depsipeptides are direct derivatives of FR900359. These findings indicate that there might be even more substrate and/or substrate sequence variability within the non-ribosomal peptide synthethases (NRPS) of the *A. crenata* endophyte. Alternatively, thus far unknown pathways may exist that mediate further modification of the non-ribosomal peptide after its synthesis.

#### Pavettamine–Produced by Bacteria or the Plant Host?

The presence of pavettamine generated our interest. It was found to be highly abundant in the mature tissues of *P. punctata* (Figure 8) and may likely serve as an herbivore defense agent. It is the causative agent of Gousiekte, a disease of ruminants. The compound has been brought into connection with leaf endophytes in Rubiaceae several times (Verstraete et al., 2011; Van Elst et al., 2013b; Brader et al., 2014) and it has been hypothesized that it is, at least partially, produced by the endophytes (Verstraete et al., 2011; Van Elst et al., 2013b). However, no production of pavettamine under *in vitro* conditions could be observed by a culturable endosymbiont of a related Rubiaceae species (Van Elst et al., 2013b). Evidence of production of other hydroxylated polyamines has been reported for Betaproteobacteria, the same order as the endophytes (Busse and Auling, 1988). Here we show that pavettamine production is strongly dependent on the developmental stage. Our results further illustrate that pavettamine production is highly species-specific. Van Elst et al. (2013a) reported the highest pavettamine concentration within the youngest leaves of a related Rubiaceae species. Our results highlight an inverse signature within *P. punctata*. Pavettamine likely diffuses through the leaf lamina or might even be transported. Considering the reports of Busse and Auling (1988), Van Elst et al. (2013a), Van Elst et al. (2013b) we suggest that pavettamine is produced by the nodulating bacteria in association with the host plant.

## Metabolites Involved in Host-Microbe-Homeostasis

### Maltose May be Central to Carbohydrate Storage in Endophytes

The low relative abundances within the lamina tissues of trehalose and maltose (Figures 7, 8) could either be due to high transport rates of carbohydrates into the nodulated tissues, or because of synthesis within the nodules. Genomic analysis from *Ca.* C. crenata and proteomic analysis of *Ca.* C. kirkii both report an alpha-alpha-trehalose-phosphate synthase and trehalose-6-phosphate phosphatase (Carlier et al., 2013; Carlier et al., 2016). The latter also found putative glucose/maltose and trehalose transporters. These findings suggest that the endophytic bacteria are capable of trehalose synthesis. Trehalose has been shown to be important in bacterial stress responses. It can stabilize protein structures through its osmoregulatory function (Ruhal et al., 2013). This coincides with the proposed high oxidative stress encountered by the endophytes within the leaf (Bringel and Couée, 2015).

Since proteomic analysis of *Ca.* C. kirkii (Carlier et al., 2013) found no evidence for a functioning class II fructose bisphosphate aldolase, preventing functional glycolysis, the increased relative abundances of maltose could suggest that it is utilized within the pentose phosphate pathway as an energy source. To be utilized it would need to be converted into glucose by action of a 4-alpha-glucanotransferase. Maltose could also act as a direct precursor for trehalose synthesis, which requires the action of a trehalose synthase. Evidence for both a 4-alpha-glucanotransferase and trehalose synthase were found within proteomic analysis of *Ca.* C. kirkii (Carlier et al., 2013). Maltose could further act as a carbohydrate storage for times of famine (Jones et al., 2008). Our results highlight that trehalose and maltose both act as key components of the endophytes carbohydrate metabolism. Evidence for maltodextrin synthesis pathways were found (Carlier et al., 2013), which suggests that these sugars serve as important carbohydrate storage.

Elevated levels of glucose (Figures 7, 8), fructose, galactose, and sucrose (data not shown) within the lamina tissues are likely a product of carbon assimilation during photosynthesis by the host plants (Halford et al., 2011).

### Variable Organic Acid Distributions Indicate Differential Regulation of Tricarboxylic Acid Energy Metabolism

Organic dicarboxylic acids have been proposed as the major energy source provided by the host plant to the bacterial endophytes (Carlier et al., 2013). Therefore, we investigated their tissue-dependent distribution carefully. Metabolic signatures of TCA intermediates not only highlight the elevated TCA activity of still developing tissues (Figures 7, 8) but also suggest that C_4_-dicarboxylic acids may indeed act as a major carbon source for *Ca.* C. crenata (Figure 7), at least during leaf growth and development. Unfortunately, information regarding relative abundances of TCA cycle intermediates within bacteria remains sparse. We therefore cannot conclude whether the observed pattern is evidence that citric acid is provided to the endophytes or if this is just indicative of high TCA cycle activity. Altogether, it is not clear which dicarboxylic acid acts as carbon source for the endophytes, therefore this needs to be further investigated.

### Leucine Supply to Endophytes May Constitute Nitrogen Source but May Also Exert Plant-Controlled Population Constraint

It has been reported that BCAAs are essential for the symbiosis between plant and bacteria in legumes (Prell et al., 2009). Endophytes within root nodules become auxotrophic to BCAAs. This mechanism allows the plant to control the development and persistence of the symbiotic bacteria by provision of these metabolites. Recent evidence suggests that the here studied leaf nodule symbionts developed an auxotrophy for leucine (Carlier et al., 2013; Carlier et al., 2016). Interestingly, leucine and other BCAAs are quite abundant in colonized tissues (Figures 7, 8). We propose that leucine is supplied to the endophytic bacteria by the plant and subsequently imported by action of a putative LIV-I BCAA transport system, which is encoded by genes BKIR_c26_5785 to BKIR_c26_5788 (Carlier and Eberl, 2012; Carlier et al., 2013). Consequently, a similar control mechanism, as found within root nodules of legumes, may also be employed to control bacterial populations in leaf nodule symbioses.

The observed amino acid compositions also illustrated that other amino acids accumulate in nodule tissue (Figures 4A, 5A, 7, 8). Whether this accumulation stems from *de novo* synthesis in the bacteria or whether AAs other than leucine are being imported needs further investigation. Evidence, however, suggests, that the bacteria are independent of nitrogen sources other than leucine, proline (*Ca*. C. crenata) (Carlier et al., 2016), methionine (*Ca*. C. kirkii) (Carlier and Eberl, 2012; Carlier et al., 2013) and ammonia. Typically, altered nitrogen assimilation processes result in highly significant metabolic signatures with decreased central metabolites such as sugars and organic acids and increased levels of nitrogen rich amino acids such as asparagine, glutamine, polyamines, but also glutamate, urea and others (Scherling et al., 2009; Scherling et al., 2010). In a previous study we analyzed metabolomic changes due to the interaction of a bacterial poplar endophyte with the plant in sterilized plant cuttings (Scherling et al., 2009) revealing a highly specific metabolic signature of the plant-microbe interaction pointing to an altered nitrogen metabolism. Results in the present studied leaf nodule interaction suggest drastically altered nitrogen assimilation/dissimilation processes in the nodules compared to the lamina tissue (Figures 7, 8). The origin of these metabolic signatures is not entirely clear and will be subject to further investigations.

### Reactive Oxygen Species Detoxification and Osmotic Stress Tolerance Pathways are Heavily Affected by Leaf Nodulation

Given the hostile environment for the bacteria within the leaf (Triantaphylides et al., 2008) an effective detoxification mechanism regarding reactive oxygen species (ROS) seems to be of utmost importance to the bacteria (Alquéres et al., 2013). We observed high amounts of oxidized glutathione (GSSG) and ophthalmic acid within the nodulated tissues relative to the non-nodulated lamina tissues in all developmental stages (Figures 7, 8). This indicates that the bacteria actually do experience oxidative stress (Farr and Kogoma, 1991; Narainsamy et al., 2016).

The relative abundances of ascorbic acid (AsA) and dehydroascorbic acid (DHA) as key components of the ascorbate-glutathione cycle support these findings (Noctor and Foyer, 1998). Especially within the young tissues ascorbic acid levels were high within the laminar tissues but depleted within the nodulated tissues. The observed pattern was inverted for DHA. This indicates high turnover of GSSG to glutathione (GSH) via the oxidation of AsA to DHA.

Enrichment of putrescine and spermidine within nodulated tissues of both species (Figures 7, 8) may further contribute toward oxidative stress resistance (Tkachenko et al., 2012).

NAD^+^ was found in higher amounts within the nodulated tissues compared to the lamina tissues. Given the involvement of NAD^+^ in many different metabolic pathways this can however not be fully attributed to effects based on oxidative stress alone (Belenky et al., 2007).

This metabolic pattern is mostly consistent between *A. crenata* and *P. punctata*. There are, however, some noteworthy differences. Whereas in *A. crenata* GSH levels are higher within the nodulated tissues compared to the lamina tissues (Figure 7), in *P. punctata* this pattern is reversed (Figure 8). There is also a dramatic difference when comparing the GSH/GSSG ratios of nodulated tissues between the two species. While in *A. crenata* GSH levels are 1–2 orders of magnitude higher the ratio is around 1 in *P. punctata*. Altogether, this could indicate that the amount of oxidative stress experienced by endophytes of *P. punctata* might be higher compared to the endophytes of *A. crenata.* Genomic analysis from *Ca.* C. crenata and proteomic analysis of *Ca.* C. kirkii report both a GSH synthetase and Glu synthase, as well as a glutamate-cystein ligase (Carlier et al., 2013; Carlier et al., 2016). This suggests that GSH, at least in part, is produced by the endophytic bacteria.

### Endophytes Delay Senescence of Surrounding Tissue—Self- or Host-Interest?

**FIGURE 9:**
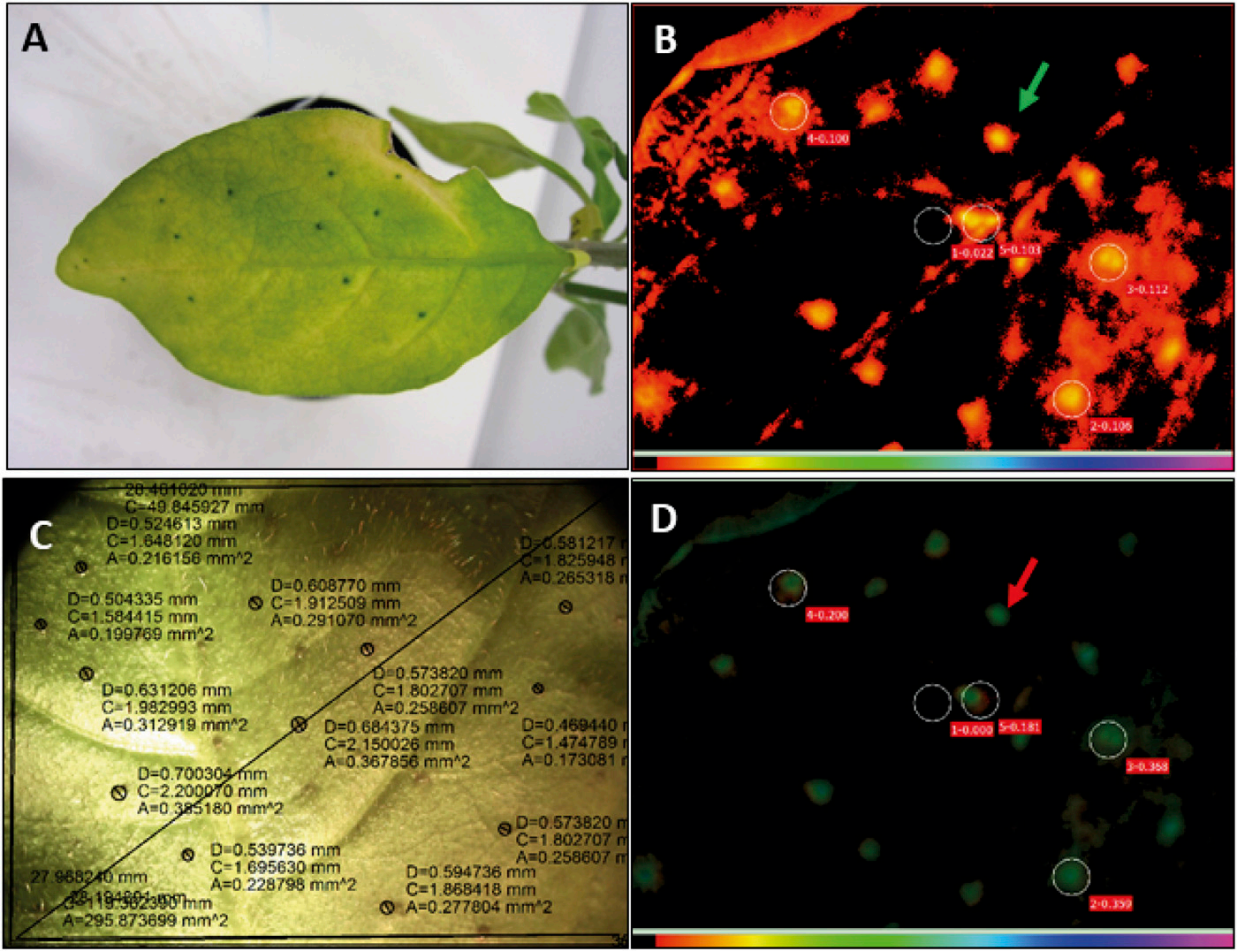
Senescent leaves of *Psychotria punctata* show green nodules and higher photosynthetic activity in nodulated areas as compared to areas of leaf lamina. **(A)** Senescent leaf of *P. punctata*, showing still green nodules. **(B)** Visualisation of the chlorophyll fluorescence parameters of a senescent leaf of *P. punctata* analysed with an Imaging PAM mini fluorometer (Walz, Germany), maximal fluorescence in light adapted state (Fm’); red colour code indicates lower levels of fluorescence light in nodulated areas (marked by green arrow) and thus higher photosynthesis rates; circels indicate the area, where the light curves have been measured. Circle number 1 indicates non nodulated leaf area, numbers 2–5 nodulated leaf areas. **(C)** Picture of the corresponding senescent leaf of *P. punctata*. Black circles surround leaf nodules, D, diameter of leaf nodule; C, circumference; A, area. **(D)** Quantum yield of photosystem II during illumination (Y(II)); green colour code indicates higher quantum yield in nodulated leaf areas (red arrow).

While auxins could not be detected, the cytokinin derivative zeatin glucoside was found at higher relative abundance in nodulated tissues of both plant species (Figures 7, 8). The role of cytokinins like zeatin has been well documented. They can function as major regulators of plant tissue development. They are involved in processes like meristem formation and maintenance as well as leaf senescence (Gan and Amasino, 1995; Su et al., 2011). In our study we also detected adenosine and adenosine monophosphate with increased relative abundances in the nodulated tissues of both species (Figures 7, 8). These compounds, educts for cytokinin biosynthesis, can be transformed via action of various enzymes including an adenosine phosphate isopentenyl transferase (Hwang and Sakakibara, 2006). Genomic analysis of *Ca.* C. crenata reports both an isopentenyl transferase and a cytokinin-β-glucosidase, while proteomic and genomic analyses of *Ca.* C. kirkii found no evidence for cytokinin biosynthesis (Carlier and Eberl, 2012; Carlier et al., 2013; Carlier et al., 2016). This result can be attributed to multiple metabolic mechanisms. It is either a (residual) plant derived defense response or the bacteria are actively producing this compound to influence the plants hormonal system. This could proceed via direct cytokinin production or control of the cytokinin pools within the tissues due to zeatin glycosylation/deglycosylation (Costacurta and Vanderleyden, 1995). The elevated adenosine and adenosine monophosphate levels within nodulated tissues, together with the findings of (Carlier et al., 2016), point towards cytokinin production by the endophytes, at least in *A. crenata*. Alteration of the plants phytohormone balance by the endophytes could explain the crippled growth of endophyte free plants (Yamada, 1955; Gordon, 1963). In *P. punctata*, however, it is unlikely that the endophyte produces the cytokinin as it lacks the genomic repertoire (Carlier and Eberl, 2012). Nevertheless, there is a strong senescence delay in *P. punctata*: the nodules stay green even when the rest of the leaf turns yellow (Figure 9). The photosynthetic activity is retained around nodule tissue, even when the leaf lamina is no more photosynthetically active (Figure 9). The presence of zeatin could explain why nodules of *P. punctata* retain their chlorophyll during leaf senescence (Rushing, 1990). Yet, there are other mechanisms that could cause this distinctive phenotype. Polyamines (PA), like putrescine and spermidine are involved in senescent mechanisms either through the competition of PAs with ethylene for their common substrate *S*-adenosylmethionine (Walden et al., 1997) or by inhibition of RNAse and protease activities (Kaur-Sawhney et al., 1982). Thus, elevated levels of polyamines (putrescine and spermidine) in leaf nodules could be involved in delay of senescence. However, PAs are also involved in other physiological and developmental processes, like cell division or root development and it is not fully understood, whether increase of PAs is a cause or a consequence of delayed senescence (Evans and Malmberg, 1989). The linkage of nitrogen metabolism in leaf nodules and photosynthesis is subject to future investigations.

### Unique Flavonoid Composition Underpins Symbioses Individuality

*A. crenata* and *P. punctata* show completely different flavonoid signatures (Figures 4B, 5B). Flavonoids are well known for their antioxidative properties and their participation in plant bacteria communications. Flavonoids have also been reported as being part of plant defense responses towards infection (Koes et al., 1994; Rice-Evans et al., 1996; Samanta et al., 2011). Given the close phylogenetic relationship of the two endophytes some convergence in flavonoid composition could be expected. Accordingly the specific flavonoid compositions might rather result from the host specificity (Liu and Murray, 2016). The flavonoid composition in colonized tissue of *A. crenata* indicated that flavonoids might play a specific role in this species’ leaf nodule symbiosis (Figures 4B, 7). These flavonoids could be part of plant-bacteria signaling pathways or could act as antioxidants further protecting the bacteria from harmful ROS (Mierziak et al., 2014). The increased amount of redox active flavonoids within nodulated tissues of *A. crenata* might also explain the reversed GSH pattern in *A. crenata* (Figure 7) compared to *P. punctata* (Figure 8). The flavonoids may contribute to detoxify ROS and thereby take pressure off the GSH available to the bacteria. A significant difference in *A. crenata* compared to *P. punctata* is an epicatechin derivative which we have structurally elucidated (Figure 6A). The modification of this in *A. crenata*, to our knowledge not yet described epicatechin derivative consists of a 3-hydroxy-2-methyl propionate-group (Figure 6A). The (R) enantiomeric form of this group is involved in the degradation pathway of valine (Letto et al., 1986). The derivatization could facilitate the transport of this metabolite. However, the exact R/L configuration needs to be been determined. 

### Bacterial cell wall composition could play an important role in host-microbe-specificity but AhMP modification could also expand the cytotoxic repertoire of leaf symbionts

In our quest to discover common nodule specific elements shared by the two symbioses we identified a broad range of AhMPs. First, our findings confirm that the peptidoglycan of both *Ca.* C. crenata and *Ca.* C. kirkii is composed of *N*-acetylglucosaminyl-*N*-acetylmuramoylalanyl-d-glutamyl-2,6-diaminopimelate-d-alanyl-d-alanine units, as suggested by analyses of *Ca.* C. crenata and *Ca.* C. kirkii, which both contain a UDP-*N*-acetylmuramoylalanyl-d-glutamyl-2,6- diaminopimelate-d-alanyl-d-alanine ligase. While this finding alone might not be surprising for gram-negative bacteria the diversity of related degradation products within the analyzed tissues is. Genomic analysis of *Ca.* C. crenata (Carlier et al., 2016) report both nagZ1 and nagZ2 β-hexosaminidases, which are involved in peptidoglycan recycling. An anhydromuropeptide permease AmpG or 1,6-anhydro-*N*-acetylmuramyl-l-alanine amidase AmpD were only reported in *Ca.* C. kirkii (Carlier et al., 2013). Our findings suggest that the peptidoglycan recycling pathway within the leaf nodule symbiosis either is broken or downregulated. This leads to a vast production of peptidoglycan degradation products in nodules. Lower relative abundances of these metabolites were also detected within the surrounding lamina tissues. Whether the AhMPs reach the laminar tissues by diffusion or are actively transported remains unclear and requires further investigation.

Cytotoxic activity has been reported for the AhMP tracheal cytotoxin (Tat-BP) (Cookson et al., 1989), which was also detected within our samples. Muropeptides have been reported to be involved in symbiotic associations, bacterial communication and pathogenesis in animals and plants. Their presence can also initiate an immune response in eukaryotes (Irazoki et al., 2019; Jõers et al., 2019). It has been reported that bacteria can evade peptidoglycan recognition by altering either glycosidic parts or stem peptides by various means. We detected native anhydromuropeptides, as well as diaminopimelic acid amidated and *N*-acetylated diaminopimelic acid amide anhydromuropeptides. This could result from mechanisms by which the bacteria try to alter their peptidoglycans to avoid triggering an immune response in the host (Yadav et al., 2018). It could however also be a post degradational modification of the anhydromuropeptides to alter their cytotoxicity. Their dynamic remodeling with age (Figures 7, 8) further supports the hypothesis that these compounds are not merely peptidoglycan degradation products but might act as molecular signals within this symbiosis.

## Conclusion

The leaf nodule symbiosis constitutes one of the most intimate kinds of plant bacteria associations known to date. Here we applied, for the first time, a holistic metabolomics-based approach to study the obligate interaction in the two phylogenetically diverse plant species *Ardisia crenata* and *Psychotria punctata*. We investigated our dataset in two respects.

We first took a look at metabolites assigned a putative symbiotic function. Generally, we could show that the leaf nodule metabolite composition follows an age dependent developmental gradient and that metabolites assigned a symbiotic function increase with leaf maturity. We identified putatively novel cyclic-depsipeptides in *A. crenata*, which indicates that the biologically active compound diversity in leaf nodules is even higher than previously thought. We also found pavettamine in tissues of *P. punctata*. We could show that the abundance of pavettamine in leaves depends on the developmental stage and is highly species-specific across Rubiaceae, which produce this polyamine.

Metabolites involved in host-microbe-homeostasis were also analysed by means of differential abundance analysis in different tissues. We found that maltose may act as central carbohydrate storage precursor in endophytes. After hypothesizing about other carbon sources supplied to the bacteria we focused on C4-dicarboxylic acids. Variable organic acid distributions indicated that regulation of the TCA energy metabolism is differentially regulated in the two endophytes. Nevertheless, we conclude that citric acid plays a crucial role in the development of both endophytes.

In terms of energy, carbon and nitrogen source supplied to the endophytes we propose that leucine or other amino acids may constitute this source but that it may also be used by the plant to control the bacterial population. A local protective mechanism for photosynthesis by leaf nodulation is observed in senescent leafs which needs further investigations.

Last, we would like to highlight the importance of bacterial cell wall composition, which could play an important role in host-microbe-specificity. AhMP modification could also expand the cytotoxic repertoire of leaf symbionts and contribute to their function as plant defense agents.

Finally, our comparative metabolomic analysis of leaf nodule and lamina tissues in *A. crenata* and *P. punctata* revealed important novel aspects of the interaction between the host and its bacterium and provided insights into this mutualistic relationship. The metabolomics data provide a first reference platform for further investigations of this highly interesting symbiotic relationship and potential resource of novel natural products.

## Data Availability

Metabolomics data have been deposited to the EMBL-EBI MetaboLights database (DOI: 10.1093/nar/gkz1019, PMID:31691833) with the identifier MTBLS2870 (Haug et al., 2019). The complete dataset can be accessed here https://www.ebi.ac.uk/metabolights/MTBLS2870.
