# Supplementary material for: Dissecting Metabolism of Leaf Nodules in Ardisia crenata and Psychotria punctata
Source: Front Mol Biosci. 2021 Jul 30;8:683671. doi: 10.3389/fmolb.2021.683671 (PMC8362603; doi:10.3389/fmolb.2021.683671)
Supplement: Supplementary file 1 [file DataSheet2.pdf]

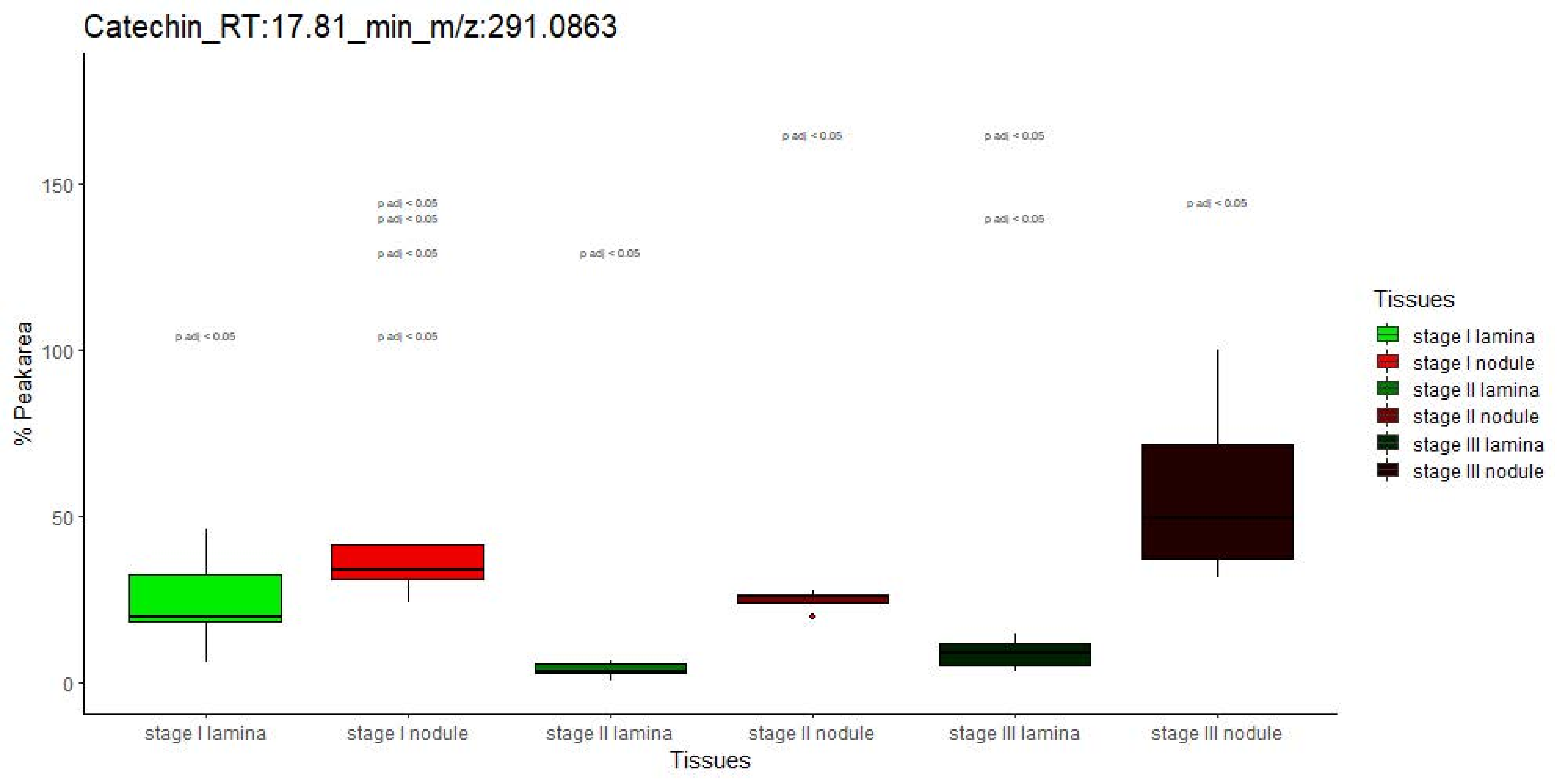

Catechin_RT:17.81_min_m/z:291.0863
% Peakarea
0
50
100
150
p adj < 0.05
stage I lamina
stage I nodule
stage II lamina
stage II nodule
stage III lamina
stage III nodule
Tissues

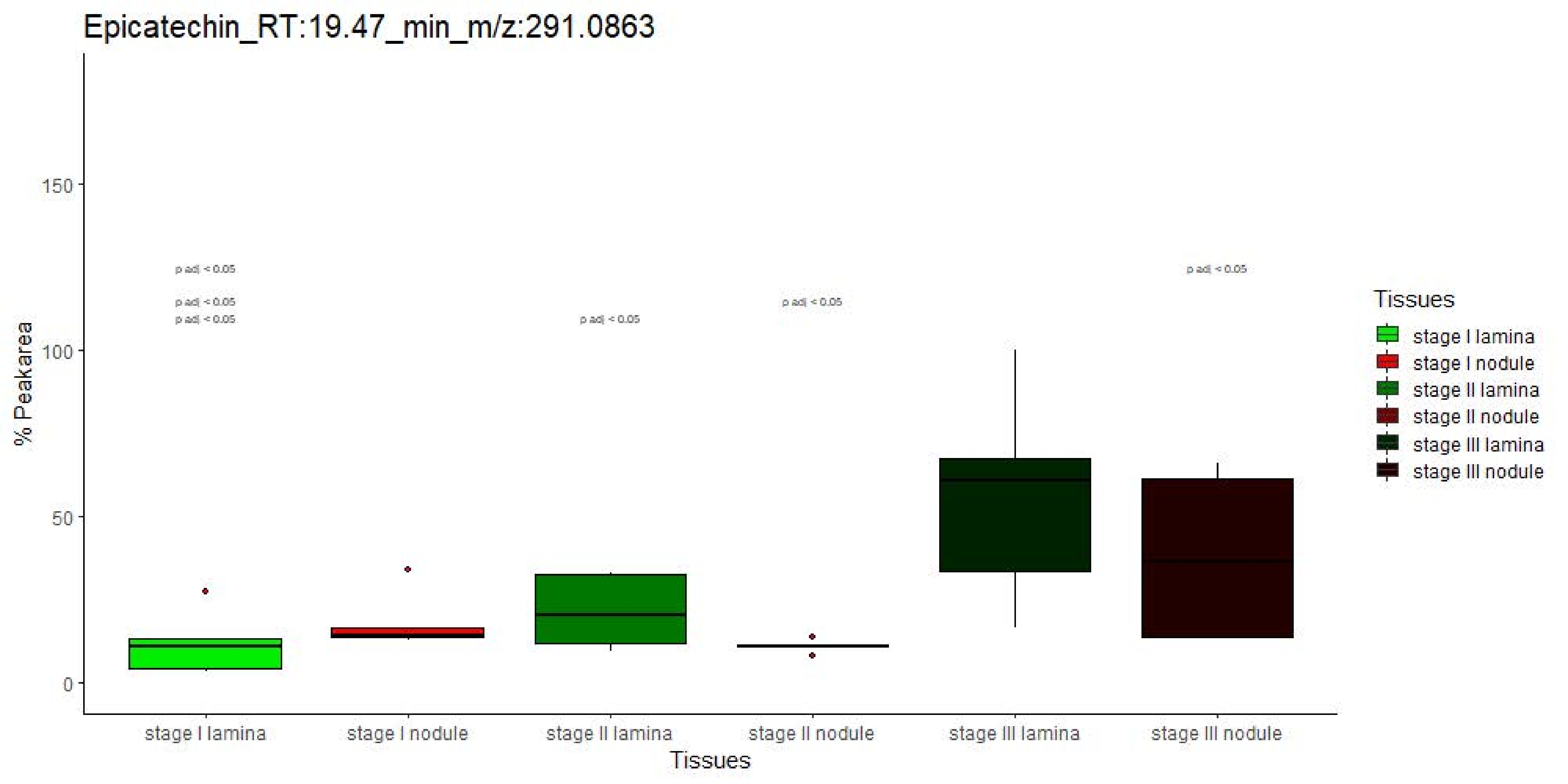
Epicatechin_RT:19.47_min_m/z:291.0863
% Peakarea
150
100
50
0
p adj < 0.05
p adj < 0.05
p adj < 0.05
p adj < 0.05
p adj < 0.05
p adj < 0.05
stage I lamina
stage I nodule
stage II lamina
stage II nodule
stage III lamina
stage III nodule
Tissues
Tissues
stage I lamina
stage I nodule
stage II lamina
stage II nodule
stage III lamina
stage III nodule

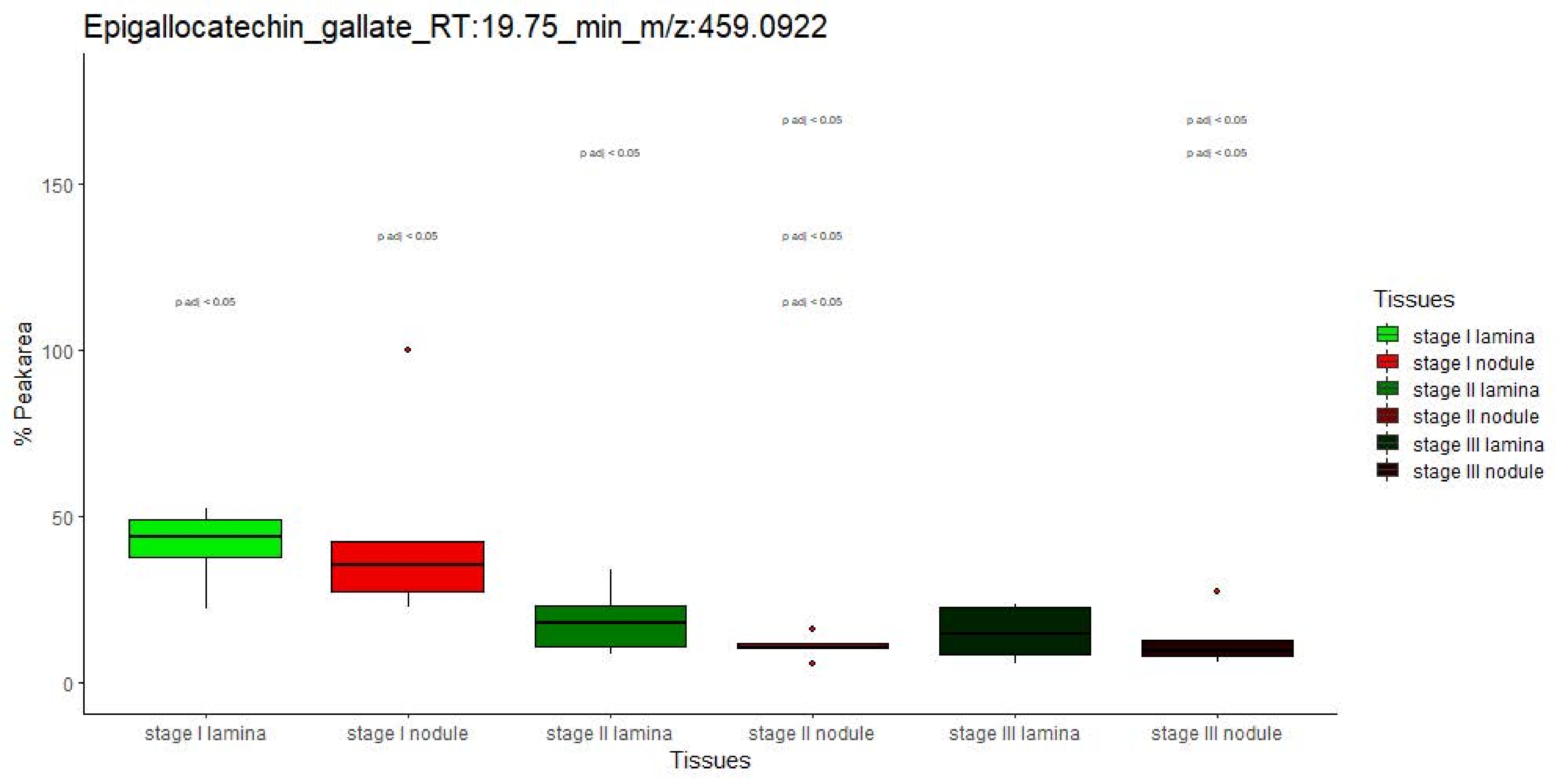
Epigallocatechin_gallate_RT:19.75_min_m/z:459.0922
% Peakarea
150
100
50
0
p adj < 0.05
p adj < 0.05
p adj < 0.05
p adj < 0.05
p adj < 0.05
p adj < 0.05
p adj < 0.05
p adj < 0.05
stage I lamina
stage I nodule
stage II lamina
stage II nodule
stage III lamina
stage III nodule
Tissues
Tissues
stage I lamina
stage I nodule
stage II lamina
stage II nodule
stage III lamina
stage III nodule

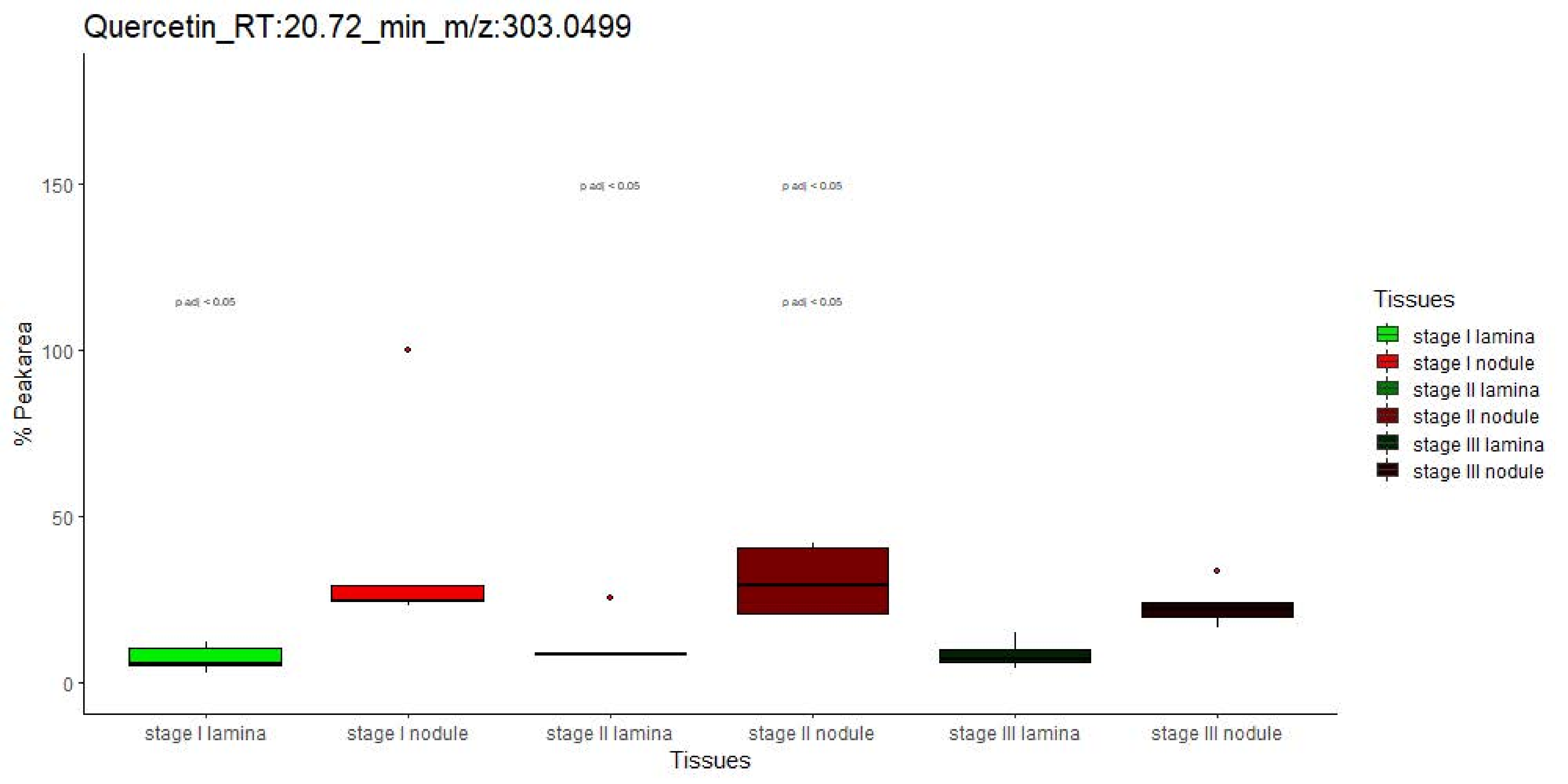

Quercetin_RT:20.72_min_m/z:303.0499
p adj < 0.05
p adj < 0.05
p adj < 0.05
p adj < 0.05
% Peakarea
150
100
50
0
stage I lamina
stage I nodule
stage II lamina
stage II nodule
stage III lamina
stage III nodule
Tissues
Tissues
stage I lamina
stage I nodule
stage II lamina
stage II nodule
stage III lamina
stage III nodule

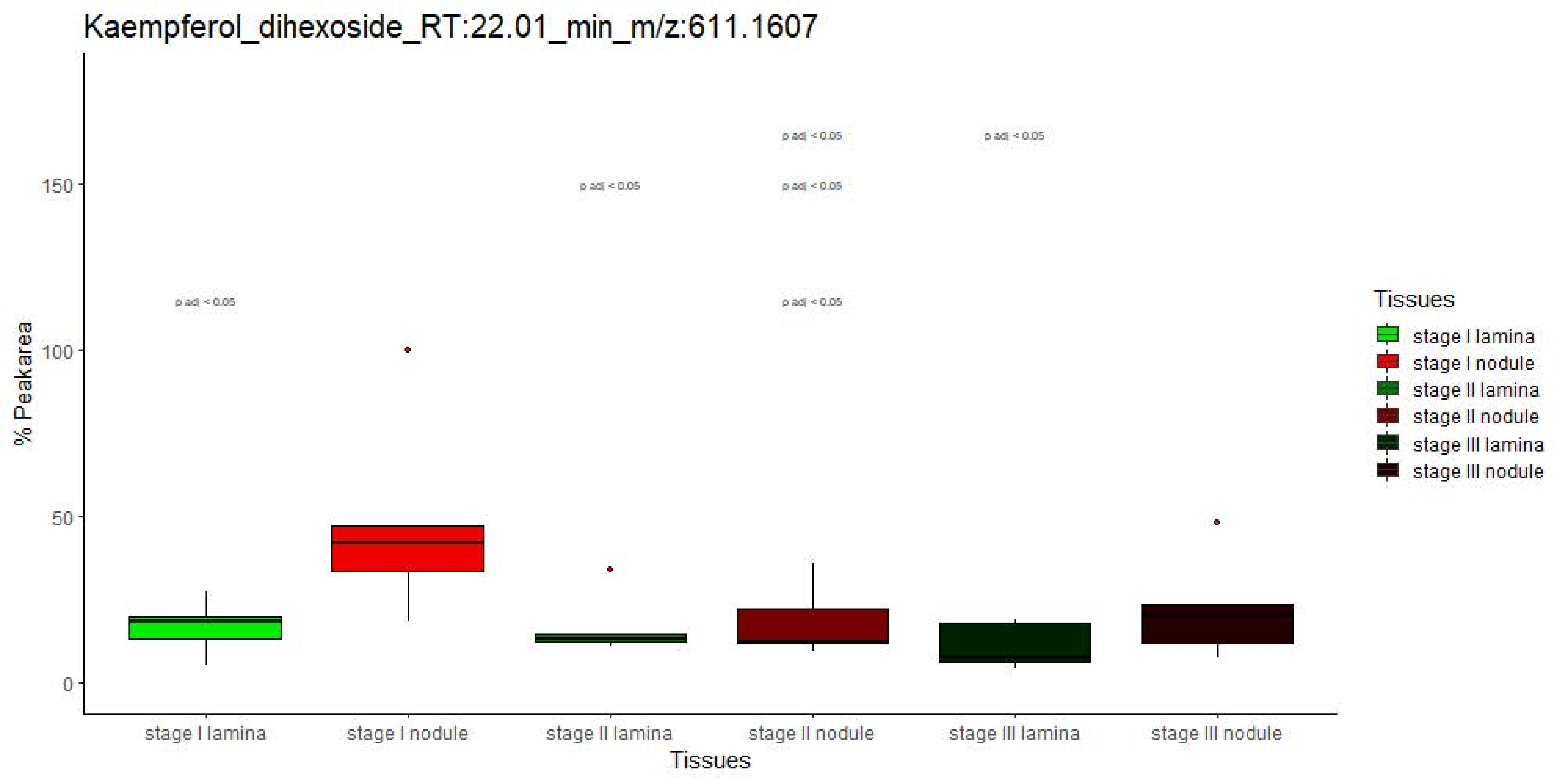

Kaempferol_dihexoside_RT:22.01_min_m/z:611.1607
% Peakarea
0
50
100
150
p adj < 0.05
p adj < 0.05
p adj < 0.05
p adj < 0.05
p adj < 0.05
p adj < 0.05
stage I lamina
stage I nodule
stage II lamina
stage II nodule
stage III lamina
stage III nodule
Tissues
Tissues
stage I lamina
stage I nodule
stage II lamina
stage II nodule
stage III lamina
stage III nodule

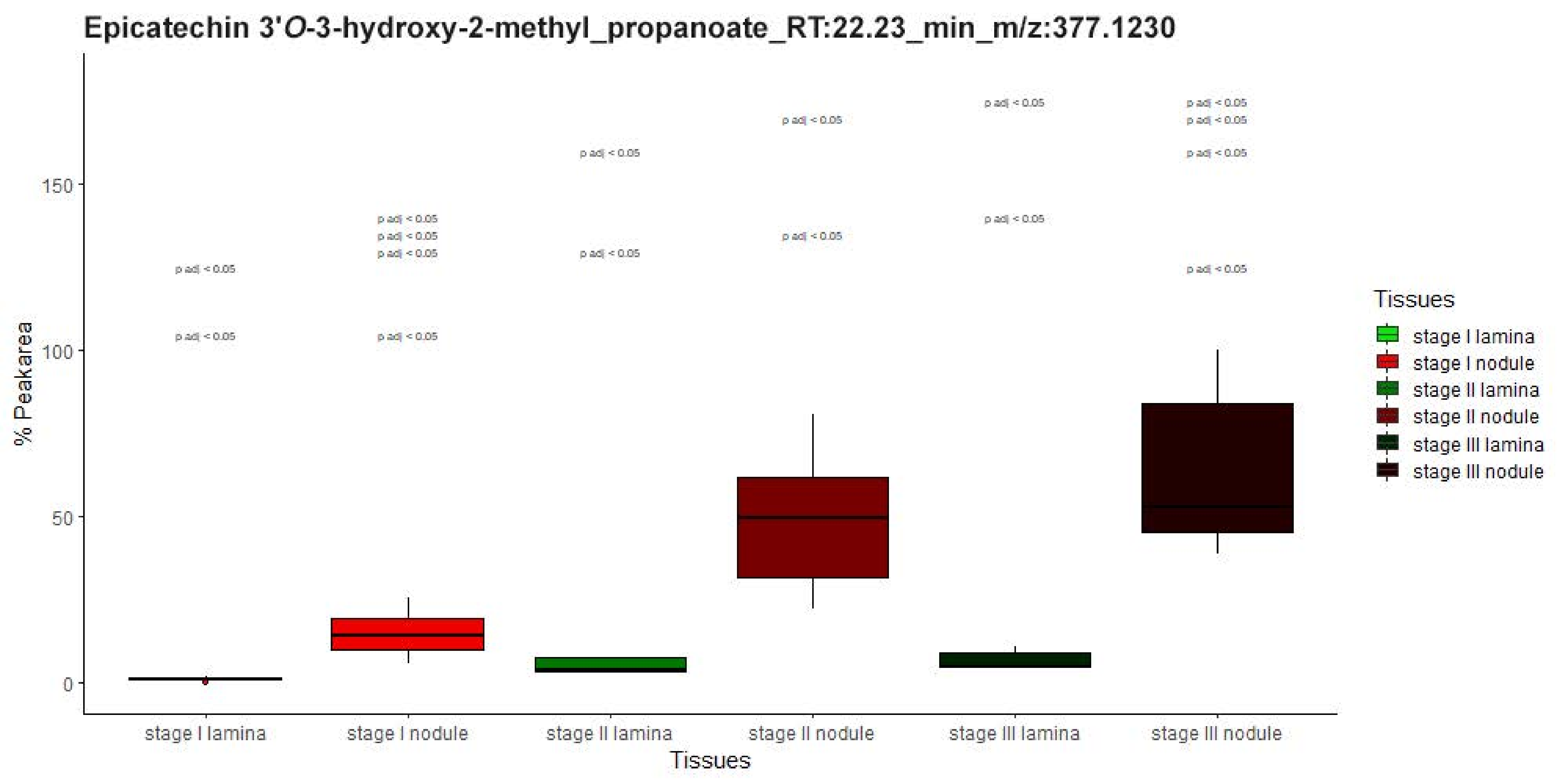

Epicatechin 3'O-3-hydroxy-2-methyl_propanoate_RT:22.23_min_m/z:377.1230
% Peakarea
Tissues
p adj < 0.05
stage I lamina
stage I nodule
stage II lamina
stage II nodule
stage III lamina
stage III nodule

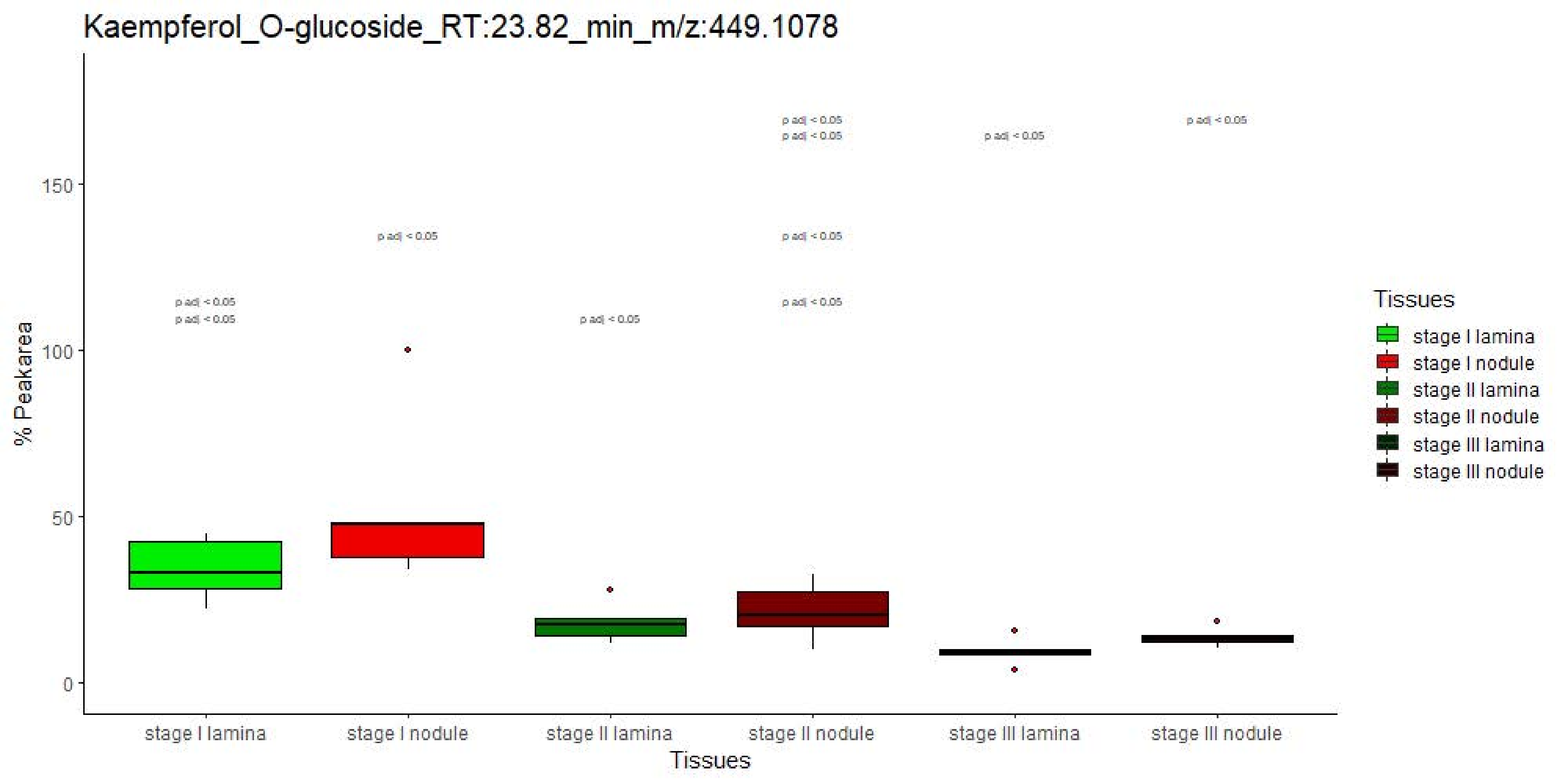

Kaempferol_O-glucoside_RT:23.82_min_m/z:449.1078
% Peakarea
150
100
50
0
p adj < 0.05
p adj < 0.05
p adj < 0.05
p adj < 0.05
p adj < 0.05
p adj < 0.05
p adj < 0.05
p adj < 0.05
p adj < 0.05
p adj < 0.05
stage I lamina
stage I nodule
stage II lamina
stage II nodule
stage III lamina
stage III nodule
Tissues
Tissues
stage I lamina
stage I nodule
stage II lamina
stage II nodule
stage III lamina
stage III nodule

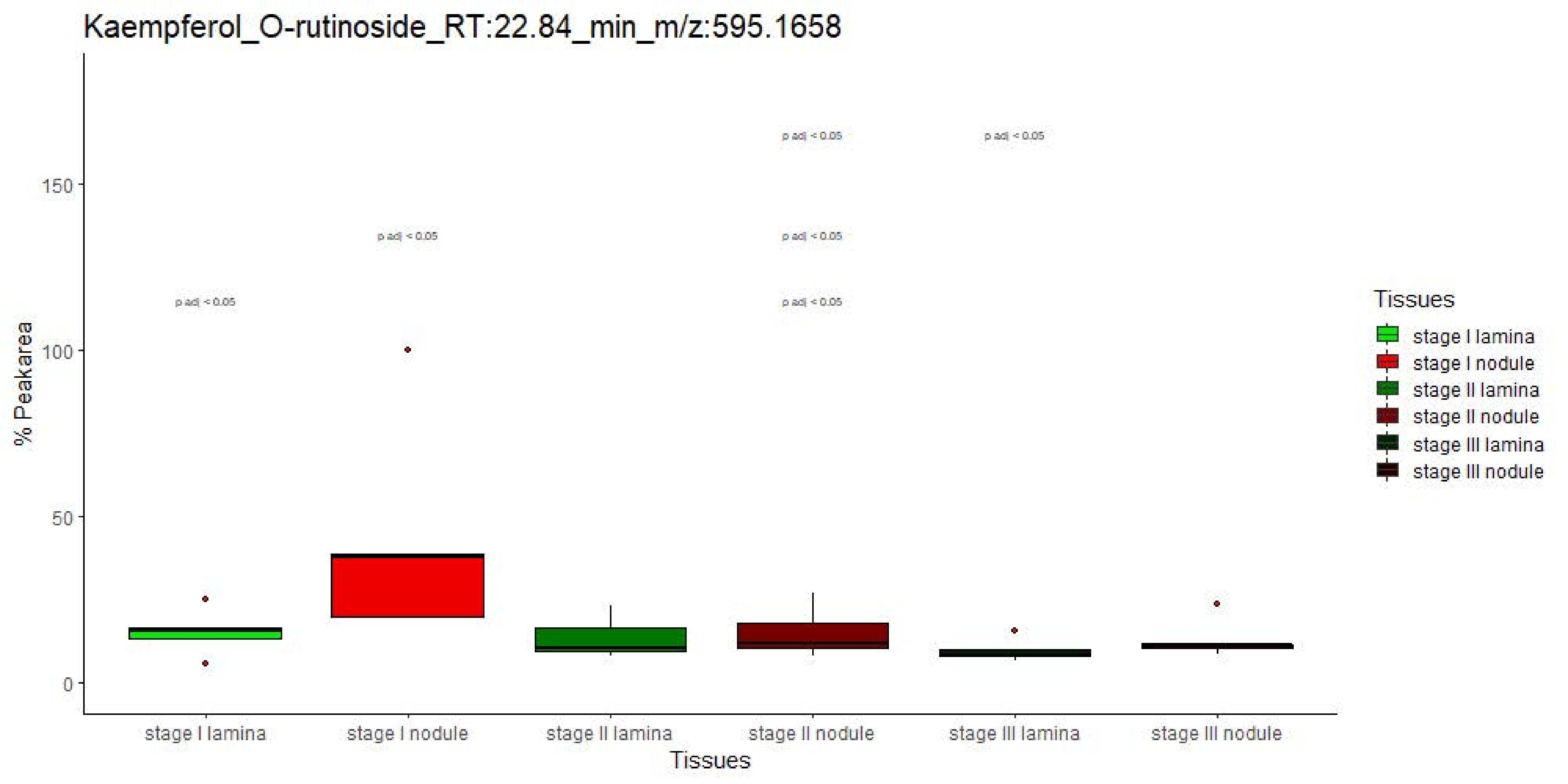

Kaempferol_O-rutinoside_RT:22.84_min_m/z:595.1658
% Peakarea
150
100
50
0
p adj < 0.05
p adj < 0.05
p adj < 0.05
p adj < 0.05
p adj < 0.05
p adj < 0.05
stage I lamina
stage I nodule
stage II lamina
stage II nodule
stage III lamina
stage III nodule
Tissues
Tissues
stage I lamina
stage I nodule
stage II lamina
stage II nodule
stage III lamina
stage III nodule

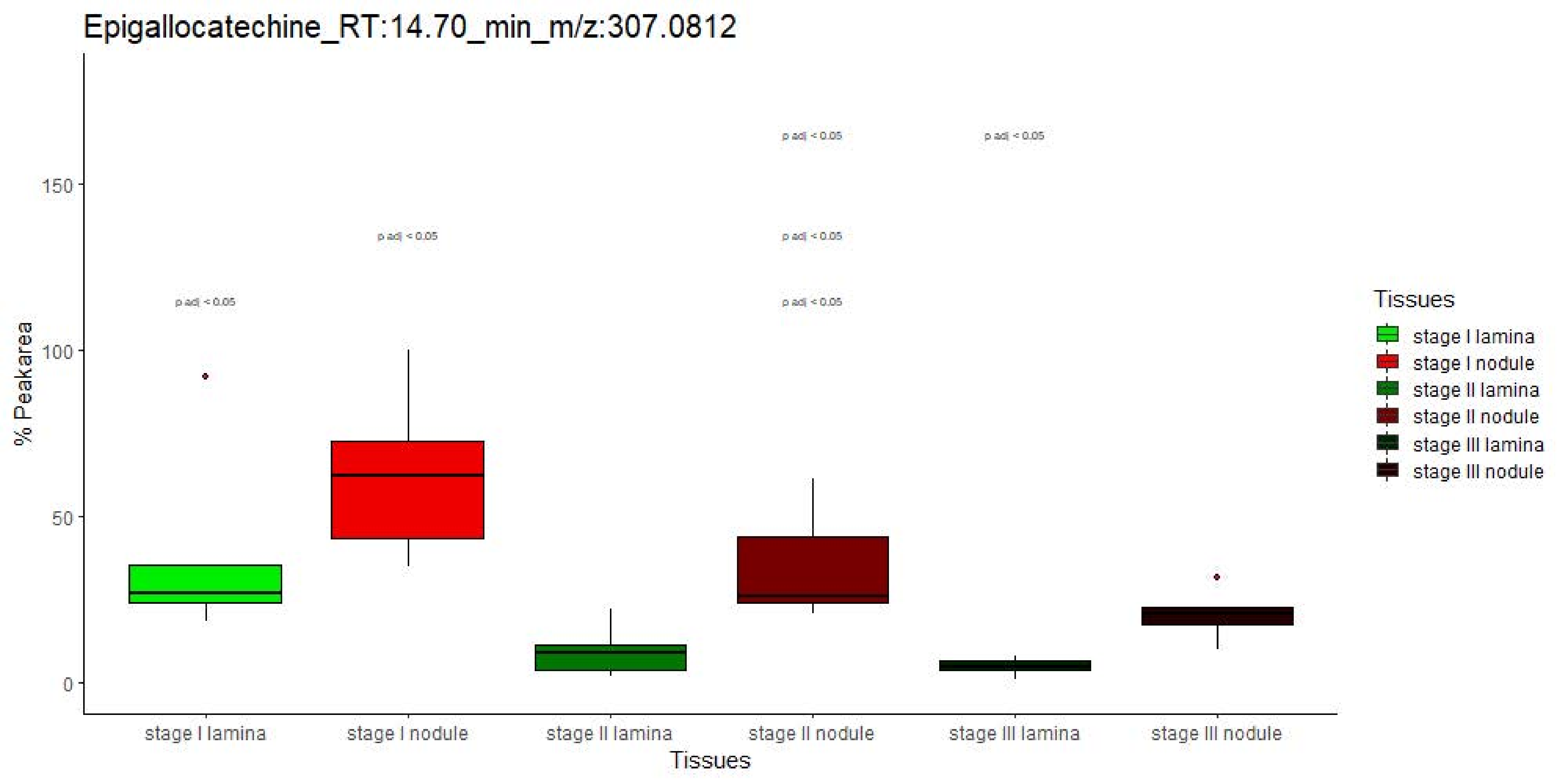
Epigallocatechine_RT:14.70_min_m/z:307.0812
% Peakarea
Tissues
150
100
50
0
p adj < 0.05
p adj < 0.05
p adj < 0.05
p adj < 0.05
p adj < 0.05
p adj < 0.05
stage I lamina
stage I nodule
stage II lamina
stage II nodule
stage III lamina
stage III nodule
Tissues
stage I lamina
stage I nodule
stage II lamina
stage II nodule
stage III lamina
stage III nodule

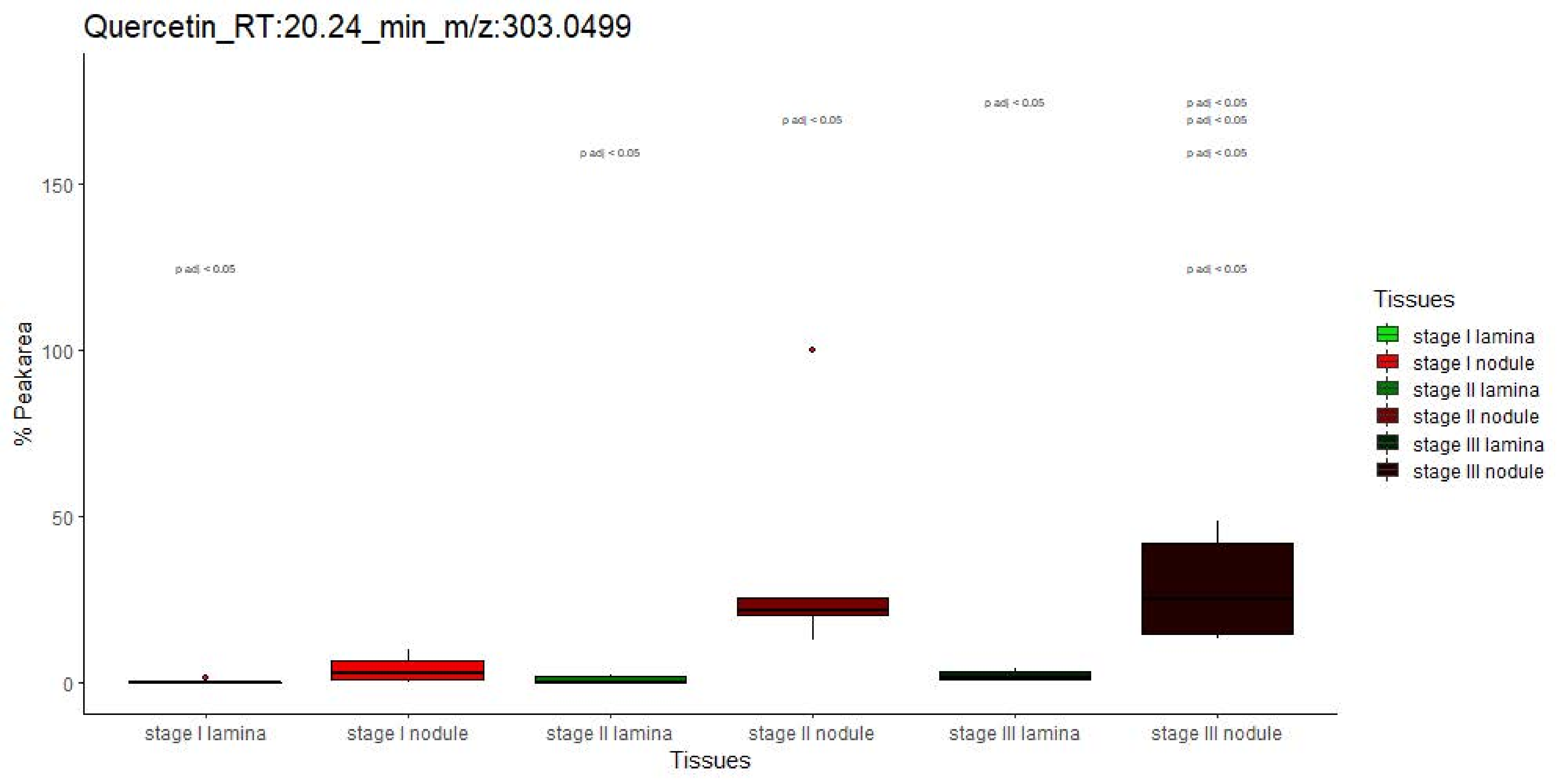

Quercetin_RT:20.24_min_m/z:303.0499
p adj < 0.05
p adj < 0.05
p adj < 0.05
p adj < 0.05
p adj < 0.05
p adj < 0.05
p adj < 0.05
p adj < 0.05
p adj < 0.05
150
100
50
0
% Peakarea
stage I lamina
stage I nodule
stage II lamina
stage II nodule
stage III lamina
stage III nodule
Tissues
Tissues
stage I lamina
stage I nodule
stage II lamina
stage II nodule
stage III lamina
stage III nodule

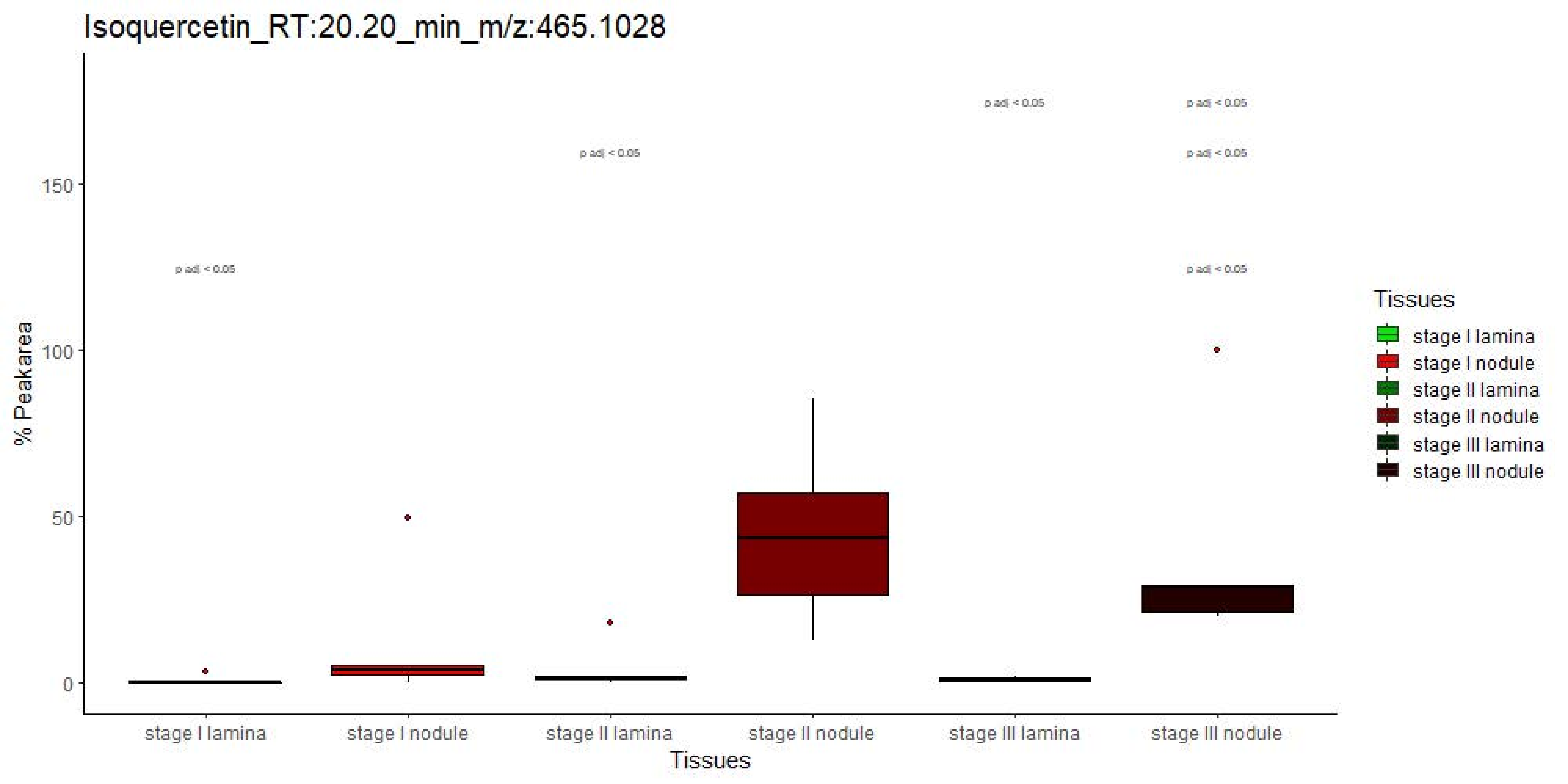

Isoquercetin_RT:20.20_min_m/z:465.1028
% Peakarea
0
50
100
150
p adj < 0.05
p adj < 0.05
p adj < 0.05
p adj < 0.05
p adj < 0.05
p adj < 0.05
stage I lamina
stage I nodule
stage II lamina
stage II nodule
stage III lamina
stage III nodule
Tissues
Tissues
stage I lamina
stage I nodule
stage II lamina
stage II nodule
stage III lamina
stage III nodule

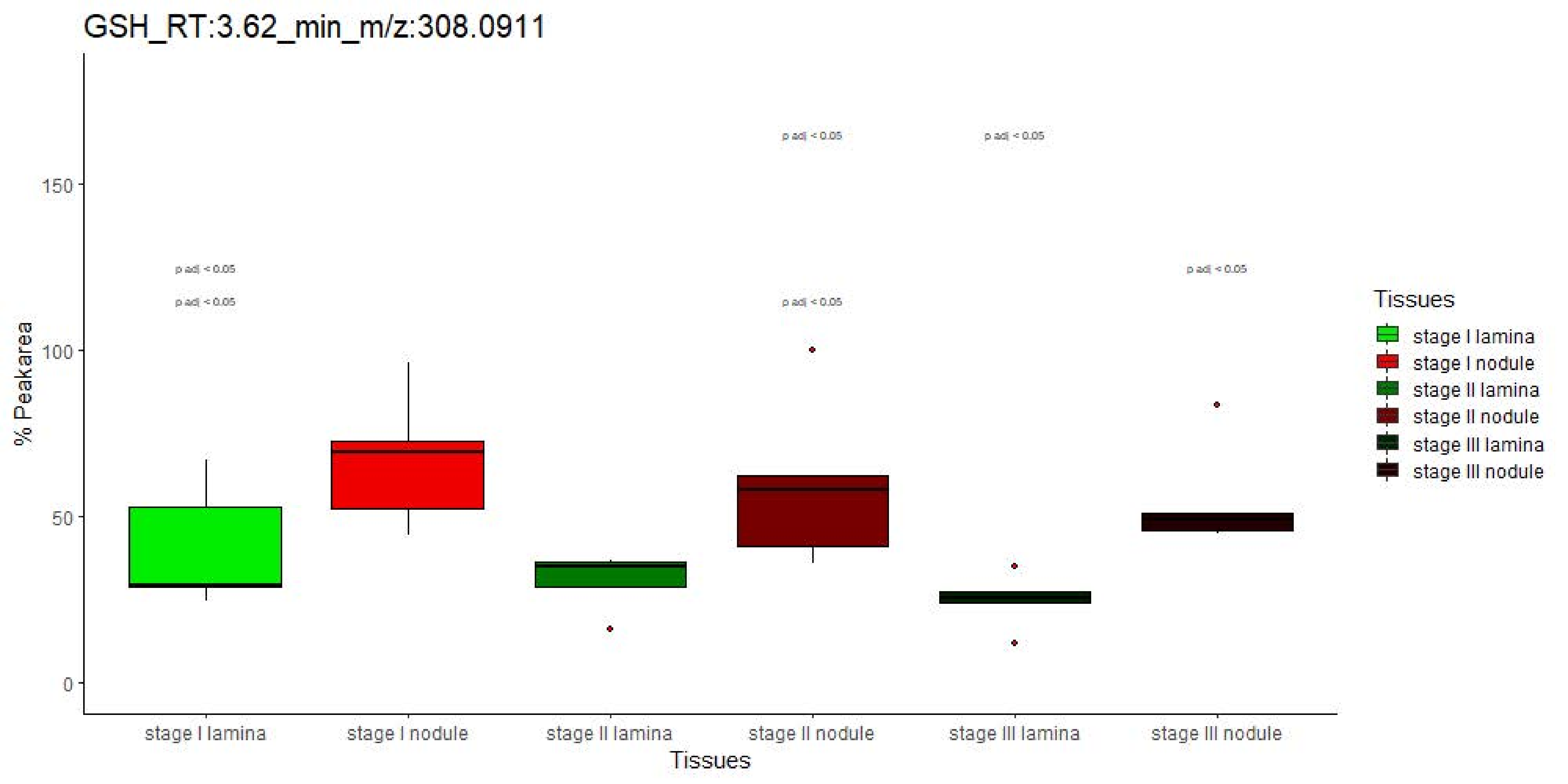
GSH_RT:3.62_min_m/z:308.0911
p adj < 0.05
p adj < 0.05
p adj < 0.05
p adj < 0.05
p adj < 0.05
p adj < 0.05
% Peakarea
150
100
50
0
stage I lamina
stage I nodule
stage II lamina
stage II nodule
stage III lamina
stage III nodule
Tissues
Tissues
stage I lamina
stage I nodule
stage II lamina
stage II nodule
stage III lamina
stage III nodule

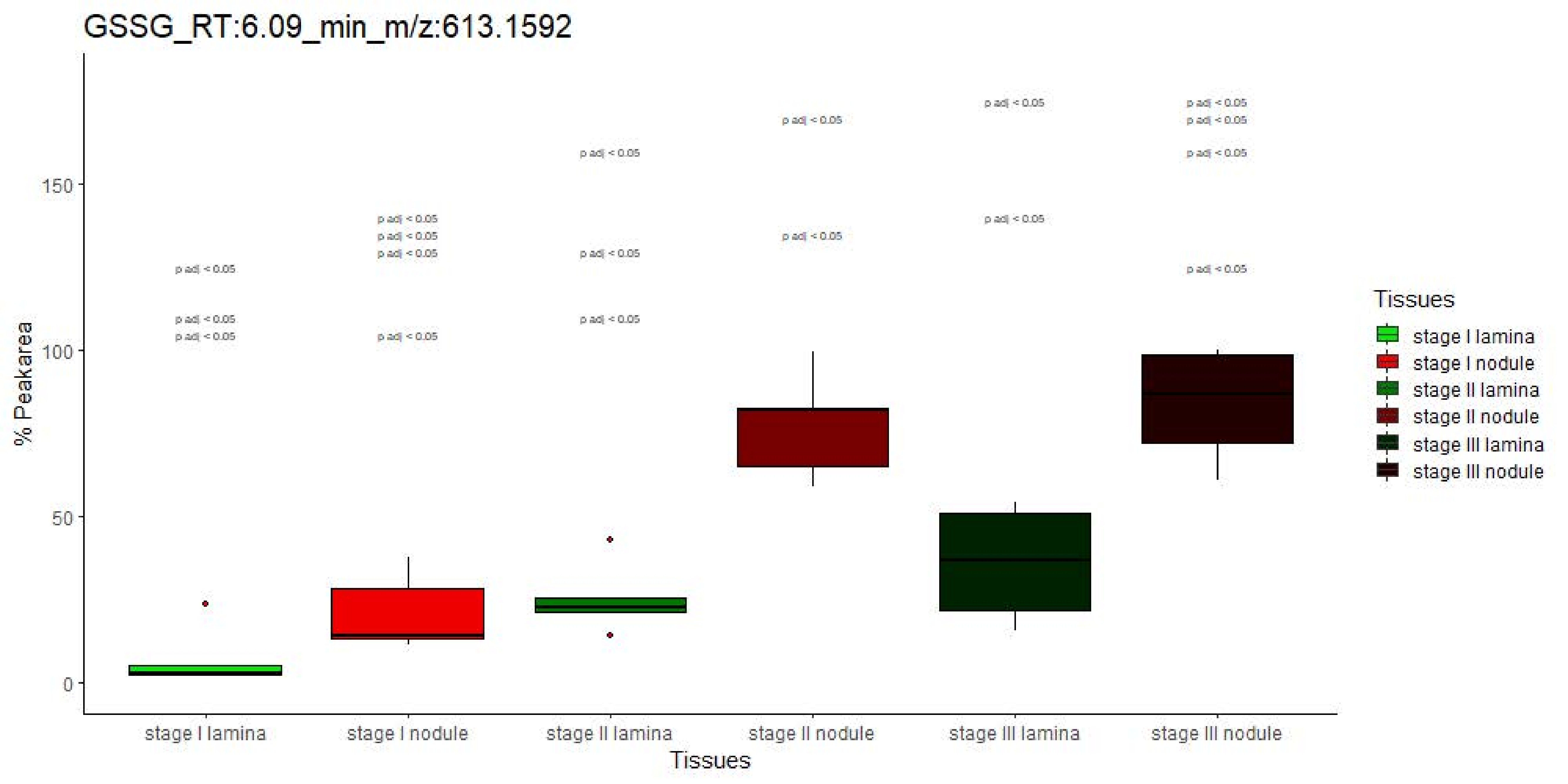
GSSG_RT:6.09_min_m/z:613.1592
% Peakarea
150
100
50
0
p adj < 0.05
stage I lamina
stage I nodule
stage II lamina
stage II nodule
stage III lamina
stage III nodule
Tissues
Tissues
stage I lamina
stage I nodule
stage II lamina
stage II nodule
stage III lamina
stage III nodule

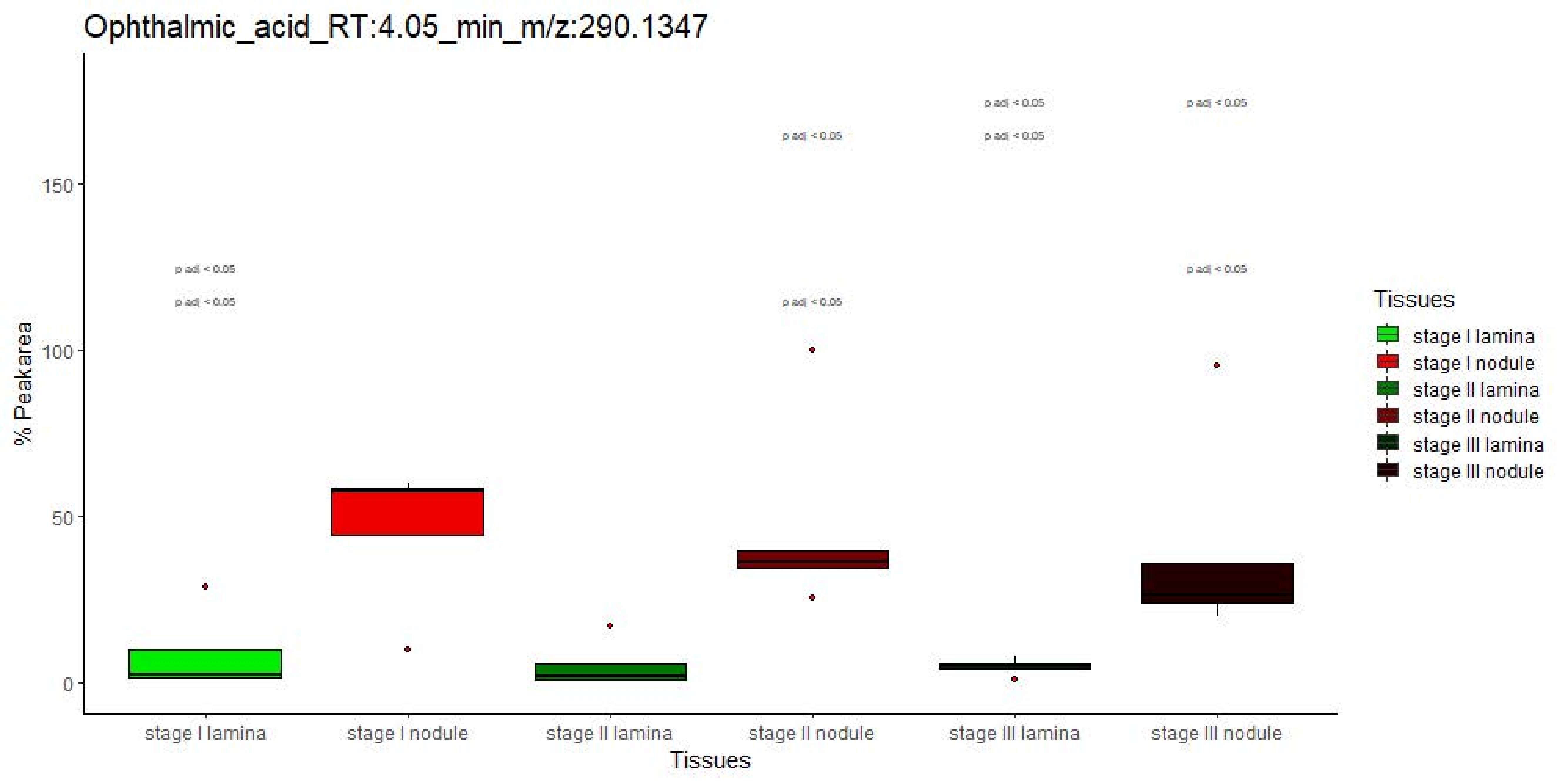
Ophthalmic_acid_RT:4.05_min_m/z:290.1347
% Peakarea
150
100
50
0
p adj < 0.05
p adj < 0.05
p adj < 0.05
p adj < 0.05
p adj < 0.05
p adj < 0.05
p adj < 0.05
p adj < 0.05
p adj < 0.05
p adj < 0.05
stage I lamina
stage I nodule
stage II lamina
stage II nodule
stage III lamina
stage III nodule
Tissues
Tissues
stage I lamina
stage I nodule
stage II lamina
stage II nodule
stage III lamina
stage III nodule

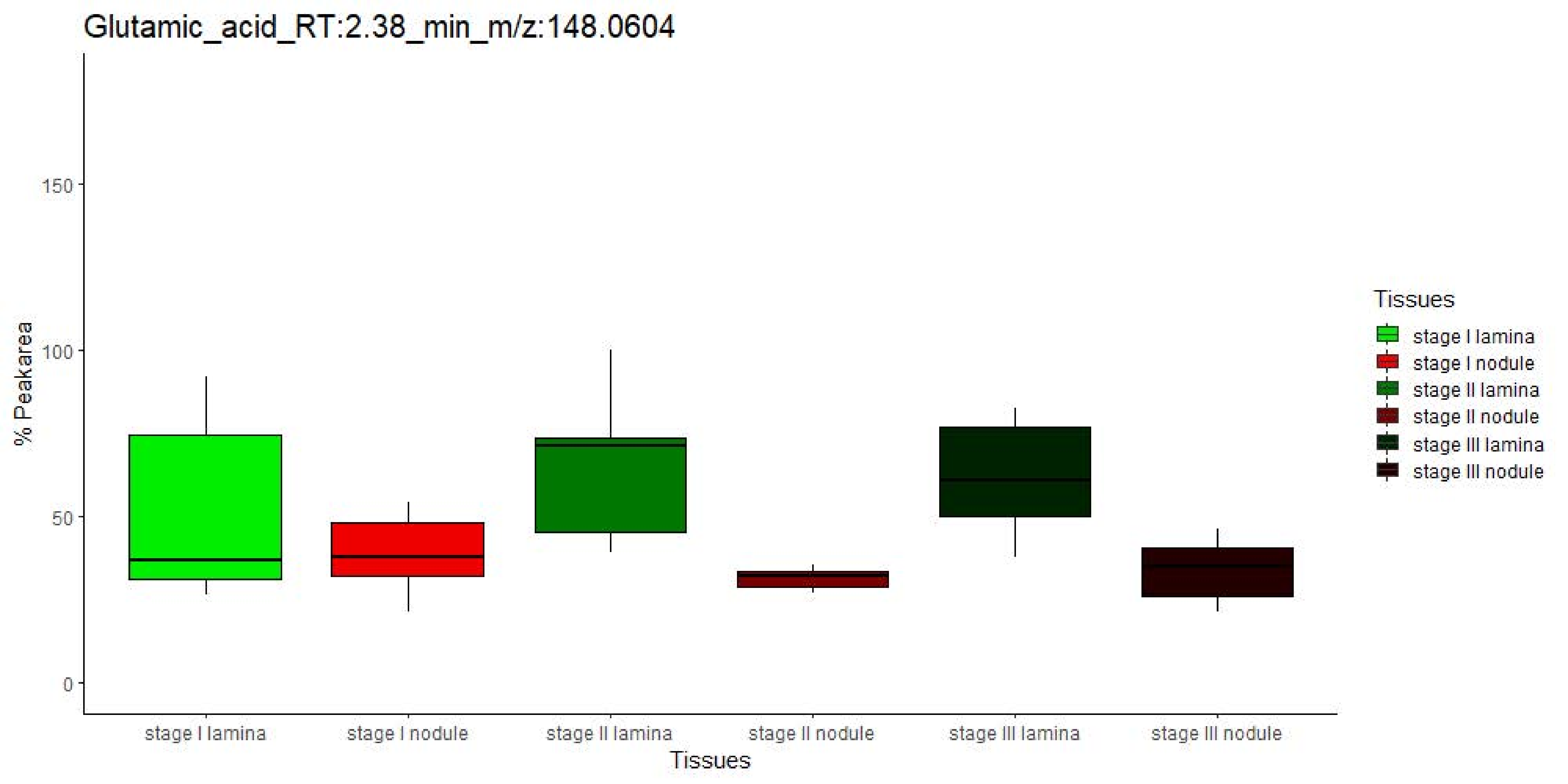

Glutamic_acid_RT:2.38_min_m/z:148.0604
% Peakarea
0
50
100
150
stage I lamina
stage I nodule
stage II lamina
stage II nodule
stage III lamina
stage III nodule
Tissues
Tissues
stage I lamina
stage I nodule
stage II lamina
stage II nodule
stage III lamina
stage III nodule

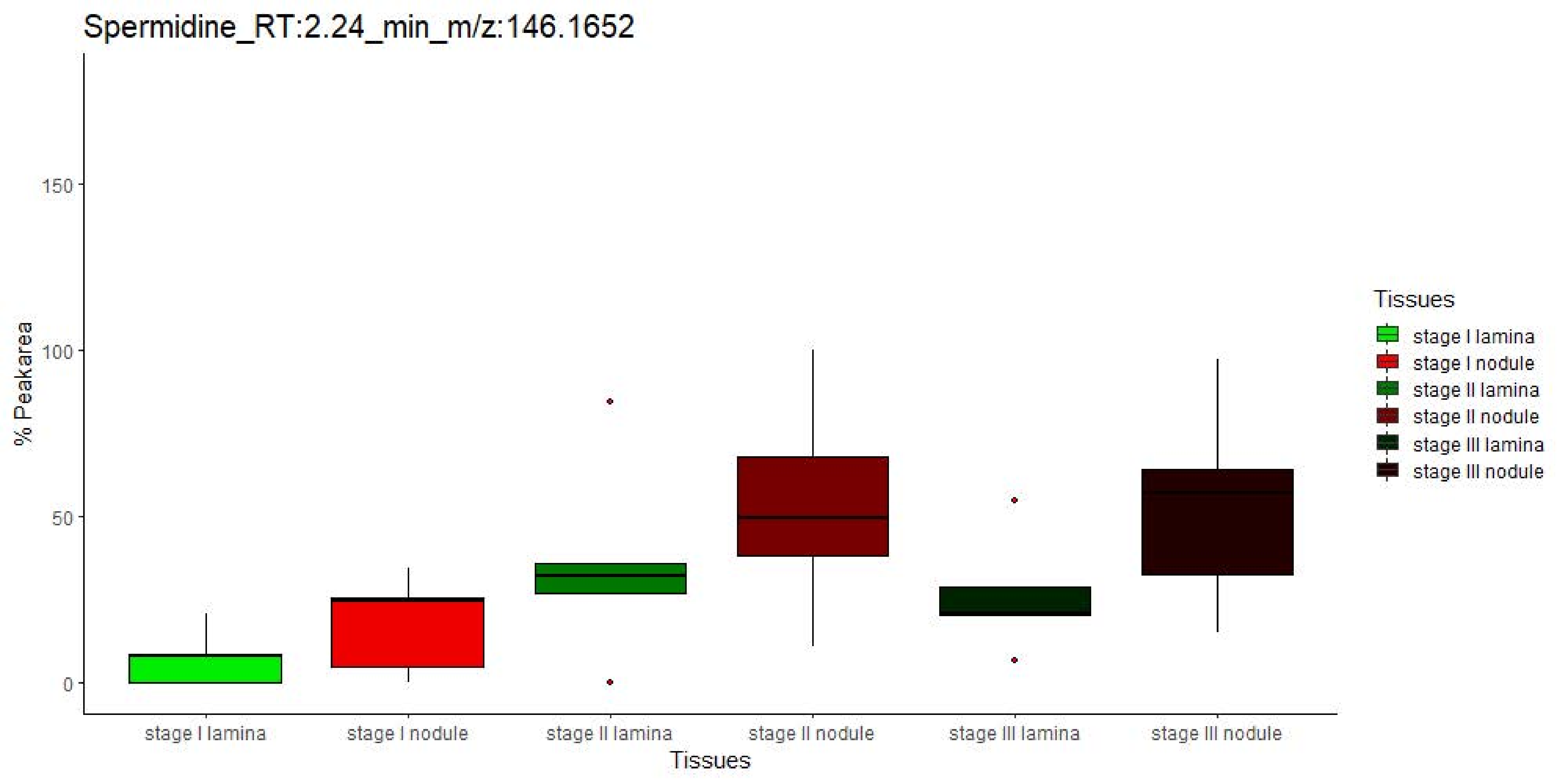

Spermidine_RT:2.24_min_m/z:146.1652
% Peakarea
150
100
50
0
stage I lamina
stage I nodule
stage II lamina
stage II nodule
stage III lamina
stage III nodule
Tissues
Tissues
stage I lamina
stage I nodule
stage II lamina
stage II nodule
stage III lamina
stage III nodule

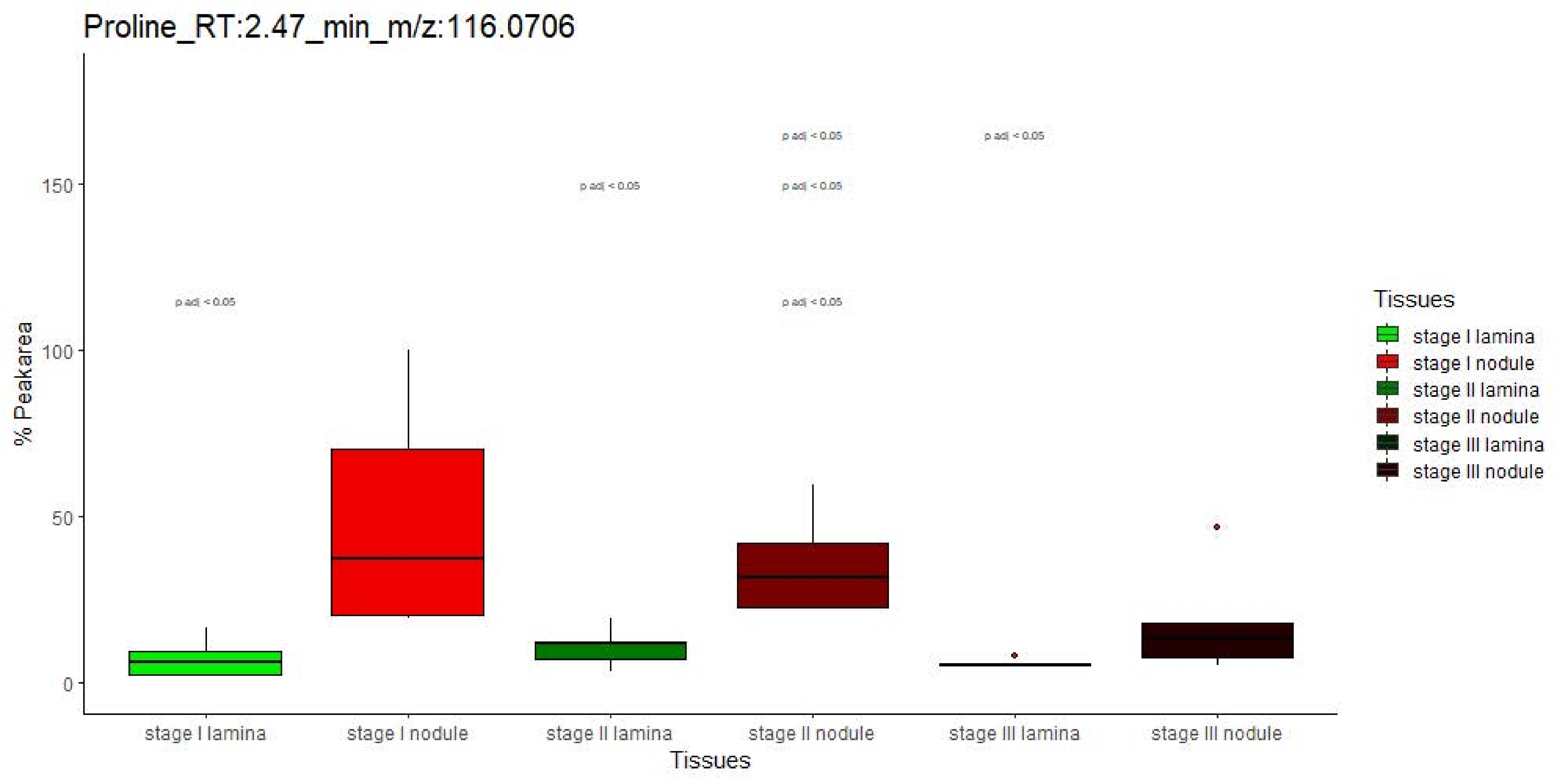
Proline_RT:2.47_min_m/z:116.0706
p adj < 0.05
p adj < 0.05
p adj < 0.05
p adj < 0.05
p adj < 0.05
p adj < 0.05
% Peakarea
150
100
50
0
stage I lamina
stage I nodule
stage II lamina
stage II nodule
stage III lamina
stage III nodule
Tissues
Tissues
stage I lamina
stage I nodule
stage II lamina
stage II nodule
stage III lamina
stage III nodule

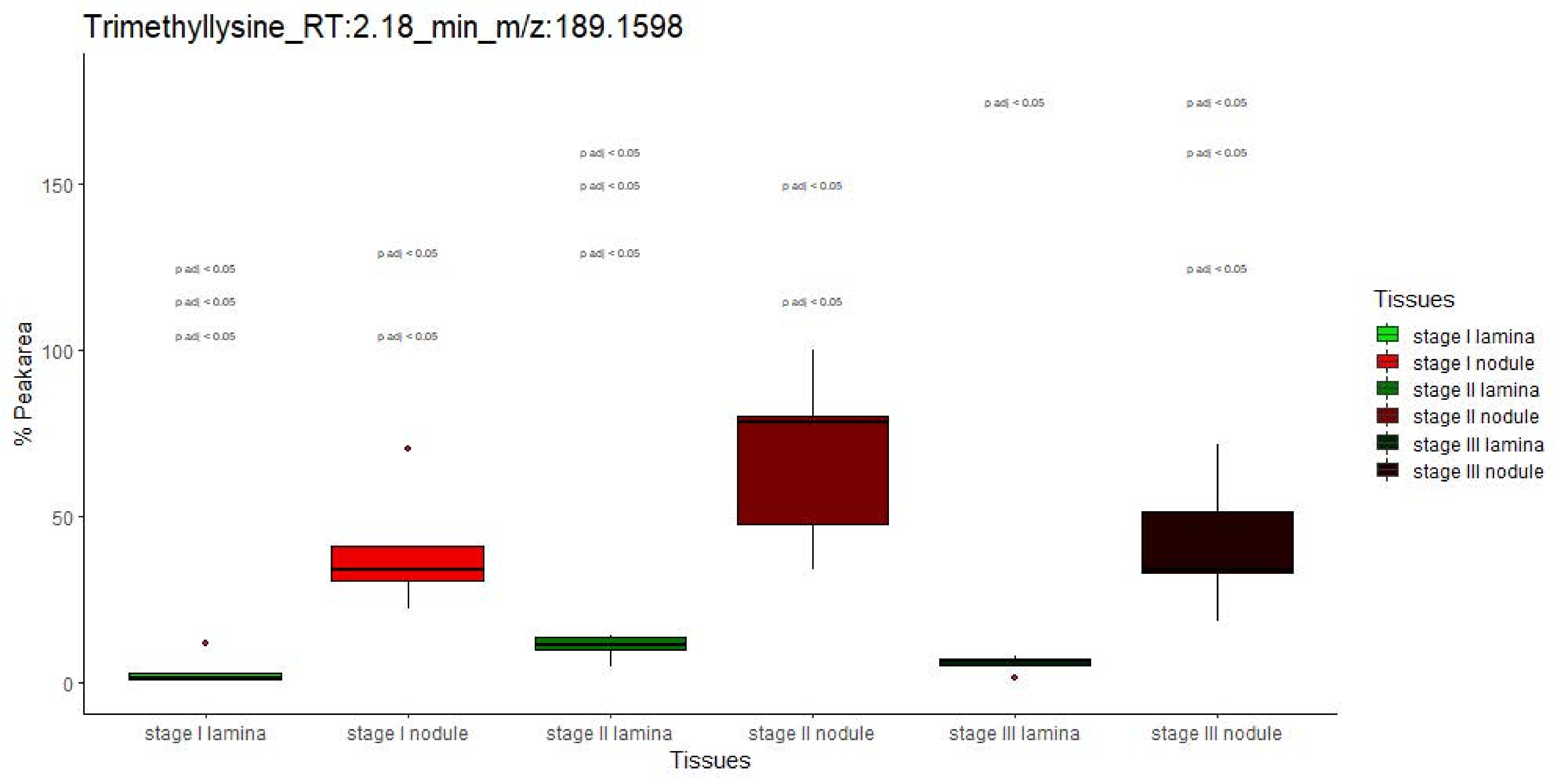

Trimethyllysine_RT:2.18_min_m/z:189.1598
% Peakarea
0
50
100
150
p adj < 0.05
Tissues
stage I lamina
stage I nodule
stage II lamina
stage II nodule
stage III lamina
stage III nodule

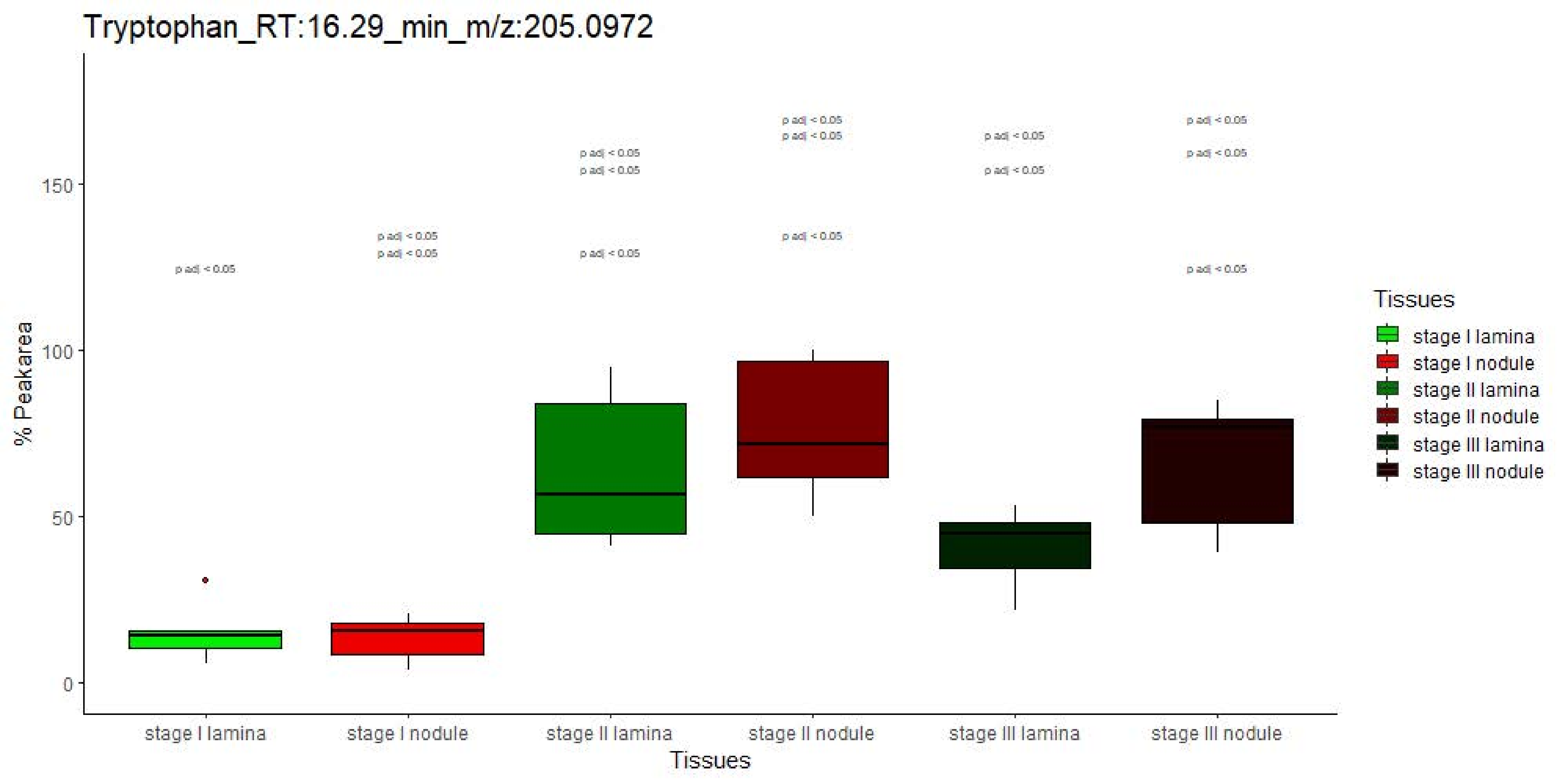

Tryptophan_RT:16.29_min_m/z:205.0972
% Peakarea
150
100
50
0
p adj < 0.05
Tissues
stage I lamina
stage I nodule
stage II lamina
stage II nodule
stage III lamina
stage III nodule

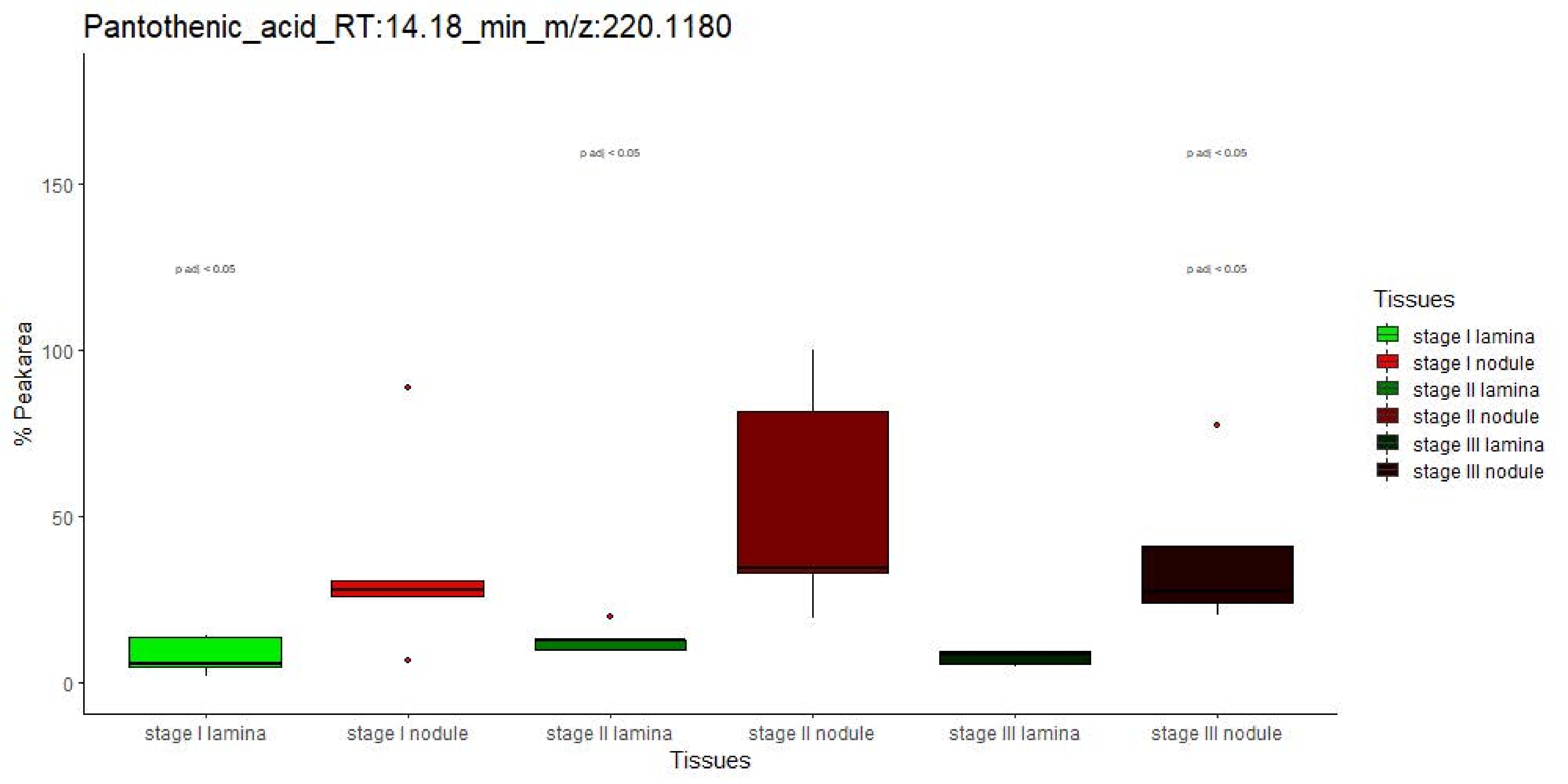

Pantothenic_acid_RT:14.18_min_m/z:220.1180
p adj < 0.05
p adj < 0.05
p adj < 0.05
p adj < 0.05
150
100
50
0
% Peakarea
stage I lamina
stage I nodule
stage II lamina
stage II nodule
stage III lamina
stage III nodule
Tissues
Tissues
stage I lamina
stage I nodule
stage II lamina
stage II nodule
stage III lamina
stage III nodule

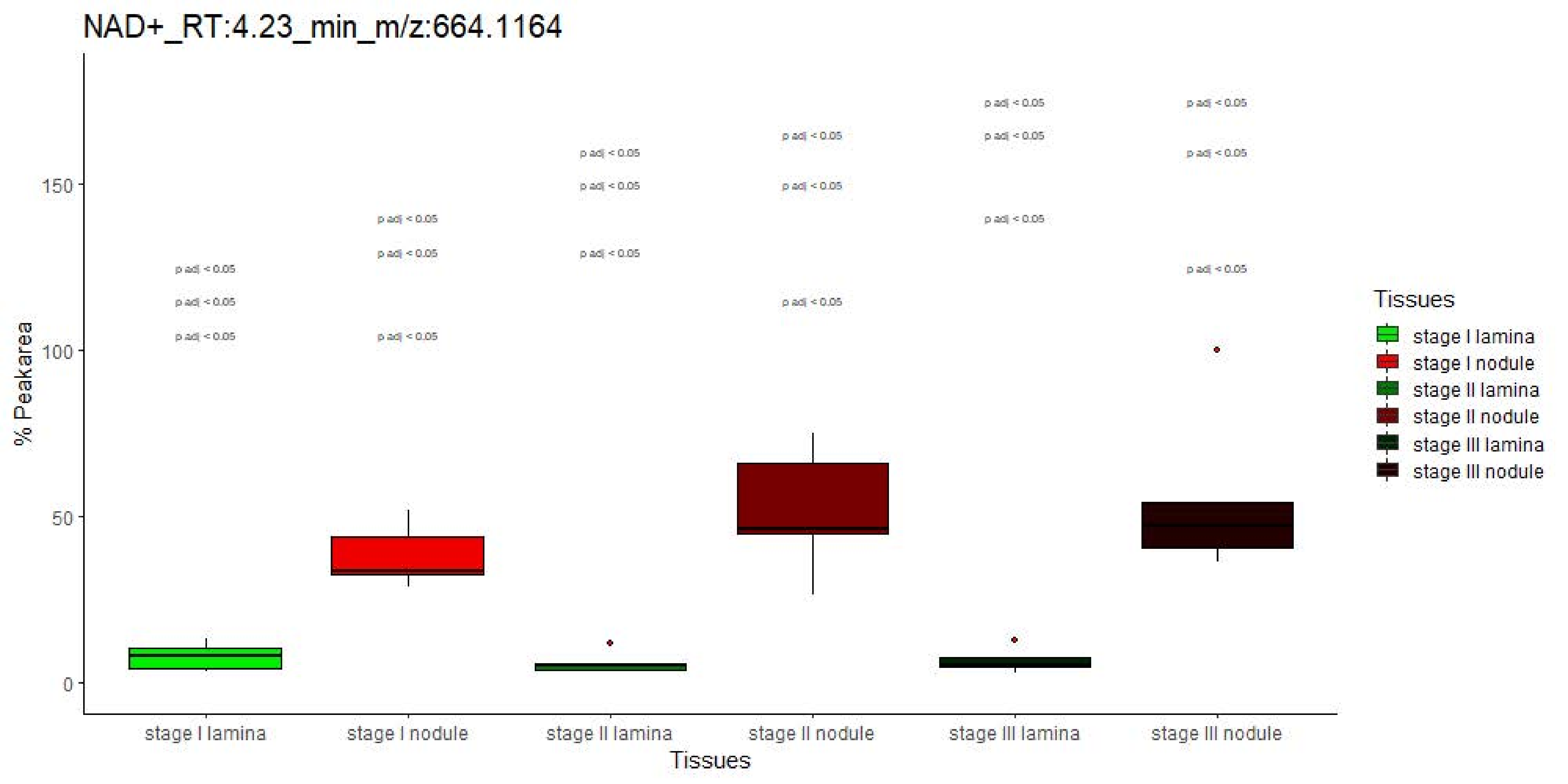

NAD+_RT:4.23_min_m/z:664.1164
% Peakarea
0
50
100
150
p adj < 0.05
Tissues
stage I lamina
stage I nodule
stage II lamina
stage II nodule
stage III lamina
stage III nodule

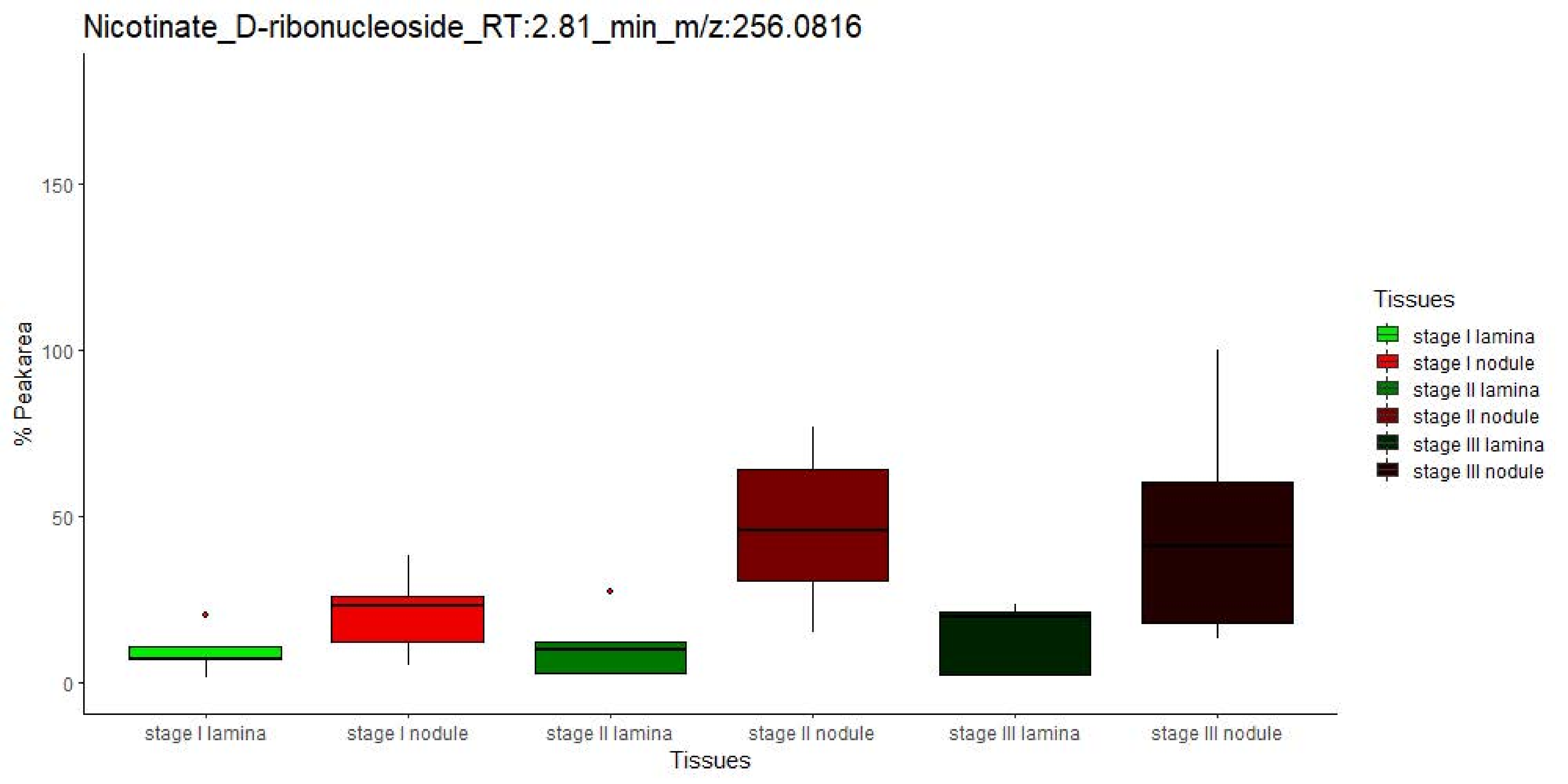

Nicotinate_D-ribonucleoside_RT:2.81_min_m/z:256.0816
% Peakarea
150
100
50
0
stage I lamina
stage I nodule
stage II lamina
stage II nodule
stage III lamina
stage III nodule
Tissues
Tissues
stage I lamina
stage I nodule
stage II lamina
stage II nodule
stage III lamina
stage III nodule

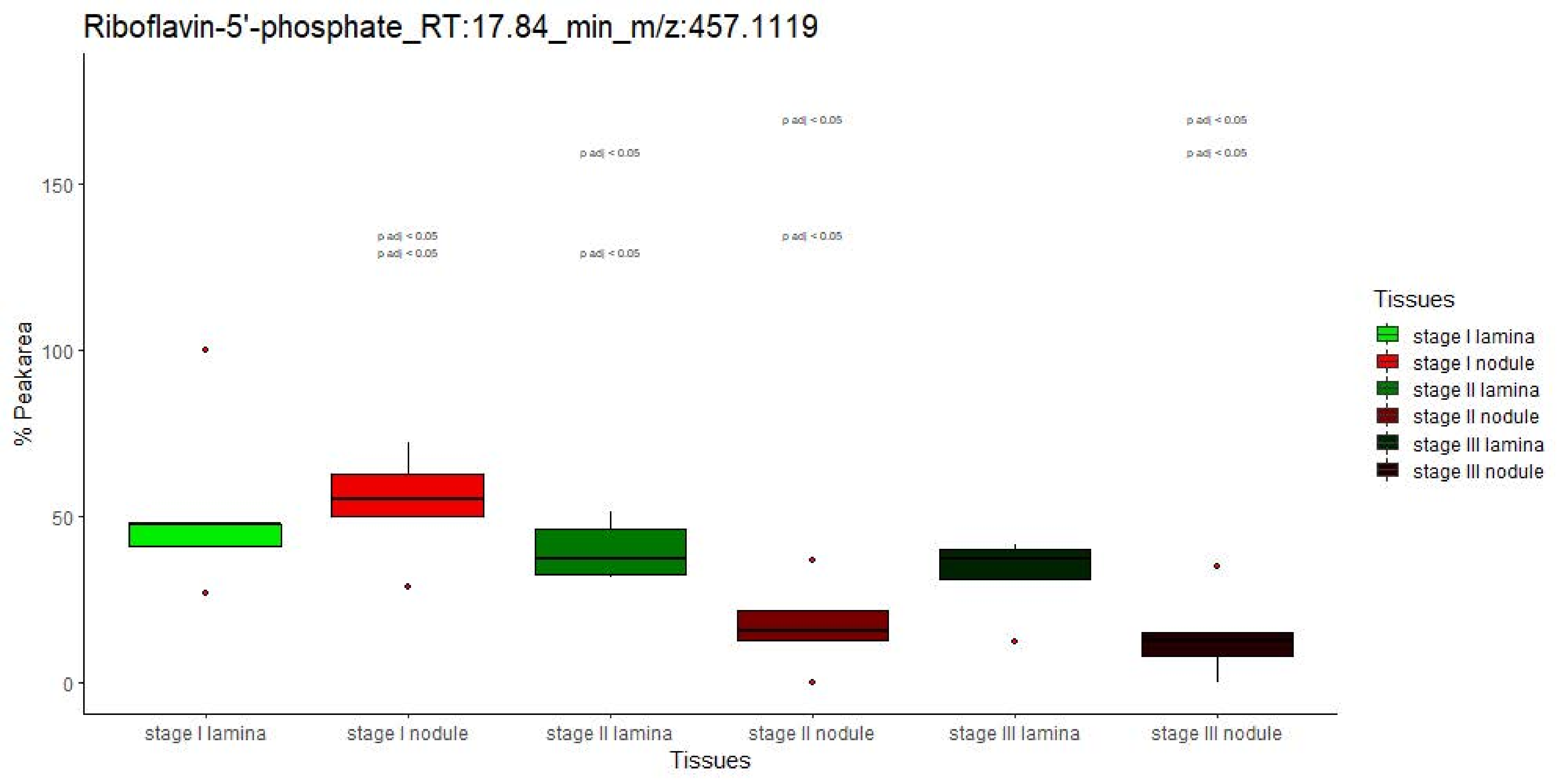

Riboflavin-5'-phosphate_RT:17.84_min_m/z:457.1119
% Peakarea
150
100
50
0
p adj < 0.05
stage I lamina
stage I nodule
stage II lamina
stage II nodule
stage III lamina
stage III nodule
Tissues

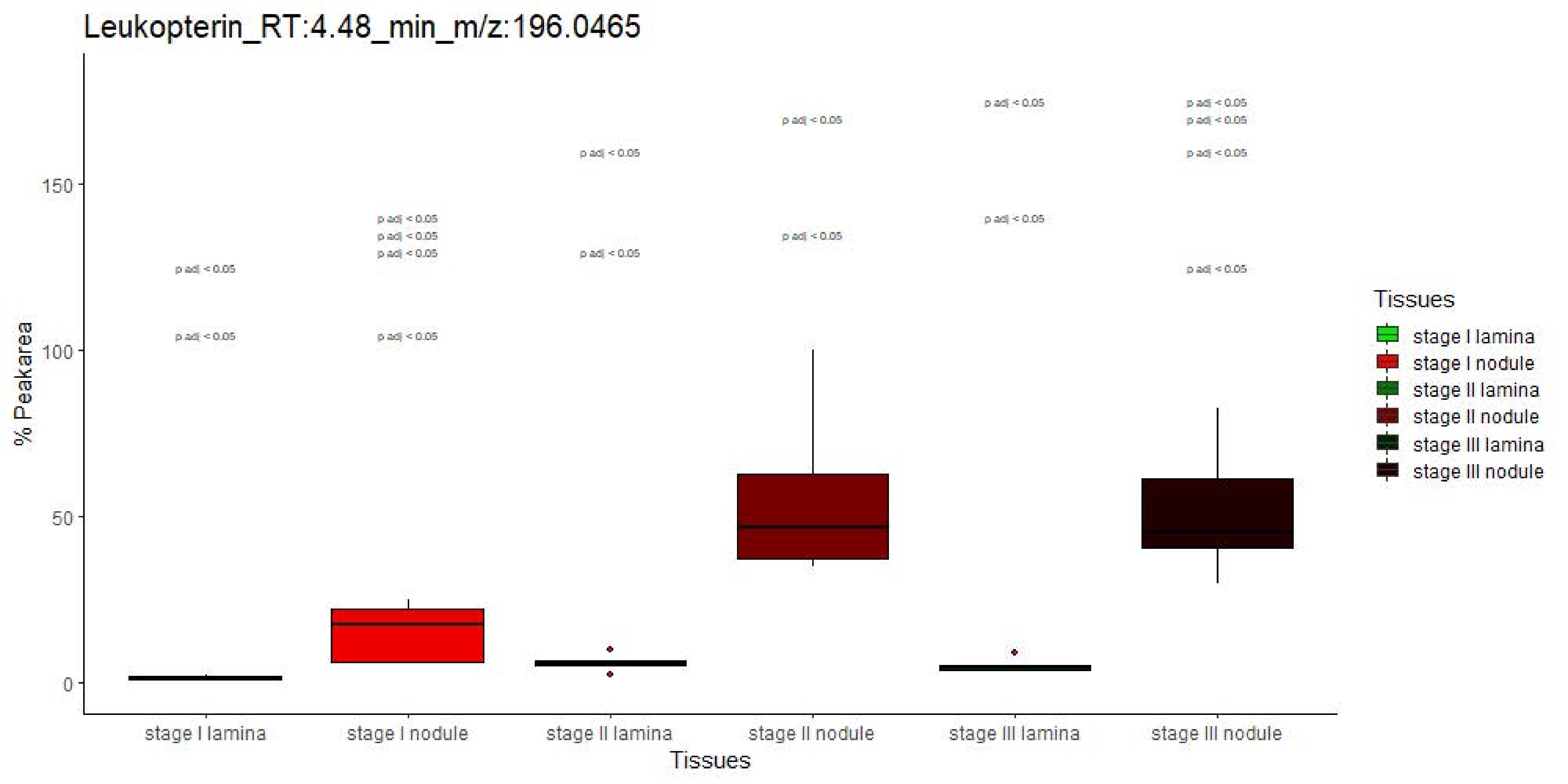

Leukopterin_RT:4.48_min_m/z:196.0465
% Peakarea
0
50
100
150
p adj < 0.05
stage I lamina
stage I nodule
stage II lamina
stage II nodule
stage III lamina
stage III nodule
Tissues
Tissues
stage I lamina
stage I nodule
stage II lamina
stage II nodule
stage III lamina
stage III nodule

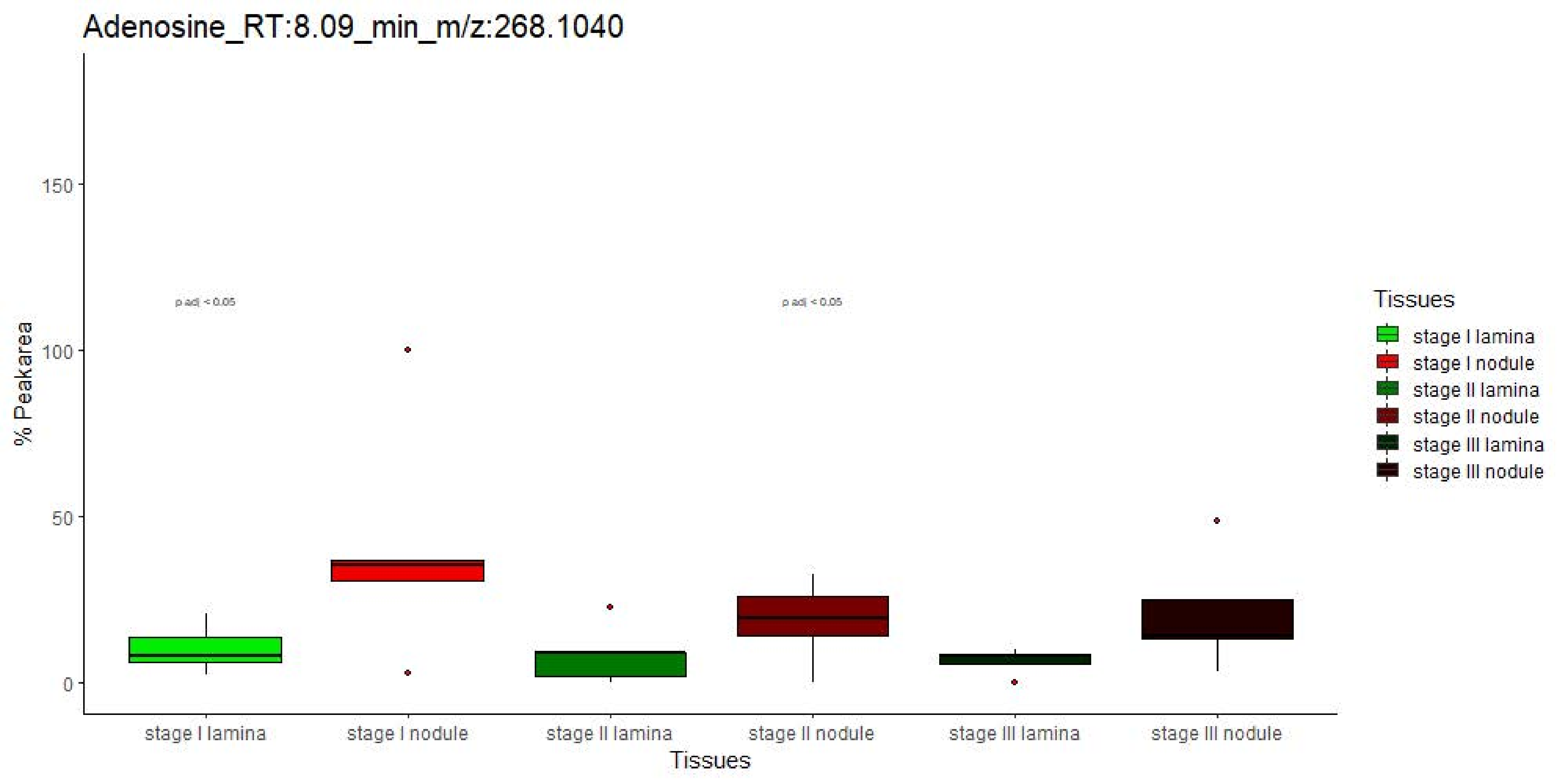
Adenosine_RT:8.09_min_m/z:268.1040
% Peakarea
150
100
50
0
p adj < 0.05
p adj < 0.05
stage I lamina
stage I nodule
stage II lamina
stage II nodule
stage III lamina
stage III nodule
Tissues
Tissues
stage I lamina
stage I nodule
stage II lamina
stage II nodule
stage III lamina
stage III nodule

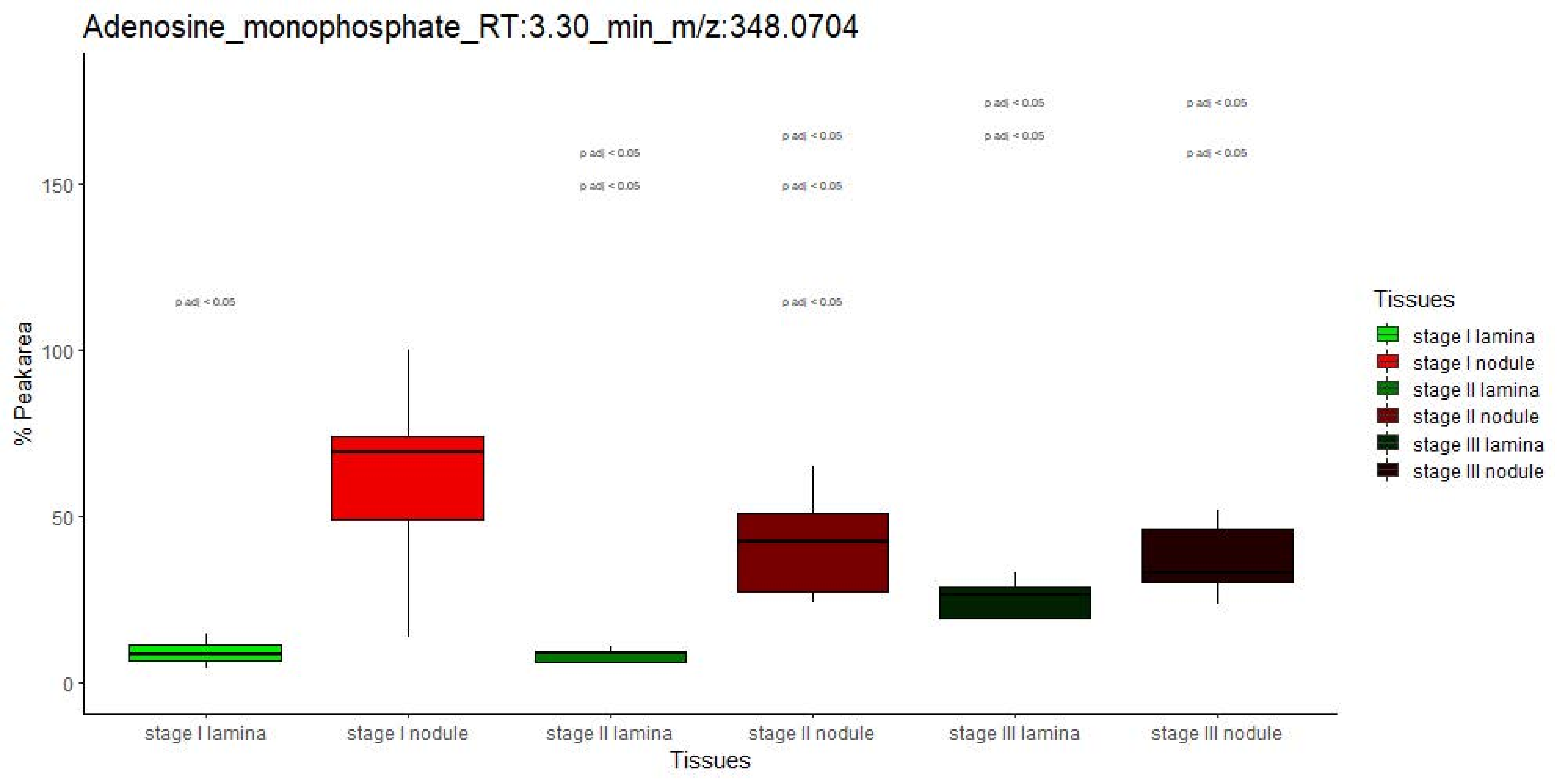

Adenosine_monophosphate_RT:3.30_min_m/z:348.0704
p adj < 0.05
p adj < 0.05
p adj < 0.05
p adj < 0.05
p adj < 0.05
p adj < 0.05
p adj < 0.05
p adj < 0.05
p adj < 0.05
p adj < 0.05
p adj < 0.05
150
100
50
0
% Peakarea
stage I lamina
stage I nodule
stage II lamina
stage II nodule
stage III lamina
stage III nodule
Tissues
Tissues
stage I lamina
stage I nodule
stage II lamina
stage II nodule
stage III lamina
stage III nodule

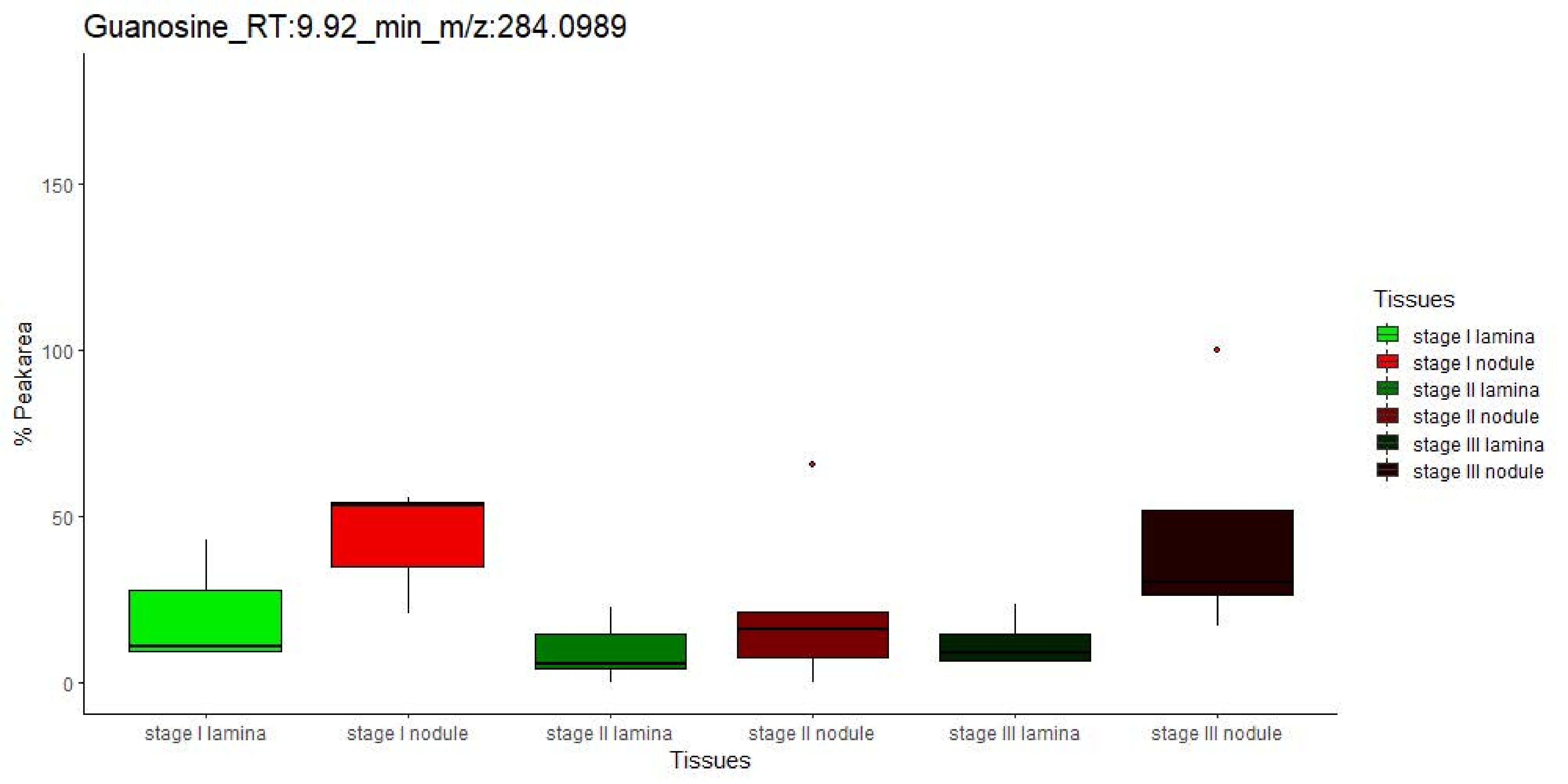
Guanosine_RT:9.92_min_m/z:284.0989
% Peakarea
150
100
50
0
stage I lamina
stage I nodule
stage II lamina
stage II nodule
stage III lamina
stage III nodule
Tissues
Tissues
stage I lamina
stage I nodule
stage II lamina
stage II nodule
stage III lamina
stage III nodule

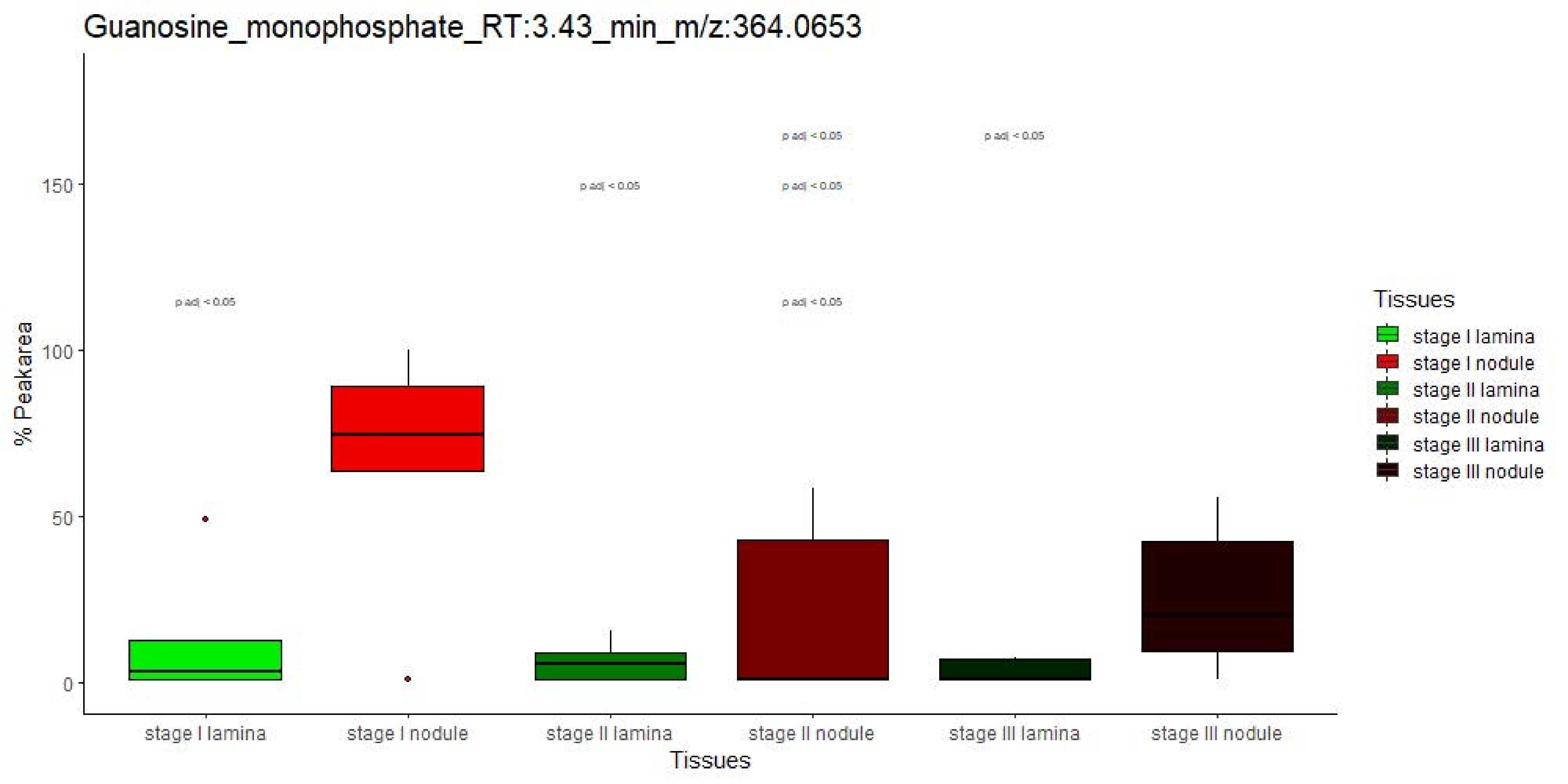

Guanosine_monophosphate_RT:3.43_min_m/z:364.0653
% Peakarea
150
100
50
0
p adj < 0.05
p adj < 0.05
p adj < 0.05
p adj < 0.05
p adj < 0.05
p adj < 0.05
stage I lamina
stage I nodule
stage II lamina
stage II nodule
stage III lamina
stage III nodule
Tissues
Tissues
stage I lamina
stage I nodule
stage II lamina
stage II nodule
stage III lamina
stage III nodule

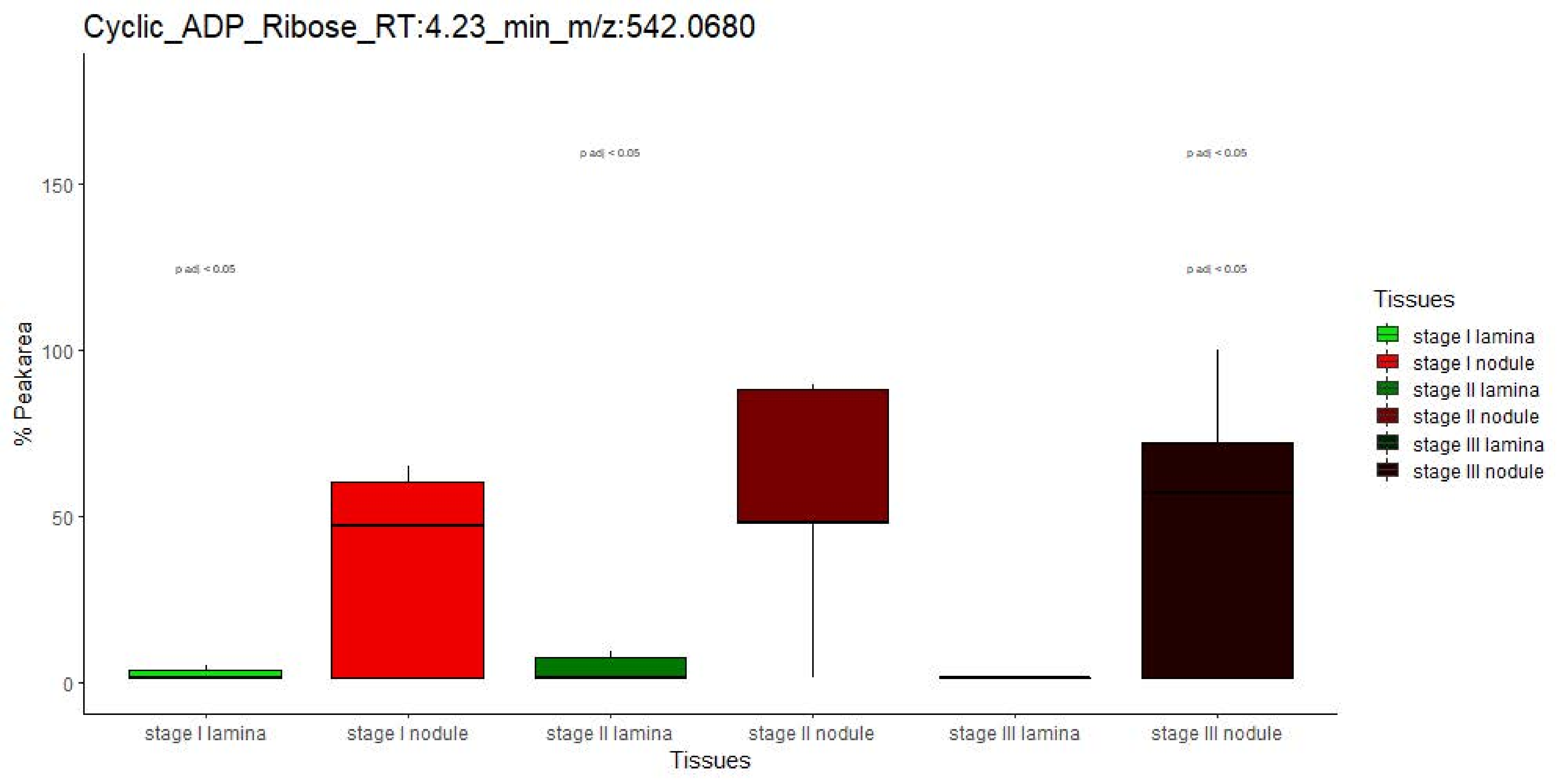
Cyclic_ADP_Ribose_RT:4.23_min_m/z:542.0680
% Peakarea
150
100
50
0
p adj < 0.05
p adj < 0.05
p adj < 0.05
p adj < 0.05
stage I lamina
stage I nodule
stage II lamina
stage II nodule
stage III lamina
stage III nodule
Tissues
Tissues
stage I lamina
stage I nodule
stage II lamina
stage II nodule
stage III lamina
stage III nodule

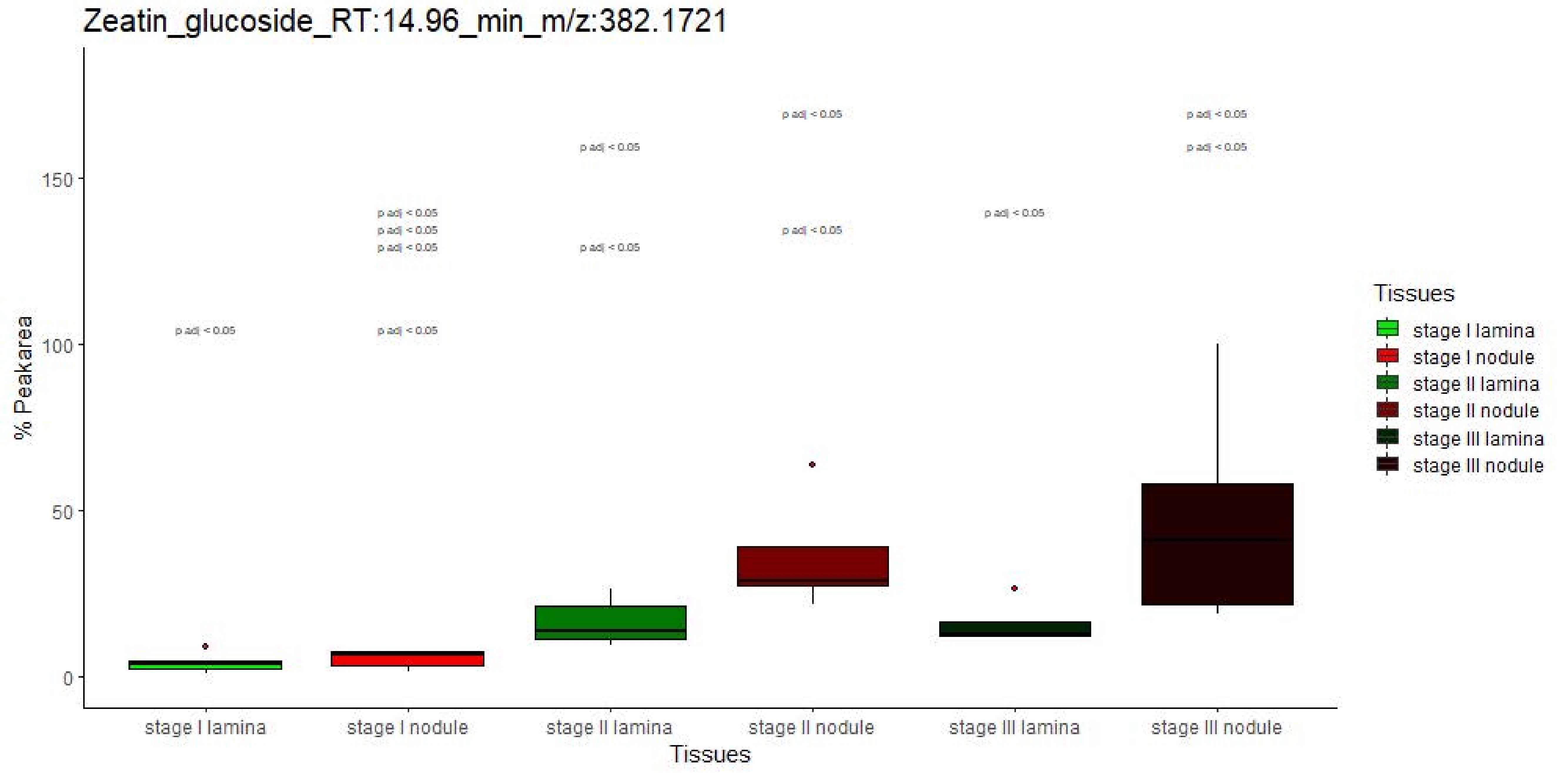

Zeatin_glucoside_RT:14.96_min_m/z:382.1721
% Peakarea
150
100
50
0
p adj < 0.05
stage I lamina
stage I nodule
stage II lamina
stage II nodule
stage III lamina
stage III nodule
Tissues

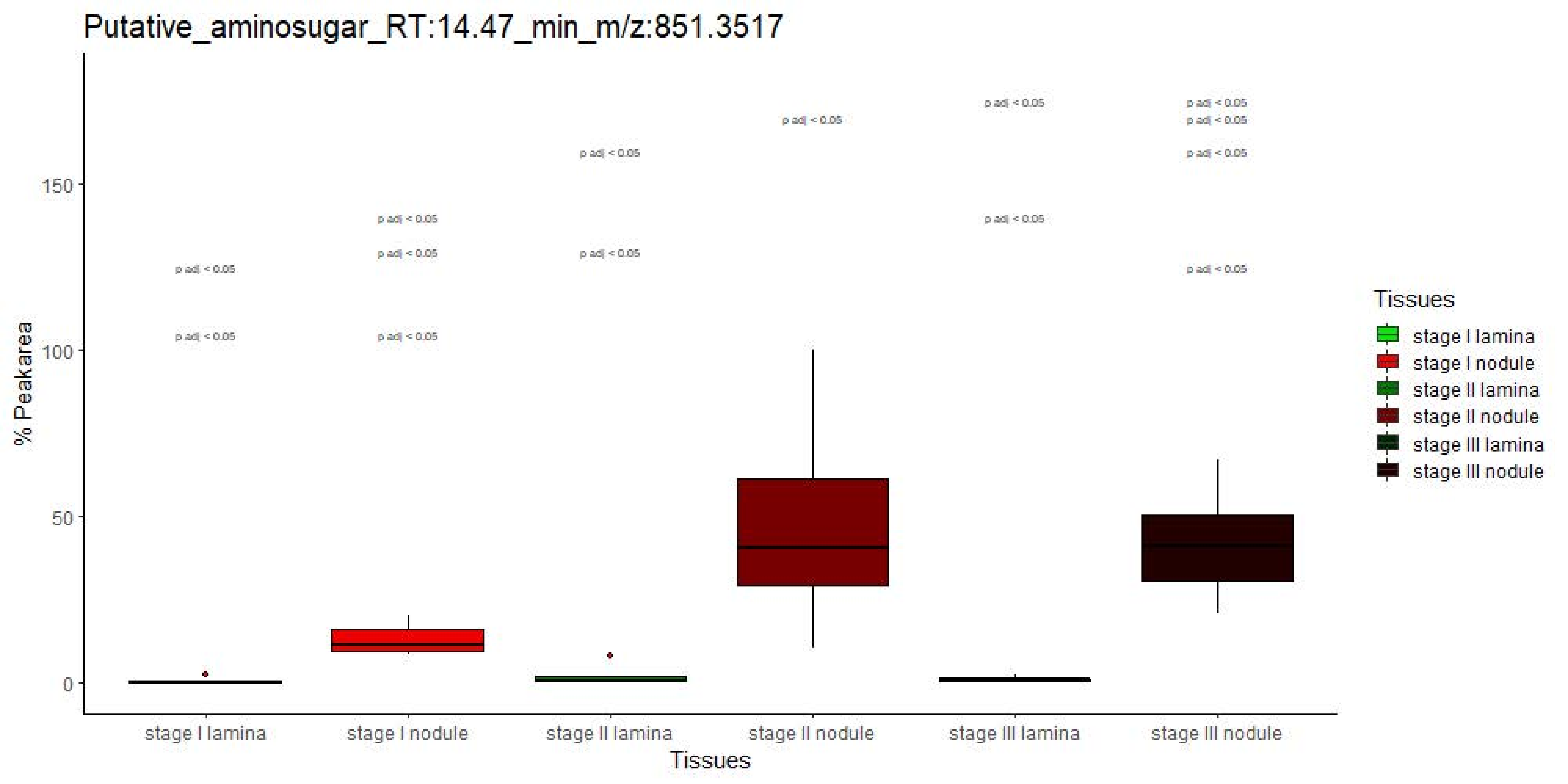

Putative_aminosugar_RT:14.47_min_m/z:851.3517
% Peakarea
0
50
100
150
p adj < 0.05
Tissues
stage I lamina
stage I nodule
stage II lamina
stage II nodule
stage III lamina
stage III nodule

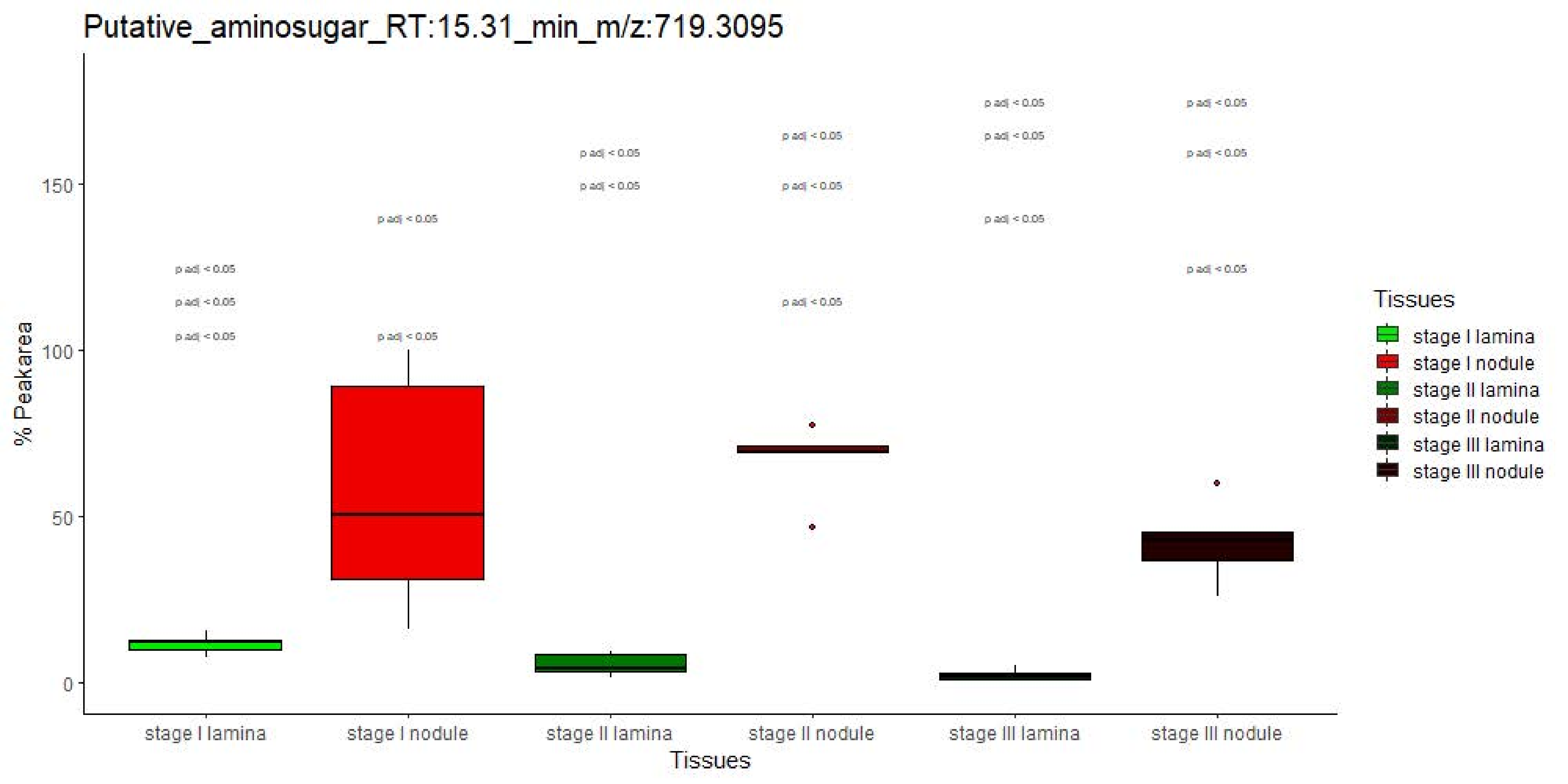

Putative_aminosugar_RT:15.31_min_m/z:719.3095
% Peakarea
0
50
100
150
p adj < 0.05
stage I lamina
stage I nodule
stage II lamina
stage II nodule
stage III lamina
stage III nodule
Tissues

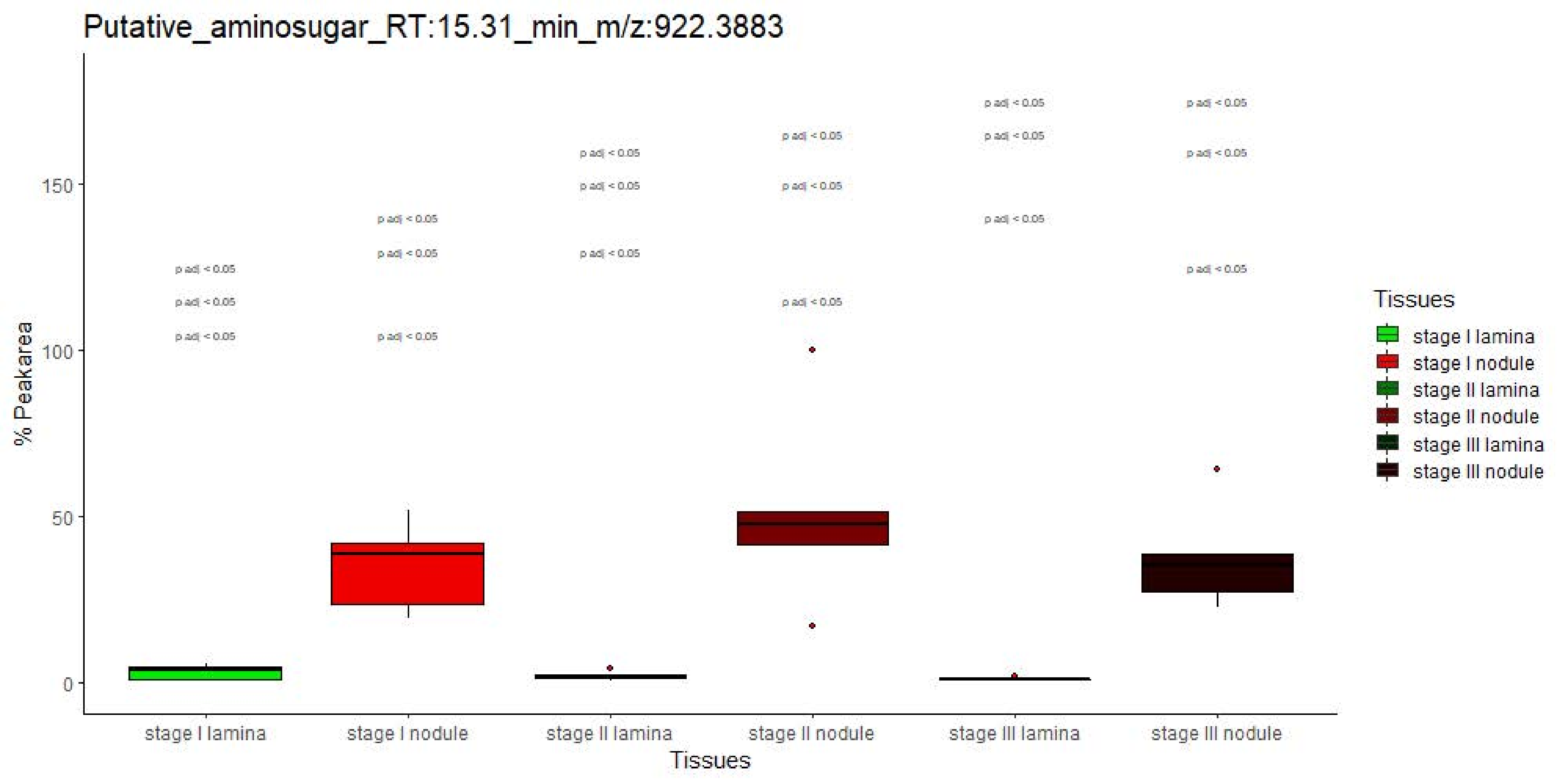

Putative_aminosugar_RT:15.31_min_m/z:922.3883
% Peakarea
150
100
50
0
p adj < 0.05
stage I lamina
stage I nodule
stage II lamina
stage II nodule
stage III lamina
stage III nodule
Tissues
Tissues
stage I lamina
stage I nodule
stage II lamina
stage II nodule
stage III lamina
stage III nodule

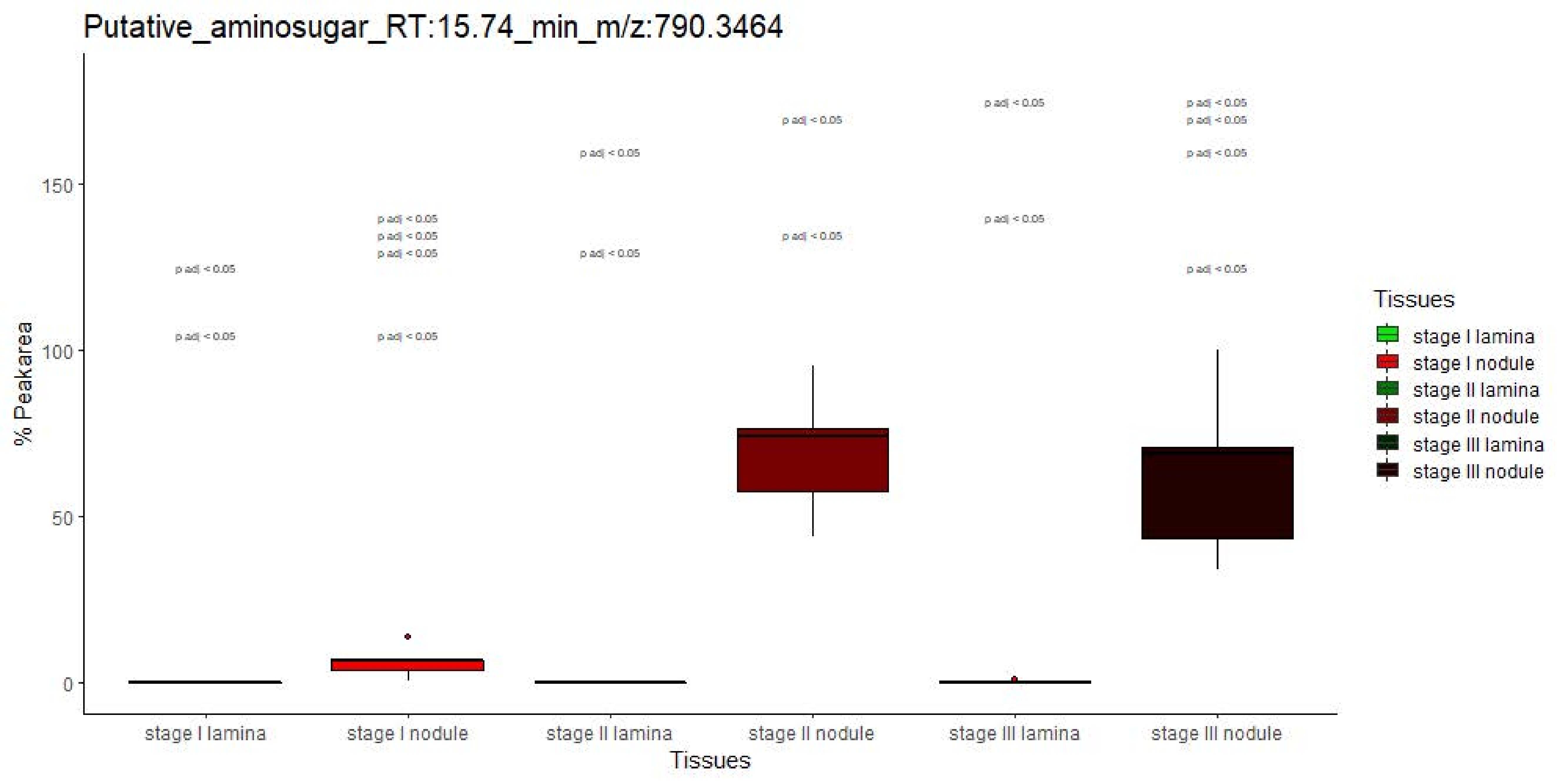

Putative_aminosugar_RT:15.74_min_m/z:790.3464
% Peakarea
0
50
100
150
p adj < 0.05
Tissues
stage I lamina
stage I nodule
stage II lamina
stage II nodule
stage III lamina
stage III nodule

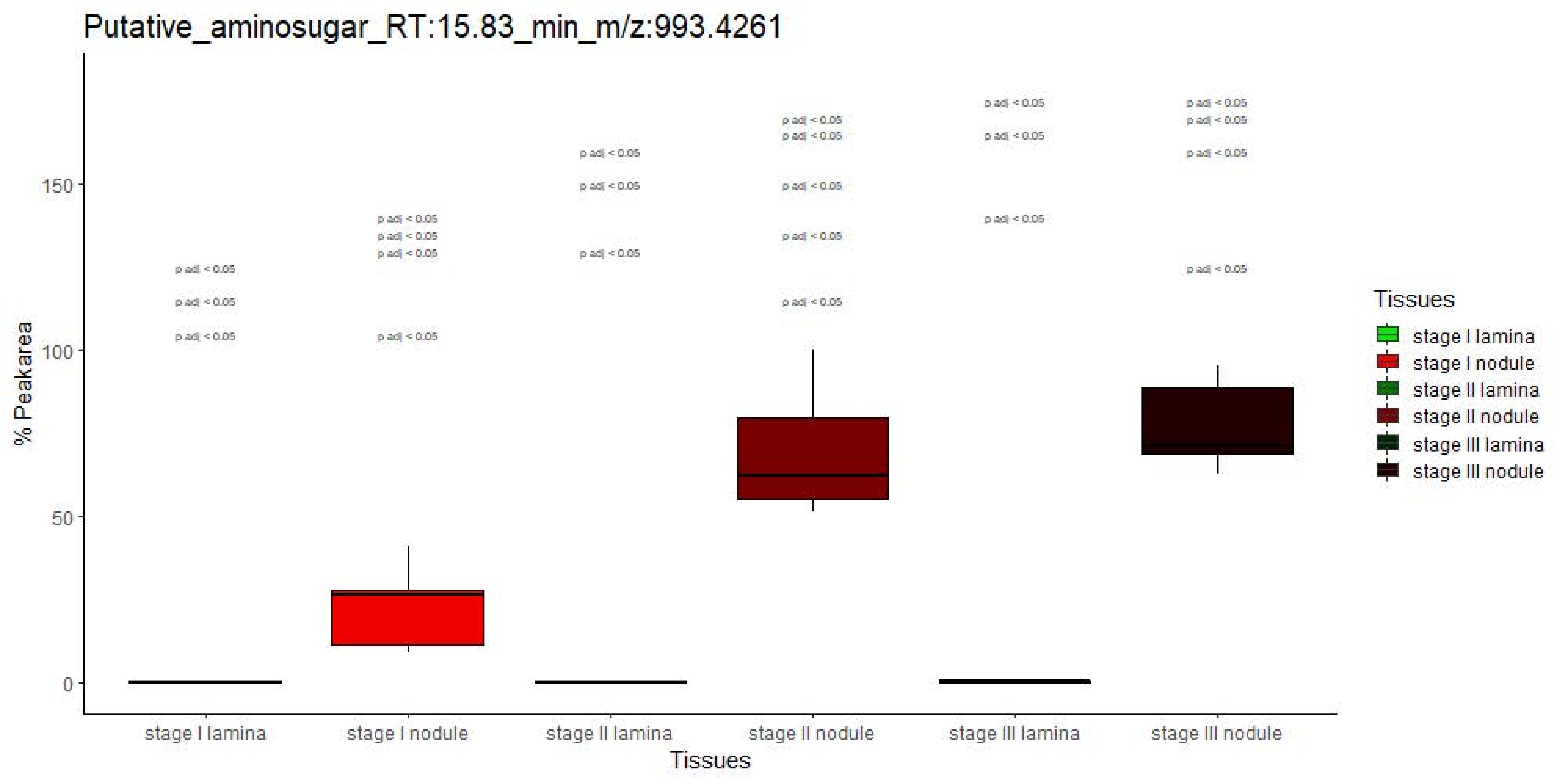

Putative_aminosugar_RT:15.83_min_m/z:993.4261
% Peakarea
Tissues
stage I lamina
stage I nodule
stage II lamina
stage II nodule
stage III lamina
stage III nodule
p adj < 0.05
0
50
100
150

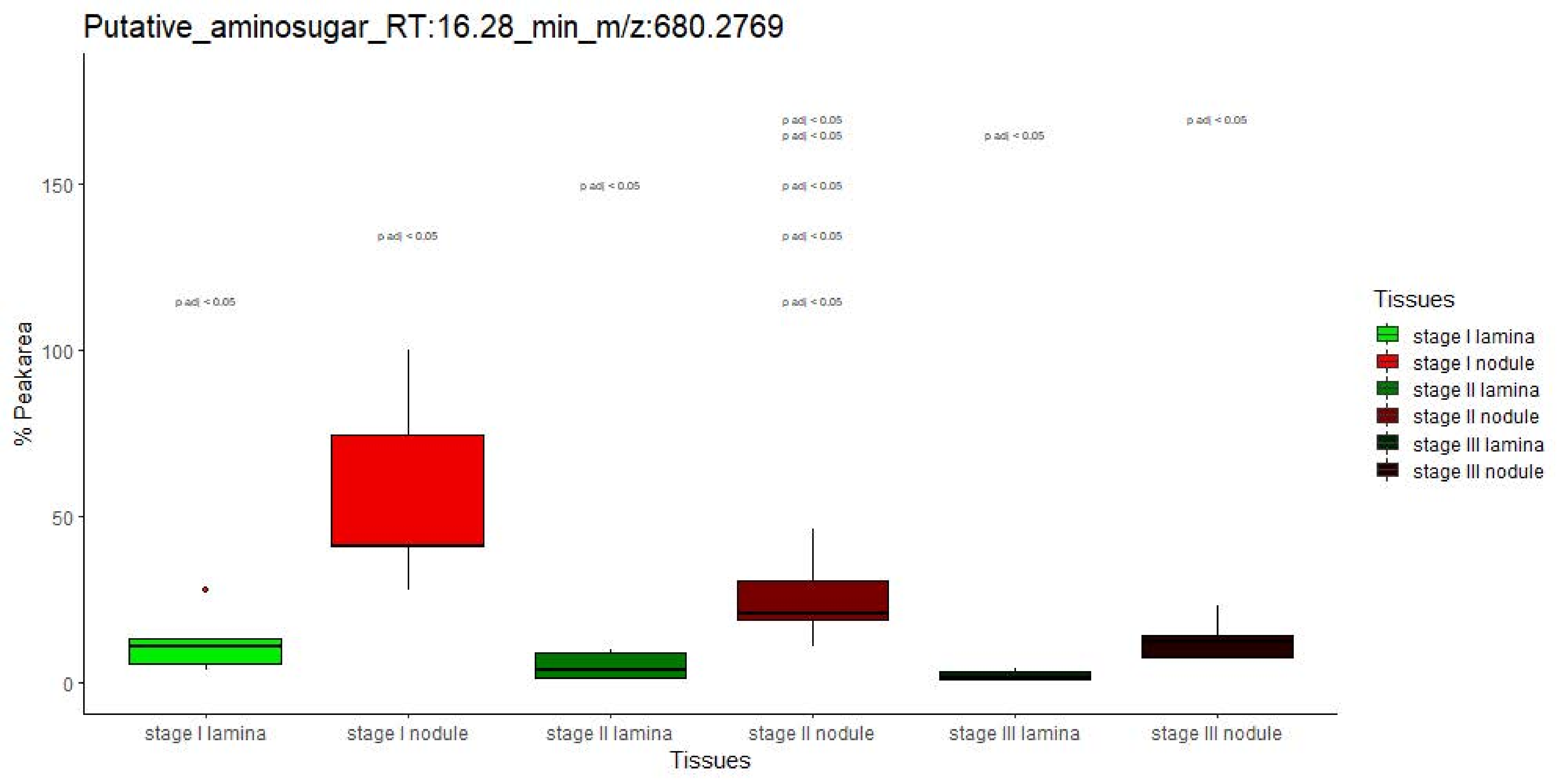

Putative_aminosugar_RT:16.28_min_m/z:680.2769
% Peakarea
0
50
100
150
p adj < 0.05
Tissues
stage I lamina
stage I nodule
stage II lamina
stage II nodule
stage III lamina
stage III nodule

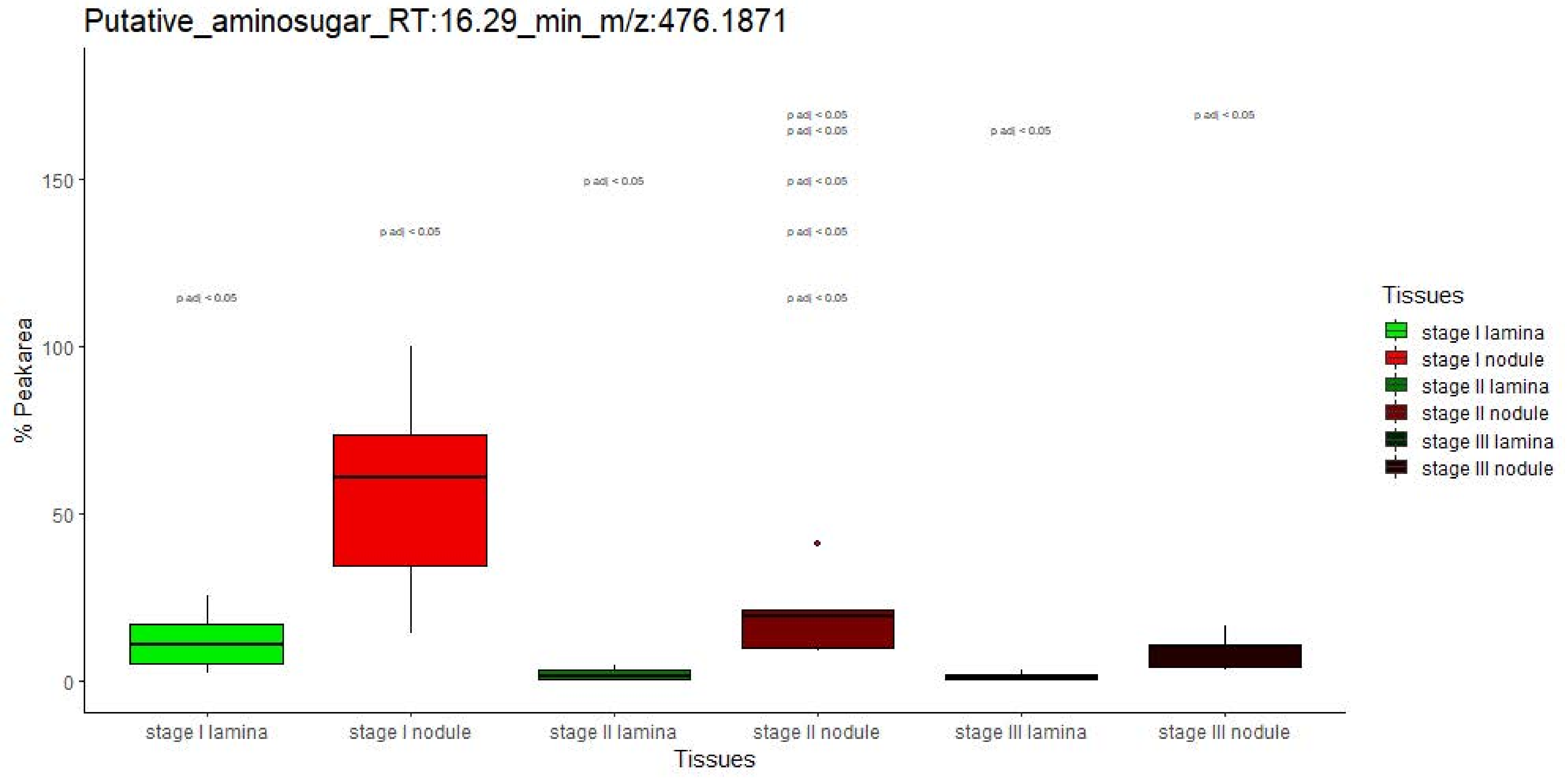

Putative_aminosugar_RT:16.29_min_m/z:476.1871
% Peakarea
0
50
100
150
p adj < 0.05
stage I lamina
stage I nodule
stage II lamina
stage II nodule
stage III lamina
stage III nodule
Tissues

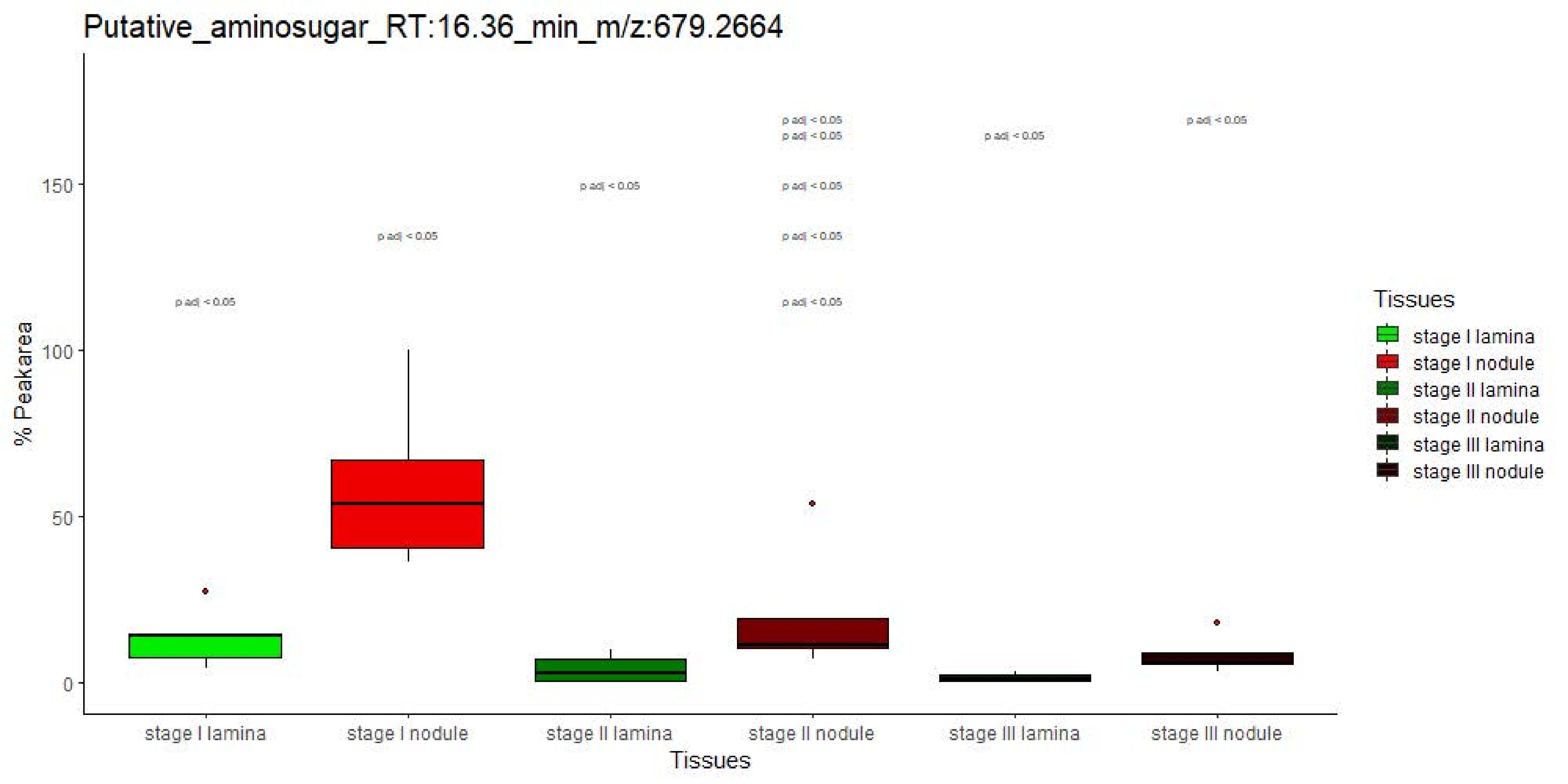
Putative_aminosugar_RT:16.36_min_m/z:679.2664
% Peakarea
0
50
100
150
p adj < 0.05
stage I lamina
stage I nodule
stage II lamina
stage II nodule
stage III lamina
stage III nodule
Tissues

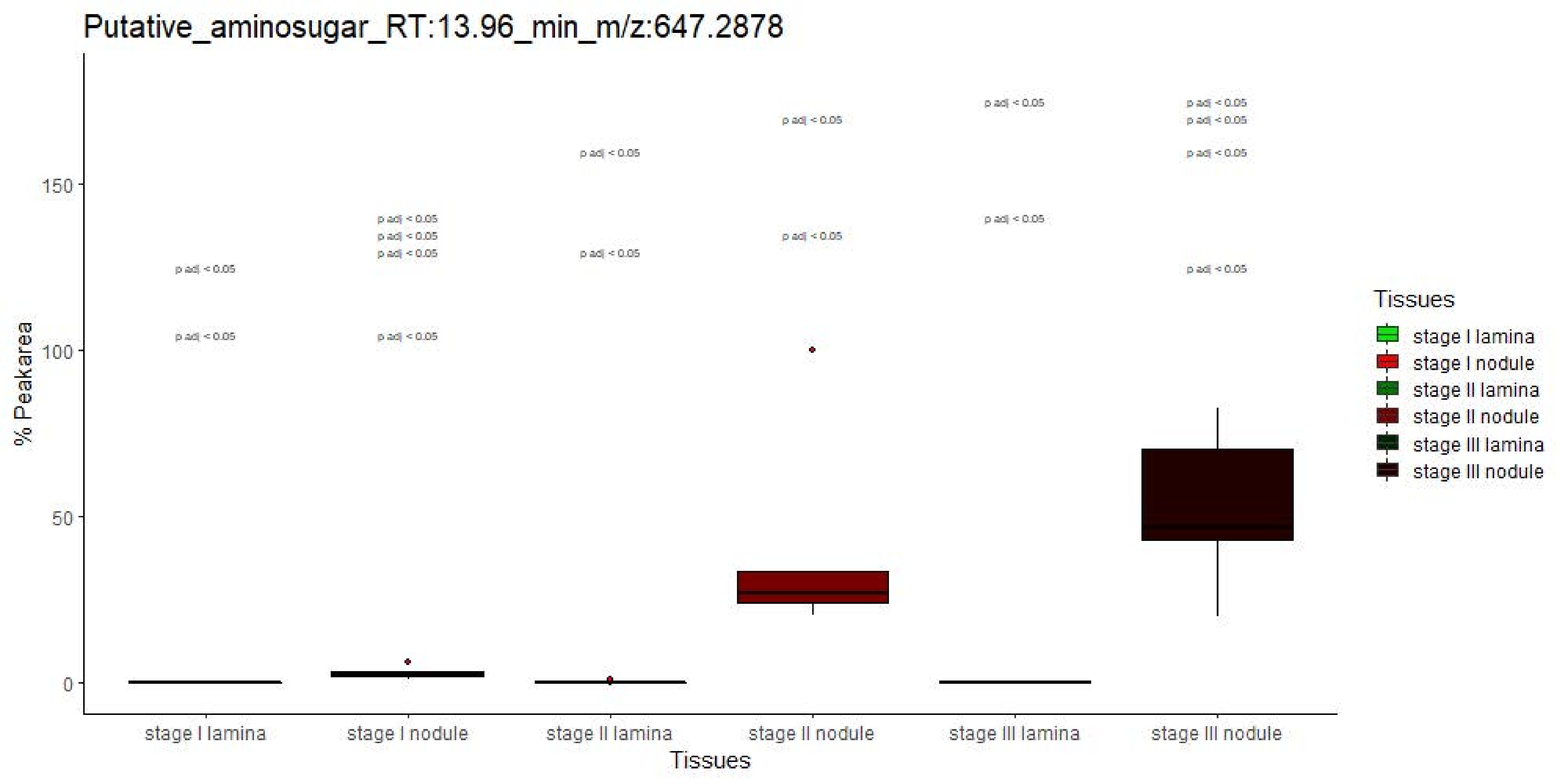

Putative_aminosugar_RT:13.96_min_m/z:647.2878
% Peakarea
150
100
50
0
p adj < 0.05
stage I lamina
stage I nodule
stage II lamina
stage II nodule
stage III lamina
stage III nodule
Tissues

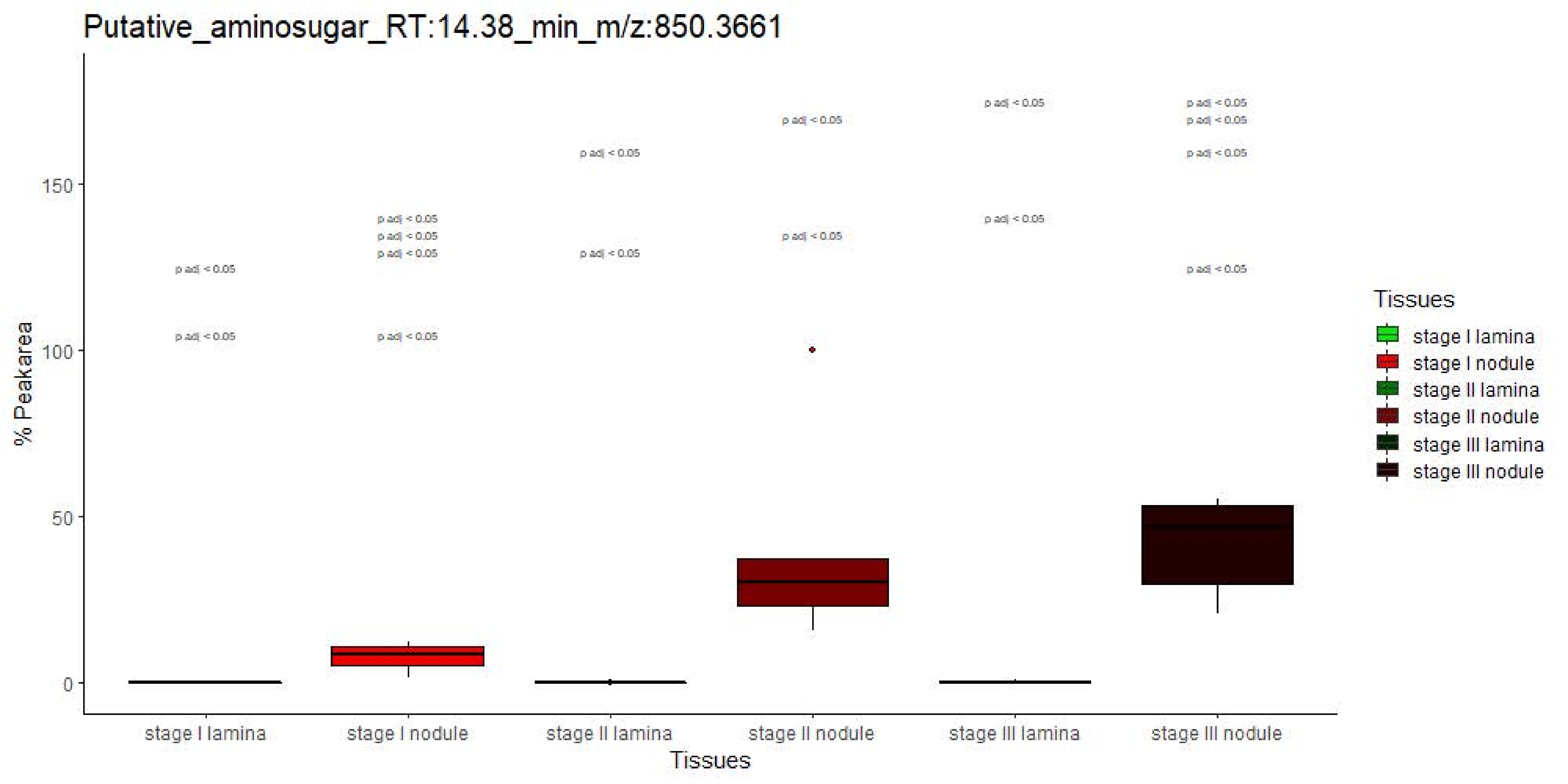

Putative_aminosugar_RT:14.38_min_m/z:850.3661
% Peakarea
Tissues
0
50
100
150
p adj < 0.05
stage I lamina
stage I nodule
stage II lamina
stage II nodule
stage III lamina
stage III nodule

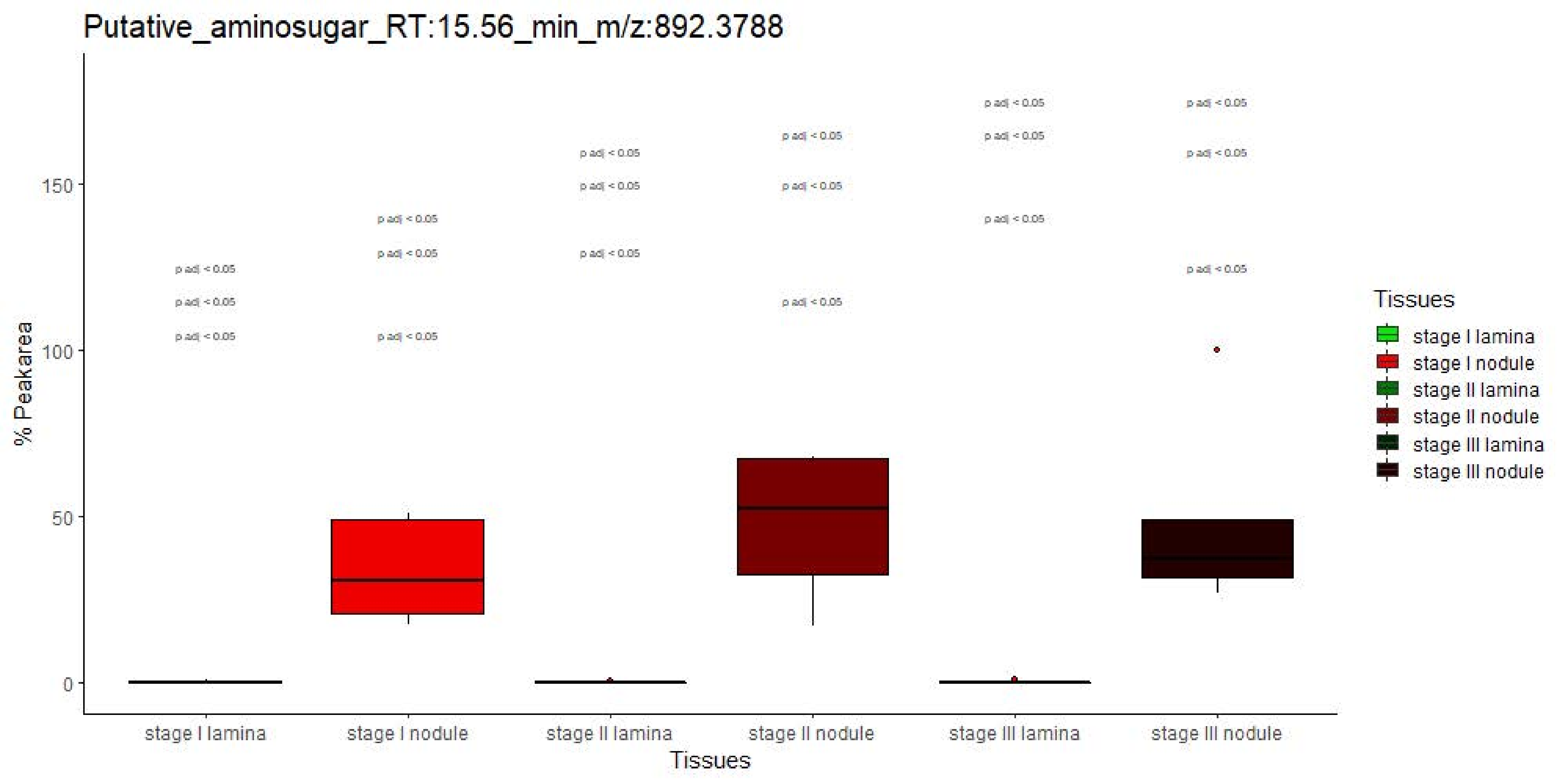

Putative_aminosugar_RT:15.56_min_m/z:892.3788
% Peakarea
Tissues
stage I lamina
stage I nodule
stage II lamina
stage II nodule
stage III lamina
stage III nodule
p adj < 0.05
0
50
100
150

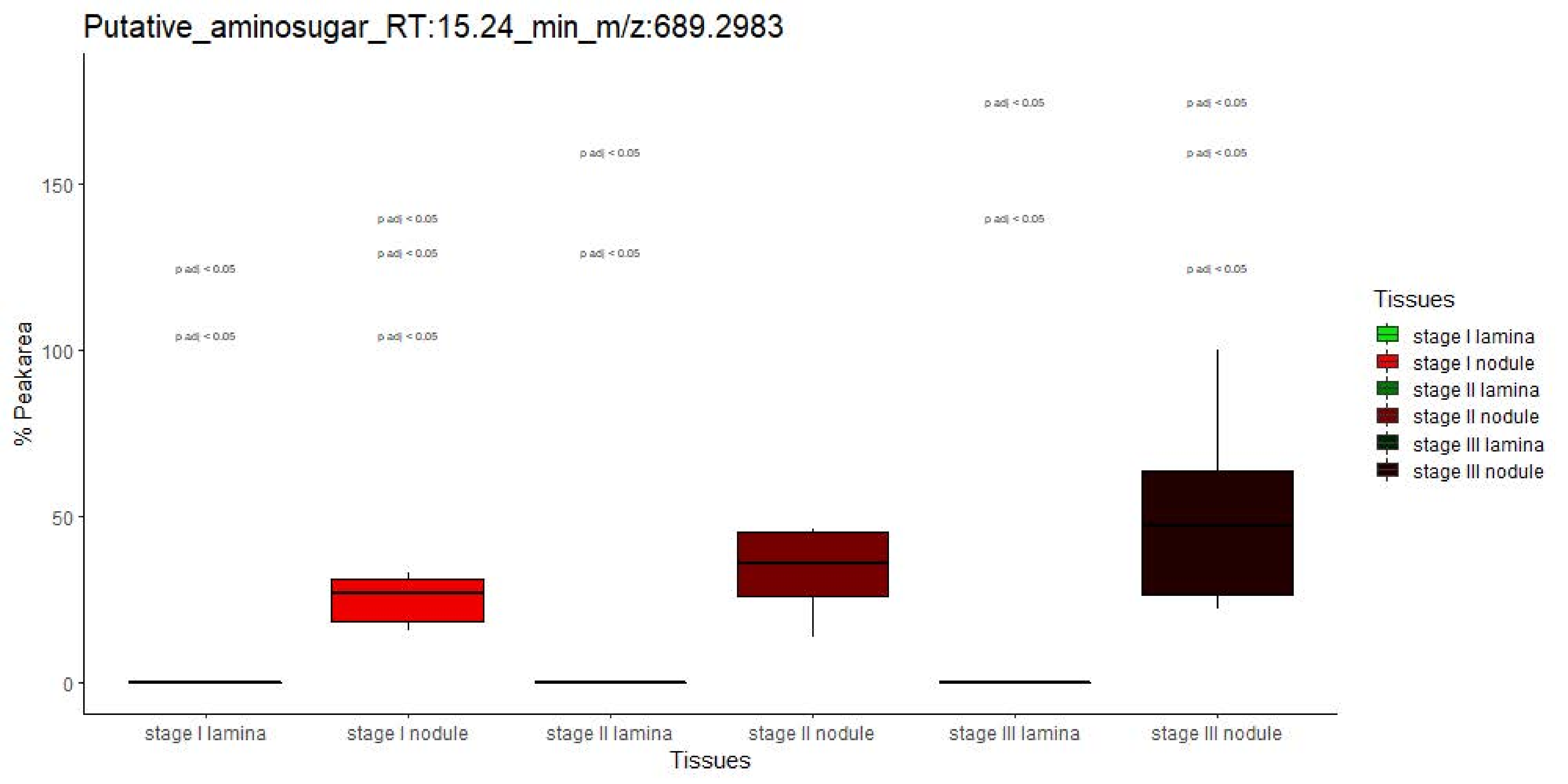

Putative_aminosugar_RT:15.24_min_m/z:689.2983
% Peakarea
0
50
100
150
p adj < 0.05
Tissues
stage I lamina
stage I nodule
stage II lamina
stage II nodule
stage III lamina
stage III nodule

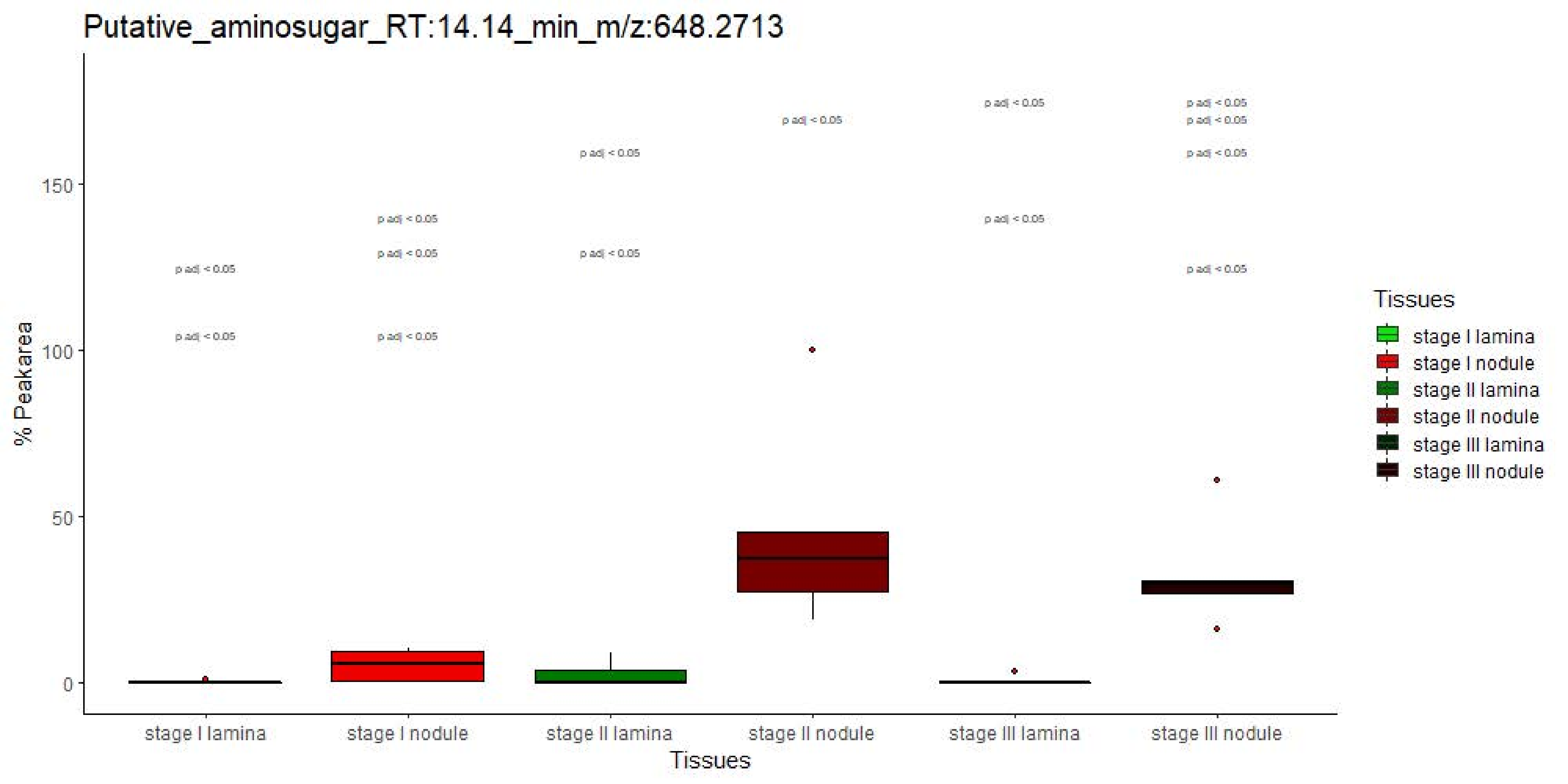
Putative_aminosugar_RT:14.14_min_m/z:648.2713
% Peakarea
0
50
100
150
p adj < 0.05
Tissues
stage I lamina
stage I nodule
stage II lamina
stage II nodule
stage III lamina
stage III nodule
Tissues
stage I lamina
stage I nodule
stage II lamina
stage II nodule
stage III lamina
stage III nodule

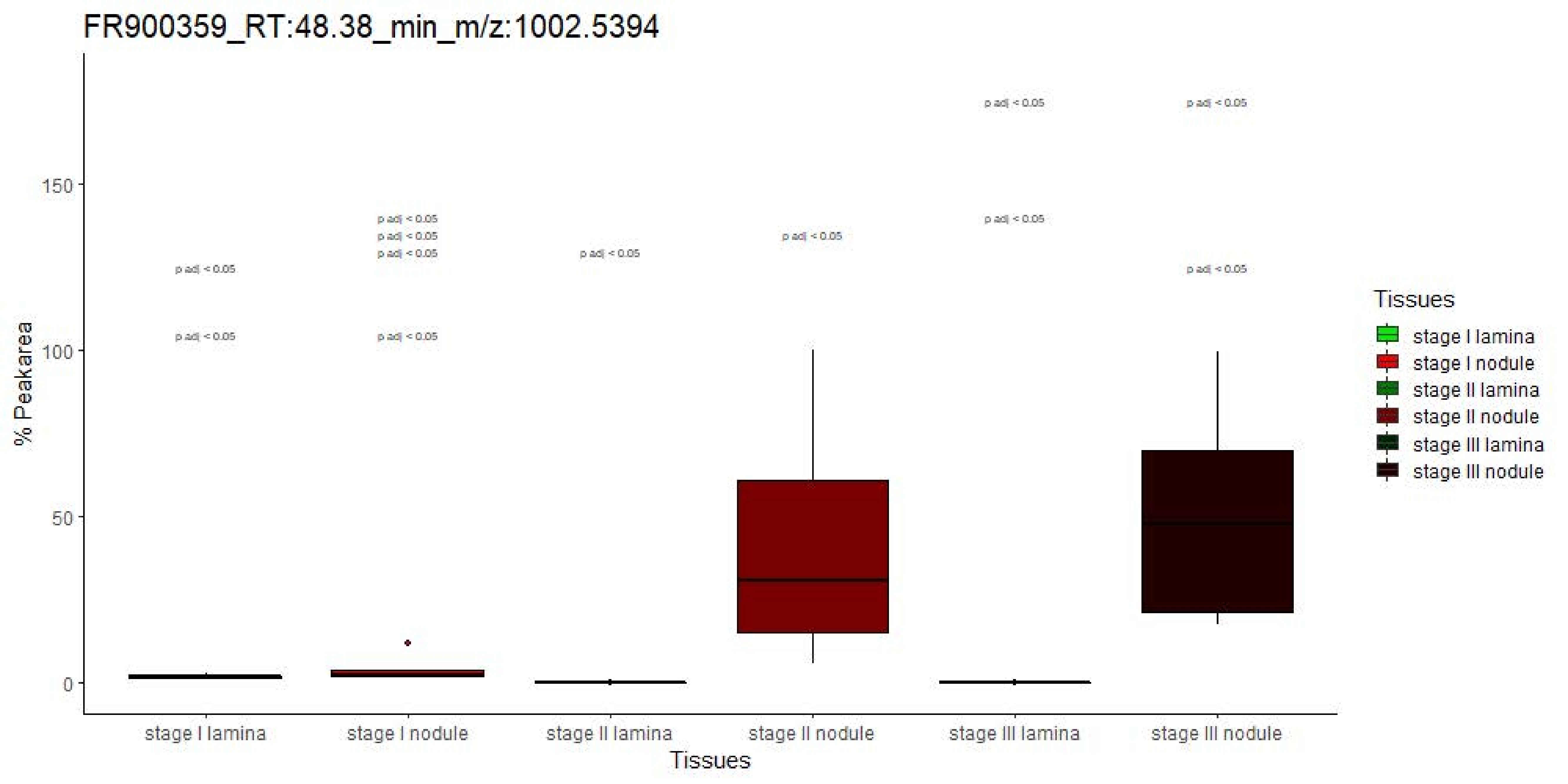
FR900359_RT:48.38_min_m/z:1002.5394
% Peakarea
150
100
50
0
p adj < 0.05
stage I lamina
stage I nodule
stage II lamina
stage II nodule
stage III lamina
stage III nodule
Tissues
Tissues
stage I lamina
stage I nodule
stage II lamina
stage II nodule
stage III lamina
stage III nodule

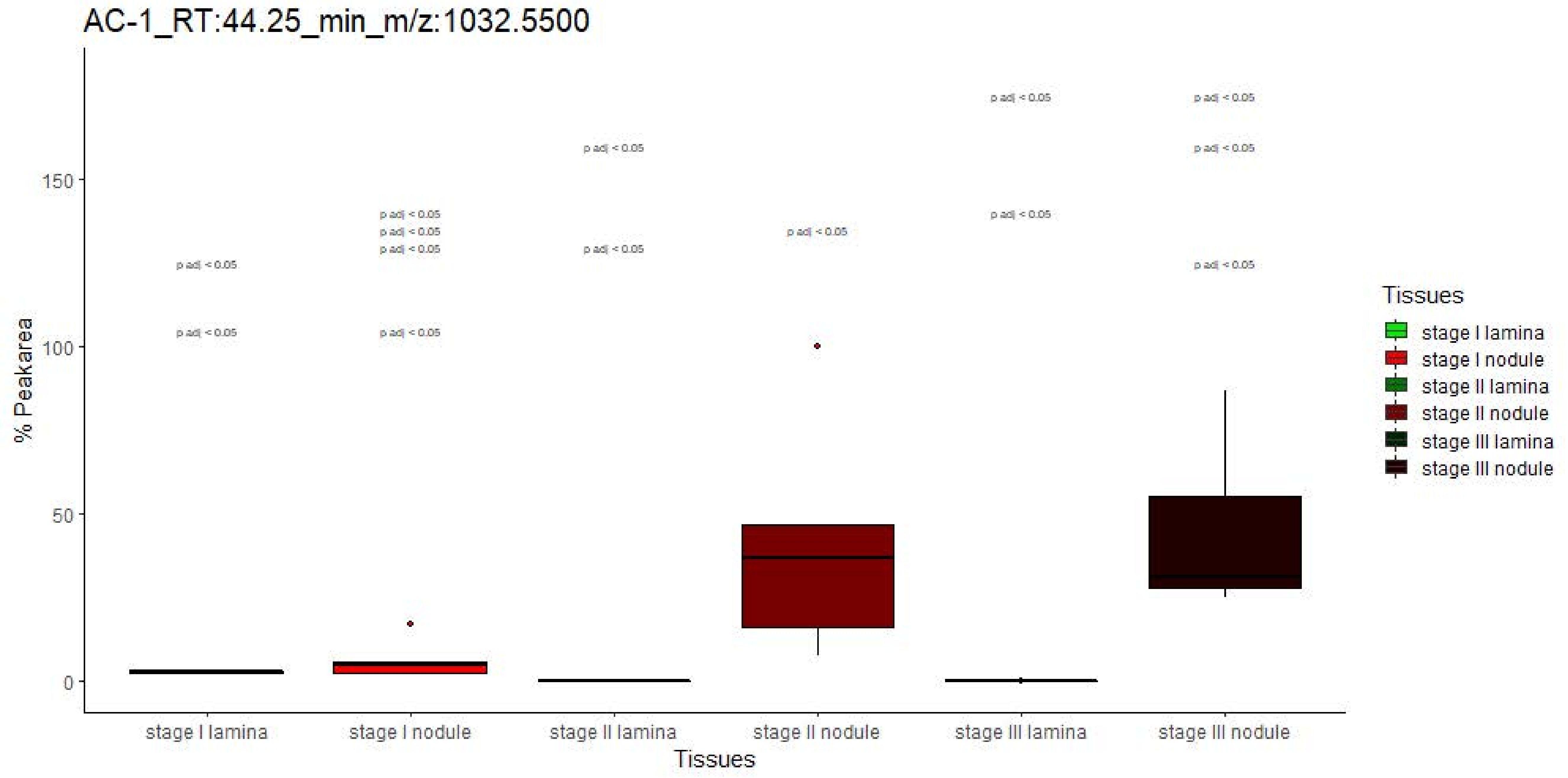

AC-1_RT:44.25_min_m/z:1032.5500
% Peakarea
0
50
100
150
p adj < 0.05
Tissues
stage I lamina
stage I nodule
stage II lamina
stage II nodule
stage III lamina
stage III nodule

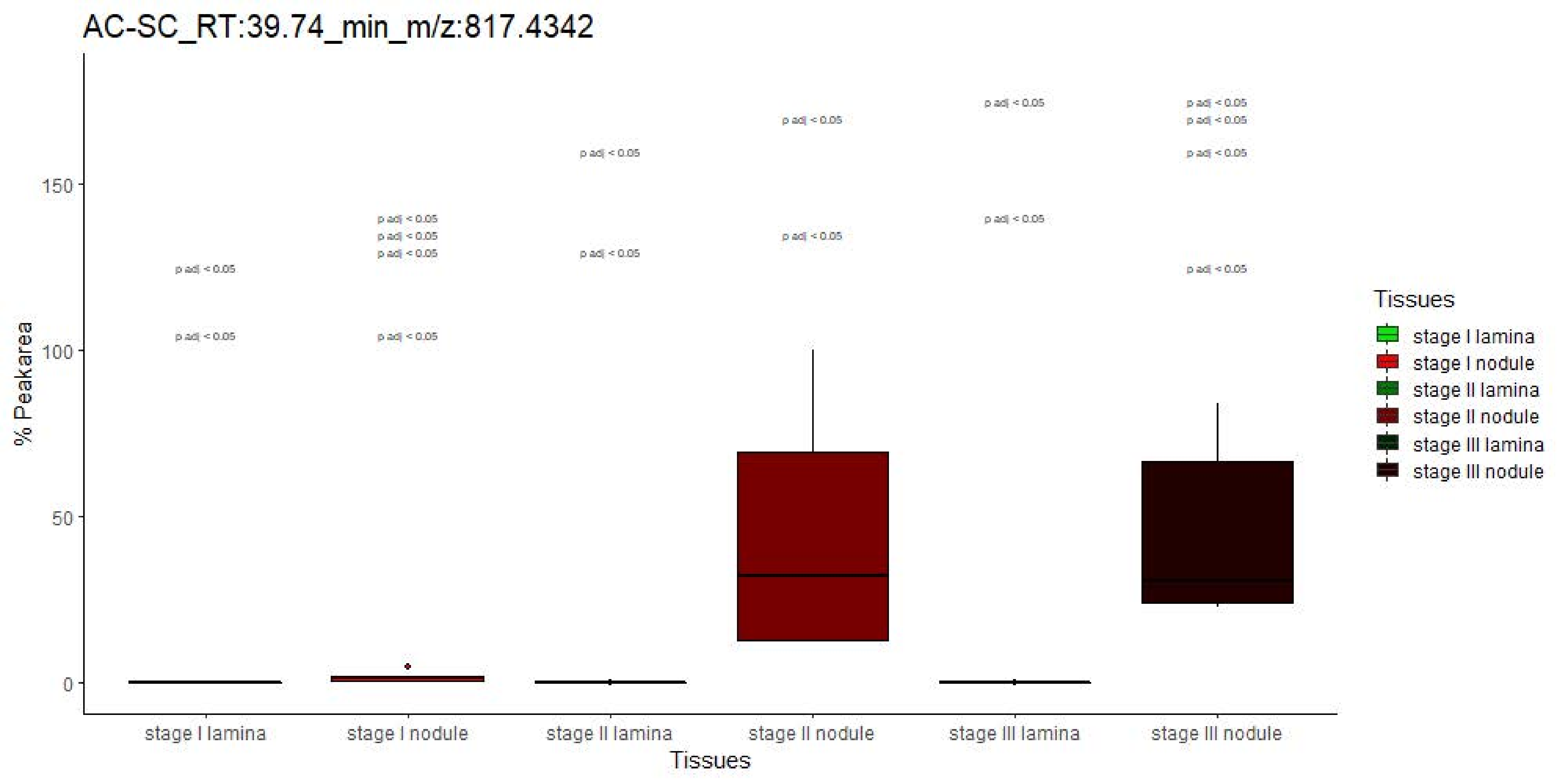
AC-SC_RT:39.74_min_m/z:817.4342
% Peakarea
0
50
100
150
p adj < 0.05
stage I lamina
stage I nodule
stage II lamina
stage II nodule
stage III lamina
stage III nodule
Tissues

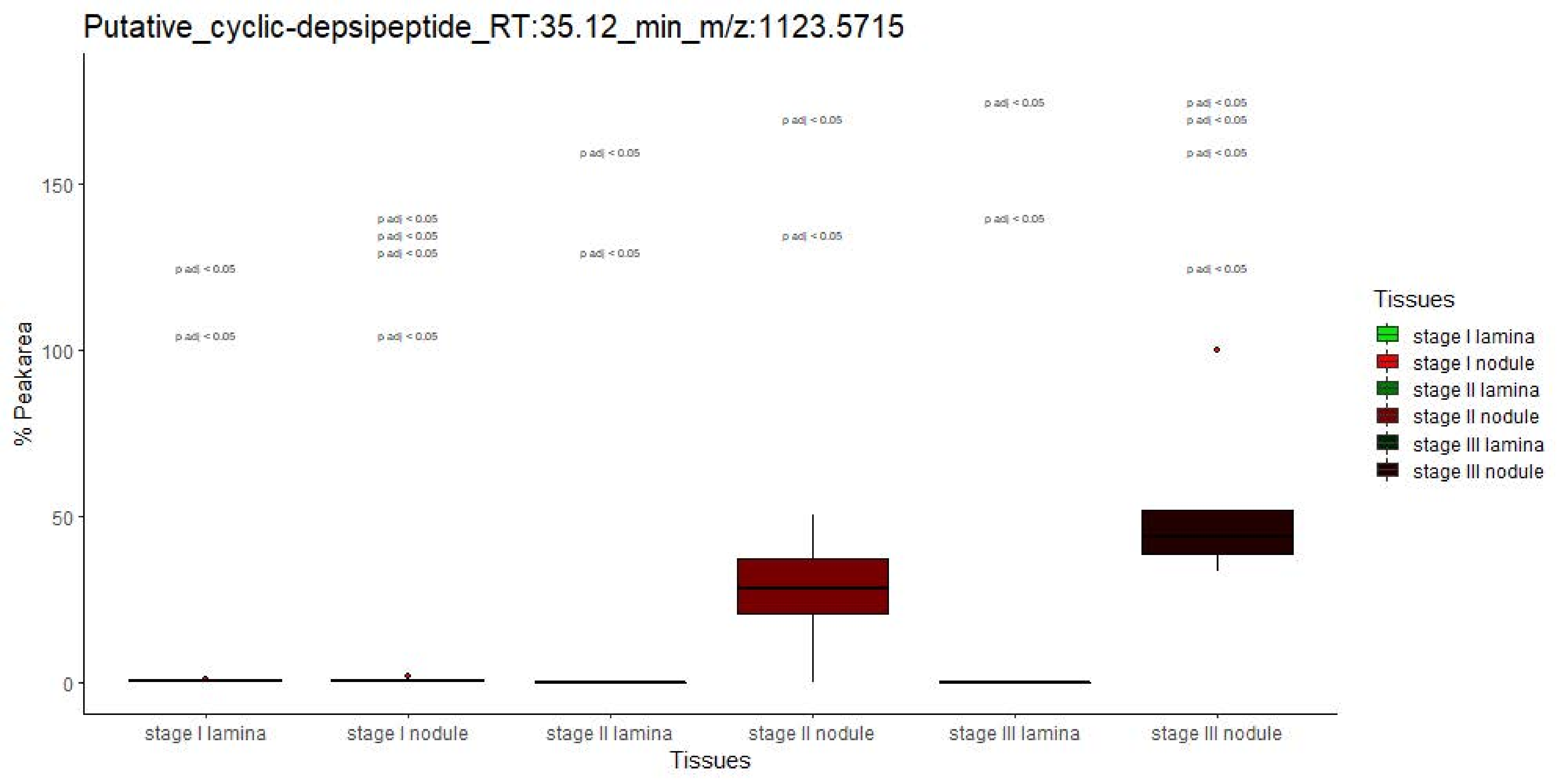

Putative_cyclic-depsipeptide_RT:35.12_min_m/z:1123.5715
% Peakarea
150
100
50
0
p adj < 0.05
stage I lamina
stage I nodule
stage II lamina
stage II nodule
stage III lamina
stage III nodule
Tissues
Tissues
stage I lamina
stage I nodule
stage II lamina
stage II nodule
stage III lamina
stage III nodule

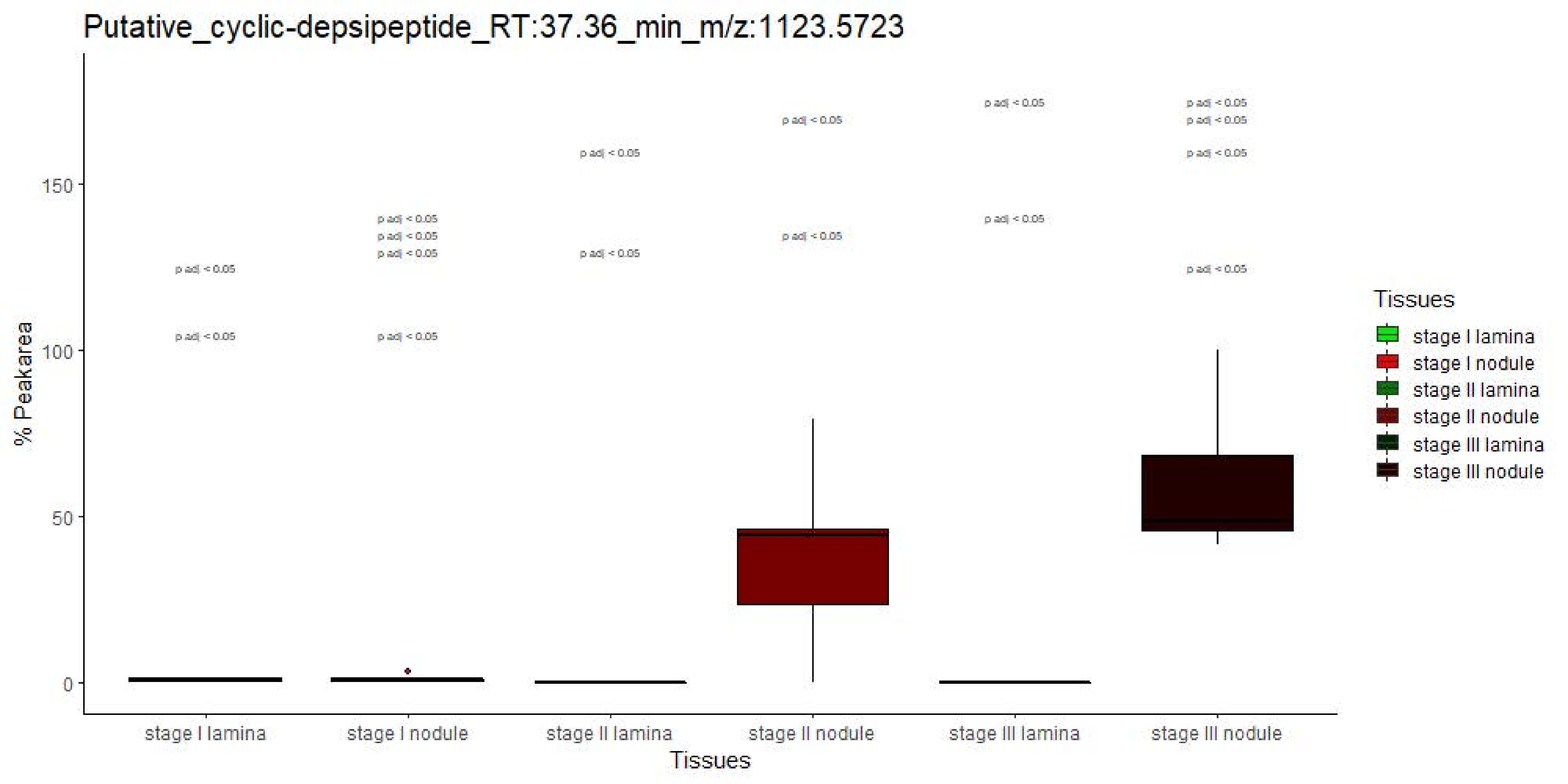
Putative_cyclic-depsipeptide_RT:37.36_min_m/z:1123.5723
% Peakarea
0
50
100
150
p adj < 0.05
Tissues
stage I lamina
stage I nodule
stage II lamina
stage II nodule
stage III lamina
stage III nodule

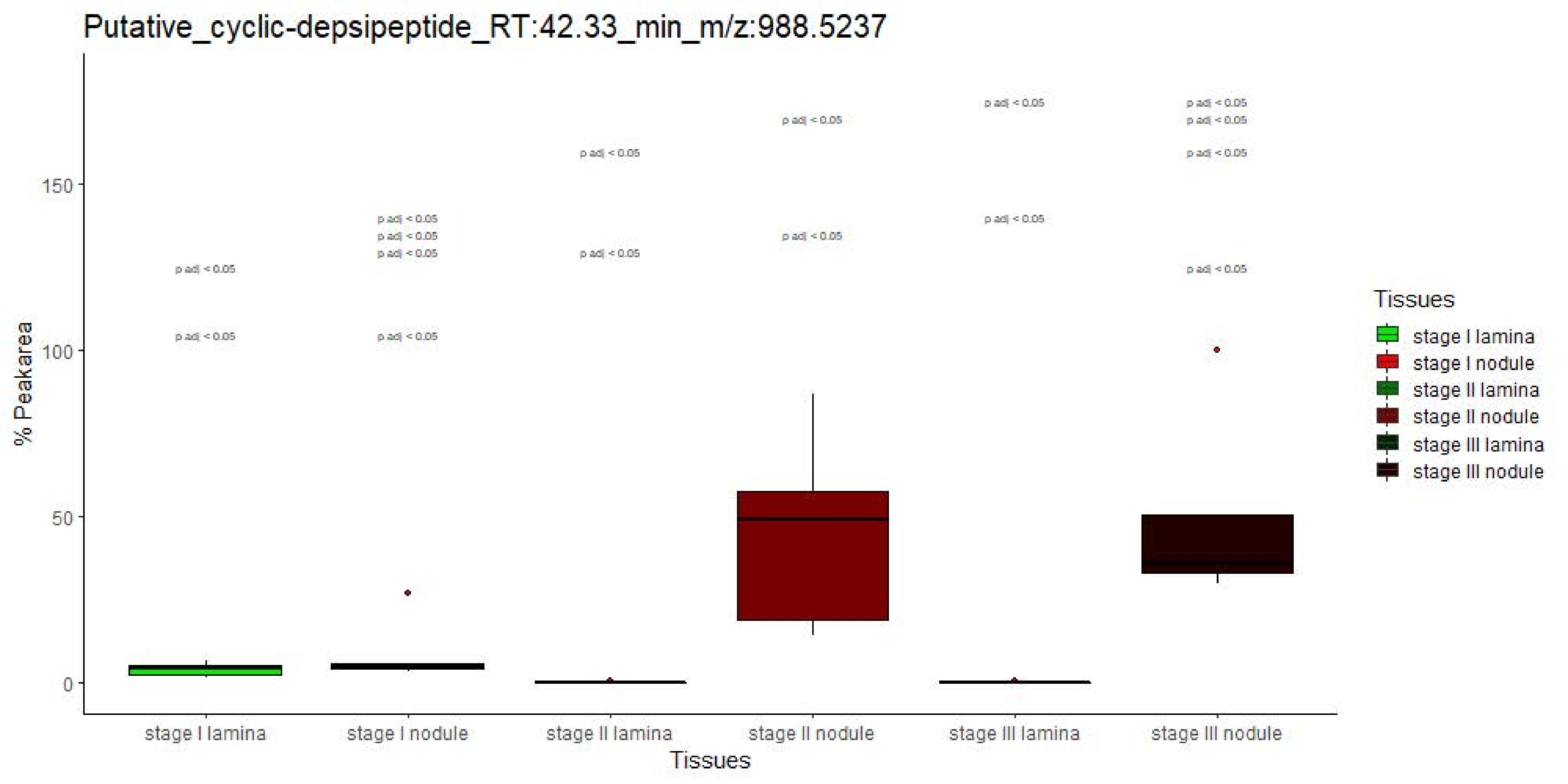

Putative_cyclic-depsipeptide_RT:42.33_min_m/z:988.5237
% Peakarea
0
50
100
150
p adj < 0.05
Tissues
stage I lamina
stage I nodule
stage II lamina
stage II nodule
stage III lamina
stage III nodule

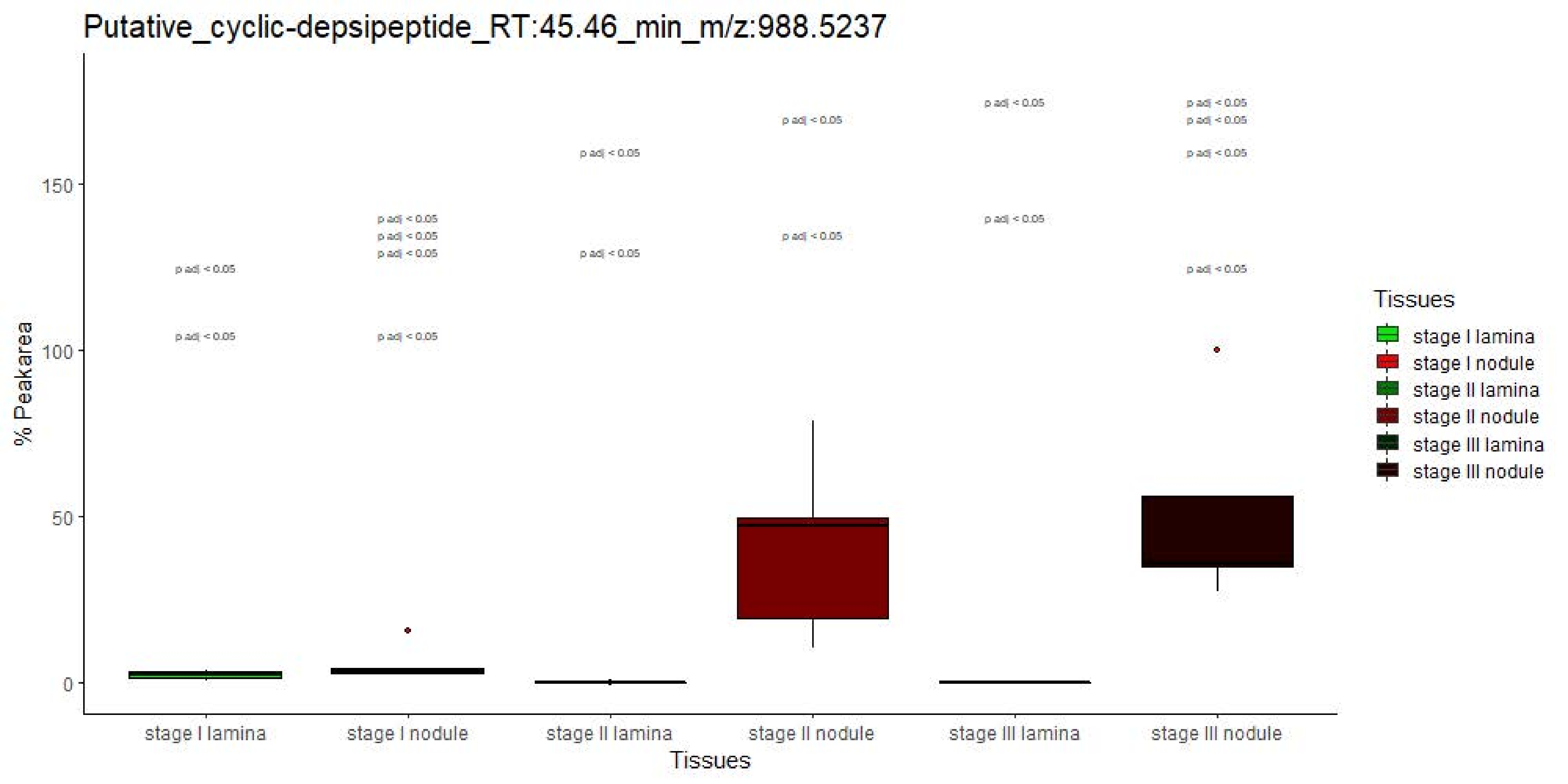

Putative_cyclic-depsipeptide_RT:45.46_min_m/z:988.5237
% Peakarea
150
100
50
0
p adj < 0.05
stage I lamina
stage I nodule
stage II lamina
stage II nodule
stage III lamina
stage III nodule
Tissues

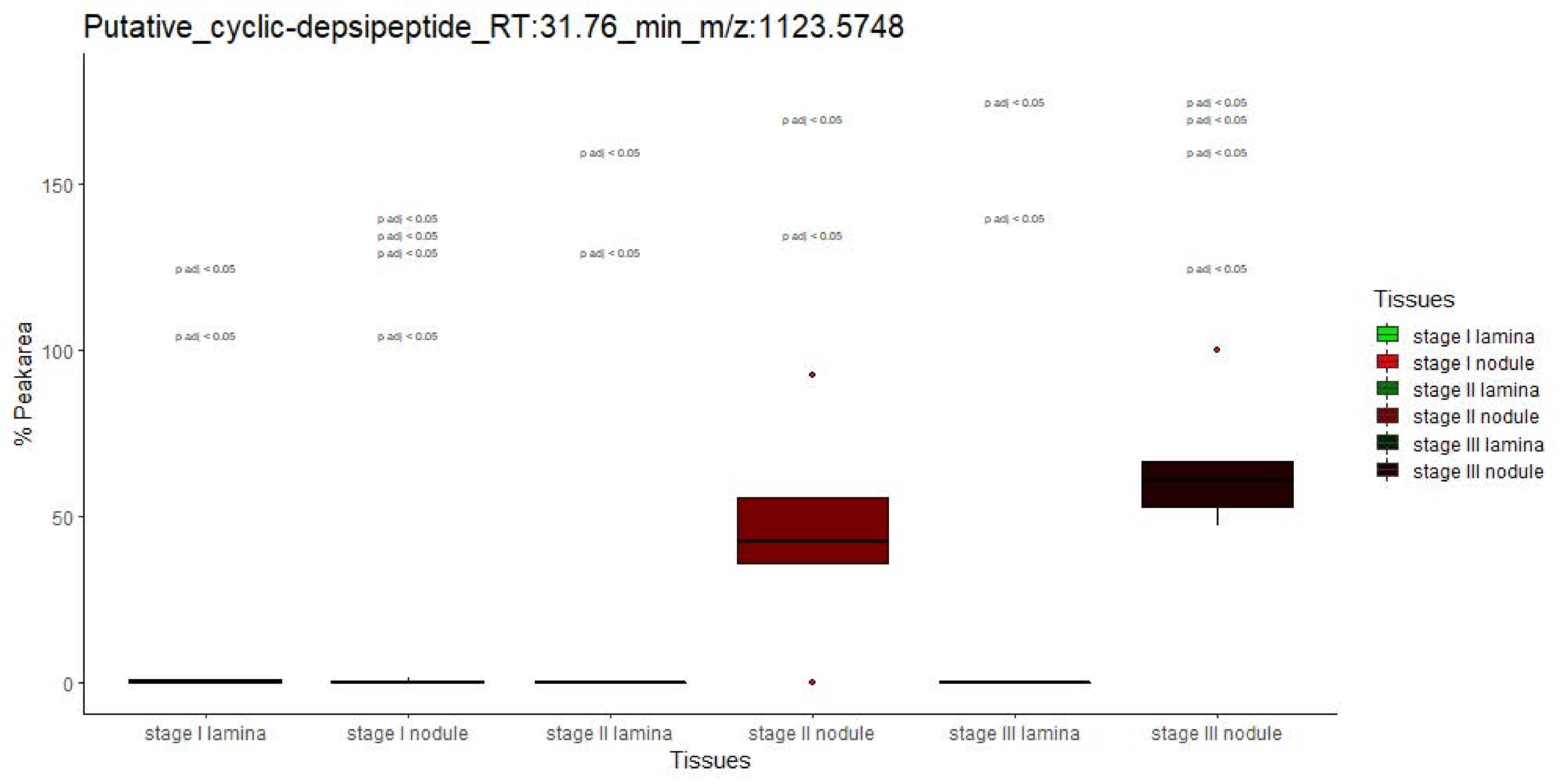

Putative_cyclic-depsipeptide_RT:31.76_min_m/z:1123.5748
% Peakarea
0
50
100
150
p adj < 0.05
stage I lamina
stage I nodule
stage II lamina
stage II nodule
stage III lamina
stage III nodule
Tissues

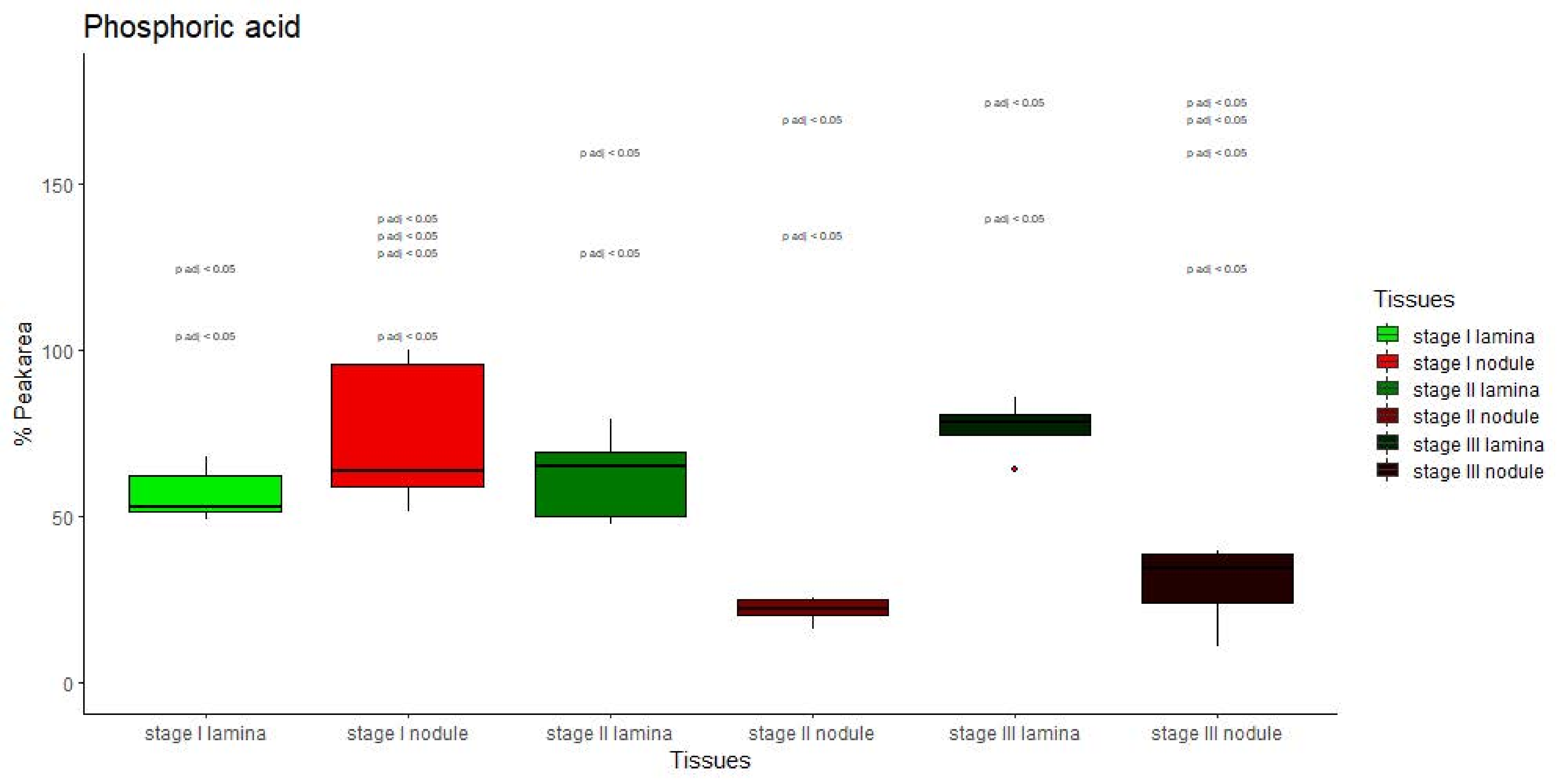
Phosphoric acid
% Peakarea
0
50
100
150
p adj < 0.05
stage I lamina
stage I nodule
stage II lamina
stage II nodule
stage III lamina
stage III nodule
Tissues
Tissues
stage I lamina
stage I nodule
stage II lamina
stage II nodule
stage III lamina
stage III nodule

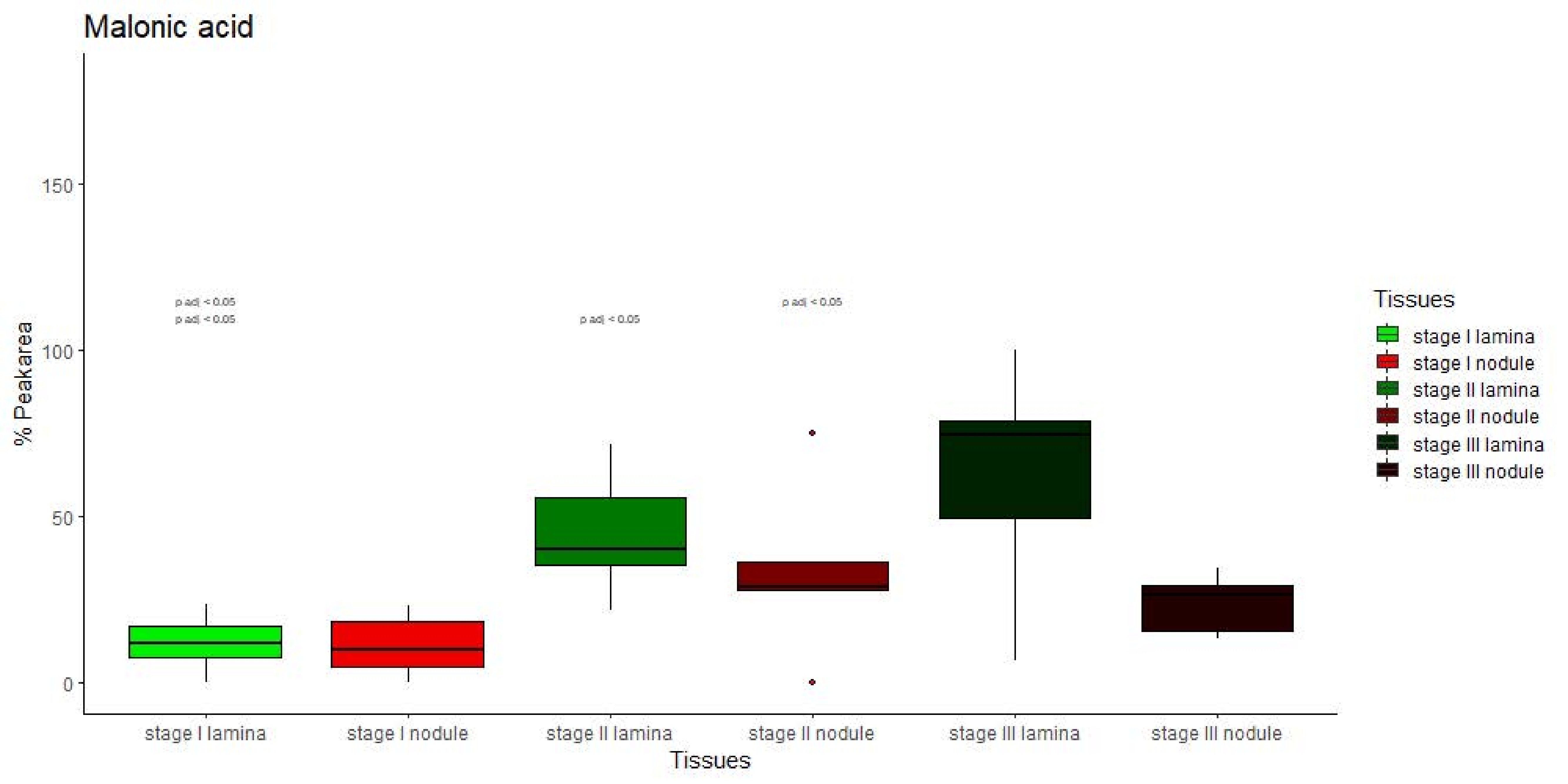

Malonic acid
% Peakarea
150
100
50
0
p adj < 0.05
p adj < 0.05
p adj < 0.05
p adj < 0.05
stage I lamina
stage I nodule
stage II lamina
stage II nodule
stage III lamina
stage III nodule
Tissues
Tissues
stage I lamina
stage I nodule
stage II lamina
stage II nodule
stage III lamina
stage III nodule

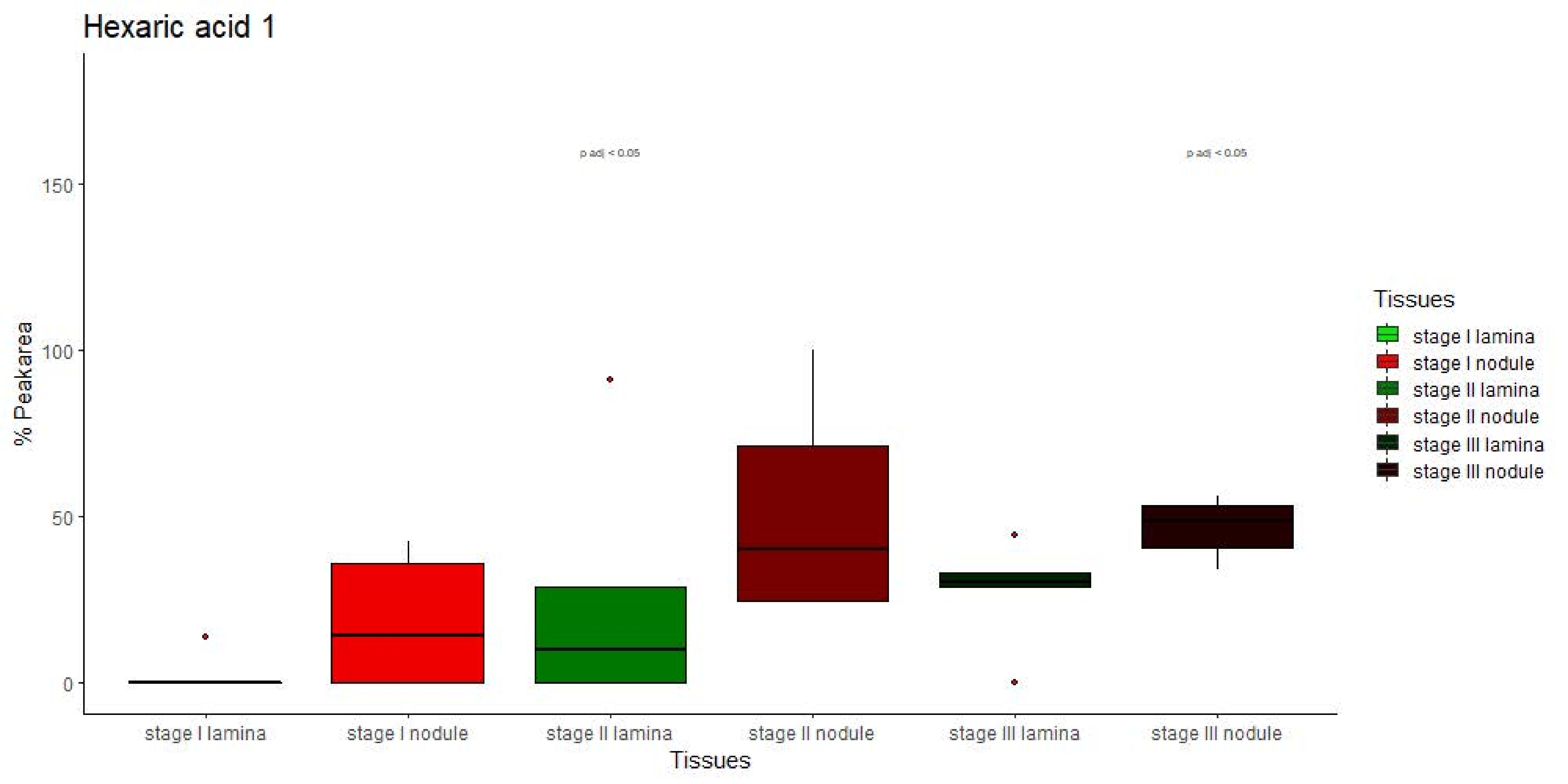
Hexaric acid 1
p adj < 0.05
p adj < 0.05
% Peakarea
150
100
50
0
stage I lamina
stage I nodule
stage II lamina
stage II nodule
stage III lamina
stage III nodule
Tissues
Tissues
stage I lamina
stage I nodule
stage II lamina
stage II nodule
stage III lamina
stage III nodule

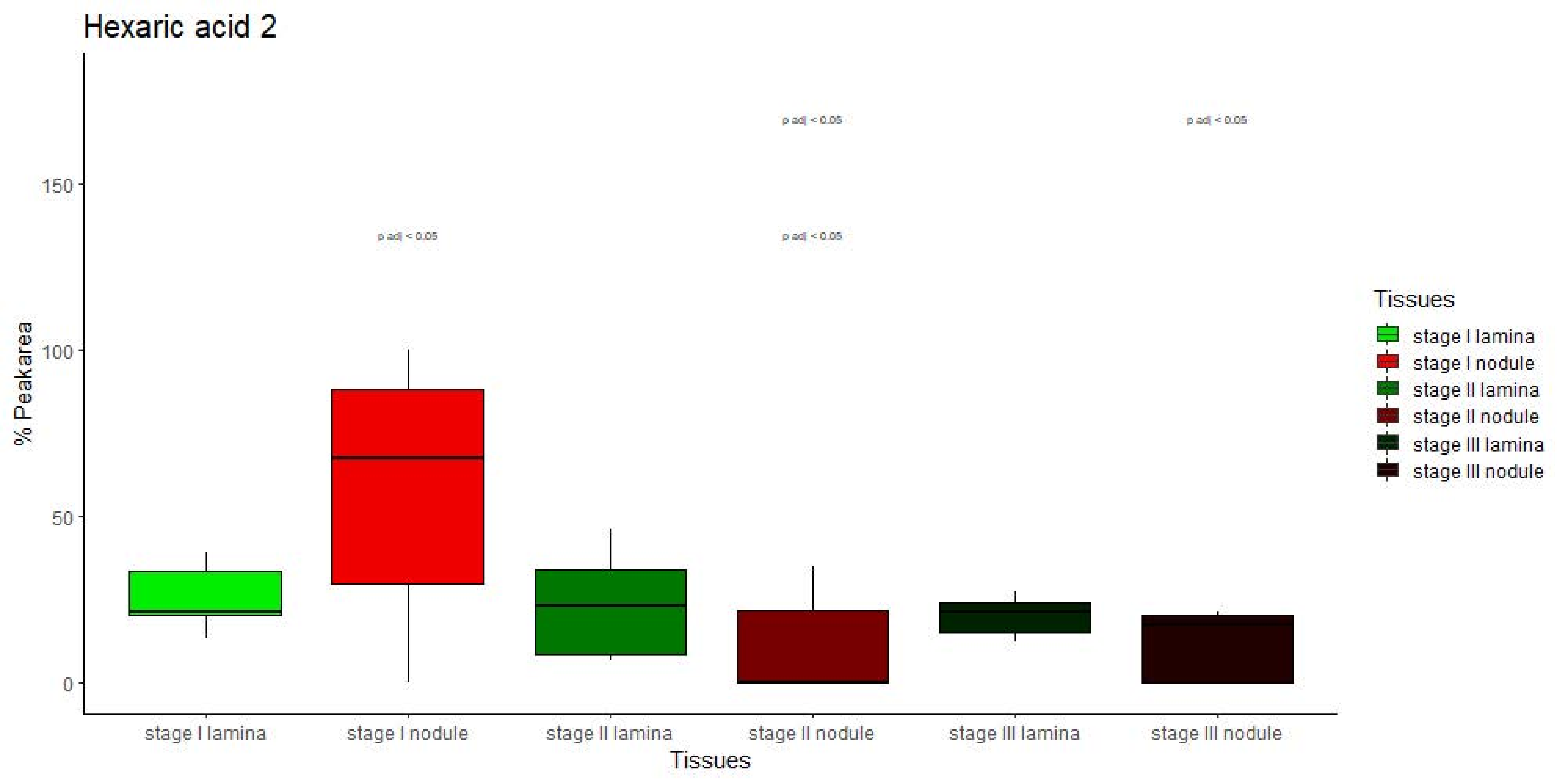

Hexaric acid 2
p adj < 0.05
p adj < 0.05
p adj < 0.05
p adj < 0.05
150
100
50
0
% Peakarea
stage I lamina
stage I nodule
stage II lamina
stage II nodule
stage III lamina
stage III nodule
Tissues
Tissues
stage I lamina
stage I nodule
stage II lamina
stage II nodule
stage III lamina
stage III nodule

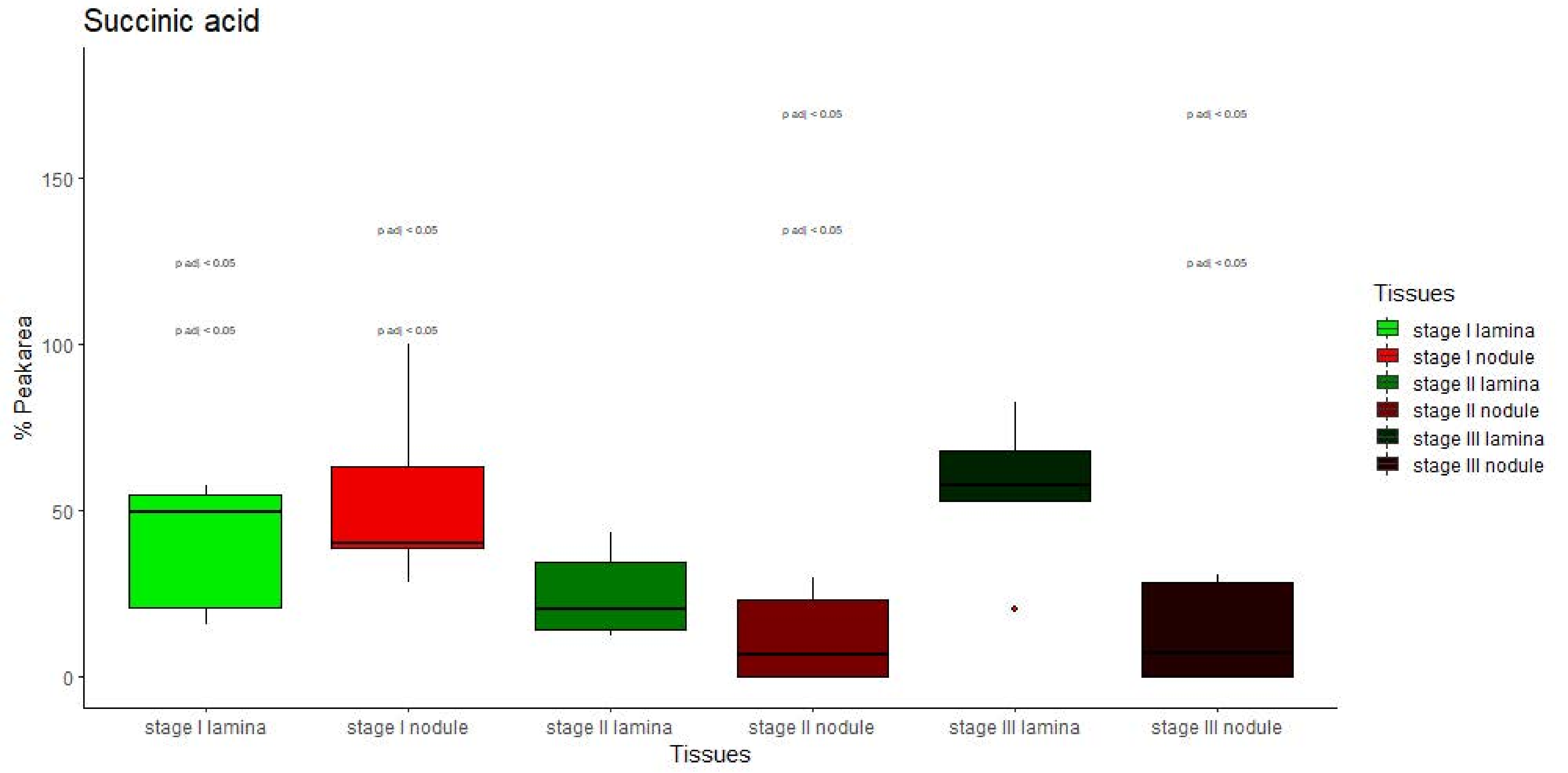
Succinic acid
% Peakarea
150
100
50
0
p adj < 0.05
p adj < 0.05
p adj < 0.05
p adj < 0.05
p adj < 0.05
p adj < 0.05
p adj < 0.05
p adj < 0.05
stage I lamina
stage I nodule
stage II lamina
stage II nodule
stage III lamina
stage III nodule
Tissues
Tissues
stage I lamina
stage I nodule
stage II lamina
stage II nodule
stage III lamina
stage III nodule

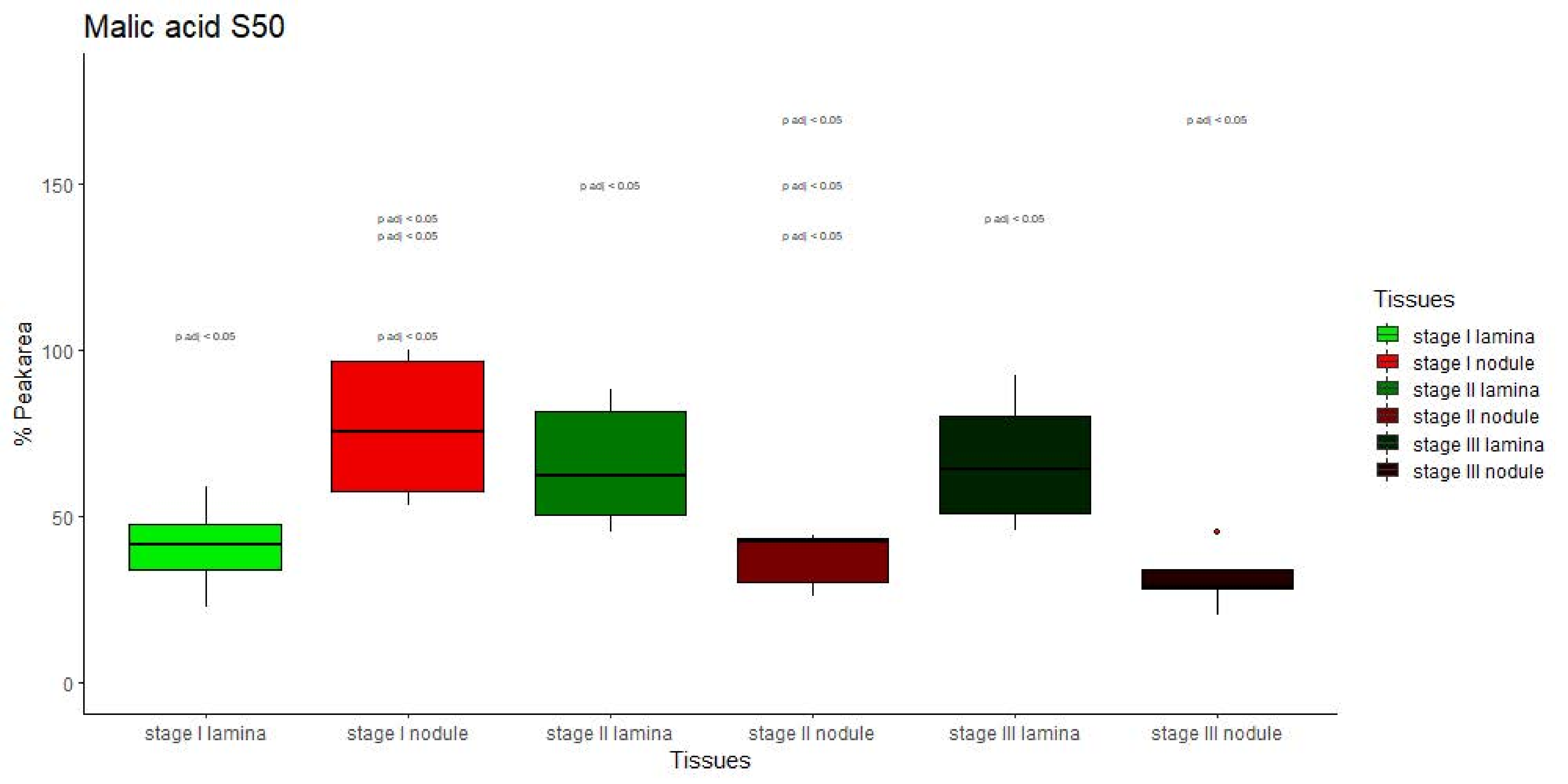

Malic acid S50
% Peakarea
0
50
100
150
p adj < 0.05
p adj < 0.05
p adj < 0.05
p adj < 0.05
p adj < 0.05
p adj < 0.05
p adj < 0.05
p adj < 0.05
p adj < 0.05
p adj < 0.05
stage I lamina
stage I nodule
stage II lamina
stage II nodule
stage III lamina
stage III nodule
Tissues
Tissues
stage I lamina
stage I nodule
stage II lamina
stage II nodule
stage III lamina
stage III nodule

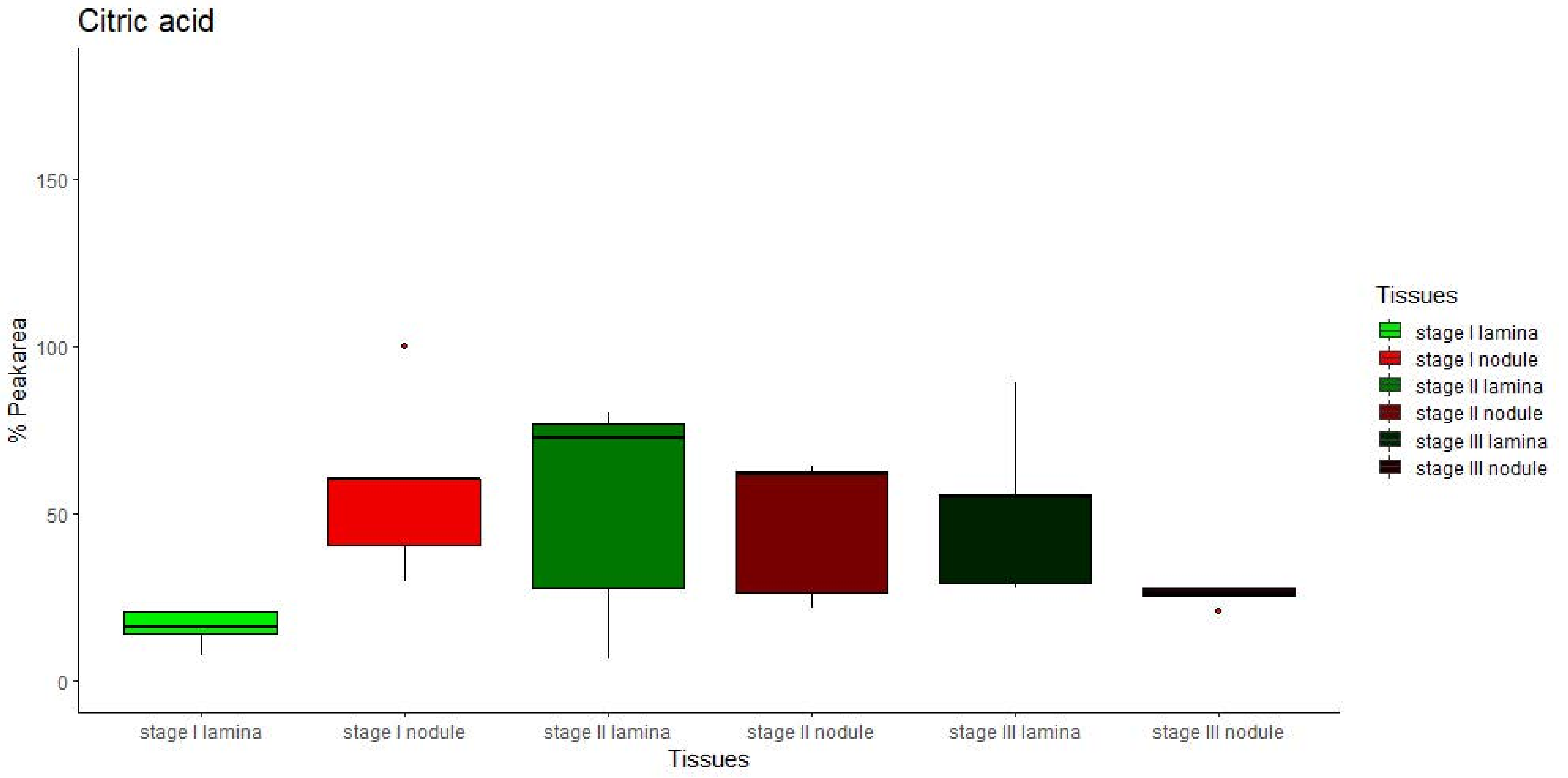
Citric acid
% Peakarea
150
100
50
0
stage I lamina
stage I nodule
stage II lamina
stage II nodule
stage III lamina
stage III nodule
Tissues
Tissues
stage I lamina
stage I nodule
stage II lamina
stage II nodule
stage III lamina
stage III nodule

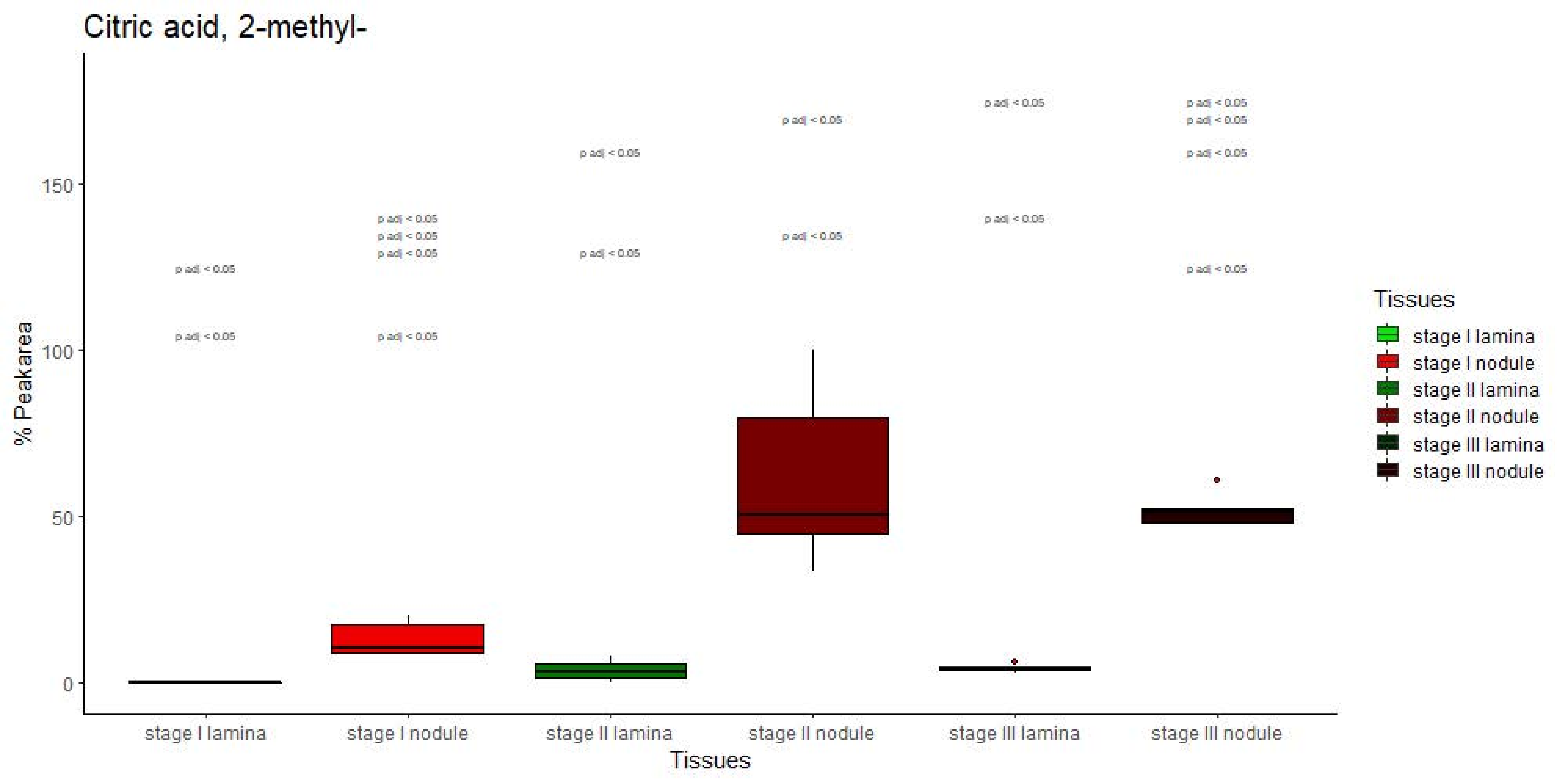

Citric acid, 2-methyl-
p adj < 0.05
150
100
50
0
% Peakarea
stage I lamina
stage I nodule
stage II lamina
stage II nodule
stage III lamina
stage III nodule
Tissues

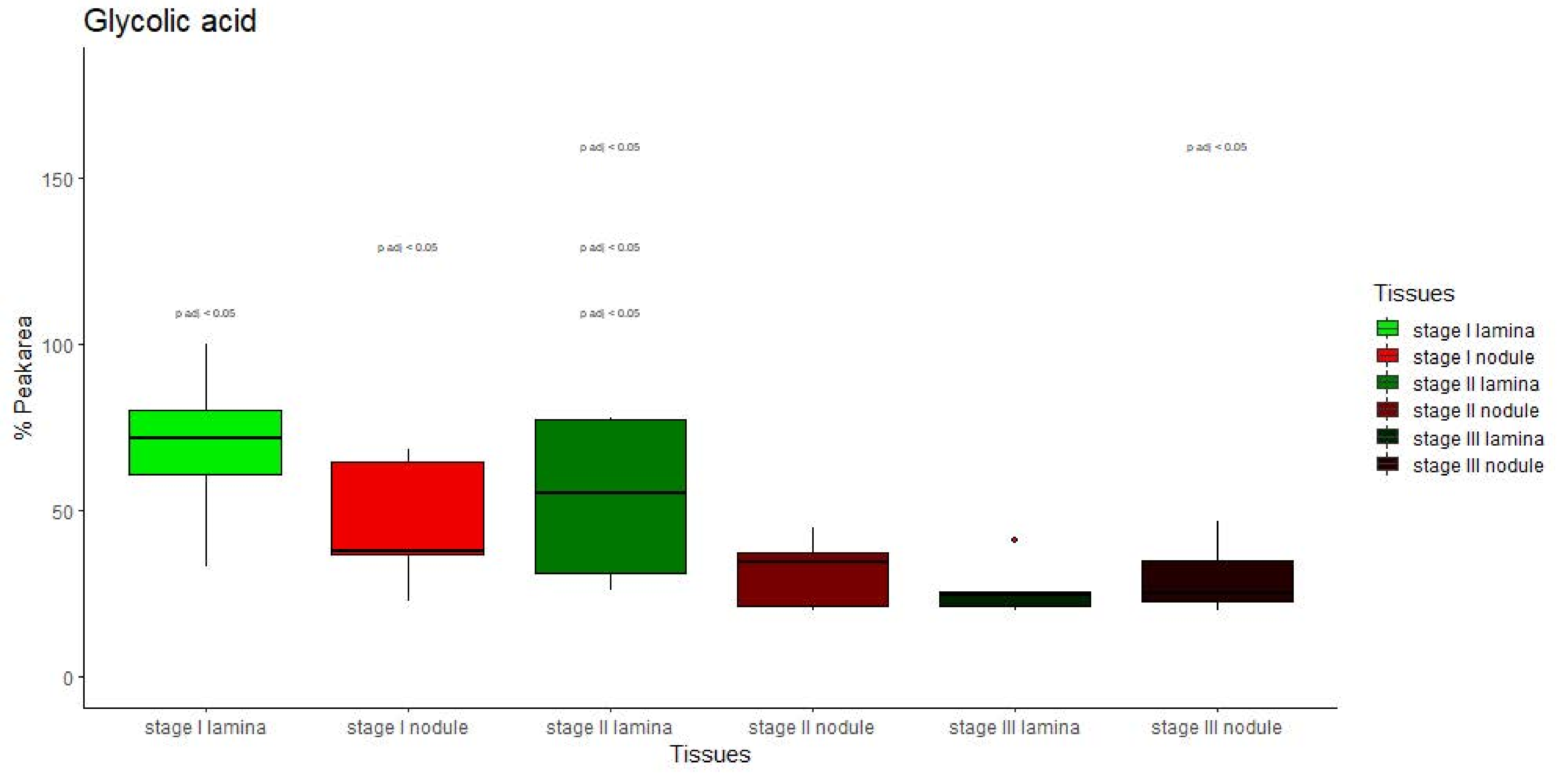

Glycolic acid
p adj < 0.05
p adj < 0.05
p adj < 0.05
p adj < 0.05
p adj < 0.05
p adj < 0.05
150
100
50
0
% Peakarea
stage I lamina
stage I nodule
stage II lamina
stage II nodule
stage III lamina
stage III nodule
Tissues
Tissues
stage I lamina
stage I nodule
stage II lamina
stage II nodule
stage III lamina
stage III nodule

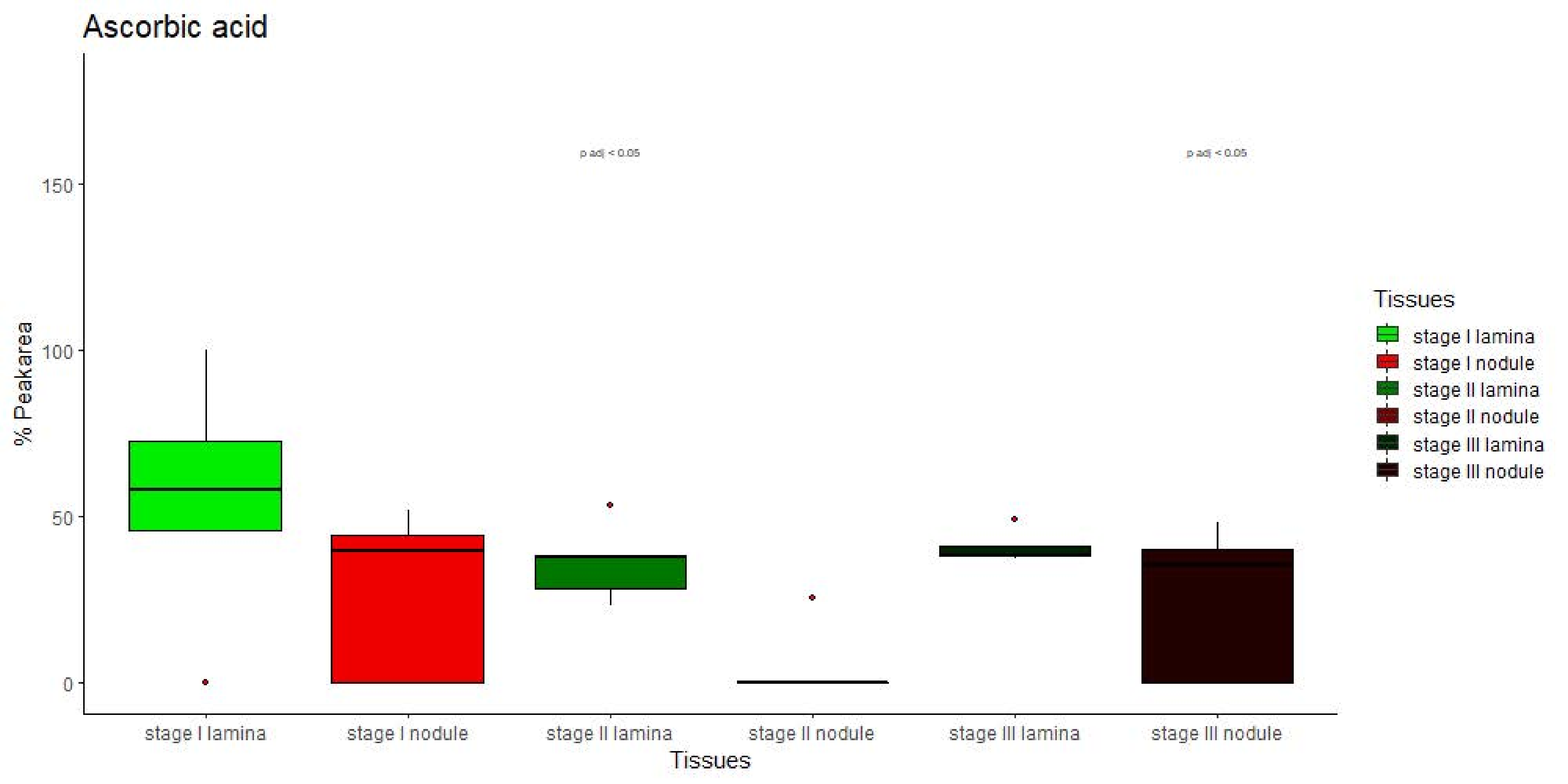

Ascorbic acid
p adj < 0.05
p adj < 0.05
% Peakarea
0
50
100
150
stage I lamina
stage I nodule
stage II lamina
stage II nodule
stage III lamina
stage III nodule
Tissues
Tissues
stage I lamina
stage I nodule
stage II lamina
stage II nodule
stage III lamina
stage III nodule

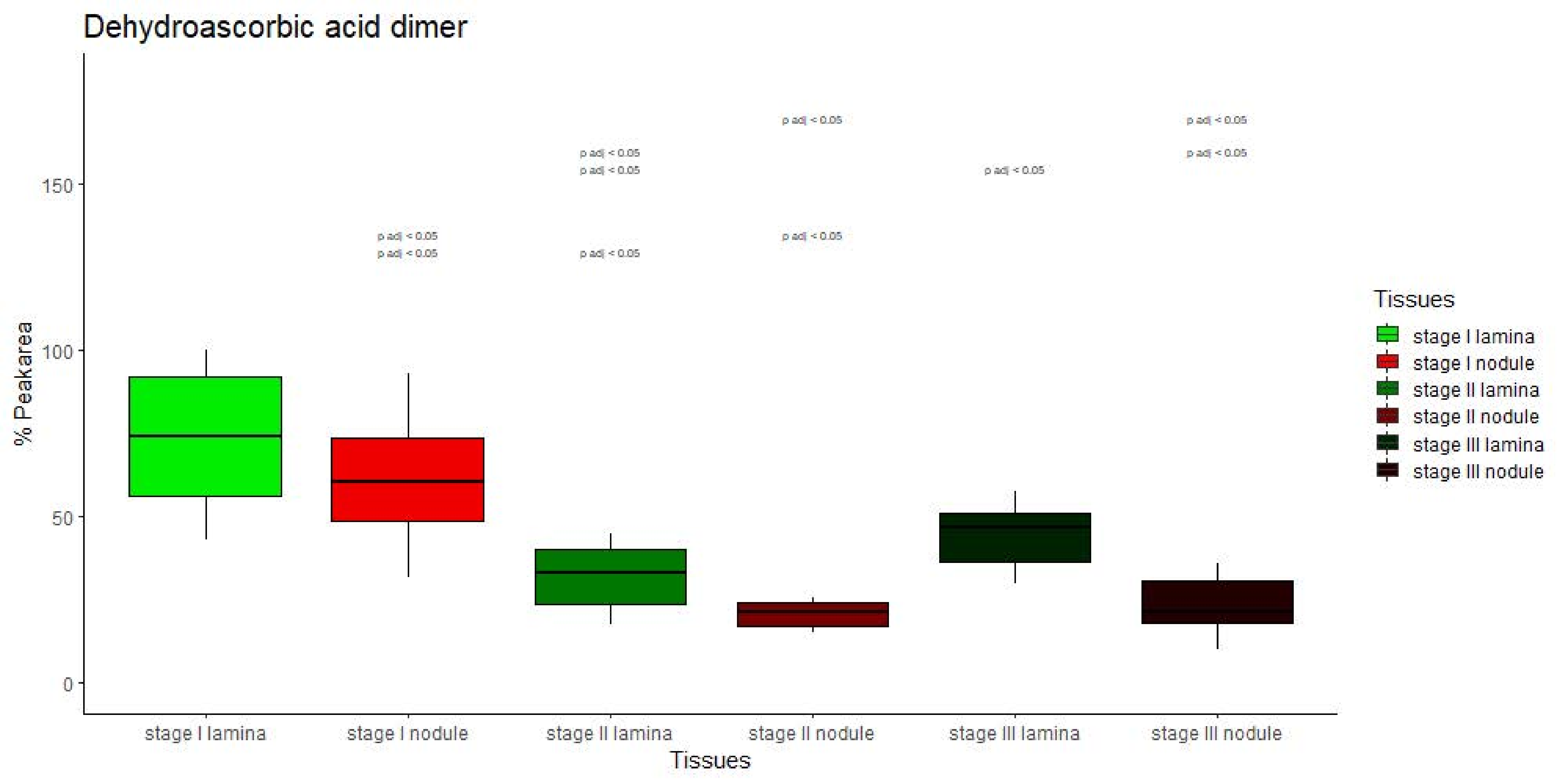
Dehydroascorbic acid dimer
% Peakarea
150
100
50
0
p adj < 0.05
stage I lamina
stage I nodule
stage II lamina
stage II nodule
stage III lamina
stage III nodule
Tissues
Tissues
stage I lamina
stage I nodule
stage II lamina
stage II nodule
stage III lamina
stage III nodule

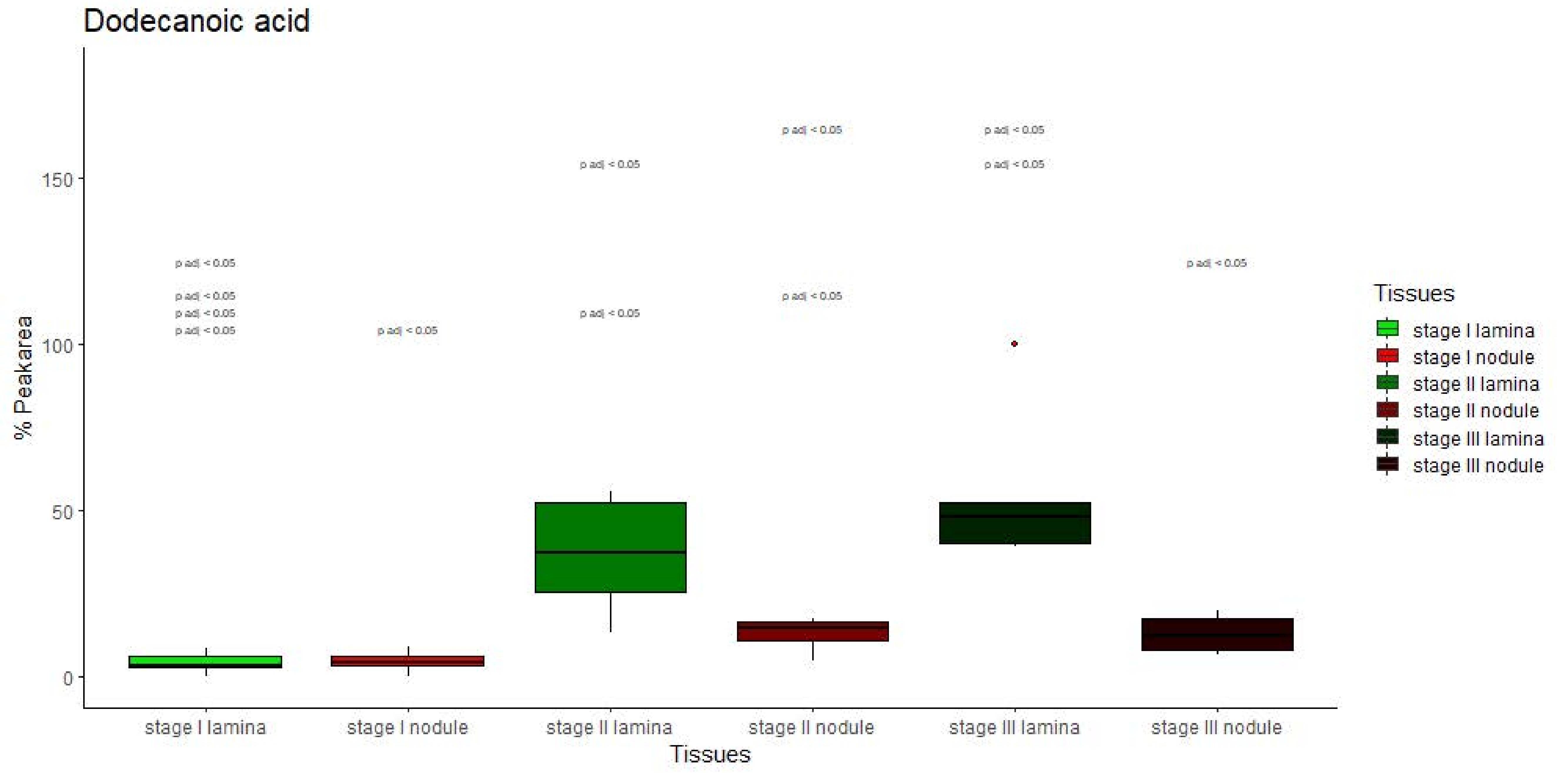

Dodecanoic acid
% Peakarea
150
100
50
0
p adj < 0.05
p adj < 0.05
p adj < 0.05
p adj < 0.05
p adj < 0.05
p adj < 0.05
p adj < 0.05
p adj < 0.05
p adj < 0.05
p adj < 0.05
p adj < 0.05
p adj < 0.05
stage I lamina
stage I nodule
stage II lamina
stage II nodule
stage III lamina
stage III nodule
Tissues
Tissues
stage I lamina
stage I nodule
stage II lamina
stage II nodule
stage III lamina
stage III nodule

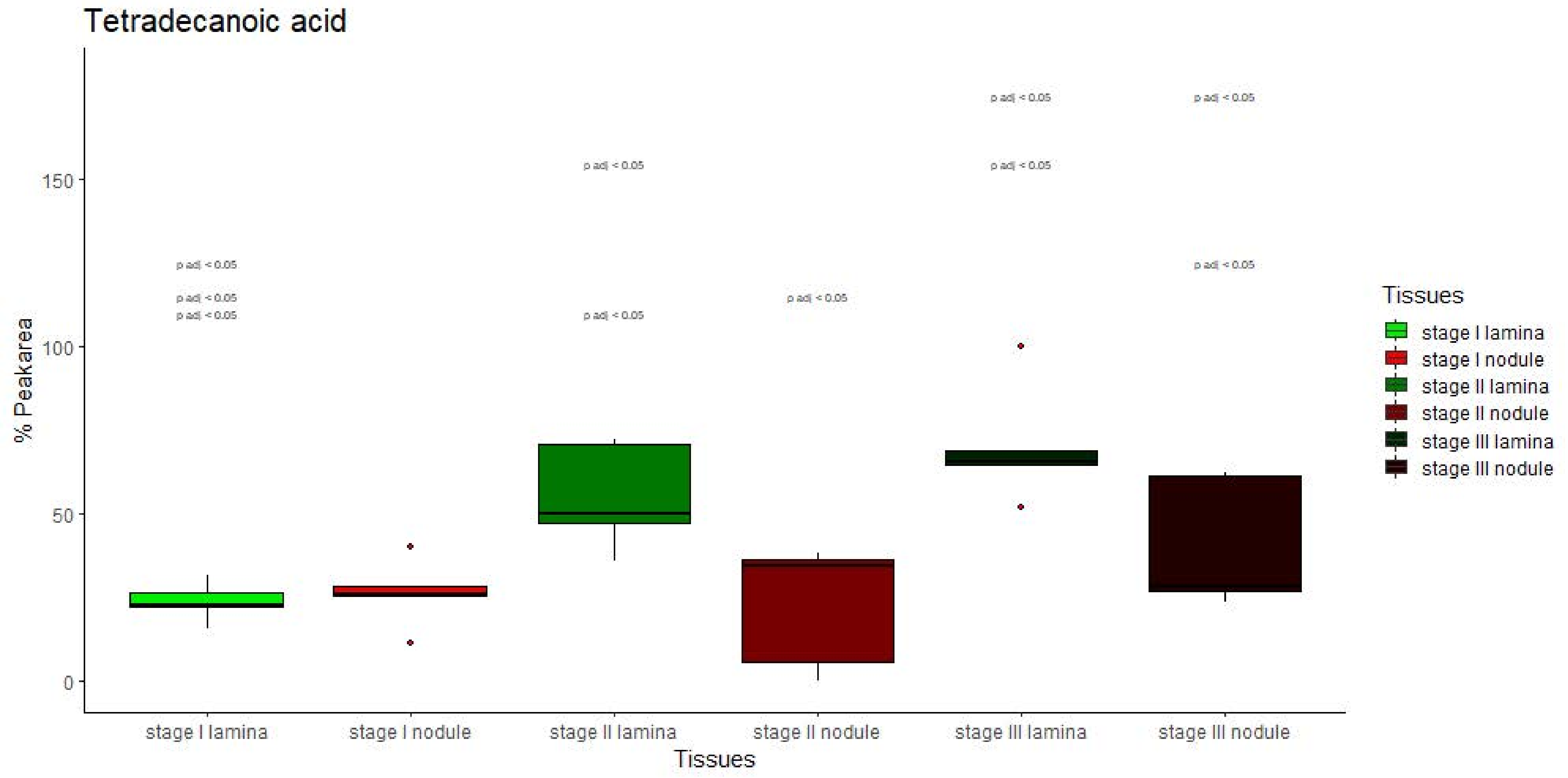
Tetradecanoic acid
% Peakarea
150
100
50
0
p adj < 0.05
p adj < 0.05
p adj < 0.05
p adj < 0.05
p adj < 0.05
p adj < 0.05
p adj < 0.05
p adj < 0.05
p adj < 0.05
p adj < 0.05
stage I lamina
stage I nodule
stage II lamina
stage II nodule
stage III lamina
stage III nodule
Tissues
Tissues
stage I lamina
stage I nodule
stage II lamina
stage II nodule
stage III lamina
stage III nodule

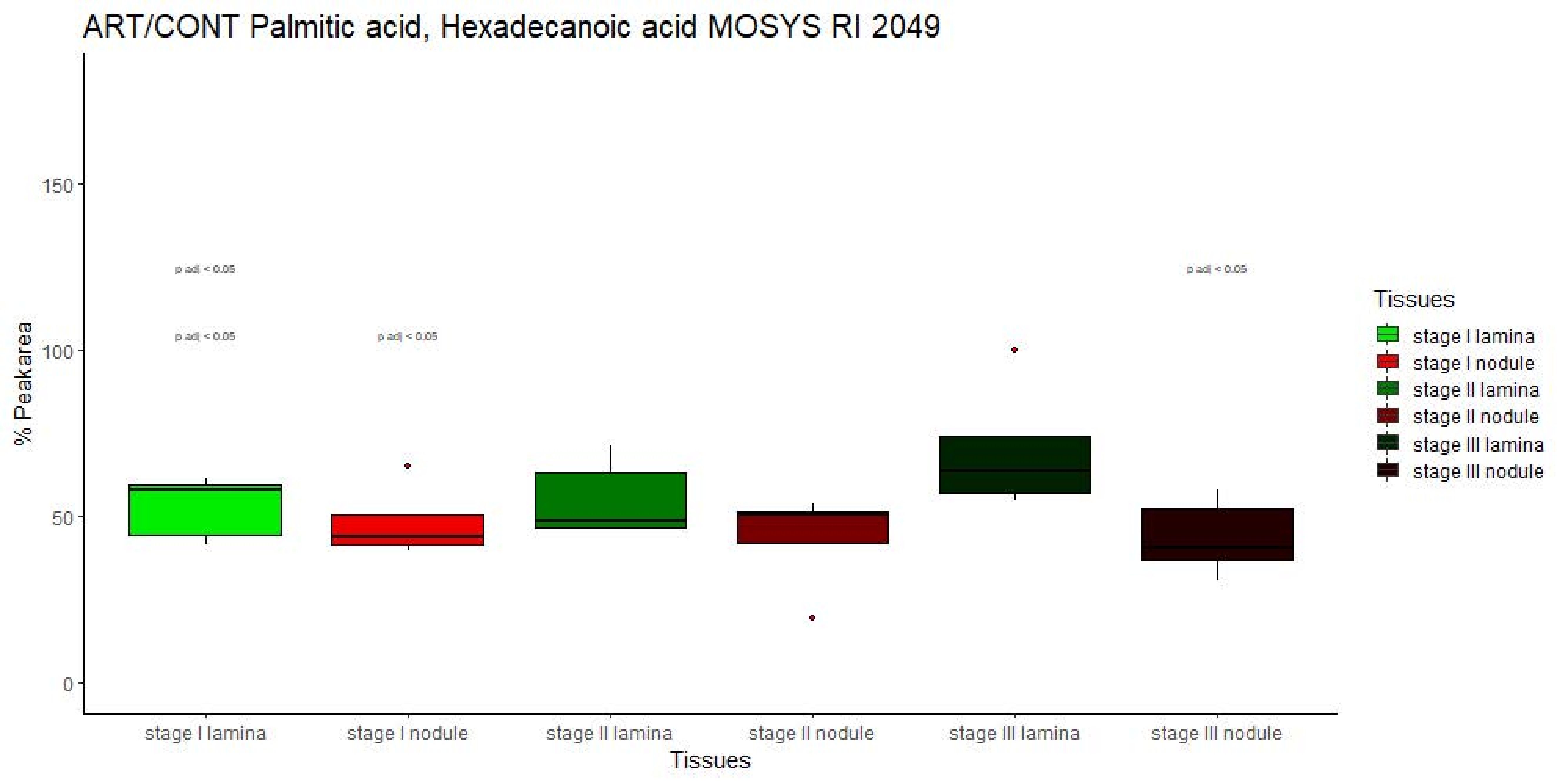

ART/CONT Palmitic acid, Hexadecanoic acid MOSYS RI 2049
% Peakarea
150
100
50
0
p adj < 0.05
p adj < 0.05
p adj < 0.05
p adj < 0.05
stage I lamina
stage I nodule
stage II lamina
stage II nodule
stage III lamina
stage III nodule
Tissues
Tissues
stage I lamina
stage I nodule
stage II lamina
stage II nodule
stage III lamina
stage III nodule

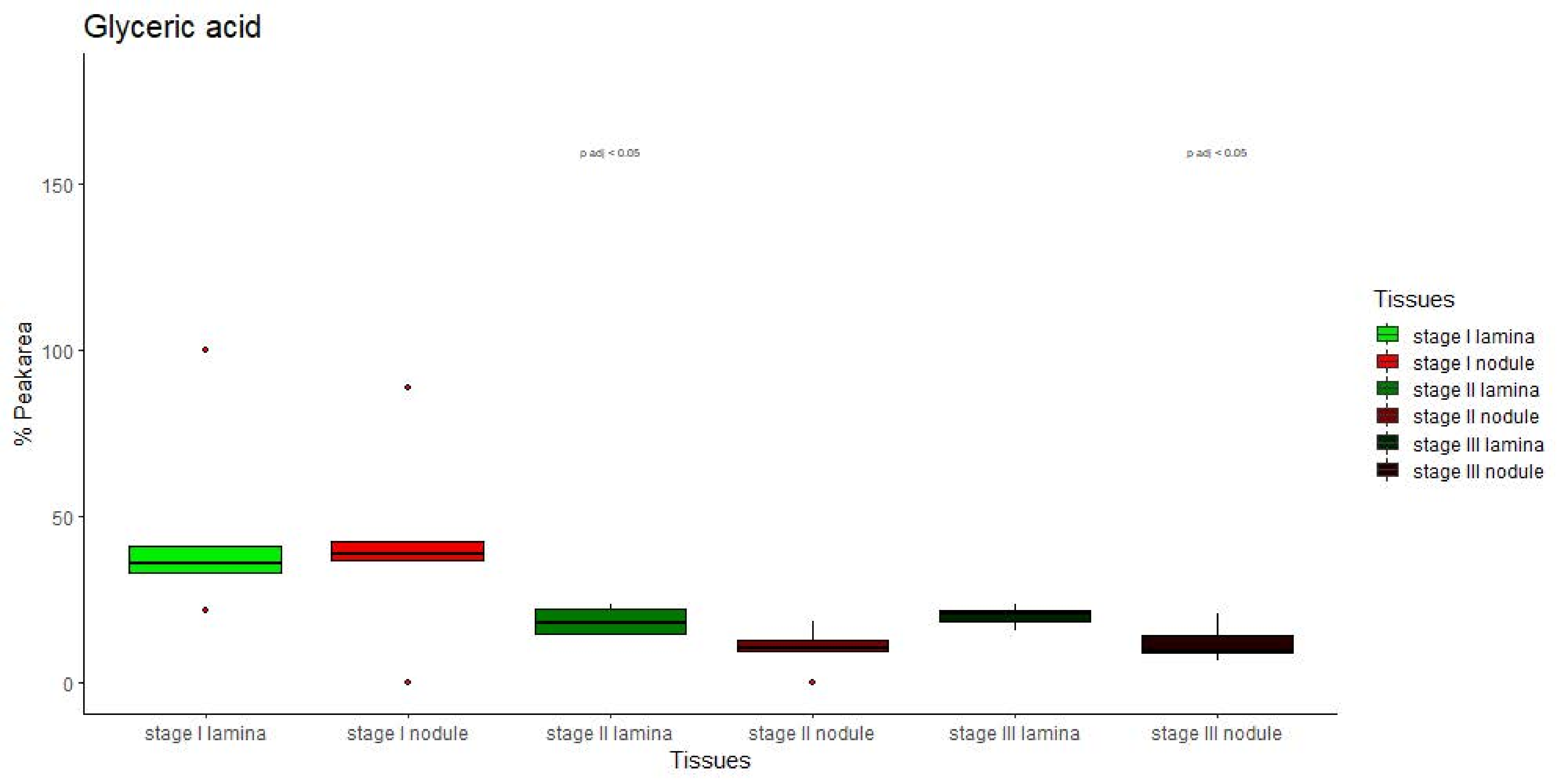

Glyceric acid
p adj < 0.05
p adj < 0.05
% Peakarea
0
50
100
150
stage I lamina
stage I nodule
stage II lamina
stage II nodule
stage III lamina
stage III nodule
Tissues
Tissues
stage I lamina
stage I nodule
stage II lamina
stage II nodule
stage III lamina
stage III nodule

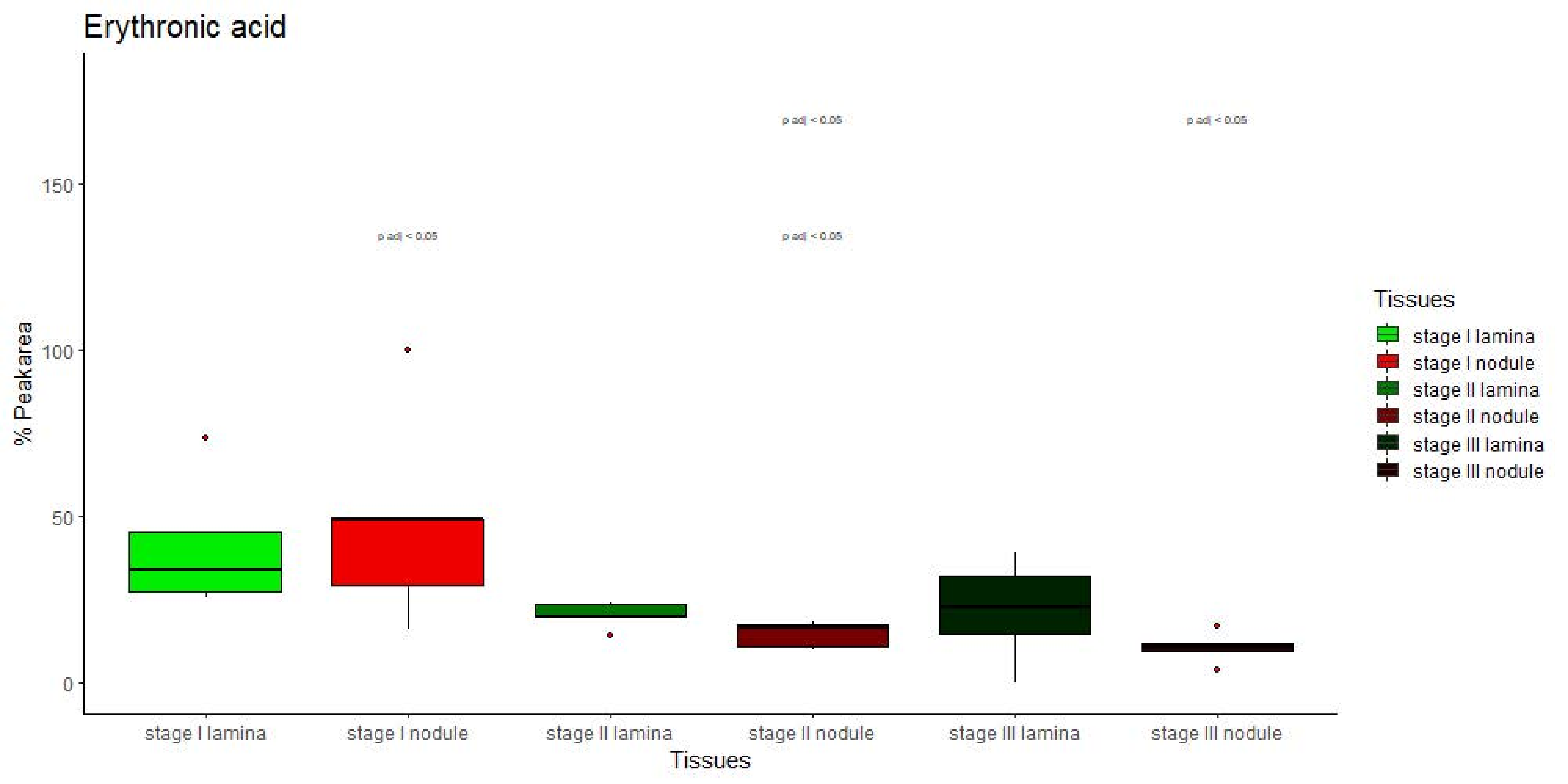

Erythronic acid
p adj < 0.05
p adj < 0.05
p adj < 0.05
p adj < 0.05
150
100
50
0
% Peakarea
stage I lamina
stage I nodule
stage II lamina
stage II nodule
stage III lamina
stage III nodule
Tissues
Tissues
stage I lamina
stage I nodule
stage II lamina
stage II nodule
stage III lamina
stage III nodule

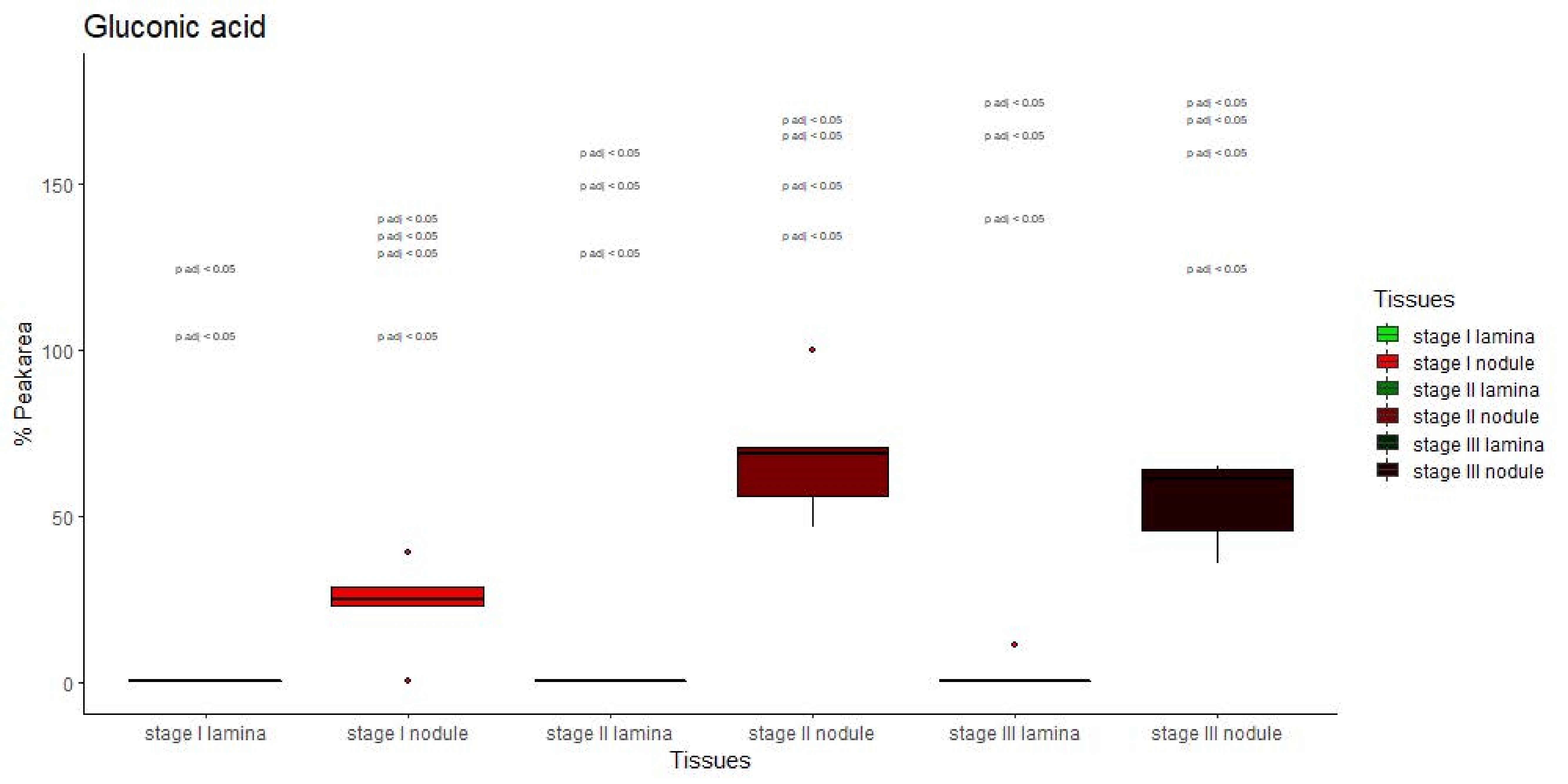

Gluconic acid
% Peakarea
0
50
100
150
p adj < 0.05
Tissues
stage I lamina
stage I nodule
stage II lamina
stage II nodule
stage III lamina
stage III nodule
Tissues
stage I lamina
stage I nodule
stage II lamina
stage II nodule
stage III lamina
stage III nodule

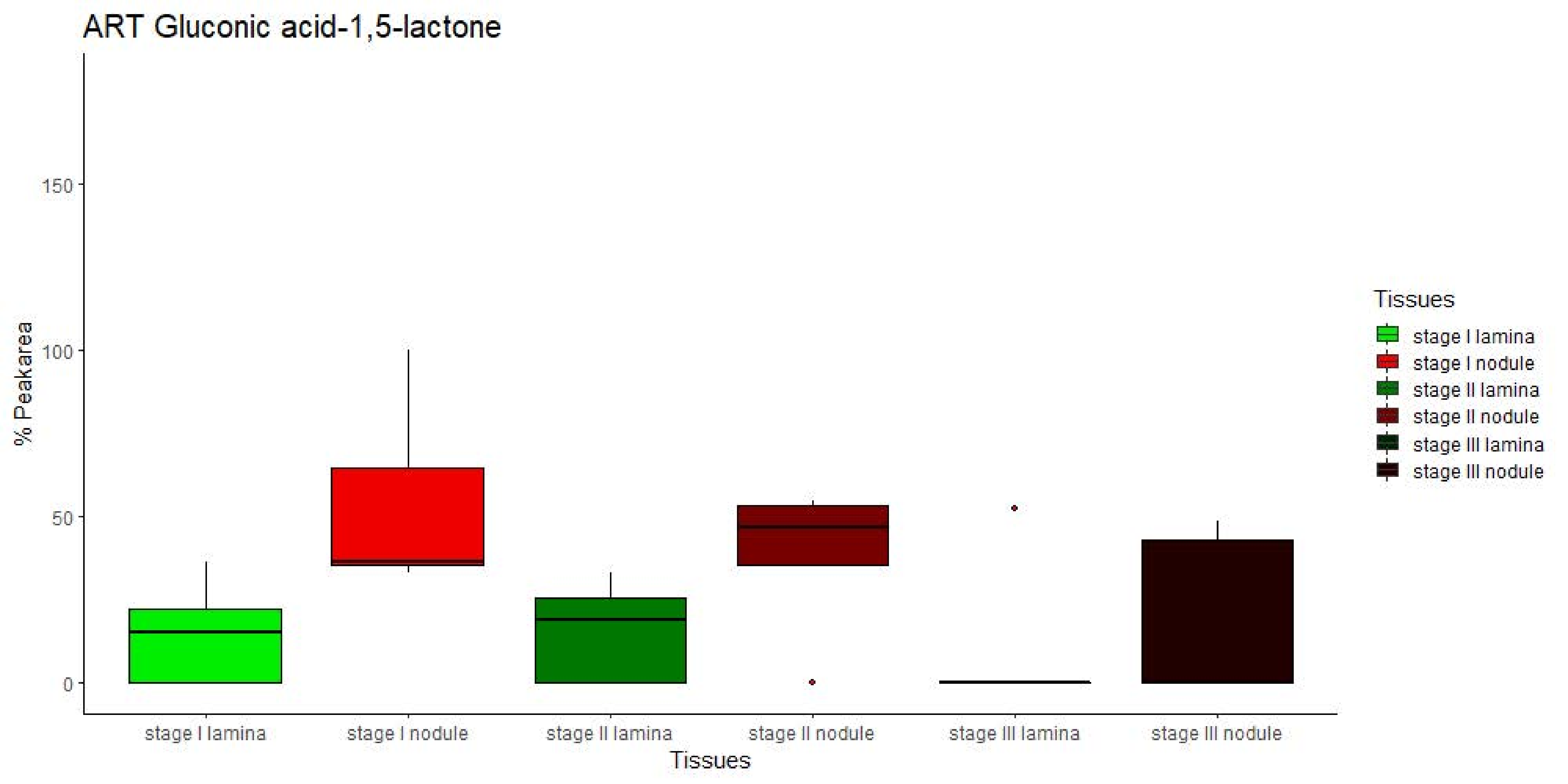

ART Gluconic acid-1,5-lactone
% Peakarea
150
100
50
0
stage I lamina
stage I nodule
stage II lamina
stage II nodule
stage III lamina
stage III nodule
Tissues
Tissues
stage I lamina
stage I nodule
stage II lamina
stage II nodule
stage III lamina
stage III nodule

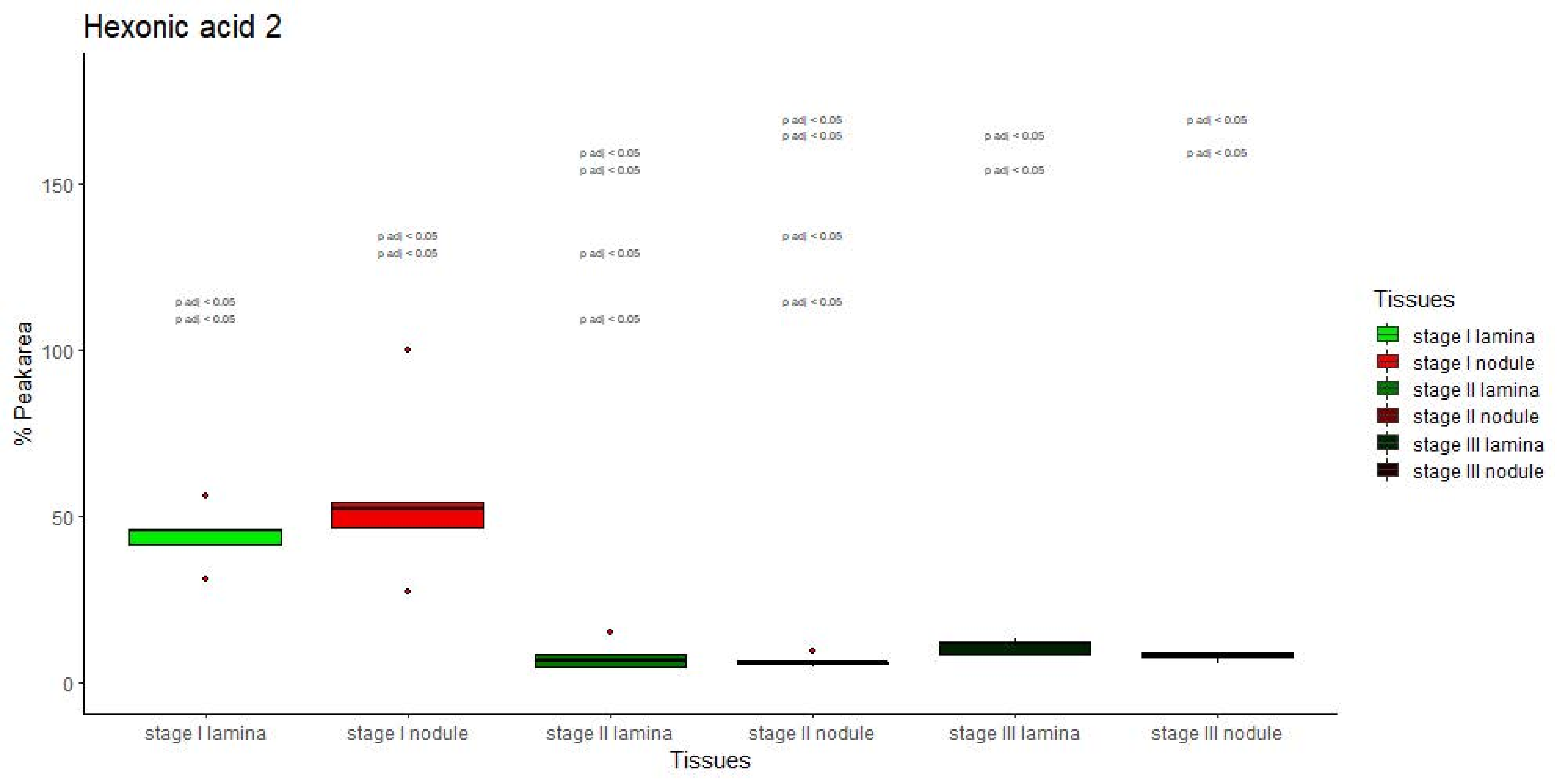

Hexonic acid 2
% Peakarea
0
50
100
150
p adj < 0.05
Tissues
stage I lamina
stage I nodule
stage II lamina
stage II nodule
stage III lamina
stage III nodule

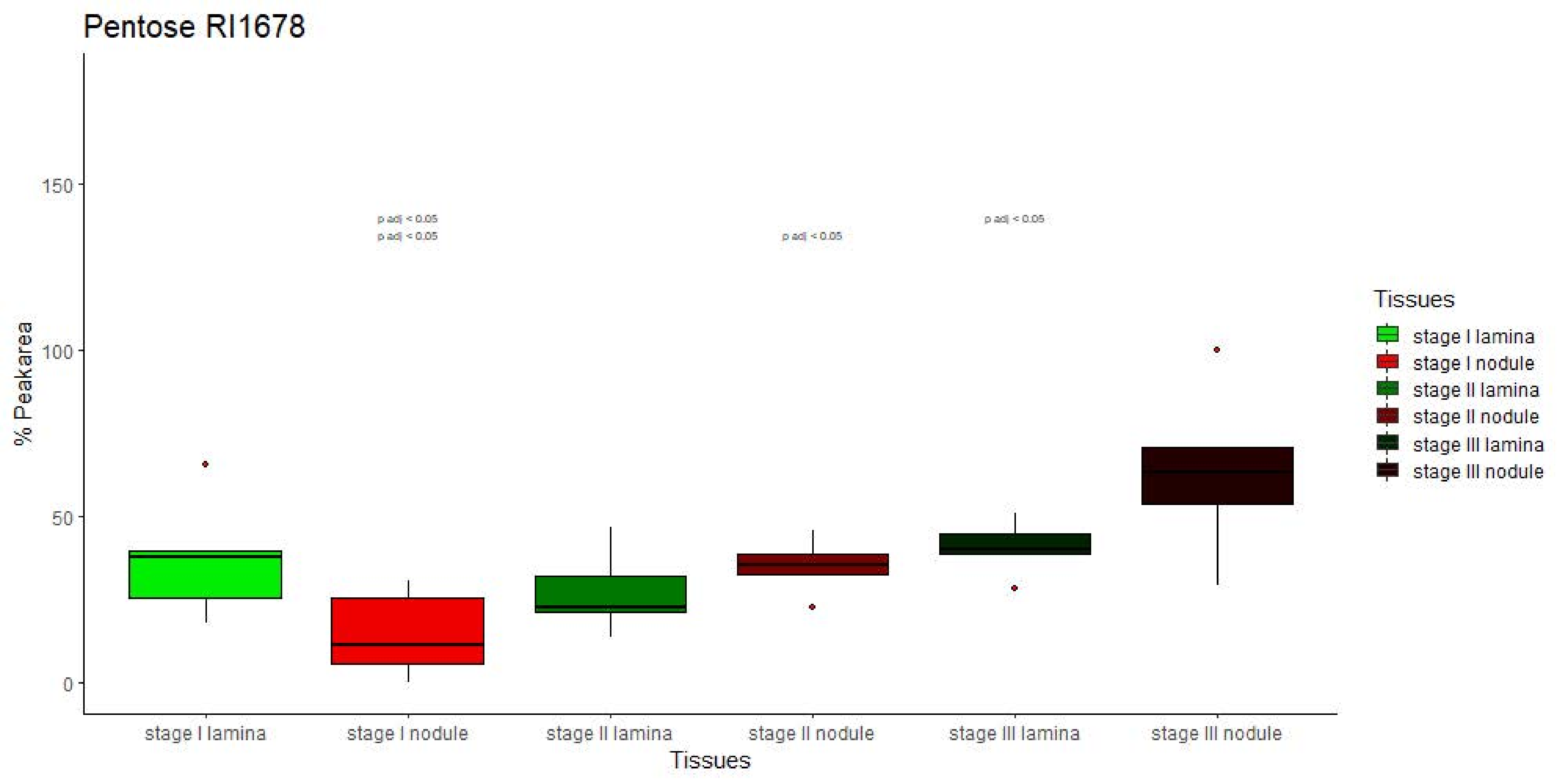

Pentose RI1678
% Peakarea
150
100
50
0
p adj < 0.05
p adj < 0.05
p adj < 0.05
p adj < 0.05
stage I lamina
stage I nodule
stage II lamina
stage II nodule
stage III lamina
stage III nodule
Tissues
Tissues
stage I lamina
stage I nodule
stage II lamina
stage II nodule
stage III lamina
stage III nodule

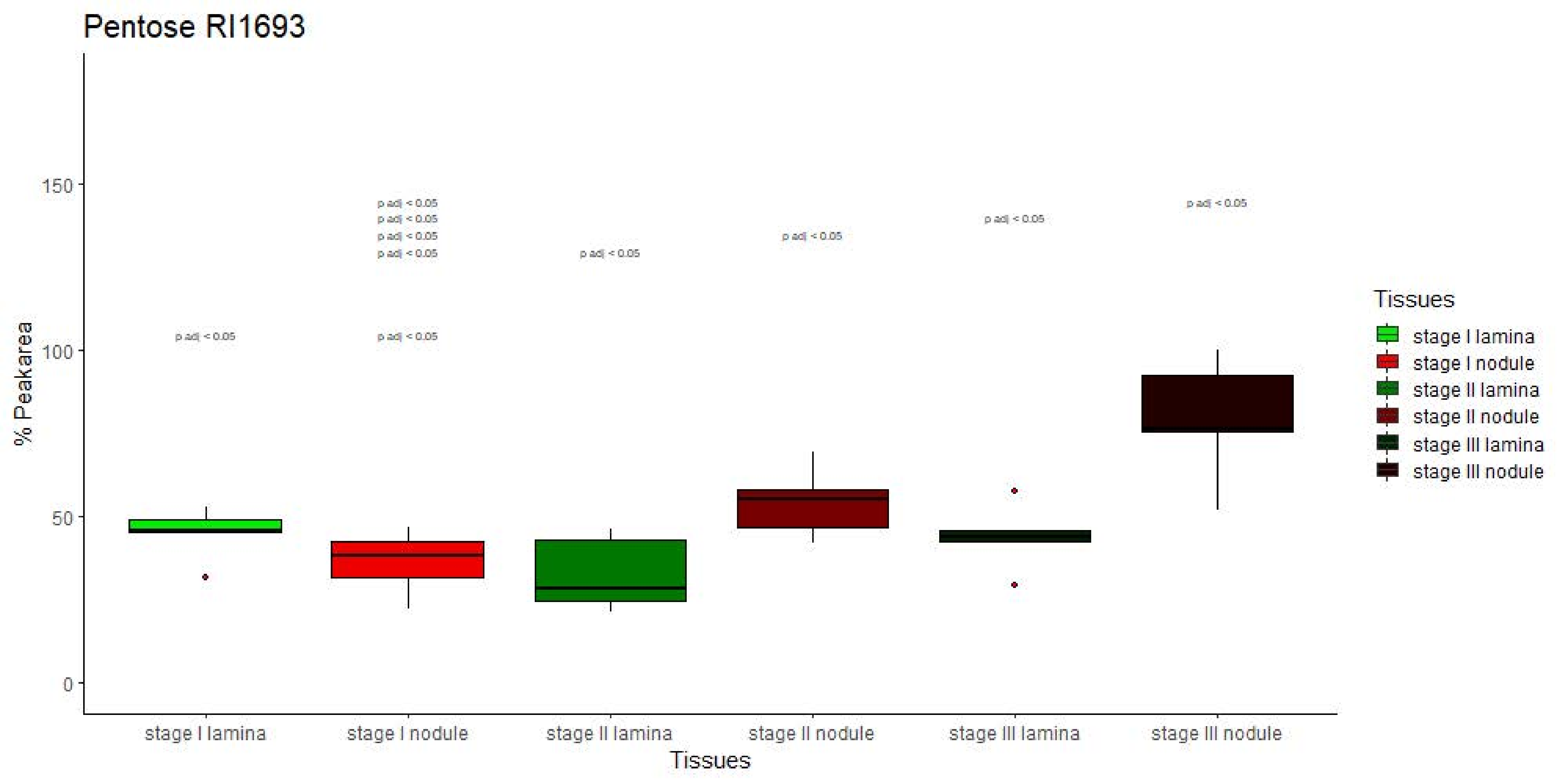

Pentose RI1693
% Peakarea
150
100
50
0
p adj < 0.05
p adj < 0.05
p adj < 0.05
p adj < 0.05
p adj < 0.05
p adj < 0.05
p adj < 0.05
p adj < 0.05
p adj < 0.05
p adj < 0.05
stage I lamina
stage I nodule
stage II lamina
stage II nodule
stage III lamina
stage III nodule
Tissues
Tissues
stage I lamina
stage I nodule
stage II lamina
stage II nodule
stage III lamina
stage III nodule

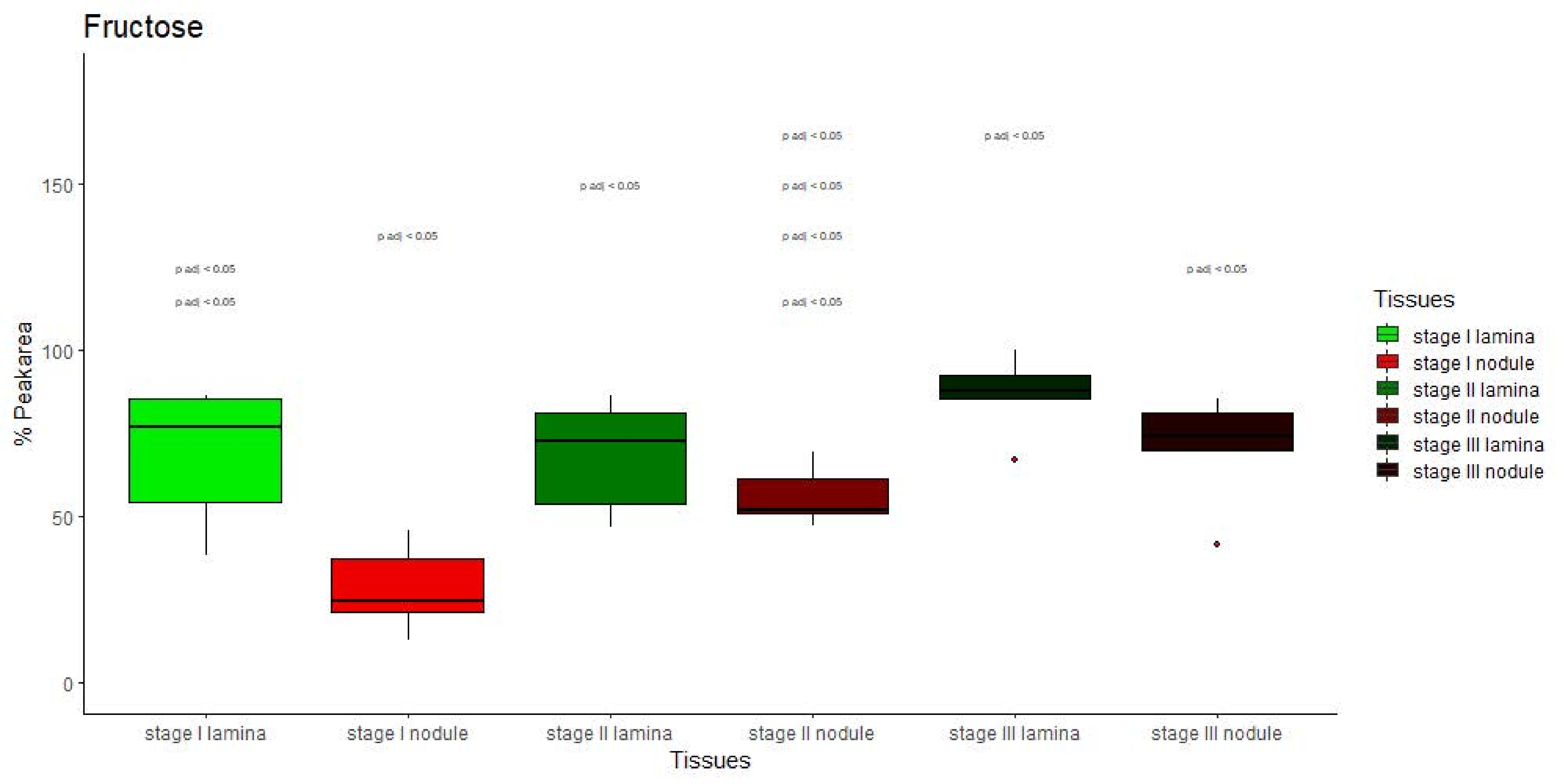

Fructose
% Peakarea
150
100
50
0
p adj < 0.05
p adj < 0.05
p adj < 0.05
p adj < 0.05
p adj < 0.05
p adj < 0.05
p adj < 0.05
p adj < 0.05
p adj < 0.05
p adj < 0.05
stage I lamina
stage I nodule
stage II lamina
stage II nodule
stage III lamina
stage III nodule
Tissues
Tissues
stage I lamina
stage I nodule
stage II lamina
stage II nodule
stage III lamina
stage III nodule

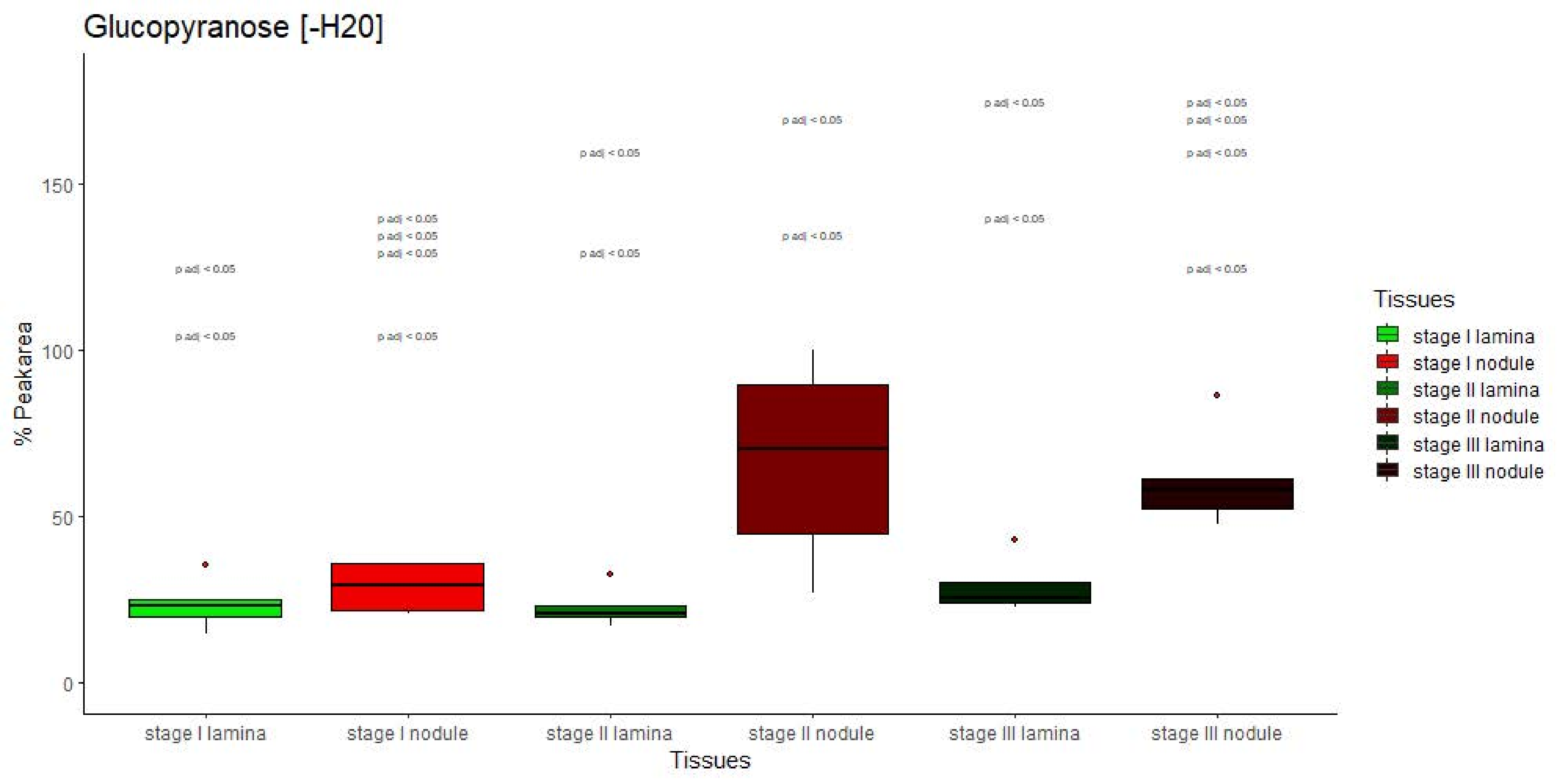
Glucopyranose [-H20]
% Peakarea
150
100
50
0
p adj < 0.05
stage I lamina
stage I nodule
stage II lamina
stage II nodule
stage III lamina
stage III nodule
Tissues
Tissues
stage I lamina
stage I nodule
stage II lamina
stage II nodule
stage III lamina
stage III nodule

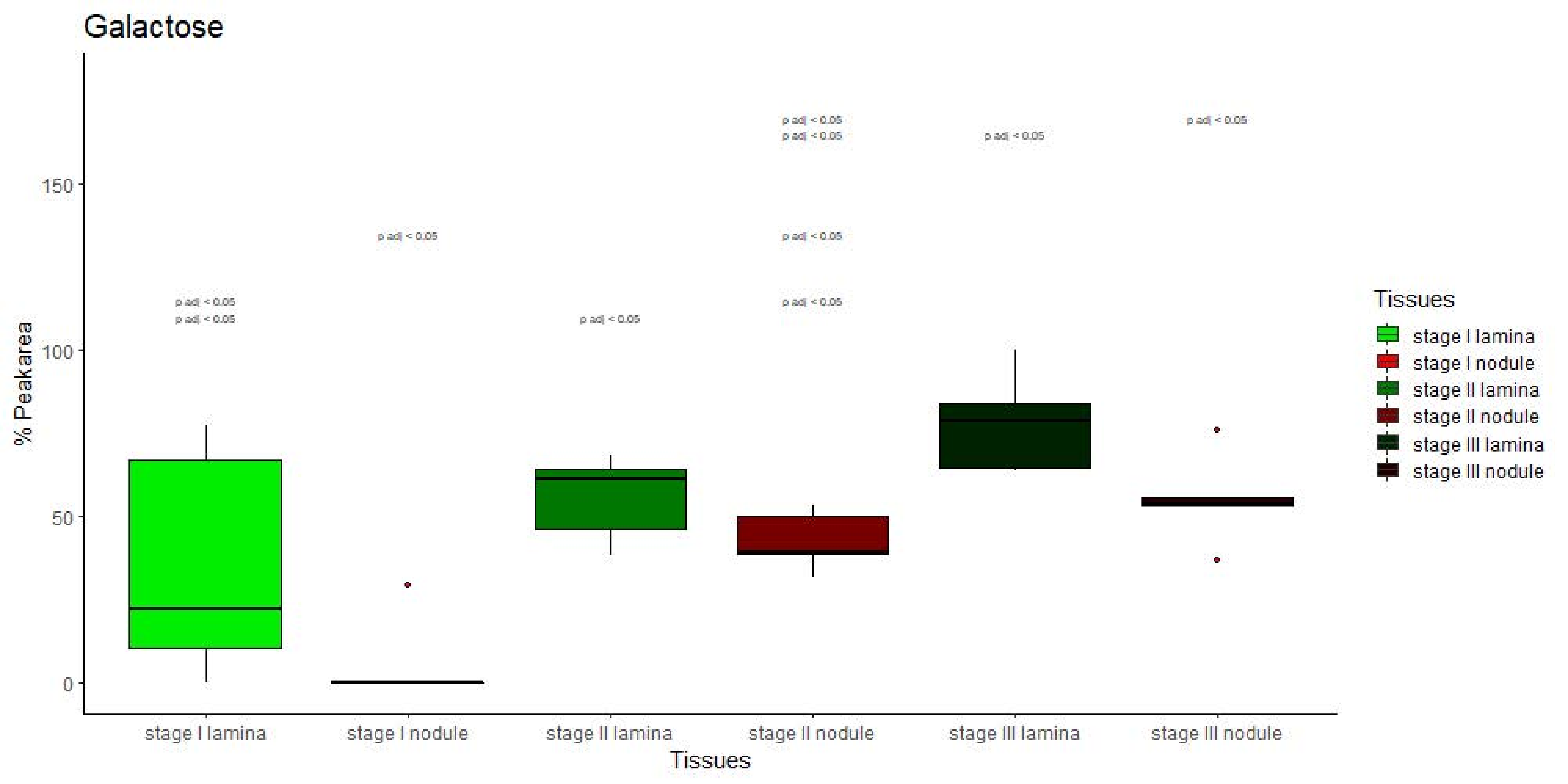

Galactose
% Peakarea
150
100
50
0
p adj < 0.05
p adj < 0.05
p adj < 0.05
p adj < 0.05
p adj < 0.05
p adj < 0.05
p adj < 0.05
p adj < 0.05
p adj < 0.05
p adj < 0.05
stage I lamina
stage I nodule
stage II lamina
stage II nodule
stage III lamina
stage III nodule
Tissues
Tissues
stage I lamina
stage I nodule
stage II lamina
stage II nodule
stage III lamina
stage III nodule

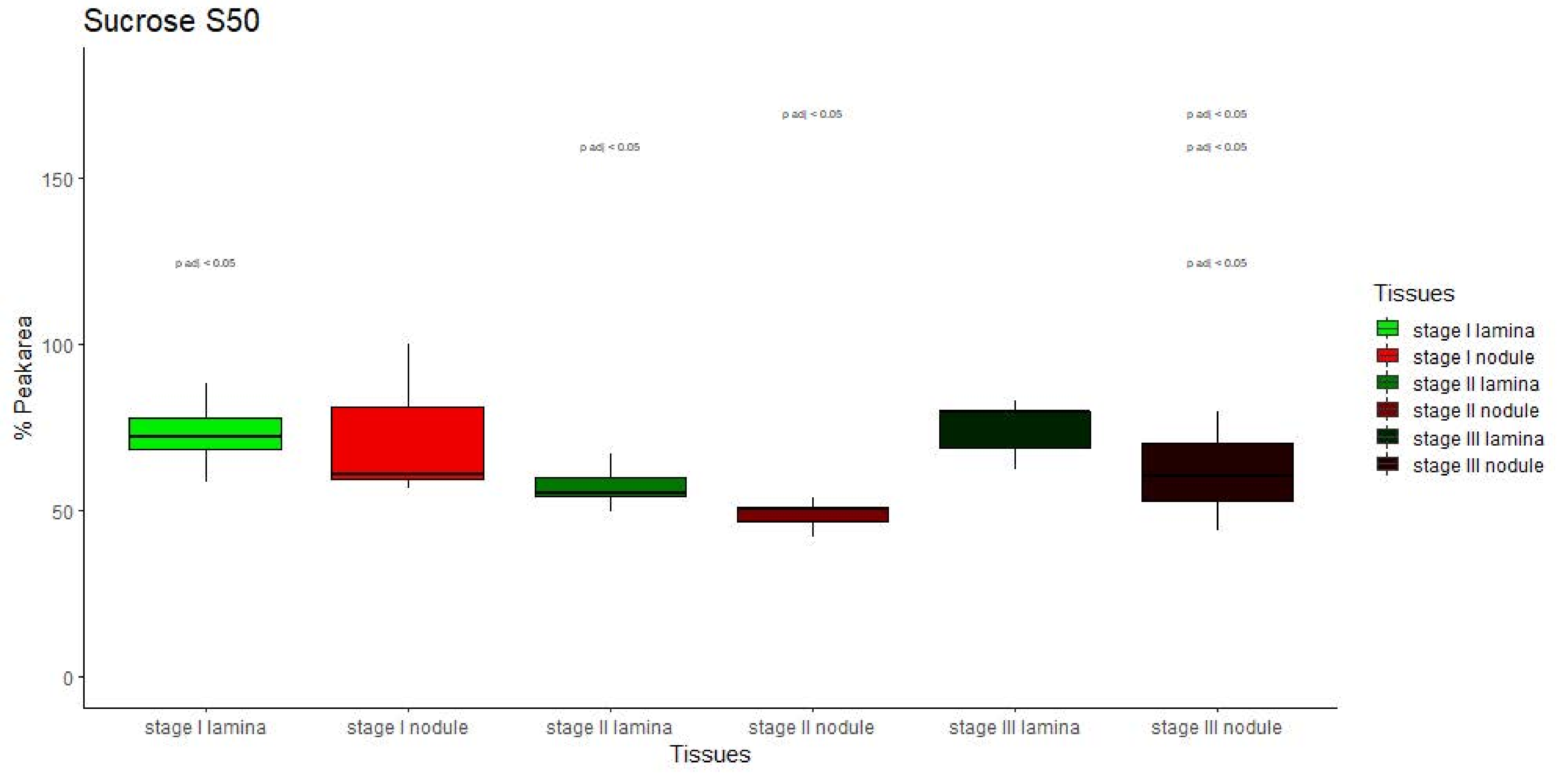
Sucrose S50
% Peakarea
150
100
50
0
p adj < 0.05
p adj < 0.05
p adj < 0.05
p adj < 0.05
p adj < 0.05
p adj < 0.05
stage I lamina
stage I nodule
stage II lamina
stage II nodule
stage III lamina
stage III nodule
Tissues
Tissues
stage I lamina
stage I nodule
stage II lamina
stage II nodule
stage III lamina
stage III nodule

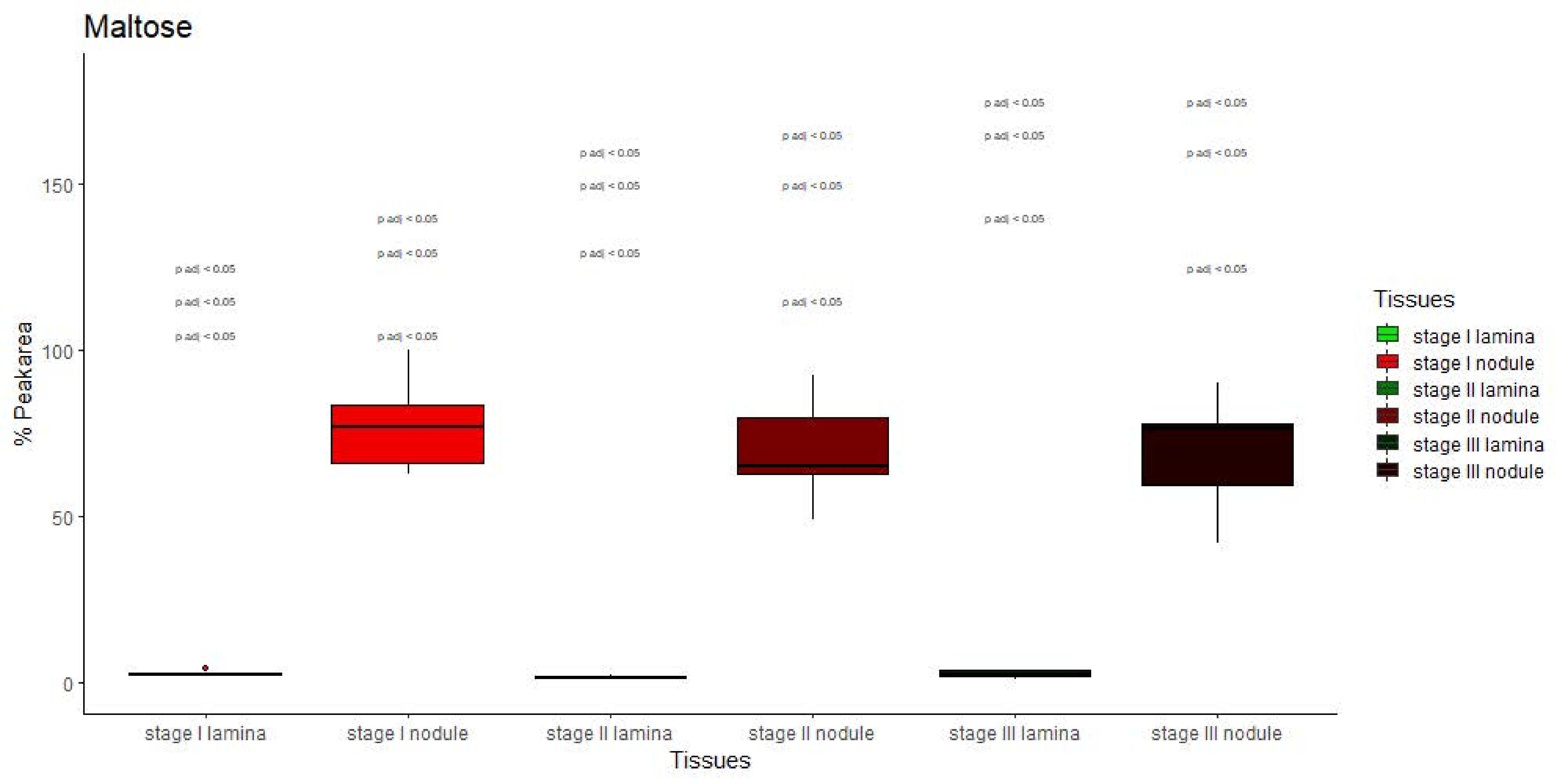
Maltose
% Peakarea
150
100
50
0
p adj < 0.05
stage I lamina
stage I nodule
stage II lamina
stage II nodule
stage III lamina
stage III nodule
Tissues

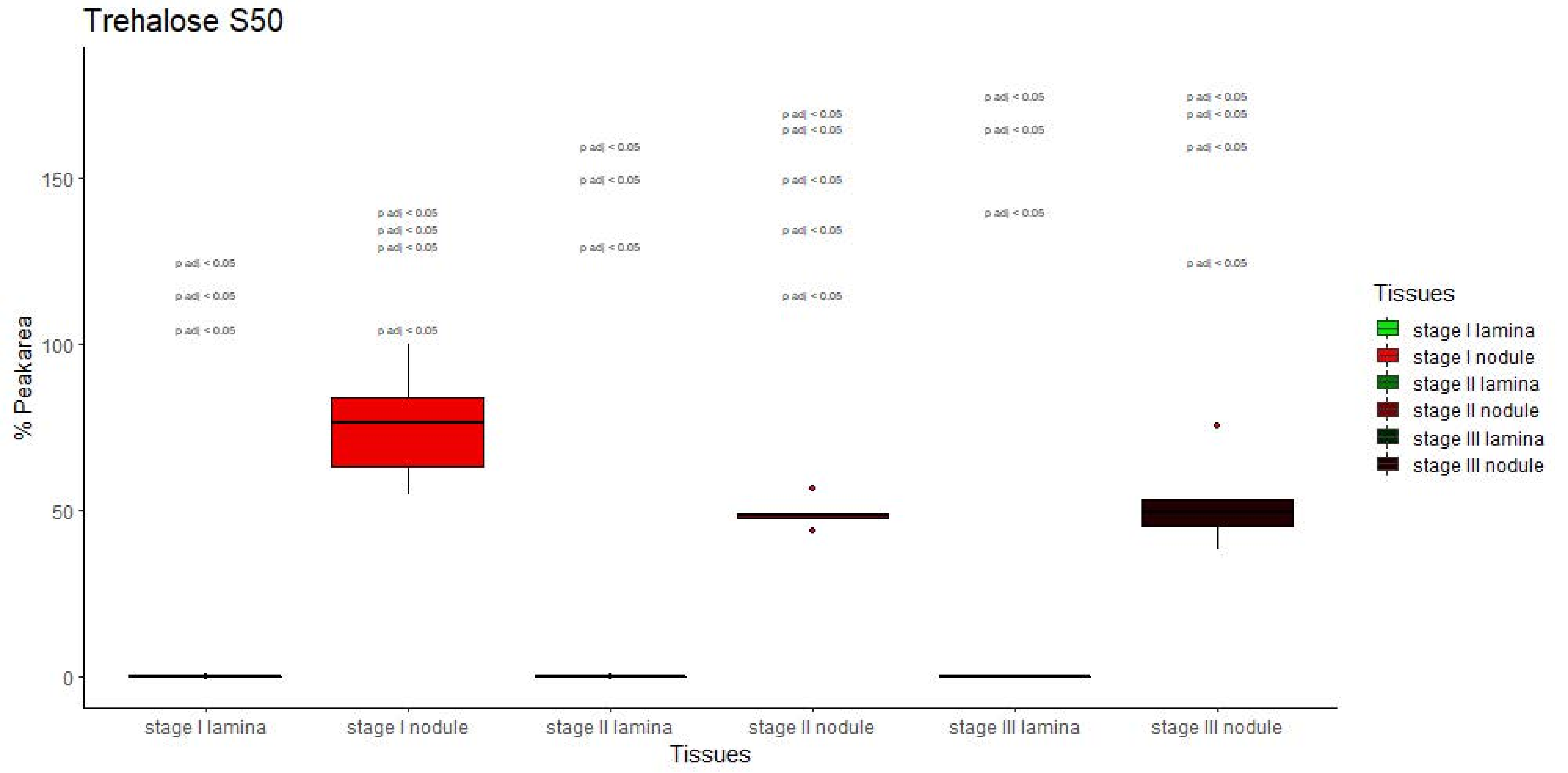
Trehalose S50
% Peakarea
0
50
100
150
p adj < 0.05
stage I lamina
stage I nodule
stage II lamina
stage II nodule
stage III lamina
stage III nodule
Tissues
Tissues
stage I lamina
stage I nodule
stage II lamina
stage II nodule
stage III lamina
stage III nodule

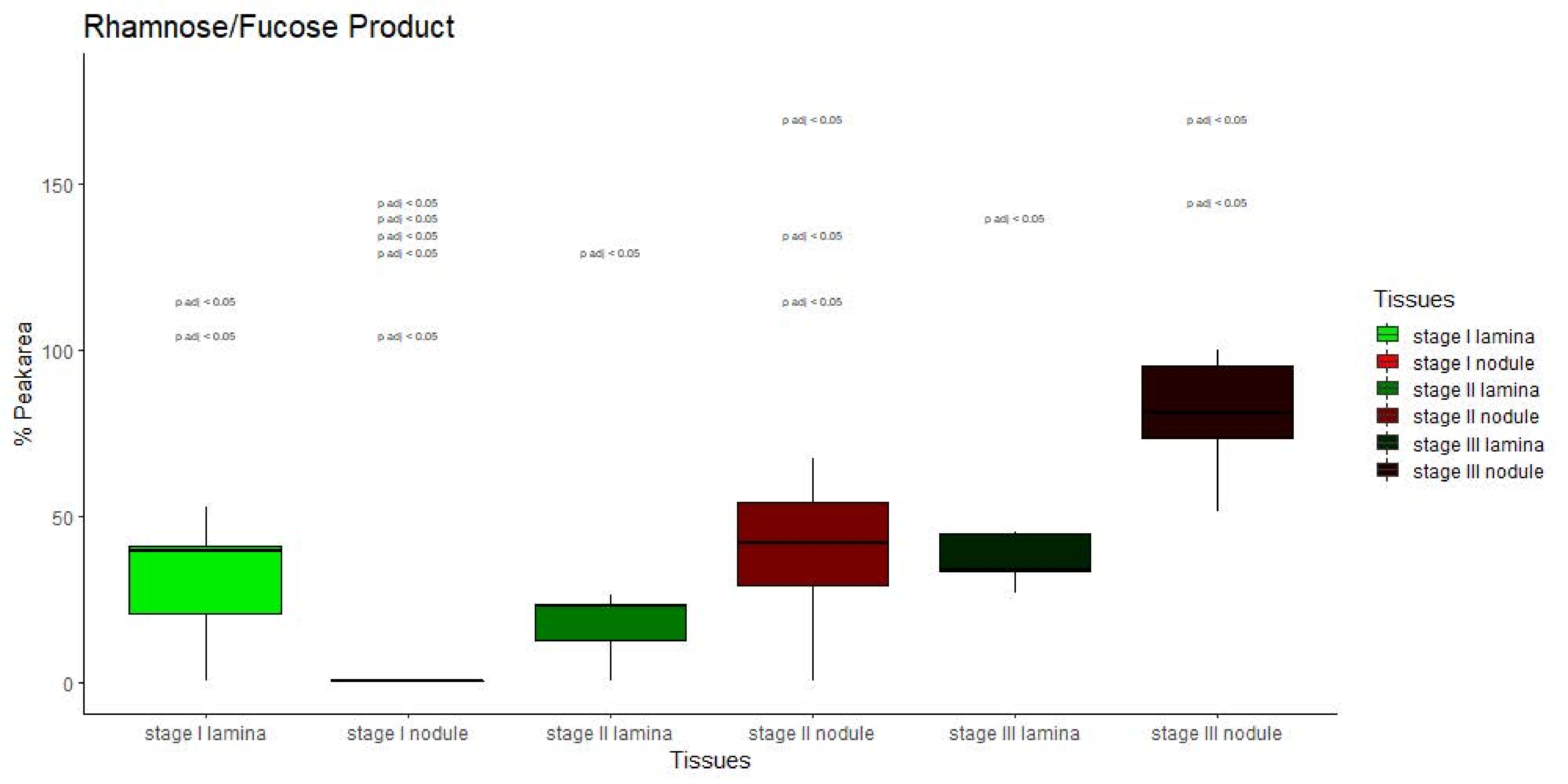

Rhamnose/Fucose Product
% Peakarea
0
50
100
150
p adj < 0.05
Tissues
stage I lamina
stage I nodule
stage II lamina
stage II nodule
stage III lamina
stage III nodule

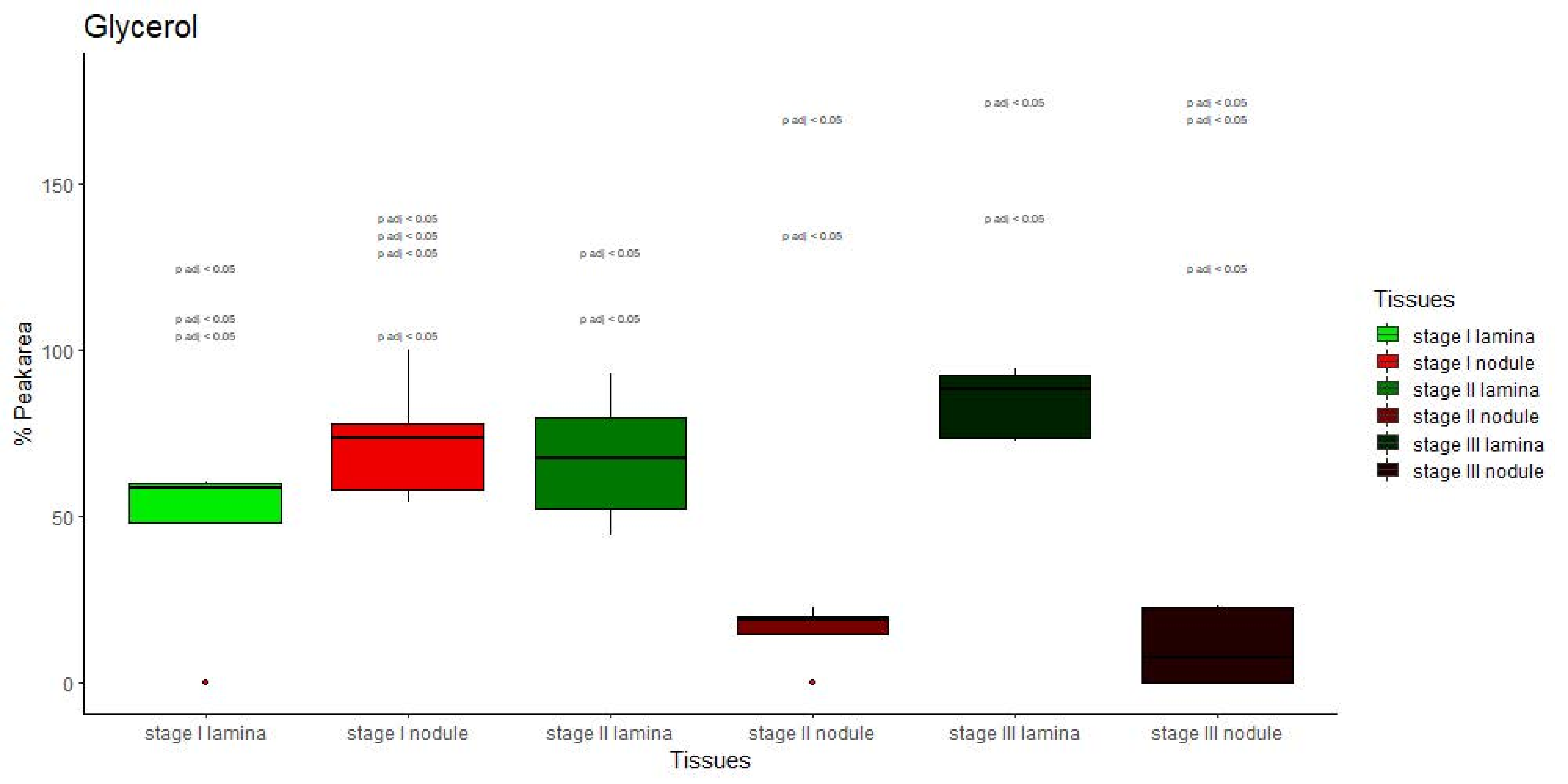
Glycerol
% Peakarea
150
100
50
0
p adj < 0.05
stage I lamina
stage I nodule
stage II lamina
stage II nodule
stage III lamina
stage III nodule
Tissues
Tissues
stage I lamina
stage I nodule
stage II lamina
stage II nodule
stage III lamina
stage III nodule

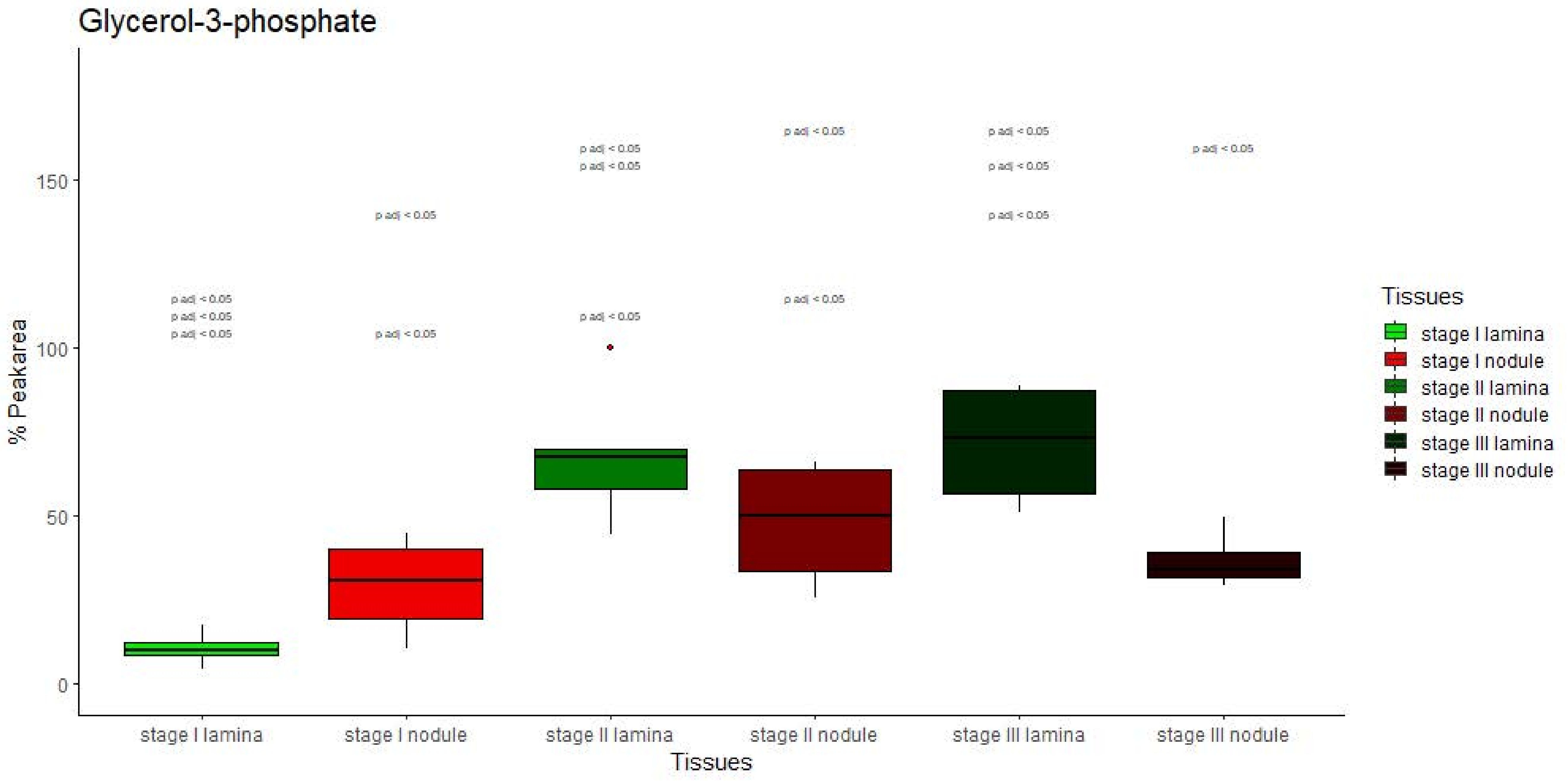
Glycerol-3-phosphate
% Peakarea
150
100
50
0
p adj < 0.05
stage I lamina
stage I nodule
stage II lamina
stage II nodule
stage III lamina
stage III nodule
Tissues
Tissues
stage I lamina
stage I nodule
stage II lamina
stage II nodule
stage III lamina
stage III nodule

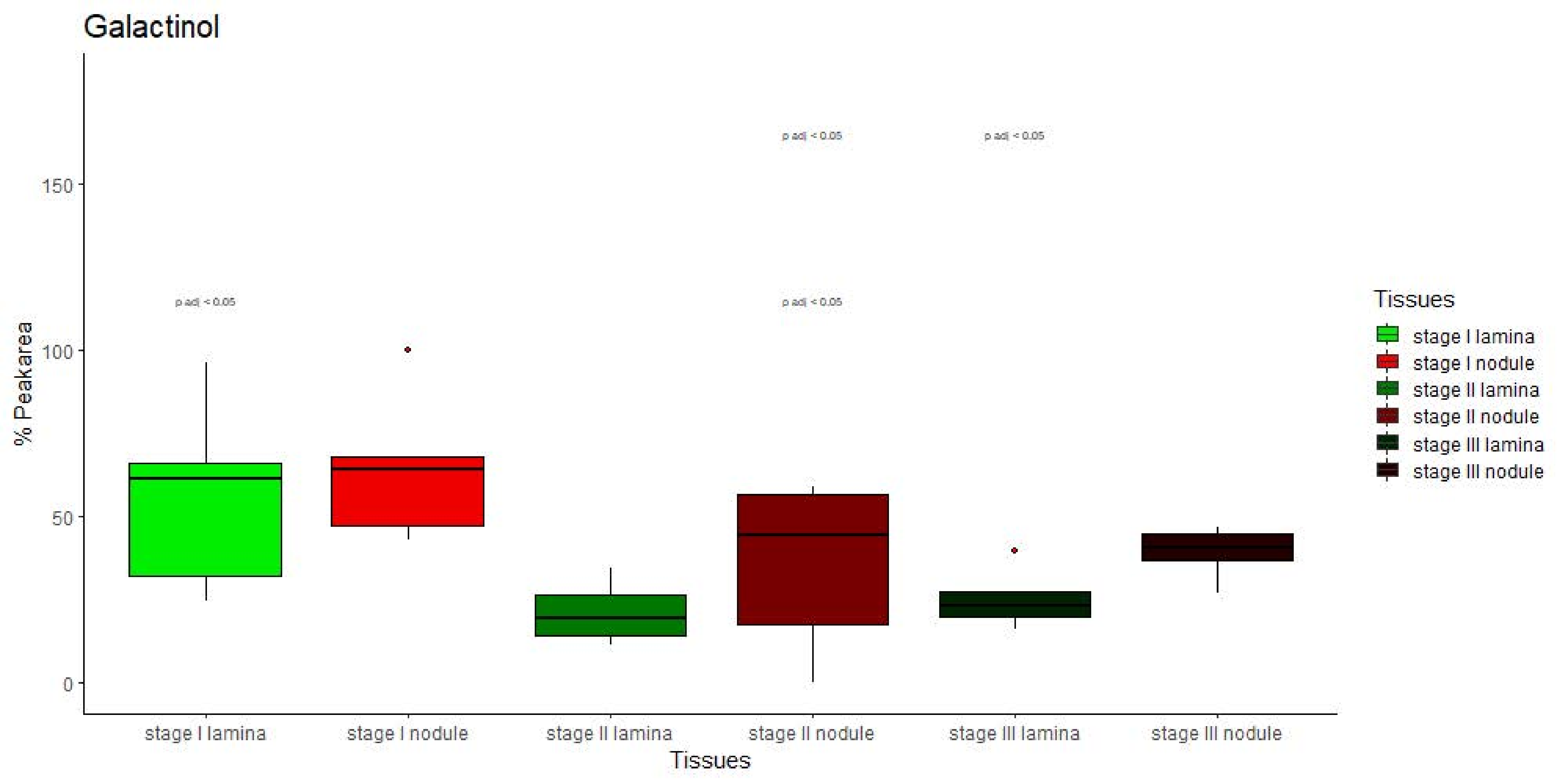
Galactinol
p adj < 0.05
p adj < 0.05
p adj < 0.05
p adj < 0.05
% Peakarea
150
100
50
0
stage I lamina
stage I nodule
stage II lamina
stage II nodule
stage III lamina
stage III nodule
Tissues
Tissues
stage I lamina
stage I nodule
stage II lamina
stage II nodule
stage III lamina
stage III nodule

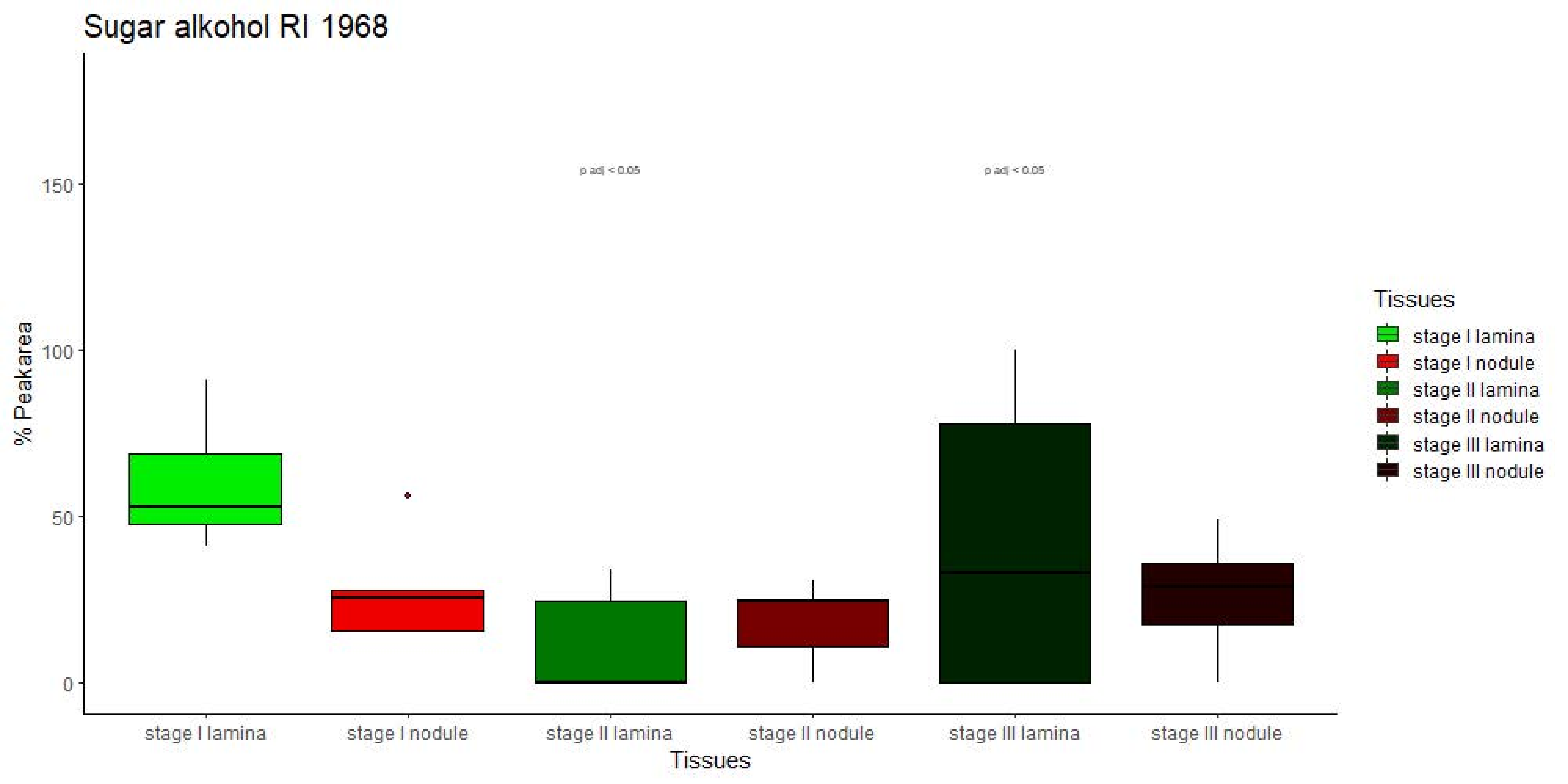

Sugar alkohol RI 1968
p adj < 0.05
p adj < 0.05
150
100
50
0
% Peakarea
stage I lamina
stage I nodule
stage II lamina
stage II nodule
stage III lamina
stage III nodule
Tissues
Tissues
stage I lamina
stage I nodule
stage II lamina
stage II nodule
stage III lamina
stage III nodule

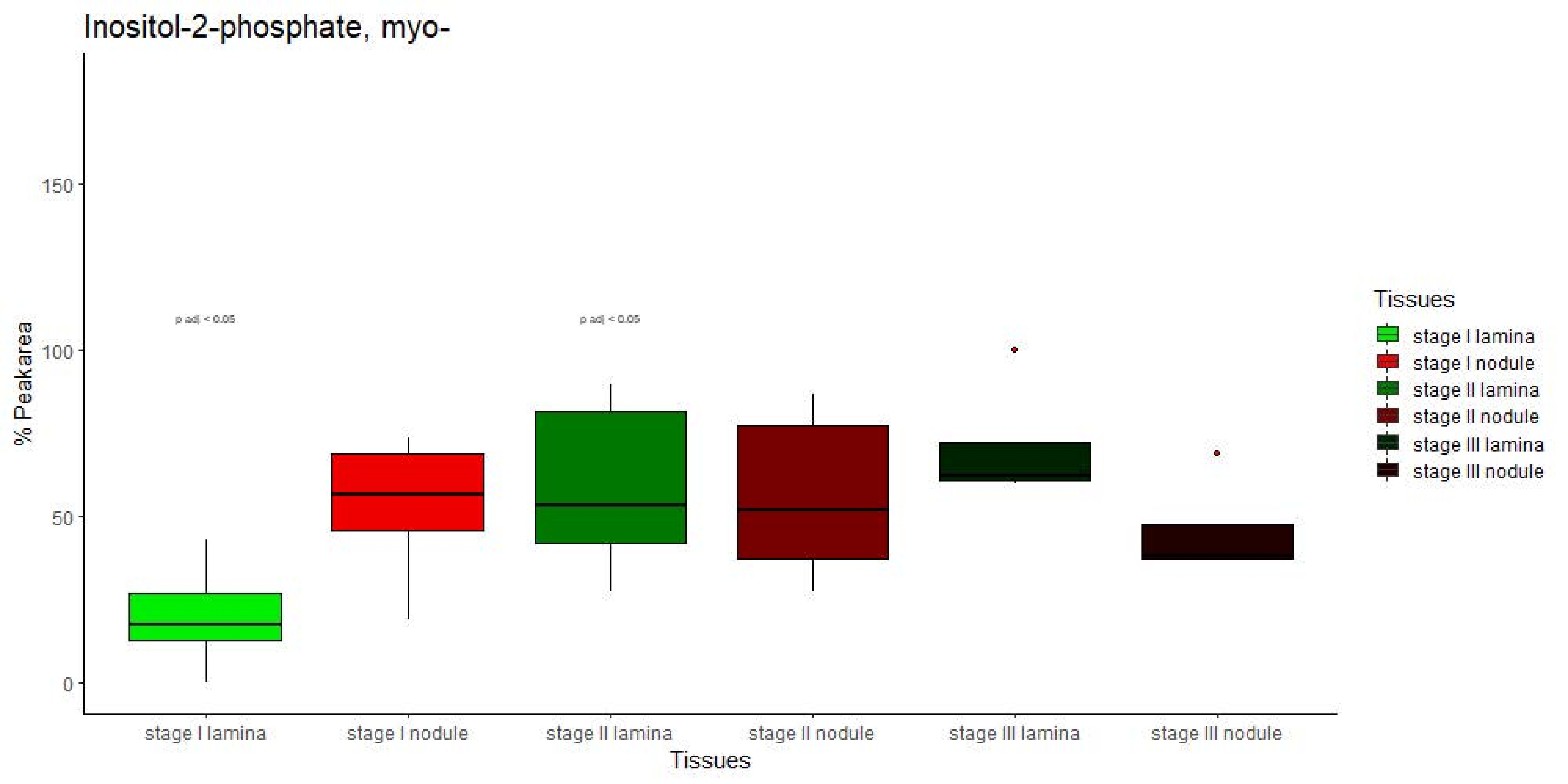

Inositol-2-phosphate, myo-
% Peakarea
150
100
50
0
p adj < 0.05
p adj < 0.05
stage I lamina
stage I nodule
stage II lamina
stage II nodule
stage III lamina
stage III nodule
Tissues
Tissues
stage I lamina
stage I nodule
stage II lamina
stage II nodule
stage III lamina
stage III nodule

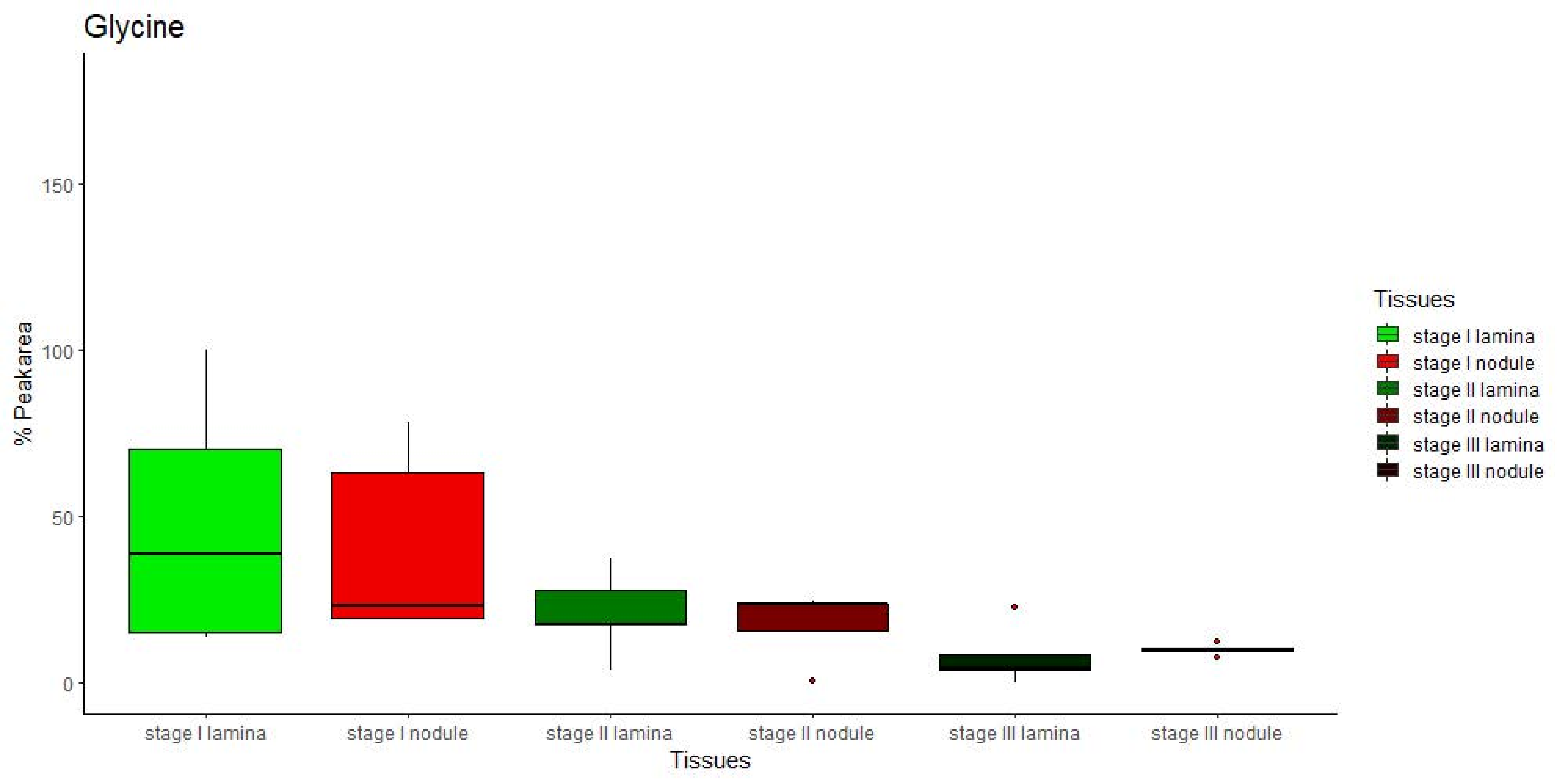
Glycine
% Peakarea
150
100
50
0
stage I lamina
stage I nodule
stage II lamina
stage II nodule
stage III lamina
stage III nodule
Tissues
Tissues
stage I lamina
stage I nodule
stage II lamina
stage II nodule
stage III lamina
stage III nodule

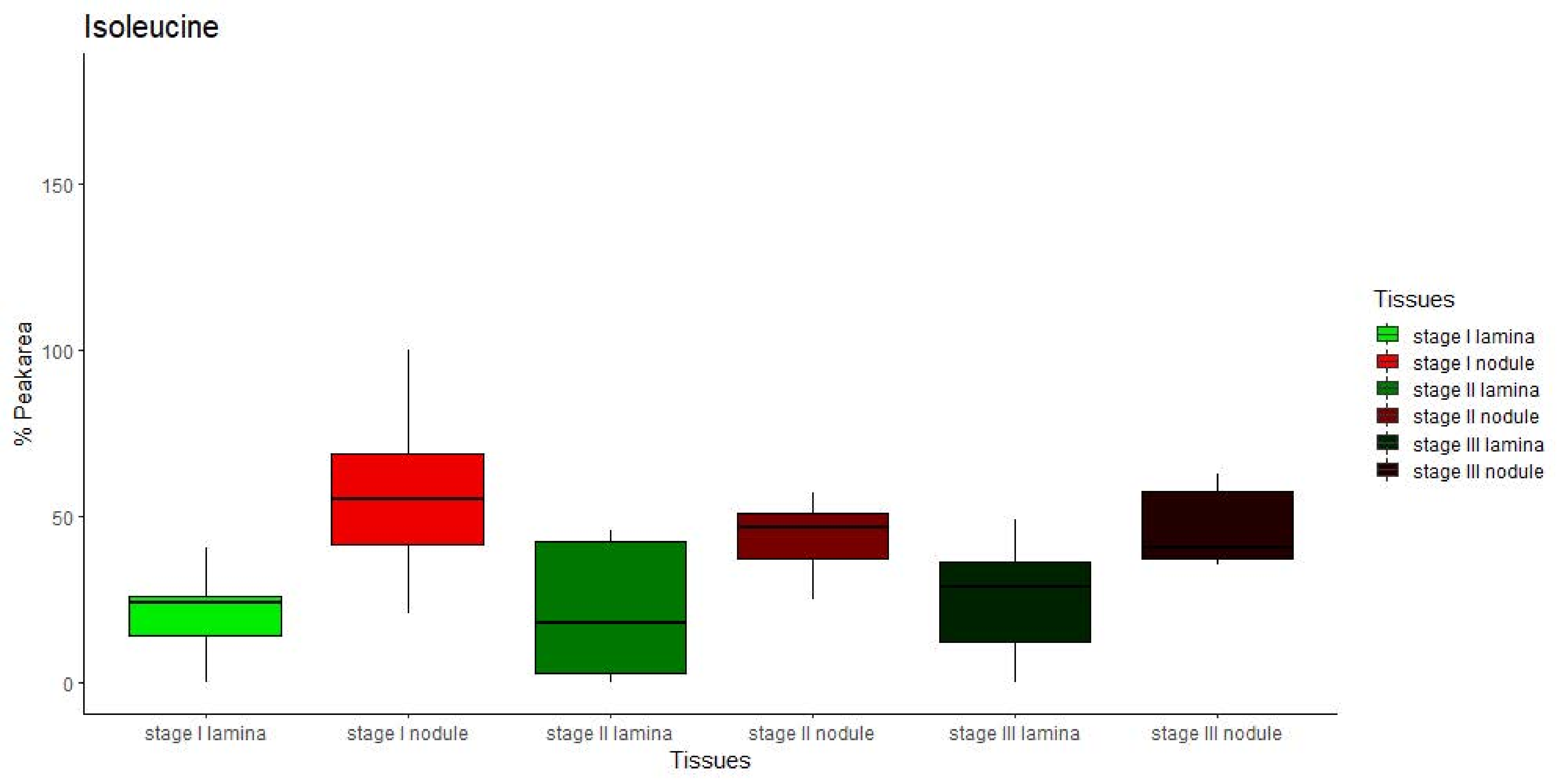

Isoleucine
% Peakarea
0
50
100
150
stage I lamina
stage I nodule
stage II lamina
stage II nodule
stage III lamina
stage III nodule
Tissues
Tissues
stage I lamina
stage I nodule
stage II lamina
stage II nodule
stage III lamina
stage III nodule

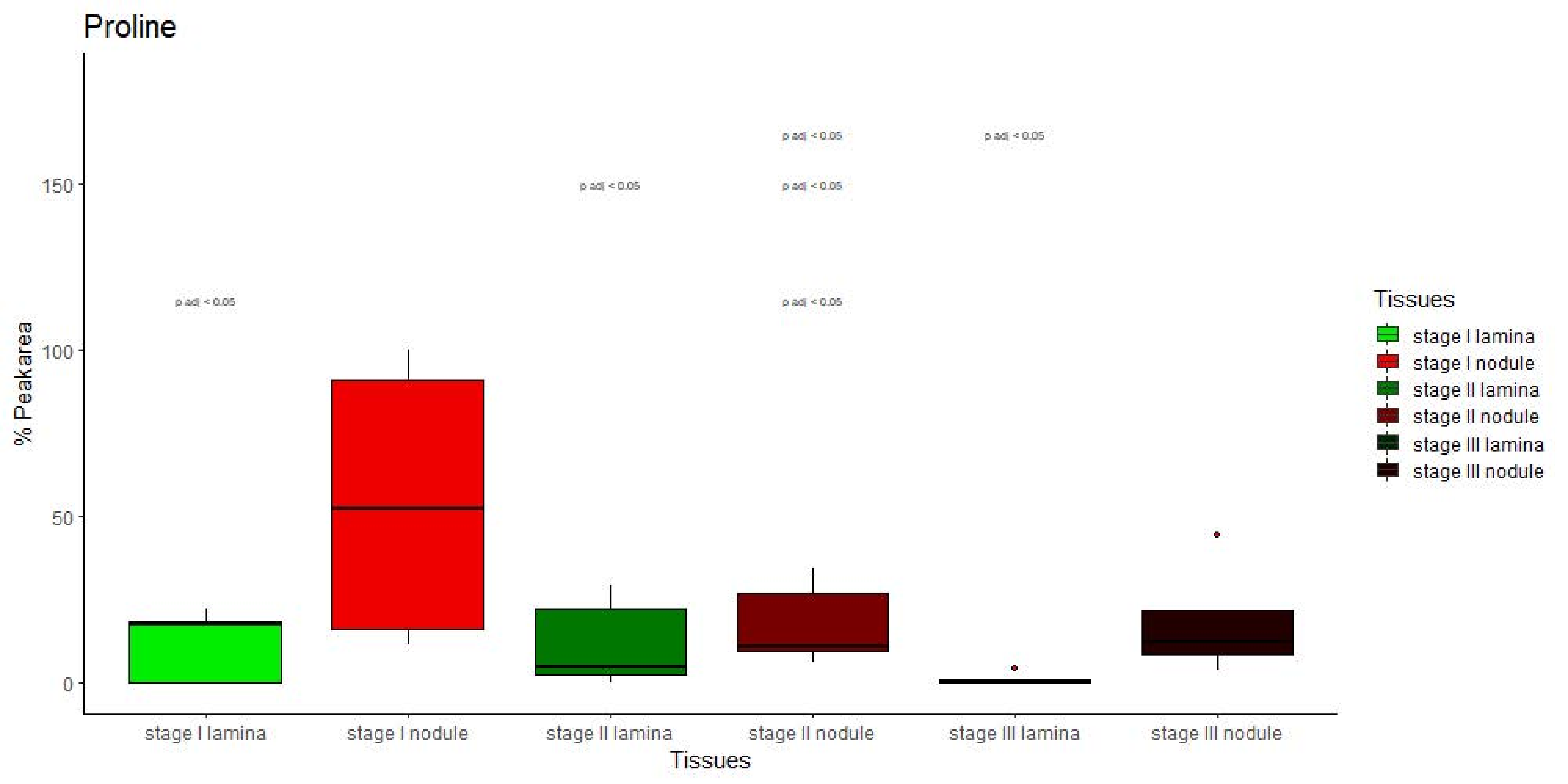
Proline
% Peakarea
150
100
50
0
p adj < 0.05
p adj < 0.05
p adj < 0.05
p adj < 0.05
p adj < 0.05
p adj < 0.05
stage I lamina
stage I nodule
stage II lamina
stage II nodule
stage III lamina
stage III nodule
Tissues
Tissues
stage I lamina
stage I nodule
stage II lamina
stage II nodule
stage III lamina
stage III nodule

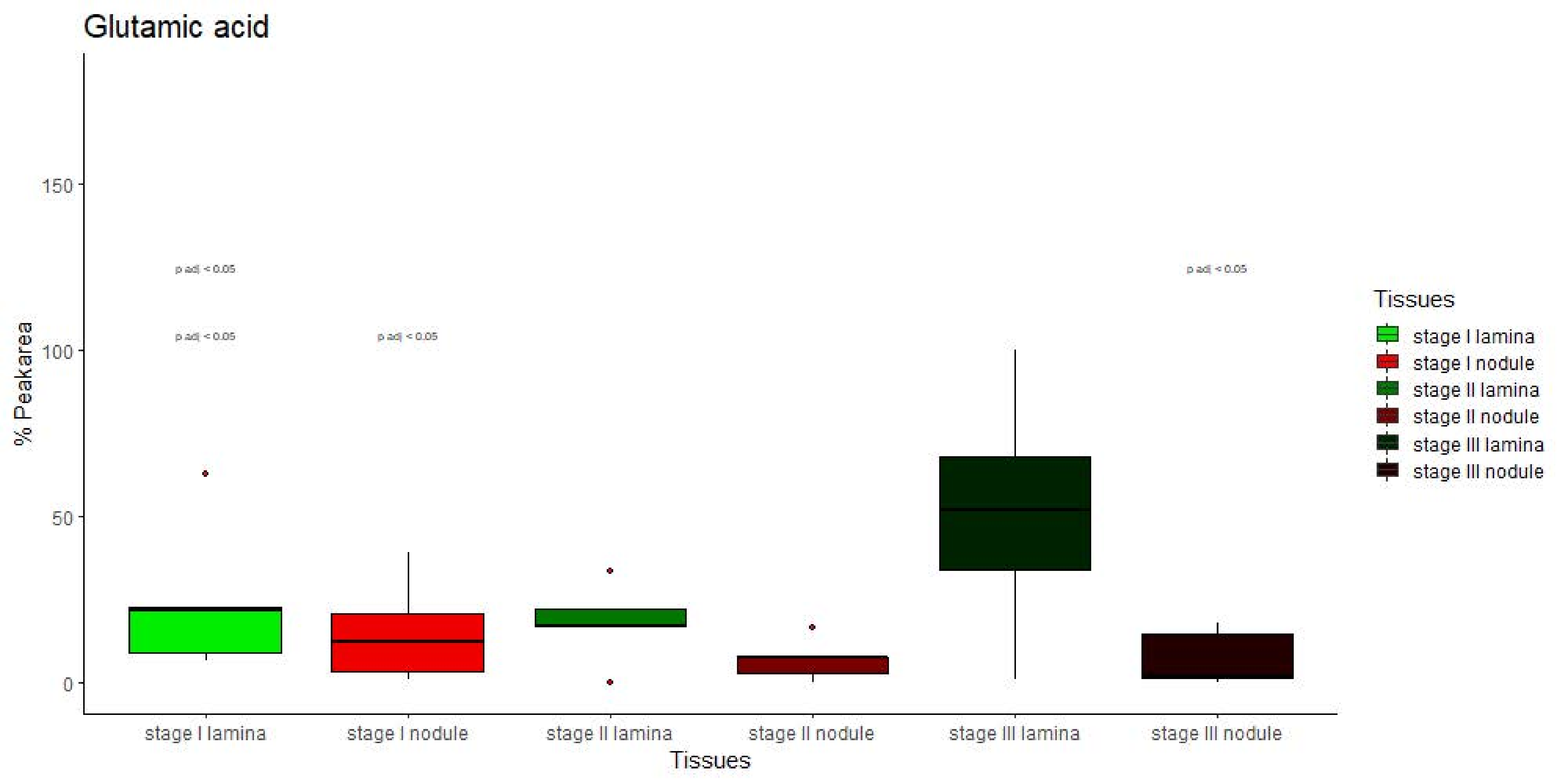

Glutamic acid
% Peakarea
150
100
50
0
p adj < 0.05
p adj < 0.05
p adj < 0.05
p adj < 0.05
stage I lamina
stage I nodule
stage II lamina
stage II nodule
stage III lamina
stage III nodule
Tissues
Tissues
stage I lamina
stage I nodule
stage II lamina
stage II nodule
stage III lamina
stage III nodule

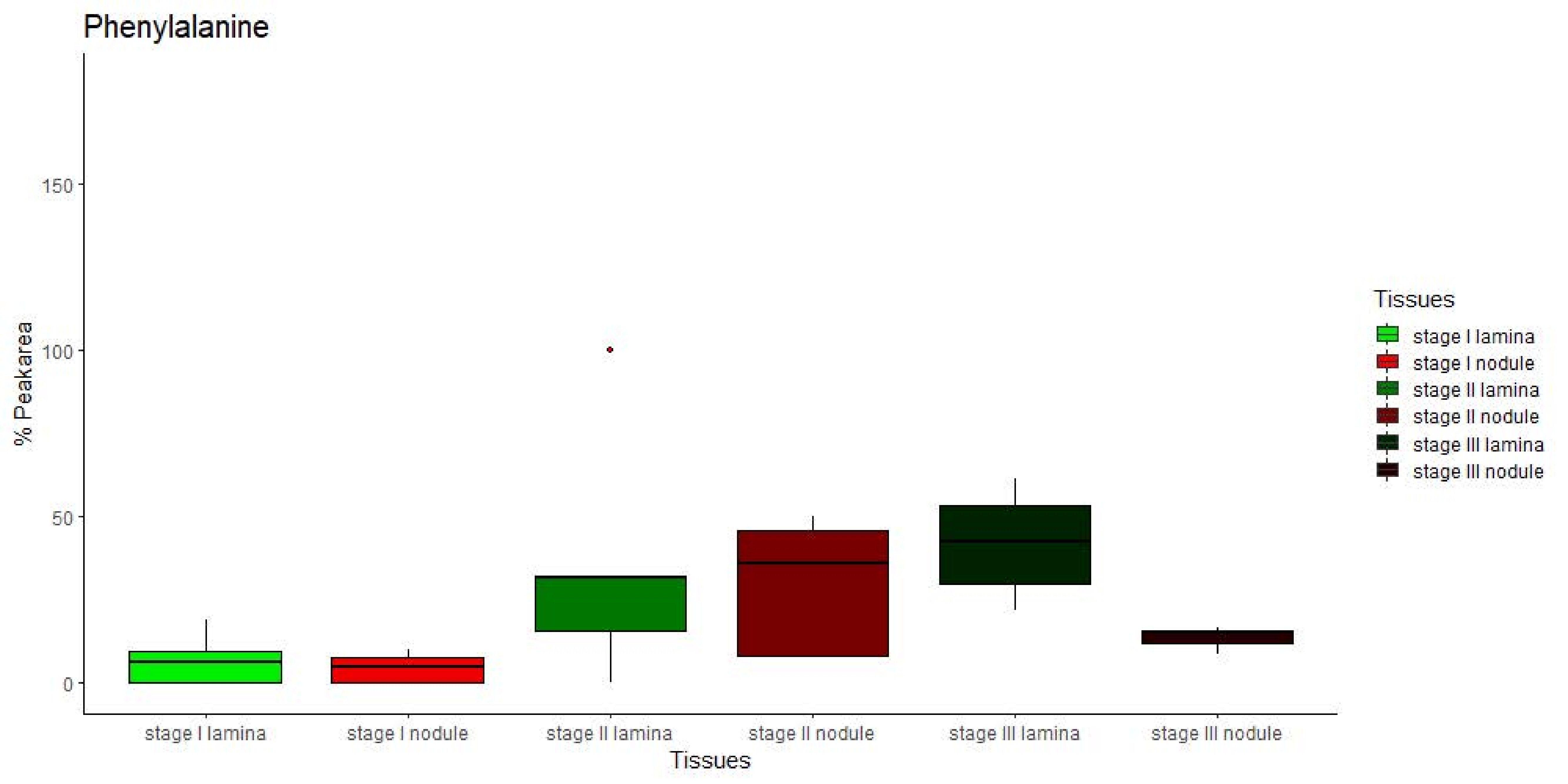
Phenylalanine
% Peakarea
150
100
50
0
stage I lamina
stage I nodule
stage II lamina
stage II nodule
stage III lamina
stage III nodule
Tissues
Tissues
stage I lamina
stage I nodule
stage II lamina
stage II nodule
stage III lamina
stage III nodule

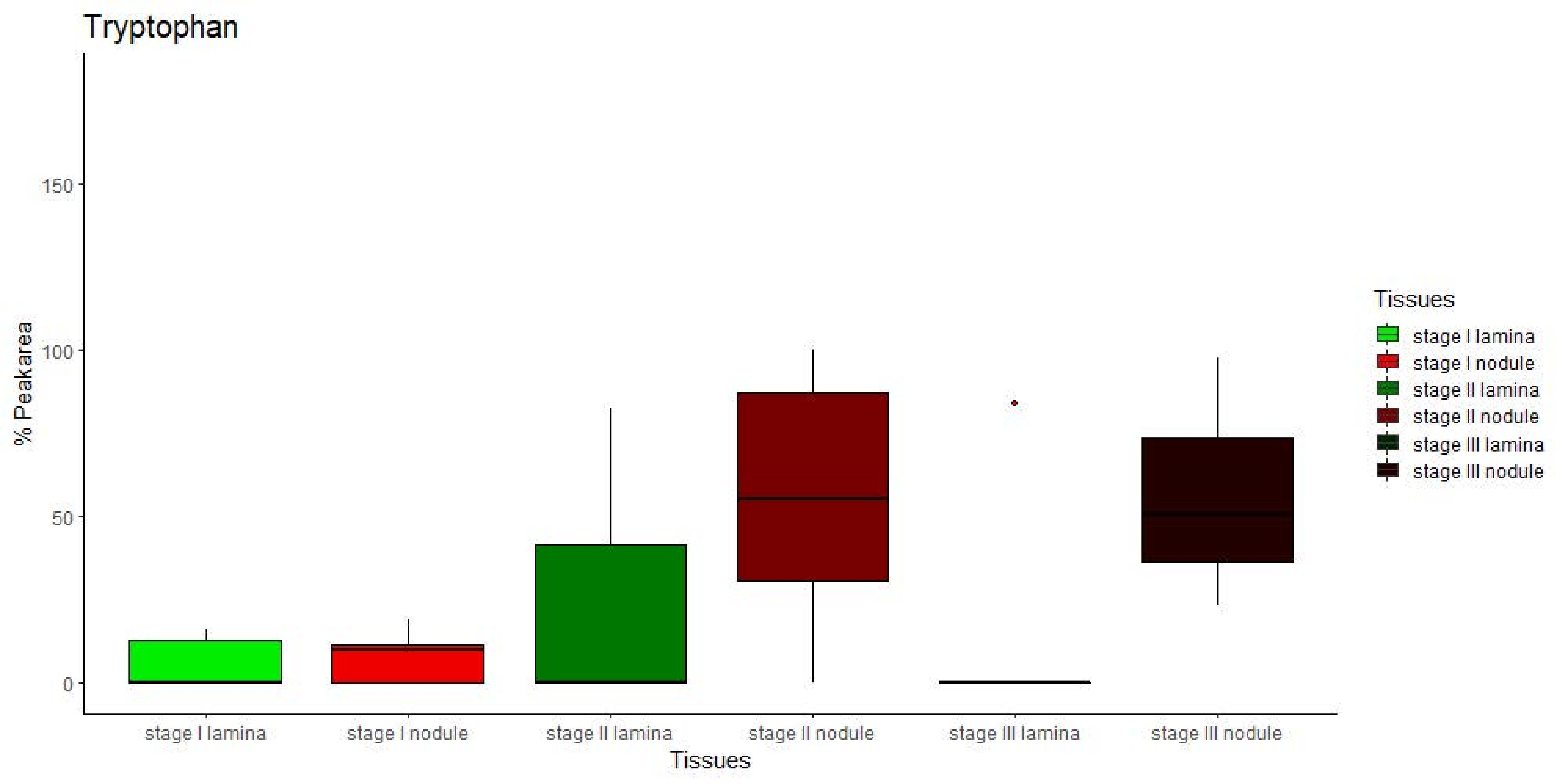
Tryptophan
% Peakarea
150
100
50
0
stage I lamina
stage I nodule
stage II lamina
stage II nodule
stage III lamina
stage III nodule
Tissues
Tissues
stage I lamina
stage I nodule
stage II lamina
stage II nodule
stage III lamina
stage III nodule

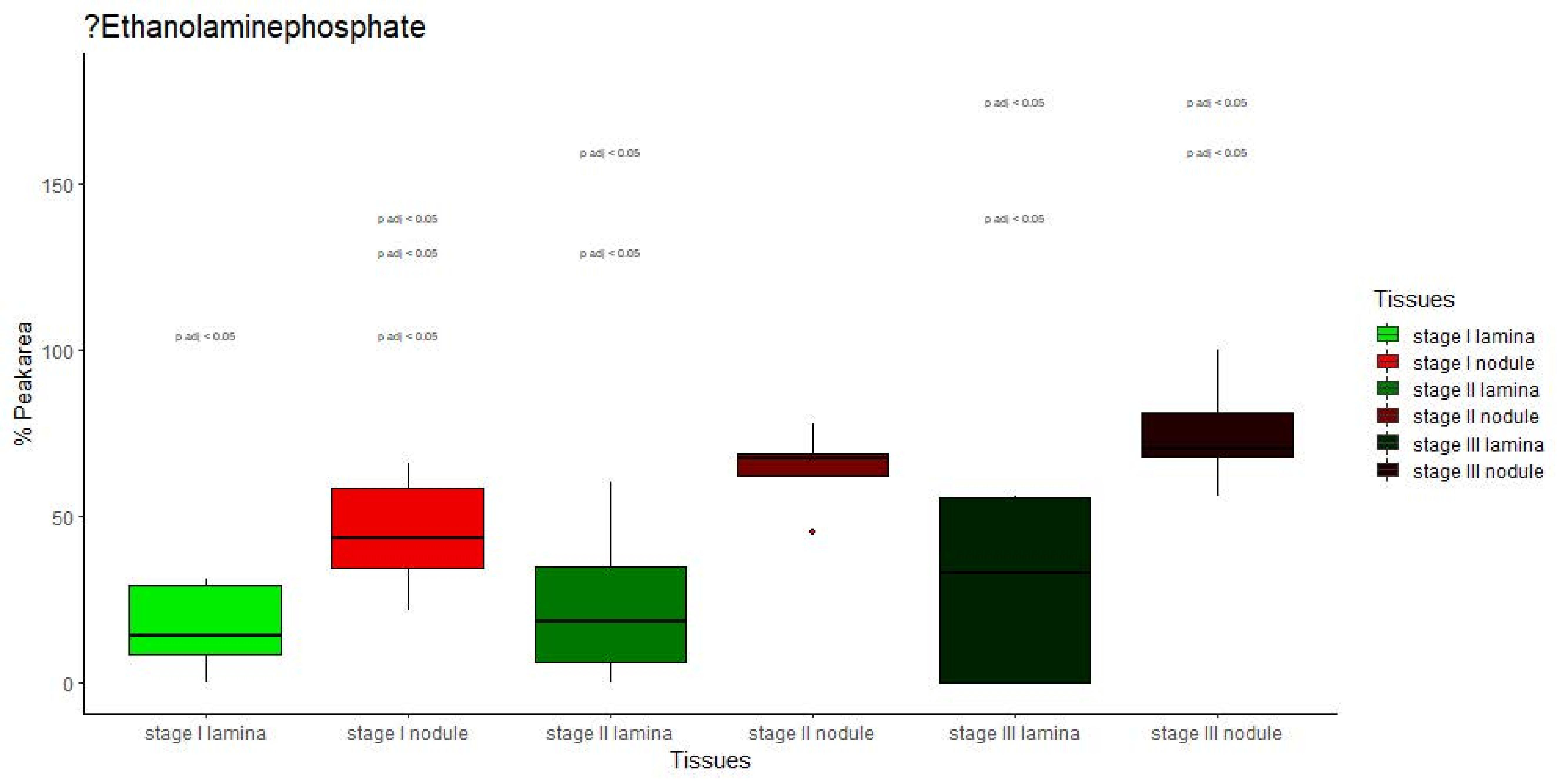

?Ethanolaminephosphate
% Peakarea
150
100
50
0
p adj < 0.05
stage I lamina
stage I nodule
stage II lamina
stage II nodule
stage III lamina
stage III nodule
Tissues
Tissues
stage I lamina
stage I nodule
stage II lamina
stage II nodule
stage III lamina
stage III nodule

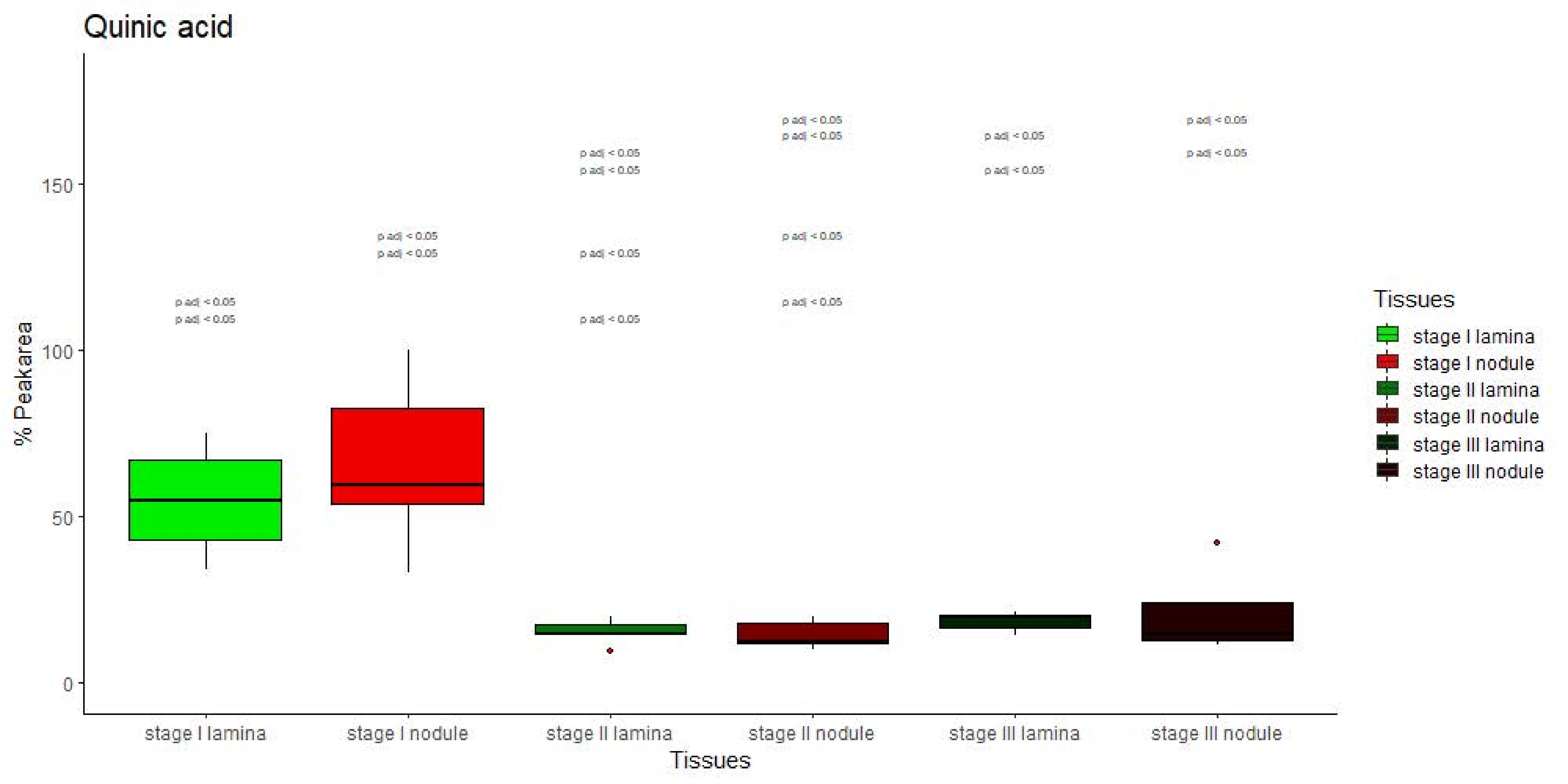
Quinic acid
% Peakarea
150
100
50
0
p adj < 0.05
stage I lamina
stage I nodule
stage II lamina
stage II nodule
stage III lamina
stage III nodule
Tissues
Tissues
stage I lamina
stage I nodule
stage II lamina
stage II nodule
stage III lamina
stage III nodule

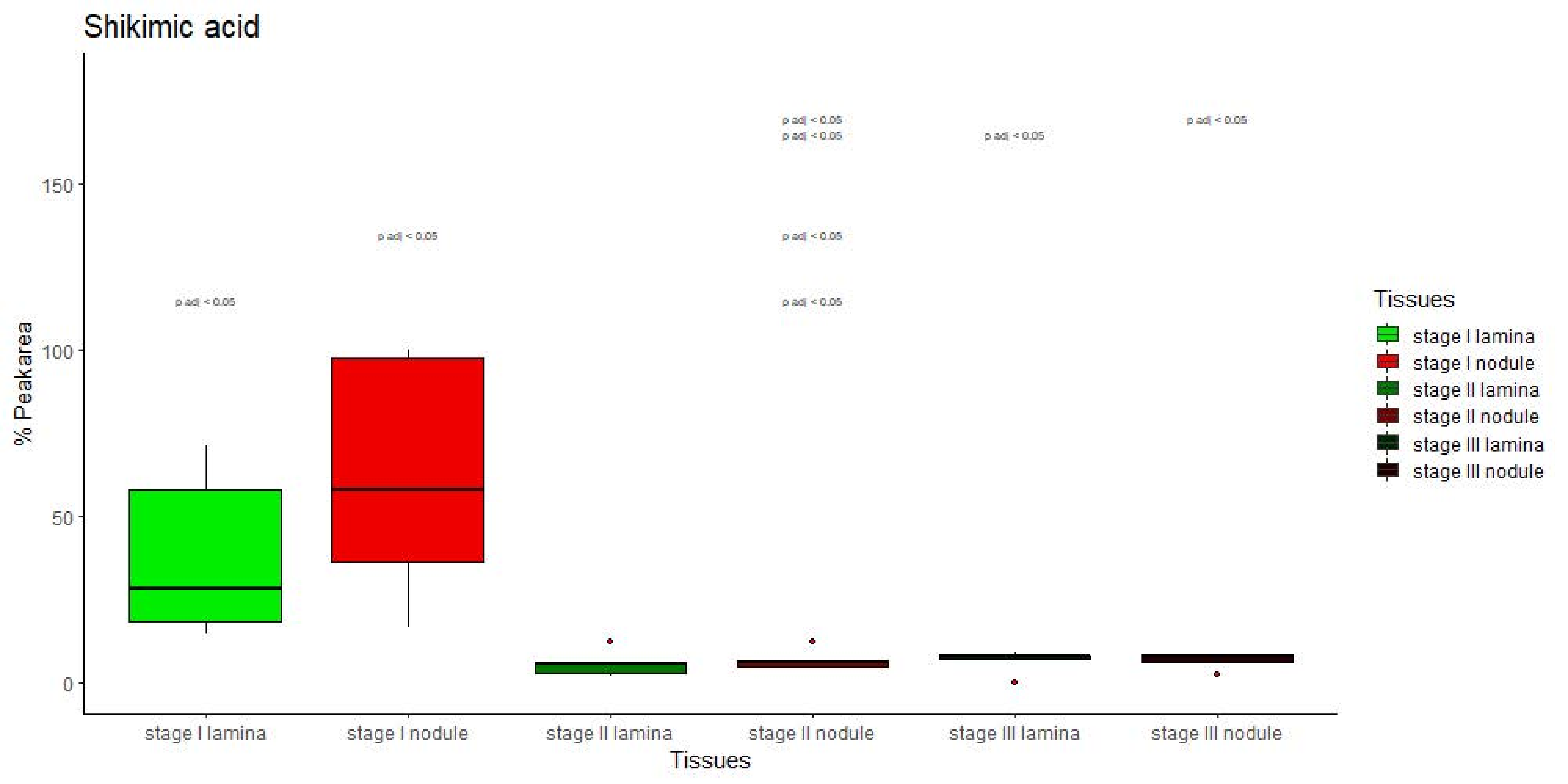
Shikimic acid
% Peakarea
0
50
100
150
p adj < 0.05
p adj < 0.05
p adj < 0.05
p adj < 0.05
p adj < 0.05
p adj < 0.05
p adj < 0.05
p adj < 0.05
stage I lamina
stage I nodule
stage II lamina
stage II nodule
stage III lamina
stage III nodule
Tissues
Tissues
stage I lamina
stage I nodule
stage II lamina
stage II nodule
stage III lamina
stage III nodule

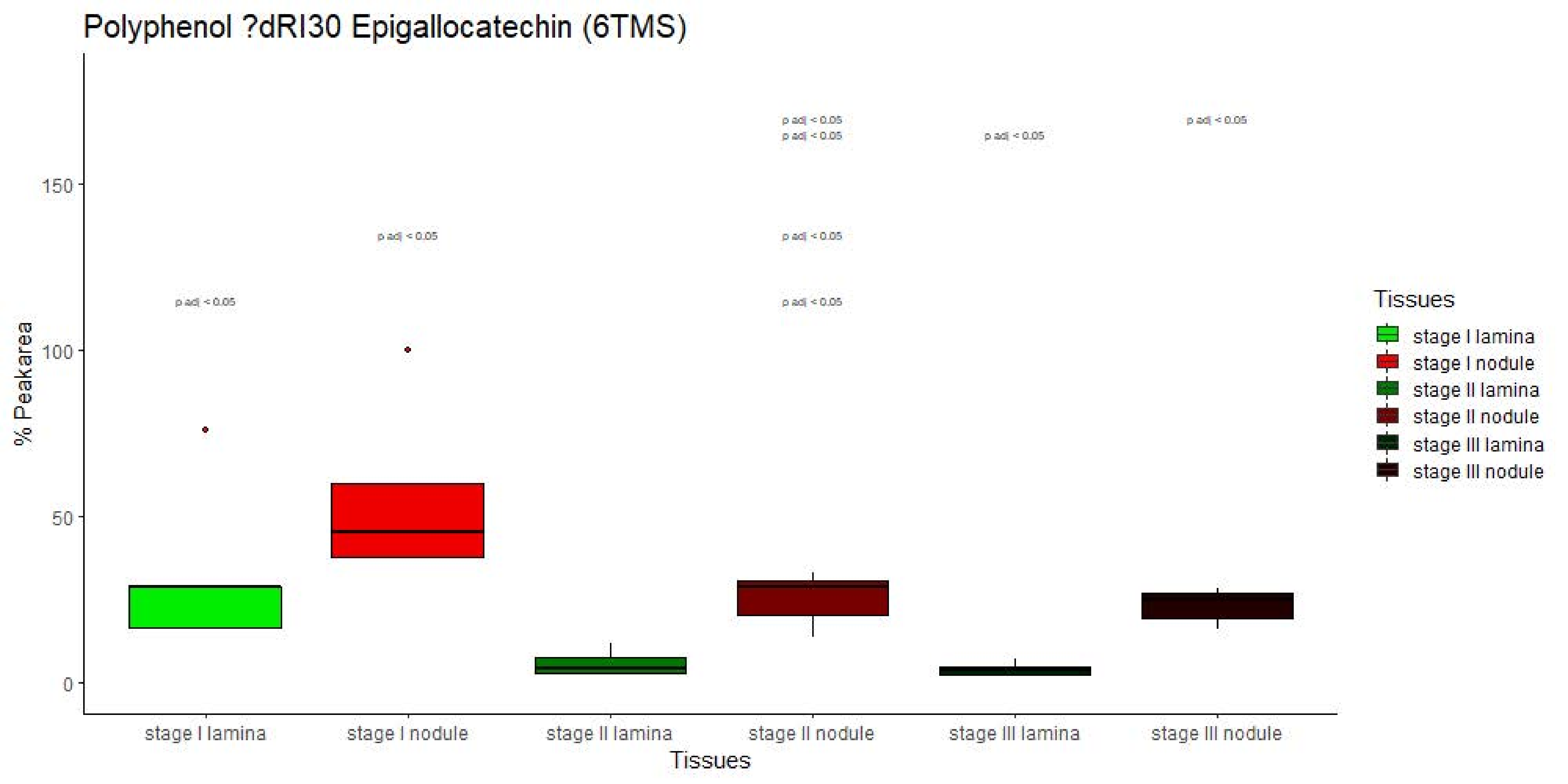

Polyphenol ?dRI30 Epigallocatechin (6TMS)
% Peakarea
0
50
100
150
p adj < 0.05
p adj < 0.05
p adj < 0.05
p adj < 0.05
p adj < 0.05
p adj < 0.05
p adj < 0.05
p adj < 0.05
stage I lamina
stage I nodule
stage II lamina
stage II nodule
stage III lamina
stage III nodule
Tissues
Tissues
stage I lamina
stage I nodule
stage II lamina
stage II nodule
stage III lamina
stage III nodule

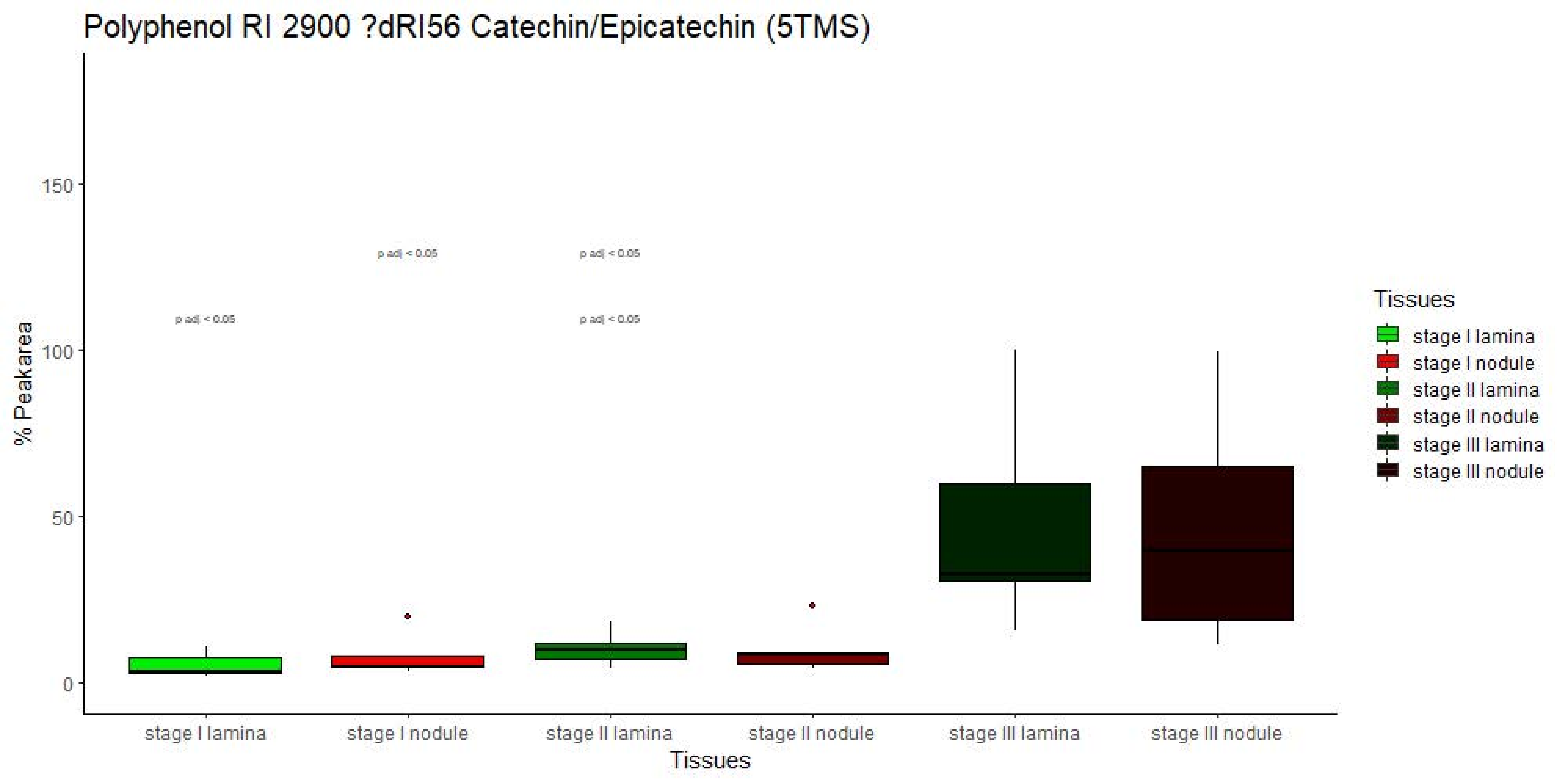

Polyphenol RI 2900 ?dRI56 Catechin/Epicatechin (5TMS)
% Peakarea
150
100
50
0
p adj < 0.05
p adj < 0.05
p adj < 0.05
p adj < 0.05
stage I lamina
stage I nodule
stage II lamina
stage II nodule
stage III lamina
stage III nodule
Tissues
Tissues
stage I lamina
stage I nodule
stage II lamina
stage II nodule
stage III lamina
stage III nodule

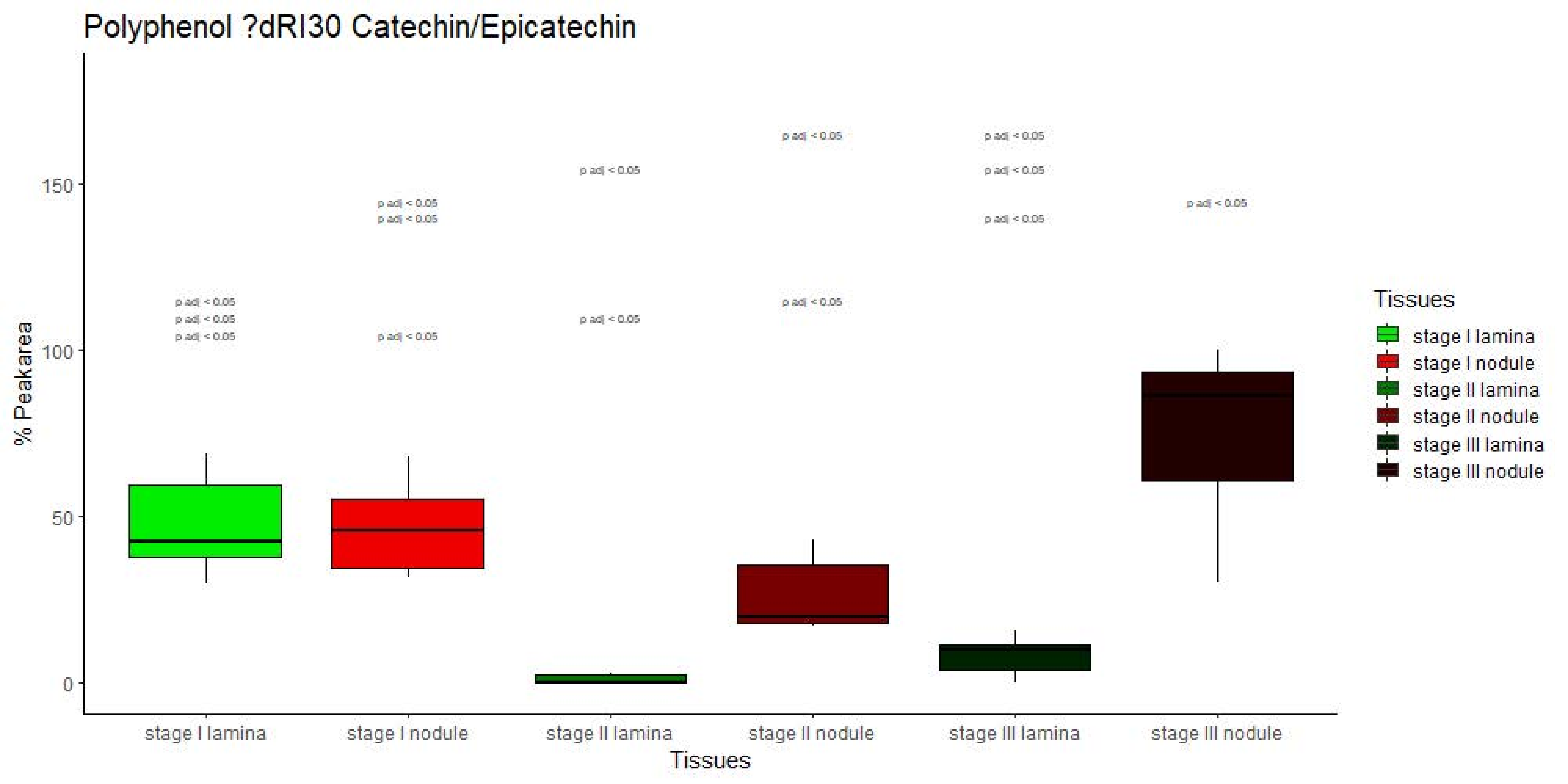
Polyphenol ?dRI30 Catechin/Epicatechin
% Peakarea
150
100
50
0
p adj < 0.05
stage I lamina
stage I nodule
stage II lamina
stage II nodule
stage III lamina
stage III nodule
Tissues
Tissues
stage I lamina
stage I nodule
stage II lamina
stage II nodule
stage III lamina
stage III nodule

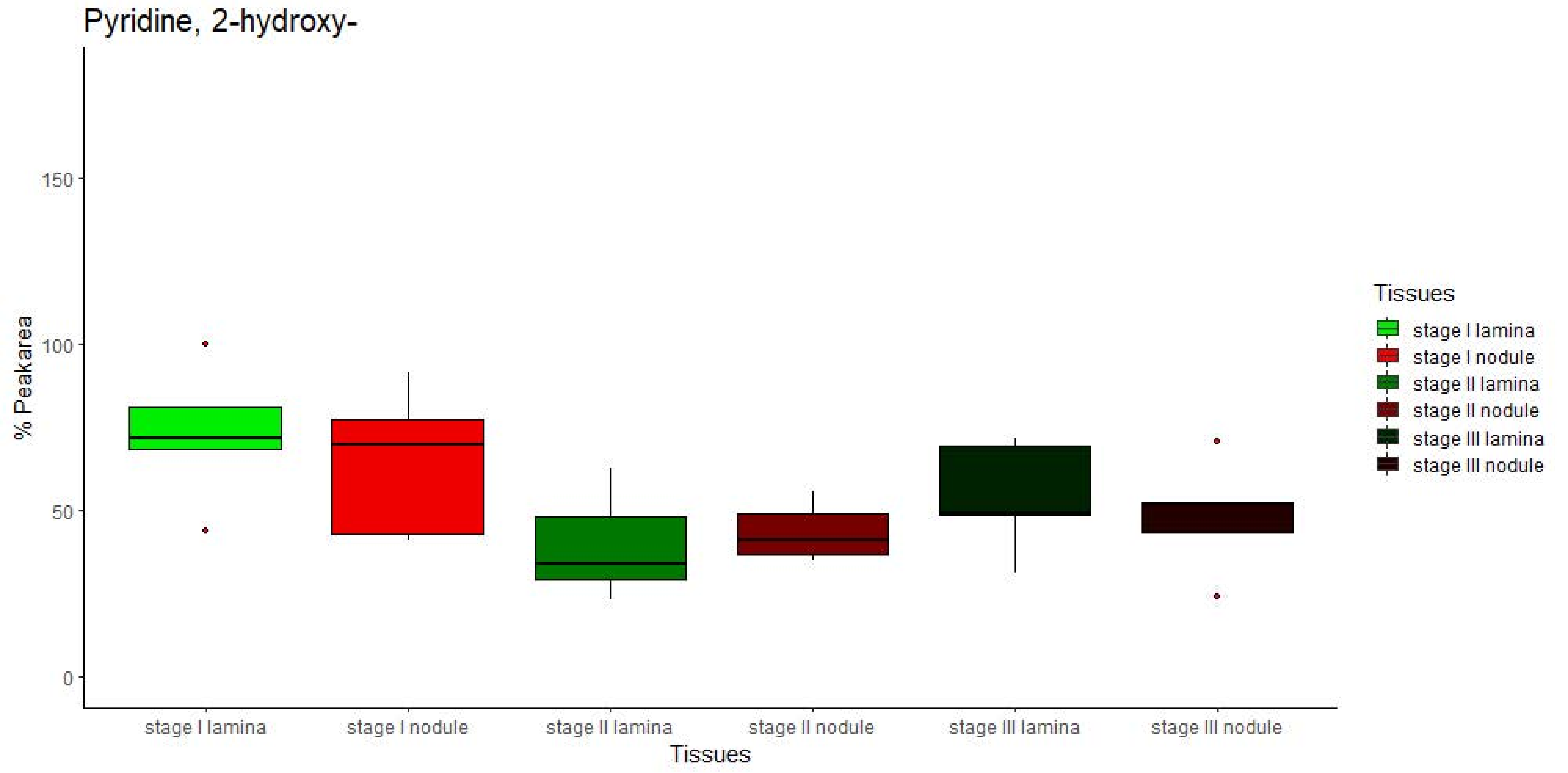

Pyridine, 2-hydroxy-
% Peakarea
150
100
50
0
stage I lamina
stage I nodule
stage II lamina
stage II nodule
stage III lamina
stage III nodule
Tissues
Tissues
stage I lamina
stage I nodule
stage II lamina
stage II nodule
stage III lamina
stage III nodule

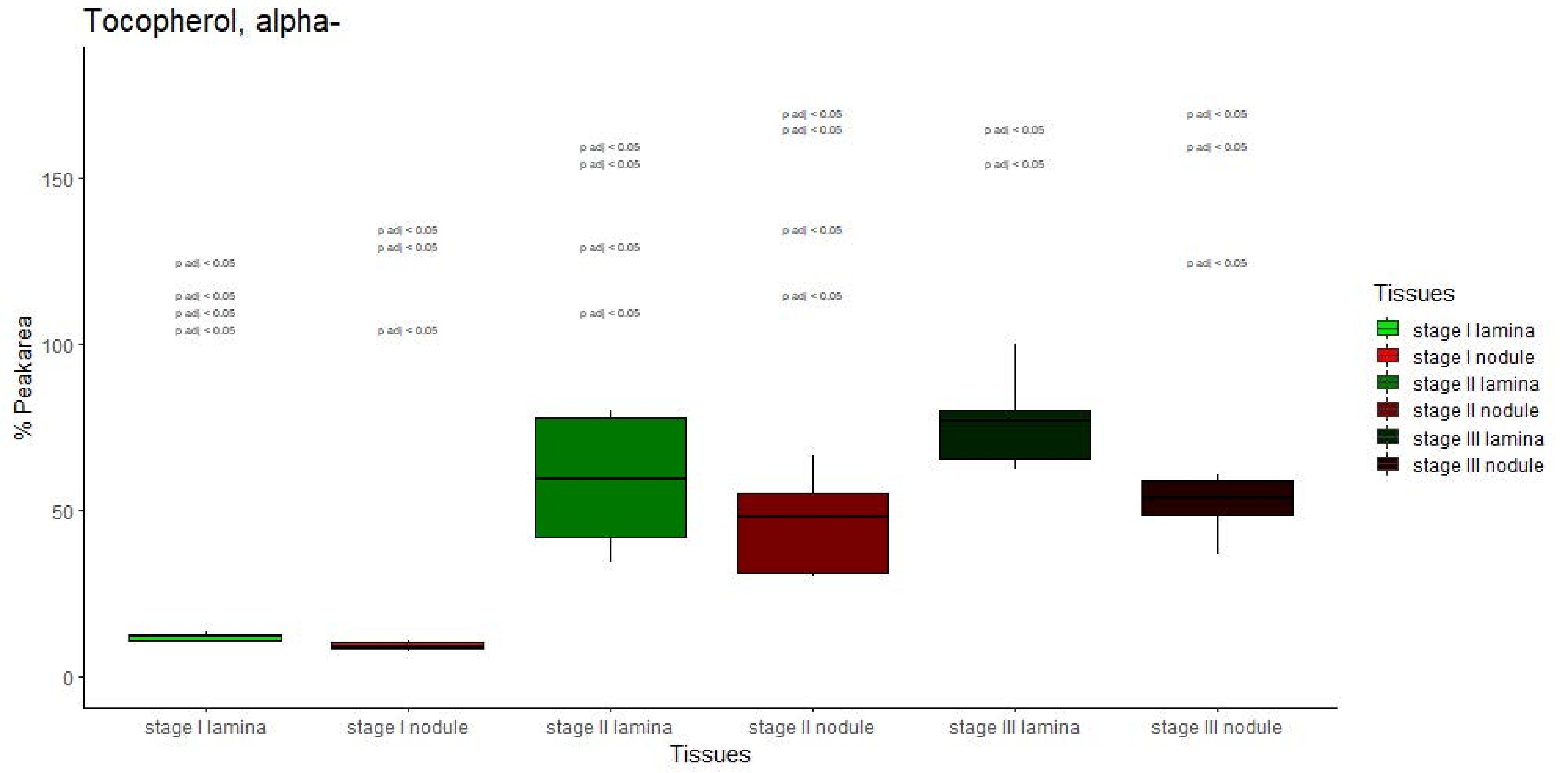

Tocopherol, alpha-
% Peakarea
0
50
100
150
p adj < 0.05
Tissues
stage I lamina
stage I nodule
stage II lamina
stage II nodule
stage III lamina
stage III nodule
Tissues
stage I lamina
stage I nodule
stage II lamina
stage II nodule
stage III lamina
stage III nodule

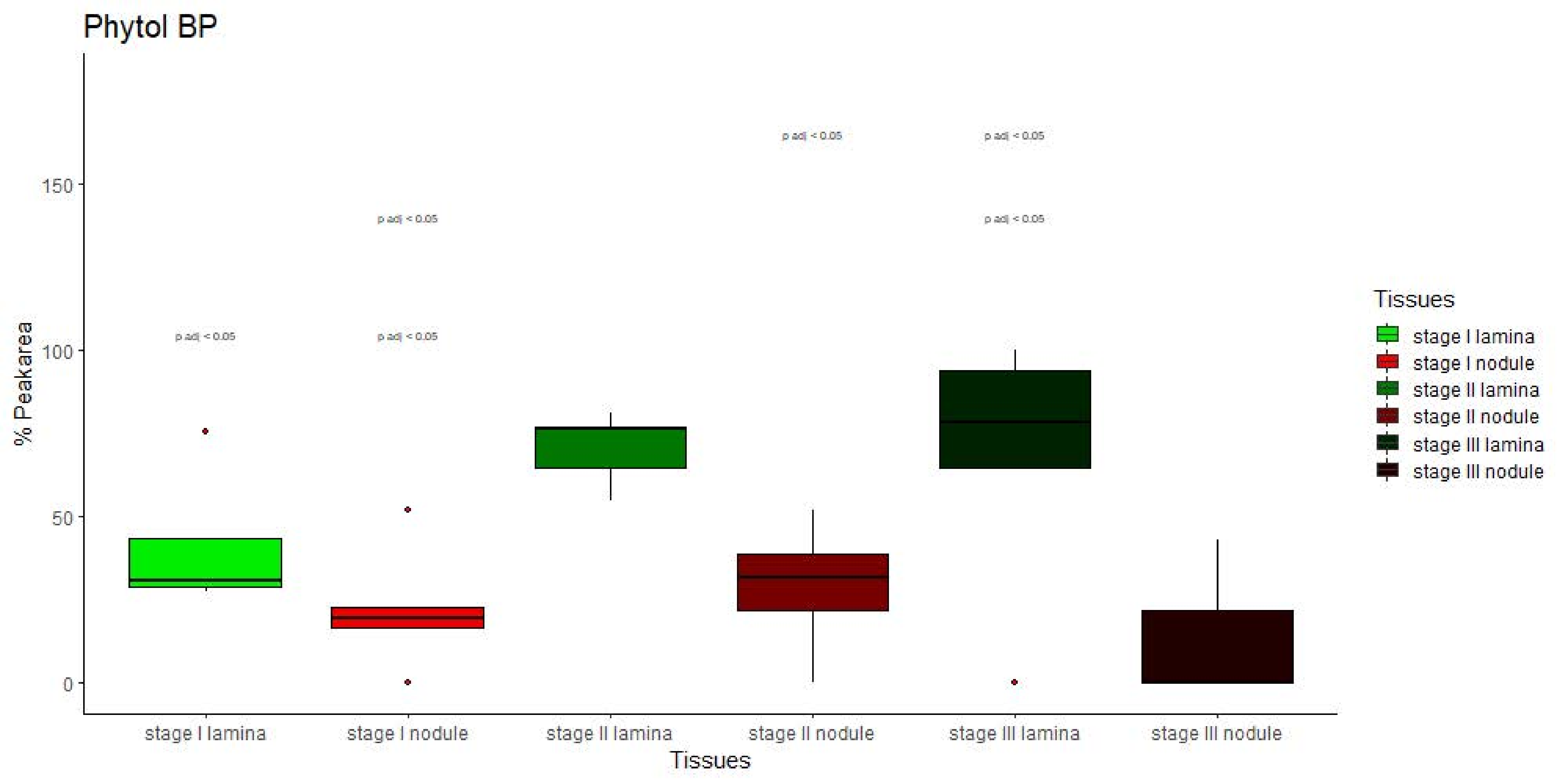
Phytol BP
% Peakarea
0
50
100
150
p adj < 0.05
p adj < 0.05
p adj < 0.05
p adj < 0.05
p adj < 0.05
p adj < 0.05
stage I lamina
stage I nodule
stage II lamina
stage II nodule
stage III lamina
stage III nodule
Tissues
Tissues
stage I lamina
stage I nodule
stage II lamina
stage II nodule
stage III lamina
stage III nodule

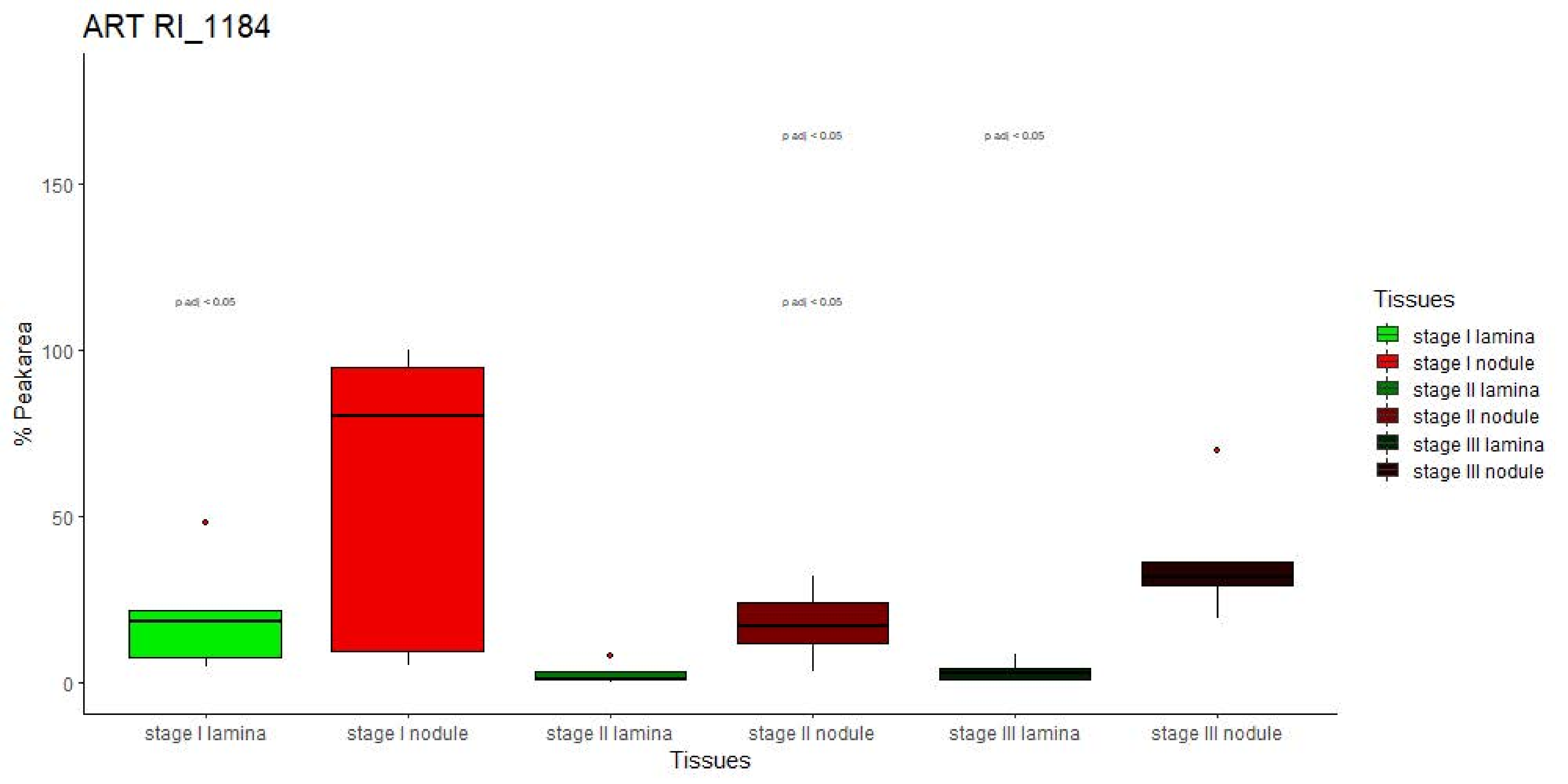

ART RI_1184
p adj < 0.05
p adj < 0.05
p adj < 0.05
p adj < 0.05
150
100
50
0
% Peakarea
stage I lamina
stage I nodule
stage II lamina
stage II nodule
stage III lamina
stage III nodule
Tissues
Tissues
stage I lamina
stage I nodule
stage II lamina
stage II nodule
stage III lamina
stage III nodule

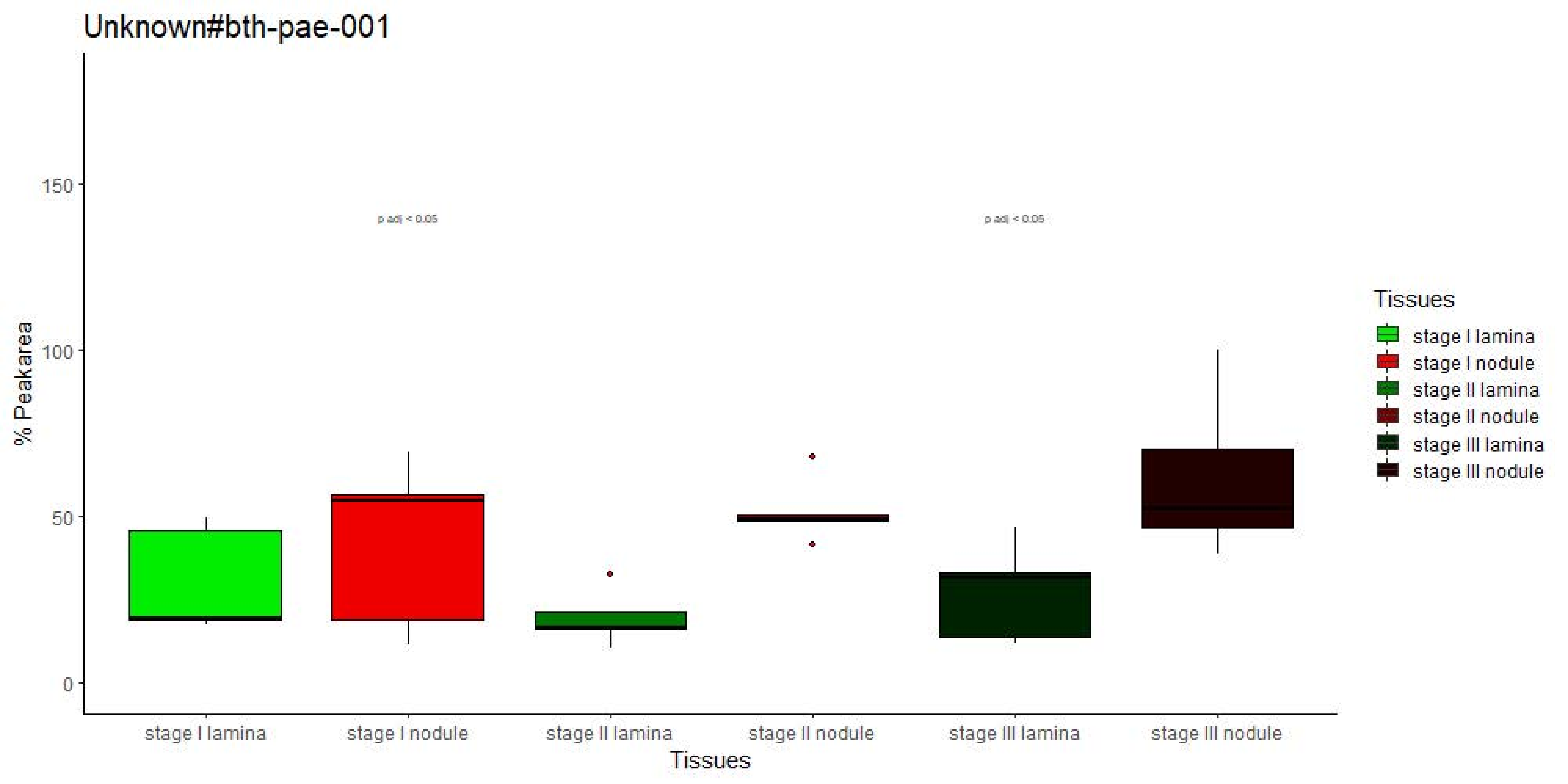
Unknown#bth-pae-001
% Peakarea
150
100
50
0
p adj < 0.05
p adj < 0.05
stage I lamina
stage I nodule
stage II lamina
stage II nodule
stage III lamina
stage III nodule
Tissues
Tissues
stage I lamina
stage I nodule
stage II lamina
stage II nodule
stage III lamina
stage III nodule

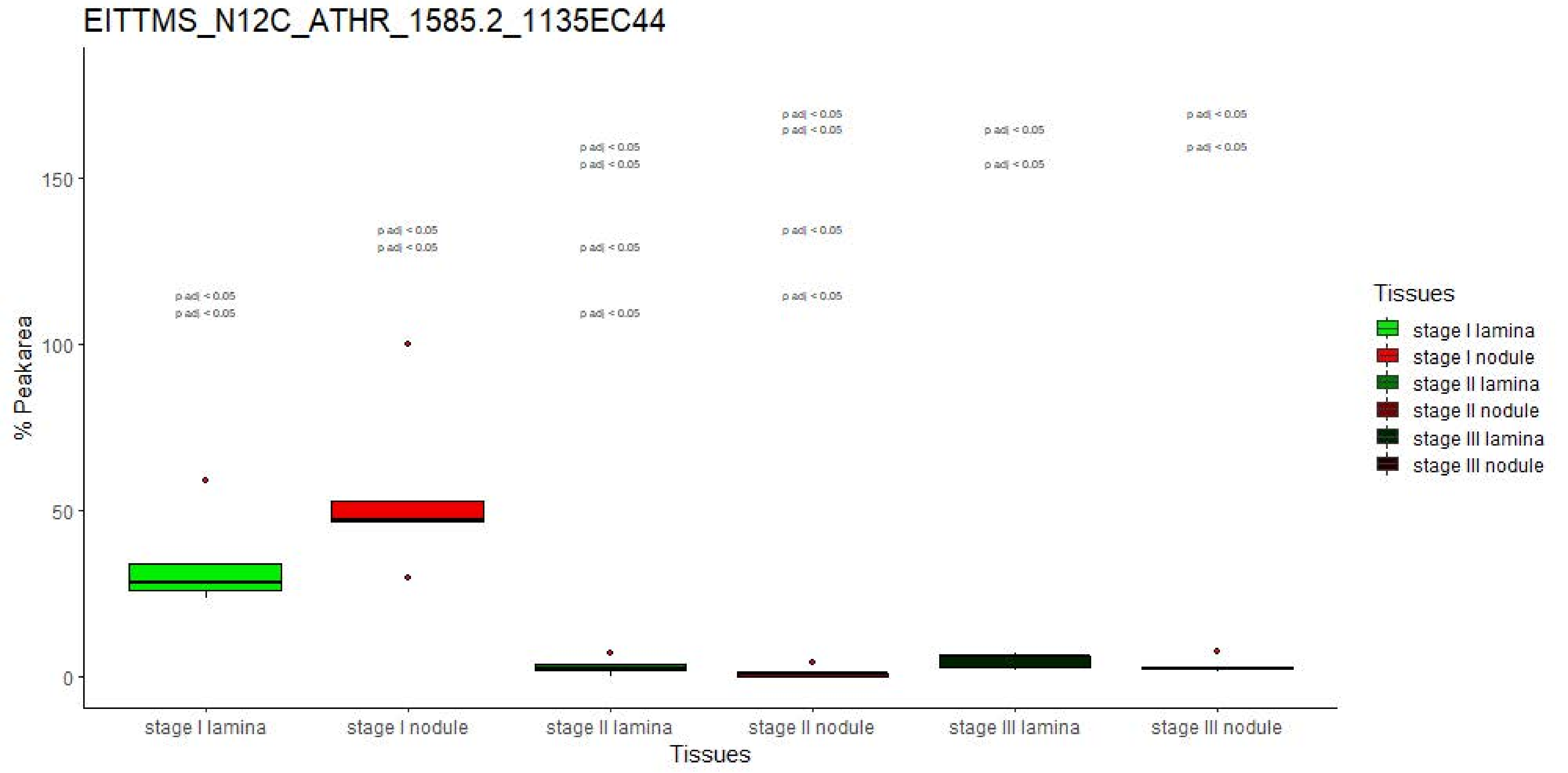

EITTMS_N12C_ATHR_1585.2_1135EC44
% Peakarea
0
50
100
150
p adj < 0.05
Tissues
stage I lamina
stage I nodule
stage II lamina
stage II nodule
stage III lamina
stage III nodule

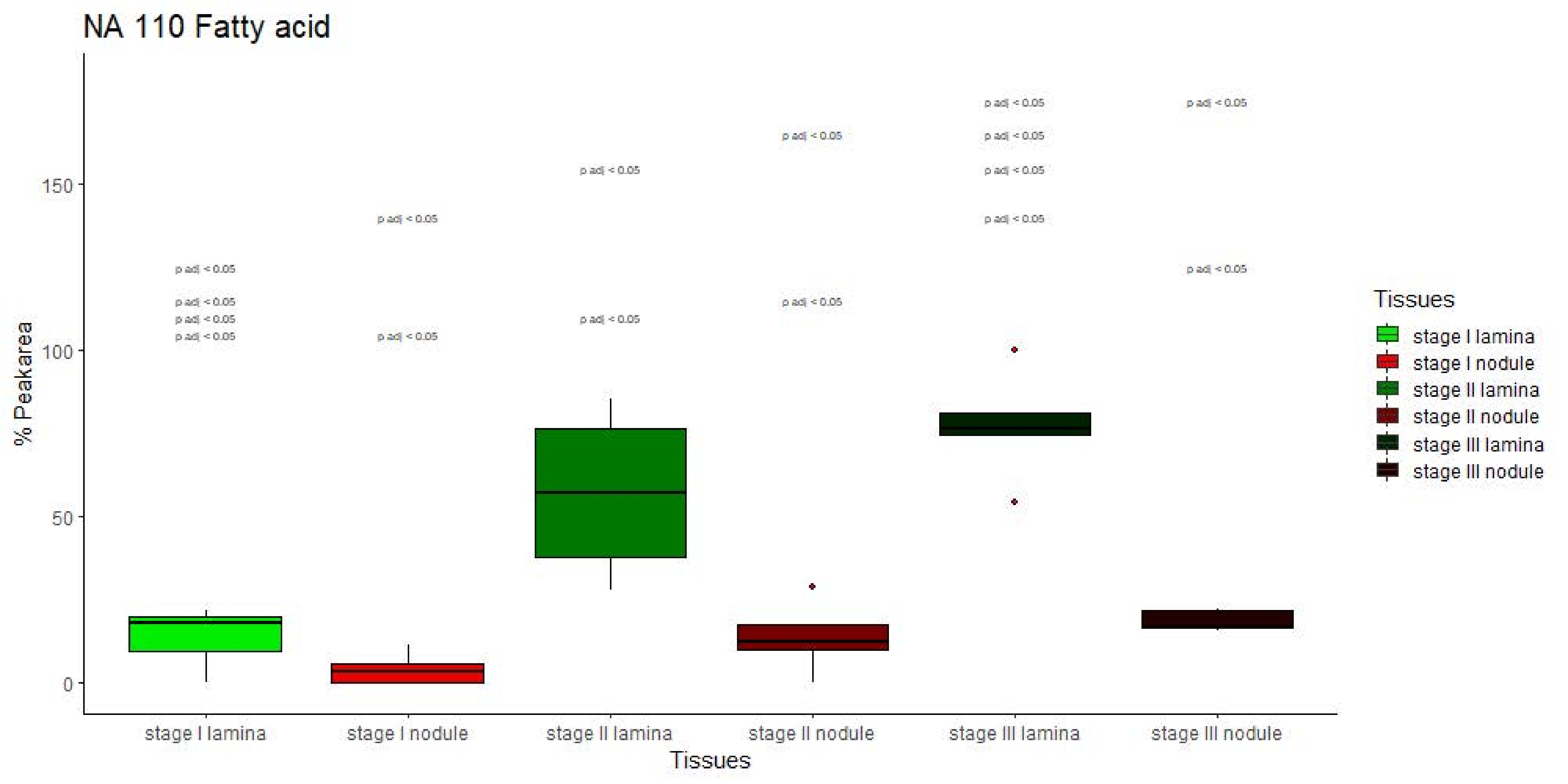

NA 110 Fatty acid
% Peakarea
0
50
100
150
p adj < 0.05
Tissues
stage I lamina
stage I nodule
stage II lamina
stage II nodule
stage III lamina
stage III nodule

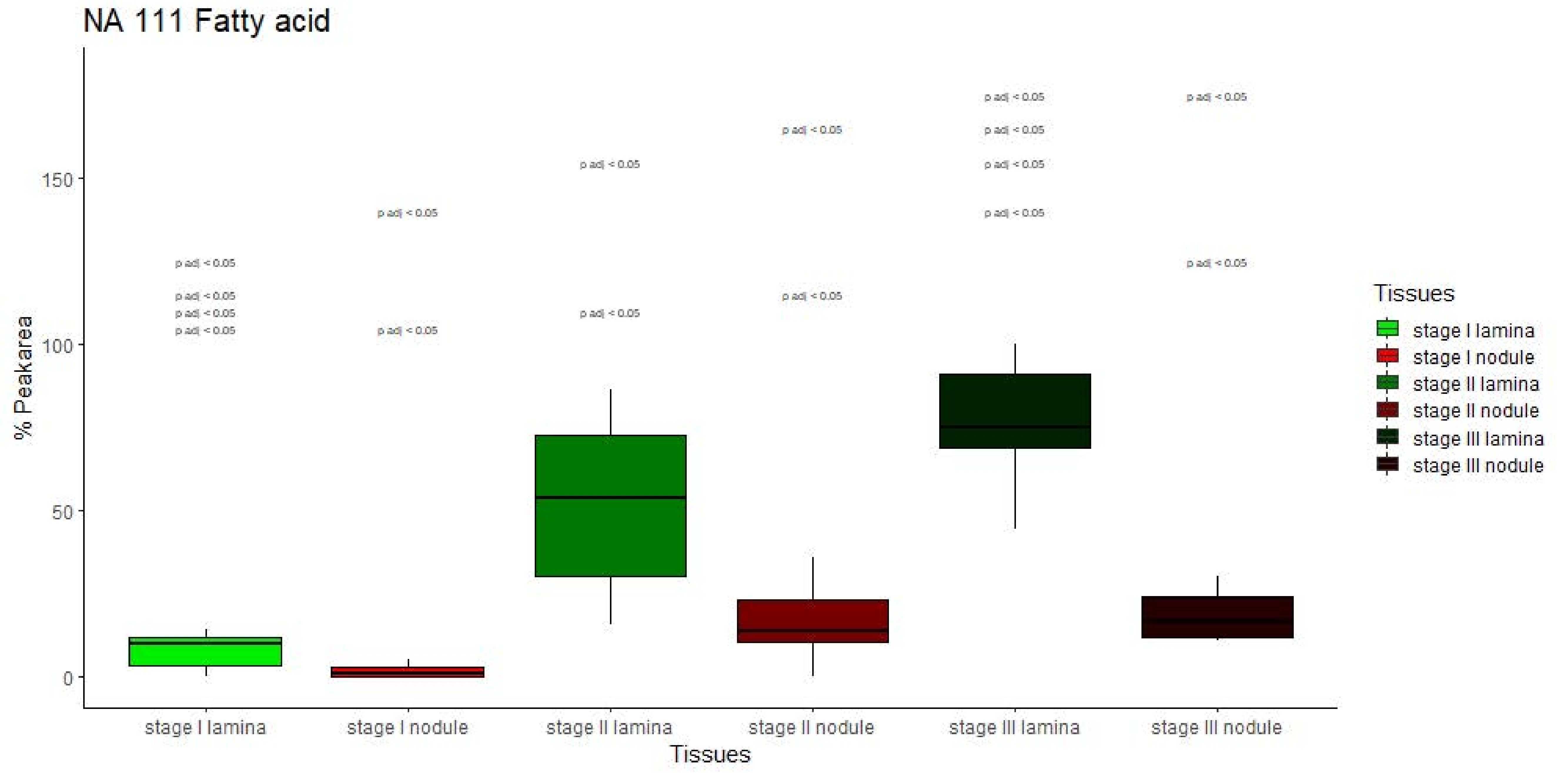

NA 111 Fatty acid
% Peakarea
150
100
50
0
p adj < 0.05
stage I lamina
stage I nodule
stage II lamina
stage II nodule
stage III lamina
stage III nodule
Tissues
Tissues
stage I lamina
stage I nodule
stage II lamina
stage II nodule
stage III lamina
stage III nodule

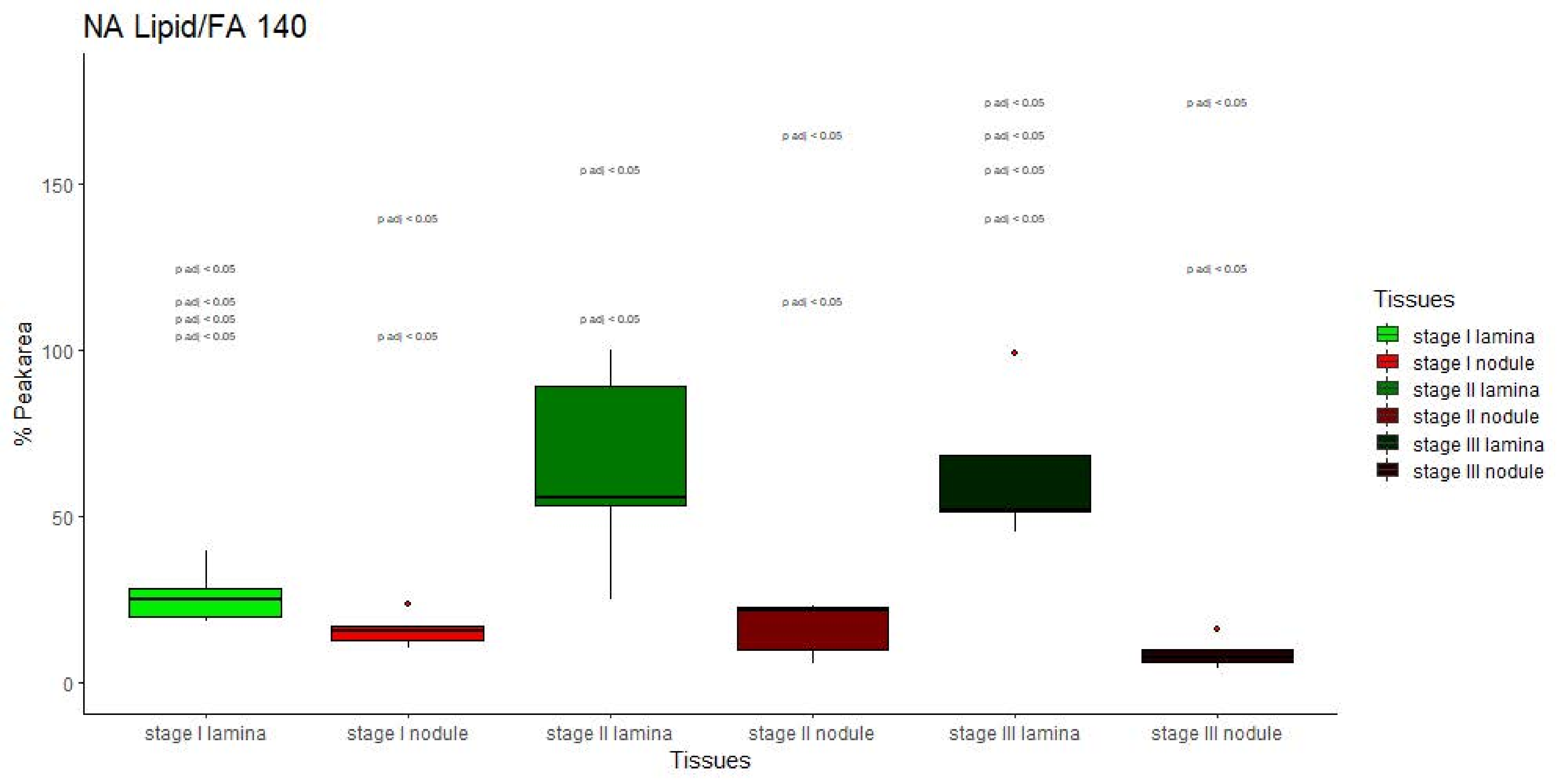

NA Lipid/FA 140
% Peakarea
0
50
100
150
p adj < 0.05
Tissues
stage I lamina
stage I nodule
stage II lamina
stage II nodule
stage III lamina
stage III nodule

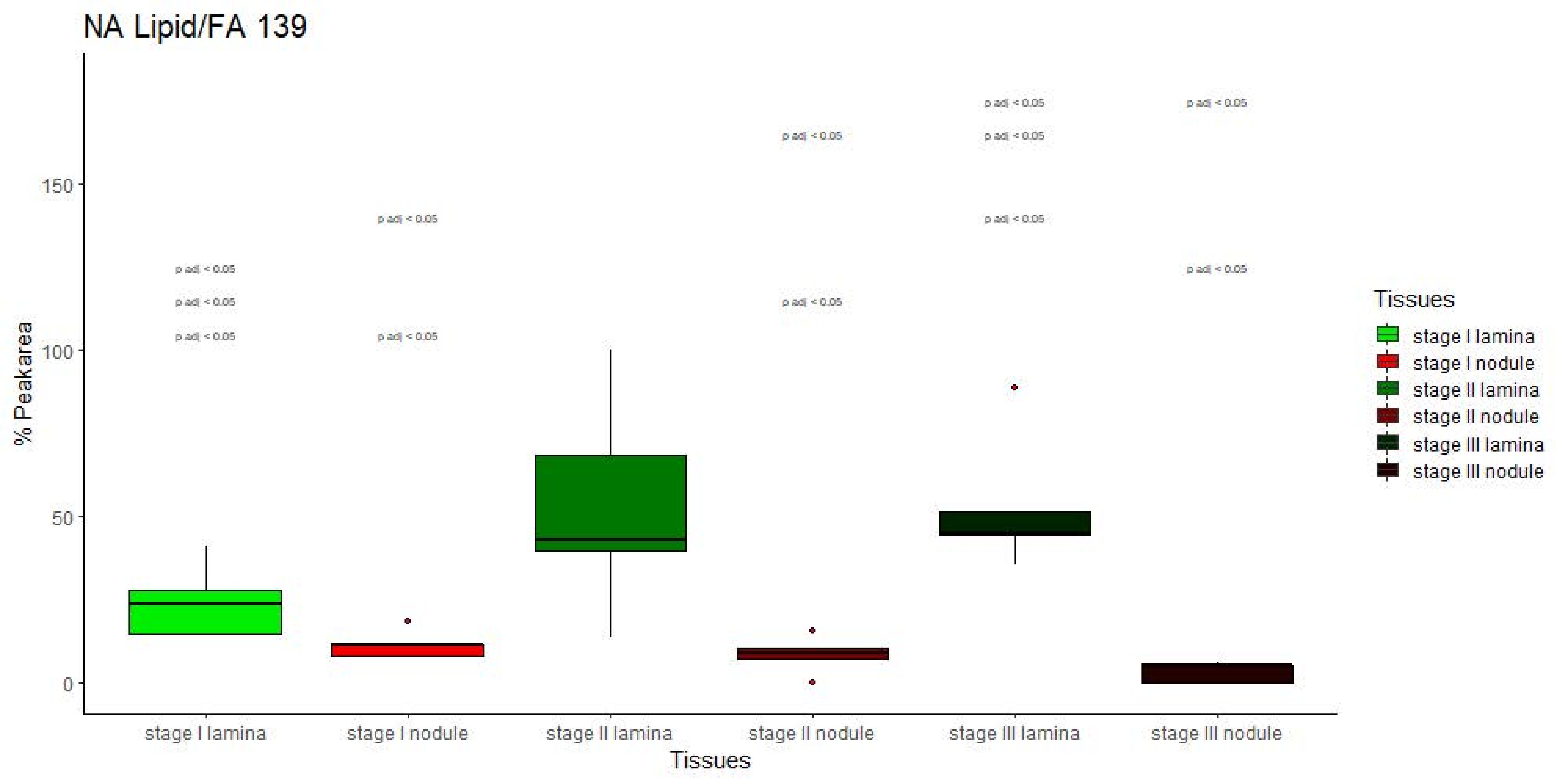

NA Lipid/FA 139
% Peakarea
150
100
50
0
p adj < 0.05
stage I lamina
stage I nodule
stage II lamina
stage II nodule
stage III lamina
stage III nodule
Tissues

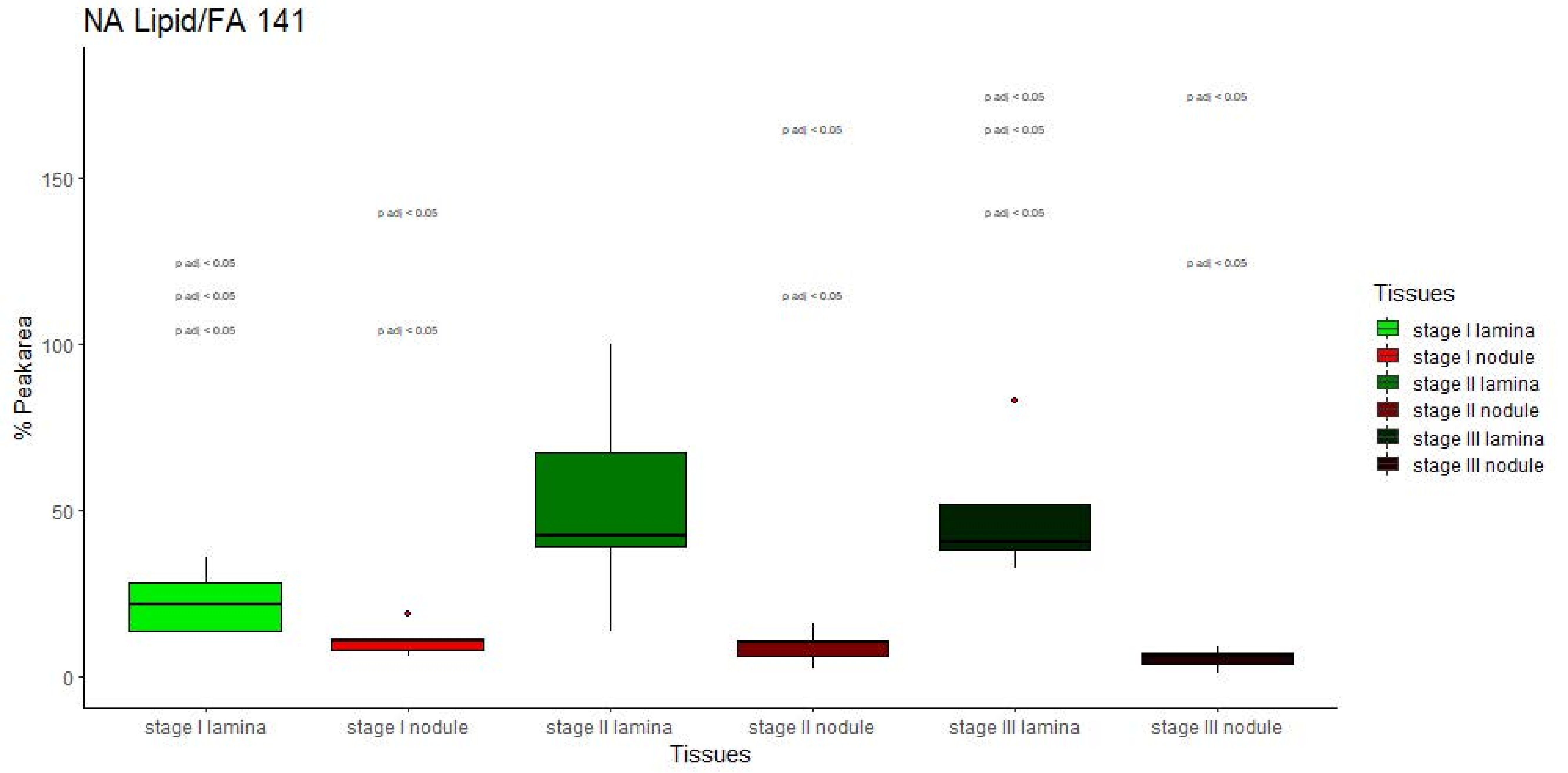

NA Lipid/FA 141
% Peakarea
150
100
50
0
p adj < 0.05
stage I lamina
stage I nodule
stage II lamina
stage II nodule
stage III lamina
stage III nodule
Tissues

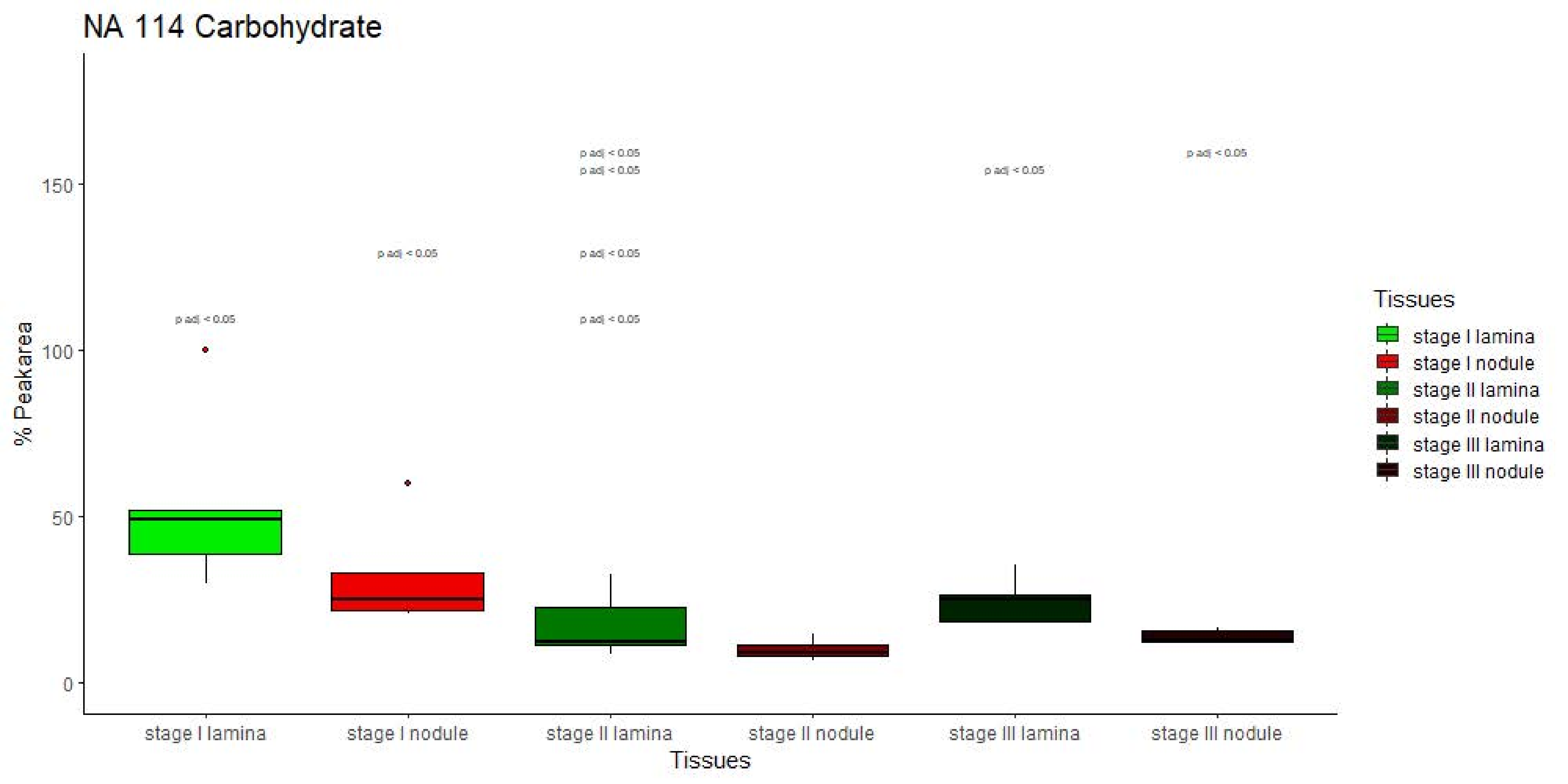
NA 114 Carbohydrate
% Peakarea
0
50
100
150
p adj < 0.05
p adj < 0.05
p adj < 0.05
p adj < 0.05
p adj < 0.05
p adj < 0.05
p adj < 0.05
p adj < 0.05
stage I lamina
stage I nodule
stage II lamina
stage II nodule
stage III lamina
stage III nodule
Tissues
Tissues
stage I lamina
stage I nodule
stage II lamina
stage II nodule
stage III lamina
stage III nodule

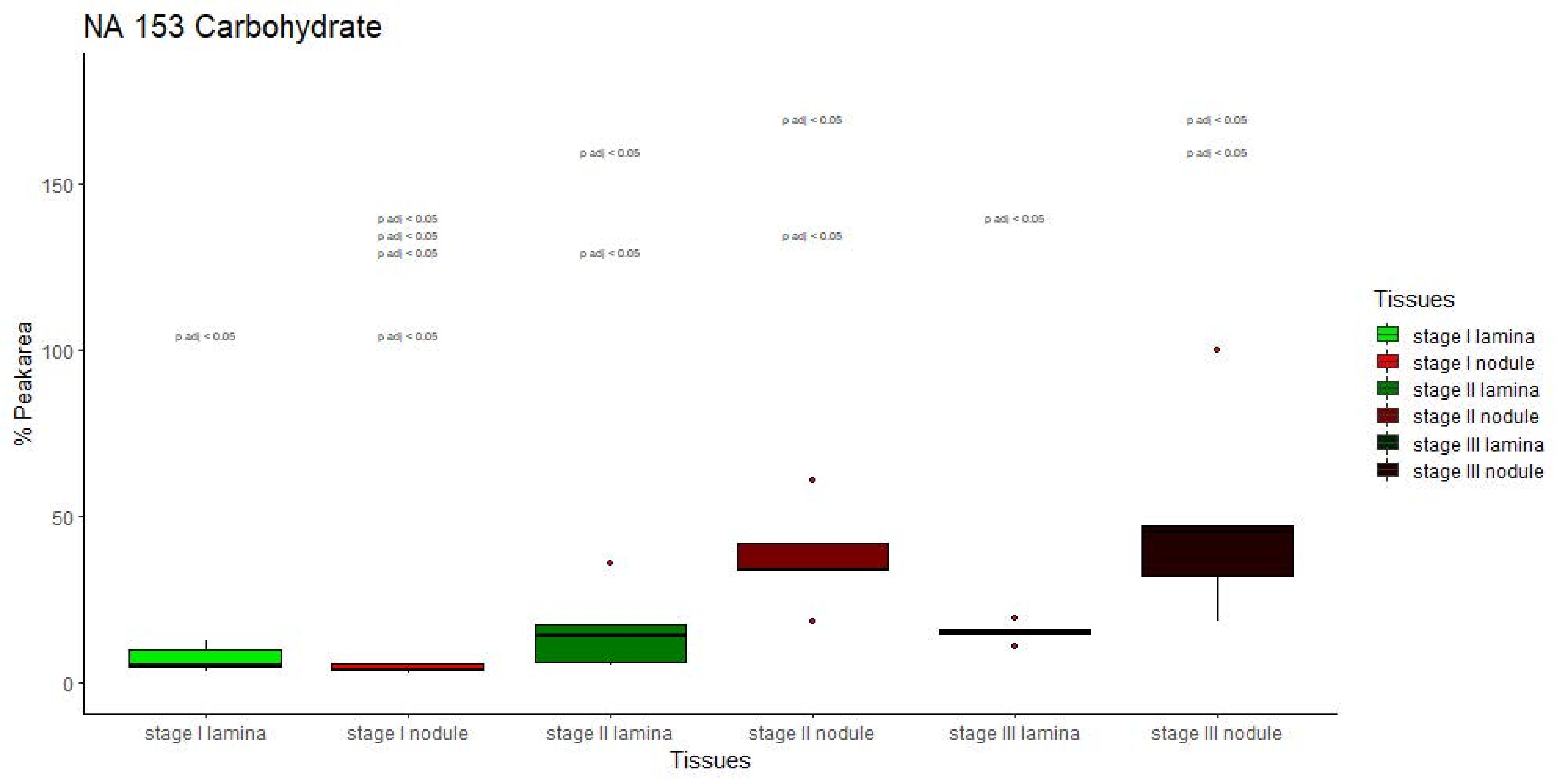

NA 153 Carbohydrate
% Peakarea
0
50
100
150
p adj < 0.05
Tissues
stage I lamina
stage I nodule
stage II lamina
stage II nodule
stage III lamina
stage III nodule

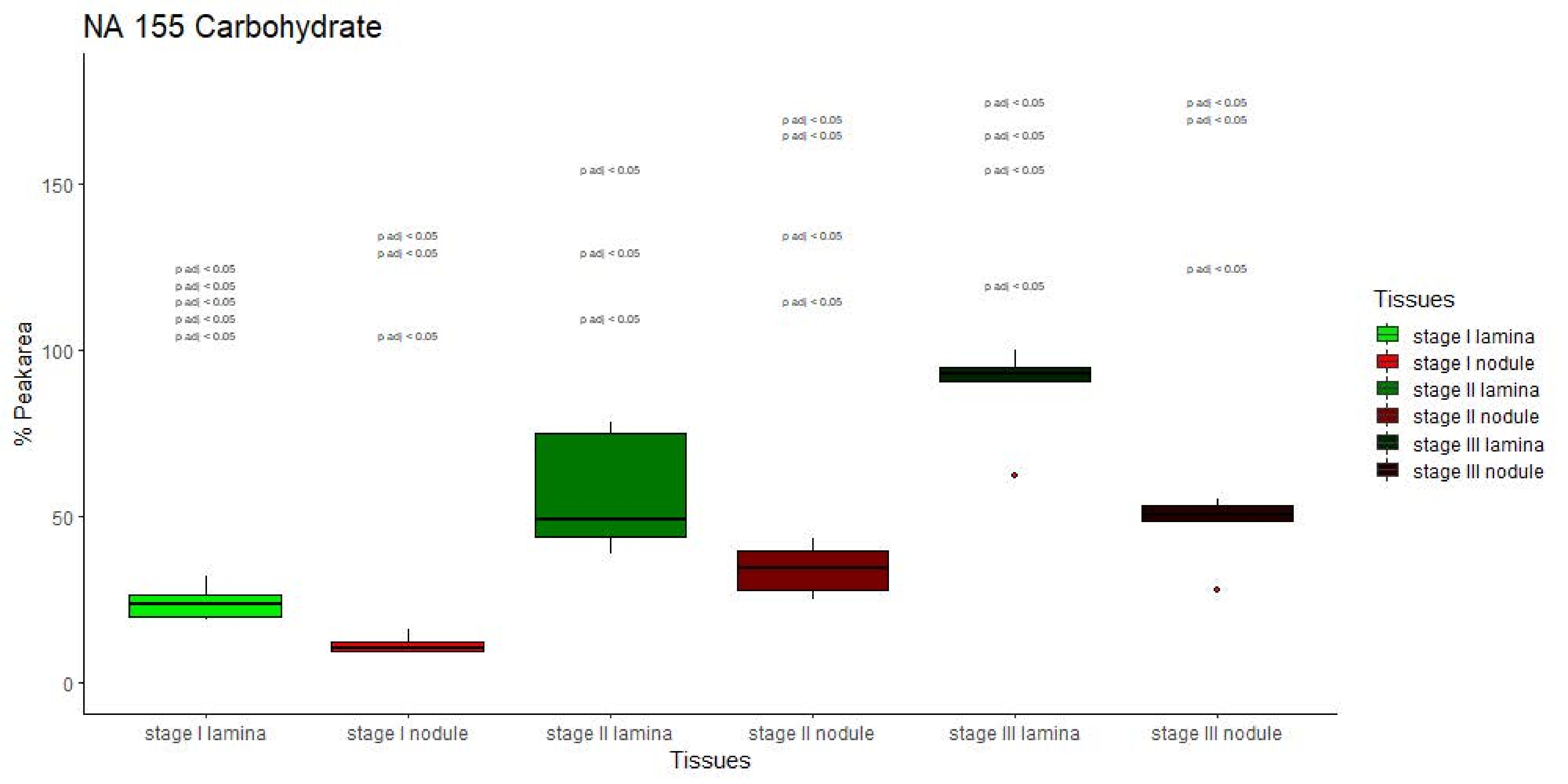

NA 155 Carbohydrate
% Peakarea
150
100
50
0
p adj < 0.05
stage I lamina
stage I nodule
stage II lamina
stage II nodule
stage III lamina
stage III nodule
Tissues

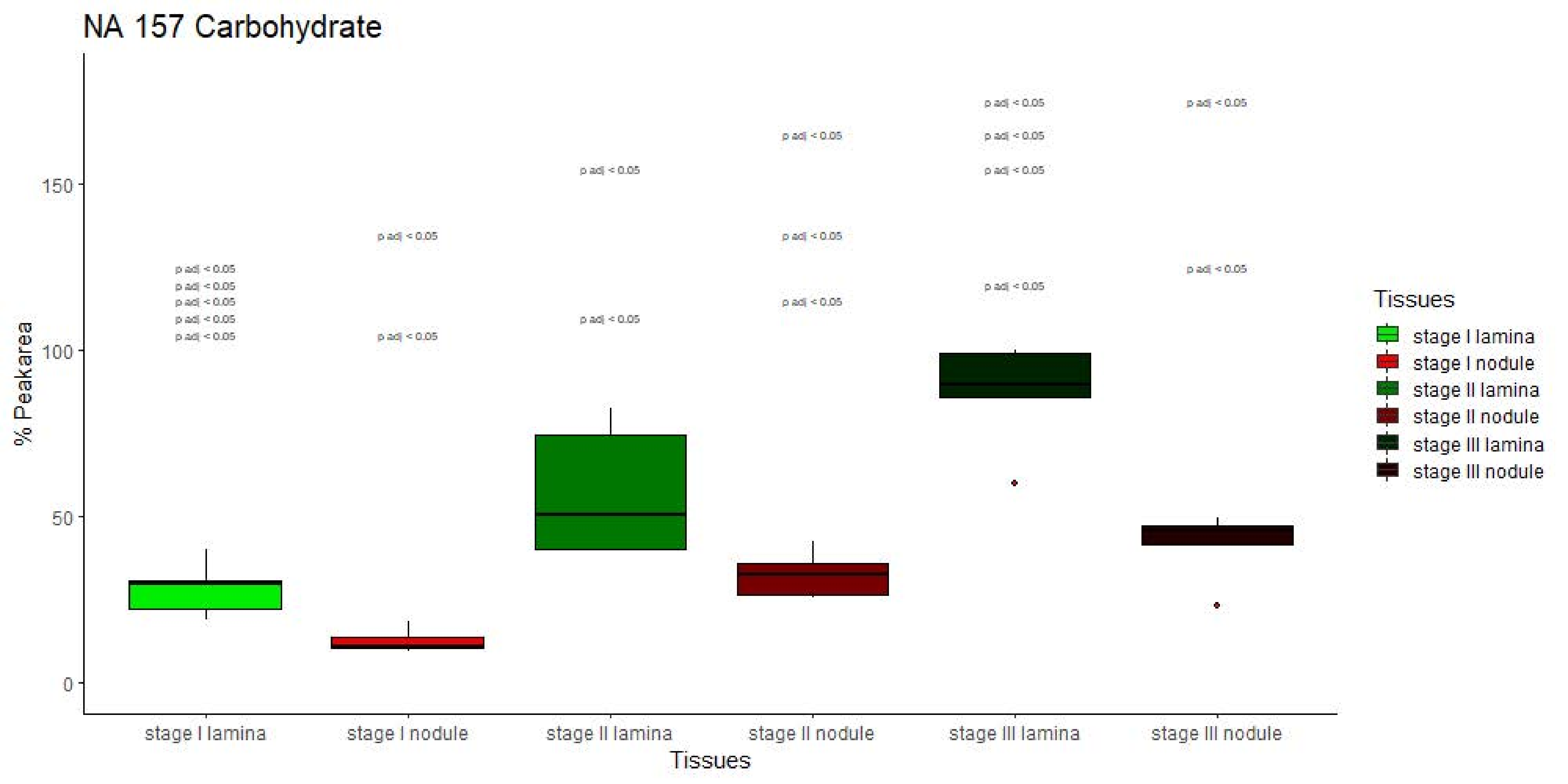

NA 157 Carbohydrate
% Peakarea
150
100
50
0
p adj < 0.05
stage I lamina
stage I nodule
stage II lamina
stage II nodule
stage III lamina
stage III nodule
Tissues
Tissues
stage I lamina
stage I nodule
stage II lamina
stage II nodule
stage III lamina
stage III nodule

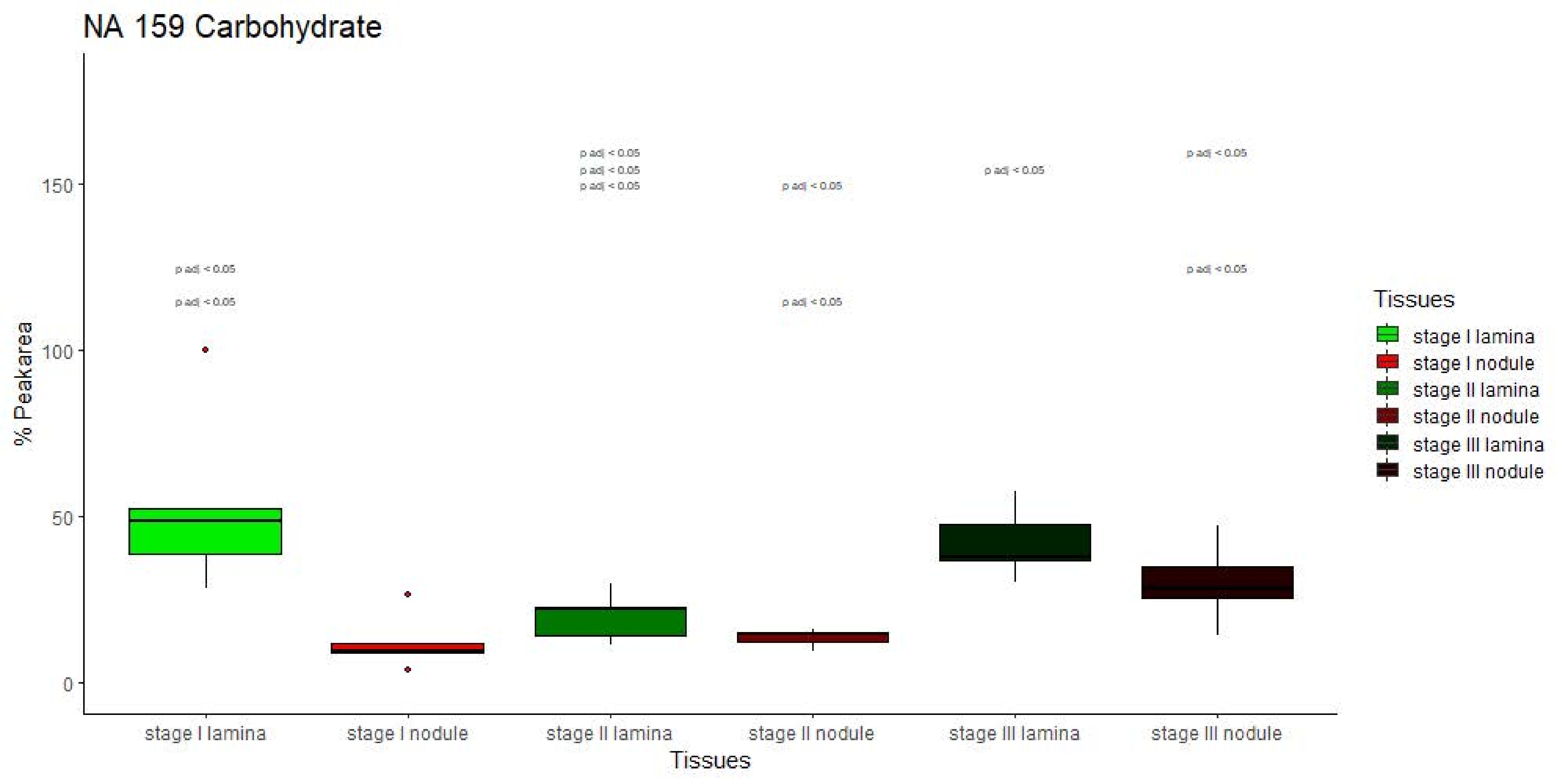
NA 159 Carbohydrate
% Peakarea
0
50
100
150
p adj < 0.05
p adj < 0.05
p adj < 0.05
p adj < 0.05
p adj < 0.05
p adj < 0.05
p adj < 0.05
p adj < 0.05
p adj < 0.05
p adj < 0.05
p adj < 0.05
stage I lamina
stage I nodule
stage II lamina
stage II nodule
stage III lamina
stage III nodule
Tissues
Tissues
stage I lamina
stage I nodule
stage II lamina
stage II nodule
stage III lamina
stage III nodule

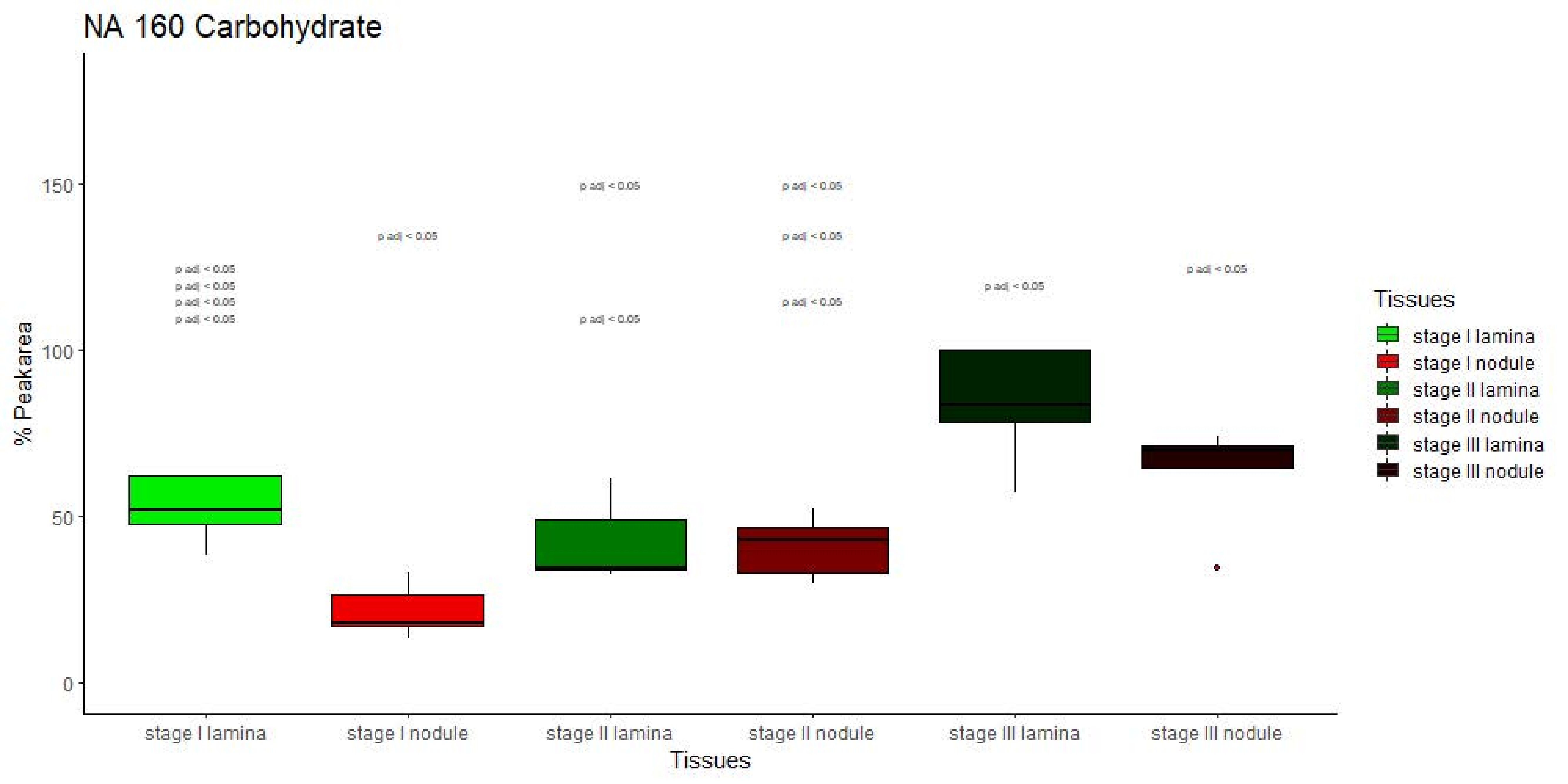
NA 160 Carbohydrate
% Peakarea
150
100
50
0
p adj < 0.05
p adj < 0.05
p adj < 0.05
p adj < 0.05
p adj < 0.05
p adj < 0.05
p adj < 0.05
p adj < 0.05
p adj < 0.05
p adj < 0.05
p adj < 0.05
p adj < 0.05
stage I lamina
stage I nodule
stage II lamina
stage II nodule
stage III lamina
stage III nodule
Tissues
Tissues
stage I lamina
stage I nodule
stage II lamina
stage II nodule
stage III lamina
stage III nodule

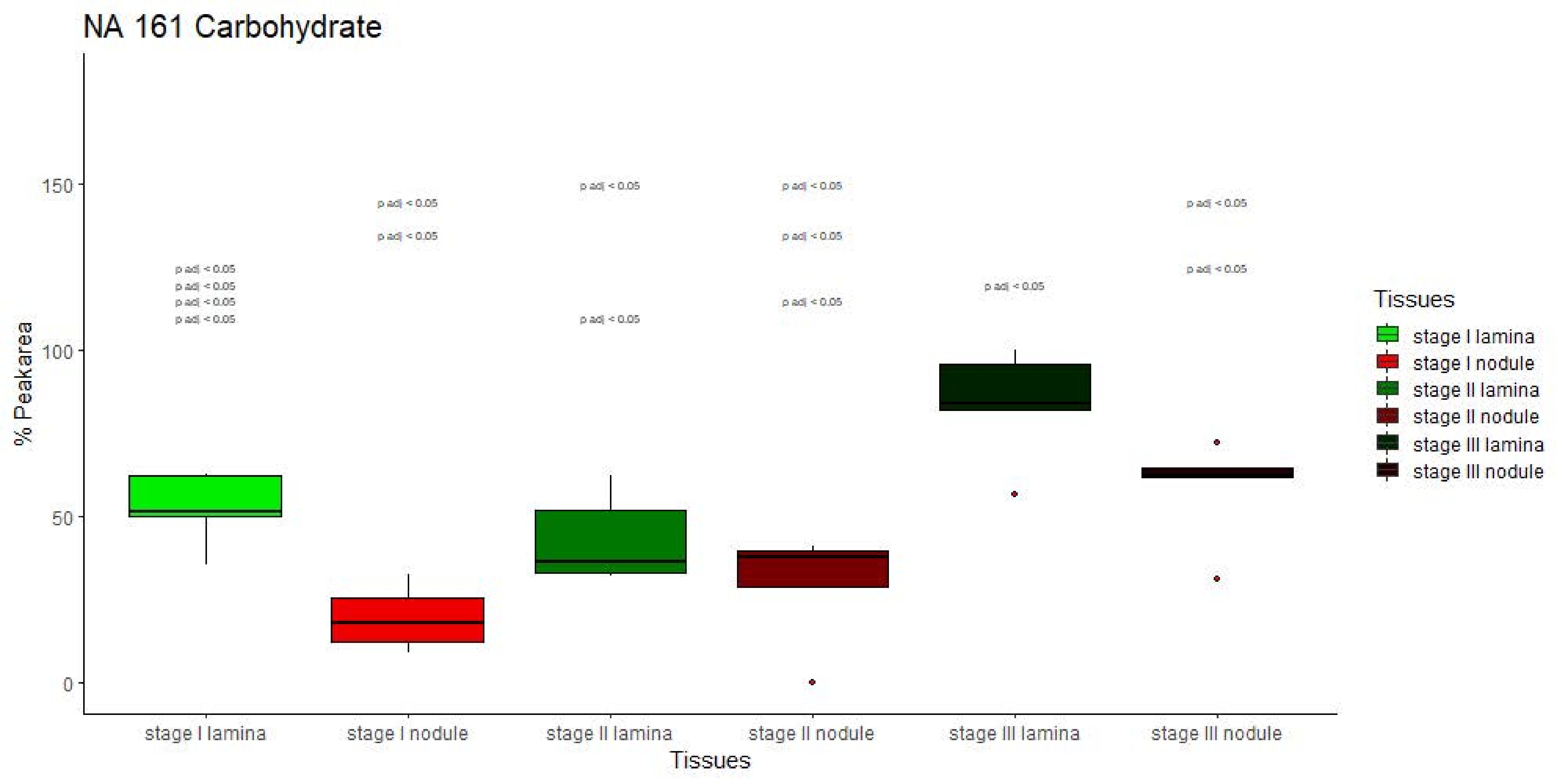

NA 161 Carbohydrate
% Peakarea
150
100
50
0
p adj < 0.05
stage I lamina
stage I nodule
stage II lamina
stage II nodule
stage III lamina
stage III nodule
Tissues

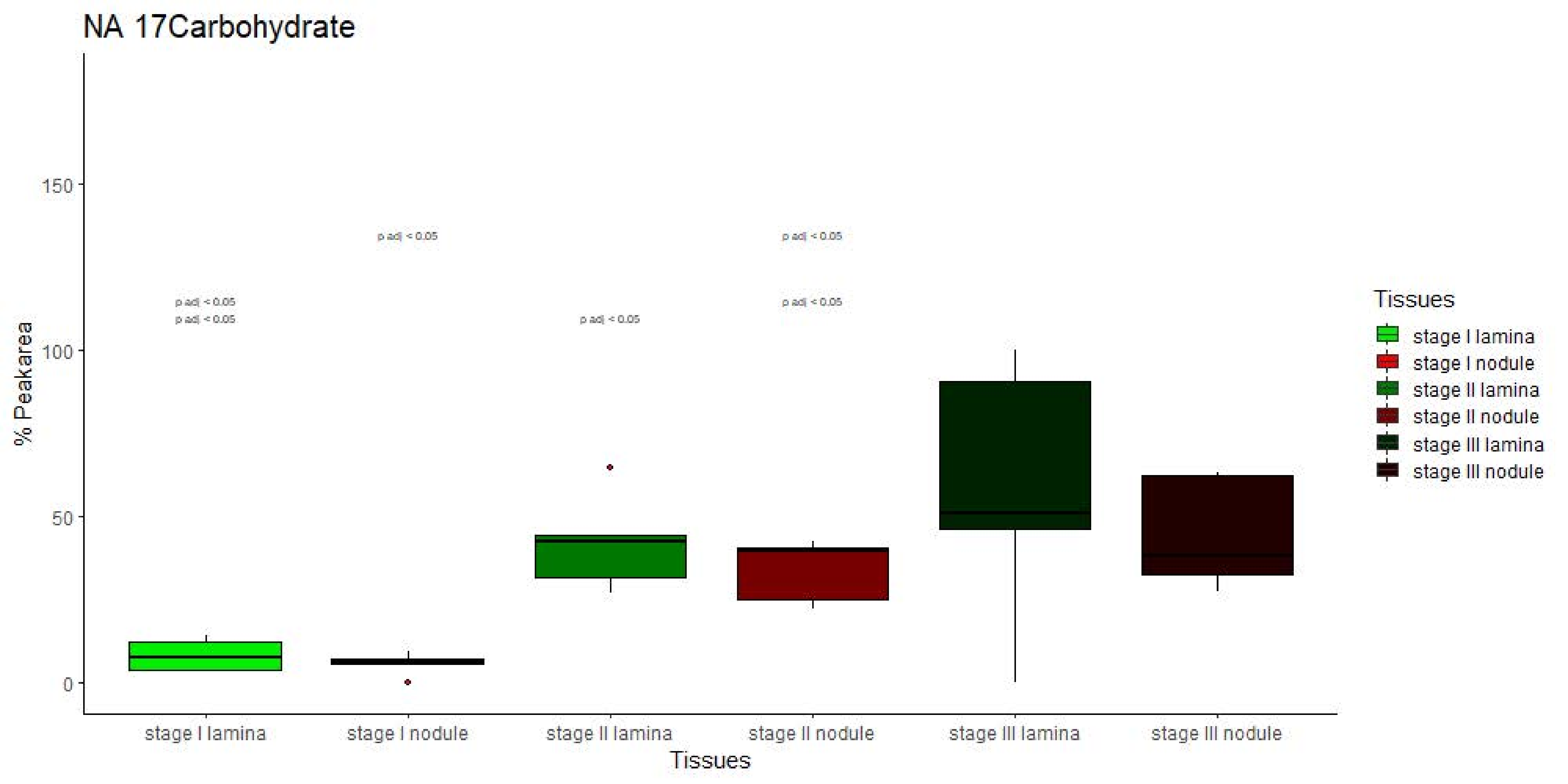

NA 17Carbohydrate
150
100
50
0
% Peakarea
p adj < 0.05
p adj < 0.05
p adj < 0.05
p adj < 0.05
p adj < 0.05
p adj < 0.05
stage I lamina
stage I nodule
stage II lamina
stage II nodule
stage III lamina
stage III nodule
Tissues
Tissues
stage I lamina
stage I nodule
stage II lamina
stage II nodule
stage III lamina
stage III nodule

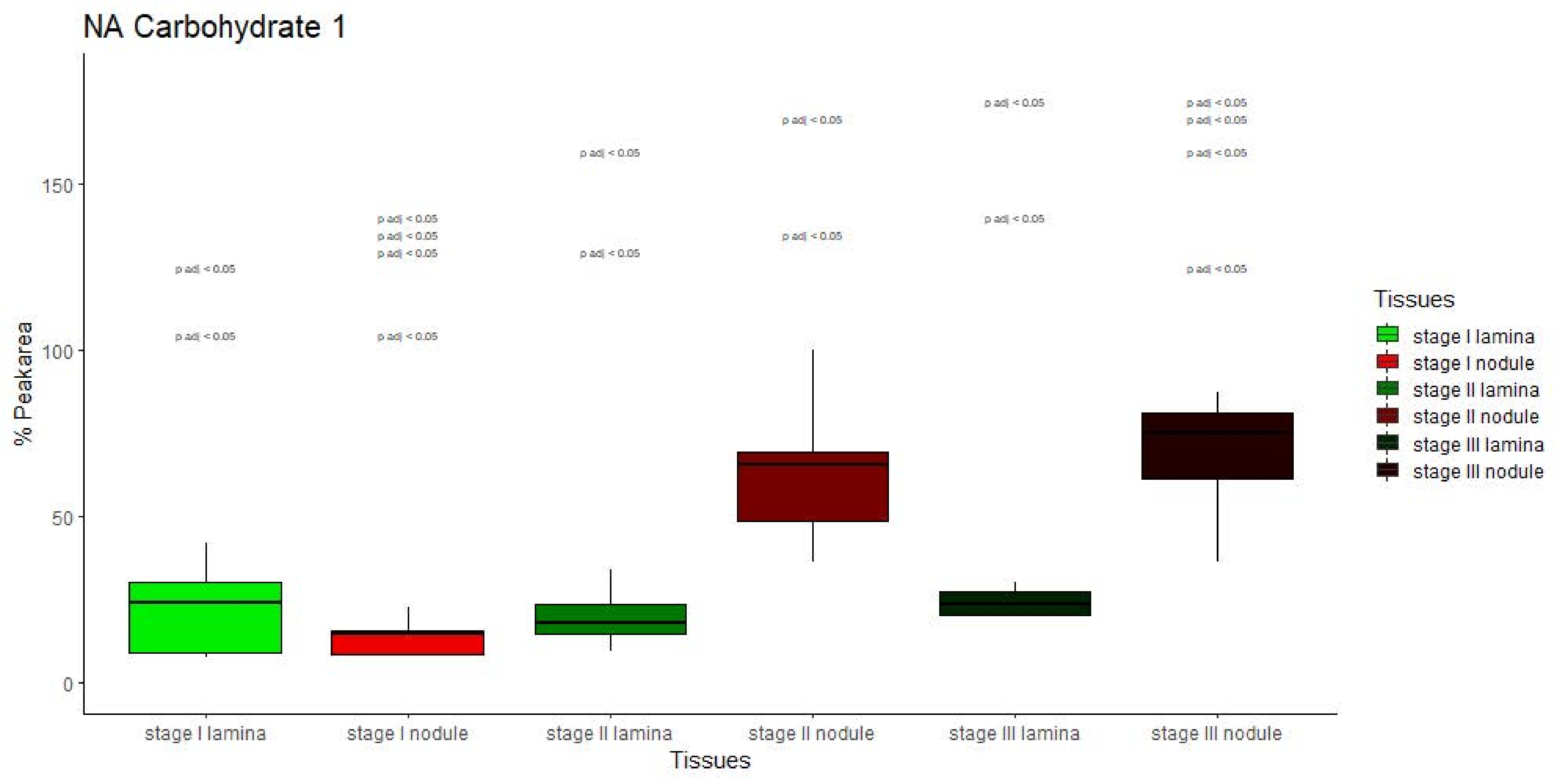

NA Carbohydrate 1
% Peakarea
150
100
50
0
p adj < 0.05
stage I lamina
stage I nodule
stage II lamina
stage II nodule
stage III lamina
stage III nodule
Tissues
Tissues
stage I lamina
stage I nodule
stage II lamina
stage II nodule
stage III lamina
stage III nodule

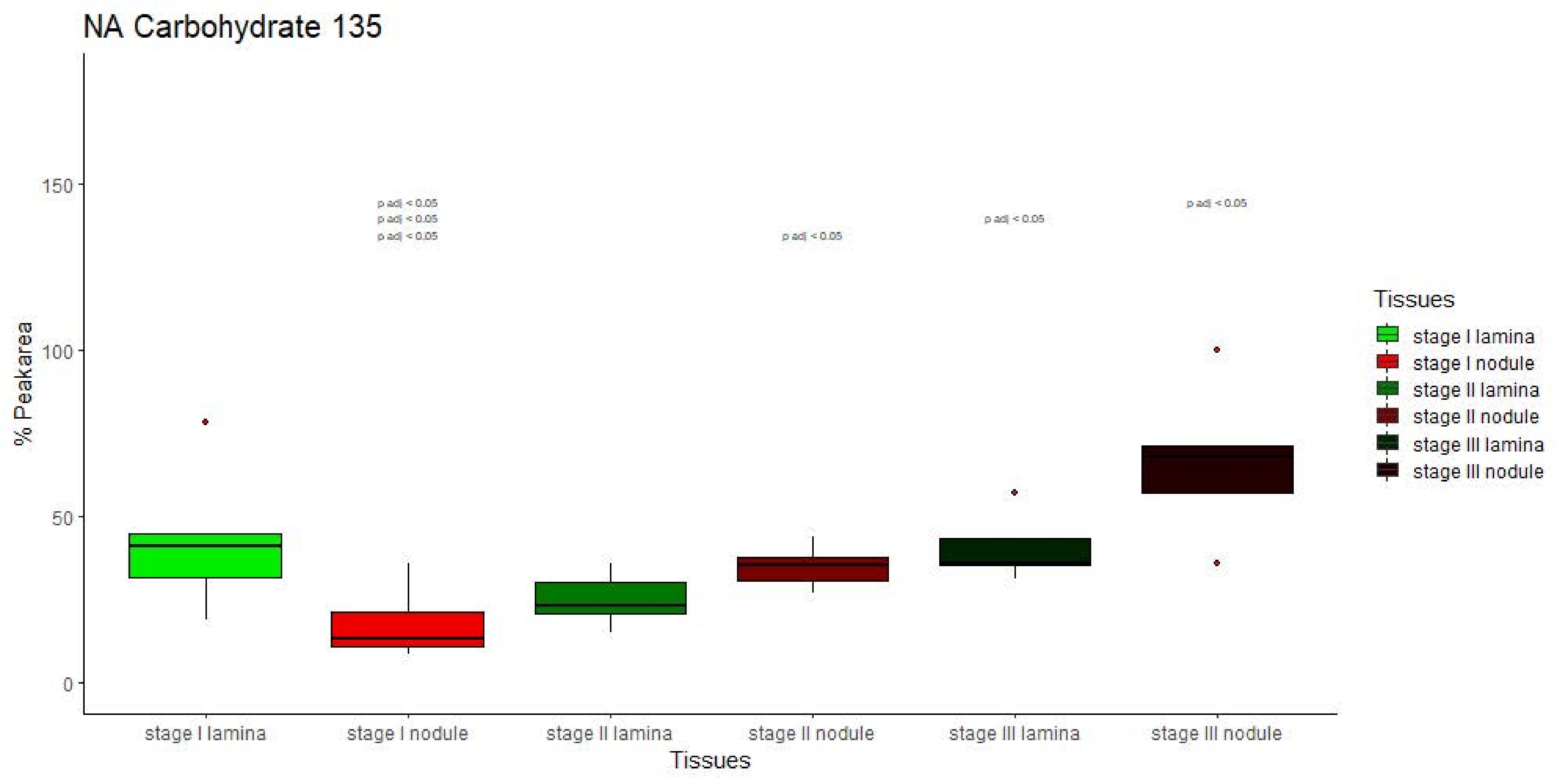
NA Carbohydrate 135
% Peakarea
150
100
50
0
p adj < 0.05
p adj < 0.05
p adj < 0.05
p adj < 0.05
p adj < 0.05
p adj < 0.05
stage I lamina
stage I nodule
stage II lamina
stage II nodule
stage III lamina
stage III nodule
Tissues
Tissues
stage I lamina
stage I nodule
stage II lamina
stage II nodule
stage III lamina
stage III nodule

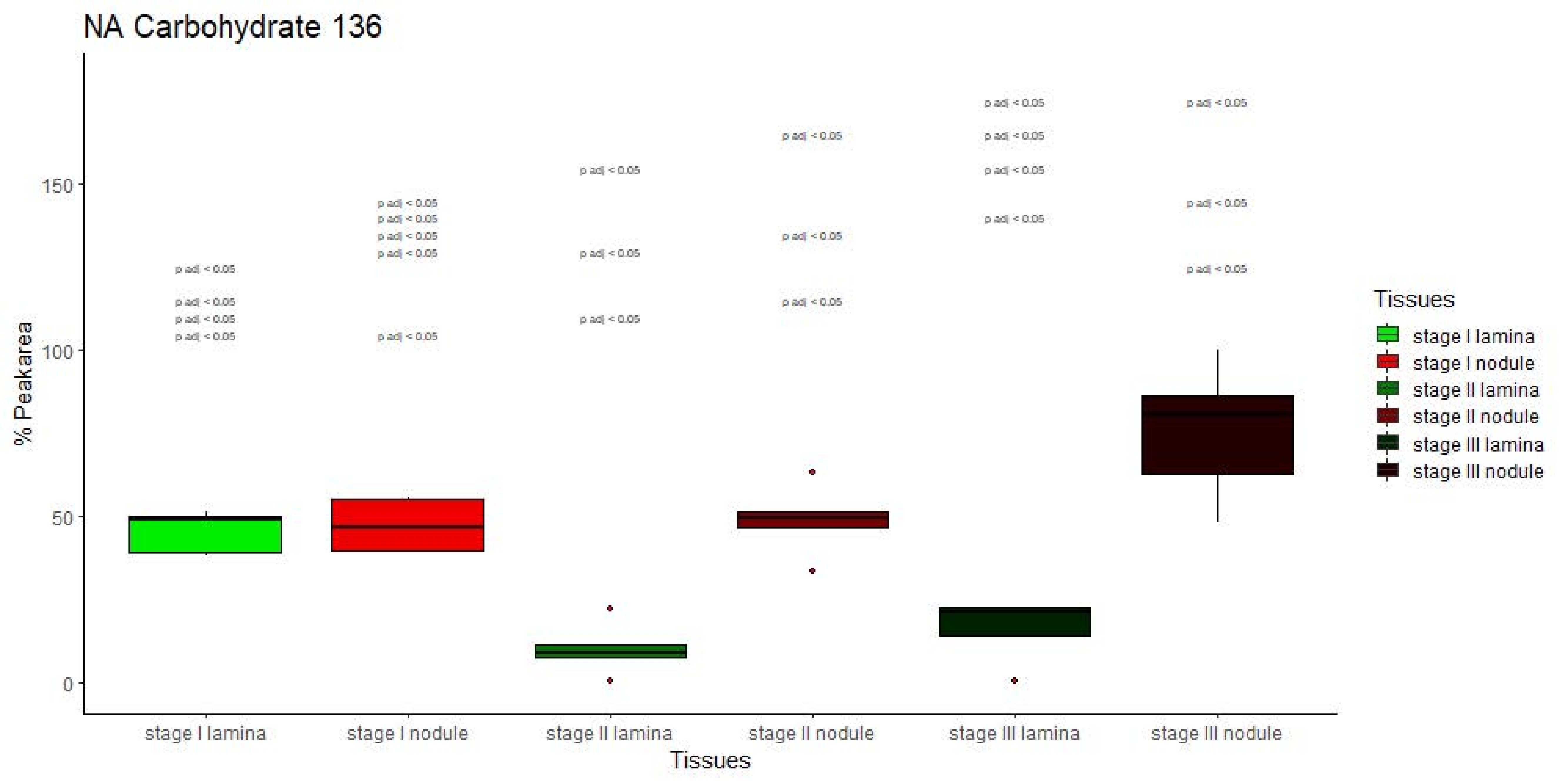

NA Carbohydrate 136
% Peakarea
150
100
50
0
p adj < 0.05
stage I lamina
stage I nodule
stage II lamina
stage II nodule
stage III lamina
stage III nodule
Tissues
Tissues
stage I lamina
stage I nodule
stage II lamina
stage II nodule
stage III lamina
stage III nodule

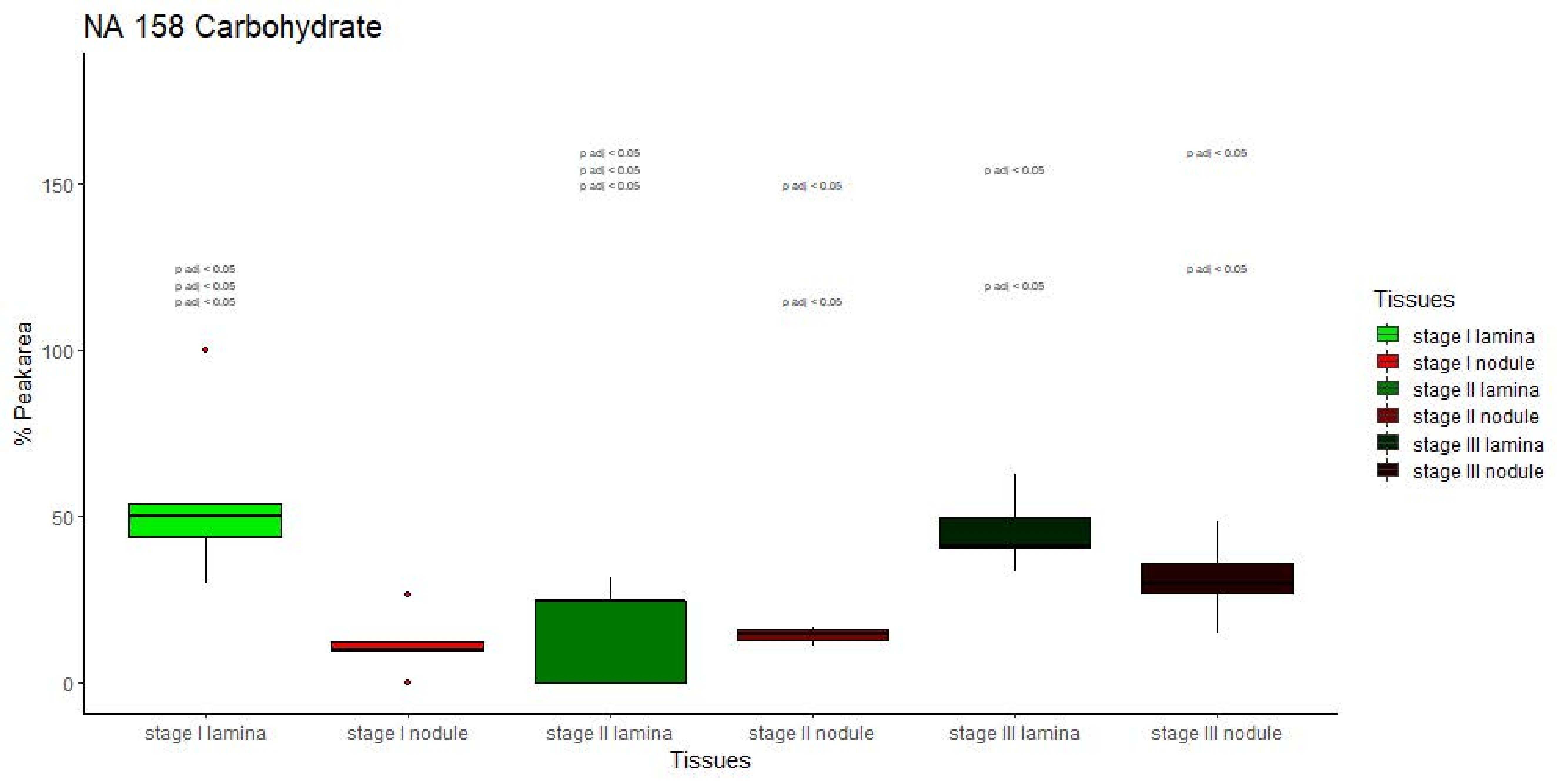

NA 158 Carbohydrate
% Peakarea
150
100
50
0
p adj < 0.05
Tissues
stage I lamina
stage I nodule
stage II lamina
stage II nodule
stage III lamina
stage III nodule

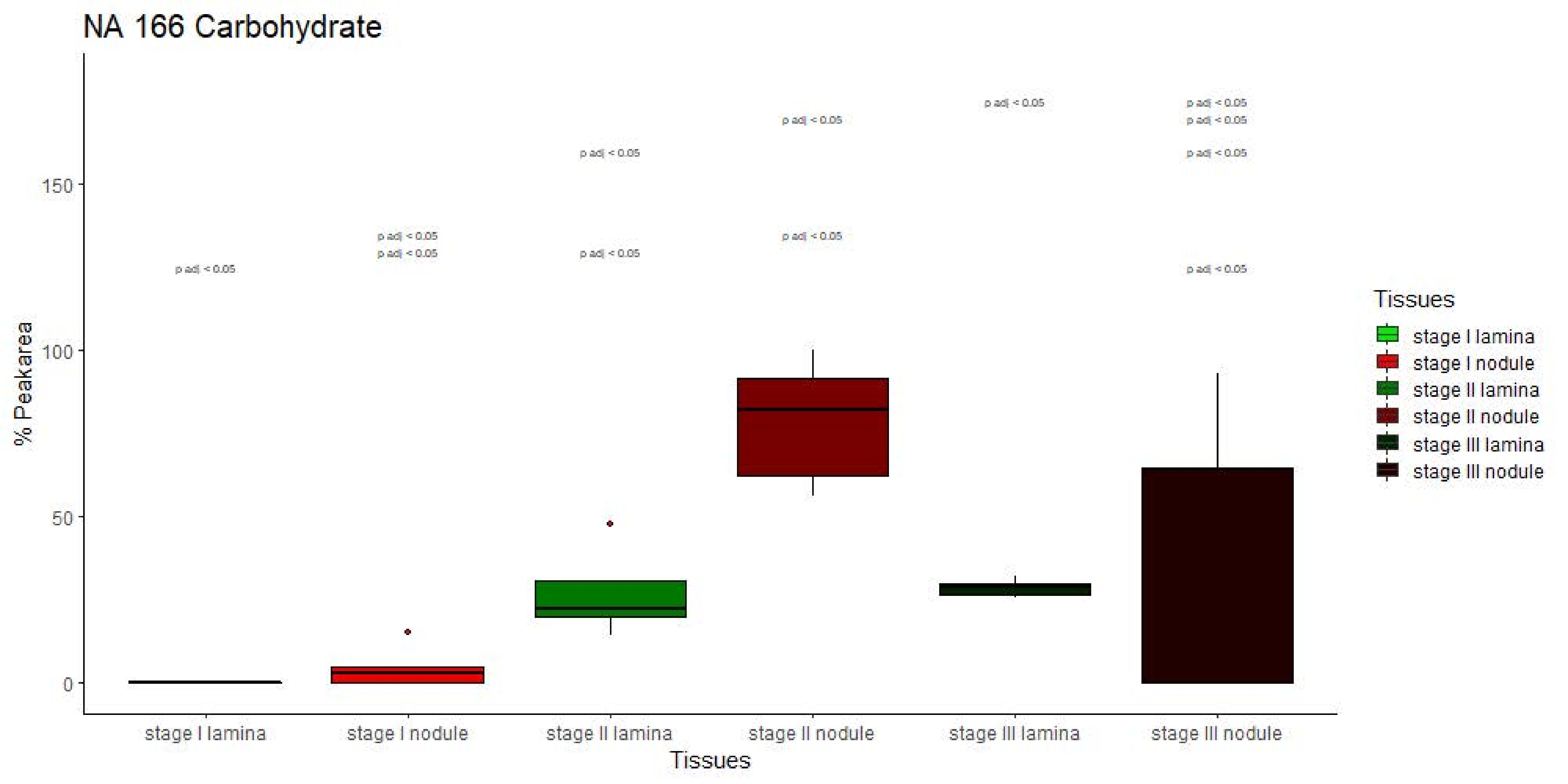
NA 166 Carbohydrate
% Peakarea
150
100
50
0
p adj < 0.05
p adj < 0.05
p adj < 0.05
p adj < 0.05
p adj < 0.05
p adj < 0.05
p adj < 0.05
p adj < 0.05
p adj < 0.05
p adj < 0.05
p adj < 0.05
p adj < 0.05
p adj < 0.05
stage I lamina
stage I nodule
stage II lamina
stage II nodule
stage III lamina
stage III nodule
Tissues
Tissues
stage I lamina
stage I nodule
stage II lamina
stage II nodule
stage III lamina
stage III nodule

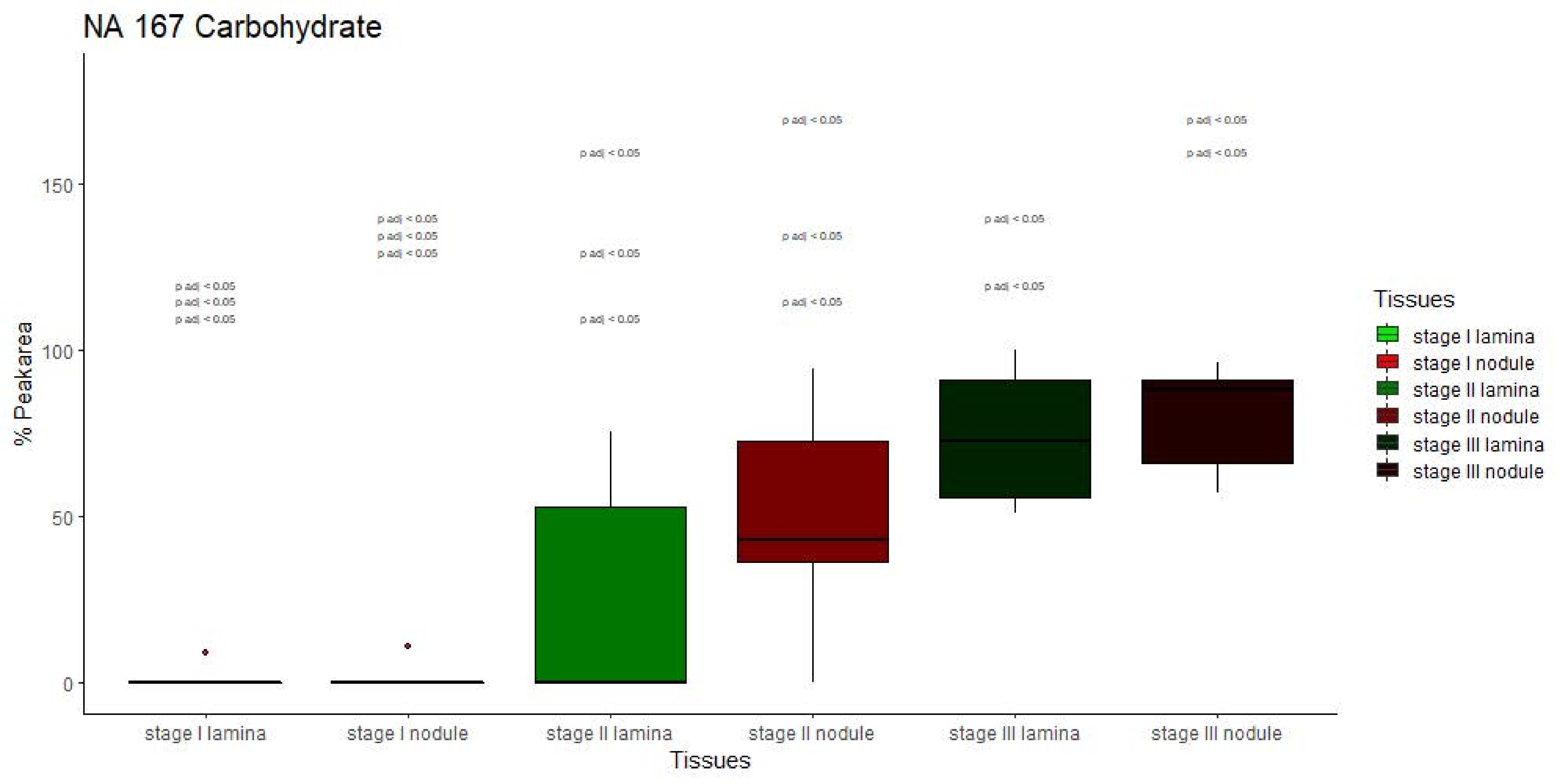
NA 167 Carbohydrate
% Peakarea
0
50
100
150
p adj < 0.05
Tissues
stage I lamina
stage I nodule
stage II lamina
stage II nodule
stage III lamina
stage III nodule

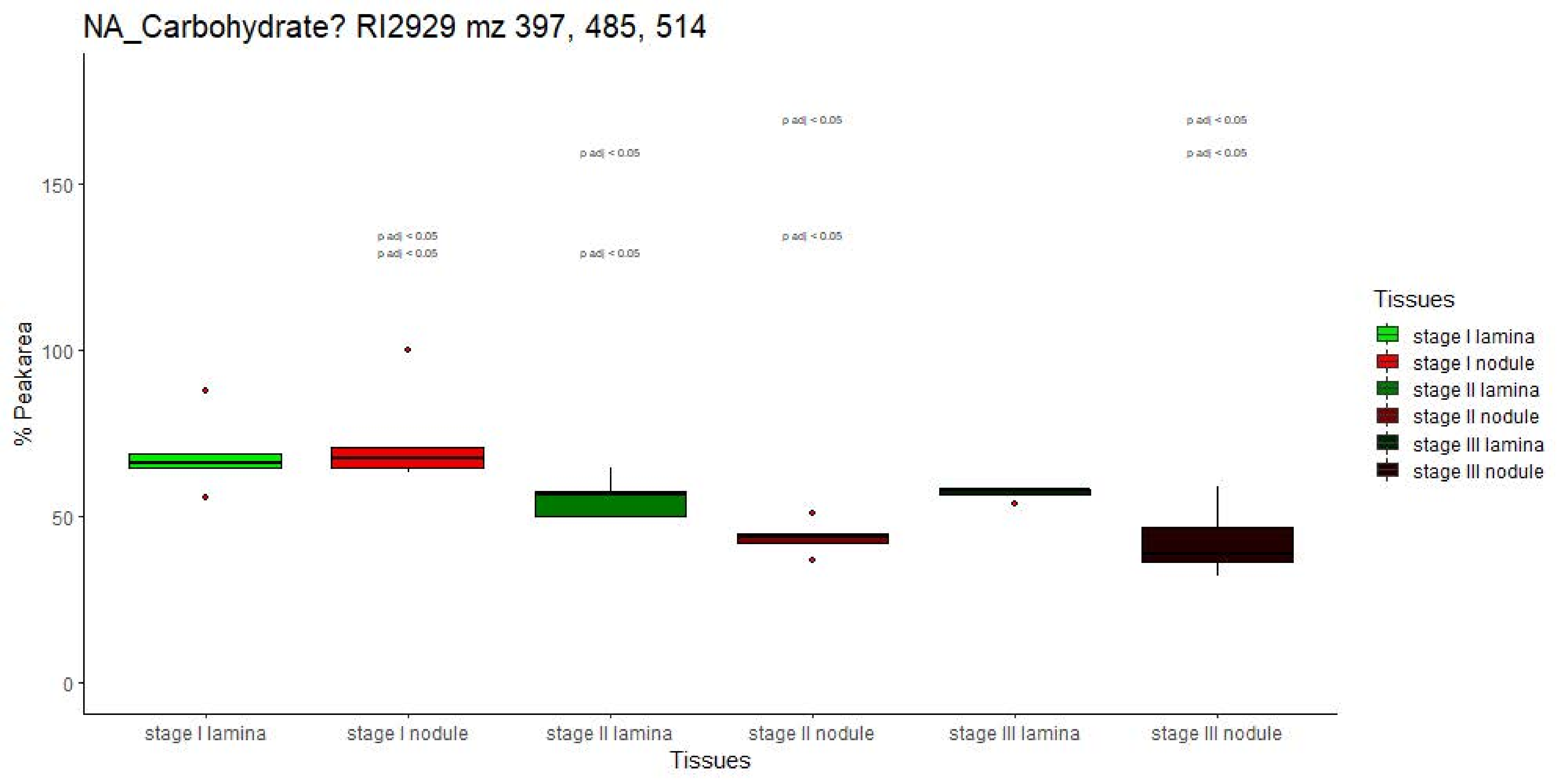

NA_Carbohydrate? RI2929 mz 397, 485, 514
p adj < 0.05
p adj < 0.05
p adj < 0.05
p adj < 0.05
p adj < 0.05
p adj < 0.05
p adj < 0.05
p adj < 0.05
150
100
50
0
% Peakarea
stage I lamina
stage I nodule
stage II lamina
stage II nodule
stage III lamina
stage III nodule
Tissues
Tissues
stage I lamina
stage I nodule
stage II lamina
stage II nodule
stage III lamina
stage III nodule

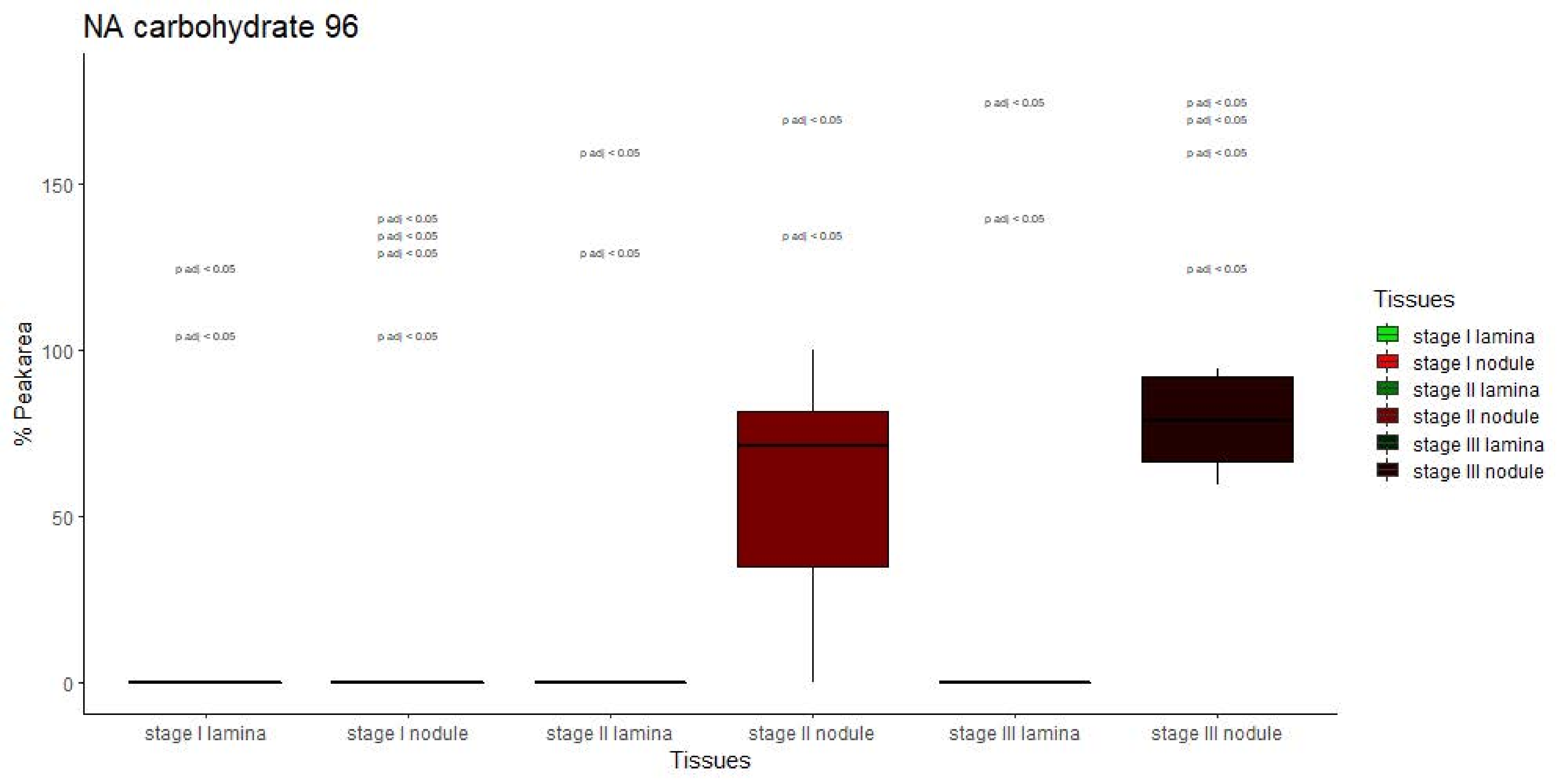

NA carbohydrate 96
% Peakarea
150
100
50
0
p adj < 0.05
Tissues
stage I lamina
stage I nodule
stage II lamina
stage II nodule
stage III lamina
stage III nodule

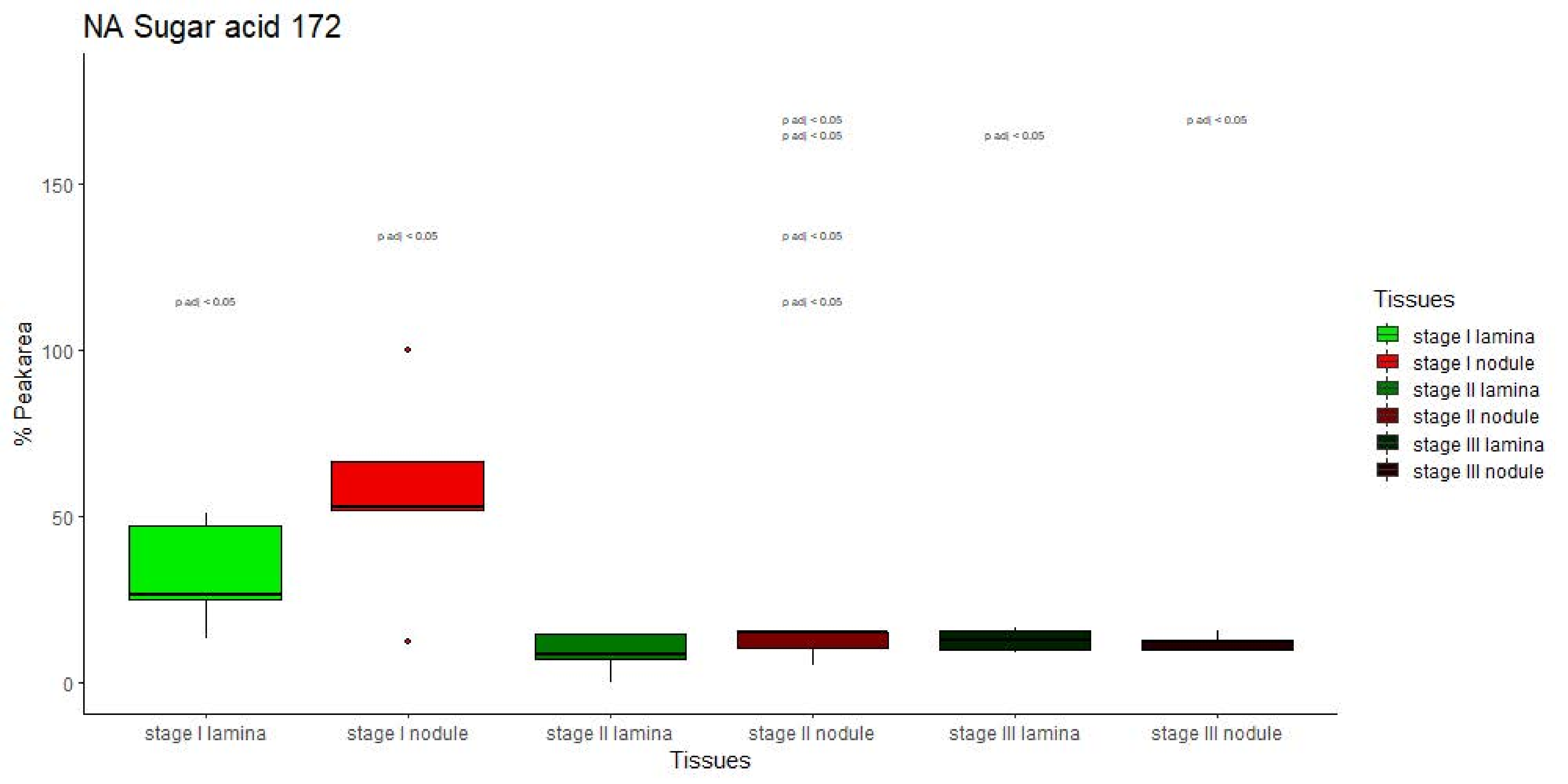
NA Sugar acid 172
p adj < 0.05
p adj < 0.05
p adj < 0.05
p adj < 0.05
p adj < 0.05
p adj < 0.05
p adj < 0.05
p adj < 0.05
150
100
50
0
% Peakarea
stage I lamina
stage I nodule
stage II lamina
stage II nodule
stage III lamina
stage III nodule
Tissues
Tissues
stage I lamina
stage I nodule
stage II lamina
stage II nodule
stage III lamina
stage III nodule

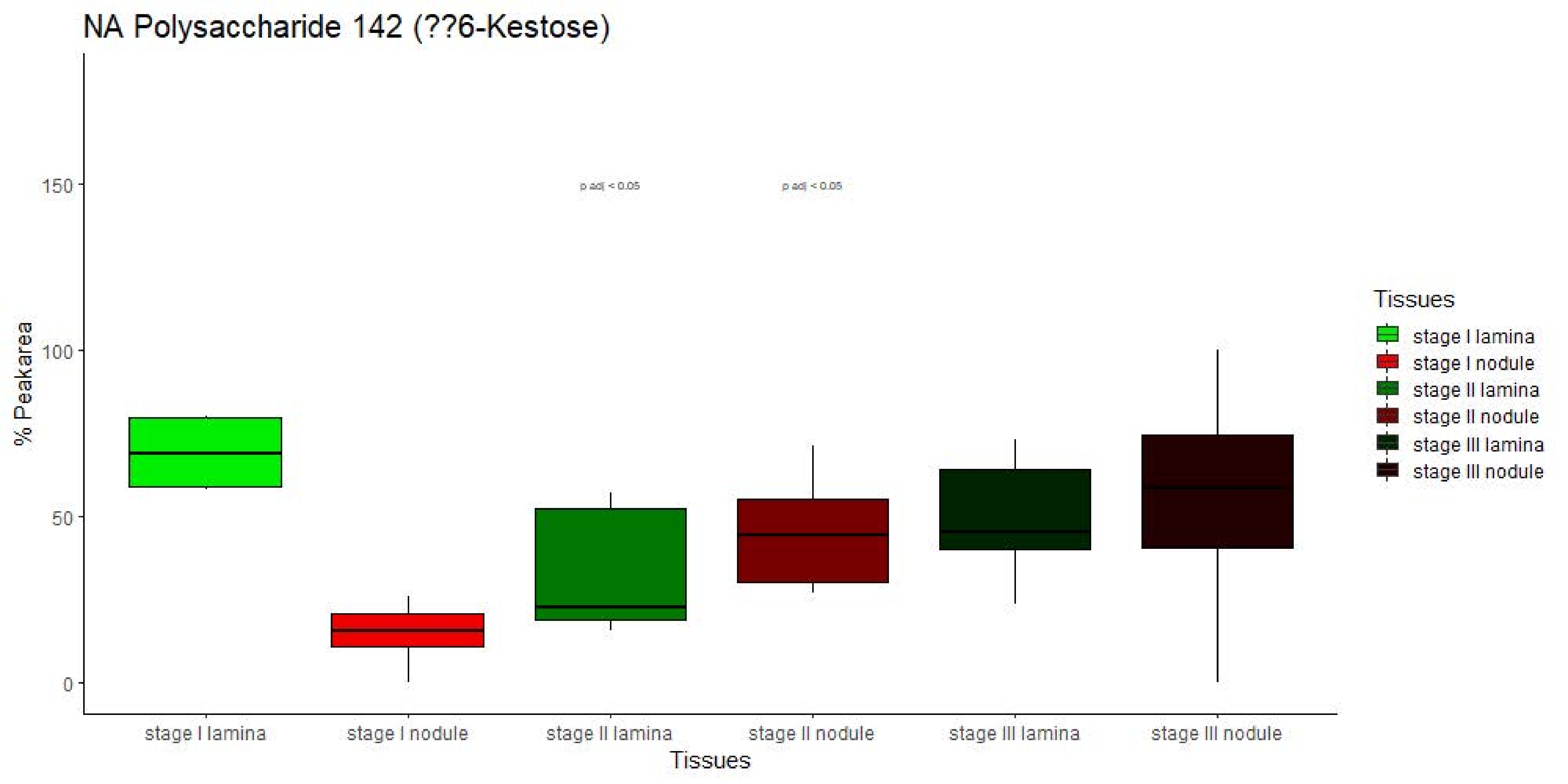
NA Polysaccharide 142 (??6-Kestose)
p adj < 0.05
p adj < 0.05
% Peakarea
0
50
100
150
stage I lamina
stage I nodule
stage II lamina
stage II nodule
stage III lamina
stage III nodule
Tissues
Tissues
stage I lamina
stage I nodule
stage II lamina
stage II nodule
stage III lamina
stage III nodule

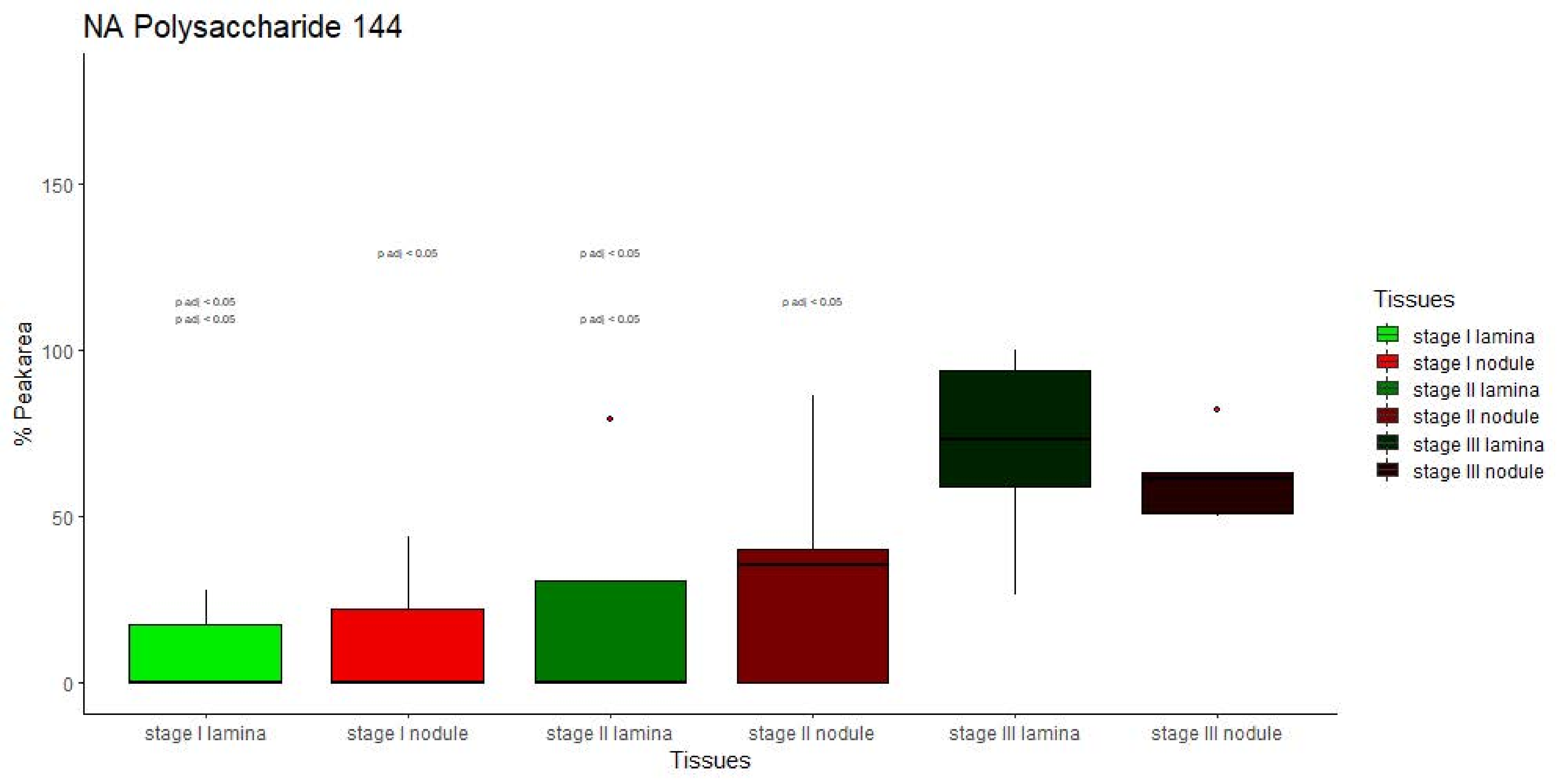
NA Polysaccharide 144
% Peakarea
150
100
50
0
p adj < 0.05
p adj < 0.05
p adj < 0.05
p adj < 0.05
p adj < 0.05
p adj < 0.05
stage I lamina
stage I nodule
stage II lamina
stage II nodule
stage III lamina
stage III nodule
Tissues
Tissues
stage I lamina
stage I nodule
stage II lamina
stage II nodule
stage III lamina
stage III nodule

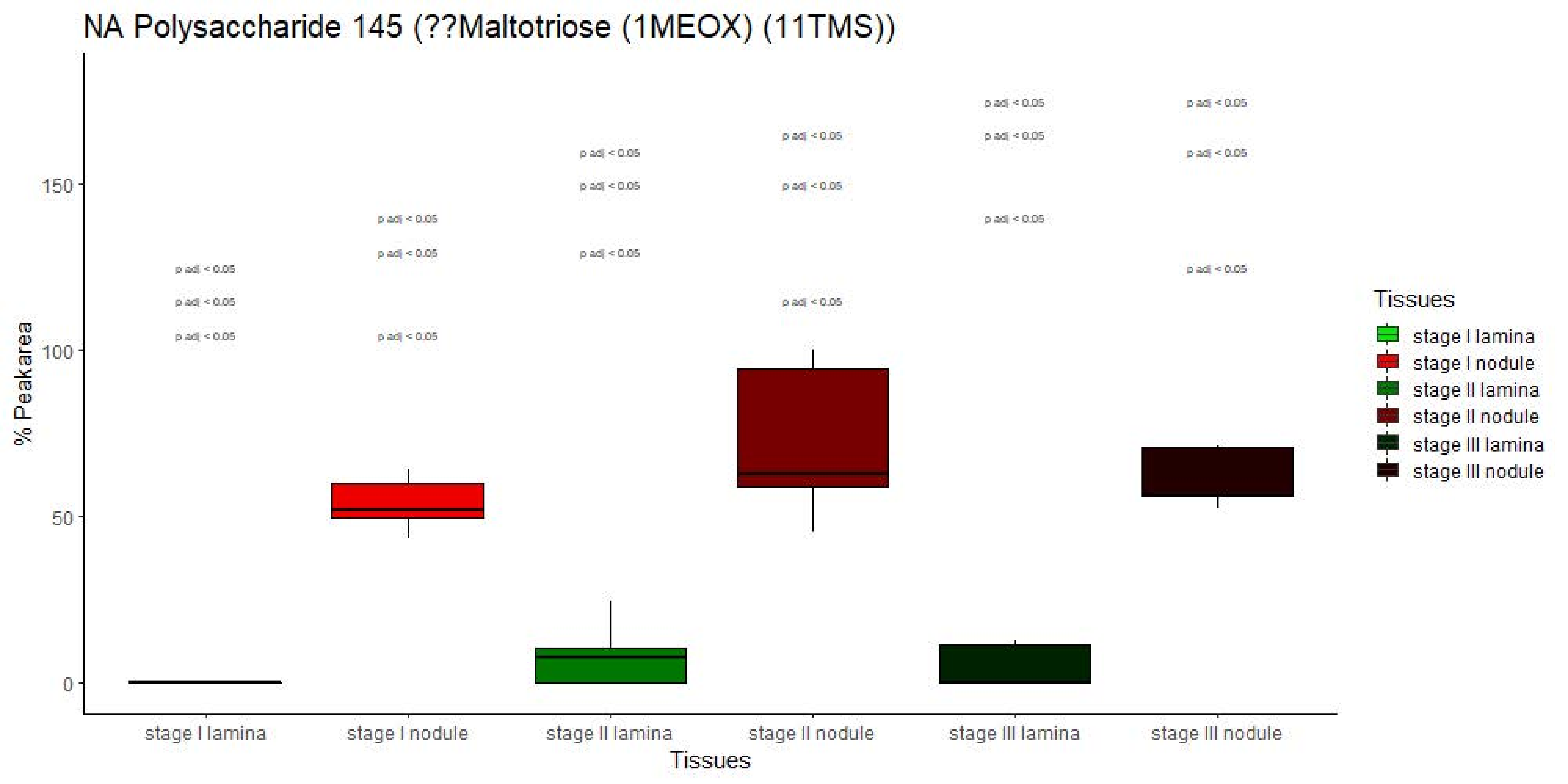
NA Polysaccharide 145 (??Maltotriose (1MEOX) (11TMS))
% Peakarea
0
50
100
150
p adj < 0.05
Tissues
stage I lamina
stage I nodule
stage II lamina
stage II nodule
stage III lamina
stage III nodule

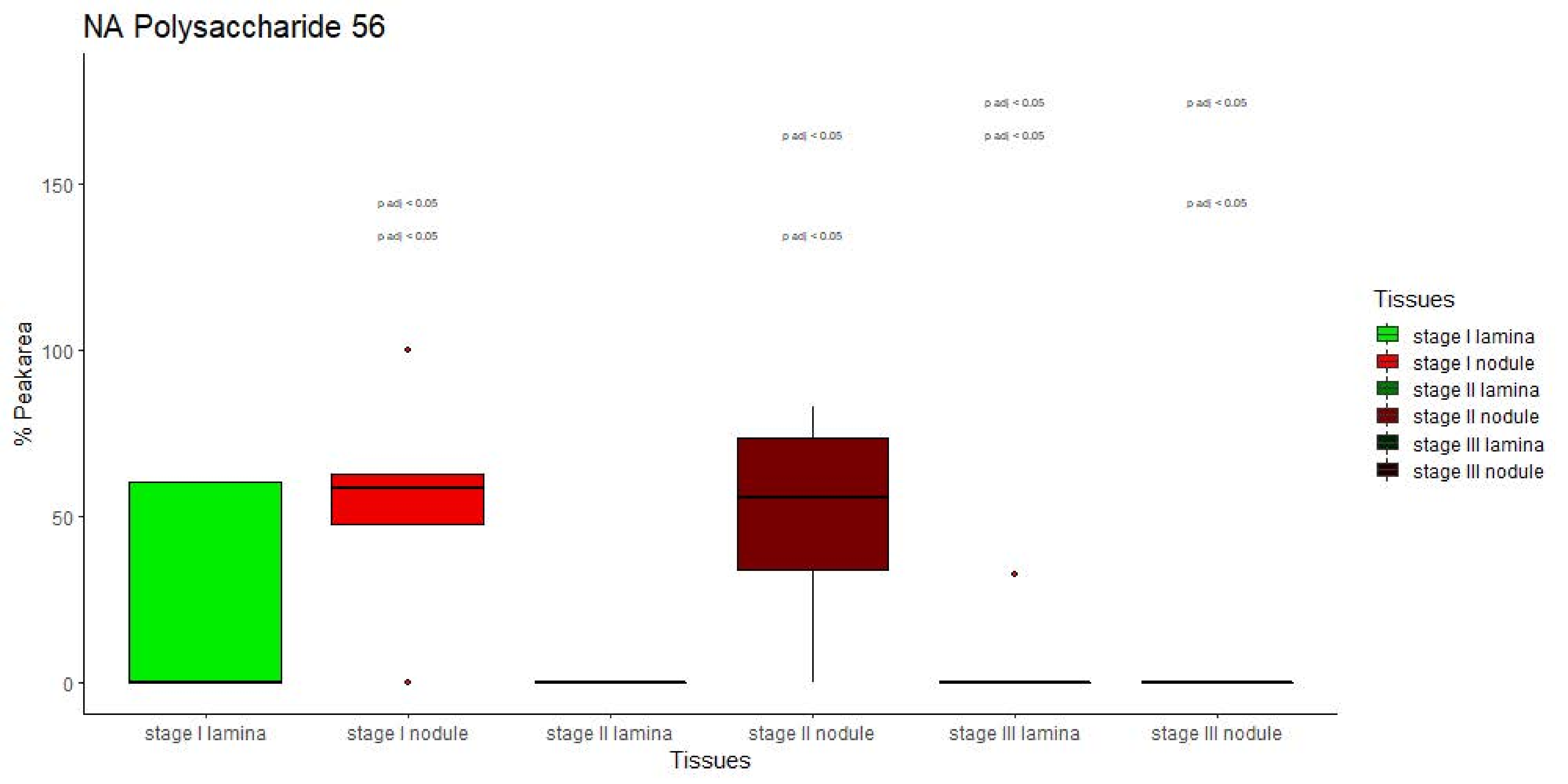
NA Polysaccharide 56
% Peakarea
0
50
100
150
p adj < 0.05
p adj < 0.05
p adj < 0.05
p adj < 0.05
p adj < 0.05
p adj < 0.05
p adj < 0.05
p adj < 0.05
stage I lamina
stage I nodule
stage II lamina
stage II nodule
stage III lamina
stage III nodule
Tissues
Tissues
stage I lamina
stage I nodule
stage II lamina
stage II nodule
stage III lamina
stage III nodule

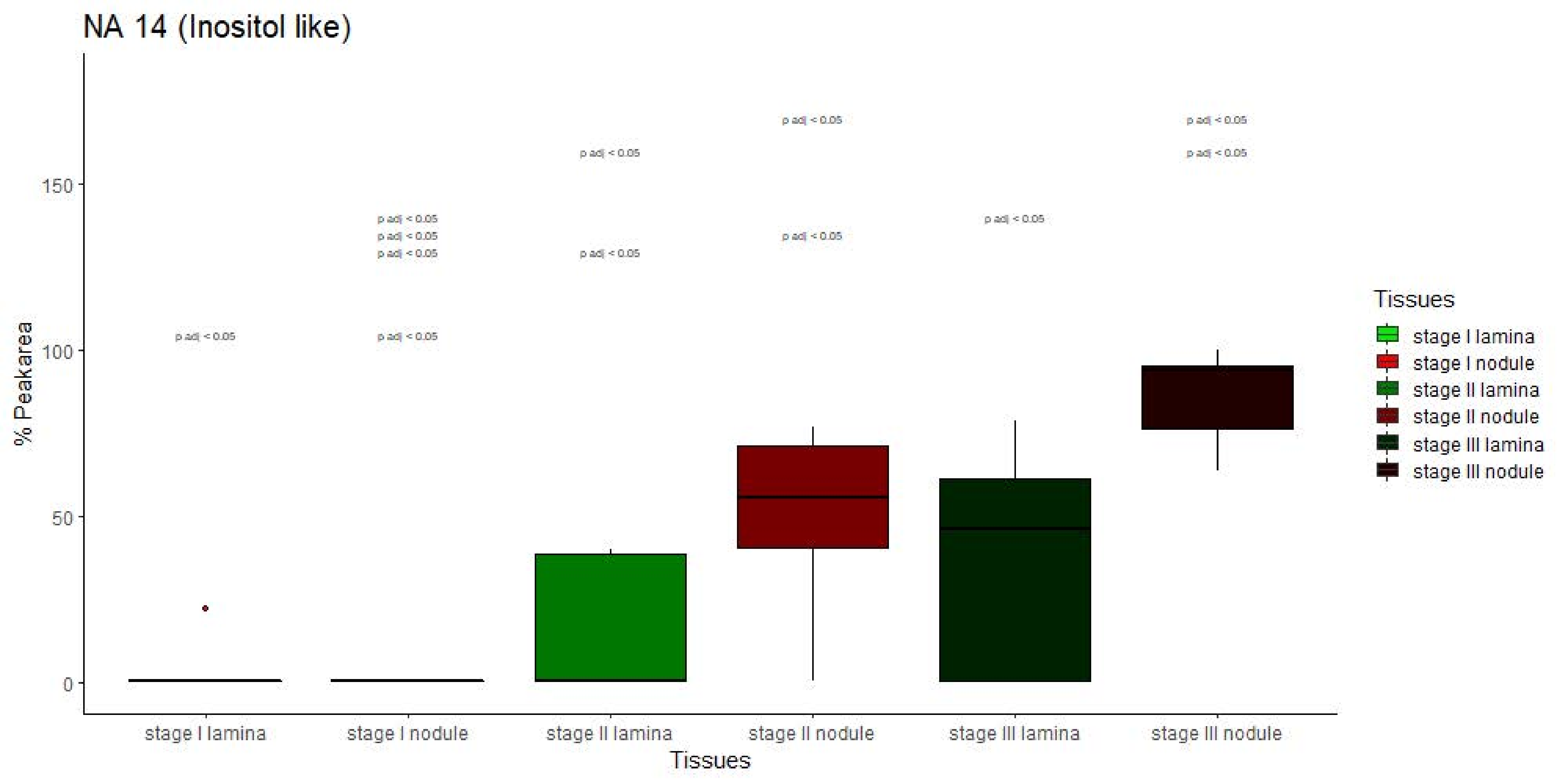
NA 14 (Inositol like)
% Peakarea
150
100
50
0
p adj < 0.05
stage I lamina
stage I nodule
stage II lamina
stage II nodule
stage III lamina
stage III nodule
Tissues
Tissues
stage I lamina
stage I nodule
stage II lamina
stage II nodule
stage III lamina
stage III nodule

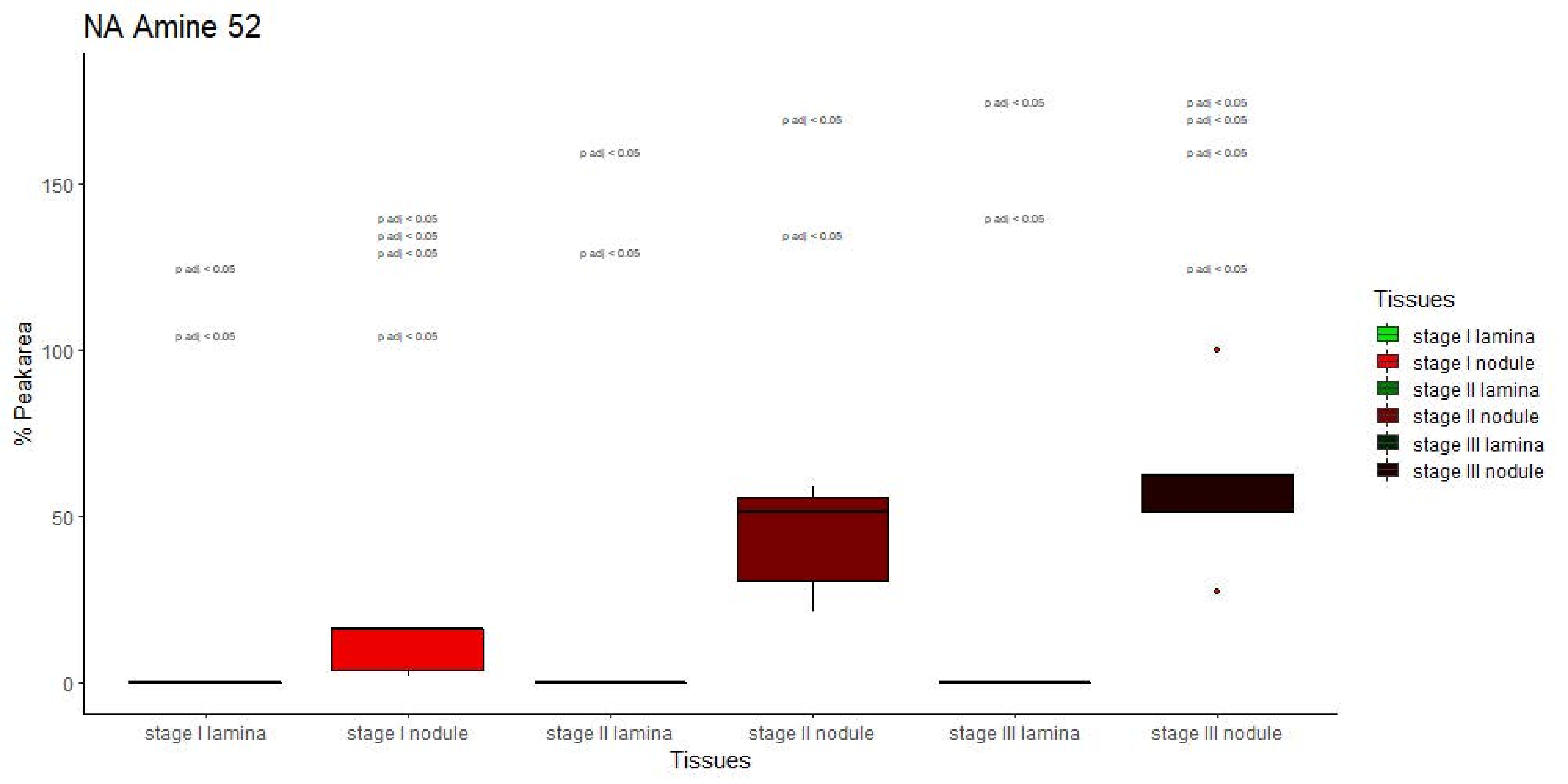

NA Amine 52
% Peakarea
0
50
100
150
p adj < 0.05
stage I lamina
stage I nodule
stage II lamina
stage II nodule
stage III lamina
stage III nodule
Tissues
Tissues
stage I lamina
stage I nodule
stage II lamina
stage II nodule
stage III lamina
stage III nodule

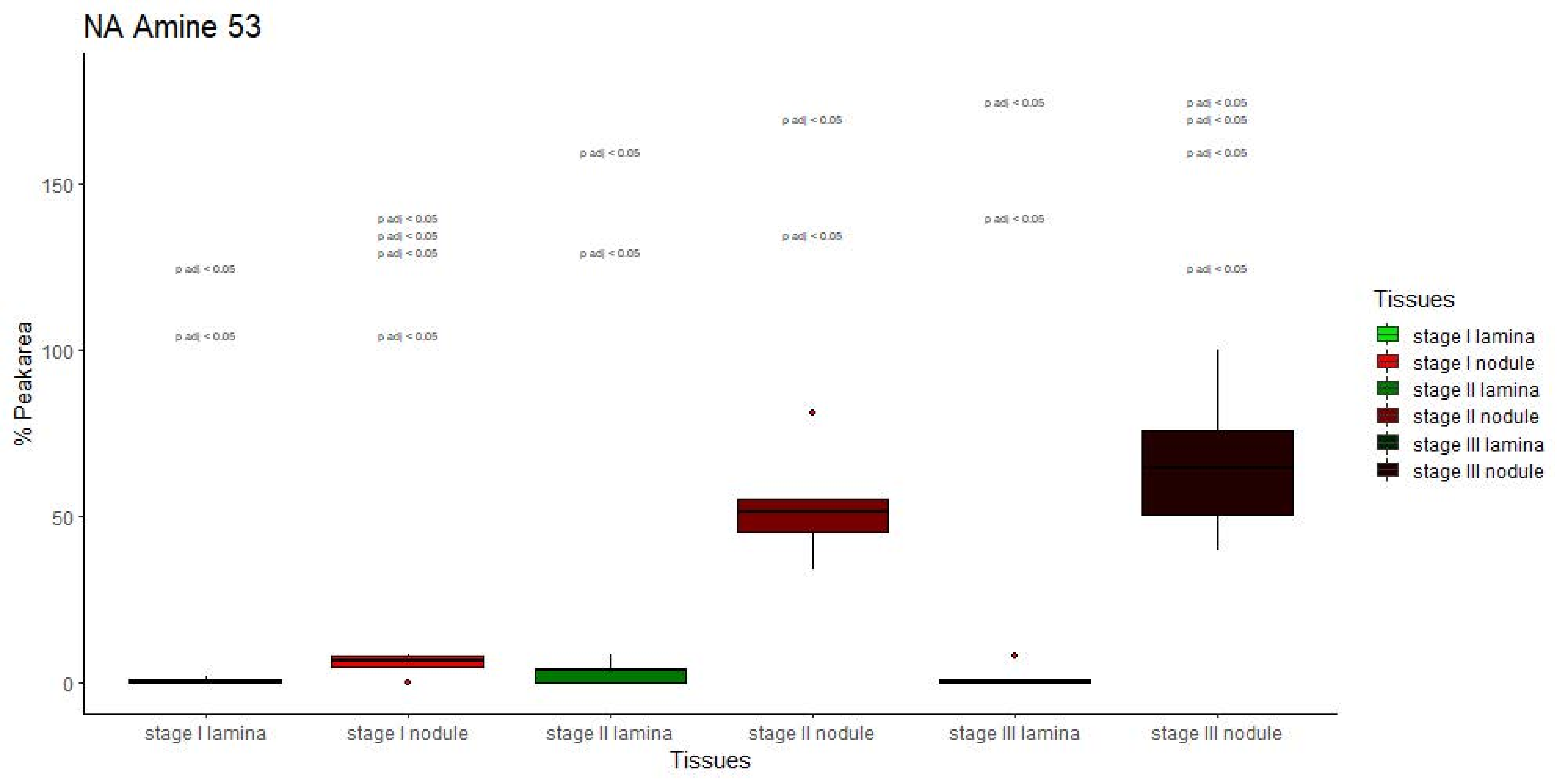
NA Amine 53
% Peakarea
150
100
50
0
p adj < 0.05
stage I lamina
stage I nodule
stage II lamina
stage II nodule
stage III lamina
stage III nodule
Tissues
Tissues
stage I lamina
stage I nodule
stage II lamina
stage II nodule
stage III lamina
stage III nodule

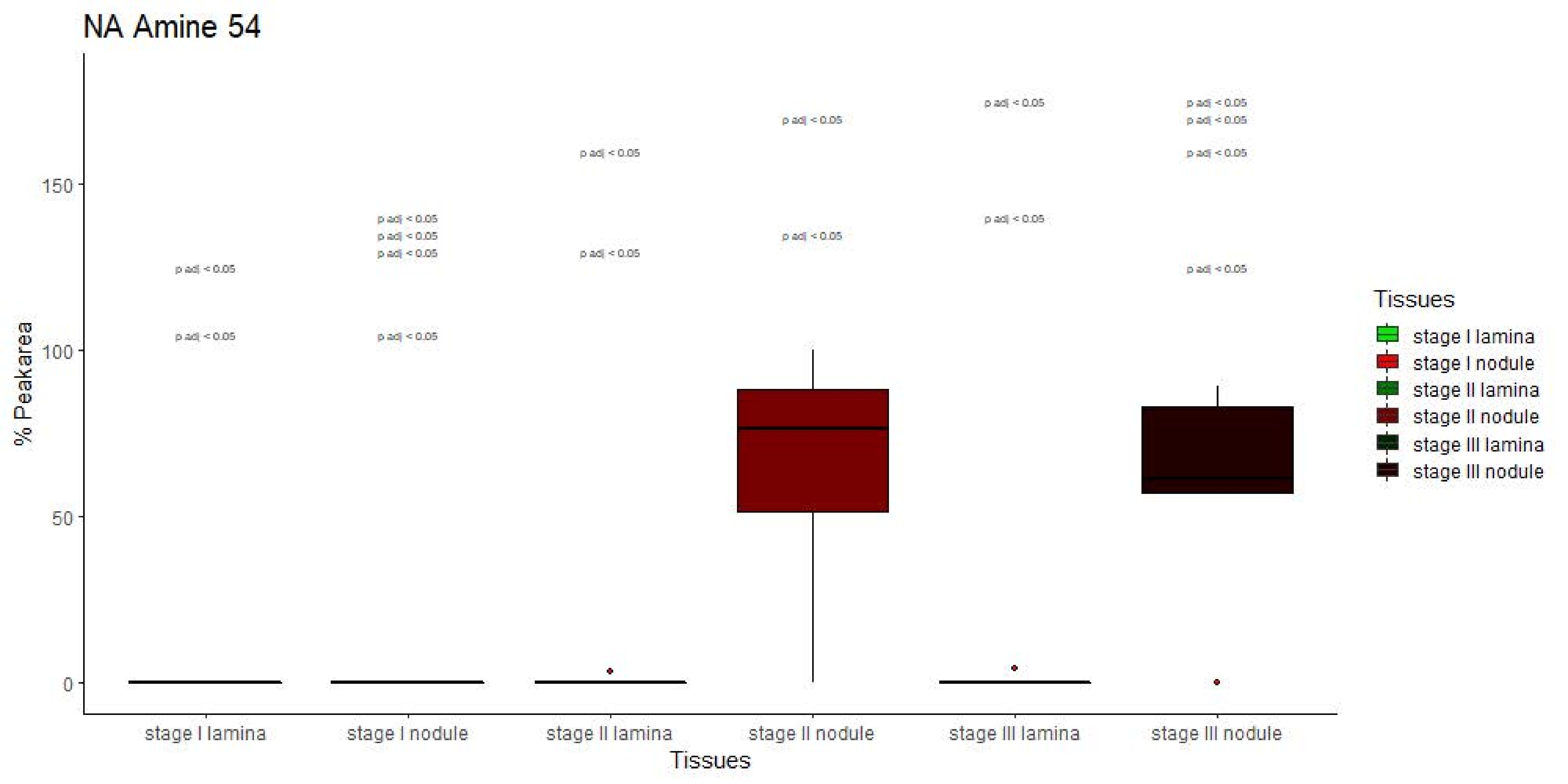

NA Amine 54
% Peakarea
150
100
50
0
p adj < 0.05
stage I lamina
stage I nodule
stage II lamina
stage II nodule
stage III lamina
stage III nodule
Tissues
Tissues
stage I lamina
stage I nodule
stage II lamina
stage II nodule
stage III lamina
stage III nodule

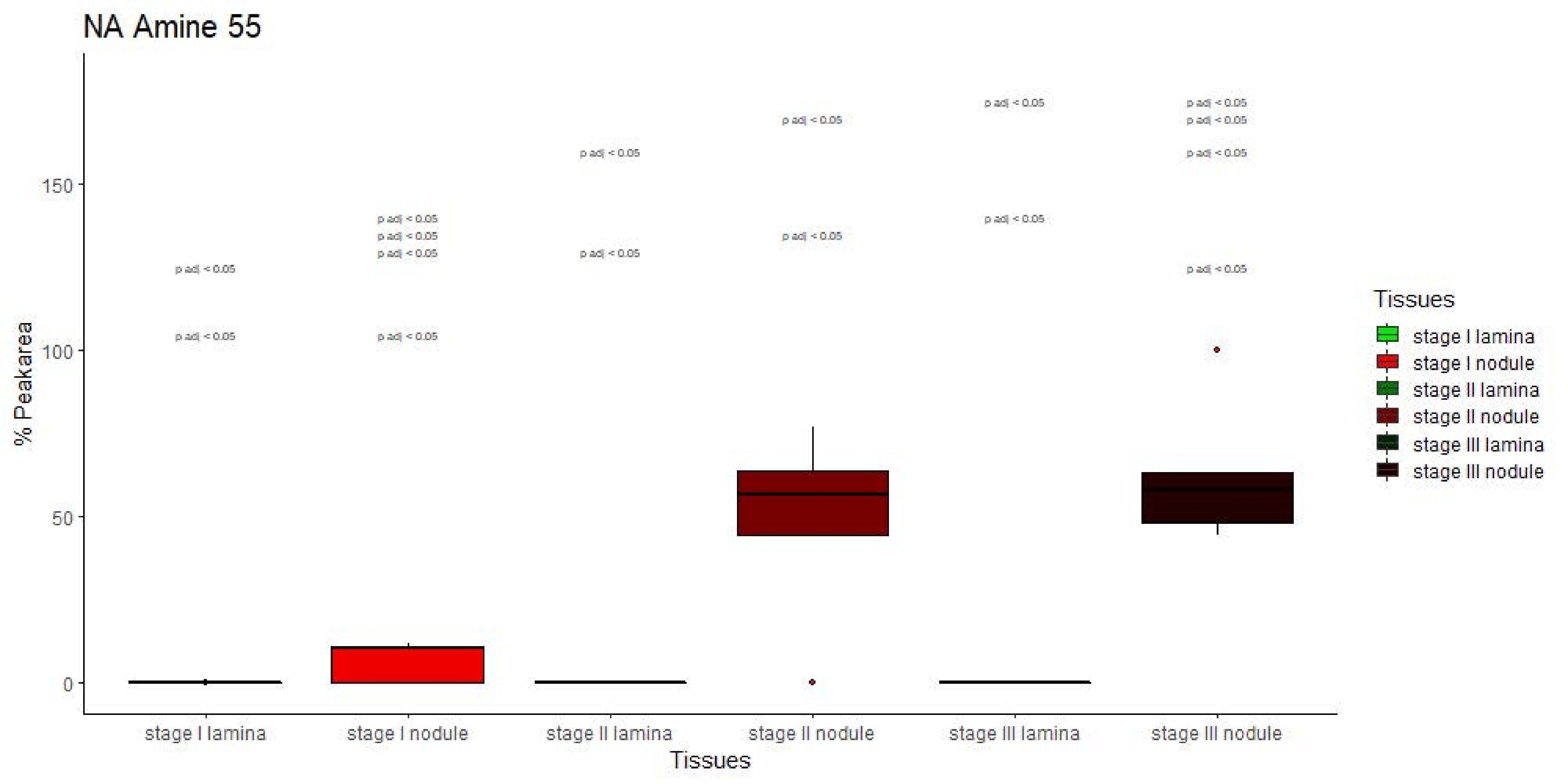
NA Amine 55
% Peakarea
150
100
50
0
p adj < 0.05
stage I lamina
stage I nodule
stage II lamina
stage II nodule
stage III lamina
stage III nodule
Tissues
Tissues
stage I lamina
stage I nodule
stage II lamina
stage II nodule
stage III lamina
stage III nodule

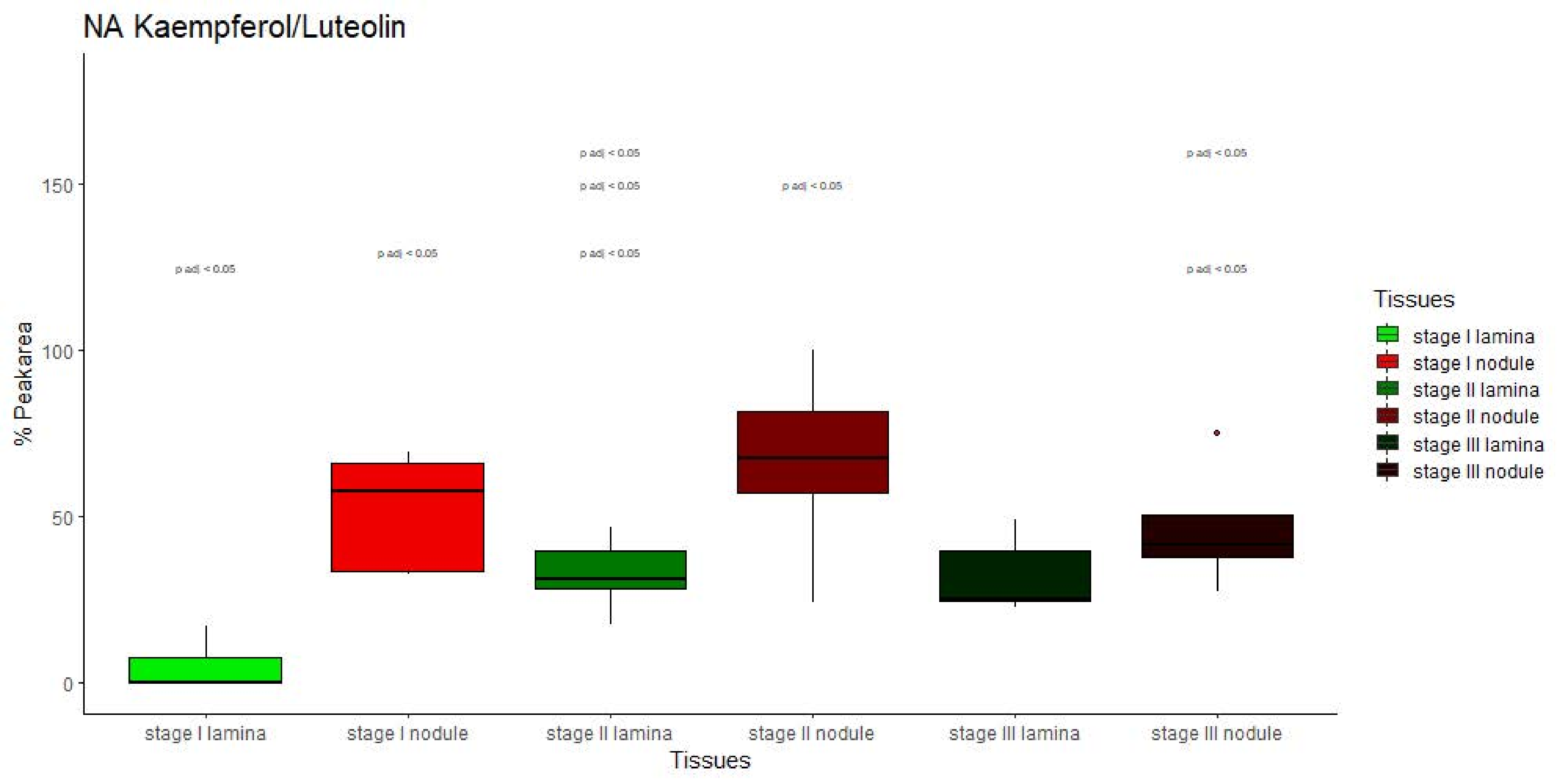

NA Kaempferol/Luteolin
p adj < 0.05
p adj < 0.05
p adj < 0.05
p adj < 0.05
p adj < 0.05
p adj < 0.05
p adj < 0.05
p adj < 0.05
p adj < 0.05
150
100
50
0
% Peakarea
stage I lamina
stage I nodule
stage II lamina
stage II nodule
stage III lamina
stage III nodule
Tissues
Tissues
stage I lamina
stage I nodule
stage II lamina
stage II nodule
stage III lamina
stage III nodule

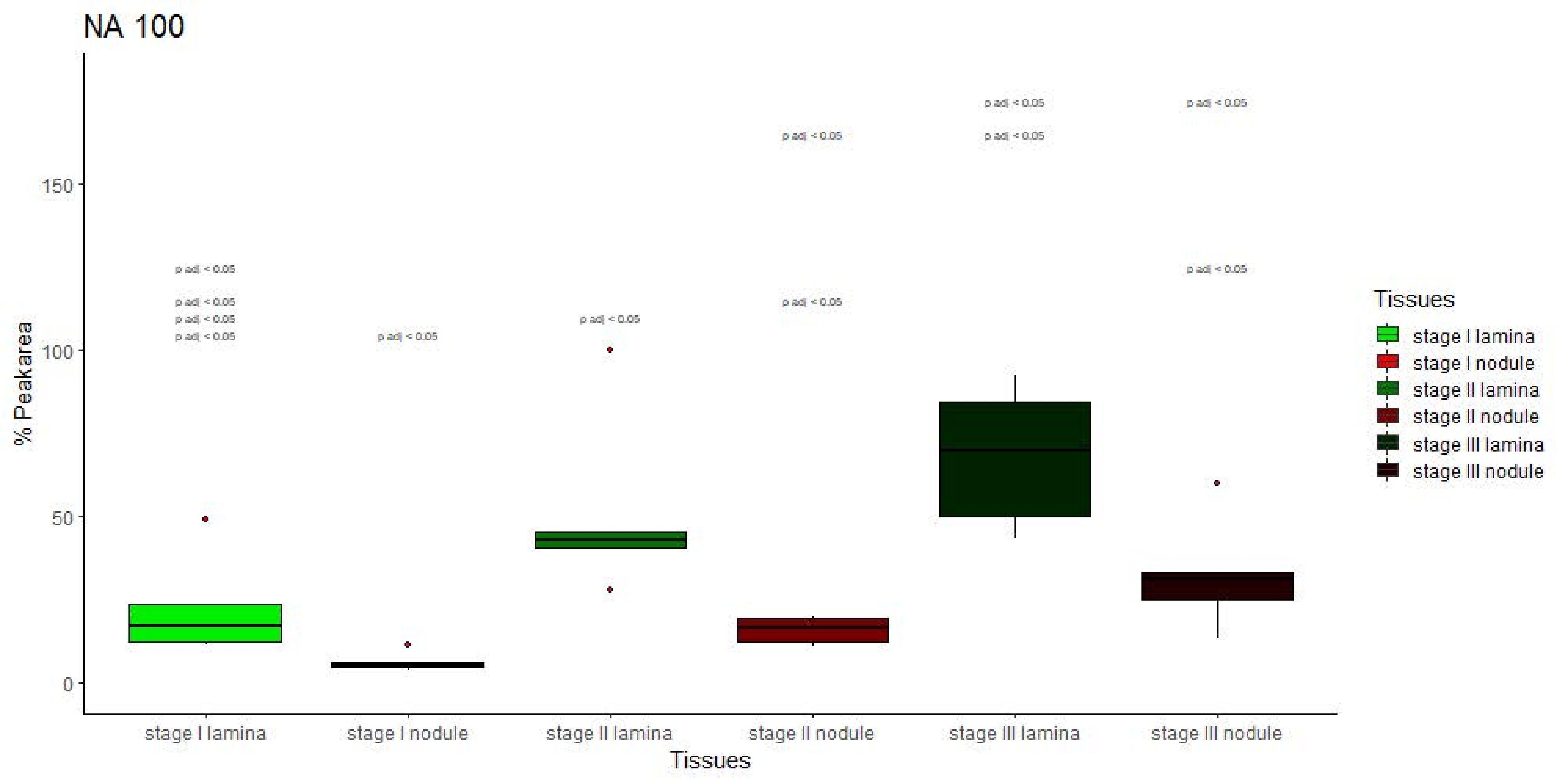

NA 100
p adj < 0.05
150
100
50
0
% Peakarea
Tissues
stage I lamina
stage I nodule
stage II lamina
stage II nodule
stage III lamina
stage III nodule

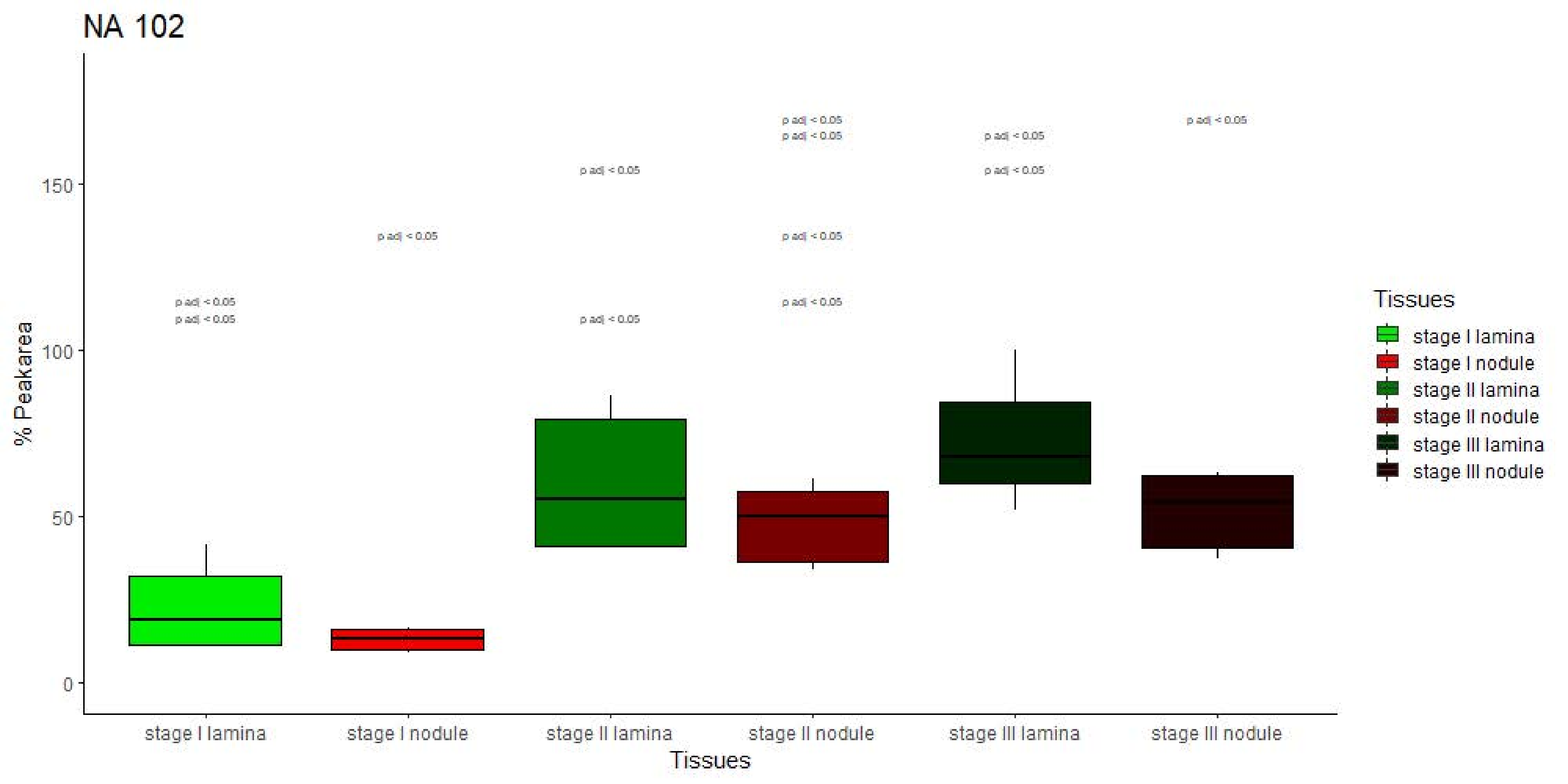

NA 102
% Peakarea
0
50
100
150
p adj < 0.05
stage I lamina
stage I nodule
stage II lamina
stage II nodule
stage III lamina
stage III nodule
Tissues
Tissues
stage I lamina
stage I nodule
stage II lamina
stage II nodule
stage III lamina
stage III nodule

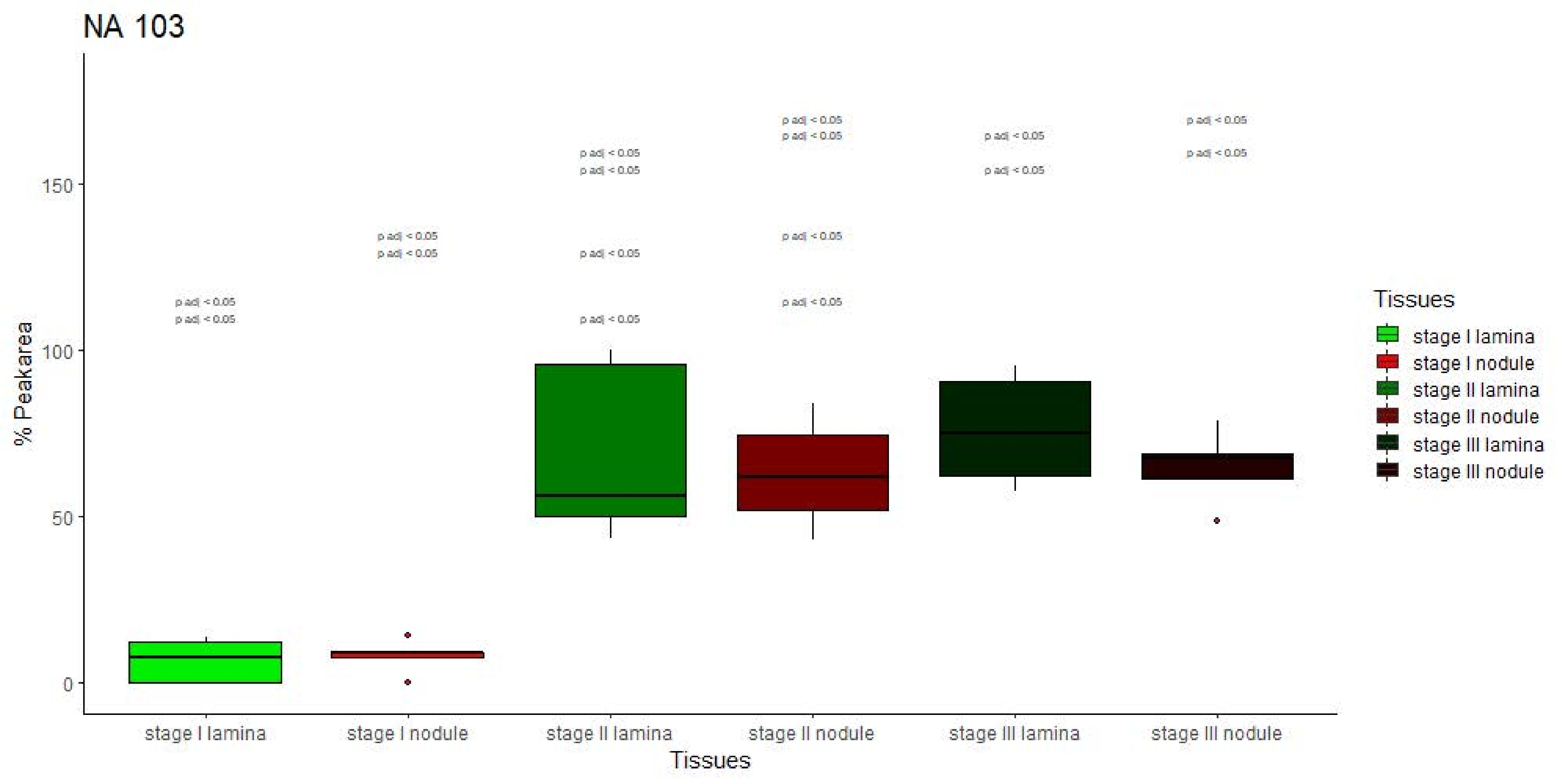

NA 103
% Peakarea
150
100
50
0
p adj < 0.05
stage I lamina
stage I nodule
stage II lamina
stage II nodule
stage III lamina
stage III nodule
Tissues
Tissues
stage I lamina
stage I nodule
stage II lamina
stage II nodule
stage III lamina
stage III nodule

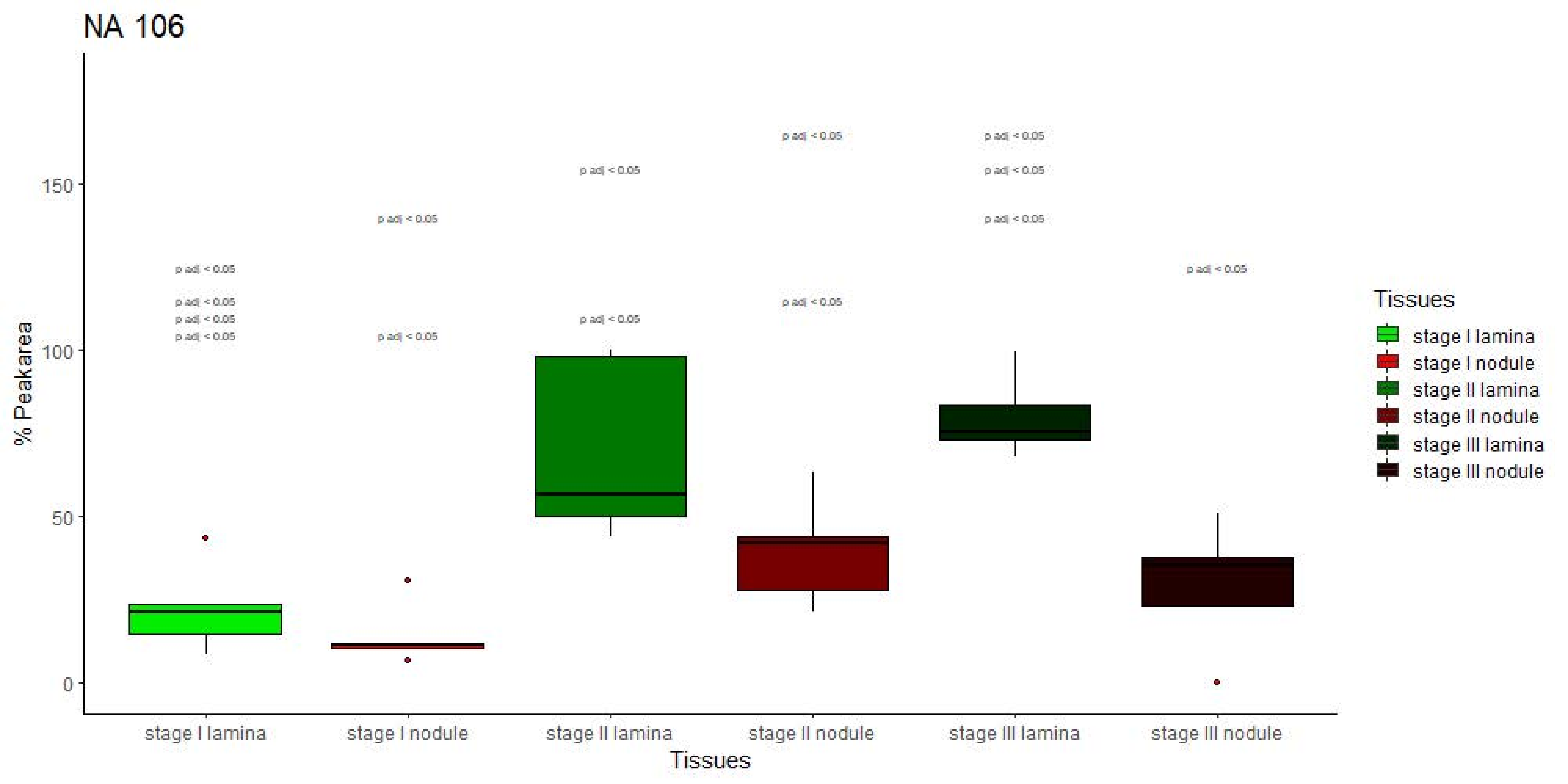

NA 106
% Peakarea
150
100
50
0
p adj < 0.05
stage I lamina
stage I nodule
stage II lamina
stage II nodule
stage III lamina
stage III nodule
Tissues
Tissues
stage I lamina
stage I nodule
stage II lamina
stage II nodule
stage III lamina
stage III nodule

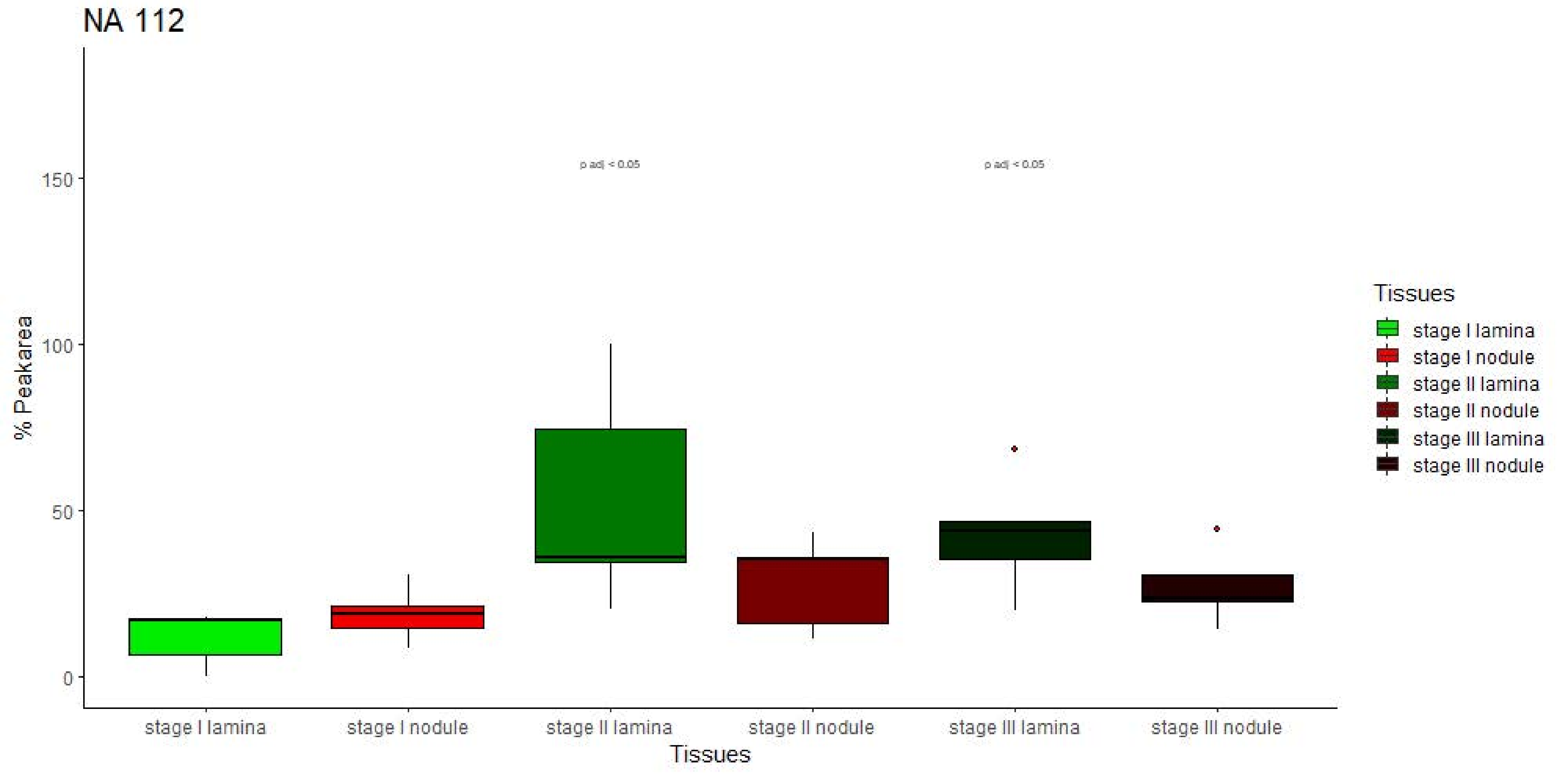
NA 112
p adj < 0.05
p adj < 0.05
150
100
50
0
% Peakarea
stage I lamina
stage I nodule
stage II lamina
stage II nodule
stage III lamina
stage III nodule
Tissues
Tissues
stage I lamina
stage I nodule
stage II lamina
stage II nodule
stage III lamina
stage III nodule

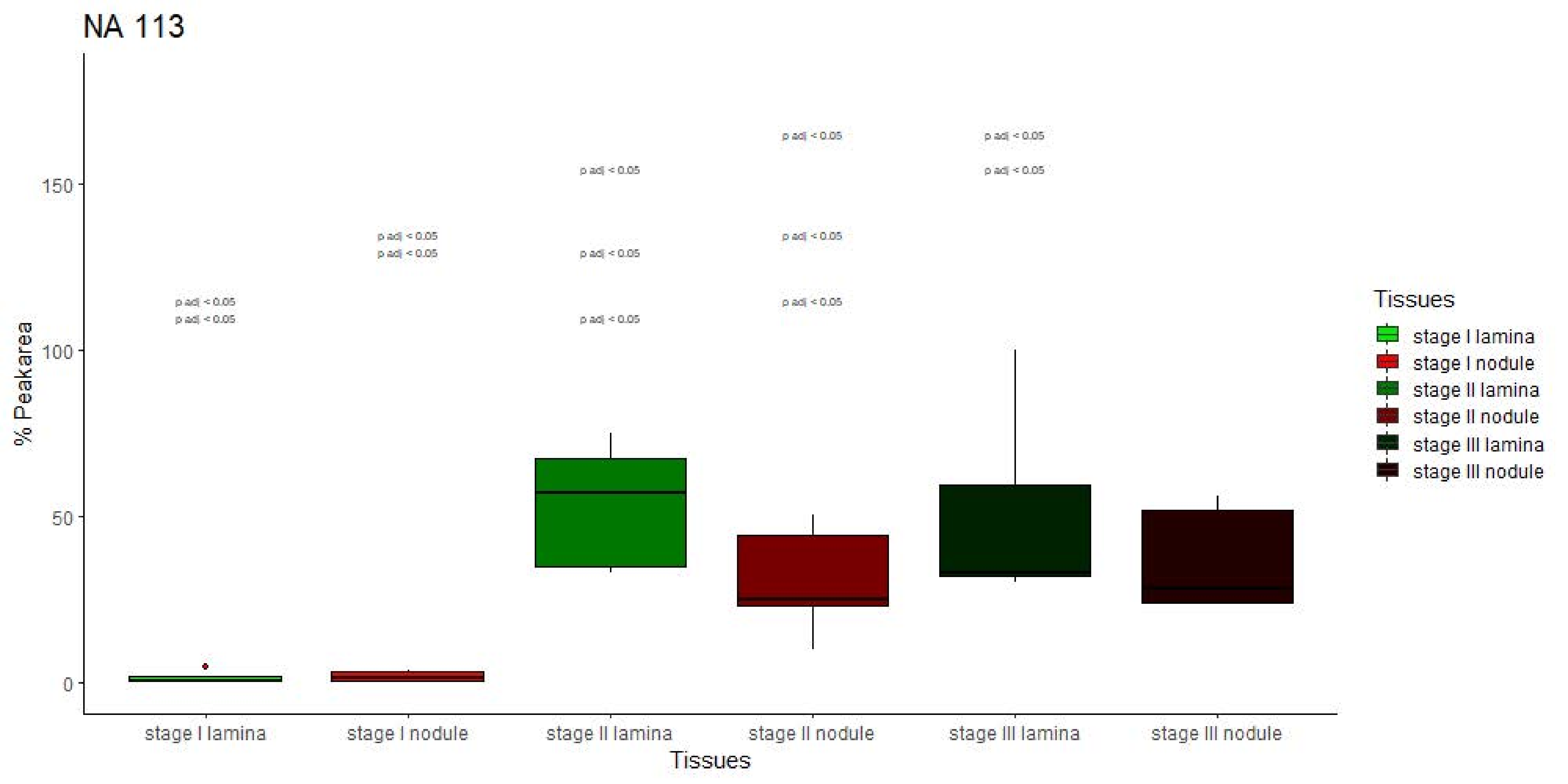
NA 113
% Peakarea
0
50
100
150
p adj < 0.05
Tissues
stage I lamina
stage I nodule
stage II lamina
stage II nodule
stage III lamina
stage III nodule

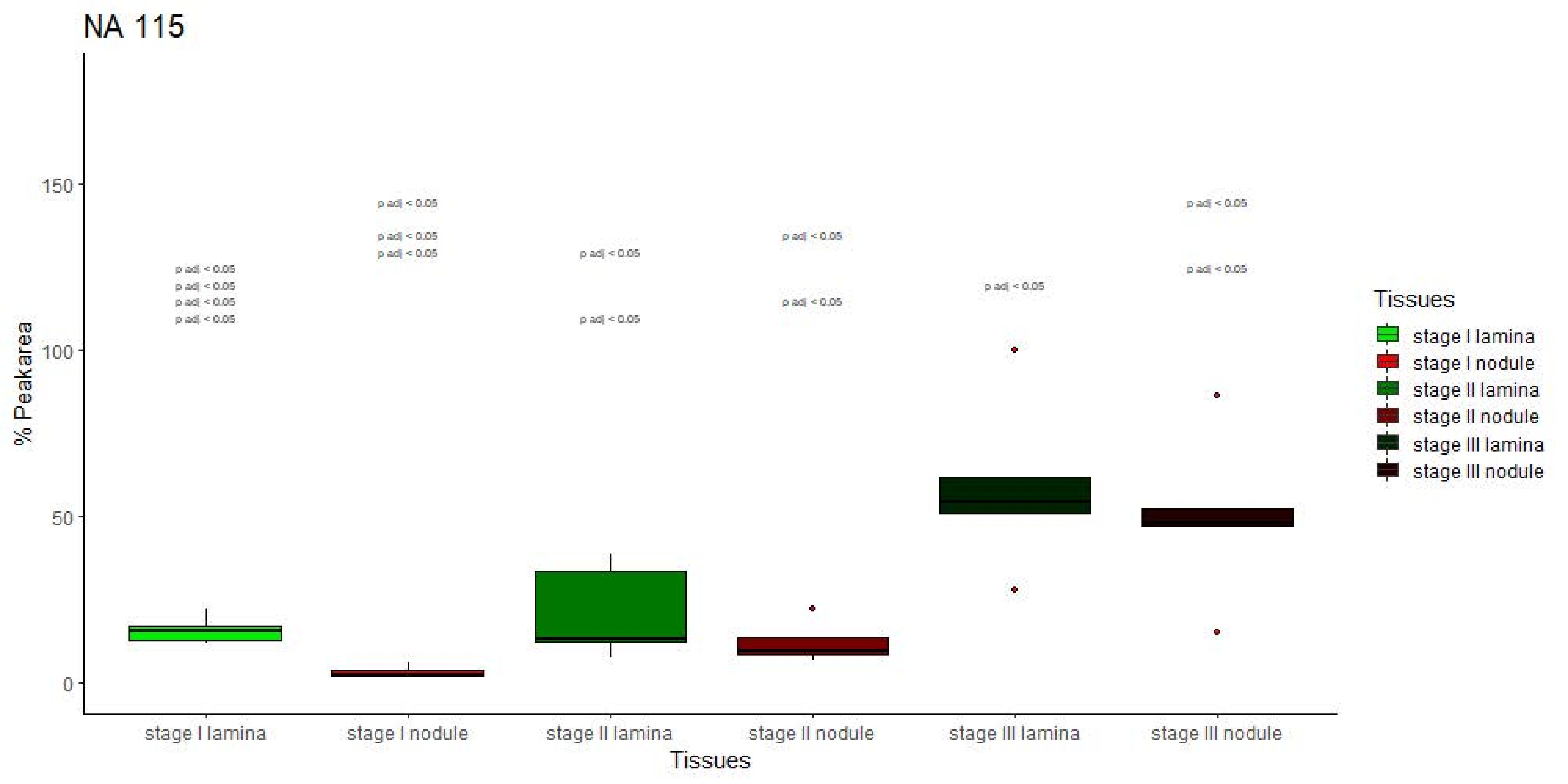

NA 115
% Peakarea
150
100
50
0
p adj < 0.05
stage I lamina
stage I nodule
stage II lamina
stage II nodule
stage III lamina
stage III nodule
Tissues
Tissues
stage I lamina
stage I nodule
stage II lamina
stage II nodule
stage III lamina
stage III nodule

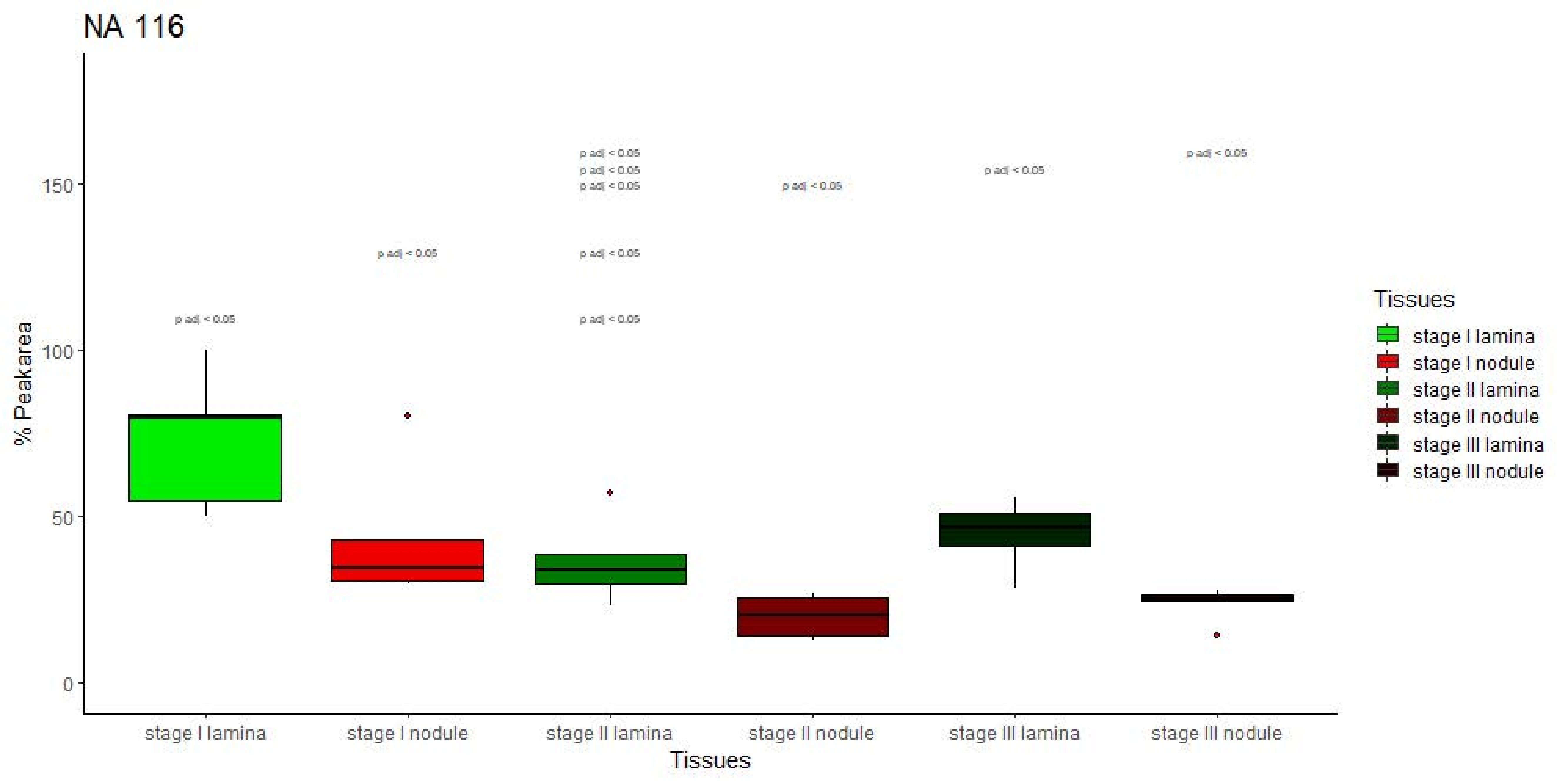
NA 116
% Peakarea
150
100
50
0
p adj < 0.05
p adj < 0.05
p adj < 0.05
p adj < 0.05
p adj < 0.05
p adj < 0.05
p adj < 0.05
p adj < 0.05
p adj < 0.05
p adj < 0.05
stage I lamina
stage I nodule
stage II lamina
stage II nodule
stage III lamina
stage III nodule
Tissues
Tissues
stage I lamina
stage I nodule
stage II lamina
stage II nodule
stage III lamina
stage III nodule

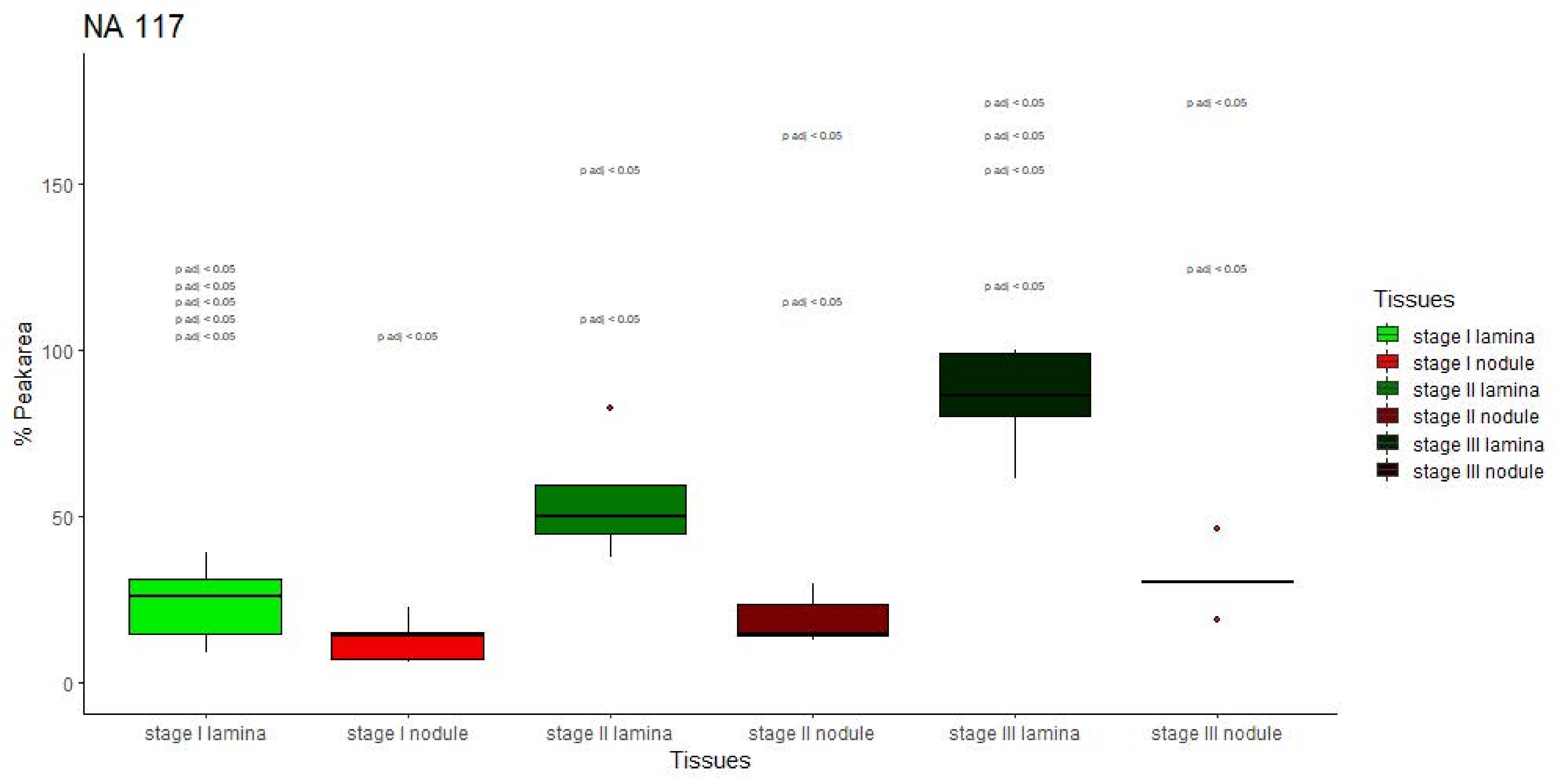

NA 117
% Peakarea
150
100
50
0
p adj < 0.05
stage I lamina
stage I nodule
stage II lamina
stage II nodule
stage III lamina
stage III nodule
Tissues
Tissues
stage I lamina
stage I nodule
stage II lamina
stage II nodule
stage III lamina
stage III nodule

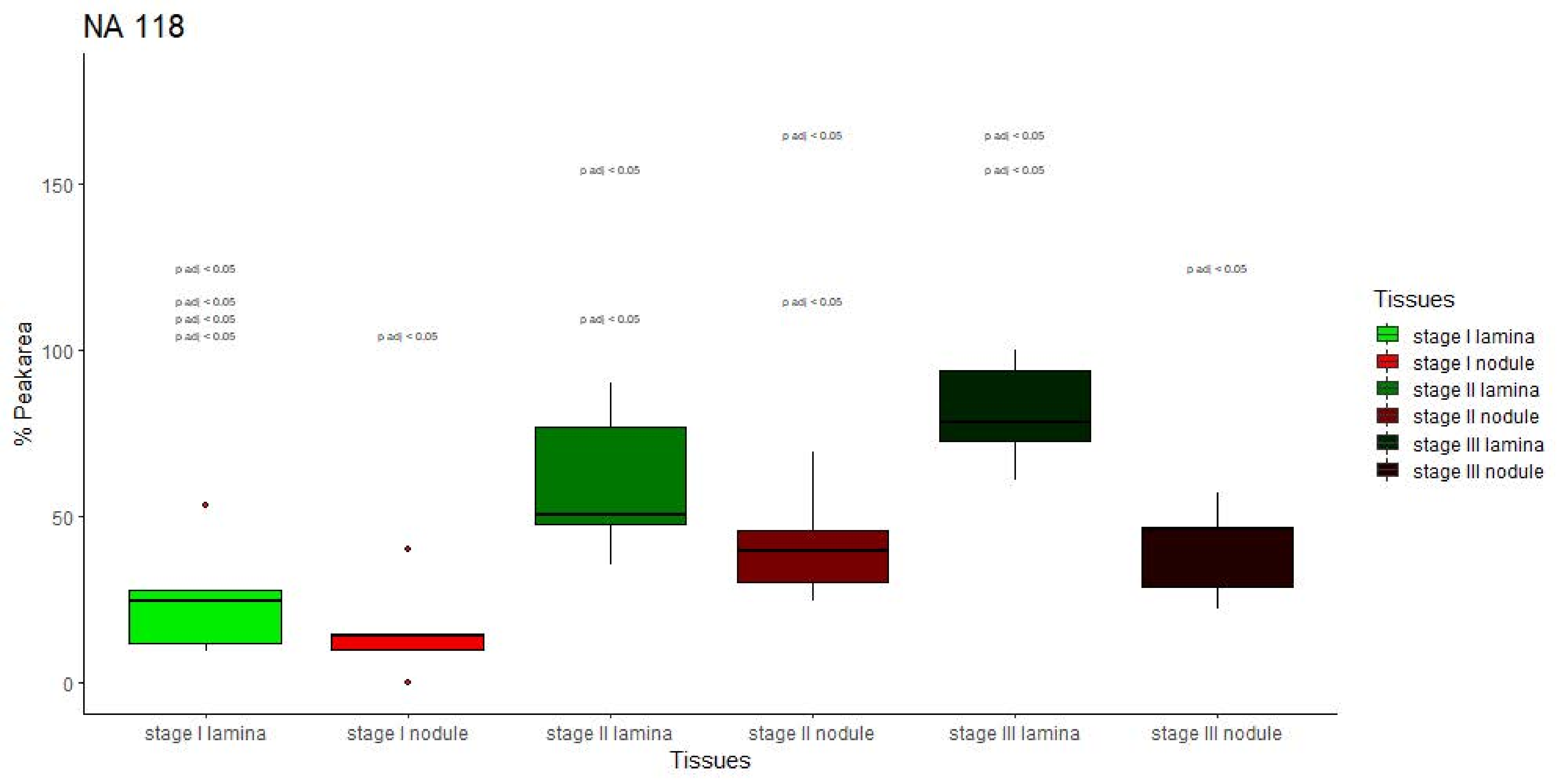
NA 118
% Peakarea
150
100
50
0
p adj < 0.05
stage I lamina
stage I nodule
stage II lamina
stage II nodule
stage III lamina
stage III nodule
Tissues

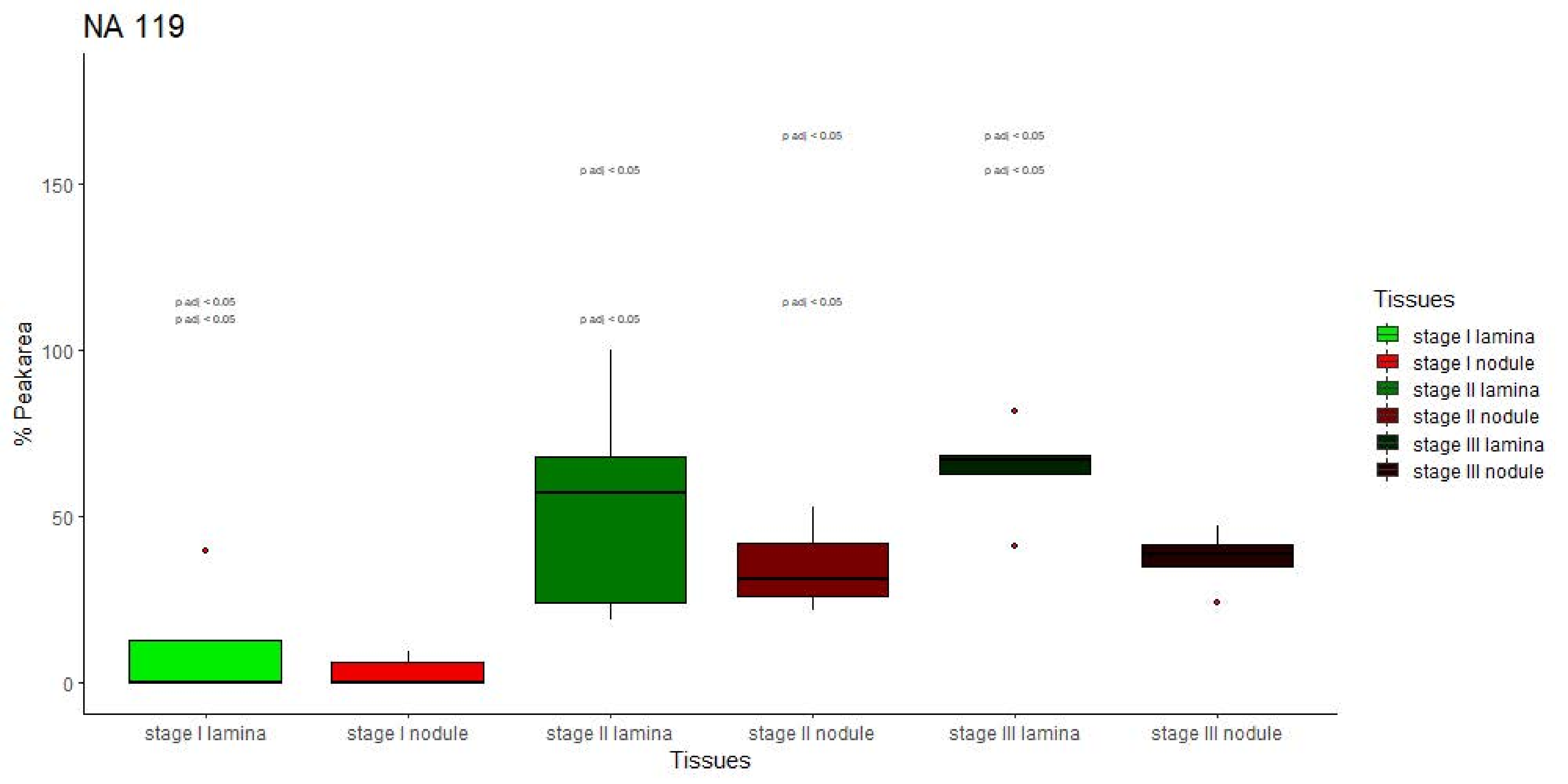

NA 119
% Peakarea
150
100
50
0
p adj < 0.05
p adj < 0.05
p adj < 0.05
p adj < 0.05
p adj < 0.05
p adj < 0.05
p adj < 0.05
p adj < 0.05
stage I lamina
stage I nodule
stage II lamina
stage II nodule
stage III lamina
stage III nodule
Tissues
Tissues
stage I lamina
stage I nodule
stage II lamina
stage II nodule
stage III lamina
stage III nodule

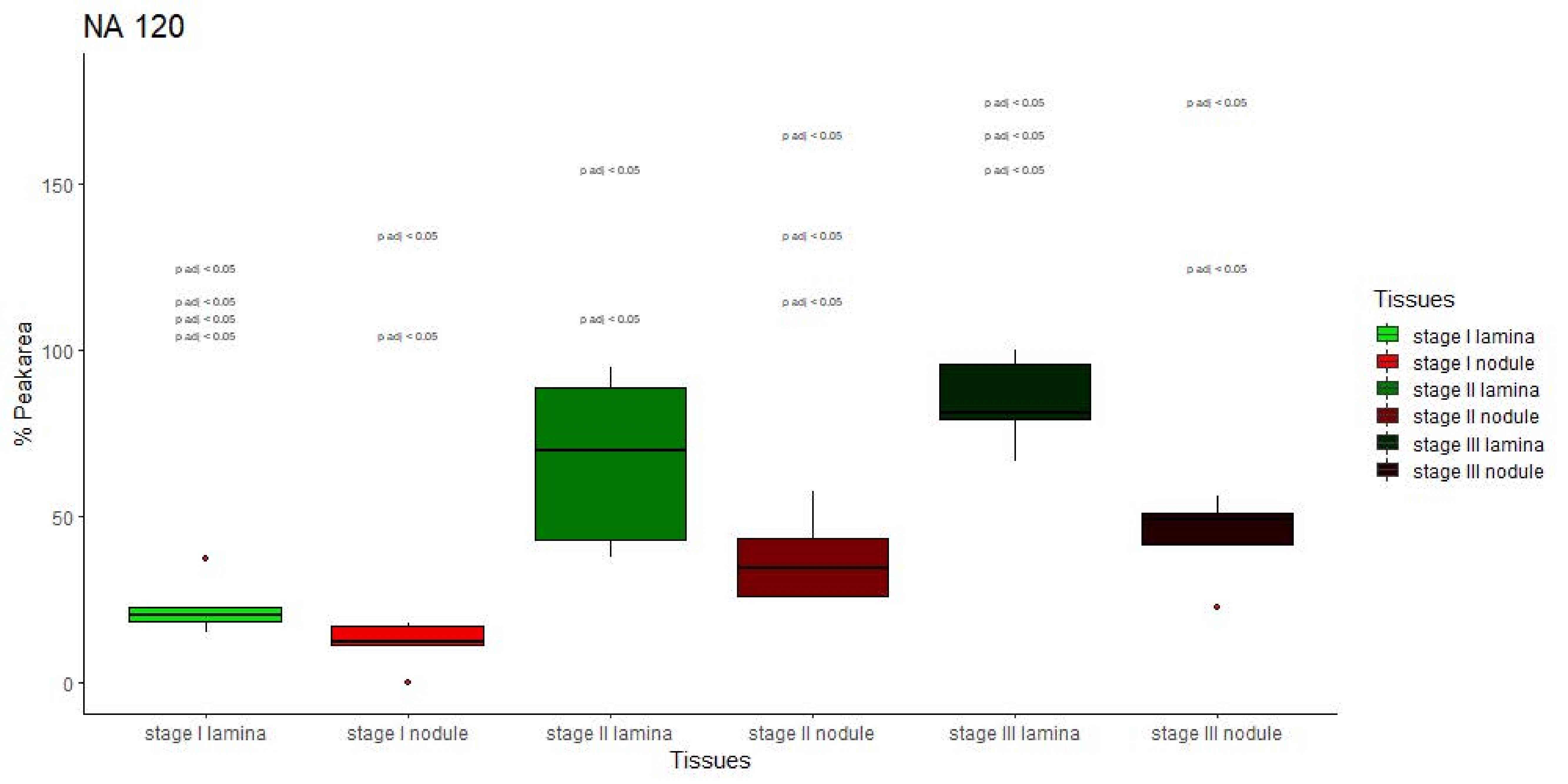
NA 120
% Peakarea
150
100
50
0
p adj < 0.05
Tissues
stage I lamina
stage I nodule
stage II lamina
stage II nodule
stage III lamina
stage III nodule

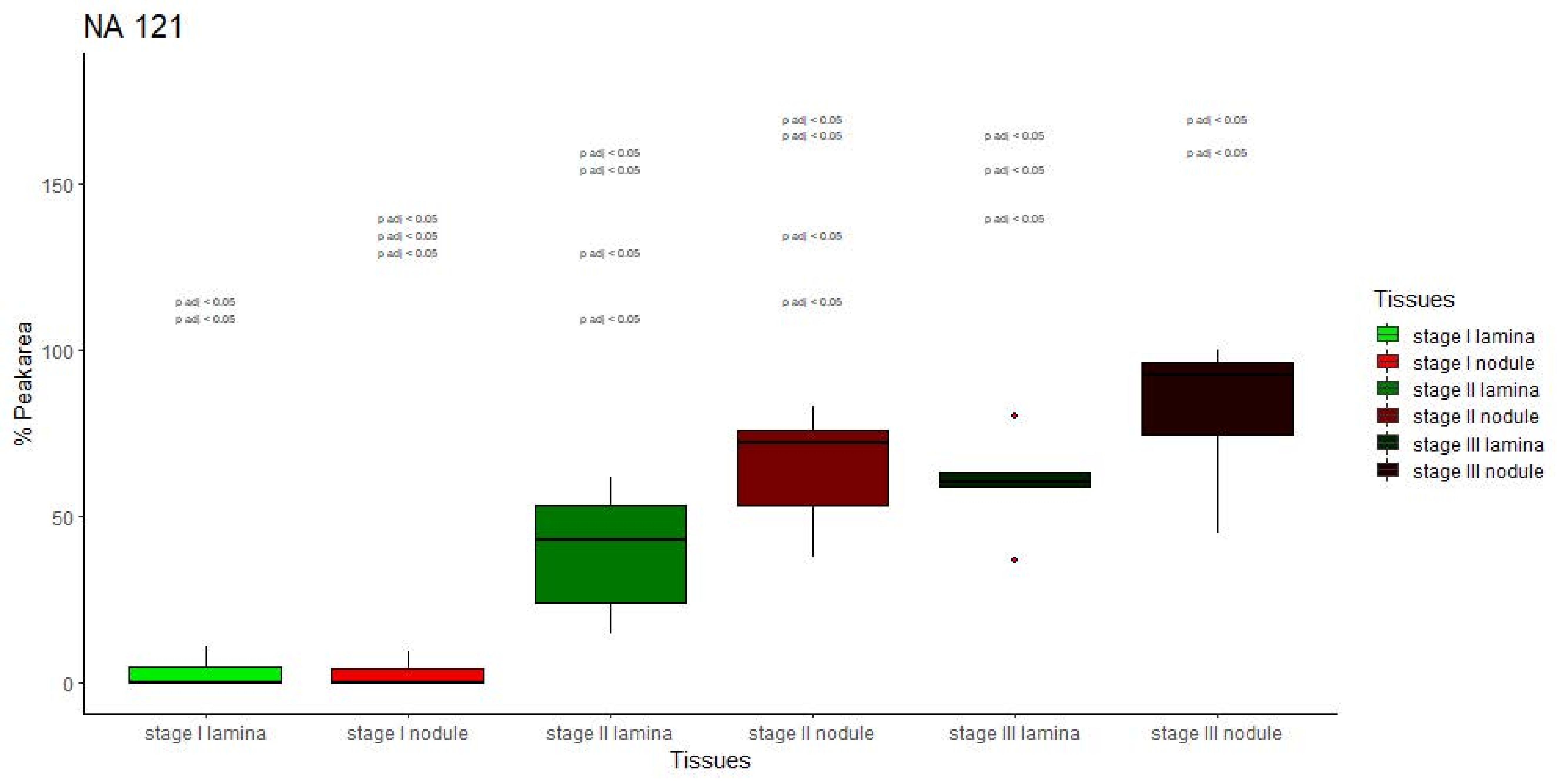
NA 121
% Peakarea
150
100
50
0
p adj < 0.05
Tissues
stage I lamina
stage I nodule
stage II lamina
stage II nodule
stage III lamina
stage III nodule

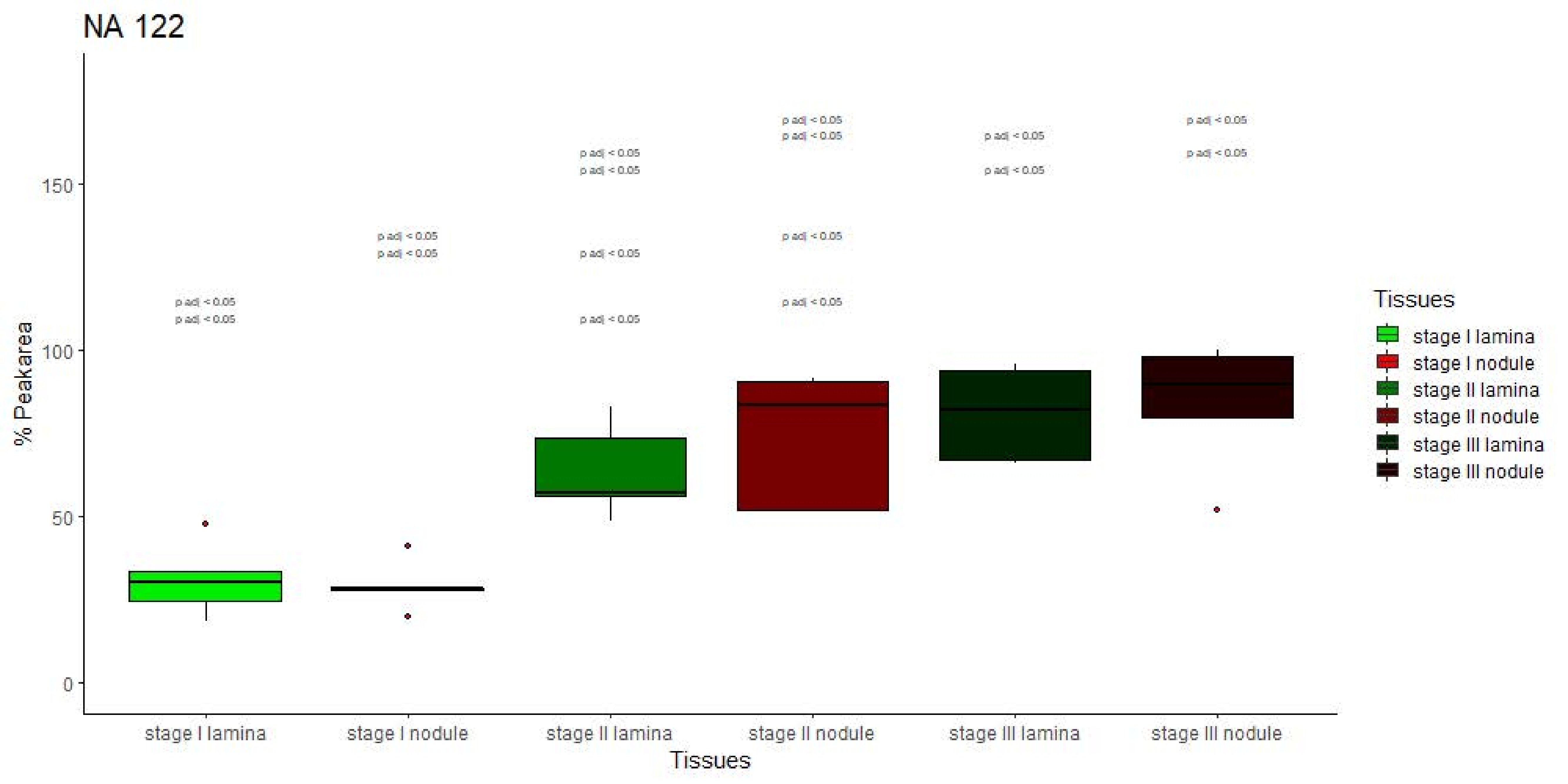

NA 122
p adj < 0.05
p adj < 0.05
% Peakarea
150
100
50
0
Tissues
stage I lamina
stage I nodule
stage II lamina
stage II nodule
stage III lamina
stage III nodule

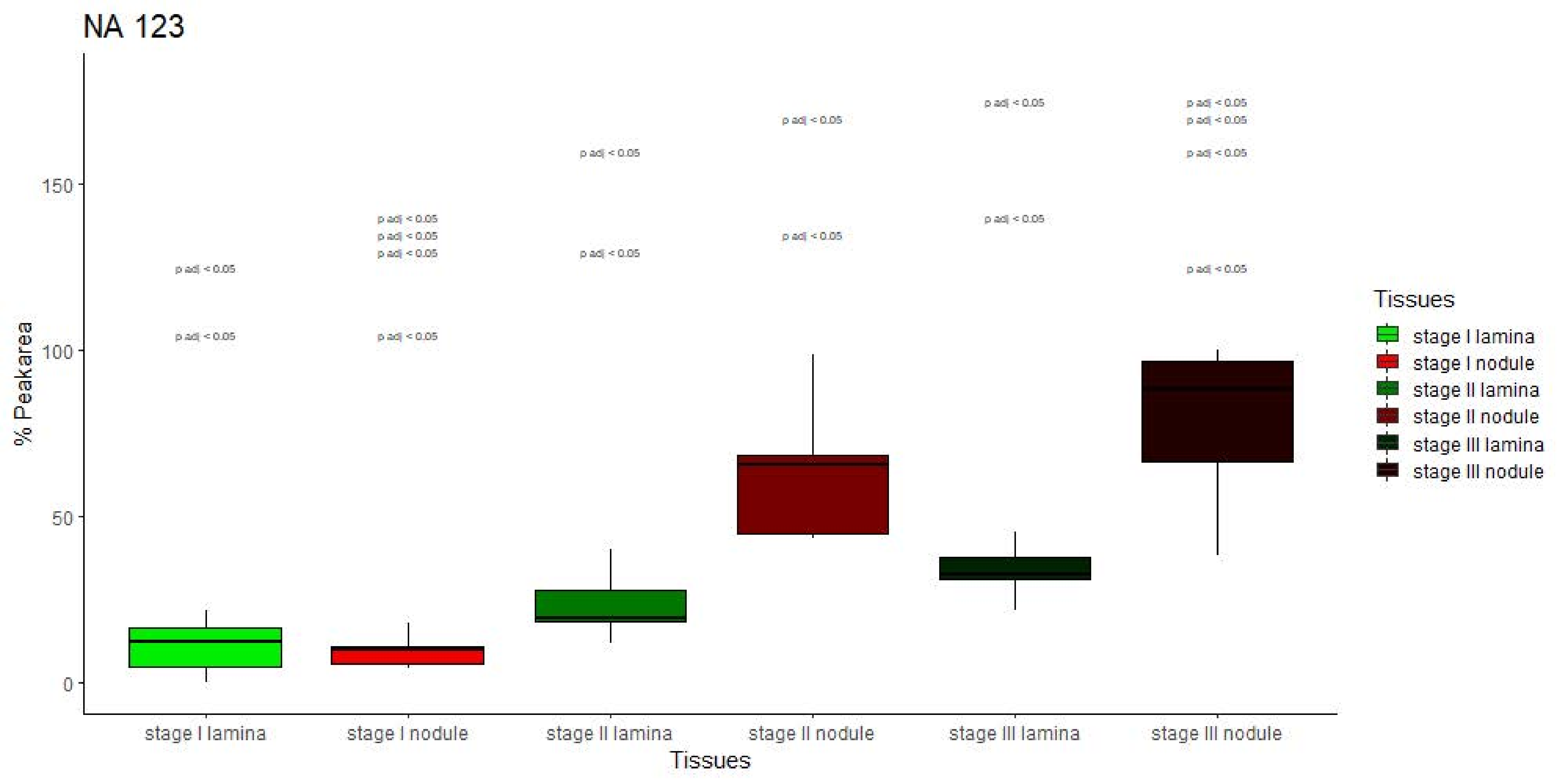
NA 123
% Peakarea
0
50
100
150
p adj < 0.05
Tissues
stage I lamina
stage I nodule
stage II lamina
stage II nodule
stage III lamina
stage III nodule

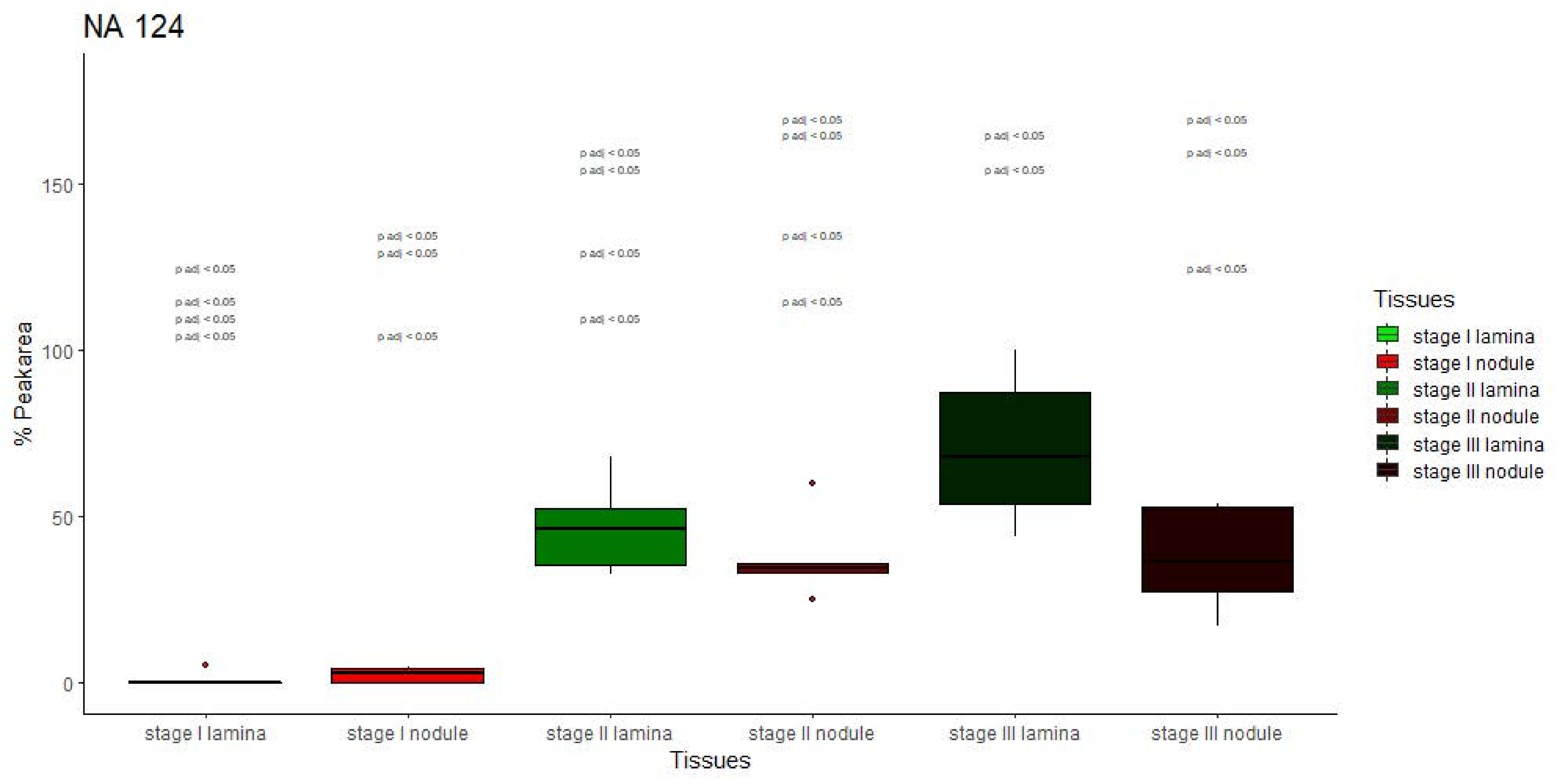

NA 124
% Peakarea
0
50
100
150
p adj < 0.05
Tissues
stage I lamina
stage I nodule
stage II lamina
stage II nodule
stage III lamina
stage III nodule

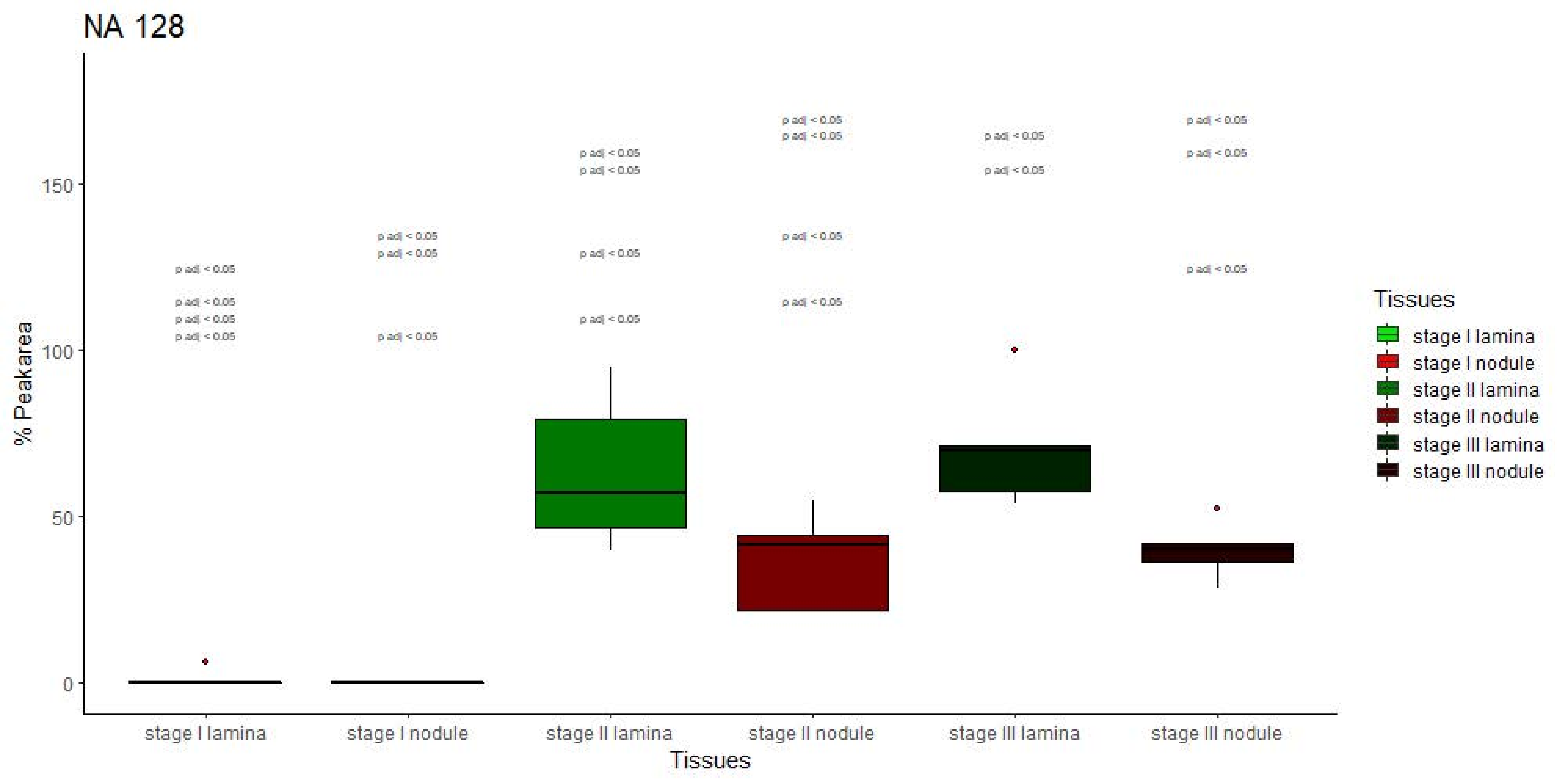

NA 128
% Peakarea
150
100
50
0
p adj < 0.05
stage I lamina
stage I nodule
stage II lamina
stage II nodule
stage III lamina
stage III nodule
Tissues
Tissues
stage I lamina
stage I nodule
stage II lamina
stage II nodule
stage III lamina
stage III nodule

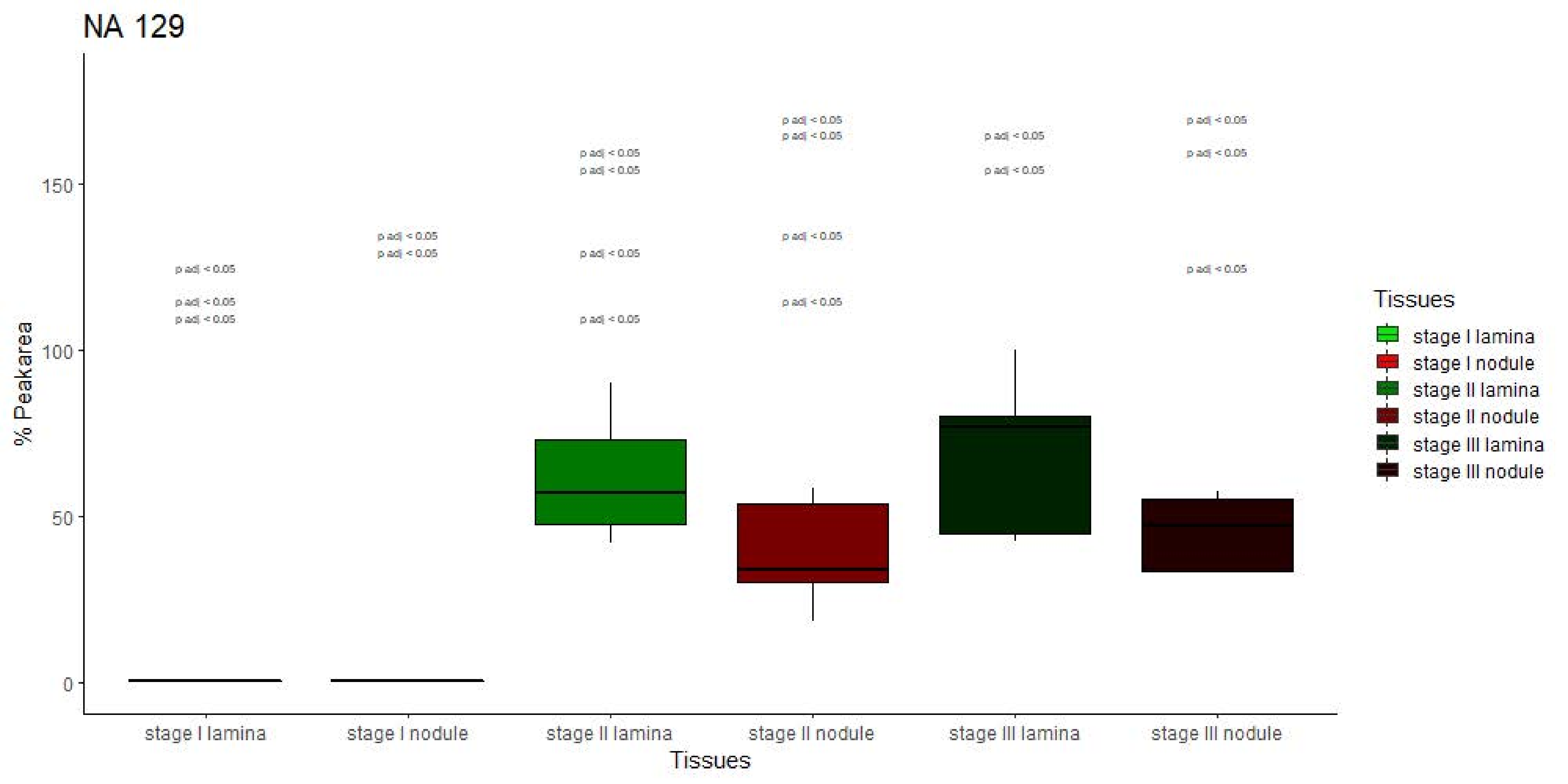

NA 129
% Peakarea
150
100
50
0
p adj < 0.05
stage I lamina
stage I nodule
stage II lamina
stage II nodule
stage III lamina
stage III nodule
Tissues
Tissues
stage I lamina
stage I nodule
stage II lamina
stage II nodule
stage III lamina
stage III nodule

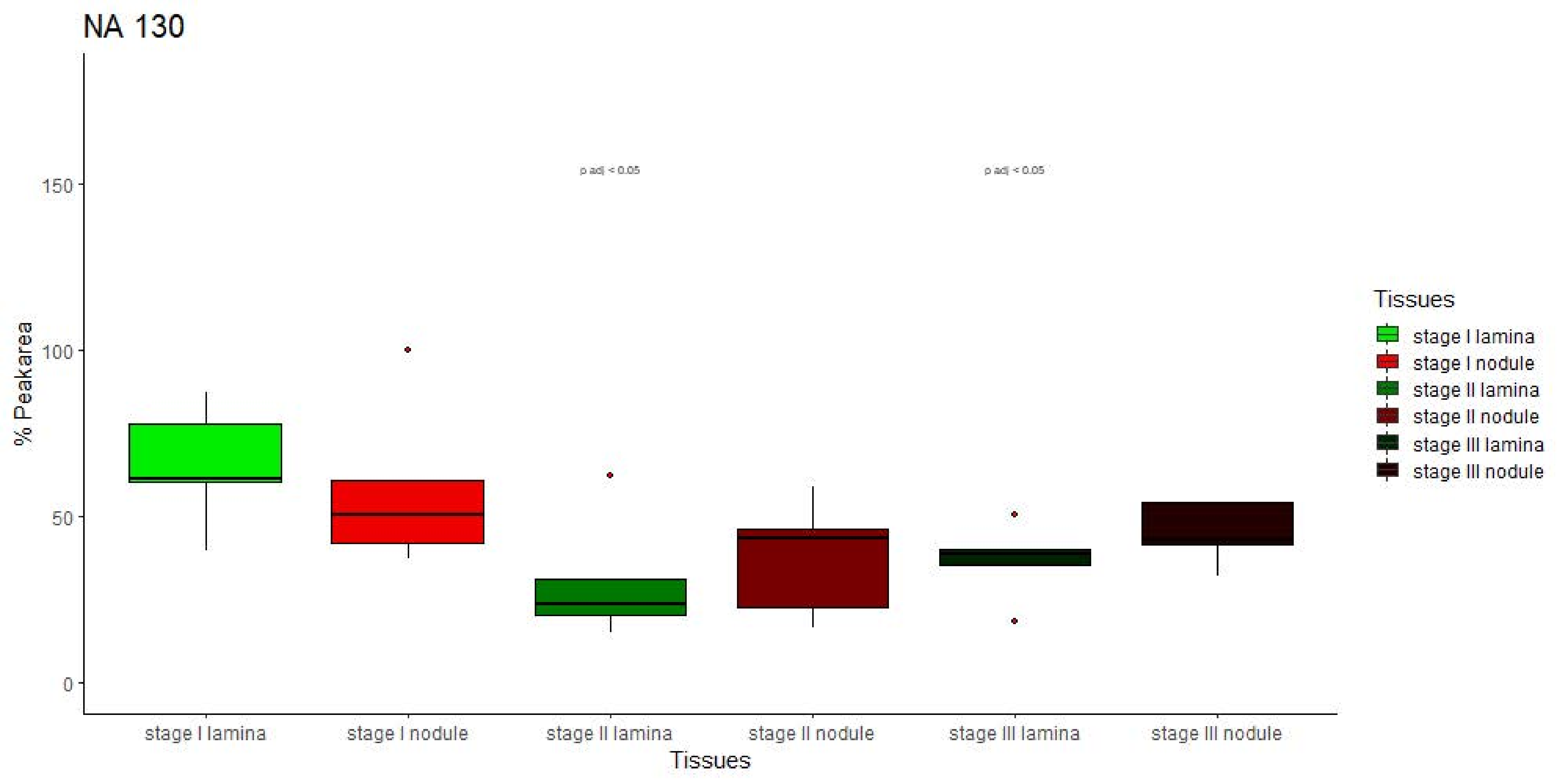
NA 130
p adj < 0.05
p adj < 0.05
150
100
50
0
% Peakarea
stage I lamina
stage I nodule
stage II lamina
stage II nodule
stage III lamina
stage III nodule
Tissues
Tissues
stage I lamina
stage I nodule
stage II lamina
stage II nodule
stage III lamina
stage III nodule

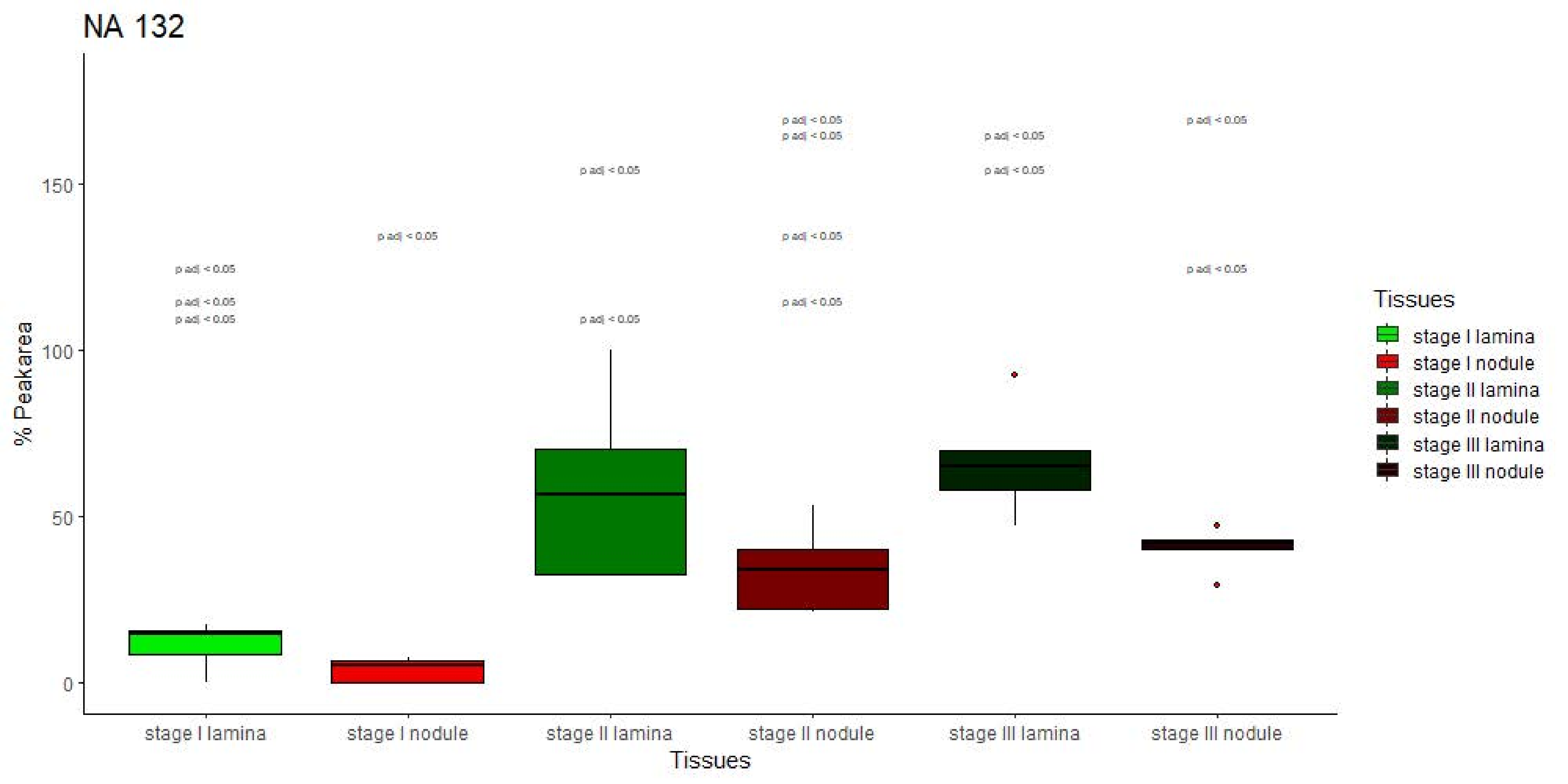
NA 132
% Peakarea
150
100
50
0
p adj < 0.05
Tissues
stage I lamina
stage I nodule
stage II lamina
stage II nodule
stage III lamina
stage III nodule

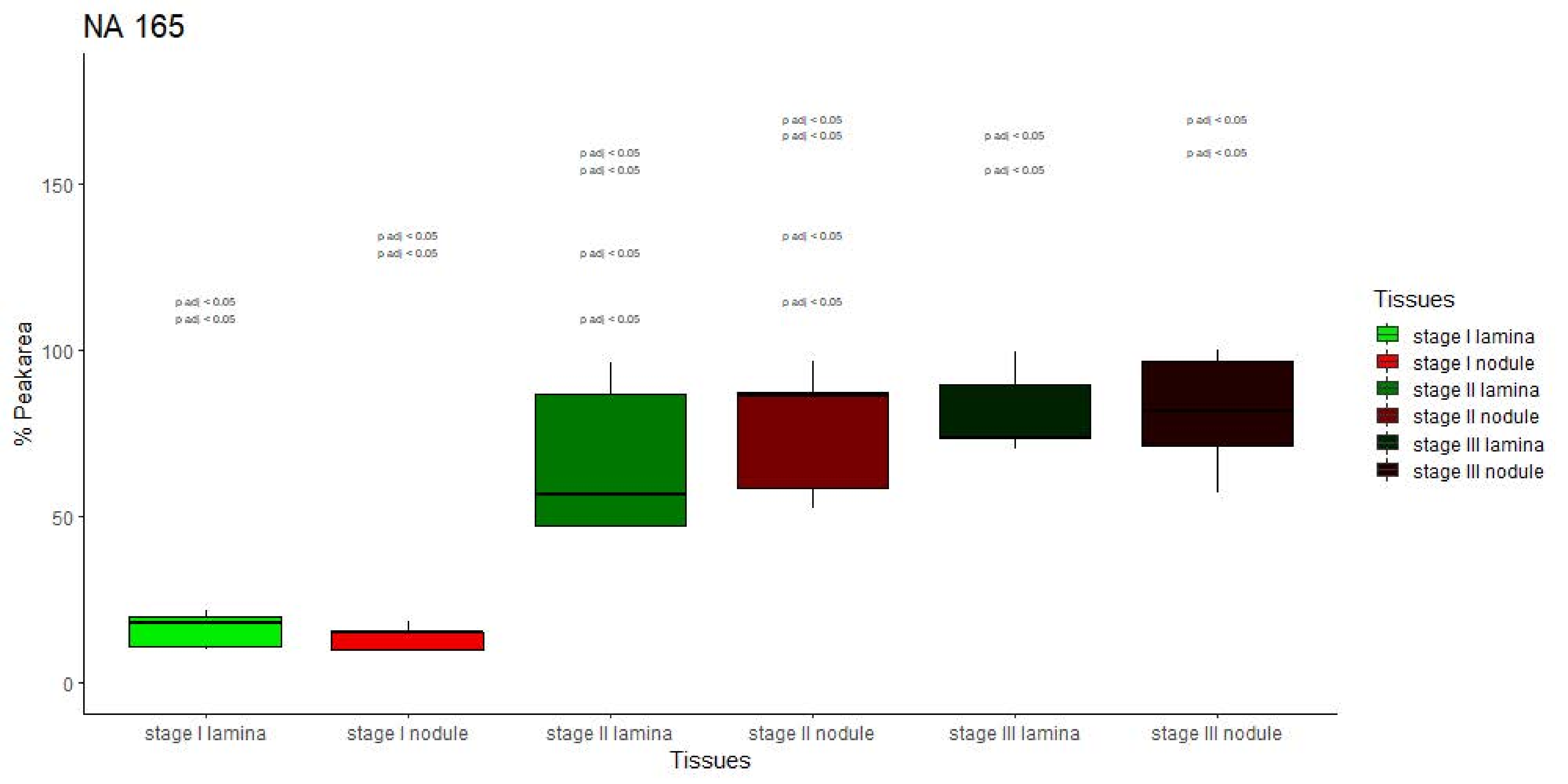

NA 165
% Peakarea
0
50
100
150
p adj < 0.05
Tissues
stage I lamina
stage I nodule
stage II lamina
stage II nodule
stage III lamina
stage III nodule

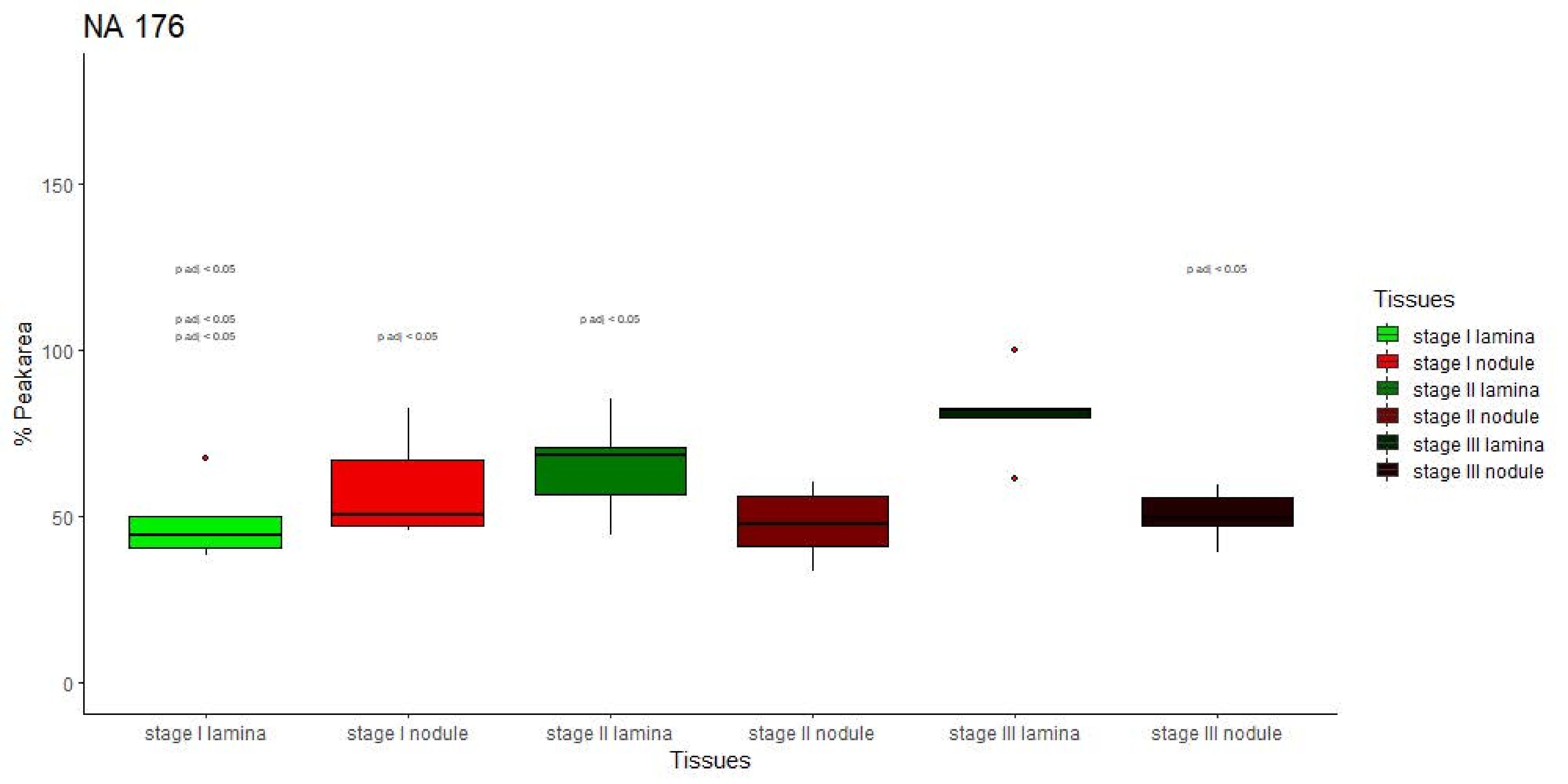

NA 176
% Peakarea
150
100
50
0
p adj < 0.05
p adj < 0.05
p adj < 0.05
p adj < 0.05
p adj < 0.05
p adj < 0.05
stage I lamina
stage I nodule
stage II lamina
stage II nodule
stage III lamina
stage III nodule
Tissues
Tissues
stage I lamina
stage I nodule
stage II lamina
stage II nodule
stage III lamina
stage III nodule

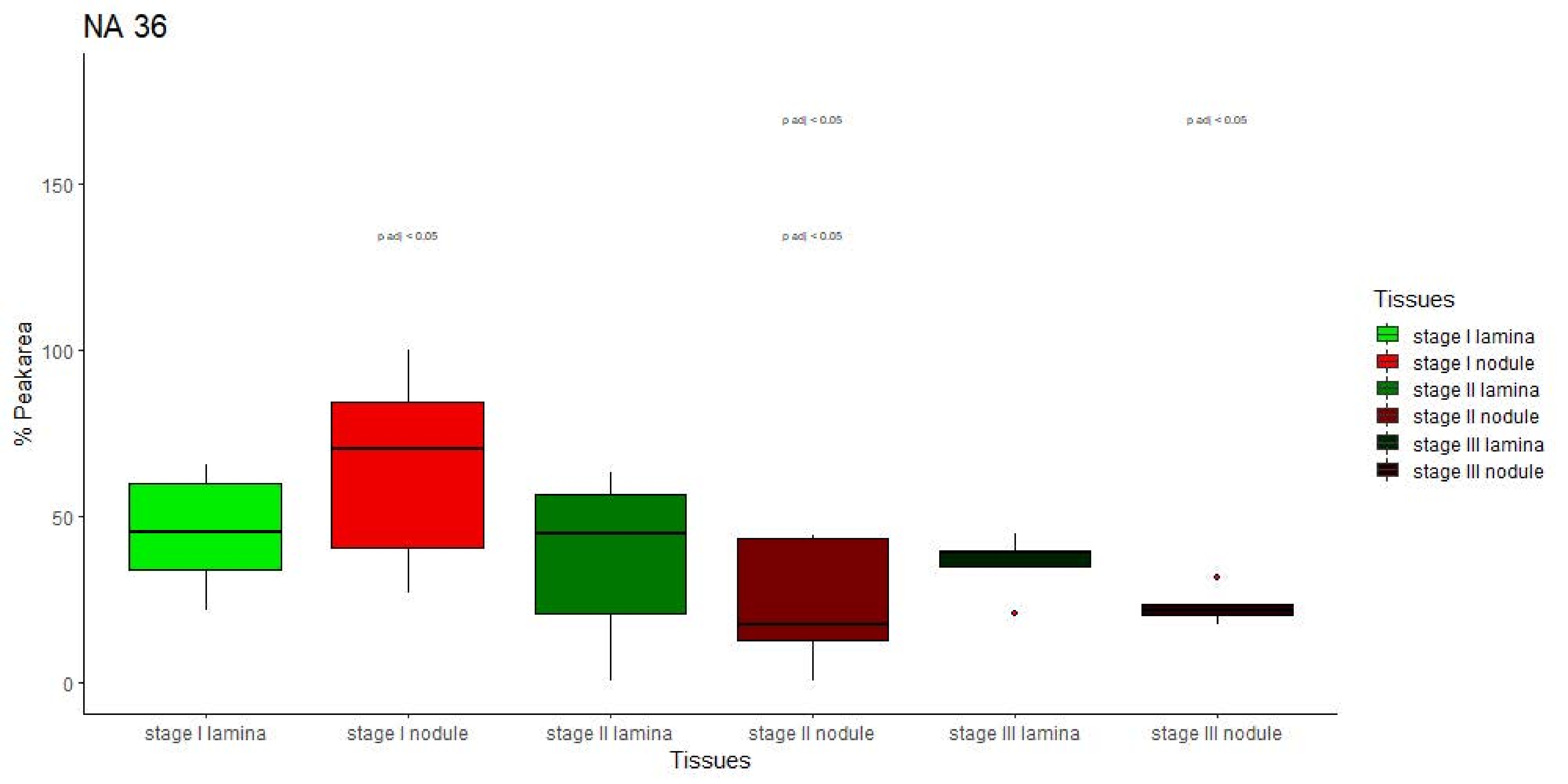
NA 36
p adj < 0.05
p adj < 0.05
p adj < 0.05
p adj < 0.05
150
100
50
0
% Peakarea
Tissues
stage I lamina
stage I nodule
stage II lamina
stage II nodule
stage III lamina
stage III nodule
Tissues
stage I lamina
stage I nodule
stage II lamina
stage II nodule
stage III lamina
stage III nodule

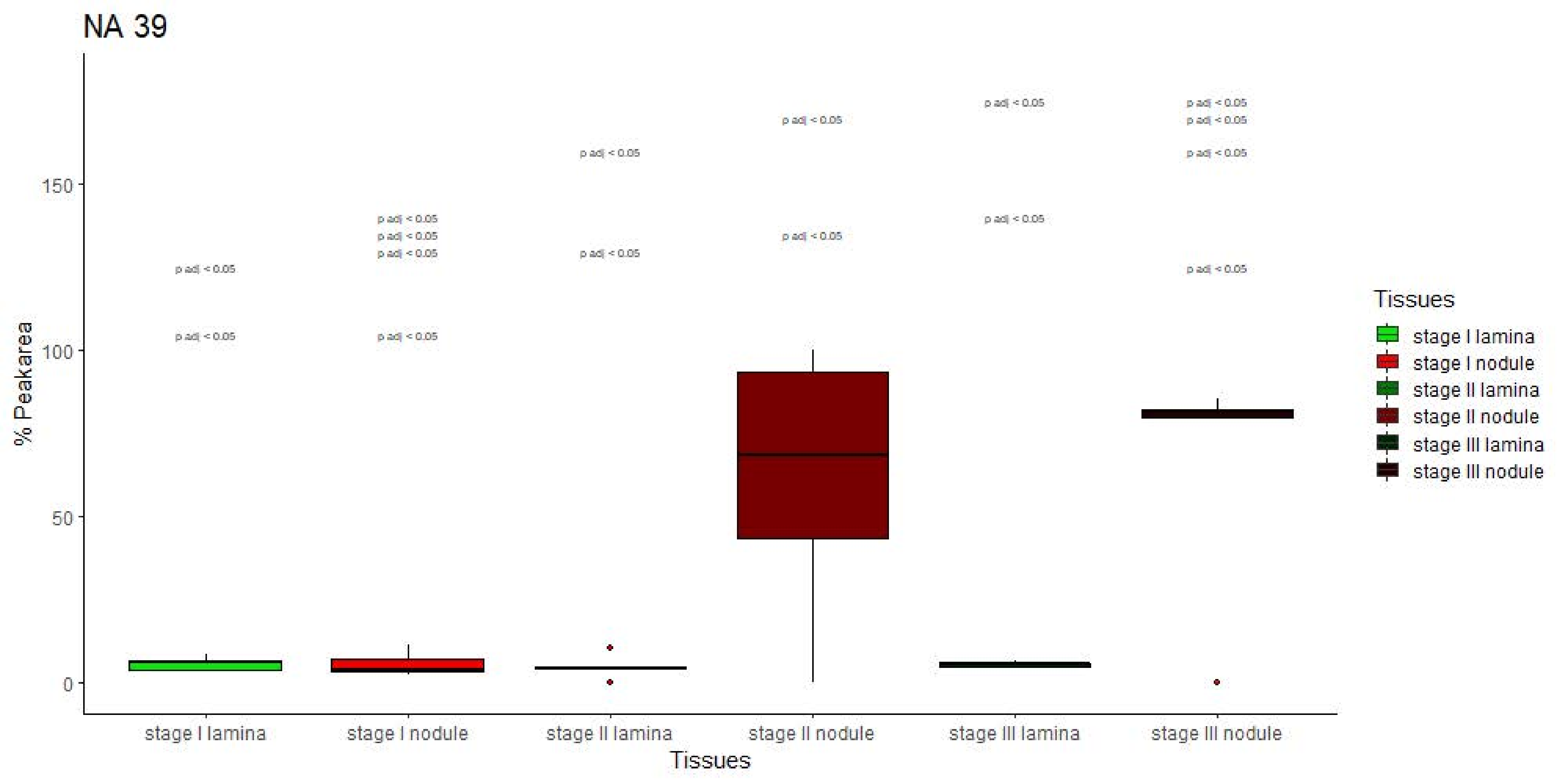

NA 39
% Peakarea
150
100
50
0
p adj < 0.05
stage I lamina
stage I nodule
stage II lamina
stage II nodule
stage III lamina
stage III nodule
Tissues
Tissues
stage I lamina
stage I nodule
stage II lamina
stage II nodule
stage III lamina
stage III nodule

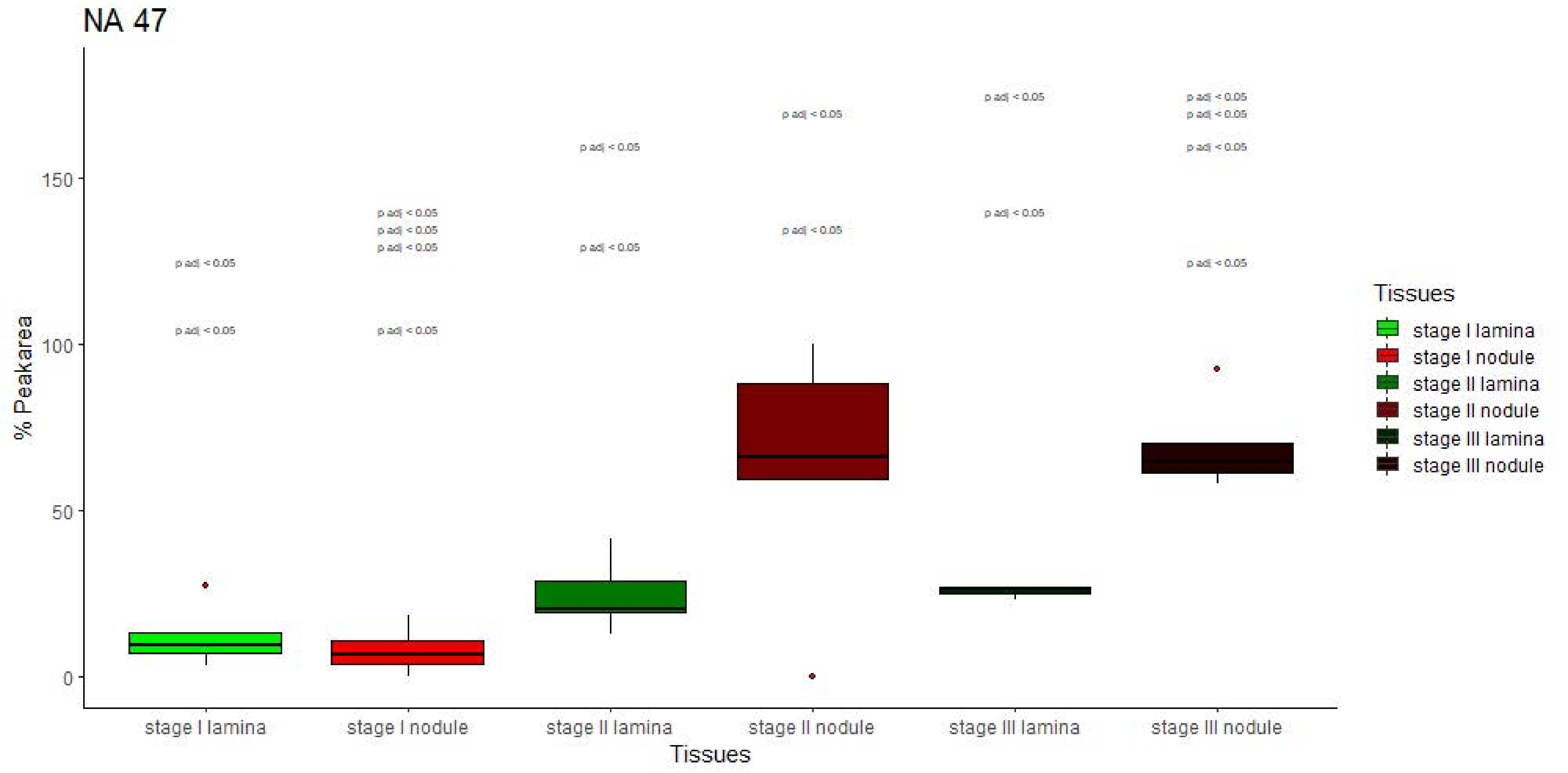
NA 47
% Peakarea
150
100
50
0
p adj < 0.05
stage I lamina
stage I nodule
stage II lamina
stage II nodule
stage III lamina
stage III nodule
Tissues
Tissues
stage I lamina
stage I nodule
stage II lamina
stage II nodule
stage III lamina
stage III nodule

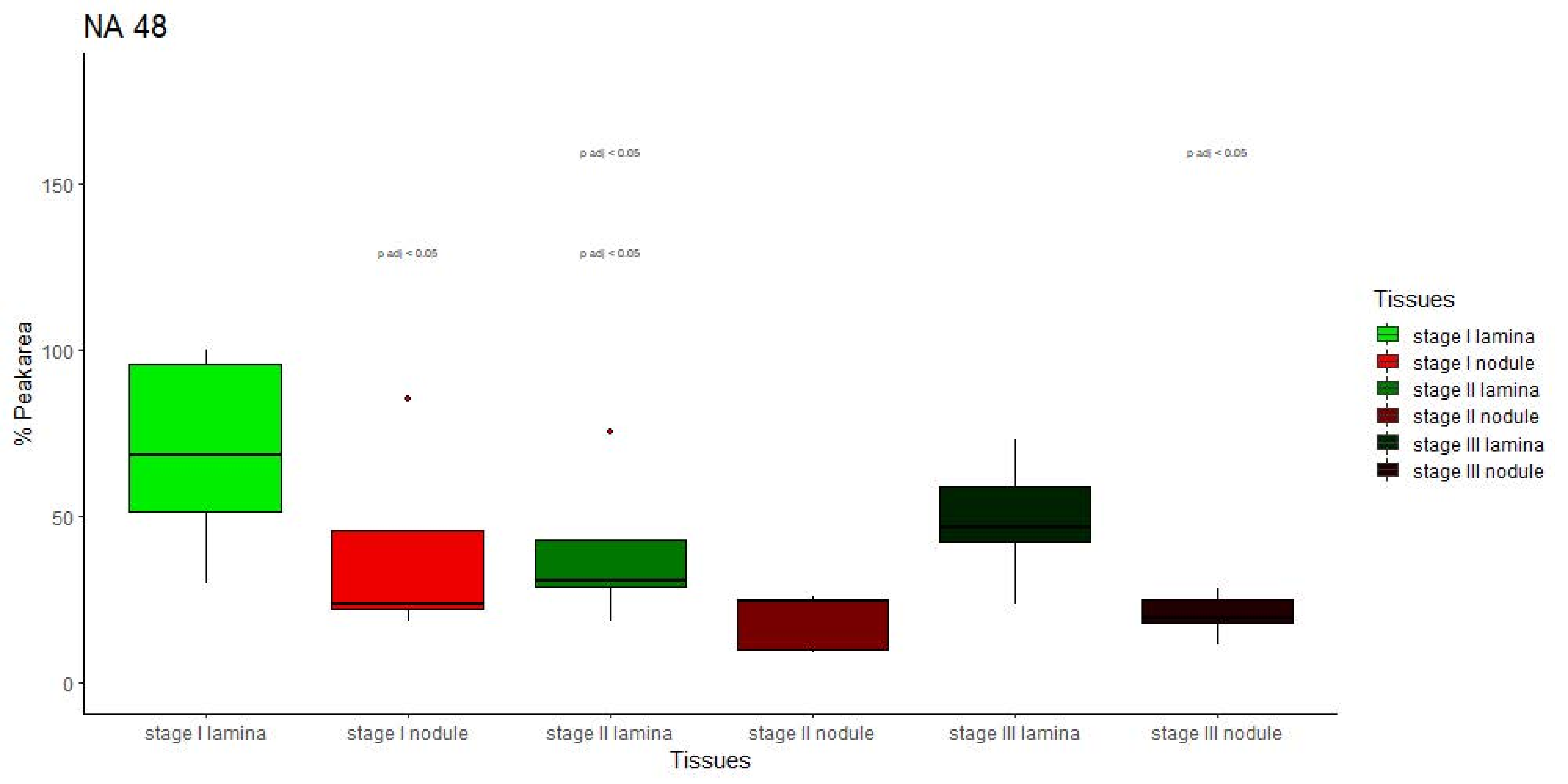
NA 48
% Peakarea
150
100
50
0
p adj < 0.05
p adj < 0.05
p adj < 0.05
p adj < 0.05
stage I lamina
stage I nodule
stage II lamina
stage II nodule
stage III lamina
stage III nodule
Tissues
Tissues
stage I lamina
stage I nodule
stage II lamina
stage II nodule
stage III lamina
stage III nodule

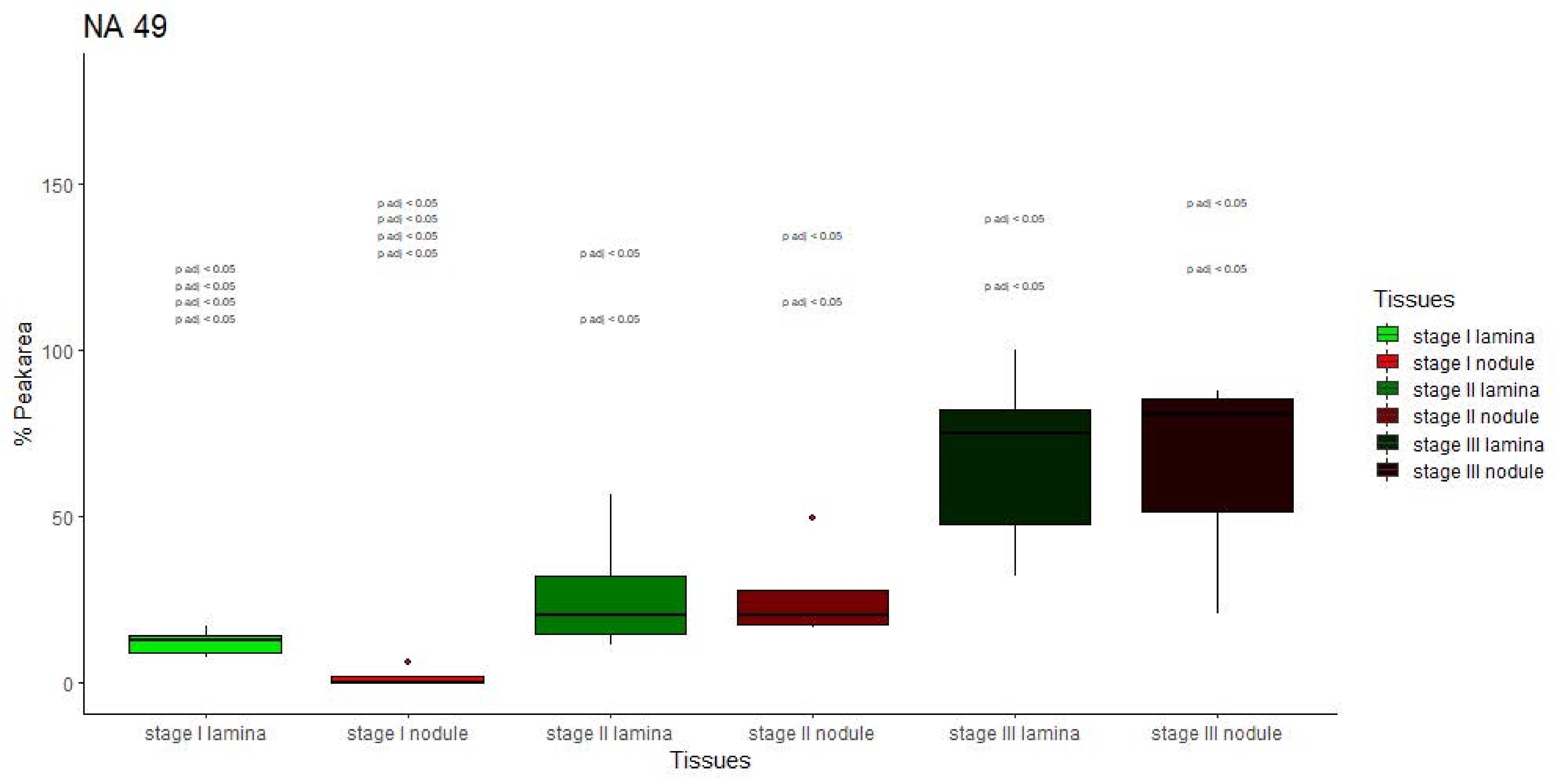

NA 49
% Peakarea
150
100
50
0
p adj < 0.05
stage I lamina
stage I nodule
stage II lamina
stage II nodule
stage III lamina
stage III nodule
Tissues
Tissues
stage I lamina
stage I nodule
stage II lamina
stage II nodule
stage III lamina
stage III nodule

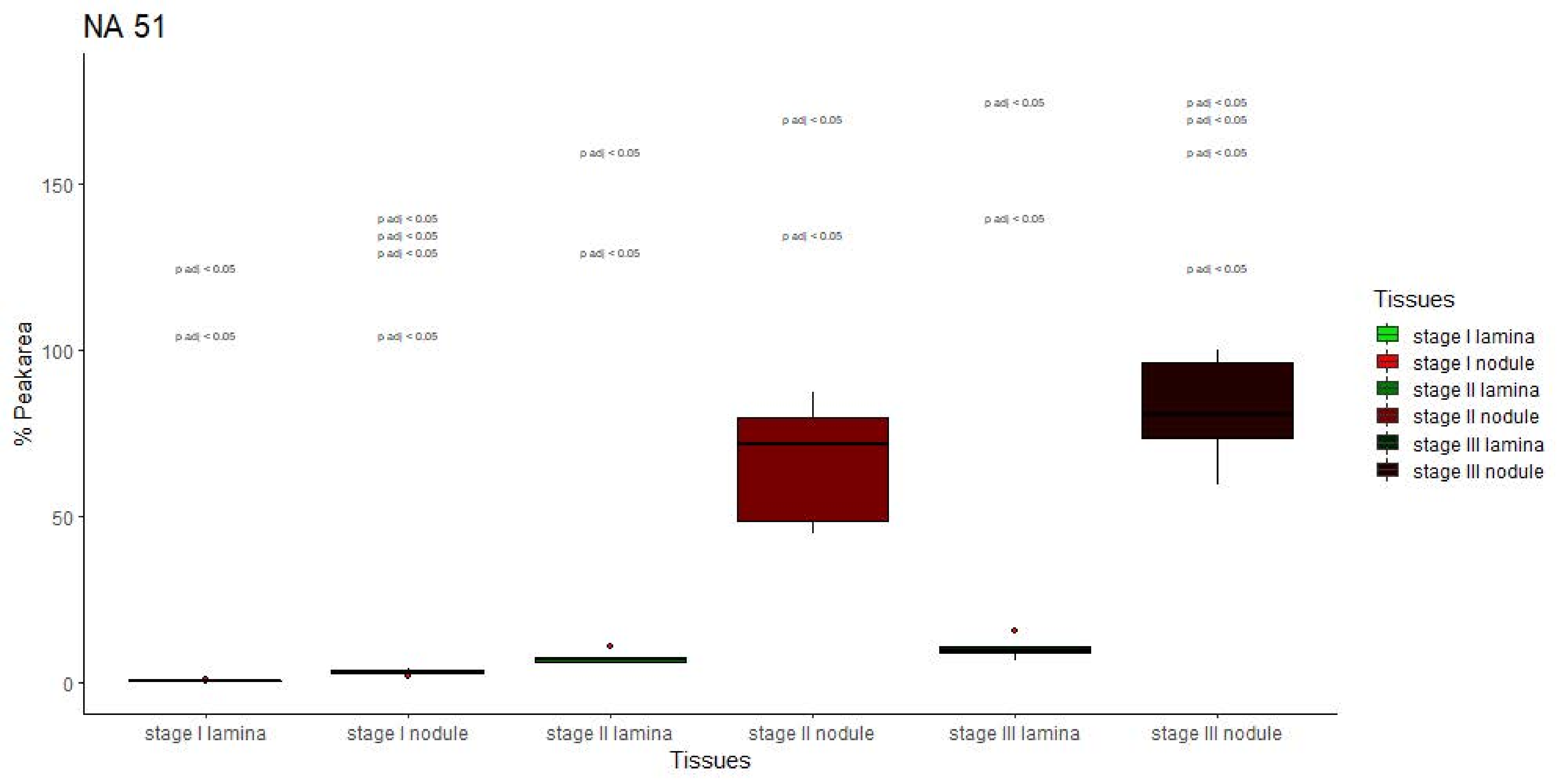

NA 51
p adj < 0.05
150
100
50
0
% Peakarea
stage I lamina
stage I nodule
stage II lamina
stage II nodule
stage III lamina
stage III nodule
Tissues
Tissues
stage I lamina
stage I nodule
stage II lamina
stage II nodule
stage III lamina
stage III nodule

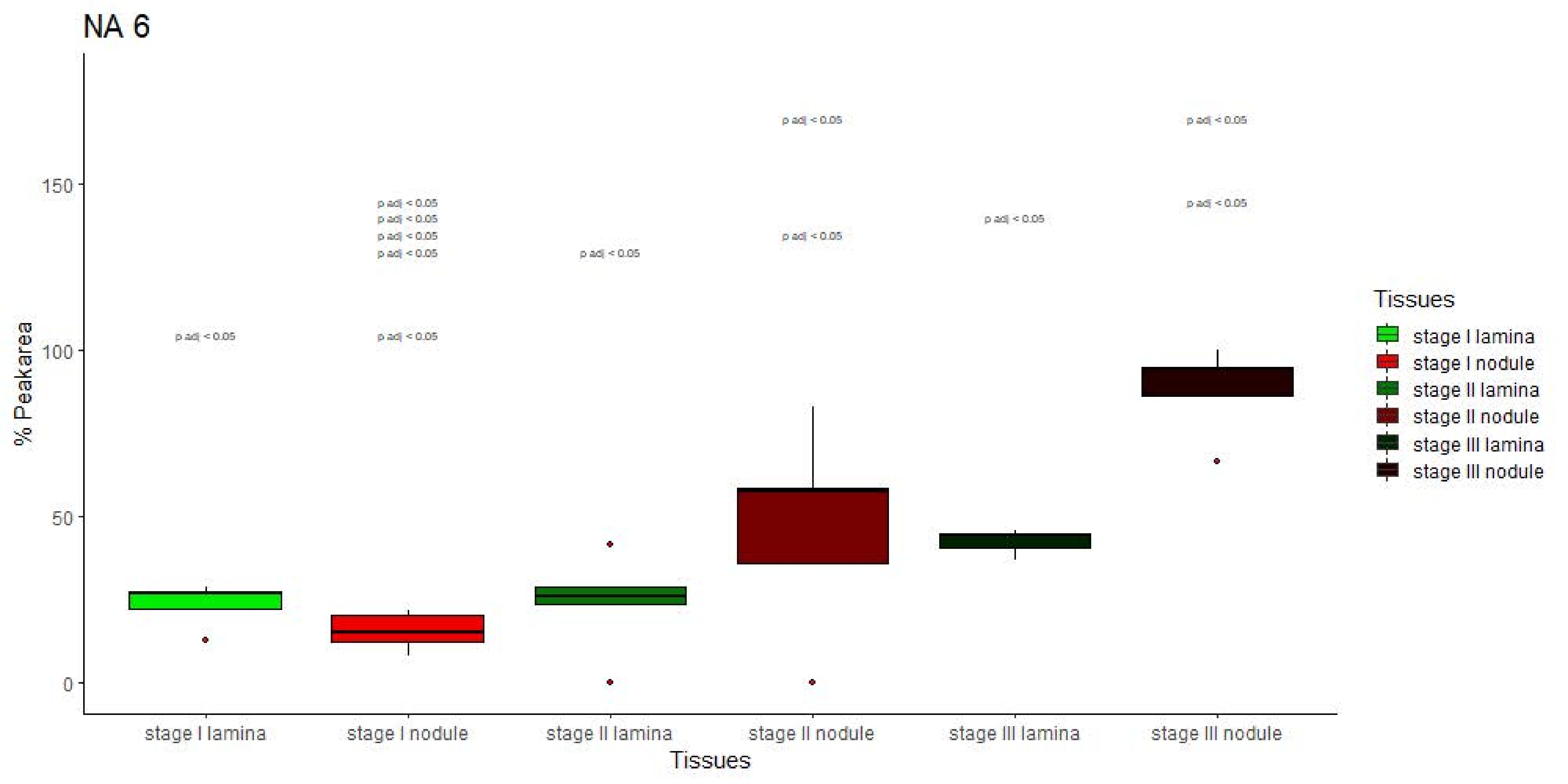
NA 6
% Peakarea
150
100
50
0
p adj < 0.05
Tissues
stage I lamina
stage I nodule
stage II lamina
stage II nodule
stage III lamina
stage III nodule

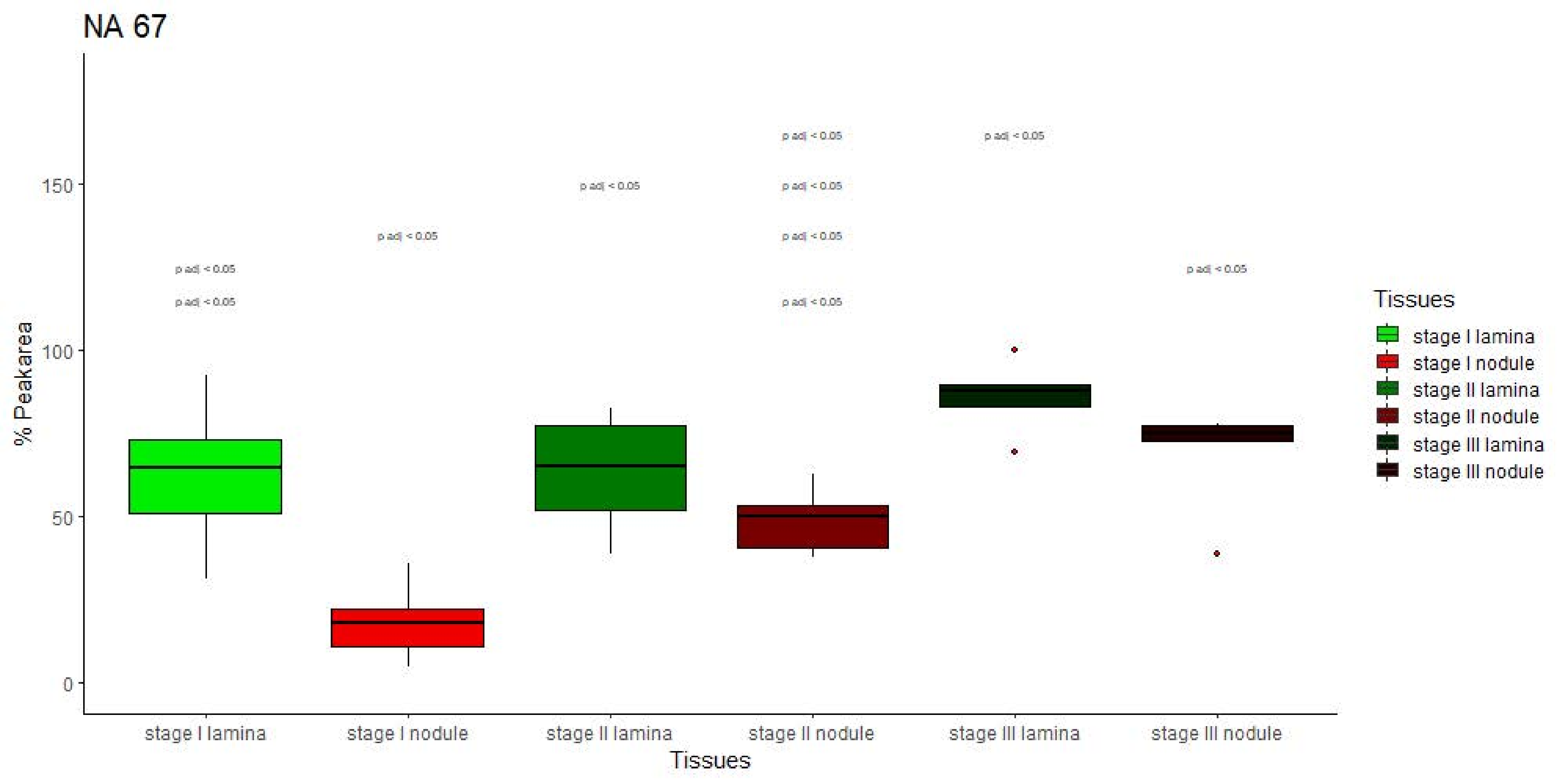
NA 67
% Peakarea
150
100
50
0
p adj < 0.05
stage I lamina
stage I nodule
stage II lamina
stage II nodule
stage III lamina
stage III nodule
Tissues
Tissues
stage I lamina
stage I nodule
stage II lamina
stage II nodule
stage III lamina
stage III nodule

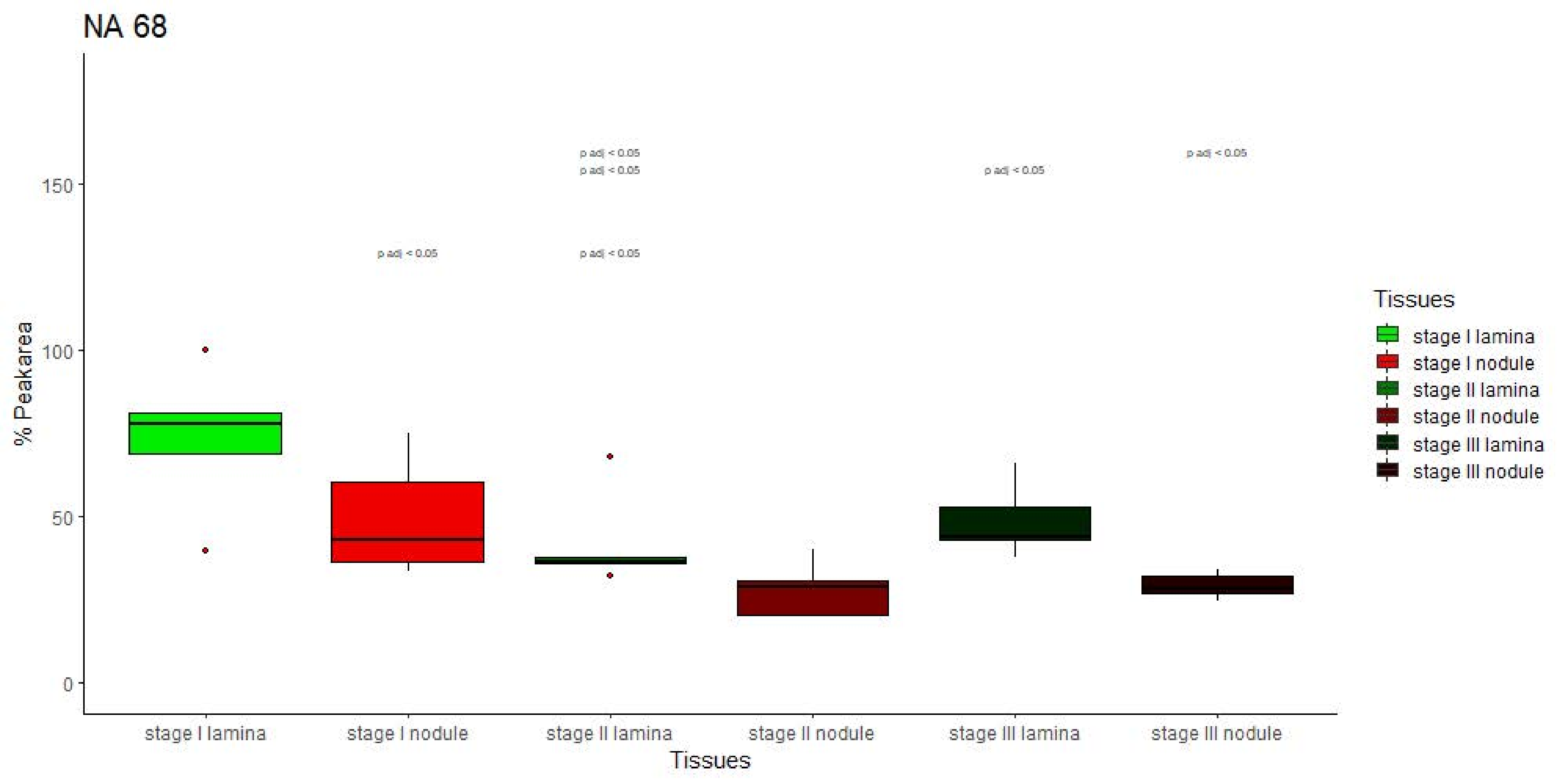
NA 68
p adj < 0.05
p adj < 0.05
p adj < 0.05
p adj < 0.05
p adj < 0.05
p adj < 0.05
150
100
50
0
% Peakarea
stage I lamina
stage I nodule
stage II lamina
stage II nodule
stage III lamina
stage III nodule
Tissues
Tissues
stage I lamina
stage I nodule
stage II lamina
stage II nodule
stage III lamina
stage III nodule

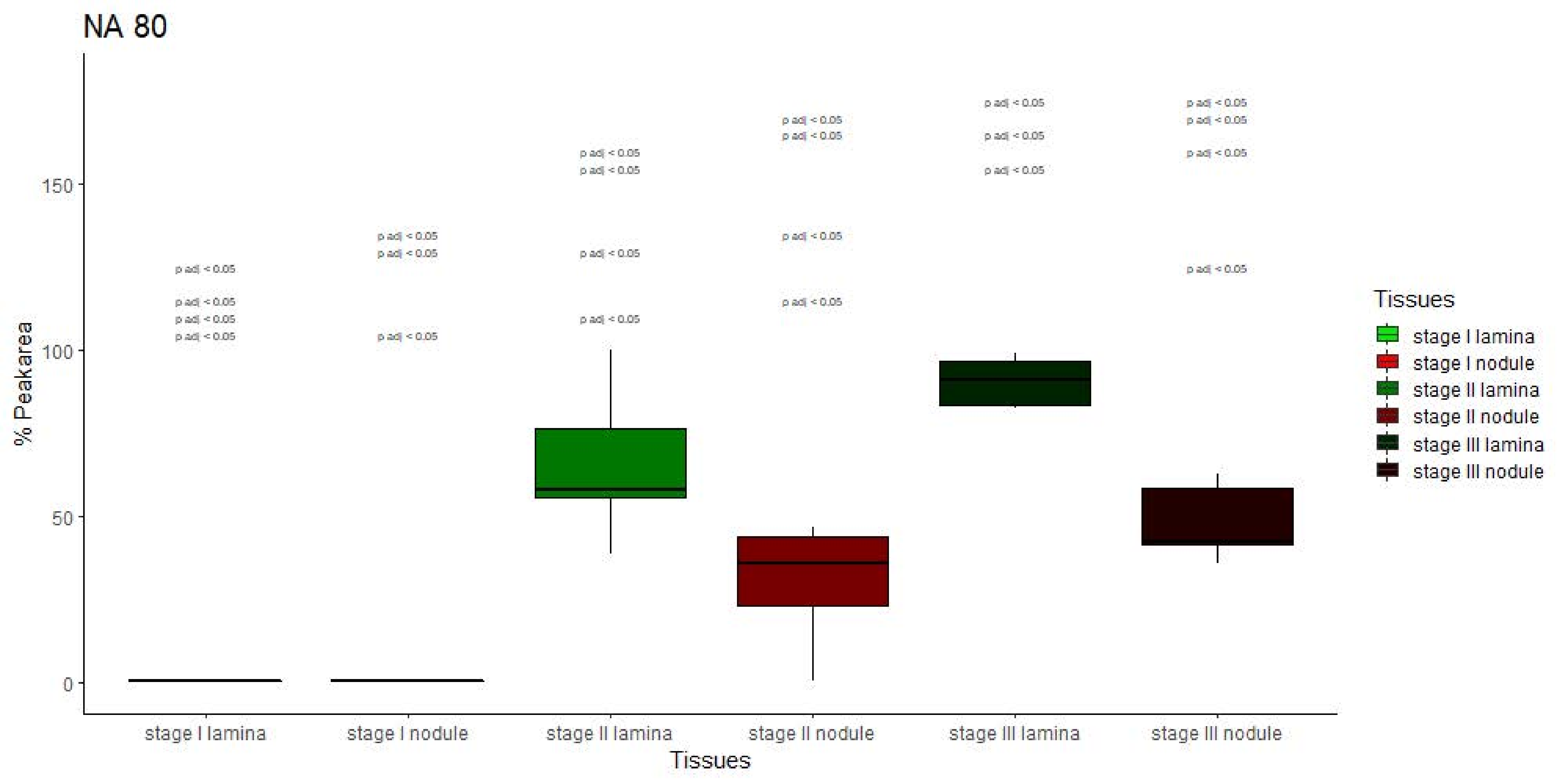

NA 80
% Peakarea
Tissues
stage I lamina
stage I nodule
stage II lamina
stage II nodule
stage III lamina
stage III nodule
p adj < 0.05
0
50
100
150

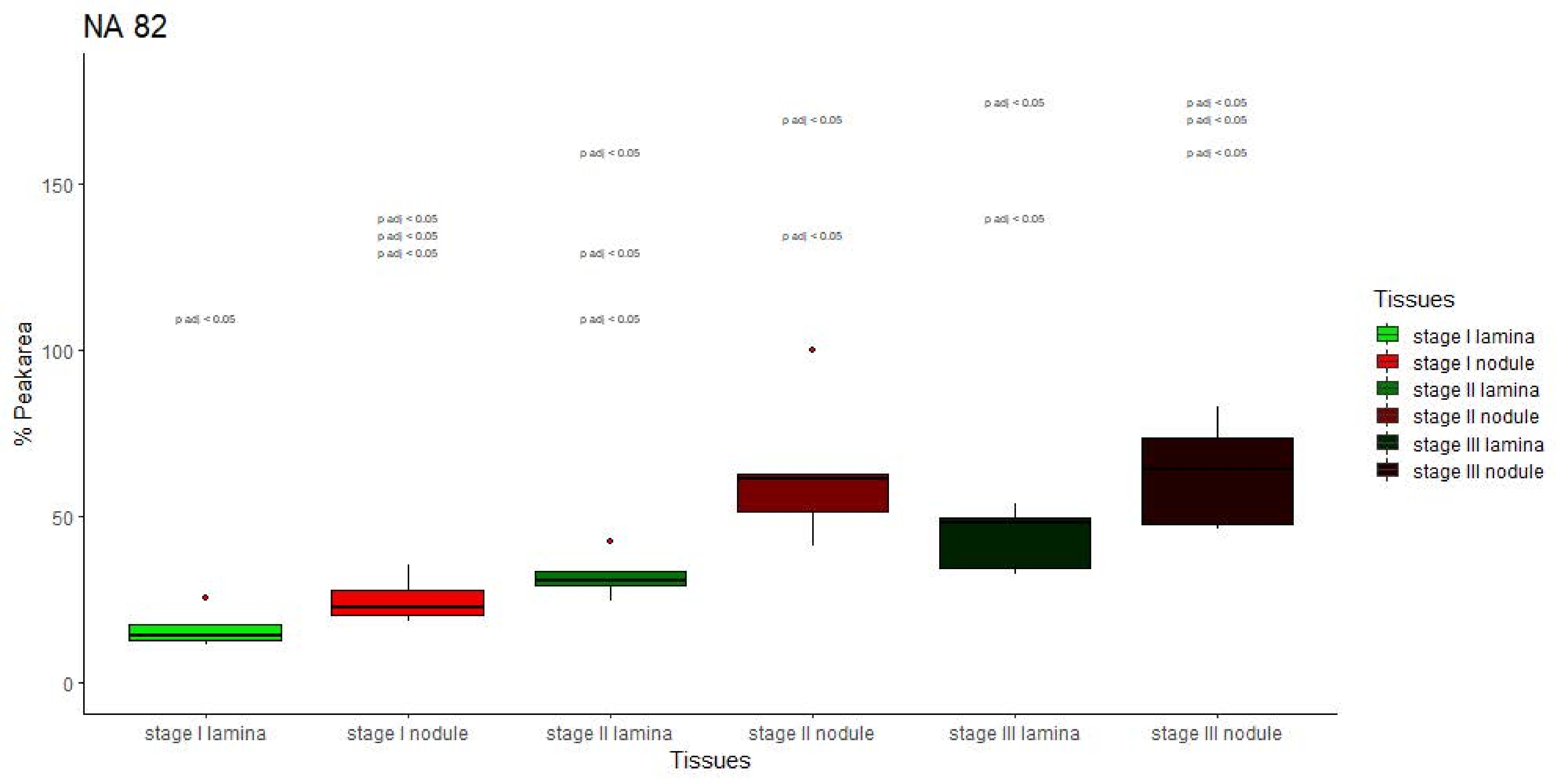
NA 82
% Peakarea
0
50
100
150
p adj < 0.05
Tissues
stage I lamina
stage I nodule
stage II lamina
stage II nodule
stage III lamina
stage III nodule

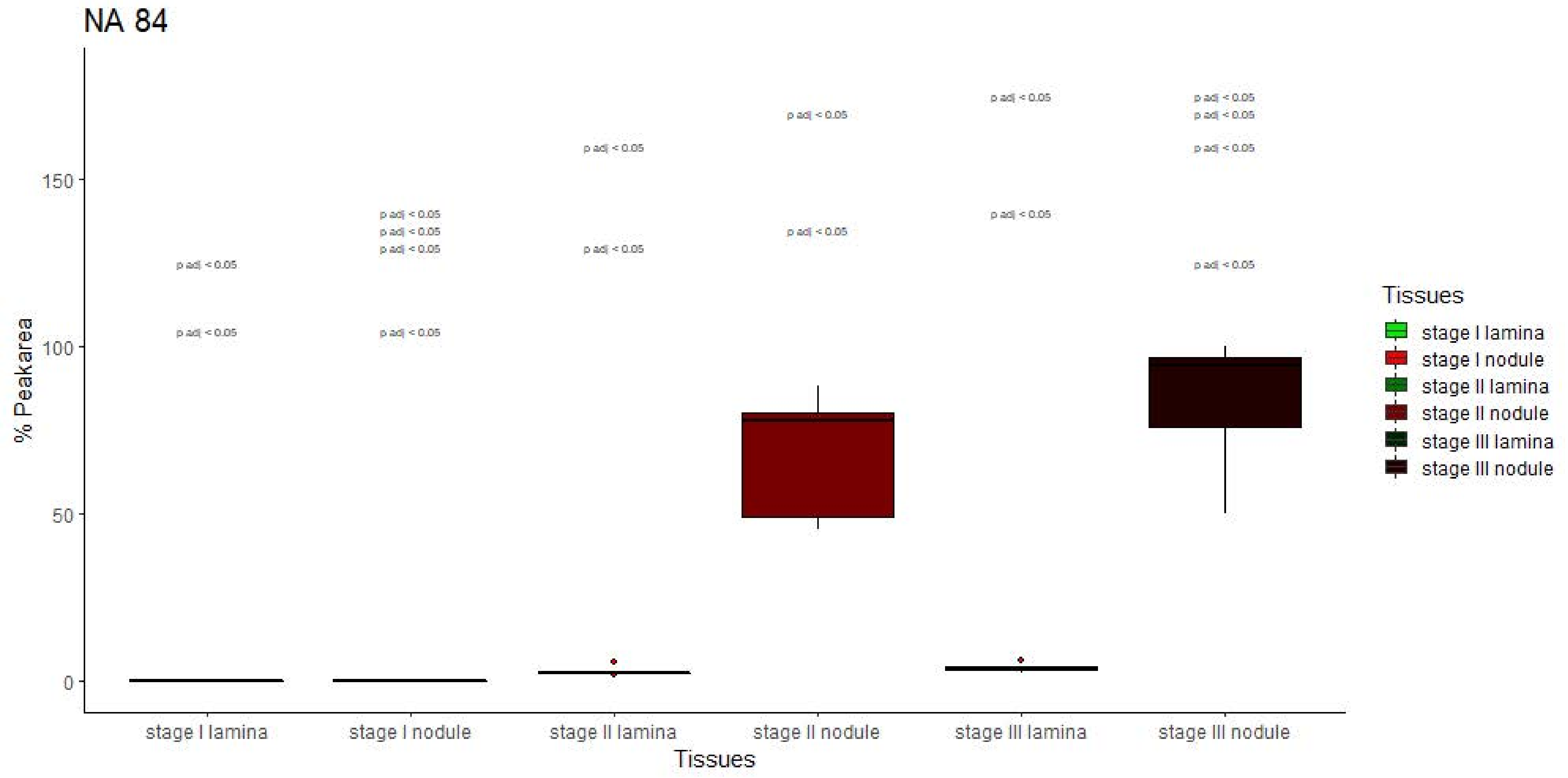
NA 84
% Peakarea
150
100
50
0
p adj < 0.05
Tissues
stage I lamina
stage I nodule
stage II lamina
stage II nodule
stage III lamina
stage III nodule

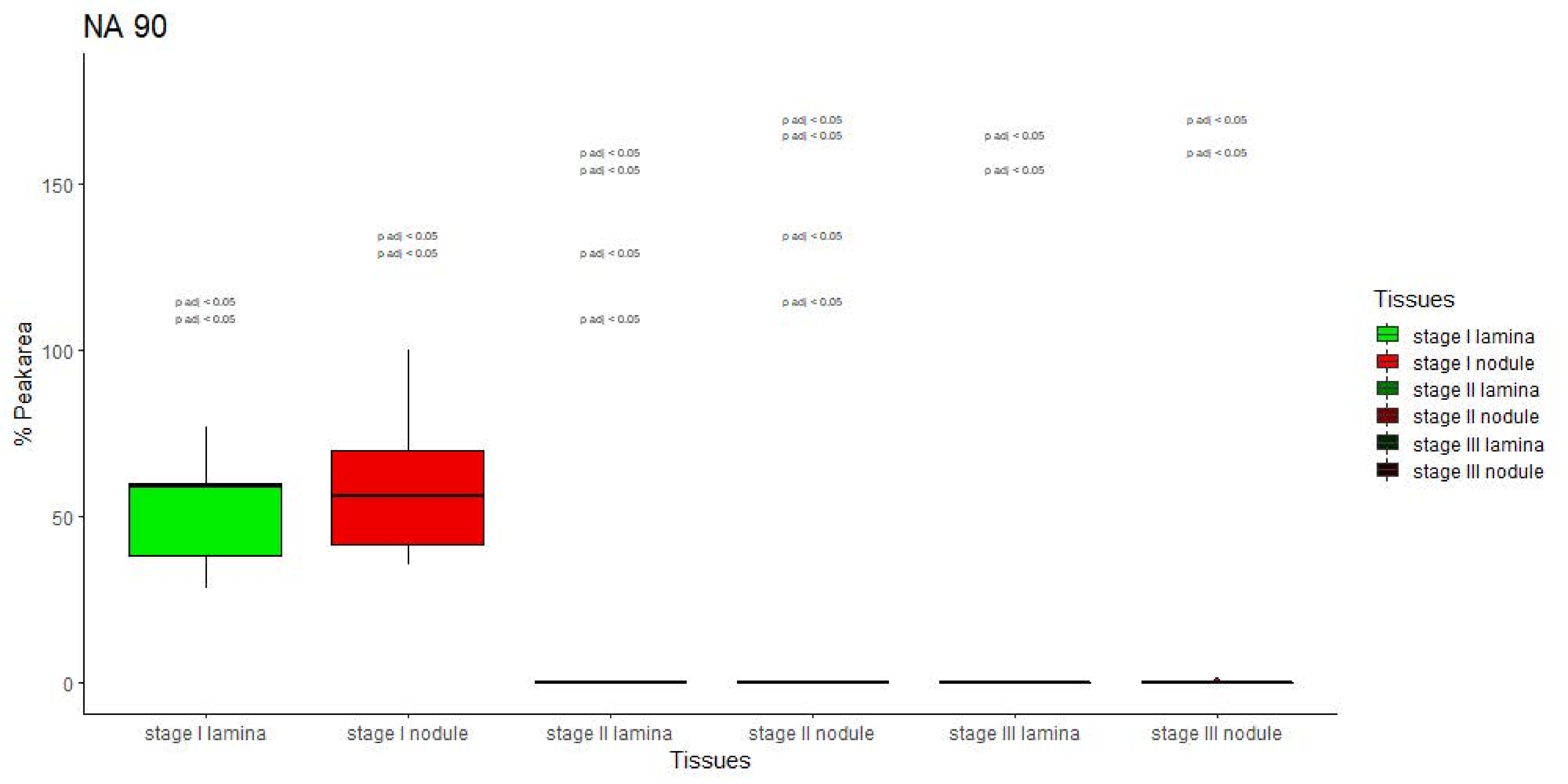
NA 90
% Peakarea
150
100
50
0
p adj < 0.05
Tissues
stage I lamina
stage I nodule
stage II lamina
stage II nodule
stage III lamina
stage III nodule
Tissues

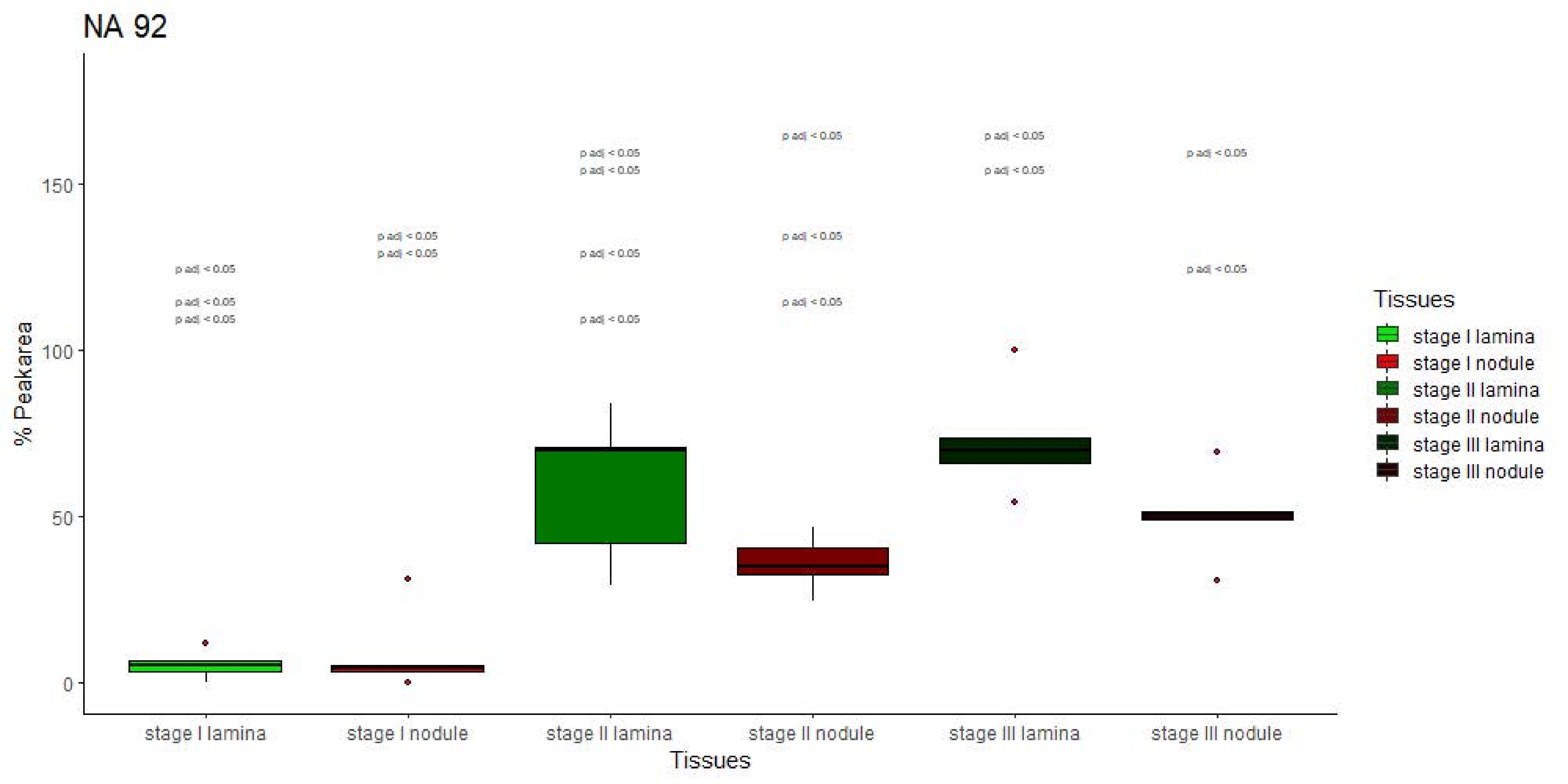
NA 92
% Peakarea
150
100
50
0
p adj < 0.05
stage I lamina
stage I nodule
stage II lamina
stage II nodule
stage III lamina
stage III nodule
Tissues
Tissues
stage I lamina
stage I nodule
stage II lamina
stage II nodule
stage III lamina
stage III nodule

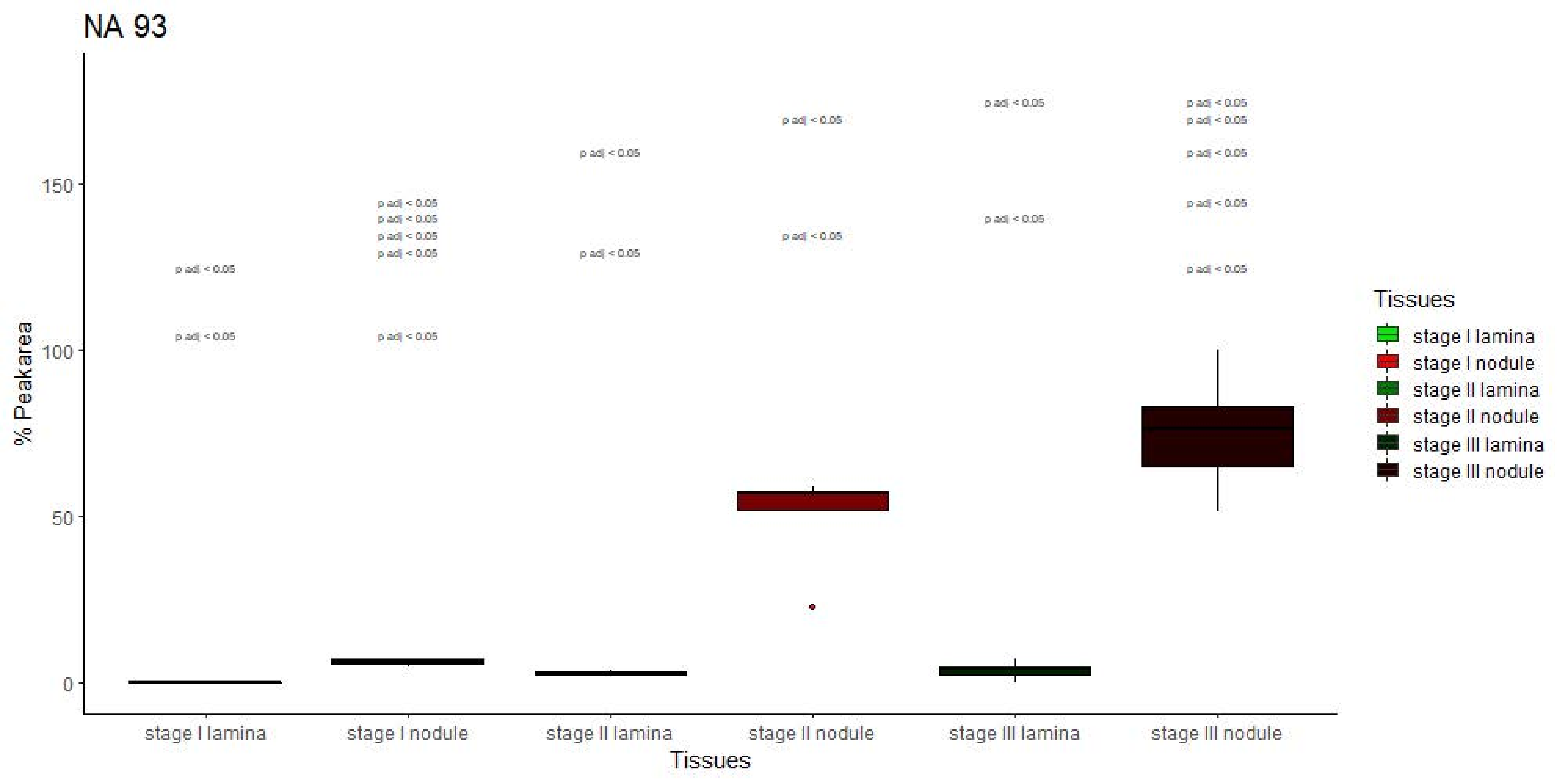

NA 93
% Peakarea
150
100
50
0
p adj < 0.05
Tissues
stage I lamina
stage I nodule
stage II lamina
stage II nodule
stage III lamina
stage III nodule

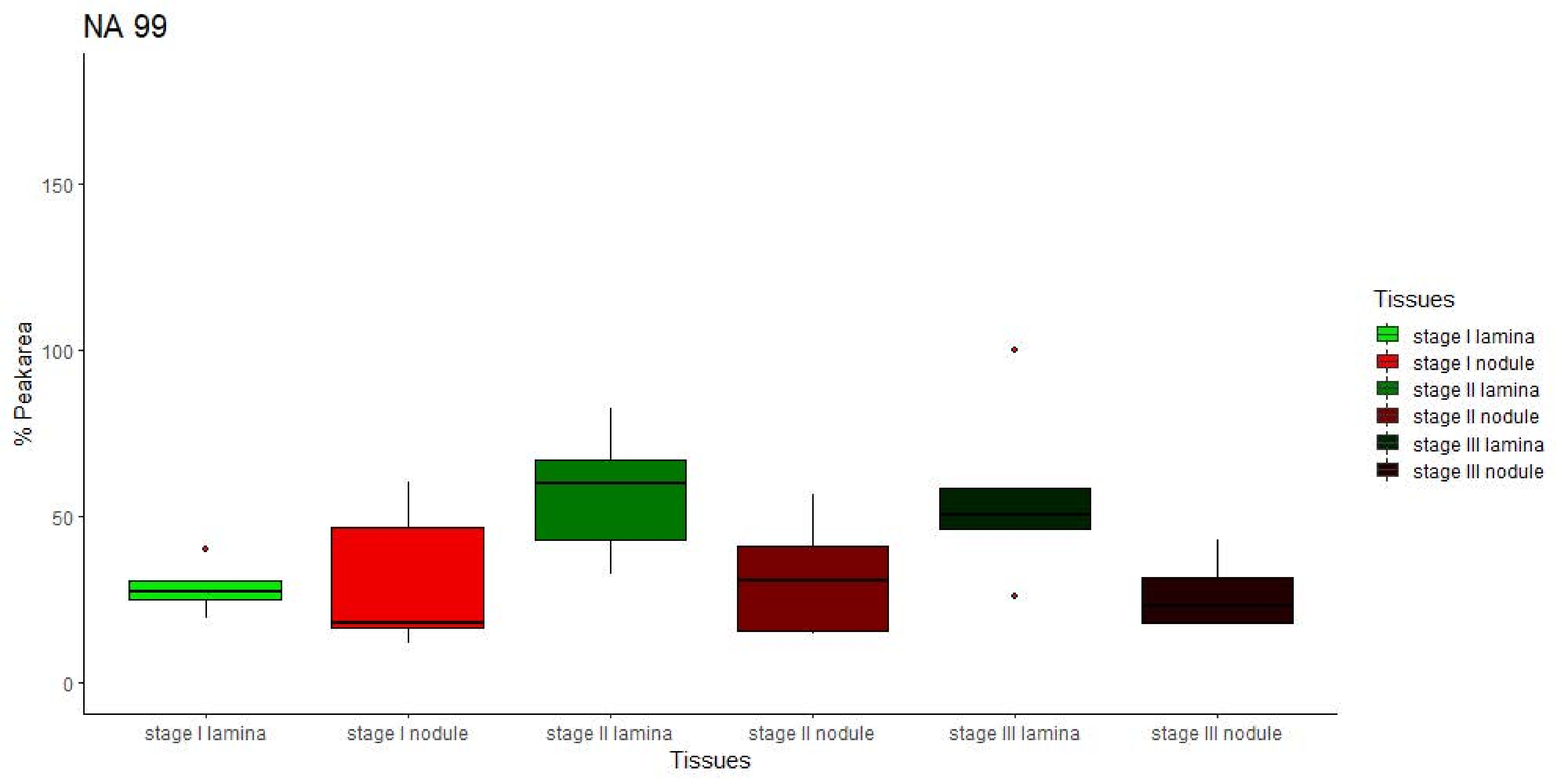

NA 99
% Peakarea
0
50
100
150
stage I lamina
stage I nodule
stage II lamina
stage II nodule
stage III lamina
stage III nodule
Tissues
Tissues
stage I lamina
stage I nodule
stage II lamina
stage II nodule
stage III lamina
stage III nodule

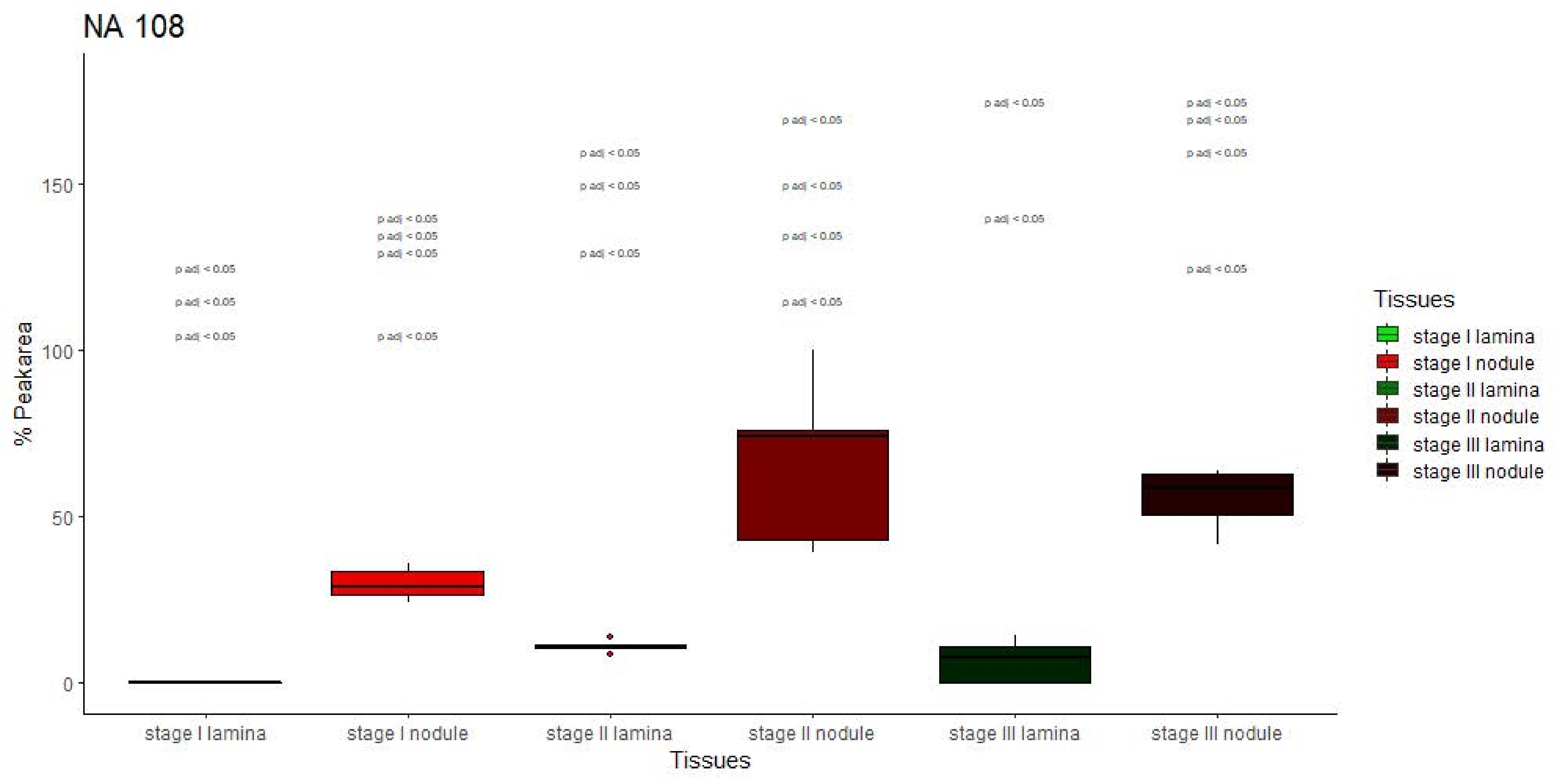

NA 108
% Peakarea
150
100
50
0
p adj < 0.05
stage I lamina
stage I nodule
stage II lamina
stage II nodule
stage III lamina
stage III nodule
Tissues
Tissues
stage I lamina
stage I nodule
stage II lamina
stage II nodule
stage III lamina
stage III nodule

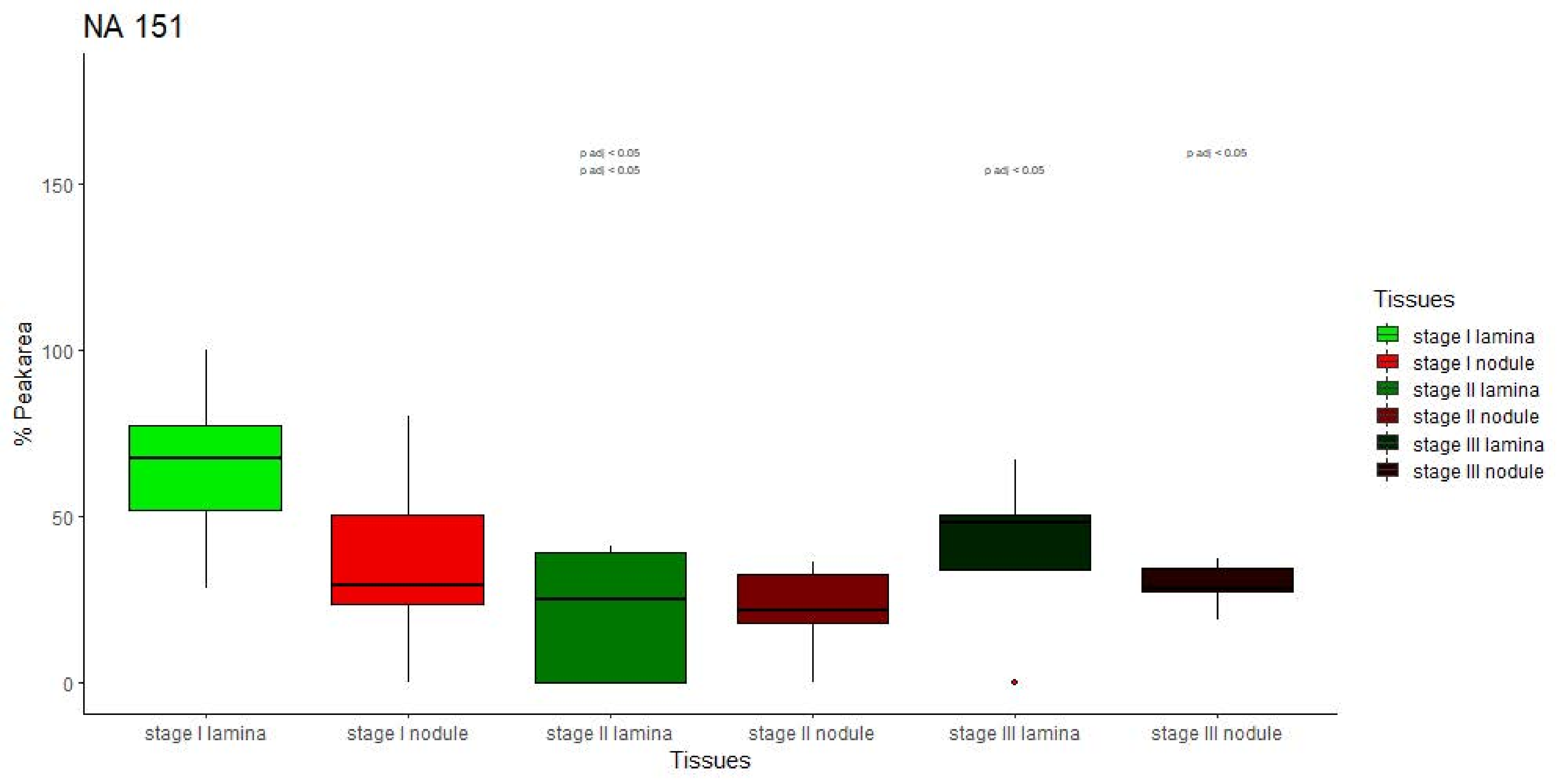

NA 151
p adj < 0.05
p adj < 0.05
p adj < 0.05
p adj < 0.05
150
100
50
0
% Peakarea
stage I lamina
stage I nodule
stage II lamina
stage II nodule
stage III lamina
stage III nodule
Tissues
Tissues
stage I lamina
stage I nodule
stage II lamina
stage II nodule
stage III lamina
stage III nodule

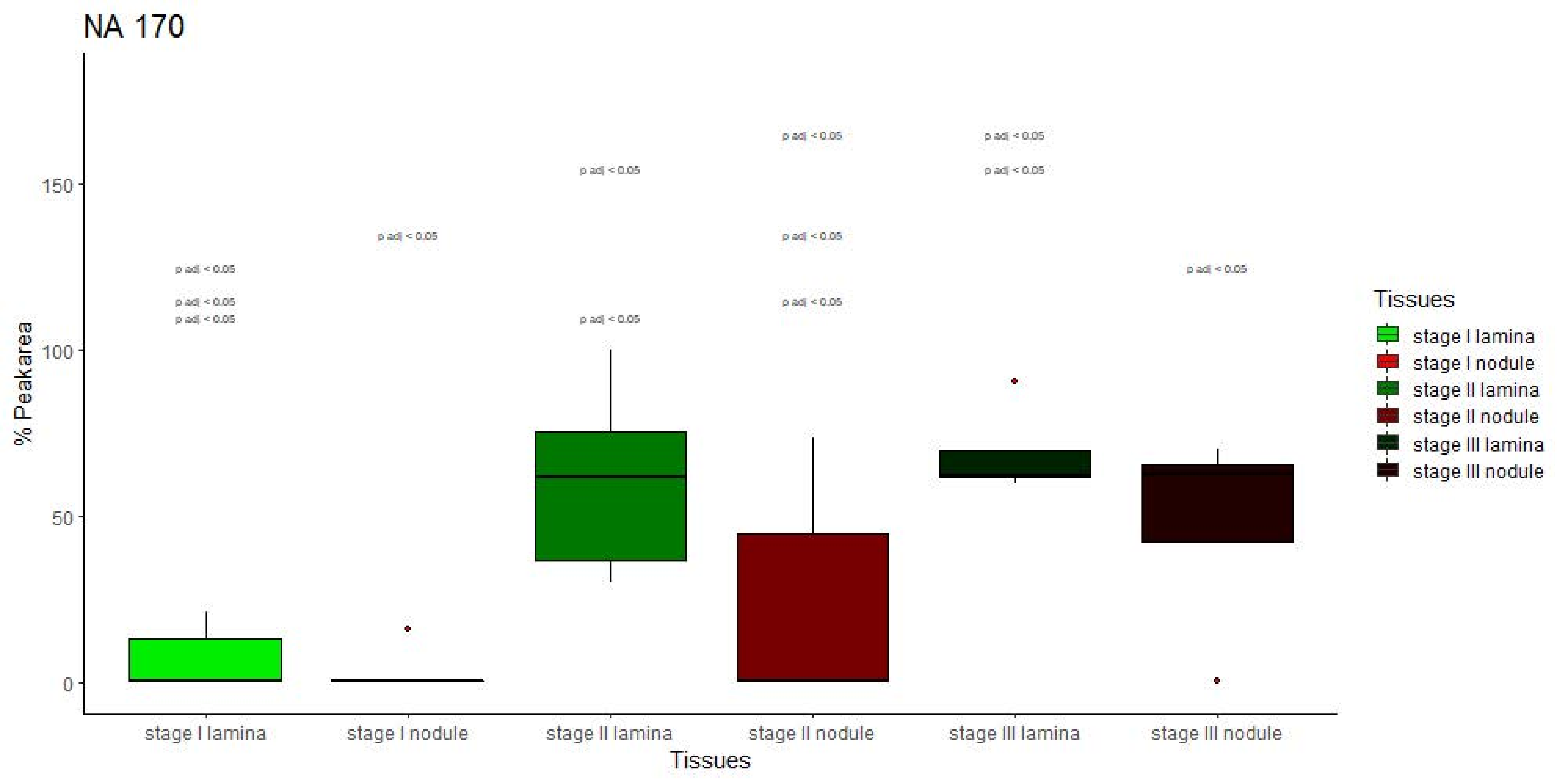

NA 170
% Peakarea
150
100
50
0
p adj < 0.05
Tissues
stage I lamina
stage I nodule
stage II lamina
stage II nodule
stage III lamina
stage III nodule

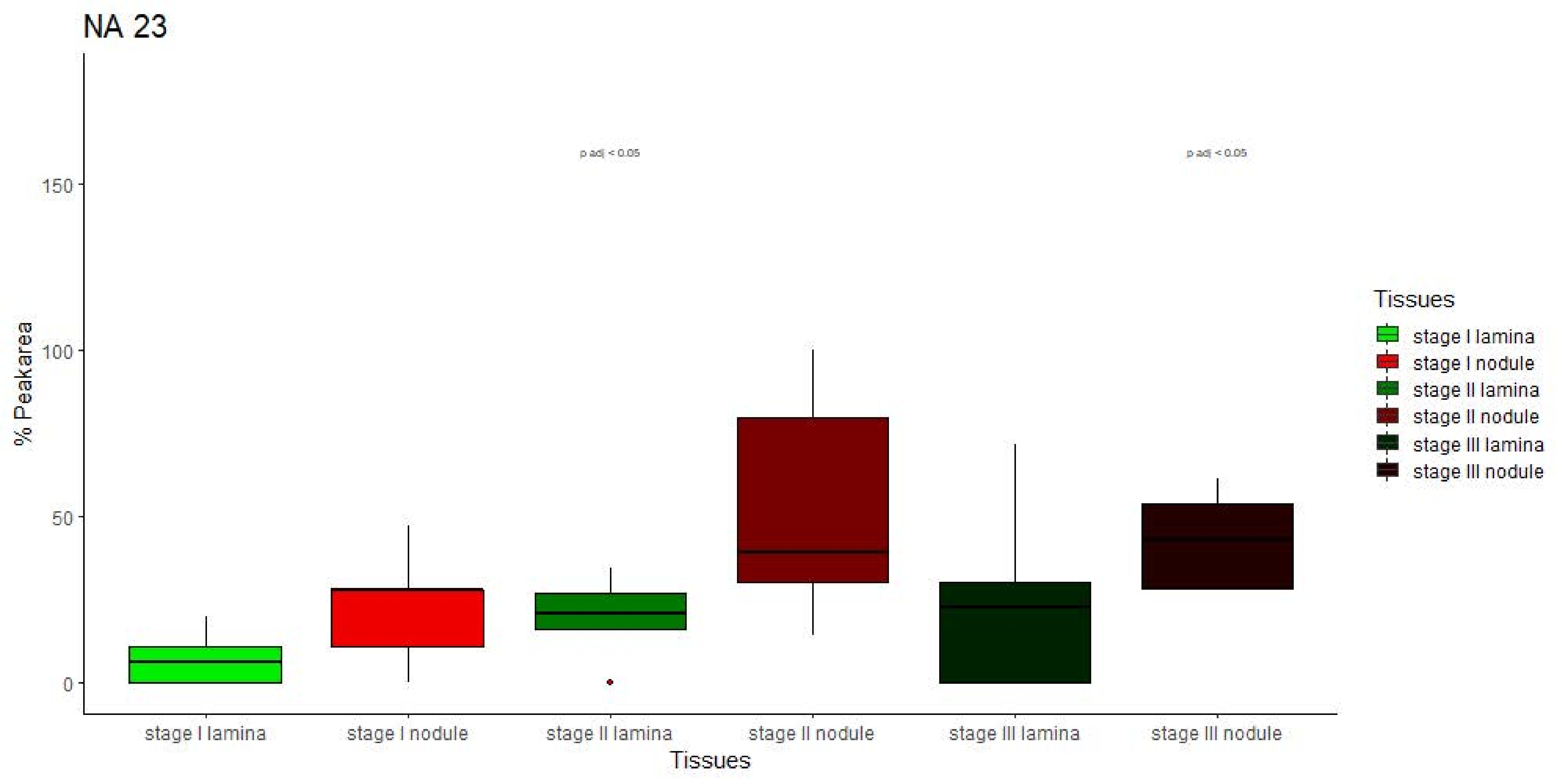

NA 23
p adj < 0.05
p adj < 0.05
150
100
50
0
% Peakarea
stage I lamina
stage I nodule
stage II lamina
stage II nodule
stage III lamina
stage III nodule
Tissues
Tissues
stage I lamina
stage I nodule
stage II lamina
stage II nodule
stage III lamina
stage III nodule

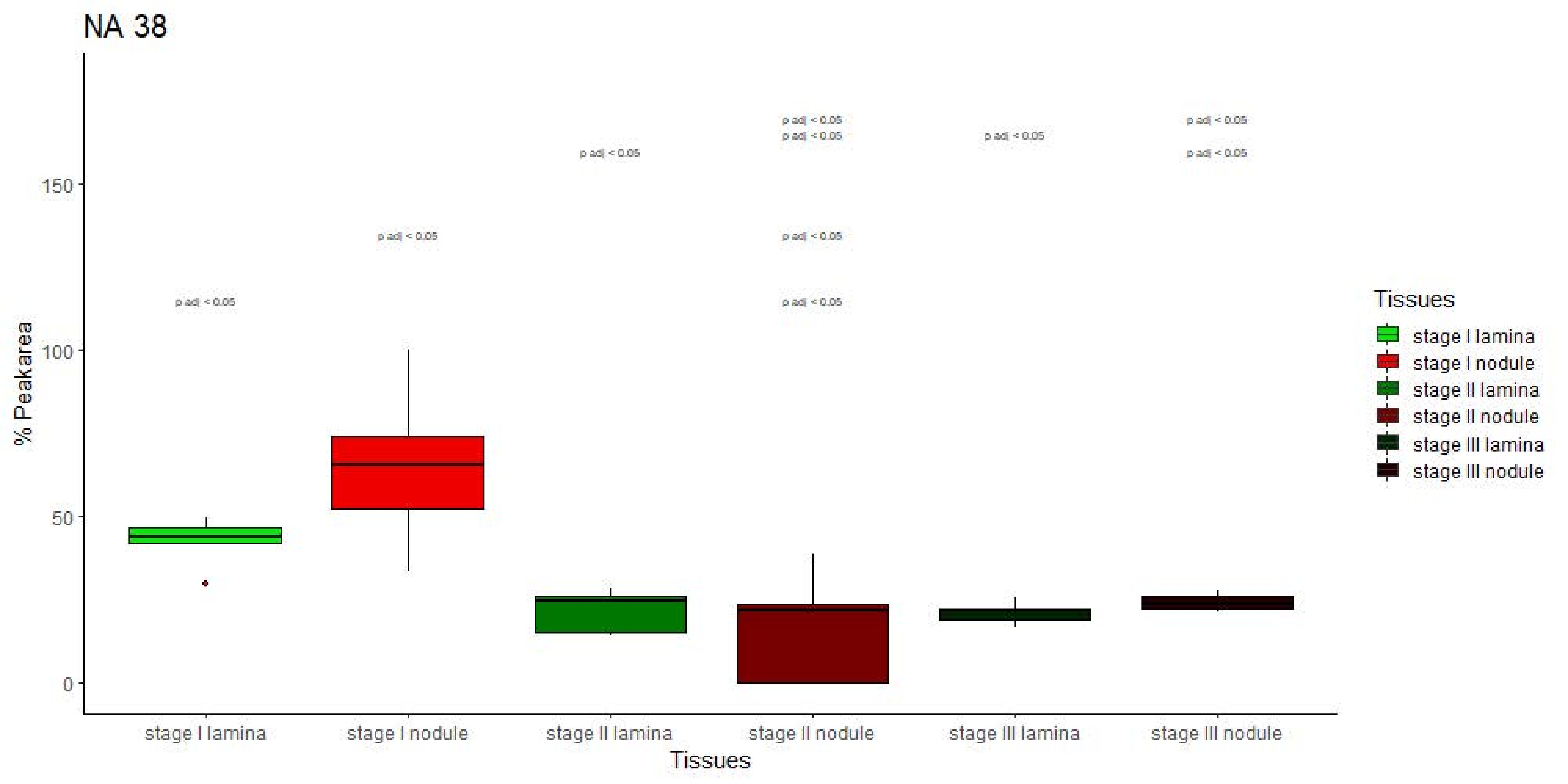

NA 38
% Peakarea
0
50
100
150
p adj < 0.05
p adj < 0.05
p adj < 0.05
p adj < 0.05
p adj < 0.05
p adj < 0.05
p adj < 0.05
p adj < 0.05
p adj < 0.05
p adj < 0.05
stage I lamina
stage I nodule
stage II lamina
stage II nodule
stage III lamina
stage III nodule
Tissues
Tissues
stage I lamina
stage I nodule
stage II lamina
stage II nodule
stage III lamina
stage III nodule

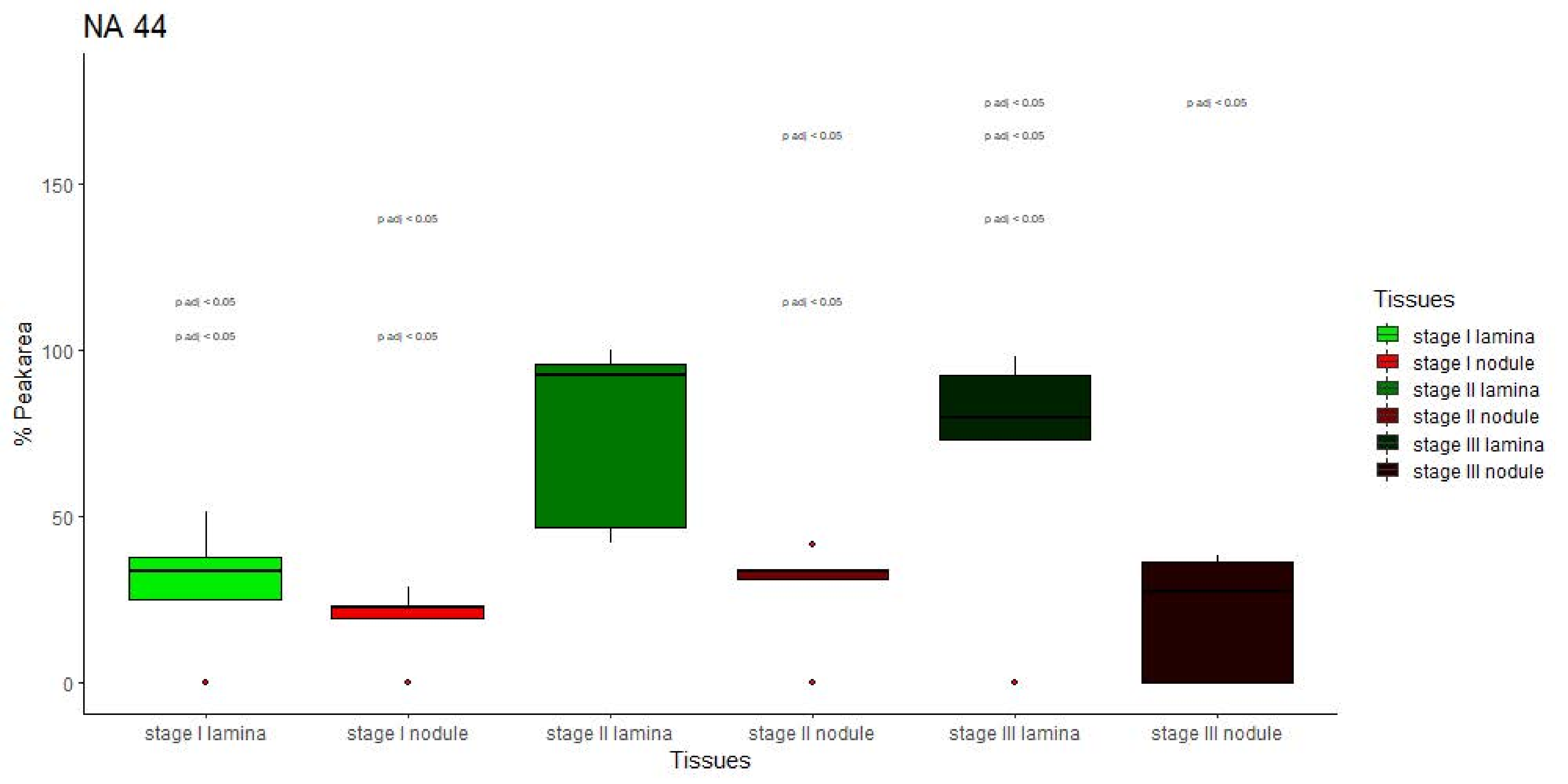
NA 44
% Peakarea
150
100
50
0
p adj < 0.05
Tissues
stage I lamina
stage I nodule
stage II lamina
stage II nodule
stage III lamina
stage III nodule

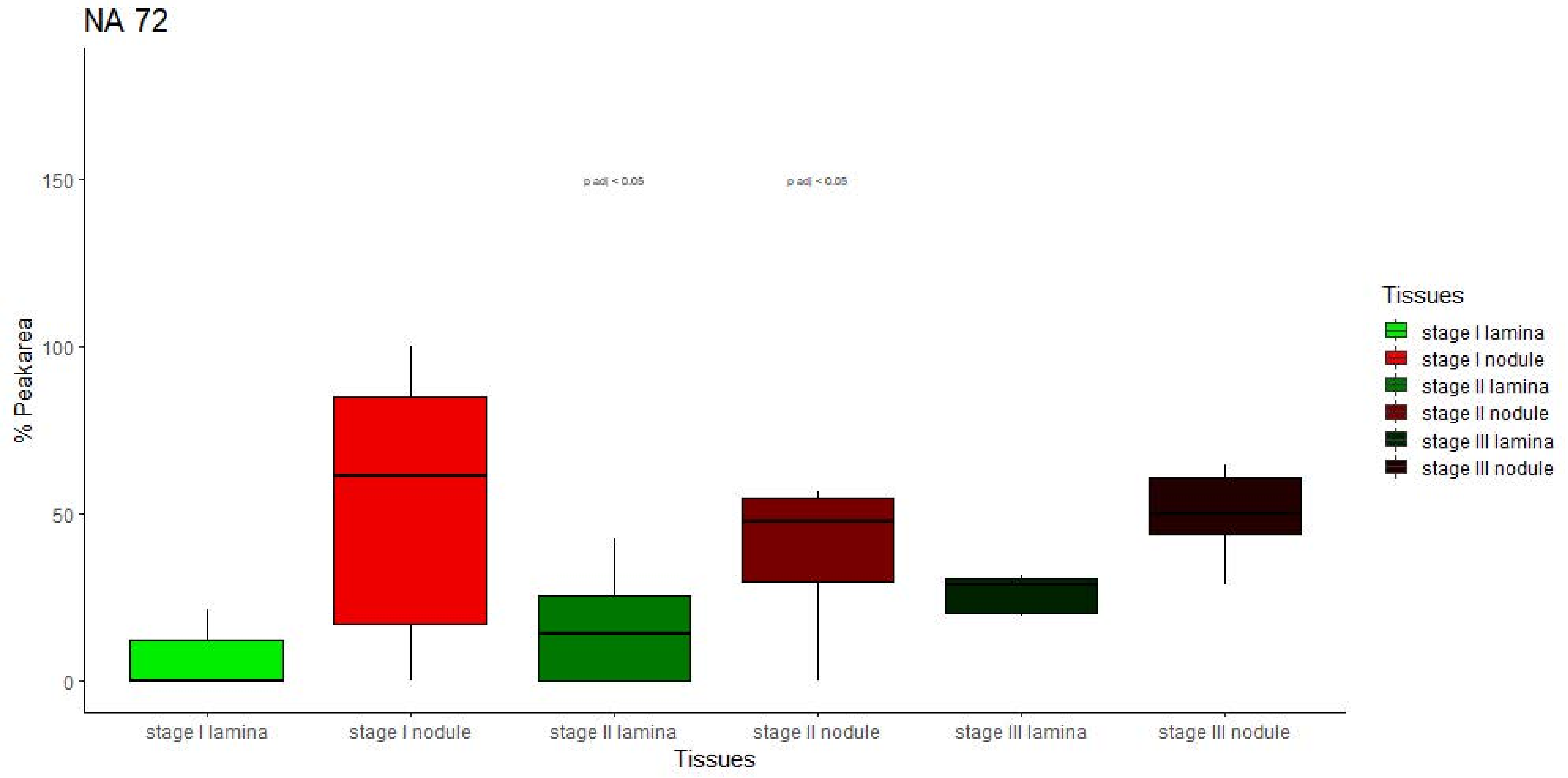
NA 72
% Peakarea
150
100
50
0
p adj < 0.05
p adj < 0.05
stage I lamina
stage I nodule
stage II lamina
stage II nodule
stage III lamina
stage III nodule
Tissues
Tissues
stage I lamina
stage I nodule
stage II lamina
stage II nodule
stage III lamina
stage III nodule

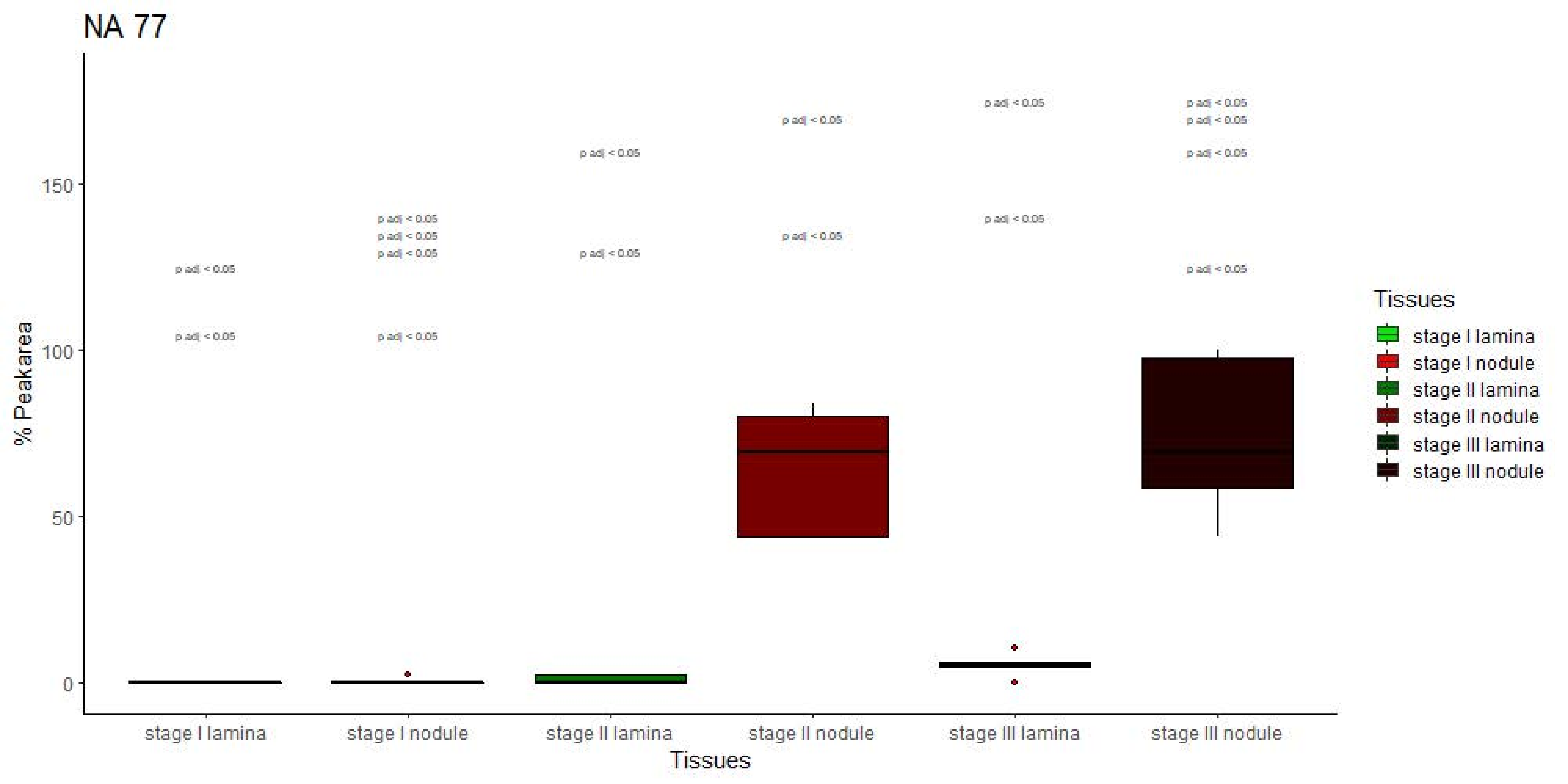

NA 77
% Peakarea
0
50
100
150
p adj < 0.05
stage I lamina
stage I nodule
stage II lamina
stage II nodule
stage III lamina
stage III nodule
Tissues

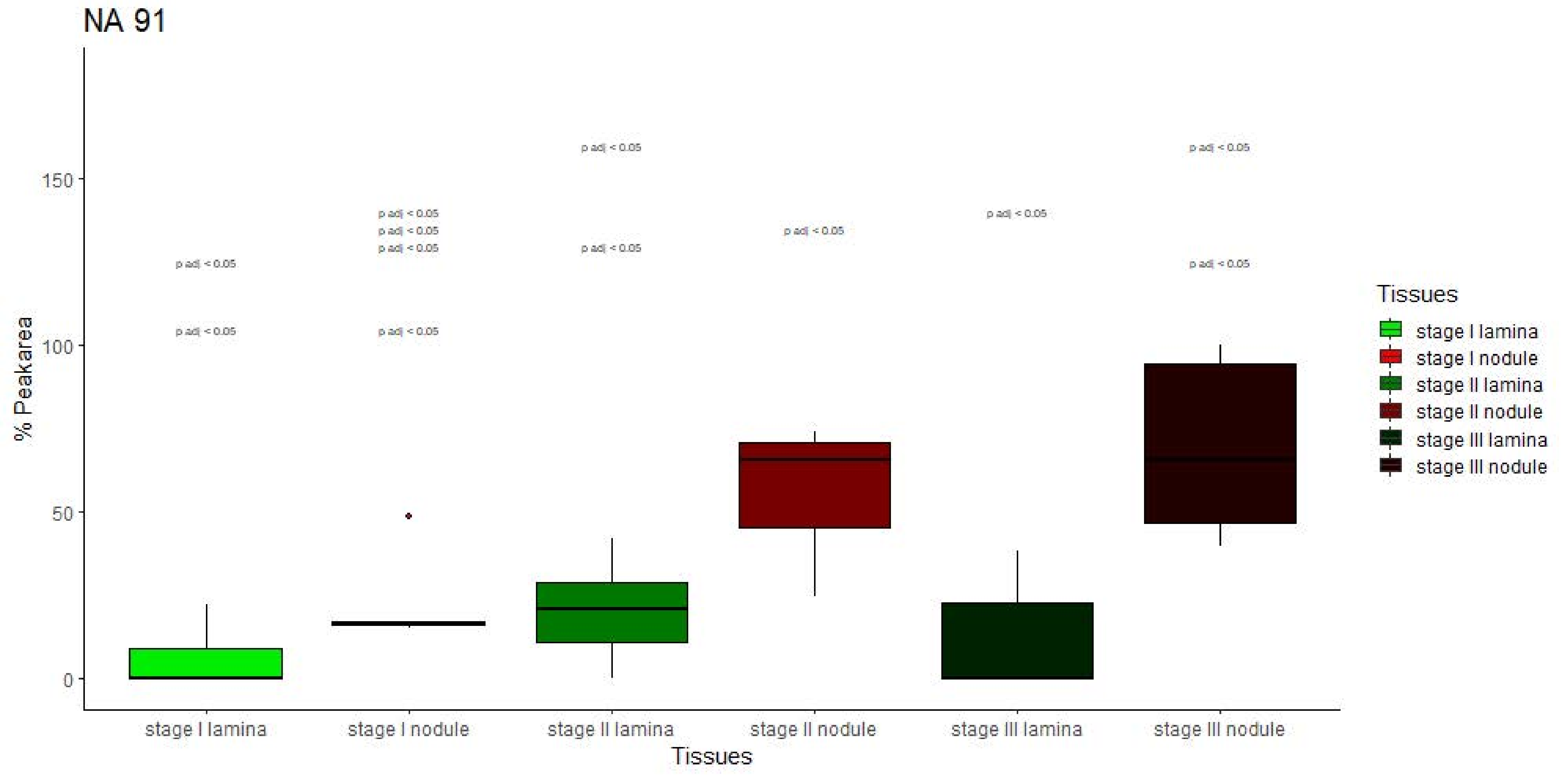

NA 91
% Peakarea
150
100
50
0
p adj < 0.05
Tissues
stage I lamina
stage I nodule
stage II lamina
stage II nodule
stage III lamina
stage III nodule

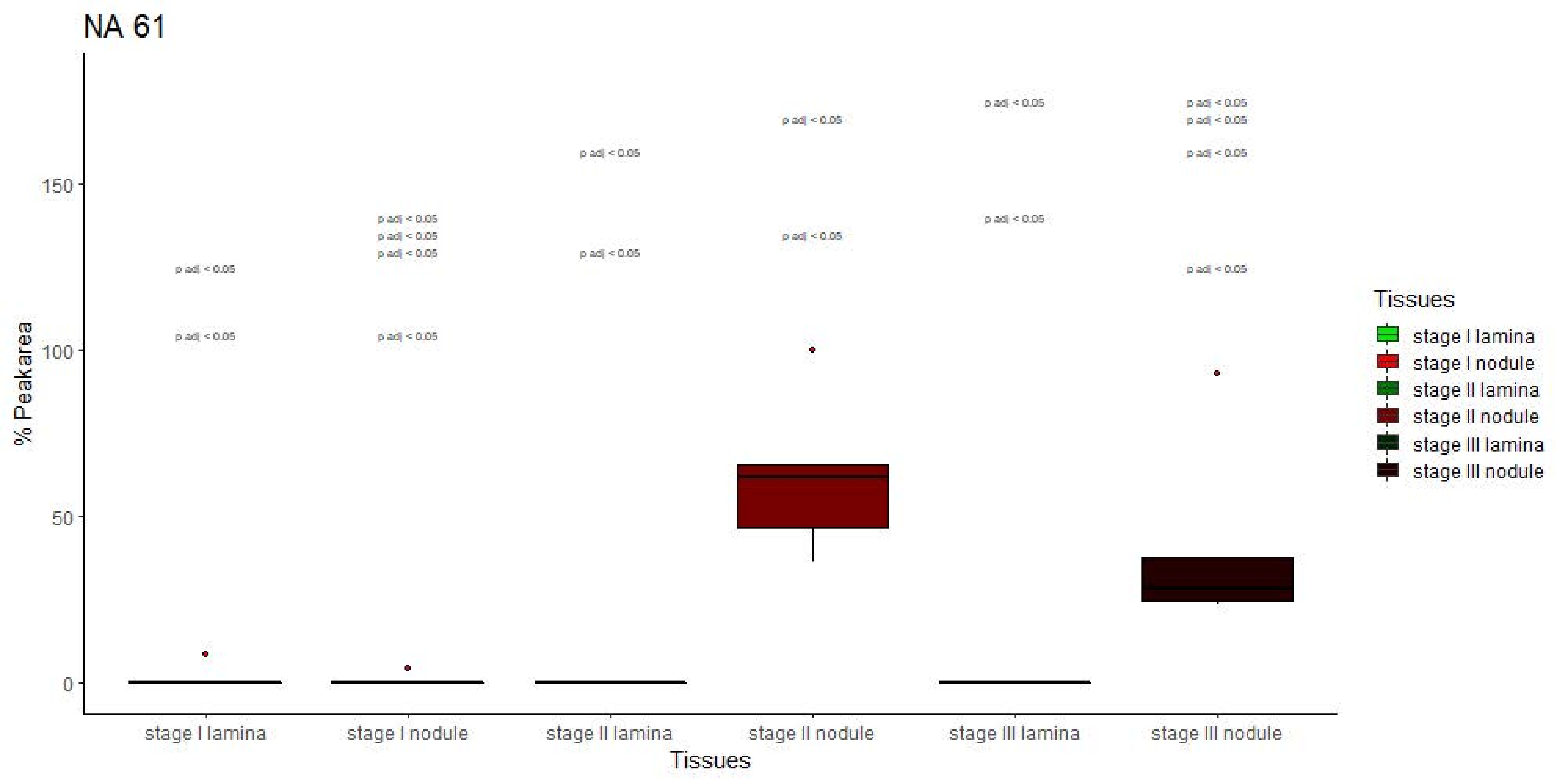

NA 61
% Peakarea
150
100
50
0
p adj < 0.05
Tissues
stage I lamina
stage I nodule
stage II lamina
stage II nodule
stage III lamina
stage III nodule

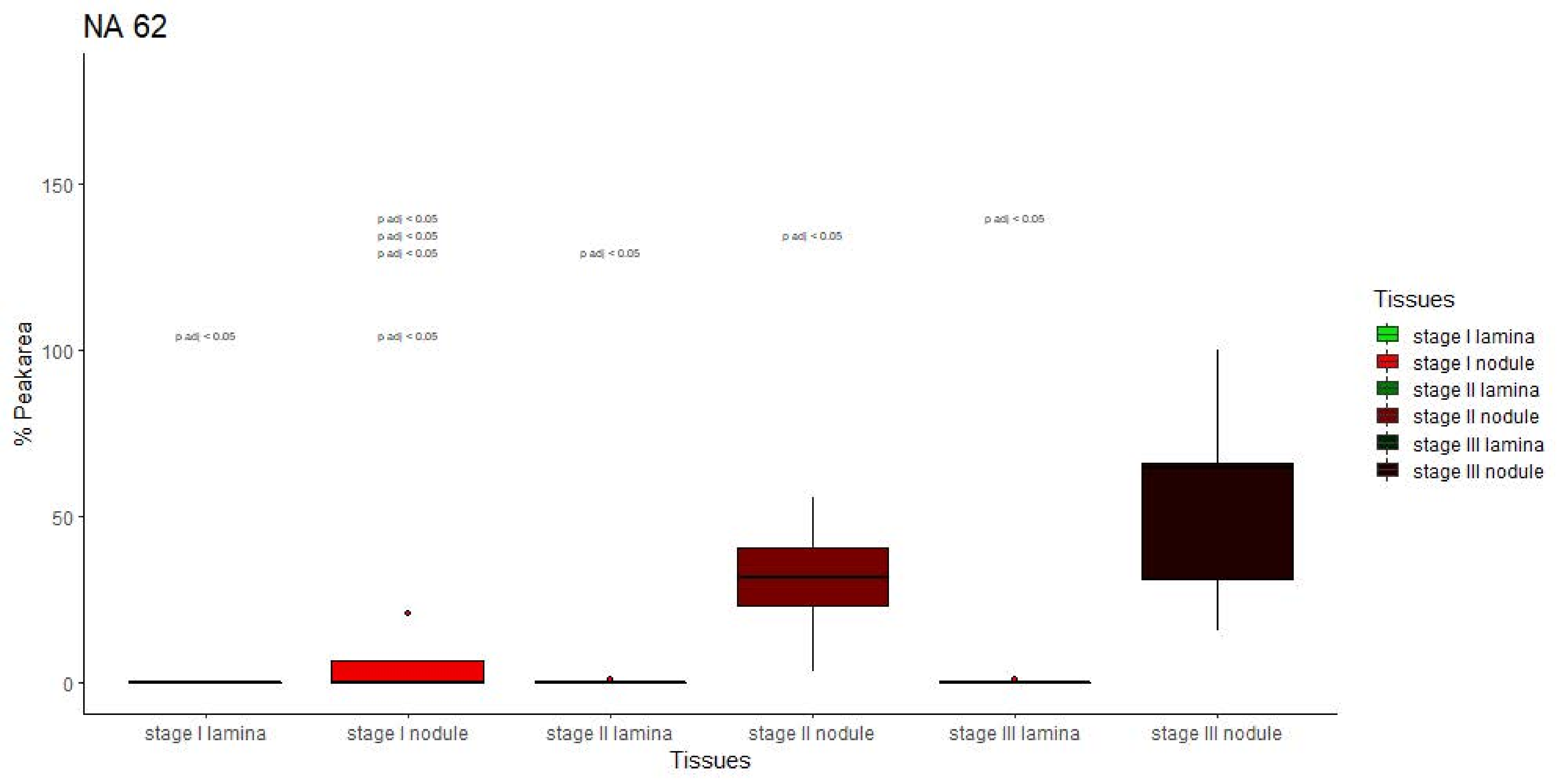
NA 62
% Peakarea
150
100
50
0
p adj < 0.05
p adj < 0.05
p adj < 0.05
p adj < 0.05
p adj < 0.05
p adj < 0.05
p adj < 0.05
p adj < 0.05
stage I lamina
stage I nodule
stage II lamina
stage II nodule
stage III lamina
stage III nodule
Tissues
Tissues
stage I lamina
stage I nodule
stage II lamina
stage II nodule
stage III lamina
stage III nodule

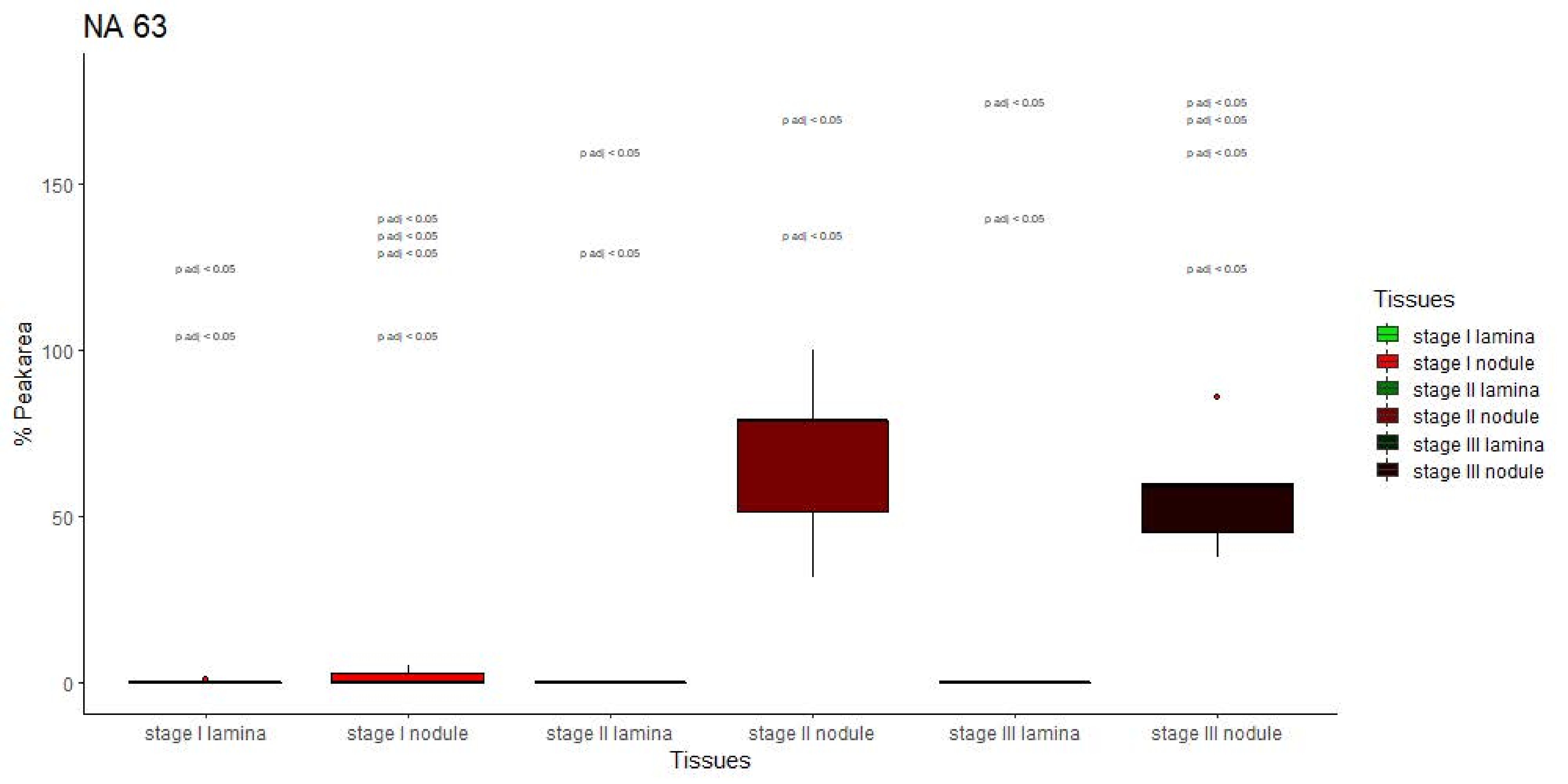

NA 63
% Peakarea
150
100
50
0
p adj < 0.05
stage I lamina
stage I nodule
stage II lamina
stage II nodule
stage III lamina
stage III nodule
Tissues
Tissues
stage I lamina
stage I nodule
stage II lamina
stage II nodule
stage III lamina
stage III nodule

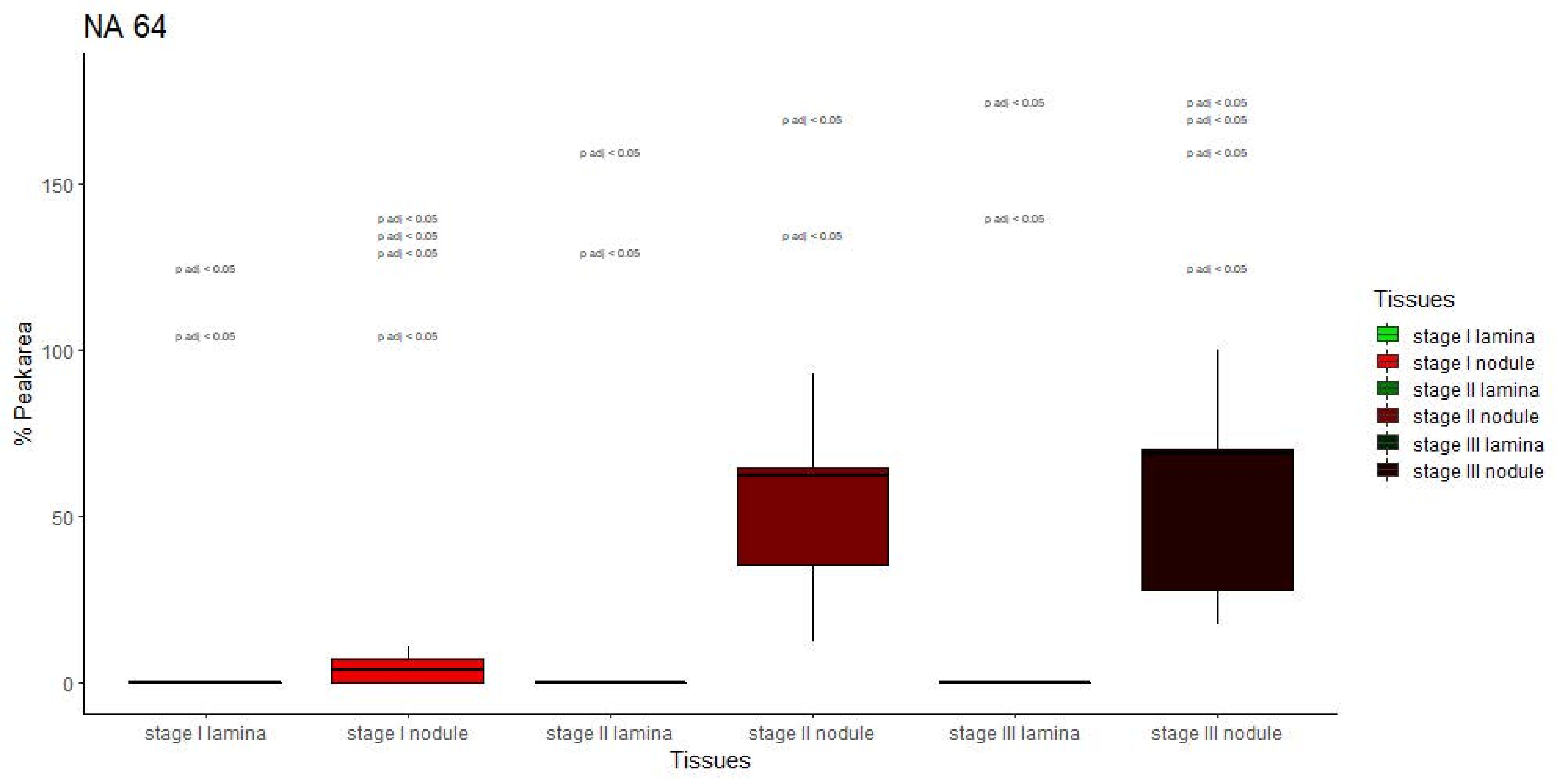
NA 64
% Peakarea
150
100
50
0
p adj < 0.05
stage I lamina
stage I nodule
stage II lamina
stage II nodule
stage III lamina
stage III nodule
Tissues
Tissues
stage I lamina
stage I nodule
stage II lamina
stage II nodule
stage III lamina
stage III nodule

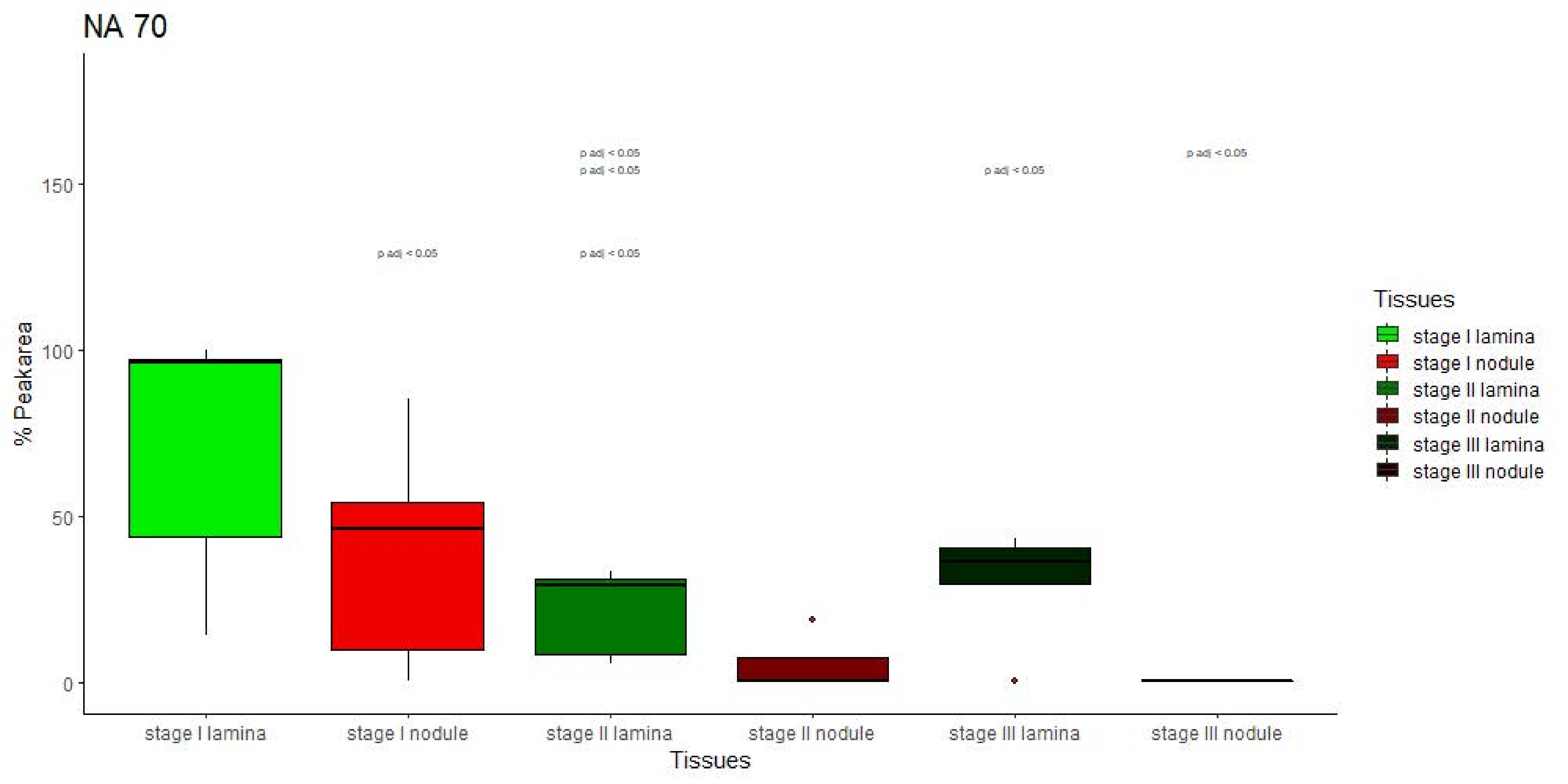
NA 70
p adj < 0.05
p adj < 0.05
p adj < 0.05
p adj < 0.05
p adj < 0.05
p adj < 0.05
p adj < 0.05
150
100
50
0
% Peakarea
stage I lamina
stage I nodule
stage II lamina
stage II nodule
stage III lamina
stage III nodule
Tissues
Tissues
stage I lamina
stage I nodule
stage II lamina
stage II nodule
stage III lamina
stage III nodule

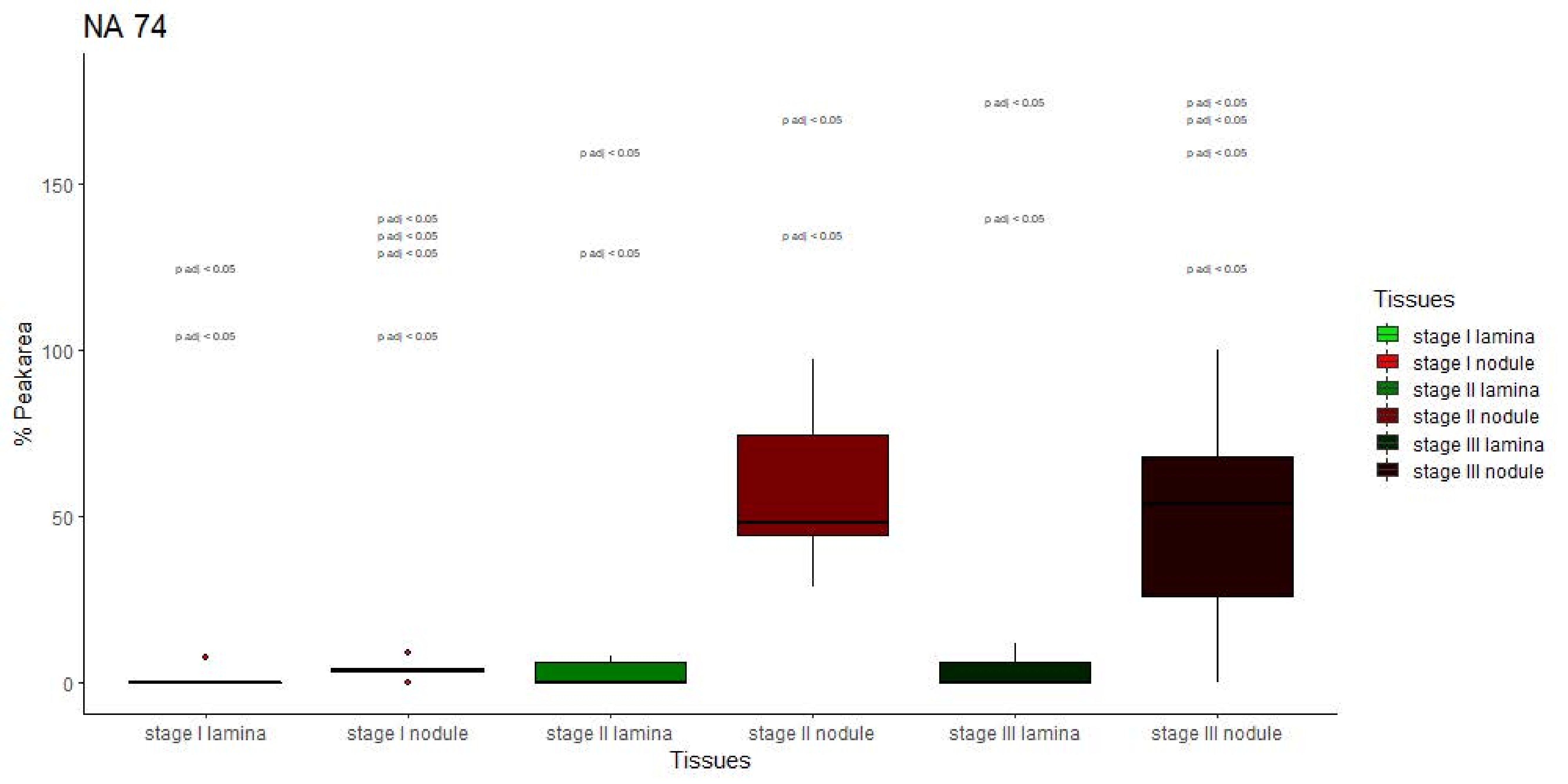

NA 74
% Peakarea
Tissues
150
100
50
0
p adj < 0.05
stage I lamina
stage I nodule
stage II lamina
stage II nodule
stage III lamina
stage III nodule

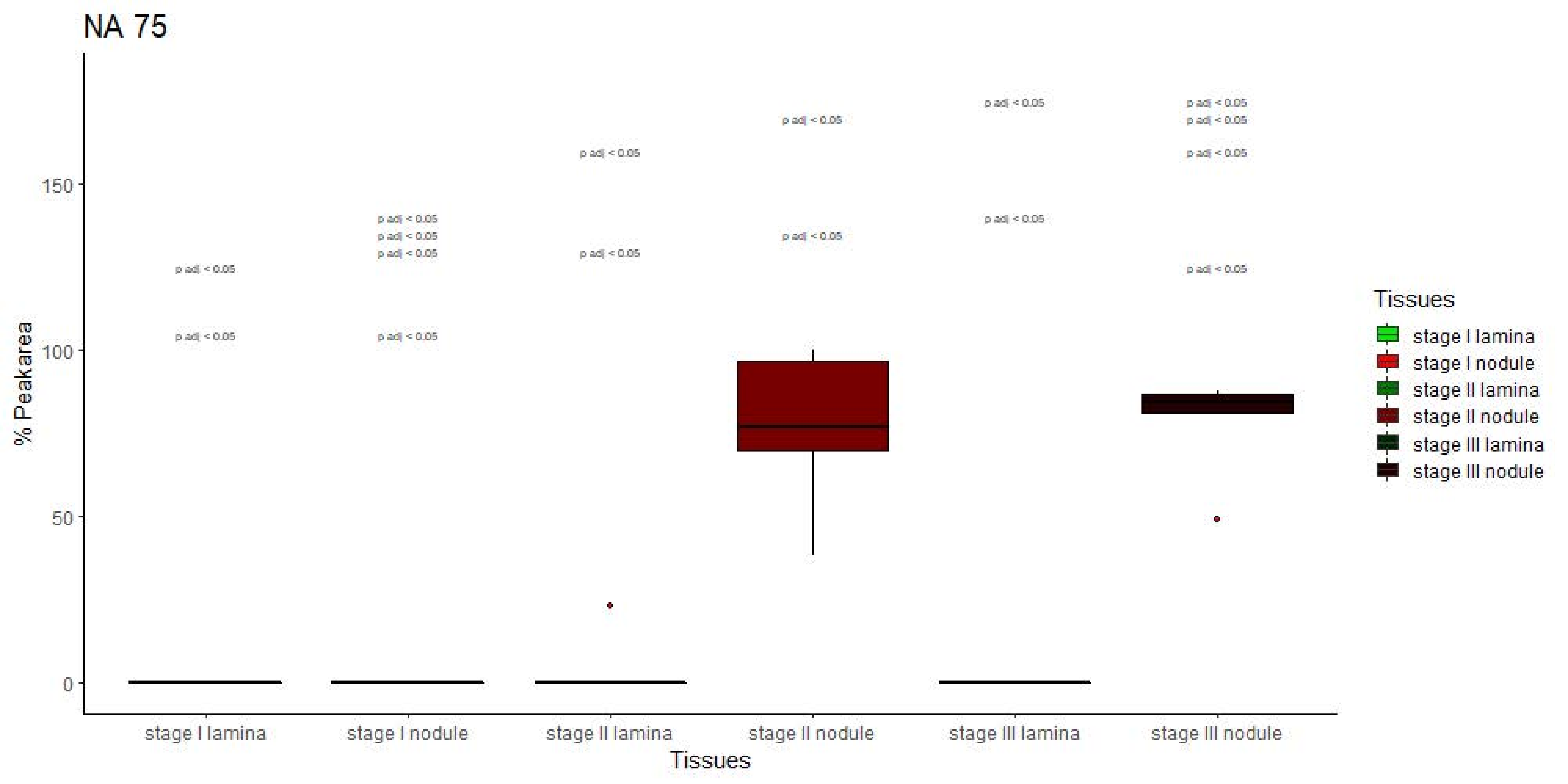

NA 75
% Peakarea
0
50
100
150
p adj < 0.05
stage I lamina
stage I nodule
stage II lamina
stage II nodule
stage III lamina
stage III nodule
Tissues

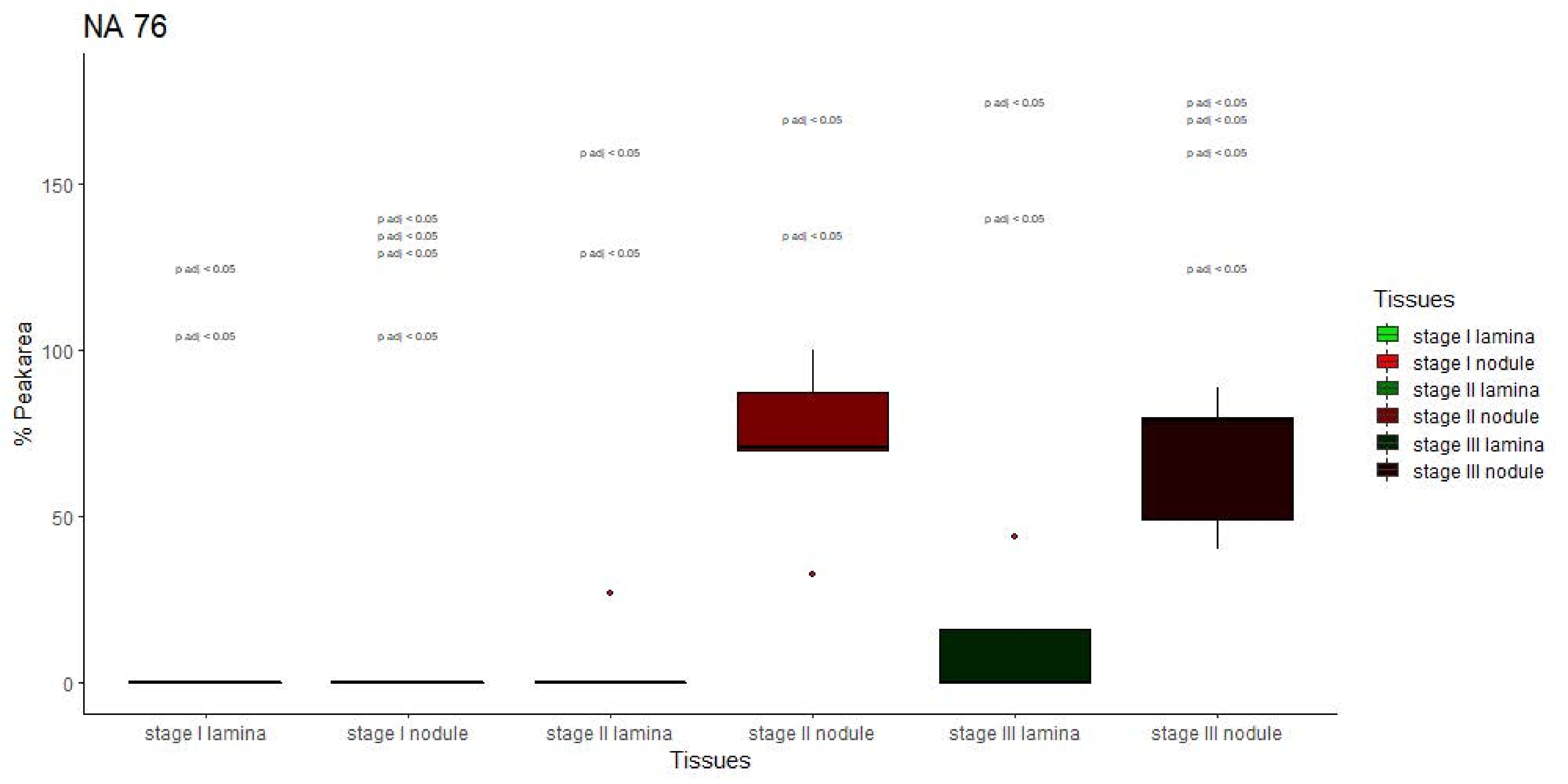

NA 76
% Peakarea
150
100
50
0
p adj < 0.05
stage I lamina
stage I nodule
stage II lamina
stage II nodule
stage III lamina
stage III nodule
Tissues
Tissues
stage I lamina
stage I nodule
stage II lamina
stage II nodule
stage III lamina
stage III nodule

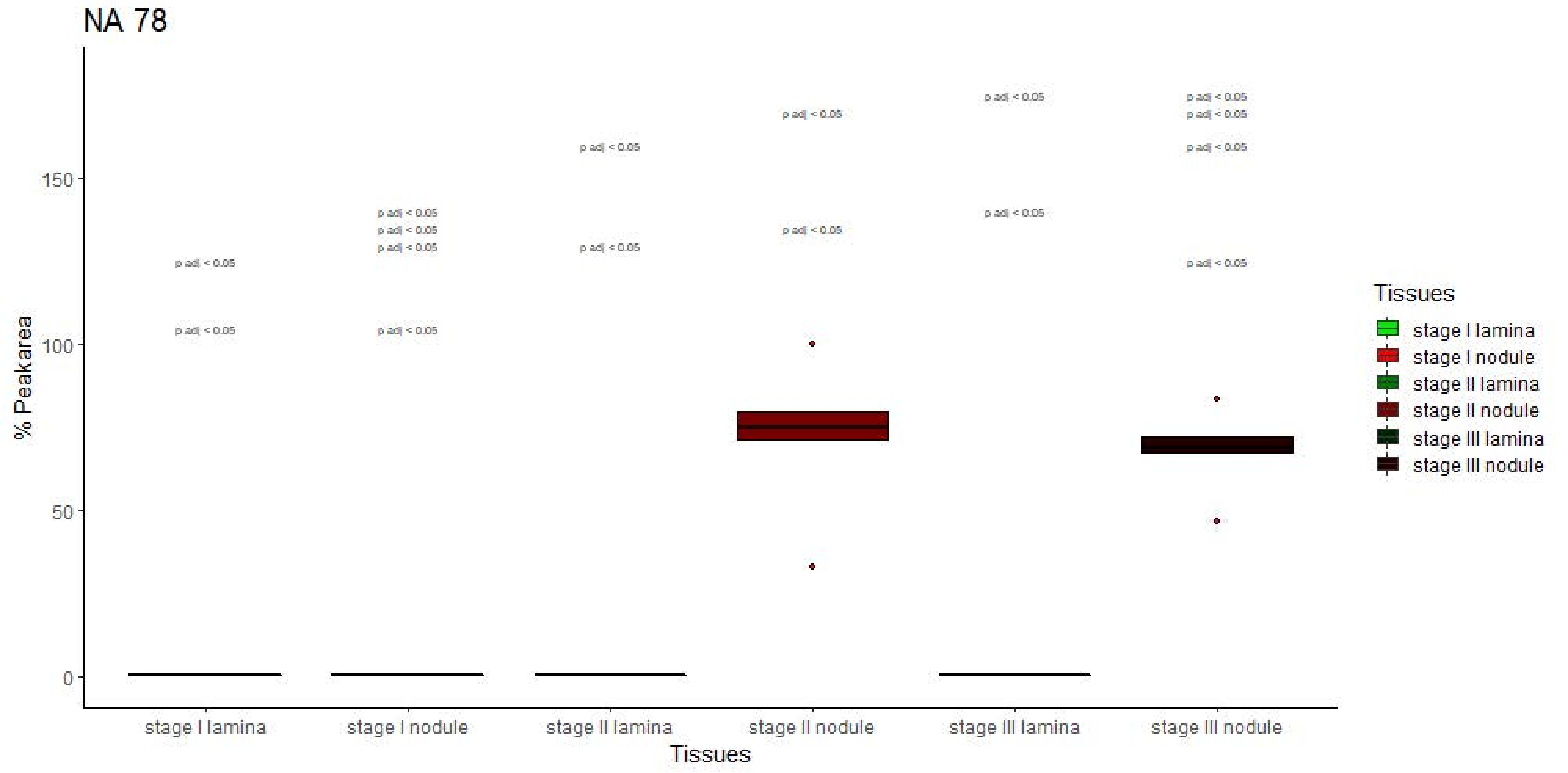
NA 78
% Peakarea
150
100
50
0
p adj < 0.05
stage I lamina
stage I nodule
stage II lamina
stage II nodule
stage III lamina
stage III nodule
Tissues
Tissues
stage I lamina
stage I nodule
stage II lamina
stage II nodule
stage III lamina
stage III nodule

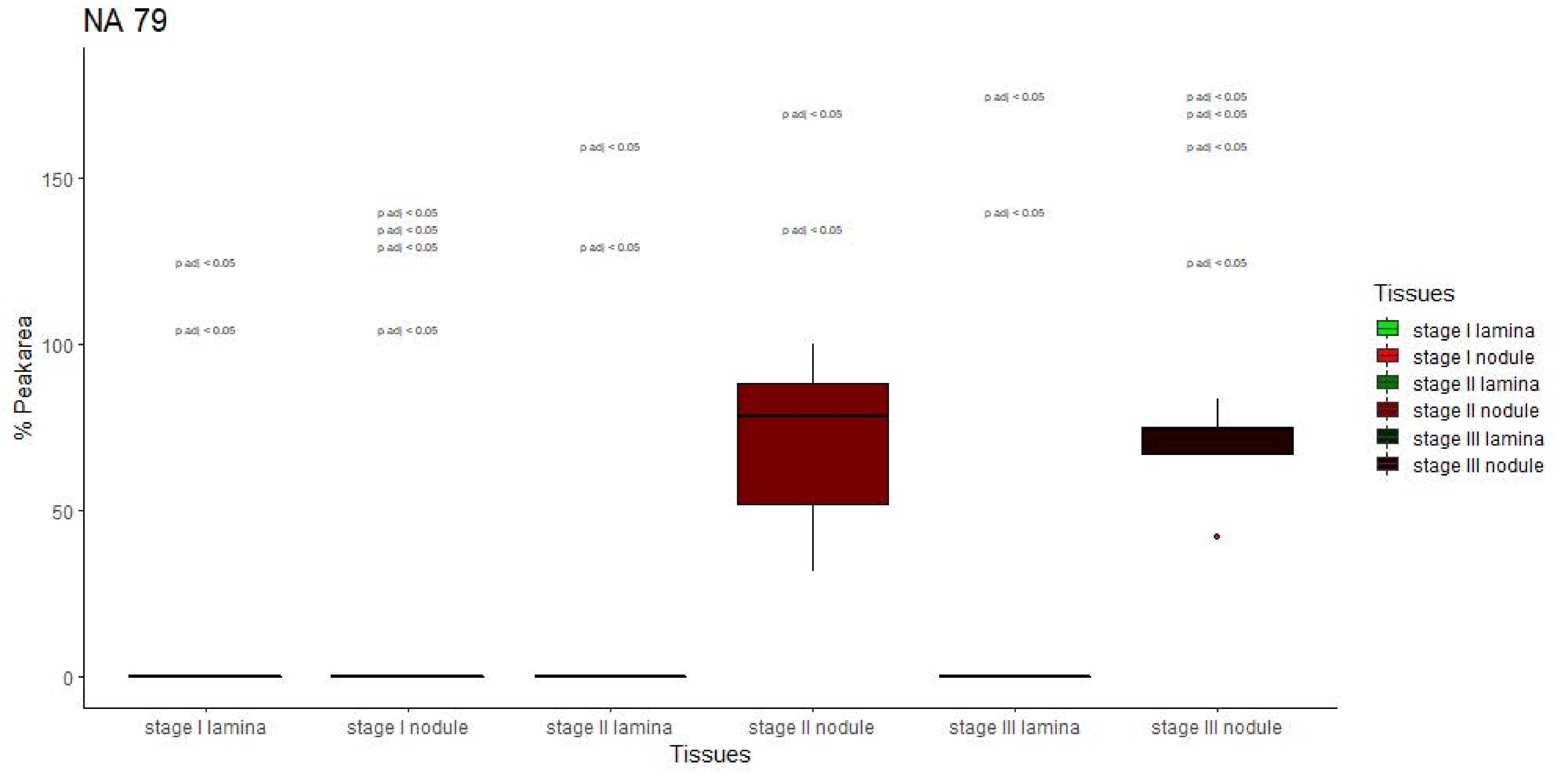

NA 79
% Peakarea
150
100
50
0
p adj < 0.05
Tissues
stage I lamina
stage I nodule
stage II lamina
stage II nodule
stage III lamina
stage III nodule

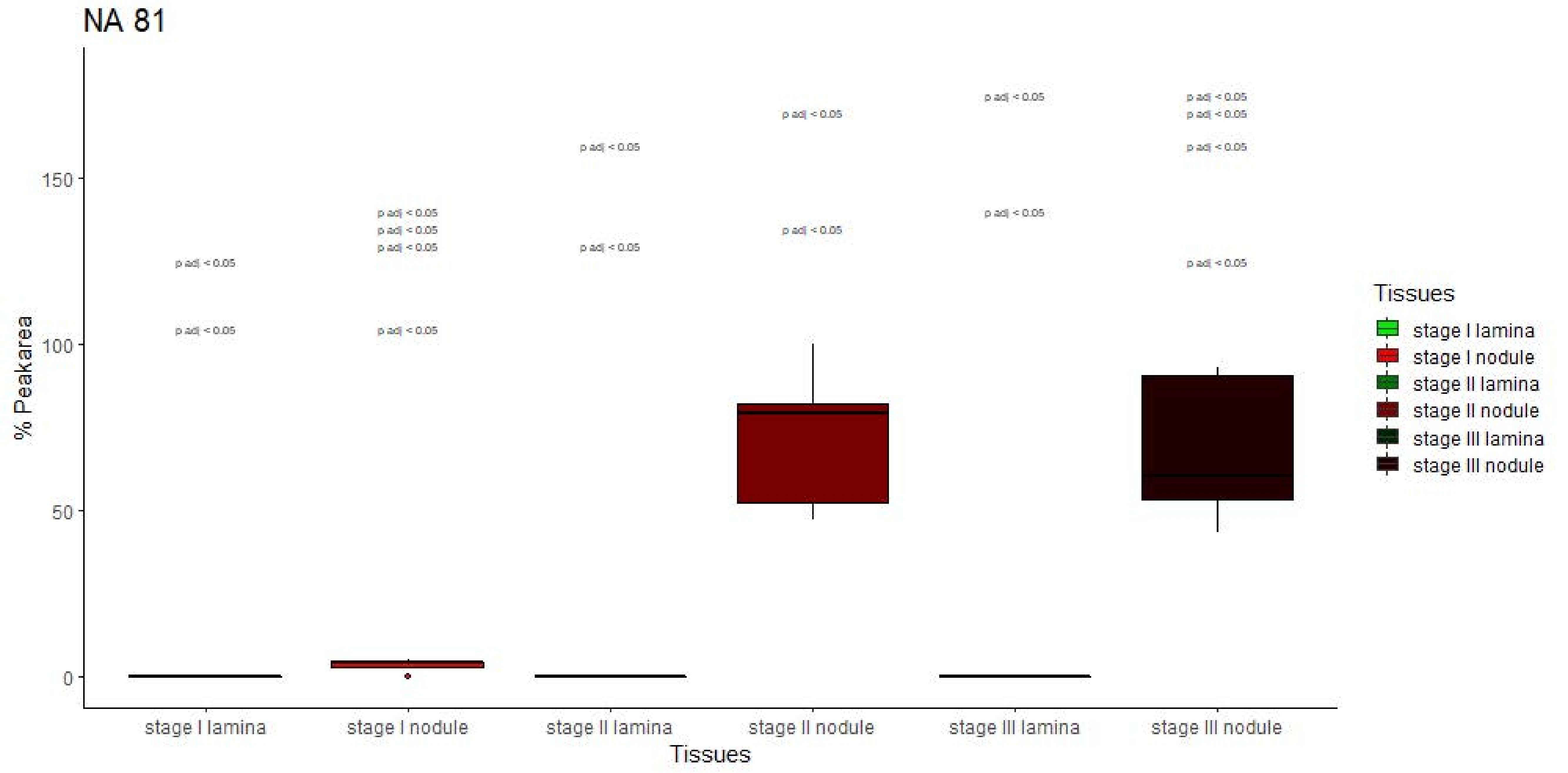

NA 81
% Peakarea
0
50
100
150
p adj < 0.05
Tissues
stage I lamina
stage I nodule
stage II lamina
stage II nodule
stage III lamina
stage III nodule

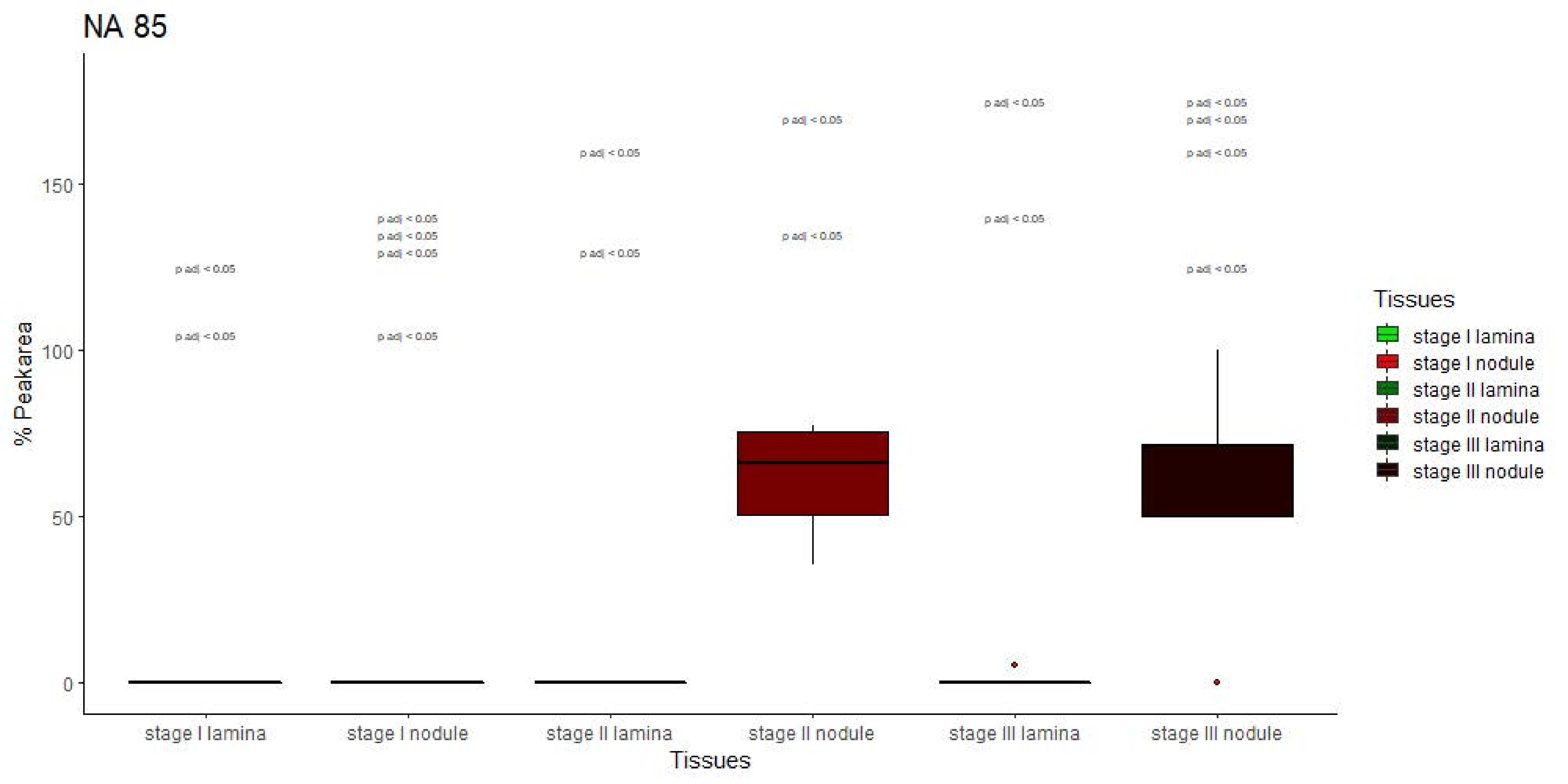

NA 85
% Peakarea
150
100
50
0
p adj < 0.05
Tissues
stage I lamina
stage I nodule
stage II lamina
stage II nodule
stage III lamina
stage III nodule

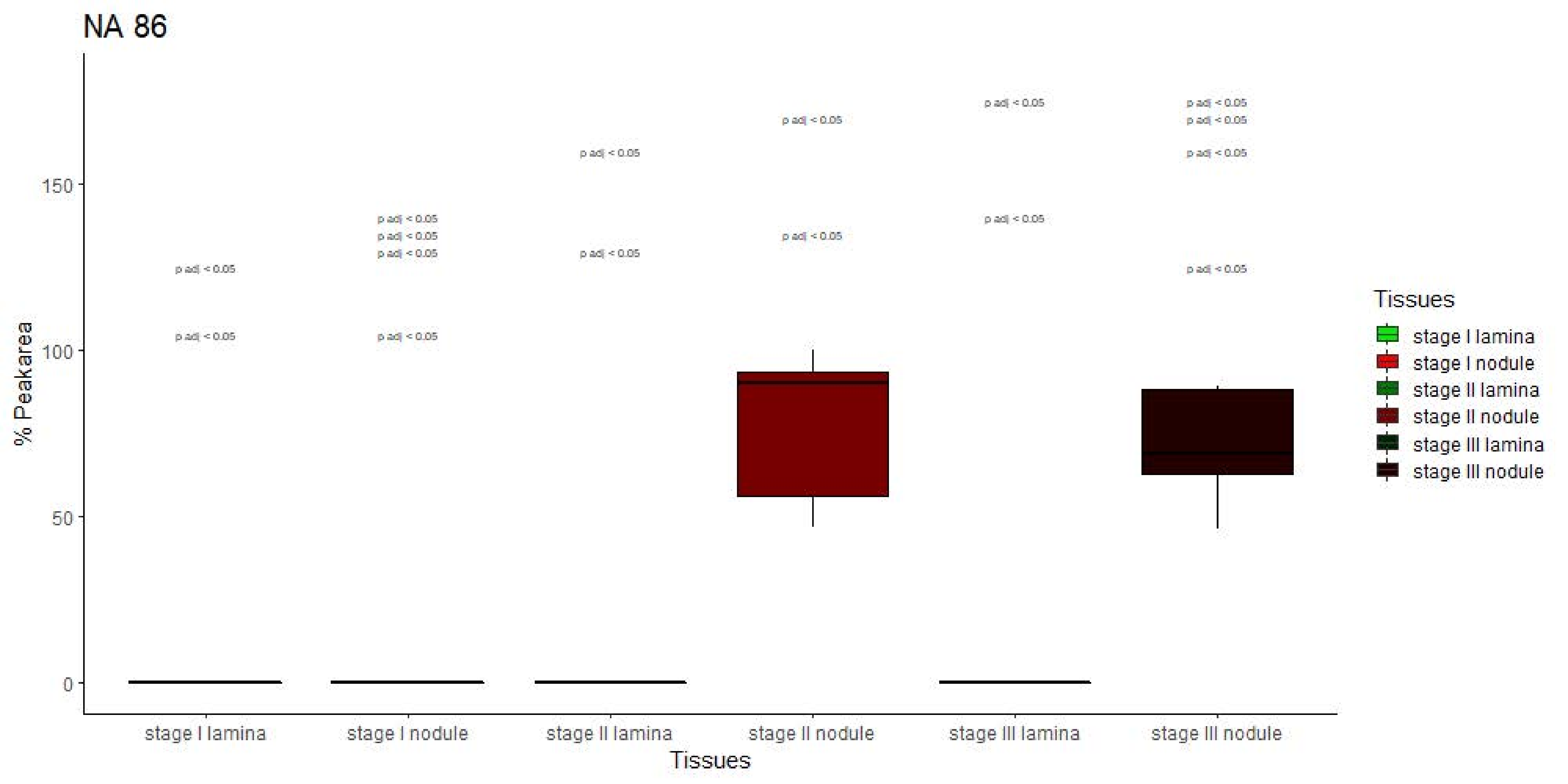
NA 86
% Peakarea
150
100
50
0
p adj < 0.05
Tissues
stage I lamina
stage I nodule
stage II lamina
stage II nodule
stage III lamina
stage III nodule
Tissues
stage I lamina
stage I nodule
stage II lamina
stage II nodule
stage III lamina
stage III nodule

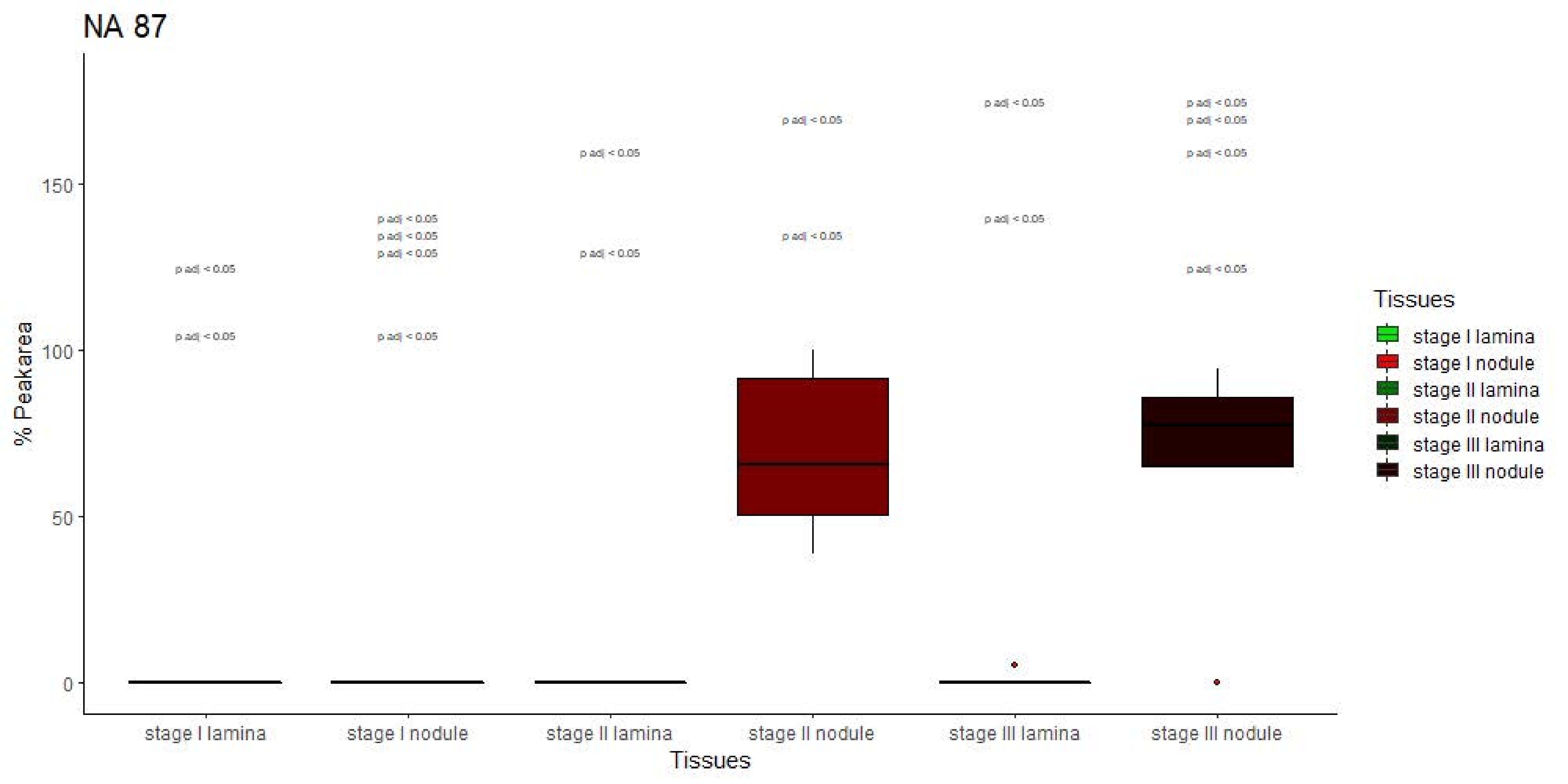

NA 87
% Peakarea
0
50
100
150
p adj < 0.05
Tissues
stage I lamina
stage I nodule
stage II lamina
stage II nodule
stage III lamina
stage III nodule

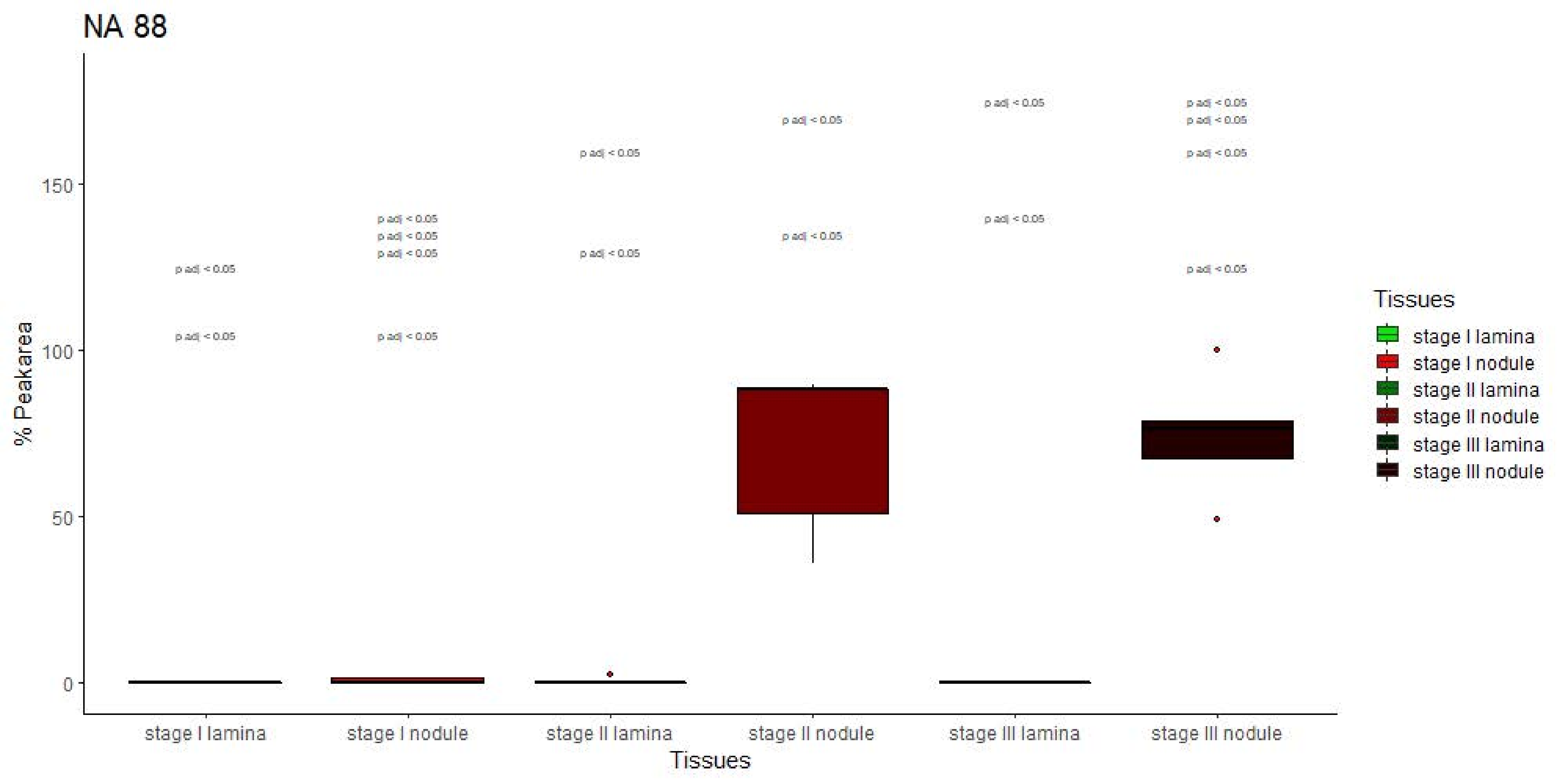
NA 88
% Peakarea
150
100
50
0
p adj < 0.05
stage I lamina
stage I nodule
stage II lamina
stage II nodule
stage III lamina
stage III nodule
Tissues
Tissues
stage I lamina
stage I nodule
stage II lamina
stage II nodule
stage III lamina
stage III nodule

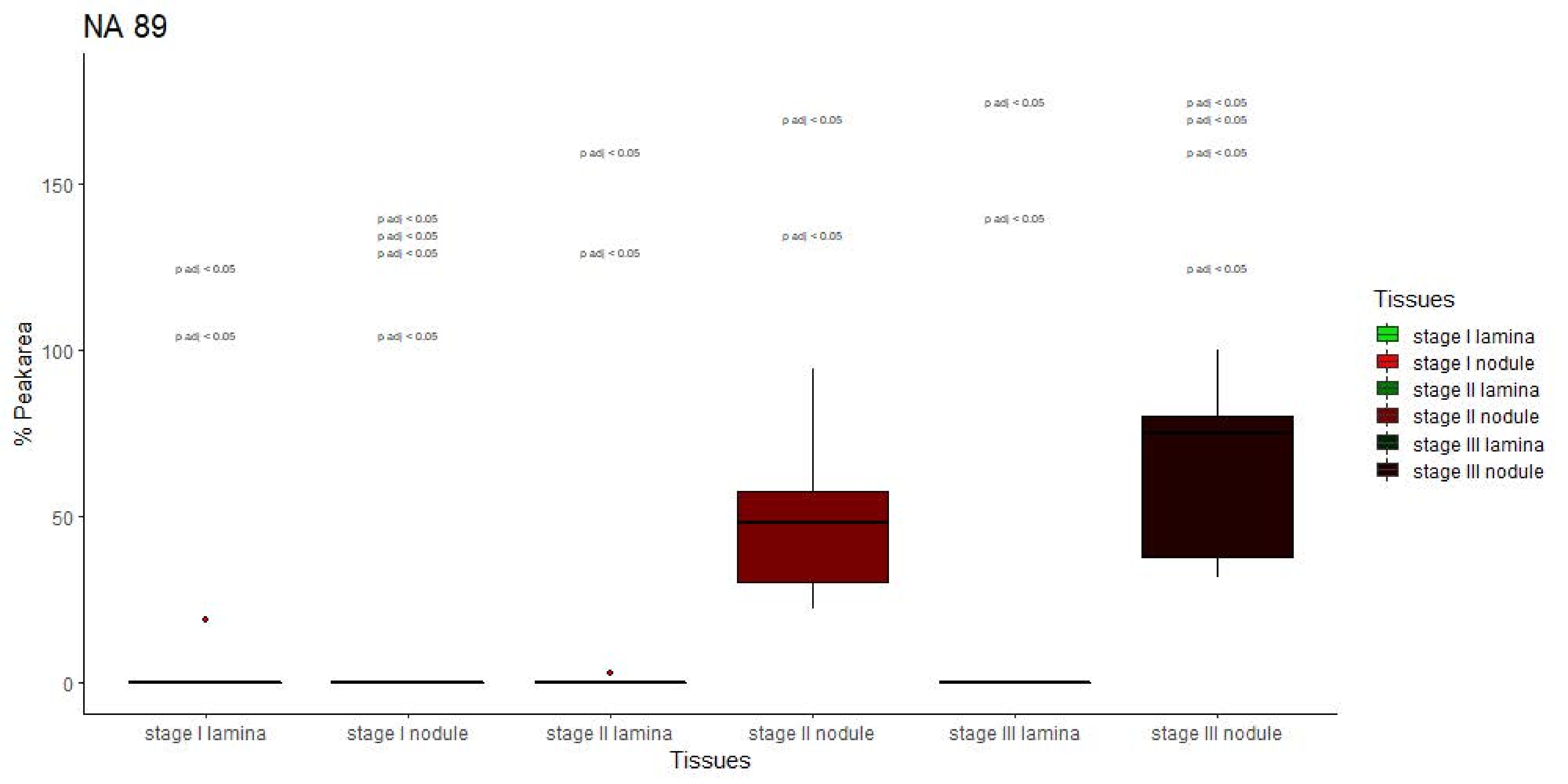

NA 89
% Peakarea
150
100
50
0
p adj < 0.05
stage I lamina
stage I nodule
stage II lamina
stage II nodule
stage III lamina
stage III nodule
Tissues
Tissues
stage I lamina
stage I nodule
stage II lamina
stage II nodule
stage III lamina
stage III nodule

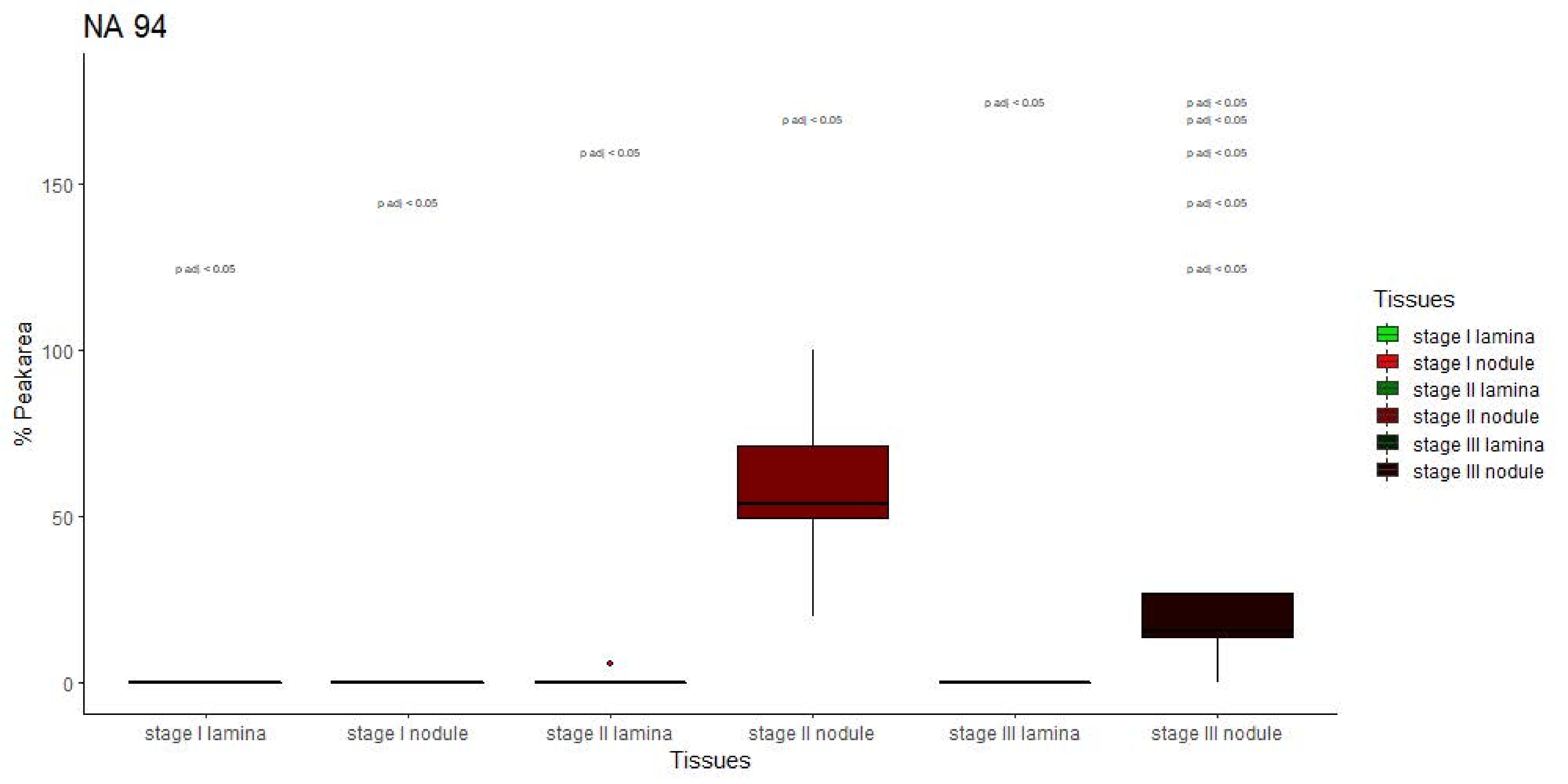

NA 94
p adj < 0.05
p adj < 0.05
p adj < 0.05
p adj < 0.05
p adj < 0.05
p adj < 0.05
p adj < 0.05
p adj < 0.05
p adj < 0.05
p adj < 0.05
150
100
50
0
% Peakarea
stage I lamina
stage I nodule
stage II lamina
stage II nodule
stage III lamina
stage III nodule
Tissues
Tissues
stage I lamina
stage I nodule
stage II lamina
stage II nodule
stage III lamina
stage III nodule
