# Supplementary material for: Dissecting Metabolism of Leaf Nodules in Ardisia crenata and Psychotria punctata
Source: Front Mol Biosci. 2021 Jul 30;8:683671. doi: 10.3389/fmolb.2021.683671 (PMC8362603; doi:10.3389/fmolb.2021.683671)
Supplement: Supplementary file 4 [file Table1.docx]

Content of supplementary material

**Table 1.** Content of supplementary material.

**Table 2.** GC-MS annotations of detected and curated peaks. MSI levels are stated according to Metabolomics Standard Initiative (Goodacre et al., 2007; Sumner et al., 2007).

**Table 3.** LC-MS annotations of detected and curated peaks including, precursor *m/z*, MS2 fragmentation patterns and references of compared literature.

**Table 4.** NMR Spectroscopic data.

**Data Sheet 1.** Manual annotation of LC-MS fragmentation spectra including, precursor *m/z*, retention time, MS2 and MS3 fragmentation patterns.

**Data Sheet 2.** Boxplots of annotated peaks in samples of *Ardisia crenata* detected by GC-MS and LC-MS approaches. Statistically significant changes between two conditions are indicated by text markers p<0.05 stated in the same height.

**Data Sheet 3.** Boxplots of annotated peaks in samples of *Psychotria punctata* detected by GC-MS and LC-MS approaches. Statistically significant changes between two conditions are indicated by text markers p<0.05 stated in the same height.

**Image 1.** Heatmap of a hierarchical bicluster analysis of all statistically significant changed metabolites of *Ardisia crenata* tissue extracts. The columns correspond to the five biological replicates of each condition.

**Image 2.** Heatmap of a hierarchical bicluster analysis of all statistically significant changed metabolites of *Psychotria punctata* tissue extracts. The columns correspond to the five biological replicates of each condition.
