# Supplementary figures and images for: Dissecting Metabolism of Leaf Nodules in Ardisia crenata and Psychotria punctata
Source: Front Mol Biosci. 2021 Jul 30;8:683671. doi: 10.3389/fmolb.2021.683671 (PMC8362603; doi:10.3389/fmolb.2021.683671)

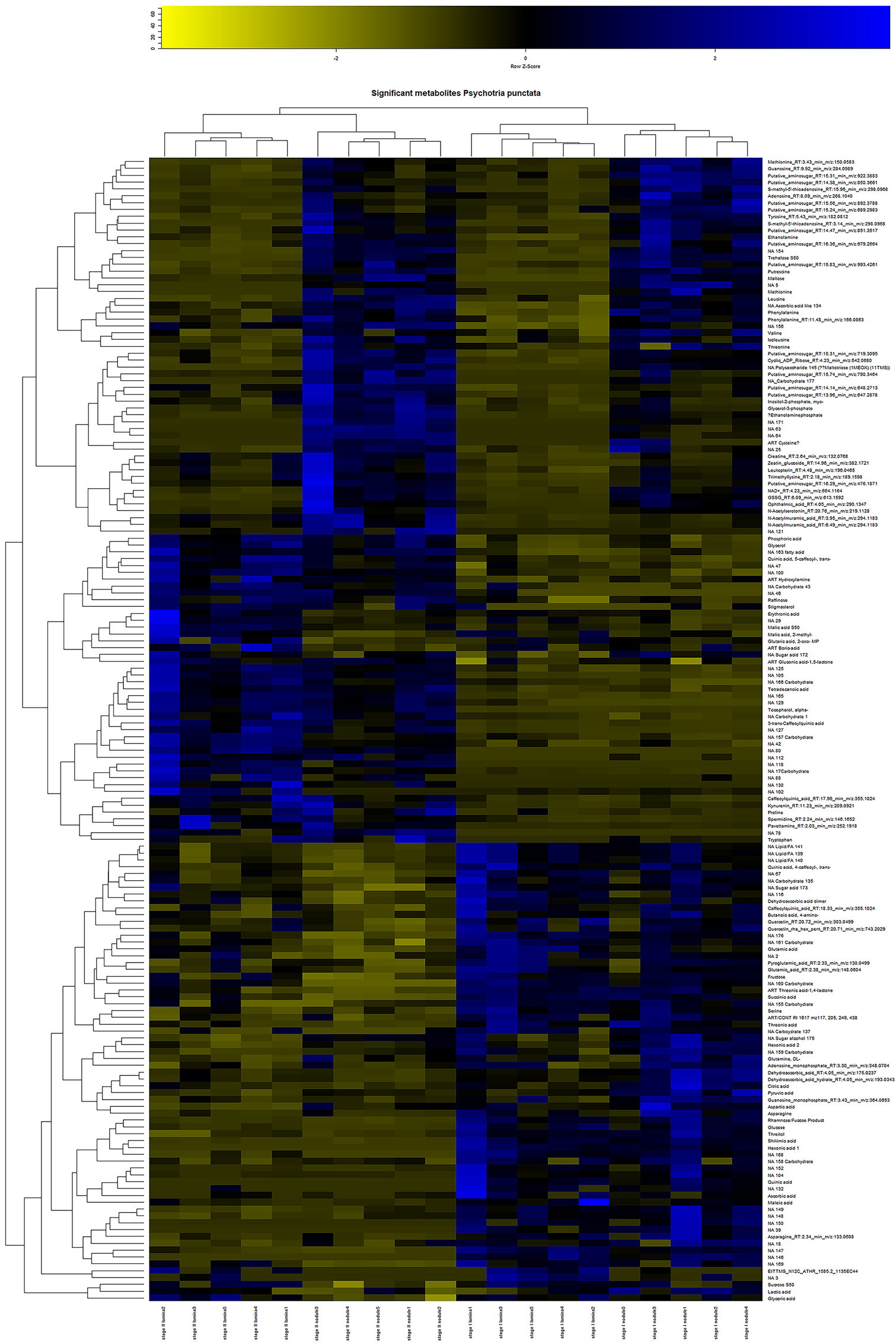

Supplement: Supplementary file 5 [file Image2.tif]

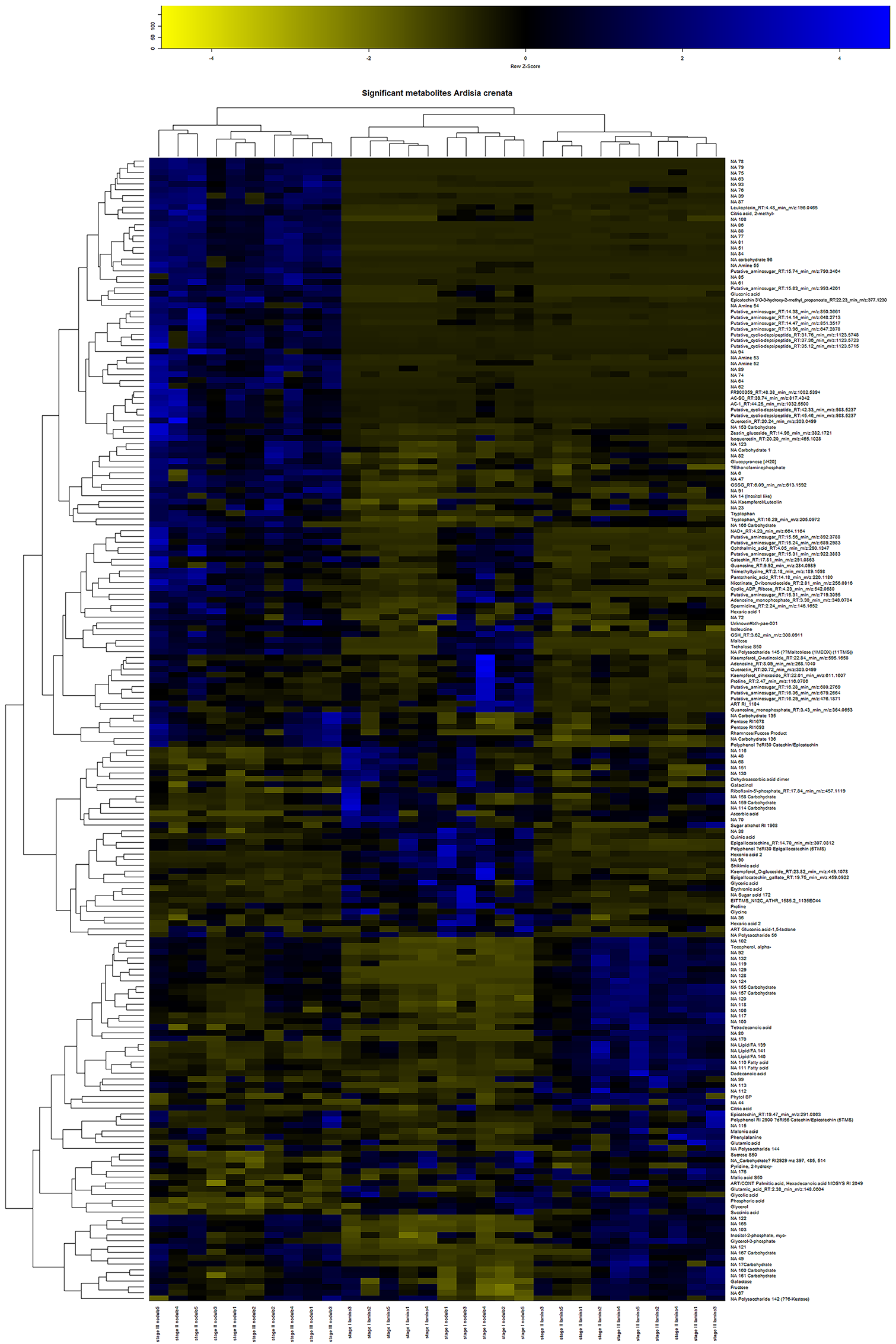

Supplement: Supplementary file 6 [file Image1.tif]
