# Supplementary material for: Dissecting Metabolism of Leaf Nodules in Ardisia crenata and Psychotria punctata
Source: Front Mol Biosci. 2021 Jul 30;8:683671. doi: 10.3389/fmolb.2021.683671 (PMC8362603; doi:10.3389/fmolb.2021.683671)
Supplement: Supplementary file 7 [file DataSheet3.pdf]

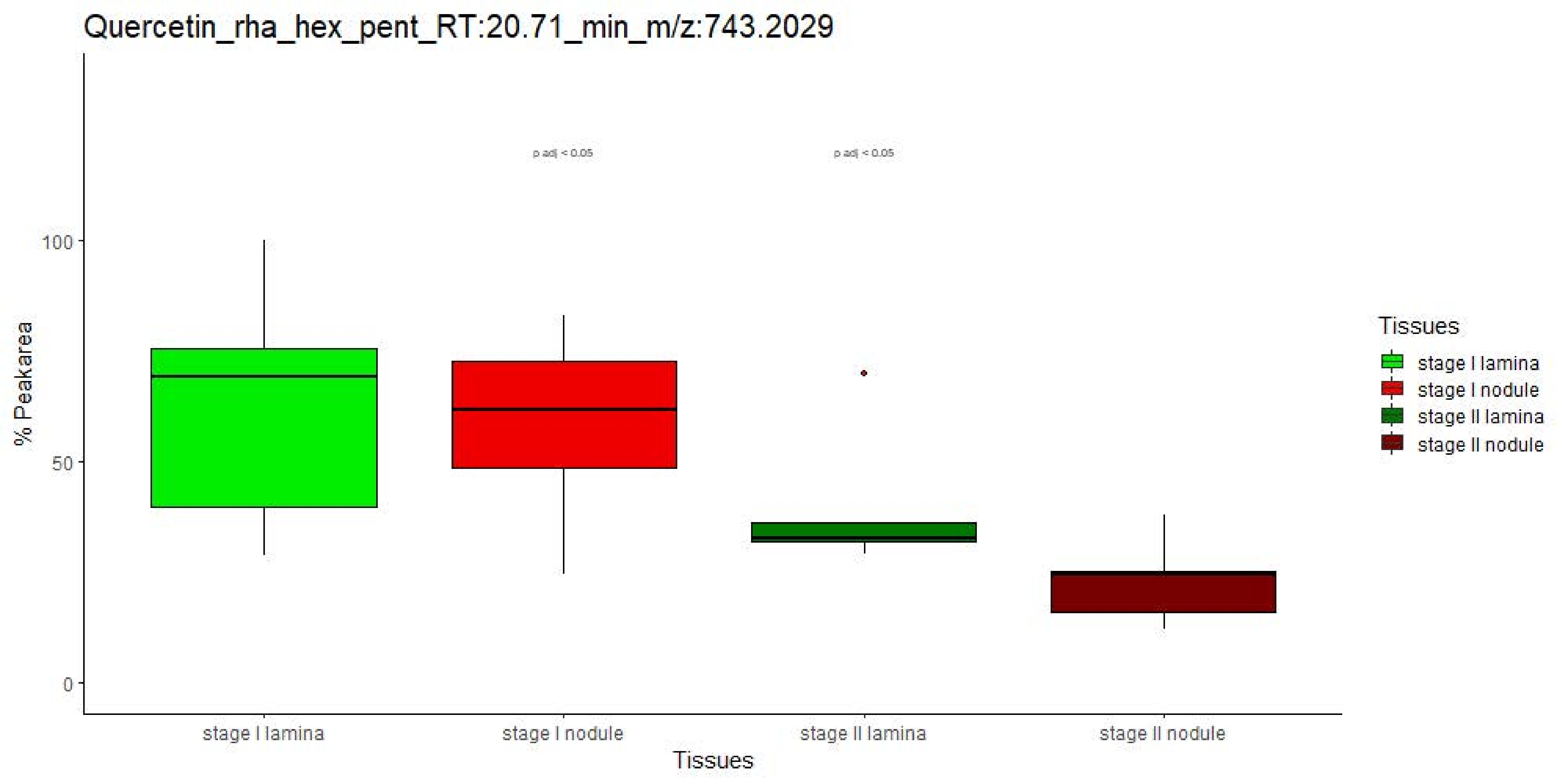

Quercetin_rha_hex_pent_RT:20.71_min_m/z:743.2029
p adj < 0.05
p adj < 0.05
100
50
0
% Peakarea
stage I lamina
stage I nodule
stage II lamina
stage II nodule
Tissues
Tissues
stage I lamina
stage I nodule
stage II lamina
stage II nodule

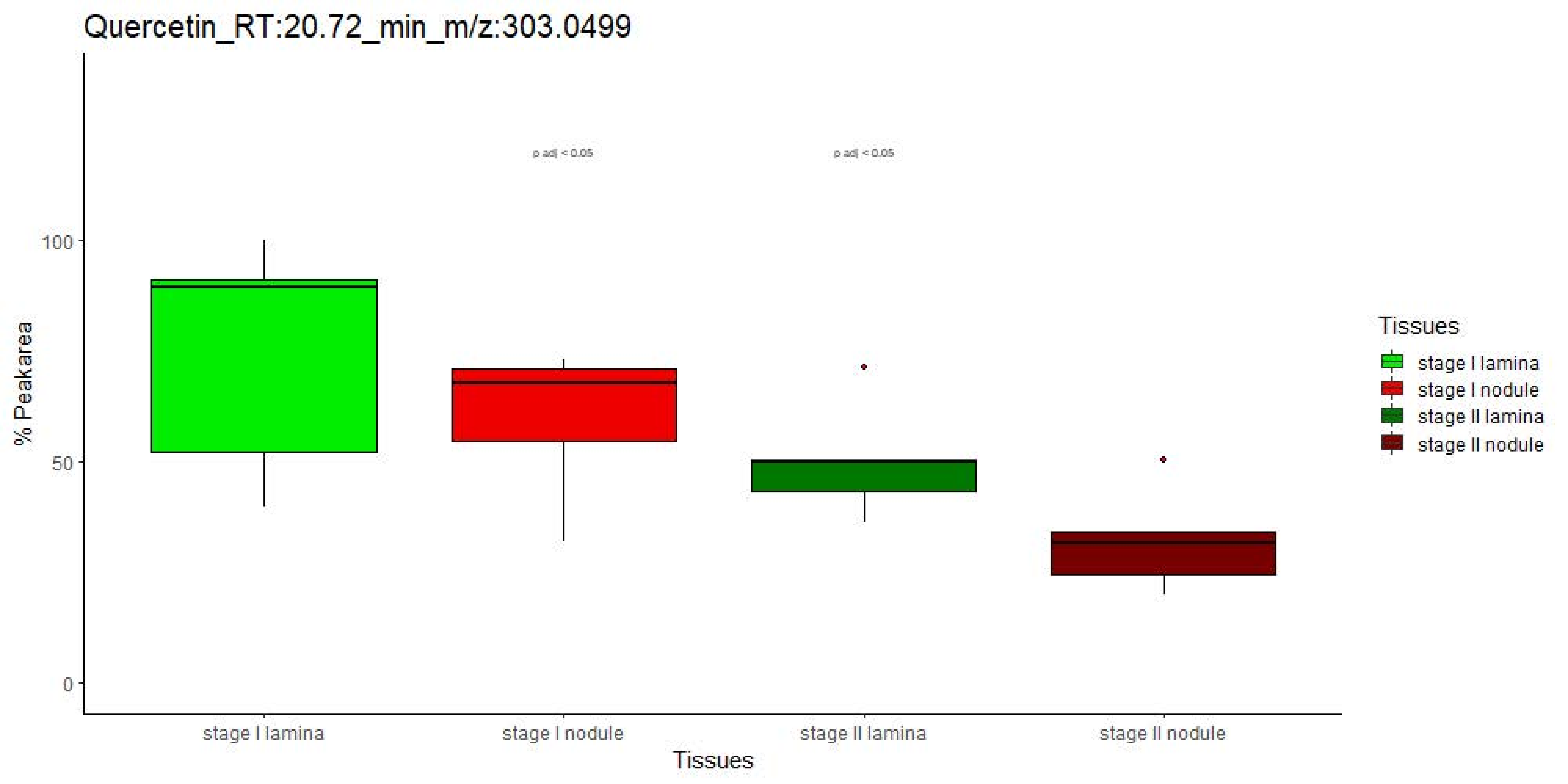

Quercetin_RT:20.72_min_m/z:303.0499
p adj < 0.05
p adj < 0.05
100
50
0
% Peakarea
stage I lamina
stage I nodule
stage II lamina
stage II nodule
Tissues
Tissues
stage I lamina
stage I nodule
stage II lamina
stage II nodule

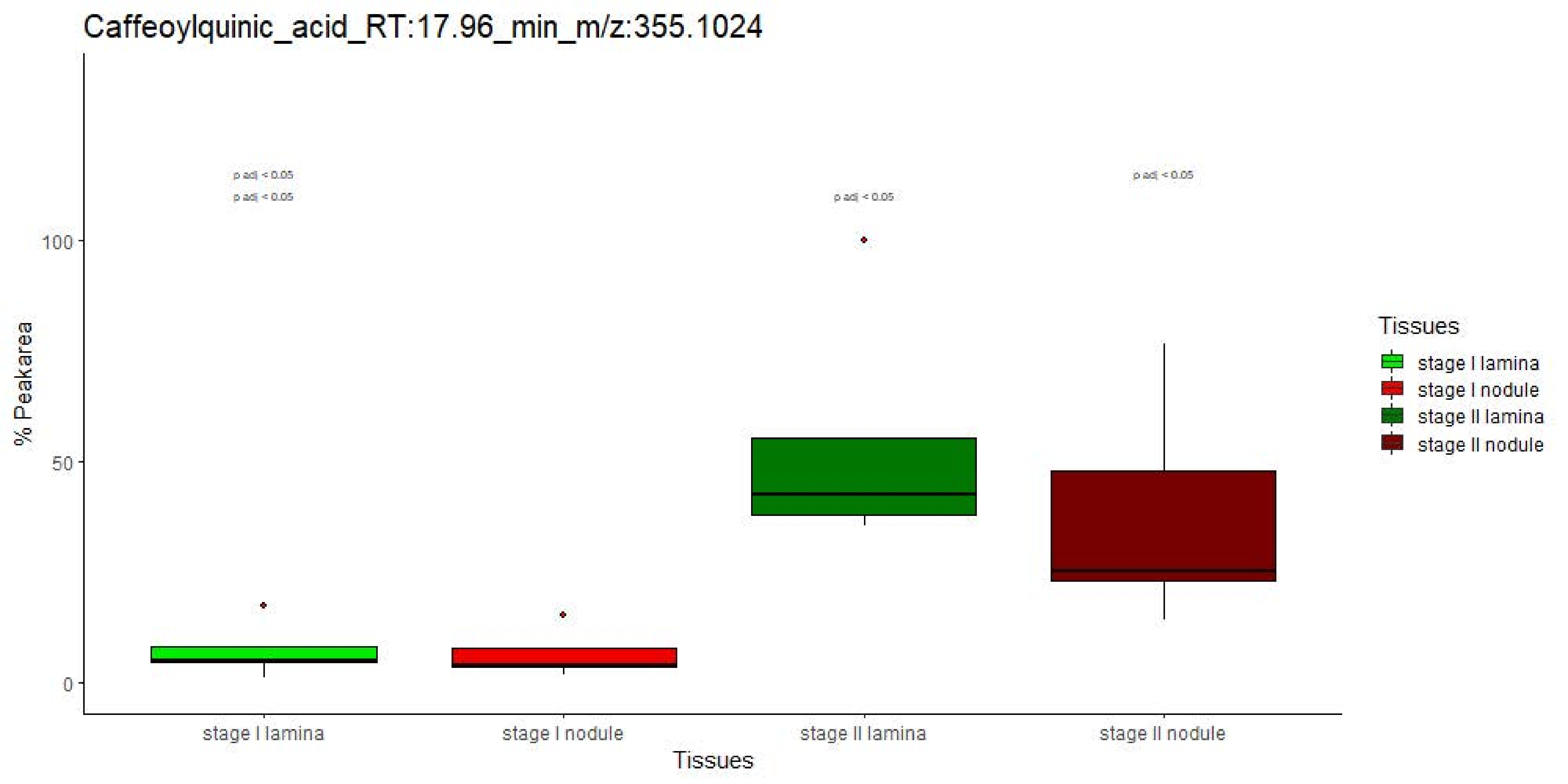

Caffeoylquinic_acid_RT:17.96_min_m/z:355.1024
p adj < 0.05
p adj < 0.05
p adj < 0.05
p adj < 0.05
100
50
0
% Peakarea
stage I lamina
stage I nodule
stage II lamina
stage II nodule
Tissues
Tissues
stage I lamina
stage I nodule
stage II lamina
stage II nodule

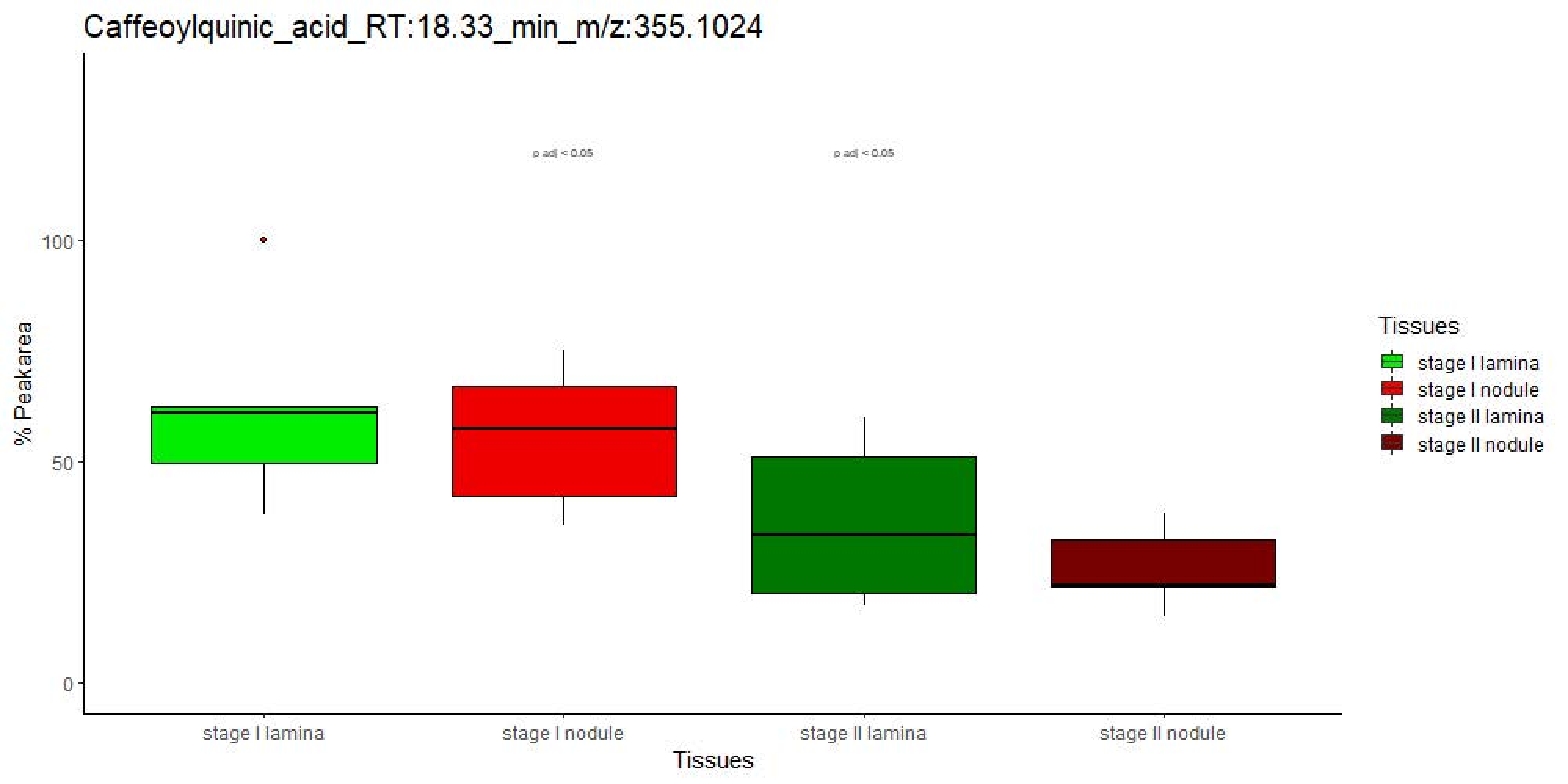

Caffeoylquinic_acid_RT:18.33_min_m/z:355.1024
p adj < 0.05
p adj < 0.05
100
50
0
% Peakarea
stage I lamina
stage I nodule
stage II lamina
stage II nodule
Tissues
Tissues
stage I lamina
stage I nodule
stage II lamina
stage II nodule

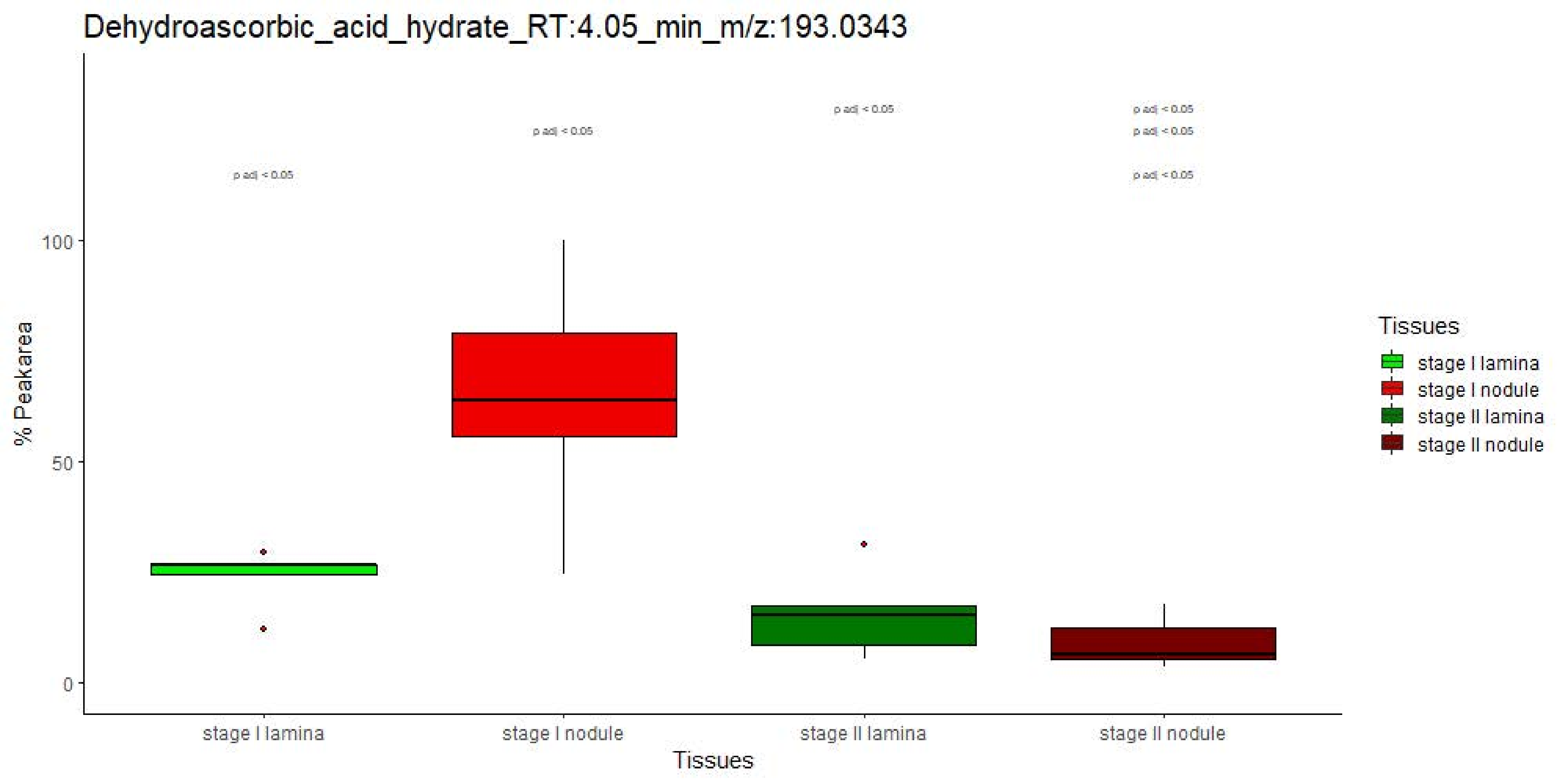
Dehydroascorbic_acid_hydrate_RT:4.05_min_m/z:193.0343
p adj < 0.05
p adj < 0.05
p adj < 0.05
p adj < 0.05
p adj < 0.05
p adj < 0.05
p adj < 0.05
100
50
0
% Peakarea
stage I lamina
stage I nodule
stage II lamina
stage II nodule
Tissues
Tissues
stage I lamina
stage I nodule
stage II lamina
stage II nodule

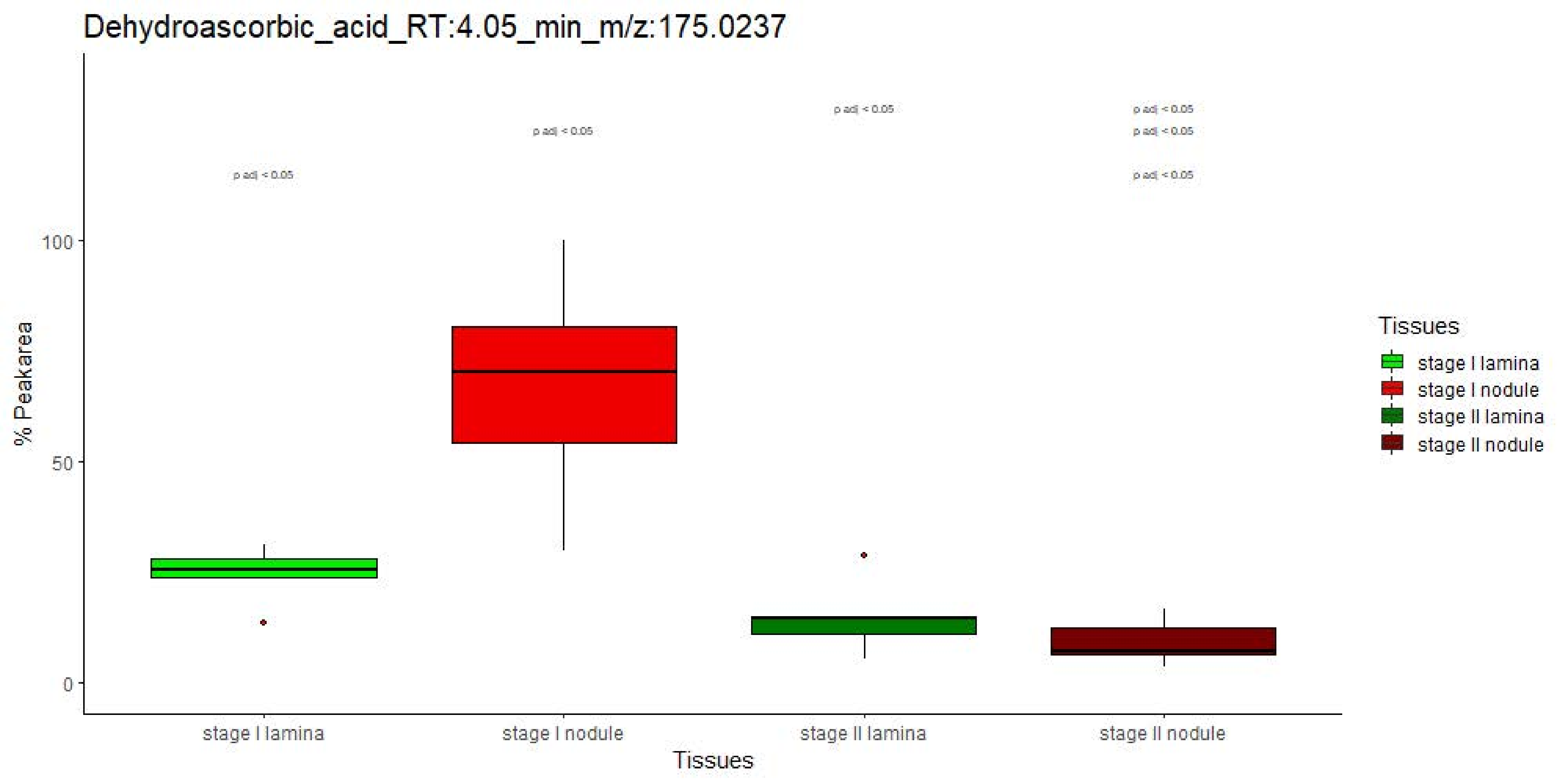

Dehydroascorbic_acid_RT:4.05_min_m/z:175.0237
p adj < 0.05
p adj < 0.05
p adj < 0.05
p adj < 0.05
p adj < 0.05
p adj < 0.05
100
50
0
% Peakarea
stage I lamina
stage I nodule
stage II lamina
stage II nodule
Tissues
Tissues
stage I lamina
stage I nodule
stage II lamina
stage II nodule

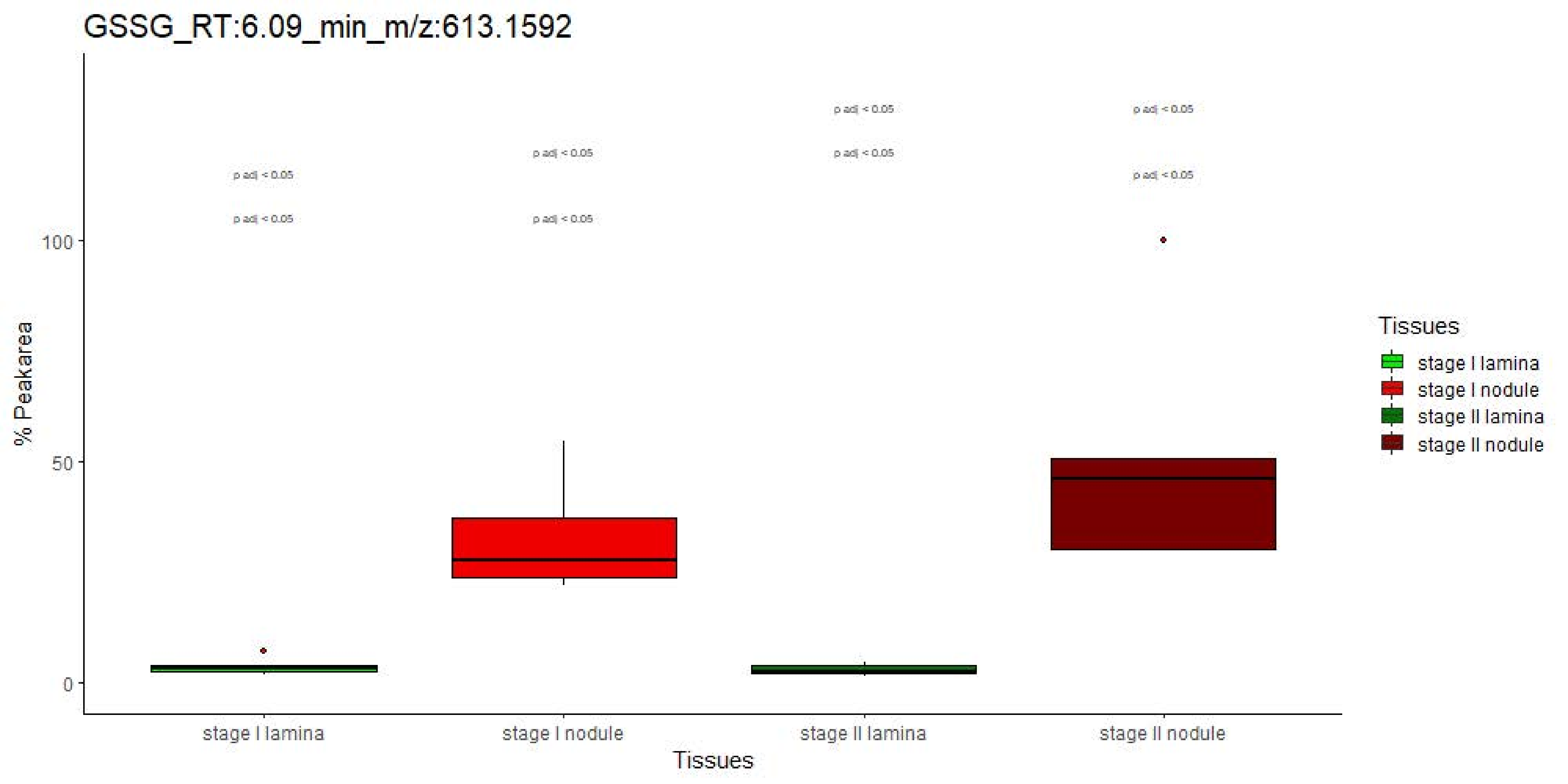
GSSG_RT:6.09_min_m/z:613.1592
% Peakarea
100
50
0
p adj < 0.05
p adj < 0.05
p adj < 0.05
p adj < 0.05
p adj < 0.05
p adj < 0.05
p adj < 0.05
p adj < 0.05
stage I lamina
stage I nodule
stage II lamina
stage II nodule
Tissues
Tissues
stage I lamina
stage I nodule
stage II lamina
stage II nodule

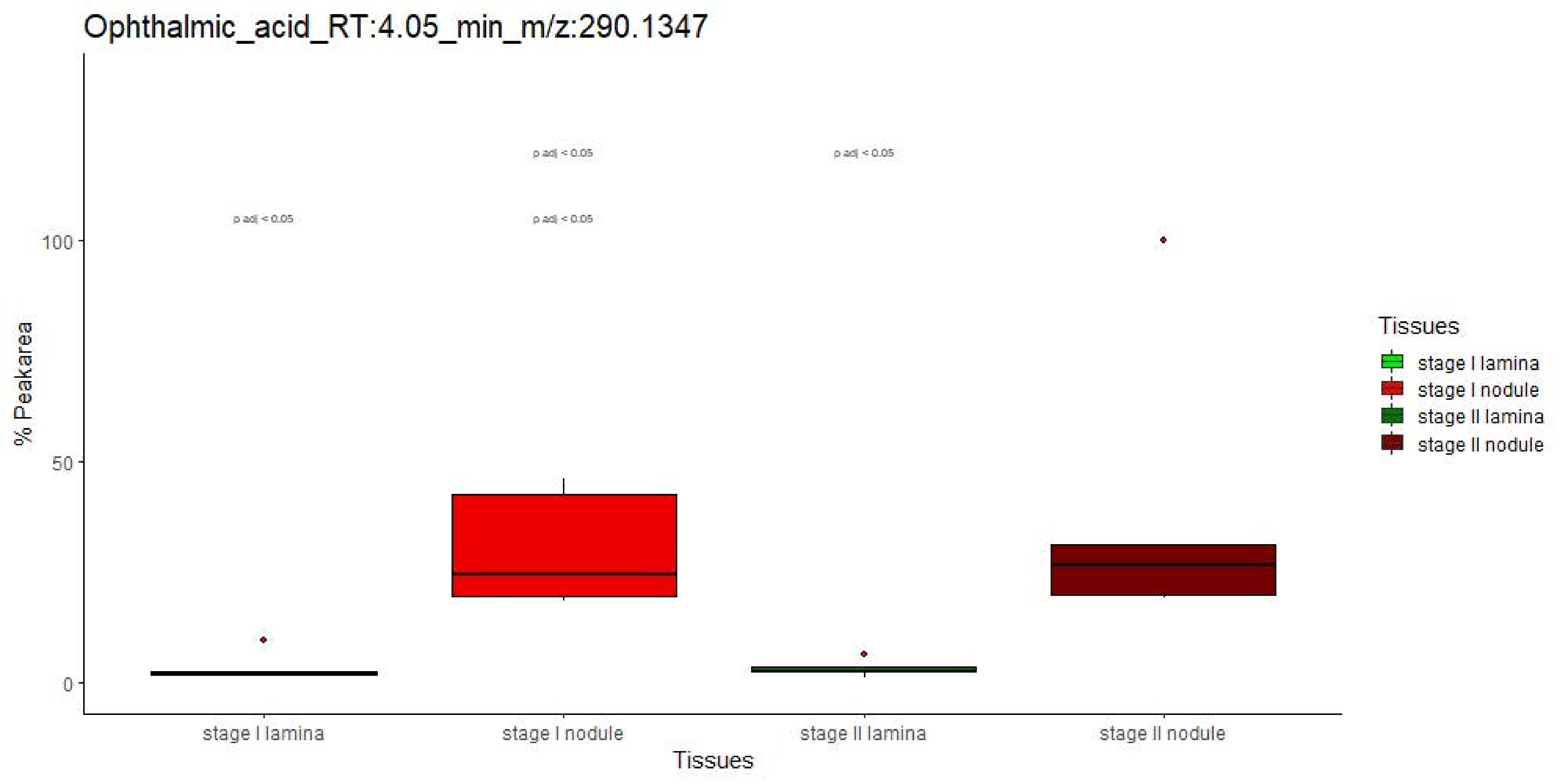

Ophthalmic_acid_RT:4.05_min_m/z:290.1347
% Peakarea
100
50
0
p adj < 0.05
p adj < 0.05
p adj < 0.05
p adj < 0.05
stage I lamina
stage I nodule
stage II lamina
stage II nodule
Tissues
Tissues
stage I lamina
stage I nodule
stage II lamina
stage II nodule

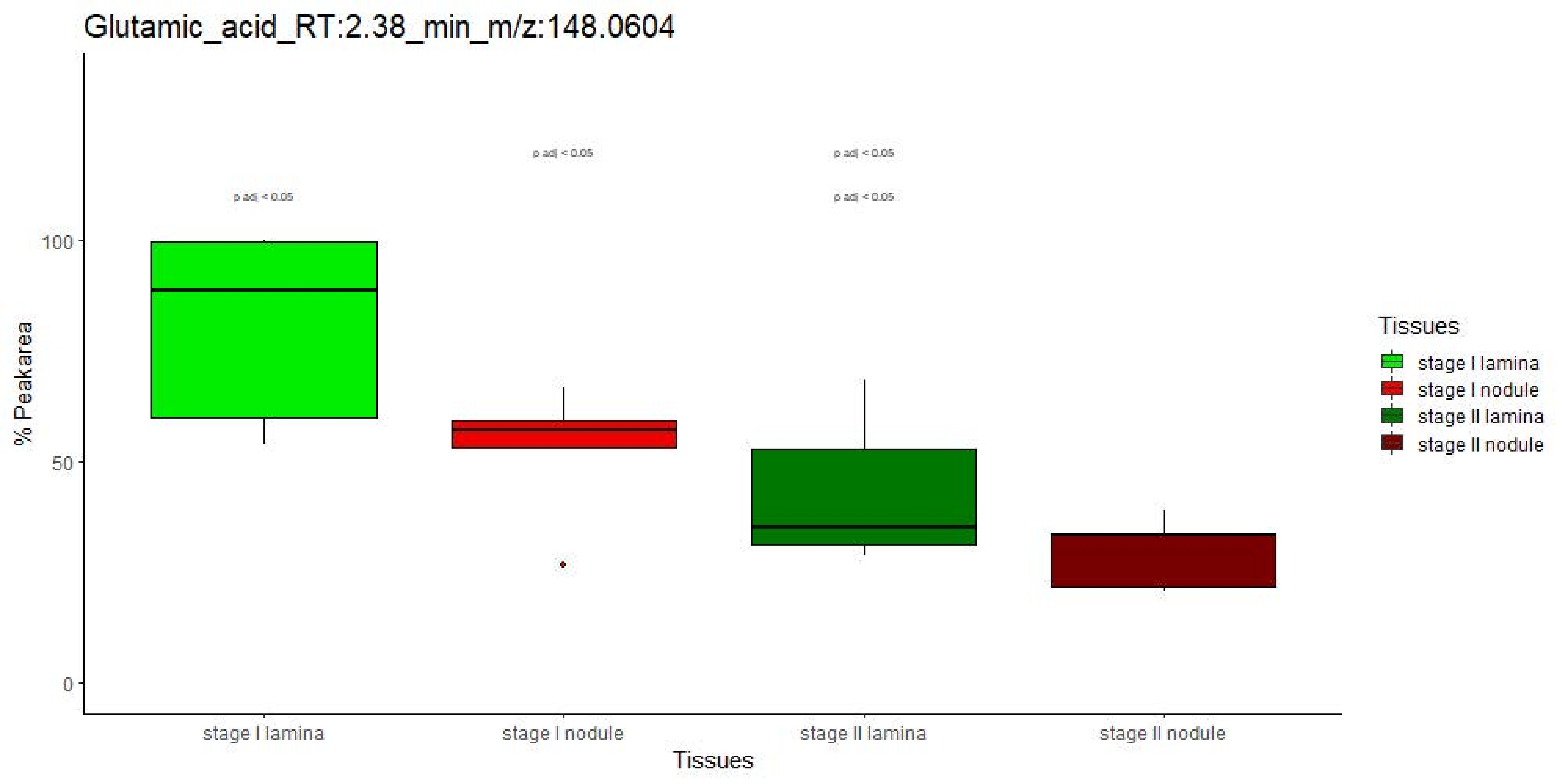
Glutamic_acid_RT:2.38_min_m/z:148.0604
p adj < 0.05
p adj < 0.05
p adj < 0.05
p adj < 0.05
100
50
0
% Peakarea
stage I lamina
stage I nodule
stage II lamina
stage II nodule
Tissues
Tissues
stage I lamina
stage I nodule
stage II lamina
stage II nodule

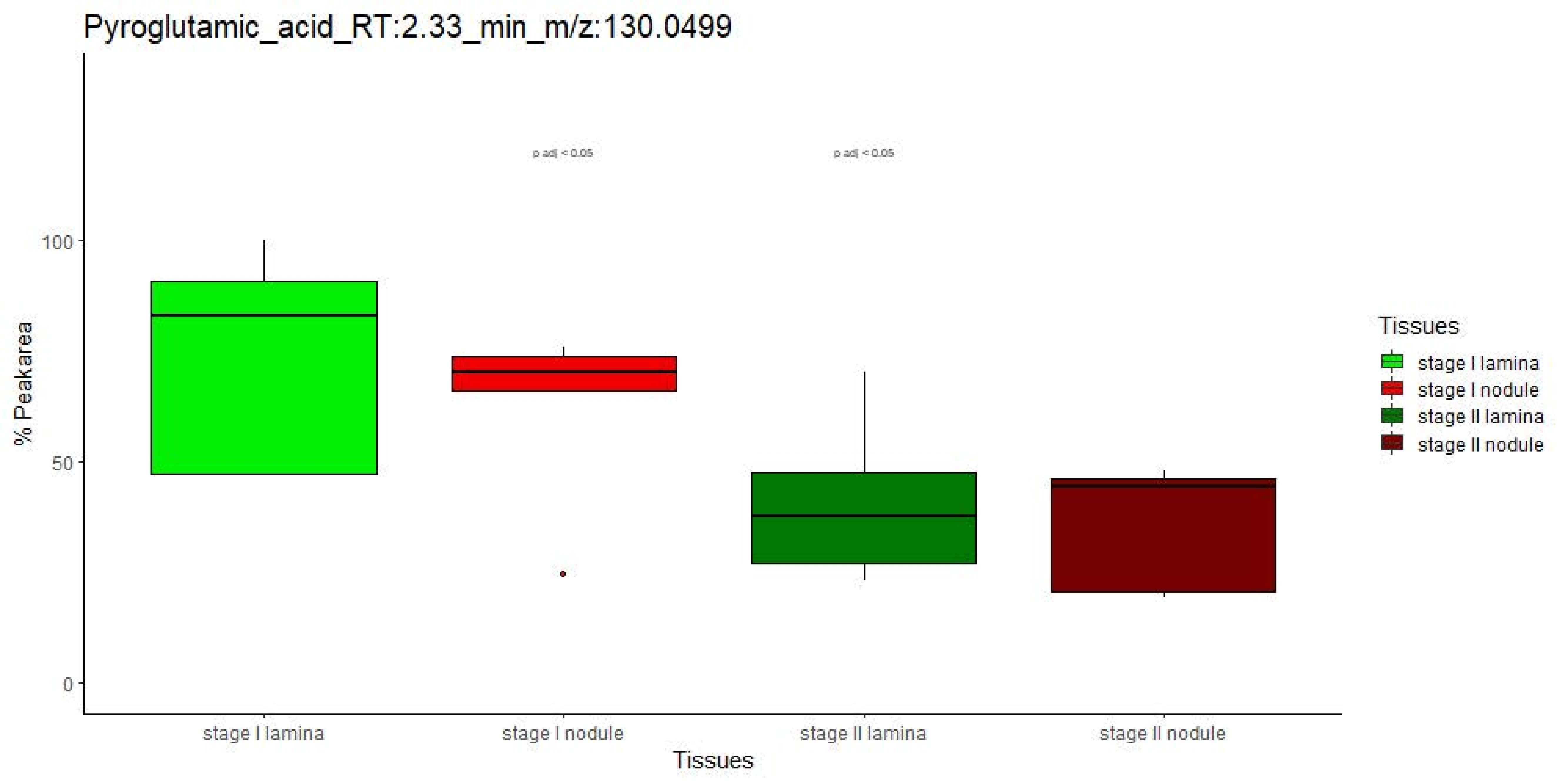

Pyroglutamic_acid_RT:2.33_min_m/z:130.0499
p adj < 0.05
p adj < 0.05
100
50
0
% Peakarea
stage I lamina
stage I nodule
stage II lamina
stage II nodule
Tissues
Tissues
stage I lamina
stage I nodule
stage II lamina
stage II nodule

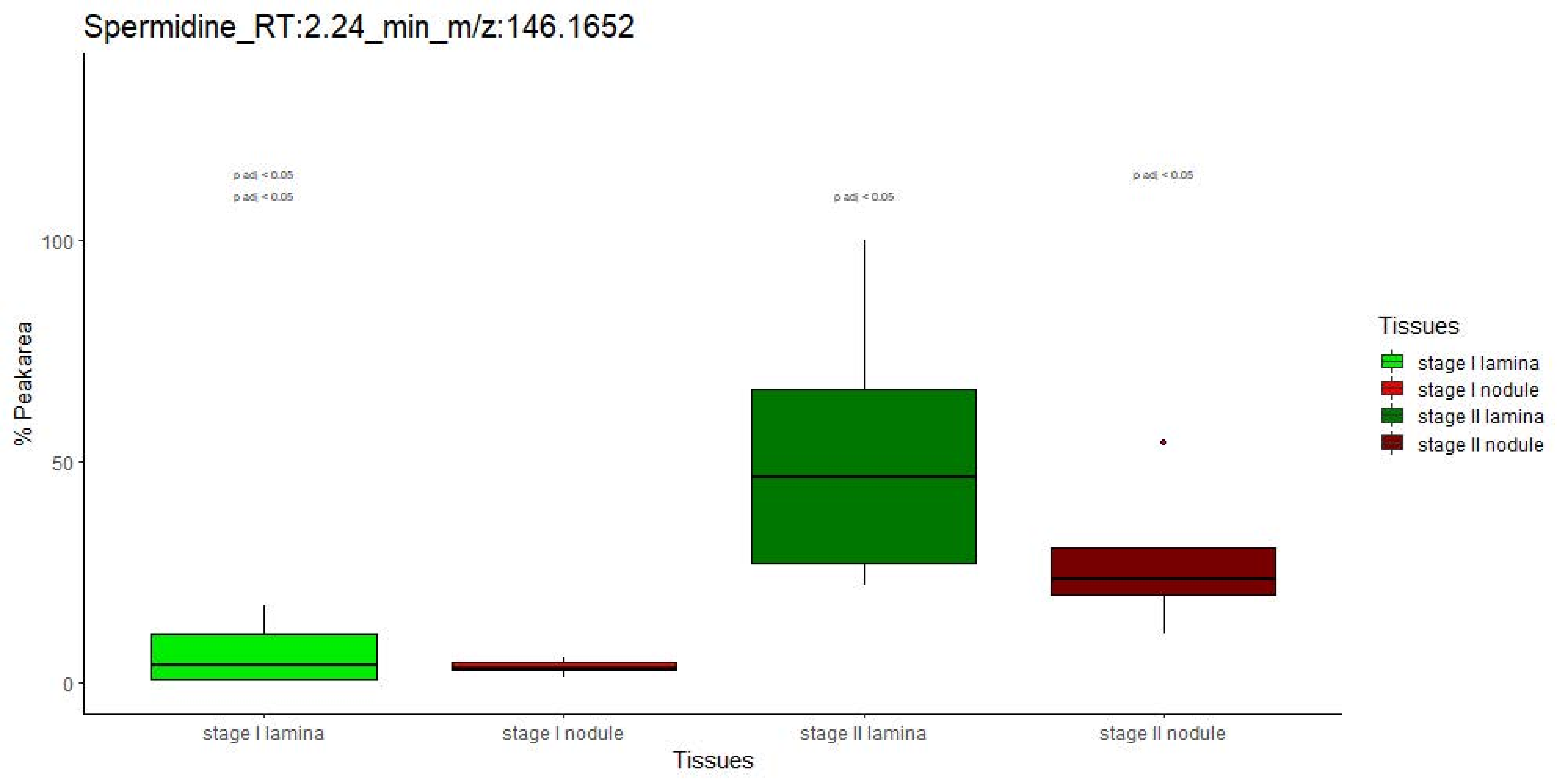

Spermidine_RT:2.24_min_m/z:146.1652
p adj < 0.05
p adj < 0.05
p adj < 0.05
p adj < 0.05
100
50
0
% Peakarea
stage I lamina
stage I nodule
stage II lamina
stage II nodule
Tissues
Tissues
stage I lamina
stage I nodule
stage II lamina
stage II nodule

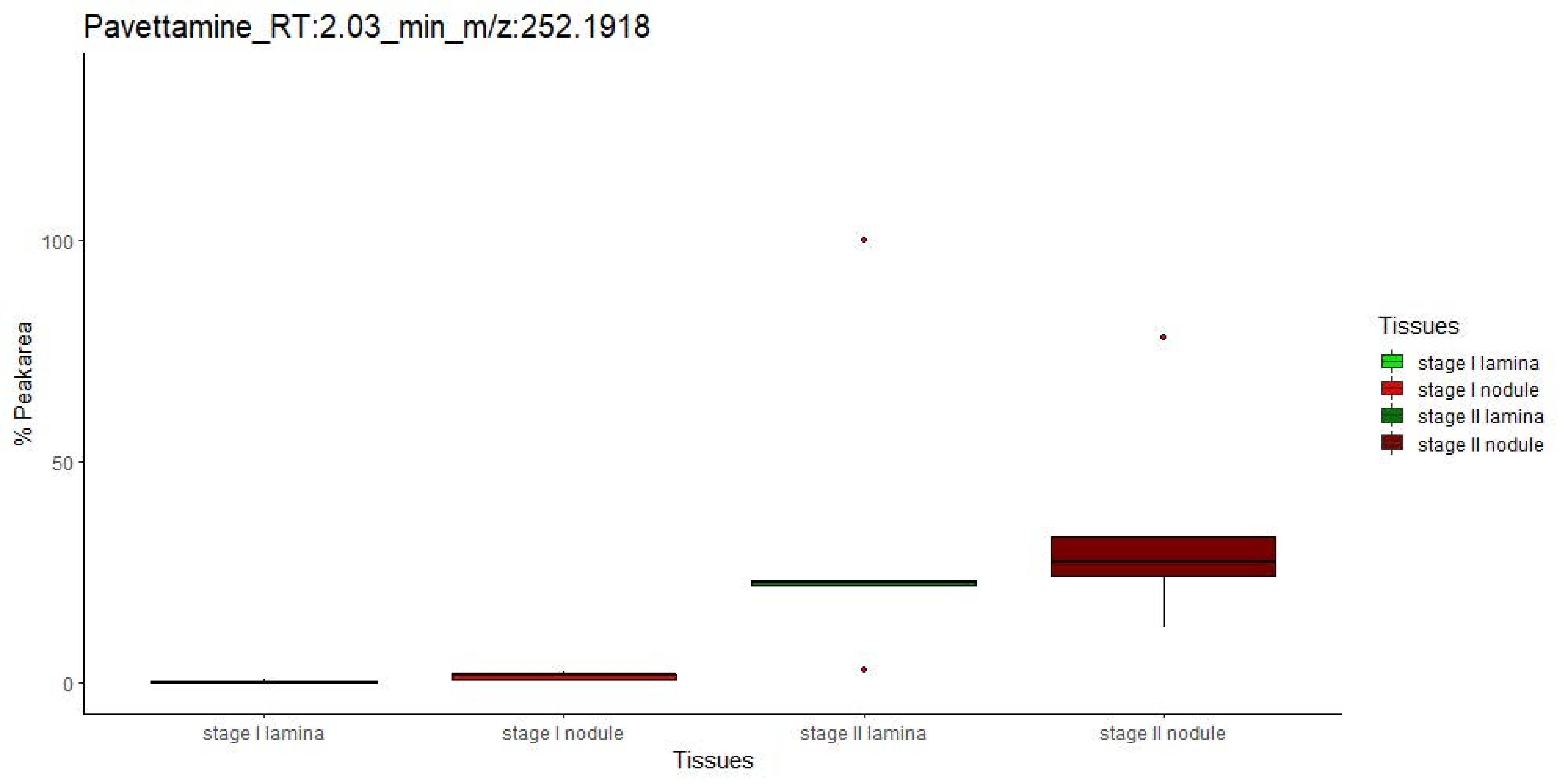
Pavettamine_RT:2.03_min_m/z:252.1918
% Peakarea
100
50
0
stage I lamina
stage I nodule
stage II lamina
stage II nodule
Tissues
Tissues
stage I lamina
stage I nodule
stage II lamina
stage II nodule

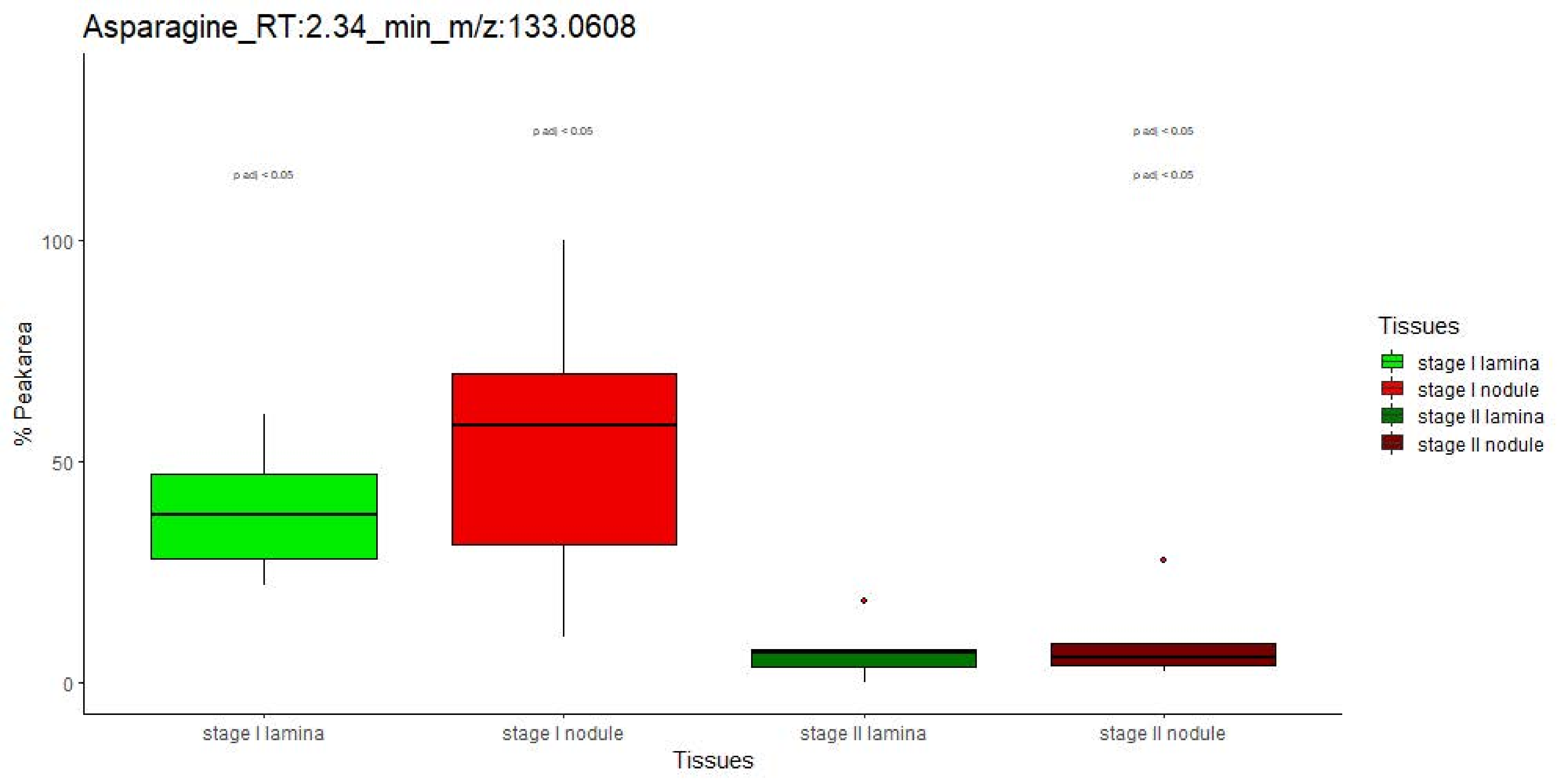

Asparagine_RT:2.34_min_m/z:133.0608
p adj < 0.05
p adj < 0.05
p adj < 0.05
p adj < 0.05
100
50
0
% Peakarea
stage I lamina
stage I nodule
stage II lamina
stage II nodule
Tissues
Tissues
stage I lamina
stage I nodule
stage II lamina
stage II nodule

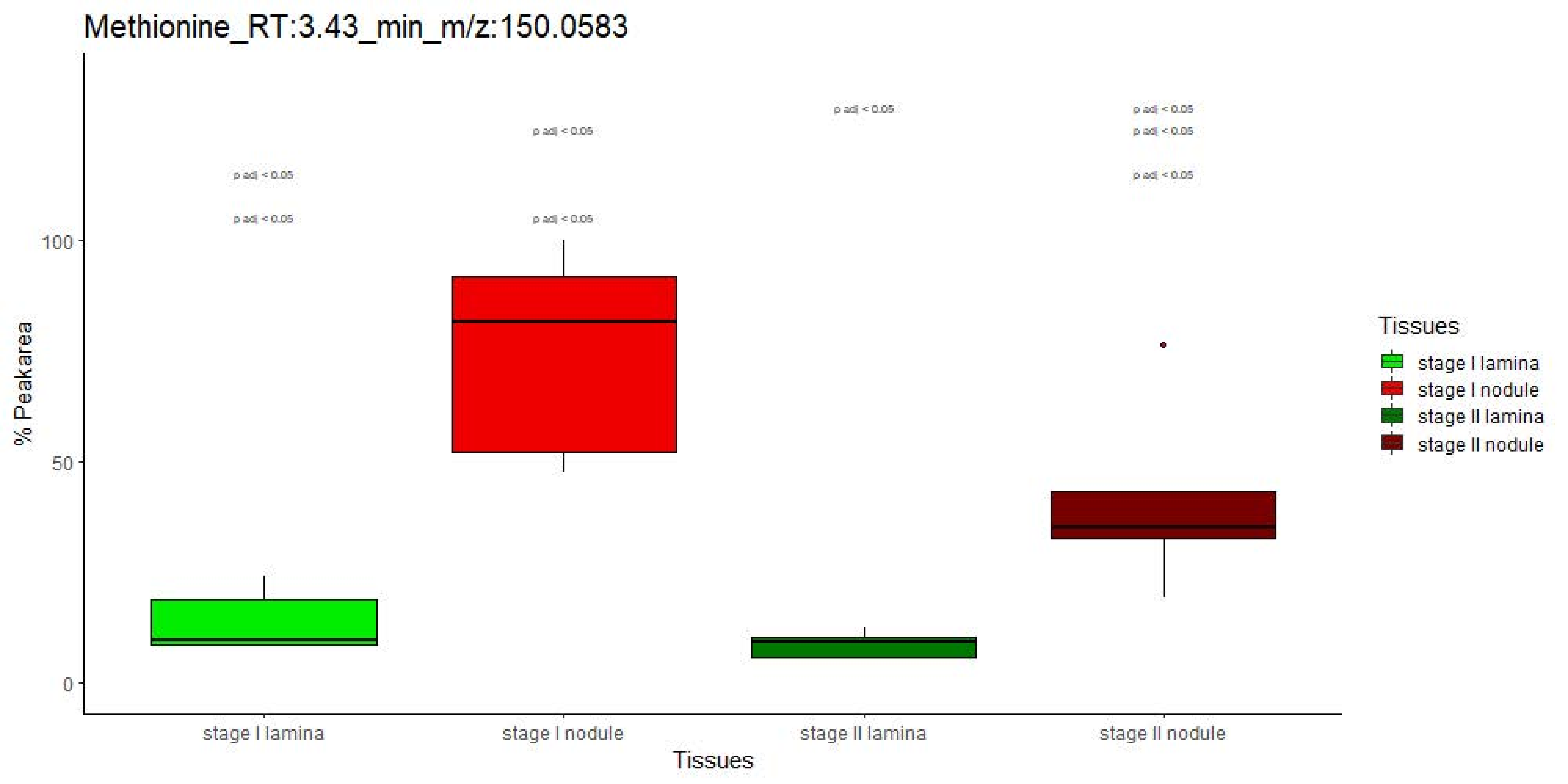

Methionine_RT:3.43_min_m/z:150.0583
% Peakarea
100
50
0
p adj < 0.05
p adj < 0.05
p adj < 0.05
p adj < 0.05
p adj < 0.05
p adj < 0.05
p adj < 0.05
p adj < 0.05
stage I lamina
stage I nodule
stage II lamina
stage II nodule
Tissues
Tissues
stage I lamina
stage I nodule
stage II lamina
stage II nodule

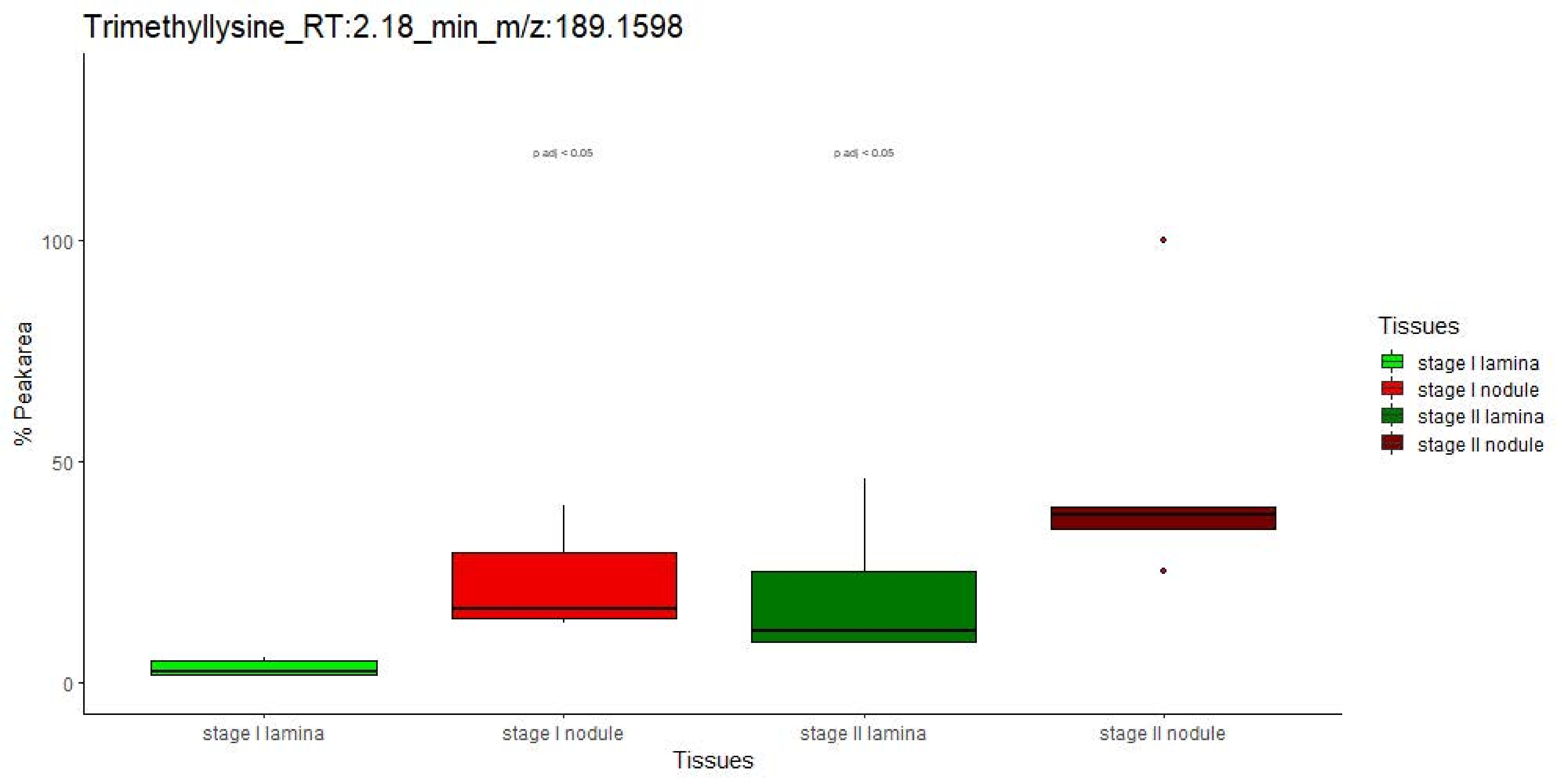

Trimethyllysine_RT:2.18_min_m/z:189.1598
p adj < 0.05
p adj < 0.05
100
50
0
% Peakarea
stage I lamina
stage I nodule
stage II lamina
stage II nodule
Tissues
Tissues
stage I lamina
stage I nodule
stage II lamina
stage II nodule

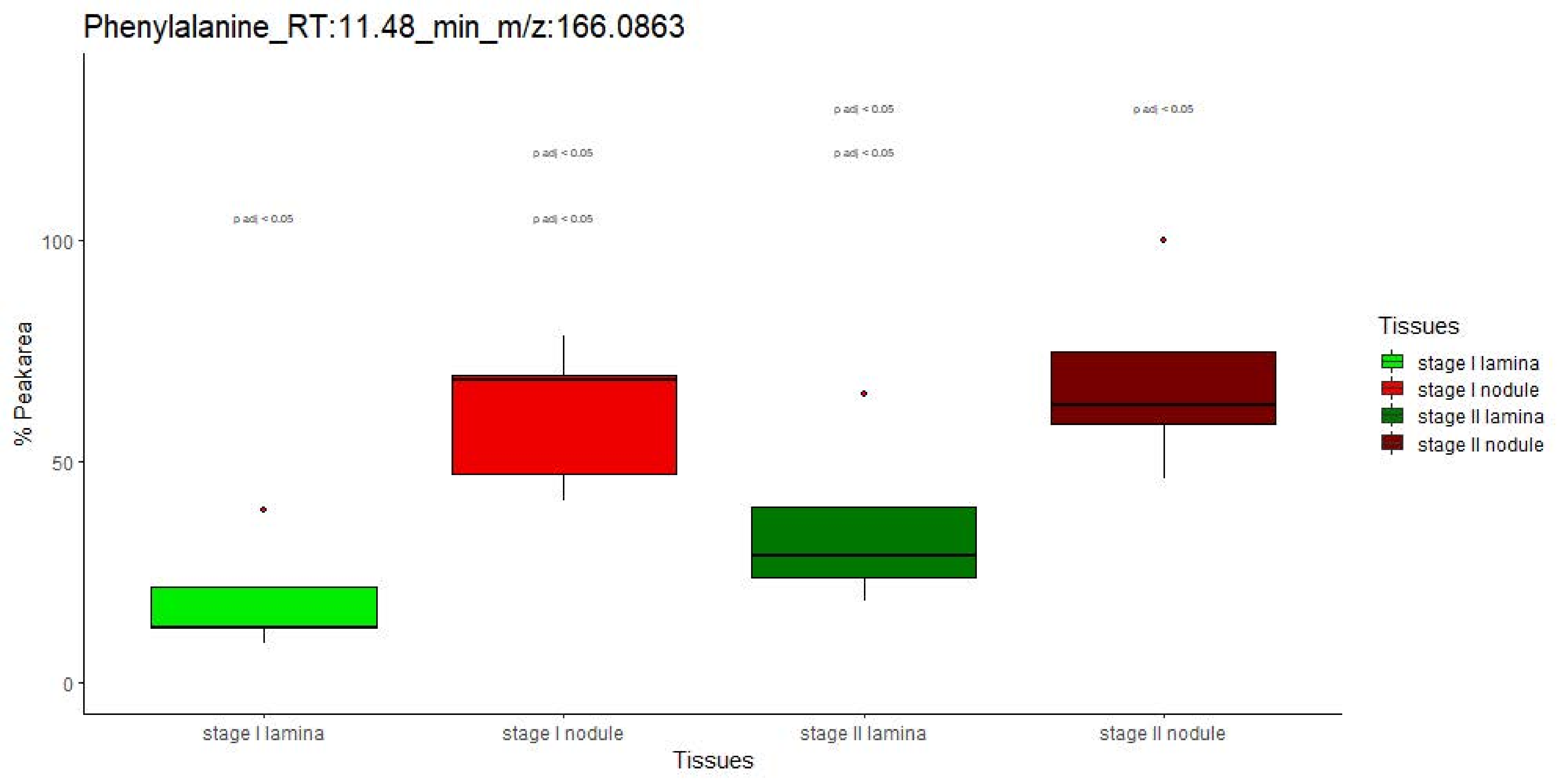

Phenylalanine_RT:11.48_min_m/z:166.0863
p adj < 0.05
p adj < 0.05
p adj < 0.05
p adj < 0.05
p adj < 0.05
p adj < 0.05
p adj < 0.05
100
50
0
% Peakarea
stage I lamina
stage I nodule
stage II lamina
stage II nodule
Tissues
Tissues
stage I lamina
stage I nodule
stage II lamina
stage II nodule

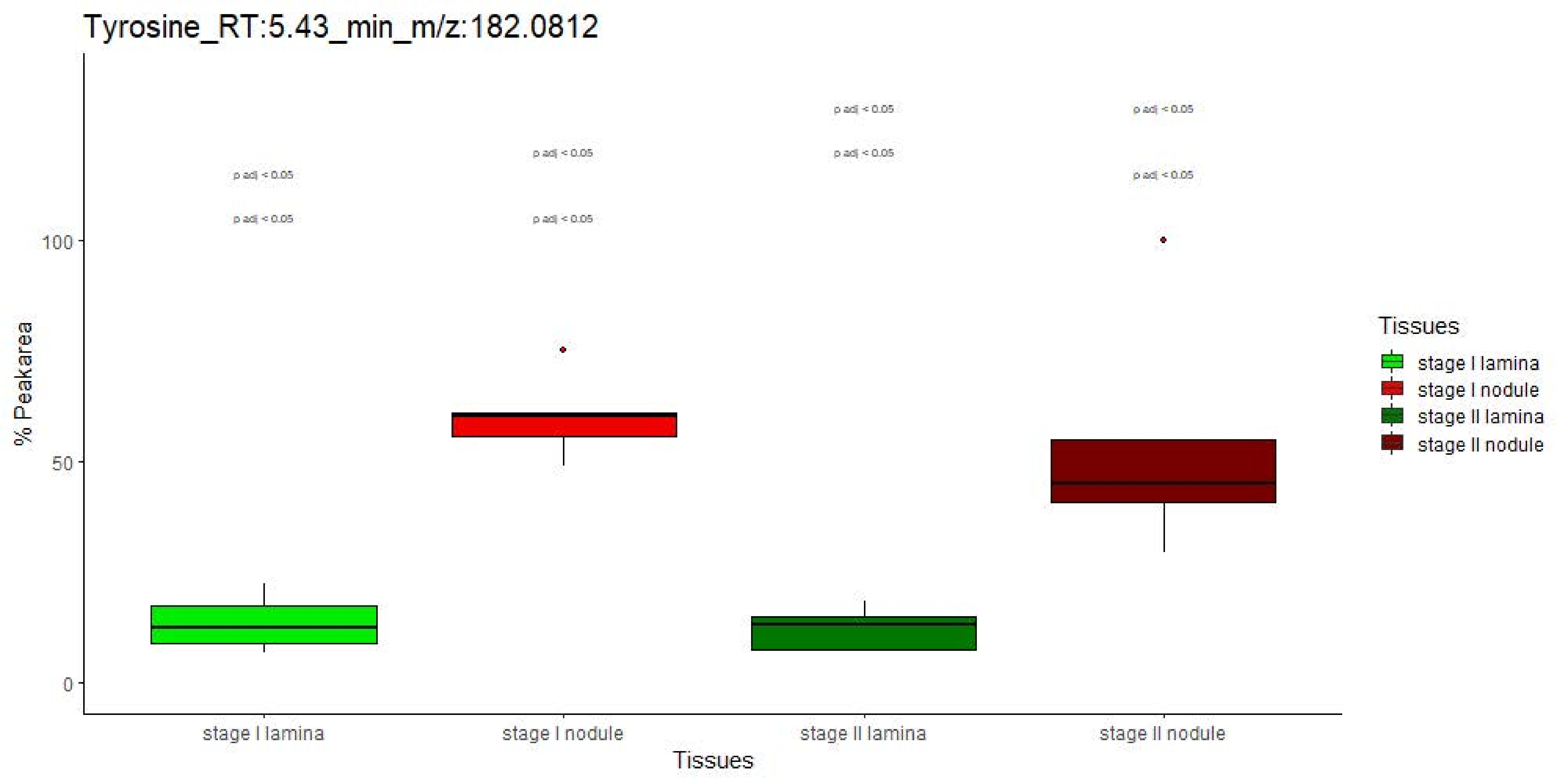

Tyrosine_RT:5.43_min_m/z:182.0812
p adj < 0.05
p adj < 0.05
p adj < 0.05
p adj < 0.05
p adj < 0.05
p adj < 0.05
p adj < 0.05
p adj < 0.05
100
50
0
% Peakarea
stage I lamina
stage I nodule
stage II lamina
stage II nodule
Tissues
Tissues
stage I lamina
stage I nodule
stage II lamina
stage II nodule

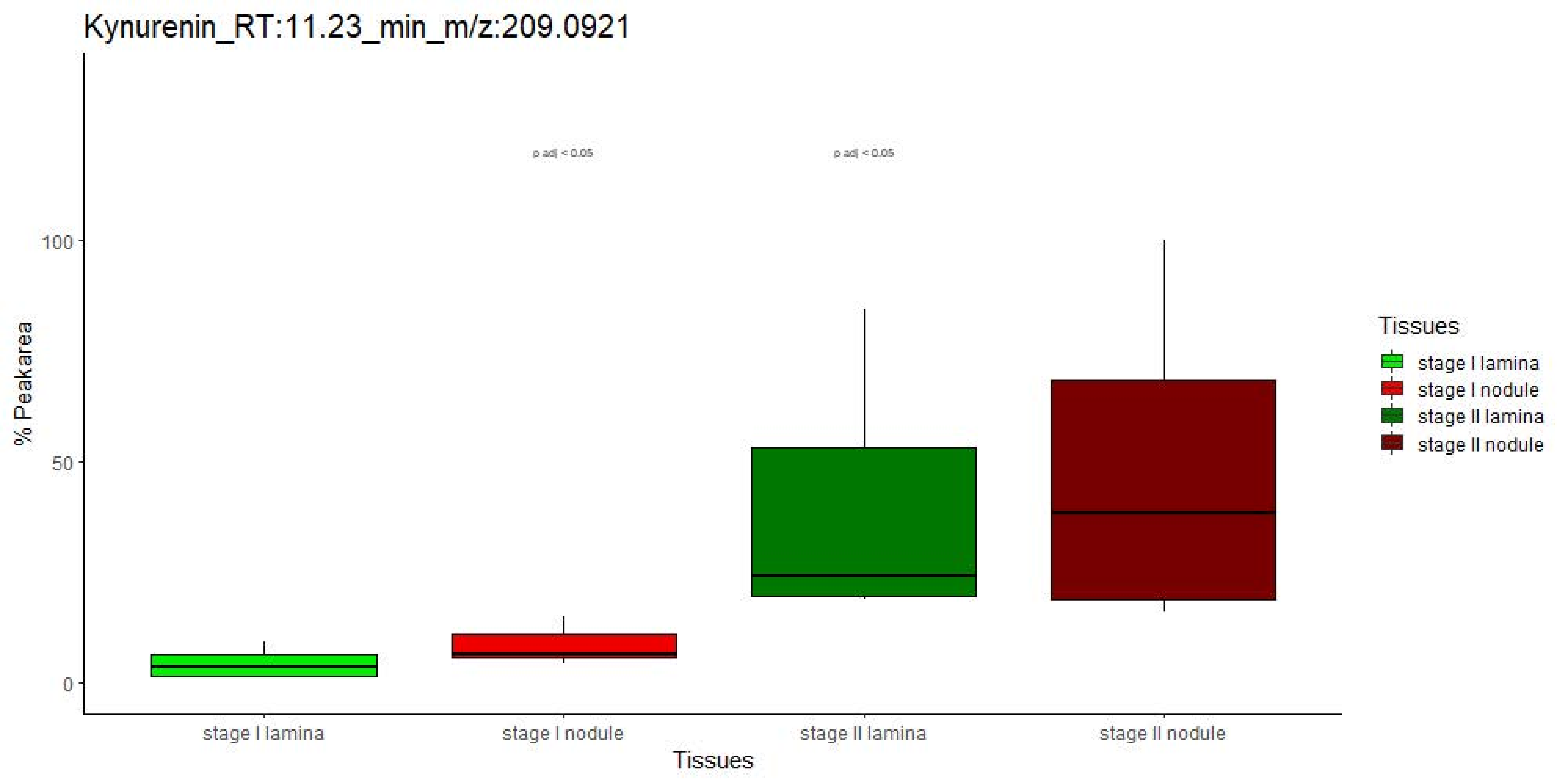

Kynurenin_RT:11.23_min_m/z:209.0921
p adj < 0.05
p adj < 0.05
100
50
0
% Peakarea
stage I lamina
stage I nodule
stage II lamina
stage II nodule
Tissues
Tissues
stage I lamina
stage I nodule
stage II lamina
stage II nodule

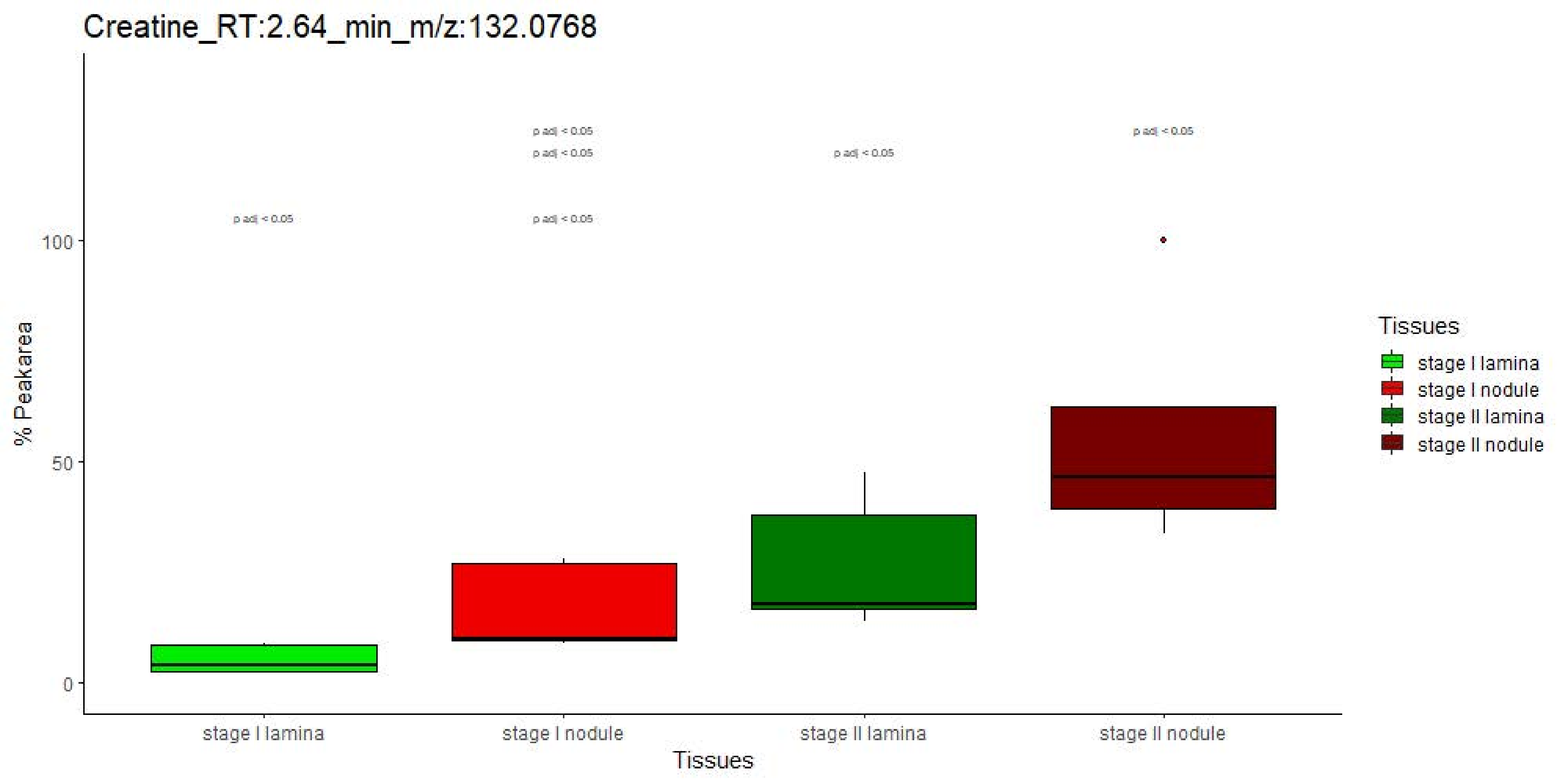

Creatine_RT:2.64_min_m/z:132.0768
p adj < 0.05
p adj < 0.05
p adj < 0.05
p adj < 0.05
p adj < 0.05
p adj < 0.05
100
50
0
% Peakarea
stage I lamina
stage I nodule
stage II lamina
stage II nodule
Tissues
Tissues
stage I lamina
stage I nodule
stage II lamina
stage II nodule

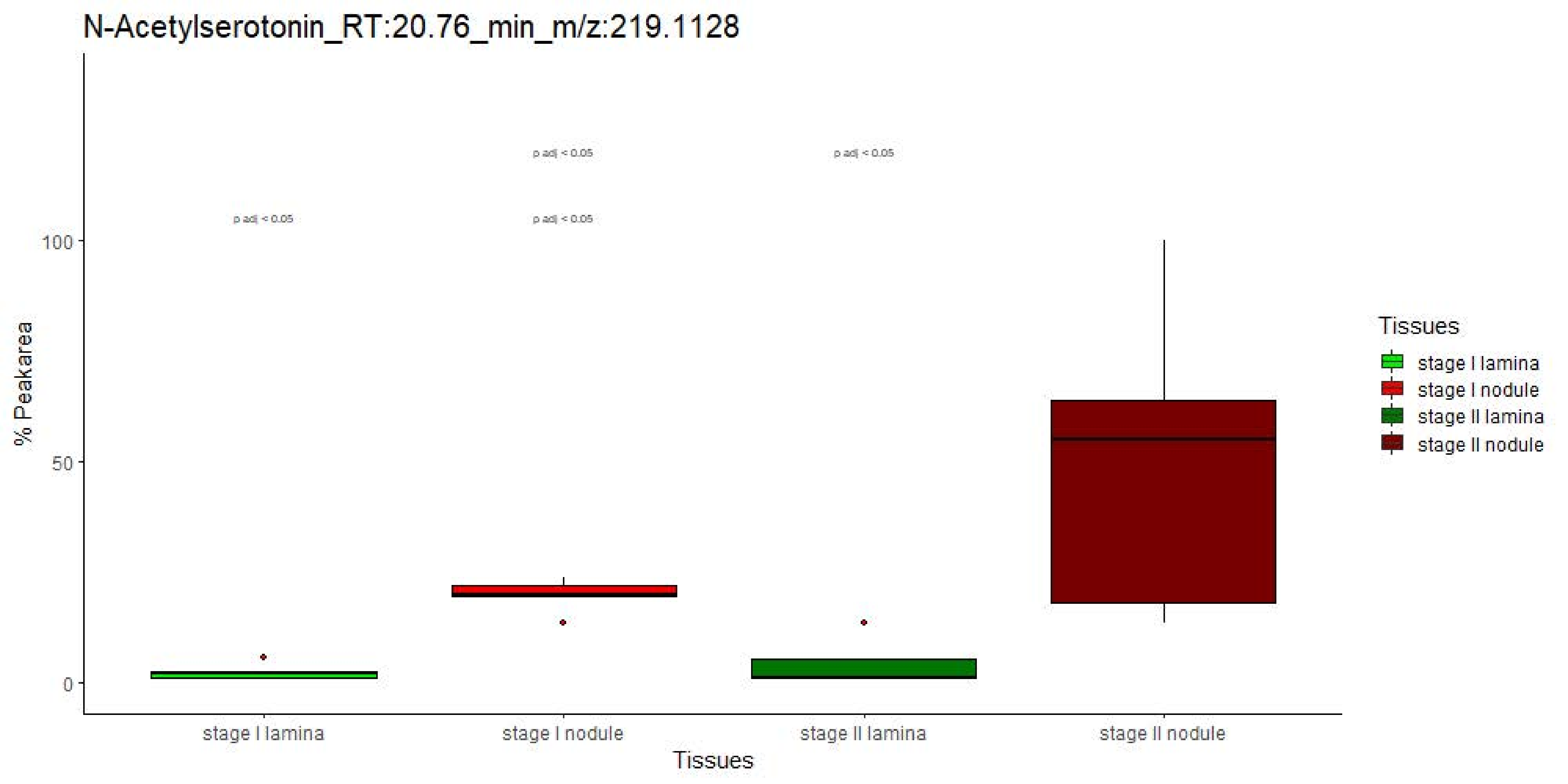

N-Acetylserotonin_RT:20.76_min_m/z:219.1128
p adj < 0.05
p adj < 0.05
p adj < 0.05
p adj < 0.05
% Peakarea
100
50
0
stage I lamina
stage I nodule
stage II lamina
stage II nodule
Tissues
Tissues
stage I lamina
stage I nodule
stage II lamina
stage II nodule

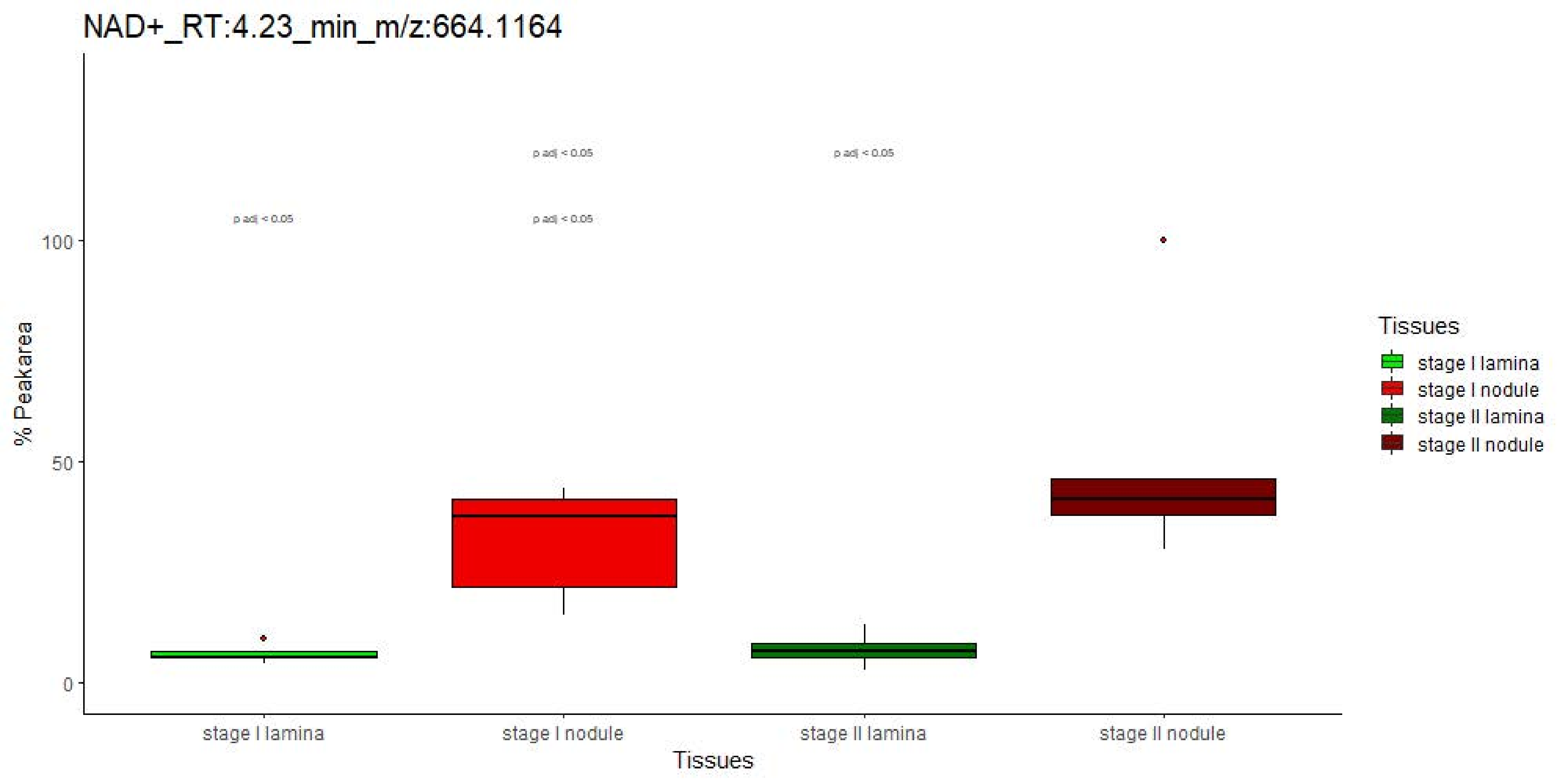

NAD+_RT:4.23_min_m/z:664.1164
p adj < 0.05
p adj < 0.05
p adj < 0.05
p adj < 0.05
100
50
0
% Peakarea
stage I lamina
stage I nodule
stage II lamina
stage II nodule
Tissues
Tissues
stage I lamina
stage I nodule
stage II lamina
stage II nodule

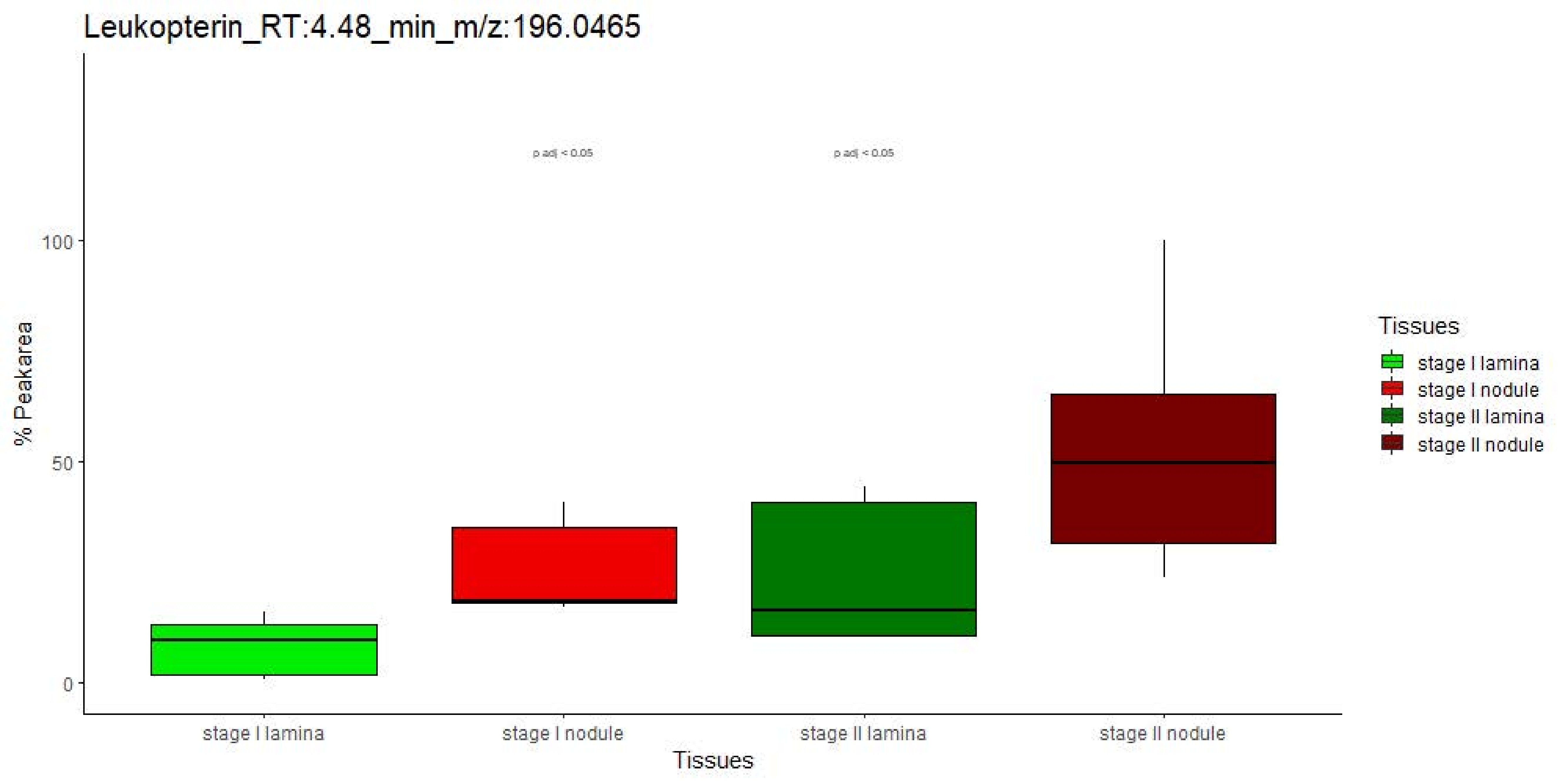

Leukopterin_RT:4.48_min_m/z:196.0465
p adj < 0.05
p adj < 0.05
100
50
0
% Peakarea
stage I lamina
stage I nodule
stage II lamina
stage II nodule
Tissues
Tissues
stage I lamina
stage I nodule
stage II lamina
stage II nodule

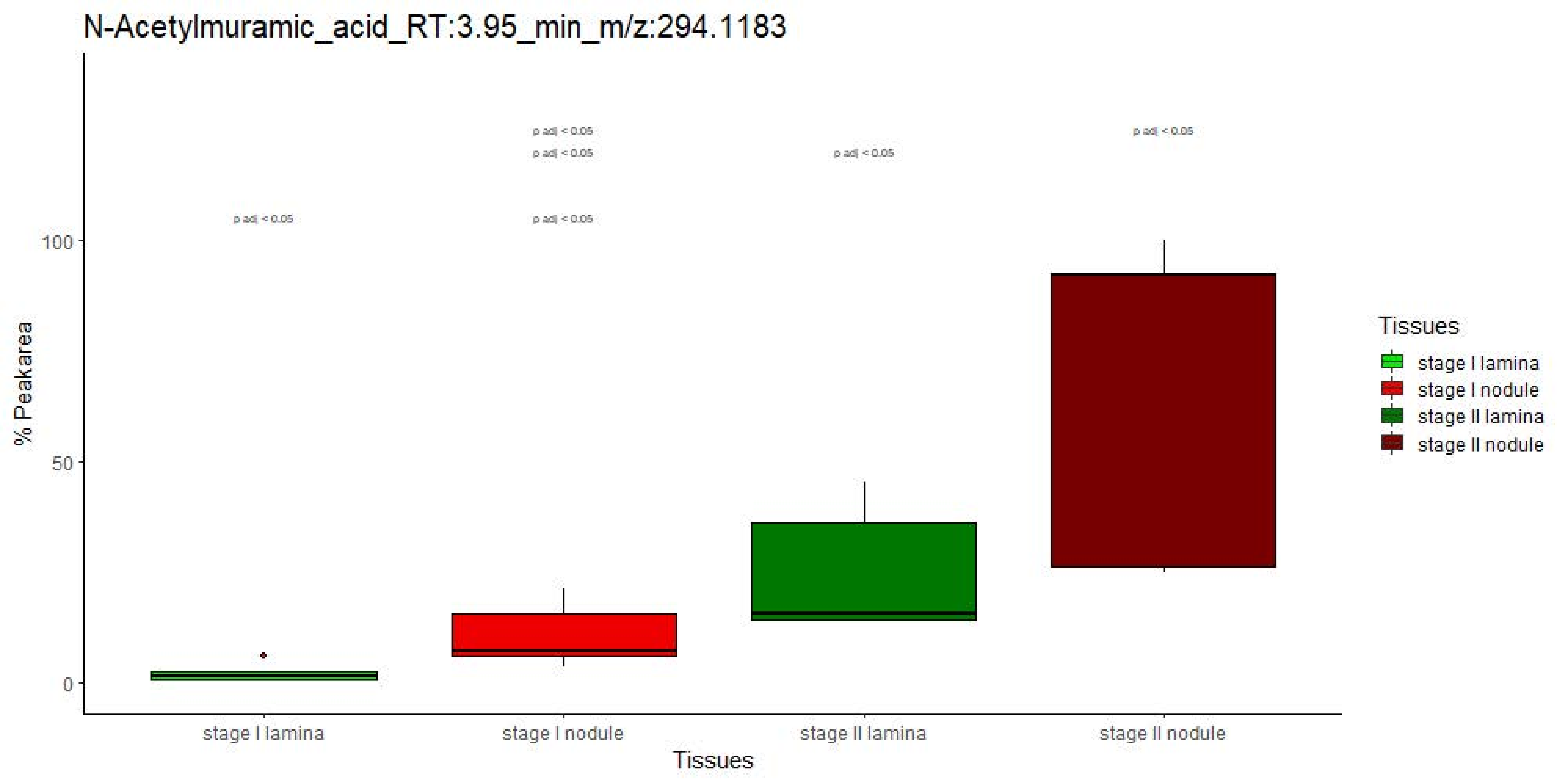
N-Acetylmuramic_acid_RT:3.95_min_m/z:294.1183
% Peakarea
100
50
0
p adj < 0.05
p adj < 0.05
p adj < 0.05
p adj < 0.05
p adj < 0.05
p adj < 0.05
stage I lamina
stage I nodule
stage II lamina
stage II nodule
Tissues
Tissues
stage I lamina
stage I nodule
stage II lamina
stage II nodule

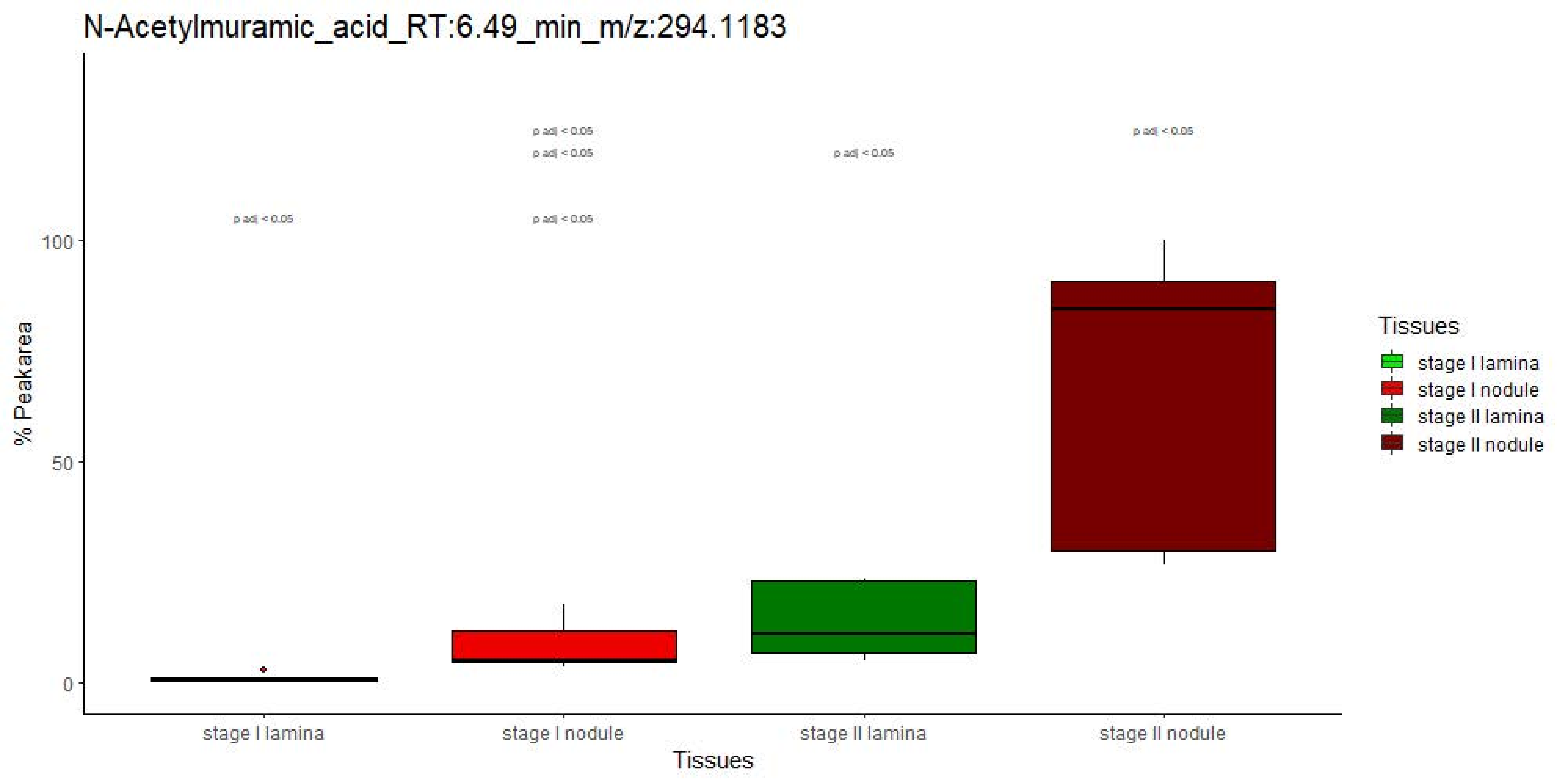

N-Acetylmuramic_acid_RT:6.49_min_m/z:294.1183
p adj < 0.05
p adj < 0.05
p adj < 0.05
p adj < 0.05
p adj < 0.05
p adj < 0.05
% Peakarea
100
50
0
stage I lamina
stage I nodule
stage II lamina
stage II nodule
Tissues
Tissues
stage I lamina
stage I nodule
stage II lamina
stage II nodule

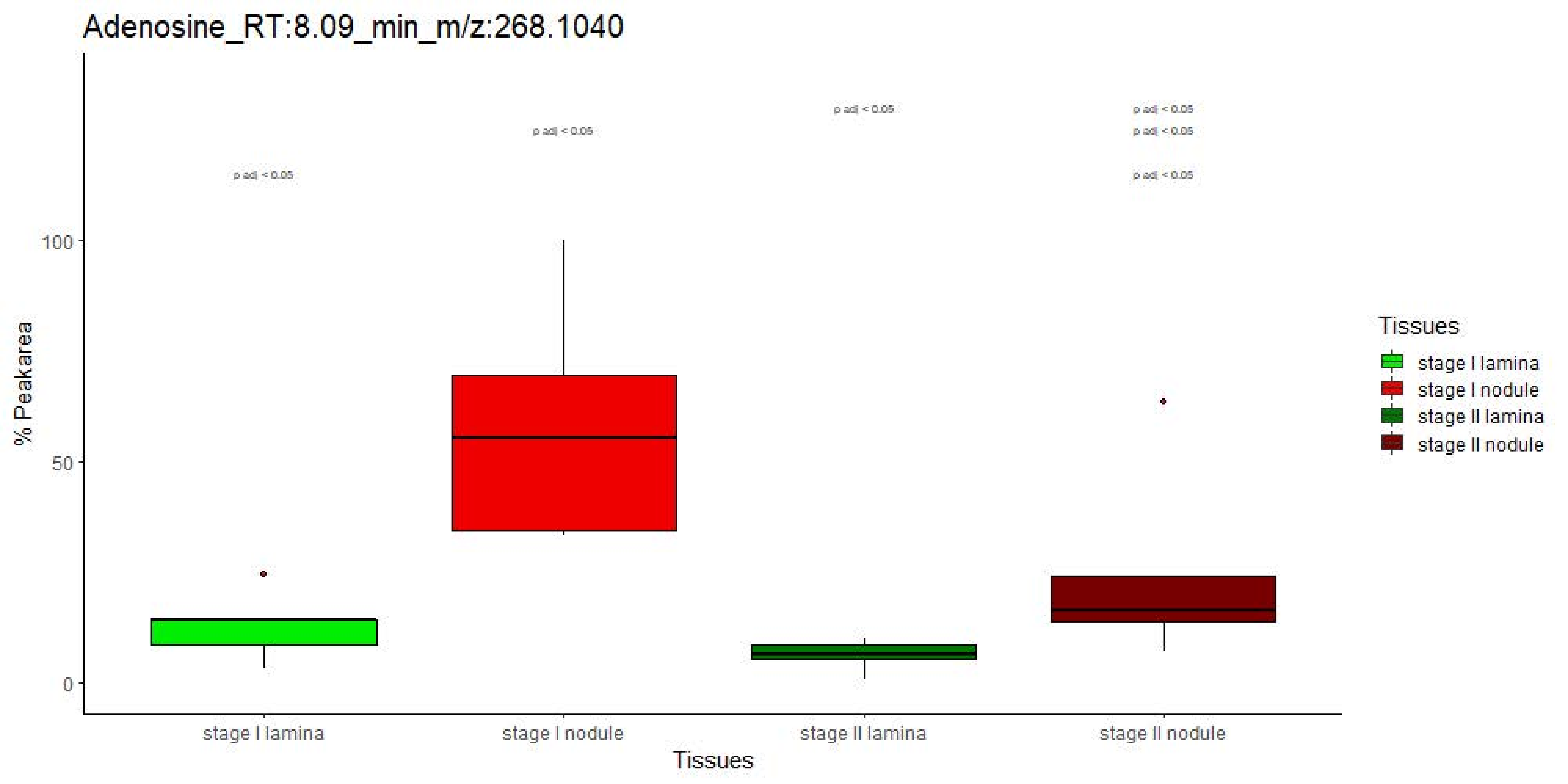
Adenosine_RT:8.09_min_m/z:268.1040
p adj < 0.05
p adj < 0.05
p adj < 0.05
p adj < 0.05
p adj < 0.05
p adj < 0.05
% Peakarea
100
50
0
stage I lamina
stage I nodule
stage II lamina
stage II nodule
Tissues
Tissues
stage I lamina
stage I nodule
stage II lamina
stage II nodule

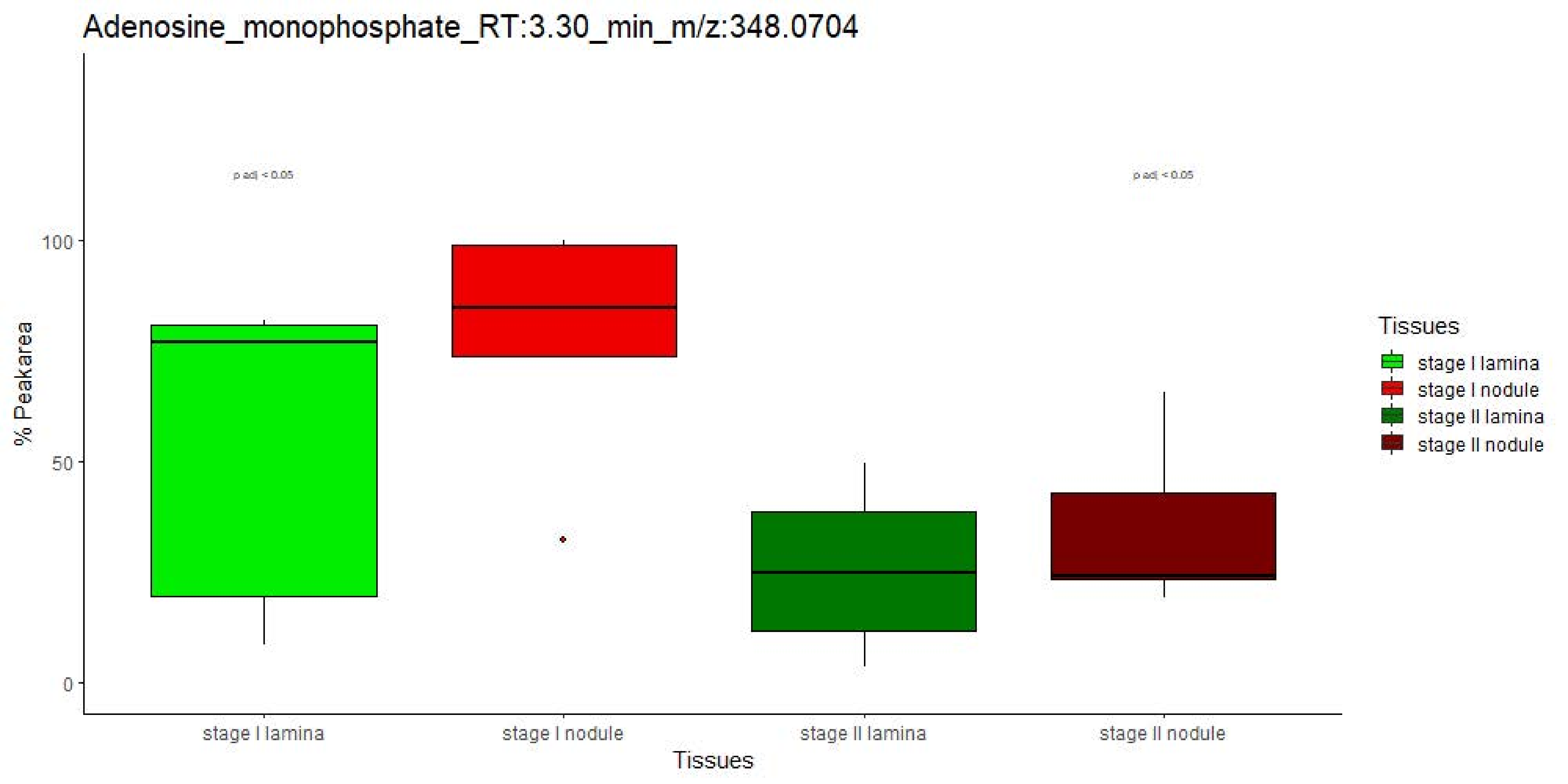
Adenosine_monophosphate_RT:3.30_min_m/z:348.0704
% Peakarea
100
50
0
p adj < 0.05
p adj < 0.05
Tissues
stage I lamina
stage I nodule
stage II lamina
stage II nodule
stage I lamina
stage I nodule
stage II lamina
stage II nodule
Tissues

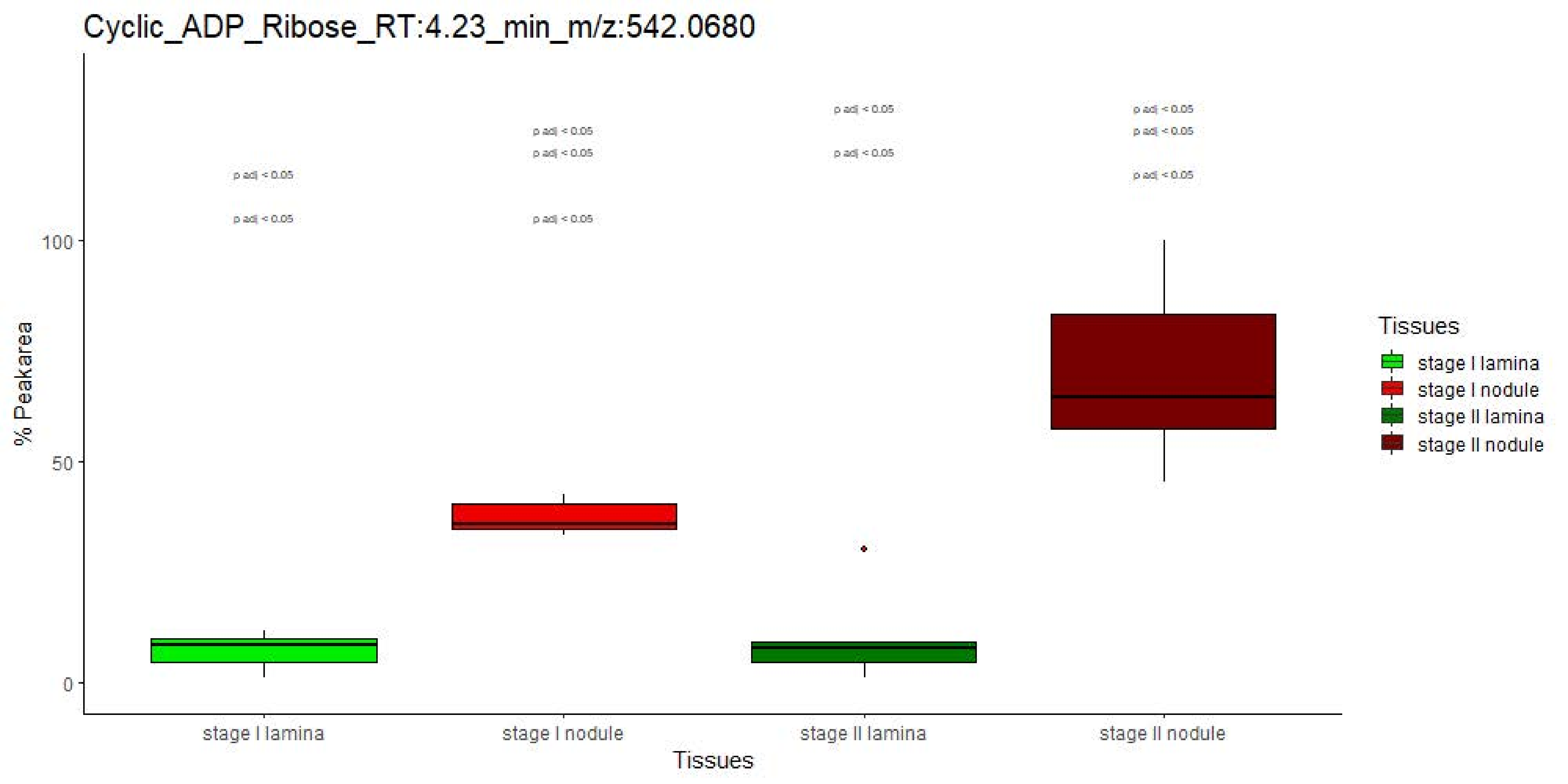

Cyclic_ADP_Ribose_RT:4.23_min_m/z:542.0680
p adj < 0.05
p adj < 0.05
p adj < 0.05
p adj < 0.05
p adj < 0.05
p adj < 0.05
p adj < 0.05
p adj < 0.05
p adj < 0.05
p adj < 0.05
p adj < 0.05
100
50
0
% Peakarea
stage I lamina
stage I nodule
stage II lamina
stage II nodule
Tissues
Tissues
stage I lamina
stage I nodule
stage II lamina
stage II nodule

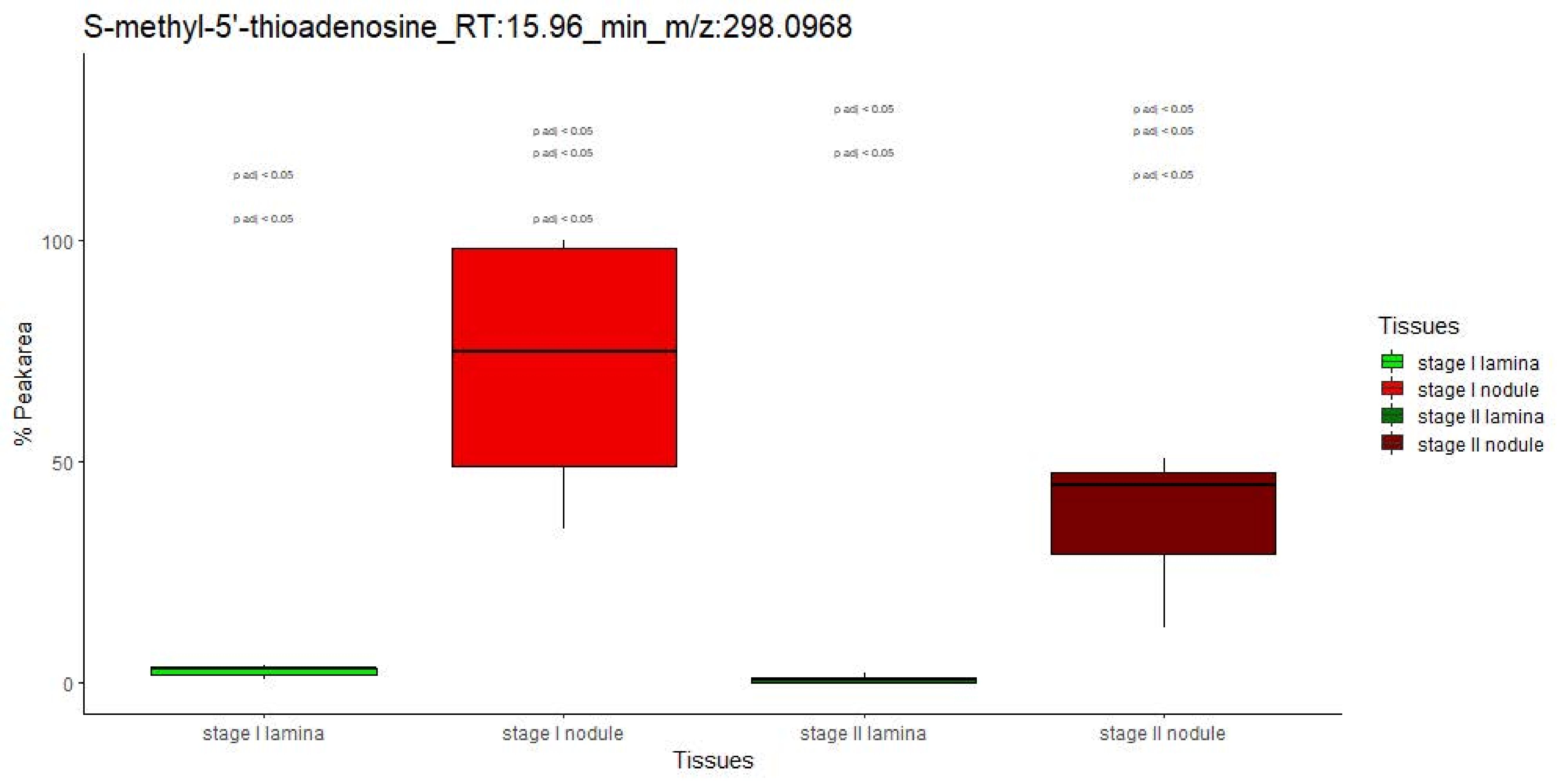
S-methyl-5'-thioadenosine_RT:15.96_min_m/z:298.0968
% Peakarea
100
50
0
p adj < 0.05
Tissues
stage I lamina
stage I nodule
stage II lamina
stage II nodule

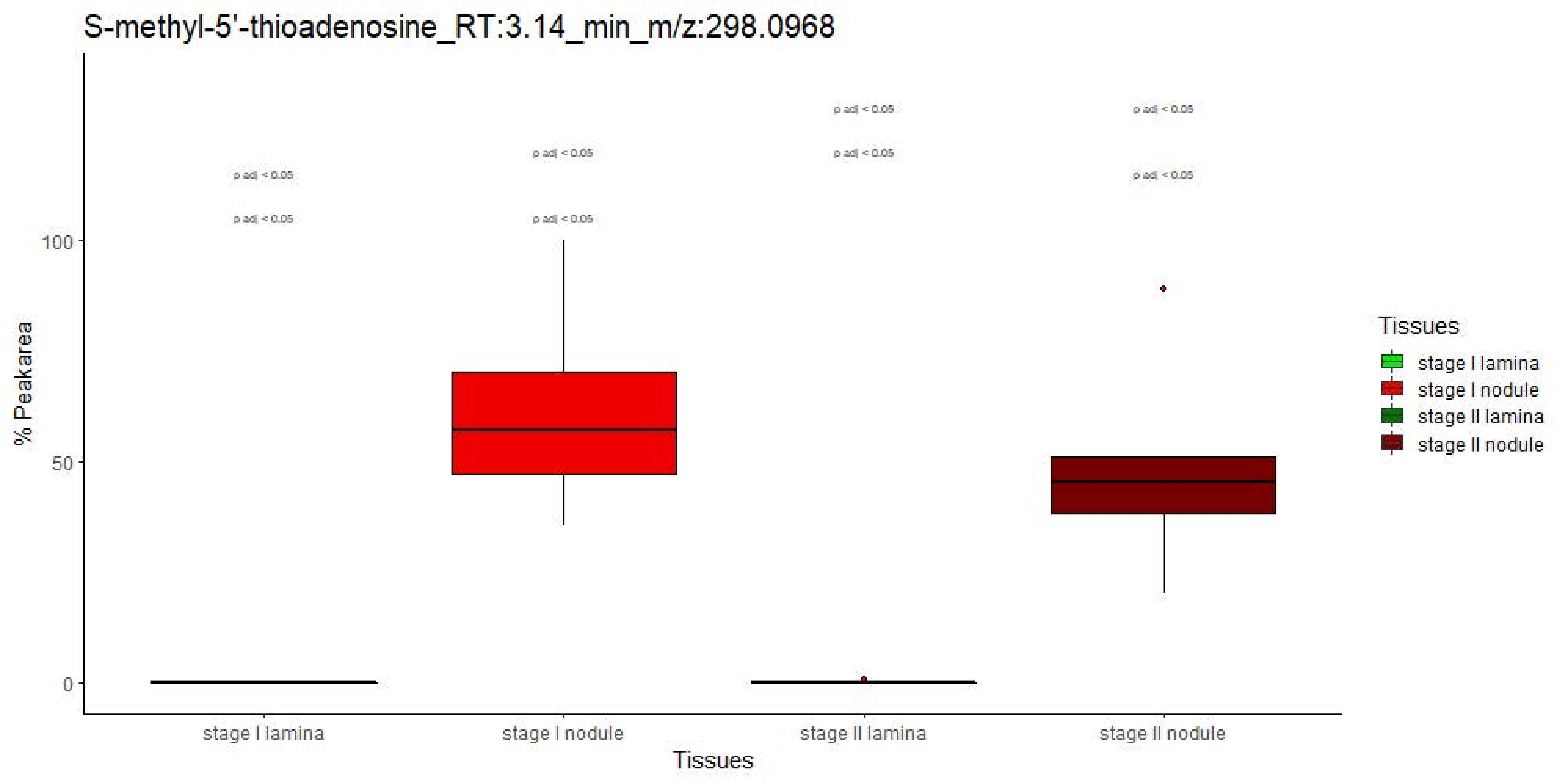
S-methyl-5'-thioadenosine_RT:3.14_min_m/z:298.0968
p adj < 0.05
p adj < 0.05
p adj < 0.05
p adj < 0.05
p adj < 0.05
p adj < 0.05
p adj < 0.05
p adj < 0.05
100
50
0
% Peakarea
Tissues
stage I lamina
stage I nodule
stage II lamina
stage II nodule
stage I lamina
stage I nodule
stage II lamina
stage II nodule
Tissues

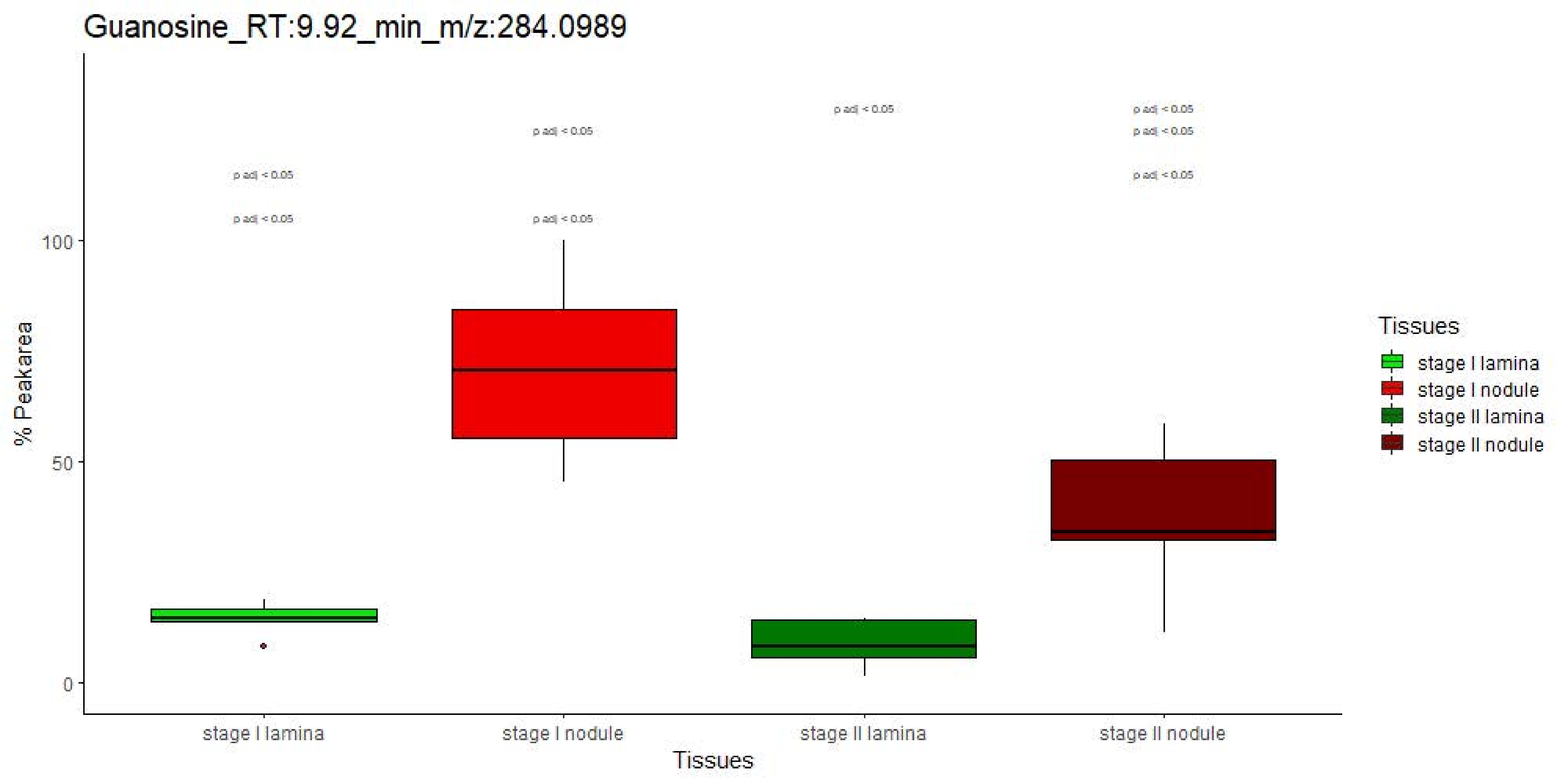

Guanosine_RT:9.92_min_m/z:284.0989
p adj < 0.05
p adj < 0.05
p adj < 0.05
p adj < 0.05
p adj < 0.05
p adj < 0.05
p adj < 0.05
p adj < 0.05
100
50
0
% Peakarea
stage I lamina
stage I nodule
stage II lamina
stage II nodule
Tissues
Tissues
stage I lamina
stage I nodule
stage II lamina
stage II nodule

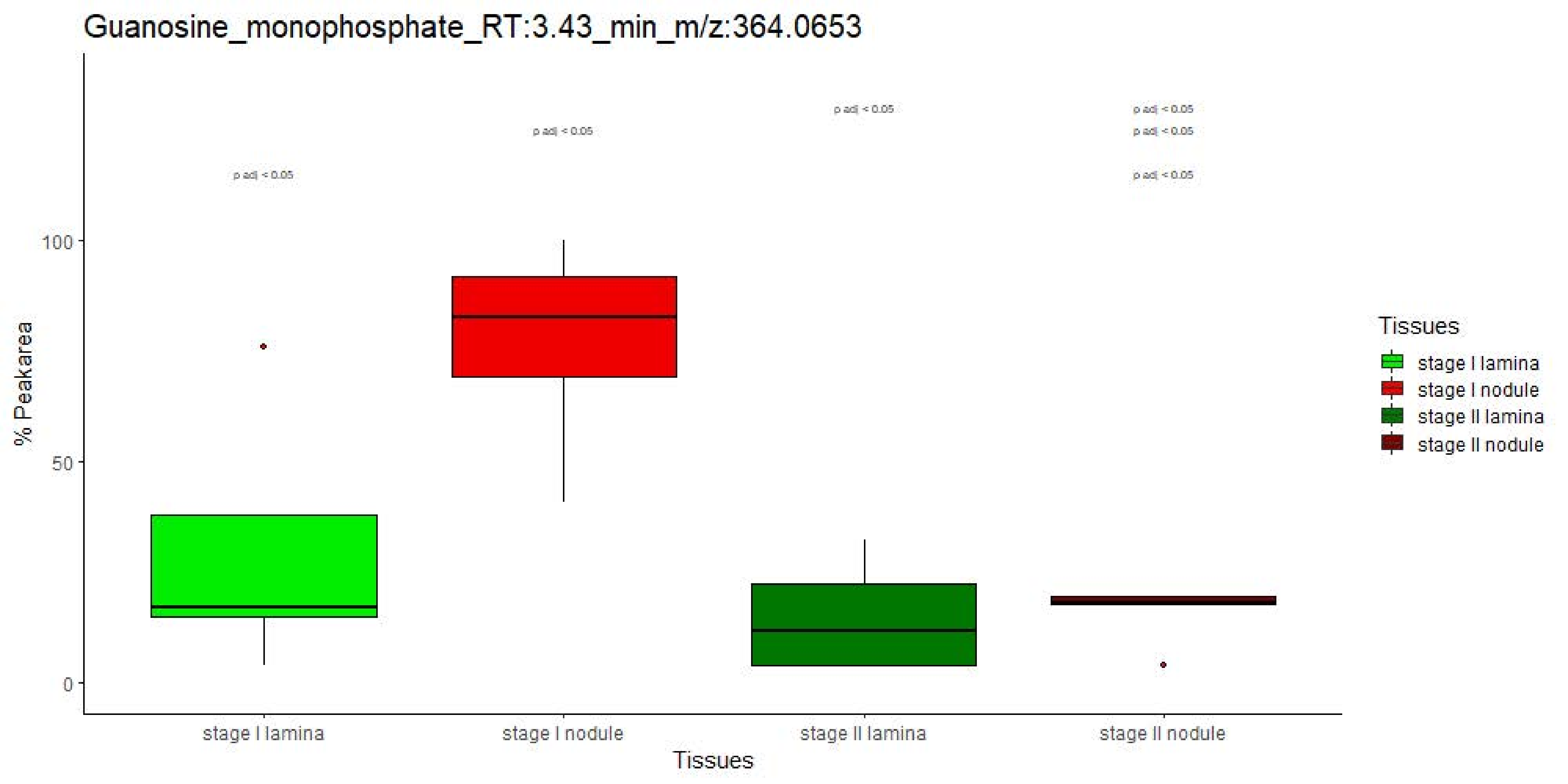
Guanosine_monophosphate_RT:3.43_min_m/z:364.0653
p adj < 0.05
p adj < 0.05
p adj < 0.05
p adj < 0.05
p adj < 0.05
p adj < 0.05
100
50
0
% Peakarea
stage I lamina
stage I nodule
stage II lamina
stage II nodule
Tissues
Tissues
stage I lamina
stage I nodule
stage II lamina
stage II nodule

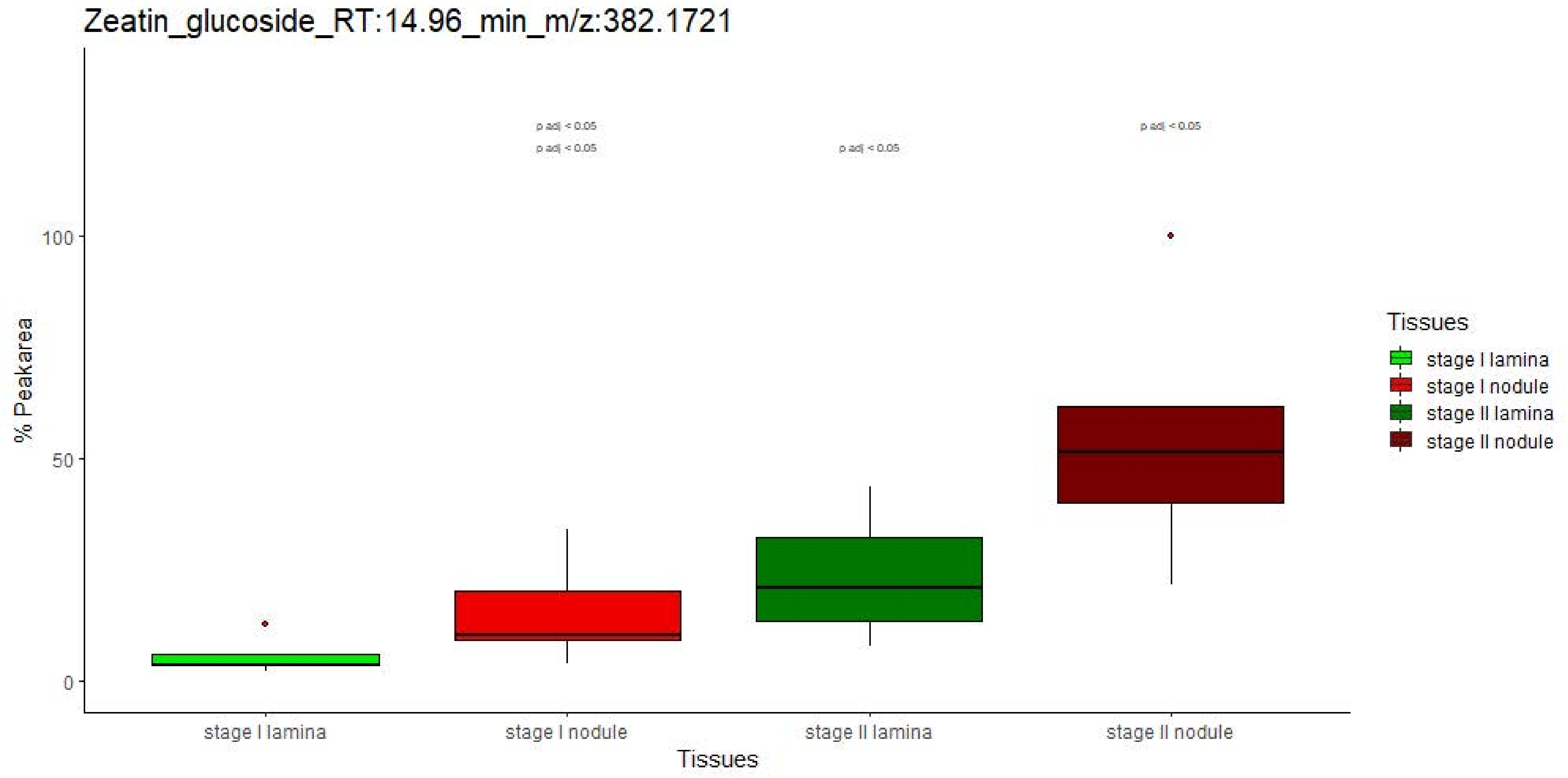

Zeatin_glucoside_RT:14.96_min_m/z:382.1721
p adj < 0.05
p adj < 0.05
p adj < 0.05
p adj < 0.05
100
50
0
% Peakarea
stage I lamina
stage I nodule
stage II lamina
stage II nodule
Tissues
Tissues
stage I lamina
stage I nodule
stage II lamina
stage II nodule

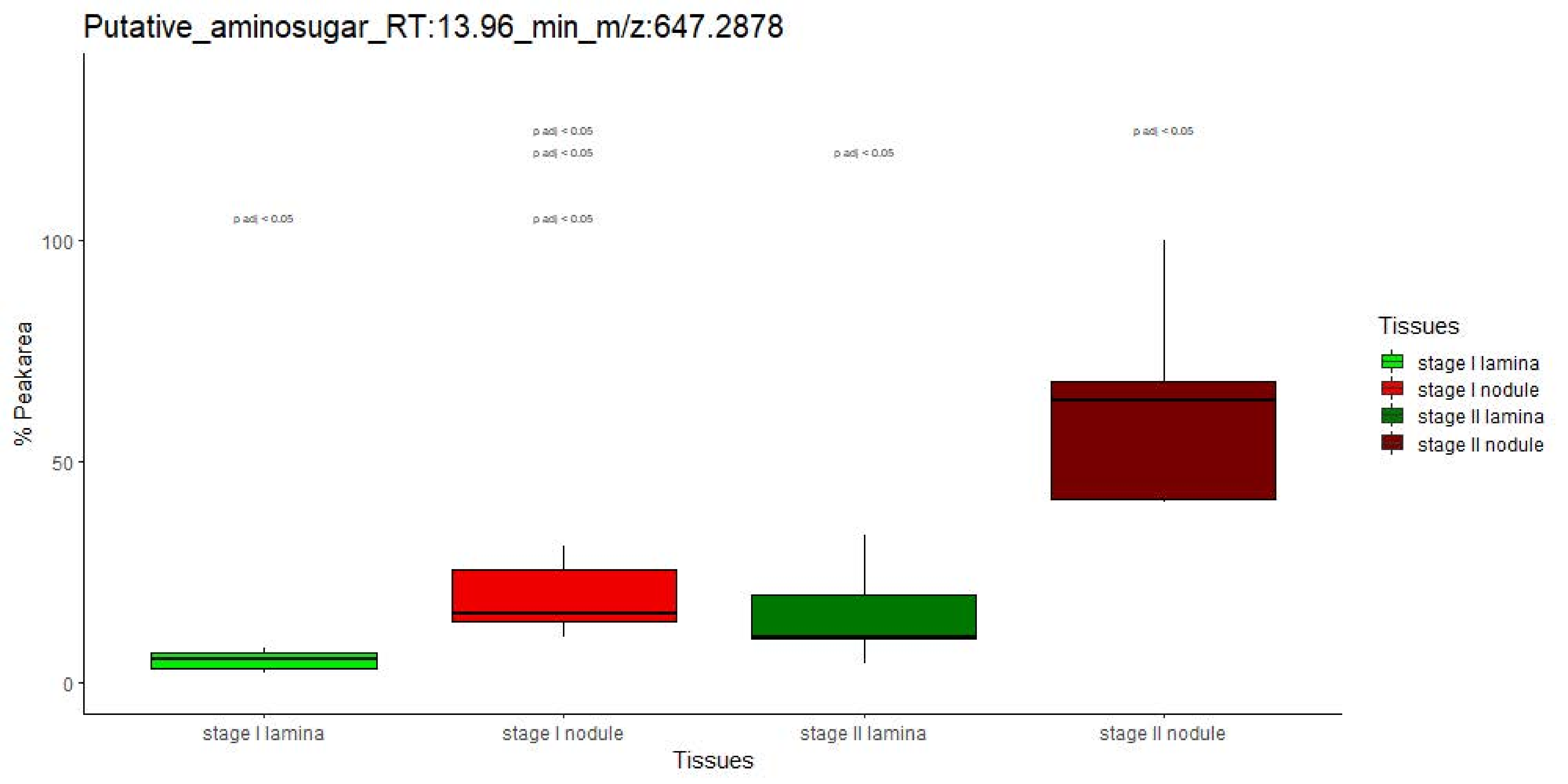
Putative_aminosugar_RT:13.96_min_m/z:647.2878
p adj < 0.05
p adj < 0.05
p adj < 0.05
p adj < 0.05
p adj < 0.05
p adj < 0.05
100
50
0
% Peakarea
stage I lamina
stage I nodule
stage II lamina
stage II nodule
Tissues
Tissues
stage I lamina
stage I nodule
stage II lamina
stage II nodule

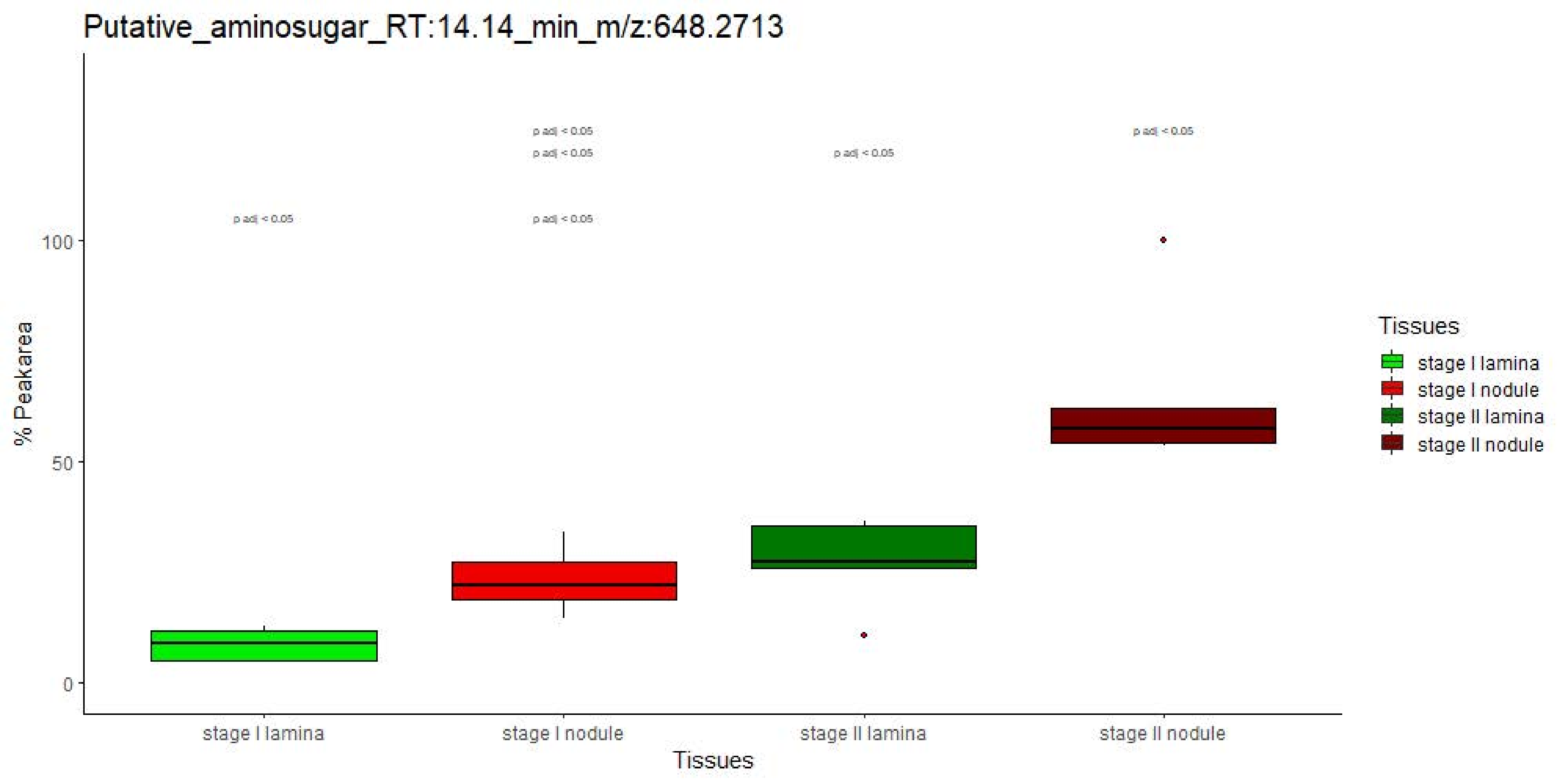

Putative_aminosugar_RT:14.14_min_m/z:648.2713
% Peakarea
100
50
0
p adj < 0.05
p adj < 0.05
p adj < 0.05
p adj < 0.05
p adj < 0.05
p adj < 0.05
stage I lamina
stage I nodule
stage II lamina
stage II nodule
Tissues
Tissues
stage I lamina
stage I nodule
stage II lamina
stage II nodule

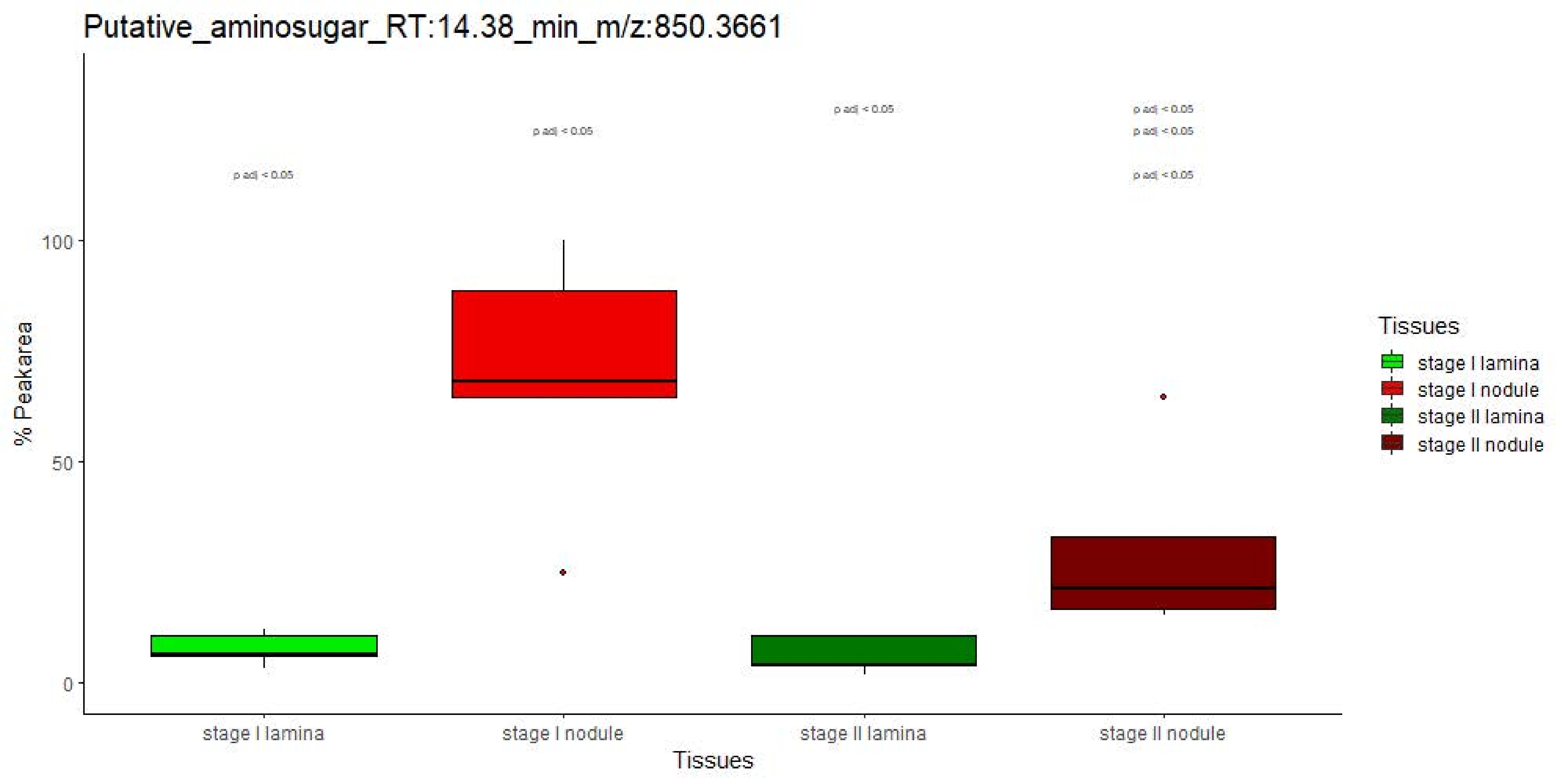

Putative_aminosugar_RT:14.38_min_m/z:850.3661
p adj < 0.05
p adj < 0.05
p adj < 0.05
p adj < 0.05
p adj < 0.05
p adj < 0.05
100
50
0
% Peakarea
stage I lamina
stage I nodule
stage II lamina
stage II nodule
Tissues
Tissues
stage I lamina
stage I nodule
stage II lamina
stage II nodule

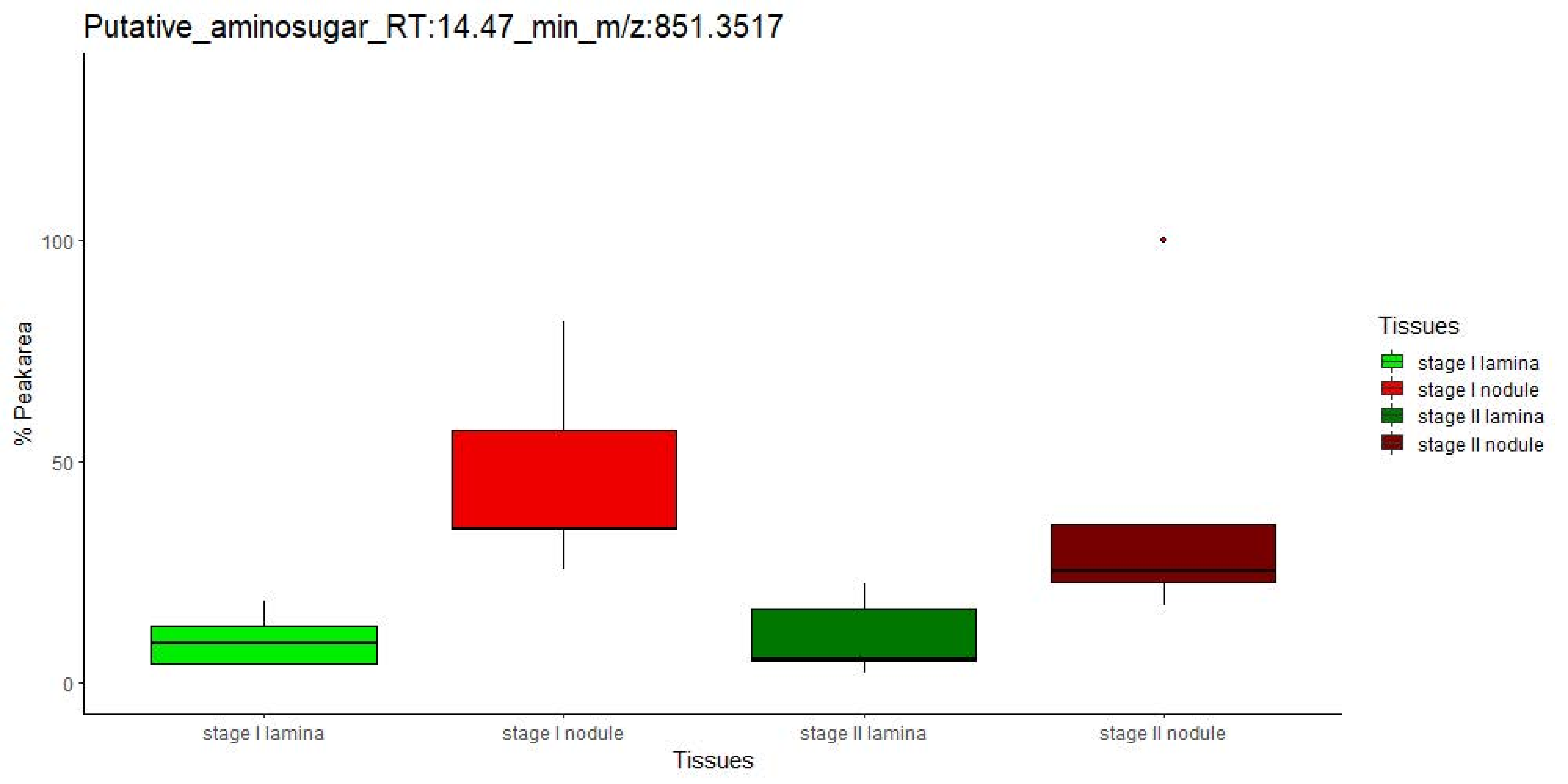

Putative_aminosugar_RT:14.47_min_m/z:851.3517
% Peakarea
100
50
0
stage I lamina
stage I nodule
stage II lamina
stage II nodule
Tissues
Tissues
stage I lamina
stage I nodule
stage II lamina
stage II nodule

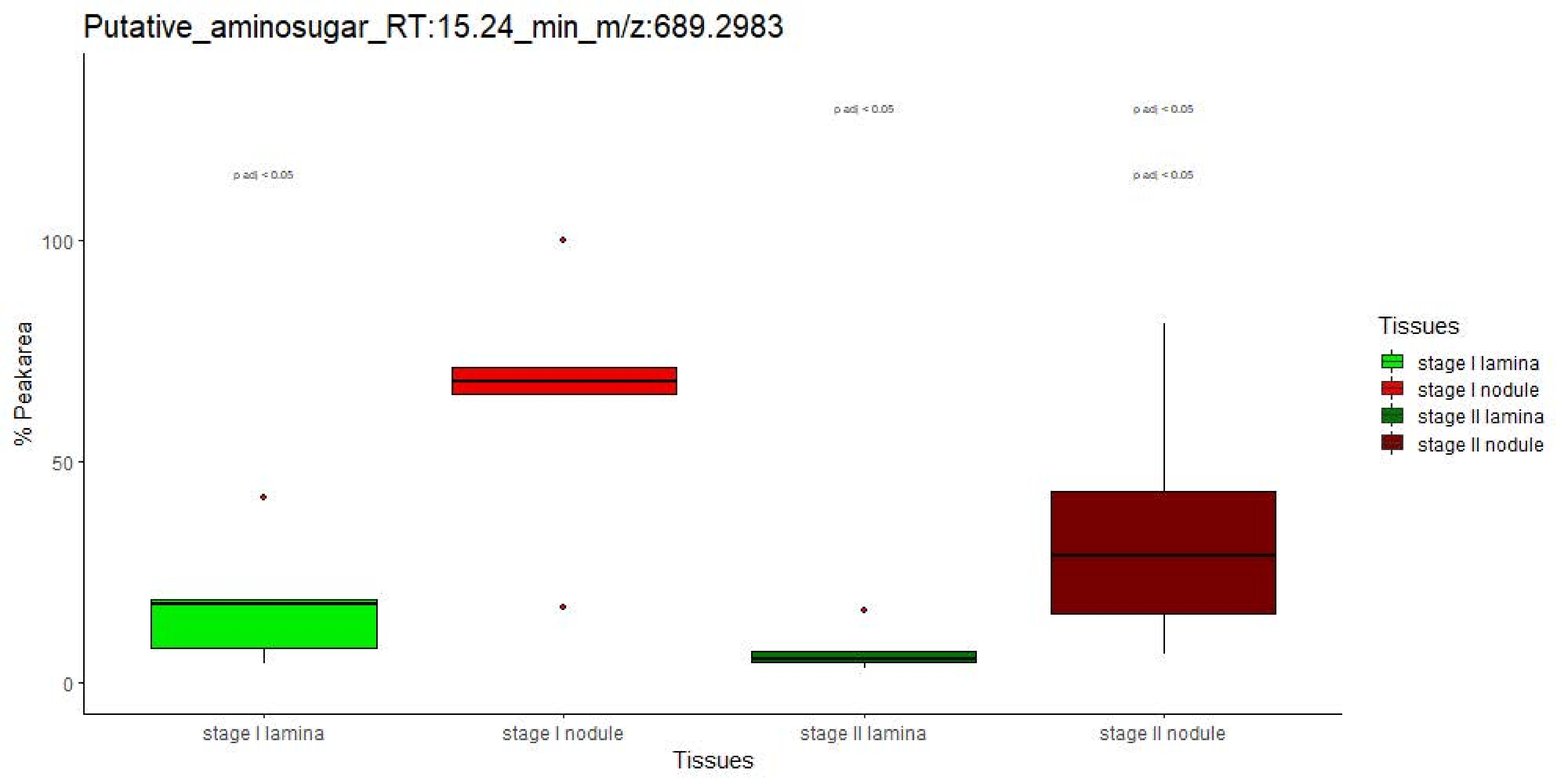

Putative_aminosugar_RT:15.24_min_m/z:689.2983
p adj < 0.05
p adj < 0.05
p adj < 0.05
p adj < 0.05
100
50
0
% Peakarea
stage I lamina
stage I nodule
stage II lamina
stage II nodule
Tissues
Tissues
stage I lamina
stage I nodule
stage II lamina
stage II nodule

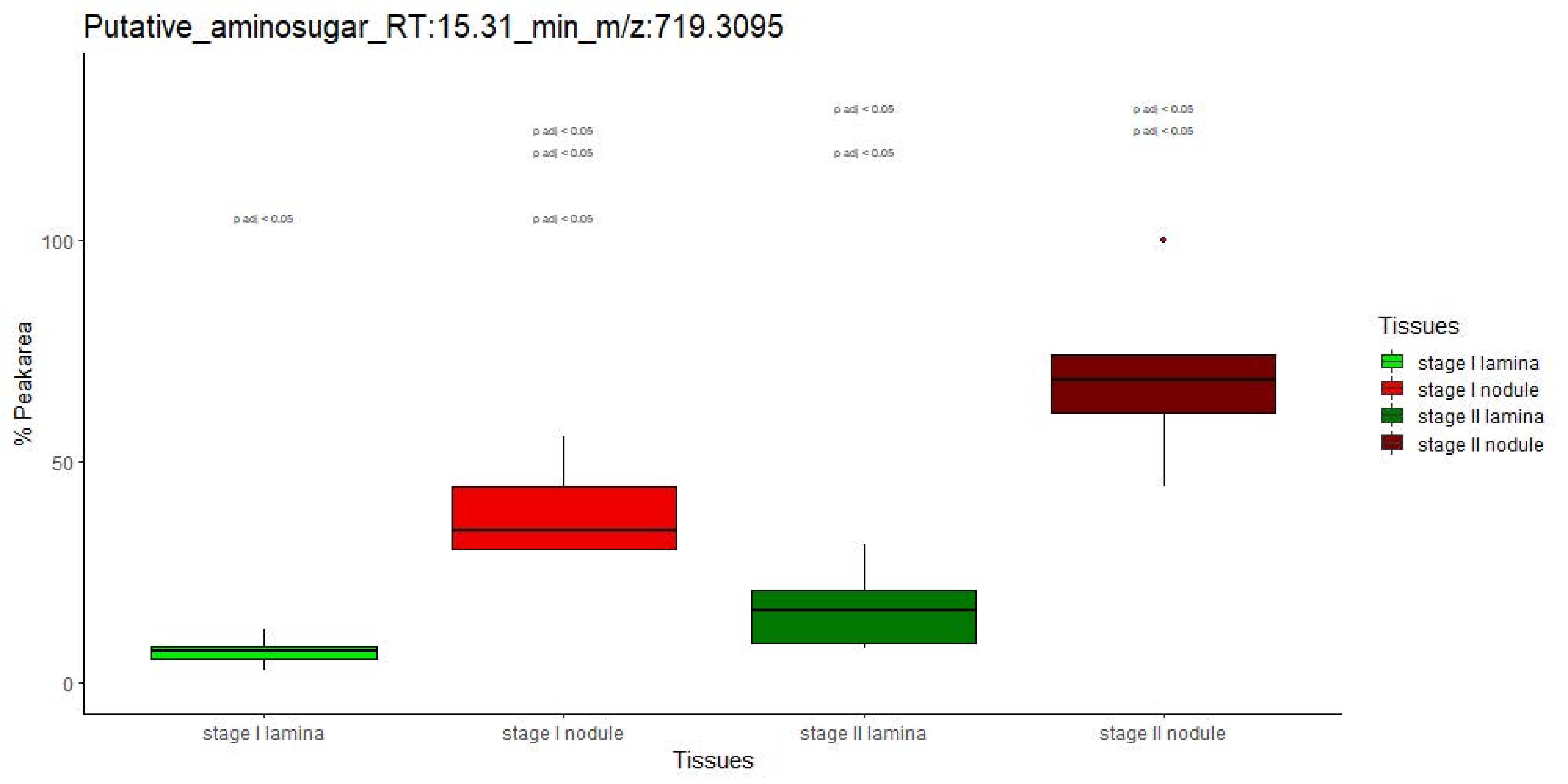

Putative_aminosugar_RT:15.31_min_m/z:719.3095
p adj < 0.05
p adj < 0.05
p adj < 0.05
p adj < 0.05
p adj < 0.05
p adj < 0.05
p adj < 0.05
p adj < 0.05
p adj < 0.05
100
50
0
% Peakarea
stage I lamina
stage I nodule
stage II lamina
stage II nodule
Tissues
Tissues
stage I lamina
stage I nodule
stage II lamina
stage II nodule

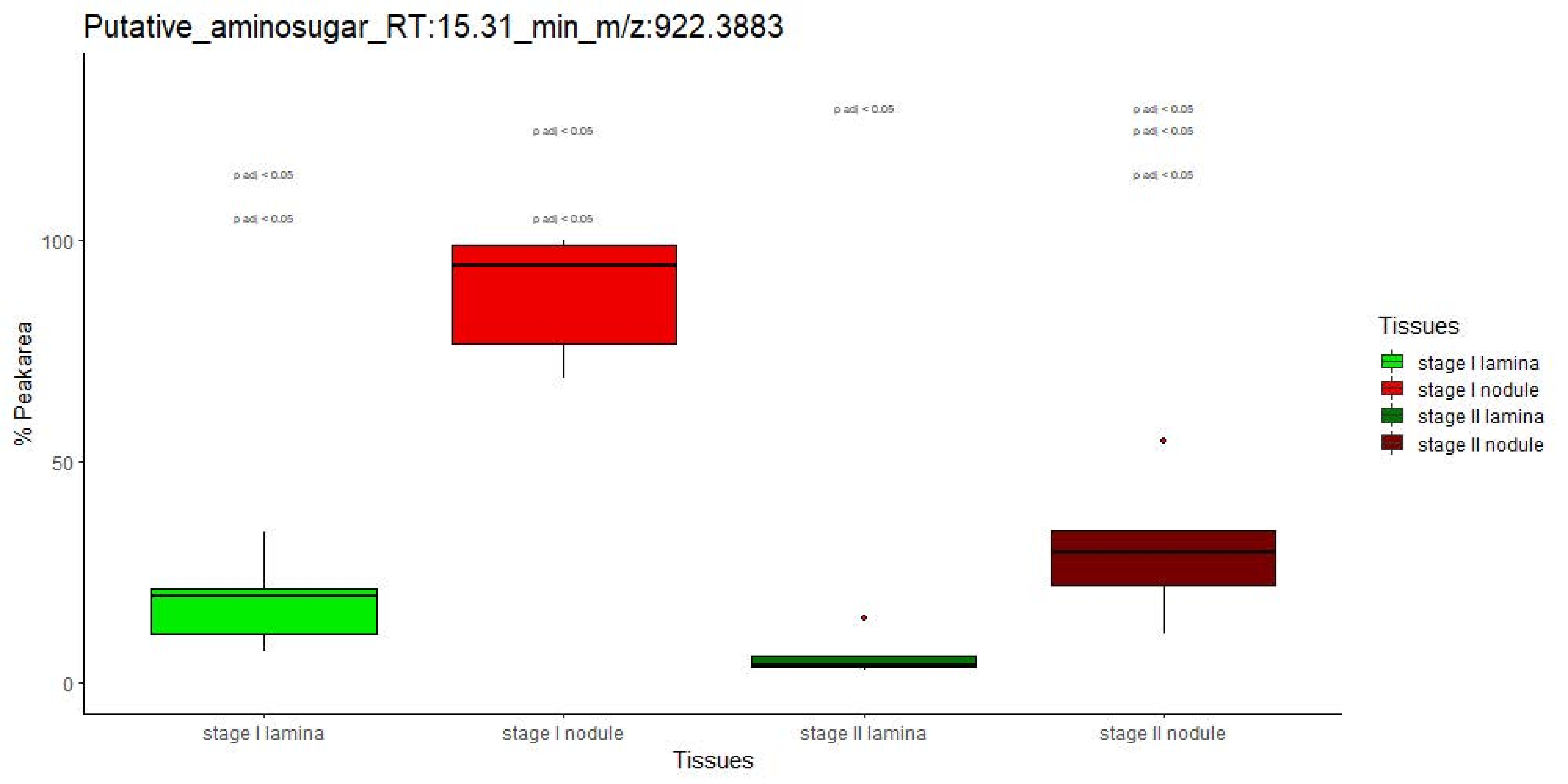

Putative_aminosugar_RT:15.31_min_m/z:922.3883
p adj < 0.05
p adj < 0.05
p adj < 0.05
p adj < 0.05
p adj < 0.05
p adj < 0.05
p adj < 0.05
p adj < 0.05
100
50
0
% Peakarea
Tissues
stage I lamina
stage I nodule
stage II lamina
stage II nodule
stage I lamina
stage I nodule
stage II lamina
stage II nodule
Tissues

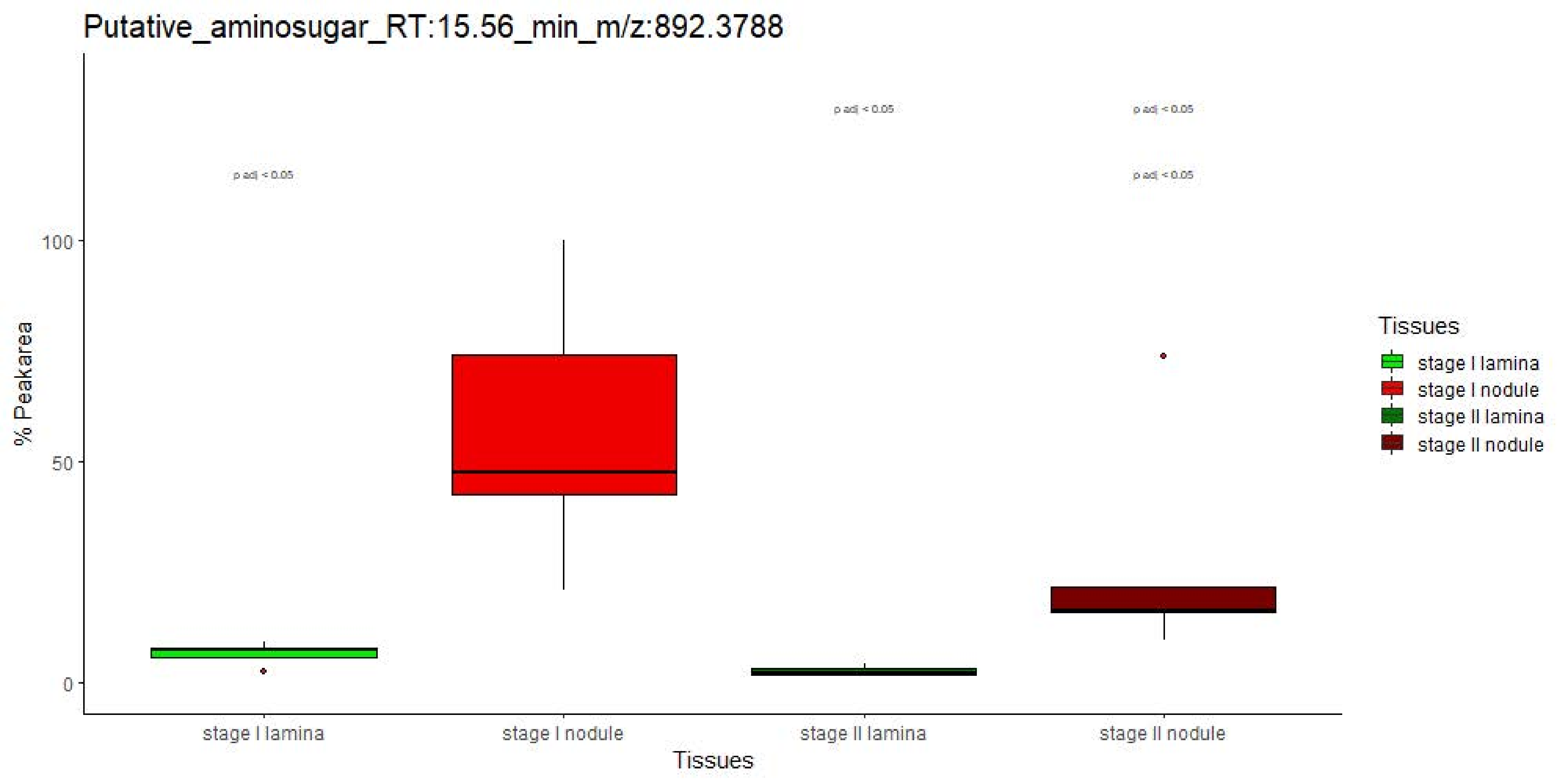
Putative_aminosugar_RT:15.56_min_m/z:892.3788
p adj < 0.05
p adj < 0.05
p adj < 0.05
p adj < 0.05
100
50
0
% Peakarea
stage I lamina
stage I nodule
stage II lamina
stage II nodule
Tissues
Tissues
stage I lamina
stage I nodule
stage II lamina
stage II nodule

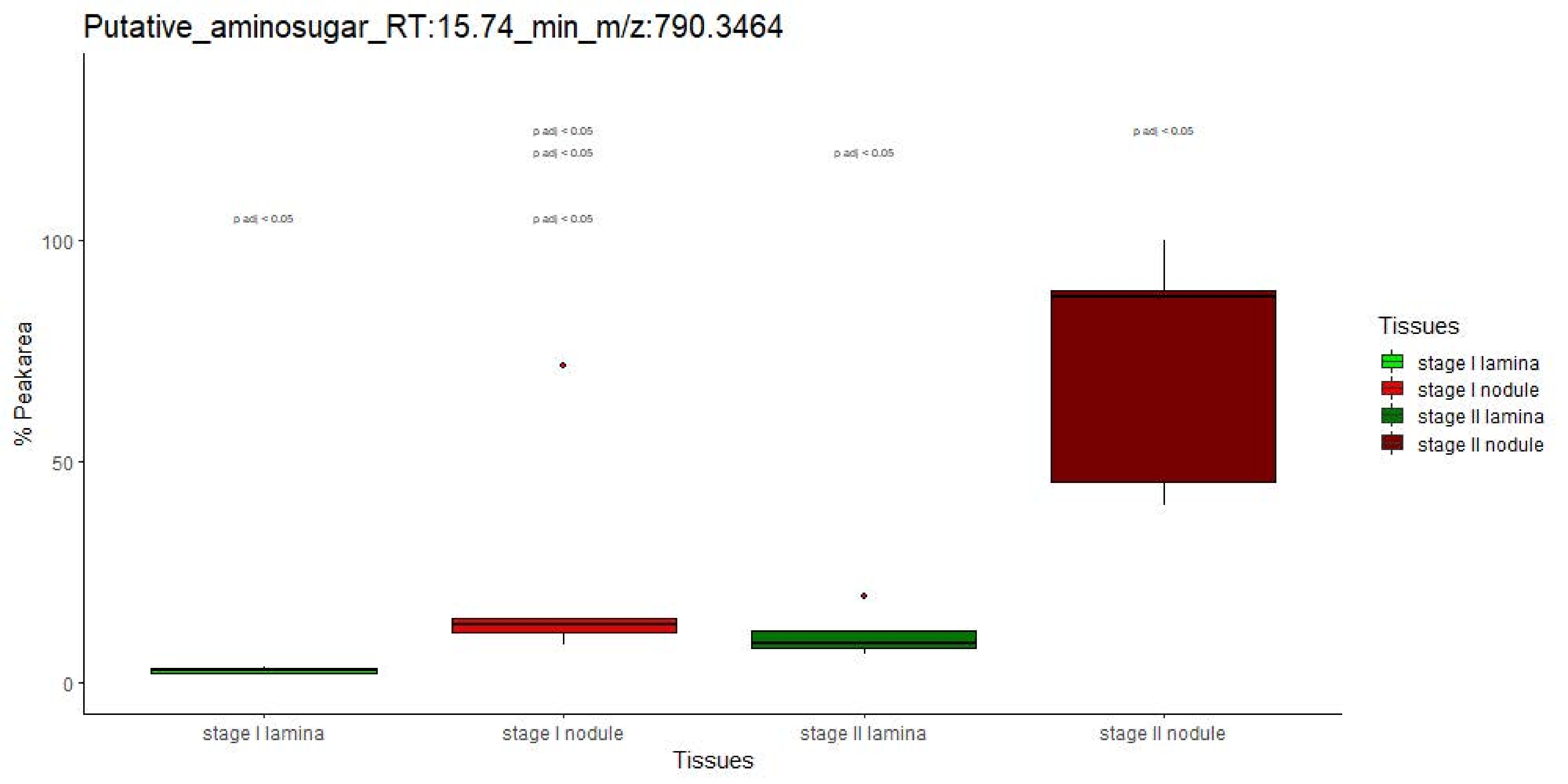
Putative_aminosugar_RT:15.74_min_m/z:790.3464
p adj < 0.05
p adj < 0.05
p adj < 0.05
p adj < 0.05
p adj < 0.05
p adj < 0.05
100
50
0
% Peakarea
stage I lamina
stage I nodule
stage II lamina
stage II nodule
Tissues
Tissues
stage I lamina
stage I nodule
stage II lamina
stage II nodule

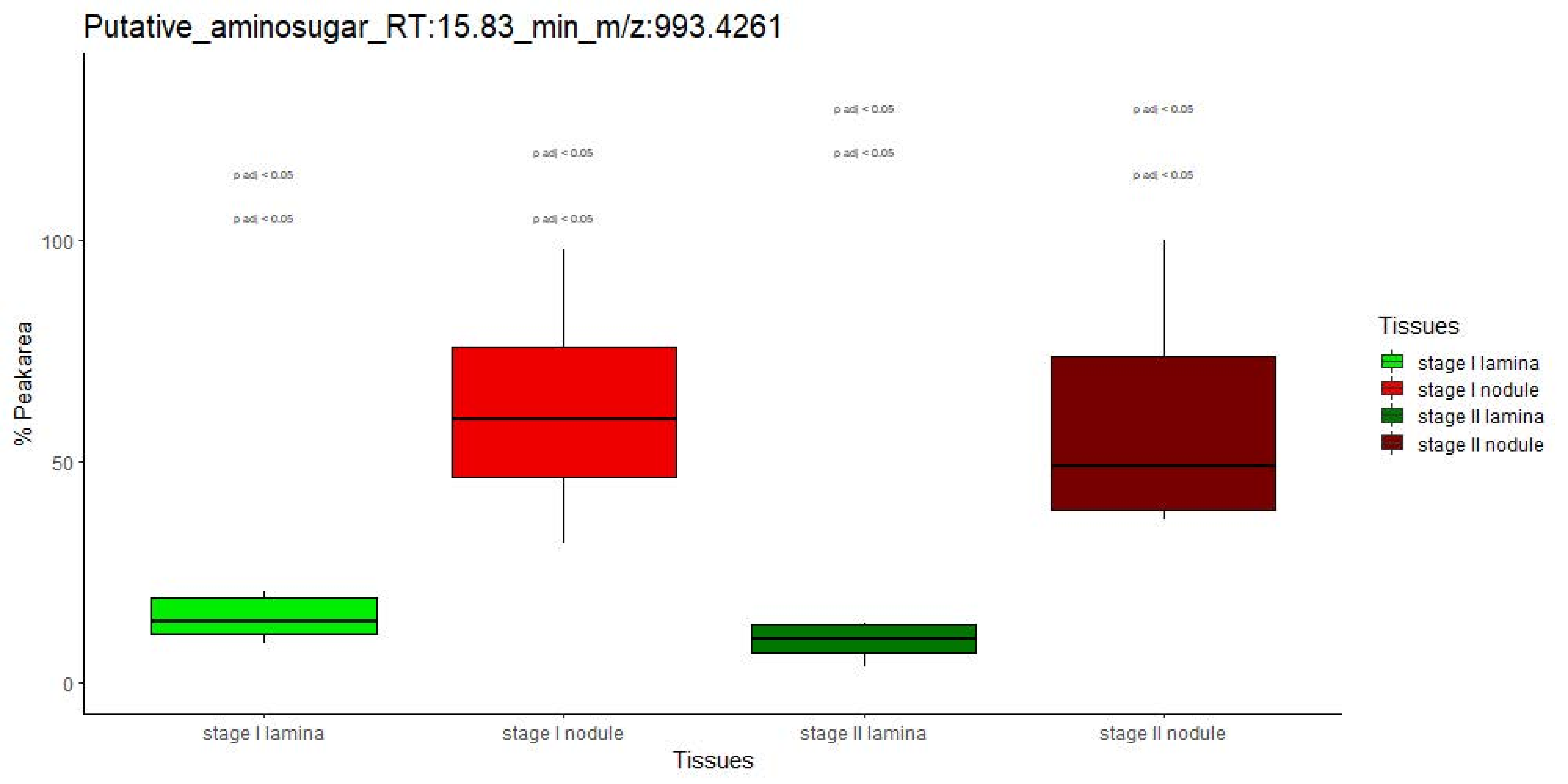
Putative_aminosugar_RT:15.83_min_m/z:993.4261
% Peakarea
0
50
100
p adj < 0.05
p adj < 0.05
p adj < 0.05
p adj < 0.05
p adj < 0.05
p adj < 0.05
p adj < 0.05
p adj < 0.05
stage I lamina
stage I nodule
stage II lamina
stage II nodule
Tissues
Tissues
stage I lamina
stage I nodule
stage II lamina
stage II nodule

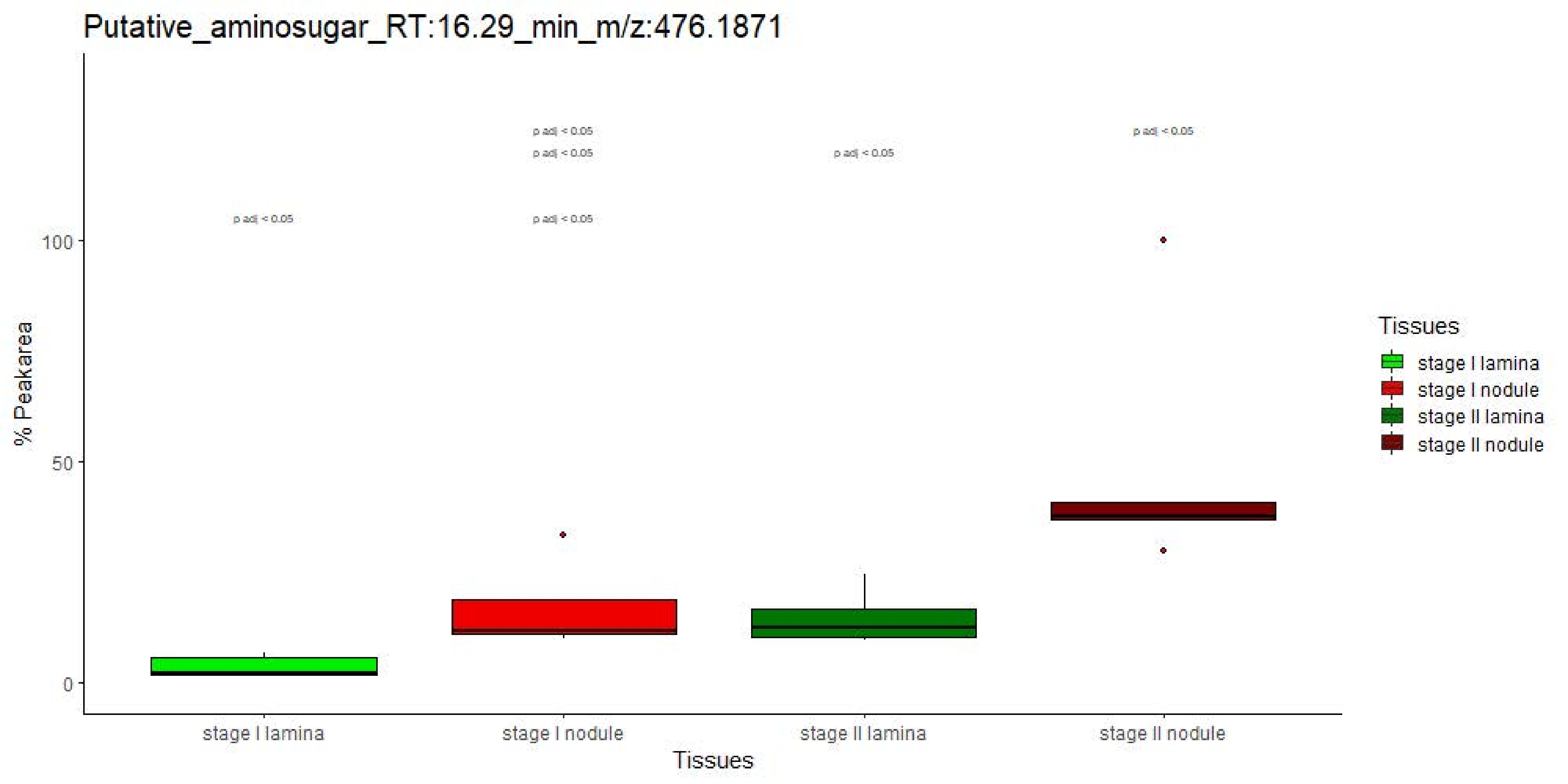

Putative_aminosugar_RT:16.29_min_m/z:476.1871
% Peakarea
100
50
0
p adj < 0.05
p adj < 0.05
p adj < 0.05
p adj < 0.05
p adj < 0.05
p adj < 0.05
stage I lamina
stage I nodule
stage II lamina
stage II nodule
Tissues
Tissues
stage I lamina
stage I nodule
stage II lamina
stage II nodule

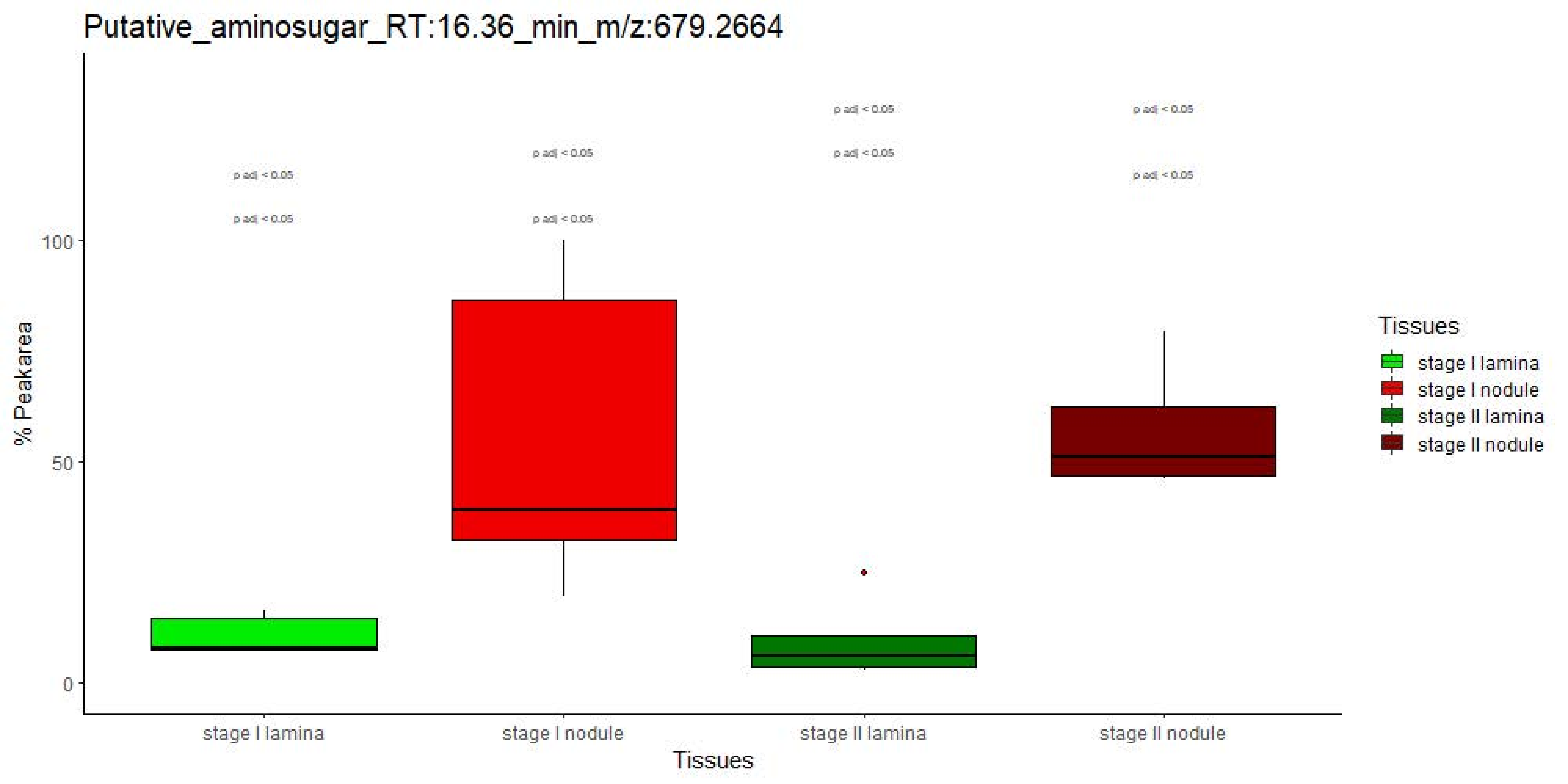
Putative_aminosugar_RT:16.36_min_m/z:679.2664
p adj < 0.05
p adj < 0.05
p adj < 0.05
p adj < 0.05
p adj < 0.05
p adj < 0.05
p adj < 0.05
p adj < 0.05
% Peakarea
100
50
0
stage I lamina
stage I nodule
stage II lamina
stage II nodule
Tissues
Tissues
stage I lamina
stage I nodule
stage II lamina
stage II nodule

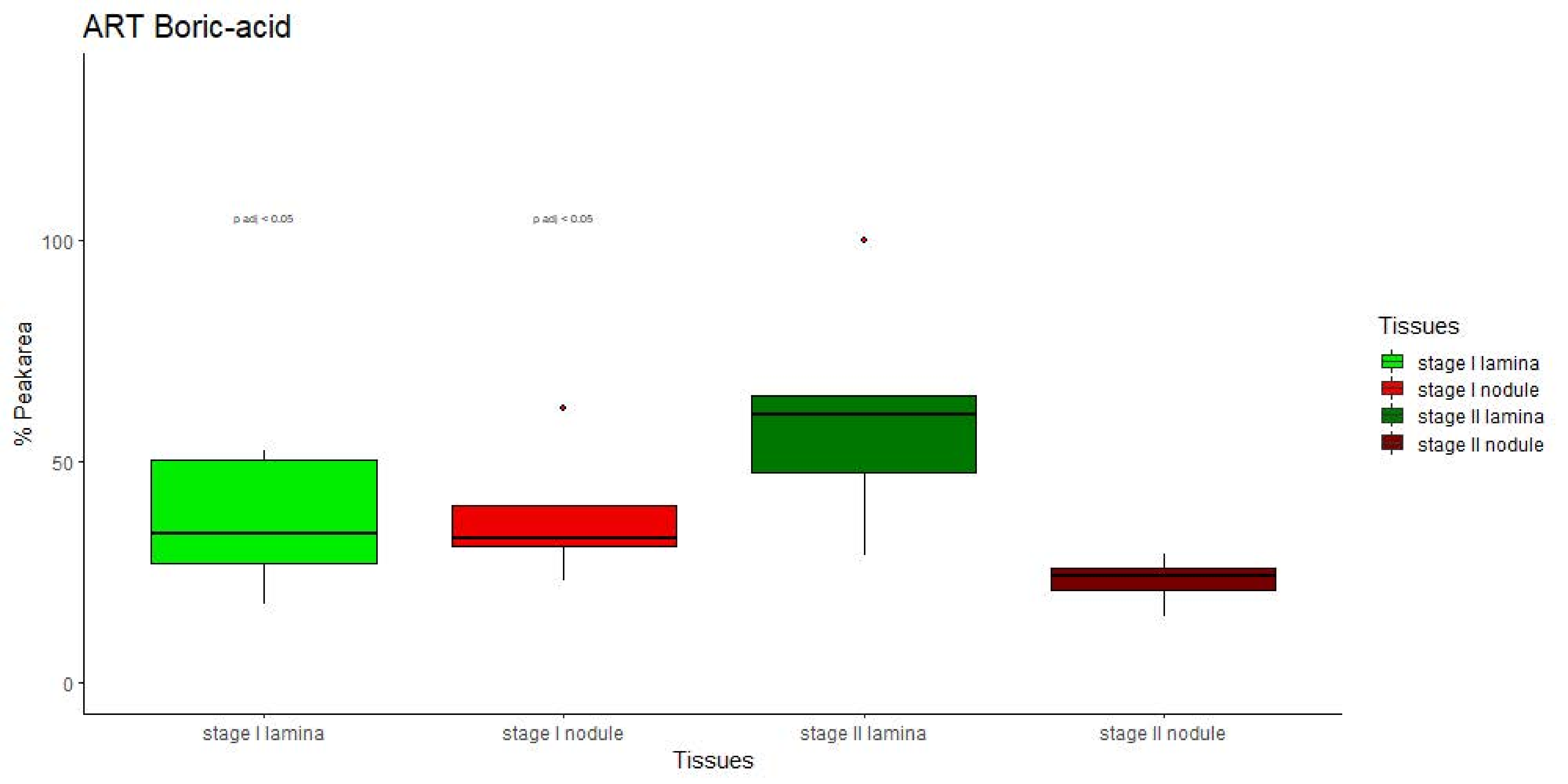
ART Boric-acid
p adj < 0.05
p adj < 0.05
100
50
0
% Peakarea
stage I lamina
stage I nodule
stage II lamina
stage II nodule
Tissues
Tissues
stage I lamina
stage I nodule
stage II lamina
stage II nodule

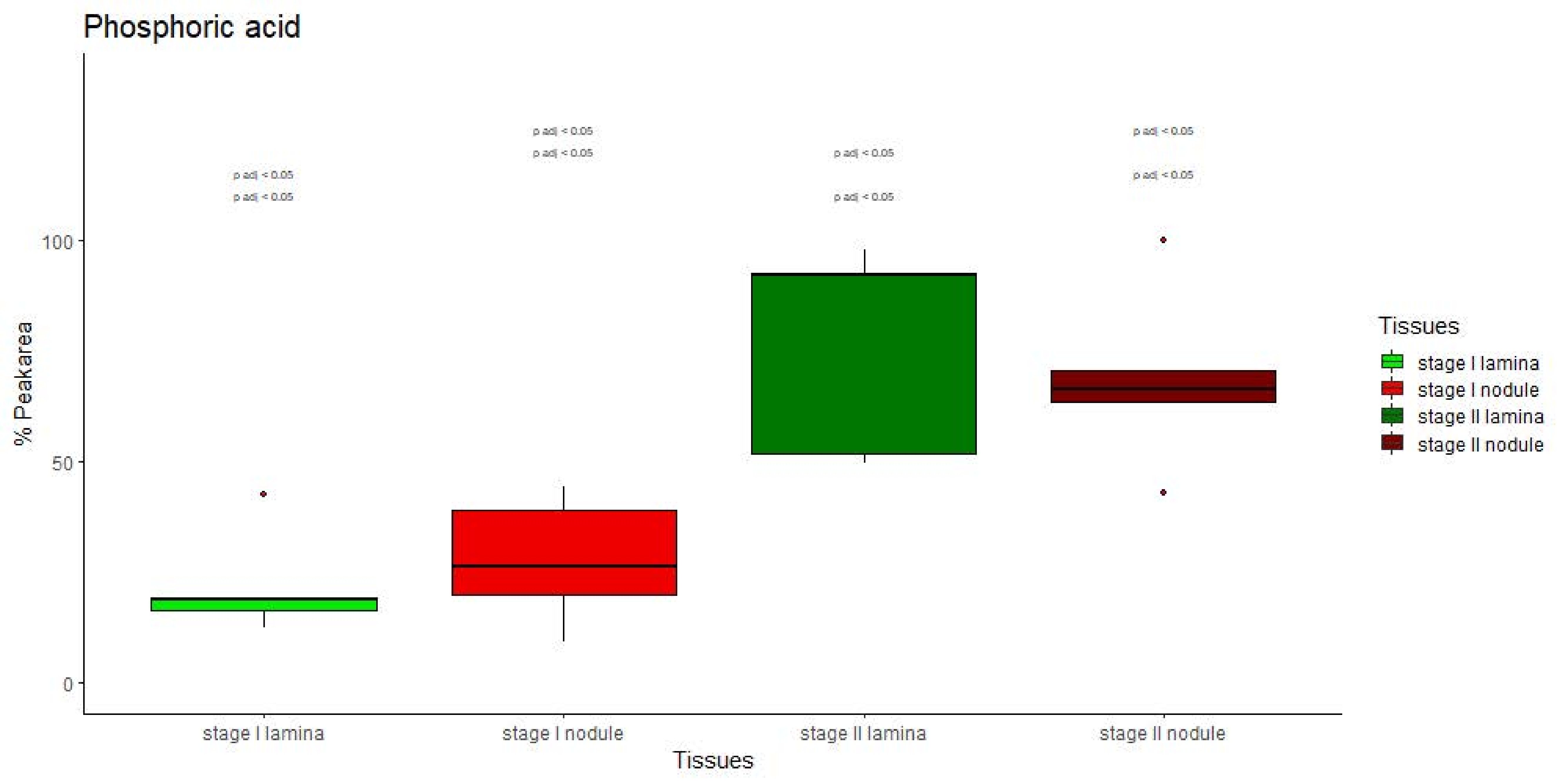

Phosphoric acid
p adj < 0.05
p adj < 0.05
p adj < 0.05
p adj < 0.05
p adj < 0.05
p adj < 0.05
p adj < 0.05
p adj < 0.05
100
50
0
% Peakarea
stage I lamina
stage I nodule
stage II lamina
stage II nodule
Tissues
Tissues
stage I lamina
stage I nodule
stage II lamina
stage II nodule

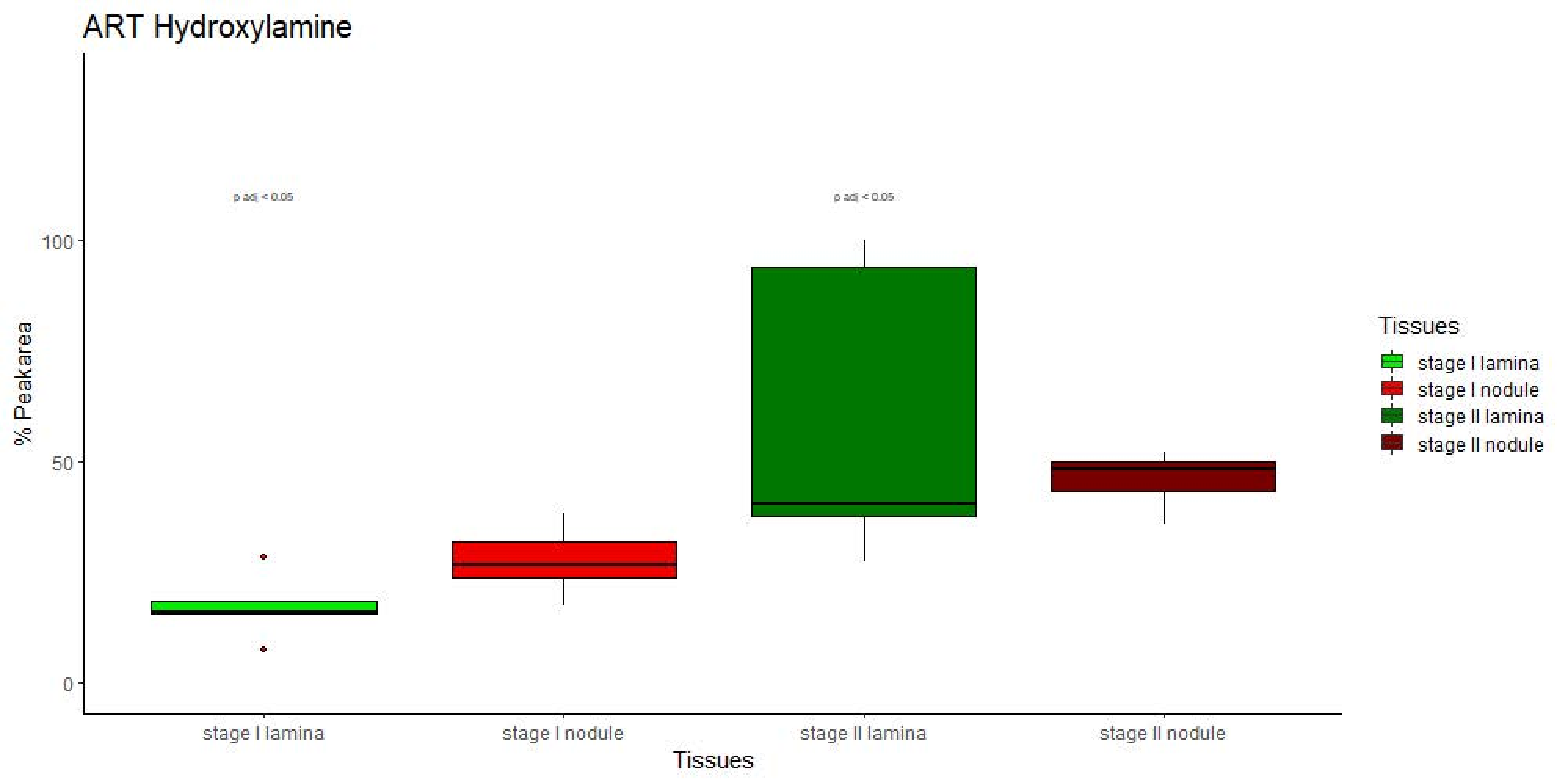

ART Hydroxylamine
p adj < 0.05
p adj < 0.05
100
50
0
% Peakarea
stage I lamina
stage I nodule
stage II lamina
stage II nodule
Tissues
Tissues
stage I lamina
stage I nodule
stage II lamina
stage II nodule

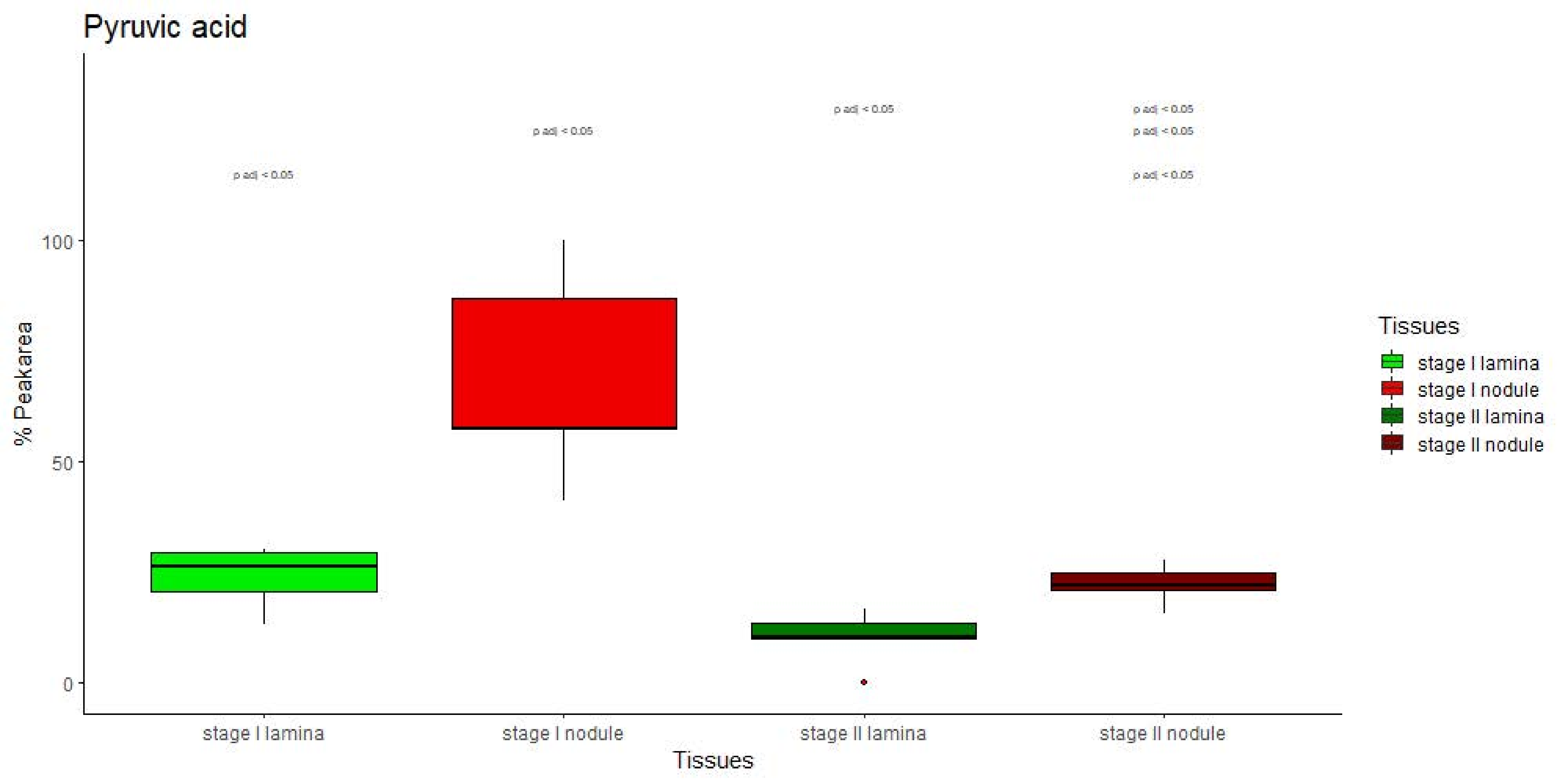

Pyruvic acid
p adj < 0.05
p adj < 0.05
p adj < 0.05
p adj < 0.05
p adj < 0.05
p adj < 0.05
100
50
0
% Peakarea
stage I lamina
stage I nodule
stage II lamina
stage II nodule
Tissues
Tissues
stage I lamina
stage I nodule
stage II lamina
stage II nodule

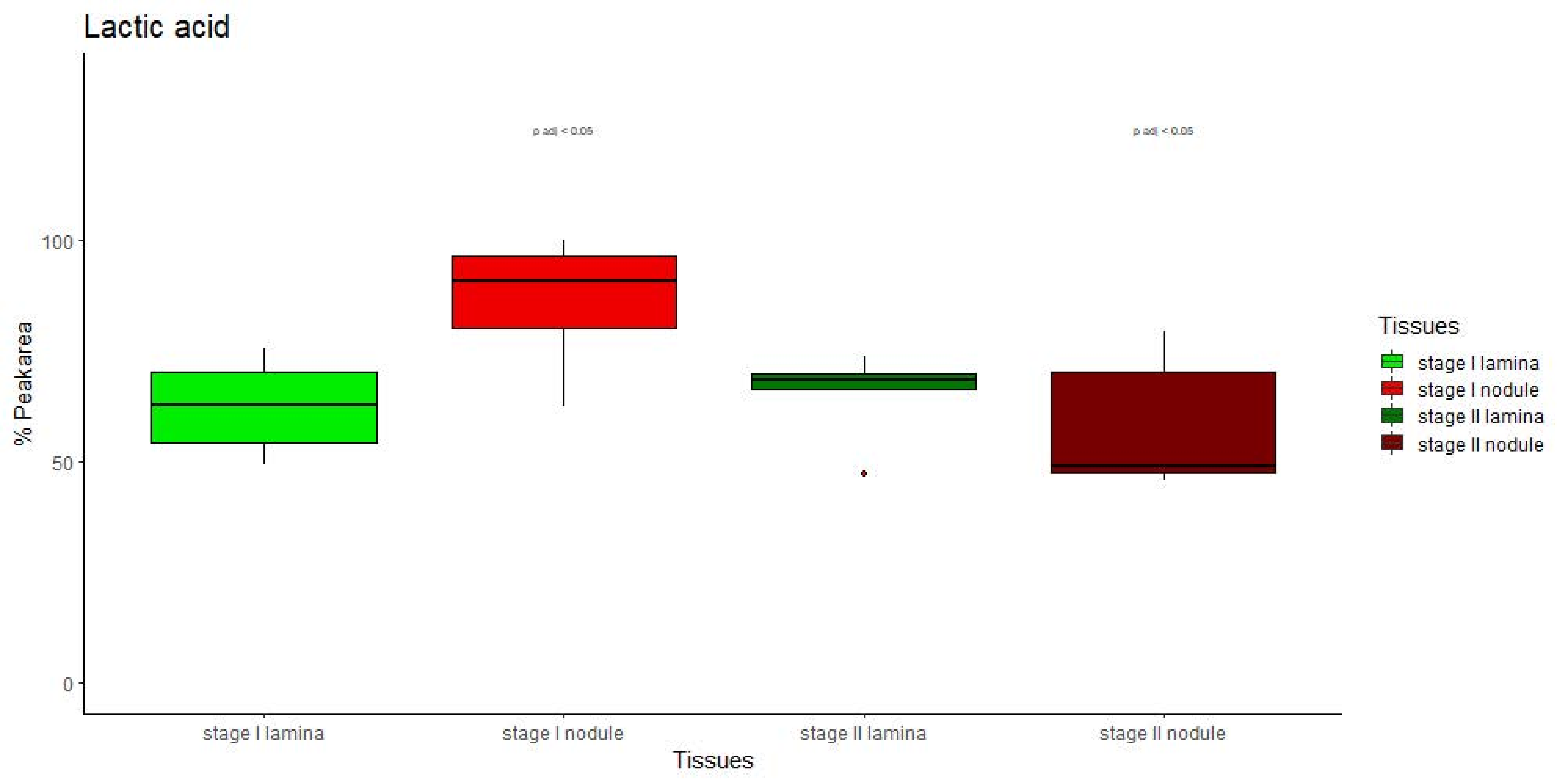
Lactic acid
p adj < 0.05
p adj < 0.05
100
50
0
% Peakarea
stage I lamina
stage I nodule
stage II lamina
stage II nodule
Tissues
Tissues
stage I lamina
stage I nodule
stage II lamina
stage II nodule

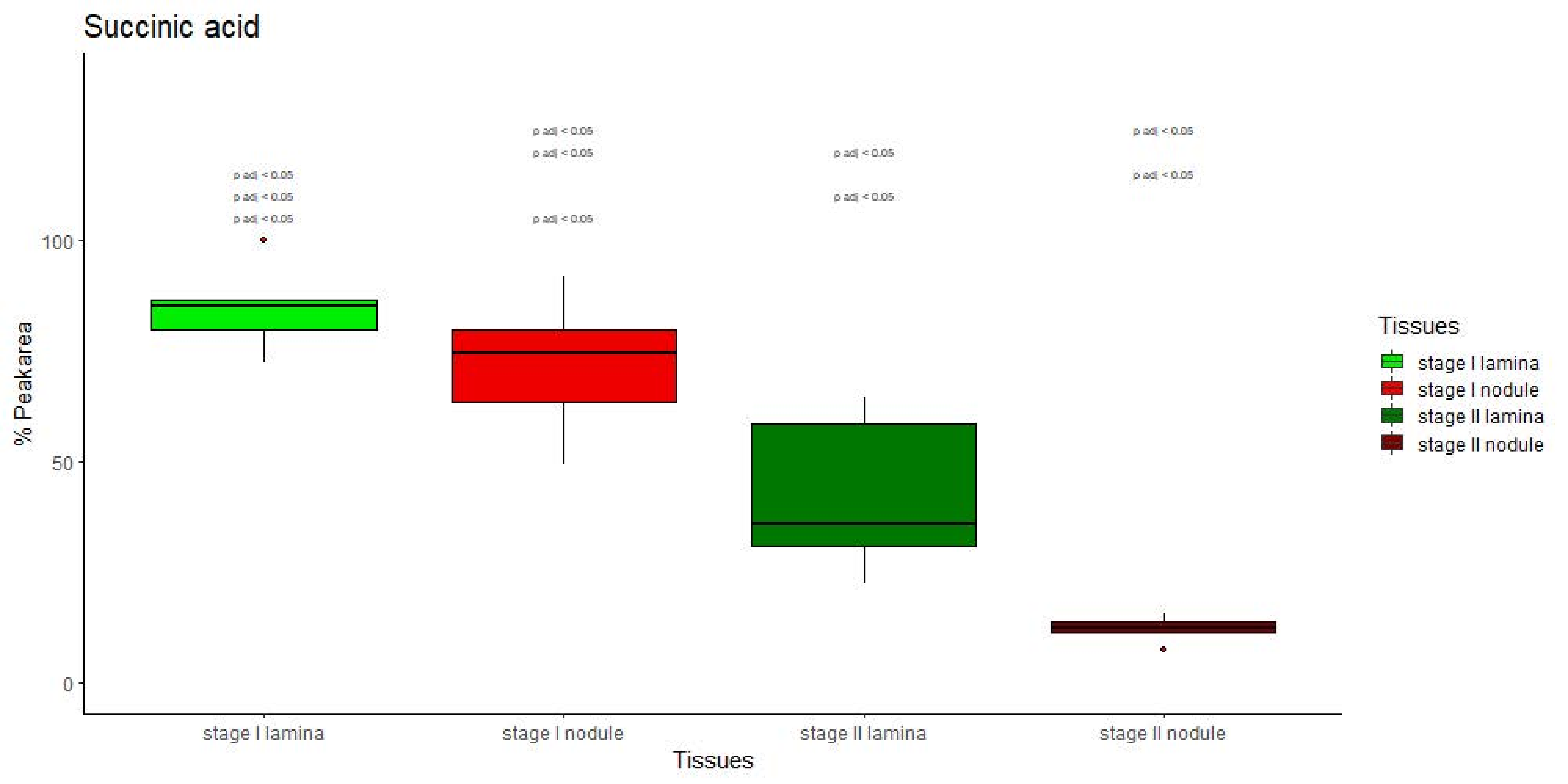

Succinic acid
p adj < 0.05
p adj < 0.05
p adj < 0.05
p adj < 0.05
p adj < 0.05
p adj < 0.05
p adj < 0.05
p adj < 0.05
p adj < 0.05
p adj < 0.05
100
50
0
% Peakarea
stage I lamina
stage I nodule
stage II lamina
stage II nodule
Tissues
Tissues
stage I lamina
stage I nodule
stage II lamina
stage II nodule

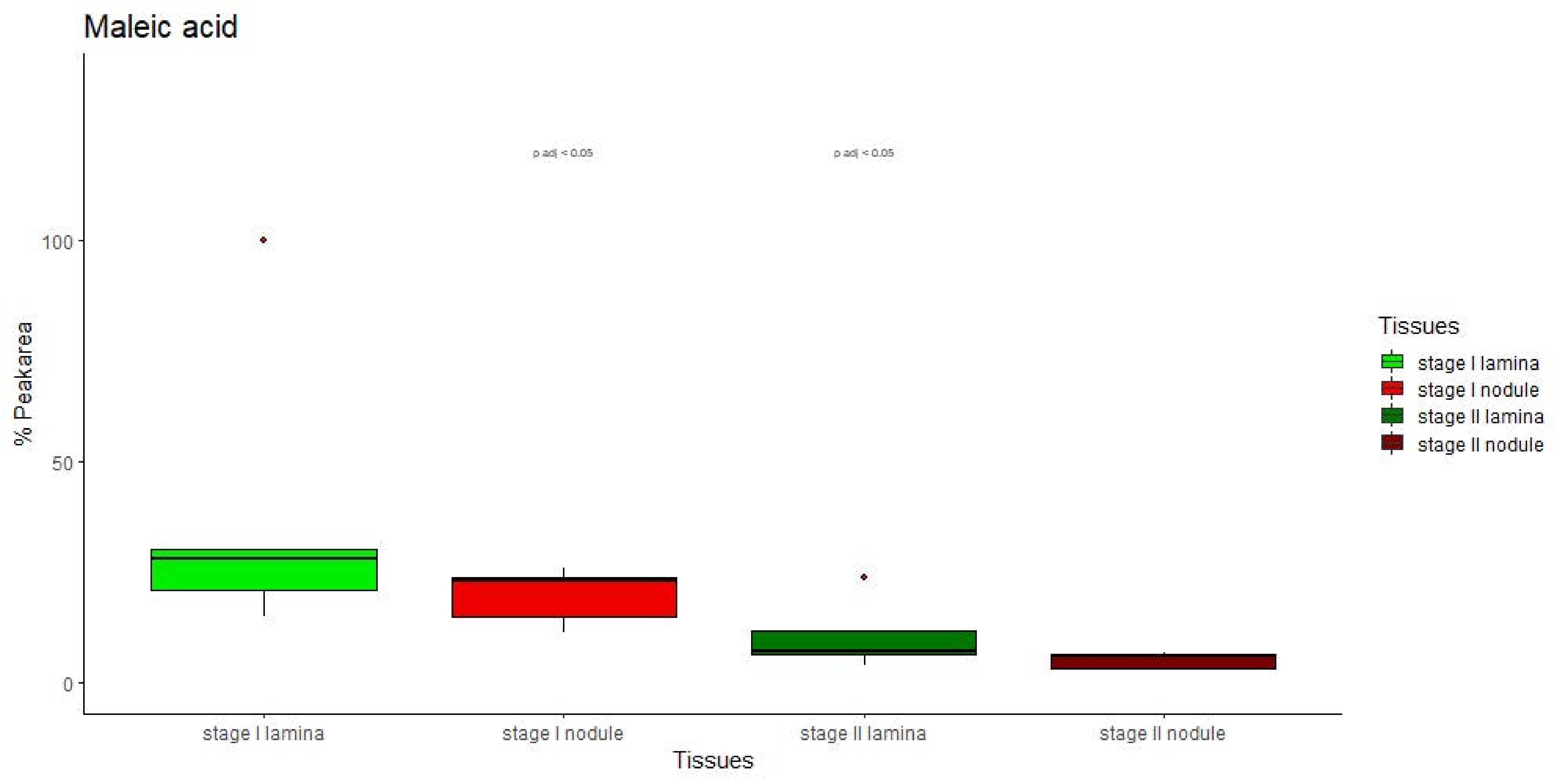

Maleic acid
p adj < 0.05
p adj < 0.05
100
50
0
% Peakarea
stage I lamina
stage I nodule
stage II lamina
stage II nodule
Tissues
Tissues
stage I lamina
stage I nodule
stage II lamina
stage II nodule

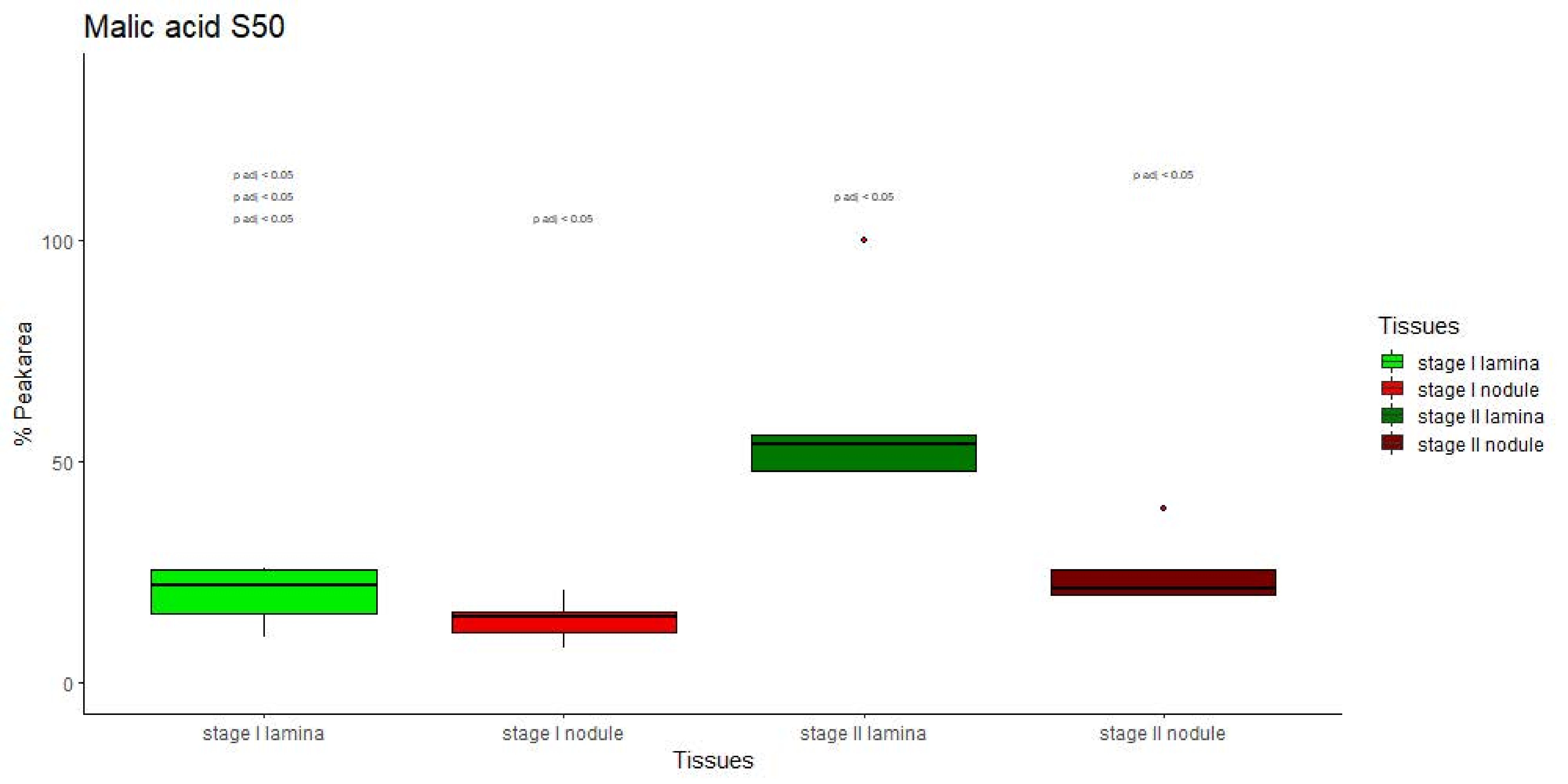

Malic acid S50
% Peakarea
100
50
0
p adj < 0.05
p adj < 0.05
p adj < 0.05
p adj < 0.05
p adj < 0.05
p adj < 0.05
stage I lamina
stage I nodule
stage II lamina
stage II nodule
Tissues
Tissues
stage I lamina
stage I nodule
stage II lamina
stage II nodule

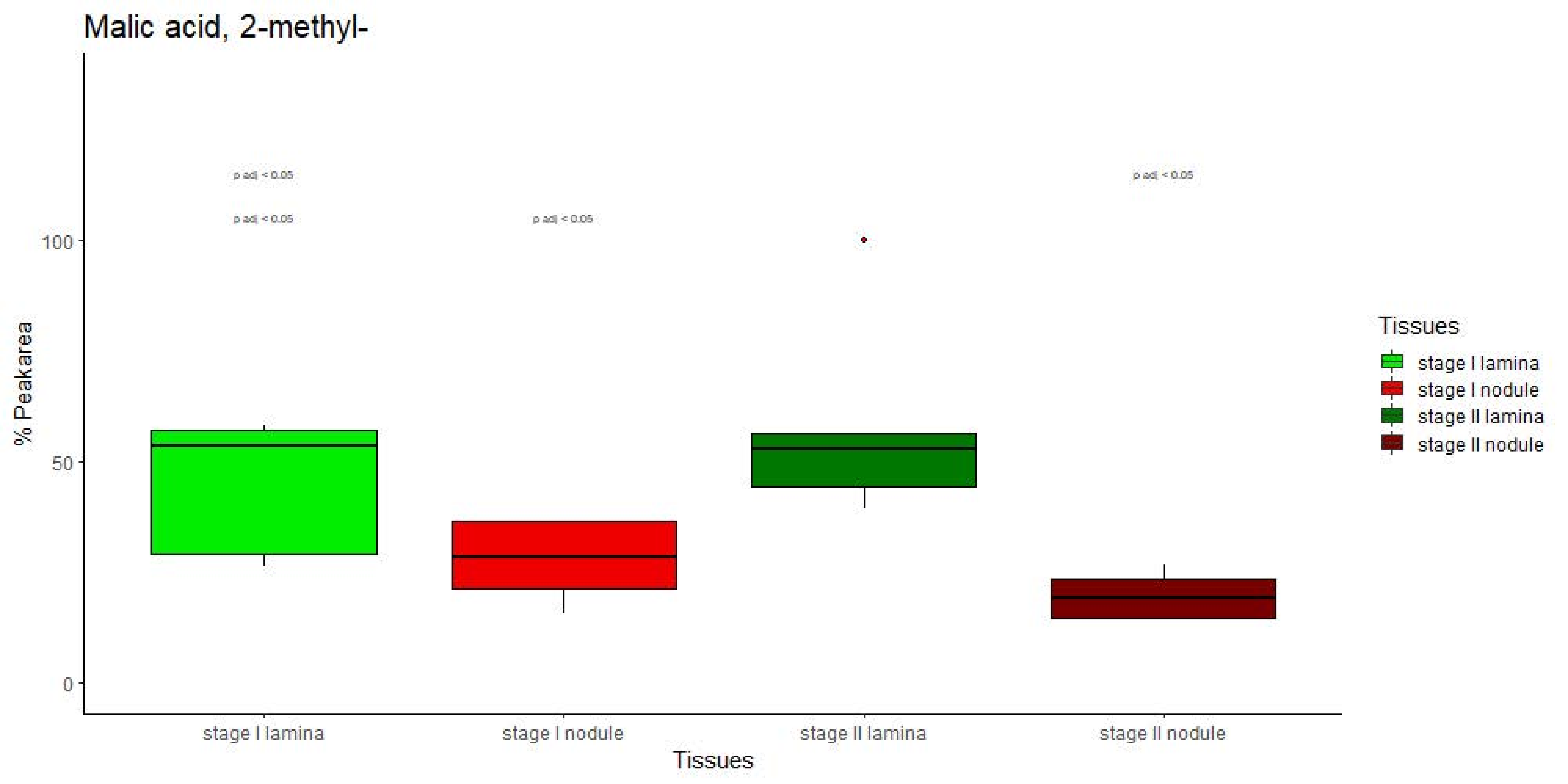

Malic acid, 2-methyl-
p adj < 0.05
p adj < 0.05
p adj < 0.05
p adj < 0.05
100
50
0
% Peakarea
stage I lamina
stage I nodule
stage II lamina
stage II nodule
Tissues
Tissues
stage I lamina
stage I nodule
stage II lamina
stage II nodule

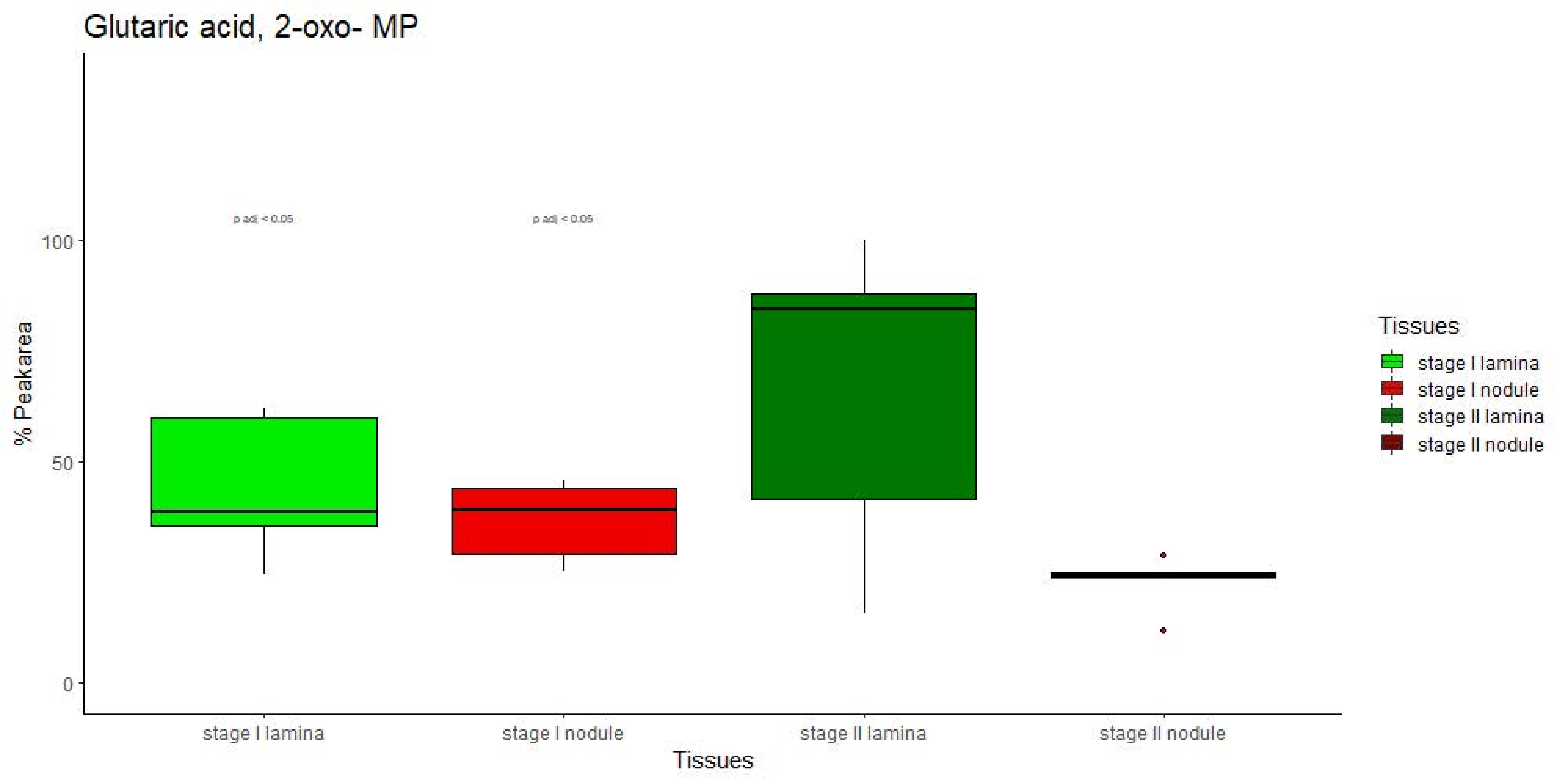
Glutaric acid, 2-oxo- MP
p adj < 0.05
p adj < 0.05
100
50
0
% Peakarea
stage I lamina
stage I nodule
stage II lamina
stage II nodule
Tissues
Tissues
stage I lamina
stage I nodule
stage II lamina
stage II nodule

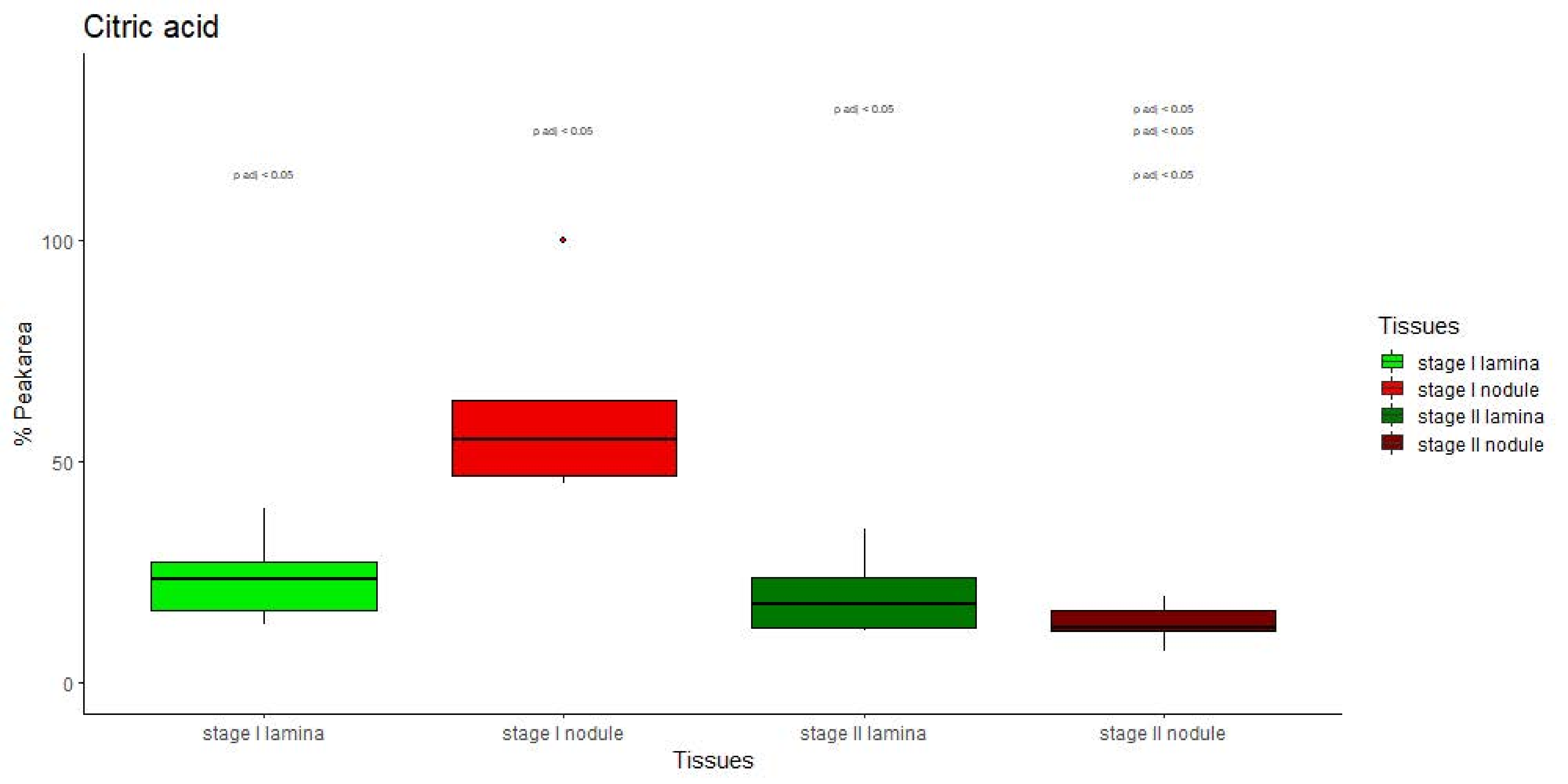

Citric acid
p adj < 0.05
p adj < 0.05
p adj < 0.05
p adj < 0.05
p adj < 0.05
p adj < 0.05
100
50
0
% Peakarea
stage I lamina
stage I nodule
stage II lamina
stage II nodule
Tissues
Tissues
stage I lamina
stage I nodule
stage II lamina
stage II nodule

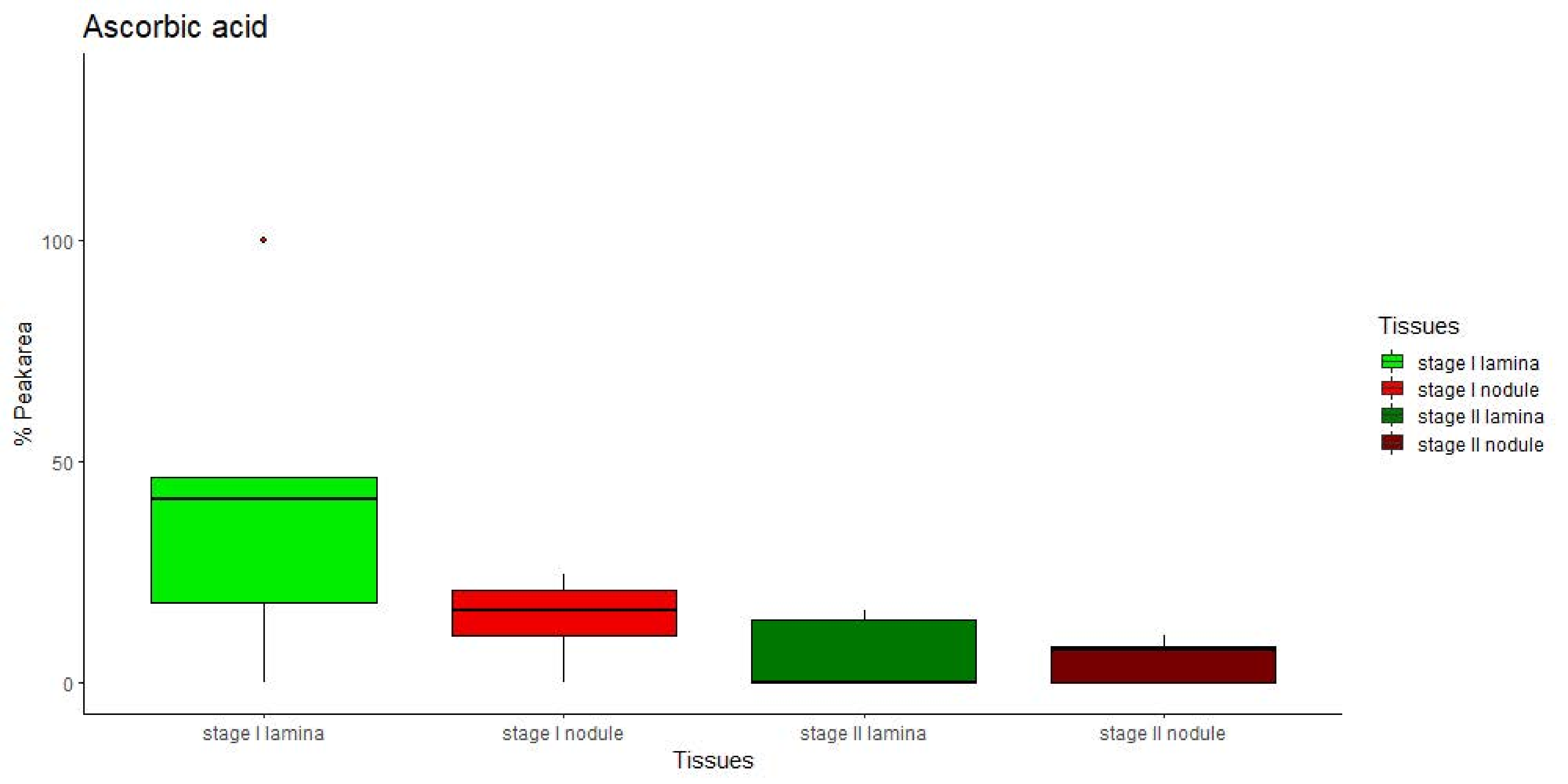
Ascorbic acid
% Peakarea
100
50
0
stage I lamina
stage I nodule
stage II lamina
stage II nodule
Tissues
Tissues
stage I lamina
stage I nodule
stage II lamina
stage II nodule

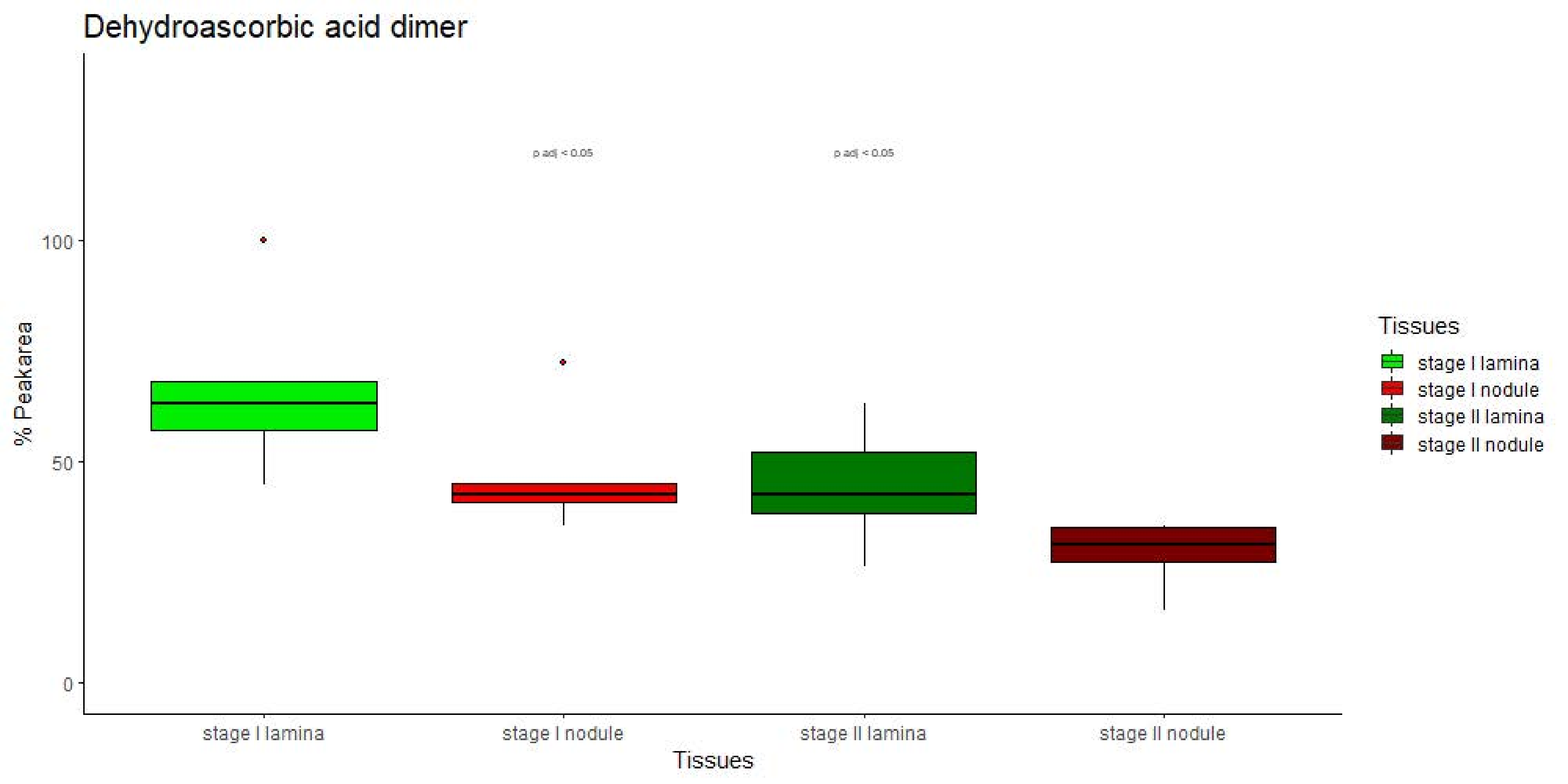

Dehydroascorbic acid dimer
p adj < 0.05
p adj < 0.05
100
50
0
% Peakarea
stage I lamina
stage I nodule
stage II lamina
stage II nodule
Tissues
Tissues
stage I lamina
stage I nodule
stage II lamina
stage II nodule

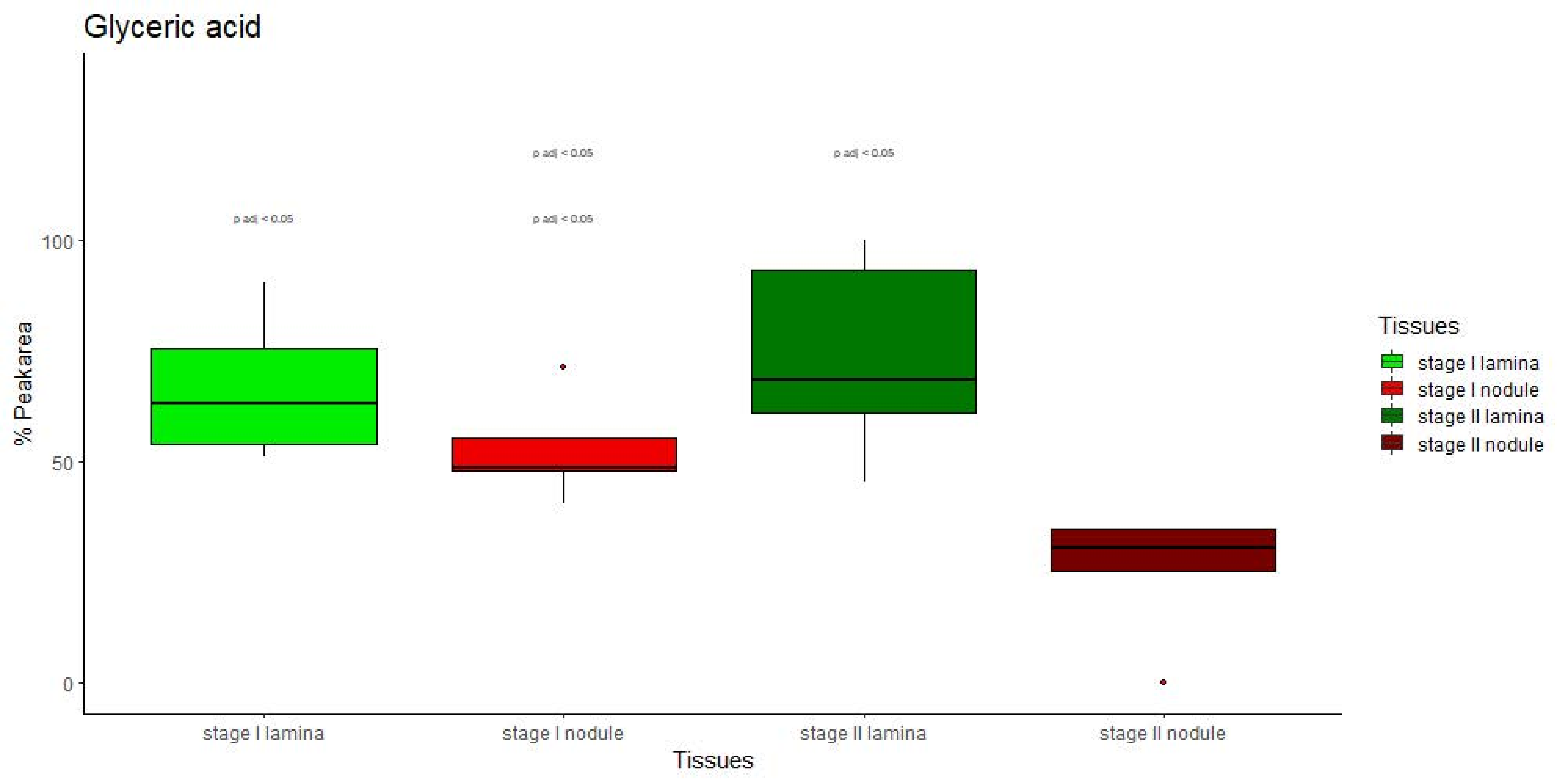
Glyceric acid
% Peakarea
100
50
0
p adj < 0.05
p adj < 0.05
p adj < 0.05
p adj < 0.05
stage I lamina
stage I nodule
stage II lamina
stage II nodule
Tissues
Tissues
stage I lamina
stage I nodule
stage II lamina
stage II nodule

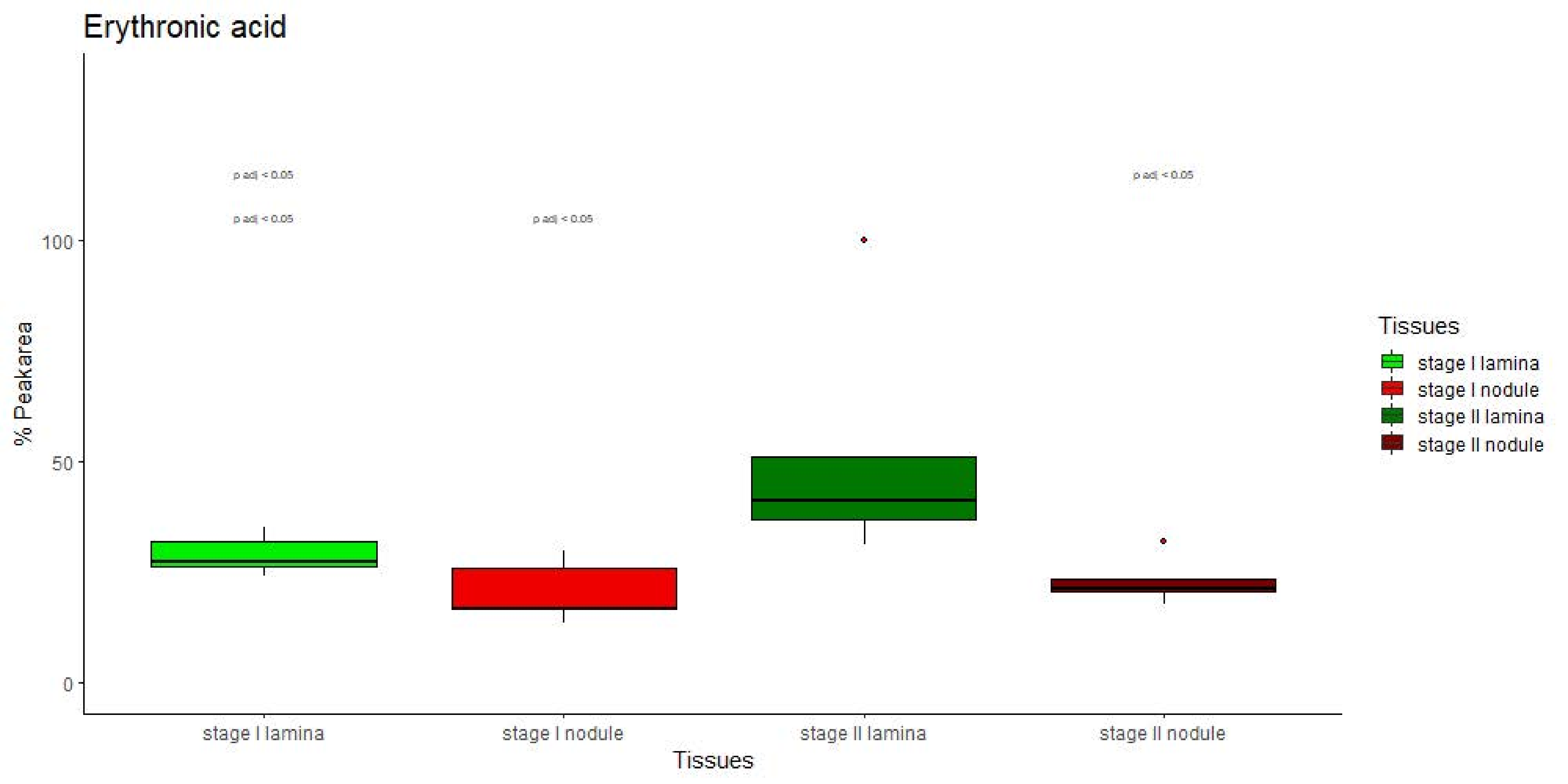
Erythronic acid
p adj < 0.05
p adj < 0.05
p adj < 0.05
p adj < 0.05
100
50
0
% Peakarea
stage I lamina
stage I nodule
stage II lamina
stage II nodule
Tissues
Tissues
stage I lamina
stage I nodule
stage II lamina
stage II nodule

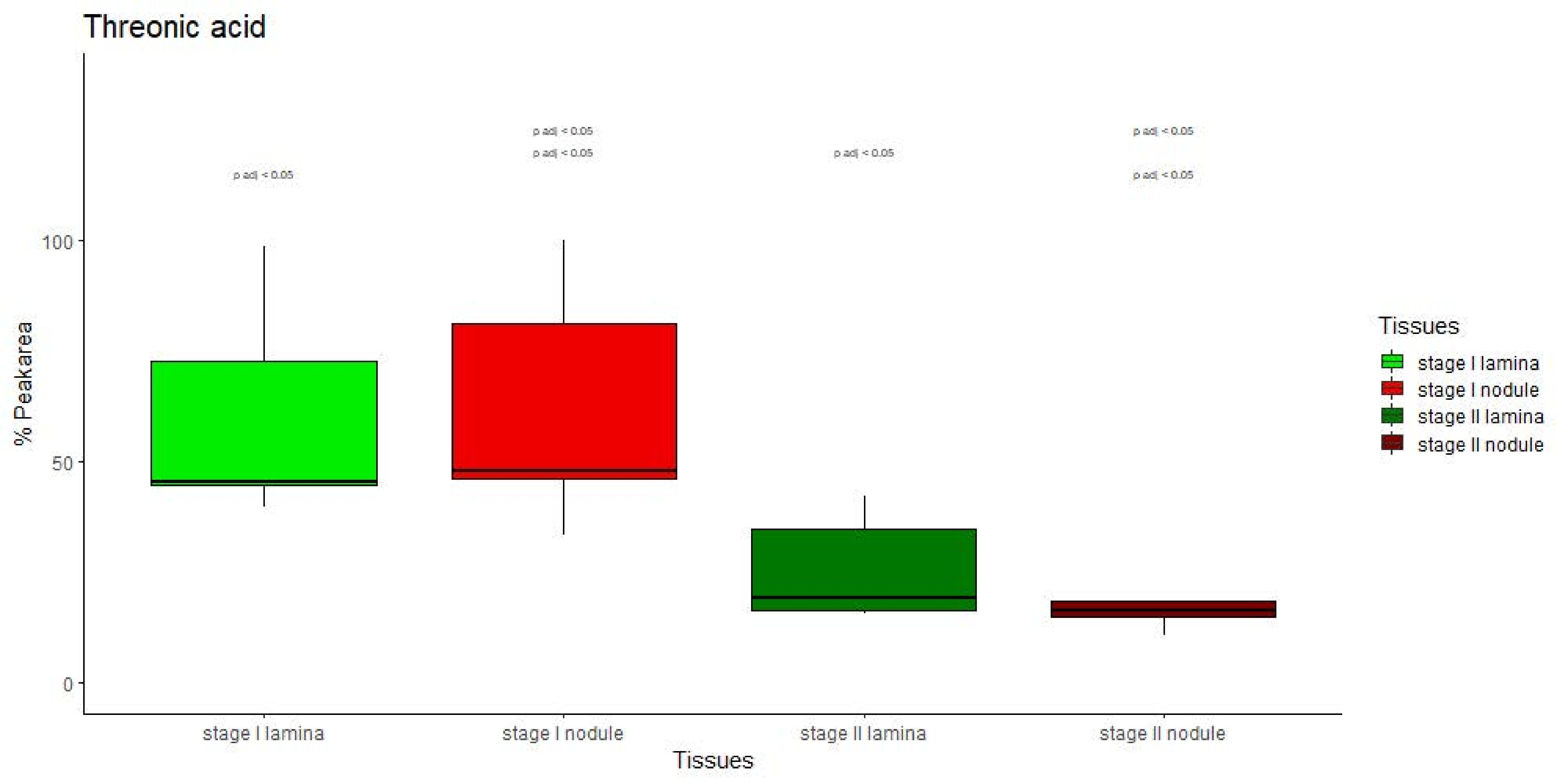
Threonic acid
p adj < 0.05
p adj < 0.05
p adj < 0.05
p adj < 0.05
p adj < 0.05
p adj < 0.05
100
50
0
% Peakarea
stage I lamina
stage I nodule
stage II lamina
stage II nodule
Tissues
Tissues
stage I lamina
stage I nodule
stage II lamina
stage II nodule

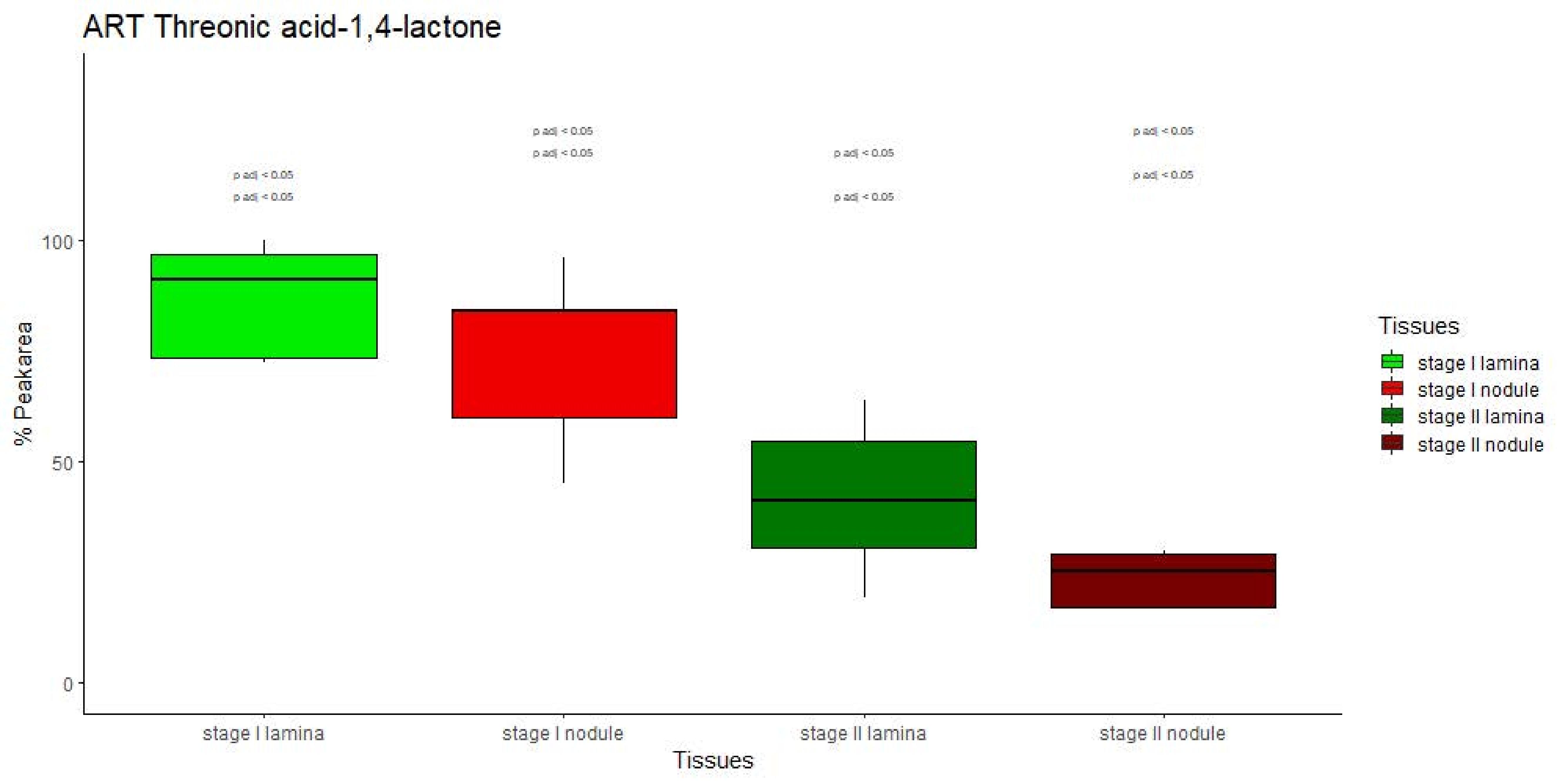

ART Threonic acid-1,4-lactone
p adj < 0.05
p adj < 0.05
p adj < 0.05
p adj < 0.05
p adj < 0.05
p adj < 0.05
p adj < 0.05
p adj < 0.05
100
50
0
% Peakarea
stage I lamina
stage I nodule
stage II lamina
stage II nodule
Tissues
Tissues
stage I lamina
stage I nodule
stage II lamina
stage II nodule

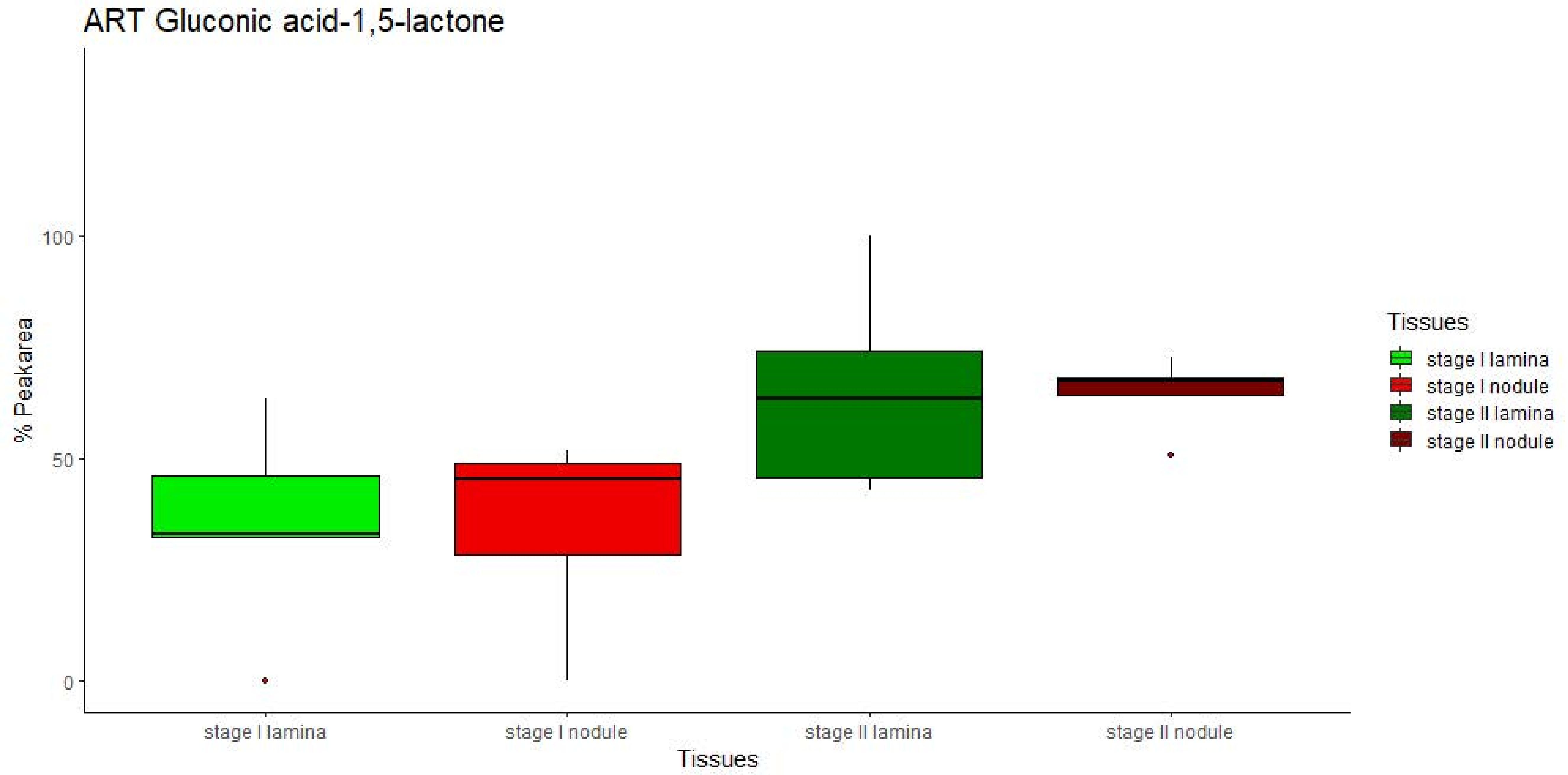

ART Gluconic acid-1,5-lactone
% Peakarea
100
50
0
stage I lamina
stage I nodule
stage II lamina
stage II nodule
Tissues
Tissues
stage I lamina
stage I nodule
stage II lamina
stage II nodule

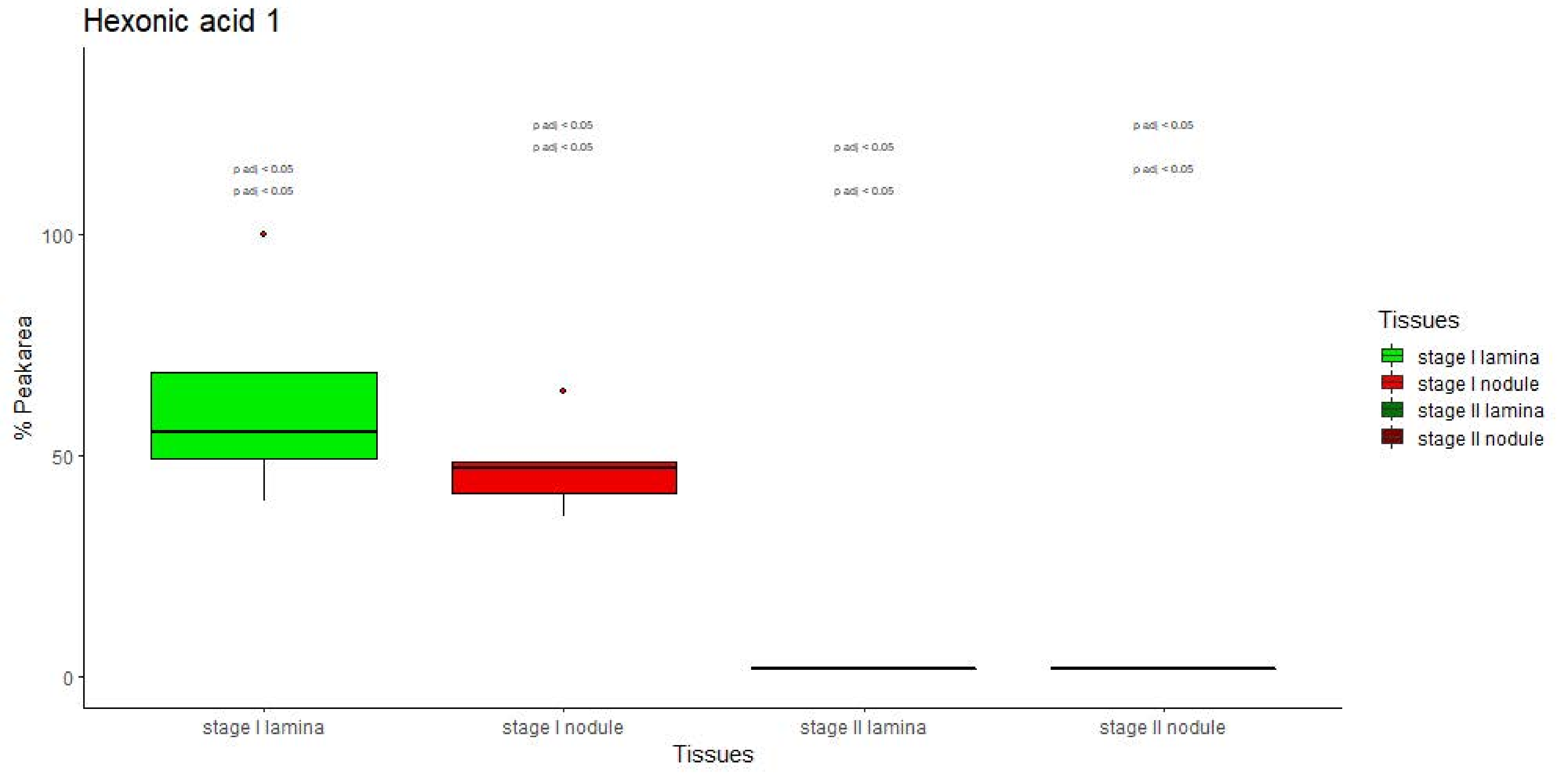
Hexonic acid 1
p adj < 0.05
p adj < 0.05
p adj < 0.05
p adj < 0.05
p adj < 0.05
p adj < 0.05
p adj < 0.05
p adj < 0.05
100
50
0
% Peakarea
stage I lamina
stage I nodule
stage II lamina
stage II nodule
Tissues
Tissues
stage I lamina
stage I nodule
stage II lamina
stage II nodule

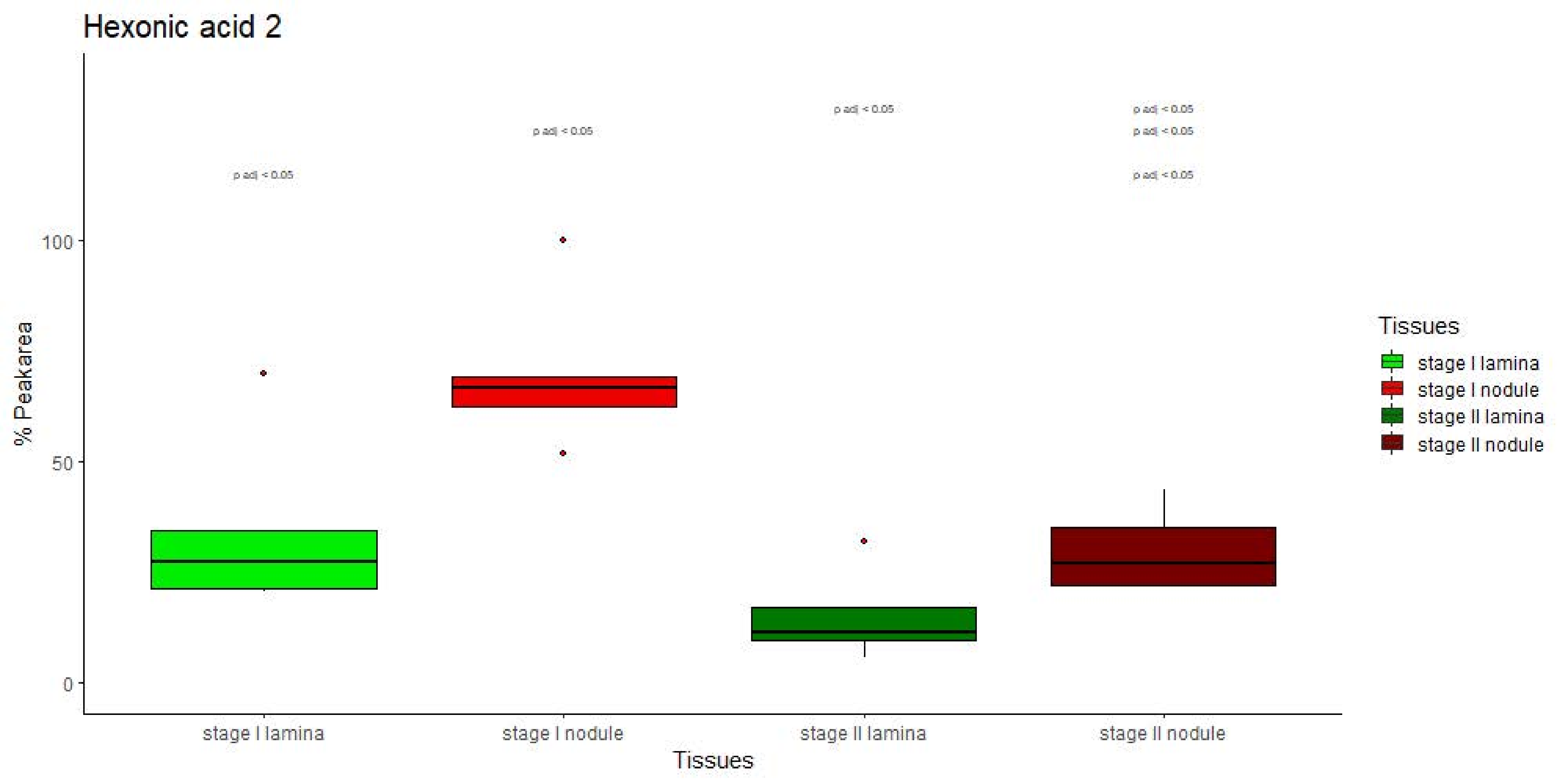

Hexonic acid 2
p adj < 0.05
p adj < 0.05
p adj < 0.05
p adj < 0.05
p adj < 0.05
p adj < 0.05
100
50
0
% Peakarea
stage I lamina
stage I nodule
stage II lamina
stage II nodule
Tissues
Tissues
stage I lamina
stage I nodule
stage II lamina
stage II nodule

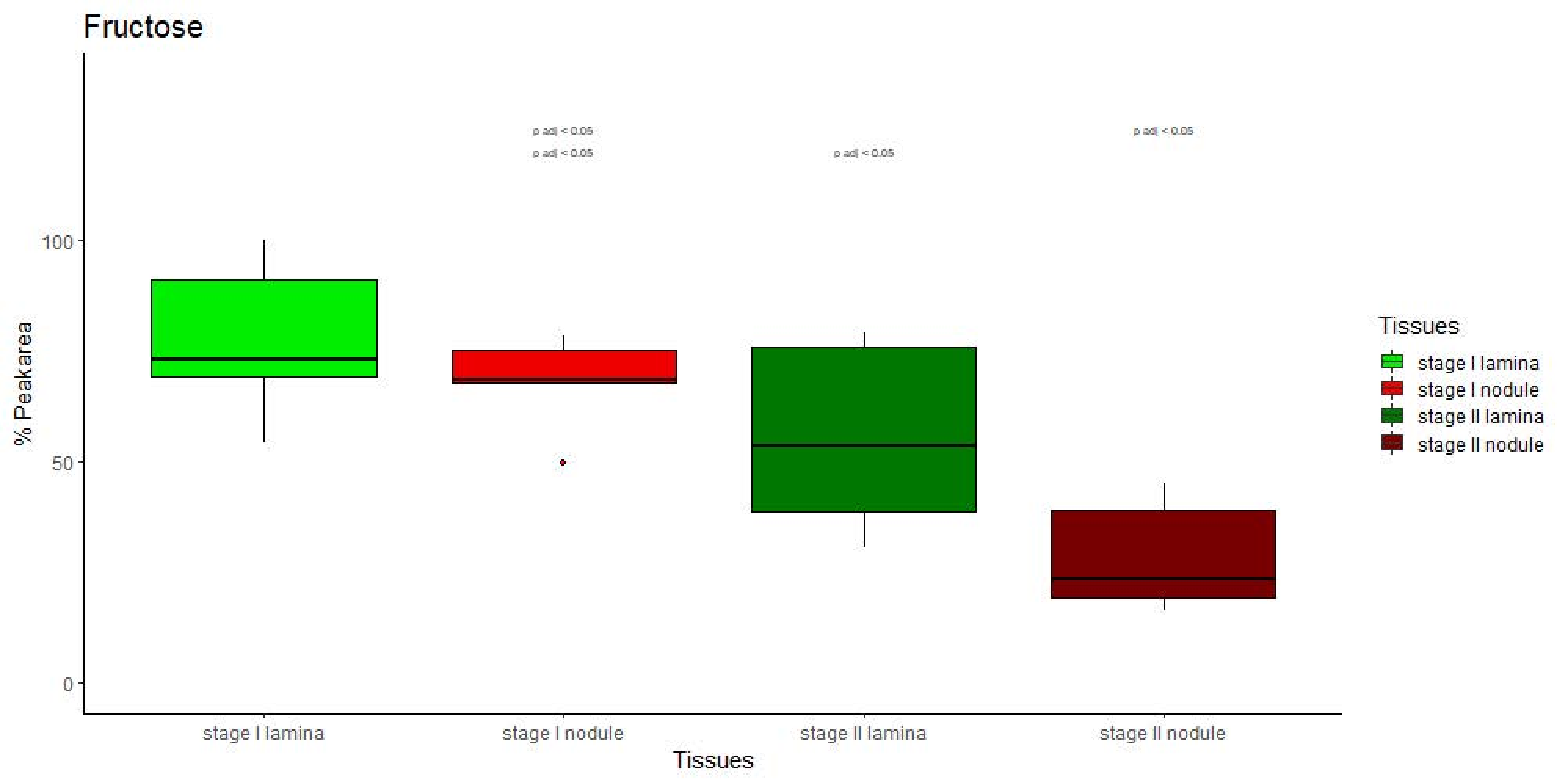

Fructose
p adj < 0.05
p adj < 0.05
p adj < 0.05
p adj < 0.05
% Peakarea
100
50
0
stage I lamina
stage I nodule
stage II lamina
stage II nodule
Tissues
Tissues
stage I lamina
stage I nodule
stage II lamina
stage II nodule

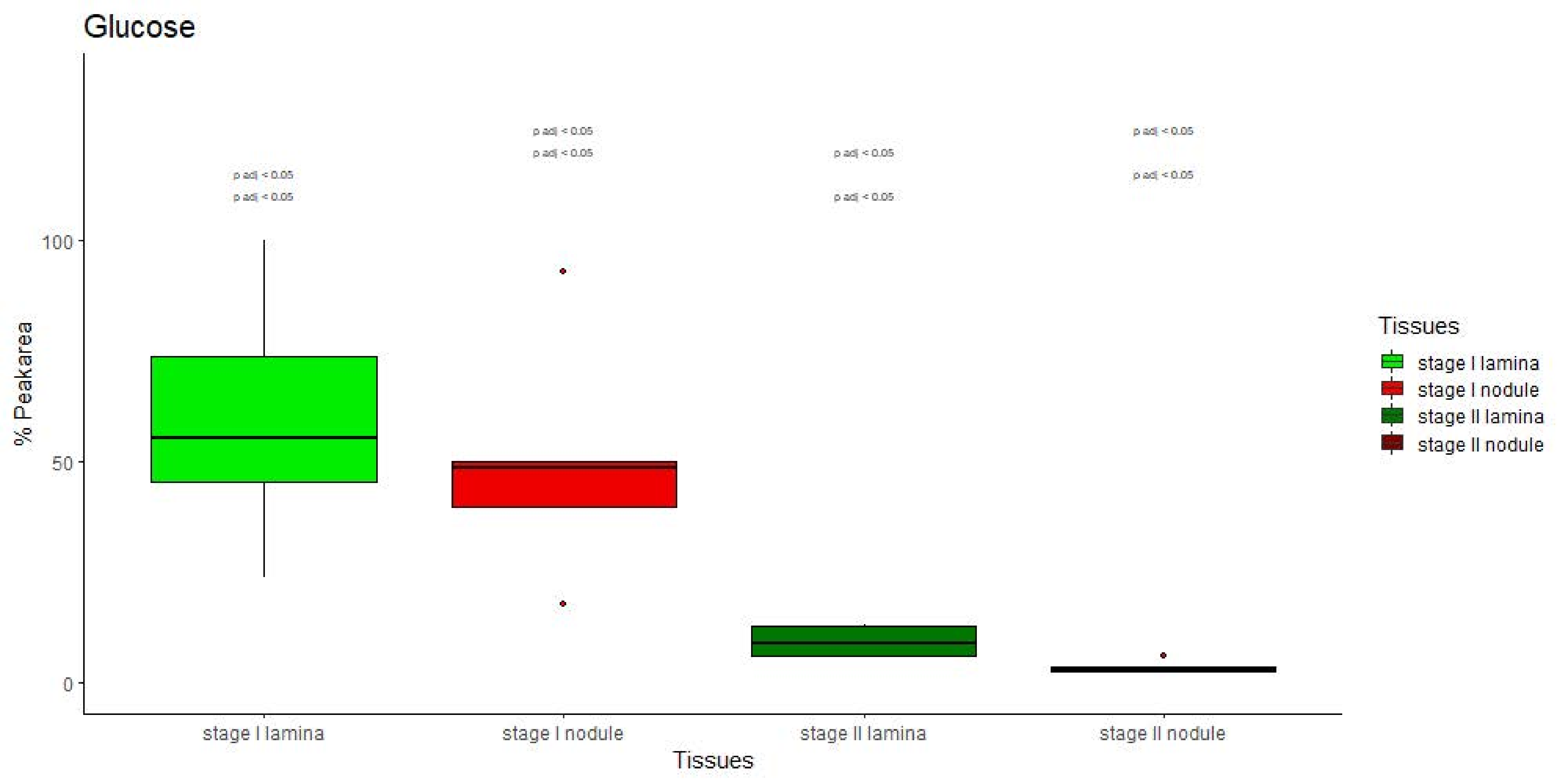
Glucose
p adj < 0.05
p adj < 0.05
p adj < 0.05
p adj < 0.05
p adj < 0.05
p adj < 0.05
p adj < 0.05
p adj < 0.05
100
50
0
% Peakarea
stage I lamina
stage I nodule
stage II lamina
stage II nodule
Tissues
Tissues
stage I lamina
stage I nodule
stage II lamina
stage II nodule

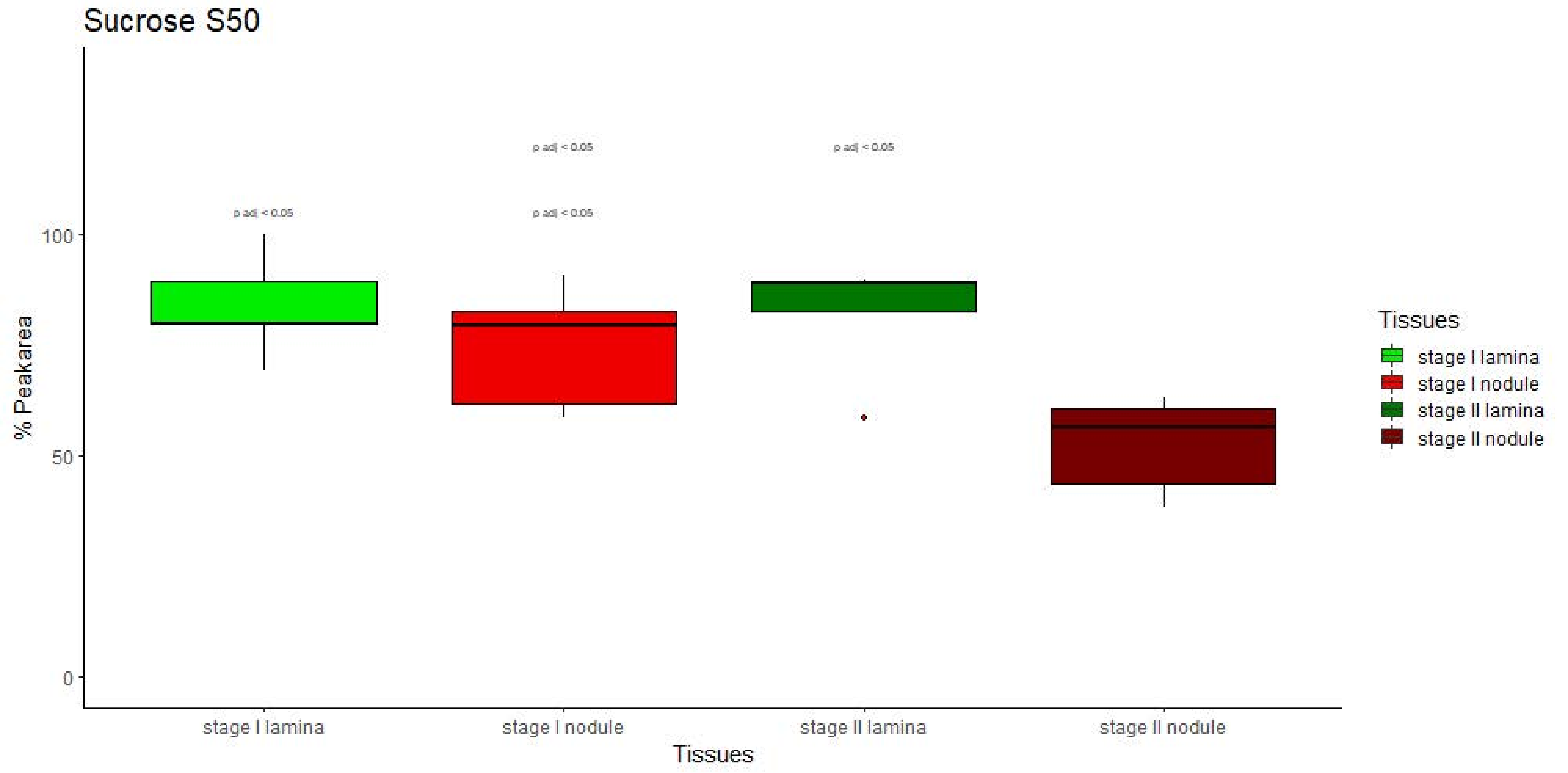

Sucrose S50
p adj < 0.05
p adj < 0.05
p adj < 0.05
p adj < 0.05
100
50
0
% Peakarea
stage I lamina
stage I nodule
stage II lamina
stage II nodule
Tissues
Tissues
stage I lamina
stage I nodule
stage II lamina
stage II nodule

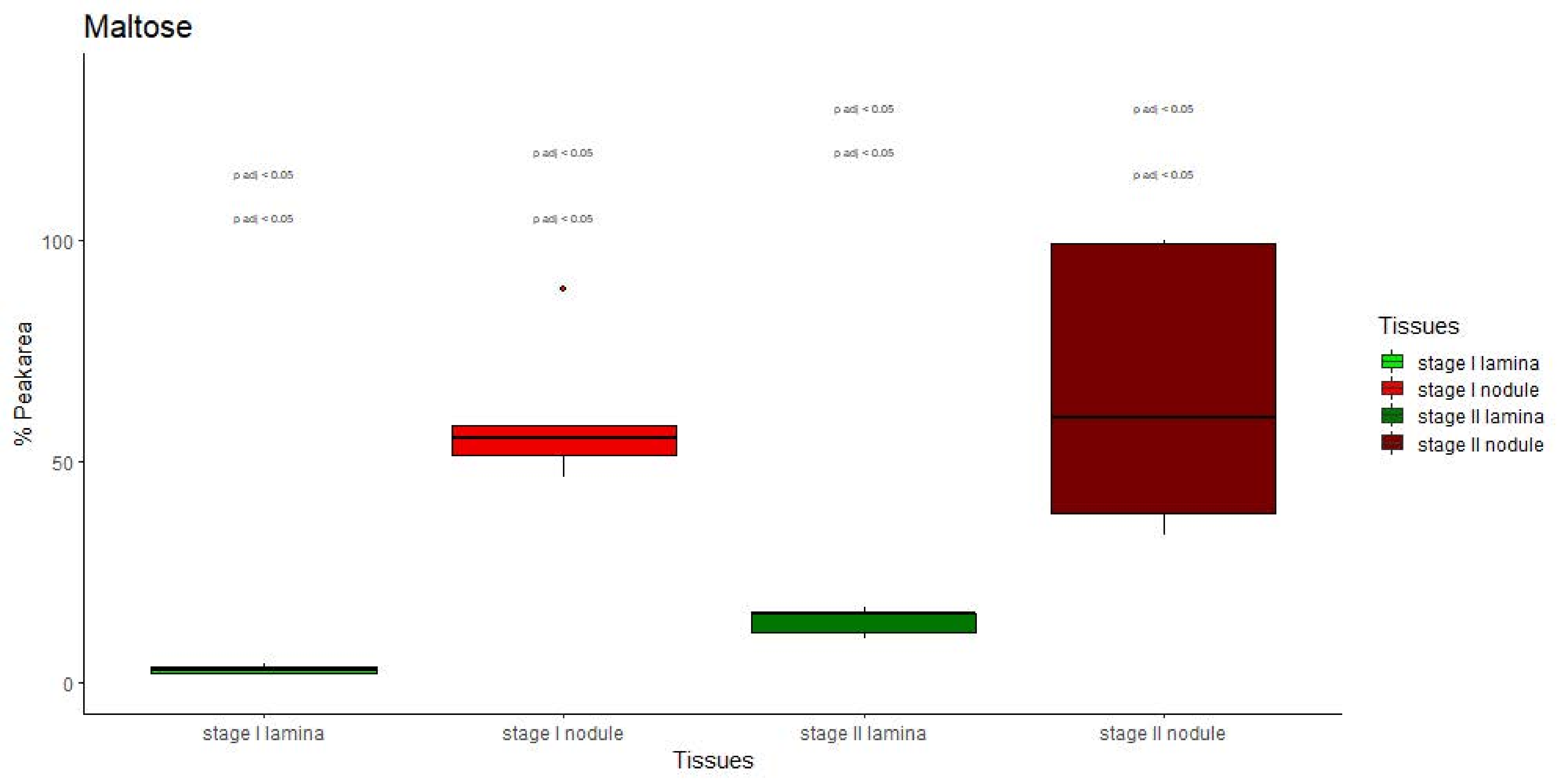
Maltose
p adj < 0.05
p adj < 0.05
p adj < 0.05
p adj < 0.05
p adj < 0.05
p adj < 0.05
p adj < 0.05
p adj < 0.05
100
50
0
% Peakarea
stage I lamina
stage I nodule
stage II lamina
stage II nodule
Tissues
Tissues
stage I lamina
stage I nodule
stage II lamina
stage II nodule

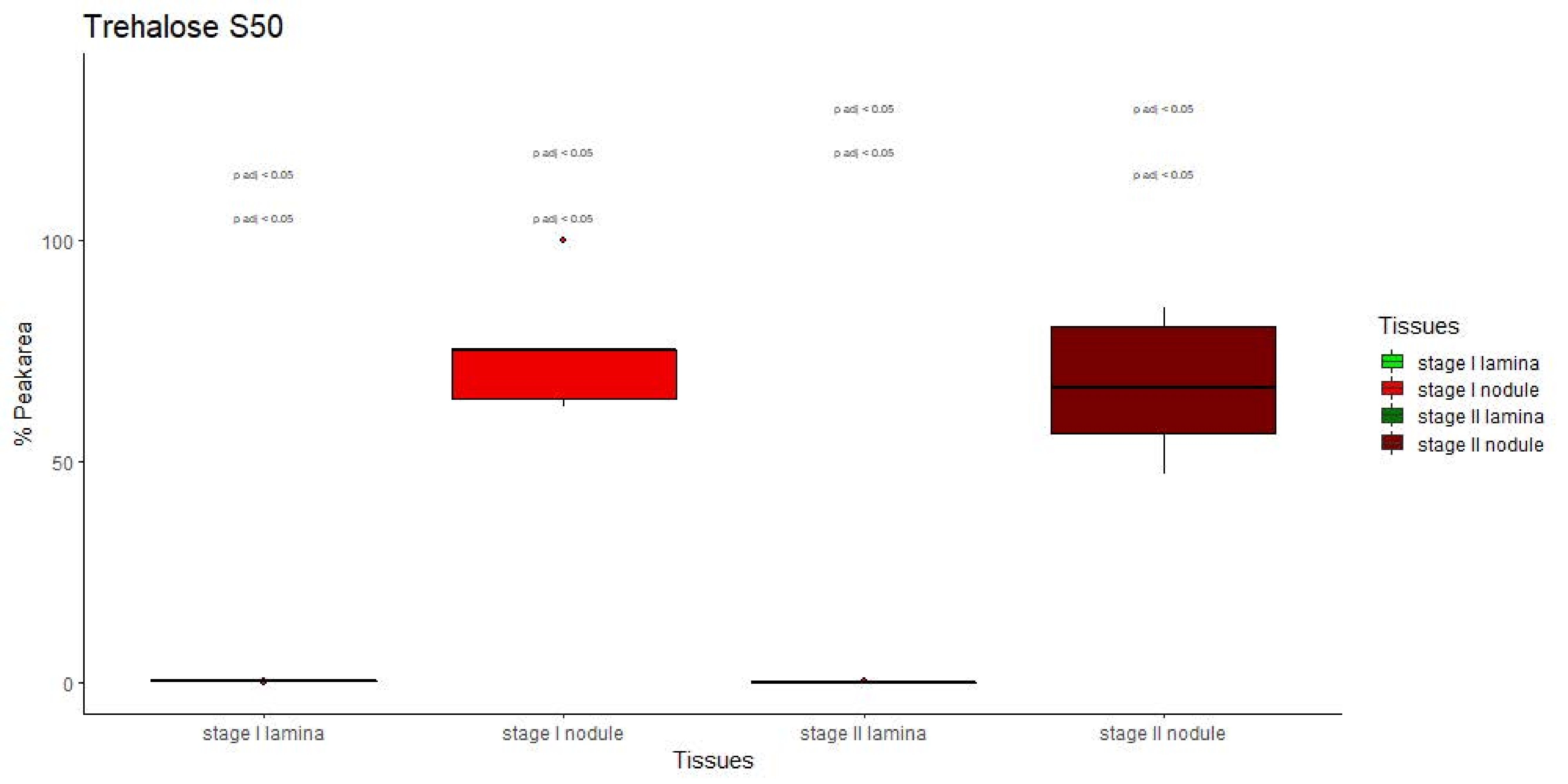
Trehalose S50
% Peakarea
100
50
0
p adj < 0.05
p adj < 0.05
p adj < 0.05
p adj < 0.05
p adj < 0.05
p adj < 0.05
p adj < 0.05
p adj < 0.05
stage I lamina
stage I nodule
stage II lamina
stage II nodule
Tissues
Tissues
stage I lamina
stage I nodule
stage II lamina
stage II nodule

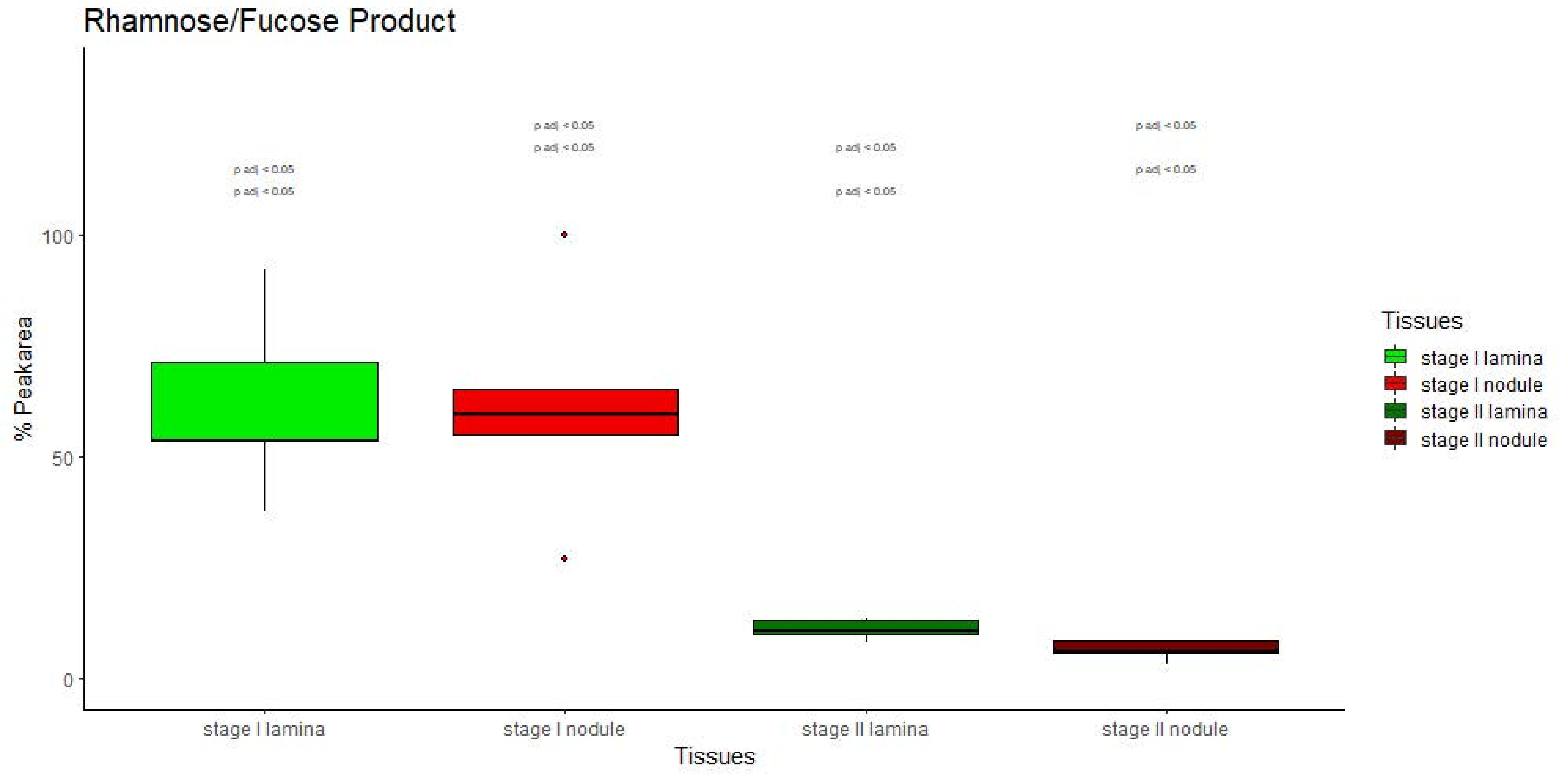
Rhamnose/Fucose Product
p adj < 0.05
p adj < 0.05
p adj < 0.05
p adj < 0.05
p adj < 0.05
p adj < 0.05
p adj < 0.05
p adj < 0.05
100
50
0
% Peakarea
stage I lamina
stage I nodule
stage II lamina
stage II nodule
Tissues
Tissues
stage I lamina
stage I nodule
stage II lamina
stage II nodule

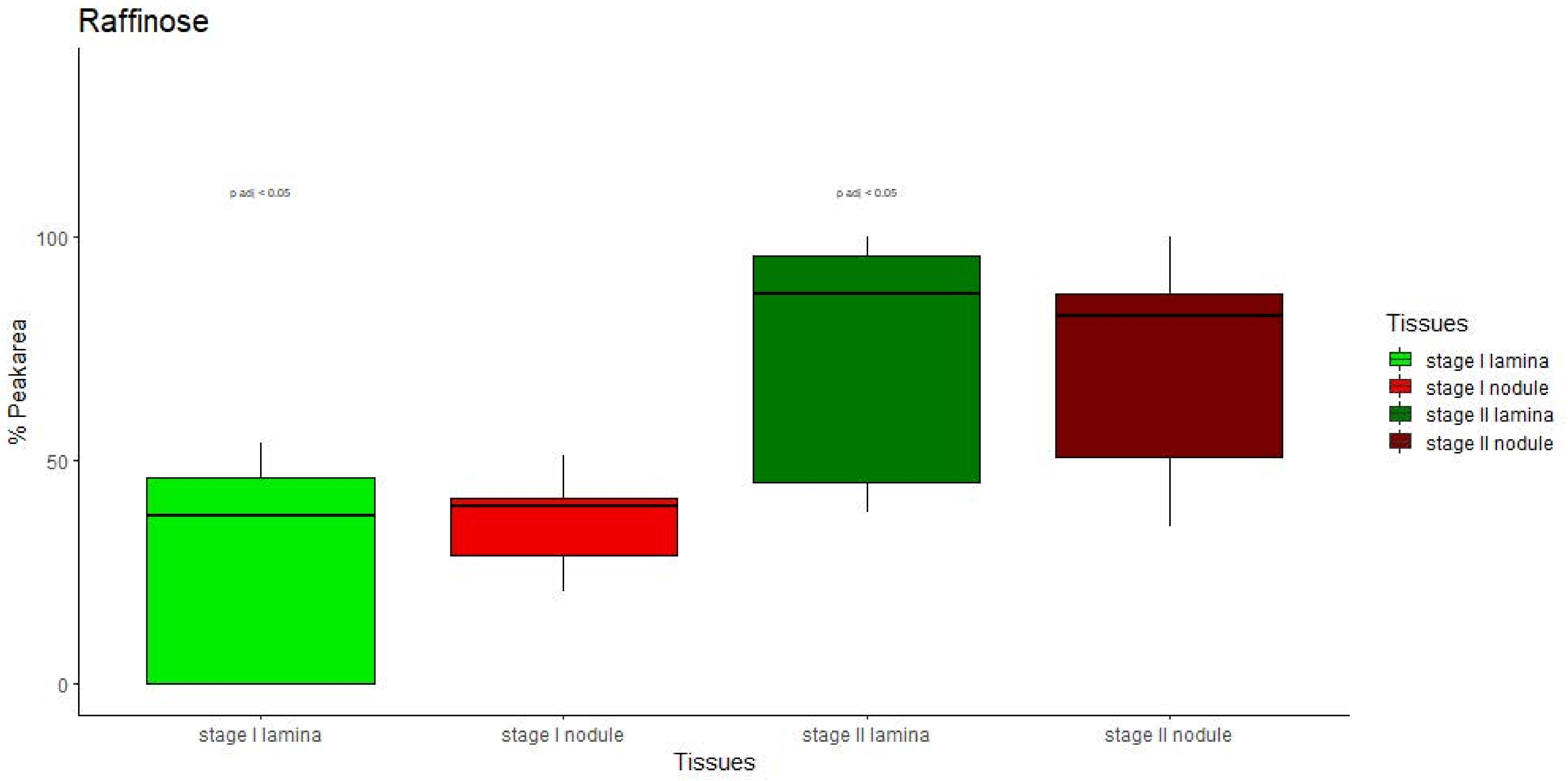

Raffinose
p adj < 0.05
p adj < 0.05
100
50
0
% Peakarea
stage I lamina
stage I nodule
stage II lamina
stage II nodule
Tissues
Tissues
stage I lamina
stage I nodule
stage II lamina
stage II nodule

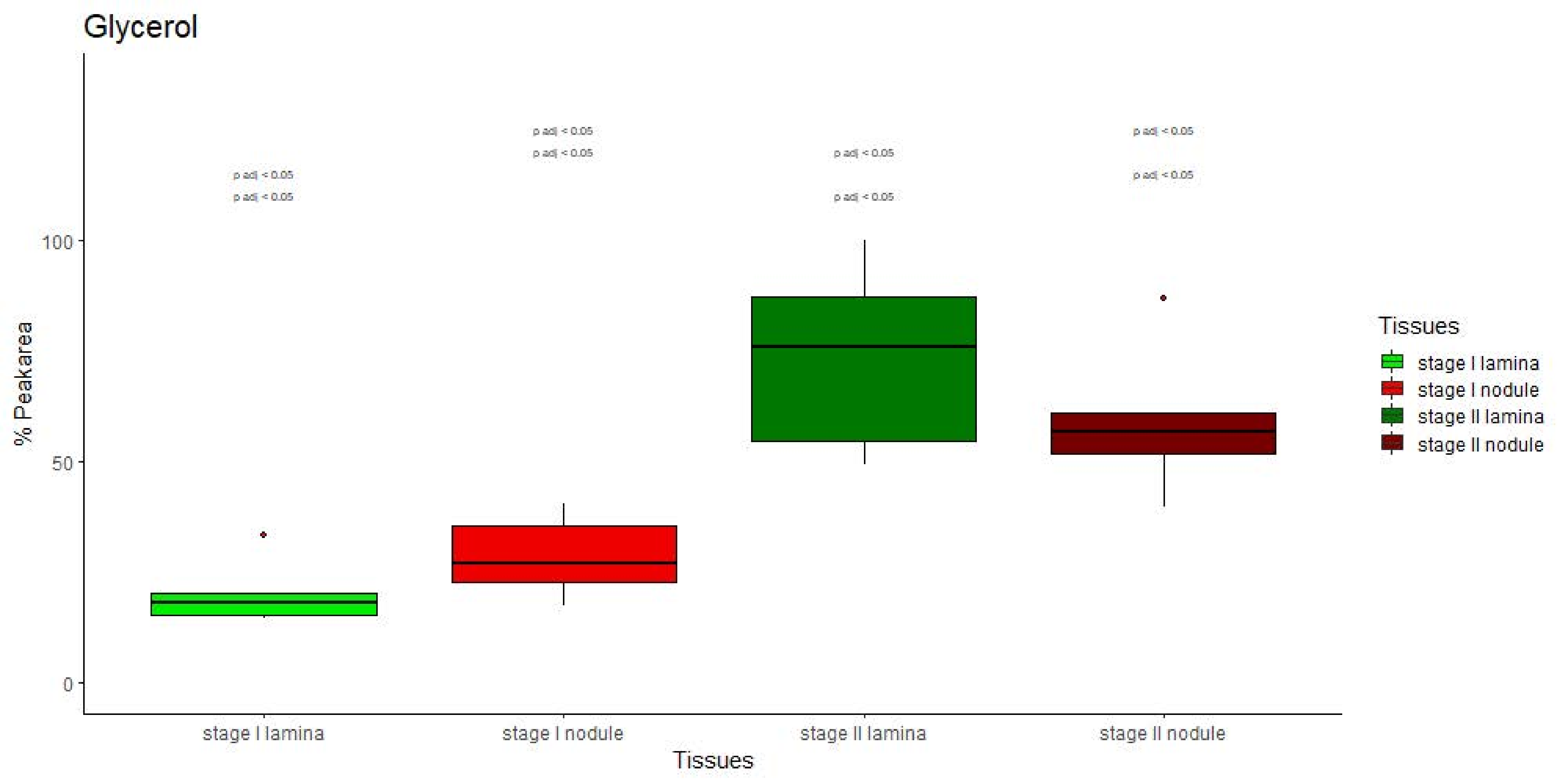

Glycerol
p adj < 0.05
p adj < 0.05
p adj < 0.05
p adj < 0.05
p adj < 0.05
p adj < 0.05
p adj < 0.05
p adj < 0.05
100
50
0
% Peakarea
stage I lamina
stage I nodule
stage II lamina
stage II nodule
Tissues
Tissues
stage I lamina
stage I nodule
stage II lamina
stage II nodule

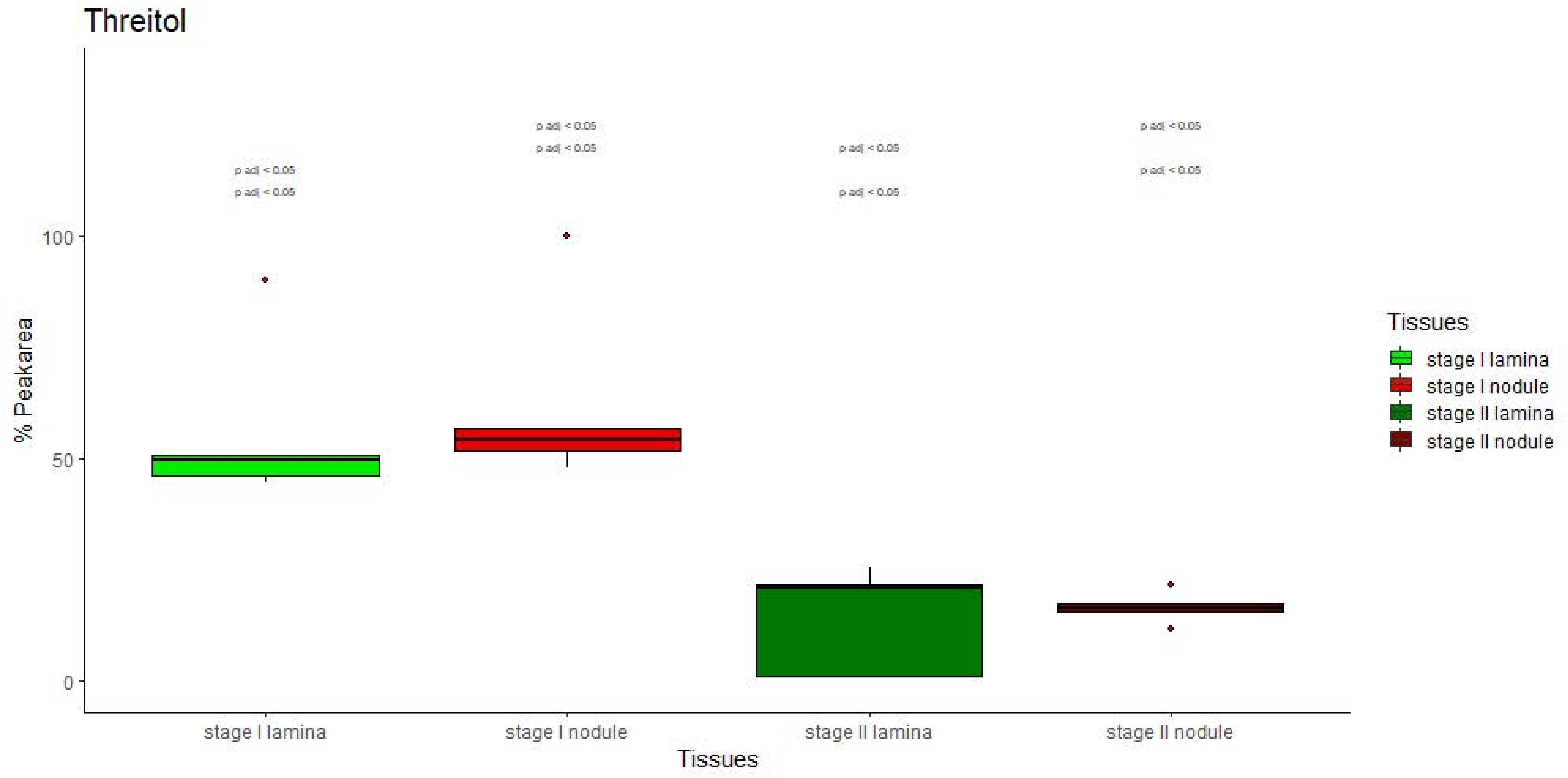

Threitol
p adj < 0.05
p adj < 0.05
p adj < 0.05
p adj < 0.05
p adj < 0.05
p adj < 0.05
p adj < 0.05
p adj < 0.05
100
50
0
% Peakarea
stage I lamina
stage I nodule
stage II lamina
stage II nodule
Tissues
Tissues
stage I lamina
stage I nodule
stage II lamina
stage II nodule

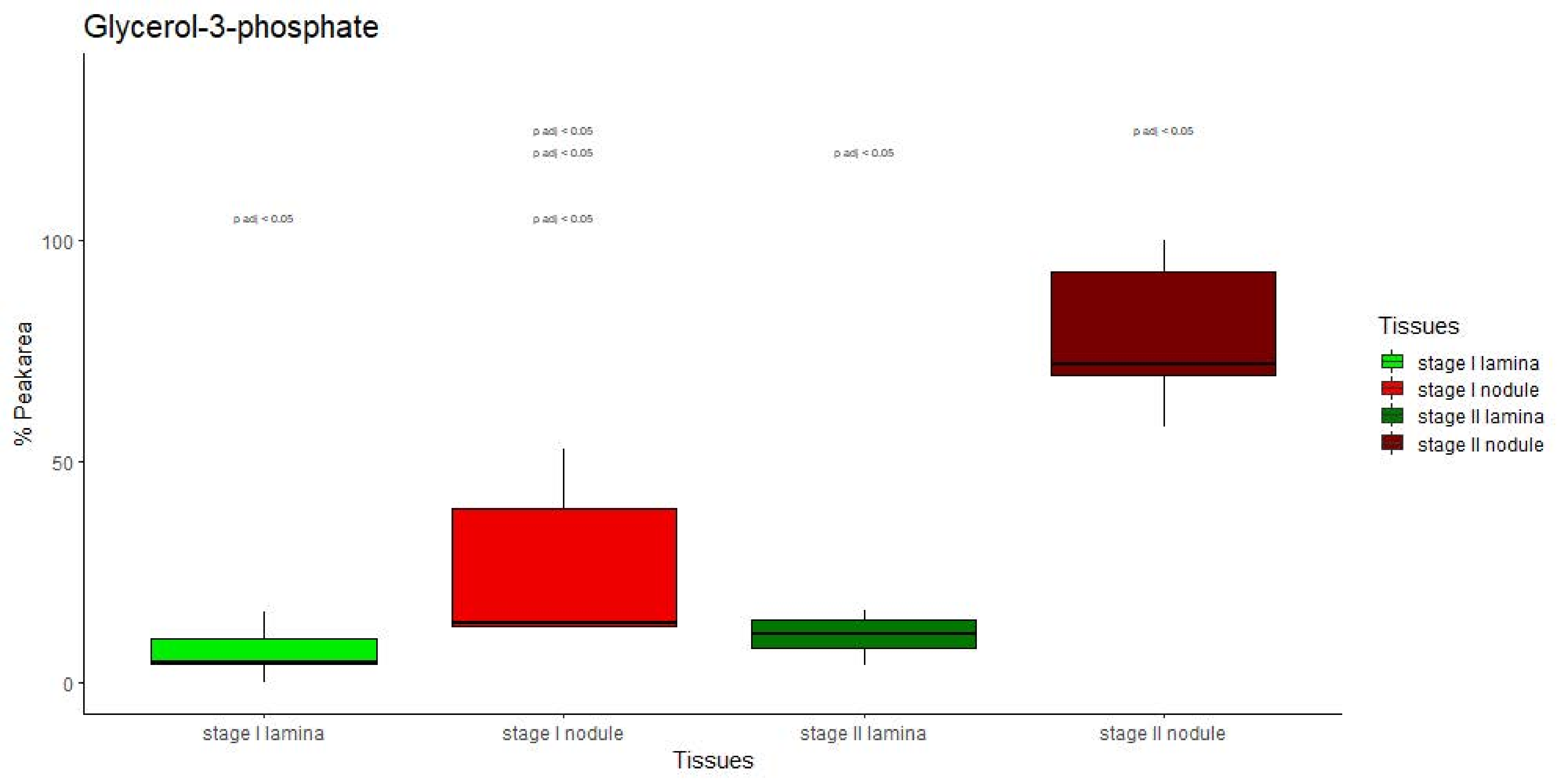
Glycerol-3-phosphate
p adj < 0.05
p adj < 0.05
p adj < 0.05
p adj < 0.05
p adj < 0.05
p adj < 0.05
% Peakarea
100
50
0
stage I lamina
stage I nodule
stage II lamina
stage II nodule
Tissues
Tissues
stage I lamina
stage I nodule
stage II lamina
stage II nodule

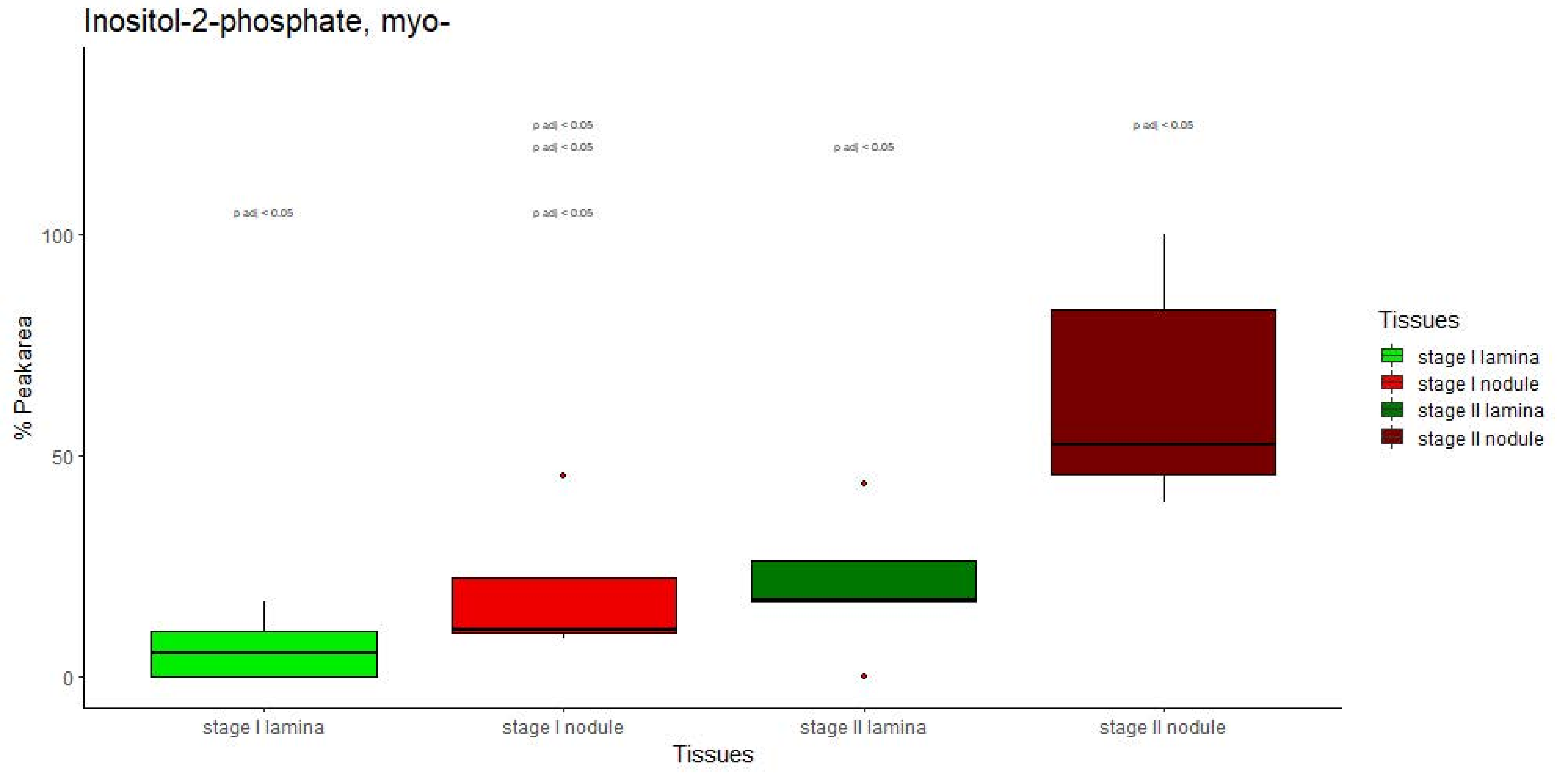

Inositol-2-phosphate, myo-
p adj < 0.05
p adj < 0.05
p adj < 0.05
p adj < 0.05
p adj < 0.05
p adj < 0.05
100
50
0
% Peakarea
stage I lamina
stage I nodule
stage II lamina
stage II nodule
Tissues
Tissues
stage I lamina
stage I nodule
stage II lamina
stage II nodule

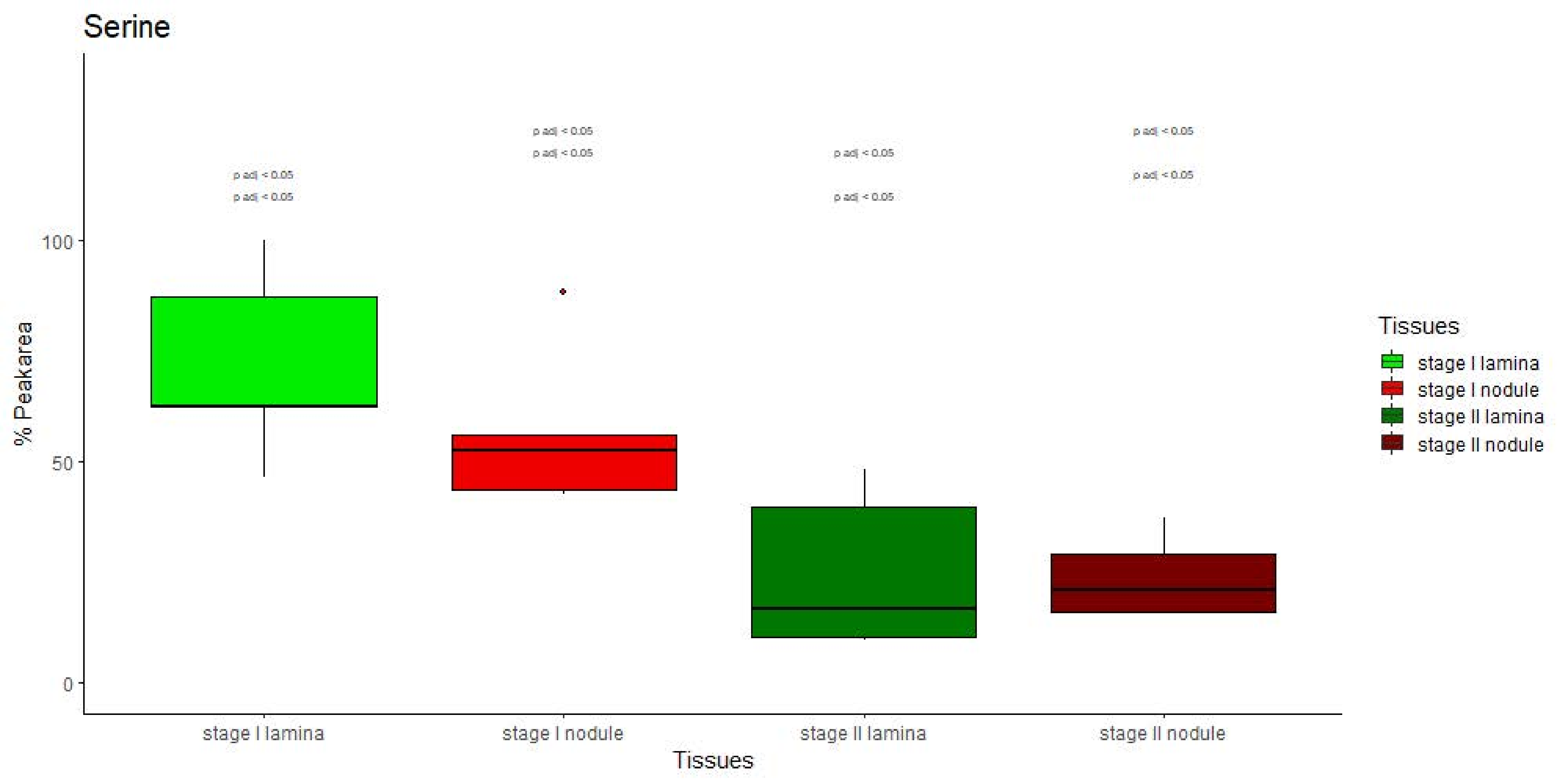

Serine
% Peakarea
100
50
0
p adj < 0.05
p adj < 0.05
p adj < 0.05
p adj < 0.05
p adj < 0.05
p adj < 0.05
p adj < 0.05
p adj < 0.05
stage I lamina
stage I nodule
stage II lamina
stage II nodule
Tissues
Tissues
stage I lamina
stage I nodule
stage II lamina
stage II nodule

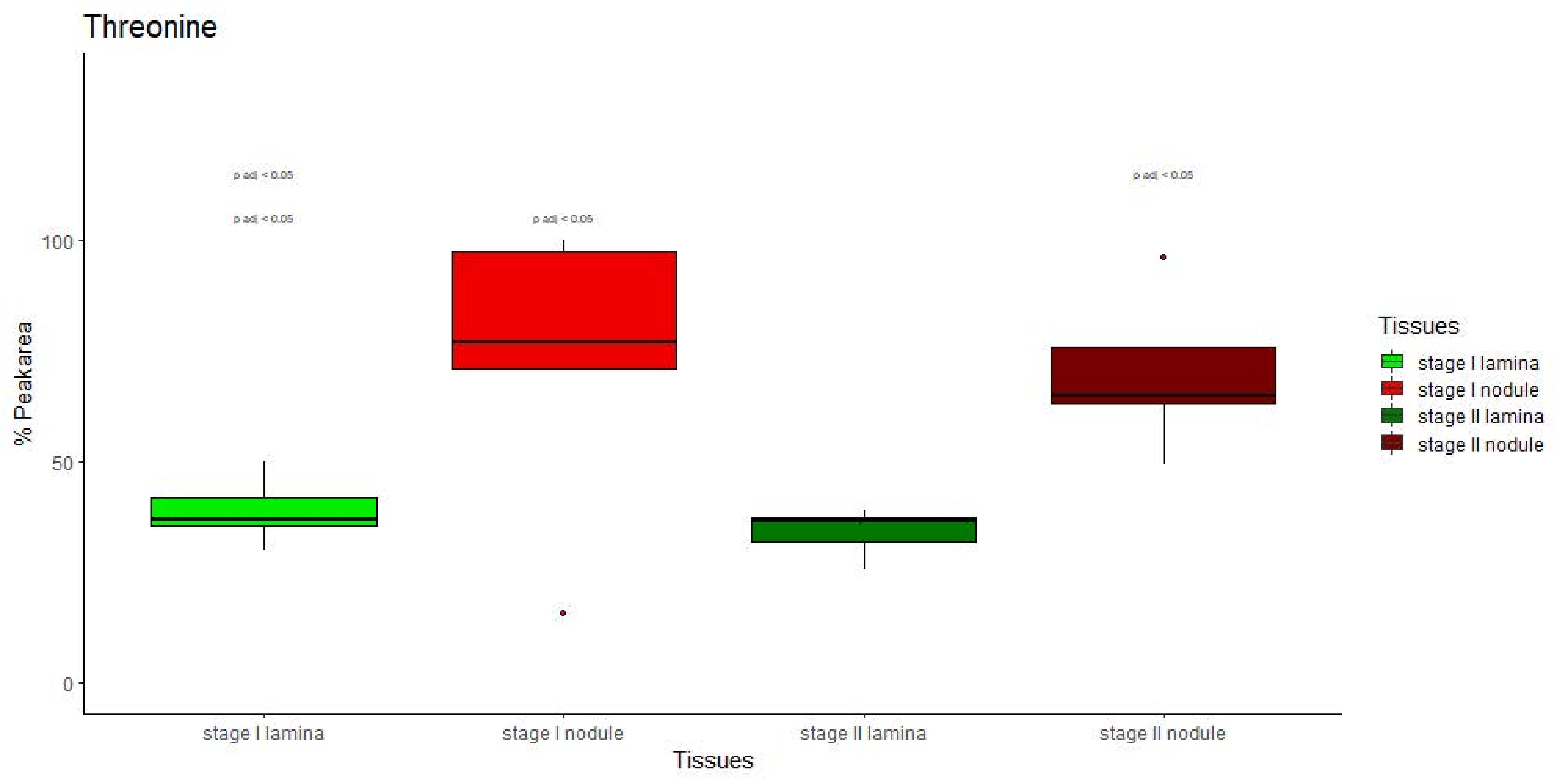

Threonine
p adj < 0.05
p adj < 0.05
p adj < 0.05
p adj < 0.05
100
50
0
% Peakarea
stage I lamina
stage I nodule
stage II lamina
stage II nodule
Tissues
Tissues
stage I lamina
stage I nodule
stage II lamina
stage II nodule

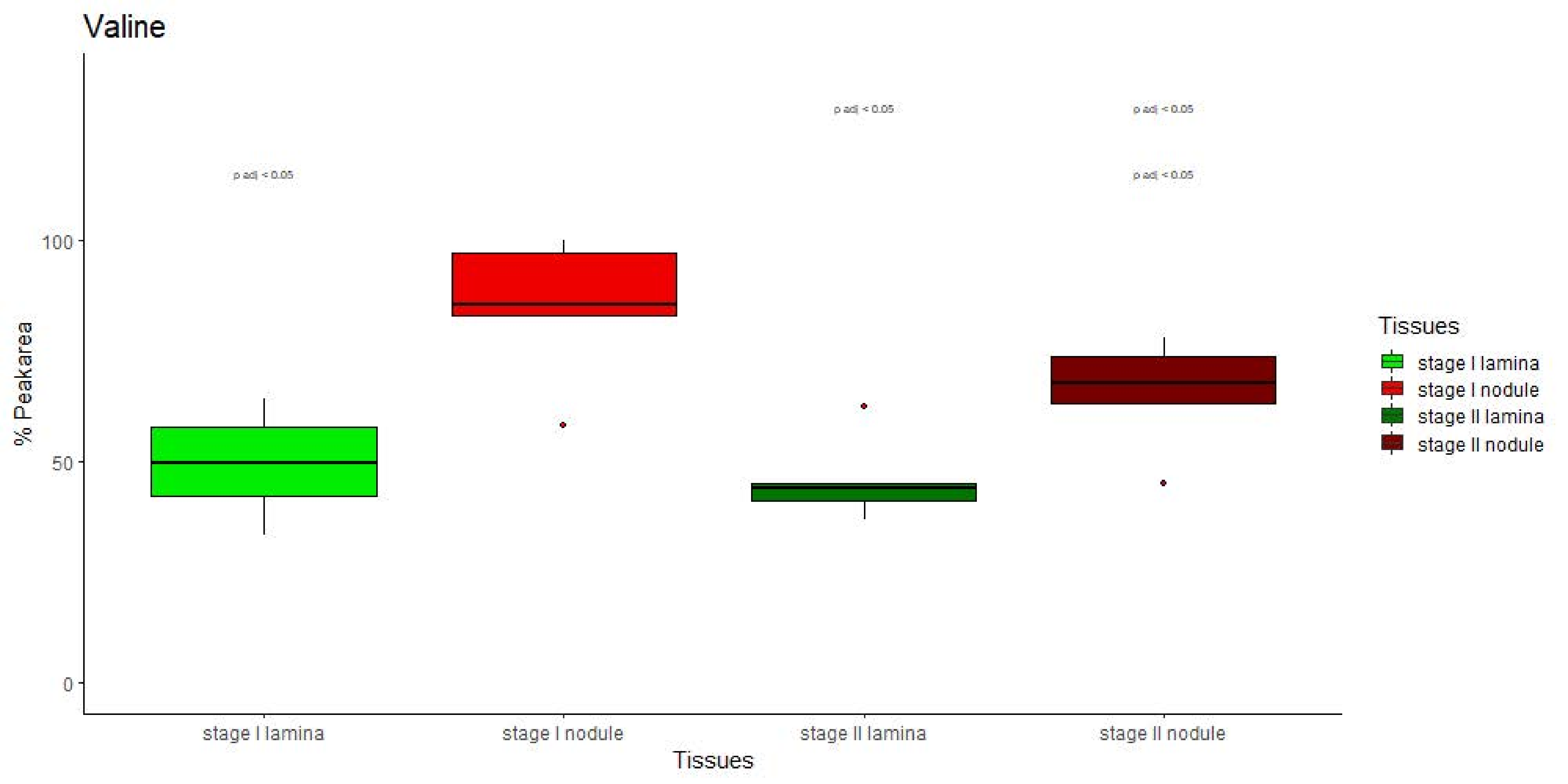

Valine
p adj < 0.05
p adj < 0.05
p adj < 0.05
p adj < 0.05
100
50
0
% Peakarea
stage I lamina
stage I nodule
stage II lamina
stage II nodule
Tissues
Tissues
stage I lamina
stage I nodule
stage II lamina
stage II nodule

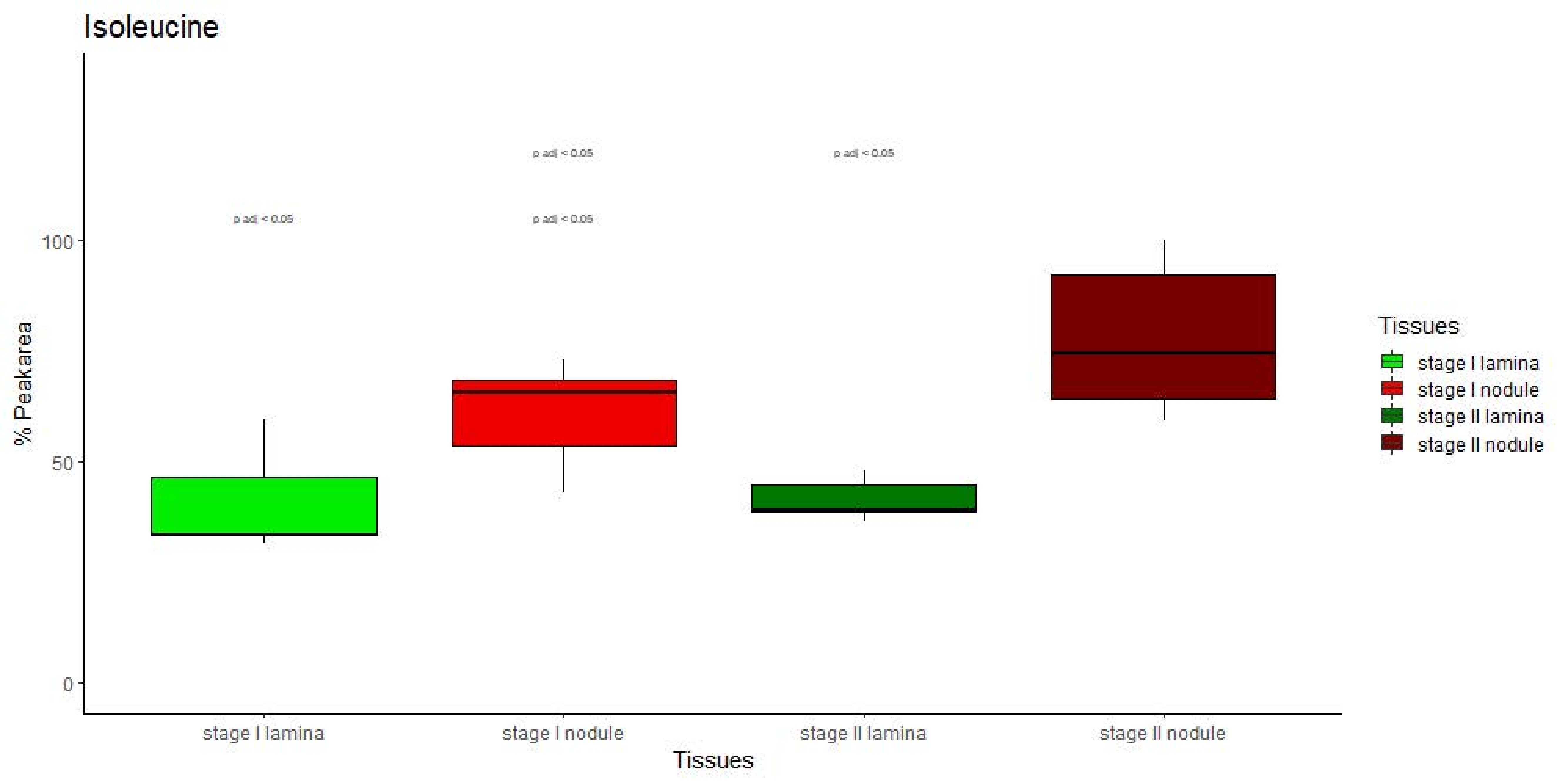

Isoleucine
p adj < 0.05
p adj < 0.05
p adj < 0.05
p adj < 0.05
100
50
0
% Peakarea
stage I lamina
stage I nodule
stage II lamina
stage II nodule
Tissues
Tissues
stage I lamina
stage I nodule
stage II lamina
stage II nodule

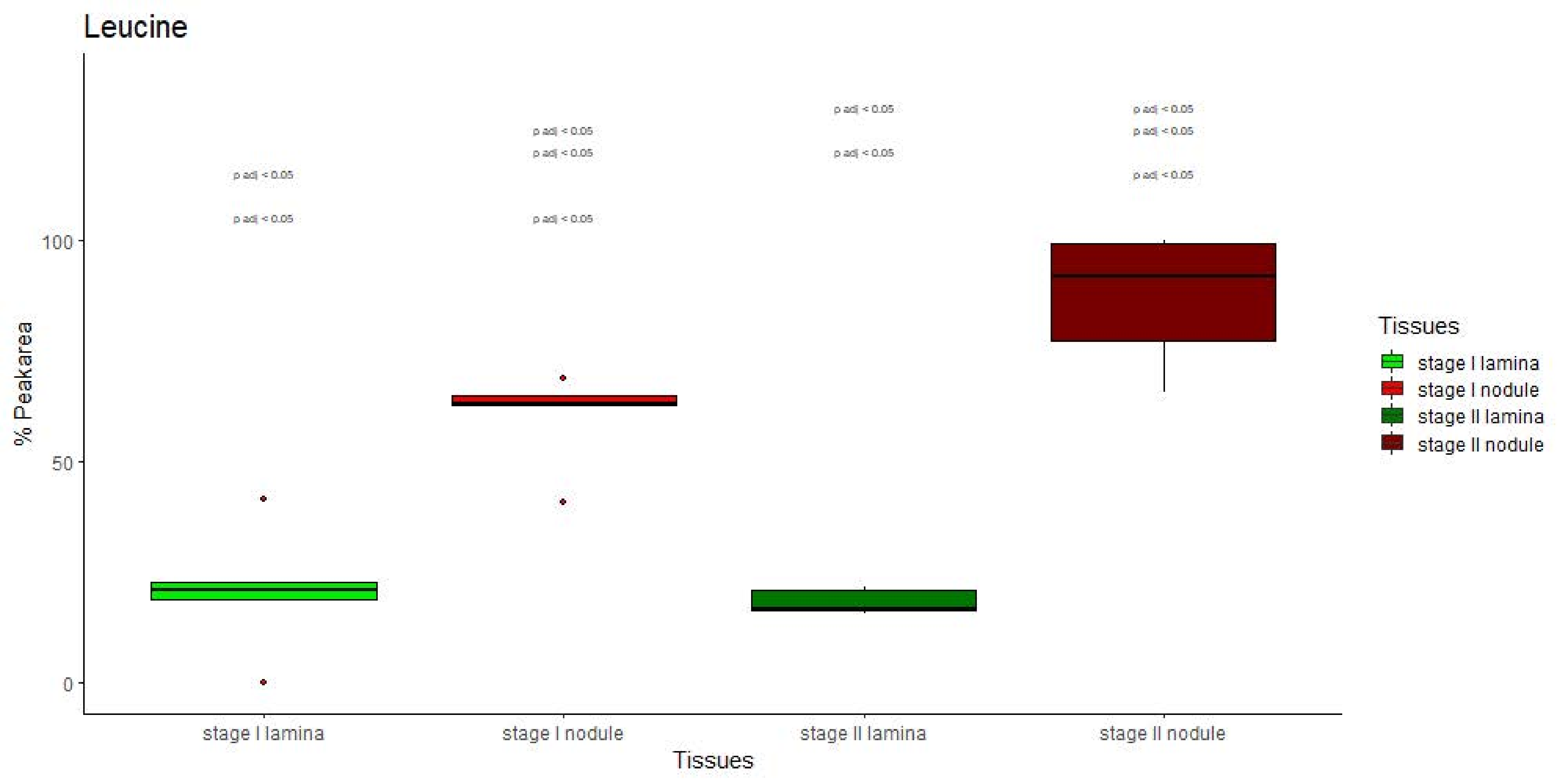
Leucine
p adj < 0.05
p adj < 0.05
p adj < 0.05
p adj < 0.05
p adj < 0.05
p adj < 0.05
p adj < 0.05
p adj < 0.05
p adj < 0.05
p adj < 0.05
100
50
0
% Peakarea
stage I lamina
stage I nodule
stage II lamina
stage II nodule
Tissues
Tissues
stage I lamina
stage I nodule
stage II lamina
stage II nodule

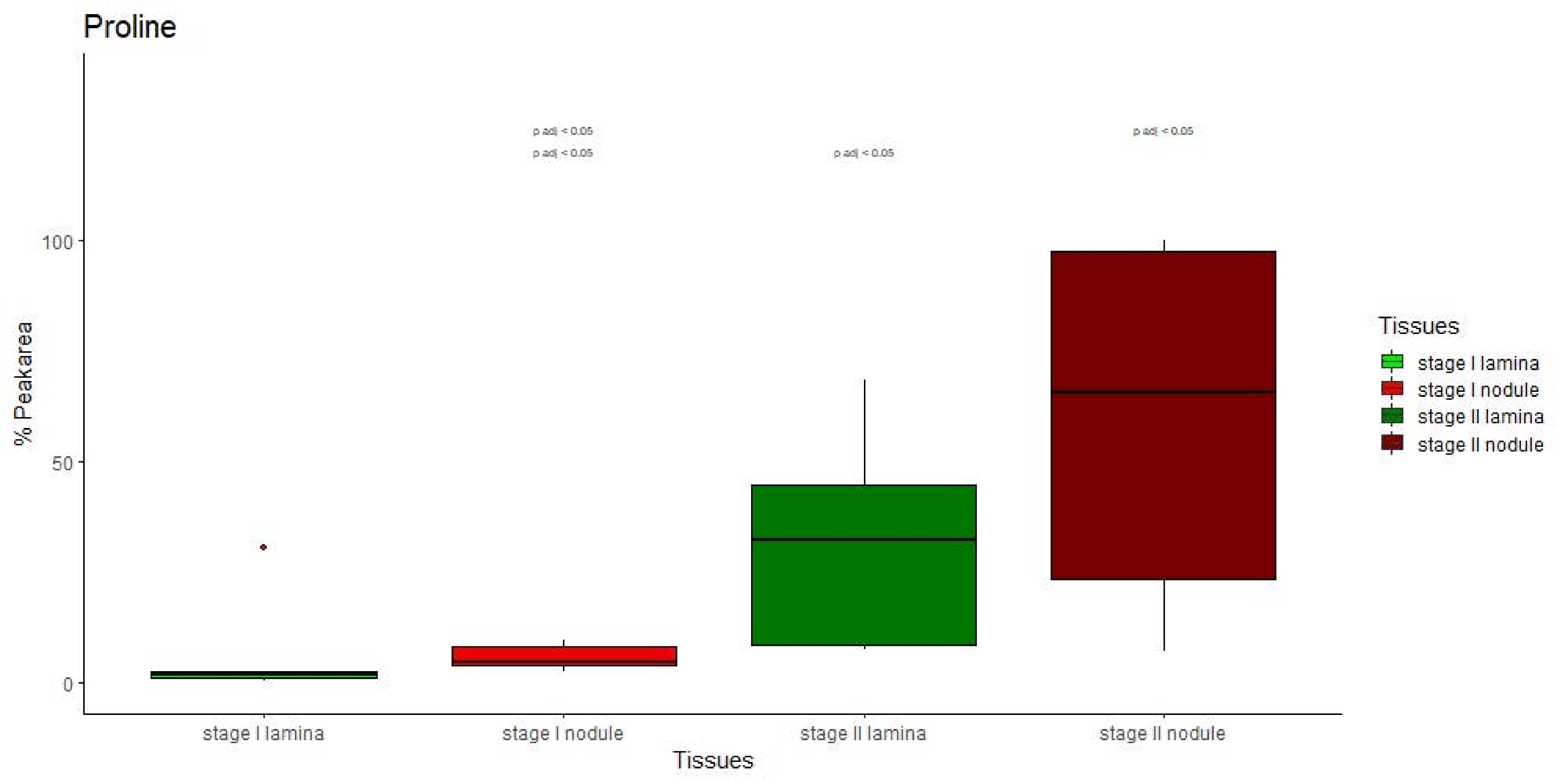

Proline
% Peakarea
100
50
0
p adj < 0.05
p adj < 0.05
p adj < 0.05
p adj < 0.05
stage I lamina
stage I nodule
stage II lamina
stage II nodule
Tissues
Tissues
stage I lamina
stage I nodule
stage II lamina
stage II nodule

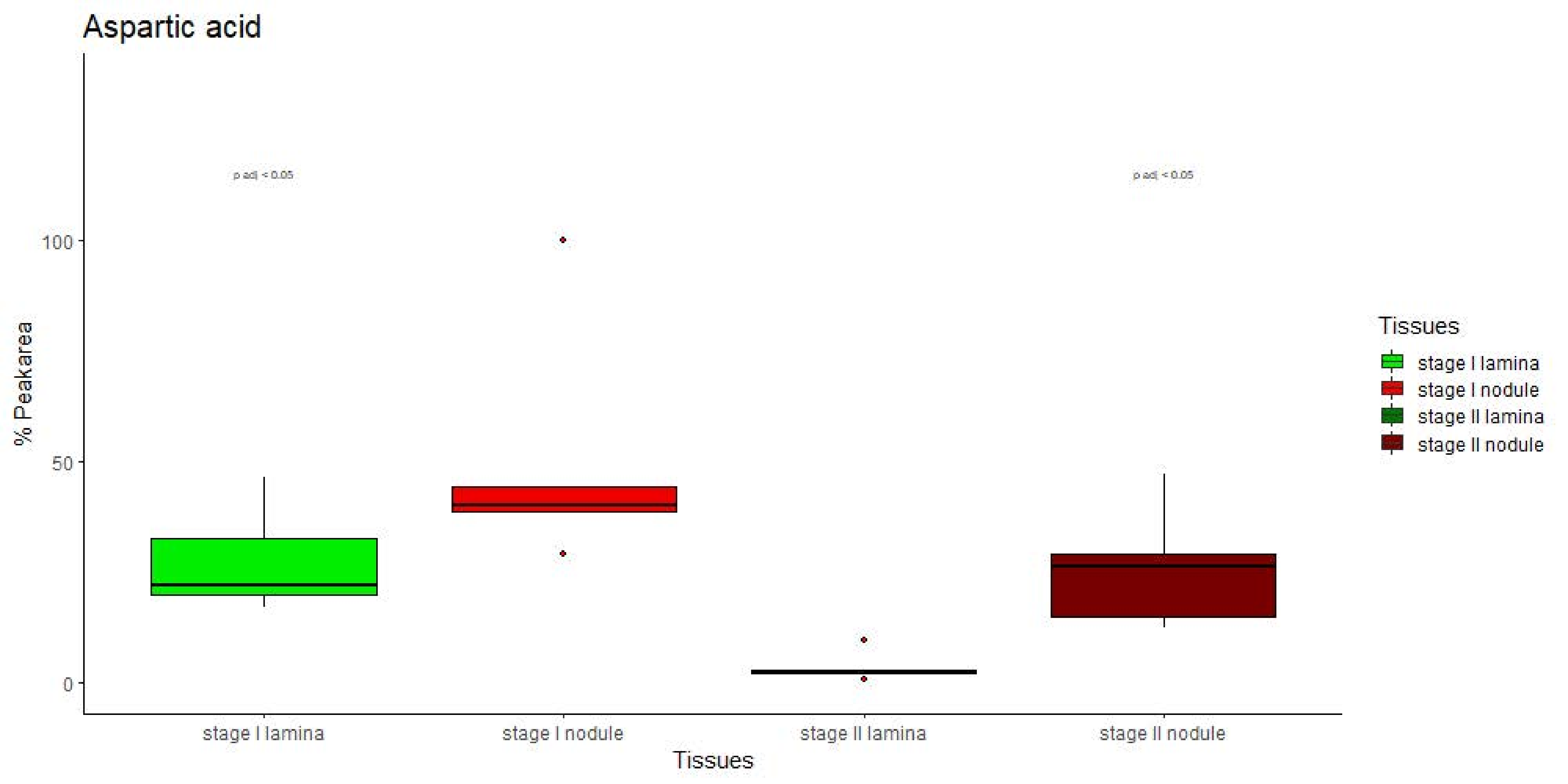

Aspartic acid
p adj < 0.05
p adj < 0.05
100
50
0
% Peakarea
Tissues
stage I lamina
stage I nodule
stage II lamina
stage II nodule
stage I lamina
stage I nodule
stage II lamina
stage II nodule
Tissues

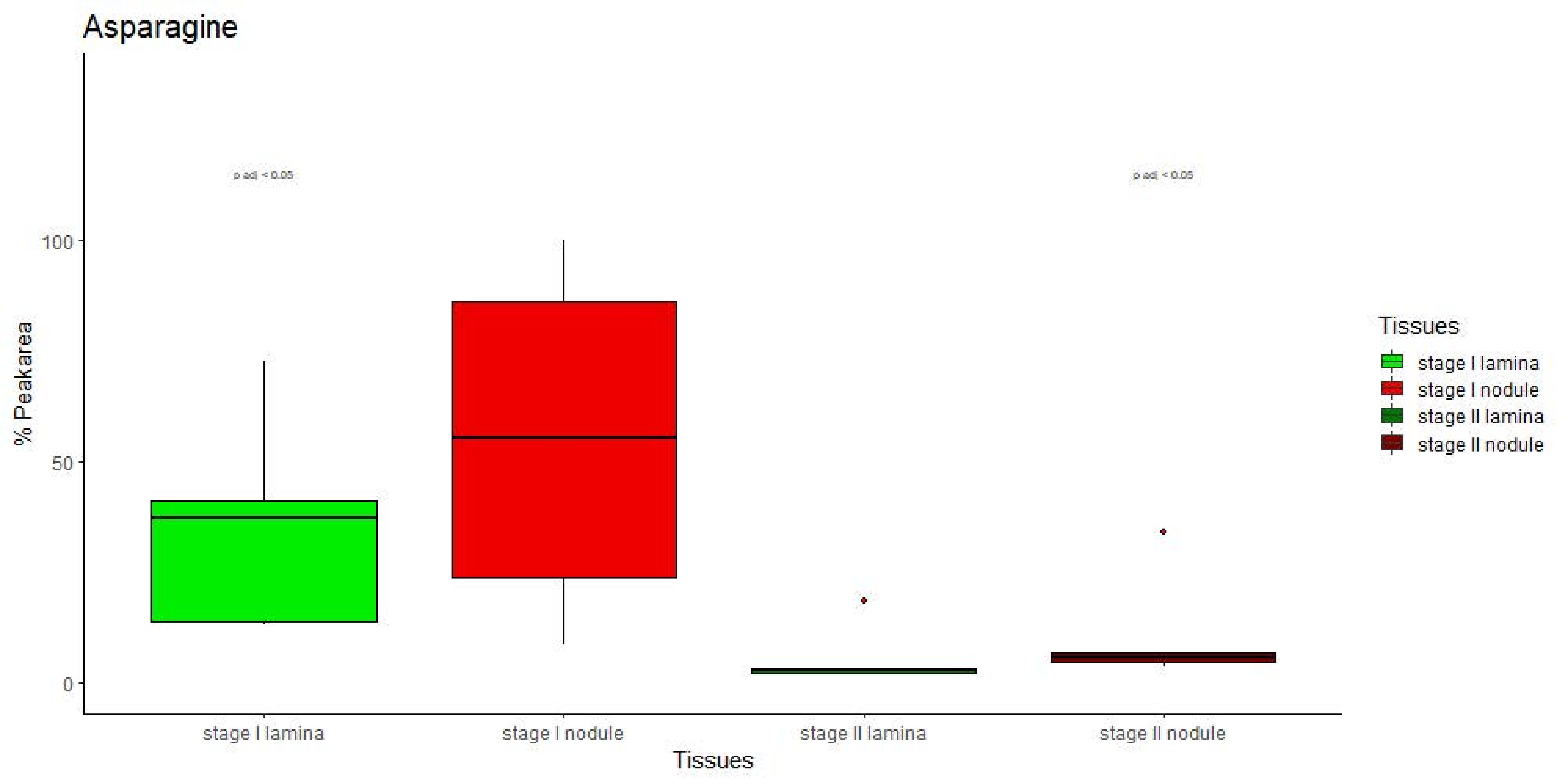

Asparagine
p adj < 0.05
p adj < 0.05
100
50
0
% Peakarea
stage I lamina
stage I nodule
stage II lamina
stage II nodule
Tissues
Tissues
stage I lamina
stage I nodule
stage II lamina
stage II nodule

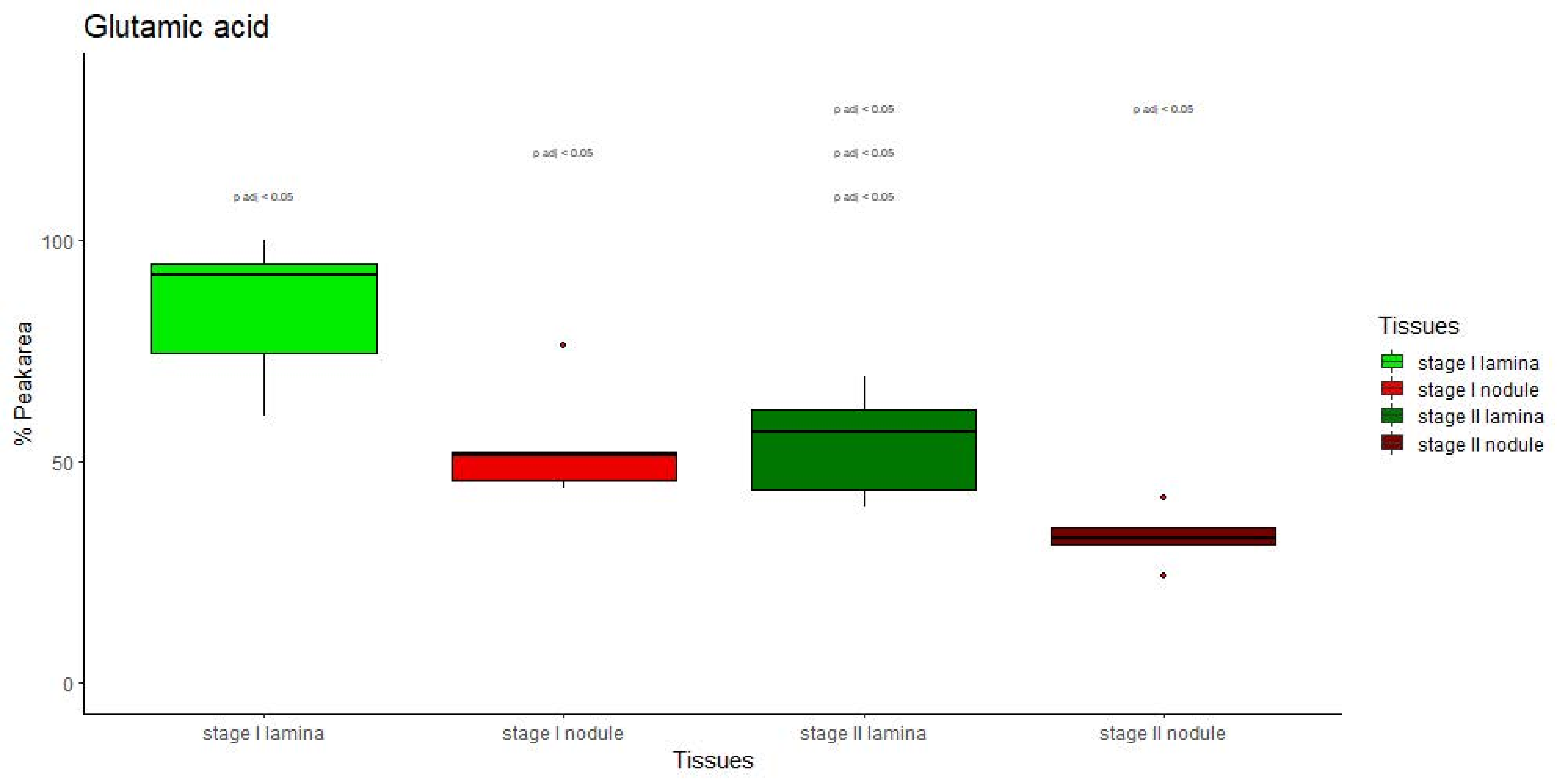

Glutamic acid
p adj < 0.05
p adj < 0.05
p adj < 0.05
p adj < 0.05
p adj < 0.05
p adj < 0.05
p adj < 0.05
100
50
0
% Peakarea
stage I lamina
stage I nodule
stage II lamina
stage II nodule
Tissues
Tissues
stage I lamina
stage I nodule
stage II lamina
stage II nodule

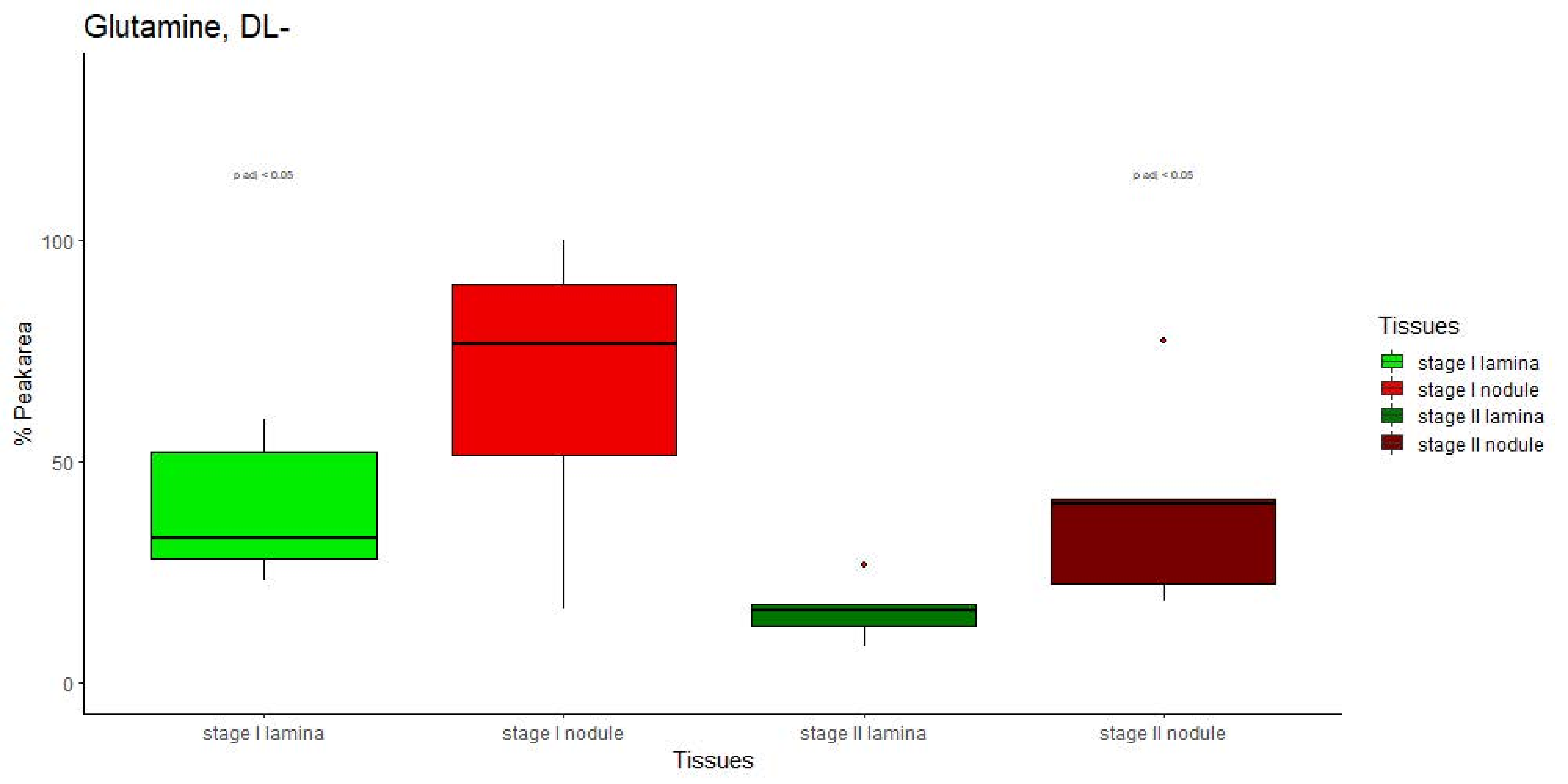
Glutamine, DL-
p adj < 0.05
p adj < 0.05
100
50
0
% Peakarea
stage I lamina
stage I nodule
stage II lamina
stage II nodule
Tissues
Tissues
stage I lamina
stage I nodule
stage II lamina
stage II nodule

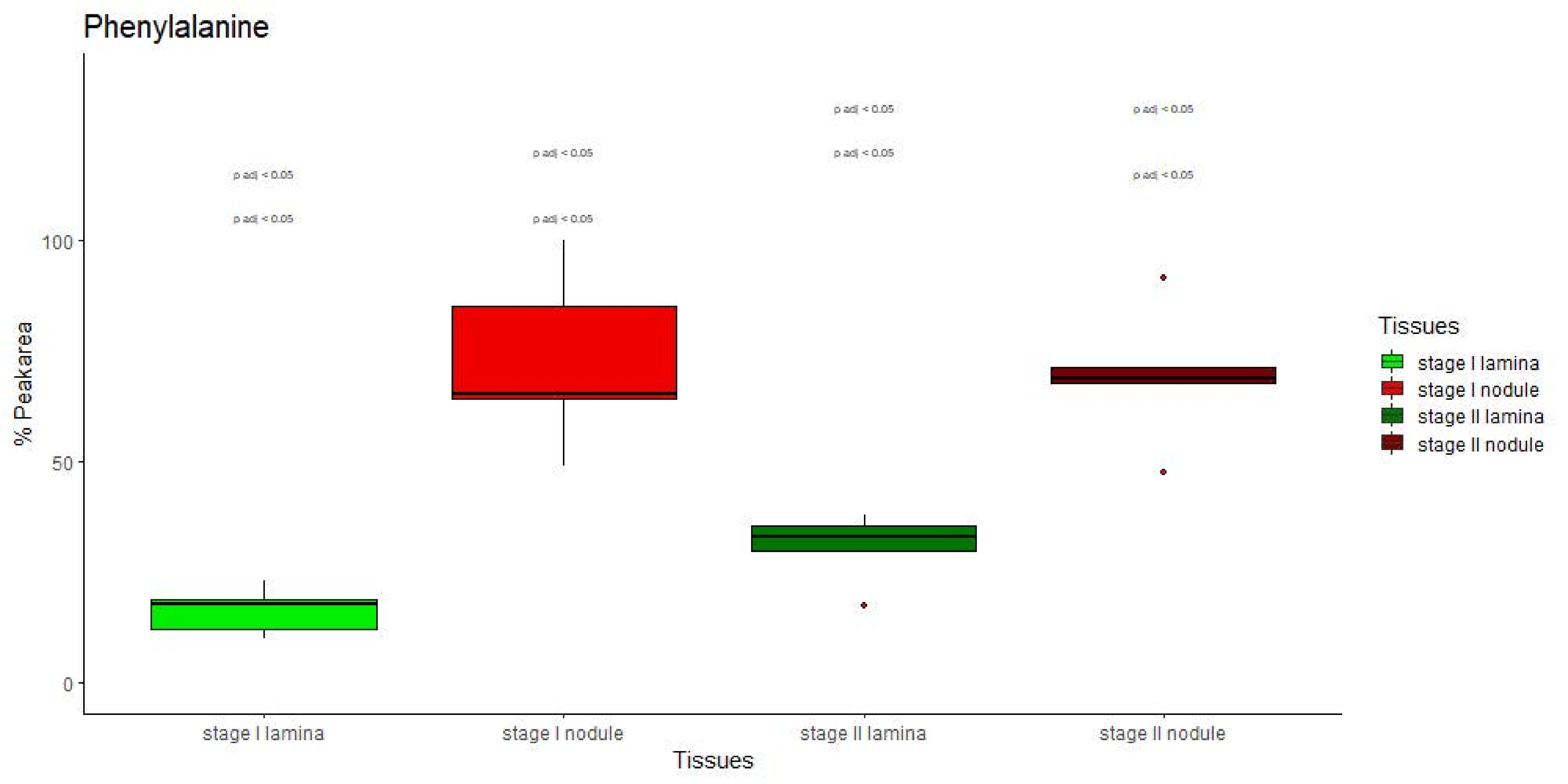
Phenylalanine
% Peakarea
100
50
0
p adj < 0.05
p adj < 0.05
p adj < 0.05
p adj < 0.05
p adj < 0.05
p adj < 0.05
p adj < 0.05
p adj < 0.05
stage I lamina
stage I nodule
stage II lamina
stage II nodule
Tissues
Tissues
stage I lamina
stage I nodule
stage II lamina
stage II nodule

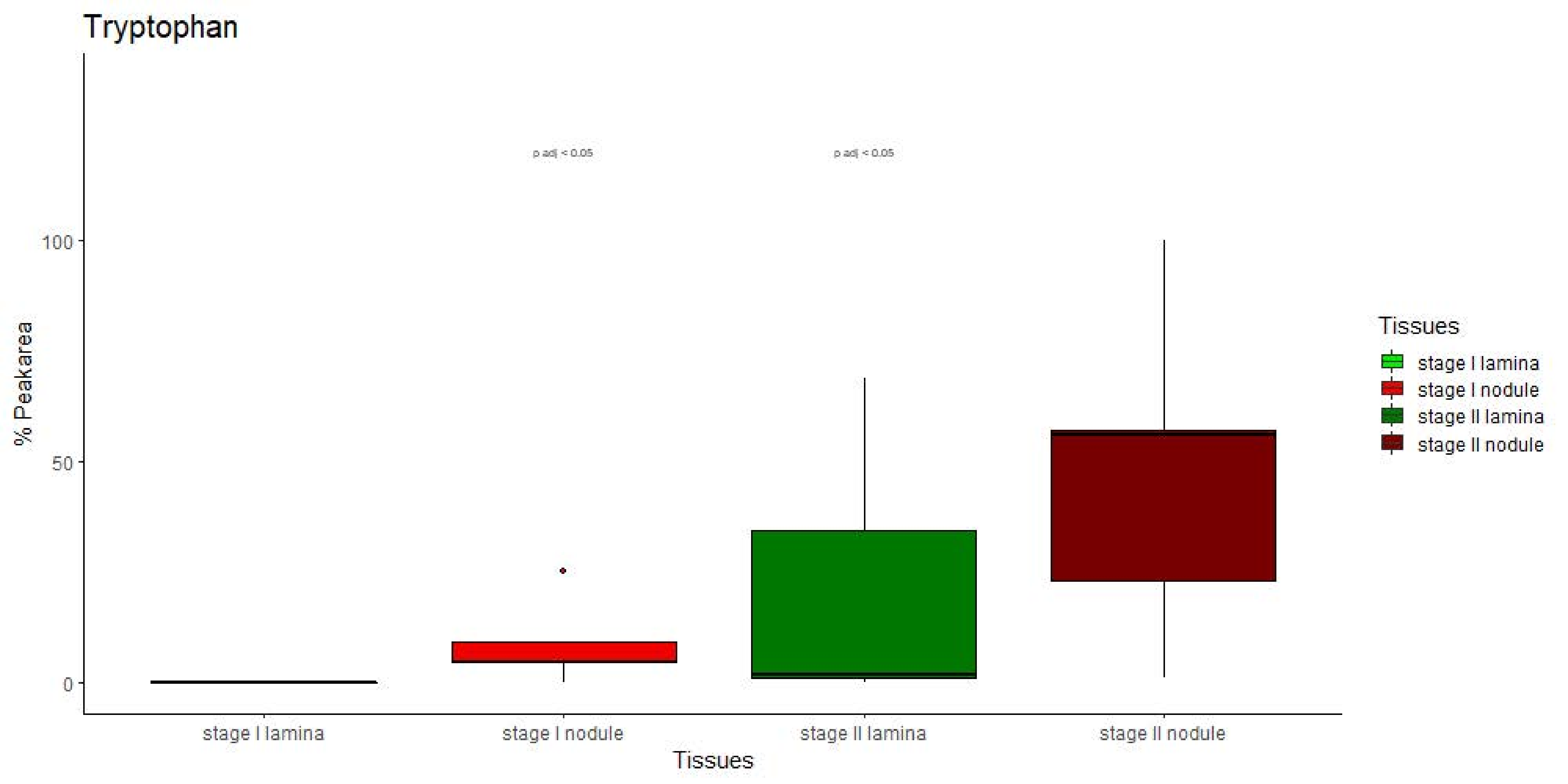
Tryptophan
p adj < 0.05
p adj < 0.05
100
50
0
% Peakarea
stage I lamina
stage I nodule
stage II lamina
stage II nodule
Tissues
Tissues
stage I lamina
stage I nodule
stage II lamina
stage II nodule

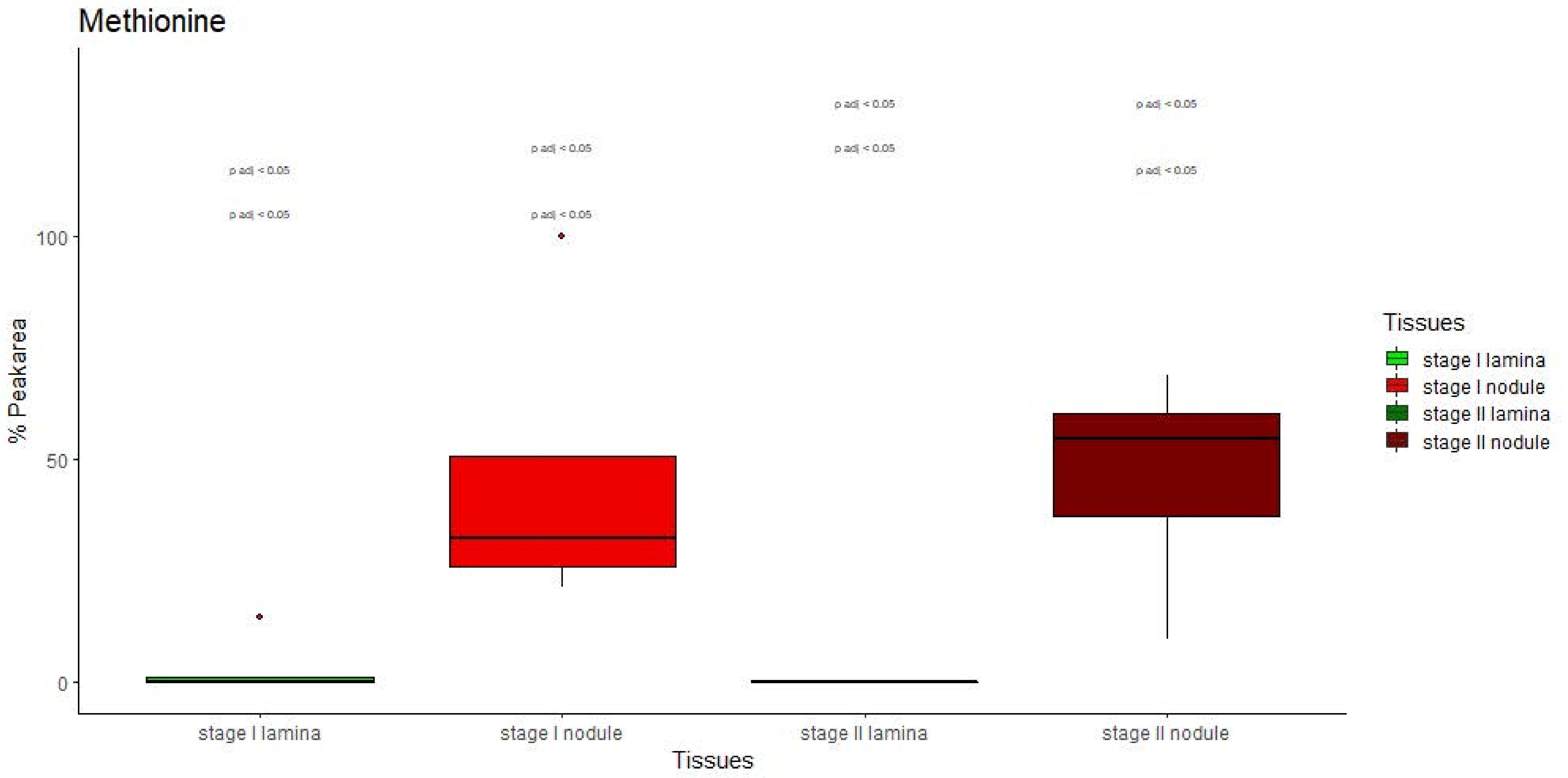

Methionine
% Peakarea
0
50
100
p adj < 0.05
p adj < 0.05
p adj < 0.05
p adj < 0.05
p adj < 0.05
p adj < 0.05
p adj < 0.05
p adj < 0.05
stage I lamina
stage I nodule
stage II lamina
stage II nodule
Tissues
Tissues
stage I lamina
stage I nodule
stage II lamina
stage II nodule

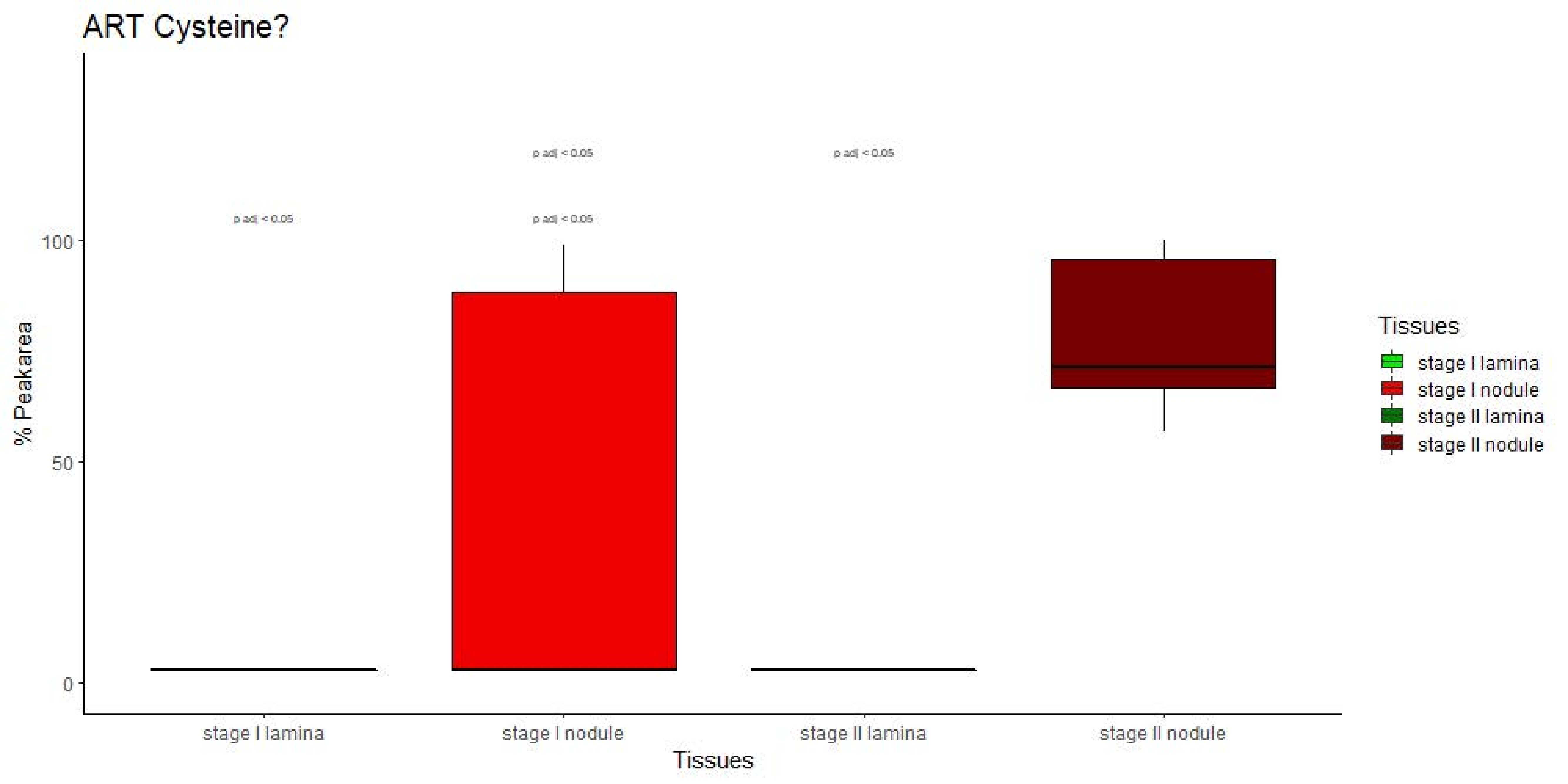
ART Cysteine?
p adj < 0.05
p adj < 0.05
p adj < 0.05
p adj < 0.05
100
50
0
% Peakarea
stage I lamina
stage I nodule
stage II lamina
stage II nodule
Tissues
Tissues
stage I lamina
stage I nodule
stage II lamina
stage II nodule

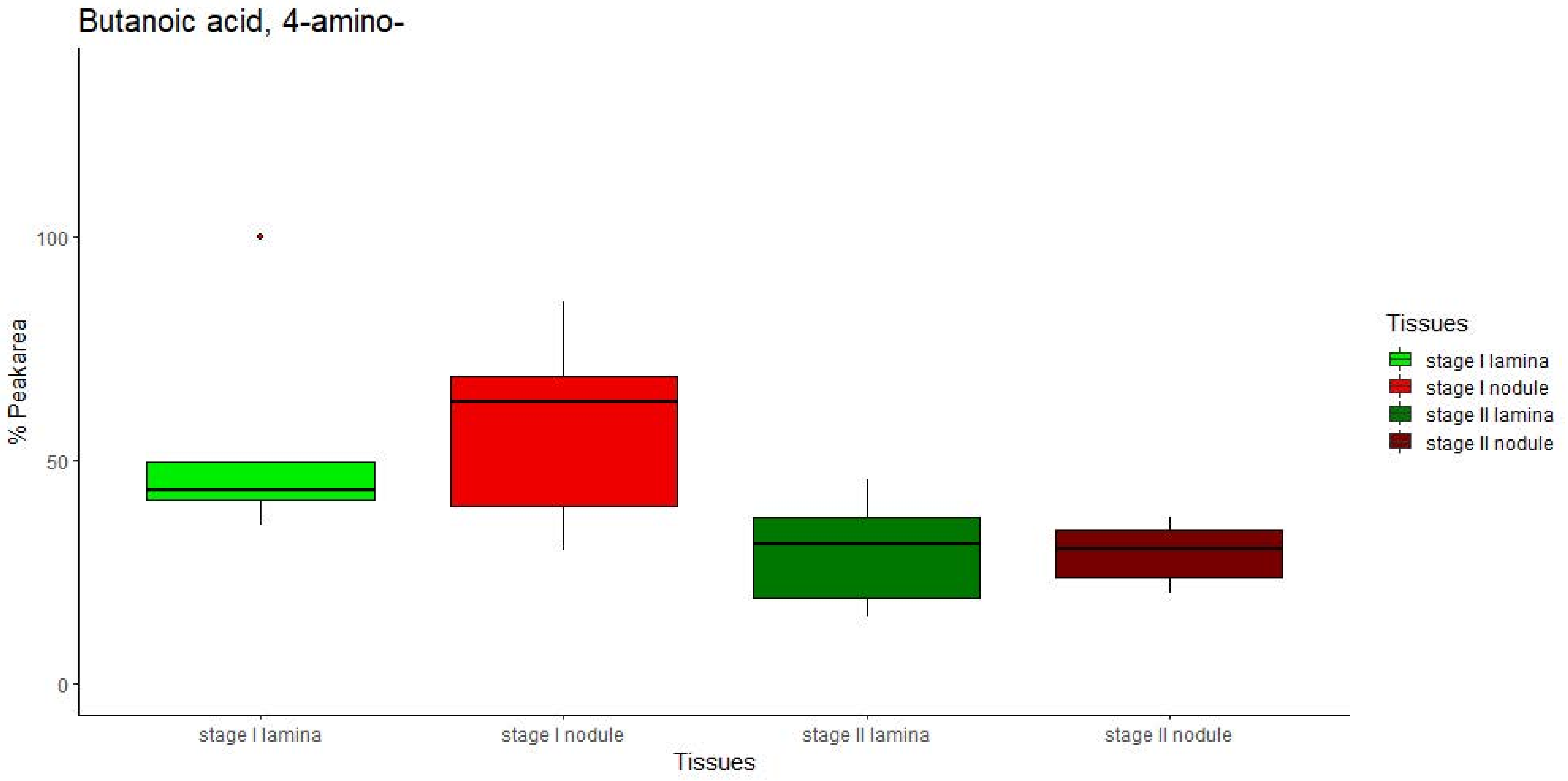

Butanoic acid, 4-amino-
% Peakarea
100
50
0
stage I lamina
stage I nodule
stage II lamina
stage II nodule
Tissues
Tissues
stage I lamina
stage I nodule
stage II lamina
stage II nodule

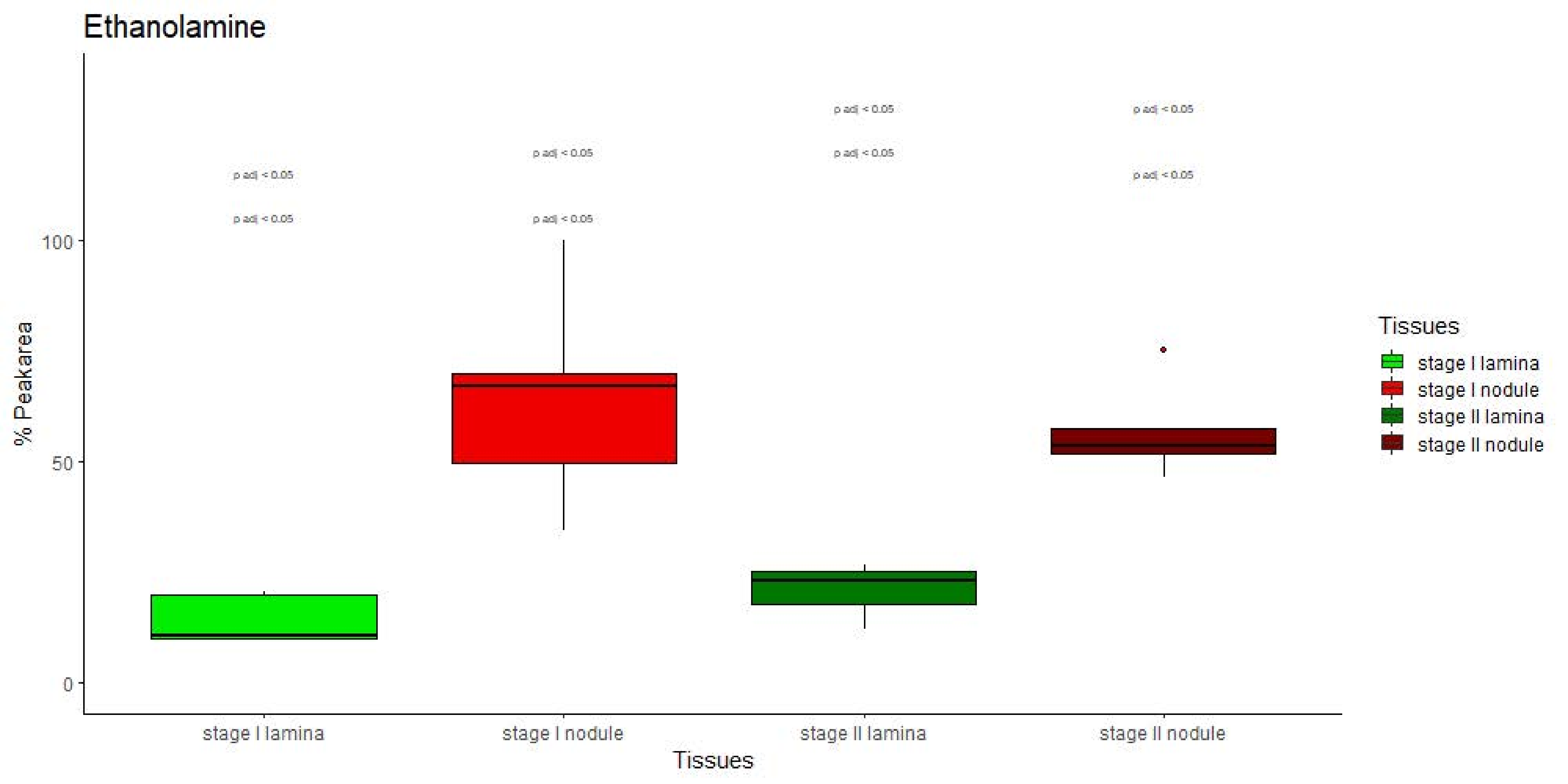

Ethanolamine
p adj < 0.05
p adj < 0.05
p adj < 0.05
p adj < 0.05
p adj < 0.05
p adj < 0.05
p adj < 0.05
p adj < 0.05
100
50
0
% Peakarea
Tissues
stage I lamina
stage I nodule
stage II lamina
stage II nodule
Tissues
stage I lamina
stage I nodule
stage II lamina
stage II nodule

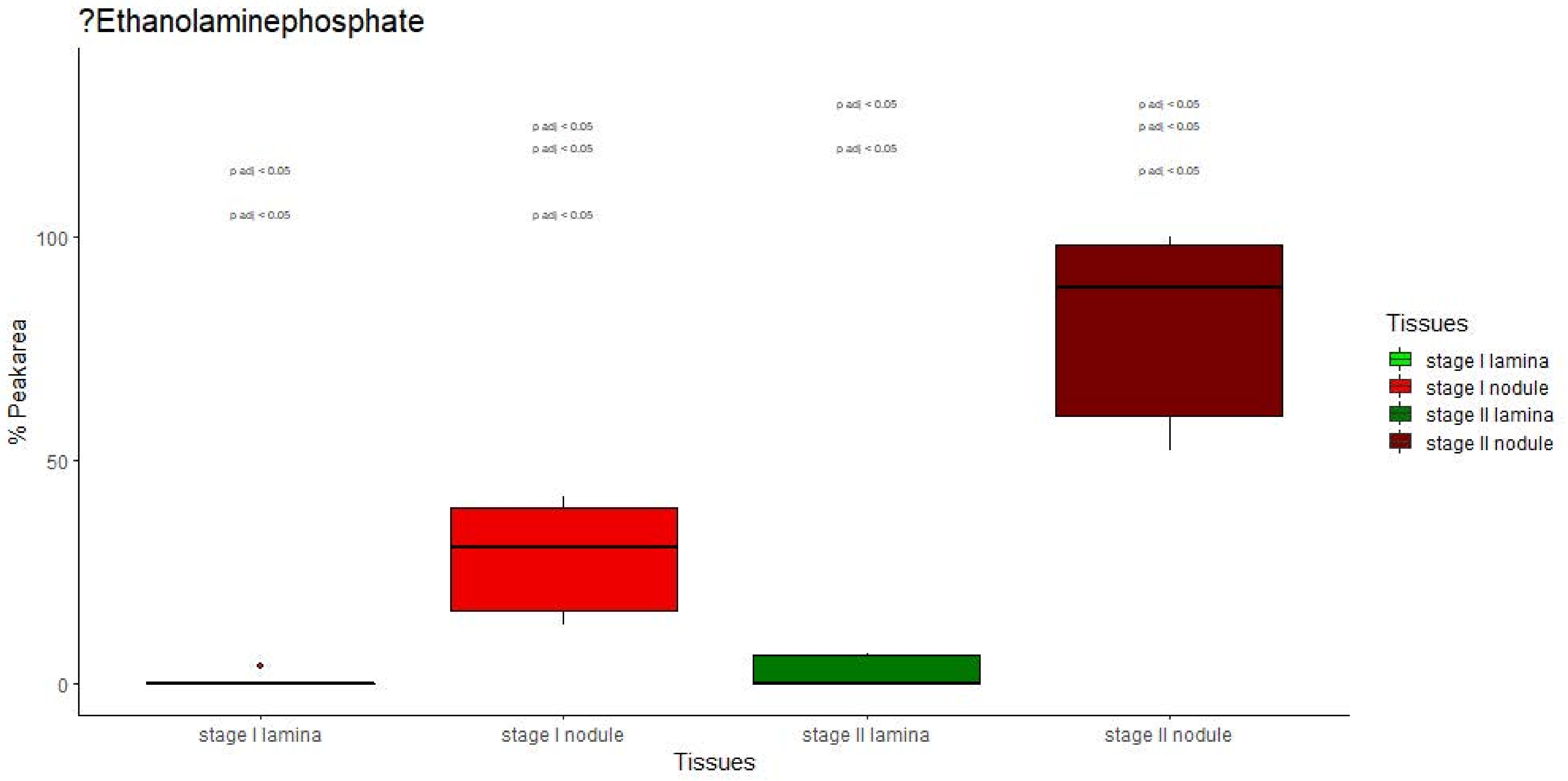

?Ethanolaminephosphate
% Peakarea
100
50
0
p adj < 0.05
stage I lamina
stage I nodule
stage II lamina
stage II nodule
Tissues
Tissues
stage I lamina
stage I nodule
stage II lamina
stage II nodule

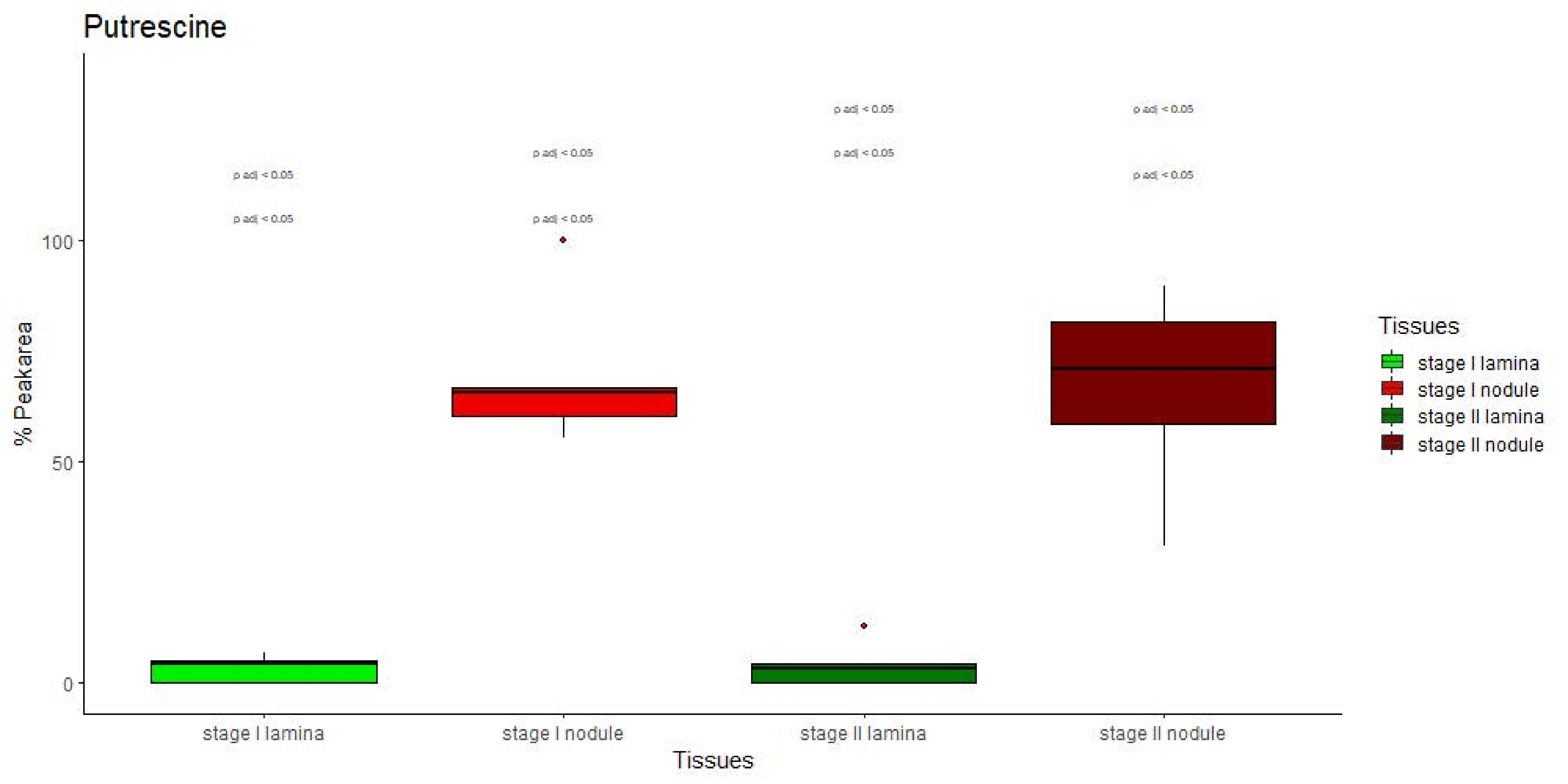

Putrescine
p adj < 0.05
p adj < 0.05
p adj < 0.05
p adj < 0.05
p adj < 0.05
p adj < 0.05
p adj < 0.05
p adj < 0.05
100
50
0
% Peakarea
stage I lamina
stage I nodule
stage II lamina
stage II nodule
Tissues
Tissues
stage I lamina
stage I nodule
stage II lamina
stage II nodule

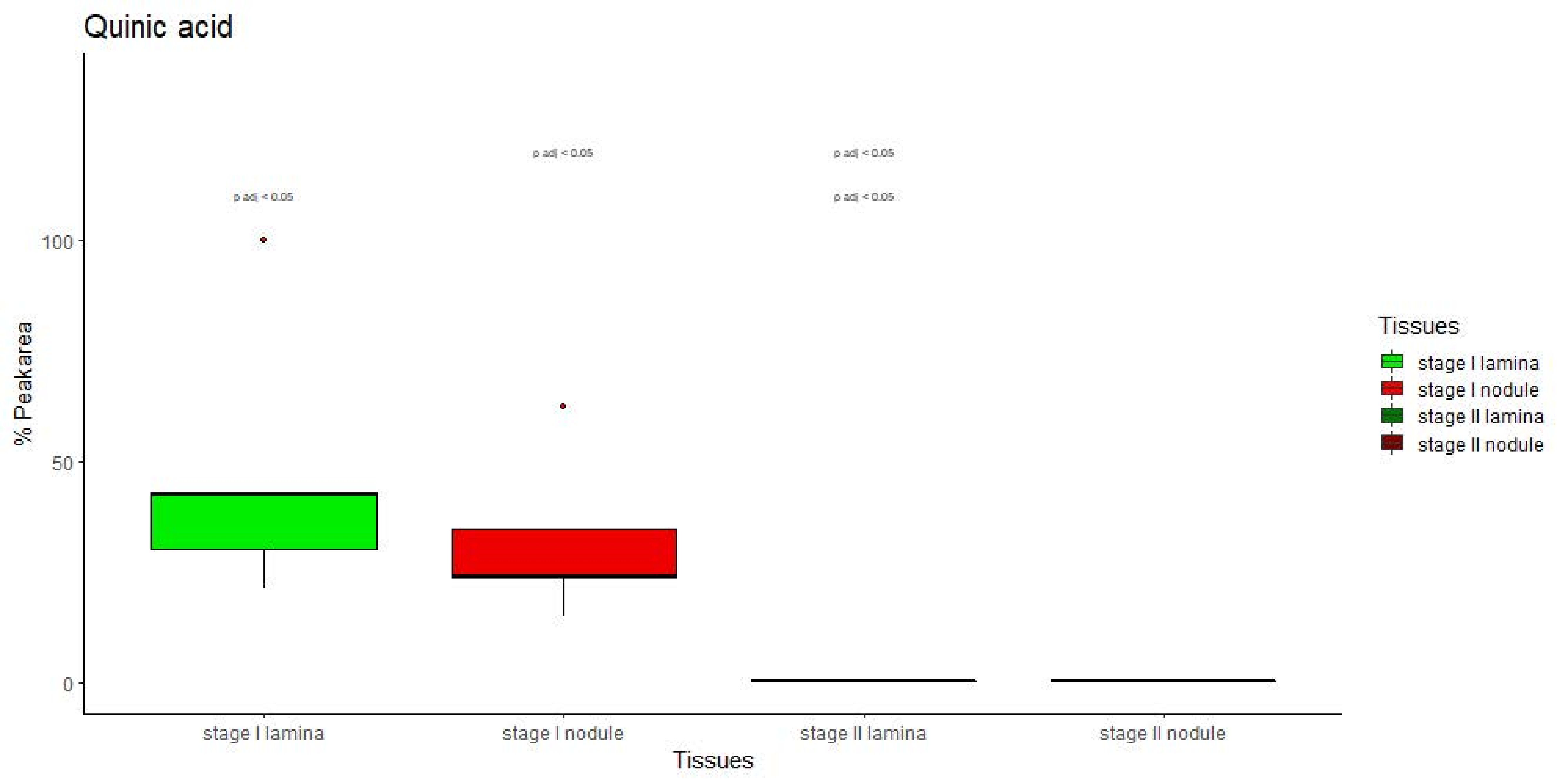

Quinic acid
p adj < 0.05
p adj < 0.05
p adj < 0.05
p adj < 0.05
100
50
0
% Peakarea
stage I lamina
stage I nodule
stage II lamina
stage II nodule
Tissues
Tissues
stage I lamina
stage I nodule
stage II lamina
stage II nodule

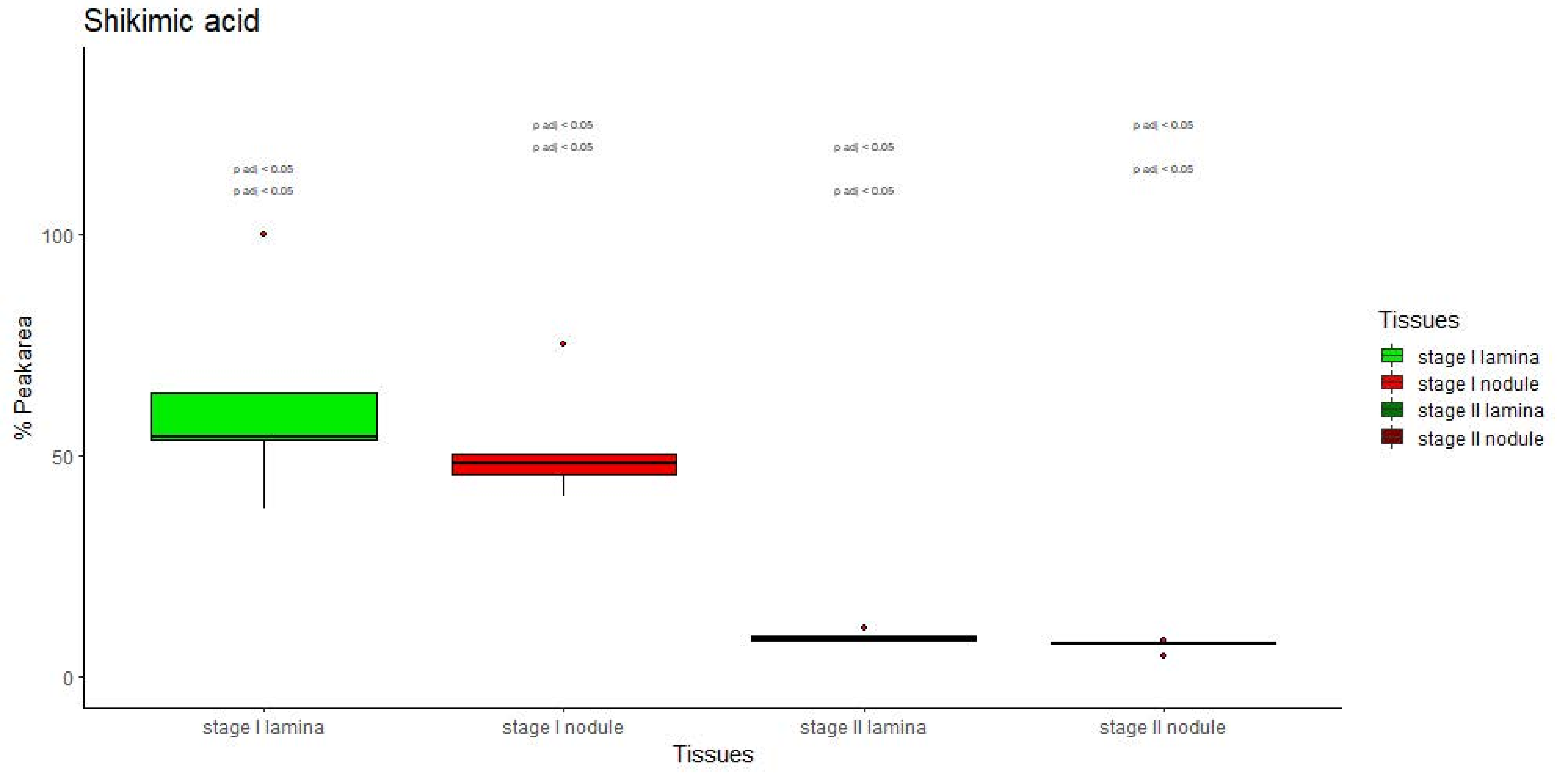

Shikimic acid
p adj < 0.05
p adj < 0.05
p adj < 0.05
p adj < 0.05
p adj < 0.05
p adj < 0.05
p adj < 0.05
p adj < 0.05
100
50
0
% Peakarea
stage I lamina
stage I nodule
stage II lamina
stage II nodule
Tissues
Tissues
stage I lamina
stage I nodule
stage II lamina
stage II nodule

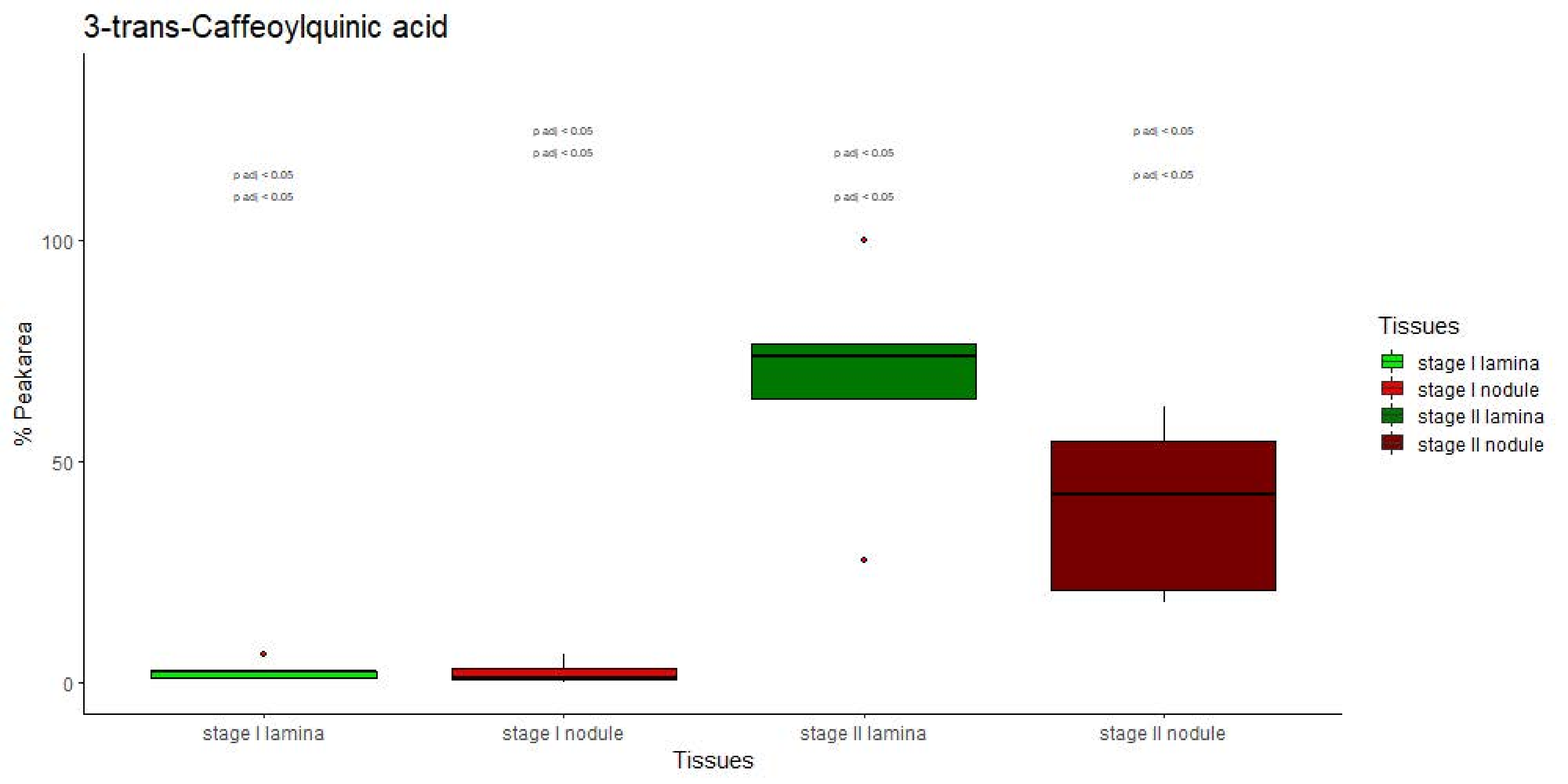

3-trans-Caffeoylquinic acid
p adj < 0.05
p adj < 0.05
p adj < 0.05
p adj < 0.05
p adj < 0.05
p adj < 0.05
p adj < 0.05
p adj < 0.05
100
50
0
% Peakarea
stage I lamina
stage I nodule
stage II lamina
stage II nodule
Tissues
Tissues
stage I lamina
stage I nodule
stage II lamina
stage II nodule

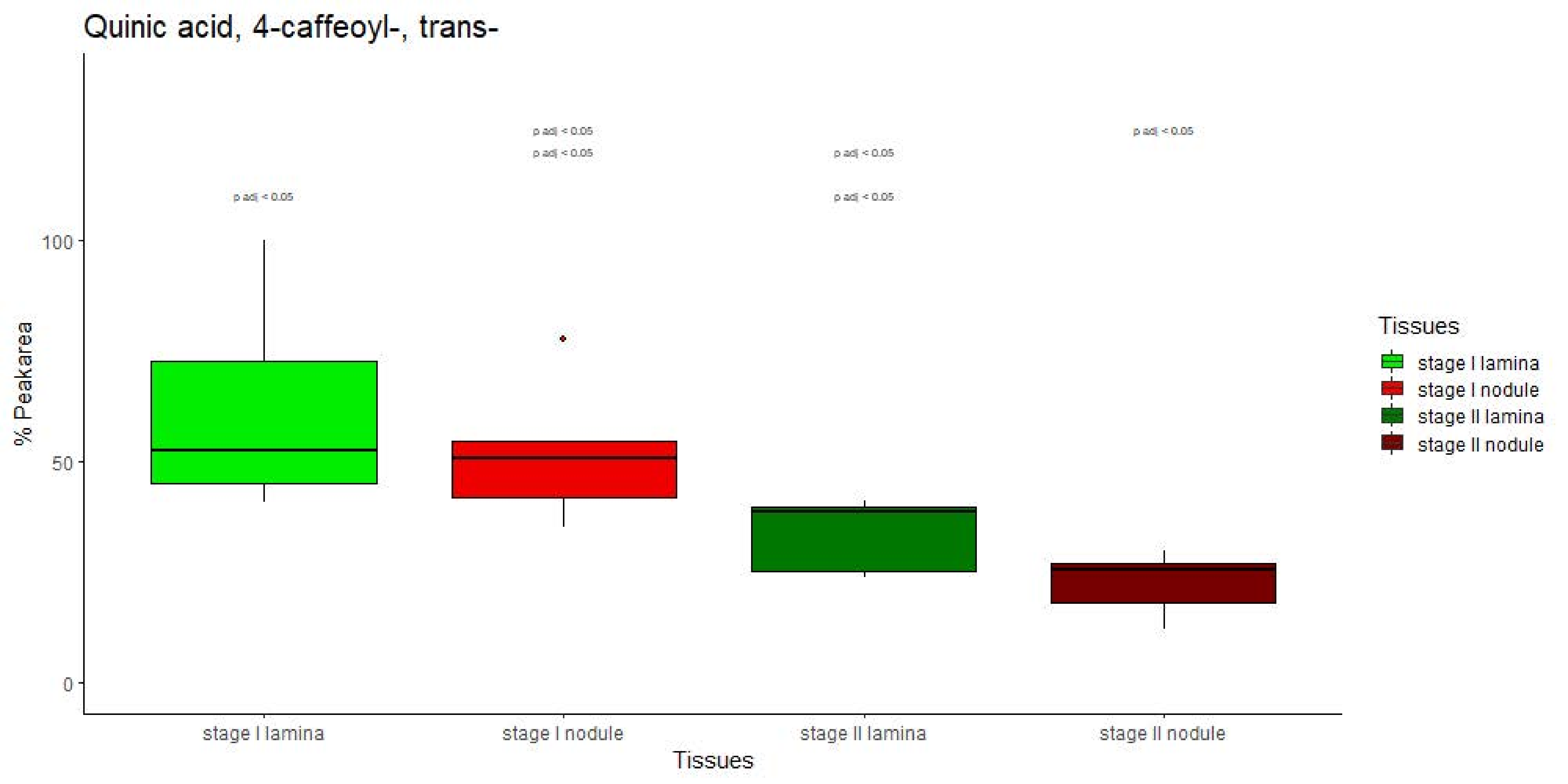
Quinic acid, 4-caffeoyl-, trans-
p adj < 0.05
p adj < 0.05
p adj < 0.05
p adj < 0.05
p adj < 0.05
p adj < 0.05
100
50
0
% Peakarea
stage I lamina
stage I nodule
stage II lamina
stage II nodule
Tissues
Tissues
stage I lamina
stage I nodule
stage II lamina
stage II nodule

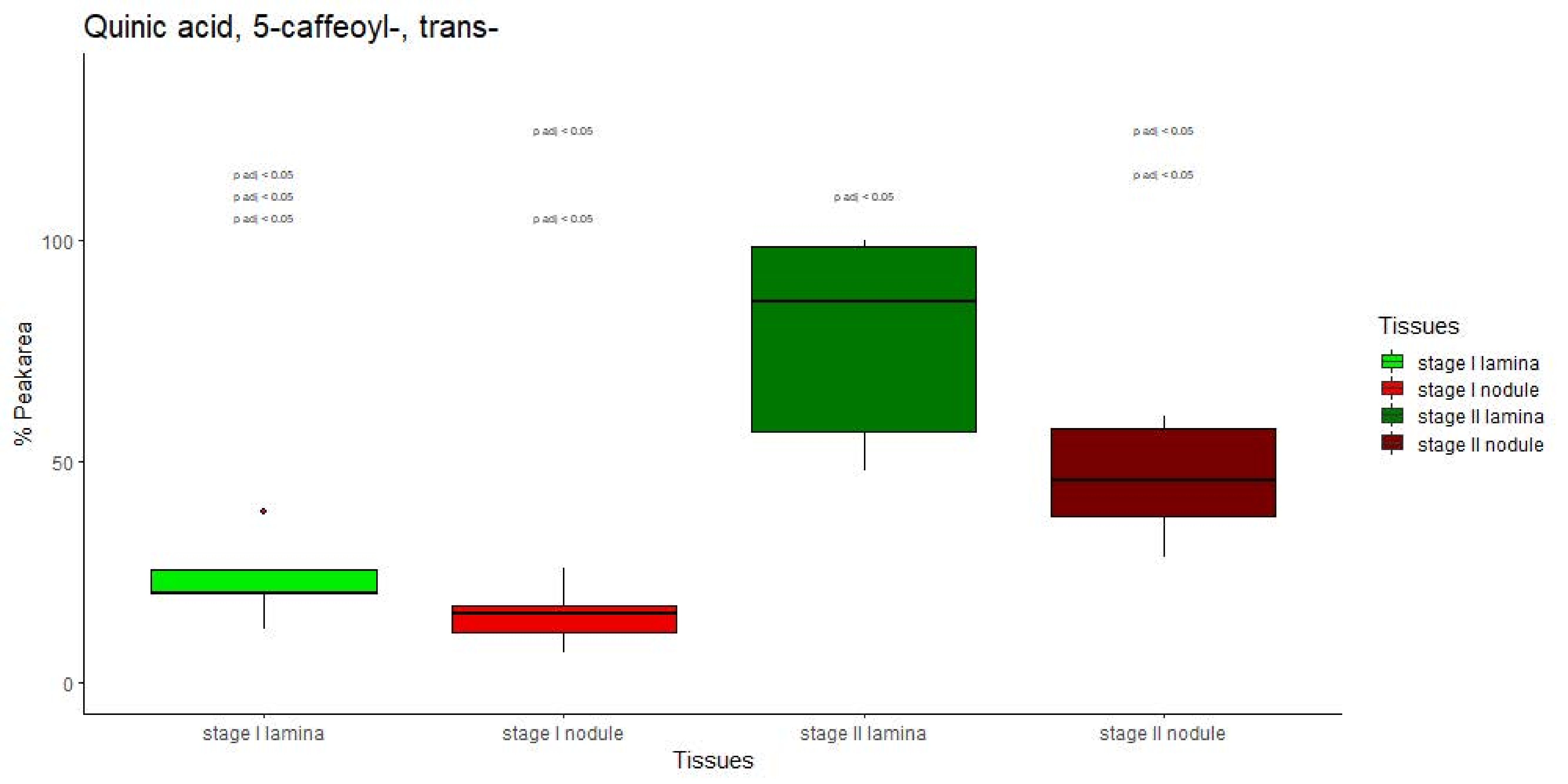
Quinic acid, 5-caffeoyl-, trans-
p adj < 0.05
p adj < 0.05
p adj < 0.05
p adj < 0.05
p adj < 0.05
p adj < 0.05
p adj < 0.05
p adj < 0.05
p adj < 0.05
100
50
0
% Peakarea
stage I lamina
stage I nodule
stage II lamina
stage II nodule
Tissues
Tissues
stage I lamina
stage I nodule
stage II lamina
stage II nodule

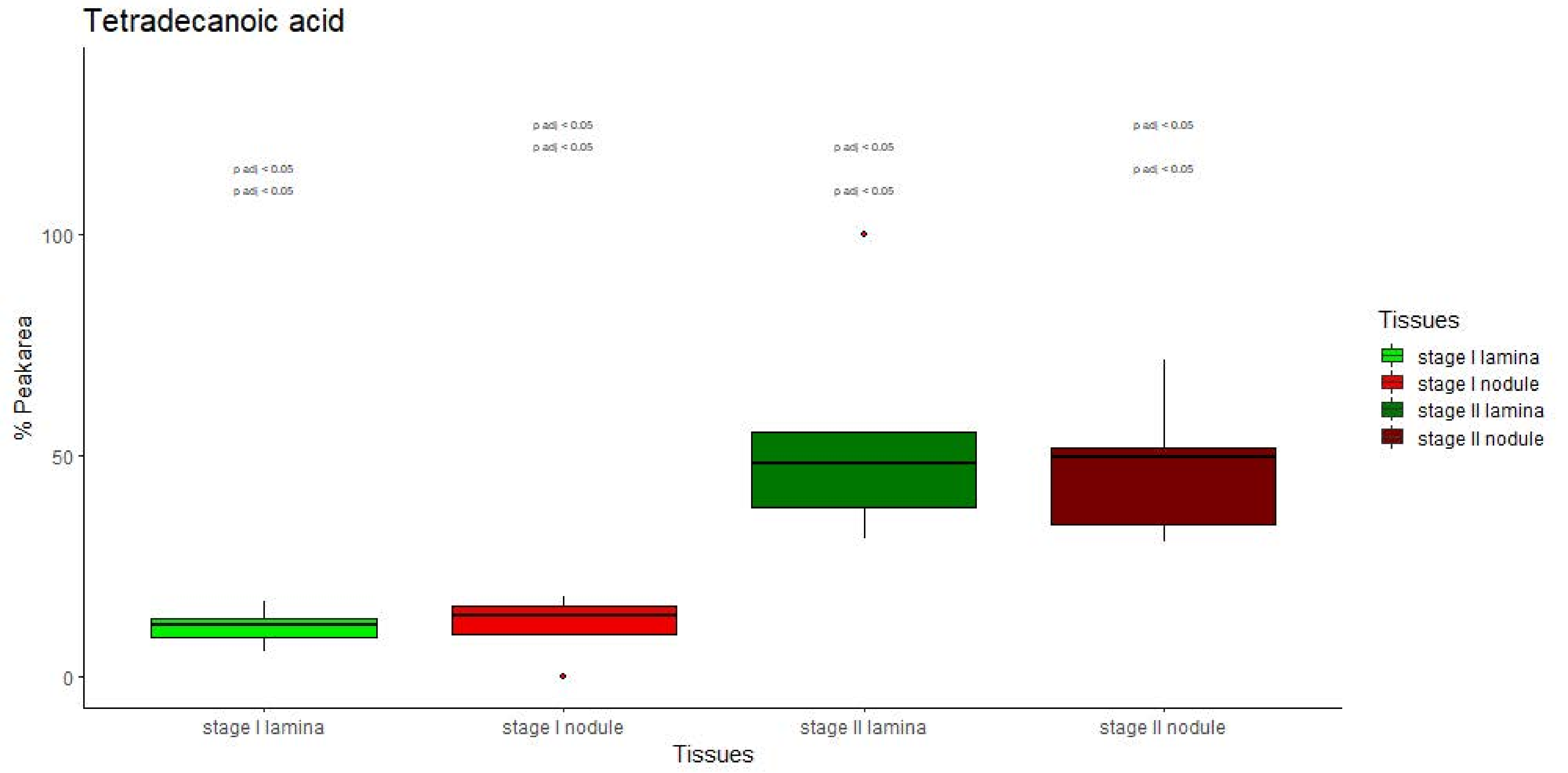

Tetradecanoic acid
p adj < 0.05
p adj < 0.05
p adj < 0.05
p adj < 0.05
p adj < 0.05
p adj < 0.05
p adj < 0.05
p adj < 0.05
100
50
0
% Peakarea
stage I lamina
stage I nodule
stage II lamina
stage II nodule
Tissues
Tissues
stage I lamina
stage I nodule
stage II lamina
stage II nodule

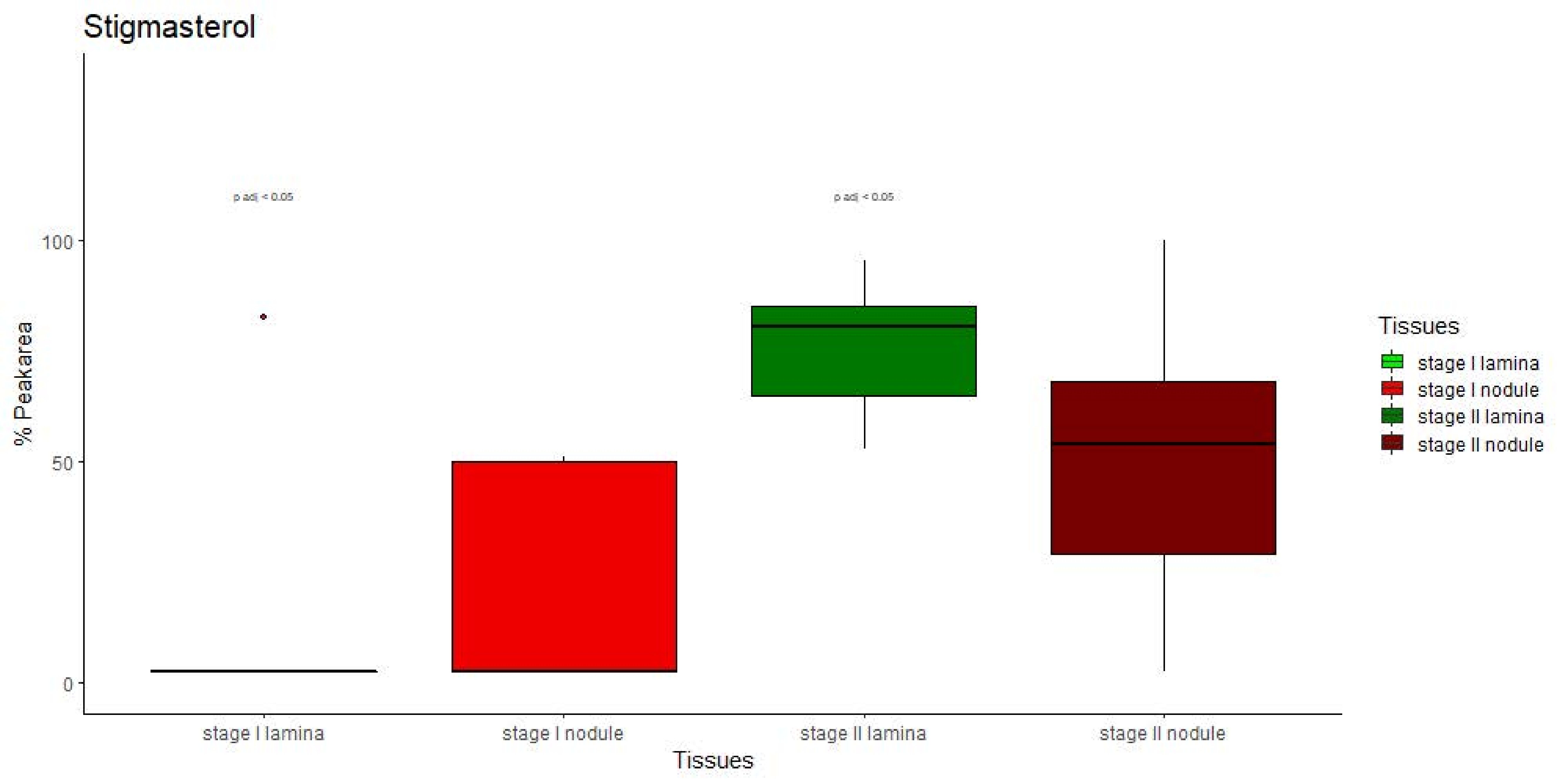

Stigmasterol
p adj < 0.05
p adj < 0.05
100
50
0
% Peakarea
stage I lamina
stage I nodule
stage II lamina
stage II nodule
Tissues
Tissues
stage I lamina
stage I nodule
stage II lamina
stage II nodule

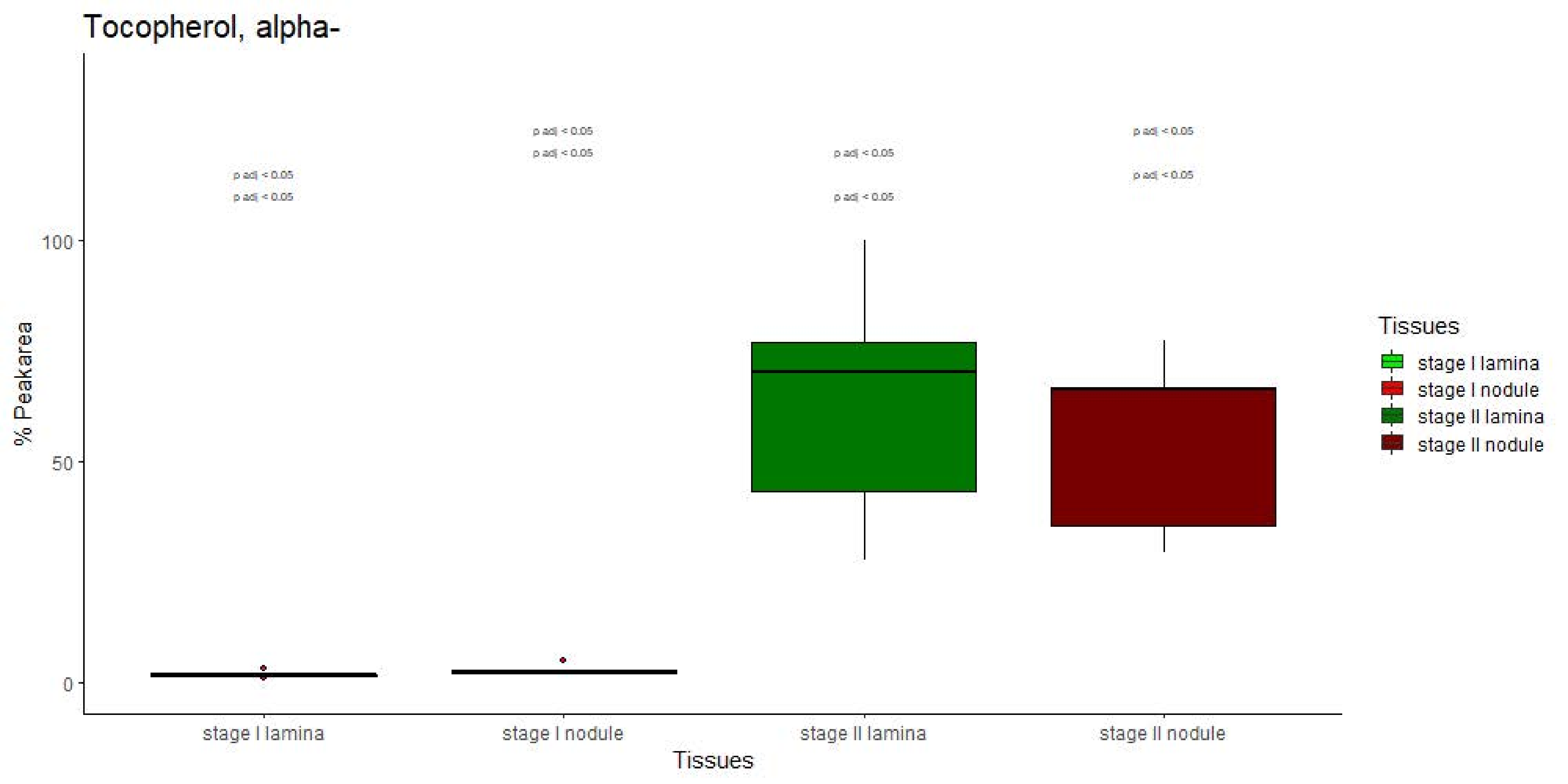

Tocopherol, alpha-
p adj < 0.05
p adj < 0.05
p adj < 0.05
p adj < 0.05
p adj < 0.05
p adj < 0.05
p adj < 0.05
p adj < 0.05
100
50
0
% Peakarea
stage I lamina
stage I nodule
stage II lamina
stage II nodule
Tissues
Tissues
stage I lamina
stage I nodule
stage II lamina
stage II nodule

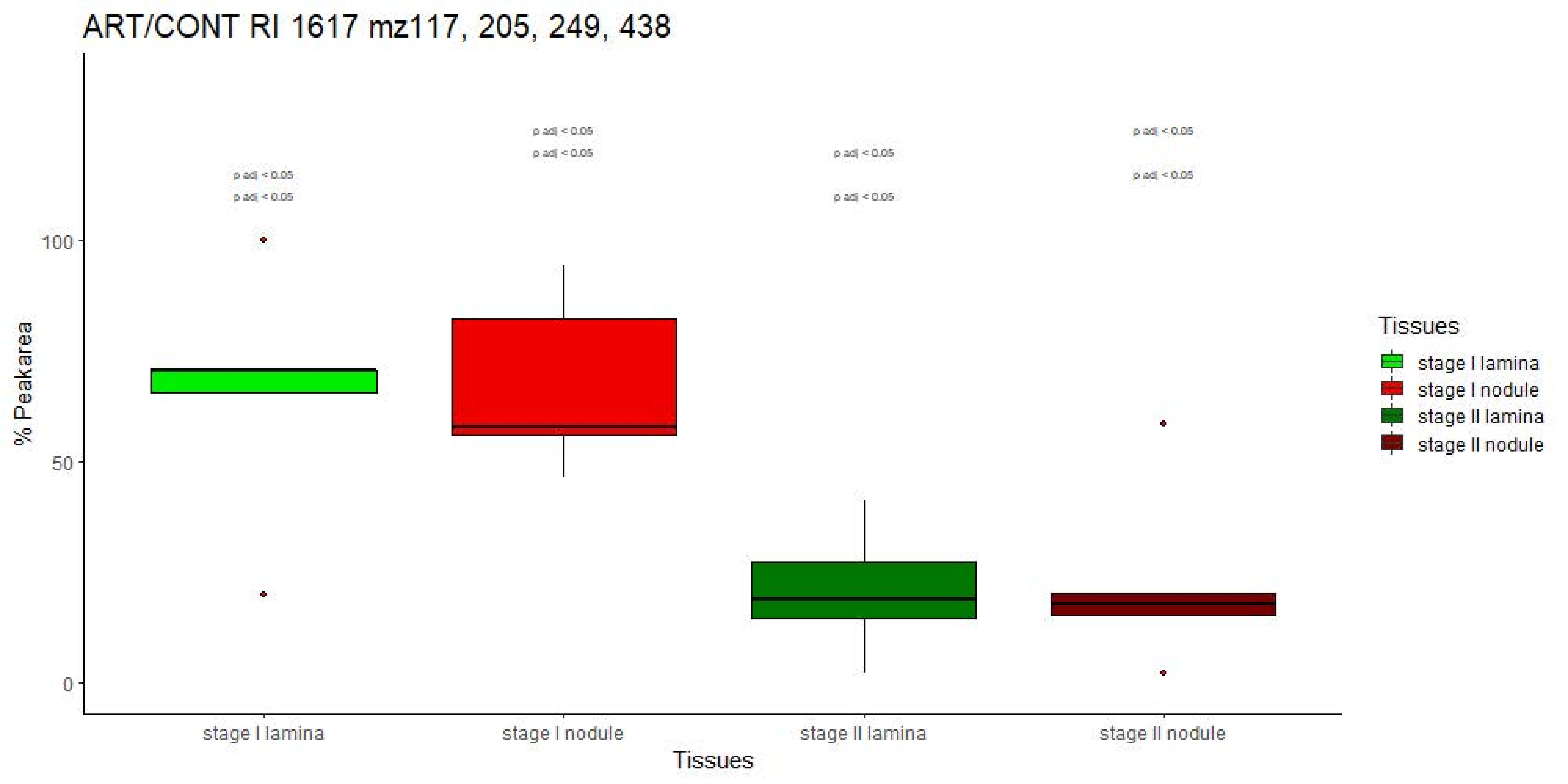

ART/CONT RI 1617 mz117, 205, 249, 438
p adj < 0.05
p adj < 0.05
p adj < 0.05
p adj < 0.05
p adj < 0.05
p adj < 0.05
p adj < 0.05
p adj < 0.05
100
50
0
% Peakarea
stage I lamina
stage I nodule
stage II lamina
stage II nodule
Tissues
Tissues
stage I lamina
stage I nodule
stage II lamina
stage II nodule

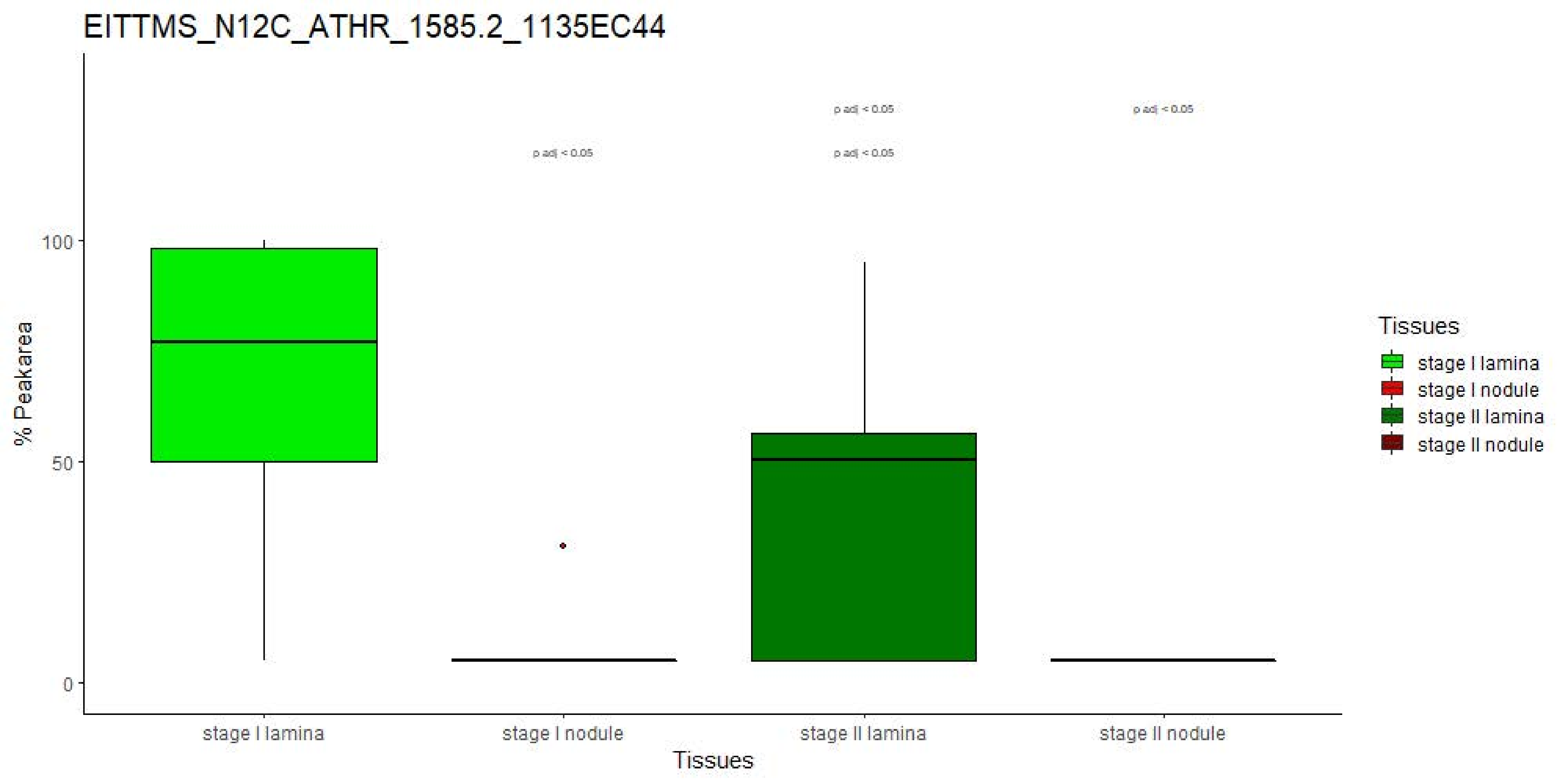
EITTMS_N12C_ATHR_1585.2_1135EC44
p adj < 0.05
p adj < 0.05
p adj < 0.05
p adj < 0.05
% Peakarea
100
50
0
stage I lamina
stage I nodule
stage II lamina
stage II nodule
Tissues
Tissues
stage I lamina
stage I nodule
stage II lamina
stage II nodule

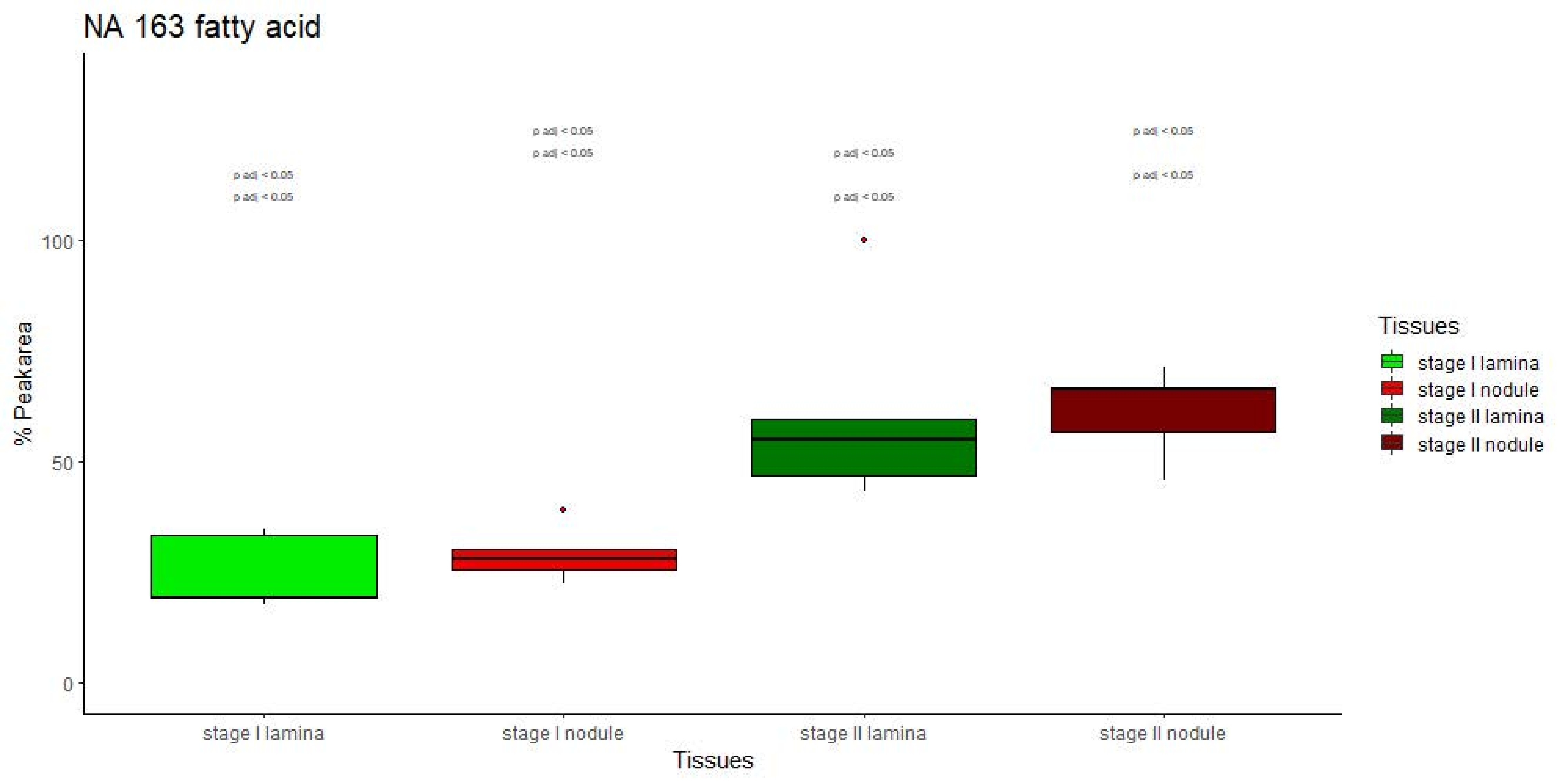

NA 163 fatty acid
% Peakarea
100
50
0
p adj < 0.05
p adj < 0.05
p adj < 0.05
p adj < 0.05
p adj < 0.05
p adj < 0.05
p adj < 0.05
p adj < 0.05
stage I lamina
stage I nodule
stage II lamina
stage II nodule
Tissues
Tissues
stage I lamina
stage I nodule
stage II lamina
stage II nodule

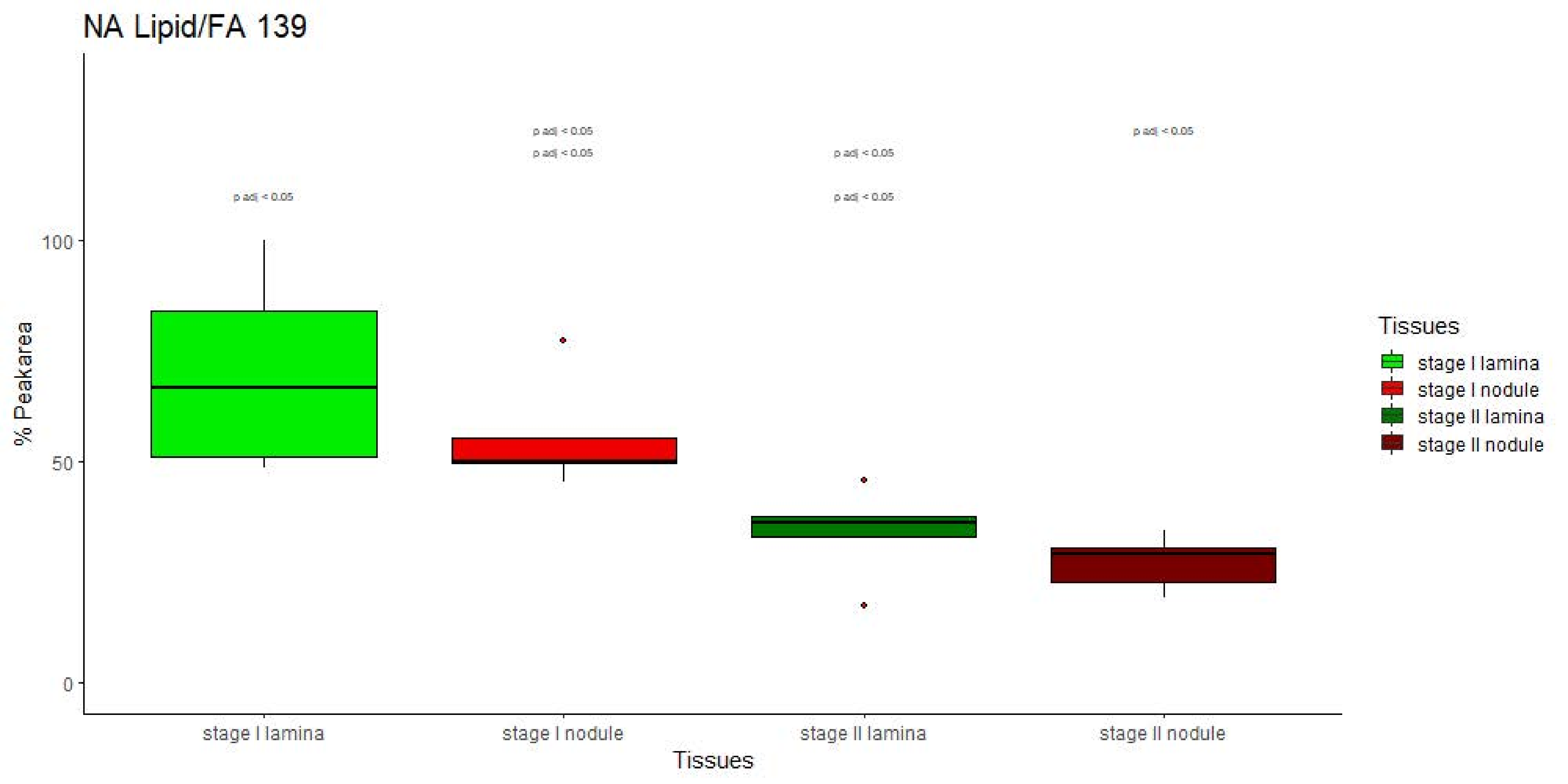
NA Lipid/FA 139
p adj < 0.05
p adj < 0.05
p adj < 0.05
p adj < 0.05
p adj < 0.05
p adj < 0.05
100
50
0
% Peakarea
stage I lamina
stage I nodule
stage II lamina
stage II nodule
Tissues
Tissues
stage I lamina
stage I nodule
stage II lamina
stage II nodule

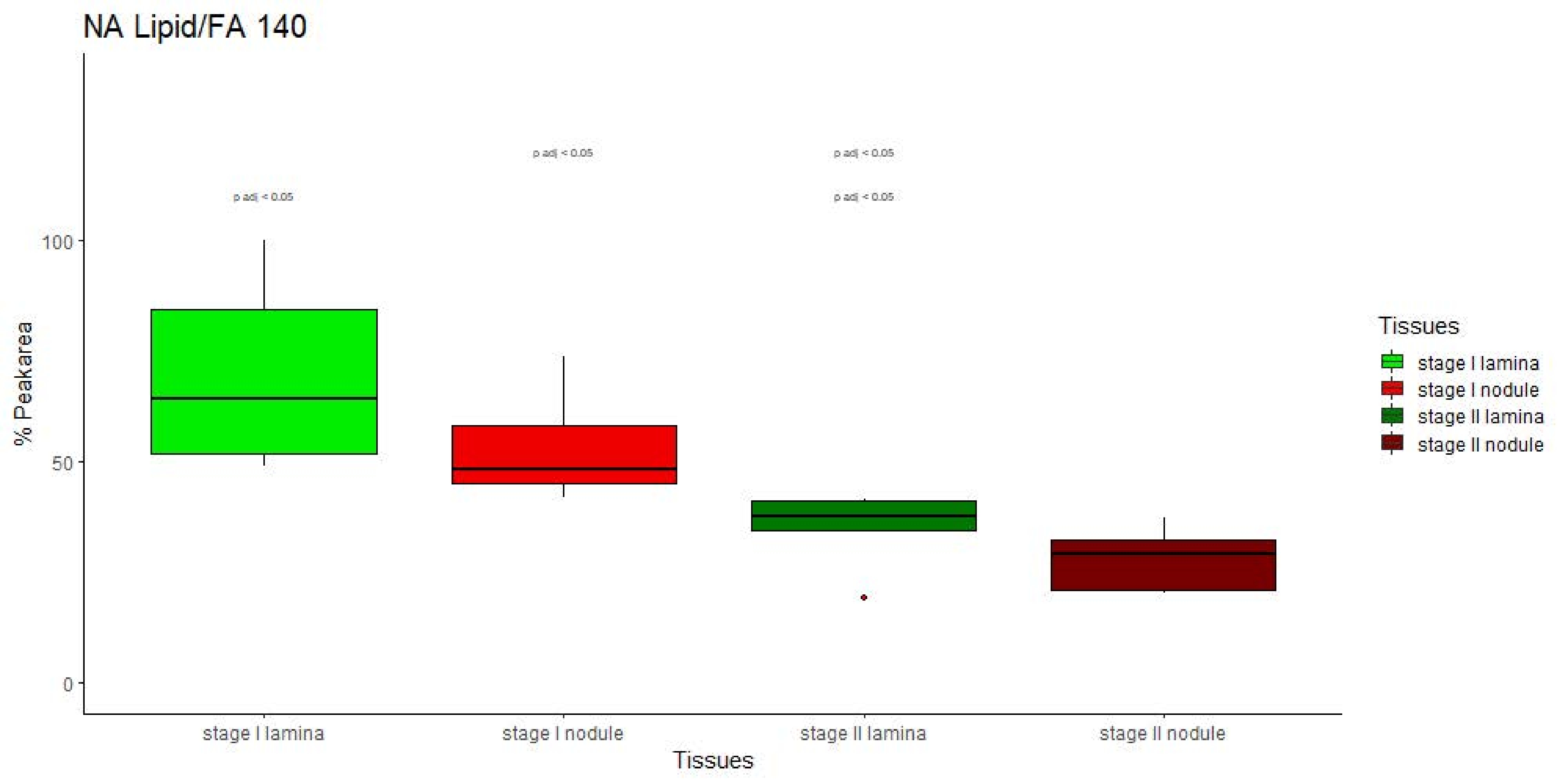

NA Lipid/FA 140
p adj < 0.05
p adj < 0.05
p adj < 0.05
p adj < 0.05
100
50
0
% Peakarea
stage I lamina
stage I nodule
stage II lamina
stage II nodule
Tissues
Tissues
stage I lamina
stage I nodule
stage II lamina
stage II nodule

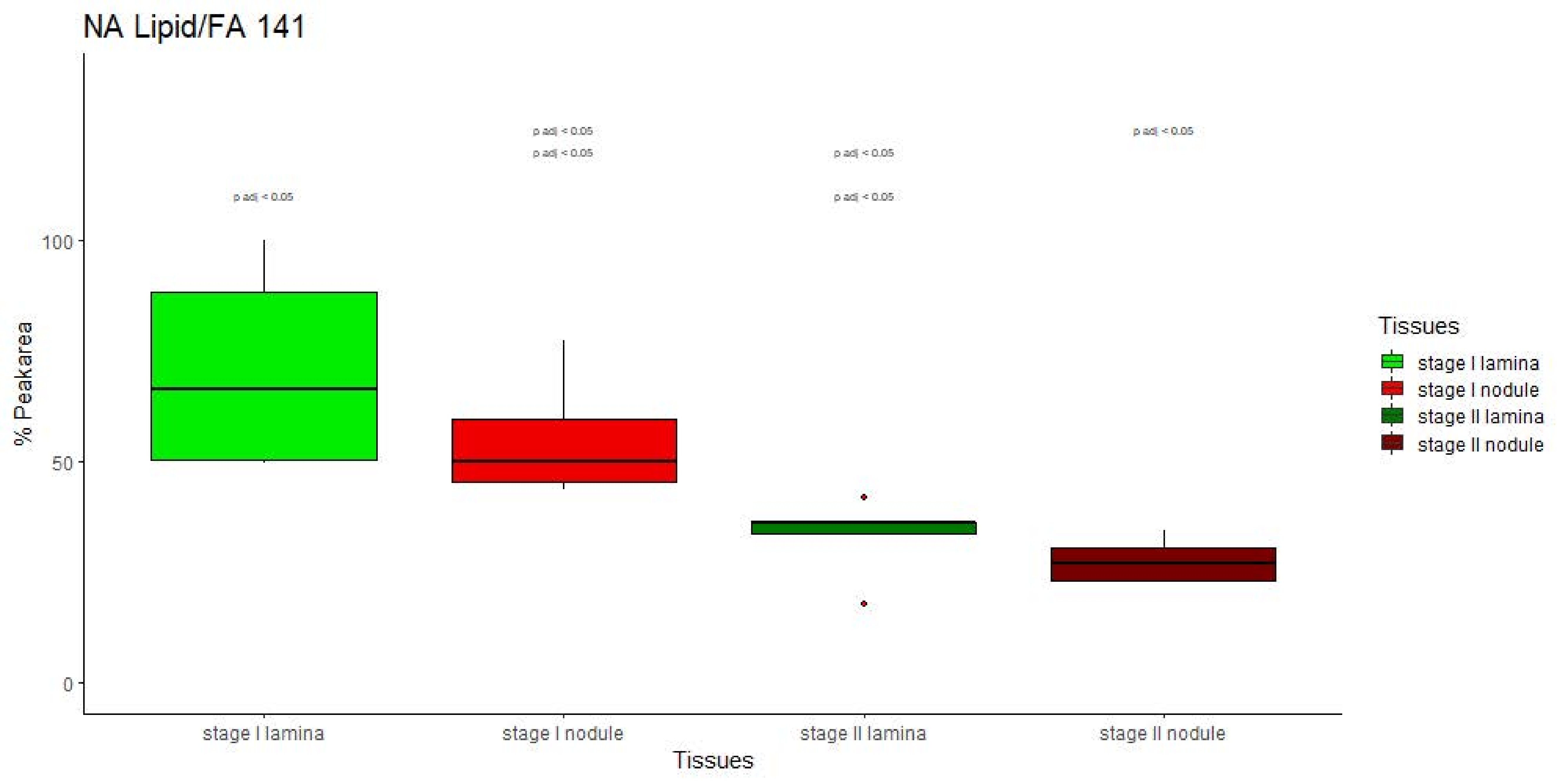
NA Lipid/FA 141
p adj < 0.05
p adj < 0.05
p adj < 0.05
p adj < 0.05
p adj < 0.05
p adj < 0.05
100
50
0
% Peakarea
stage I lamina
stage I nodule
stage II lamina
stage II nodule
Tissues
Tissues
stage I lamina
stage I nodule
stage II lamina
stage II nodule

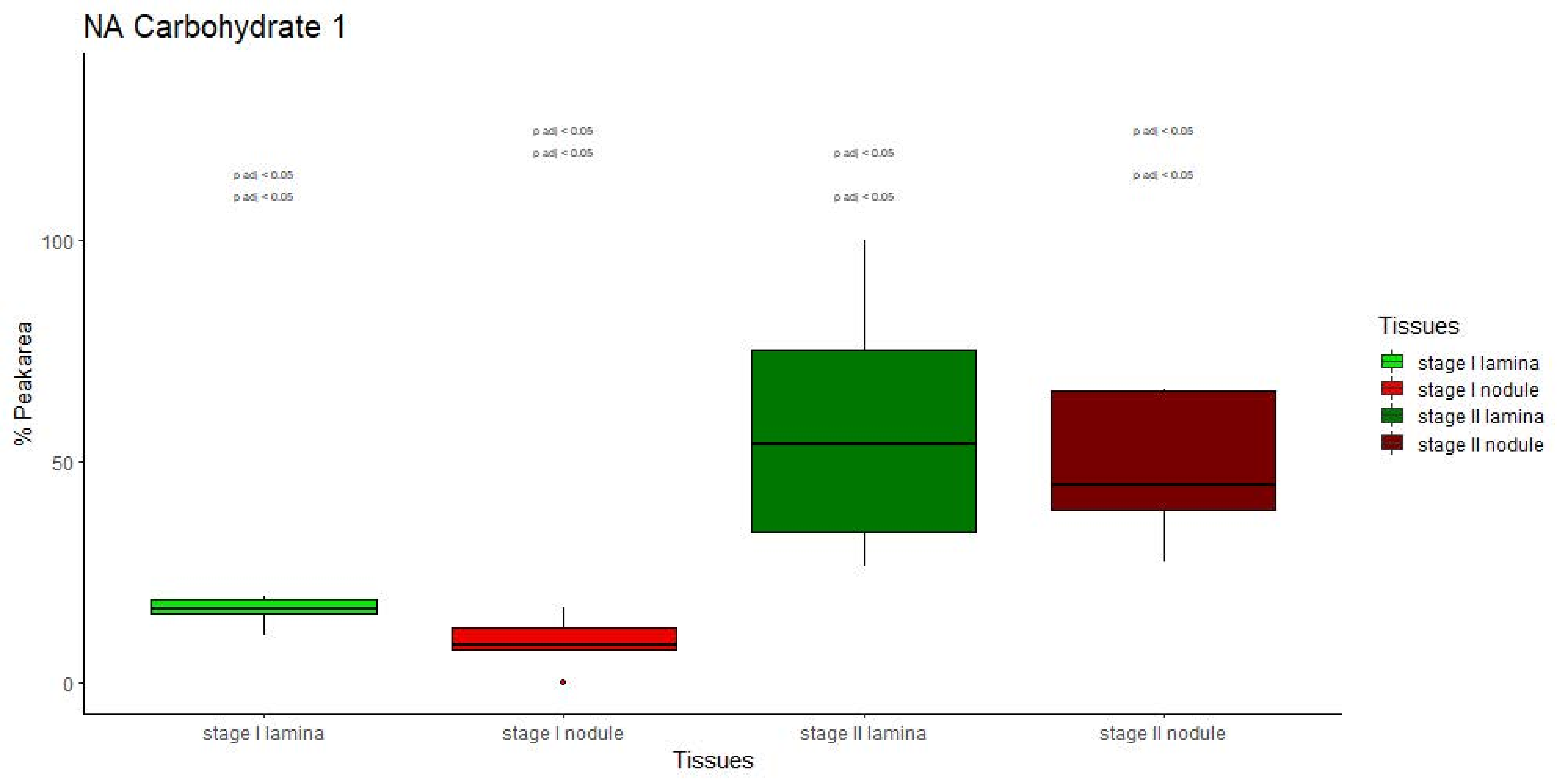
NA Carbohydrate 1
p adj < 0.05
p adj < 0.05
p adj < 0.05
p adj < 0.05
p adj < 0.05
p adj < 0.05
p adj < 0.05
p adj < 0.05
100
50
0
% Peakarea
stage I lamina
stage I nodule
stage II lamina
stage II nodule
Tissues
Tissues
stage I lamina
stage I nodule
stage II lamina
stage II nodule

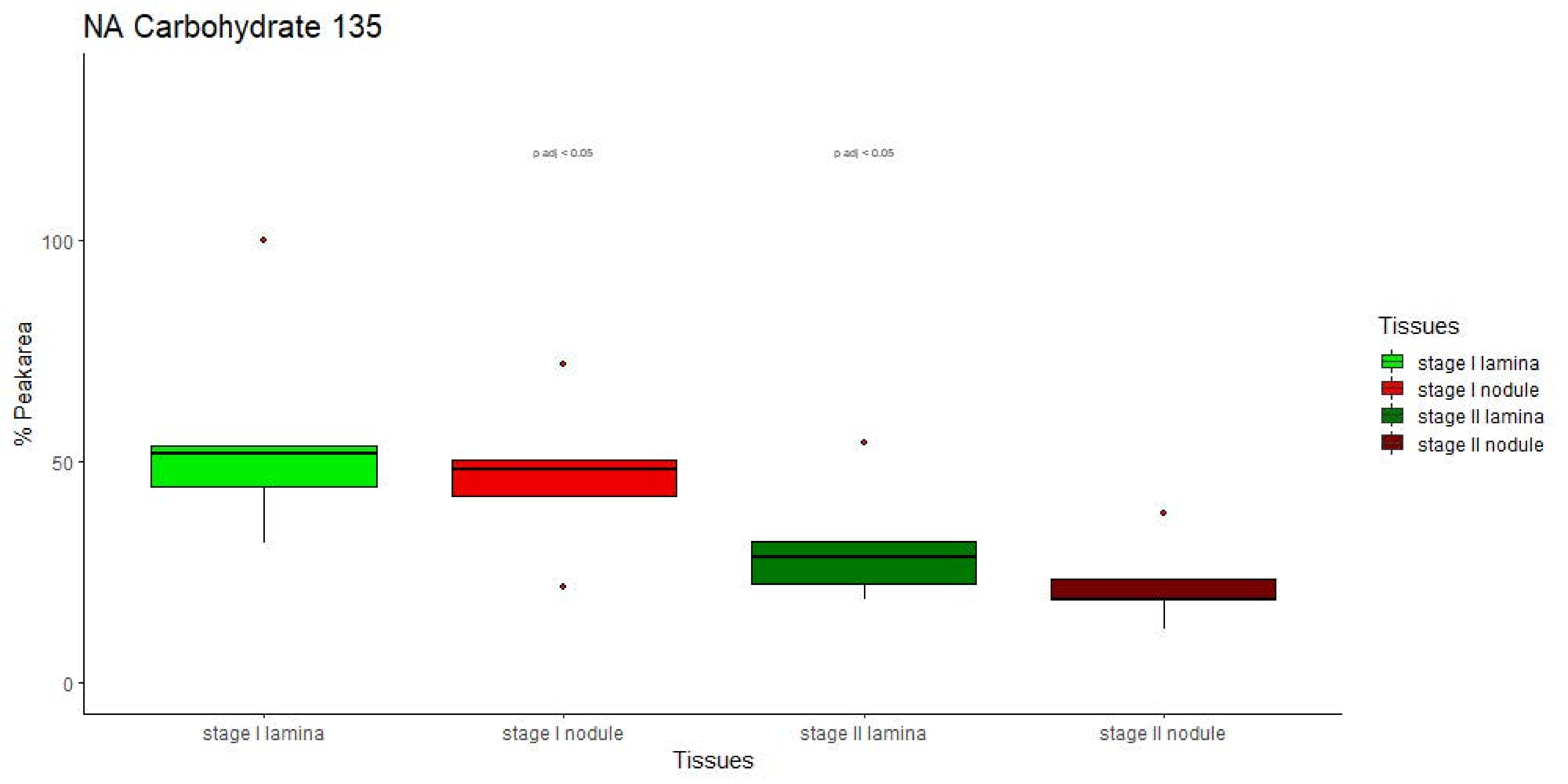

NA Carbohydrate 135
p adj < 0.05
p adj < 0.05
% Peakarea
100
50
0
stage I lamina
stage I nodule
stage II lamina
stage II nodule
Tissues
Tissues
stage I lamina
stage I nodule
stage II lamina
stage II nodule

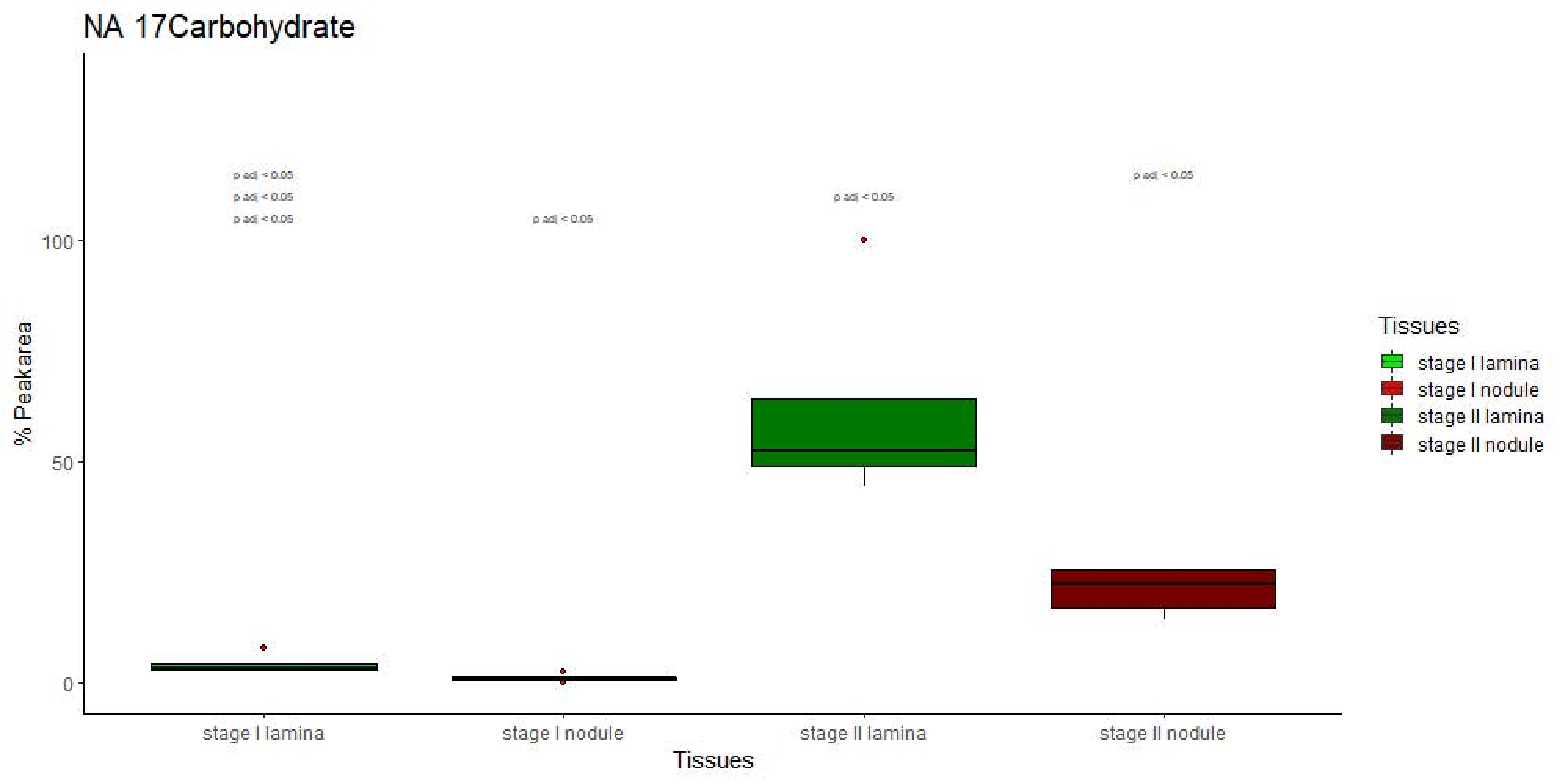
NA 17Carbohydrate
p adj < 0.05
p adj < 0.05
p adj < 0.05
p adj < 0.05
p adj < 0.05
p adj < 0.05
100
50
0
% Peakarea
stage I lamina
stage I nodule
stage II lamina
stage II nodule
Tissues
Tissues
stage I lamina
stage I nodule
stage II lamina
stage II nodule

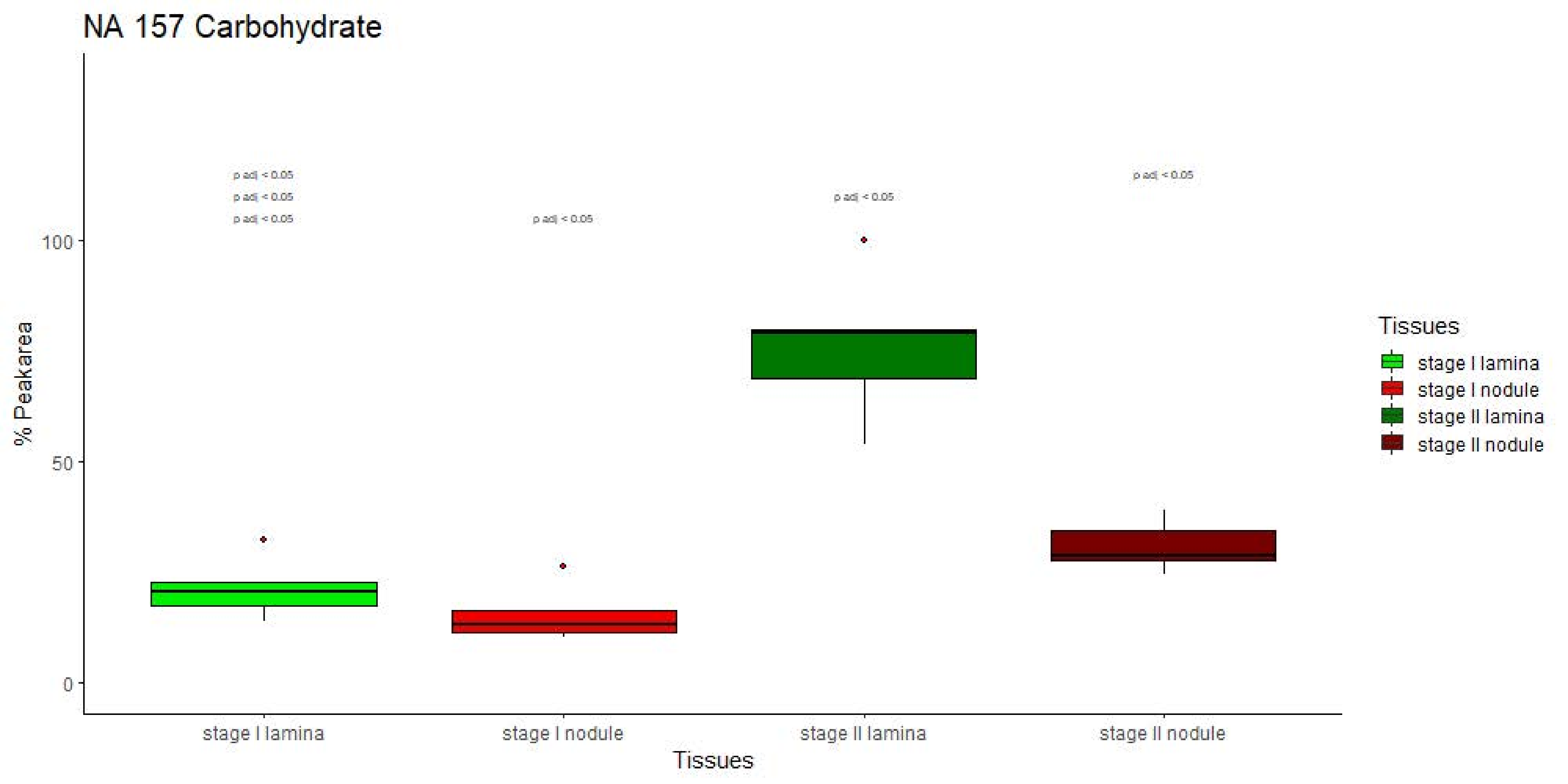
NA 157 Carbohydrate
p adj < 0.05
p adj < 0.05
p adj < 0.05
p adj < 0.05
p adj < 0.05
p adj < 0.05
100
50
0
% Peakarea
stage I lamina
stage I nodule
stage II lamina
stage II nodule
Tissues
Tissues
stage I lamina
stage I nodule
stage II lamina
stage II nodule

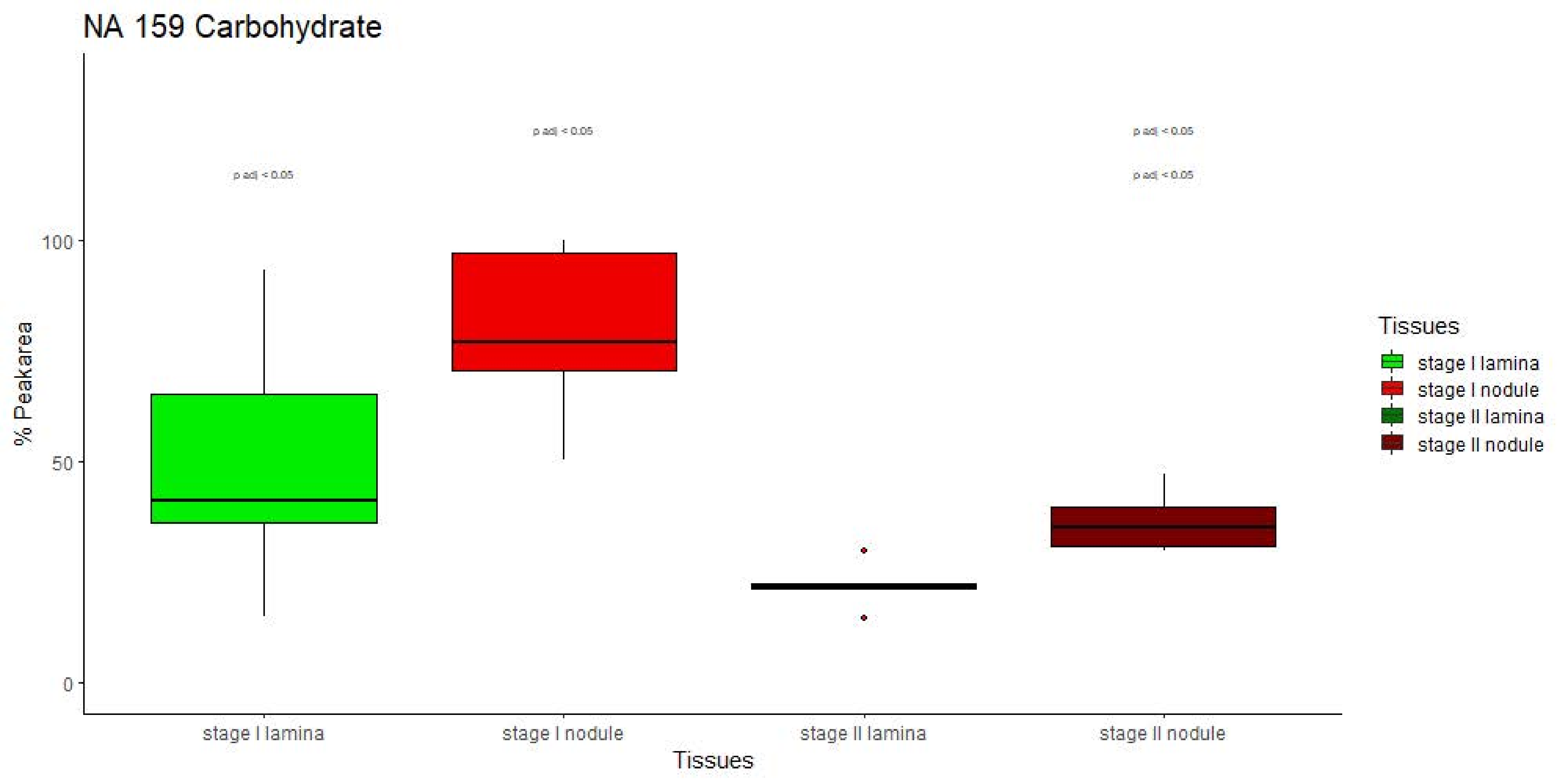
NA 159 Carbohydrate
p adj < 0.05
p adj < 0.05
p adj < 0.05
p adj < 0.05
100
50
0
% Peakarea
stage I lamina
stage I nodule
stage II lamina
stage II nodule
Tissues
Tissues
stage I lamina
stage I nodule
stage II lamina
stage II nodule

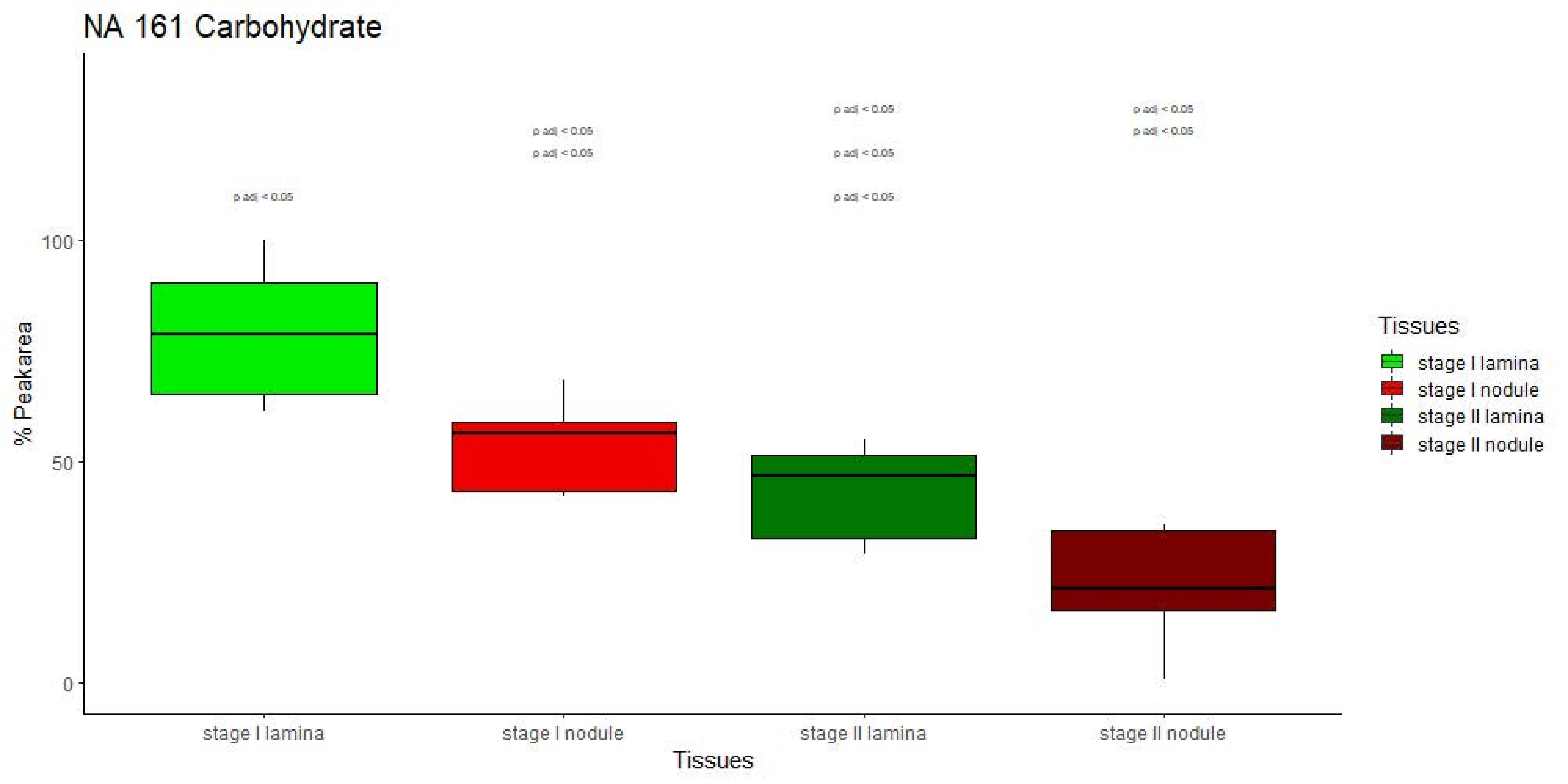
NA 161 Carbohydrate
p adj < 0.05
p adj < 0.05
p adj < 0.05
p adj < 0.05
p adj < 0.05
p adj < 0.05
p adj < 0.05
p adj < 0.05
100
50
0
% Peakarea
stage I lamina
stage I nodule
stage II lamina
stage II nodule
Tissues
Tissues
stage I lamina
stage I nodule
stage II lamina
stage II nodule

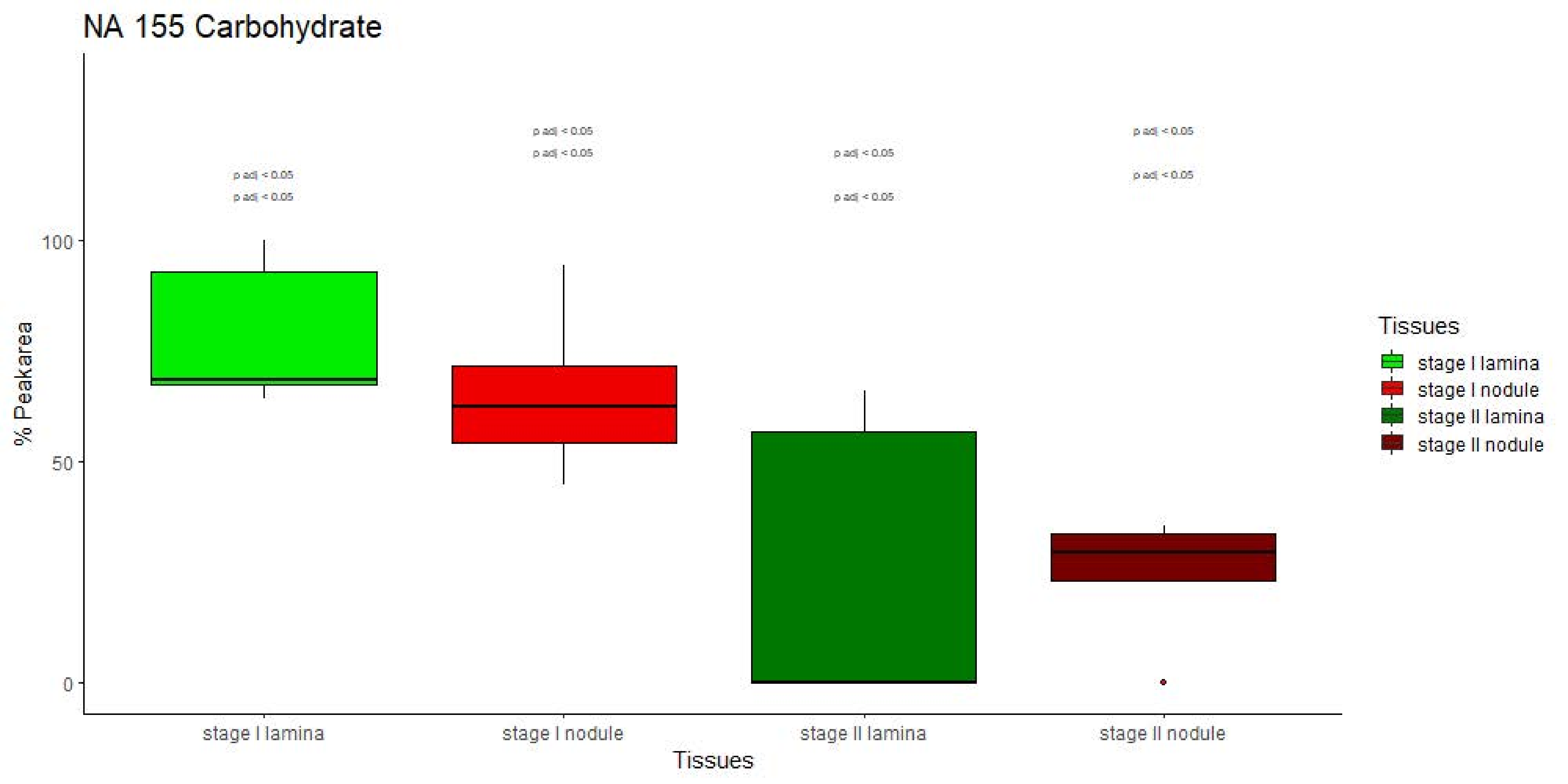

NA 155 Carbohydrate
p adj < 0.05
p adj < 0.05
p adj < 0.05
p adj < 0.05
p adj < 0.05
p adj < 0.05
p adj < 0.05
p adj < 0.05
100
50
0
% Peakarea
stage I lamina
stage I nodule
stage II lamina
stage II nodule
Tissues
Tissues
stage I lamina
stage I nodule
stage II lamina
stage II nodule

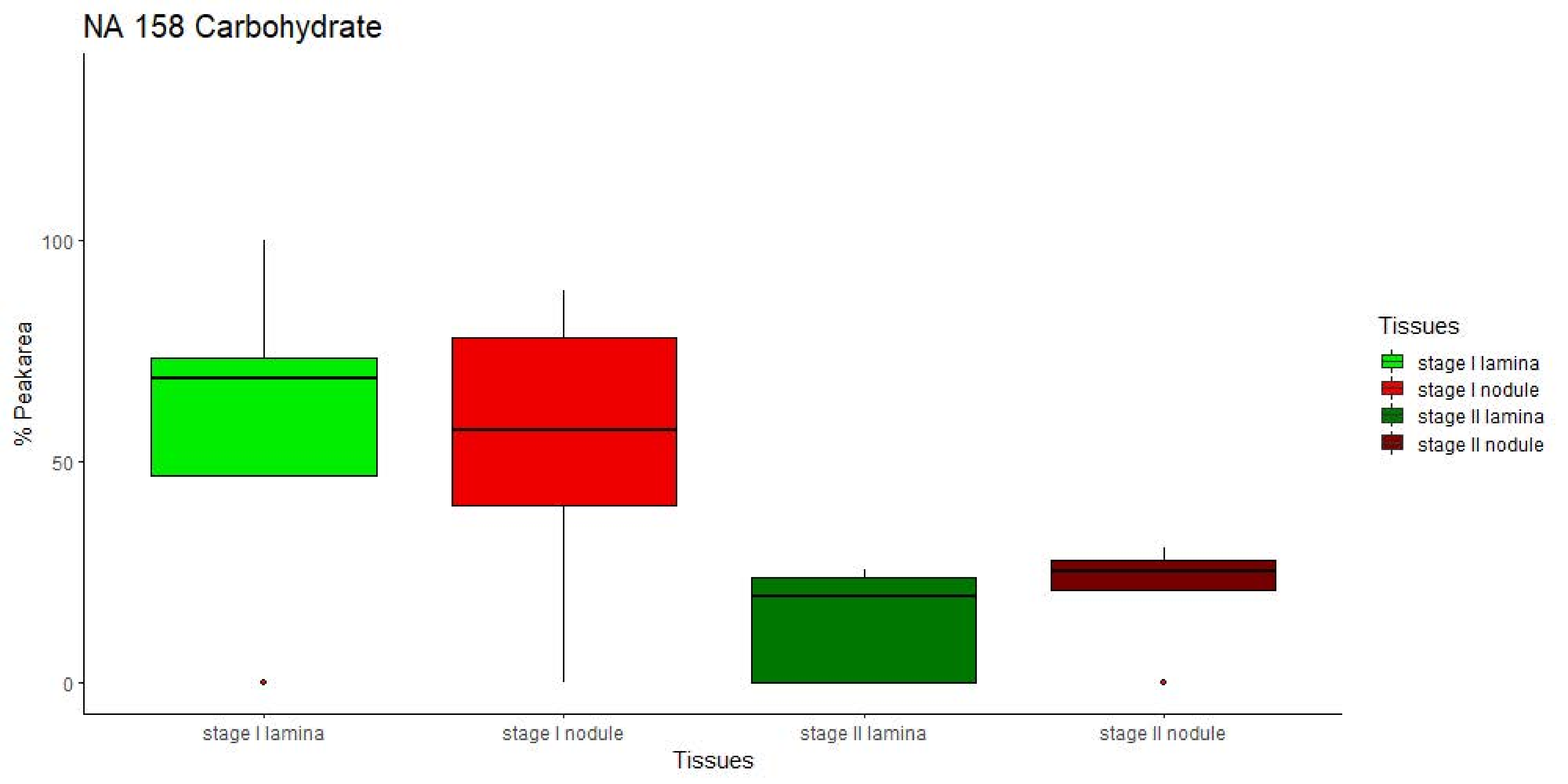

NA 158 Carbohydrate
% Peakarea
100
50
0
stage I lamina
stage I nodule
stage II lamina
stage II nodule
Tissues
Tissues
stage I lamina
stage I nodule
stage II lamina
stage II nodule

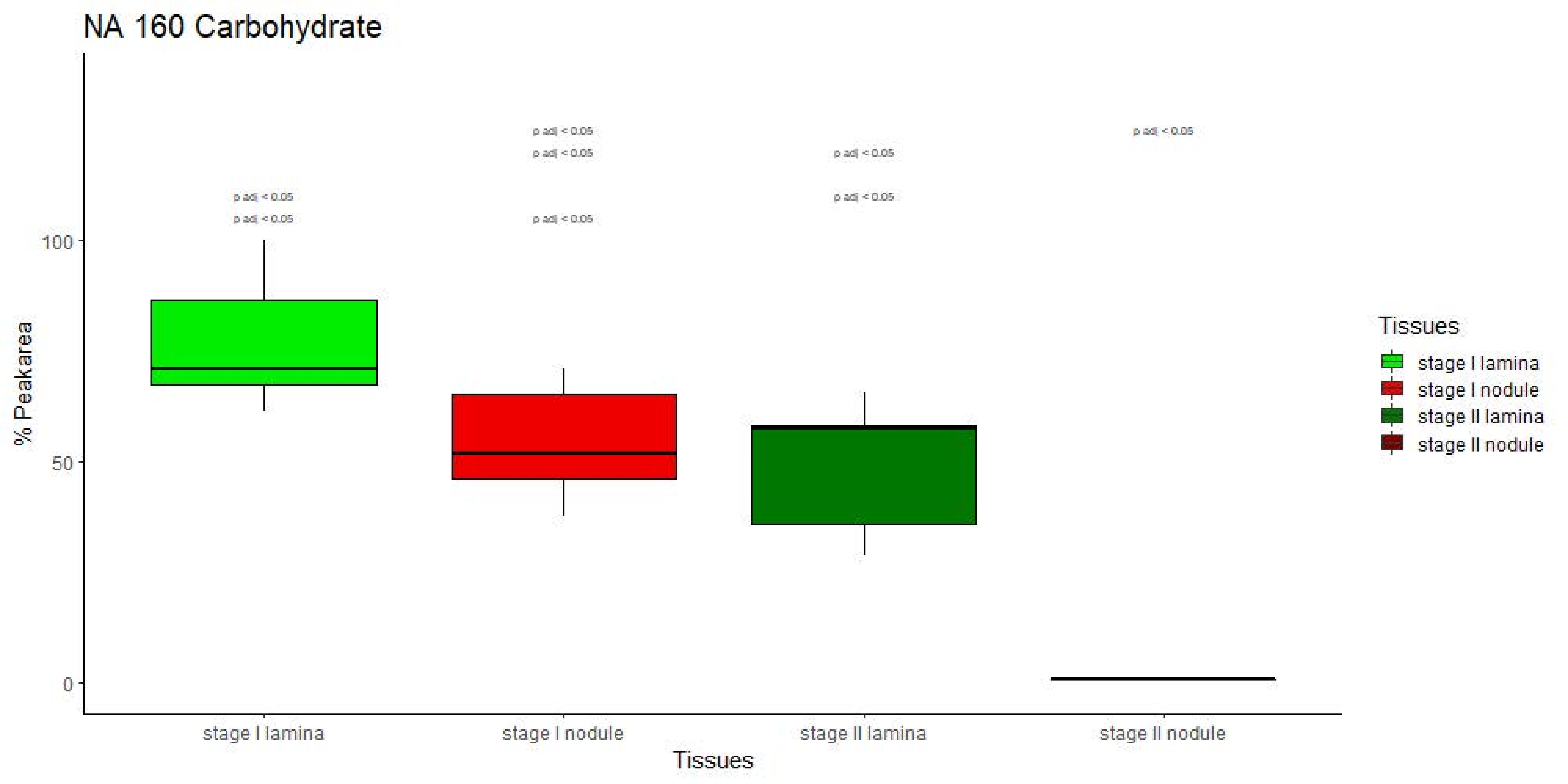

NA 160 Carbohydrate
% Peakarea
100
50
0
p adj < 0.05
p adj < 0.05
p adj < 0.05
p adj < 0.05
p adj < 0.05
p adj < 0.05
p adj < 0.05
p adj < 0.05
stage I lamina
stage I nodule
stage II lamina
stage II nodule
Tissues
Tissues
stage I lamina
stage I nodule
stage II lamina
stage II nodule

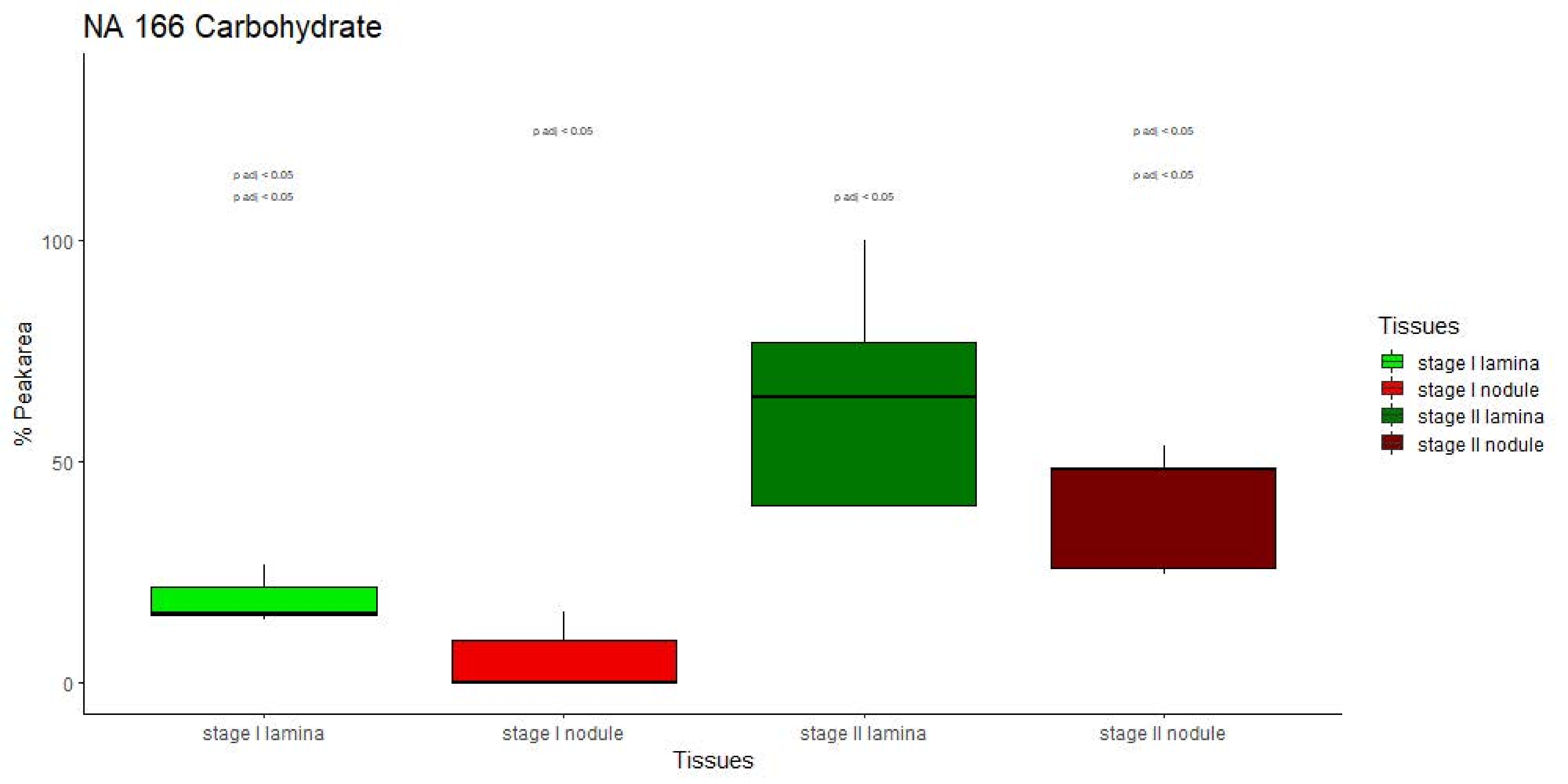
NA 166 Carbohydrate
p adj < 0.05
p adj < 0.05
p adj < 0.05
p adj < 0.05
p adj < 0.05
p adj < 0.05
p adj < 0.05
100
50
0
% Peakarea
stage I lamina
stage I nodule
stage II lamina
stage II nodule
Tissues
Tissues
stage I lamina
stage I nodule
stage II lamina
stage II nodule

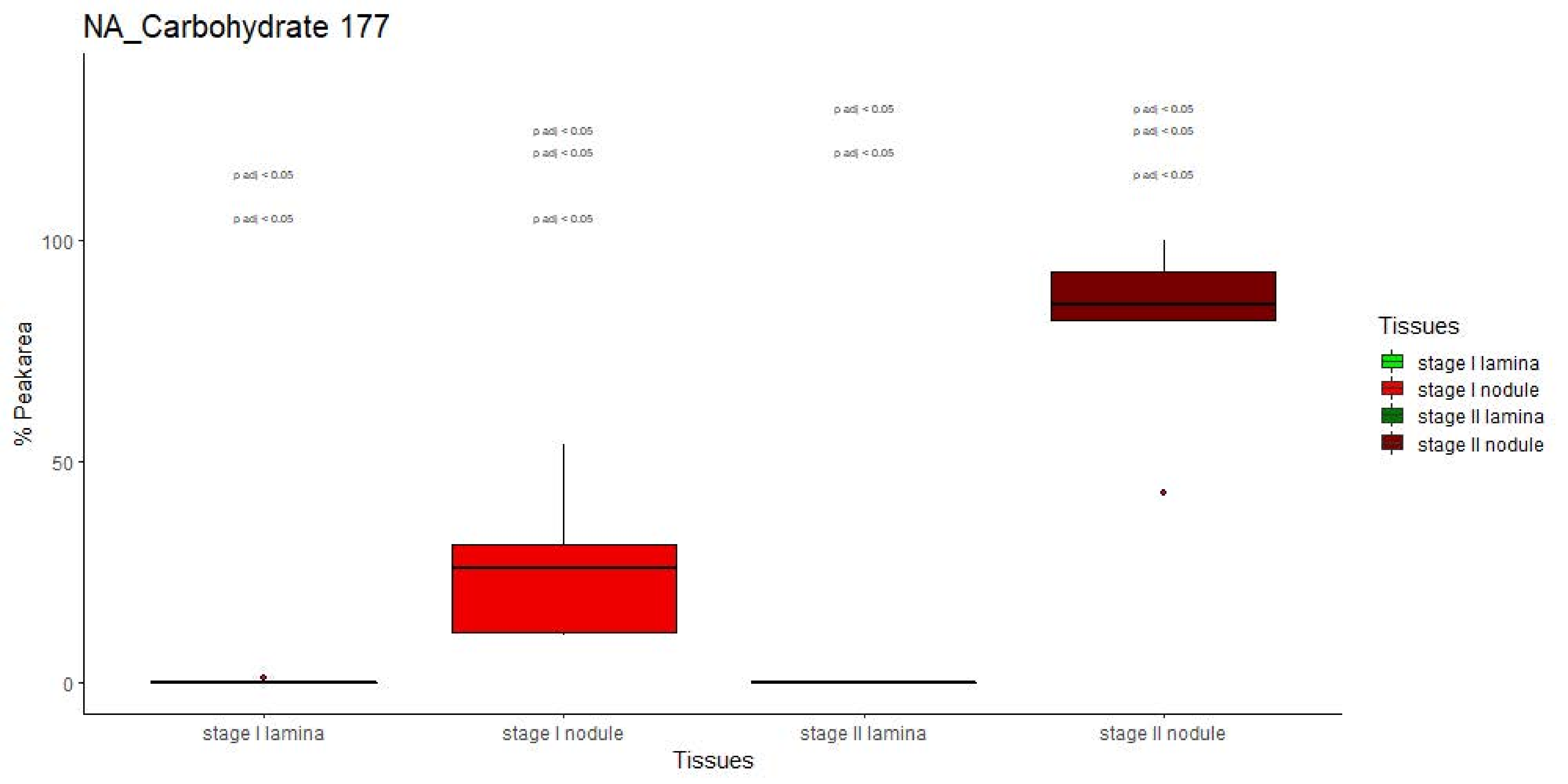

NA_Carbohydrate 177
p adj < 0.05
p adj < 0.05
p adj < 0.05
p adj < 0.05
p adj < 0.05
p adj < 0.05
p adj < 0.05
p adj < 0.05
p adj < 0.05
p adj < 0.05
p adj < 0.05
100
50
0
% Peakarea
stage I lamina
stage I nodule
stage II lamina
stage II nodule
Tissues
Tissues
stage I lamina
stage I nodule
stage II lamina
stage II nodule

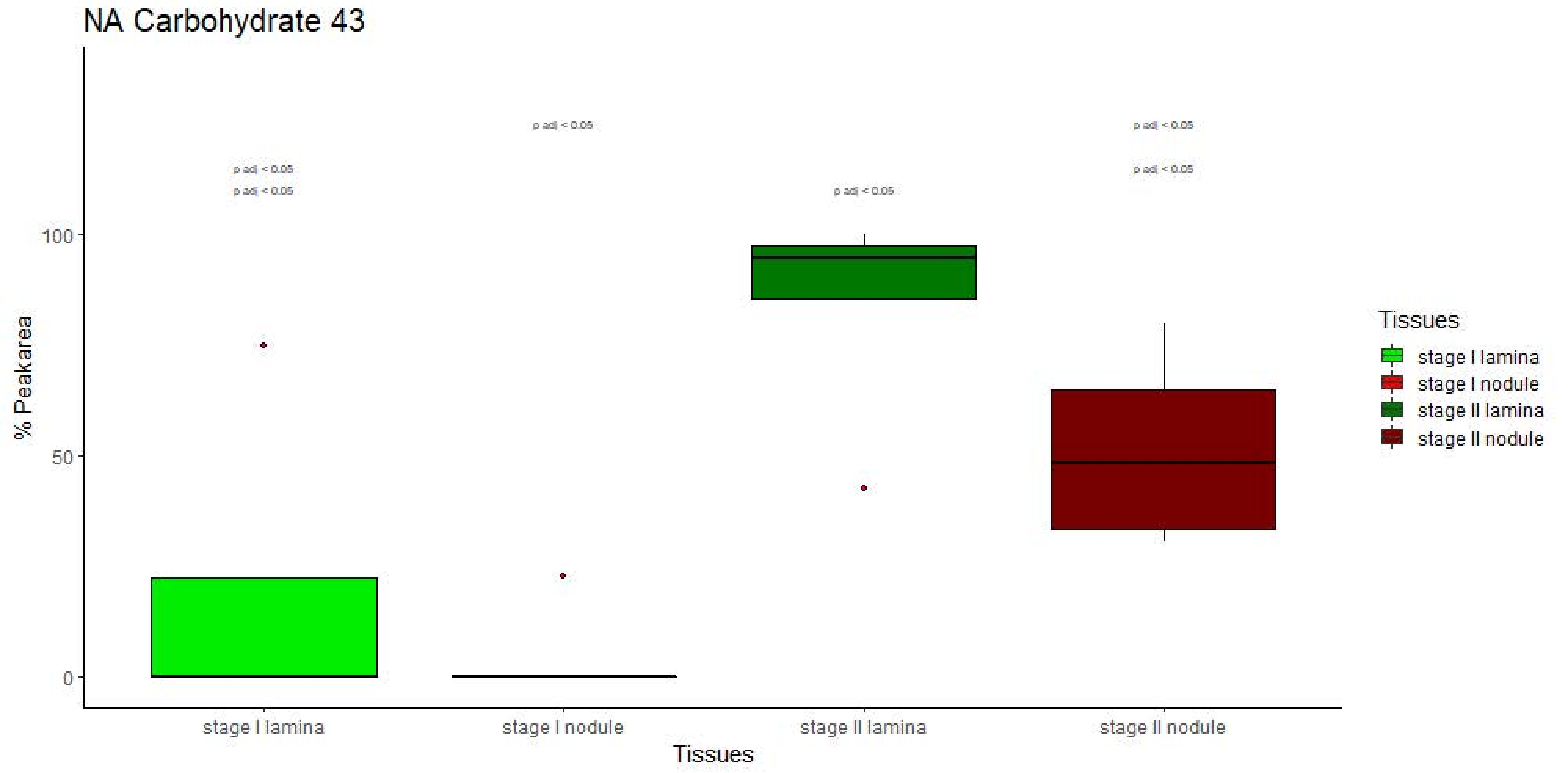

NA Carbohydrate 43
p adj < 0.05
p adj < 0.05
p adj < 0.05
p adj < 0.05
p adj < 0.05
p adj < 0.05
p adj < 0.05
100
50
0
% Peakarea
stage I lamina
stage I nodule
stage II lamina
stage II nodule
Tissues
Tissues
stage I lamina
stage I nodule
stage II lamina
stage II nodule

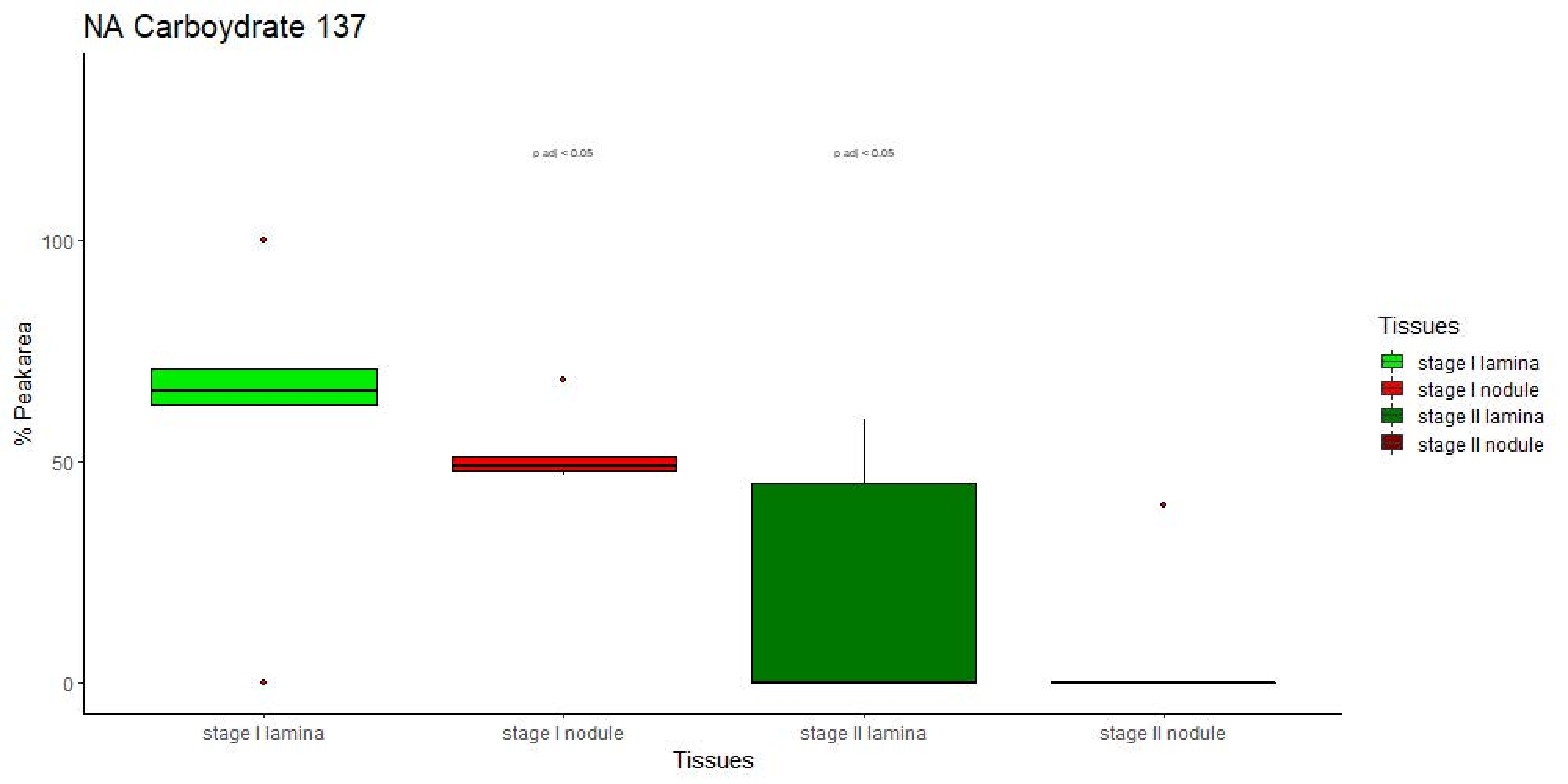

NA Carboydrate 137
p adj < 0.05
p adj < 0.05
100
50
0
% Peakarea
stage I lamina
stage I nodule
stage II lamina
stage II nodule
Tissues
Tissues
stage I lamina
stage I nodule
stage II lamina
stage II nodule

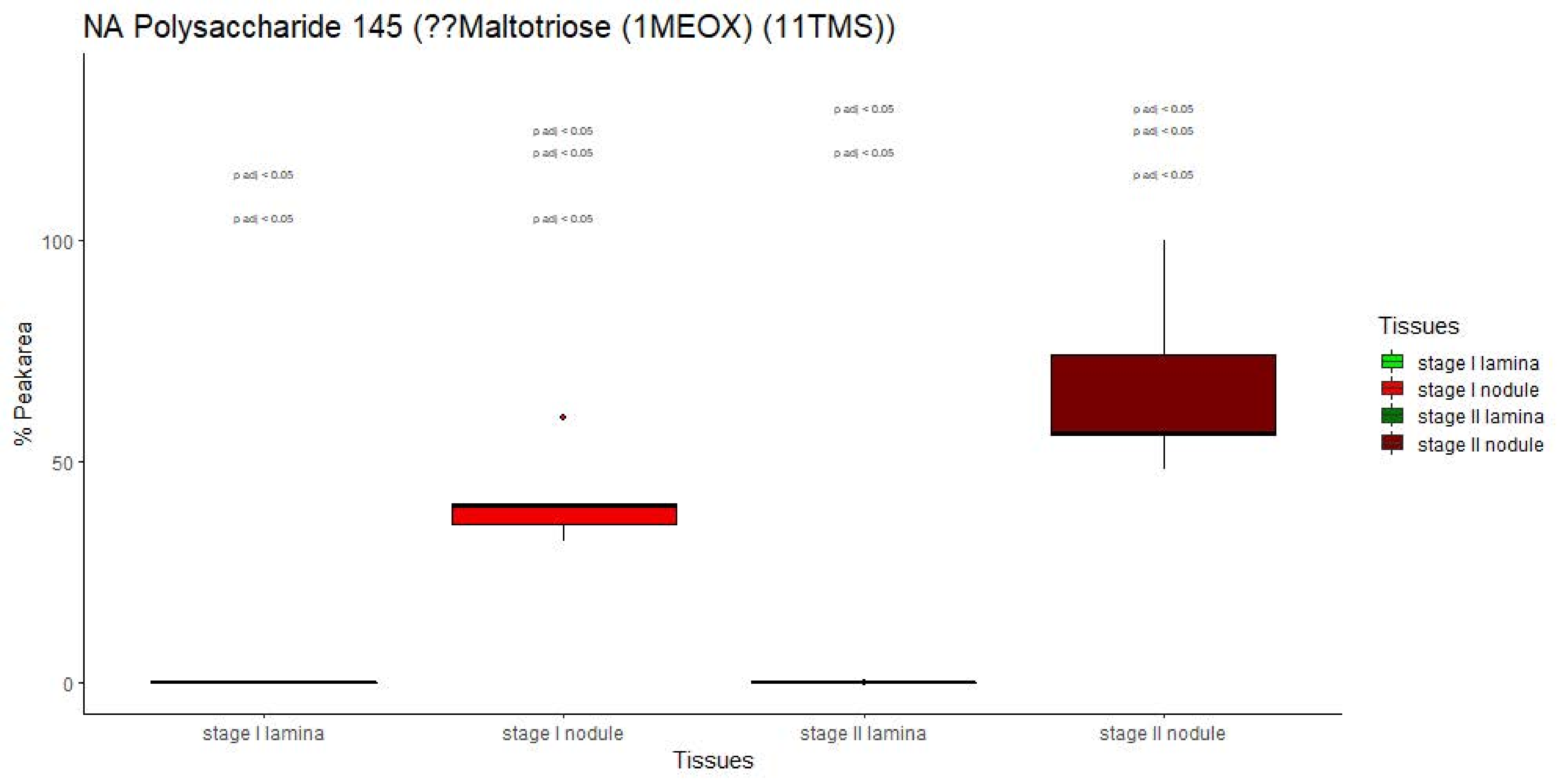

NA Polysaccharide 145 (??Maltotriose (1MEOX) (11TMS))
p adj < 0.05
p adj < 0.05
p adj < 0.05
p adj < 0.05
p adj < 0.05
p adj < 0.05
p adj < 0.05
p adj < 0.05
p adj < 0.05
p adj < 0.05
p adj < 0.05
% Peakarea
100
50
0
stage I lamina
stage I nodule
stage II lamina
stage II nodule
Tissues
Tissues
stage I lamina
stage I nodule
stage II lamina
stage II nodule

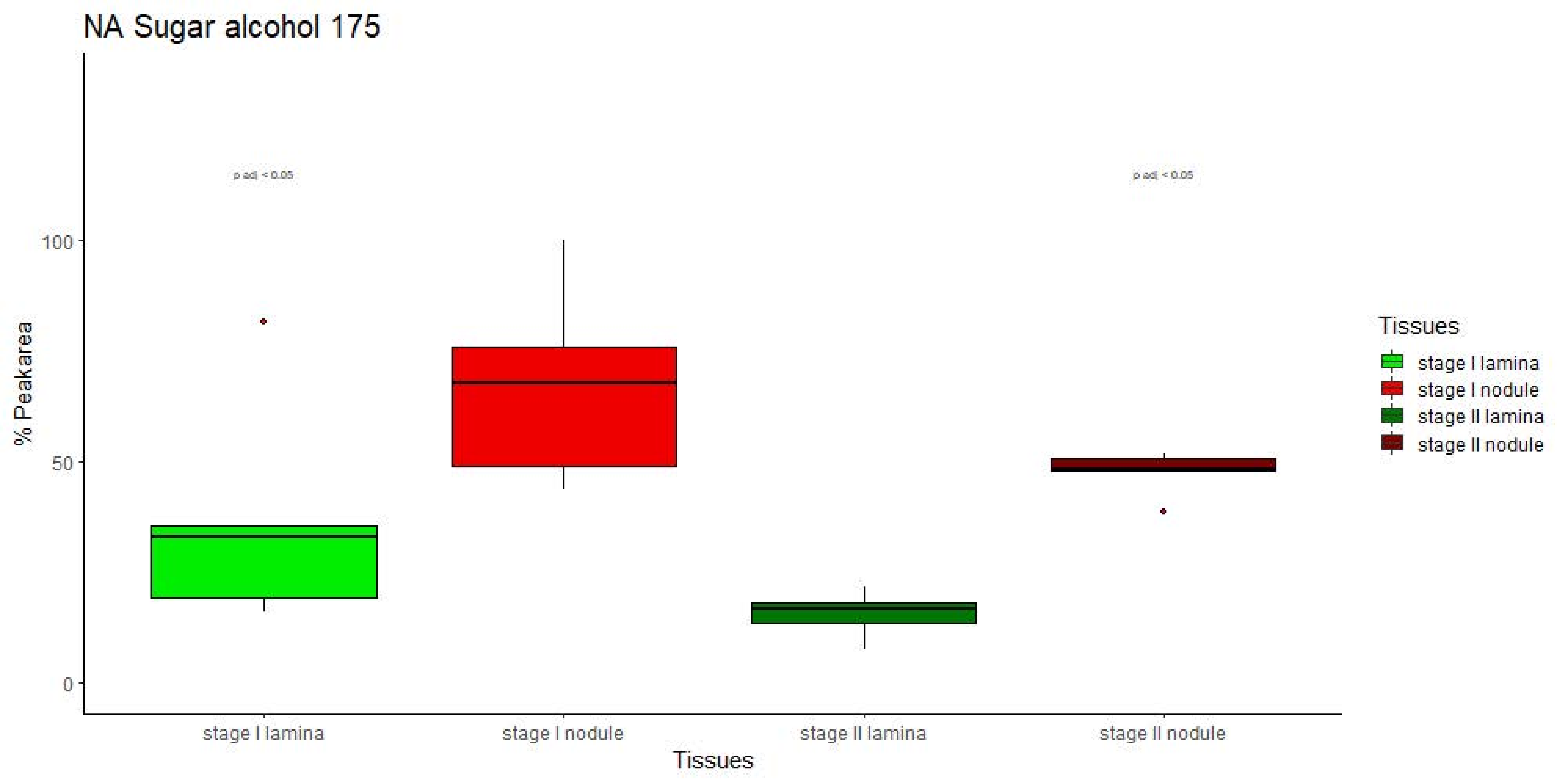

NA Sugar alcohol 175
p adj < 0.05
p adj < 0.05
100
50
0
% Peakarea
stage I lamina
stage I nodule
stage II lamina
stage II nodule
Tissues
Tissues
stage I lamina
stage I nodule
stage II lamina
stage II nodule

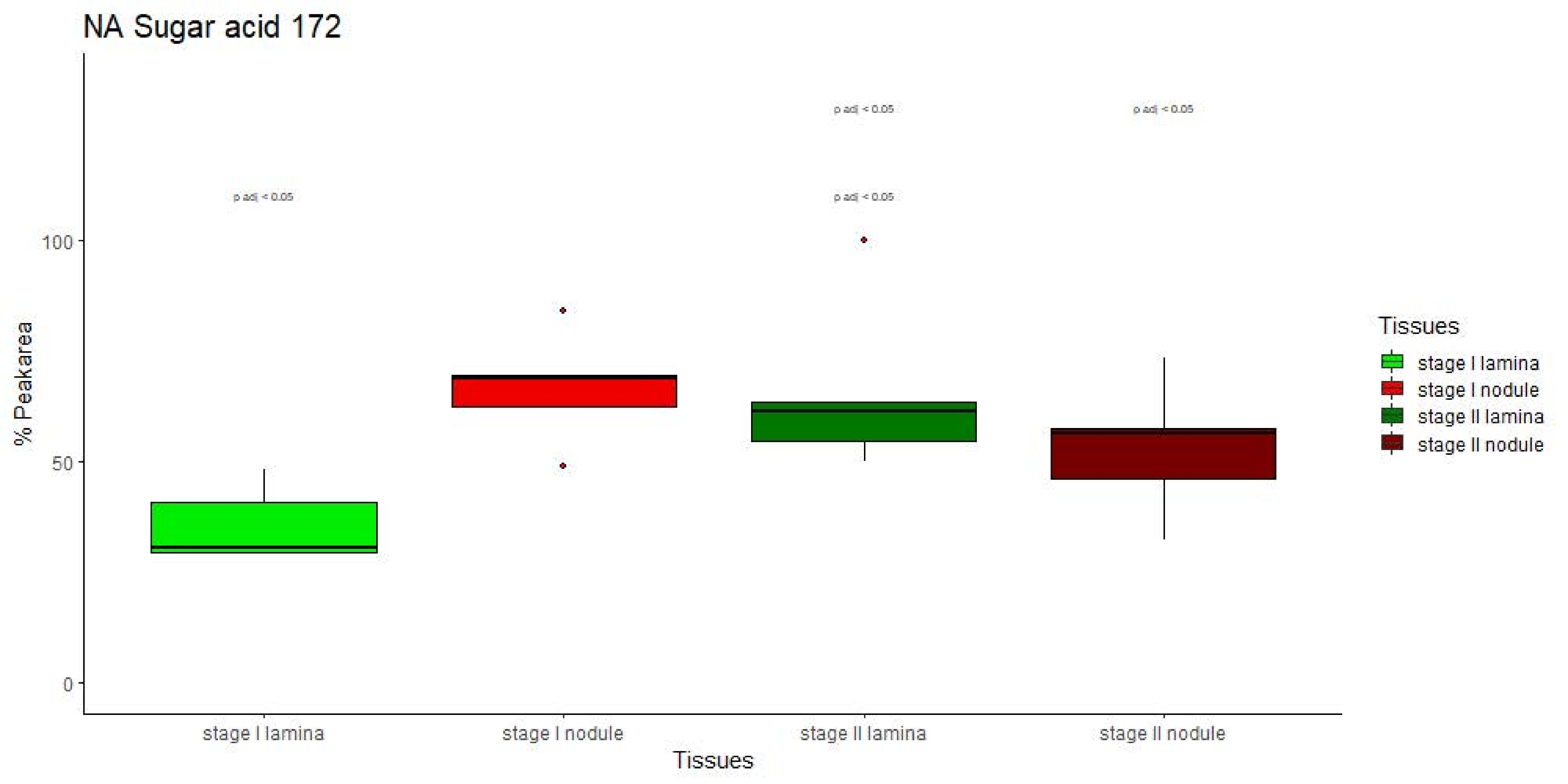
NA Sugar acid 172
p adj < 0.05
p adj < 0.05
p adj < 0.05
p adj < 0.05
100
50
0
% Peakarea
stage I lamina
stage I nodule
stage II lamina
stage II nodule
Tissues
Tissues
stage I lamina
stage I nodule
stage II lamina
stage II nodule

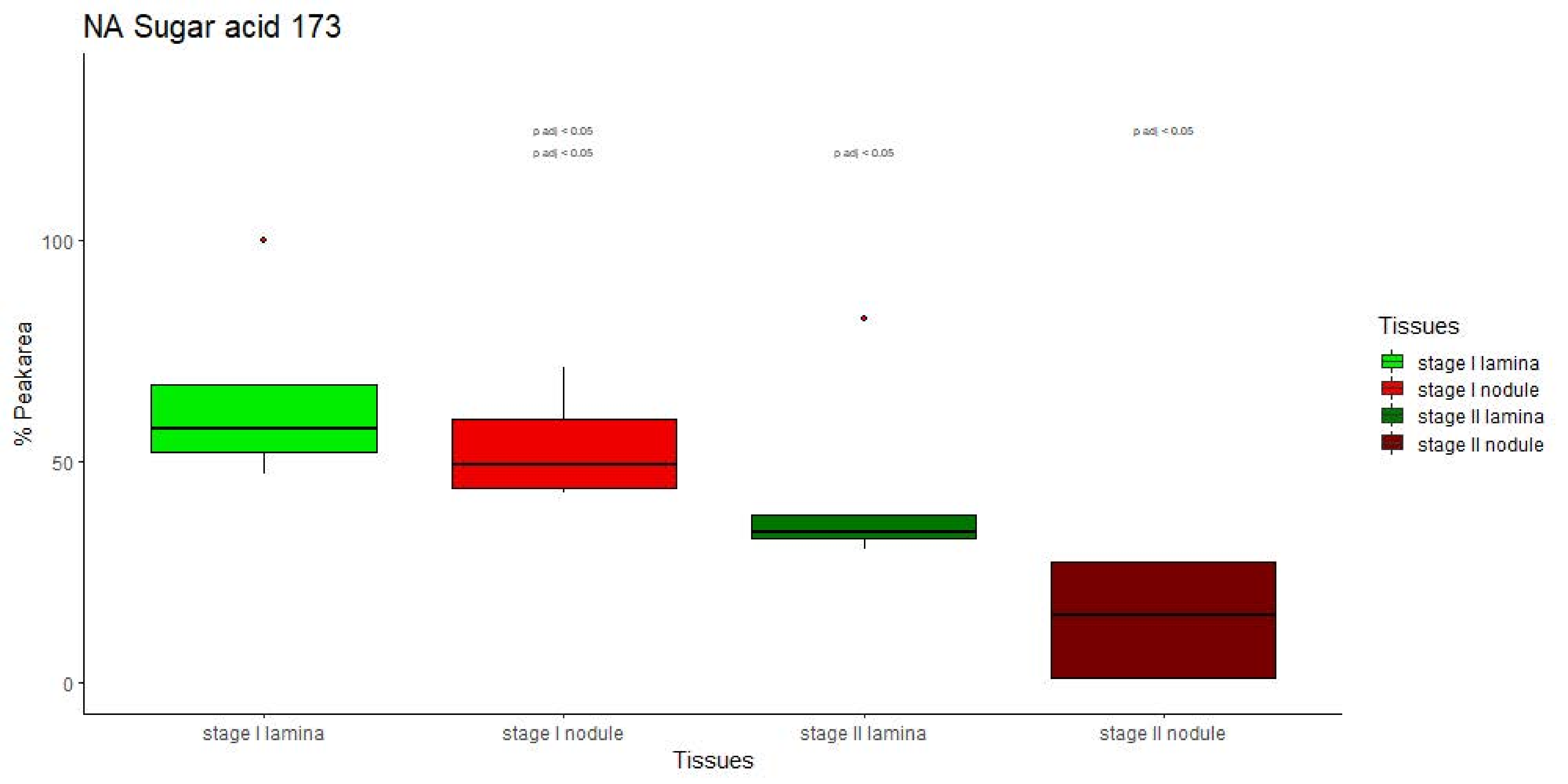

NA Sugar acid 173
p adj < 0.05
p adj < 0.05
p adj < 0.05
p adj < 0.05
100
50
0
% Peakarea
stage I lamina
stage I nodule
stage II lamina
stage II nodule
Tissues
Tissues
stage I lamina
stage I nodule
stage II lamina
stage II nodule

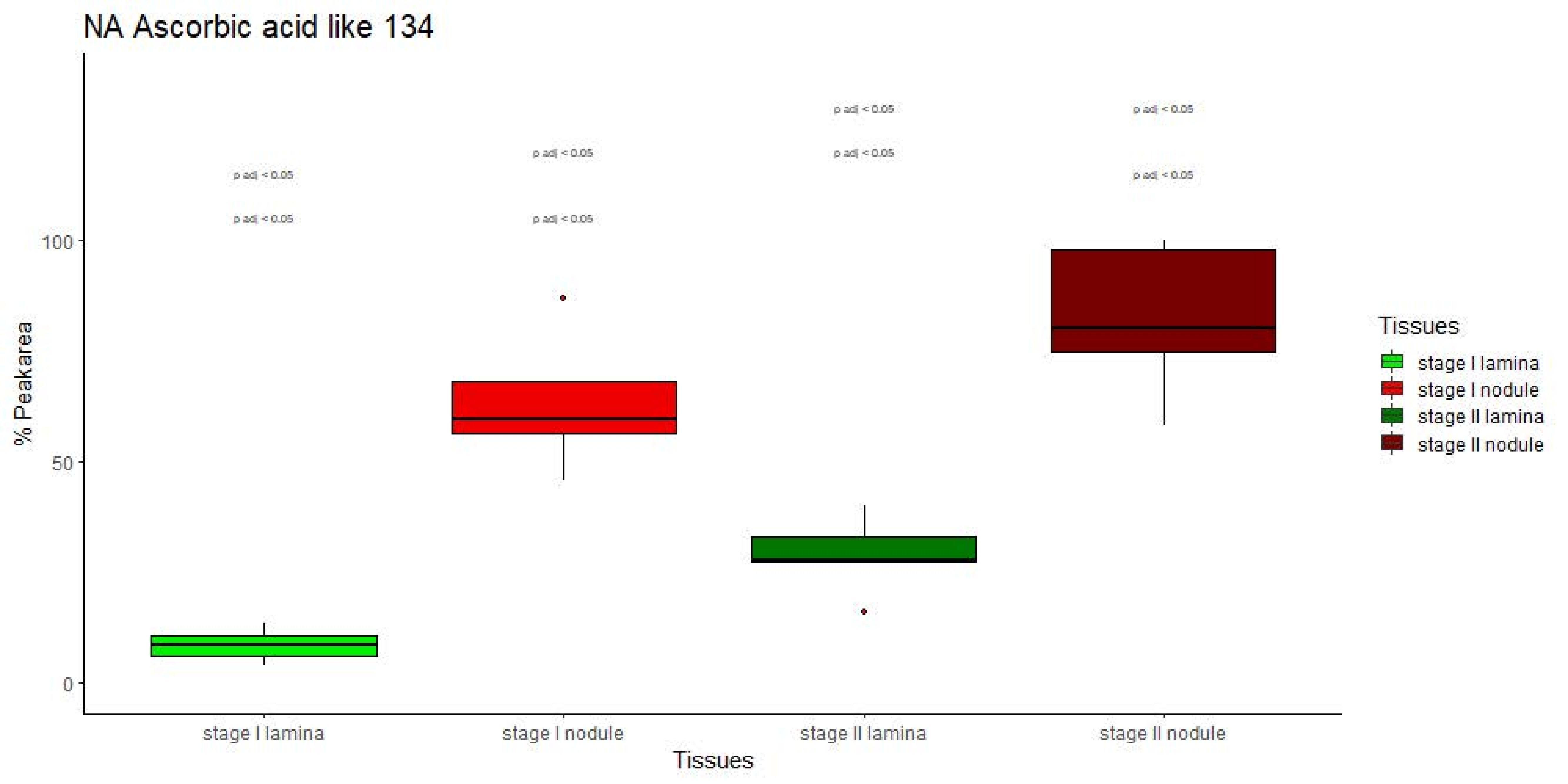

NA Ascorbic acid like 134
p adj < 0.05
p adj < 0.05
p adj < 0.05
p adj < 0.05
p adj < 0.05
p adj < 0.05
p adj < 0.05
p adj < 0.05
100
50
0
% Peakarea
stage I lamina
stage I nodule
stage II lamina
stage II nodule
Tissues
Tissues
stage I lamina
stage I nodule
stage II lamina
stage II nodule

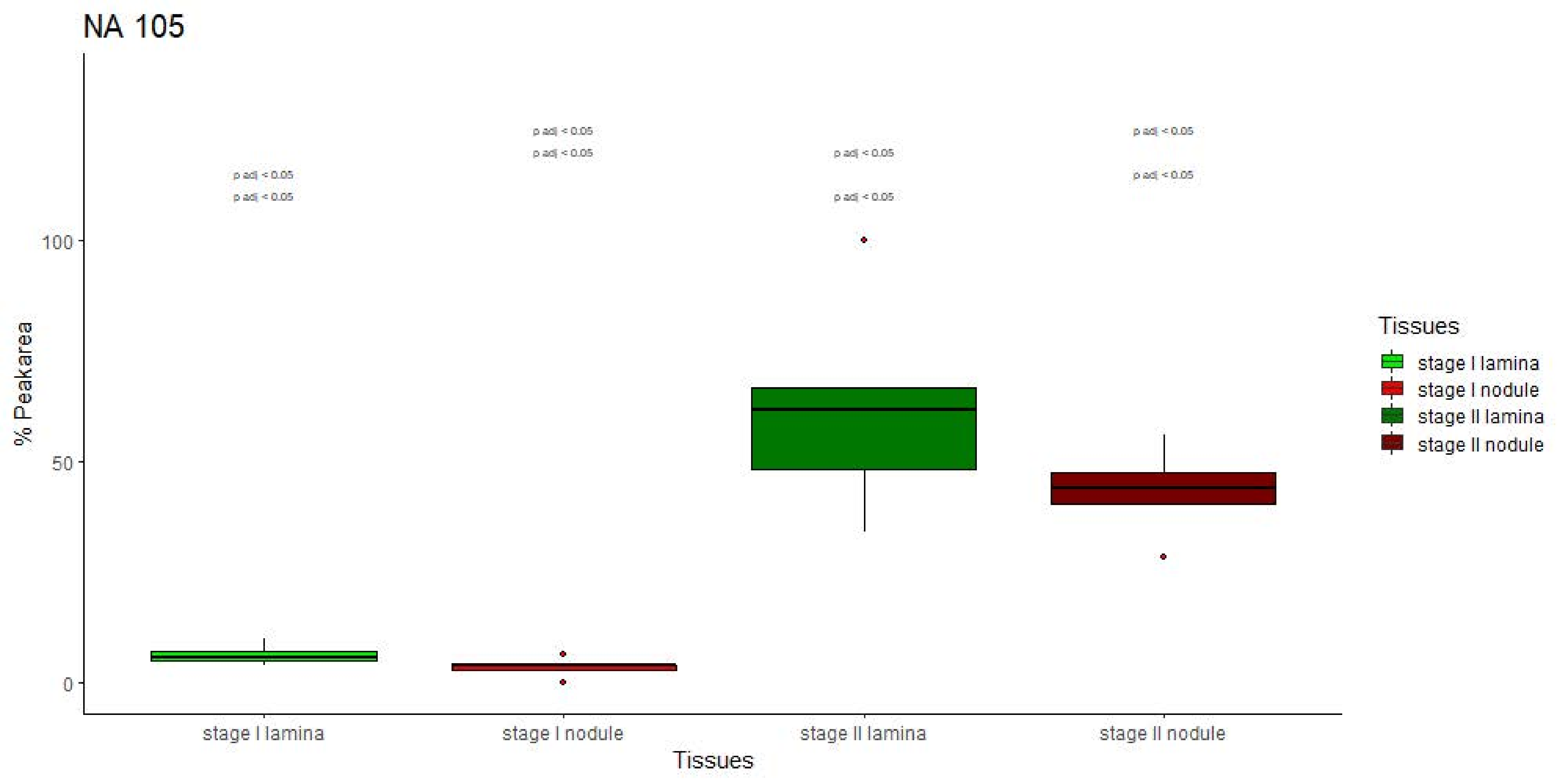

NA 105
p adj < 0.05
p adj < 0.05
p adj < 0.05
p adj < 0.05
p adj < 0.05
p adj < 0.05
p adj < 0.05
p adj < 0.05
100
50
0
% Peakarea
stage I lamina
stage I nodule
stage II lamina
stage II nodule
Tissues
Tissues
stage I lamina
stage I nodule
stage II lamina
stage II nodule

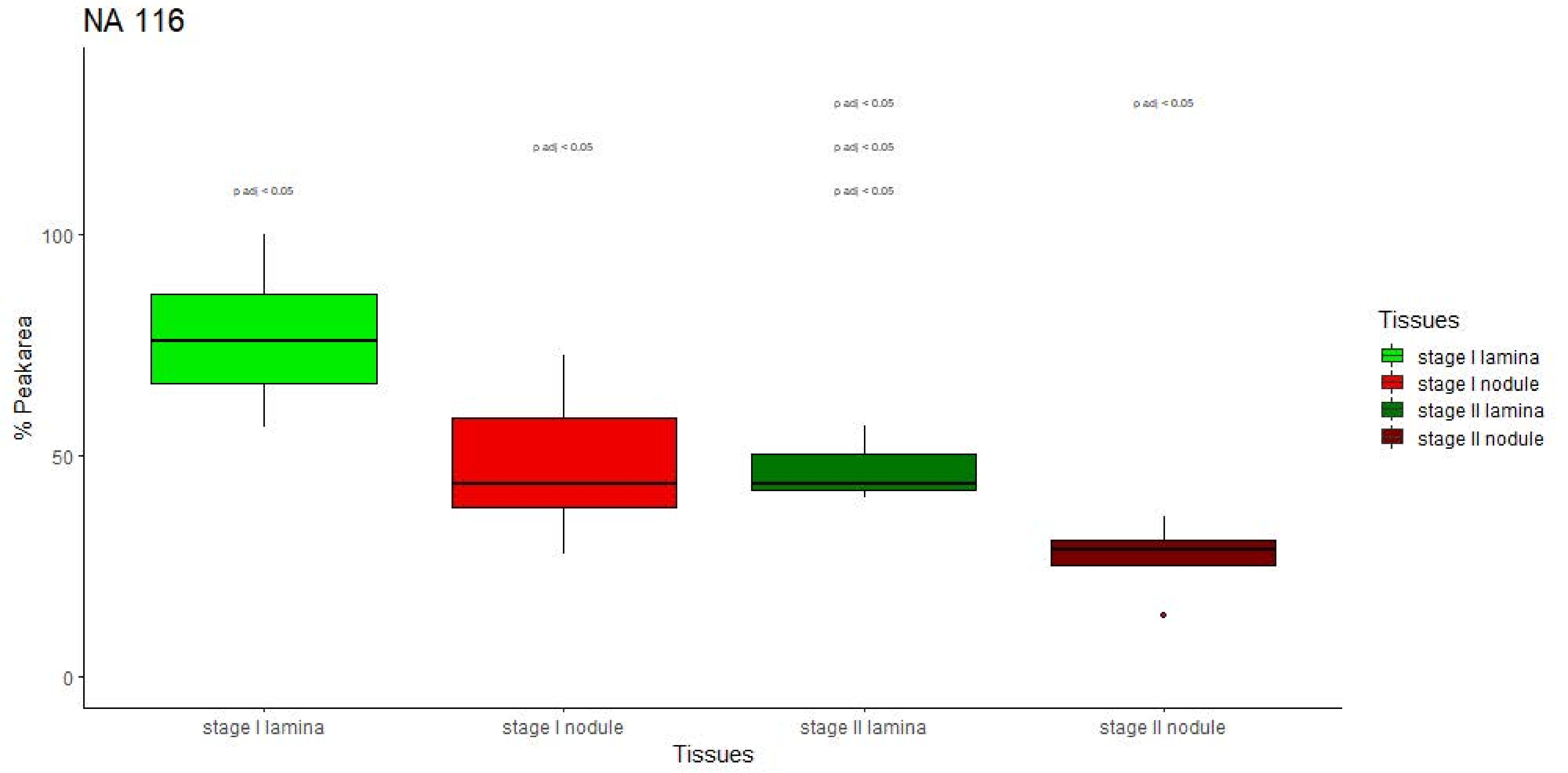
NA 116
p adj < 0.05
p adj < 0.05
p adj < 0.05
p adj < 0.05
p adj < 0.05
p adj < 0.05
% Peakarea
100
50
0
stage I lamina
stage I nodule
stage II lamina
stage II nodule
Tissues
Tissues
stage I lamina
stage I nodule
stage II lamina
stage II nodule

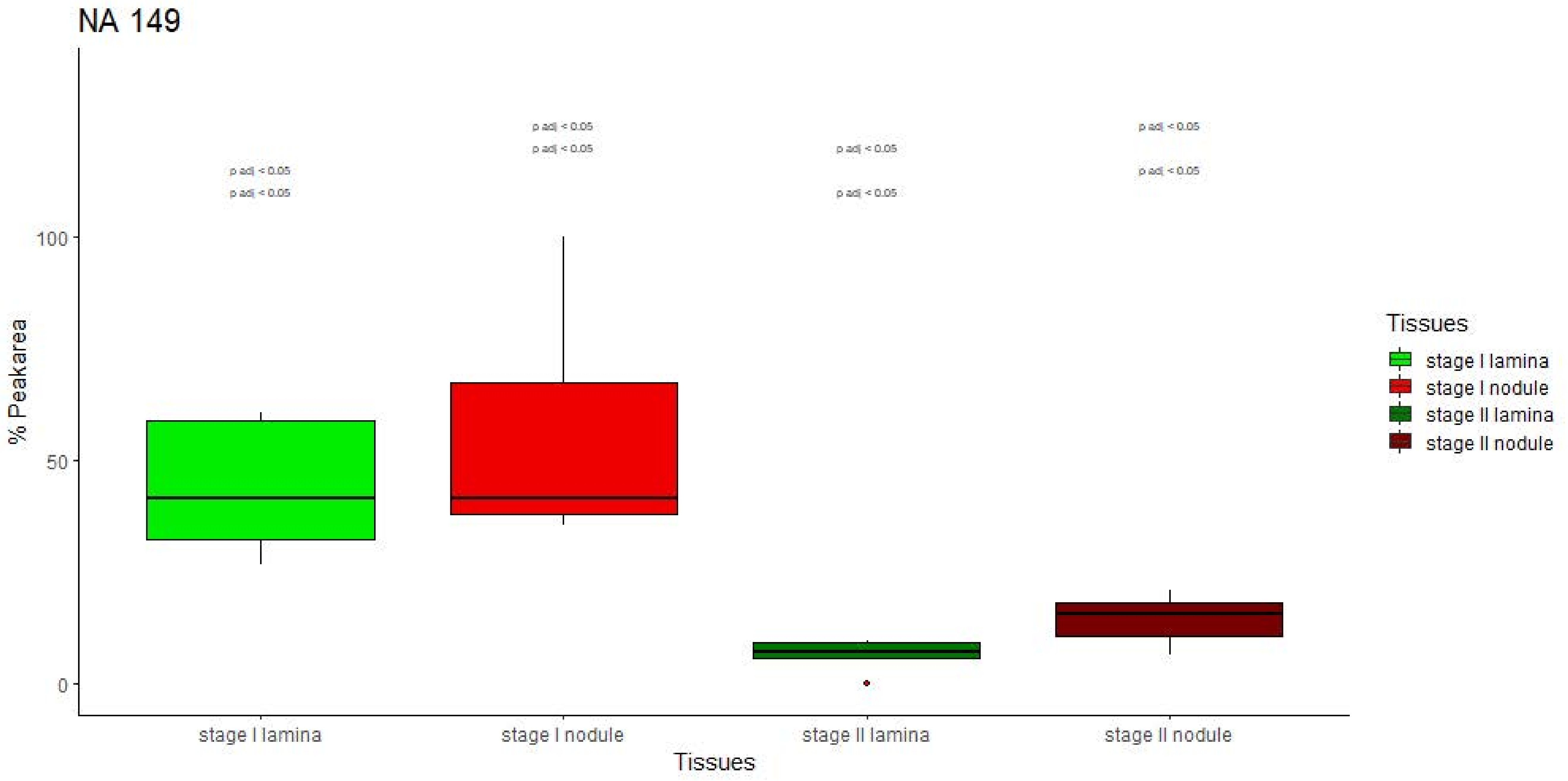

NA 149
p adj < 0.05
p adj < 0.05
p adj < 0.05
p adj < 0.05
p adj < 0.05
p adj < 0.05
p adj < 0.05
p adj < 0.05
100
50
0
% Peakarea
stage I lamina
stage I nodule
stage II lamina
stage II nodule
Tissues
Tissues
stage I lamina
stage I nodule
stage II lamina
stage II nodule

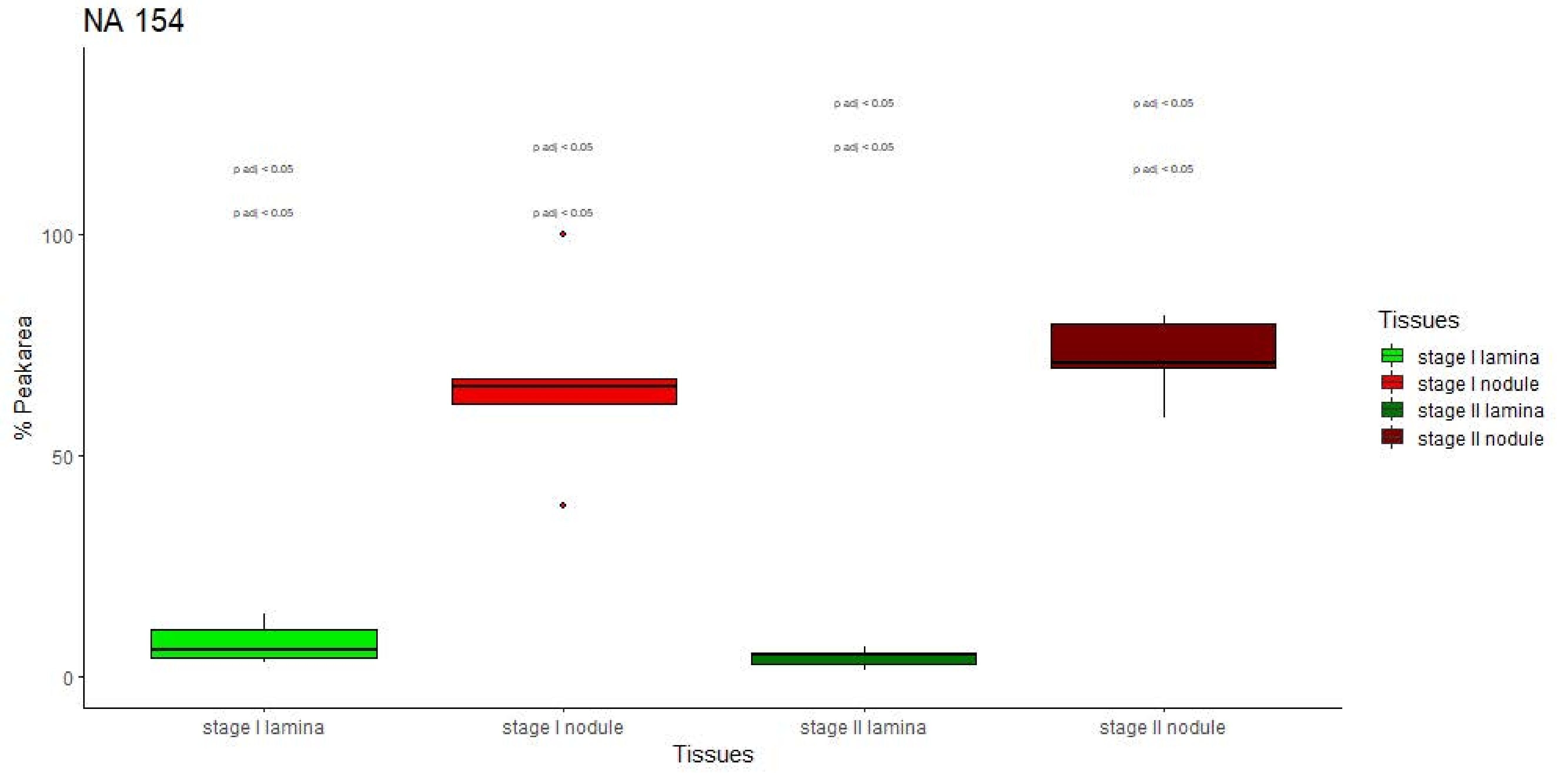

NA 154
p adj < 0.05
p adj < 0.05
p adj < 0.05
p adj < 0.05
p adj < 0.05
p adj < 0.05
p adj < 0.05
p adj < 0.05
100
50
0
% Peakarea
stage I lamina
stage I nodule
stage II lamina
stage II nodule
Tissues
Tissues
stage I lamina
stage I nodule
stage II lamina
stage II nodule

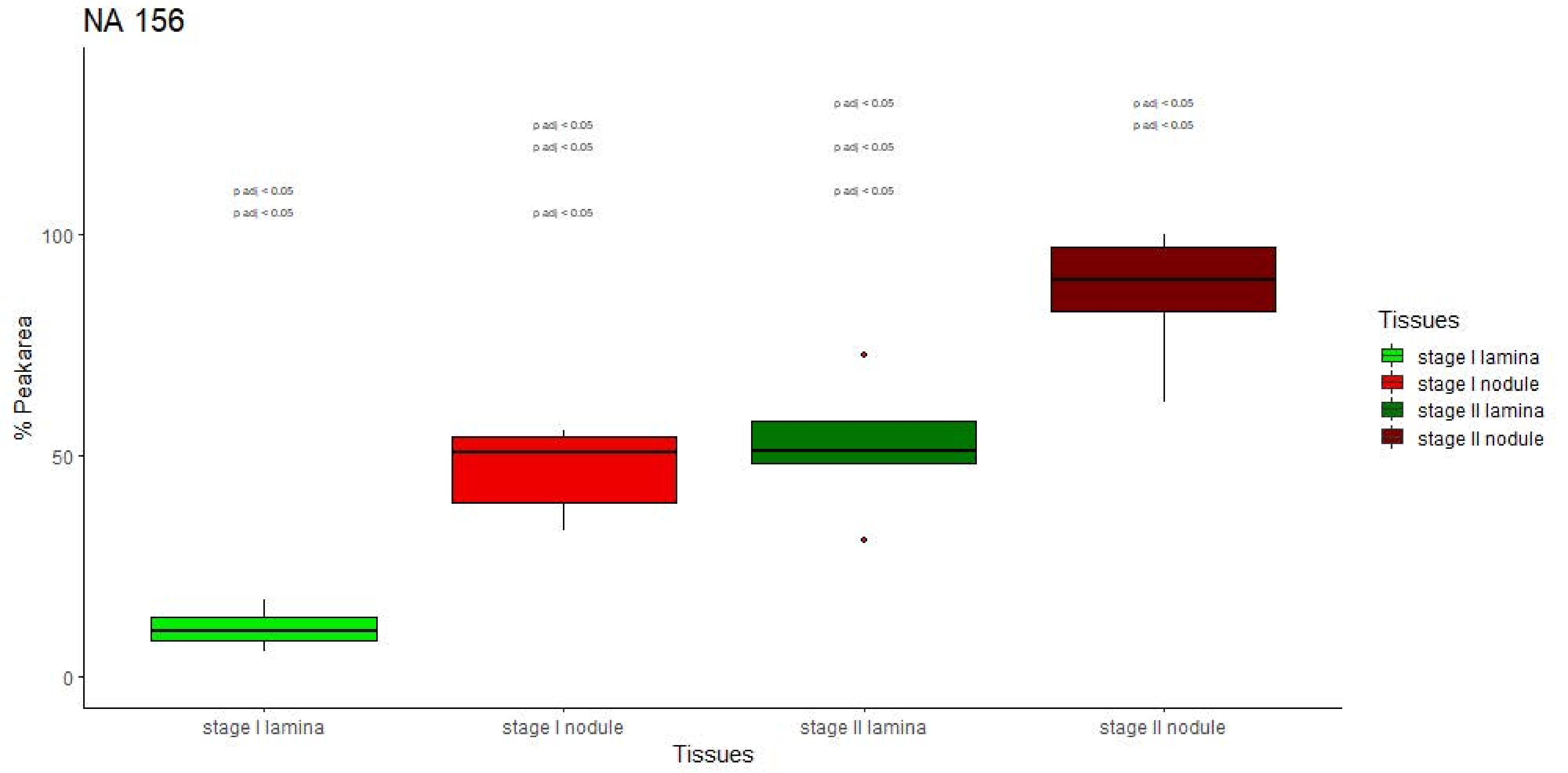
NA 156
% Peakarea
Tissues
p adj < 0.05
0
50
100
stage I lamina
stage I nodule
stage II lamina
stage II nodule
Tissues
stage I lamina
stage I nodule
stage II lamina
stage II nodule

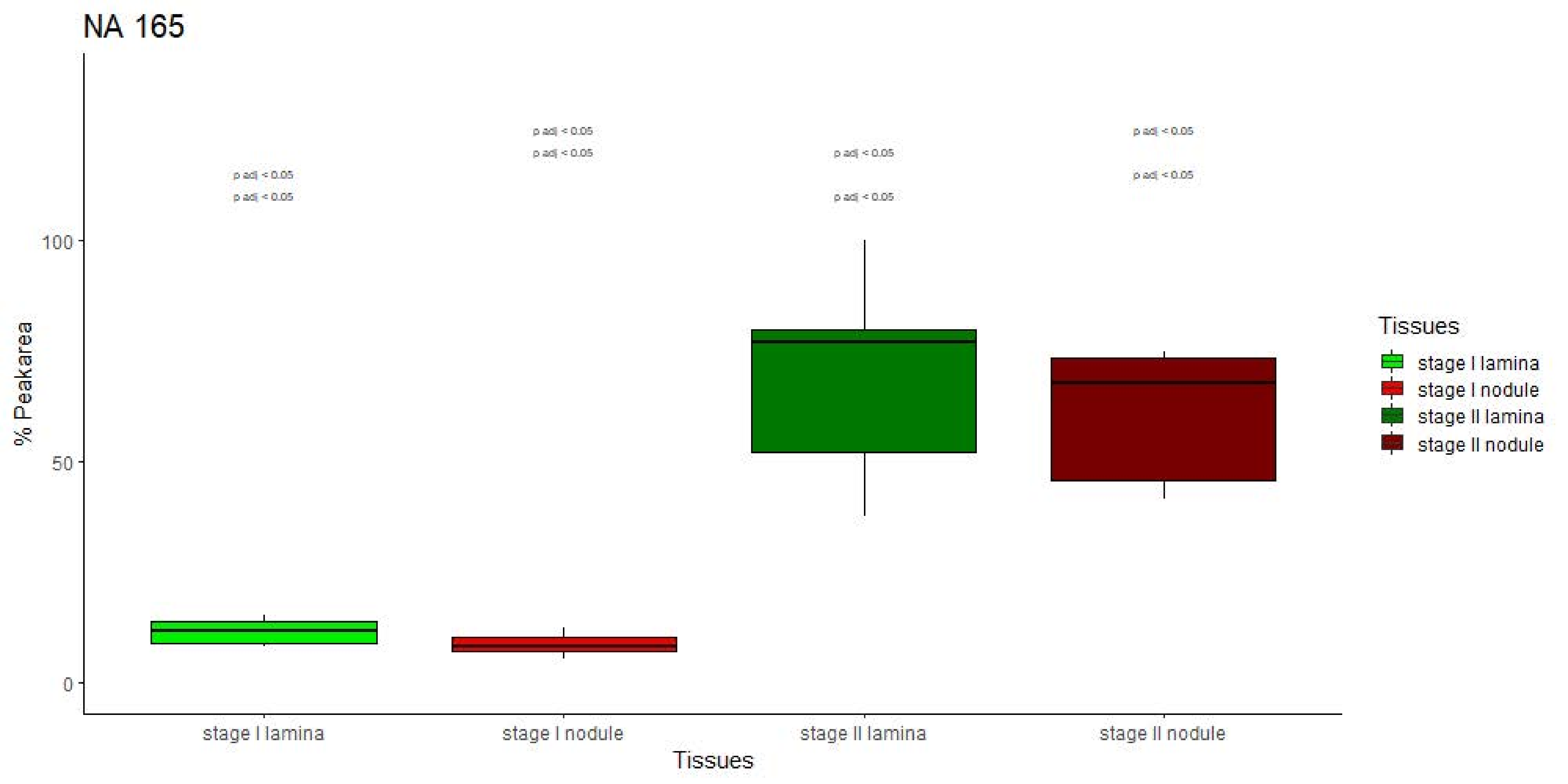
NA 165
% Peakarea
0
50
100
p adj < 0.05
p adj < 0.05
p adj < 0.05
p adj < 0.05
p adj < 0.05
p adj < 0.05
p adj < 0.05
p adj < 0.05
stage I lamina
stage I nodule
stage II lamina
stage II nodule
Tissues
Tissues
stage I lamina
stage I nodule
stage II lamina
stage II nodule

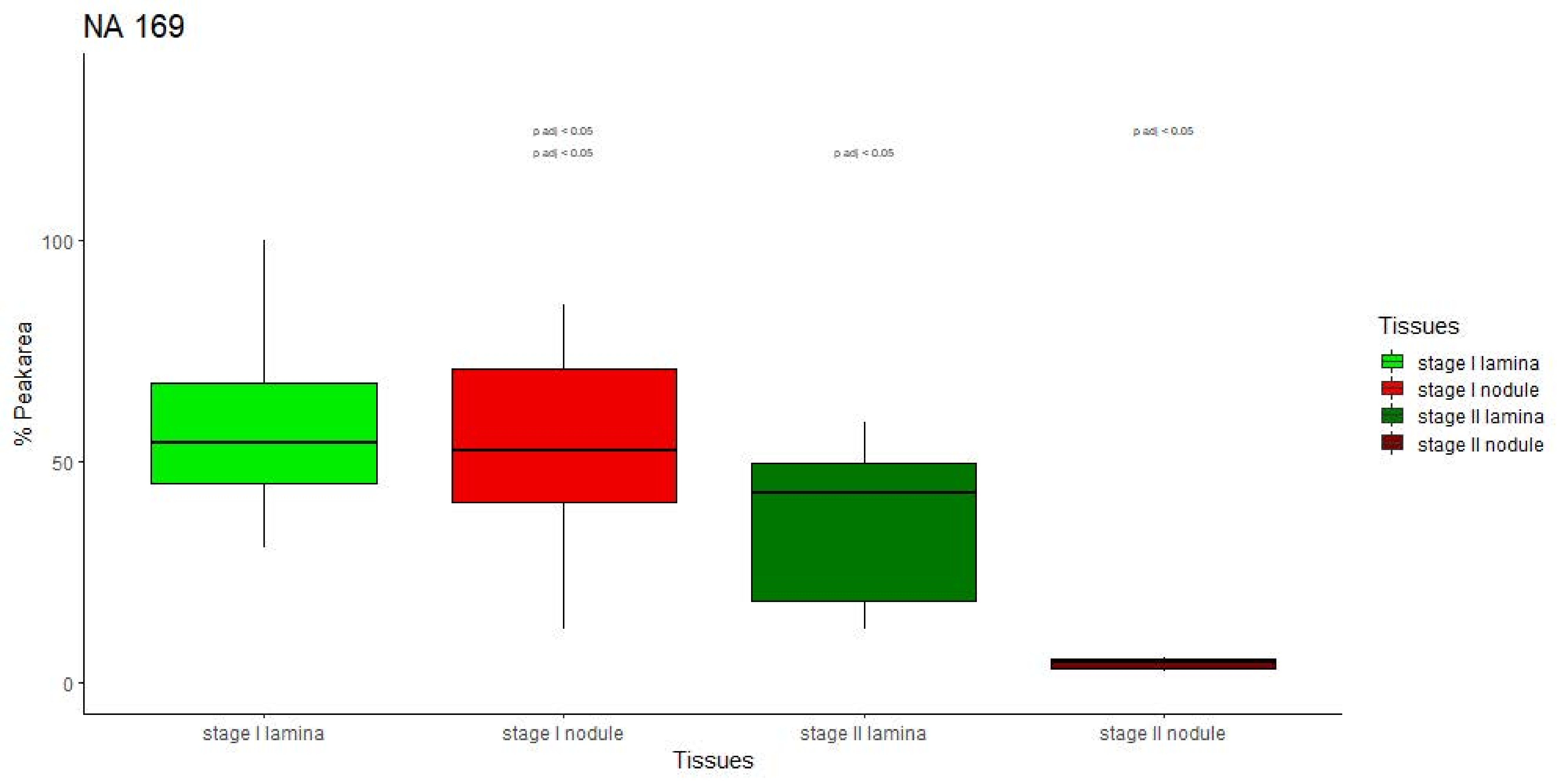
NA 169
p adj < 0.05
p adj < 0.05
p adj < 0.05
p adj < 0.05
100
50
0
% Peakarea
stage I lamina
stage I nodule
stage II lamina
stage II nodule
Tissues
Tissues
stage I lamina
stage I nodule
stage II lamina
stage II nodule

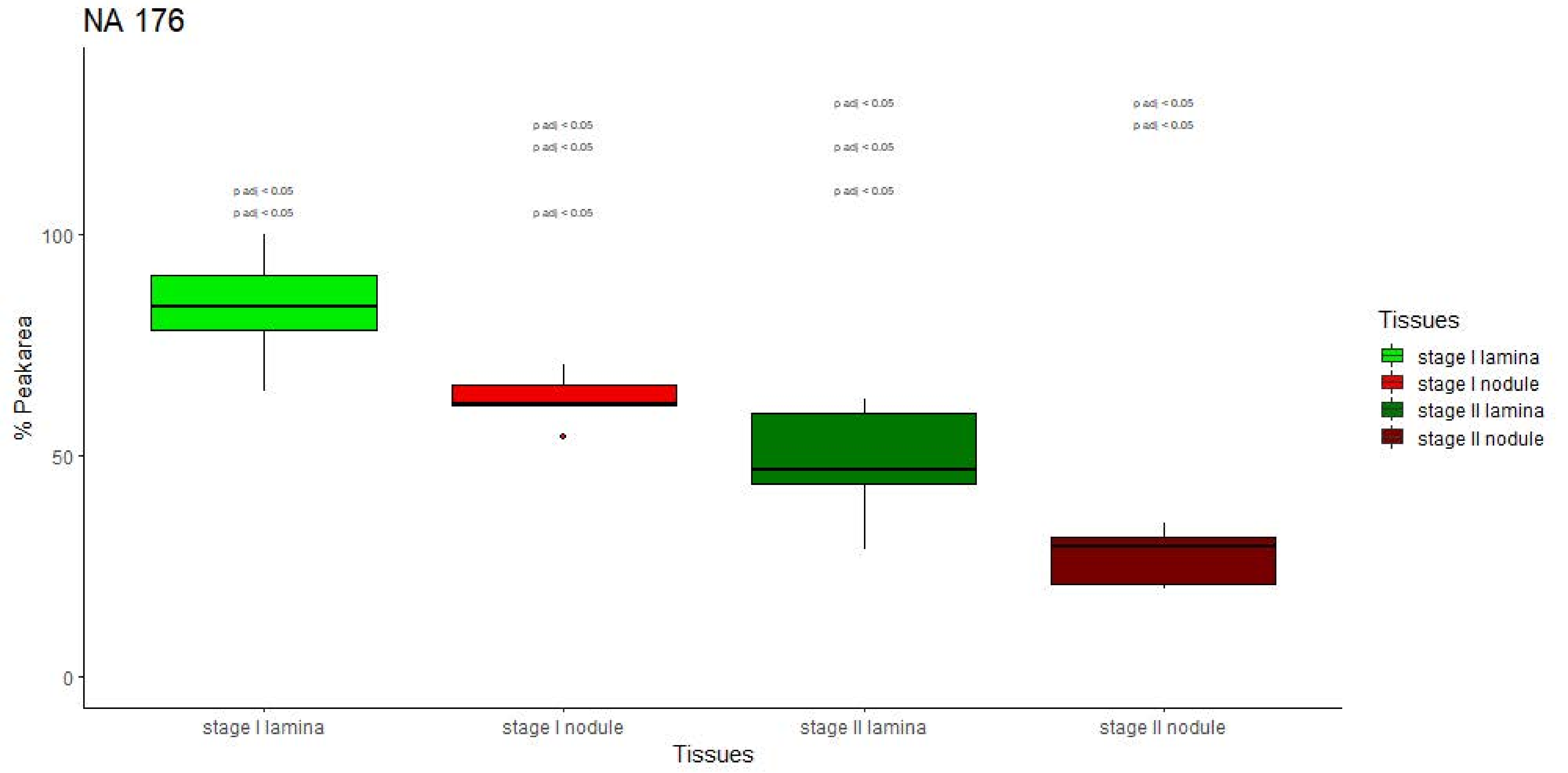
NA 176
p adj < 0.05
p adj < 0.05
p adj < 0.05
p adj < 0.05
p adj < 0.05
p adj < 0.05
p adj < 0.05
p adj < 0.05
p adj < 0.05
p adj < 0.05
100
50
0
% Peakarea
stage I lamina
stage I nodule
stage II lamina
stage II nodule
Tissues
Tissues
stage I lamina
stage I nodule
stage II lamina
stage II nodule

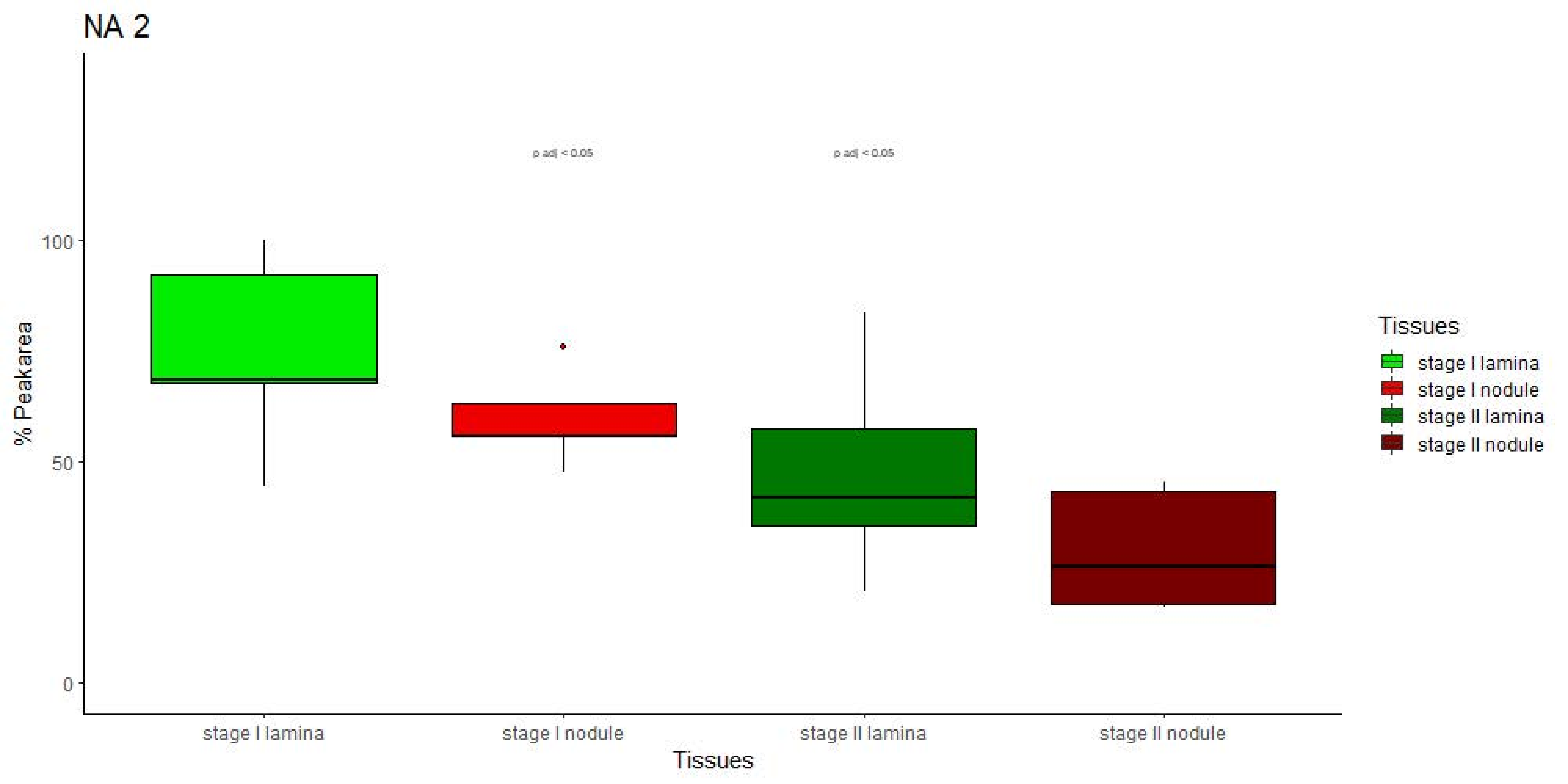

NA 2
p adj < 0.05
p adj < 0.05
100
50
0
% Peakarea
stage I lamina
stage I nodule
stage II lamina
stage II nodule
Tissues
Tissues
stage I lamina
stage I nodule
stage II lamina
stage II nodule

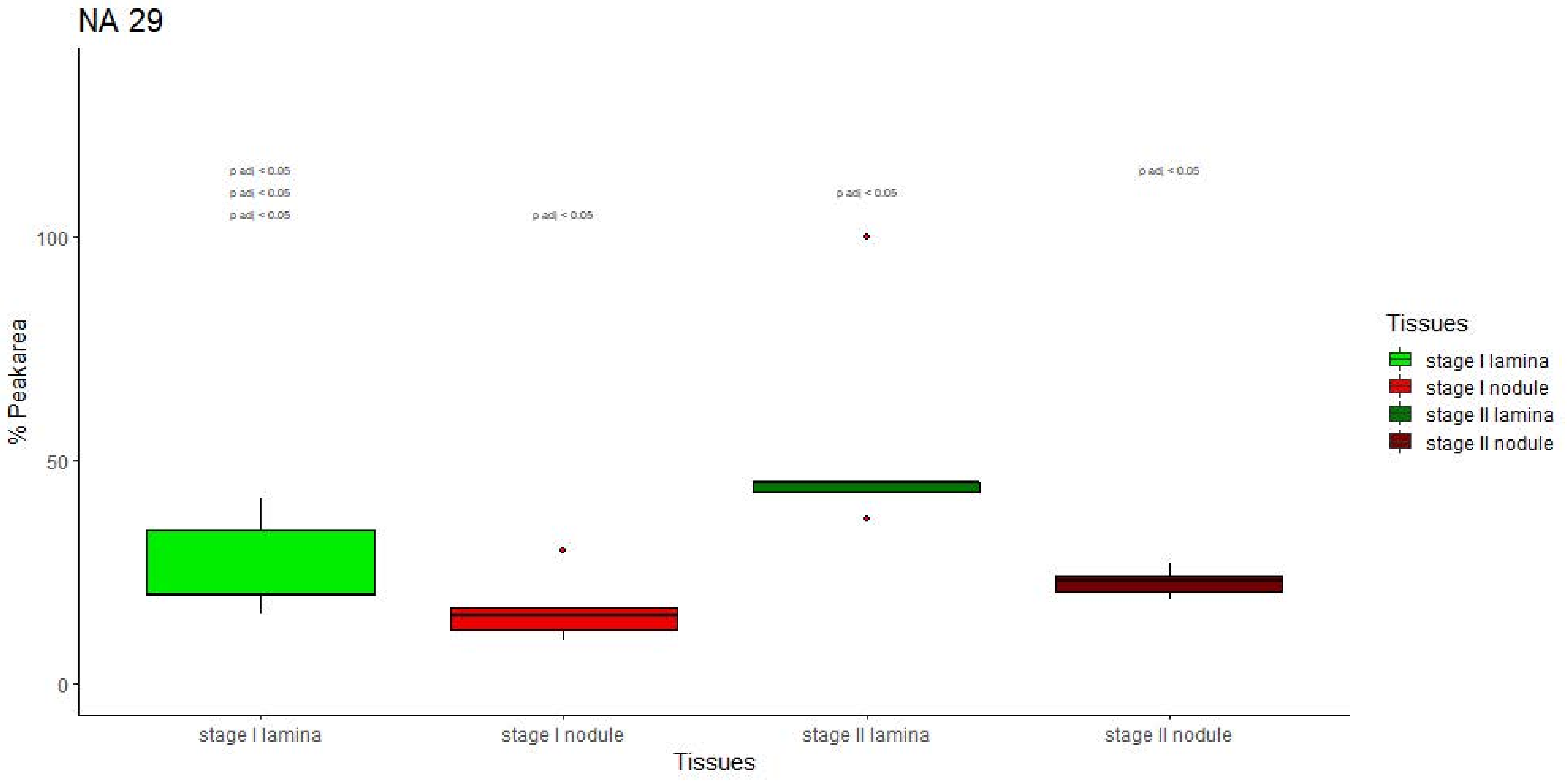
NA 29
% Peakarea
100
50
0
p adj < 0.05
p adj < 0.05
p adj < 0.05
p adj < 0.05
p adj < 0.05
p adj < 0.05
stage I lamina
stage I nodule
stage II lamina
stage II nodule
Tissues
Tissues
stage I lamina
stage I nodule
stage II lamina
stage II nodule

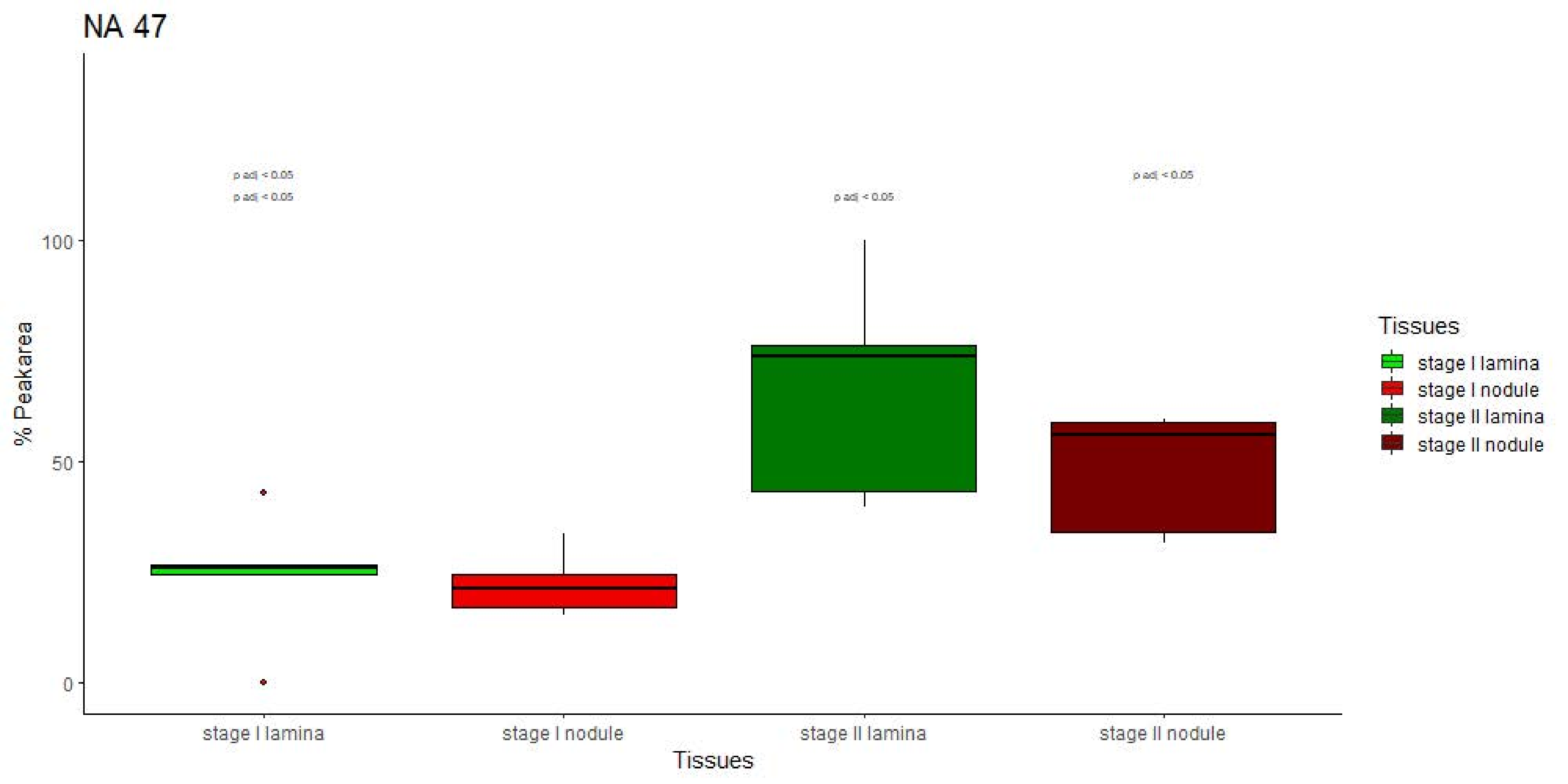

NA 47
p adj < 0.05
p adj < 0.05
p adj < 0.05
p adj < 0.05
100
50
0
% Peakarea
stage I lamina
stage I nodule
stage II lamina
stage II nodule
Tissues
Tissues
stage I lamina
stage I nodule
stage II lamina
stage II nodule

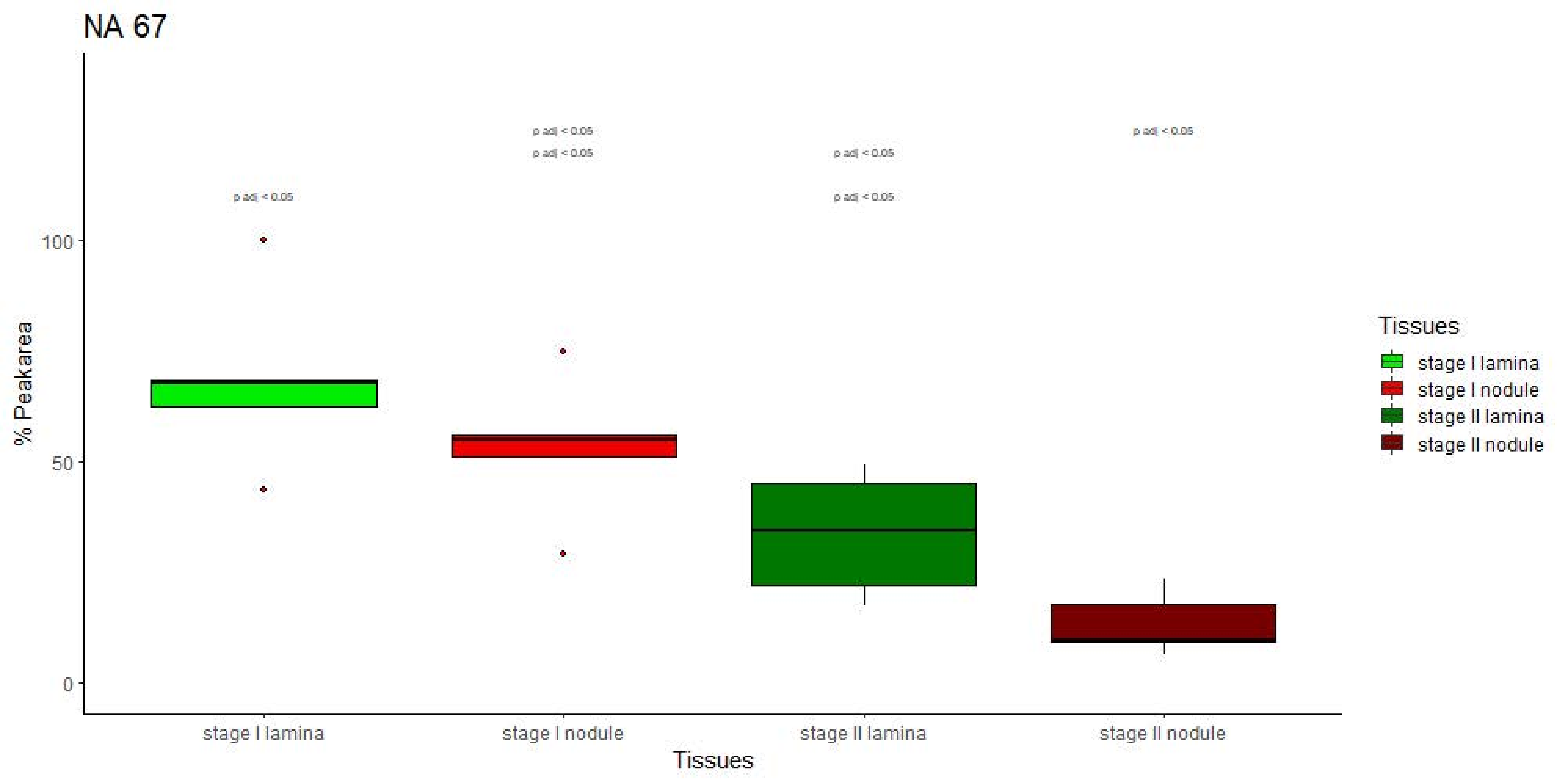
NA 67
p adj < 0.05
p adj < 0.05
p adj < 0.05
p adj < 0.05
p adj < 0.05
p adj < 0.05
100
50
0
% Peakarea
stage I lamina
stage I nodule
stage II lamina
stage II nodule
Tissues
Tissues
stage I lamina
stage I nodule
stage II lamina
stage II nodule

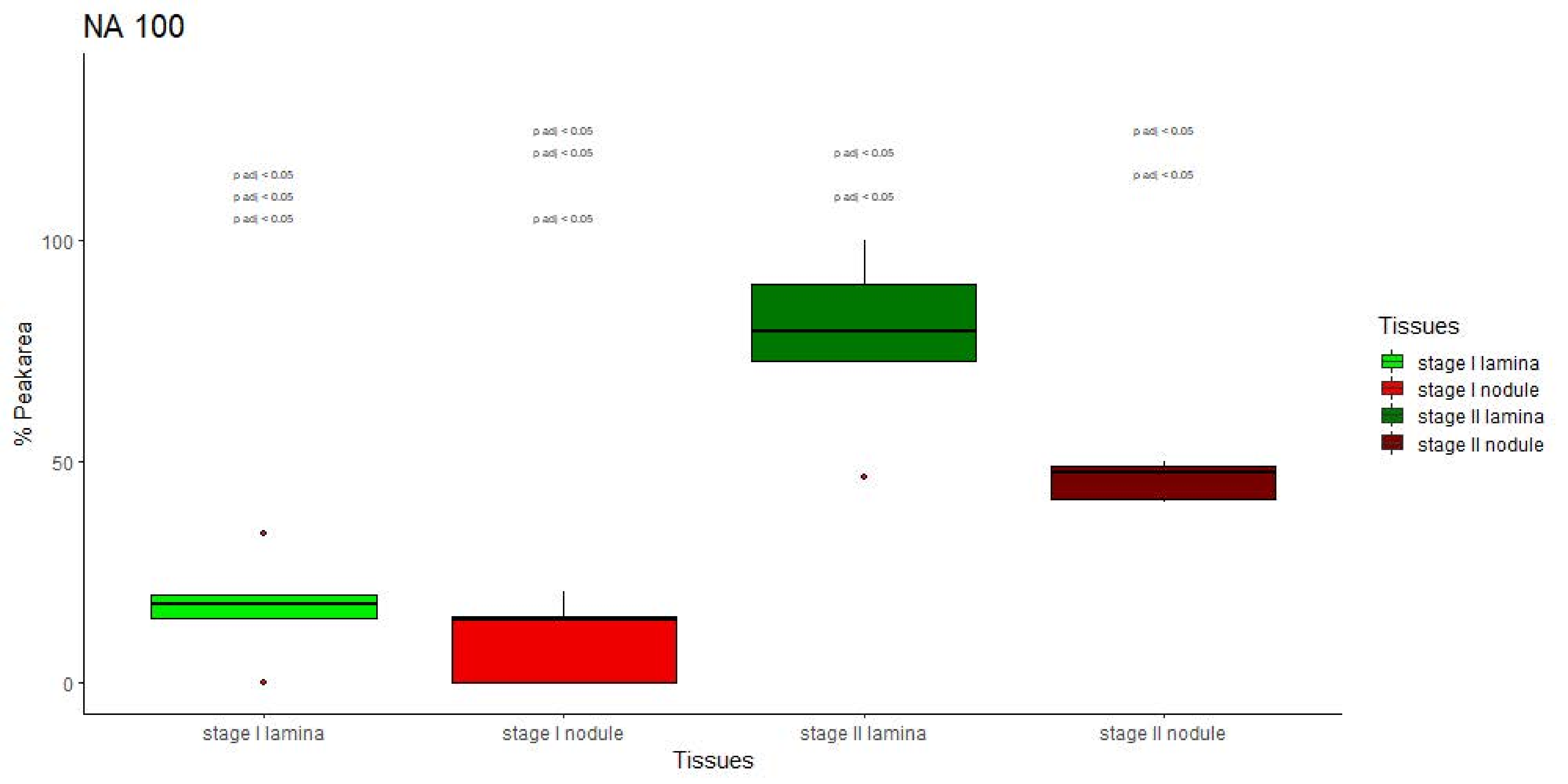
NA 100
p adj < 0.05
p adj < 0.05
p adj < 0.05
p adj < 0.05
p adj < 0.05
p adj < 0.05
p adj < 0.05
p adj < 0.05
p adj < 0.05
p adj < 0.05
100
50
0
% Peakarea
stage I lamina
stage I nodule
stage II lamina
stage II nodule
Tissues
Tissues
stage I lamina
stage I nodule
stage II lamina
stage II nodule

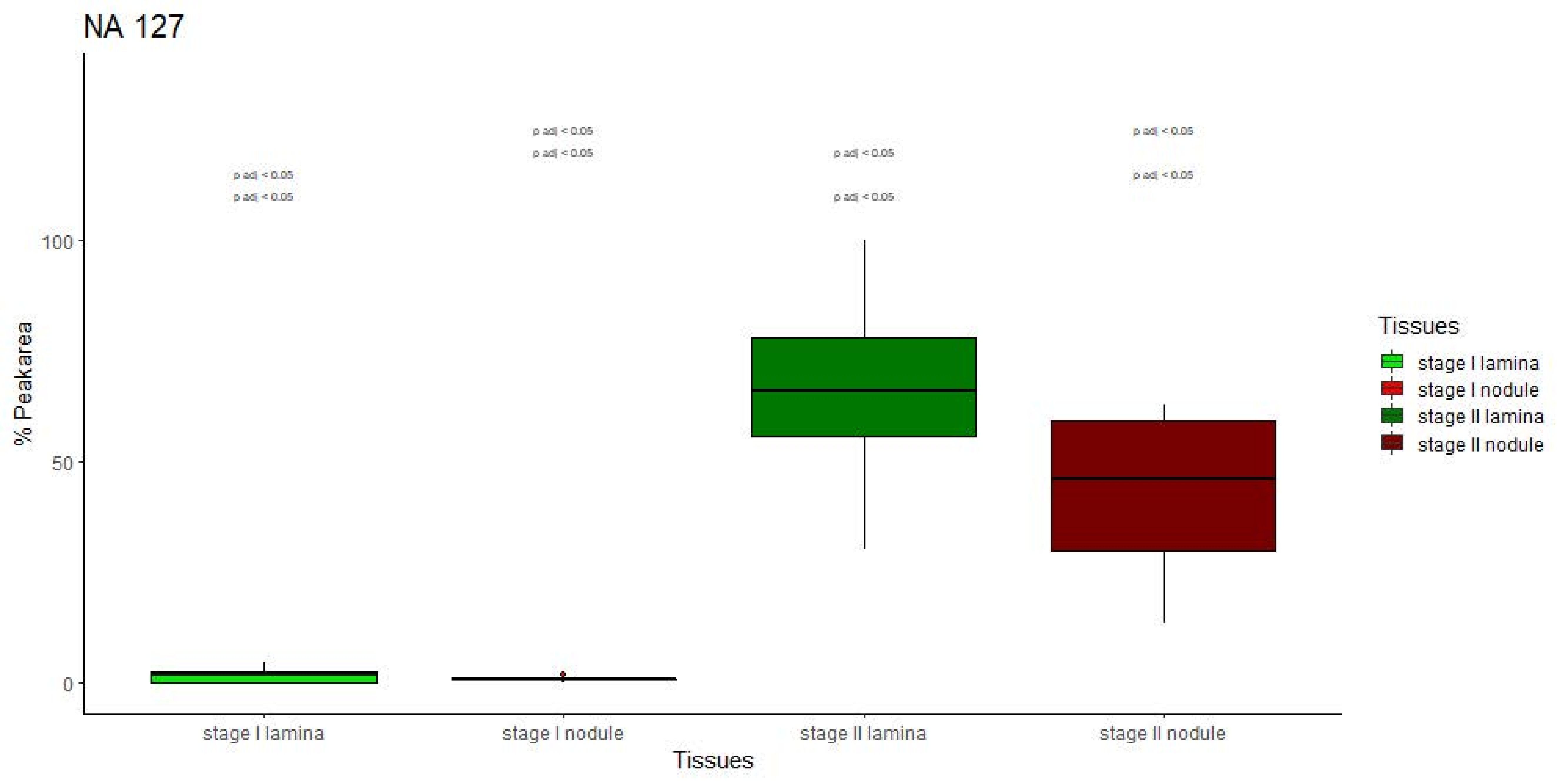
NA 127
p adj < 0.05
p adj < 0.05
p adj < 0.05
p adj < 0.05
p adj < 0.05
p adj < 0.05
p adj < 0.05
p adj < 0.05
% Peakarea
100
50
0
stage I lamina
stage I nodule
stage II lamina
stage II nodule
Tissues
Tissues
stage I lamina
stage I nodule
stage II lamina
stage II nodule

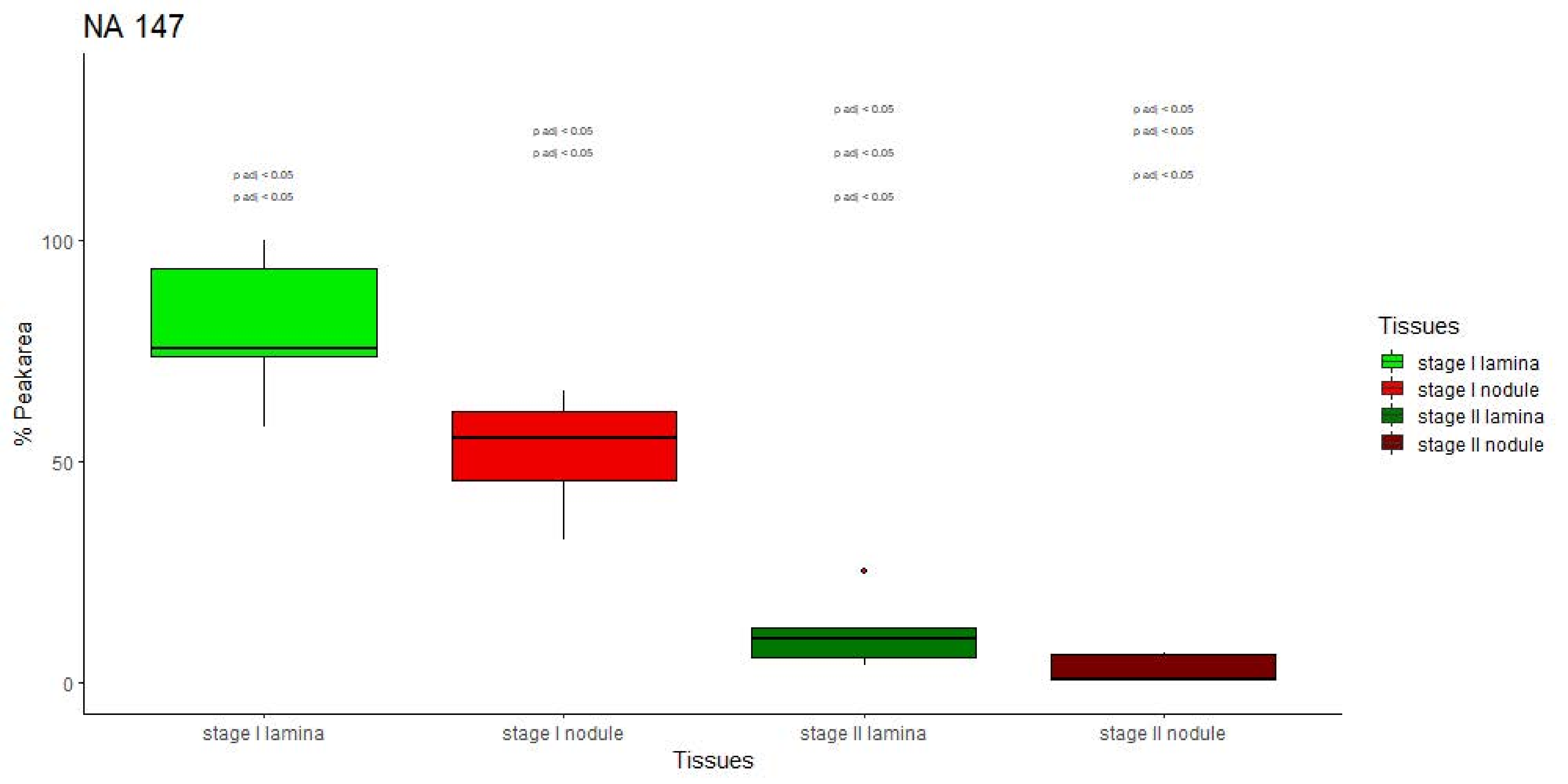

NA 147
p adj < 0.05
p adj < 0.05
p adj < 0.05
p adj < 0.05
p adj < 0.05
p adj < 0.05
p adj < 0.05
p adj < 0.05
p adj < 0.05
p adj < 0.05
% Peakarea
100
50
0
stage I lamina
stage I nodule
stage II lamina
stage II nodule
Tissues
Tissues
stage I lamina
stage I nodule
stage II lamina
stage II nodule

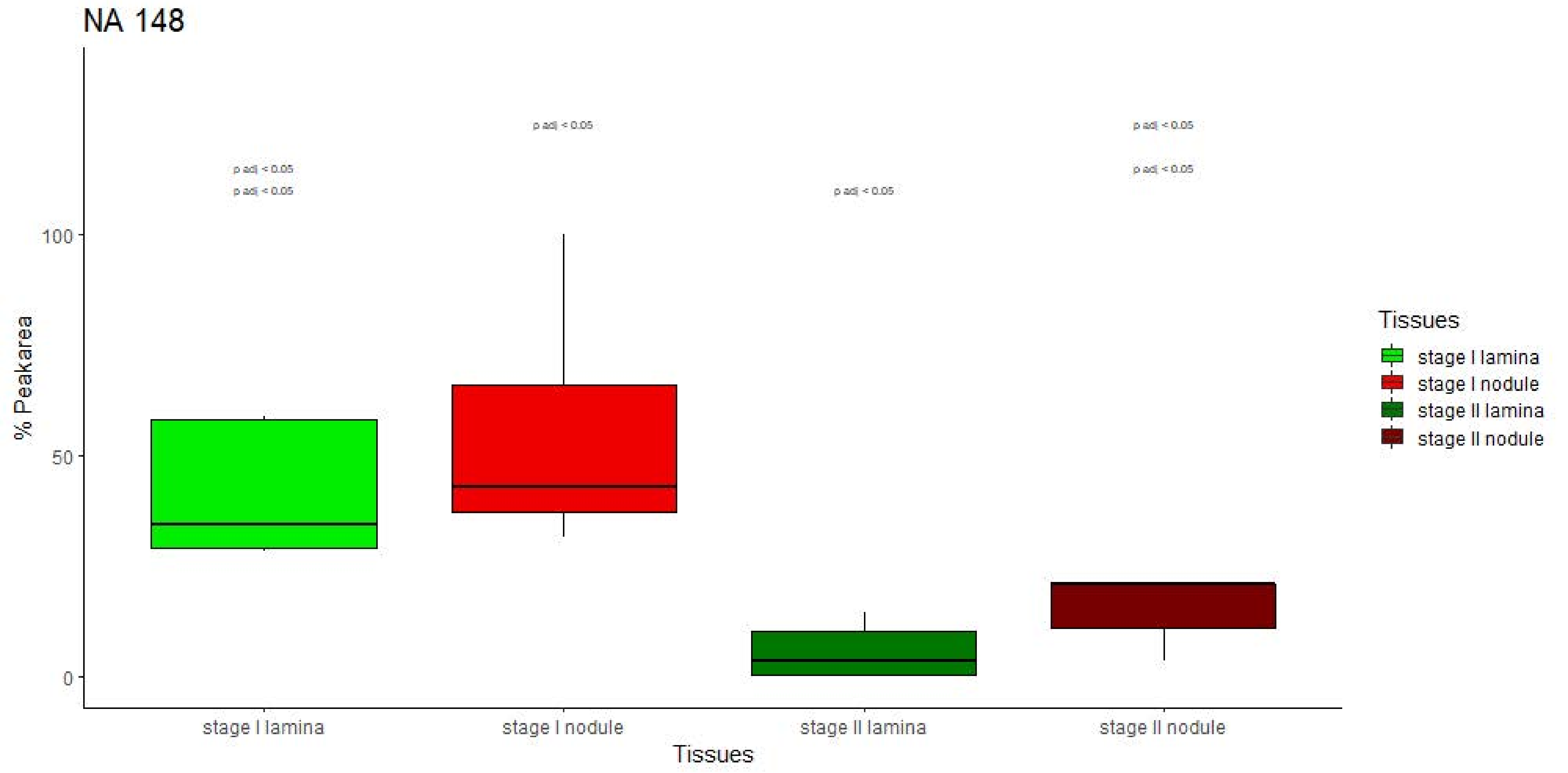
NA 148
p adj < 0.05
p adj < 0.05
p adj < 0.05
p adj < 0.05
p adj < 0.05
p adj < 0.05
p adj < 0.05
100
50
0
% Peakarea
stage I lamina
stage I nodule
stage II lamina
stage II nodule
Tissues
Tissues
stage I lamina
stage I nodule
stage II lamina
stage II nodule

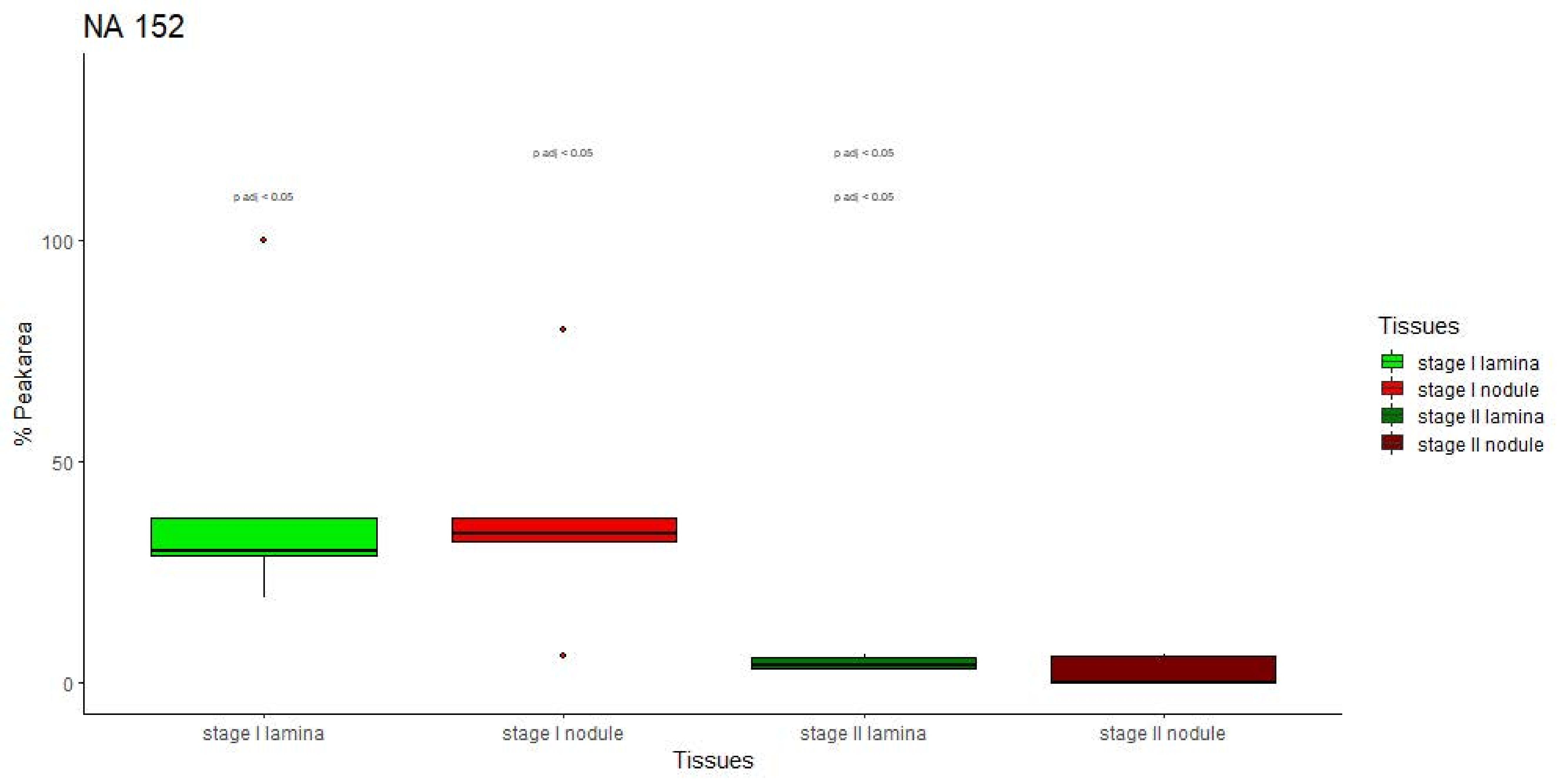

NA 152
p adj < 0.05
p adj < 0.05
p adj < 0.05
p adj < 0.05
100
50
0
% Peakarea
stage I lamina
stage I nodule
stage II lamina
stage II nodule
Tissues
Tissues
stage I lamina
stage I nodule
stage II lamina
stage II nodule

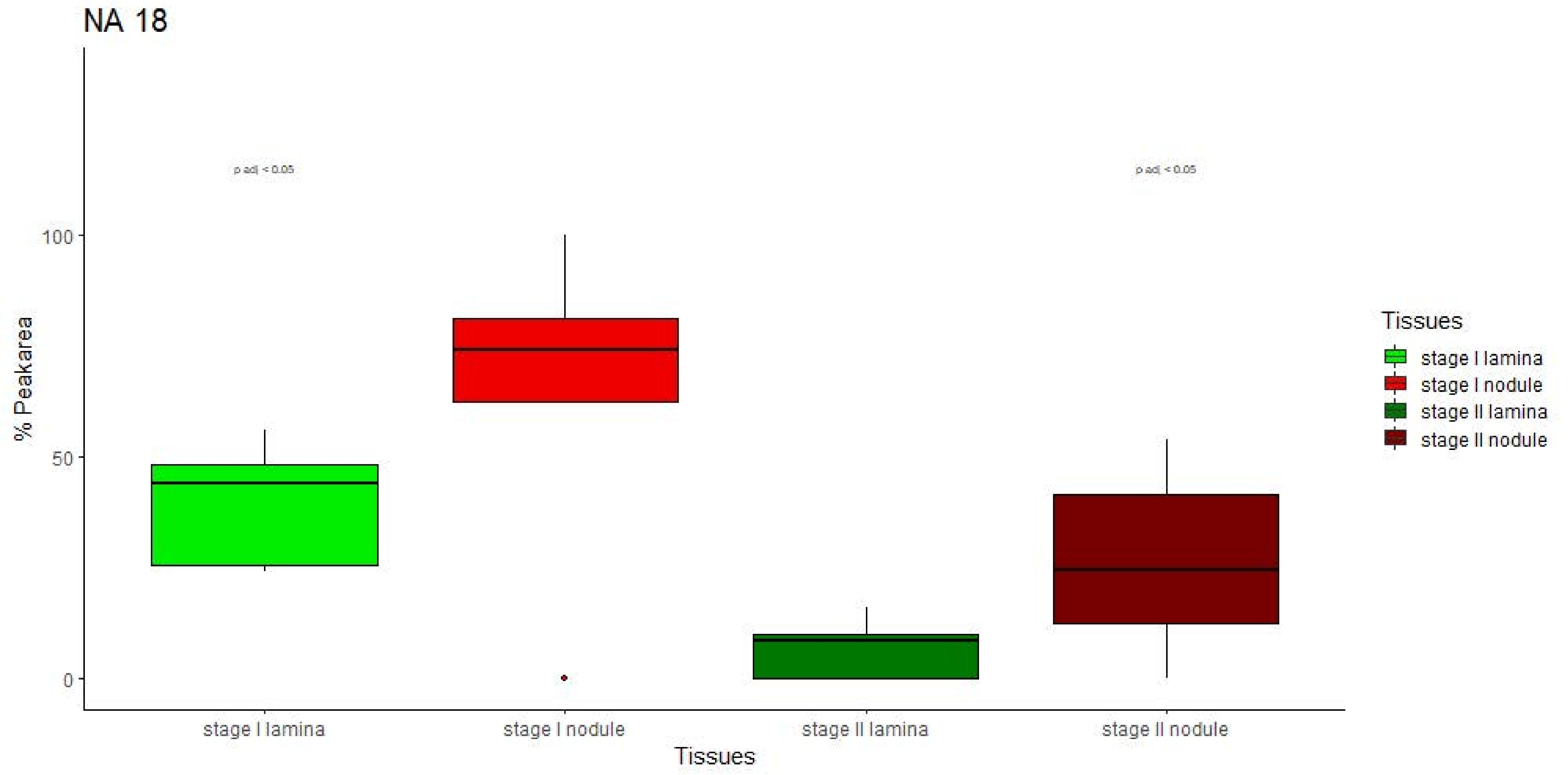
NA 18
p adj < 0.05
p adj < 0.05
100
50
0
% Peakarea
stage I lamina
stage I nodule
stage II lamina
stage II nodule
Tissues
Tissues
stage I lamina
stage I nodule
stage II lamina
stage II nodule

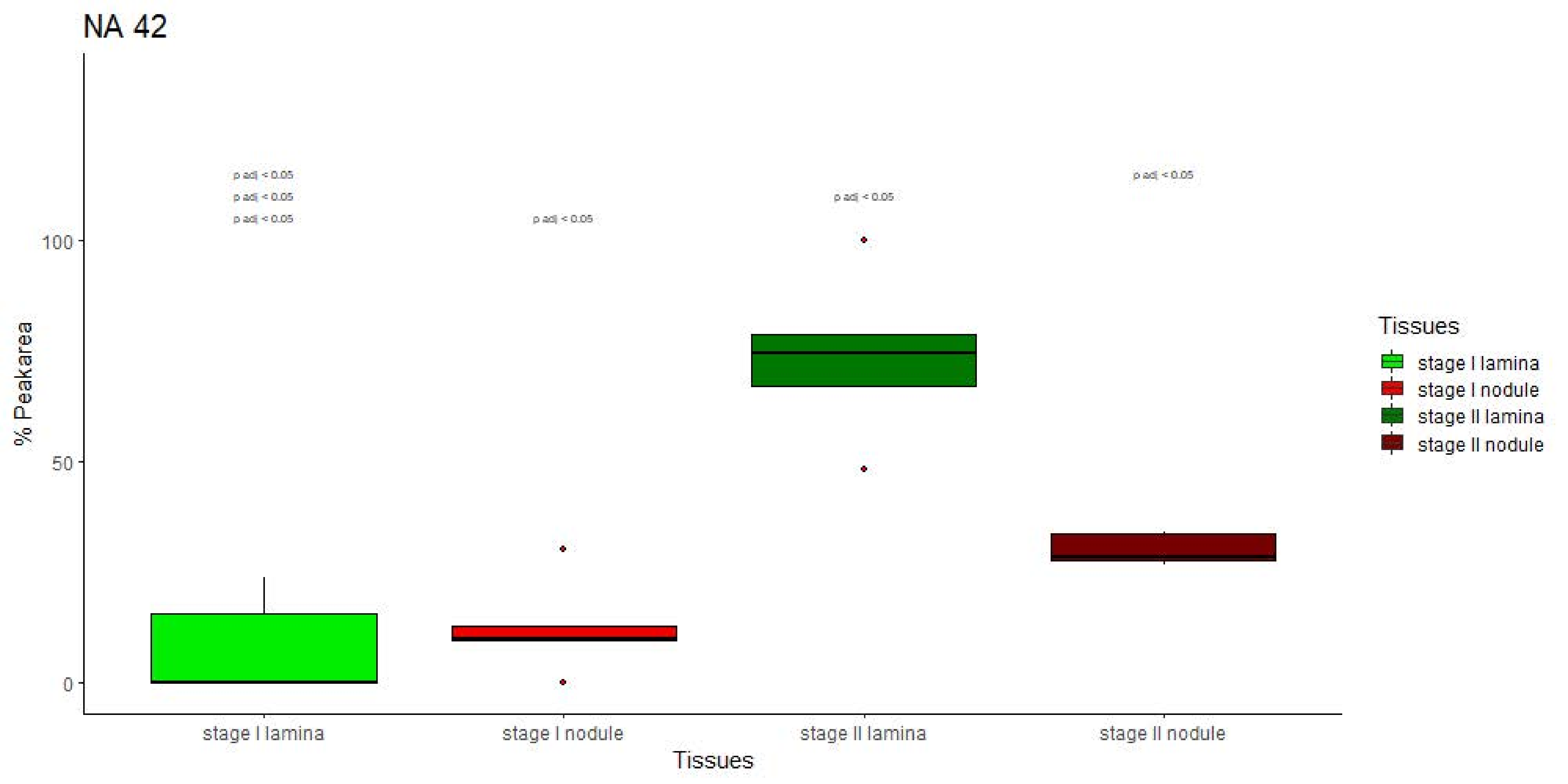
NA 42
p adj < 0.05
p adj < 0.05
p adj < 0.05
p adj < 0.05
p adj < 0.05
p adj < 0.05
100
50
0
% Peakarea
stage I lamina
stage I nodule
stage II lamina
stage II nodule
Tissues
Tissues
stage I lamina
stage I nodule
stage II lamina
stage II nodule

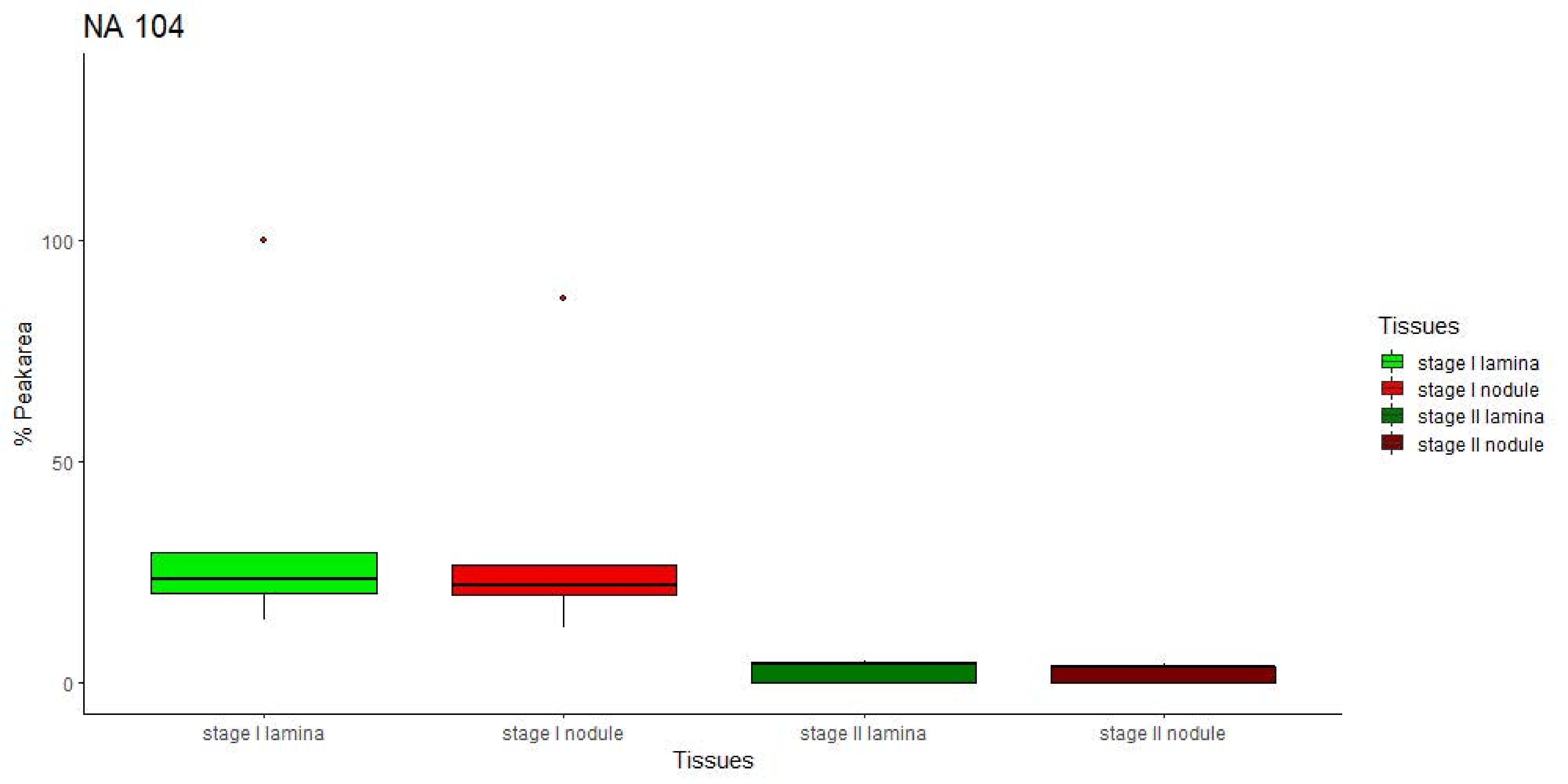

NA 104
% Peakarea
100
50
0
stage I lamina
stage I nodule
stage II lamina
stage II nodule
Tissues
Tissues
stage I lamina
stage I nodule
stage II lamina
stage II nodule

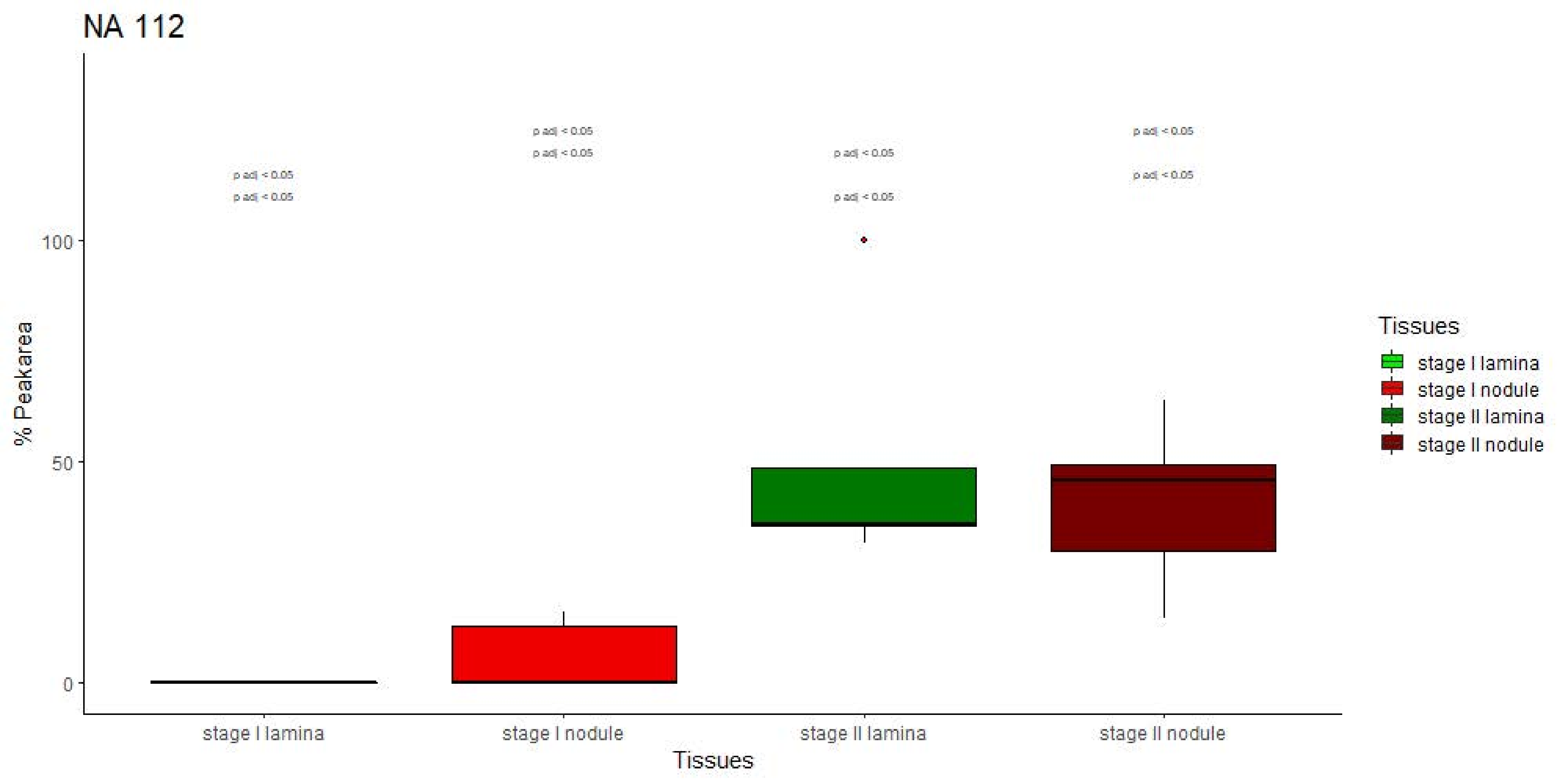
NA 112
p adj < 0.05
p adj < 0.05
p adj < 0.05
p adj < 0.05
p adj < 0.05
p adj < 0.05
p adj < 0.05
p adj < 0.05
100
50
0
% Peakarea
stage I lamina
stage I nodule
stage II lamina
stage II nodule
Tissues
Tissues
stage I lamina
stage I nodule
stage II lamina
stage II nodule

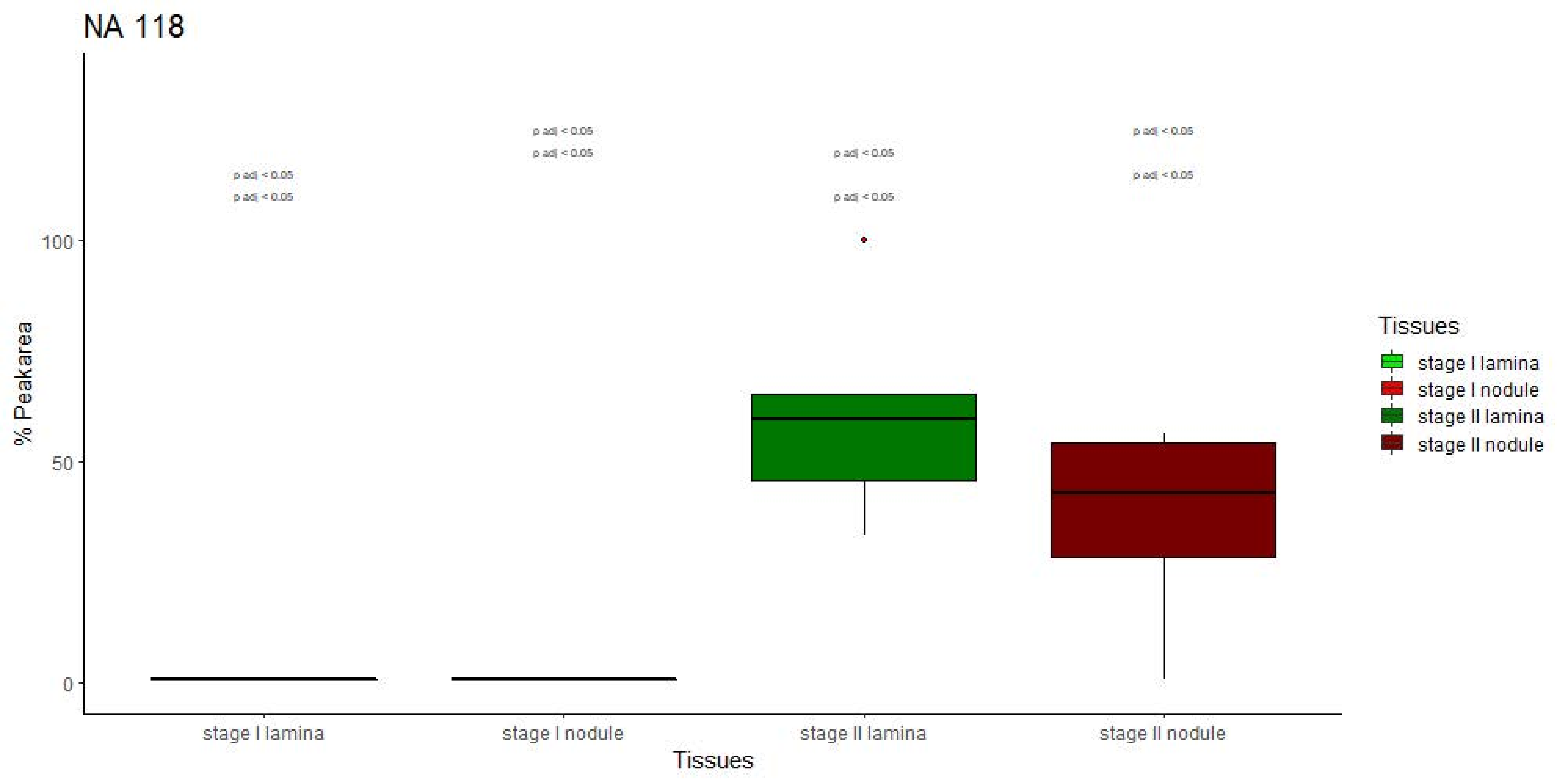

NA 118
p adj < 0.05
p adj < 0.05
p adj < 0.05
p adj < 0.05
p adj < 0.05
p adj < 0.05
p adj < 0.05
p adj < 0.05
100
50
0
% Peakarea
stage I lamina
stage I nodule
stage II lamina
stage II nodule
Tissues
Tissues
stage I lamina
stage I nodule
stage II lamina
stage II nodule

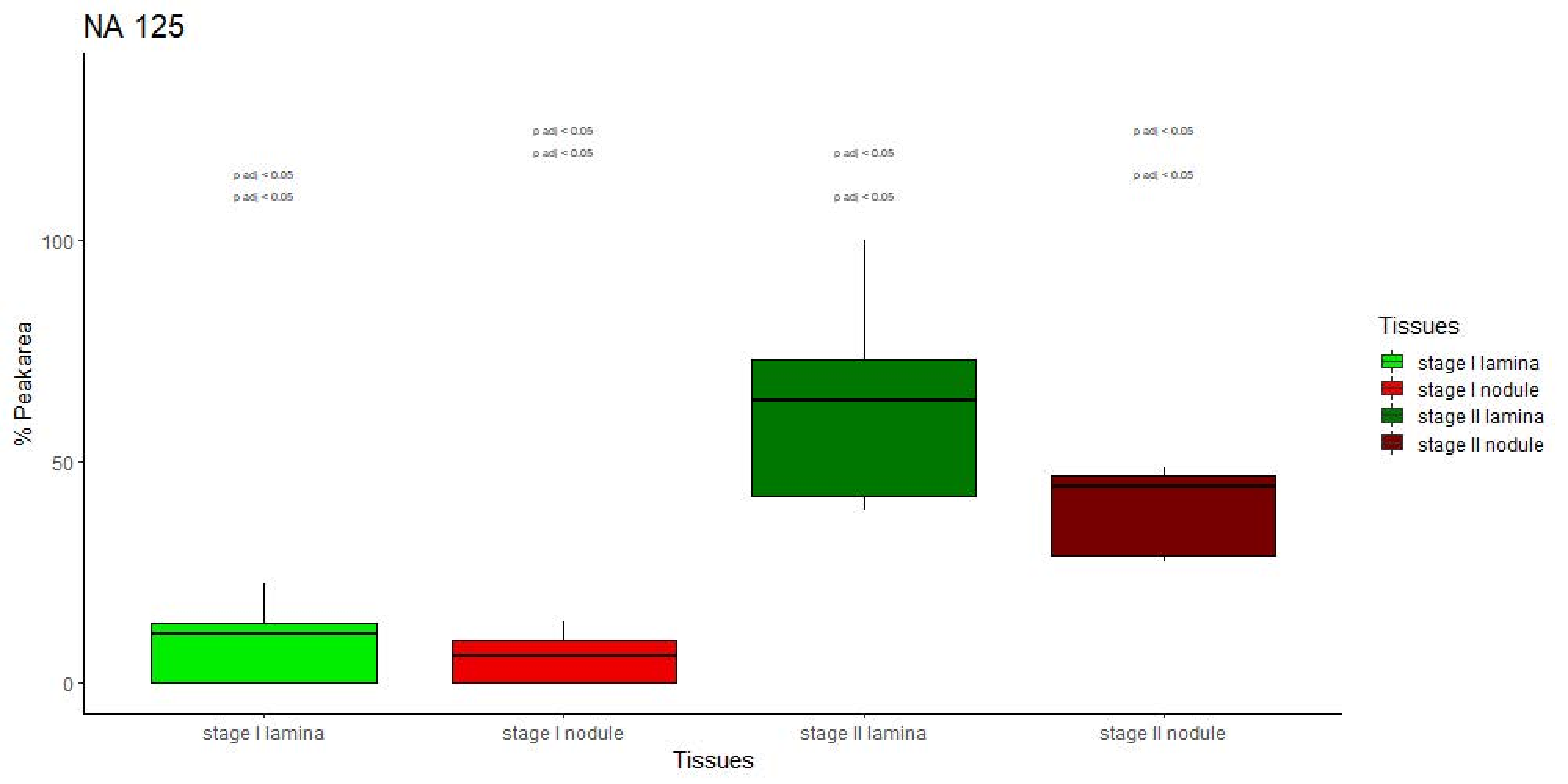
NA 125
p adj < 0.05
p adj < 0.05
p adj < 0.05
p adj < 0.05
p adj < 0.05
p adj < 0.05
p adj < 0.05
p adj < 0.05
100
50
0
% Peakarea
stage I lamina
stage I nodule
stage II lamina
stage II nodule
Tissues
Tissues
stage I lamina
stage I nodule
stage II lamina
stage II nodule

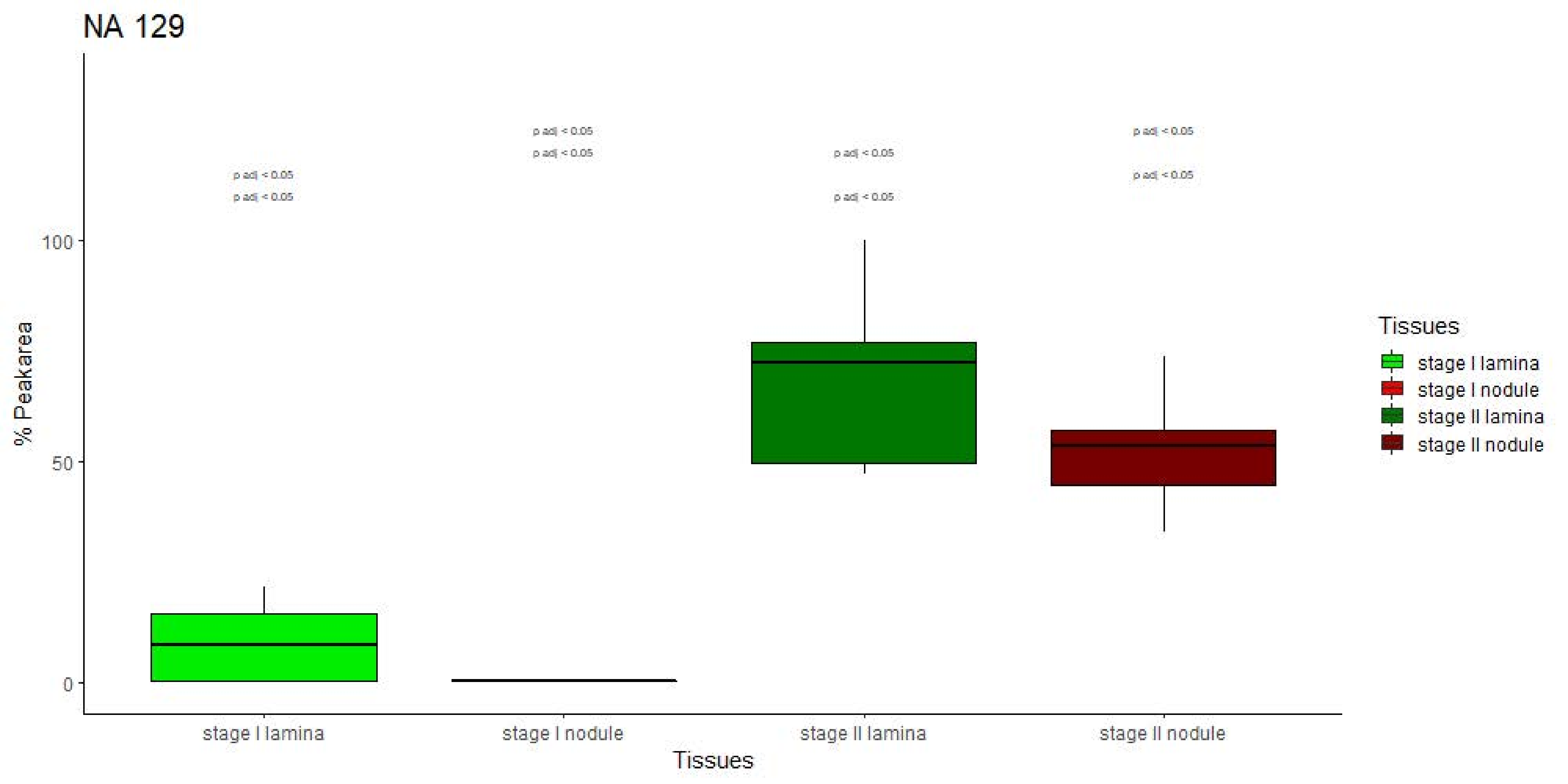

NA 129
p adj < 0.05
p adj < 0.05
p adj < 0.05
p adj < 0.05
p adj < 0.05
p adj < 0.05
p adj < 0.05
p adj < 0.05
100
50
0
% Peakarea
stage I lamina
stage I nodule
stage II lamina
stage II nodule
Tissues
Tissues
stage I lamina
stage I nodule
stage II lamina
stage II nodule

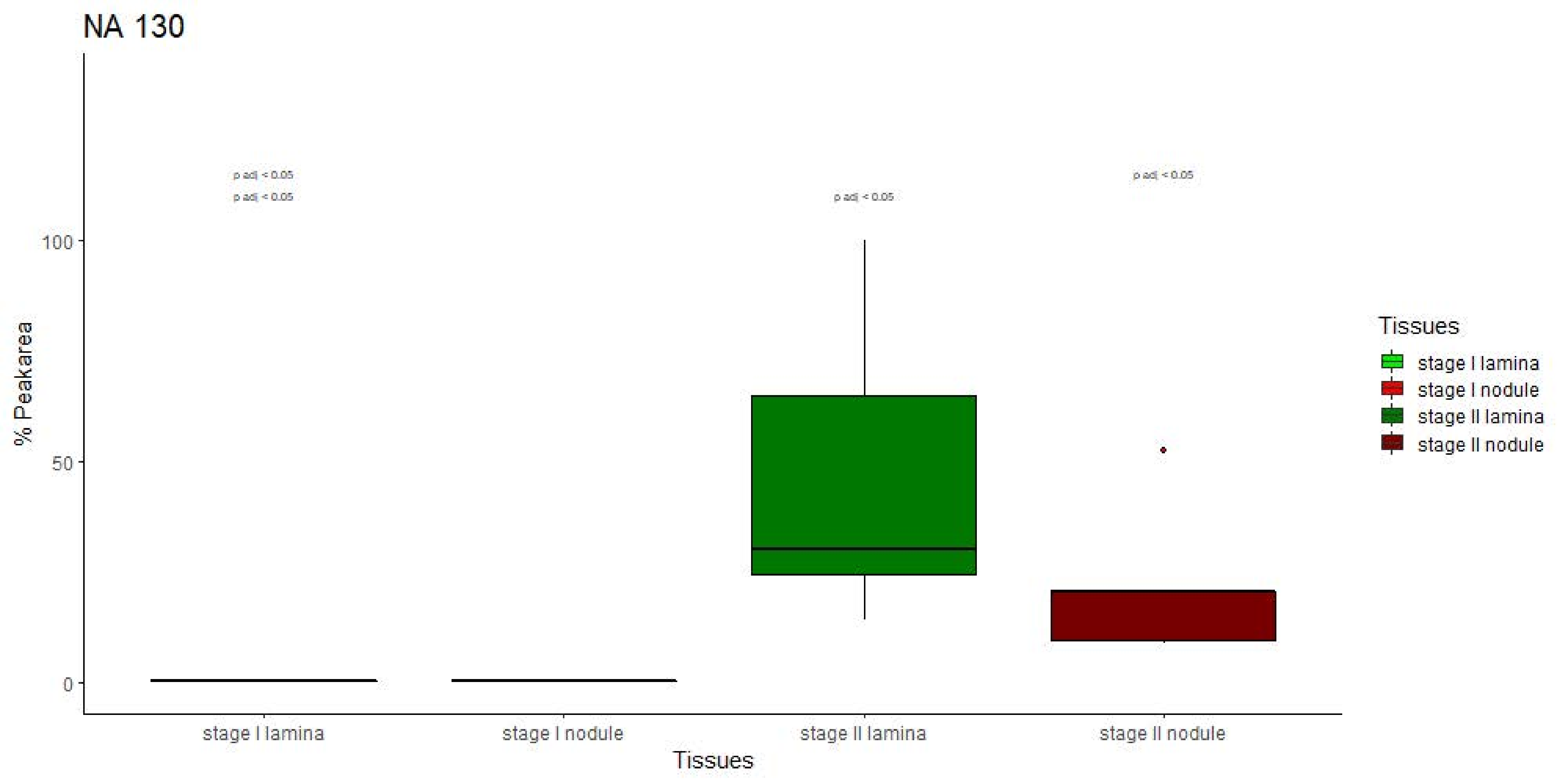
NA 130
p adj < 0.05
p adj < 0.05
p adj < 0.05
p adj < 0.05
100
50
0
% Peakarea
stage I lamina
stage I nodule
stage II lamina
stage II nodule
Tissues
Tissues
stage I lamina
stage I nodule
stage II lamina
stage II nodule

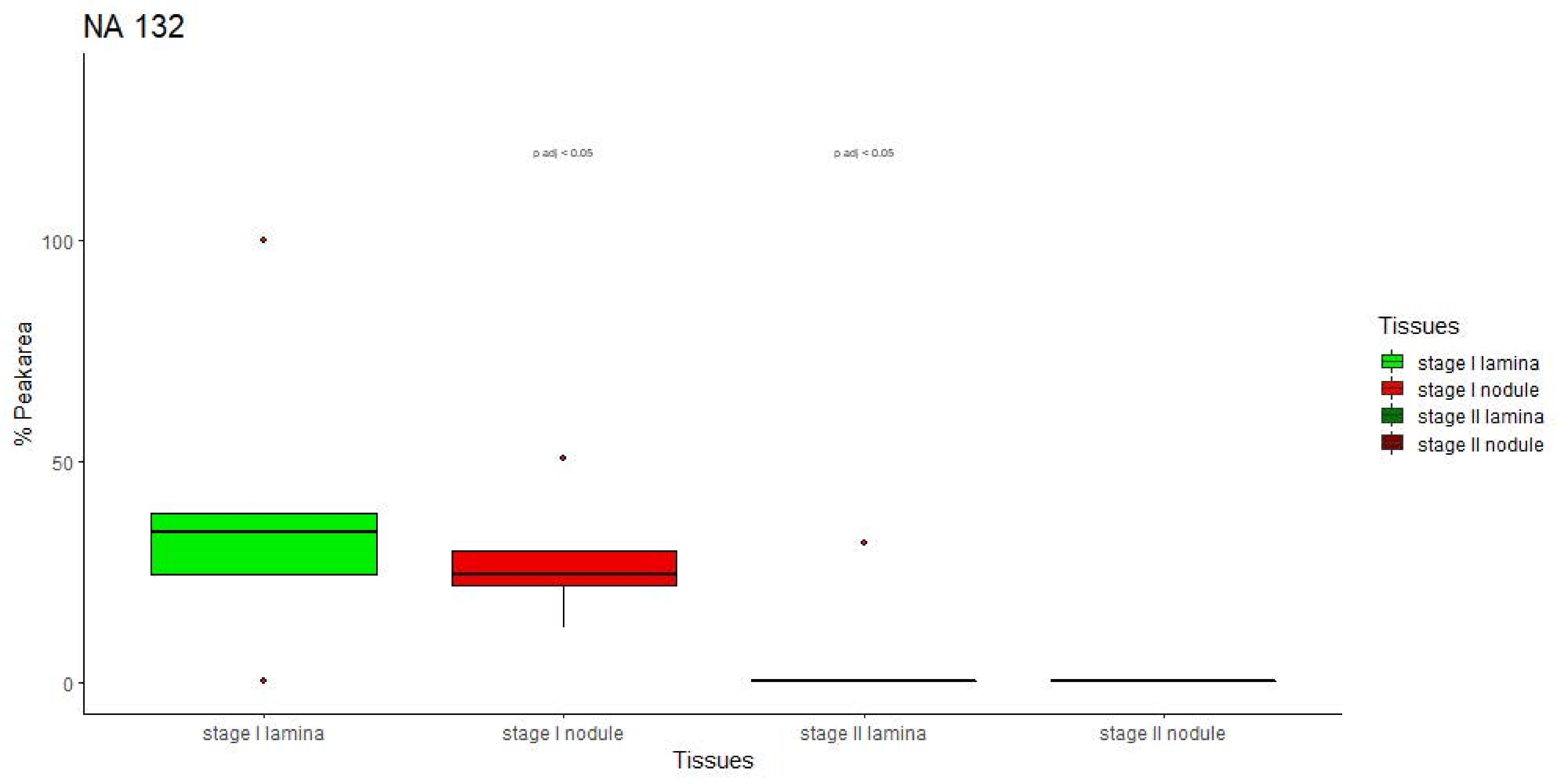
NA 132
p adj < 0.05
p adj < 0.05
100
50
0
% Peakarea
stage I lamina
stage I nodule
stage II lamina
stage II nodule
Tissues
Tissues
stage I lamina
stage I nodule
stage II lamina
stage II nodule

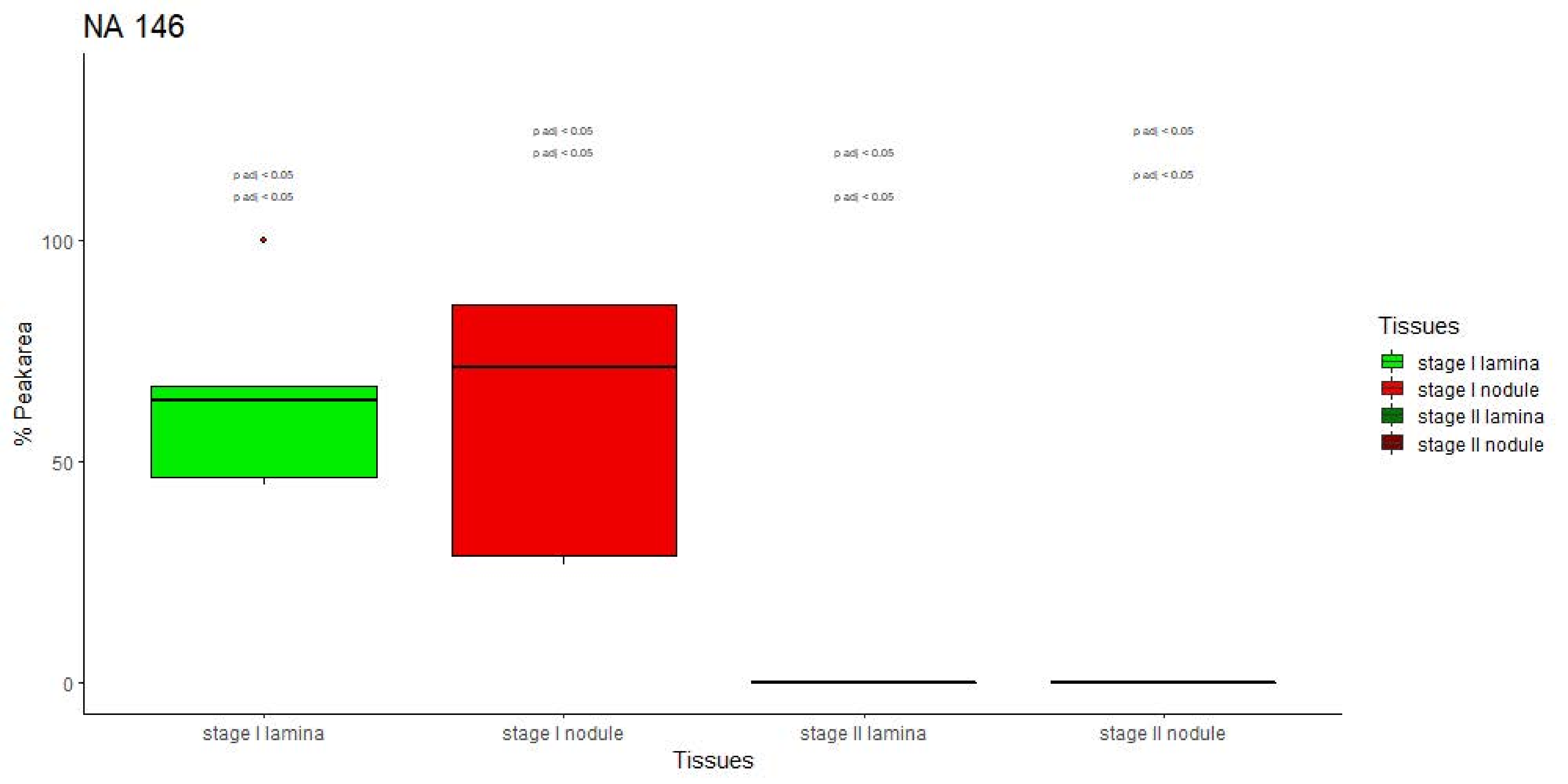

NA 146
p adj < 0.05
p adj < 0.05
p adj < 0.05
p adj < 0.05
p adj < 0.05
p adj < 0.05
p adj < 0.05
p adj < 0.05
100
50
0
% Peakarea
stage I lamina
stage I nodule
stage II lamina
stage II nodule
Tissues
Tissues
stage I lamina
stage I nodule
stage II lamina
stage II nodule

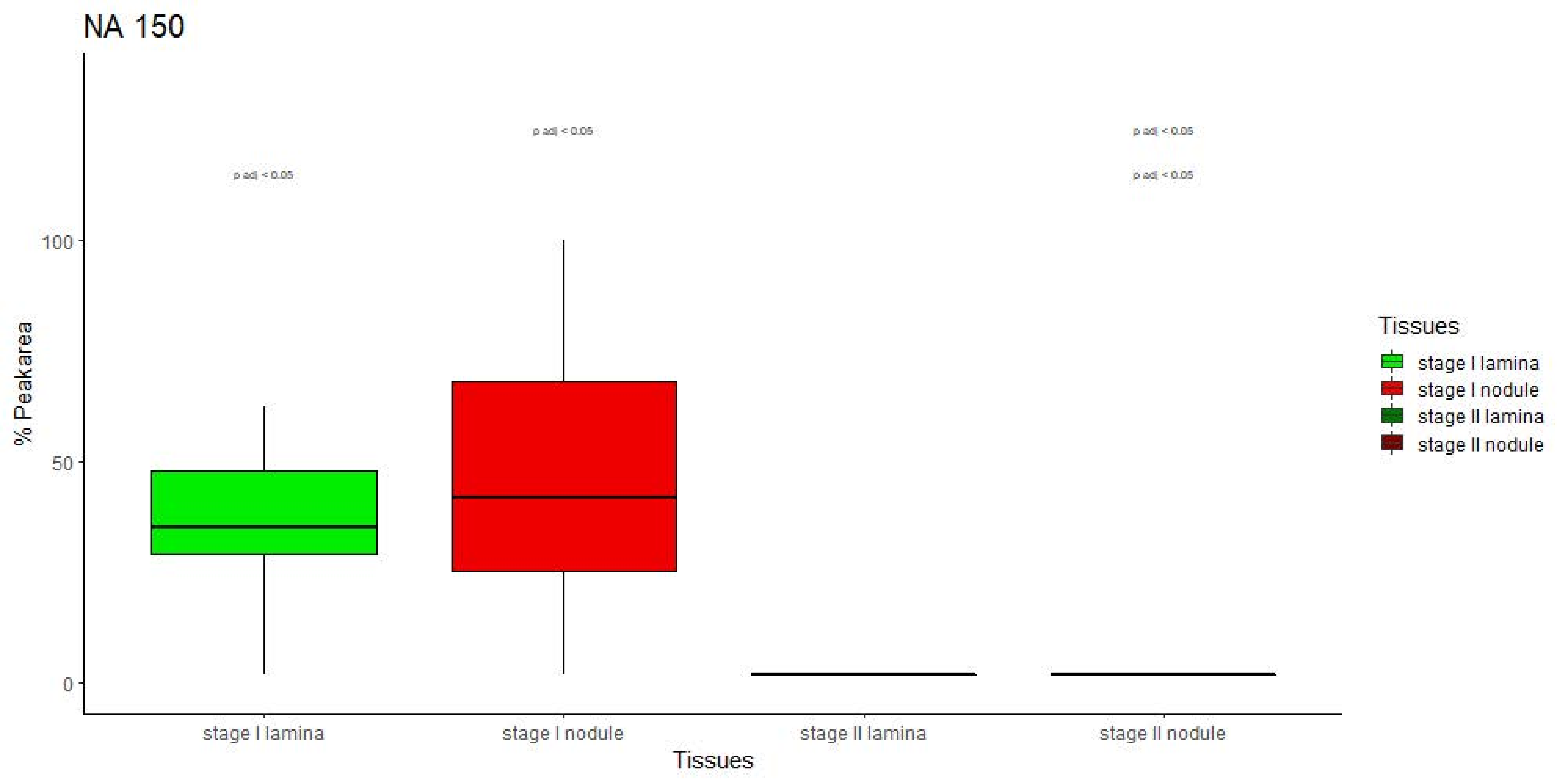
NA 150
% Peakarea
100
50
0
p adj < 0.05
p adj < 0.05
p adj < 0.05
p adj < 0.05
stage I lamina
stage I nodule
stage II lamina
stage II nodule
Tissues
Tissues
stage I lamina
stage I nodule
stage II lamina
stage II nodule

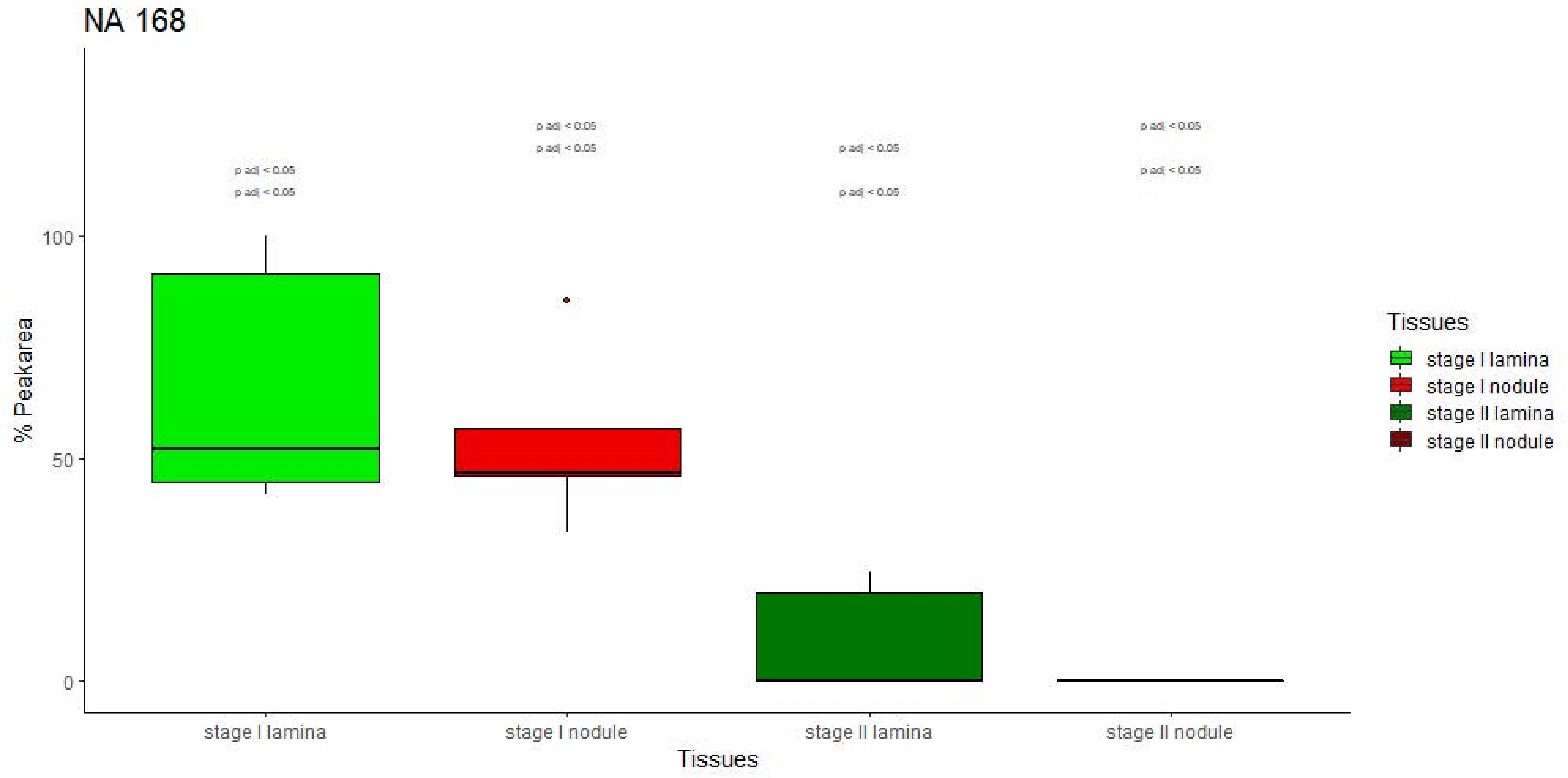

NA 168
p adj < 0.05
p adj < 0.05
p adj < 0.05
p adj < 0.05
p adj < 0.05
p adj < 0.05
p adj < 0.05
p adj < 0.05
100
50
0
% Peakarea
stage I lamina
stage I nodule
stage II lamina
stage II nodule
Tissues
Tissues
stage I lamina
stage I nodule
stage II lamina
stage II nodule

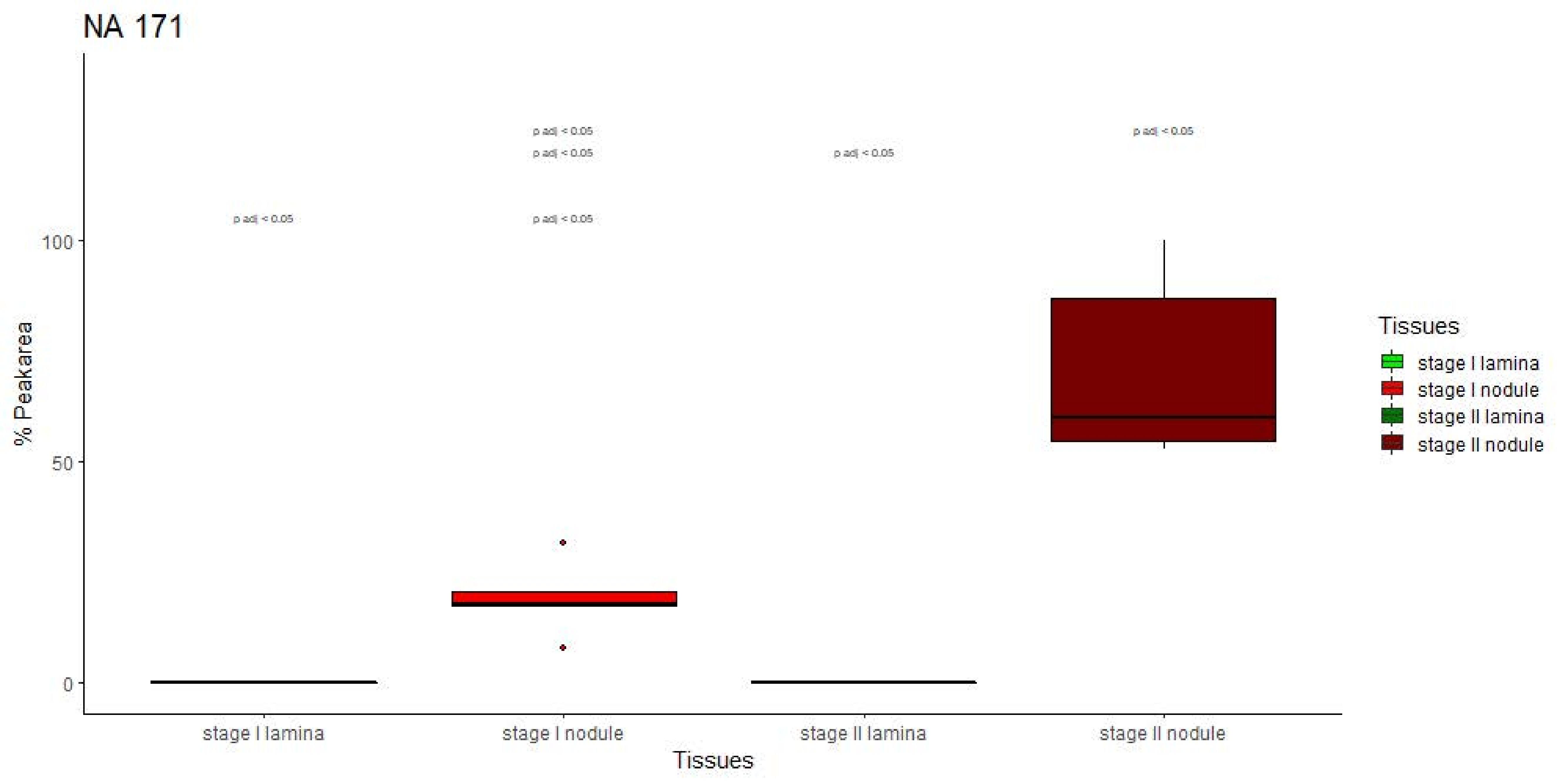

NA 171
p adj < 0.05
p adj < 0.05
p adj < 0.05
p adj < 0.05
p adj < 0.05
p adj < 0.05
100
50
0
% Peakarea
stage I lamina
stage I nodule
stage II lamina
stage II nodule
Tissues
Tissues
stage I lamina
stage I nodule
stage II lamina
stage II nodule

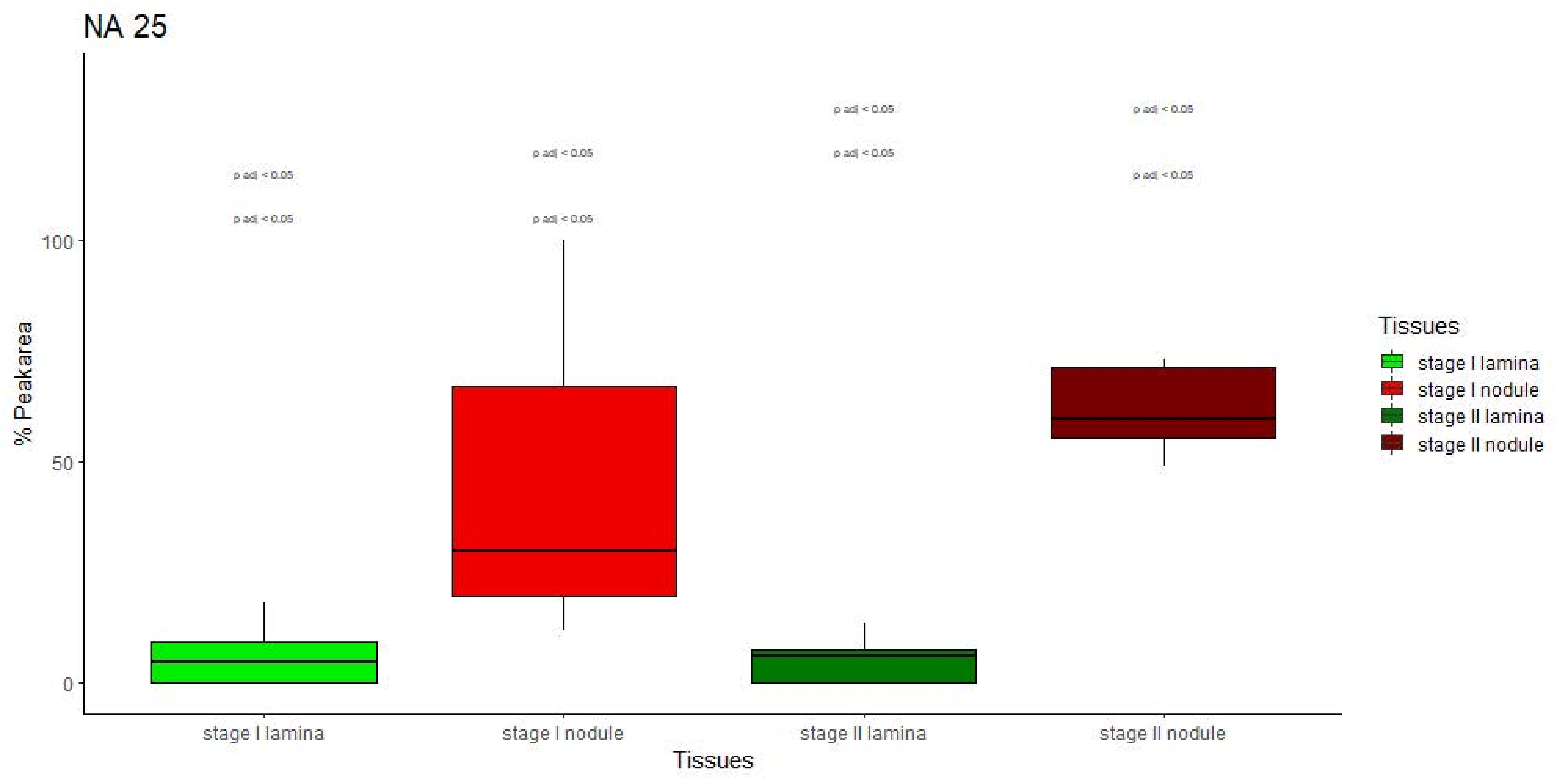
NA 25
p adj < 0.05
p adj < 0.05
p adj < 0.05
p adj < 0.05
p adj < 0.05
p adj < 0.05
p adj < 0.05
p adj < 0.05
100
50
0
% Peakarea
stage I lamina
stage I nodule
stage II lamina
stage II nodule
Tissues
Tissues
stage I lamina
stage I nodule
stage II lamina
stage II nodule

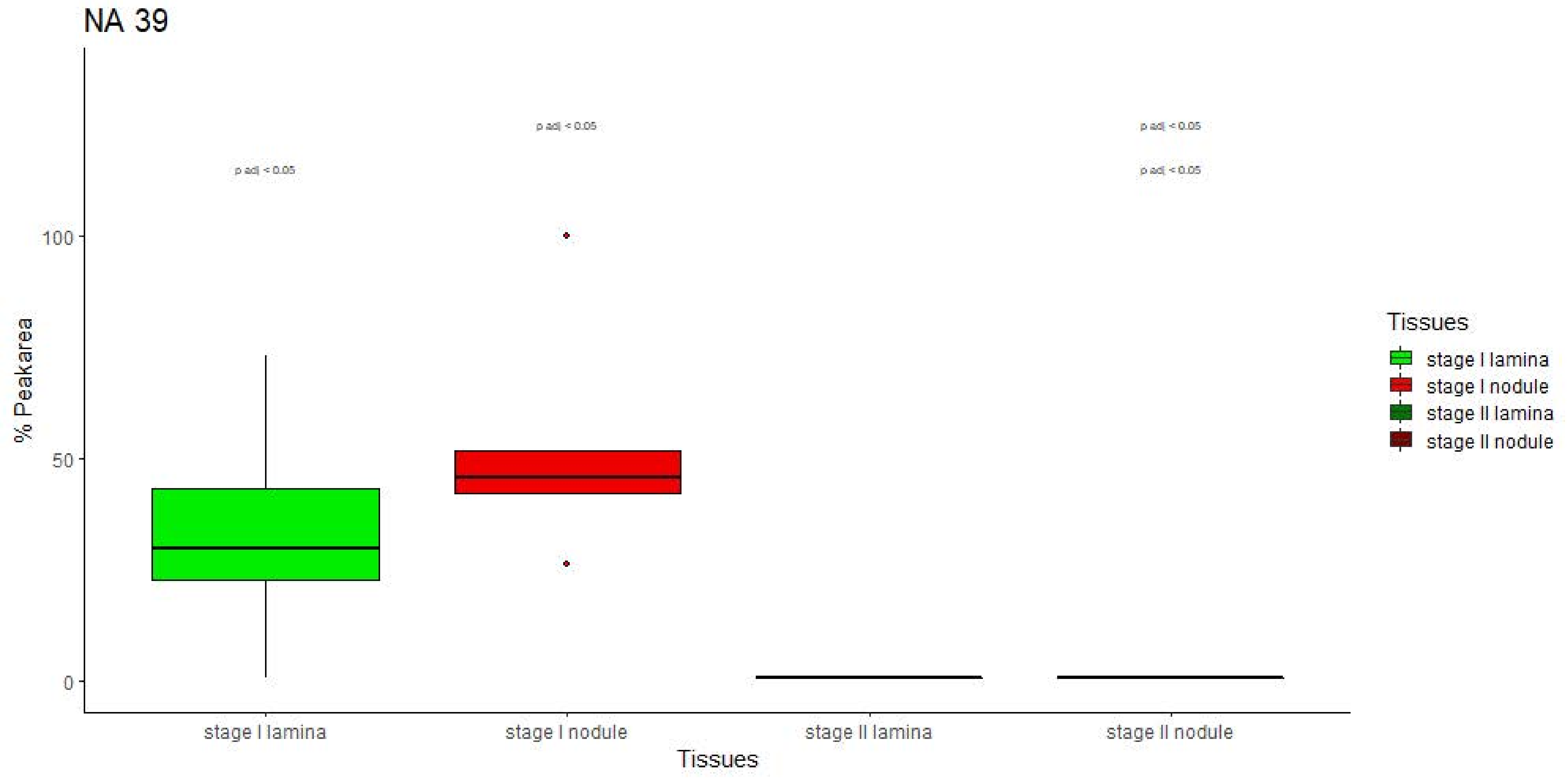
NA 39
p adj < 0.05
p adj < 0.05
p adj < 0.05
p adj < 0.05
100
50
0
% Peakarea
stage I lamina
stage I nodule
stage II lamina
stage II nodule
Tissues
Tissues
stage I lamina
stage I nodule
stage II lamina
stage II nodule

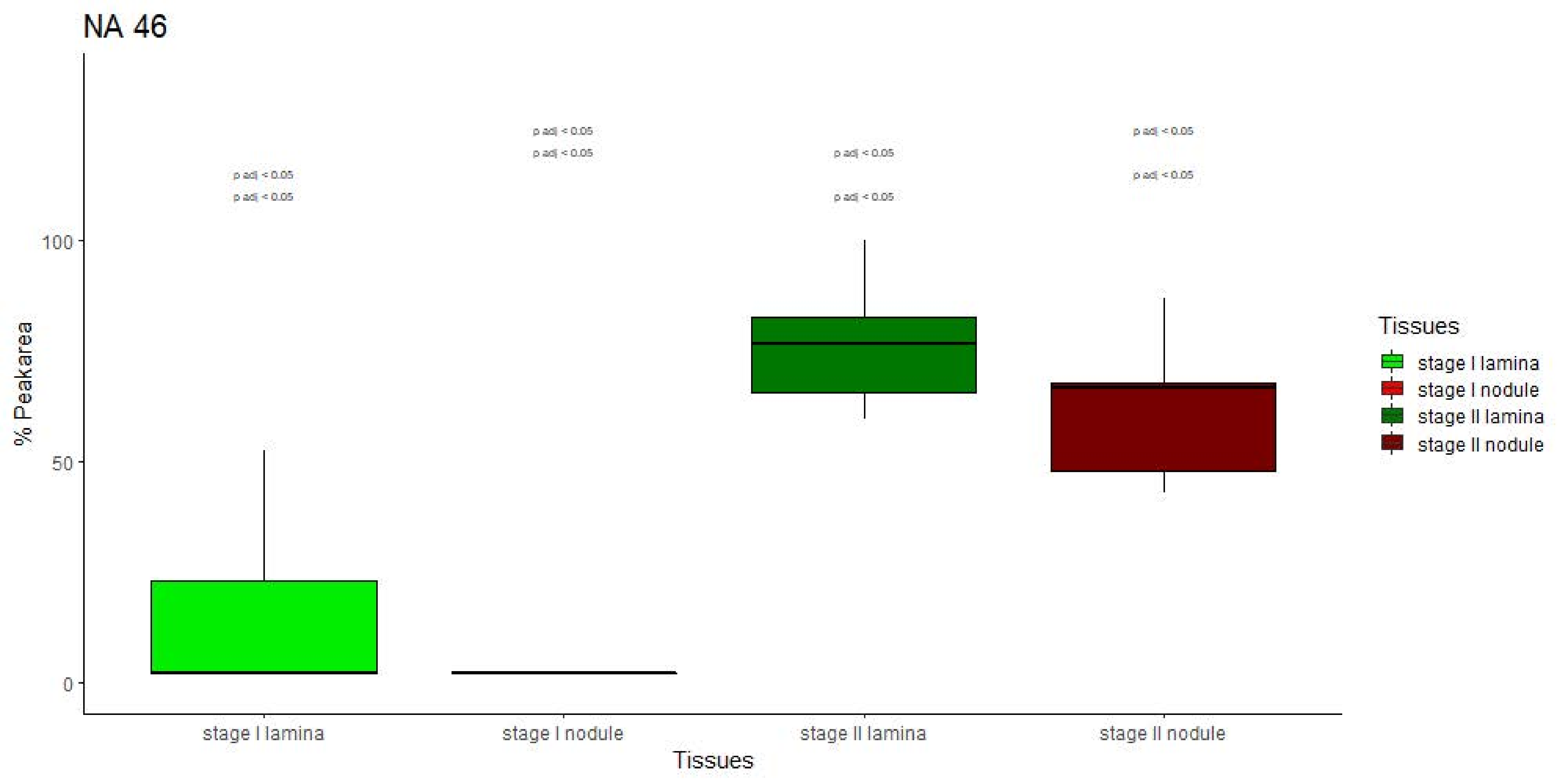

NA 46
p adj < 0.05
p adj < 0.05
p adj < 0.05
p adj < 0.05
p adj < 0.05
p adj < 0.05
p adj < 0.05
p adj < 0.05
100
50
0
% Peakarea
stage I lamina
stage I nodule
stage II lamina
stage II nodule
Tissues
Tissues
stage I lamina
stage I nodule
stage II lamina
stage II nodule

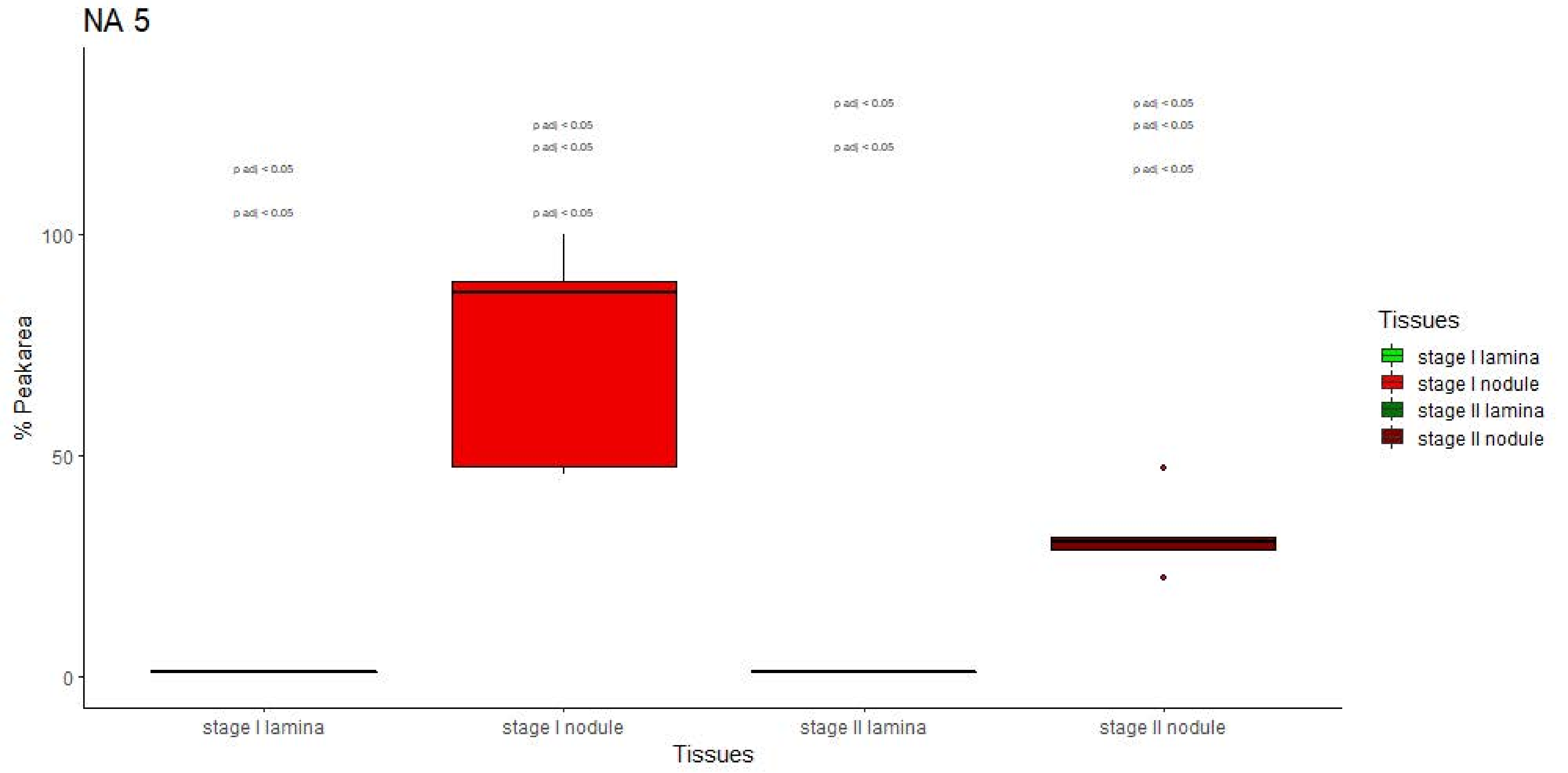

NA 5
p adj < 0.05
p adj < 0.05
p adj < 0.05
p adj < 0.05
p adj < 0.05
p adj < 0.05
p adj < 0.05
p adj < 0.05
p adj < 0.05
p adj < 0.05
100
50
0
% Peakarea
stage I lamina
stage I nodule
stage II lamina
stage II nodule
Tissues
Tissues
stage I lamina
stage I nodule
stage II lamina
stage II nodule

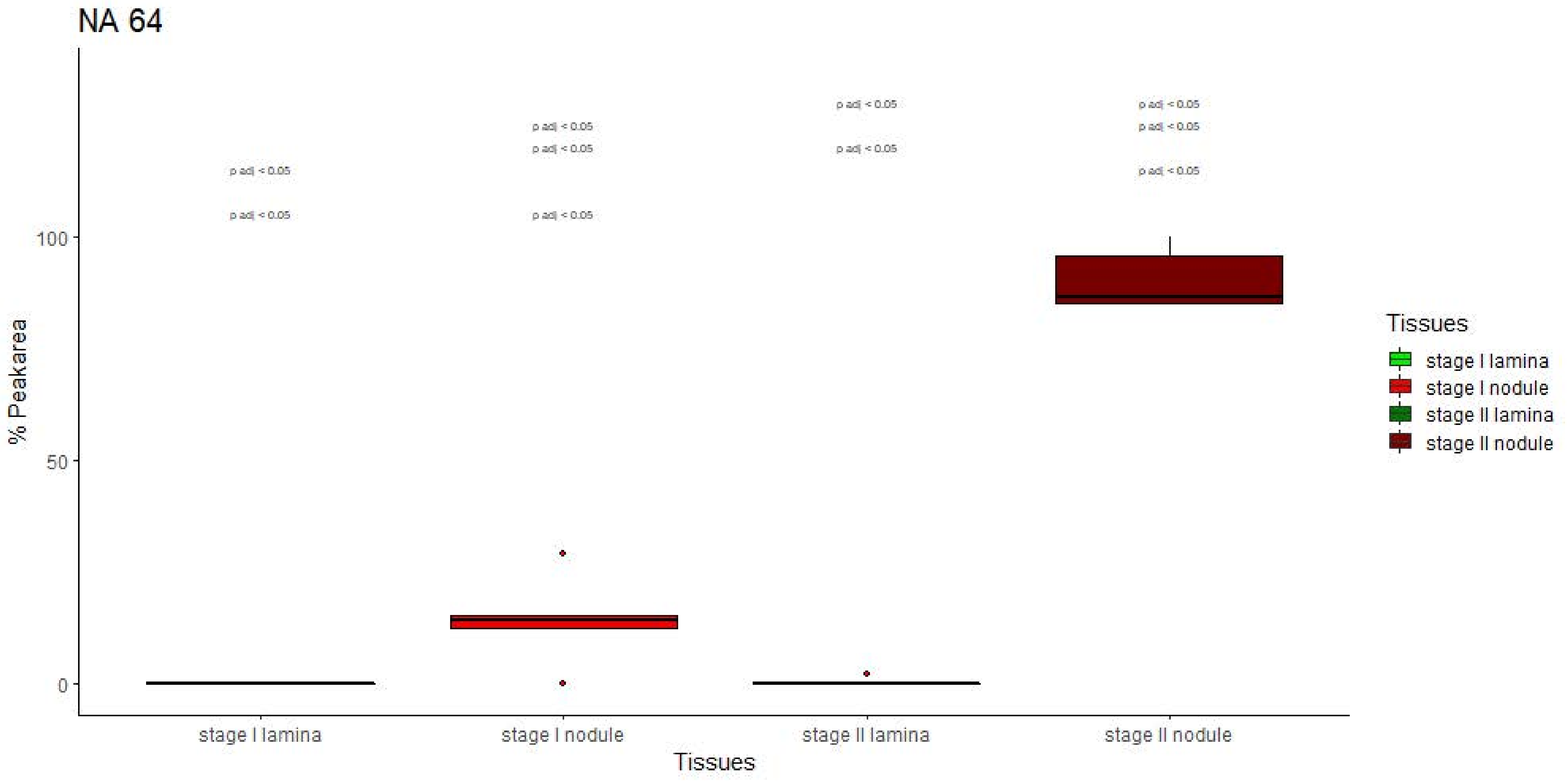
NA 64
p adj < 0.05
p adj < 0.05
p adj < 0.05
p adj < 0.05
p adj < 0.05
p adj < 0.05
p adj < 0.05
p adj < 0.05
p adj < 0.05
p adj < 0.05
100
50
0
% Peakarea
stage I lamina
stage I nodule
stage II lamina
stage II nodule
Tissues
Tissues
stage I lamina
stage I nodule
stage II lamina
stage II nodule

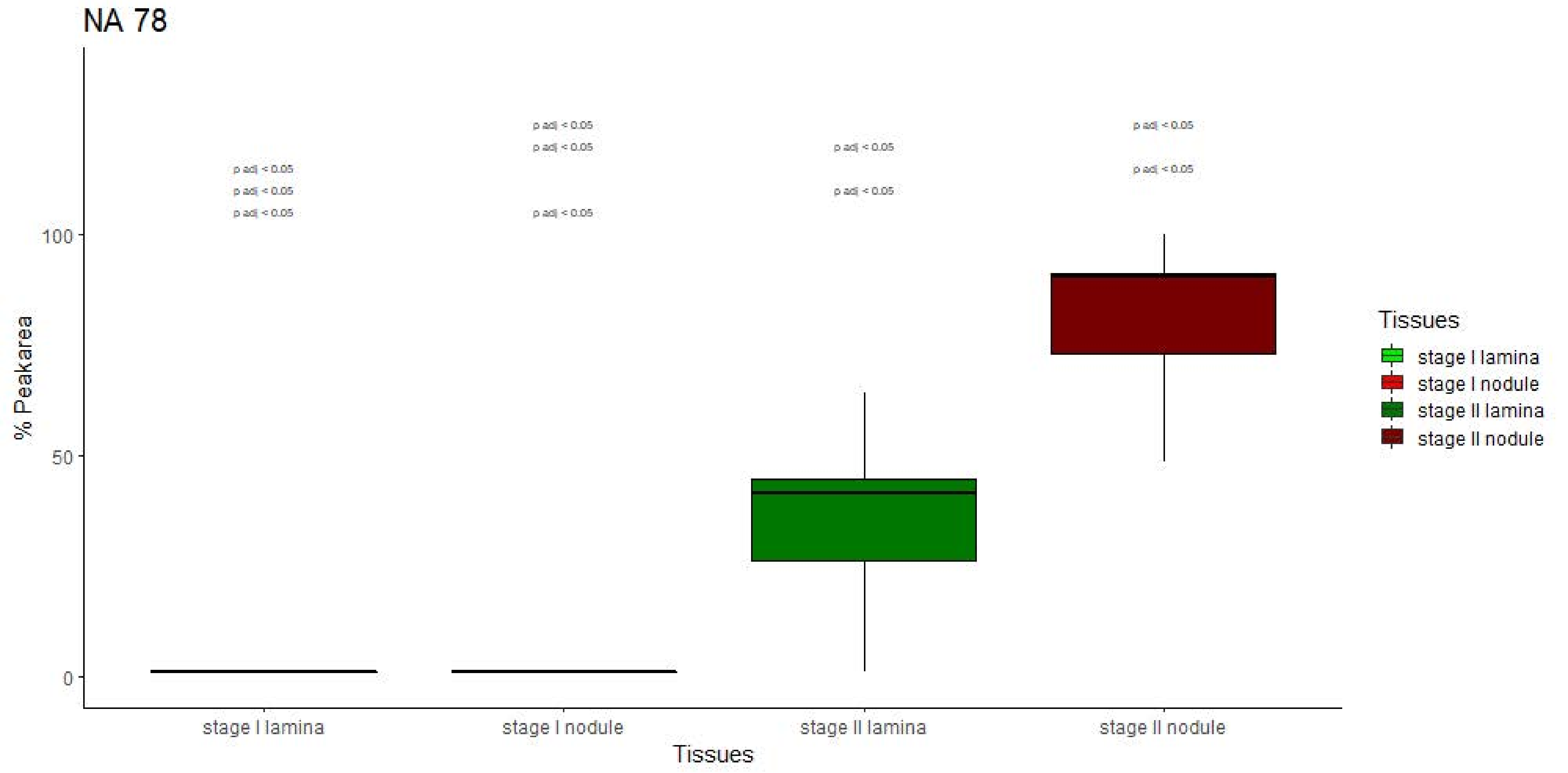
NA 78
p adj < 0.05
p adj < 0.05
p adj < 0.05
p adj < 0.05
p adj < 0.05
p adj < 0.05
p adj < 0.05
p adj < 0.05
p adj < 0.05
p adj < 0.05
100
50
0
% Peakarea
stage I lamina
stage I nodule
stage II lamina
stage II nodule
Tissues
Tissues
stage I lamina
stage I nodule
stage II lamina
stage II nodule

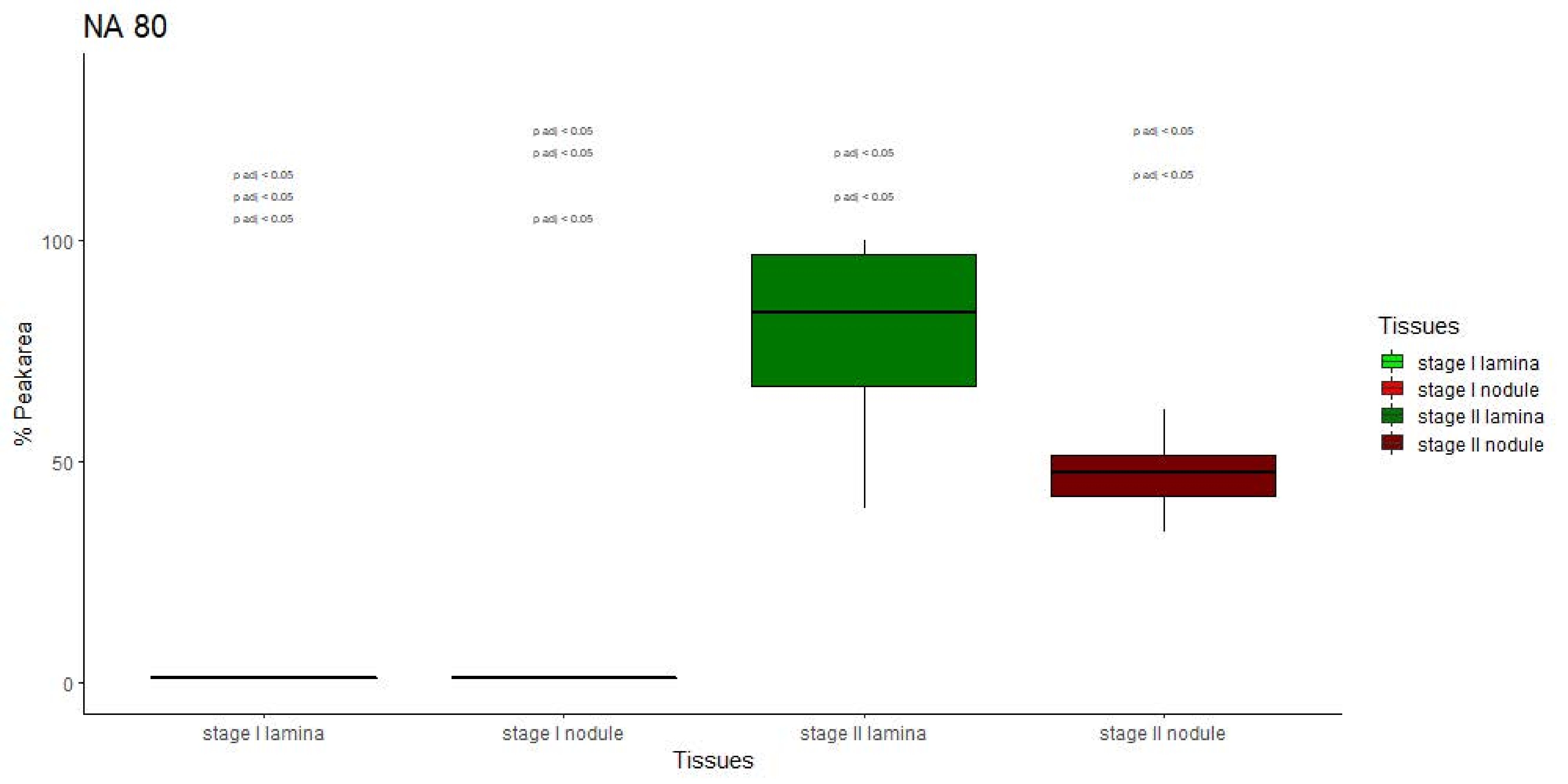

NA 80
p adj < 0.05
p adj < 0.05
p adj < 0.05
p adj < 0.05
p adj < 0.05
p adj < 0.05
p adj < 0.05
p adj < 0.05
p adj < 0.05
p adj < 0.05
100
50
0
% Peakarea
stage I lamina
stage I nodule
stage II lamina
stage II nodule
Tissues
Tissues
stage I lamina
stage I nodule
stage II lamina
stage II nodule

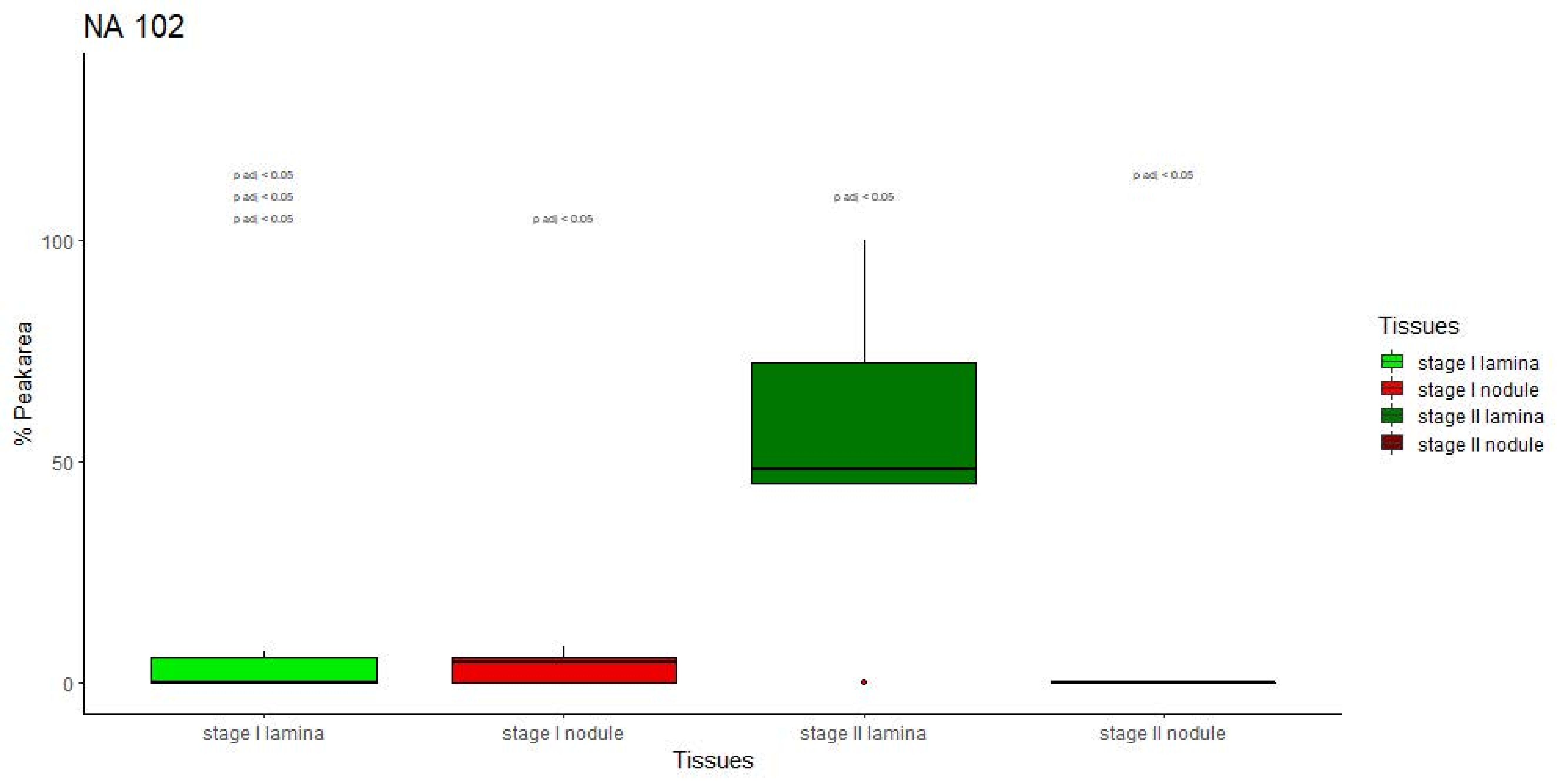
NA 102
p adj < 0.05
p adj < 0.05
p adj < 0.05
p adj < 0.05
p adj < 0.05
p adj < 0.05
100
50
0
% Peakarea
stage I lamina
stage I nodule
stage II lamina
stage II nodule
Tissues
Tissues
stage I lamina
stage I nodule
stage II lamina
stage II nodule

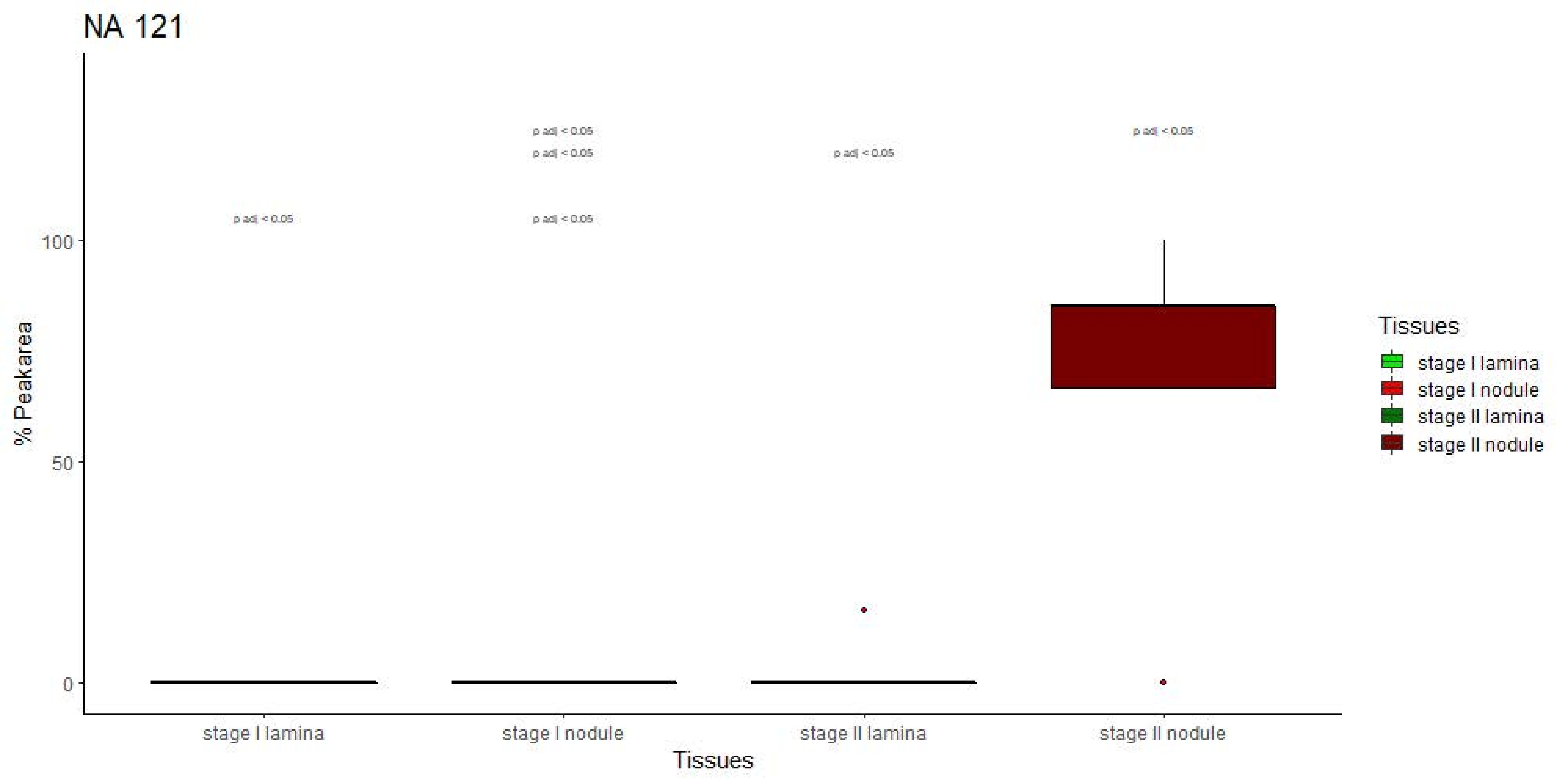

NA 121
p adj < 0.05
p adj < 0.05
p adj < 0.05
p adj < 0.05
p adj < 0.05
p adj < 0.05
p adj < 0.05
100
50
0
% Peakarea
stage I lamina
stage I nodule
stage II lamina
stage II nodule
Tissues
Tissues
stage I lamina
stage I nodule
stage II lamina
stage II nodule

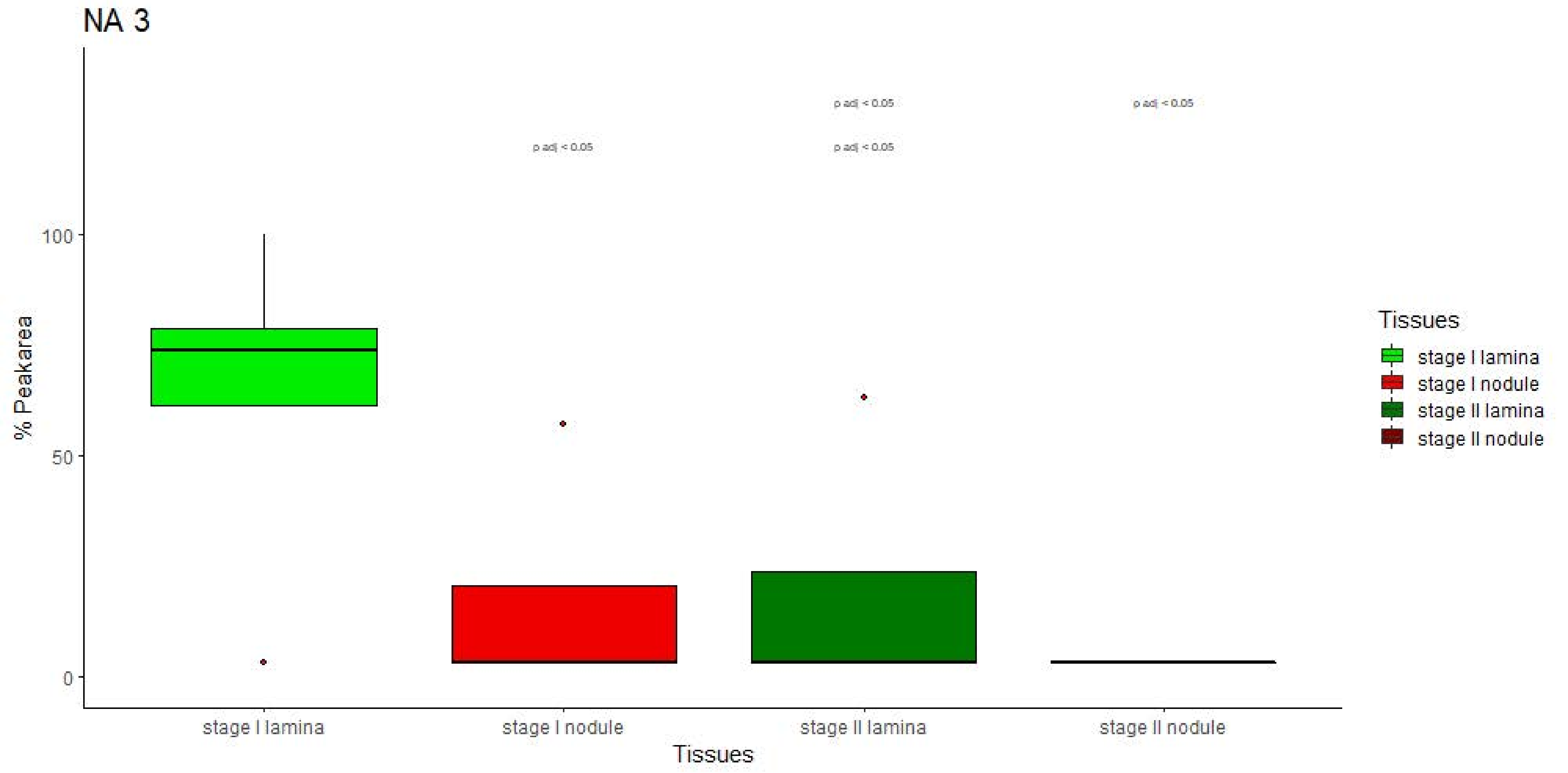
NA 3
% Peakarea
100
50
0
p adj < 0.05
p adj < 0.05
p adj < 0.05
p adj < 0.05
stage I lamina
stage I nodule
stage II lamina
stage II nodule
Tissues
Tissues
stage I lamina
stage I nodule
stage II lamina
stage II nodule

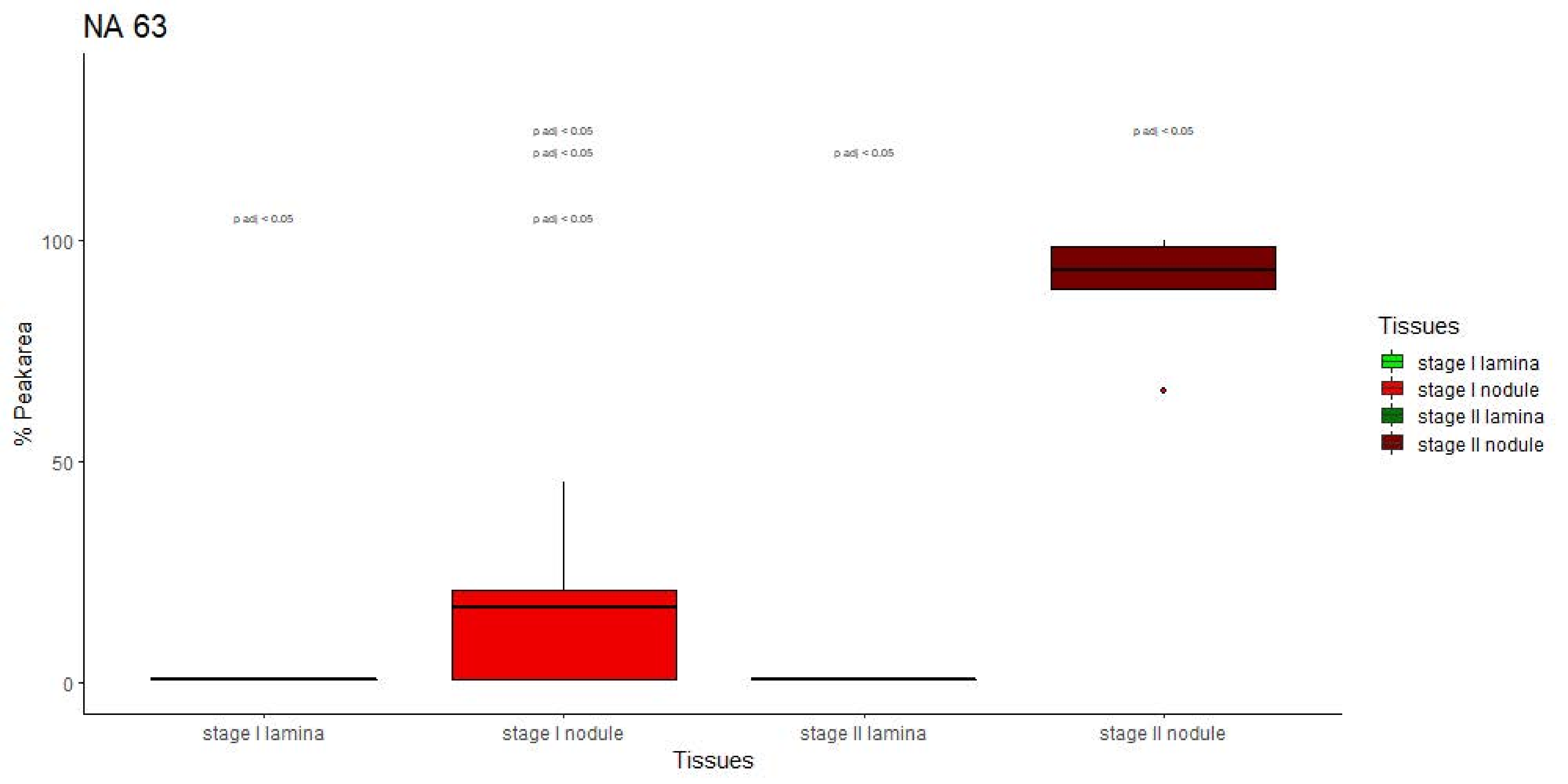

NA 63
p adj < 0.05
p adj < 0.05
p adj < 0.05
p adj < 0.05
p adj < 0.05
p adj < 0.05
100
50
0
% Peakarea
stage I lamina
stage I nodule
stage II lamina
stage II nodule
Tissues
Tissues
stage I lamina
stage I nodule
stage II lamina
stage II nodule

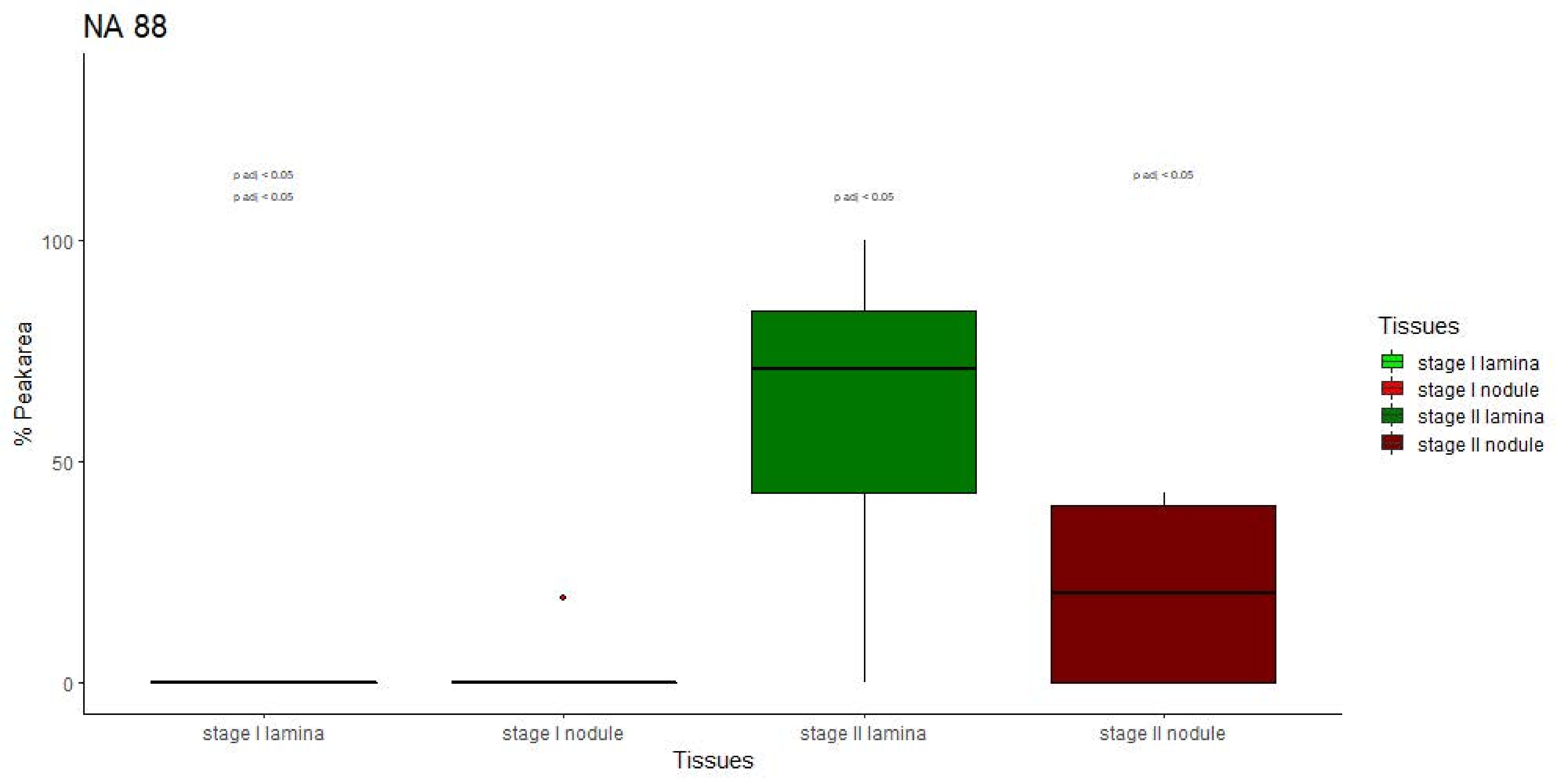

NA 88
p adj < 0.05
p adj < 0.05
p adj < 0.05
p adj < 0.05
100
50
0
% Peakarea
stage I lamina
stage I nodule
stage II lamina
stage II nodule
Tissues
Tissues
stage I lamina
stage I nodule
stage II lamina
stage II nodule
