# Supplementary material for: Dissecting Metabolism of Leaf Nodules in Ardisia crenata and Psychotria punctata
Source: Front Mol Biosci. 2021 Jul 30;8:683671. doi: 10.3389/fmolb.2021.683671 (PMC8362603; doi:10.3389/fmolb.2021.683671)
Supplement: Supplementary file 8 [file DataSheet1.pdf]

Epigallocatechin RT:14.70 min *m/z*:307.0812

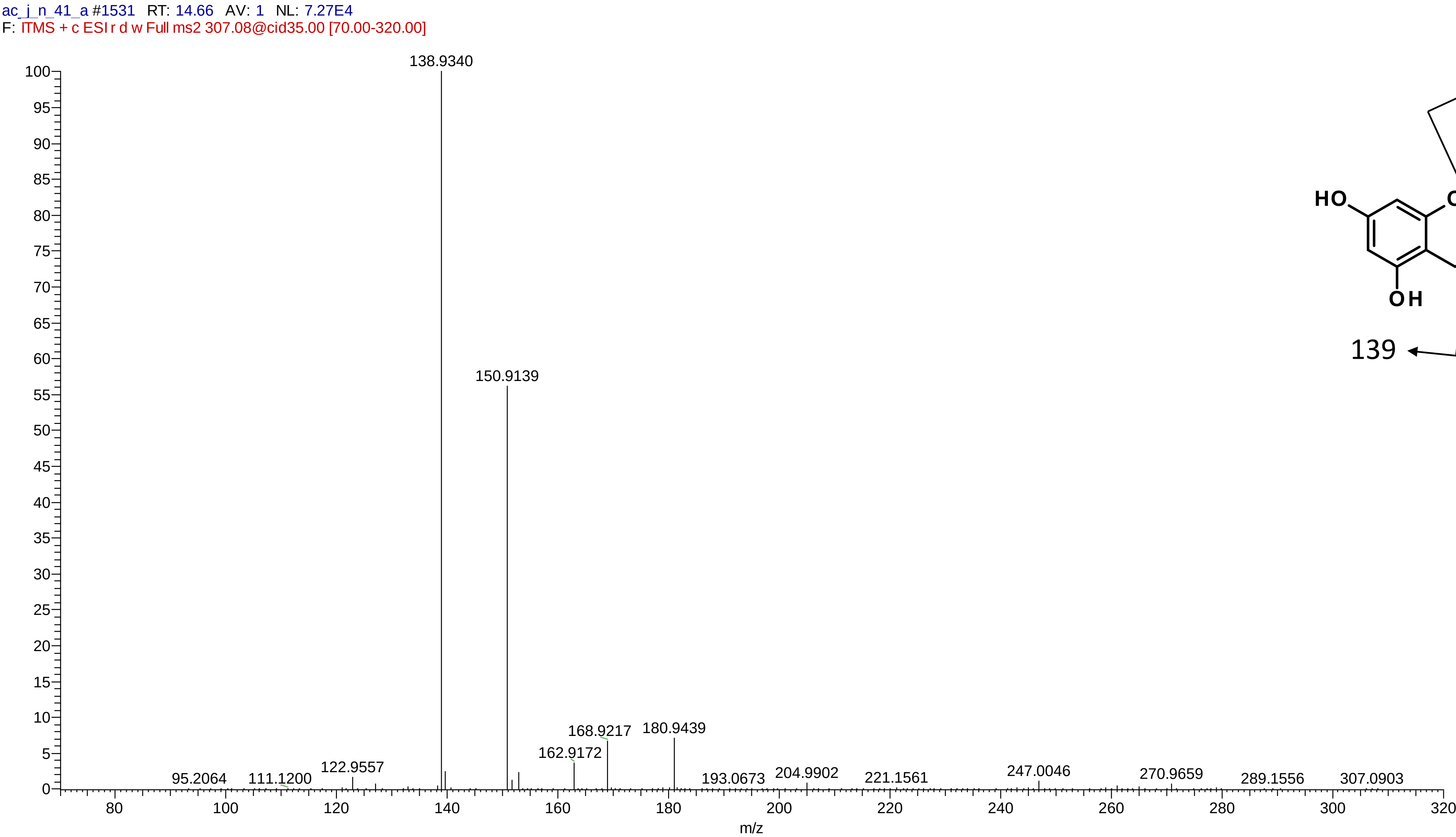

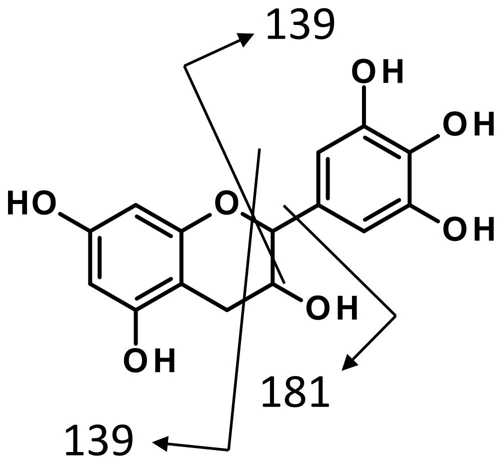

Catechin RT:17.81 min *m/z*:291.0863

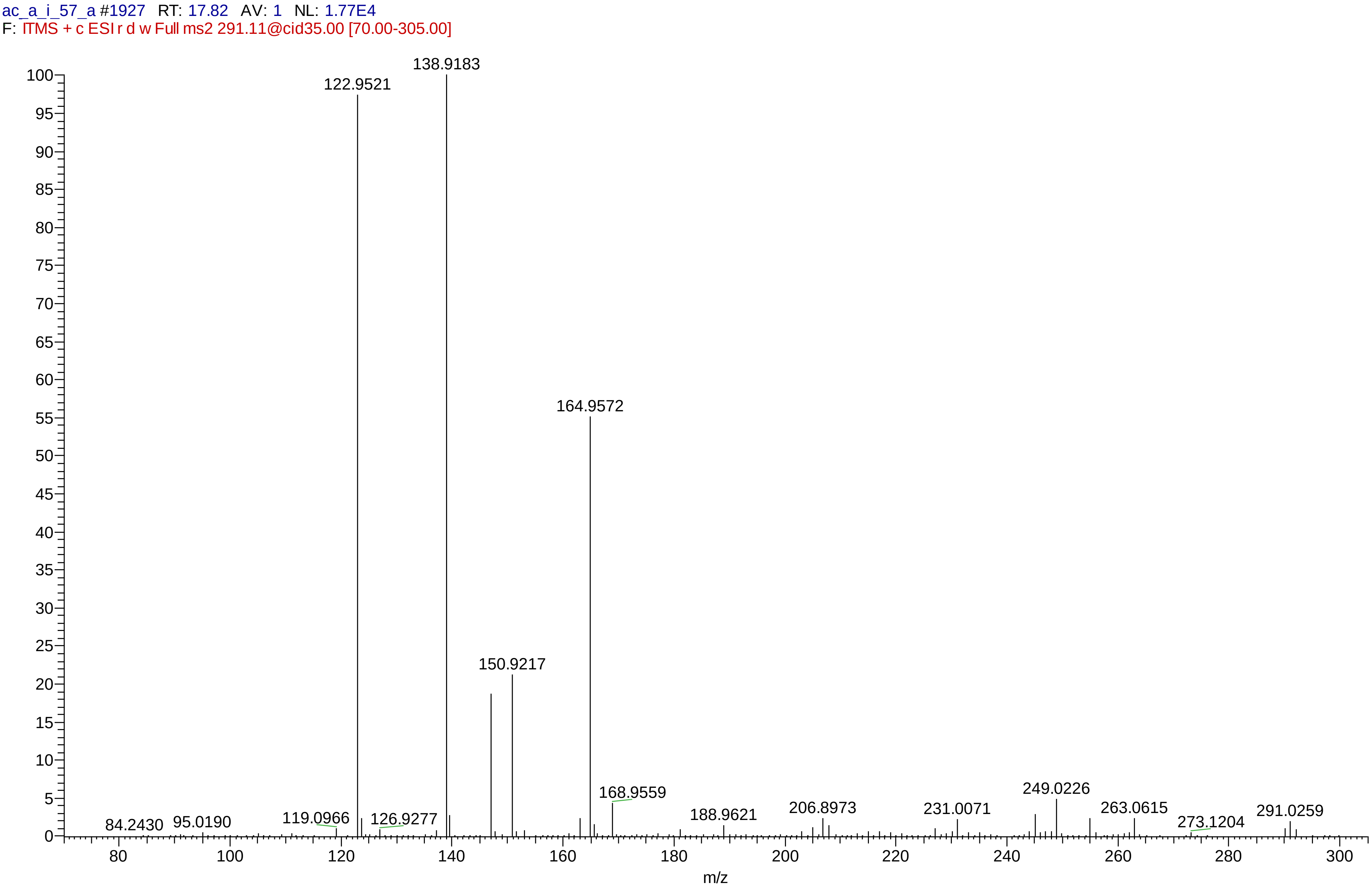

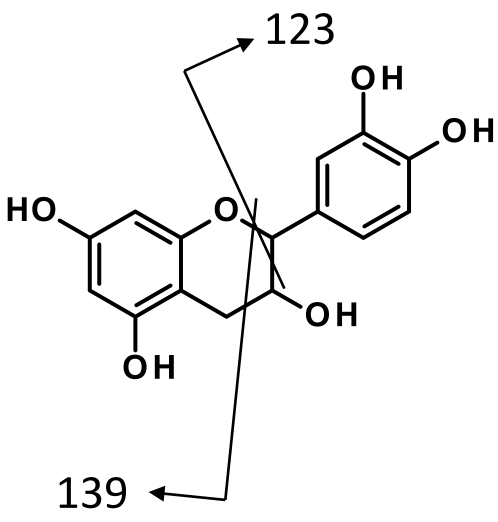

Epicatechin RT:19.47 min *m/z*:291.0863

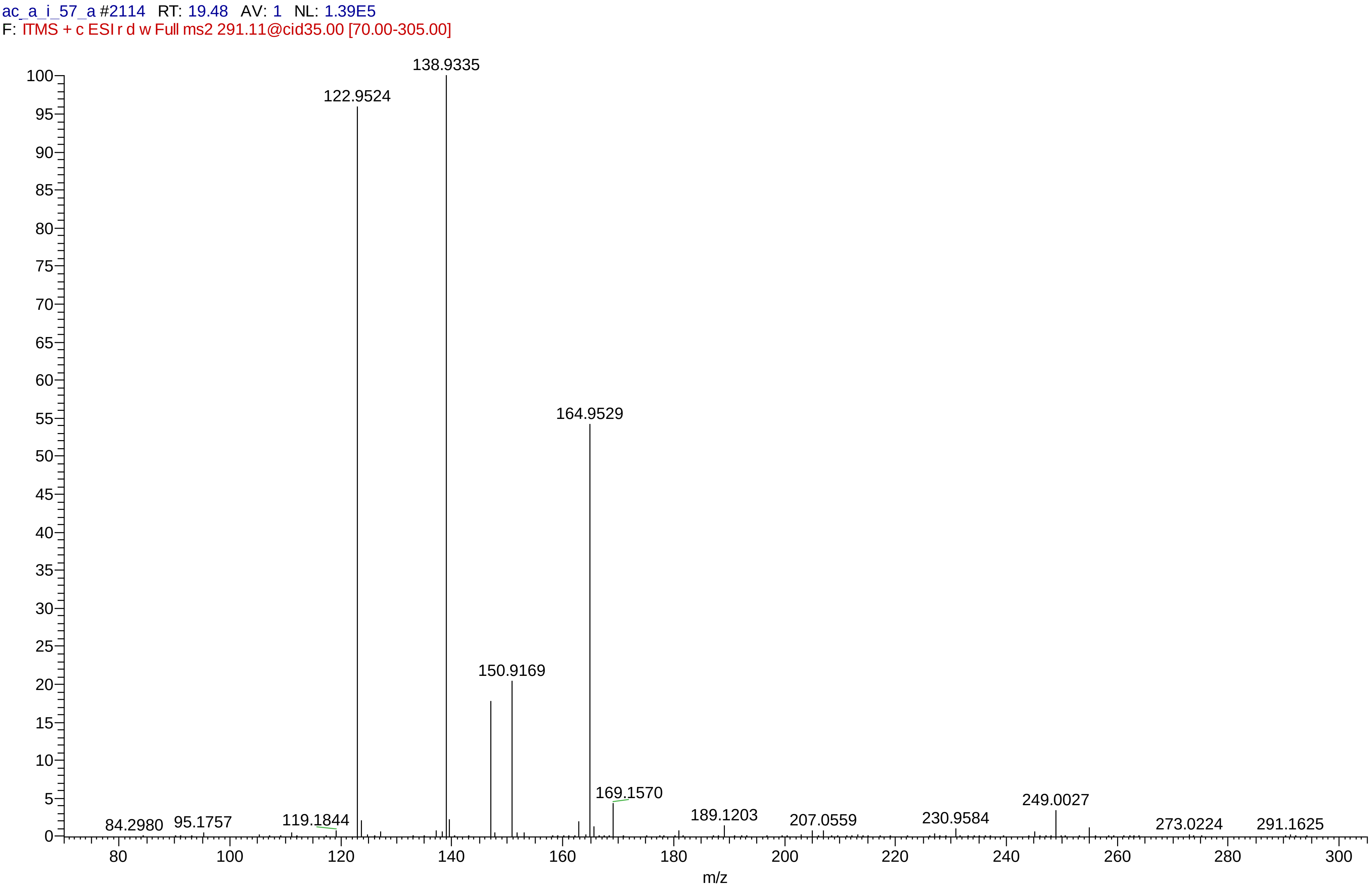

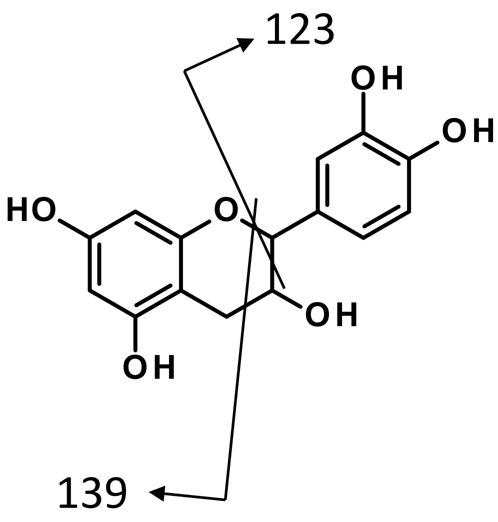

Epicatechin 3'*O*-3-hydroxy-2-methyl propanoate RT:22.23 min *m/z*:377.123

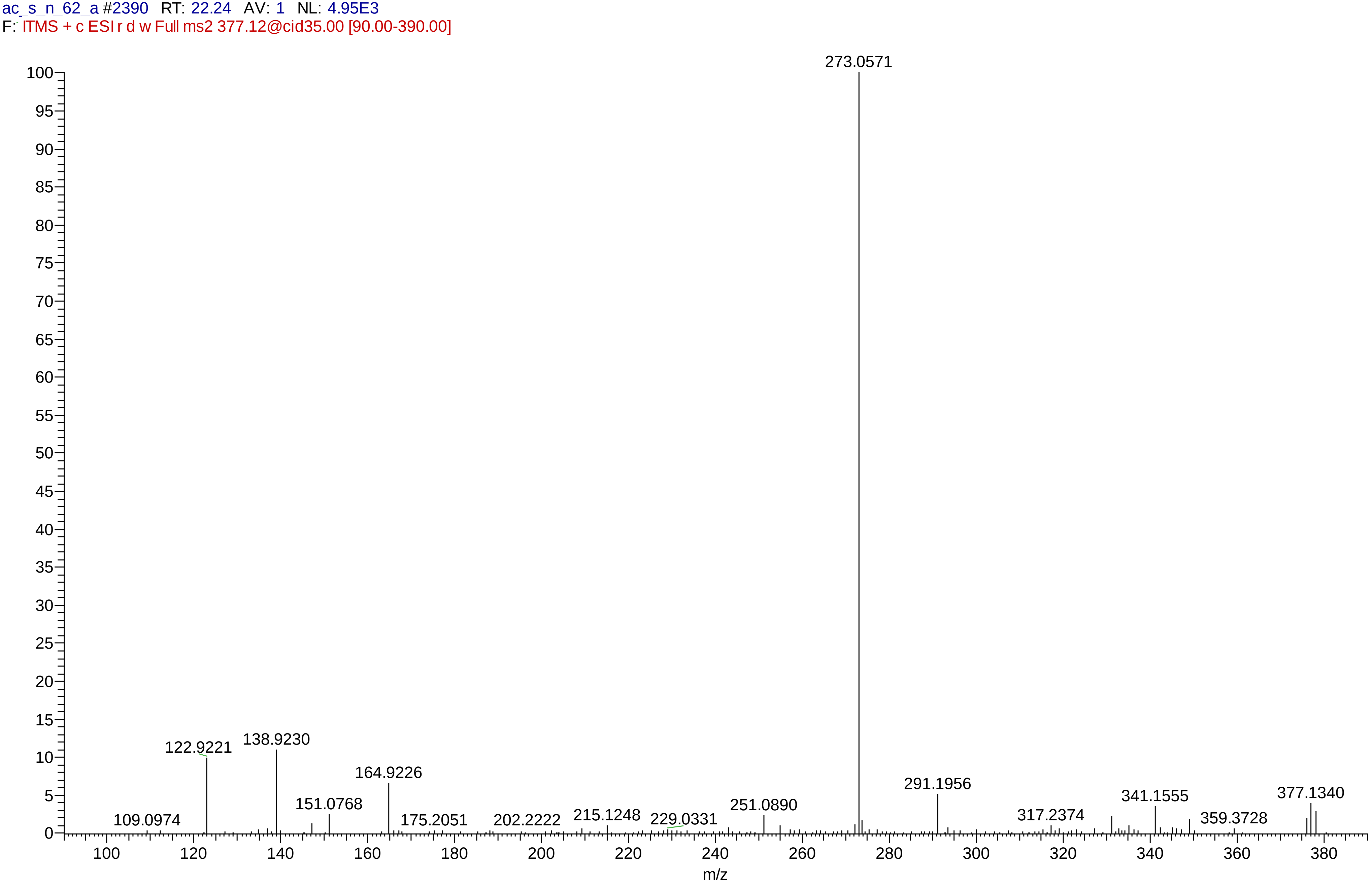

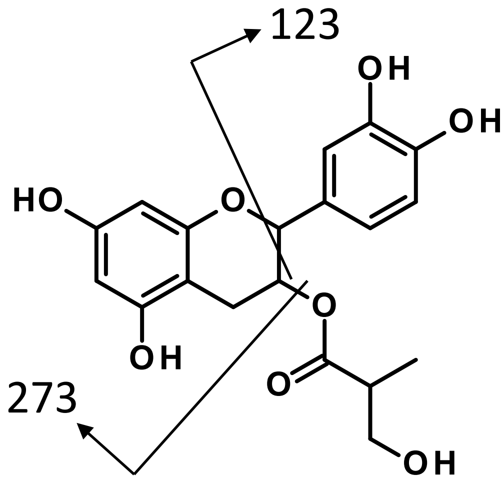

Epigallocatechin gallate RT:19.75 min *m/z*:459.0922

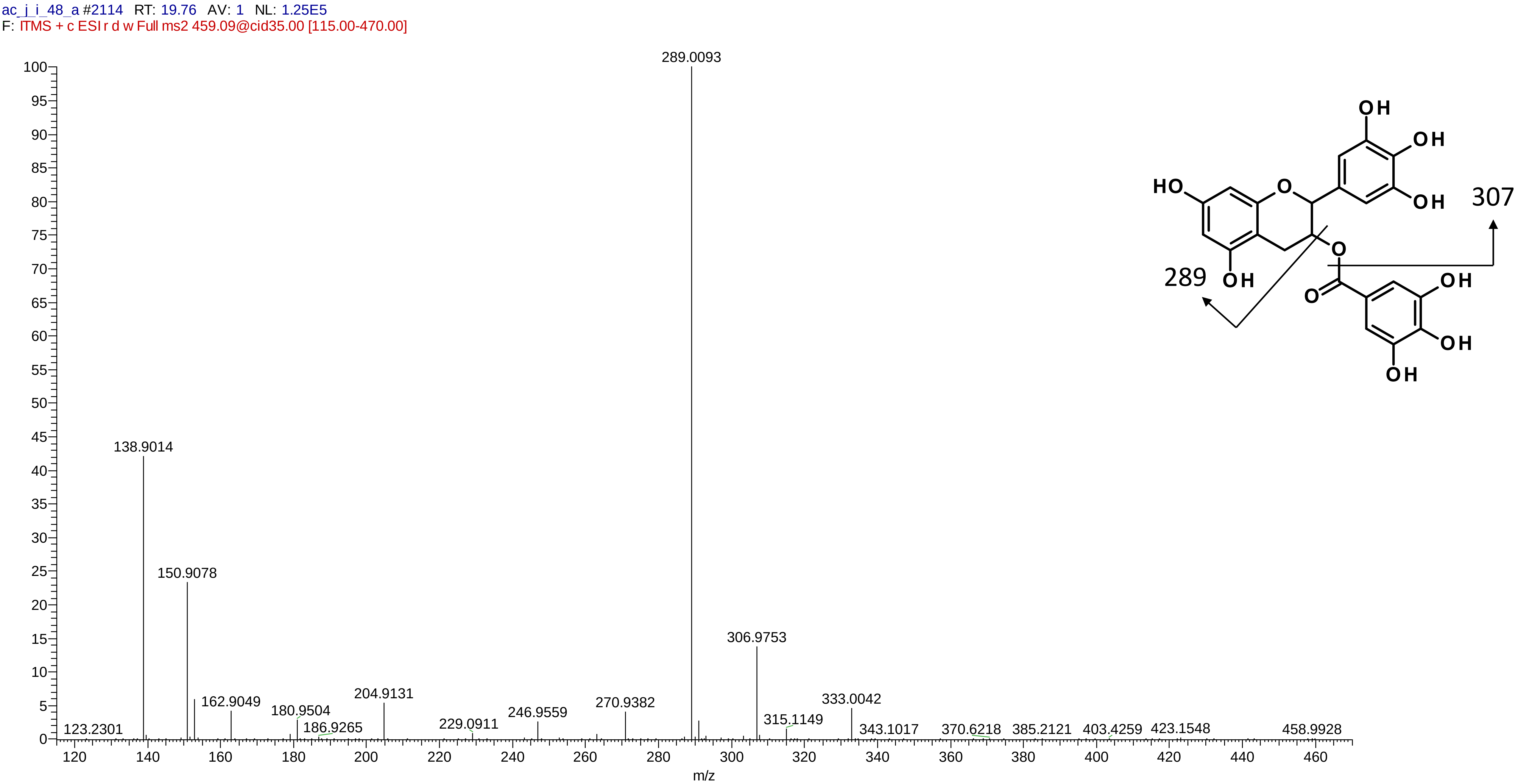

Quercetin RT:20.24 min *m/z*:303.0499

pk_a_n_13_a #2312 RT: 20.25 AV: 1 NL: 9.13E4
F: ITMS + c ESI r d w Full ms2 303.22@cid35.00 [70.00-315.00]

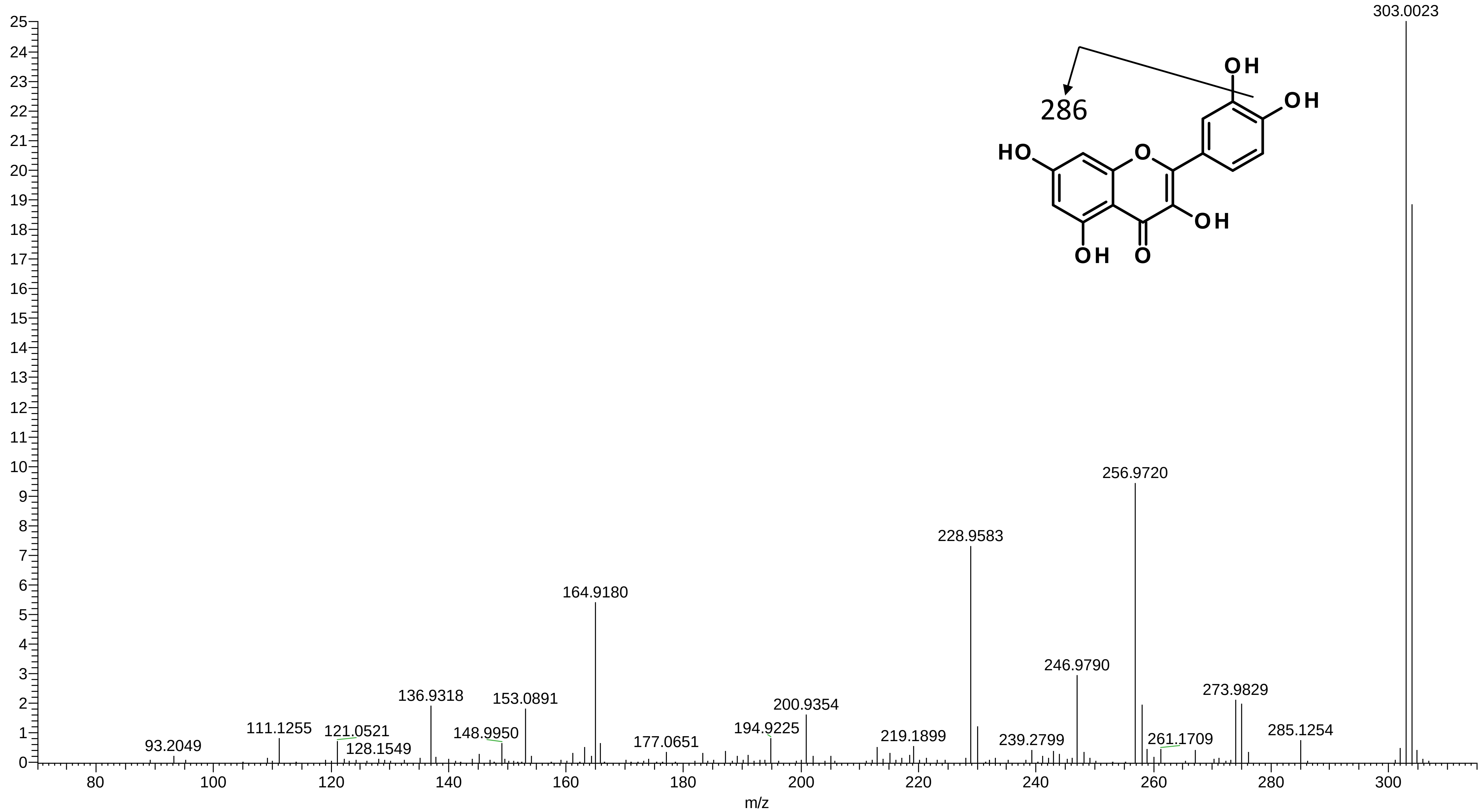

Isoquercetin RT:20.20 min *m/z*:465.1028

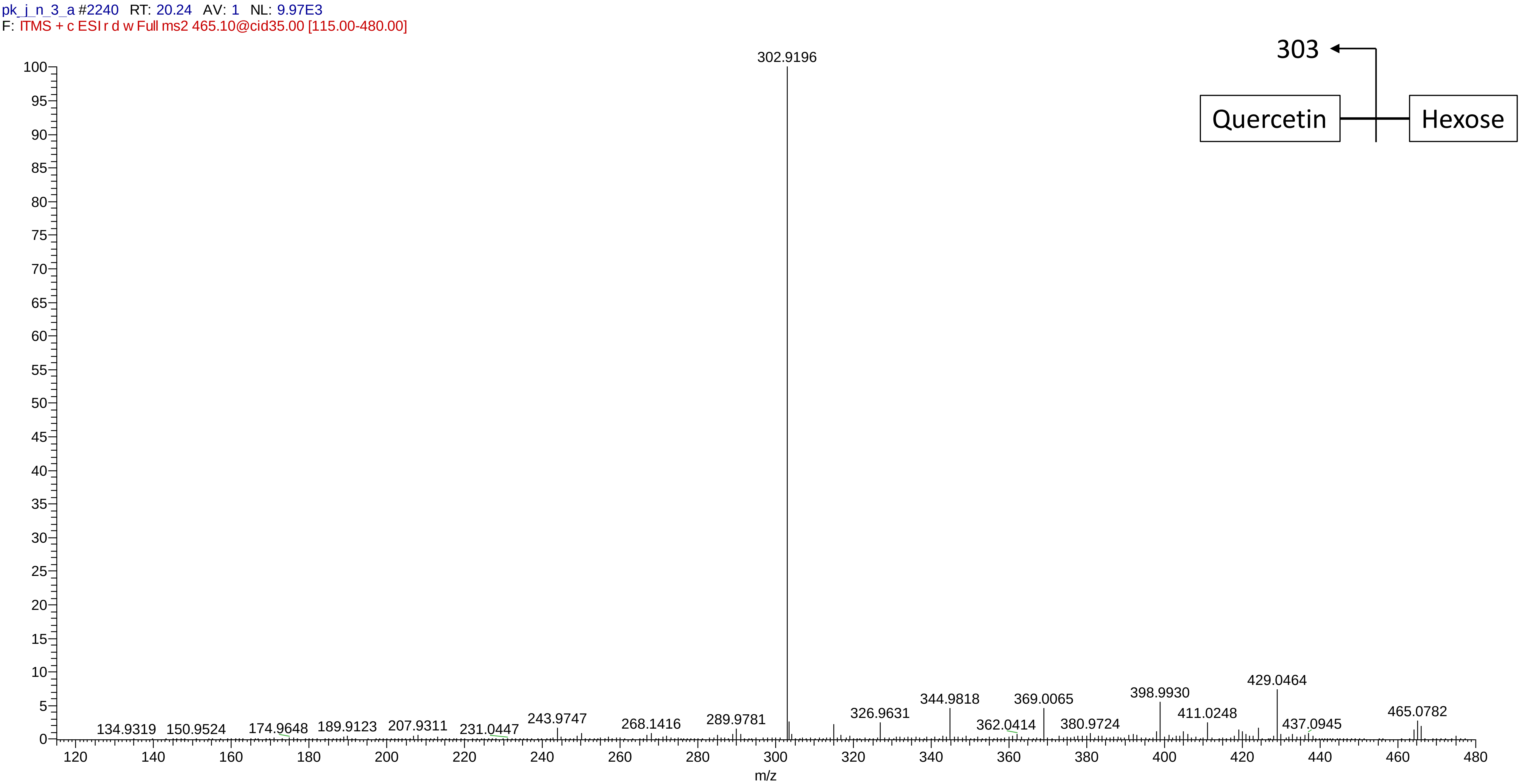

Quercetin-dirhamnopyranoside-hexoside RT:20.20 min *m/z*:757.2186

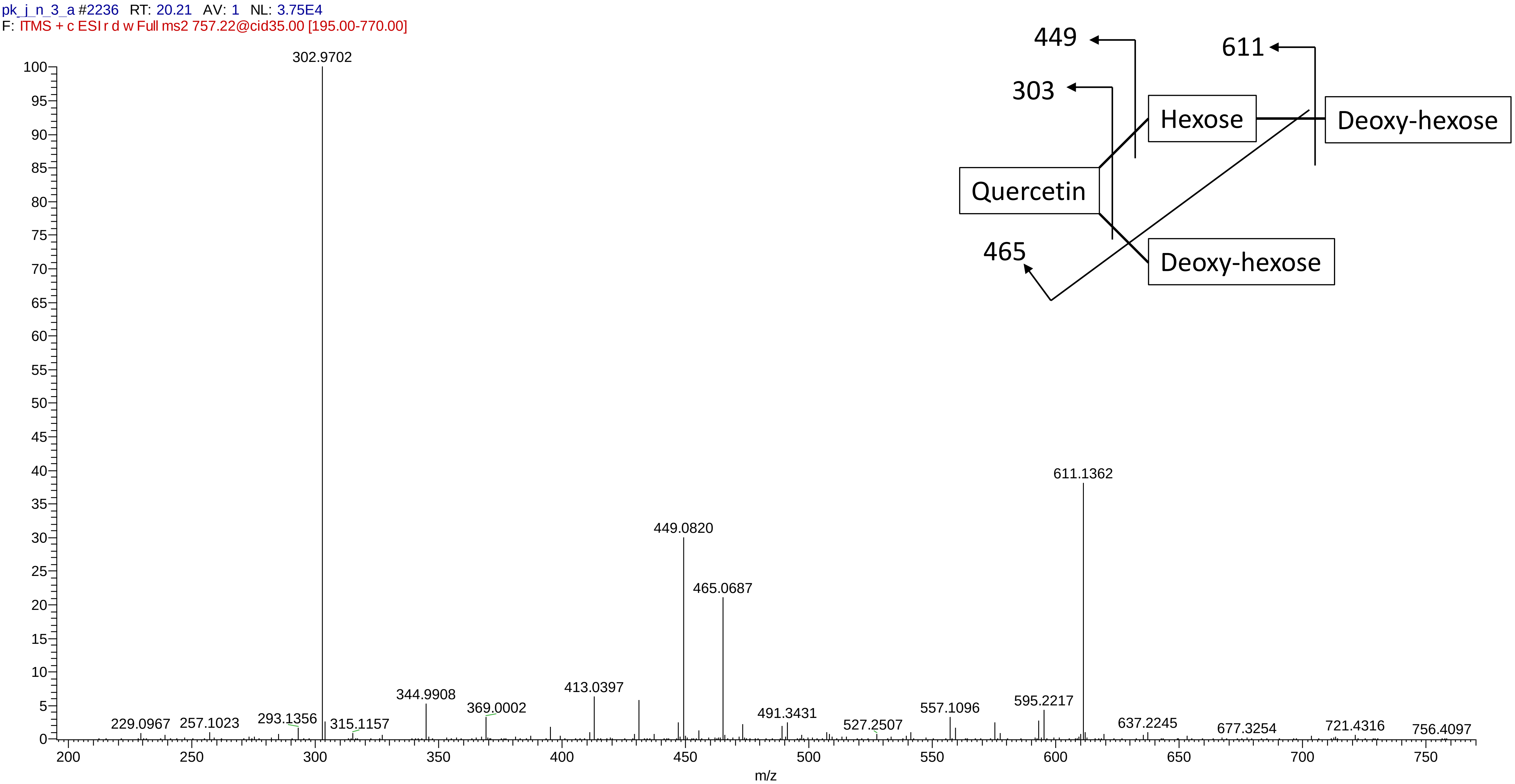

Quercetin RT:20.72 min *m/z*:303.0499

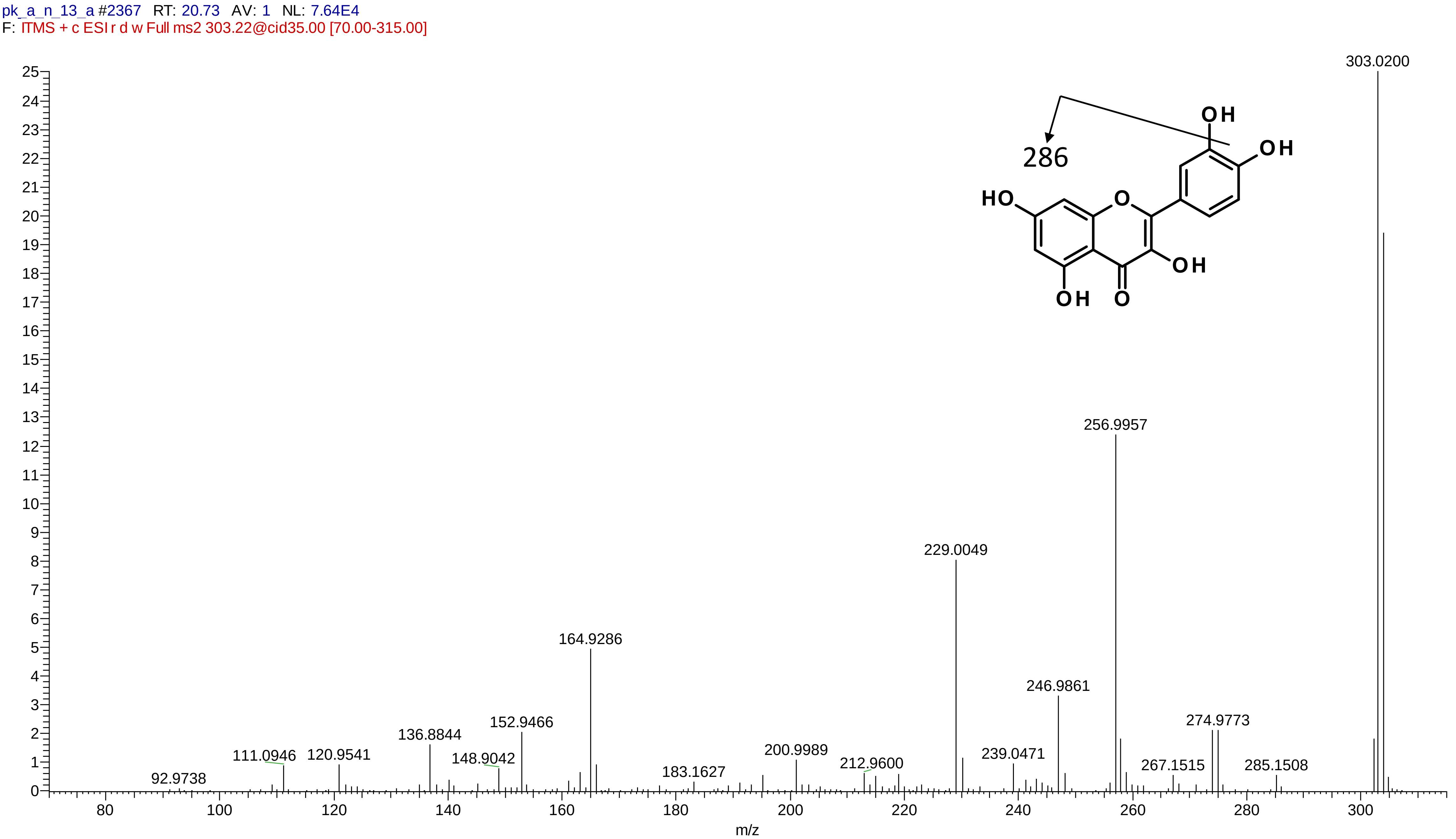

Isoquercetin RT:20.71 min *m/z*:465.1028

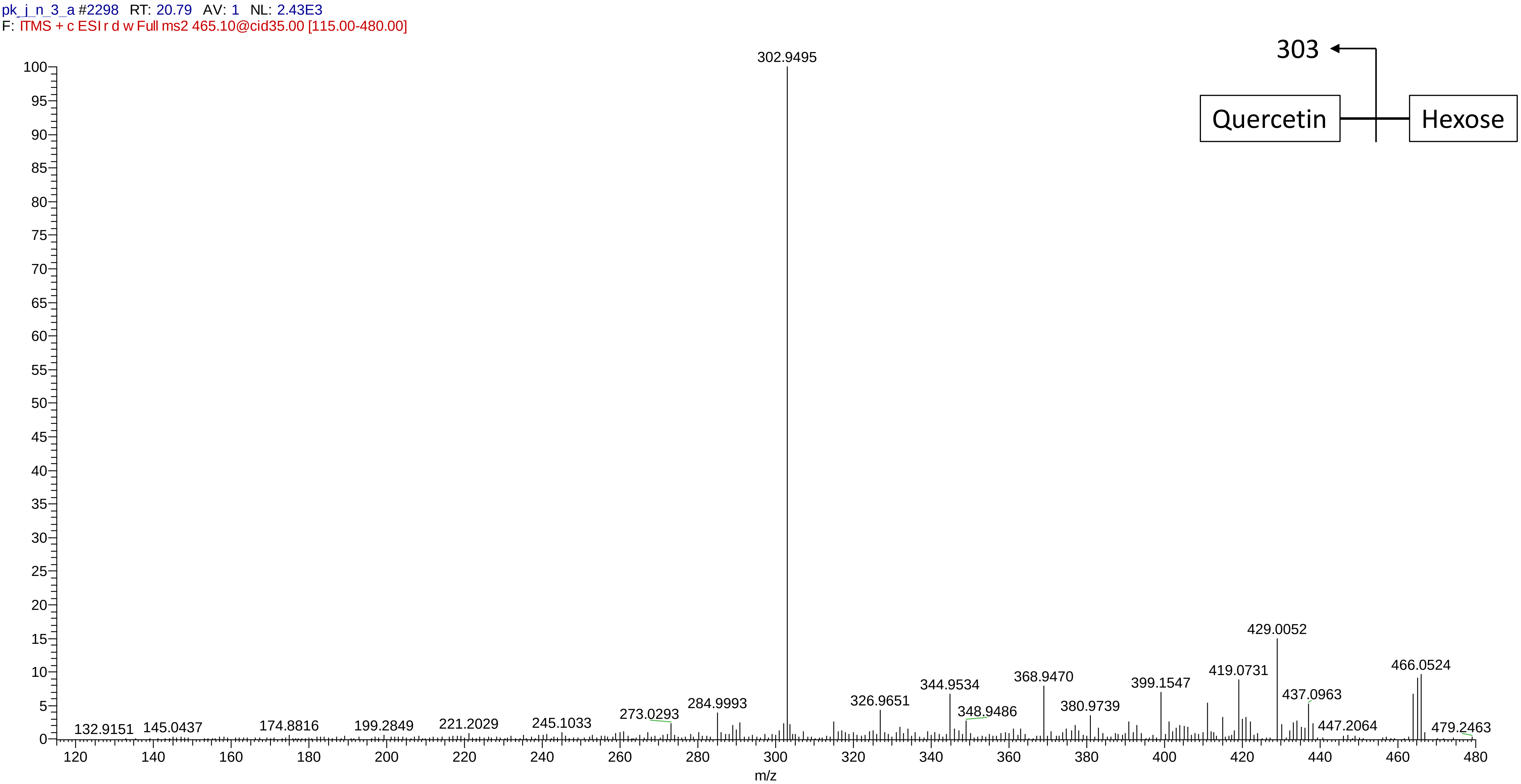

Quercetin rha hex pent RT:20.71 min *m/z*:743.2029

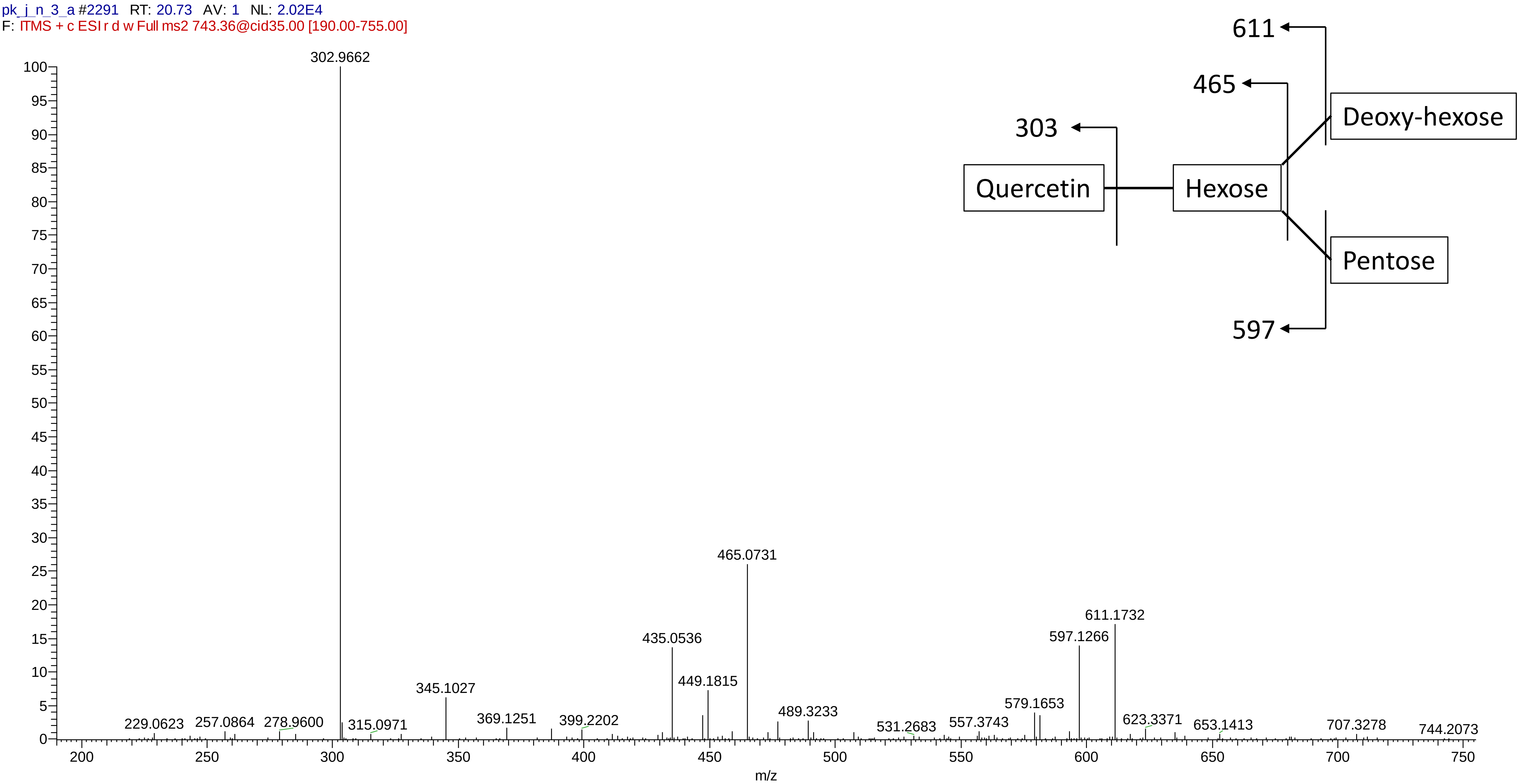

Kaempferol RT:21.11 min *m/z*:287.0550

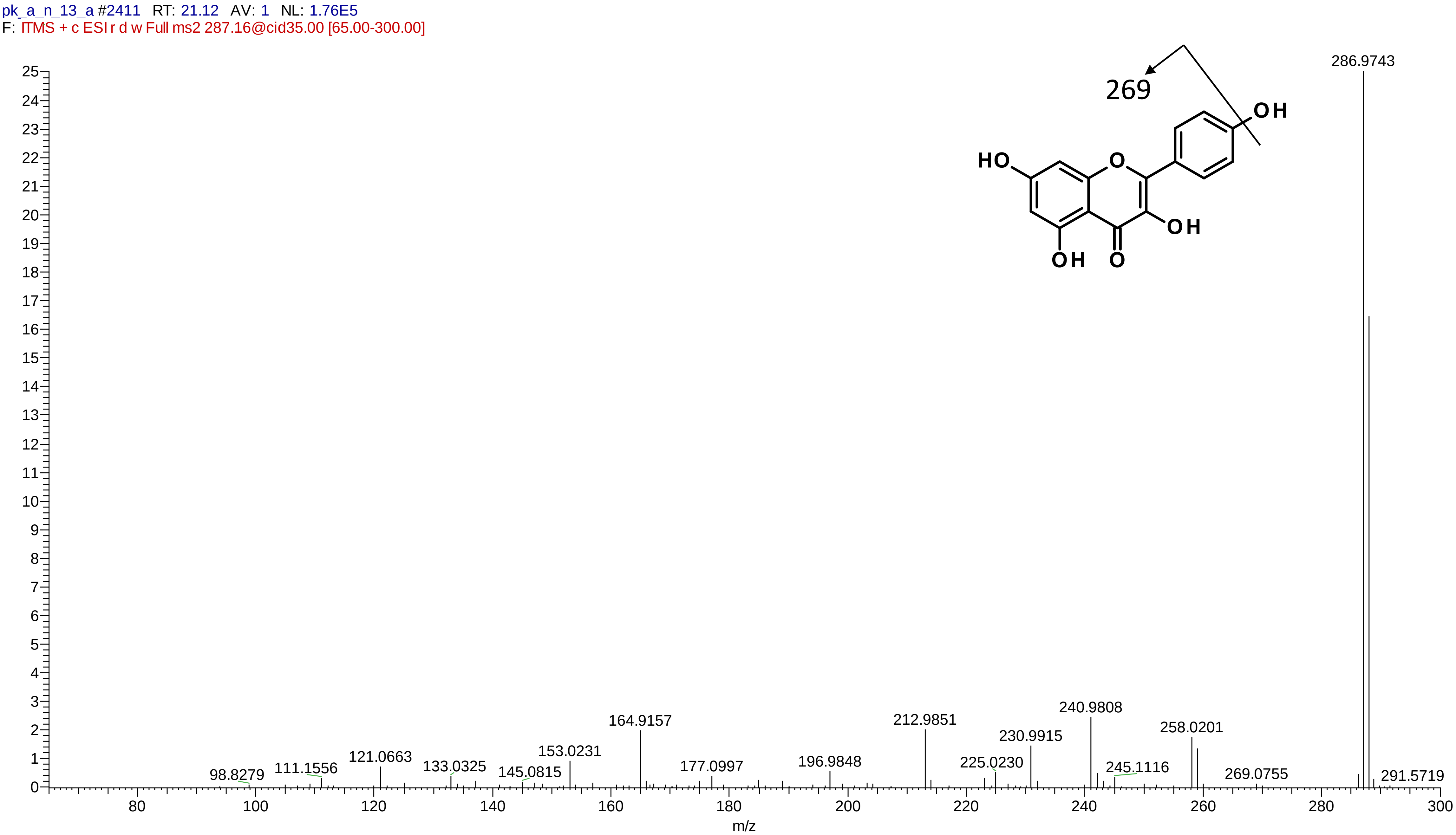

Kaempferol *O*-glucoside RT:21.11 min *m/z*:449.1078

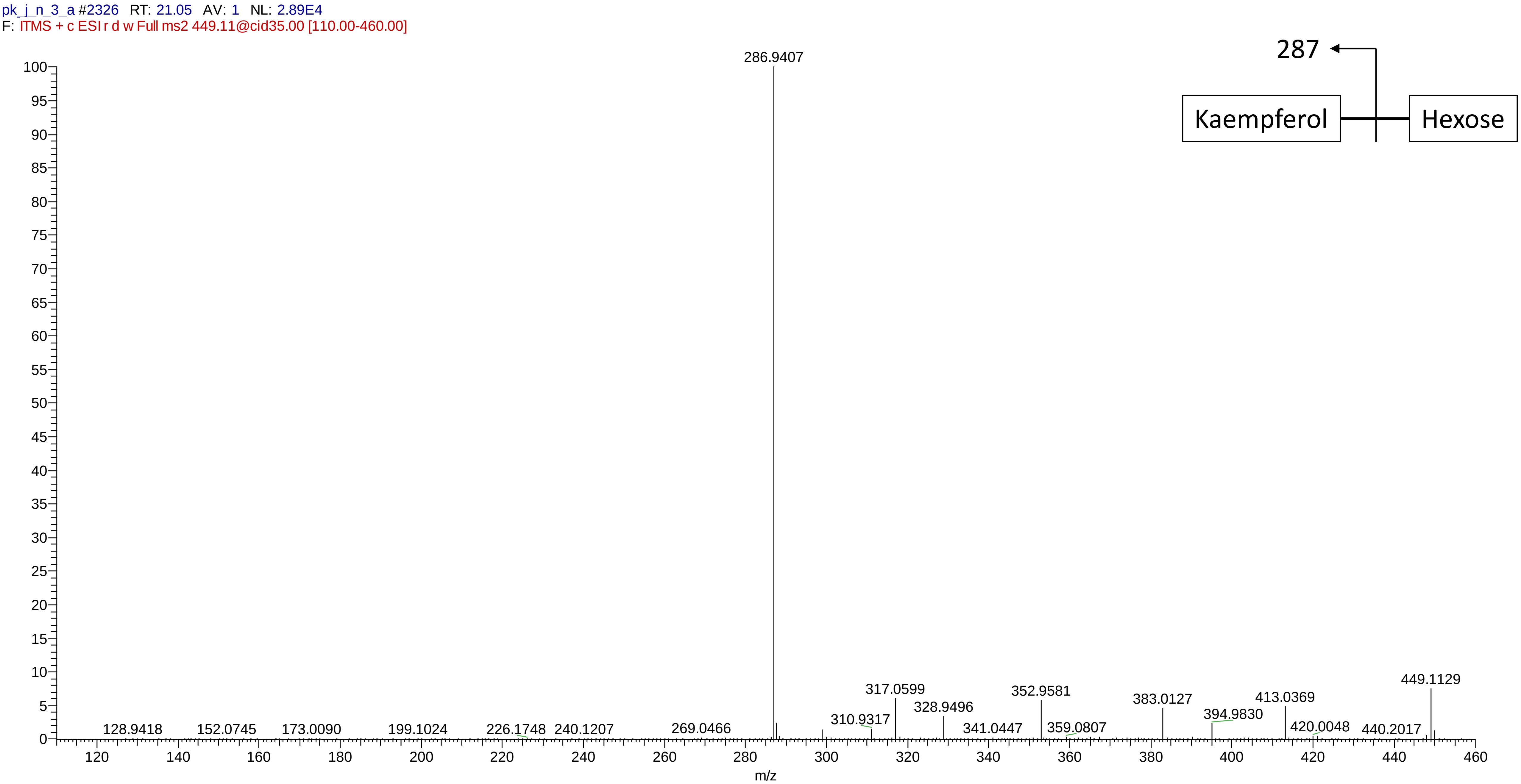

Kaempferol *O*-rutinoside RT:21.02 min *m/z*:595.1658

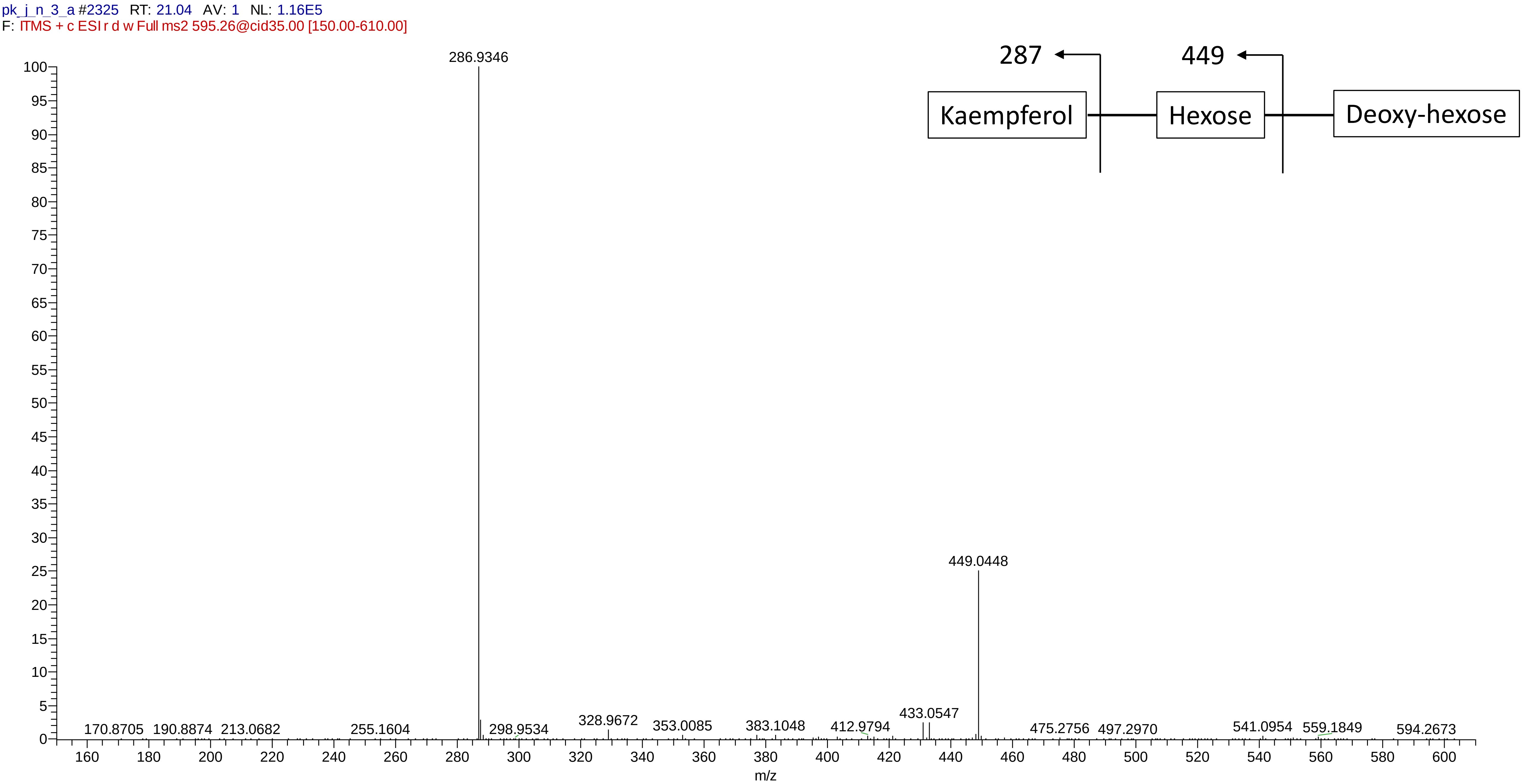

Robinin or isomer RT:21.11 min *m/z*:741.2237

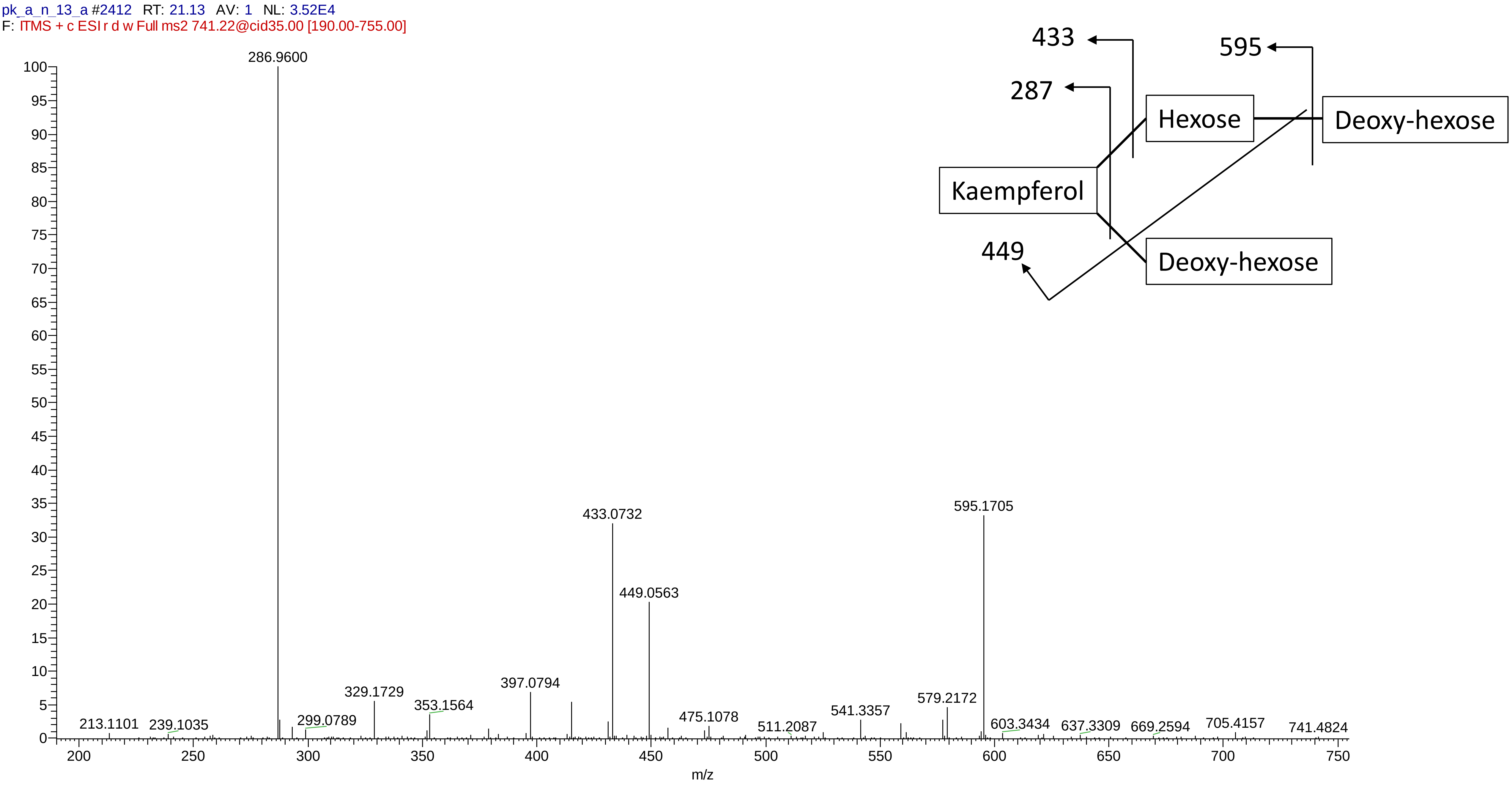

Kaempferol *O*-glucoside RT:21.65 min *m/z*:449.1078

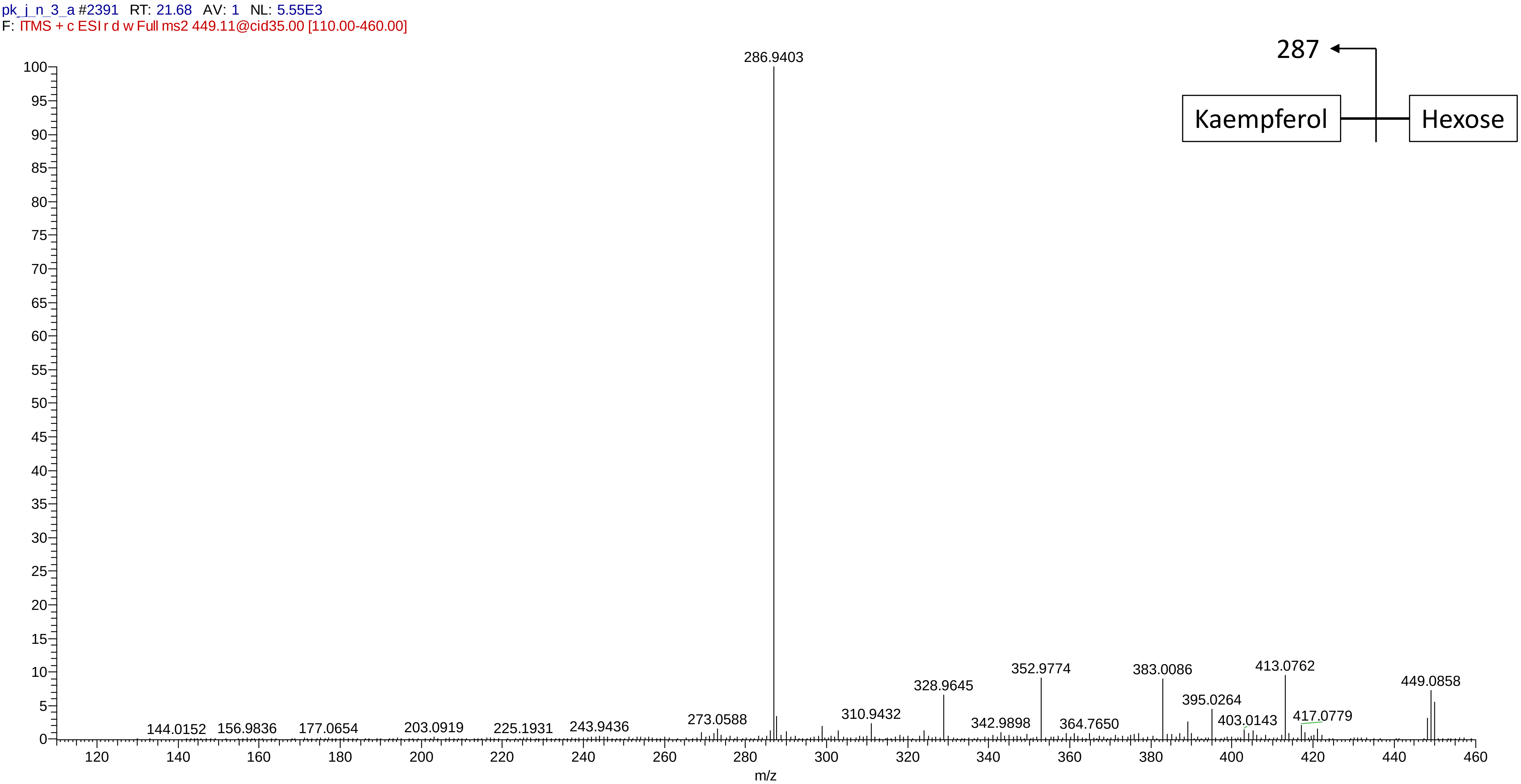

Kaempferol *O*-rutinoside RT:21.65 min *m/z*:595.1658

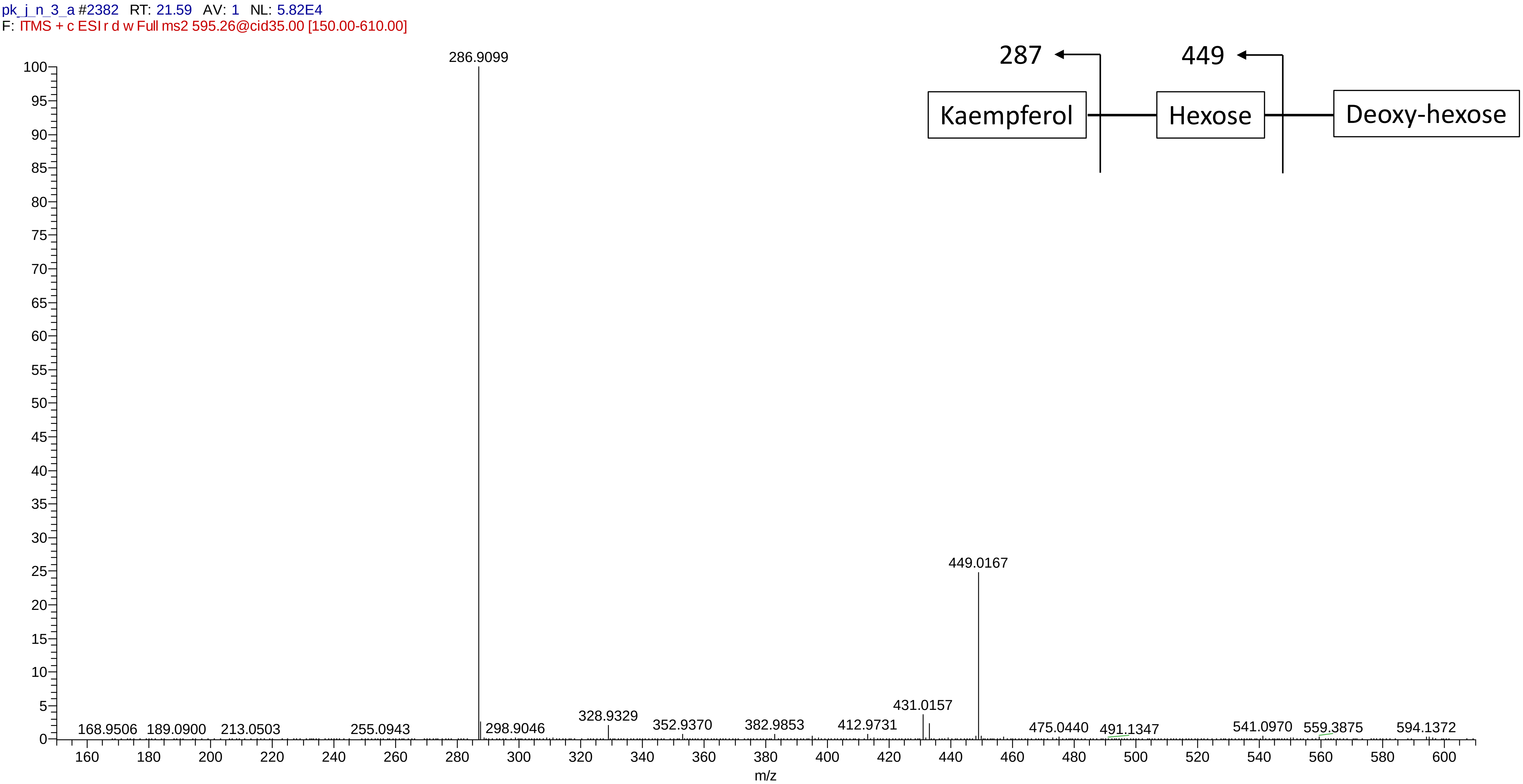

Kaempferol dihexoside RT:22.01 min *m/z*:611.1607

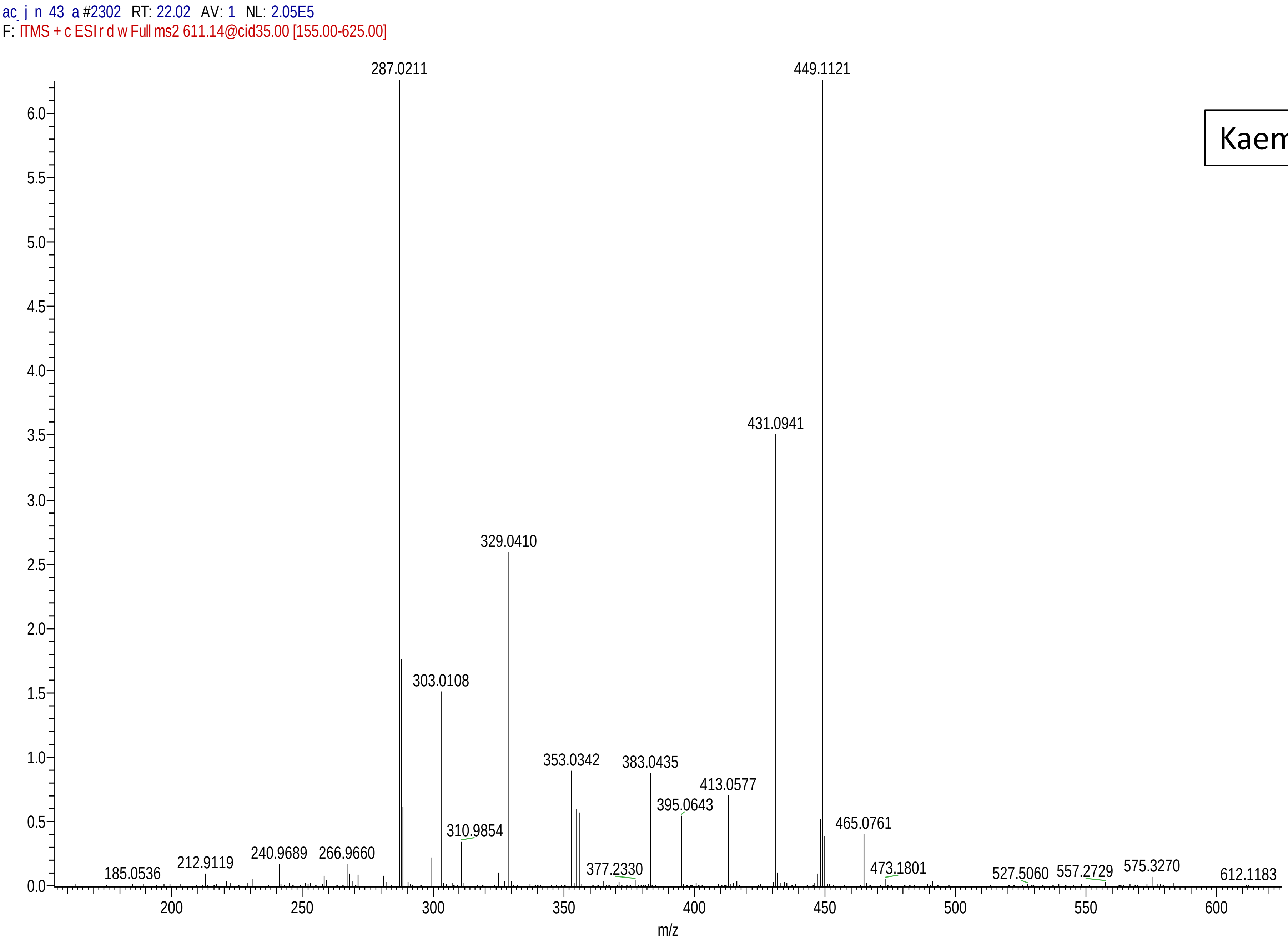

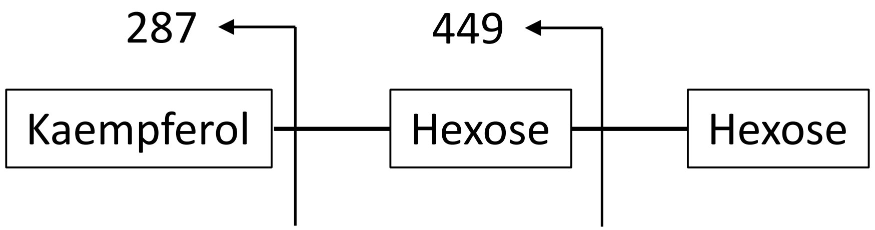

Quercetin RT:22.08 min *m/z*:303.0499

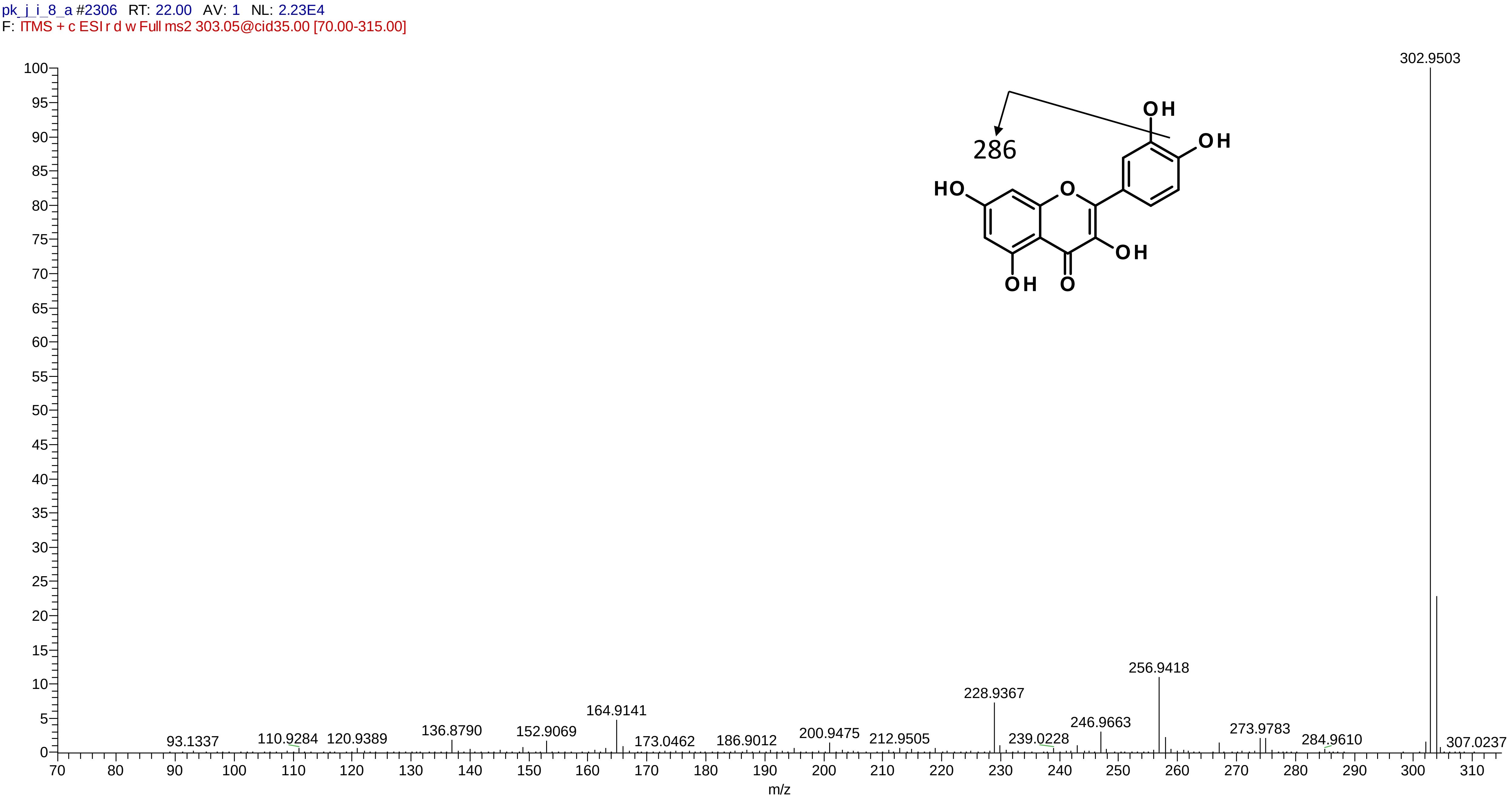

Rutin RT:22.08 min *m/z*:611.1607

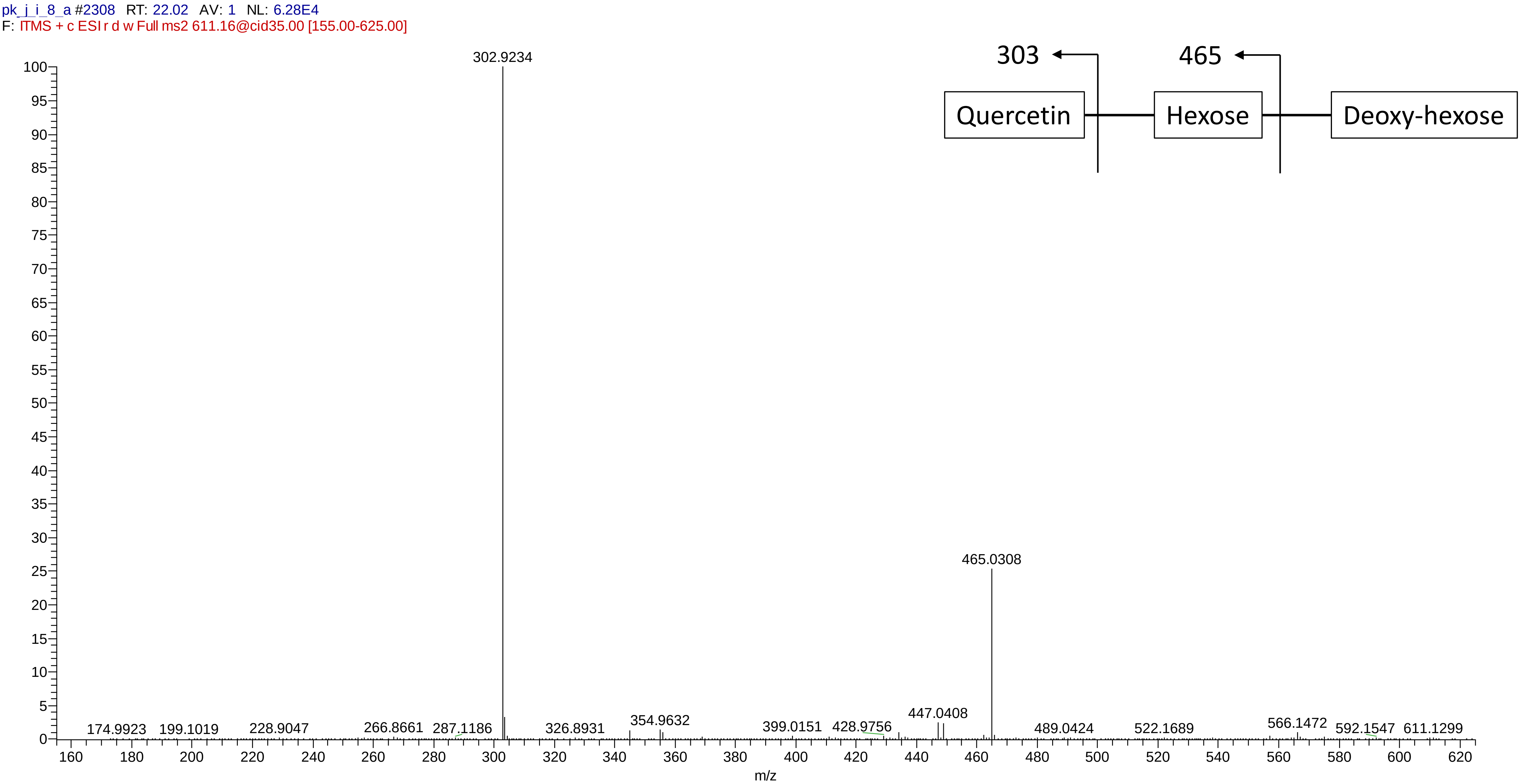

Kaempferol *O*-glucoside RT:22.84 min *m/z*:449.1078

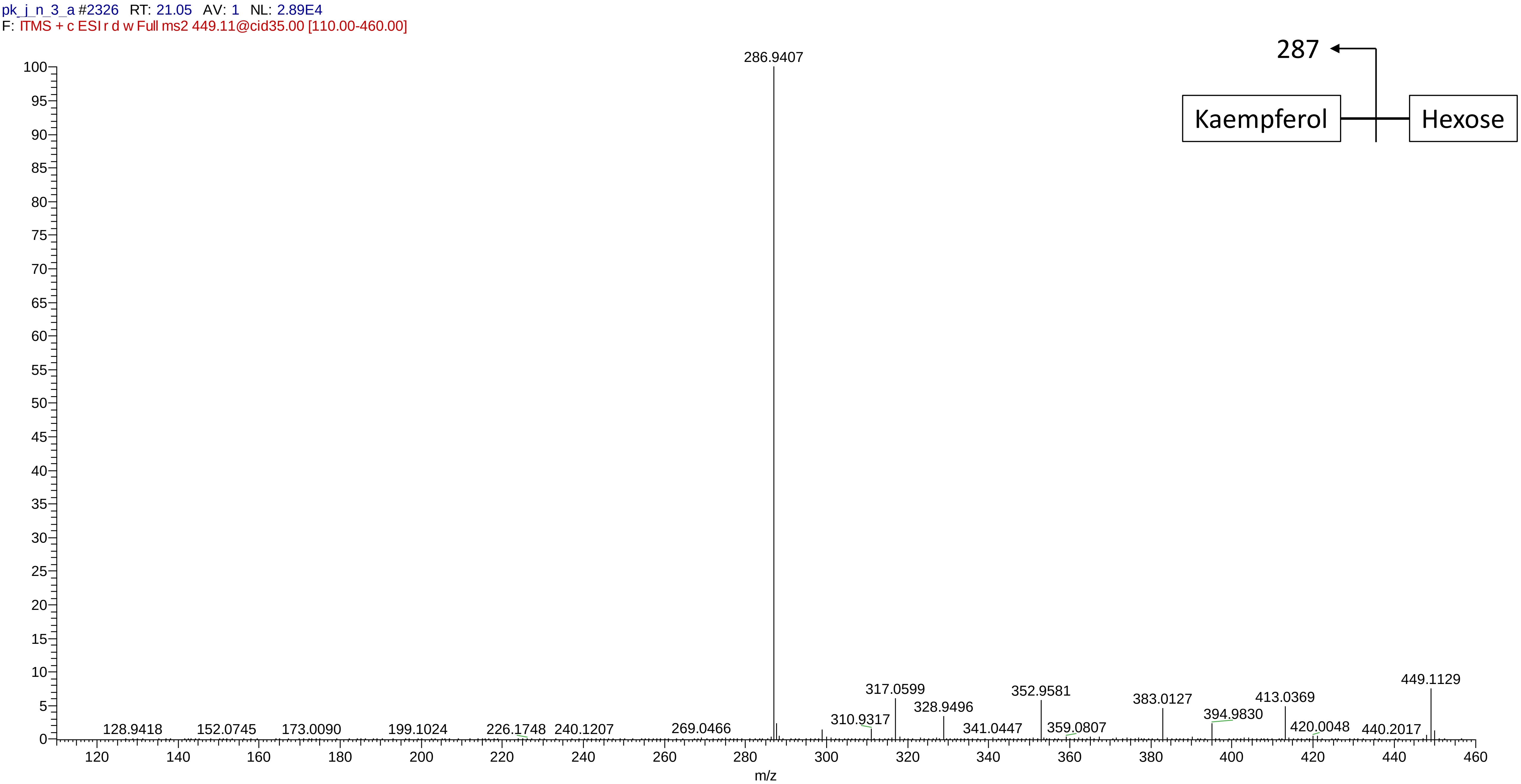

Kaempferol *O*-rutinoside RT:22.84 min *m/z*:595.1658

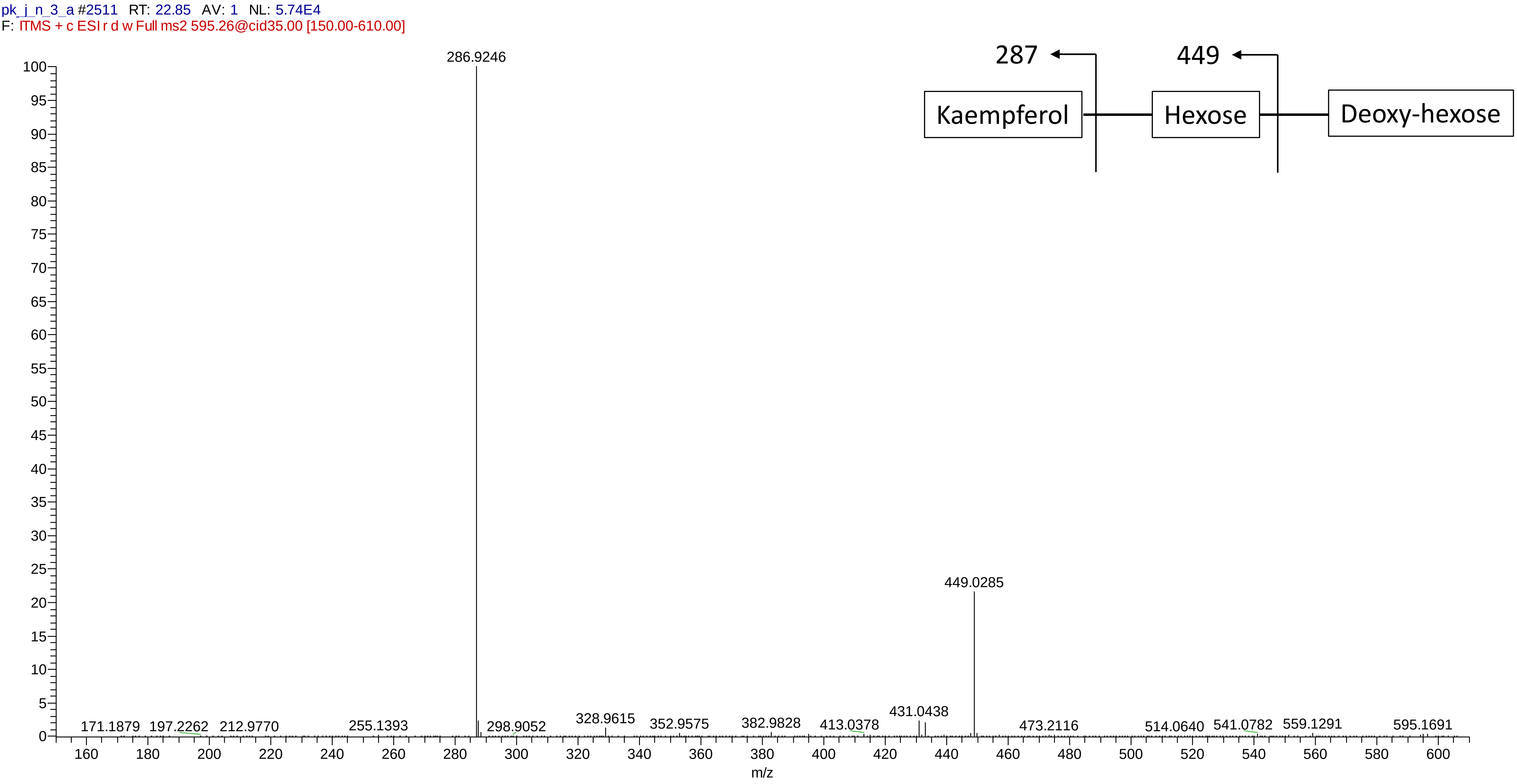

Kaempferol *O*-glucoside RT:23.82 min *m/z*:449.1078

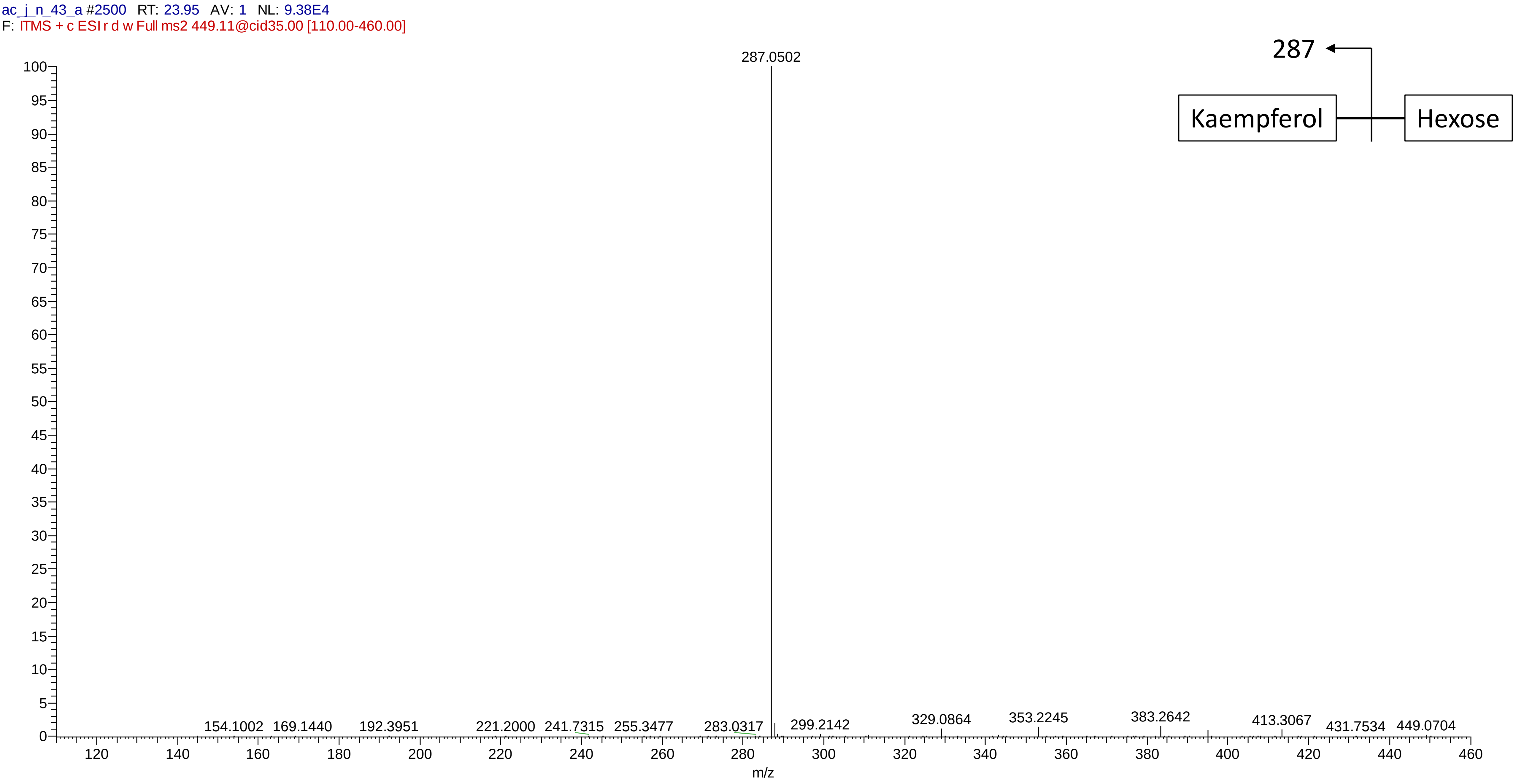

Caffeoylquinic acid RT:15.97 min *m/z*:355.1024

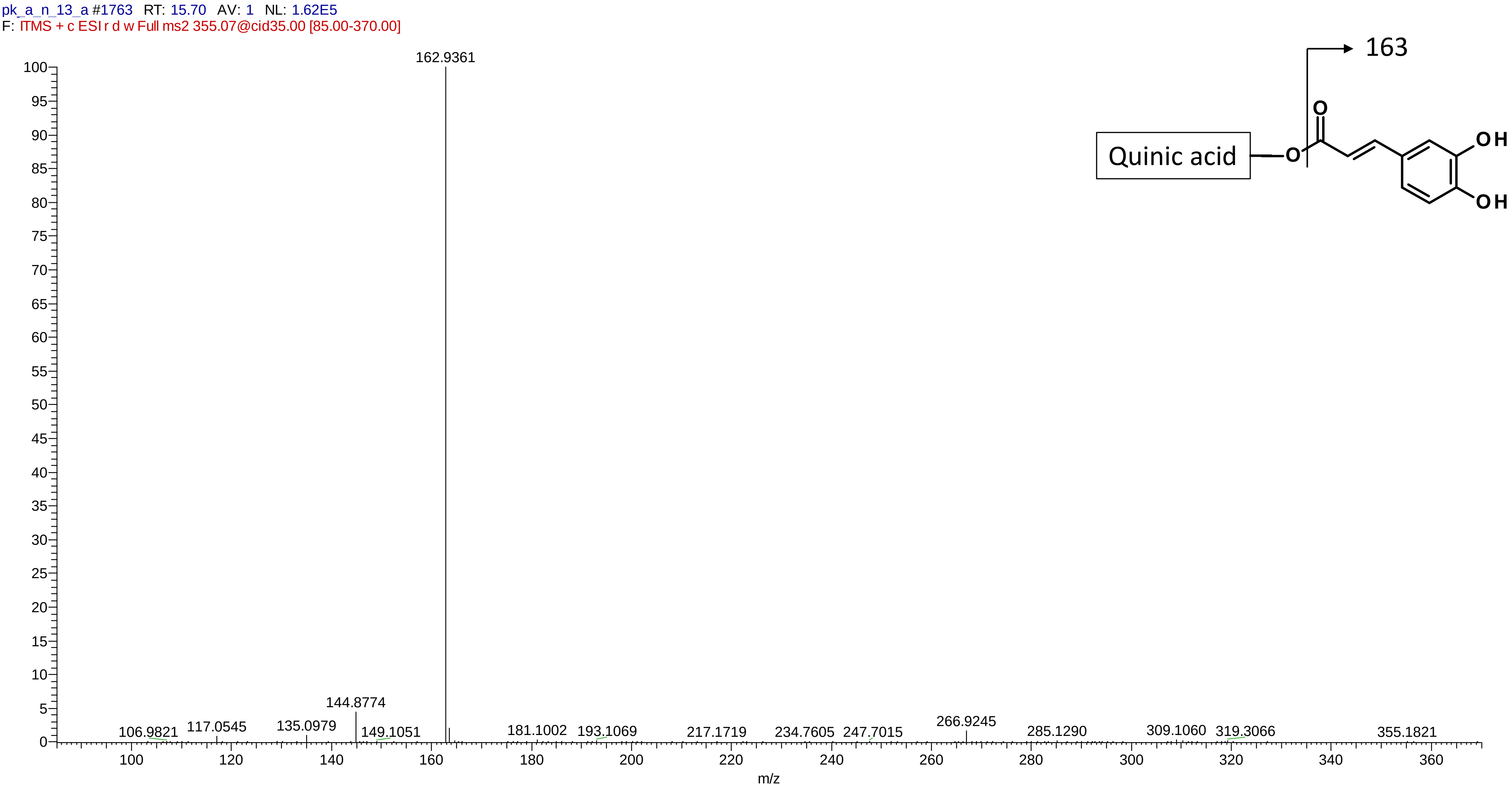

Caffeoylquinic acid RT:17.67 min *m/z*:355.1024

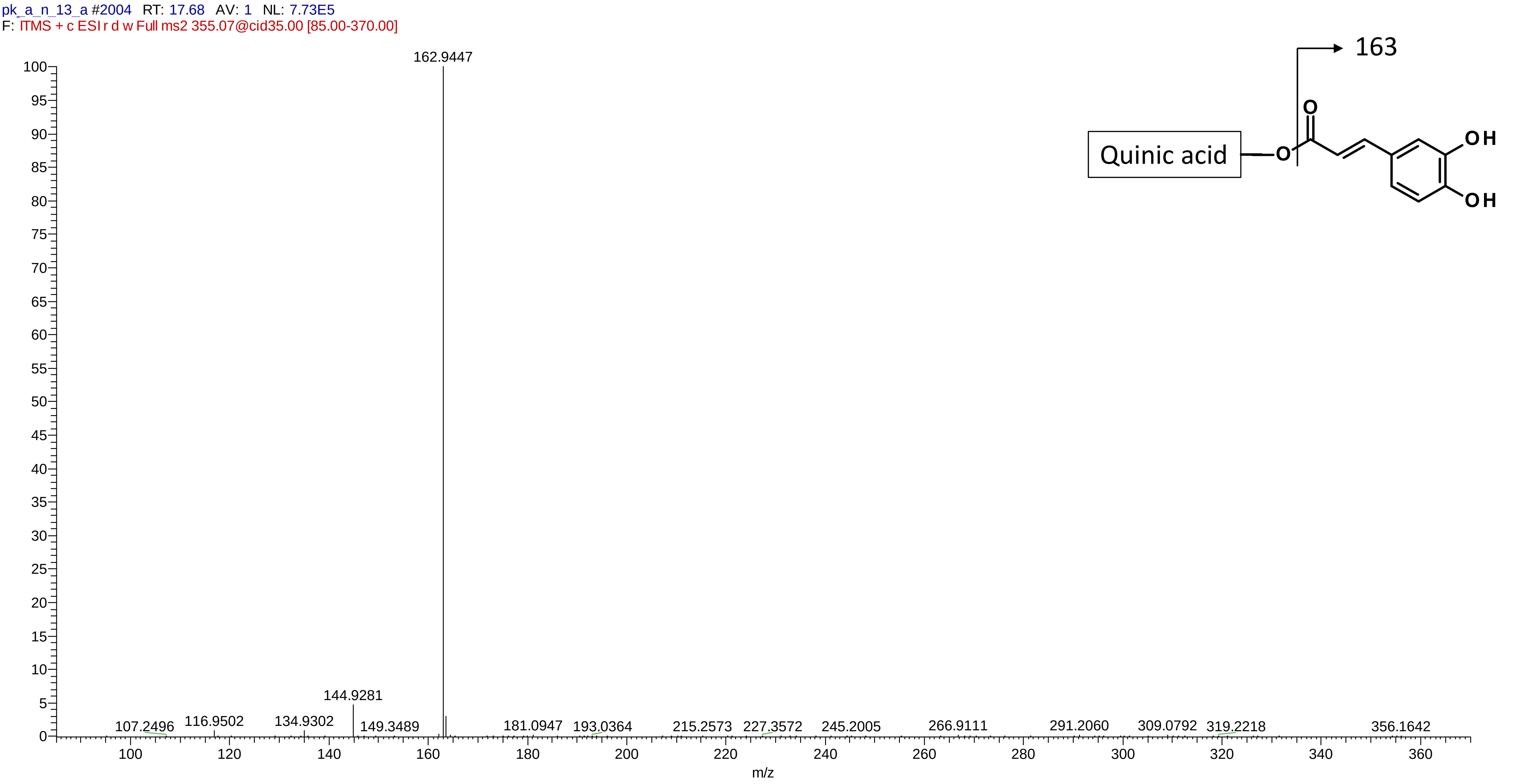

Caffeoylquinic acid RT:18.34 min *m/z*:355.1024

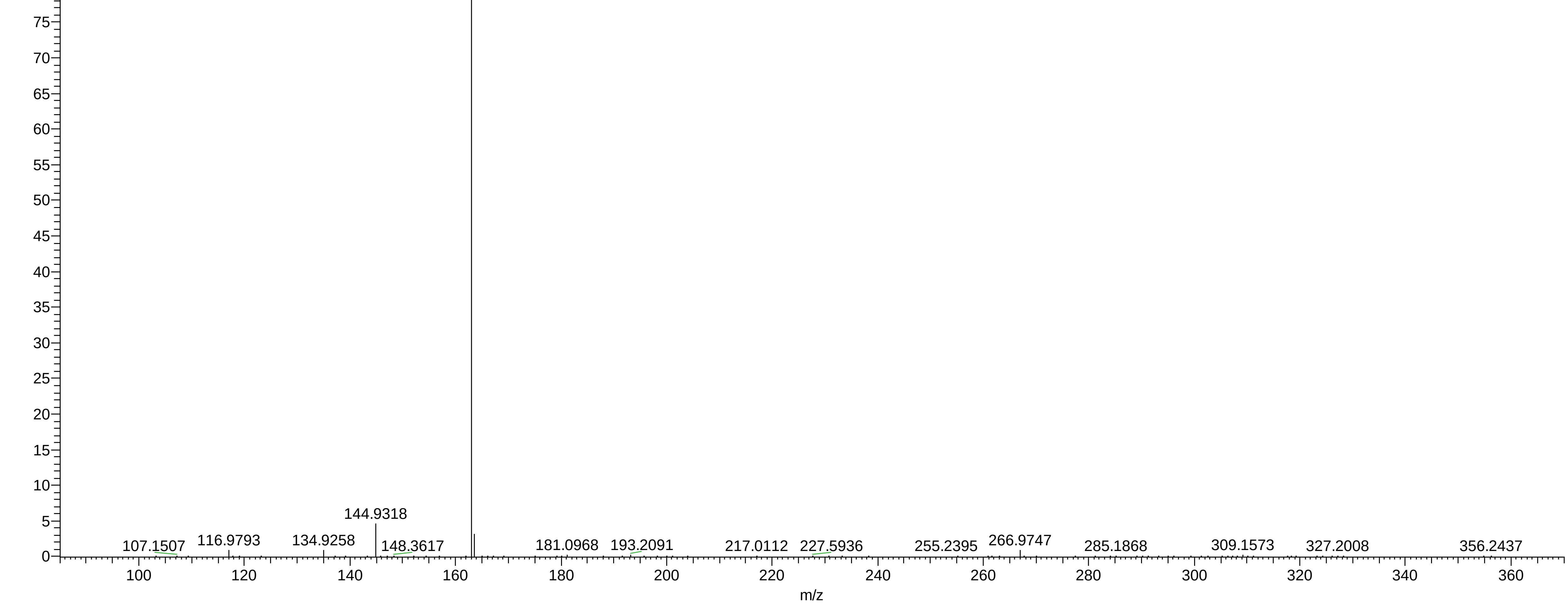

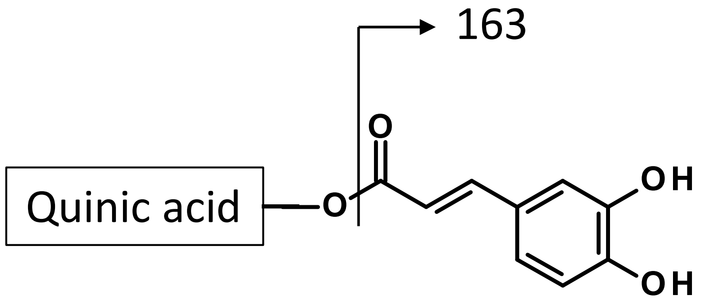

Ascorbic acid RT:3.03 min *m/z*:177.0394

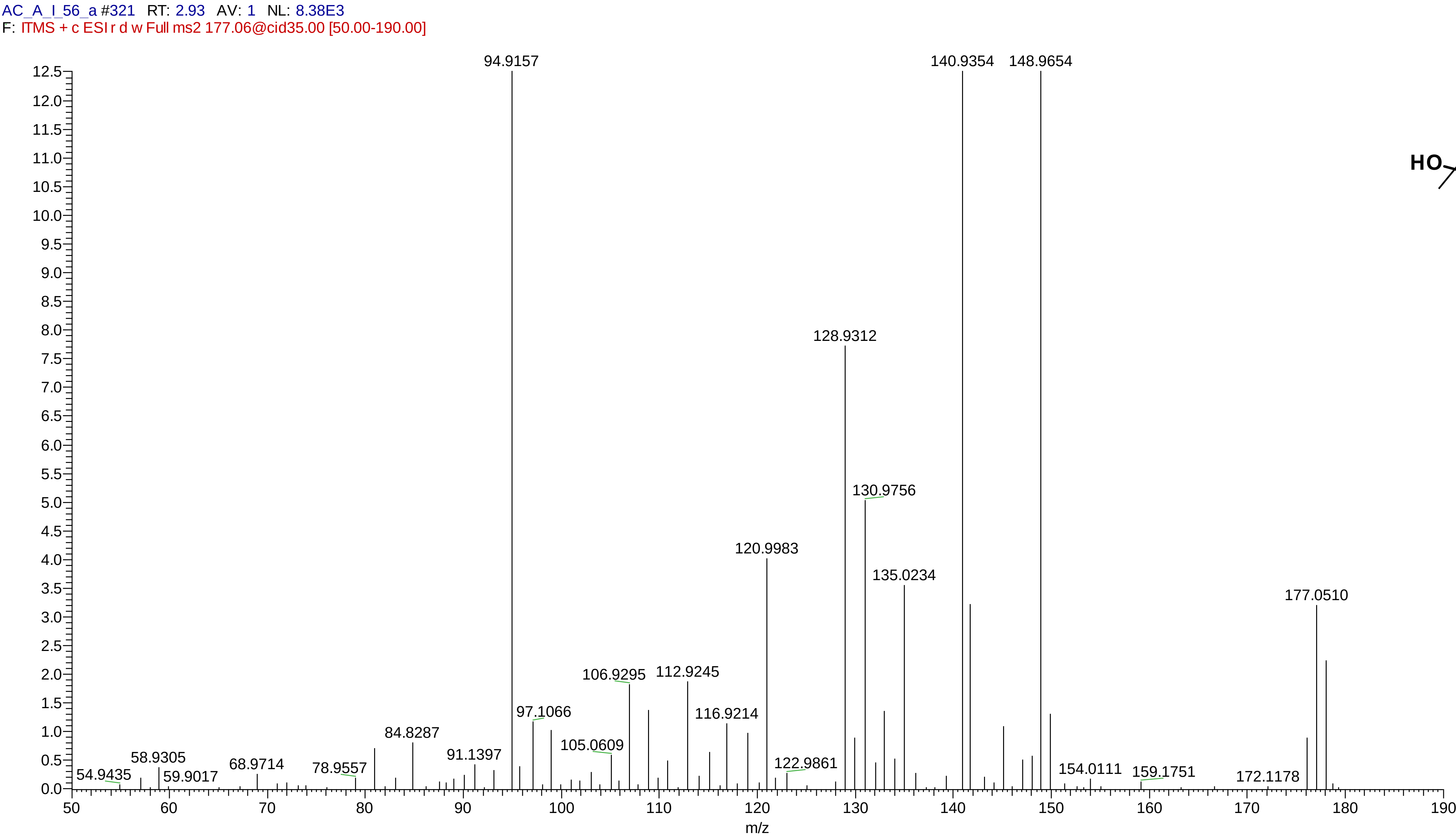

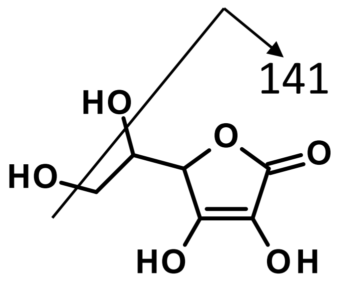

Dehydroascorbic acid hydrate RT:4.05 min *m/z*:193.0342

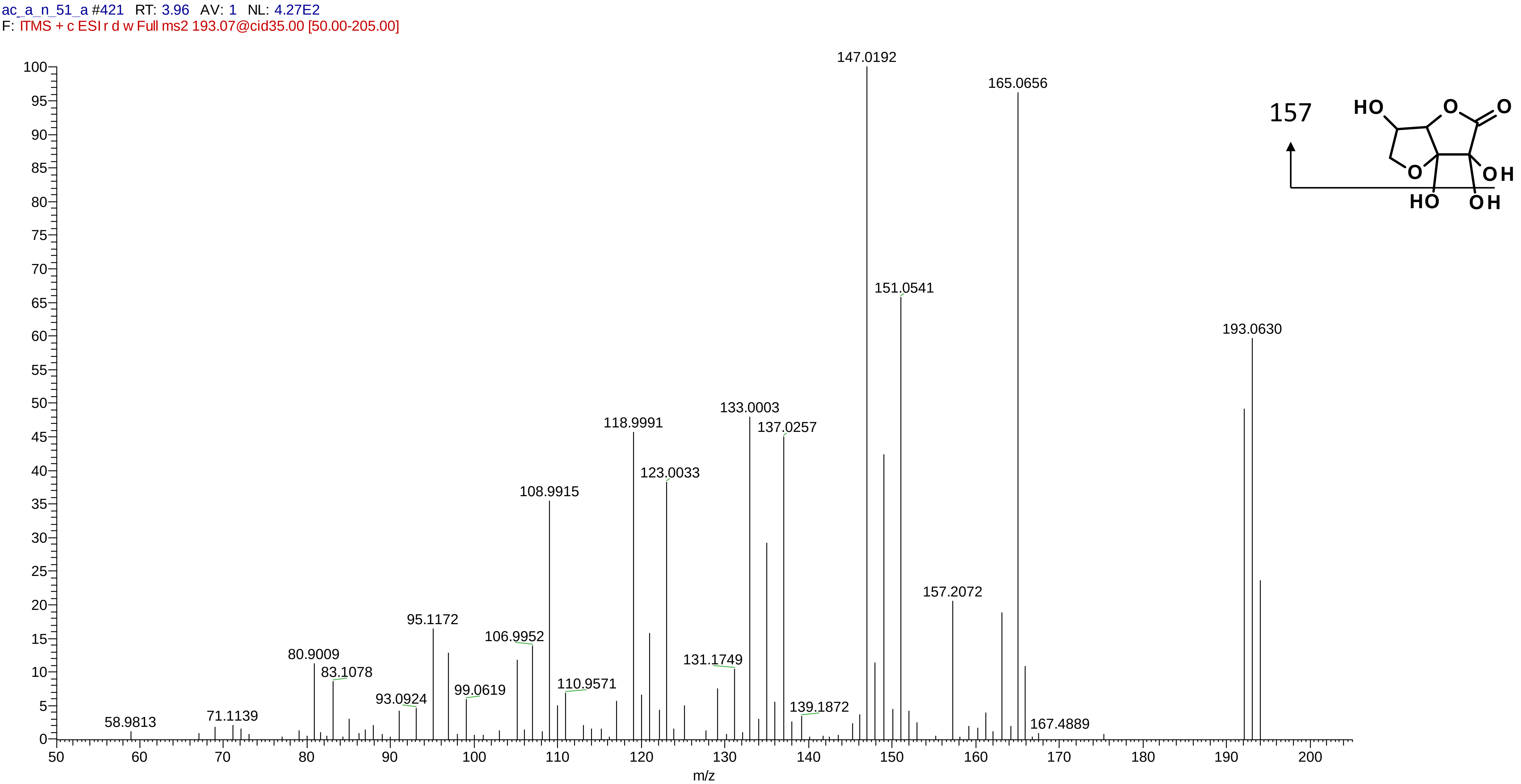

Dehydroascorbic acid RT:4.05 min *m/z*:175.0237

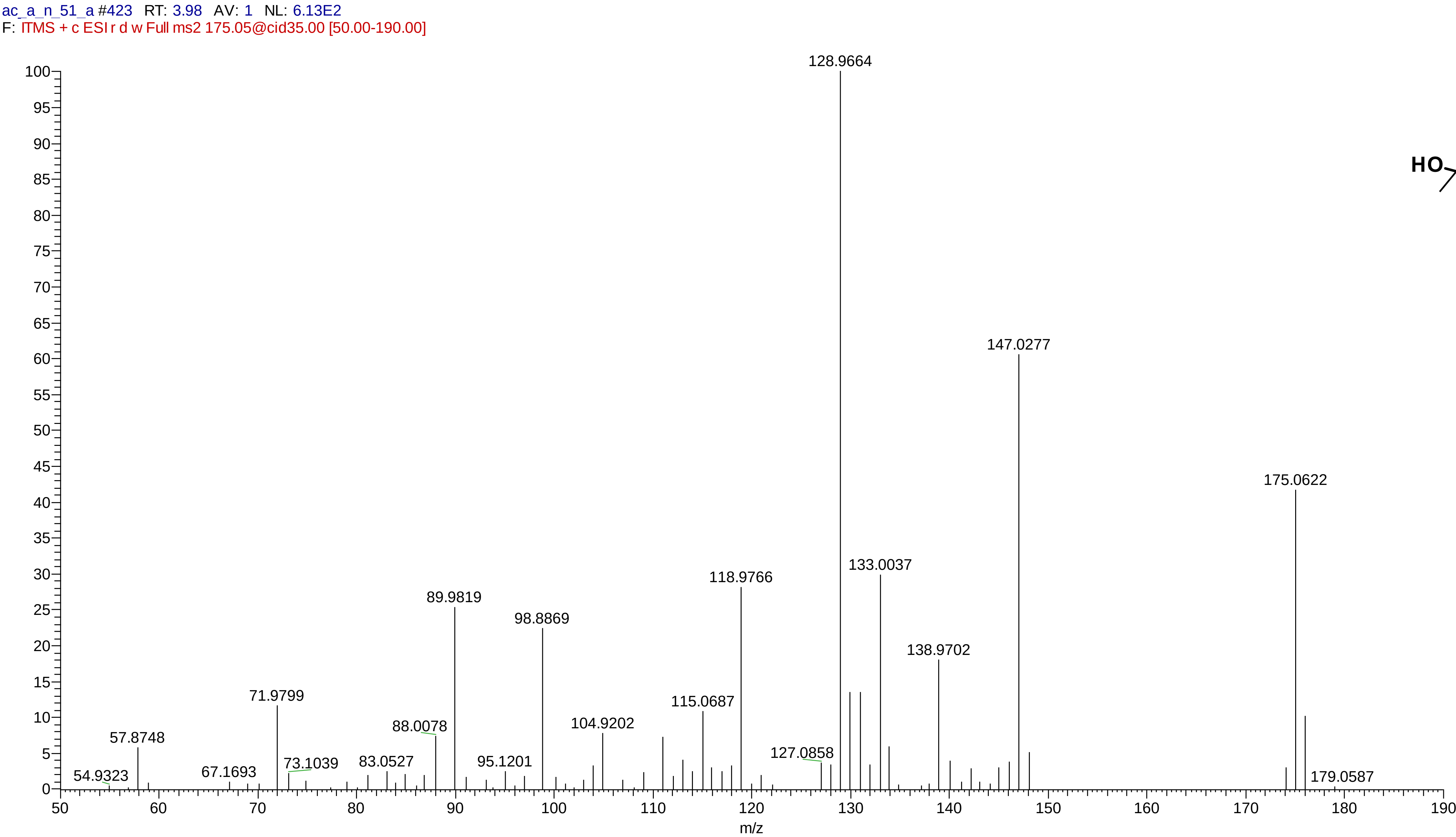

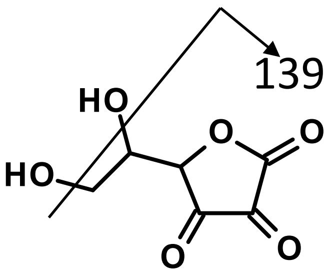

GSH RT:3.62 min *m/z*:308.0911

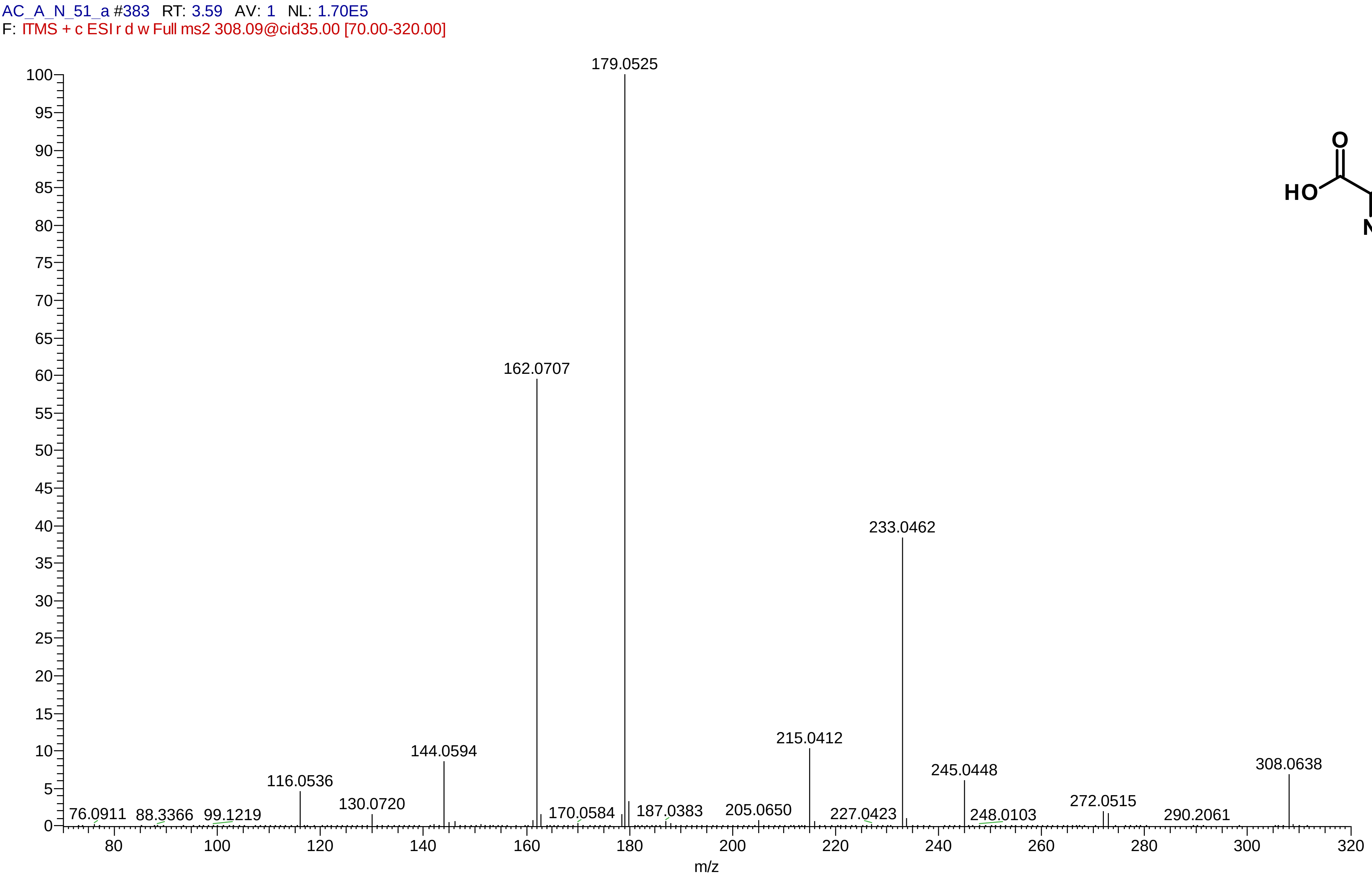

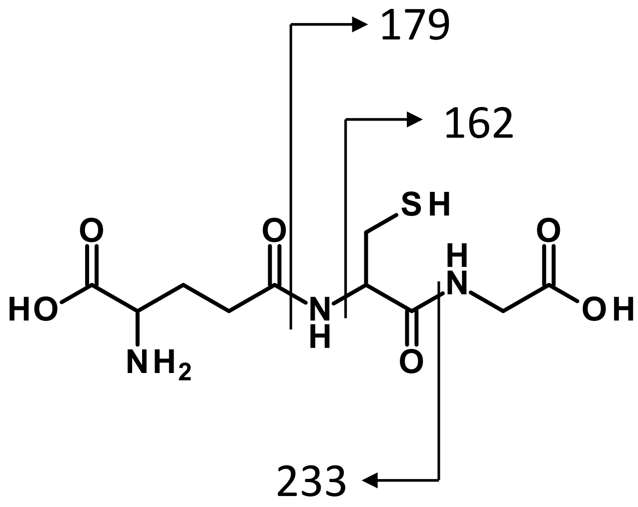

GSSG RT:5.78 min *m/z*:613.1592

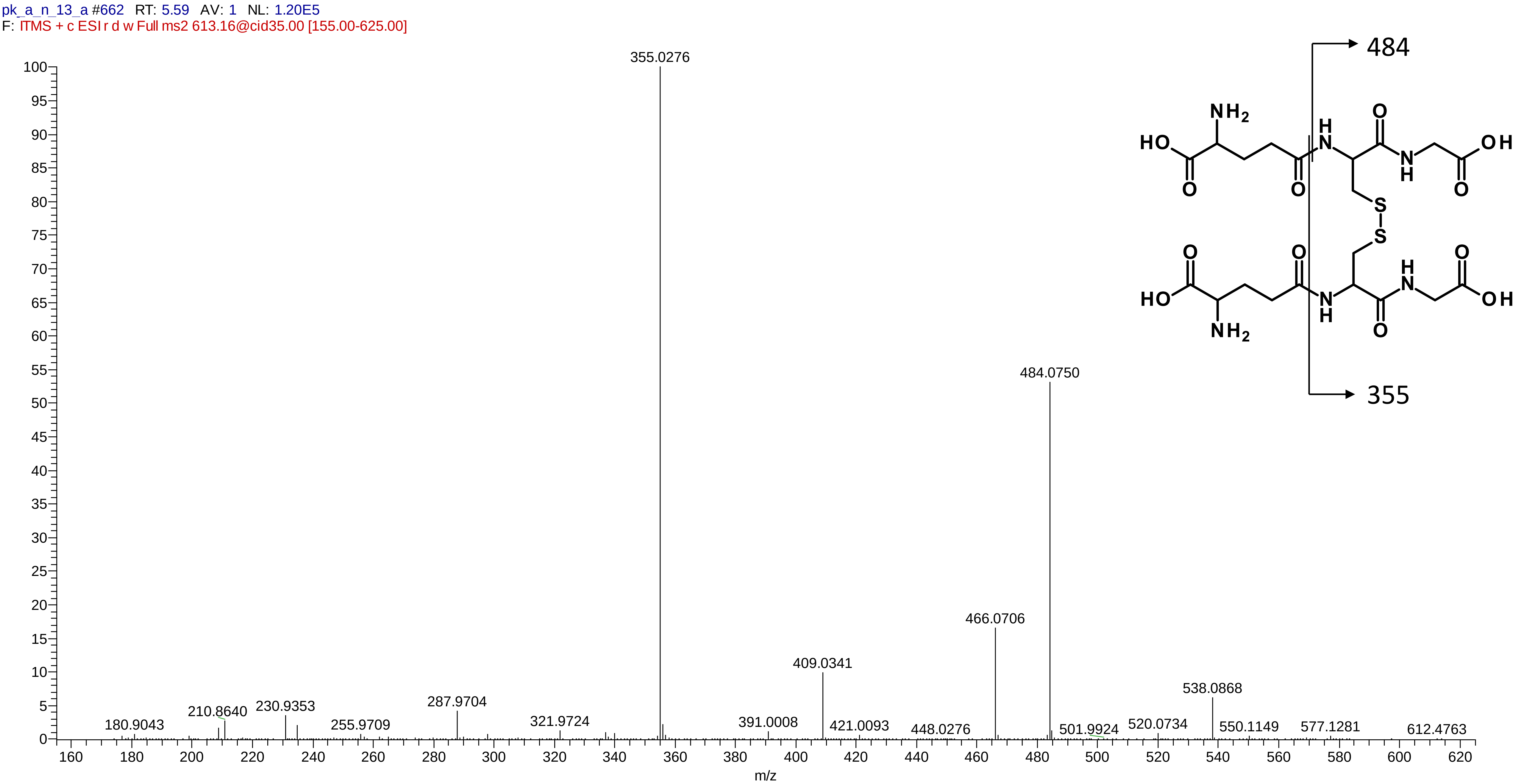

Ophthalmic acid RT:4.05 min *m/z*:290.1347

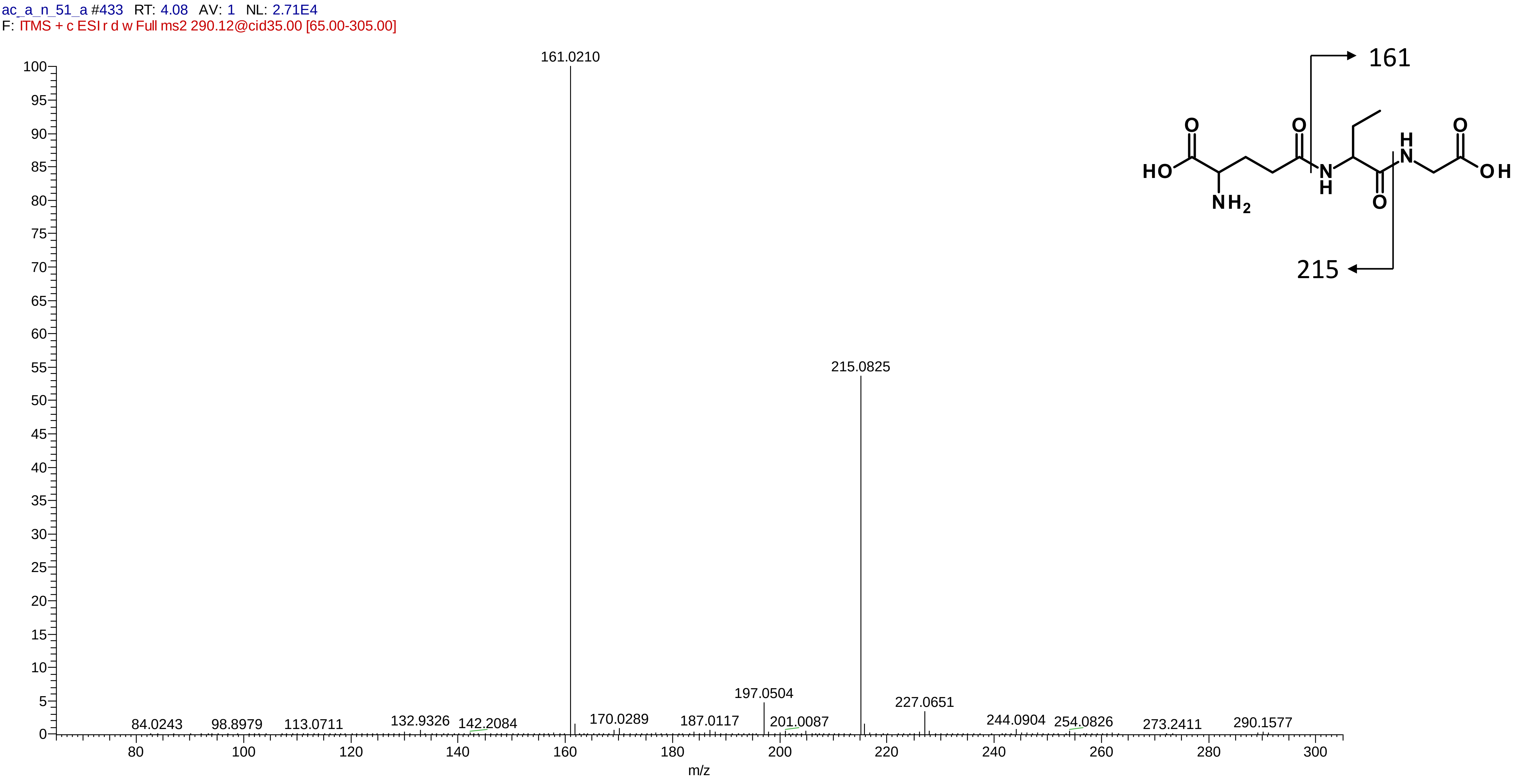

Glutamic acid RT:2.38 min *m/z*:148.0604

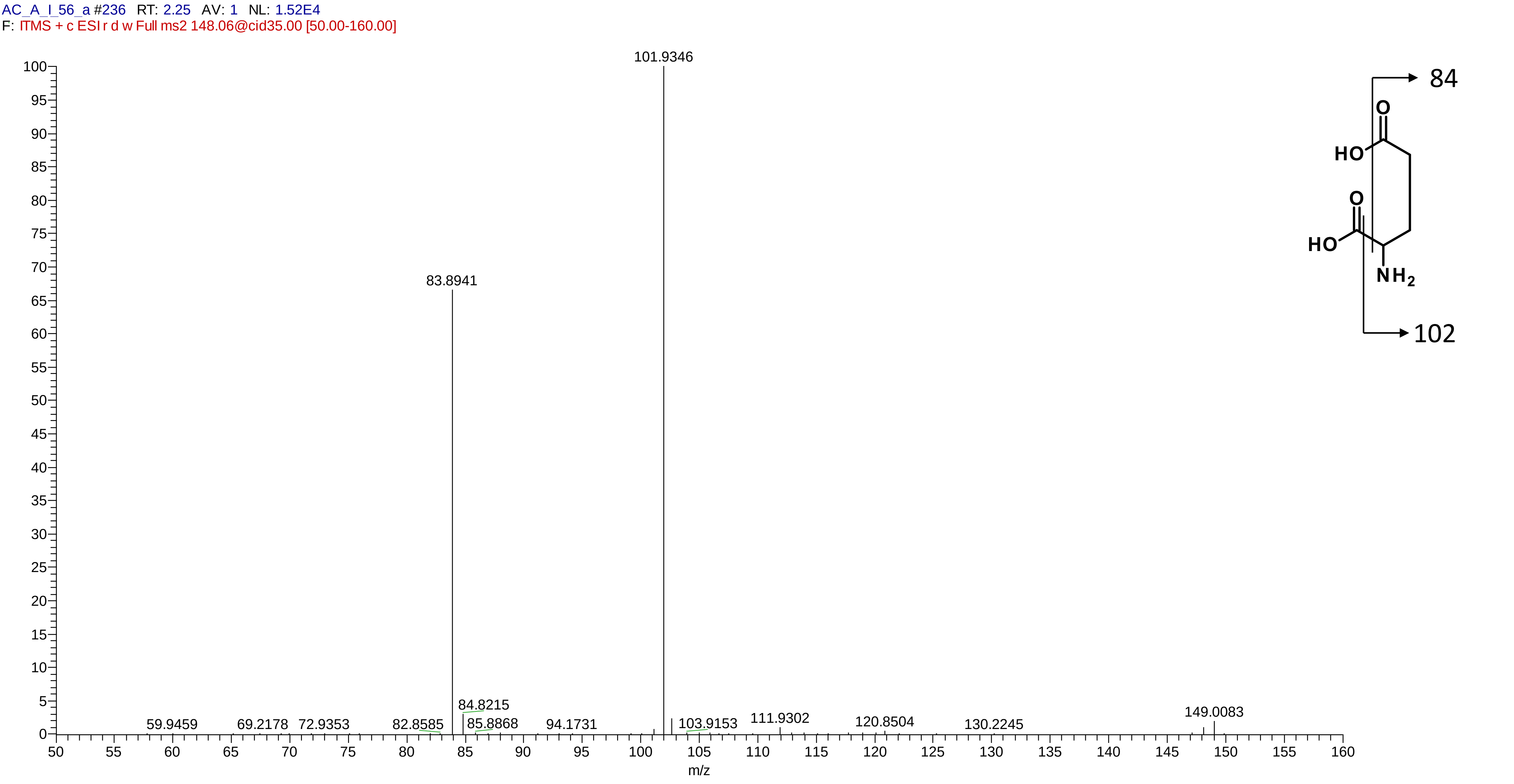

Pyroglutamic acid RT:2.33 min *m/z*:130.0499

ac_a_n_51_a #247 RT: 2.35 AV: 1 NL: 2.65E4
F: ITMS + c ESI r d w Full ms2 130.07@cid35.00 [50.00-145.00]

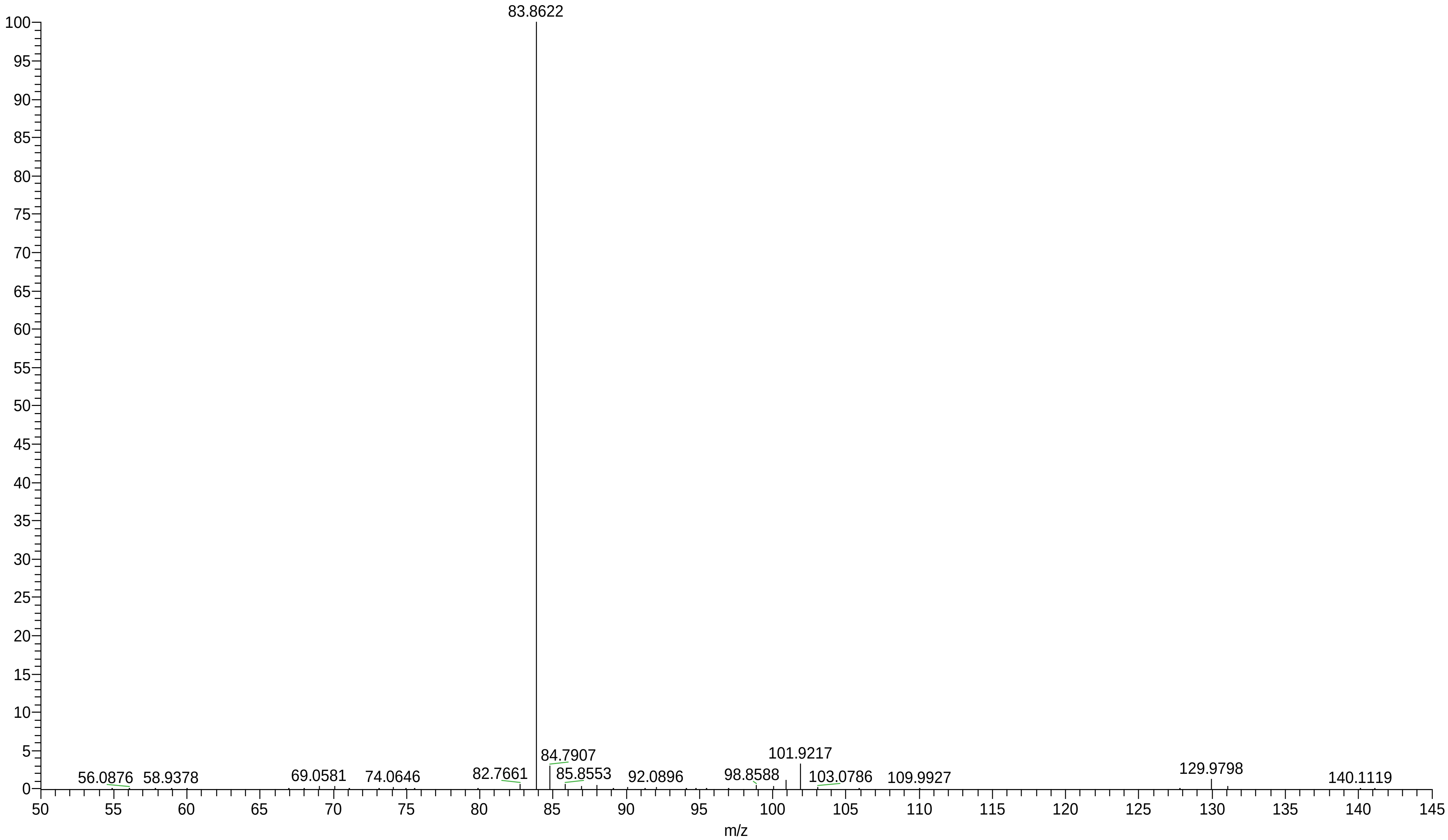

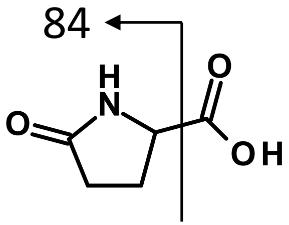

Pyroglutamic acid RT:4.16 min *m/z*:130.0499

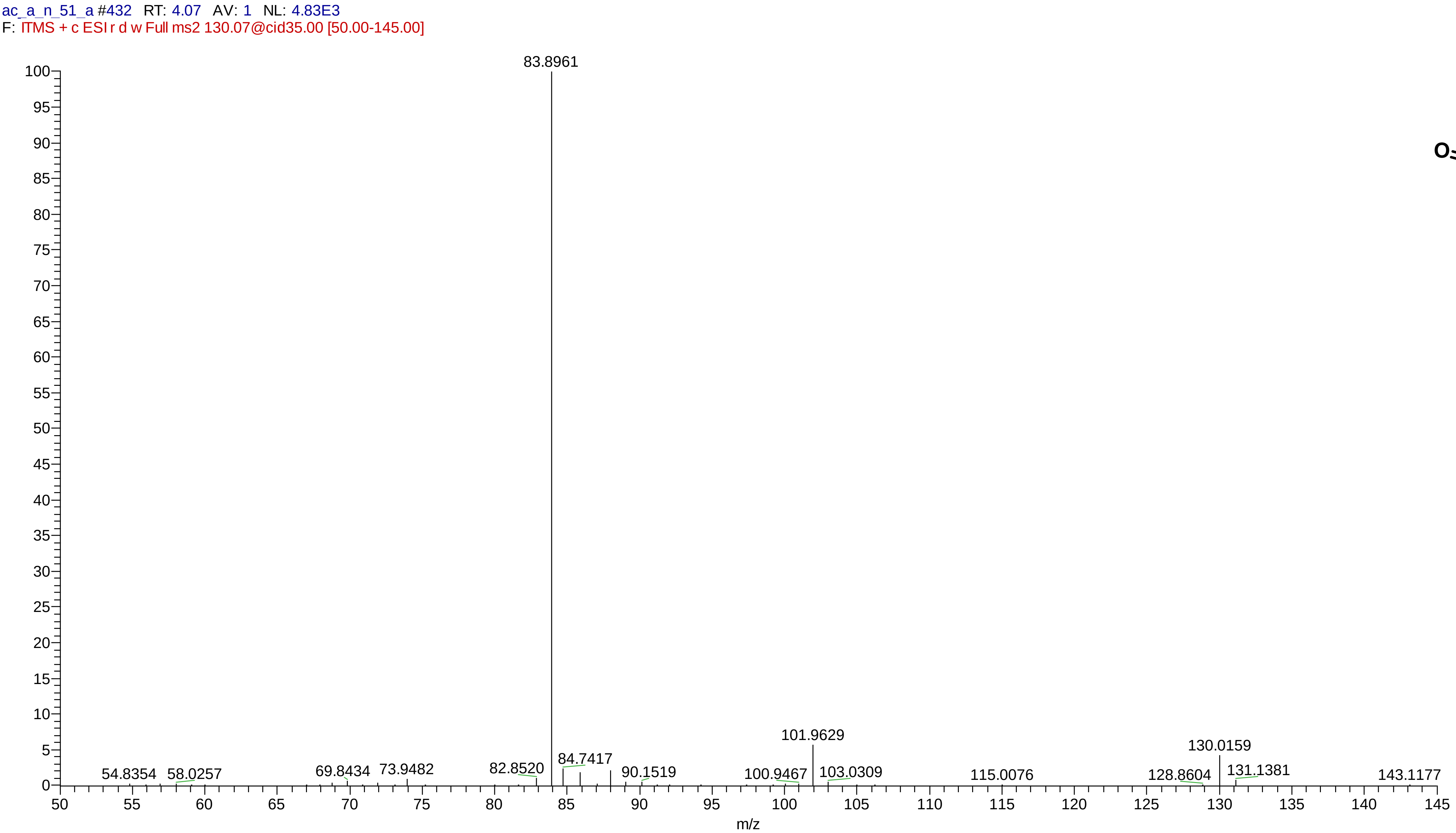

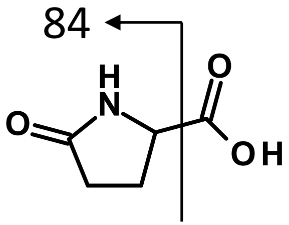

Asparagine RT:2.34 min *m/z*:133.0608

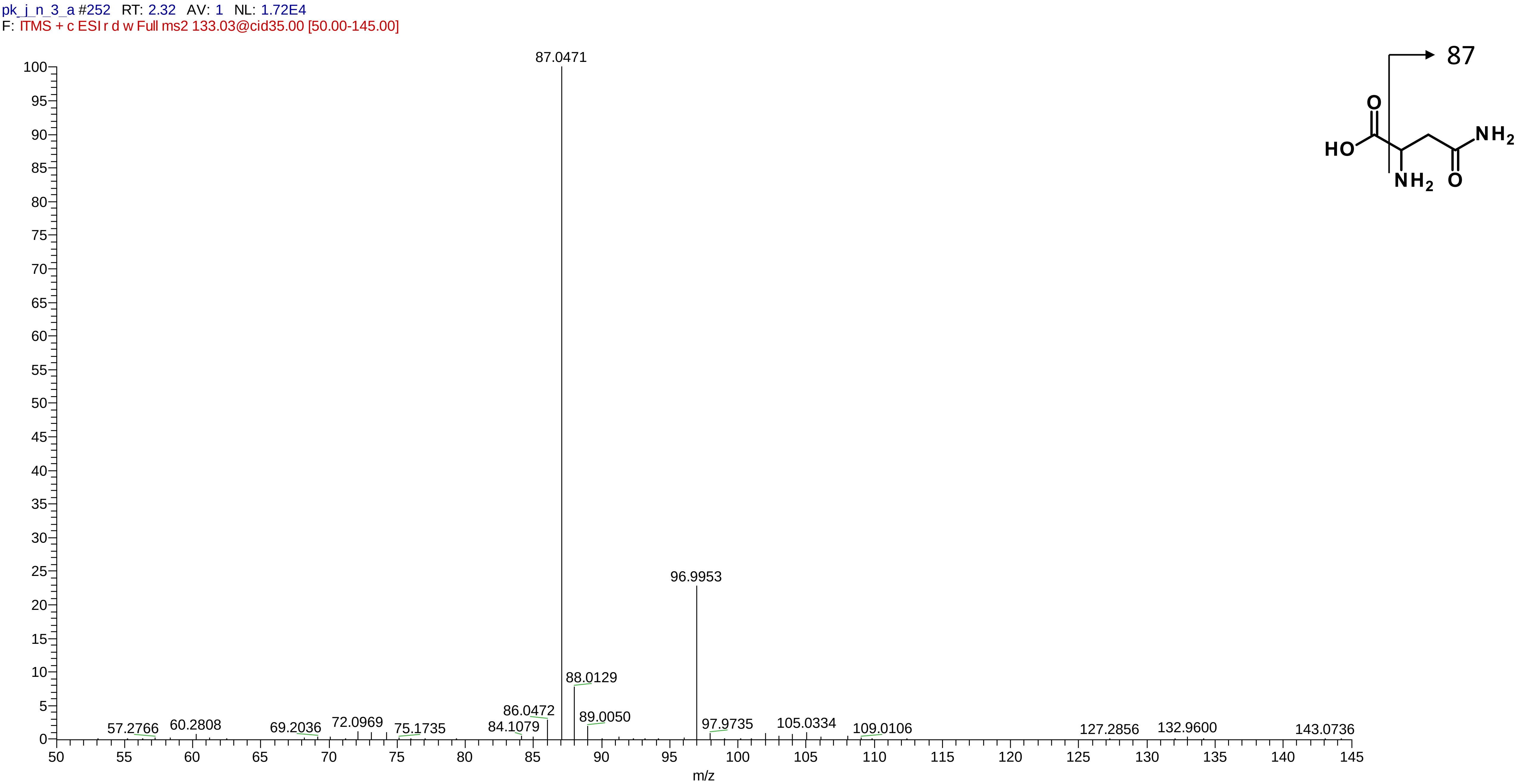

Proline RT:2.47 min *m/z*:116.0702

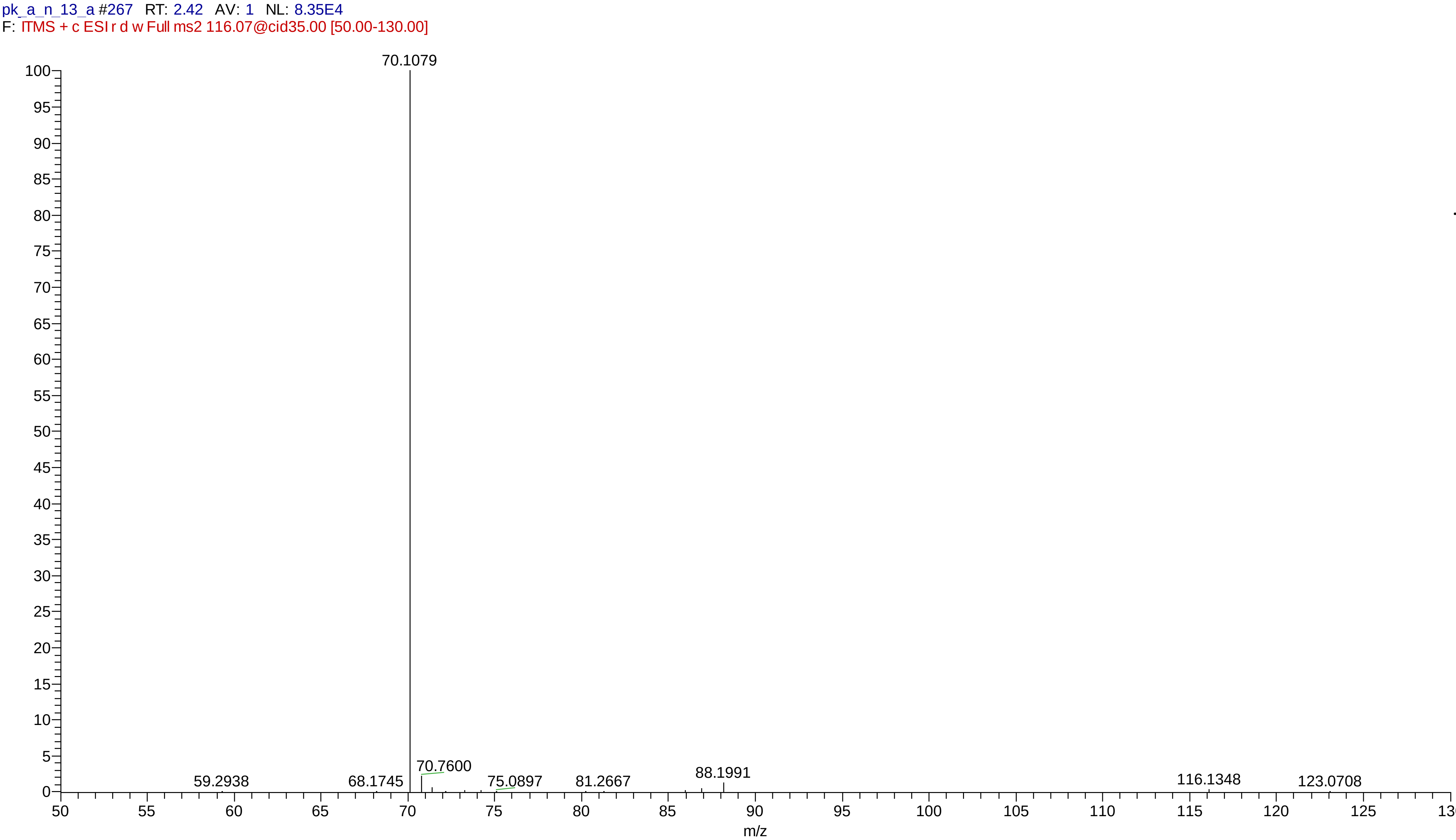

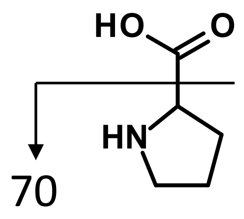

Methionine RT:3.43 min *m/z*:150.0583

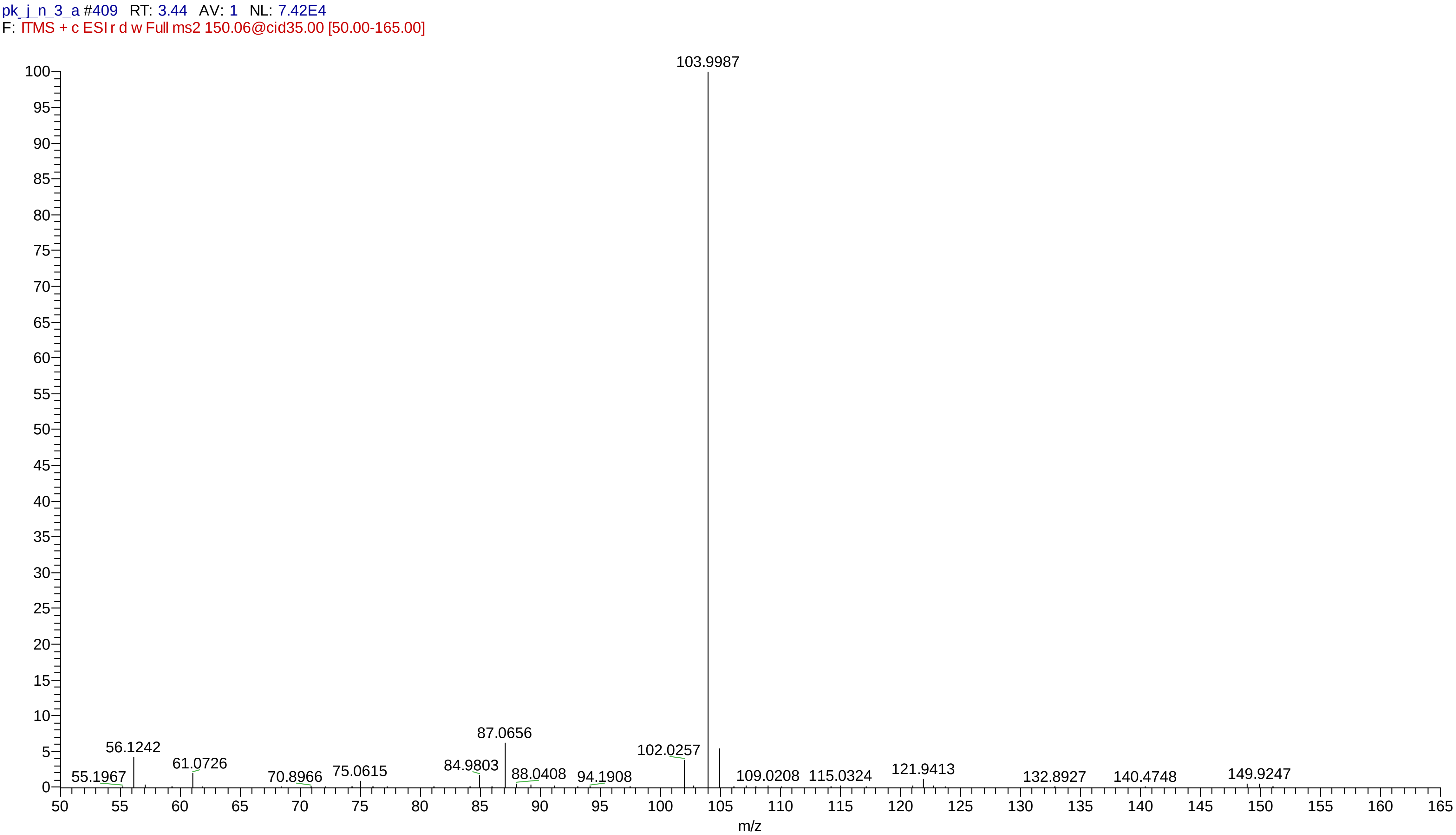

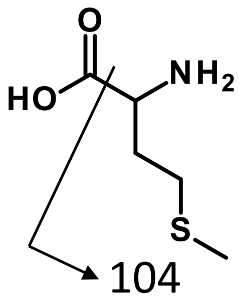

Trimethyllysine RT:2.18 min *m/z*:189.1598

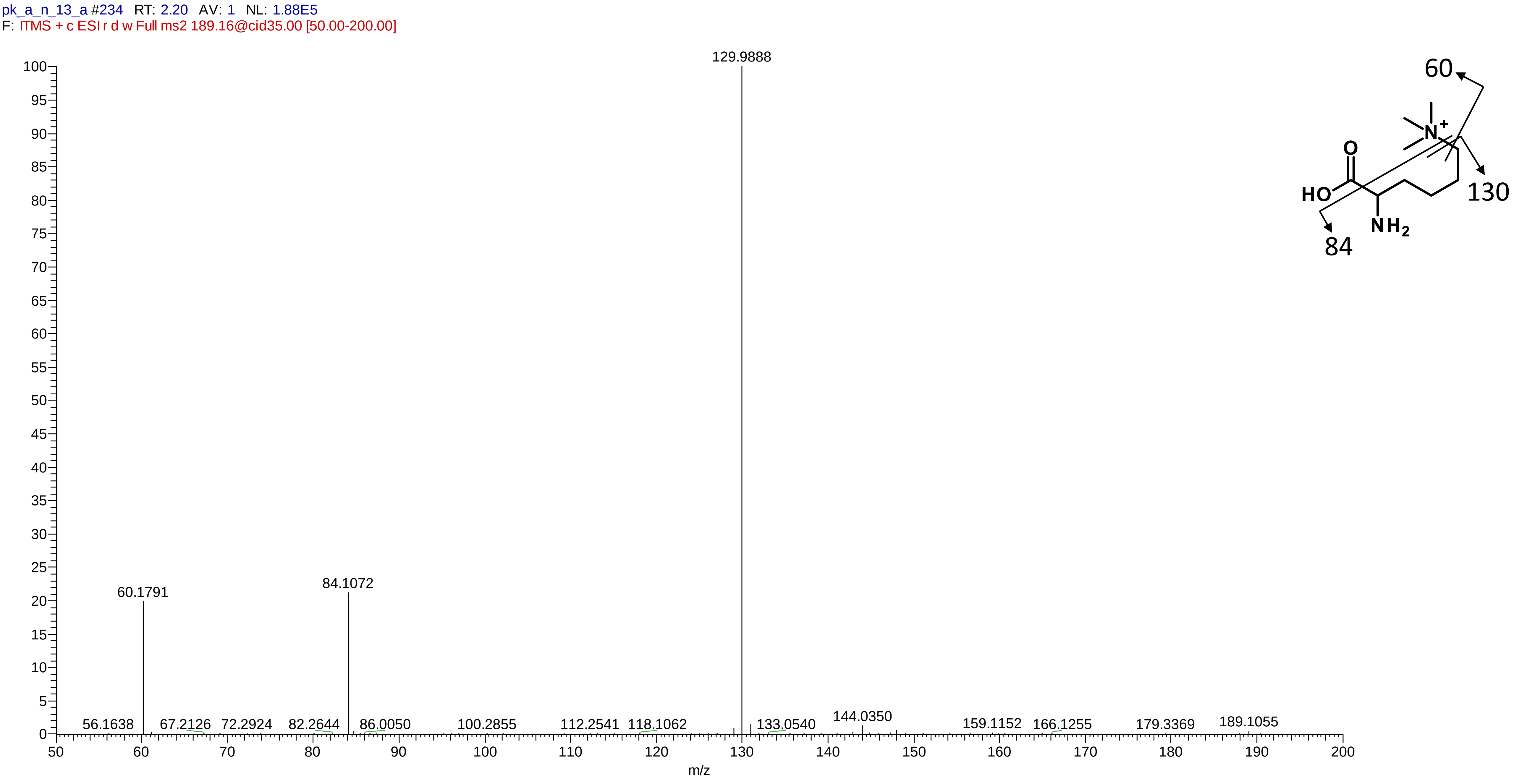

Phenylalanine RT:11.36 min *m/z*:166.0859

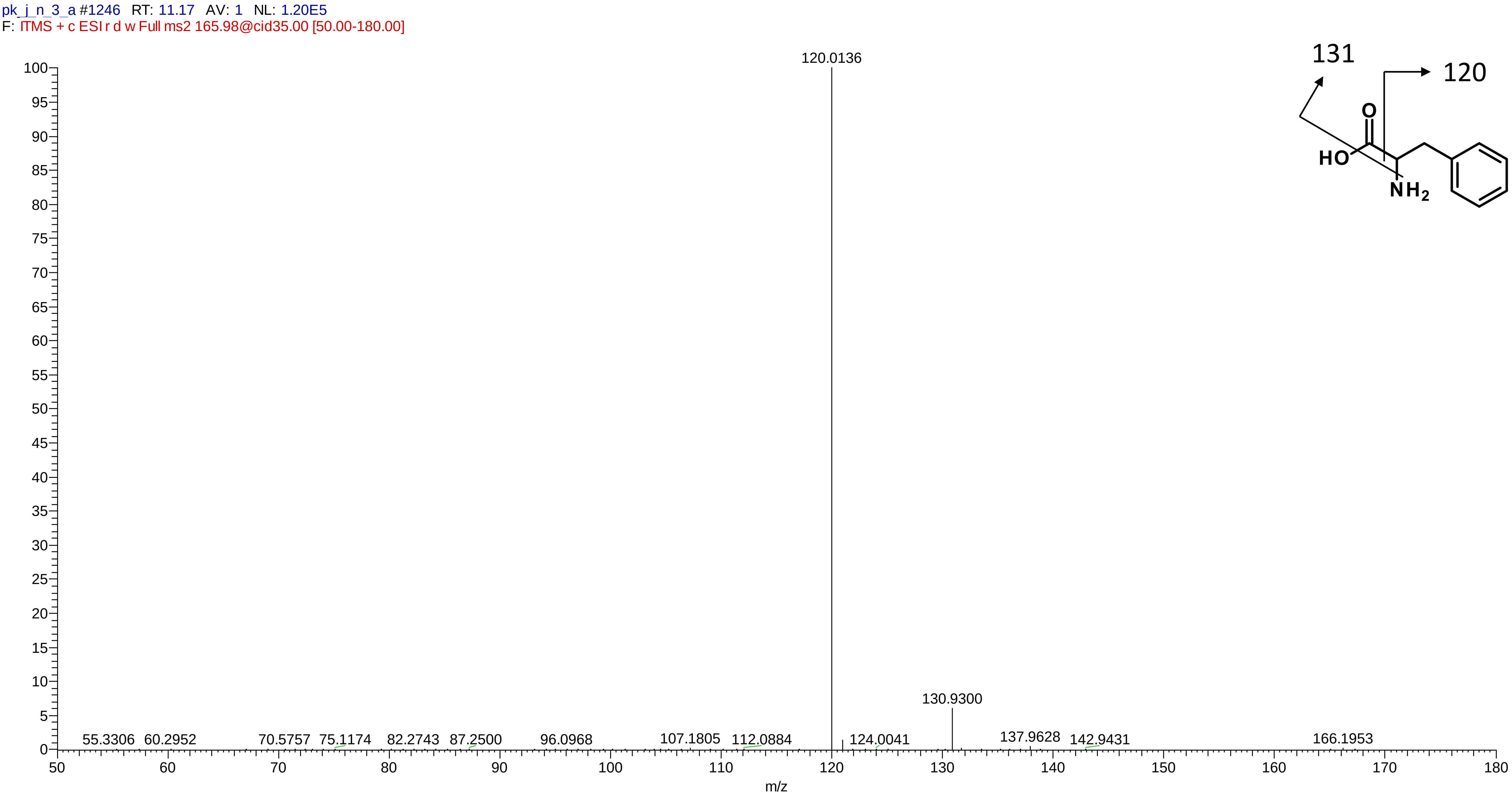

Tyrosine RT:5.43 min *m/z*:182.0812

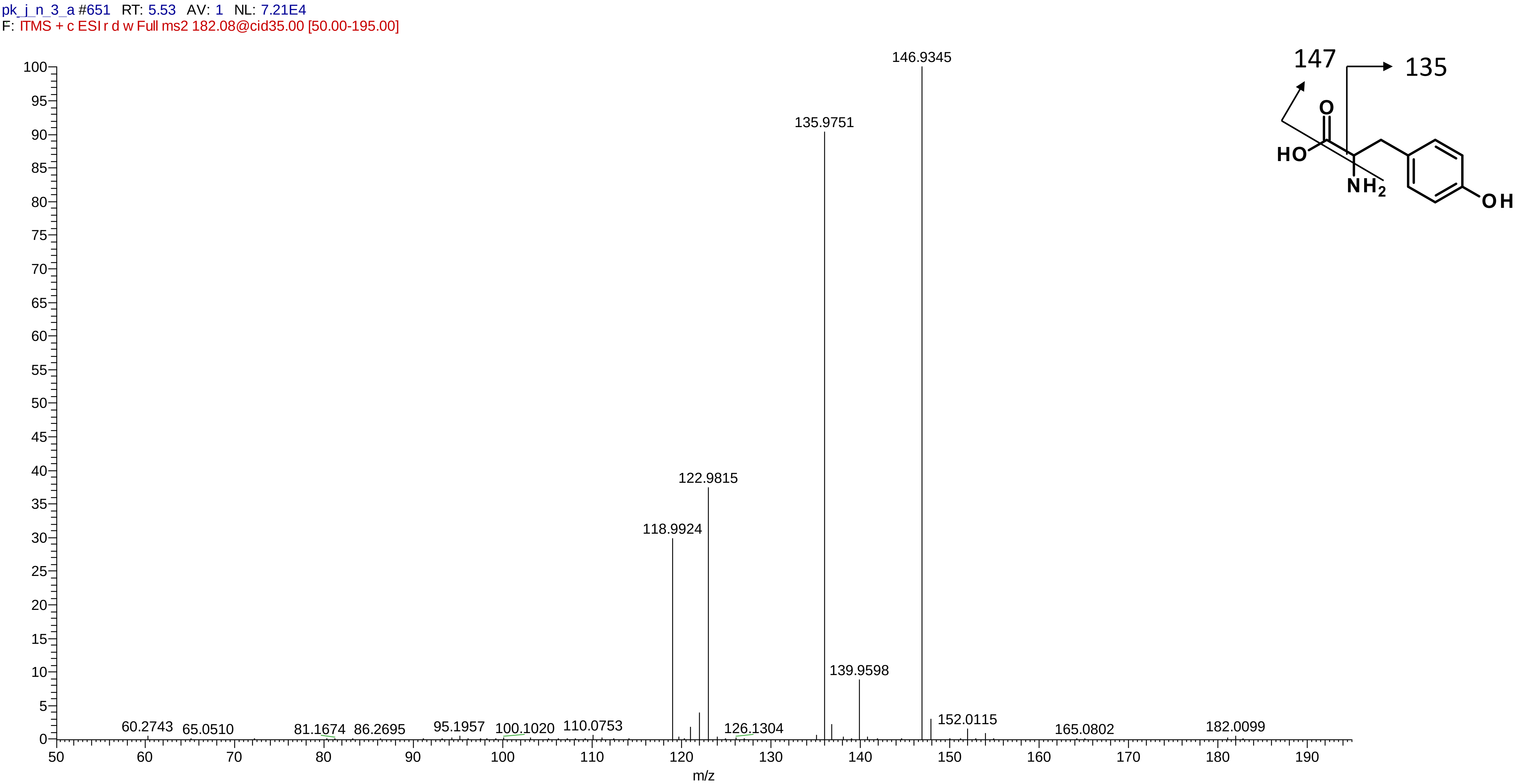

Tryptophan RT:16.29 min *m/z*:205.0972

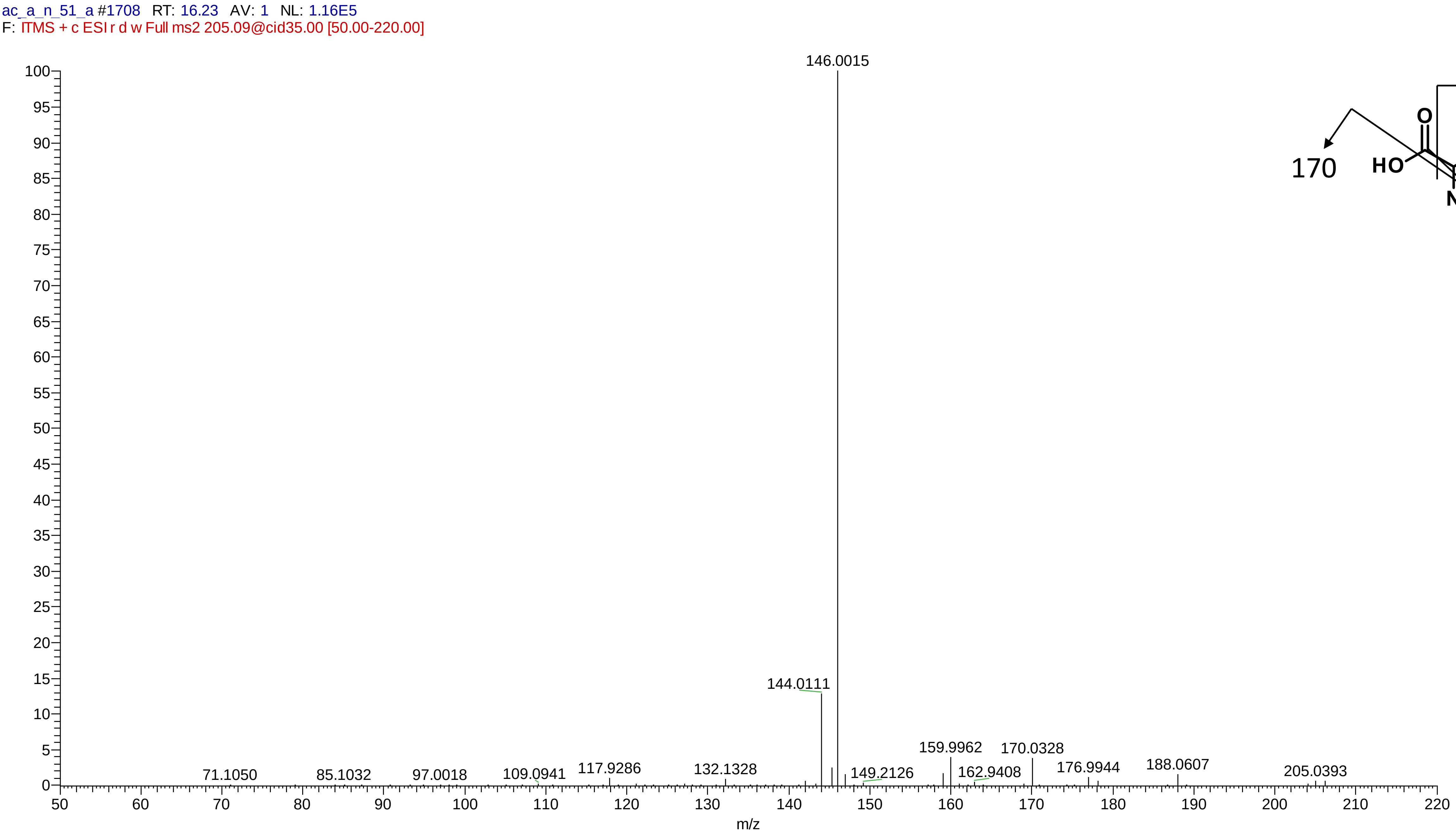

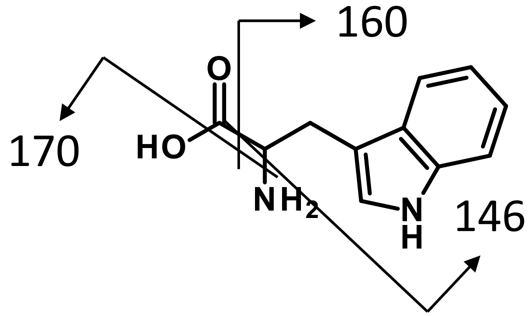

Kynurenin RT:11.23 min *m/z*:209.0921

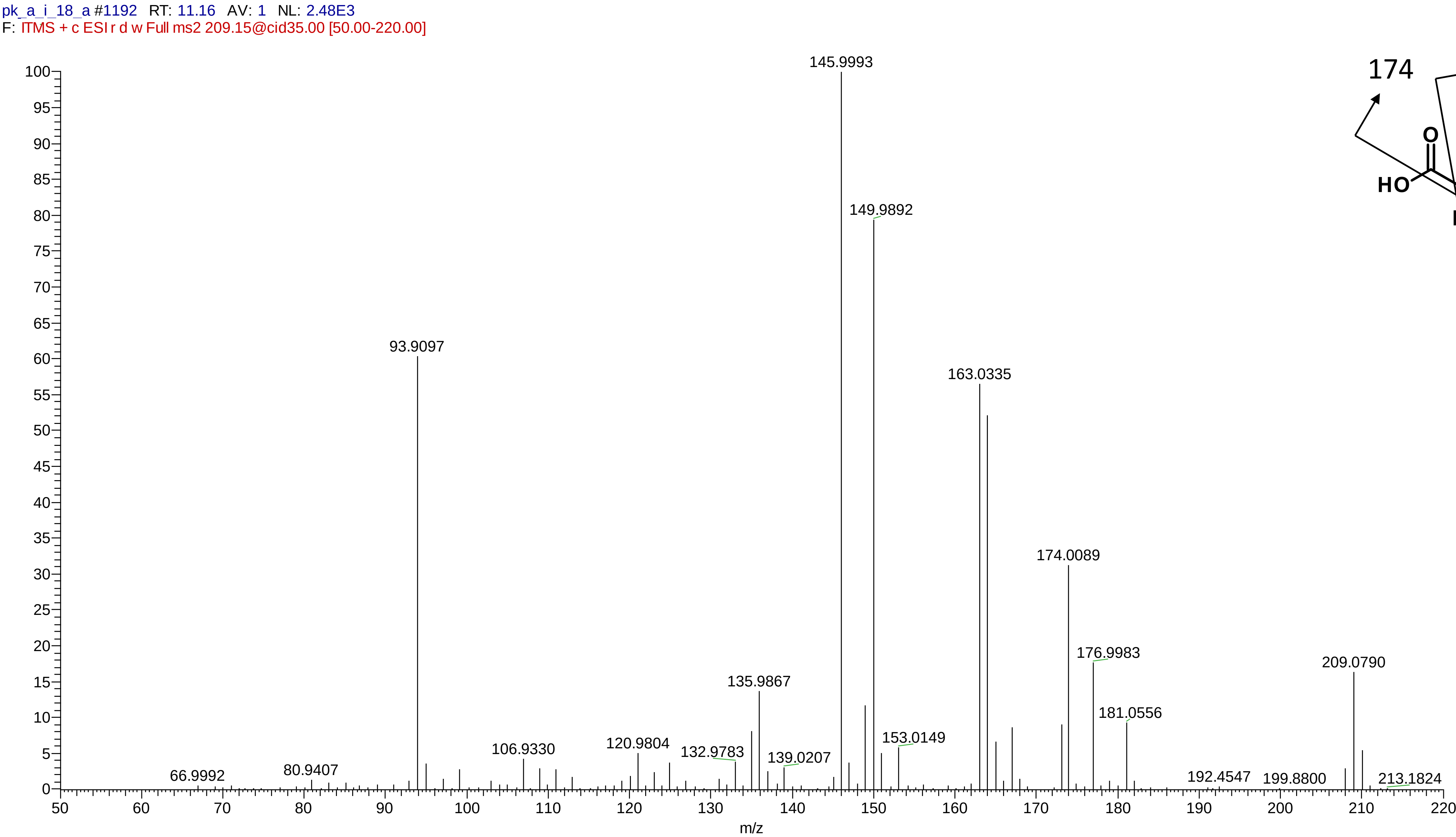

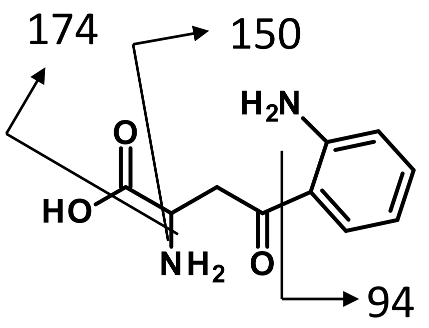

Cholin RT:2.34 min *m/z*:104.1070

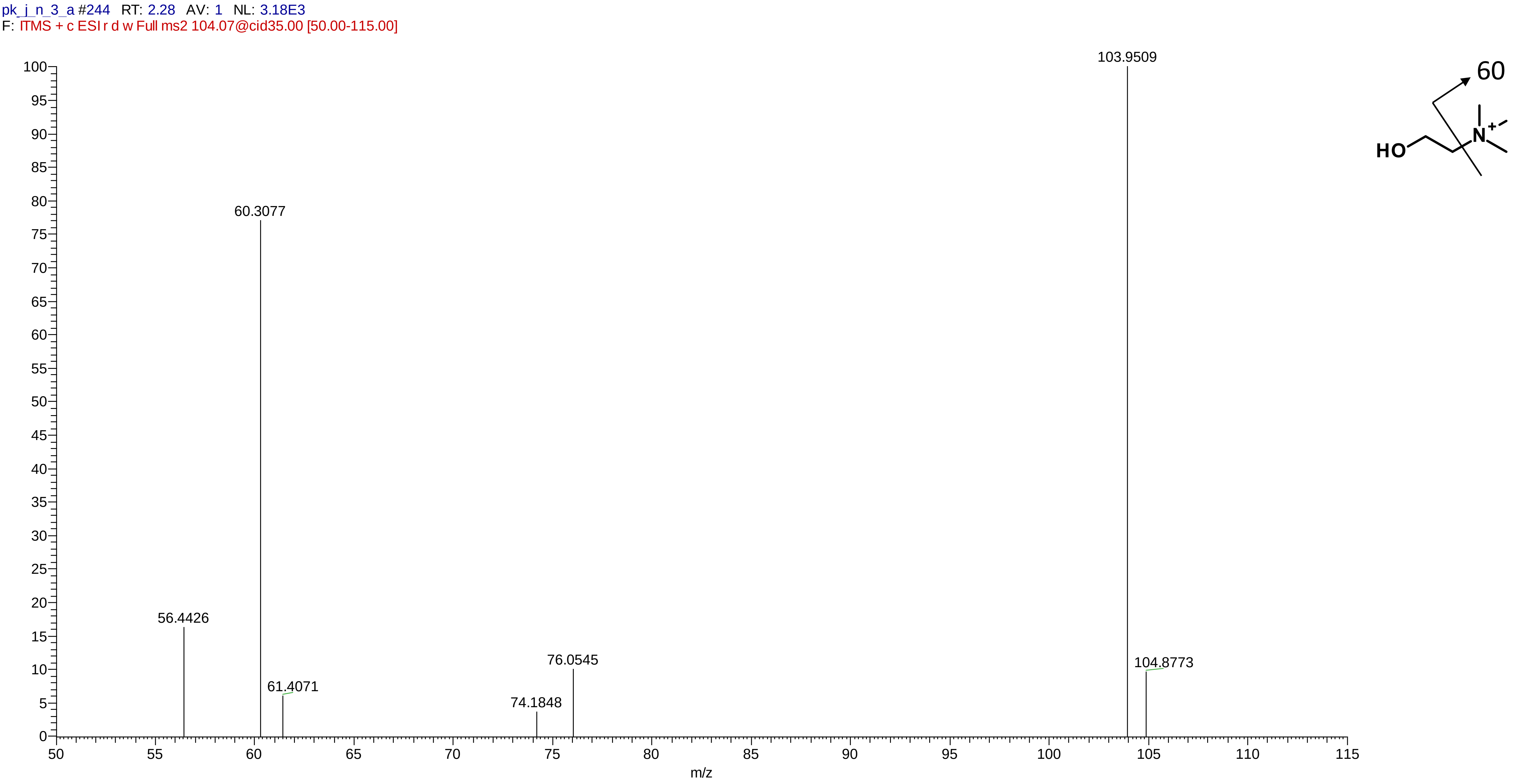

Creatine RT:2.64 min *m/z*:132.0768

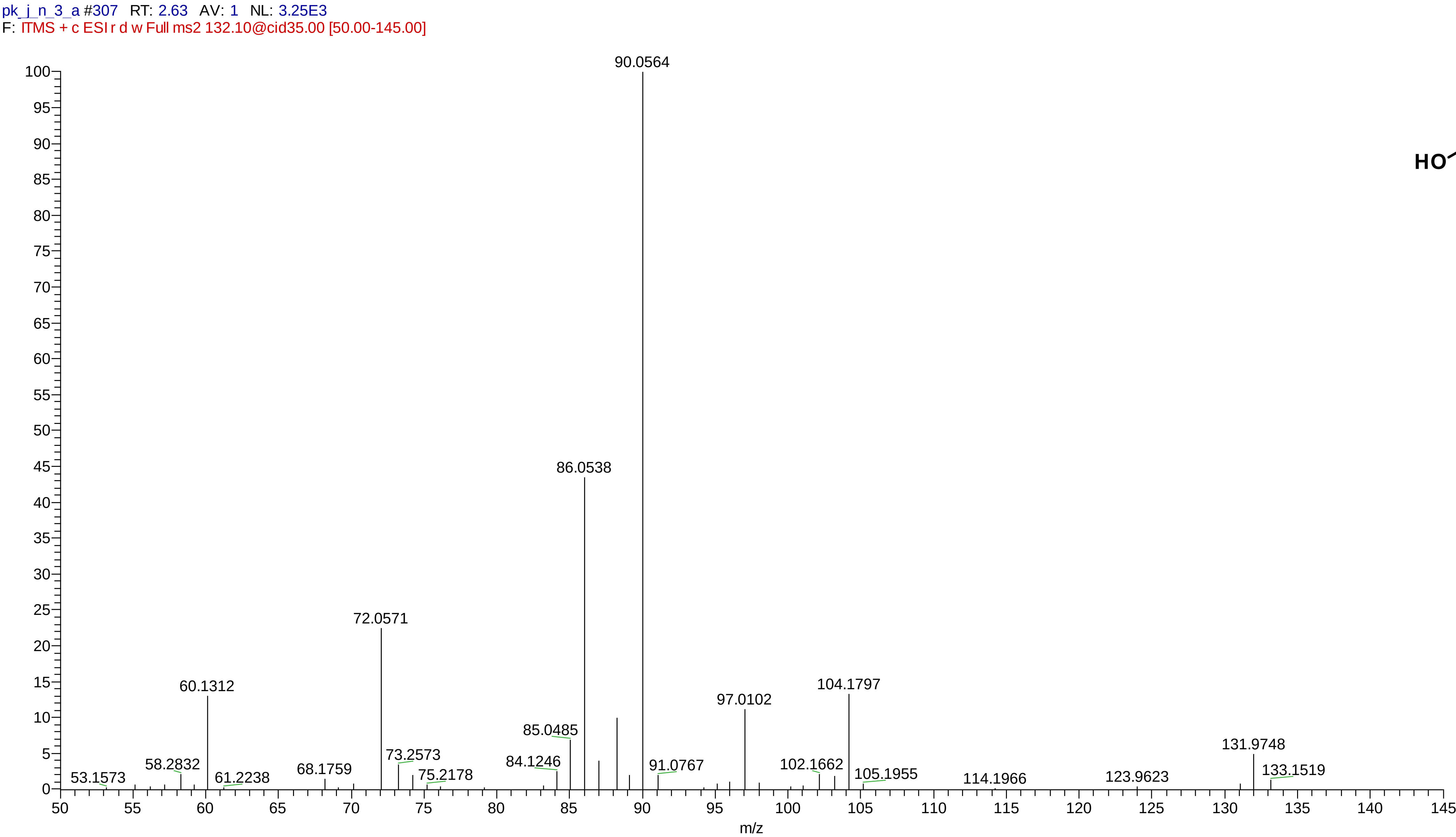

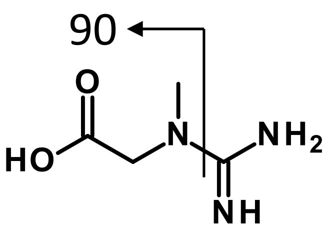

N-Acetylserotonin RT:20.76 min *m/z*:219.1128

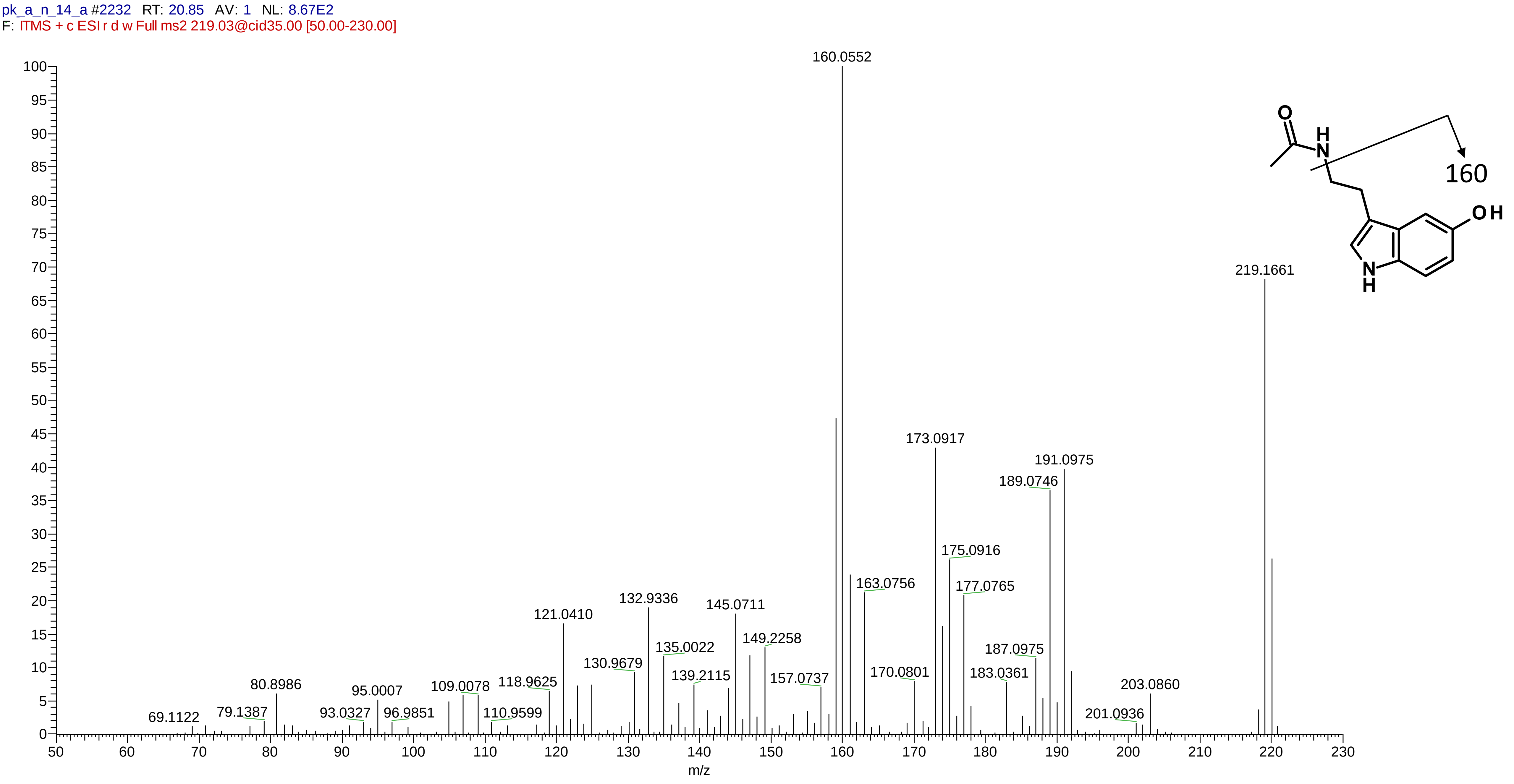

Spermidine RT:2.24 min *m/z*:146.1652

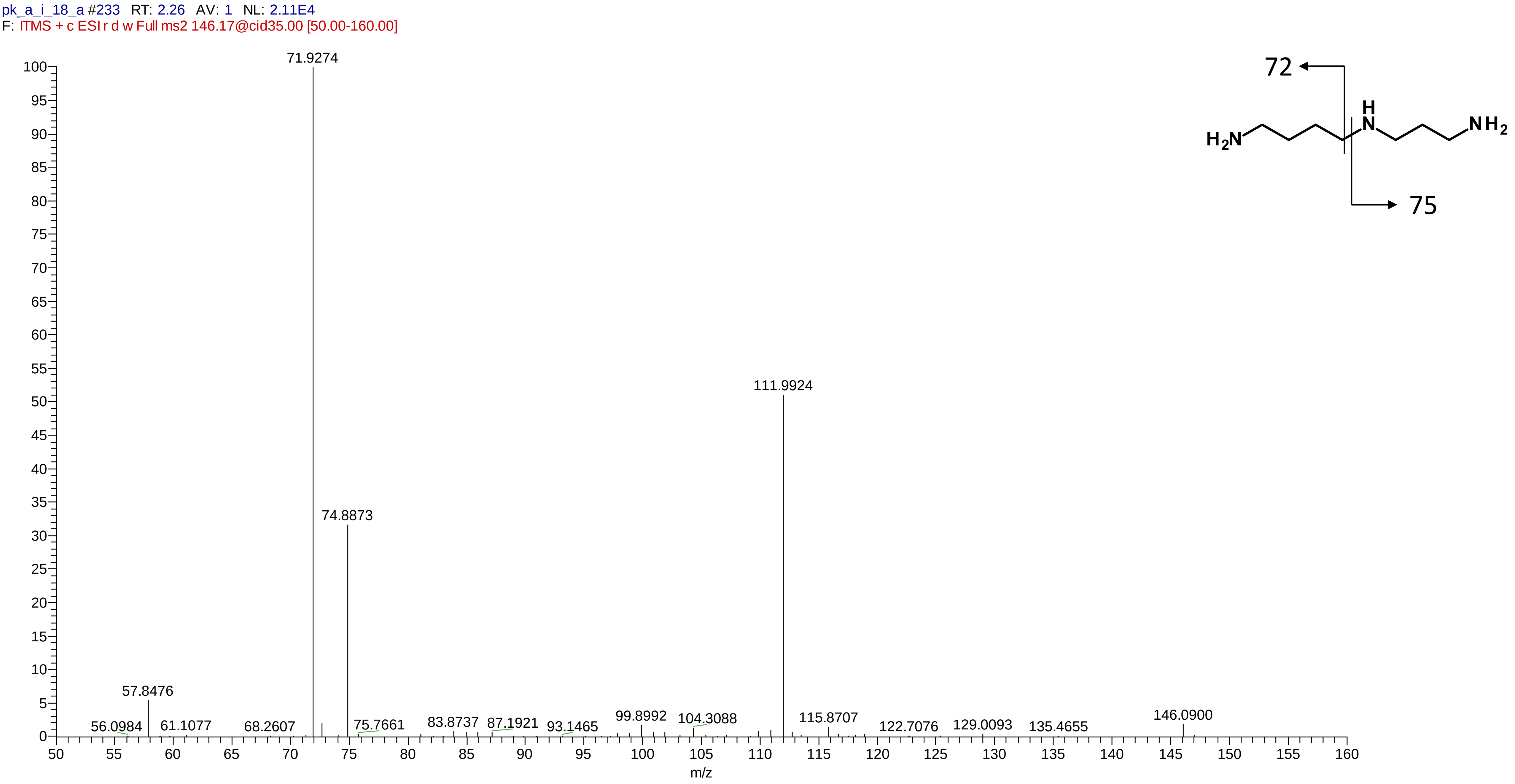

Pavettamine RT:2.03 min m/z:252.1918

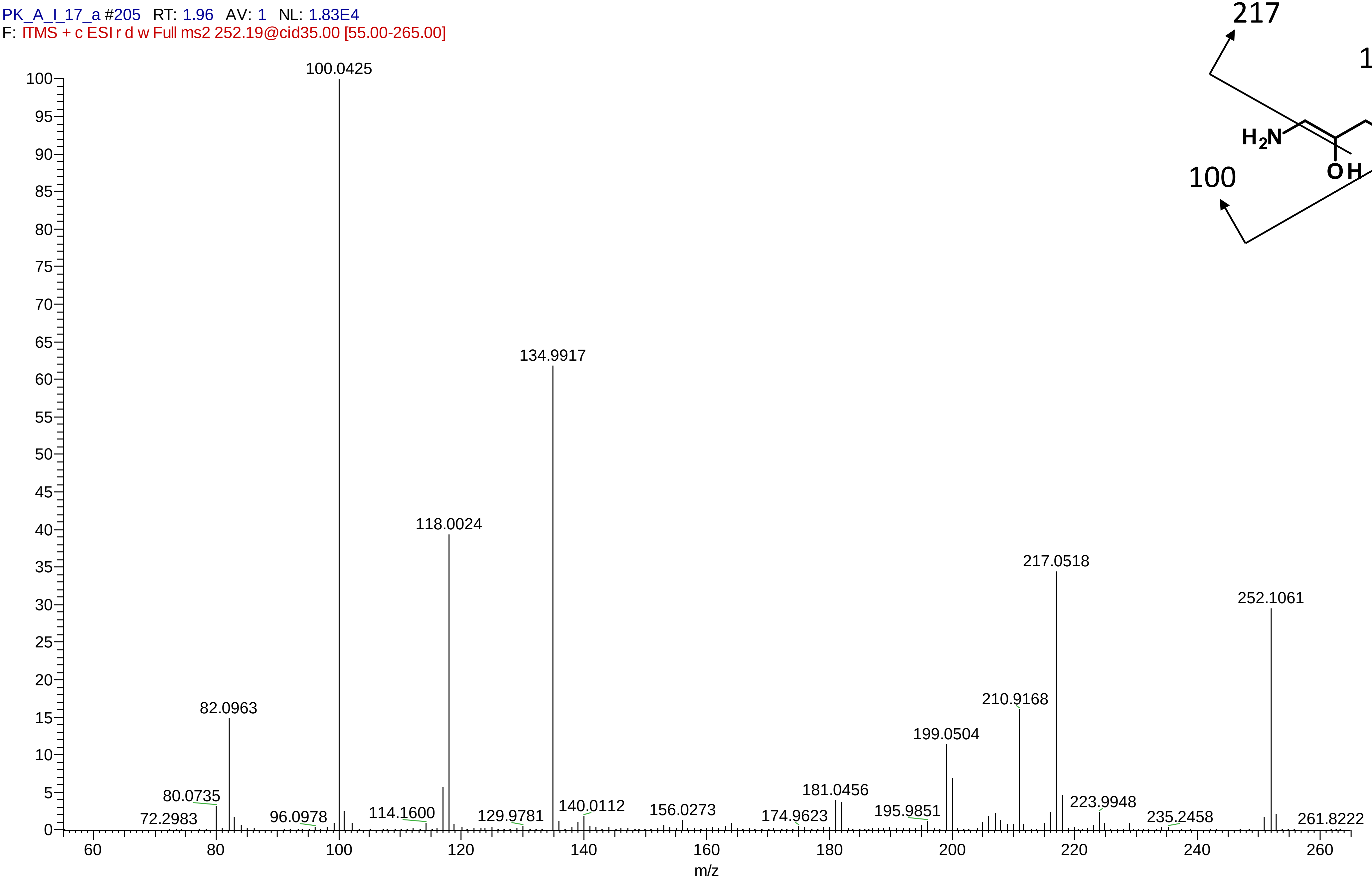

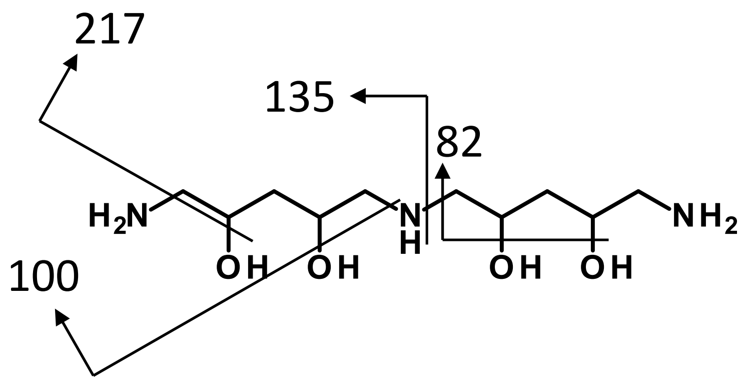

Pantothenic acid RT:14.18 min *m/z*:220.1180

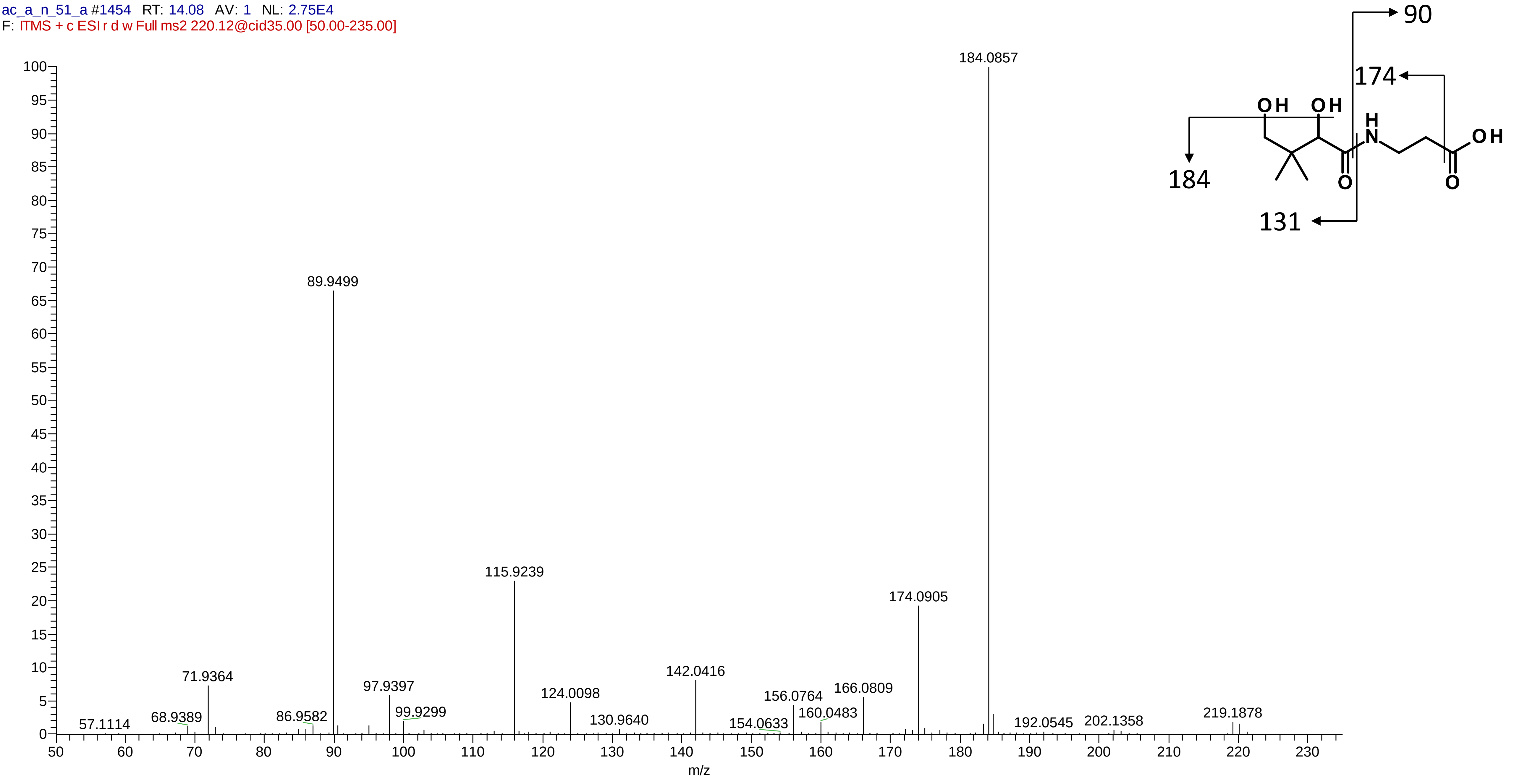

NAD⁺ RT:3.89 min *m/z*:664.1150

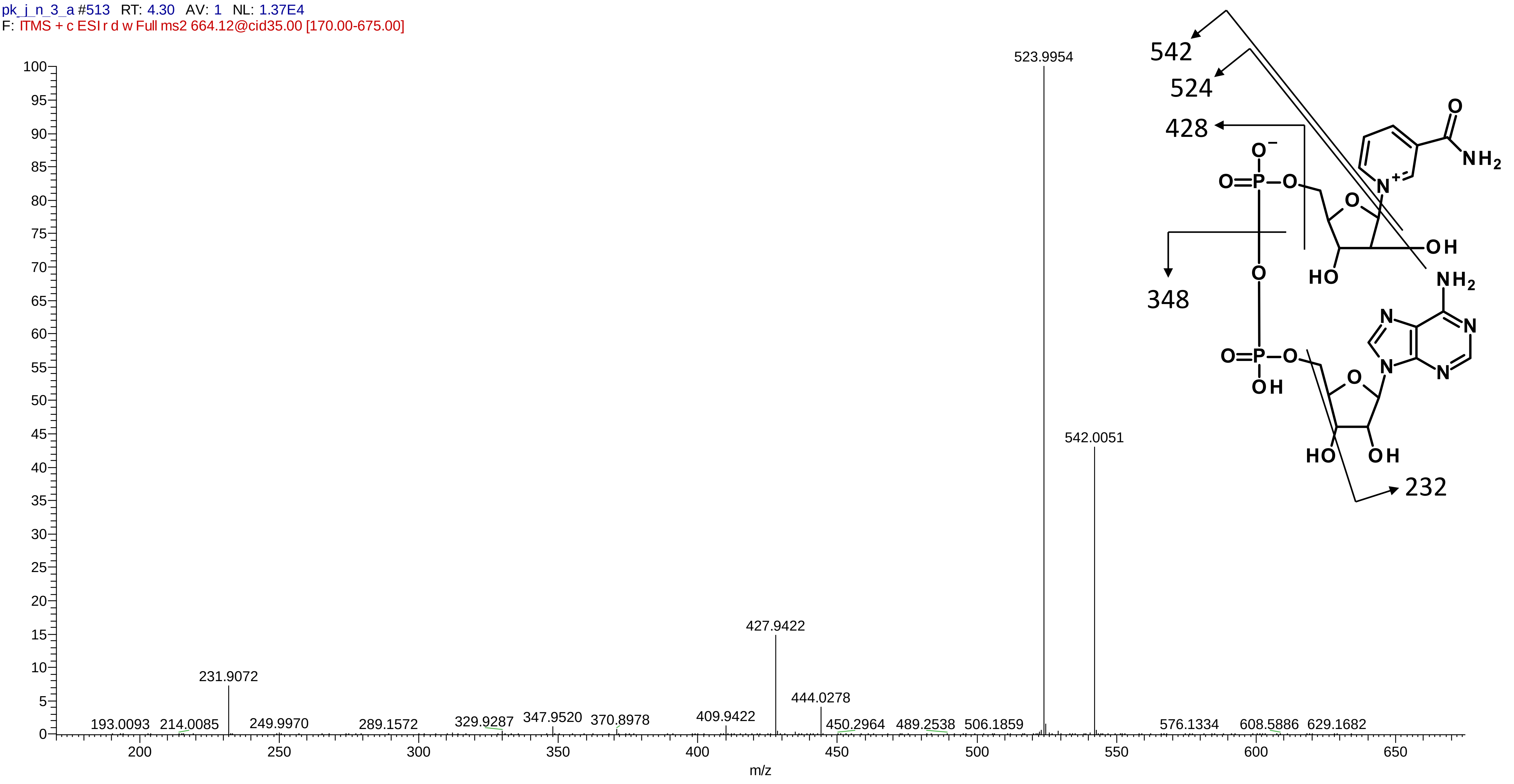

Nicotinate D-ribonucleoside RT:2.74 min *m/z*:256.0810

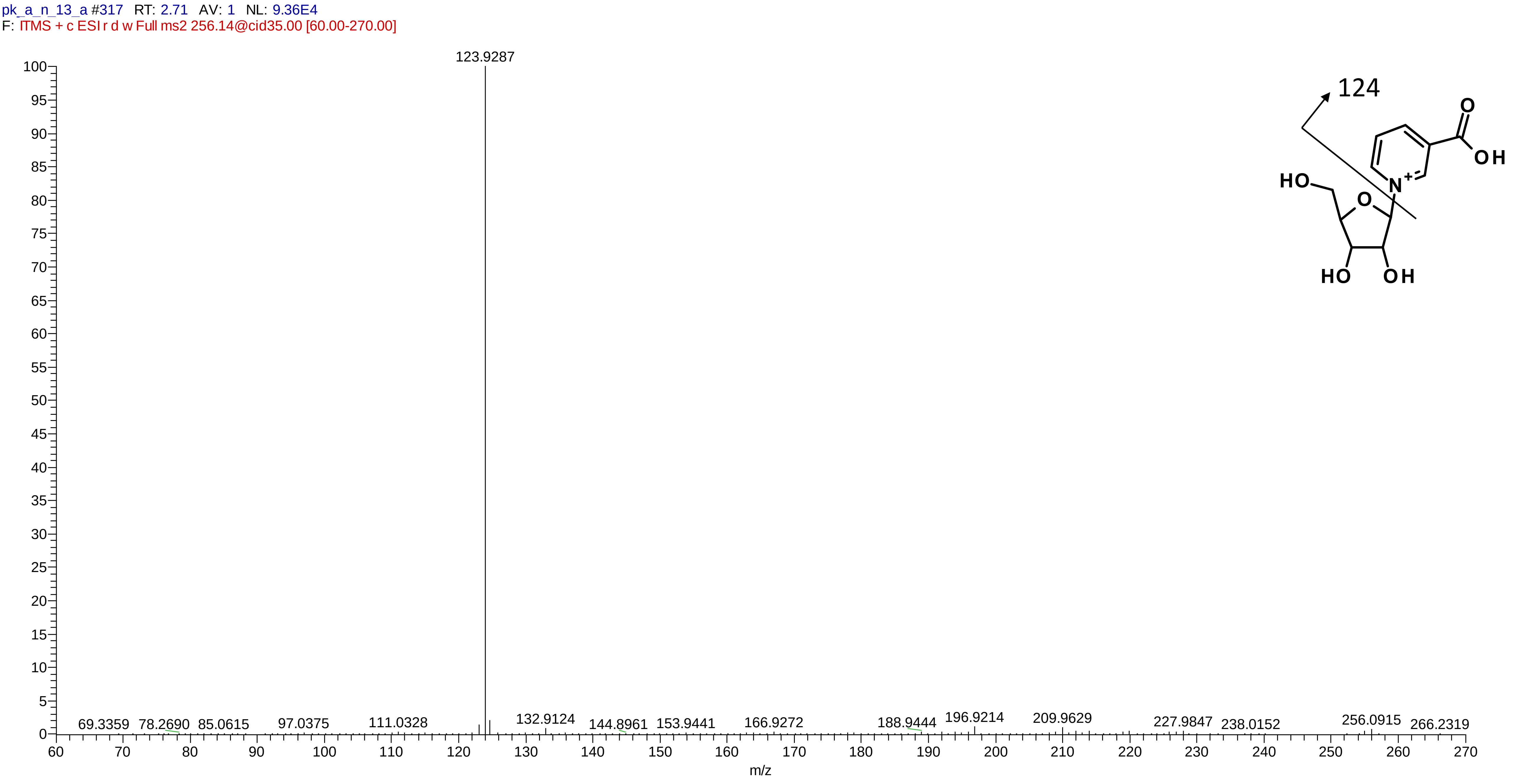

Riboflavin-5'-phosphate RT:17.84 min *m/z*:457.1119

pk_a_n_15_a #1943 RT: 17.86 AV: 1 NL: 5.35E4
F: ITMS + c ESI r d w Full ms2 457.11@cid35.00 [115.00-470.00]

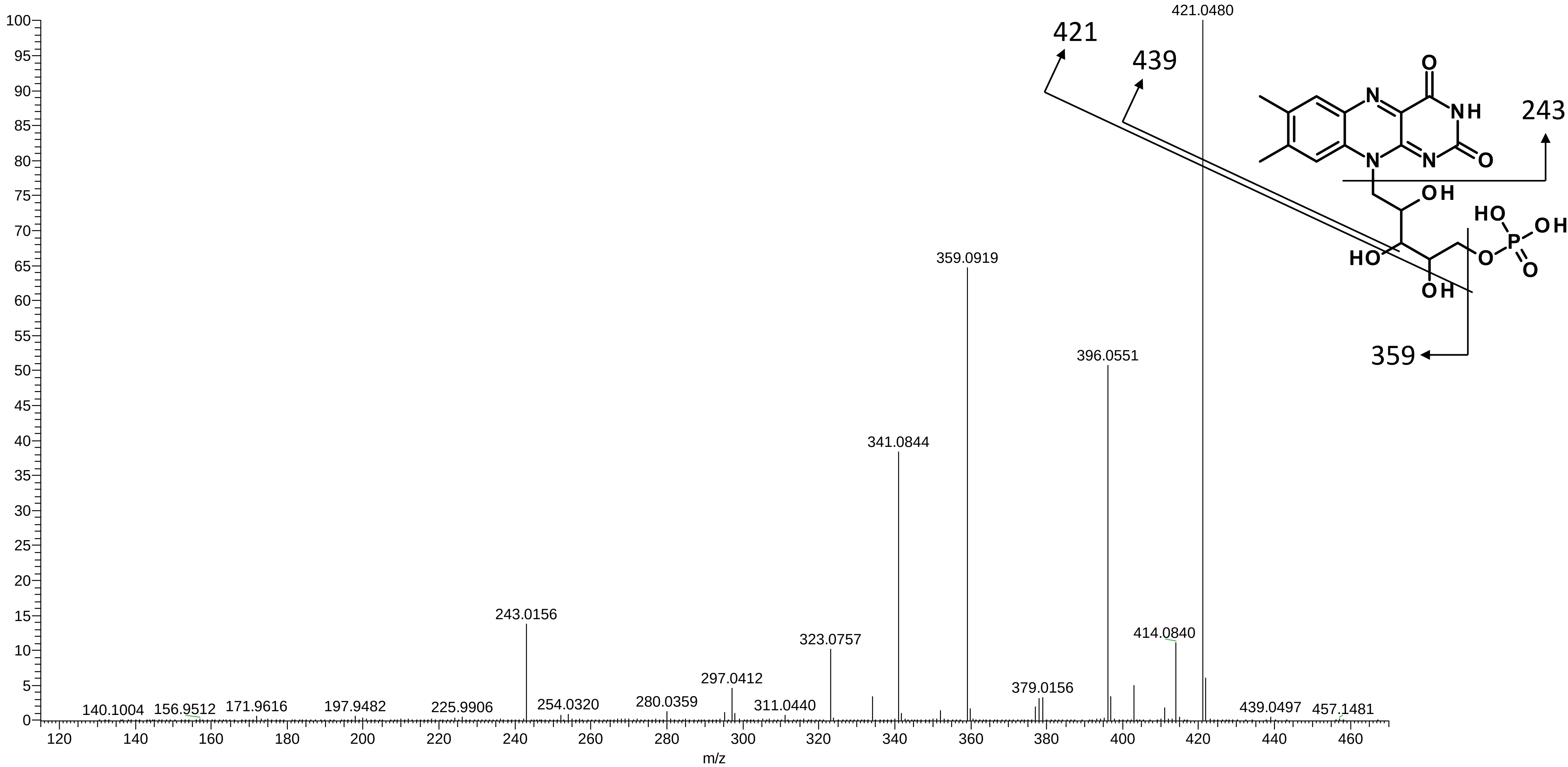

Leukopterin RT:4.48 min *m/z*:196.0465

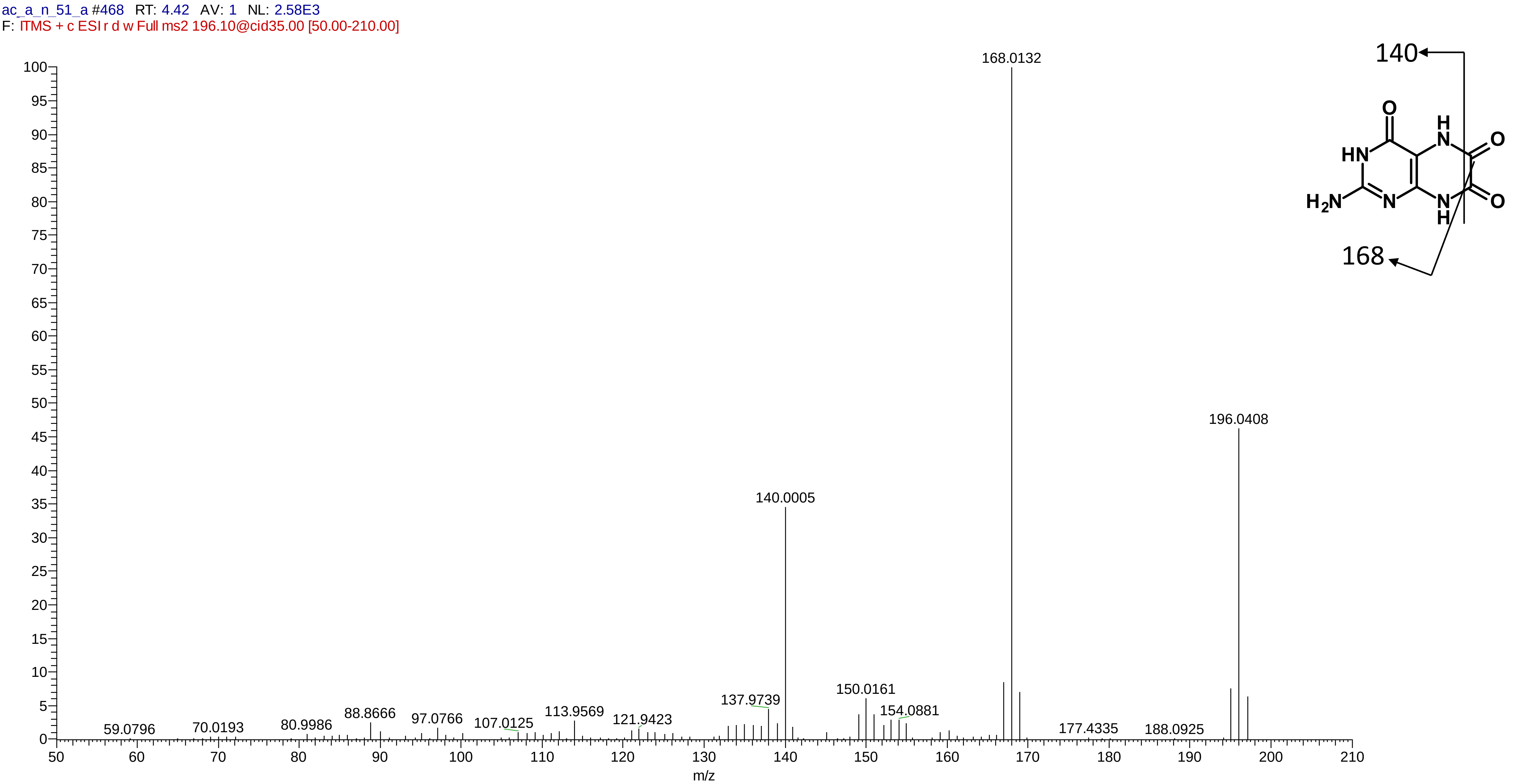

Adenosine RT:8.09 min *m/z*:268.104

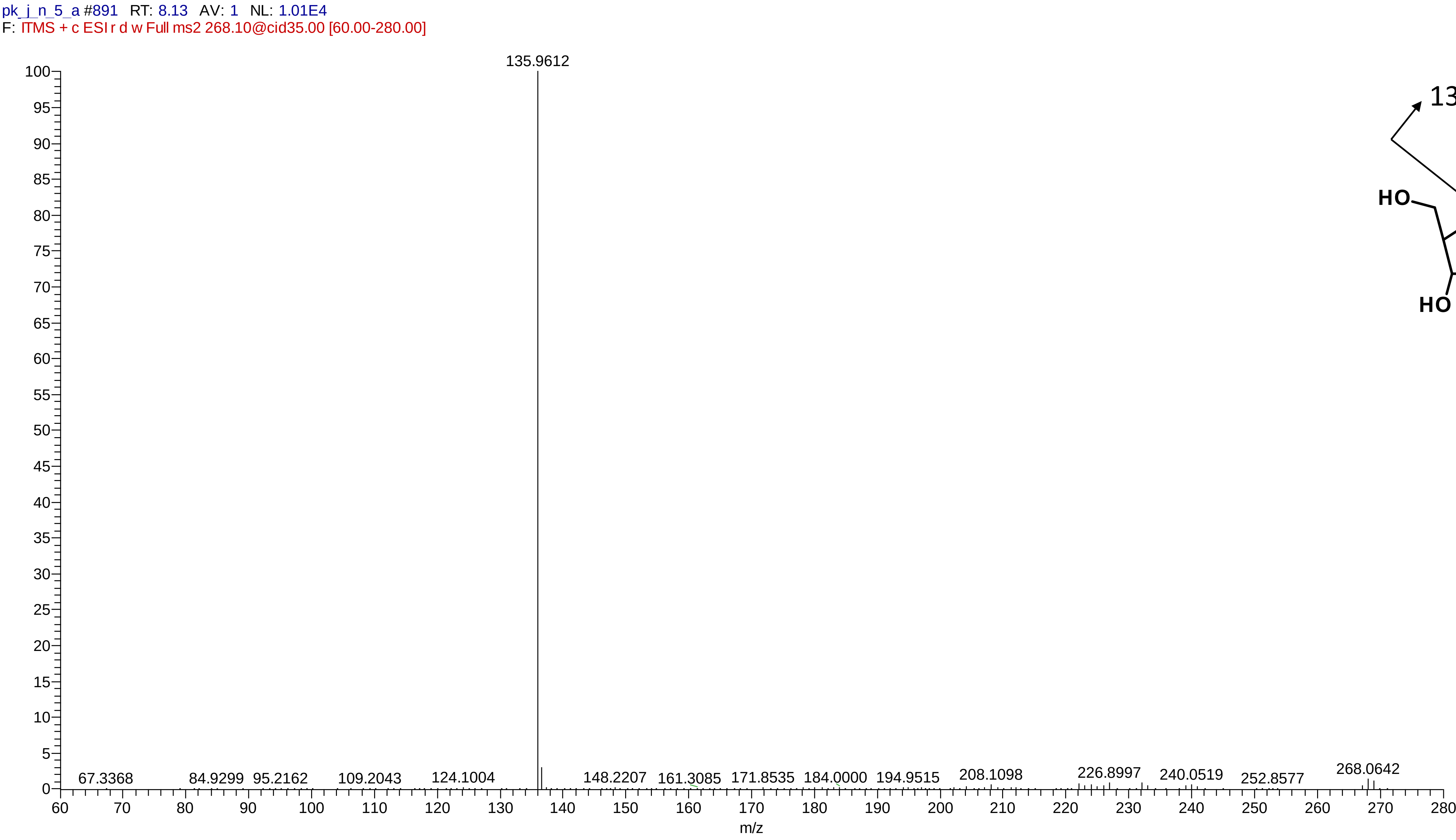

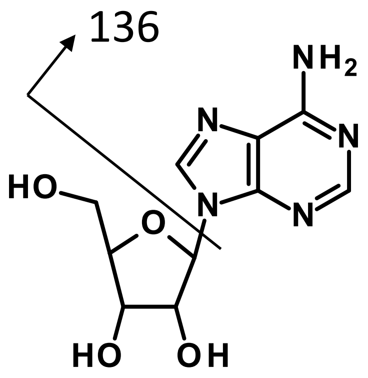

Adenosine monophosphate RT:3.30 min *m/z*:348.0704

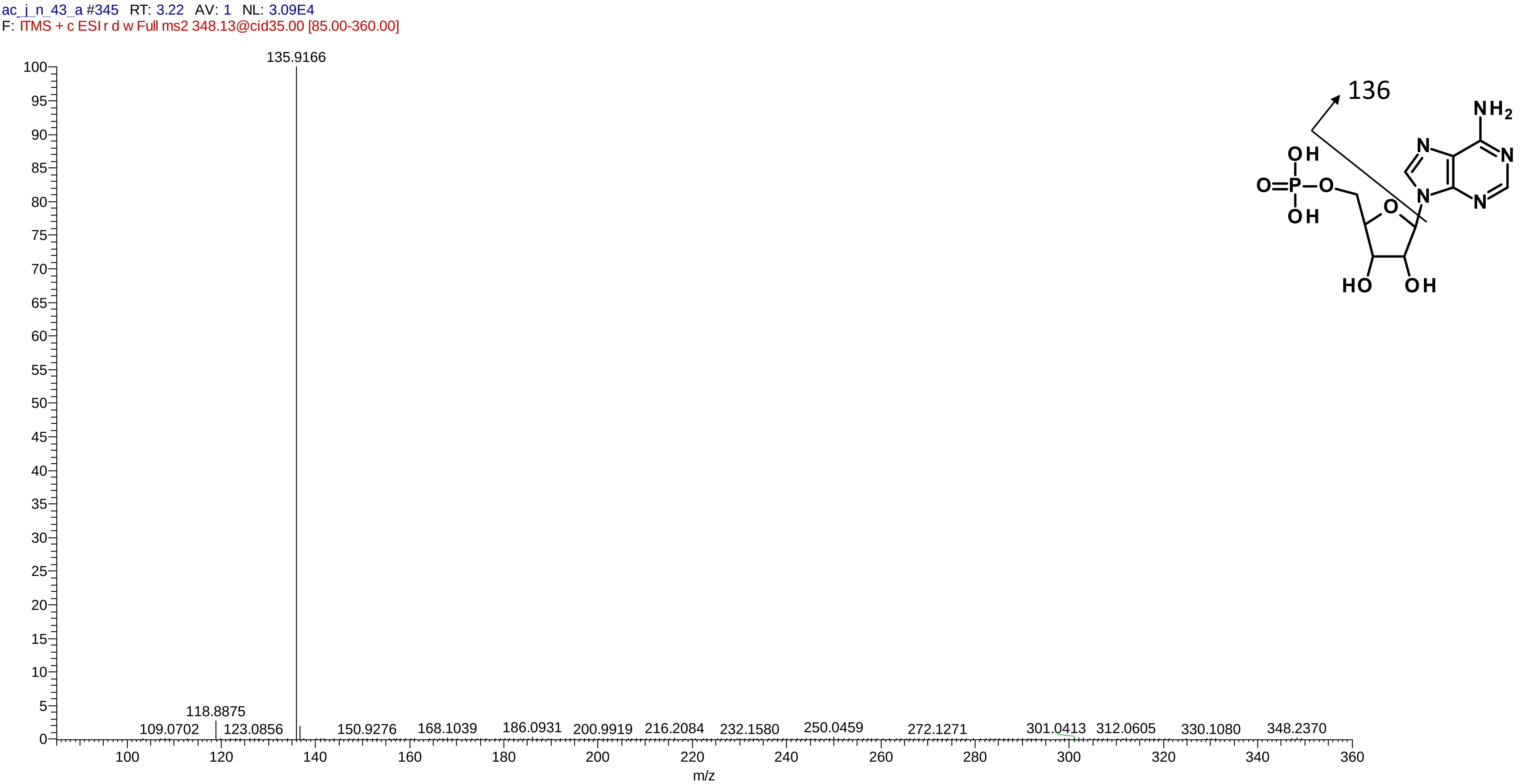

Guanosine RT:9.92 min *m/z*:284.0989

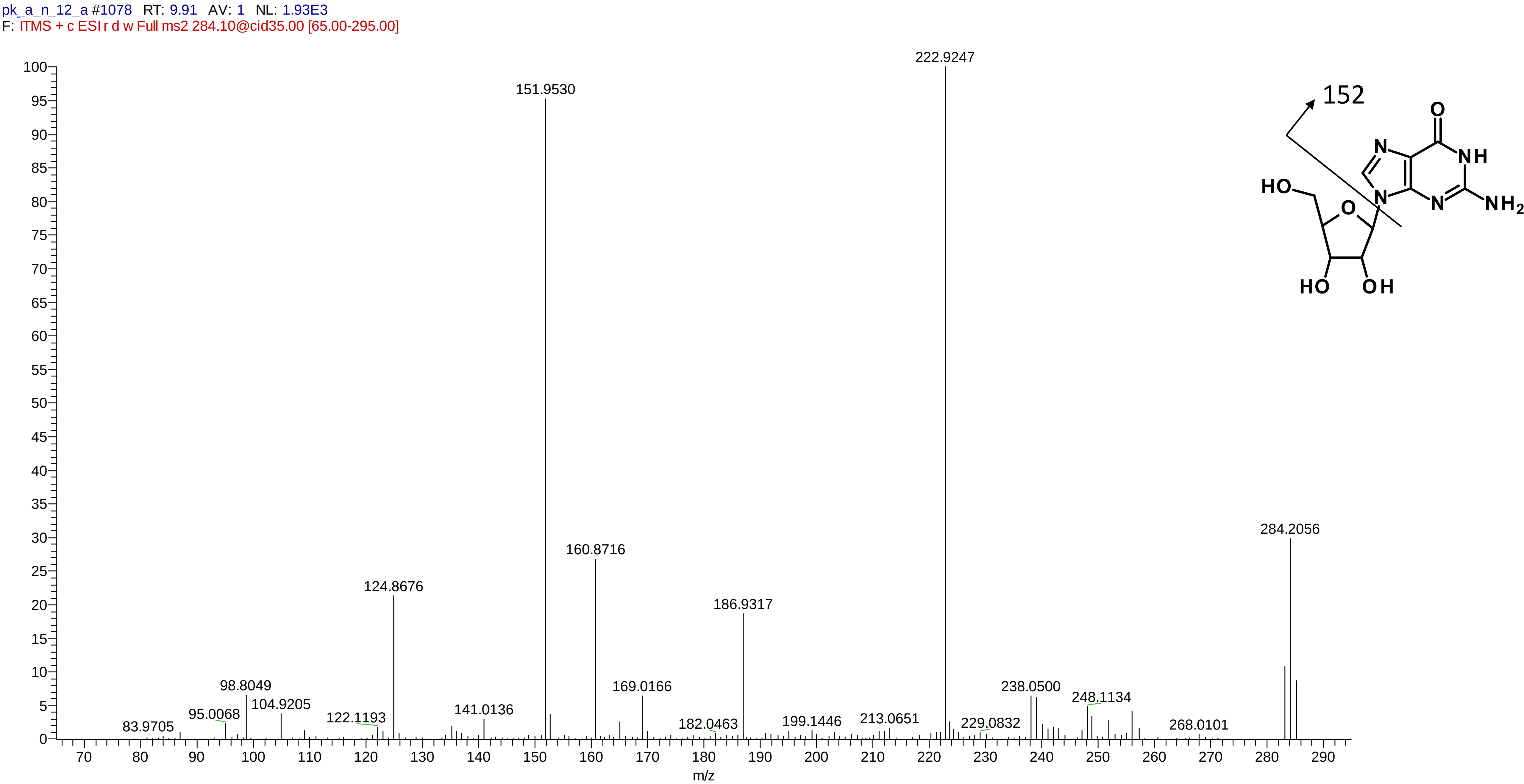

Cyclic ADP Ribose RT:4.07 min *m/z*:542.0666

pk_a_n_13_a #495 RT: 4.14 AV: 1 NL: 1.48E3
F: ITMS + c ESI r d w Full ms2 542.07@cid35.00 [135.00-555.00]

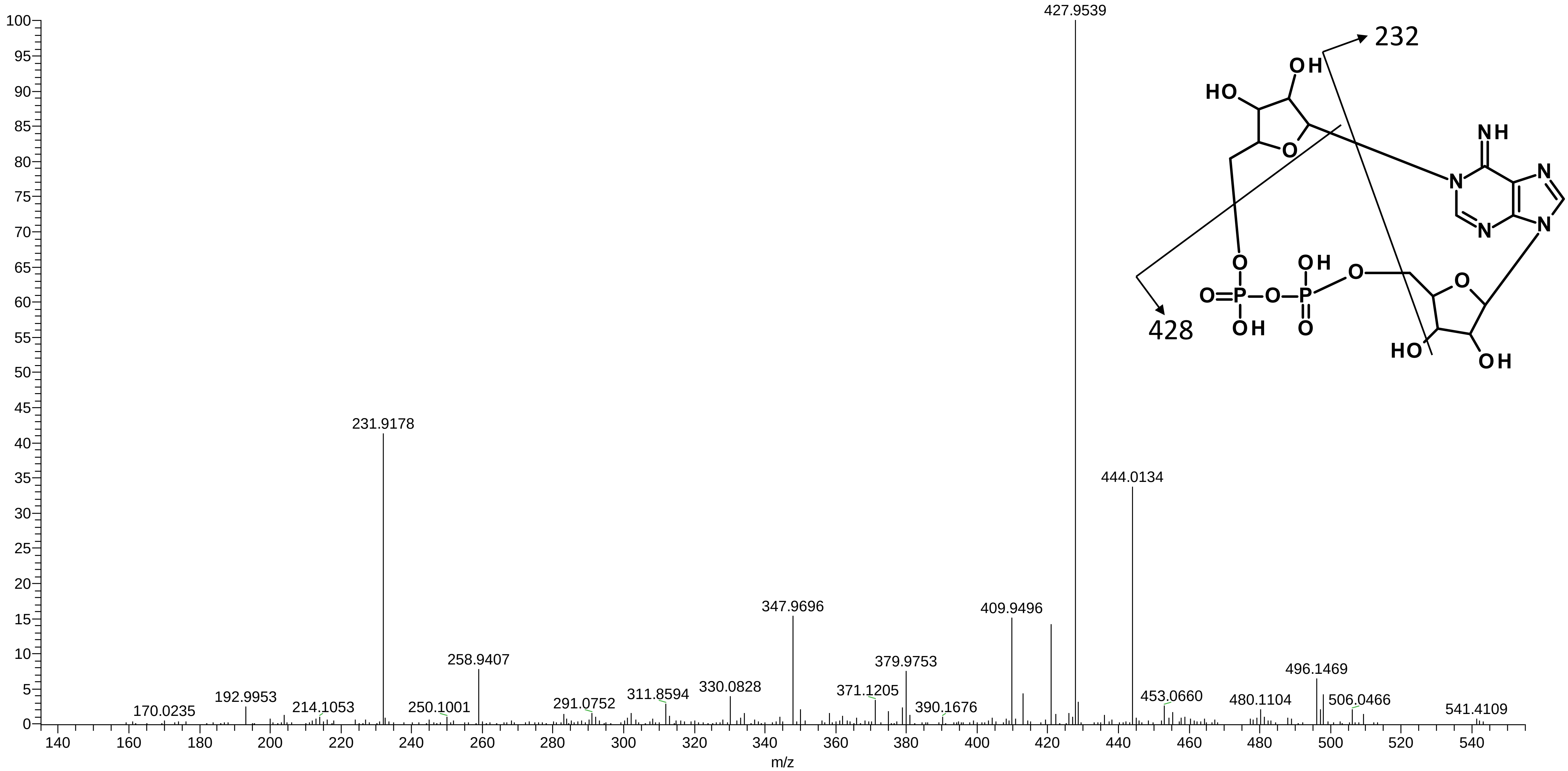

S-methyl-5'-thioadenosine RT:15.96 min *m/z*:298.0973

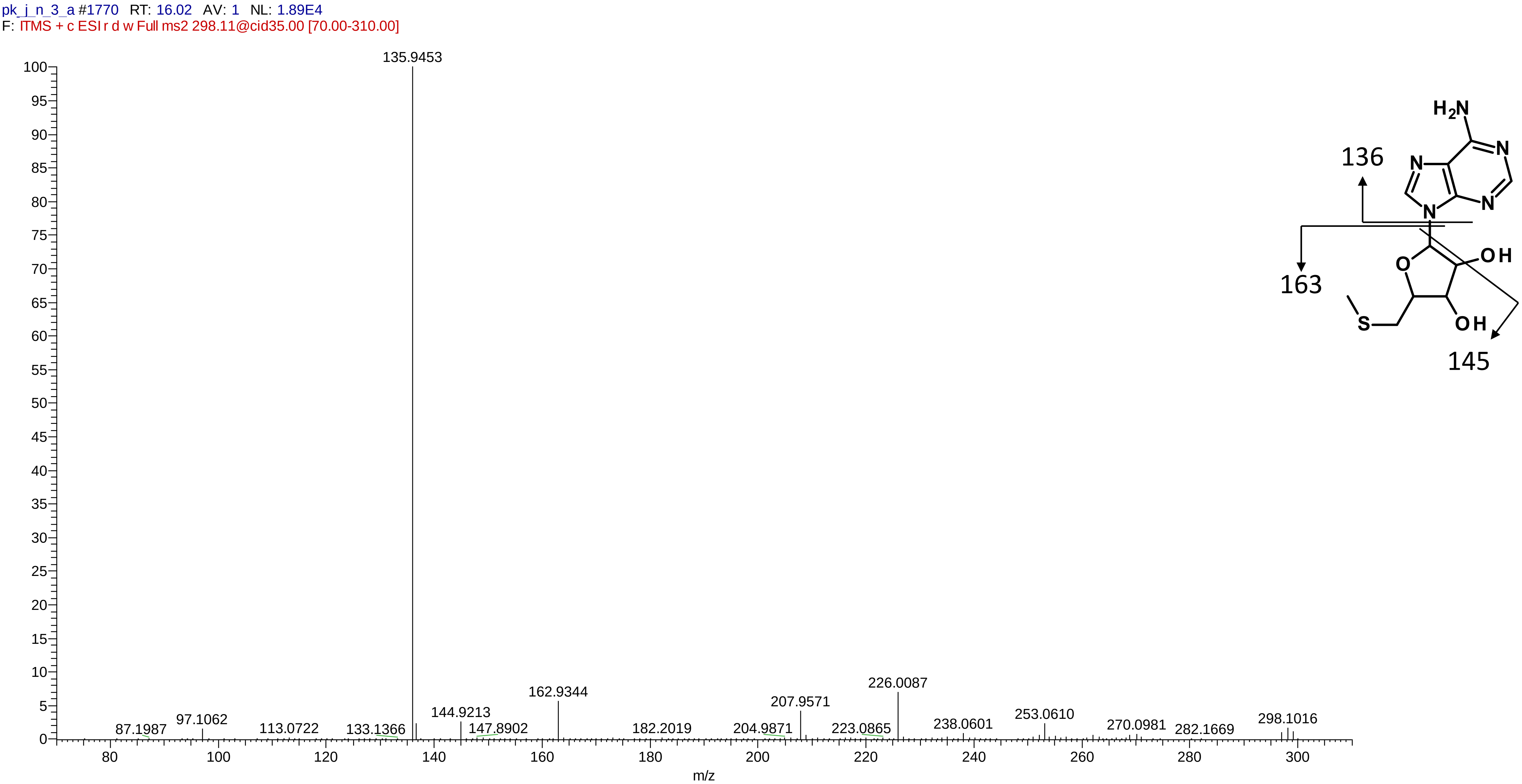

S-methyl-5'-thioadenosine RT:3.14 min *m/z*:298.0968

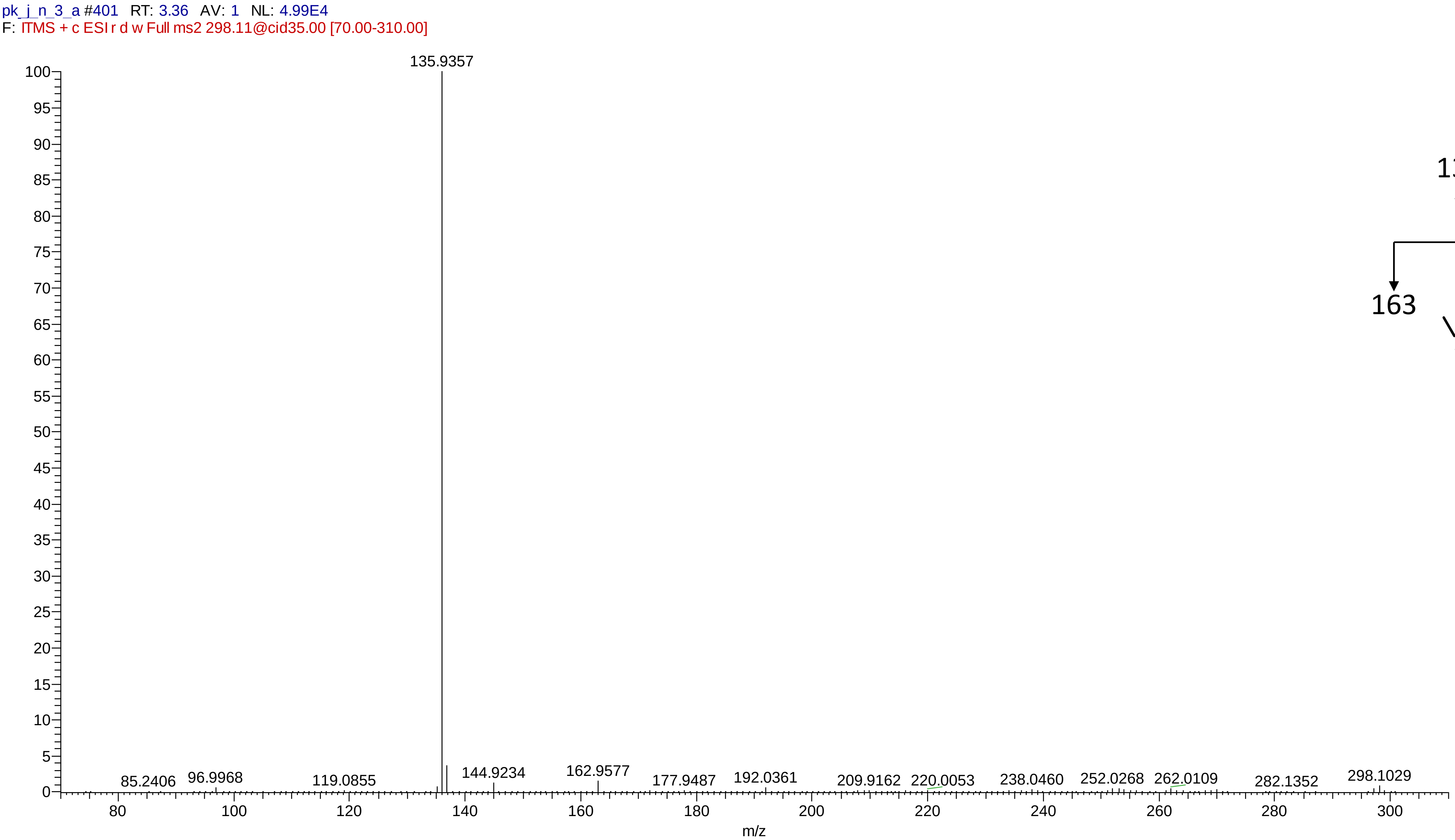

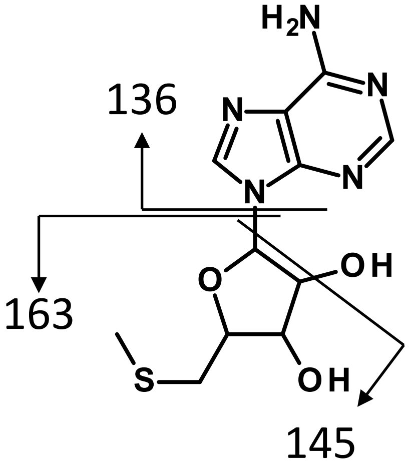

Zeatin glucoside RT:14.96 min *m/z*:382.1721

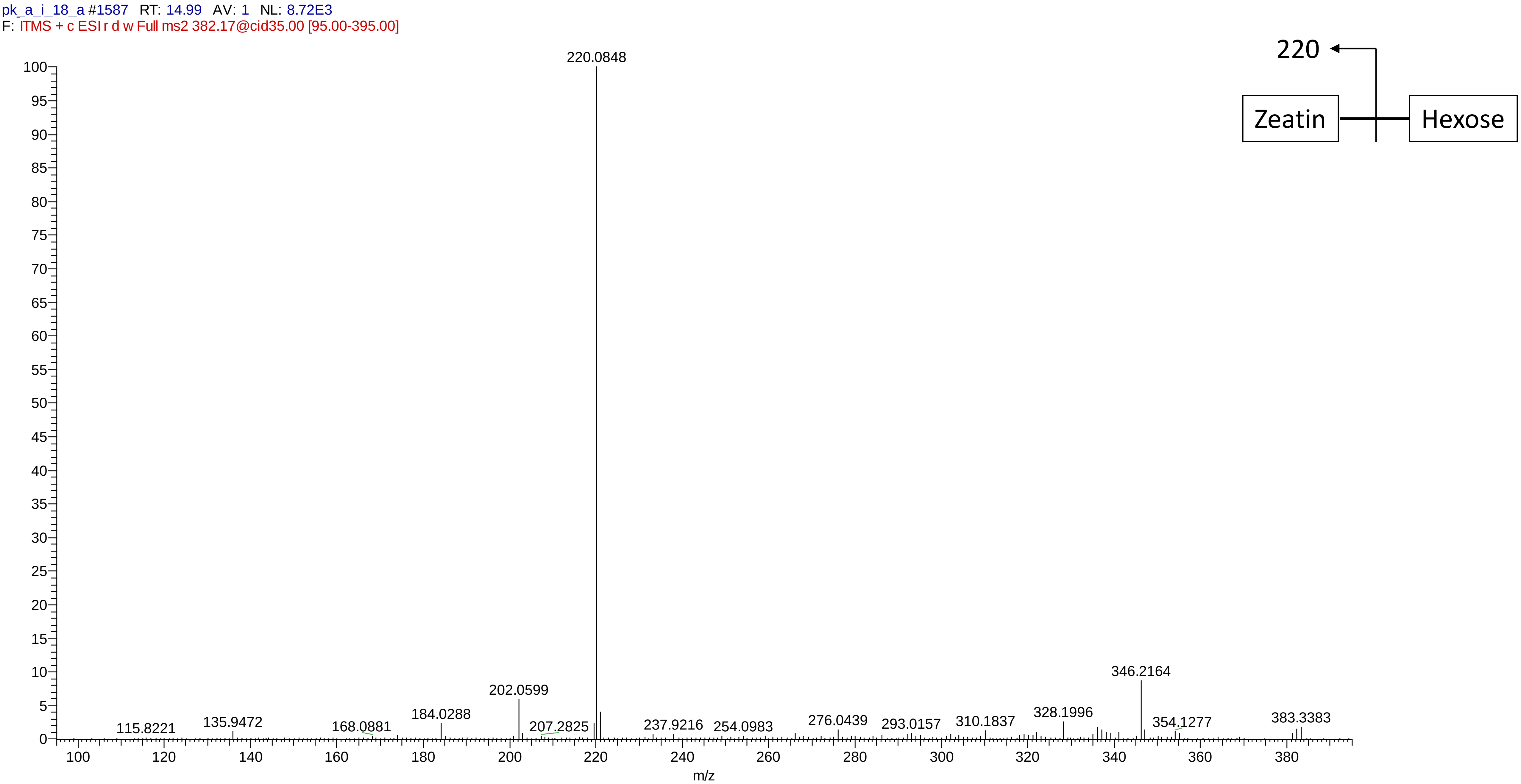

Disaccharide RT:2.63 min *m/z*:343.1235

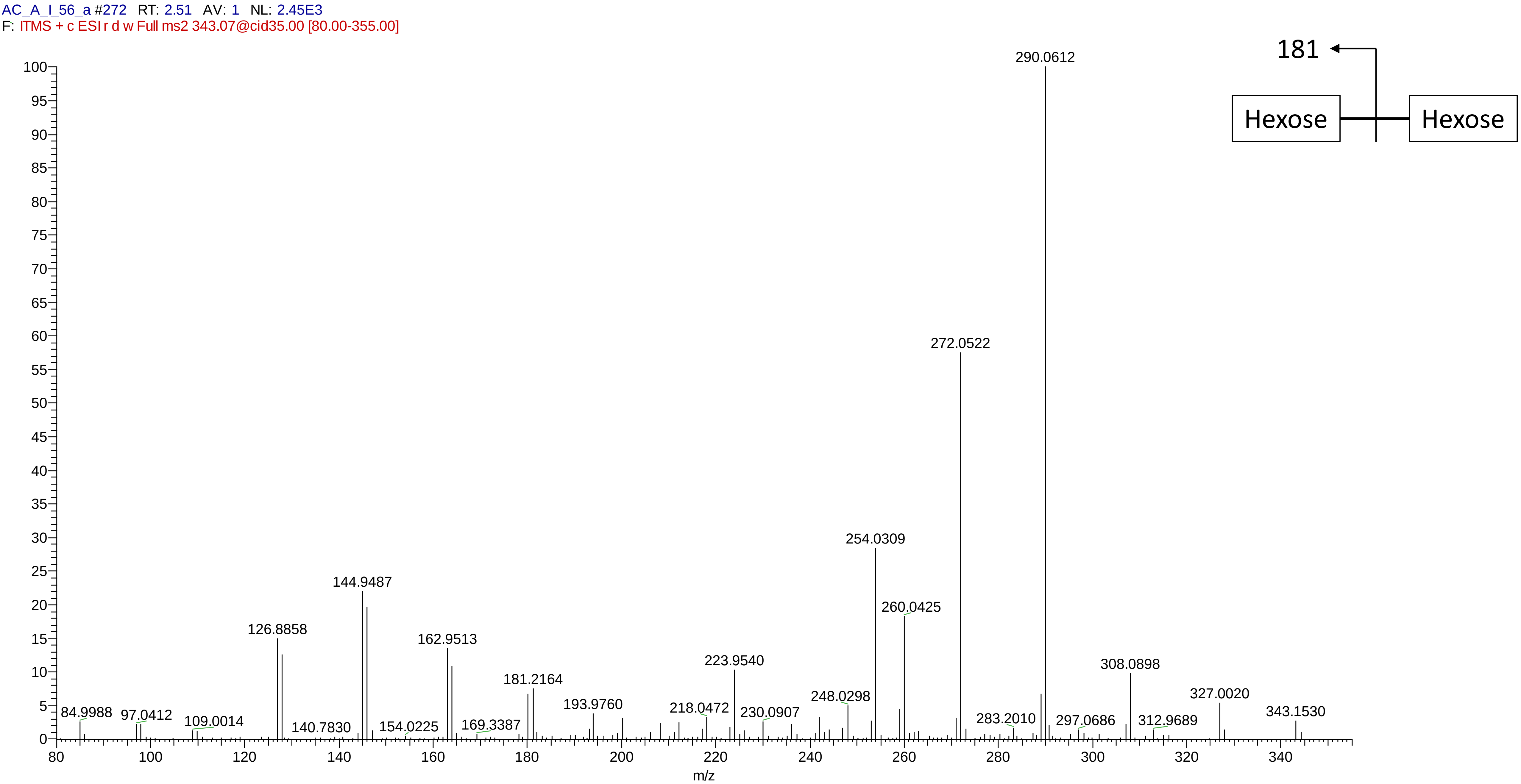

N-Acetylmuramic acid RT:3.95 min *m/z*:294.1183

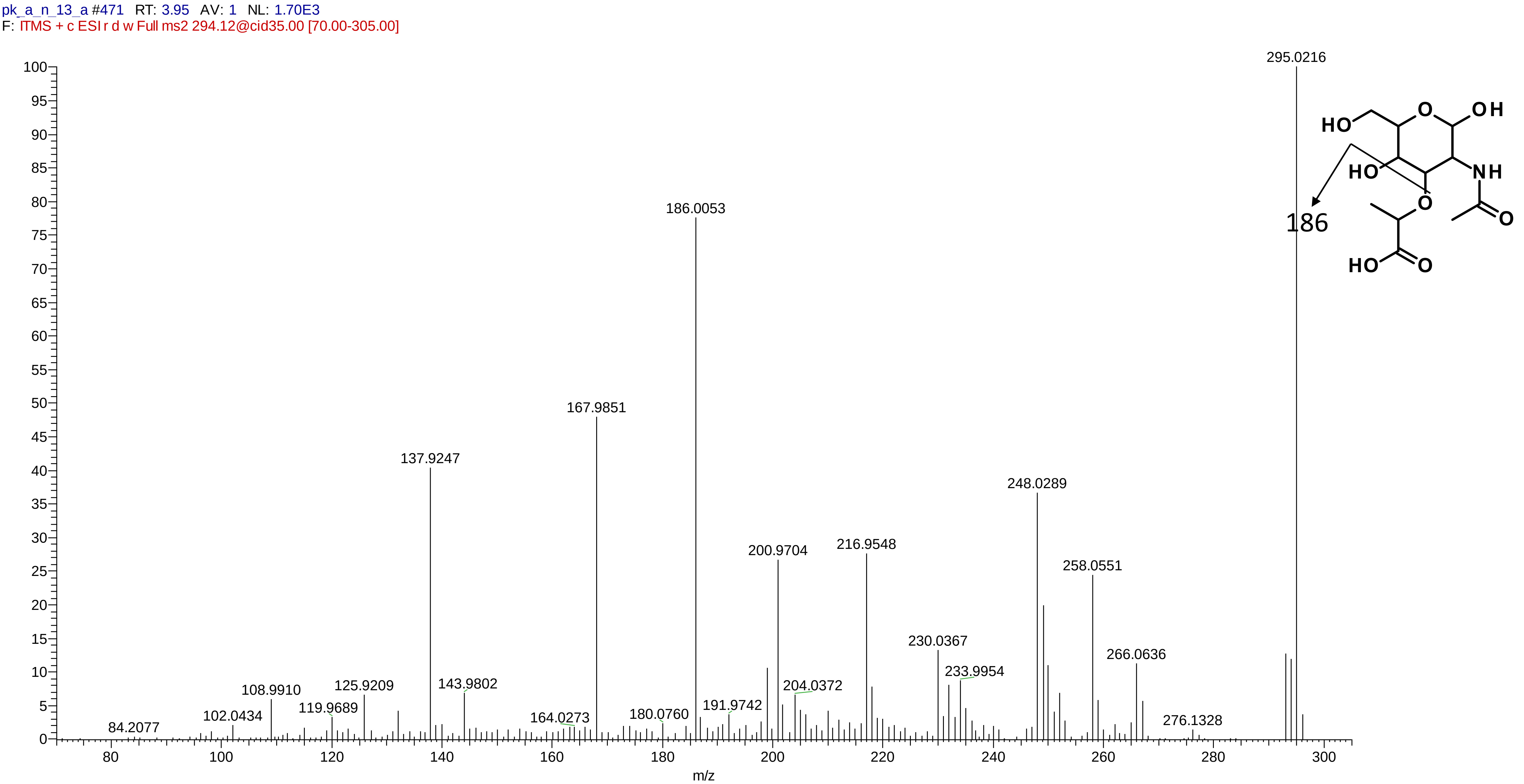

N-Acetylmuramic acid RT:6.49 min *m/z*:294.1183

pk_a_n_13_a #752 RT: 6.41 AV: 1 NL: 4.12E3
F: ITMS + c ESI r d w Full ms2 294.12@cid35.00 [70.00-305.00]

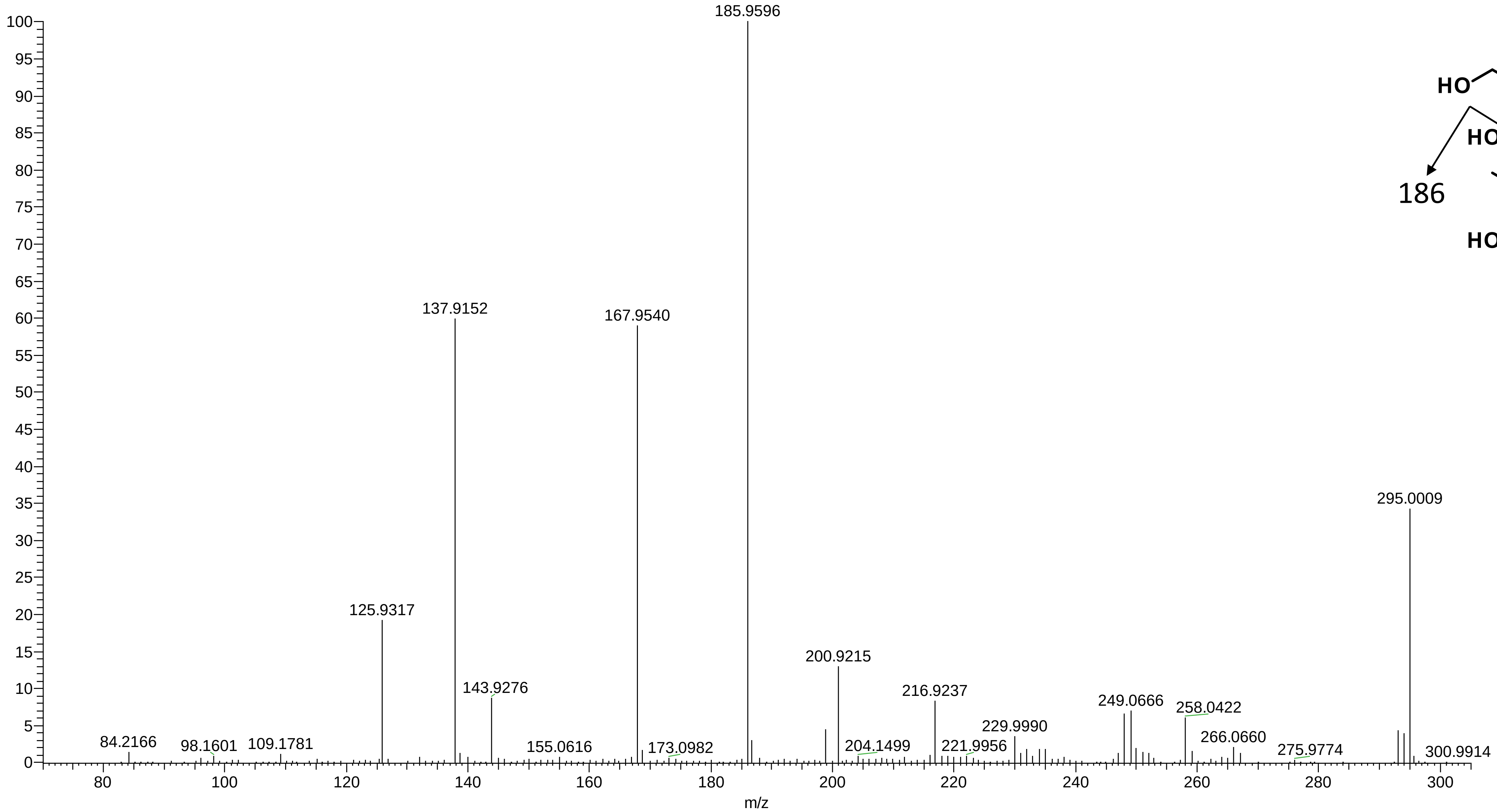

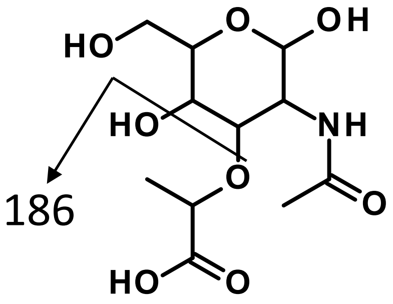

Putative aminosugar RT:13.96 min *m/z*:647.2878

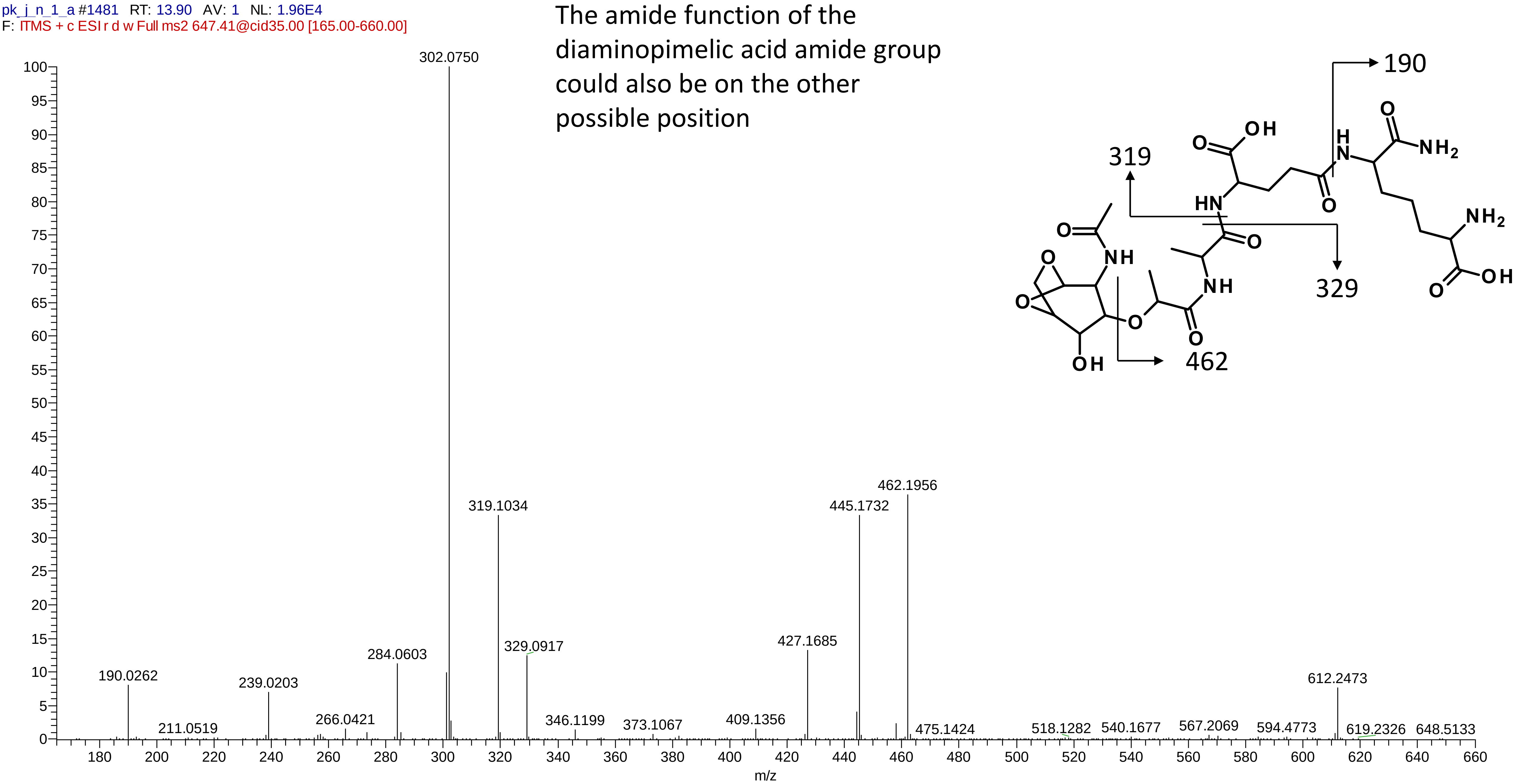

The amide function of the diaminopimelic acid amide group could also be on the other possible position

Putative aminosugar RT:14.31 min *m/z*:648.2719

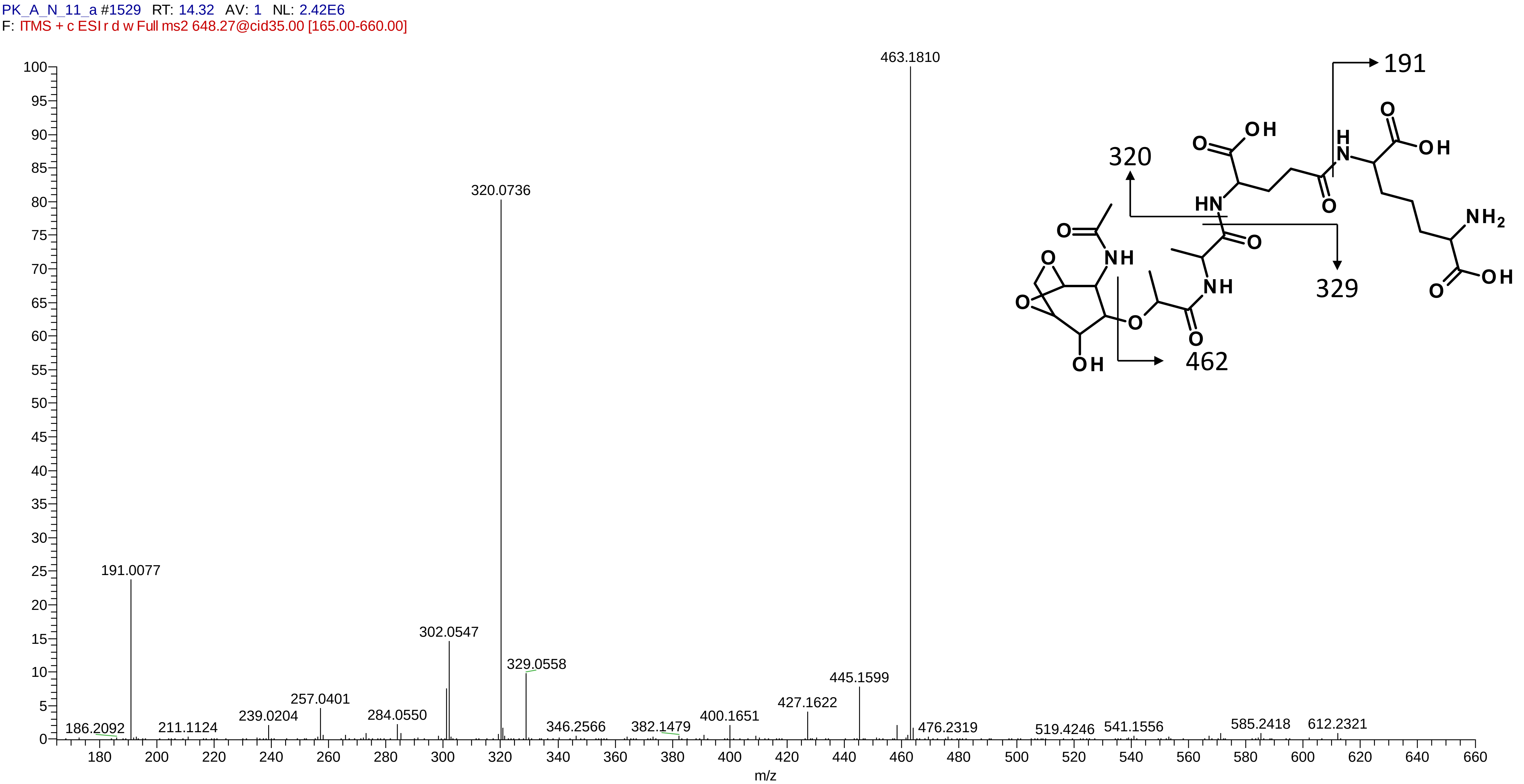

Putative aminosugar RT:14.38 min *m/z*:850.3661

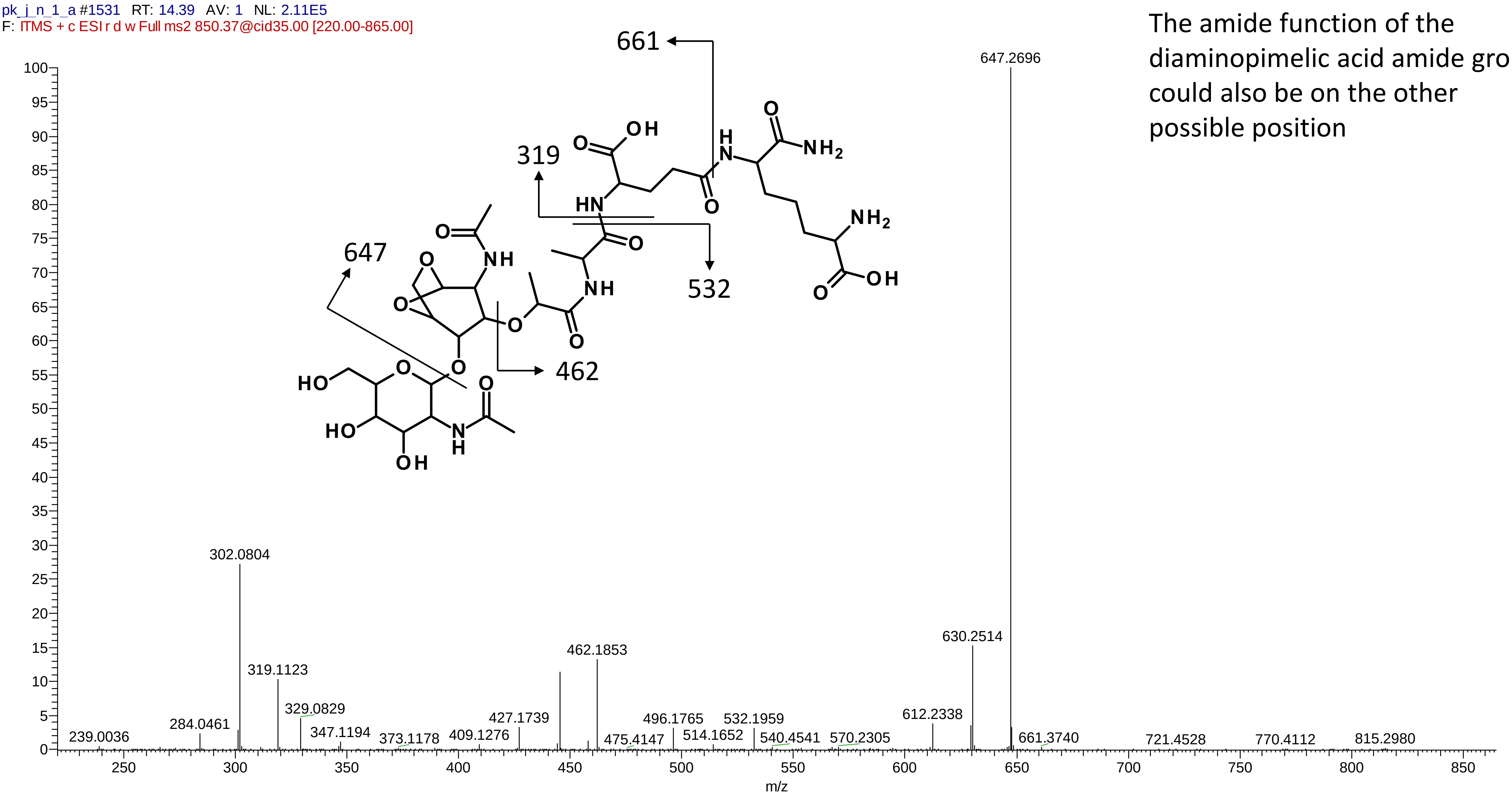

The amide function of the diaminopimelic acid amide group could also be on the other possible position

Putative aminosugar RT:14.47 min *m/z*:851.3517

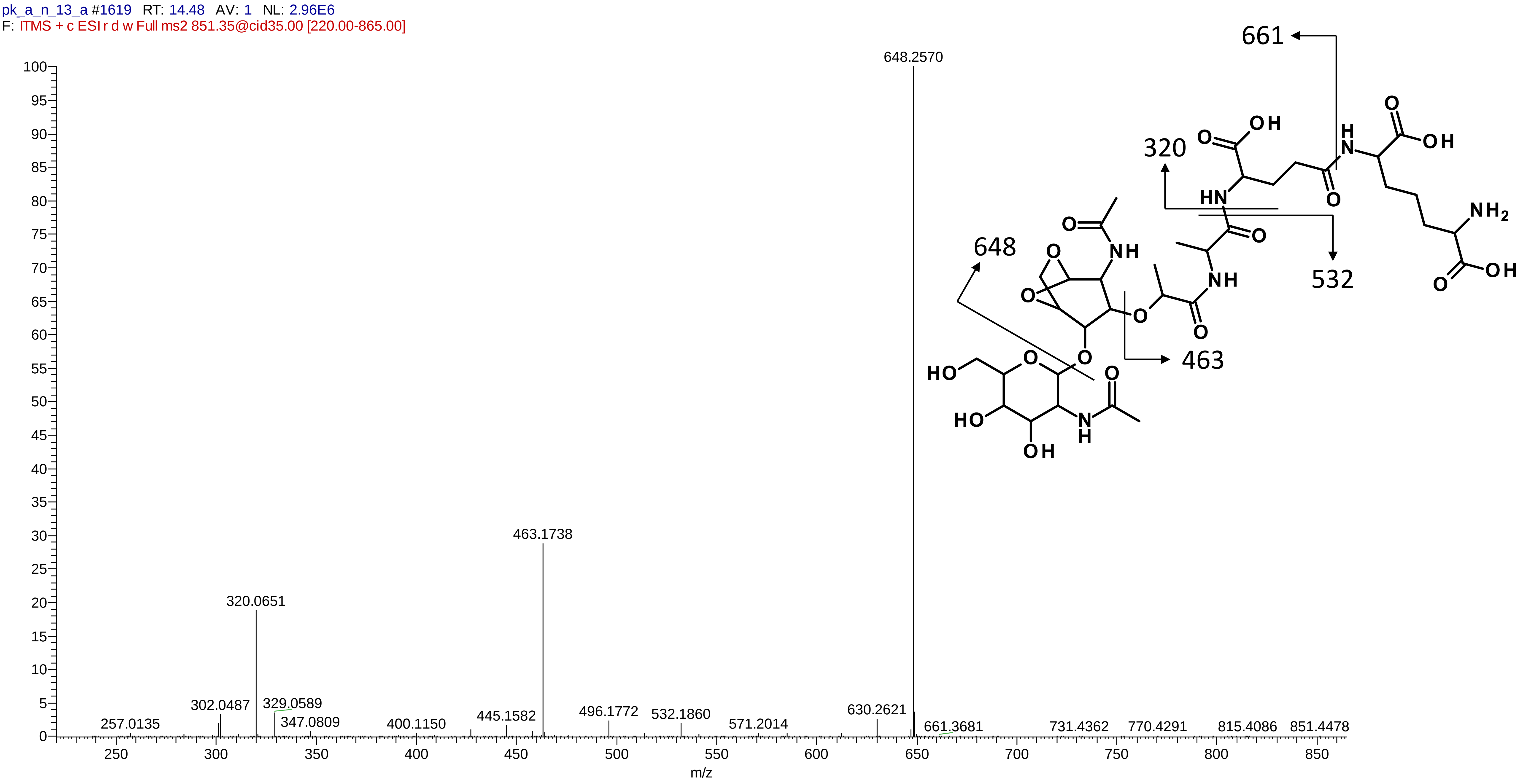

Putative aminosugar RT:15.24 min *m/z*:689.2983

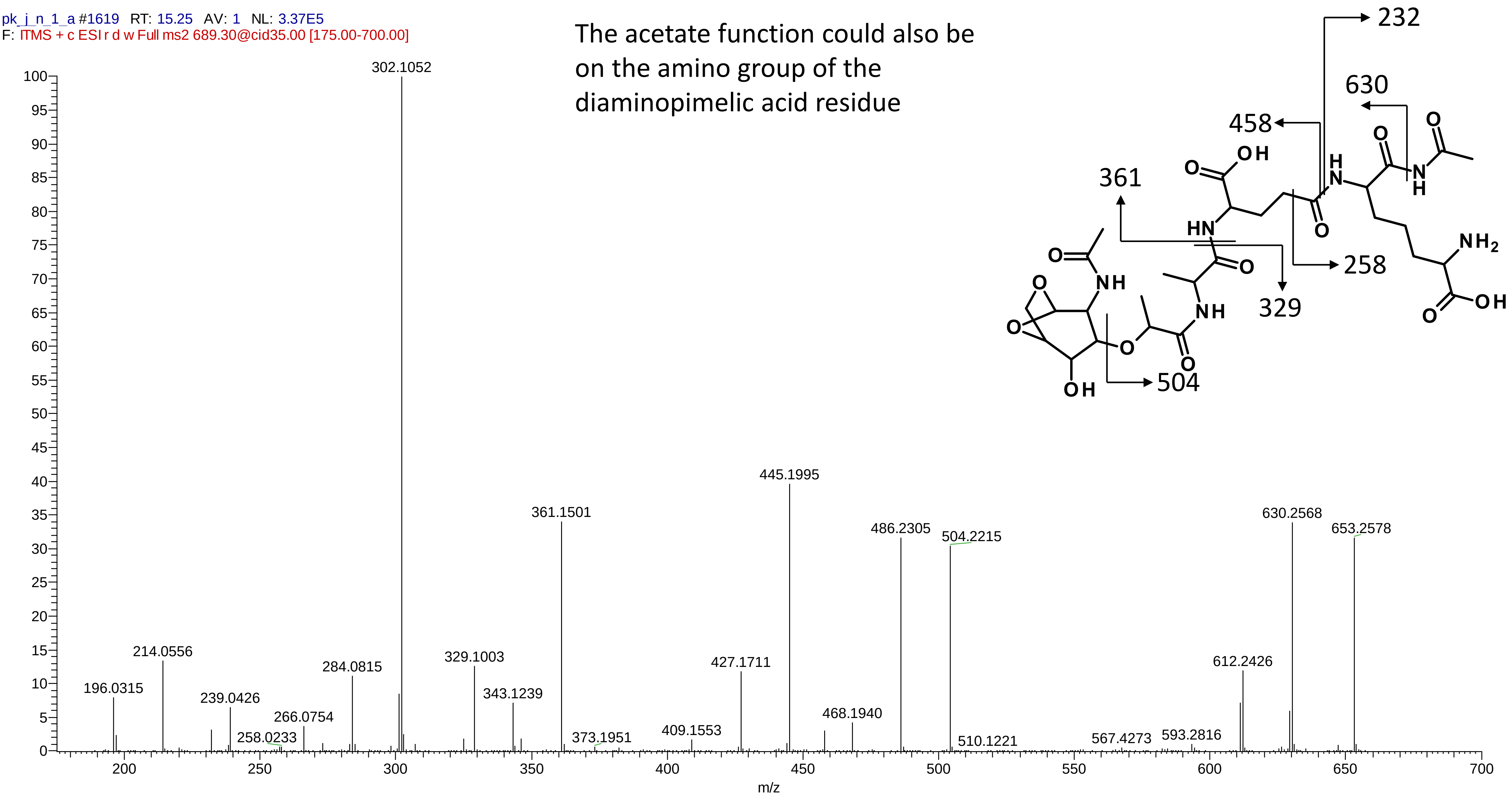

The acetate function could also be on the amino group of the diaminopimelic acid residue

Putative aminosugar RT:15.31 min *m/z*:719.3095

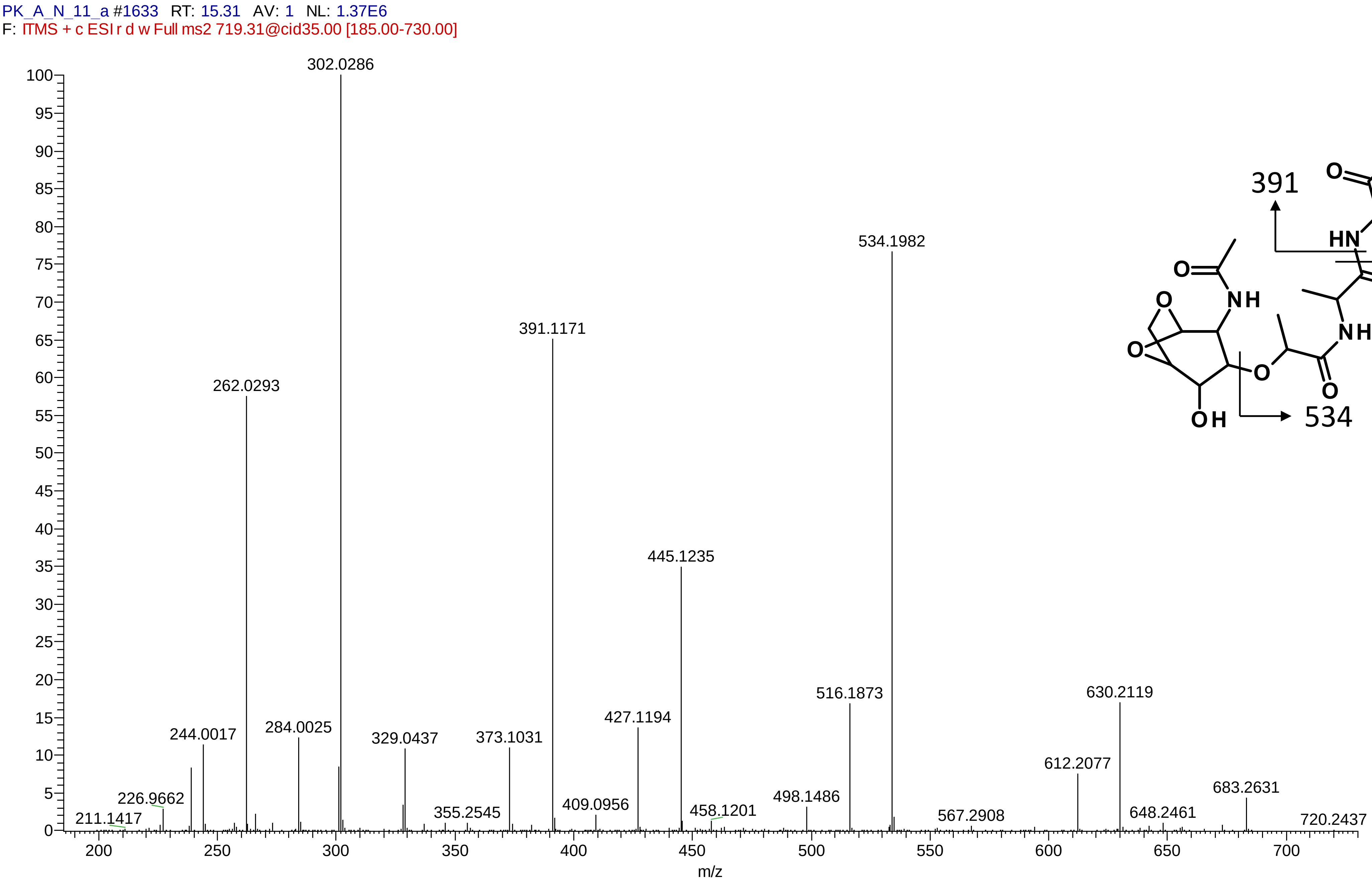

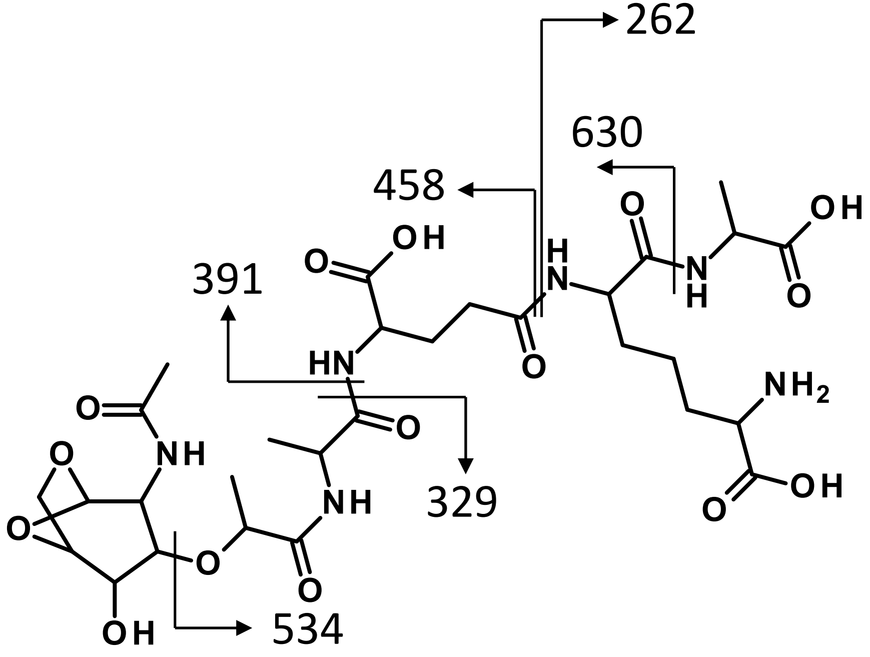

Putative aminosugar RT:15.31 min *m/z*:922.3883

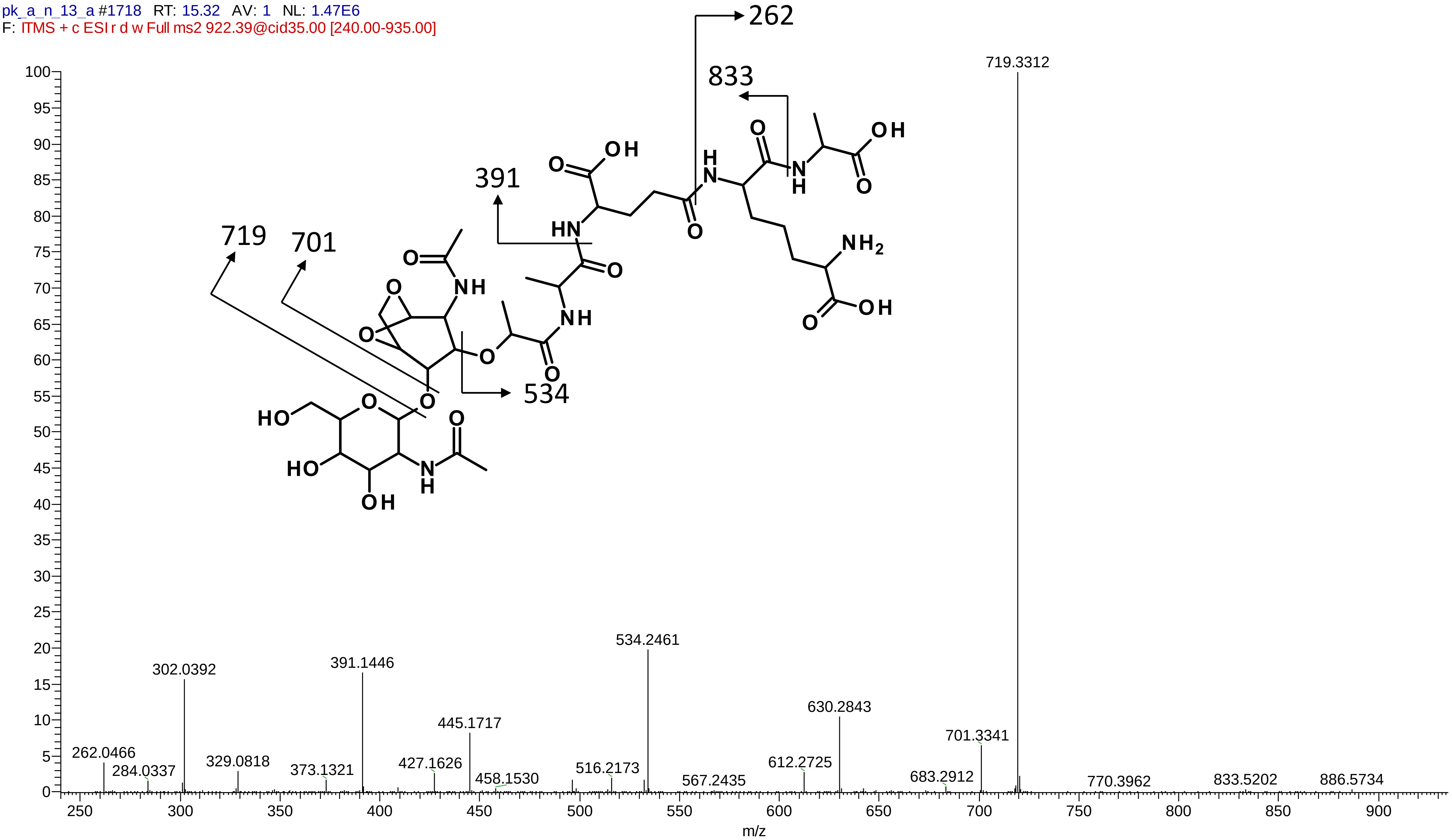

Putative aminosugar RT:15.56 min *m/z*:892.3788

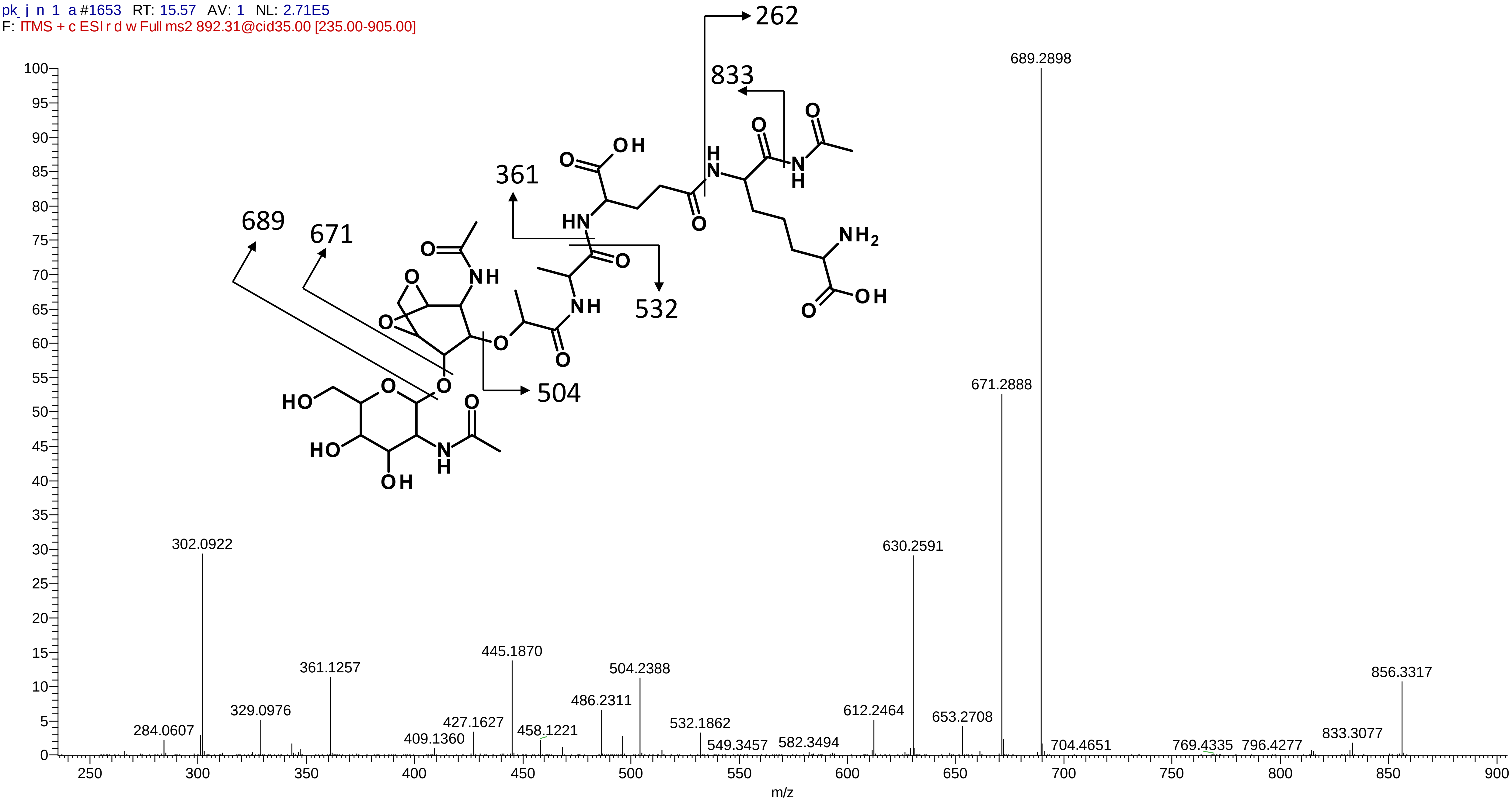

Putative aminosugar RT:15.74 min *m/z*:790.3464

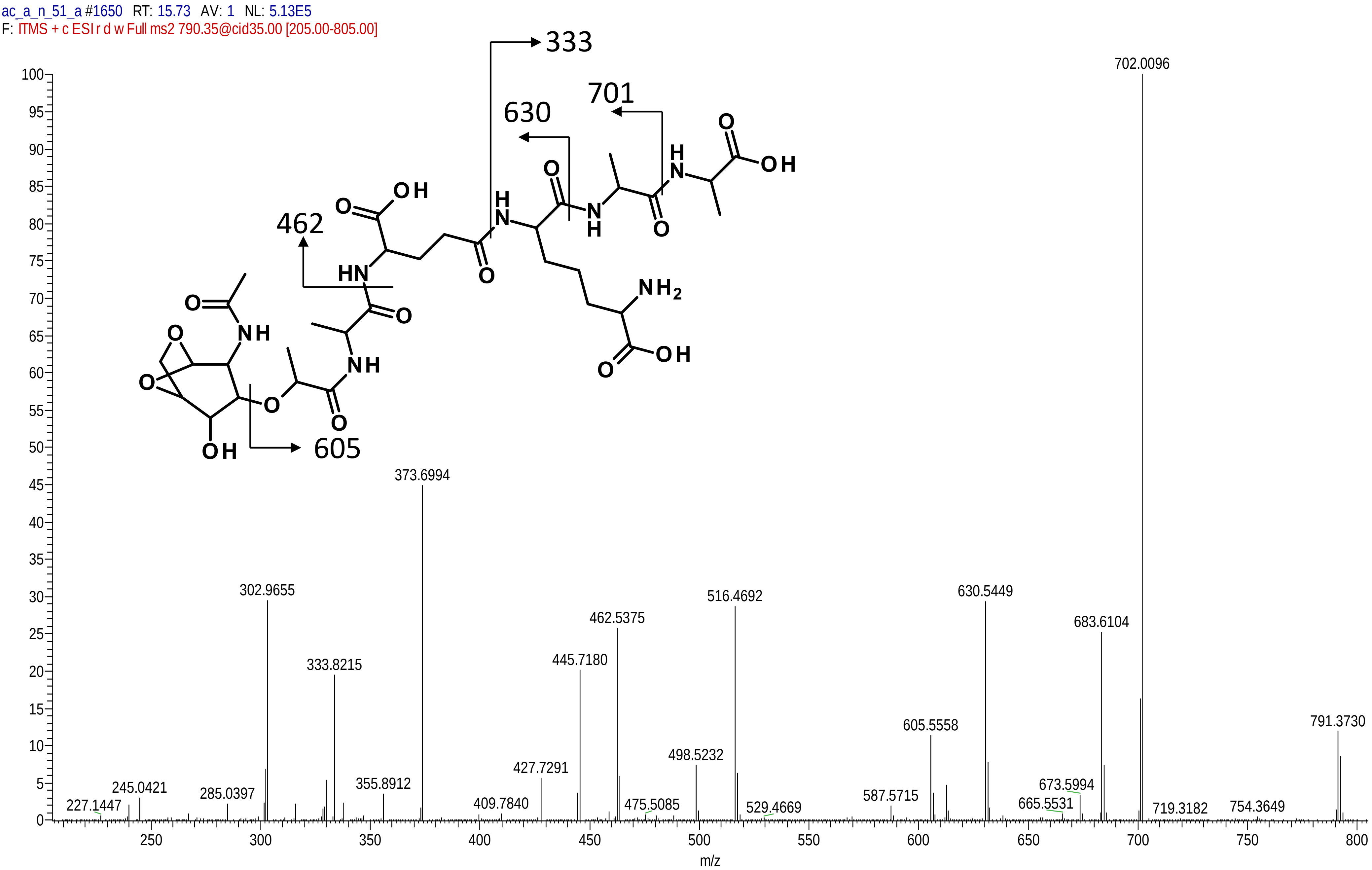

Putative aminosugar RT:15.83 min *m/z*:993.4261

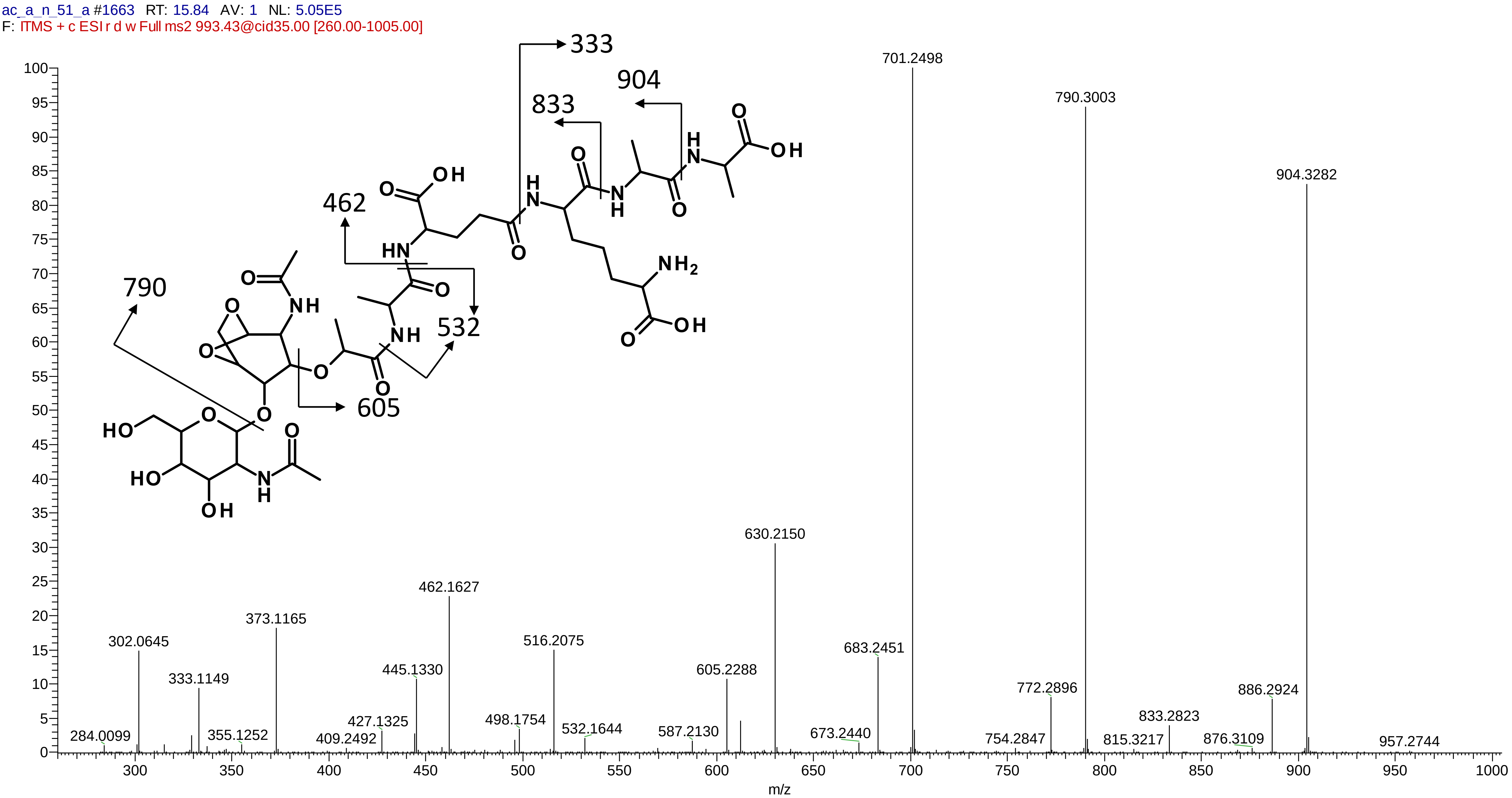

Putative aminosugar RT:16.28 min *m/z*:680.2769

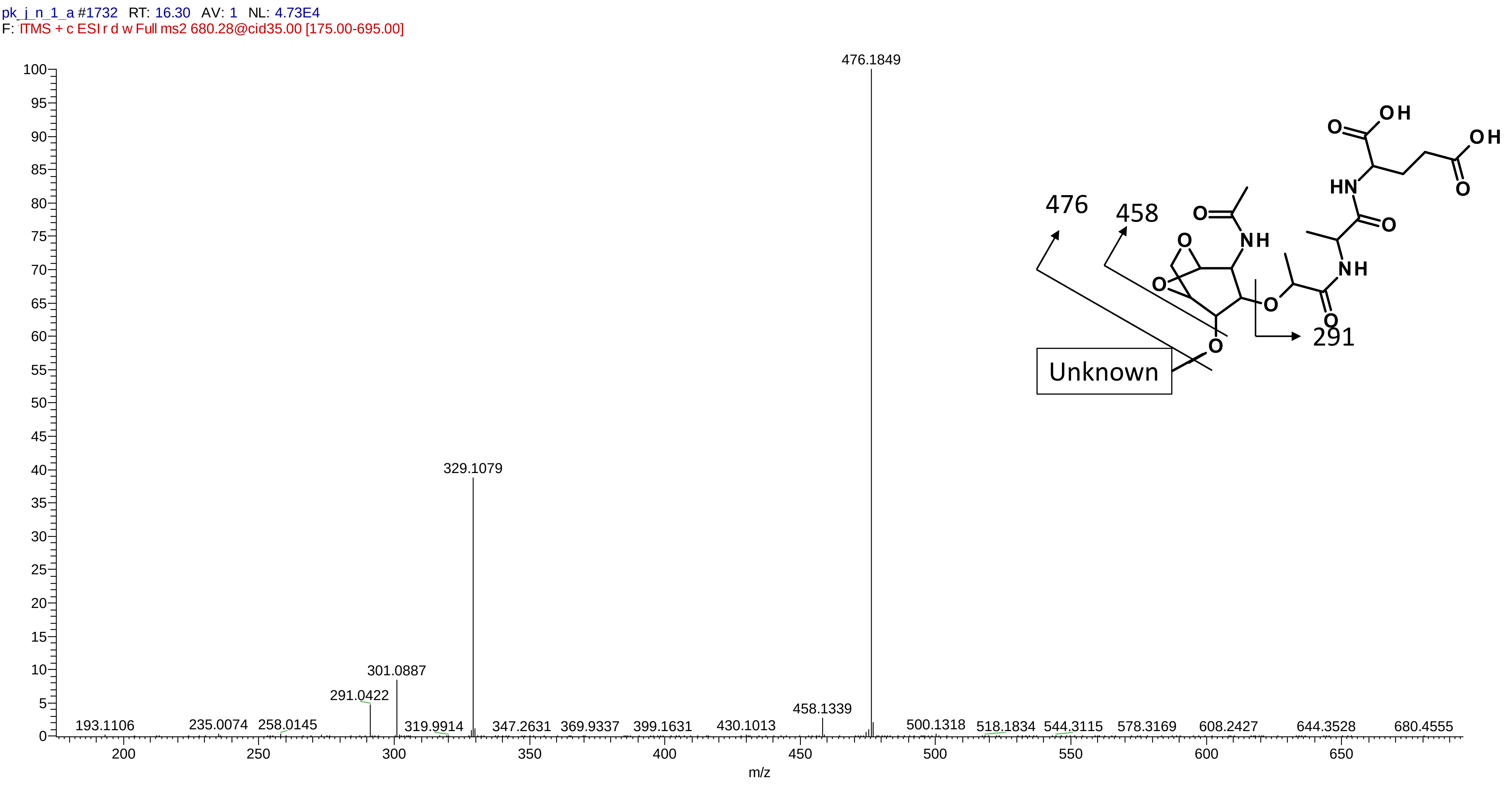

Putative aminosugar RT:16.29 min *m/z*:476.1871

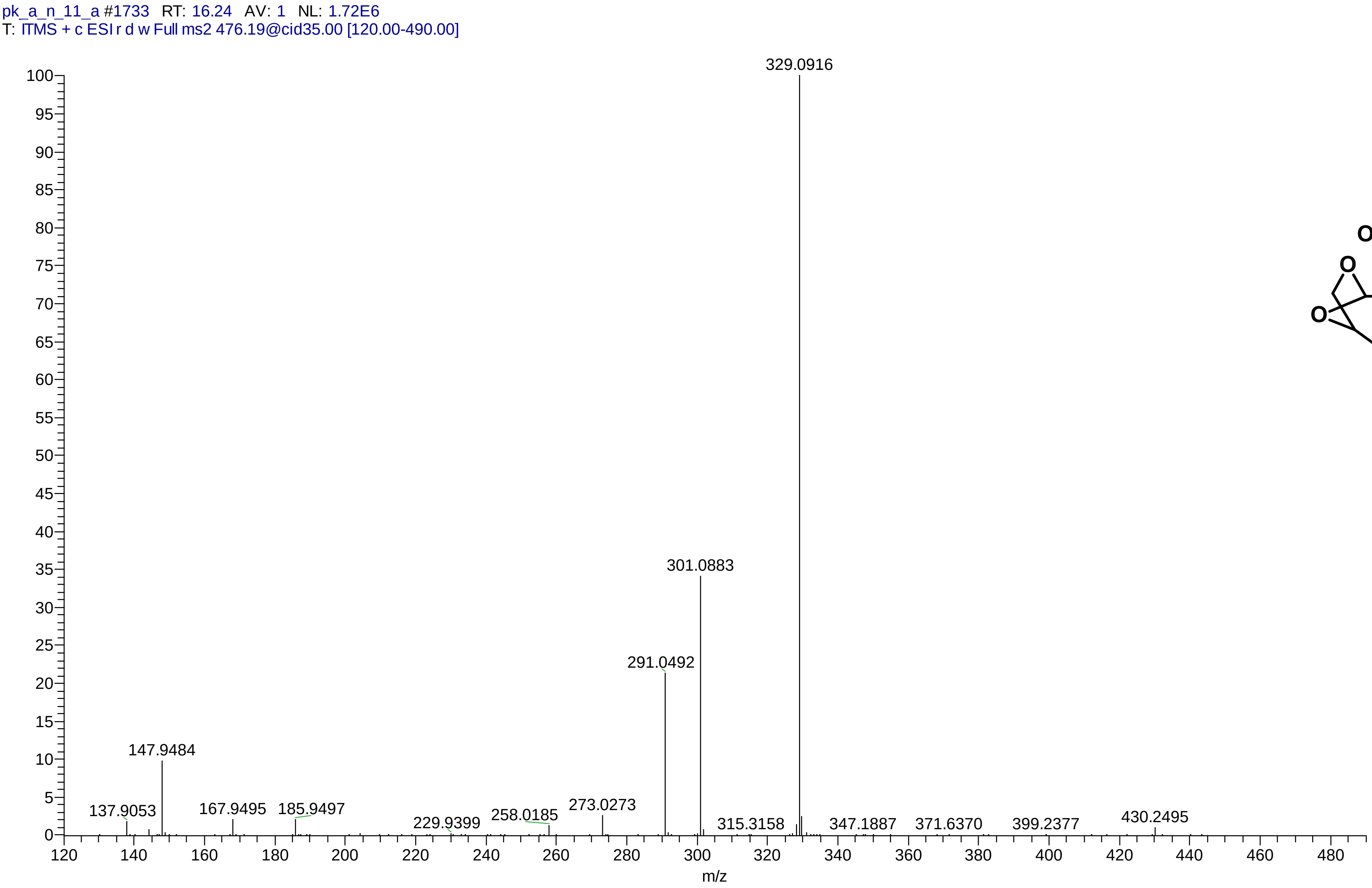

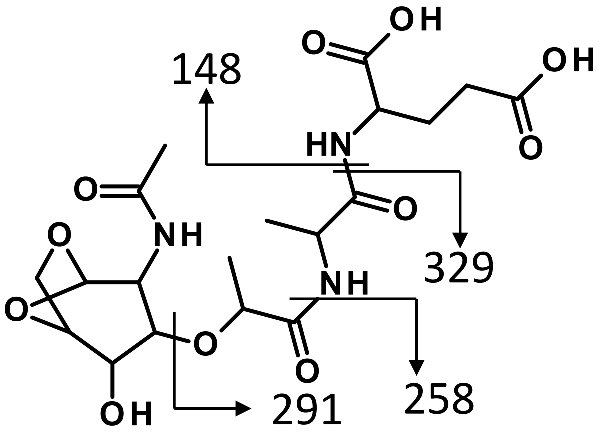

Putative aminosugar RT:16.36 min *m/z*:679.2664

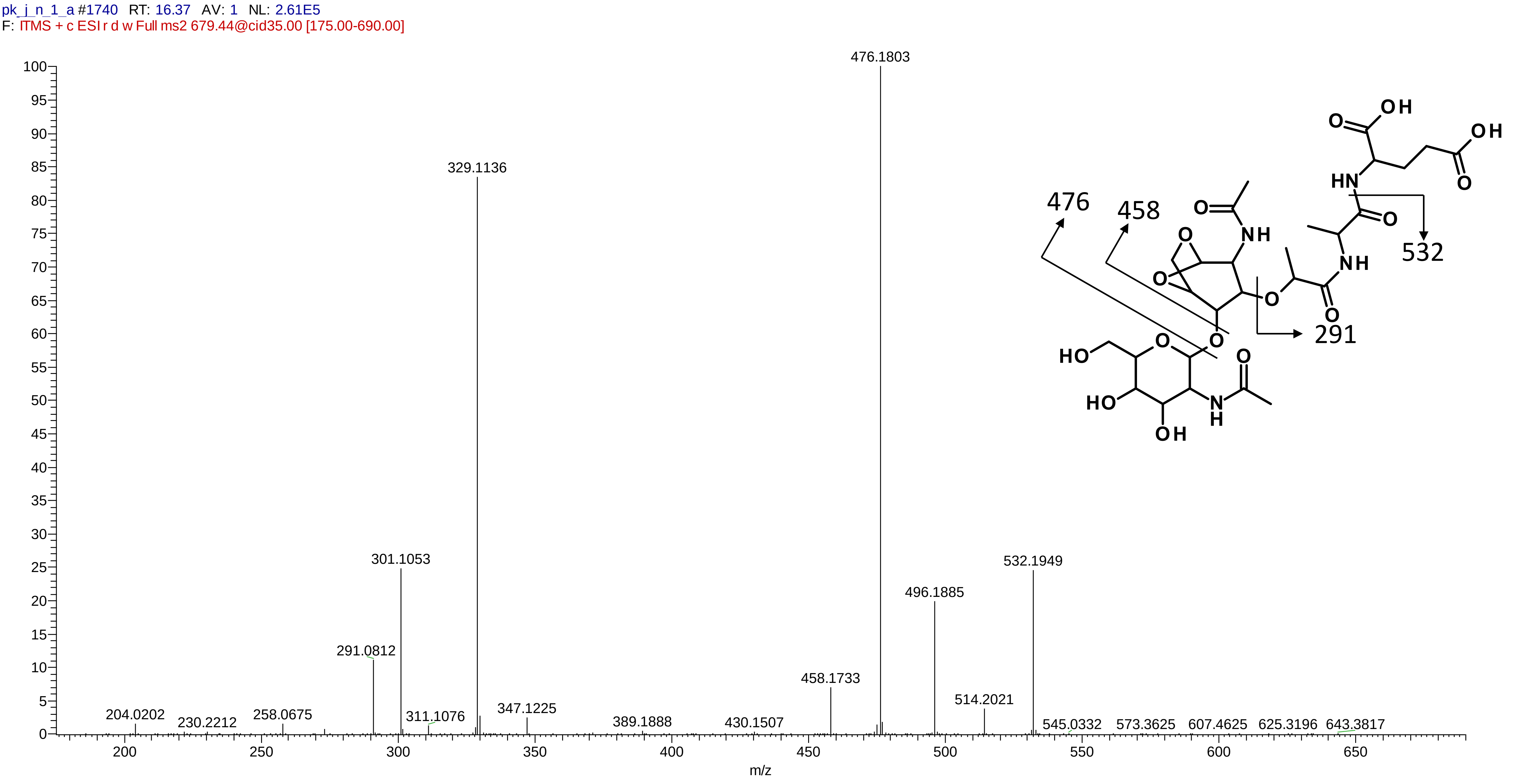

FR900359 RT:48.38 min *m/z*:1002.5394

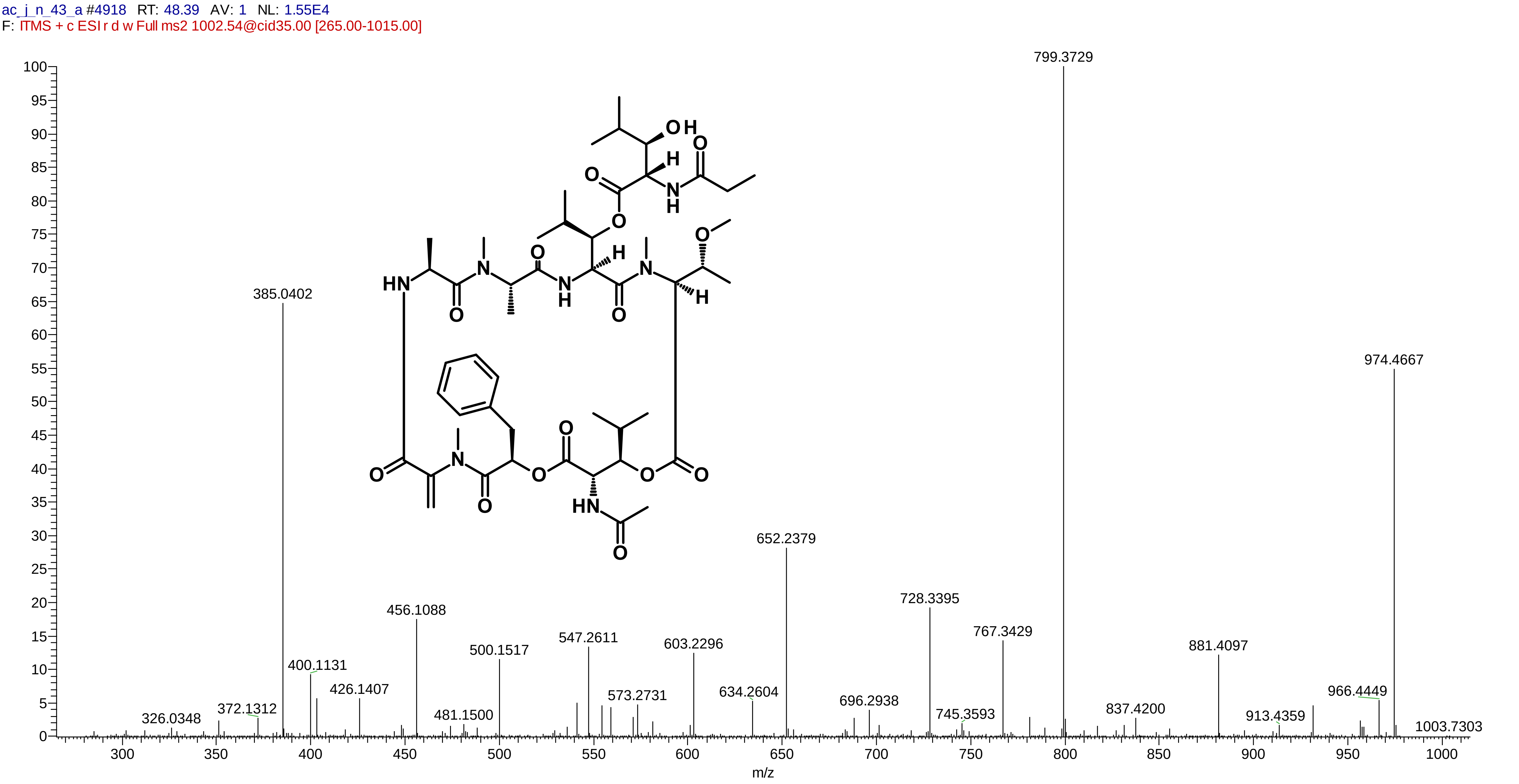

FR900359 RT:48.38 min *m/z*:1002.5394

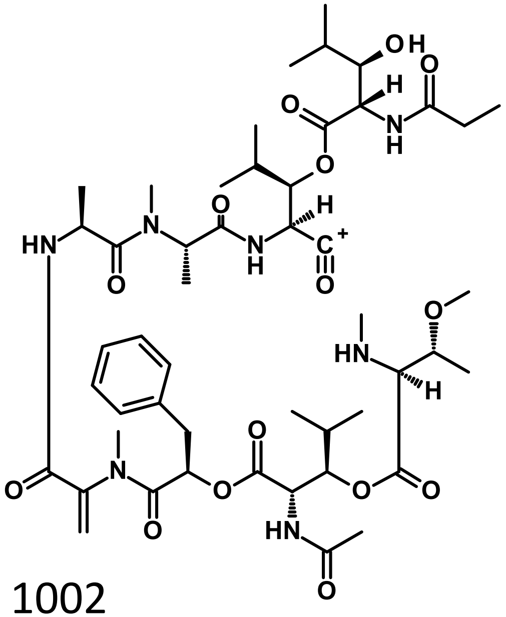

1002

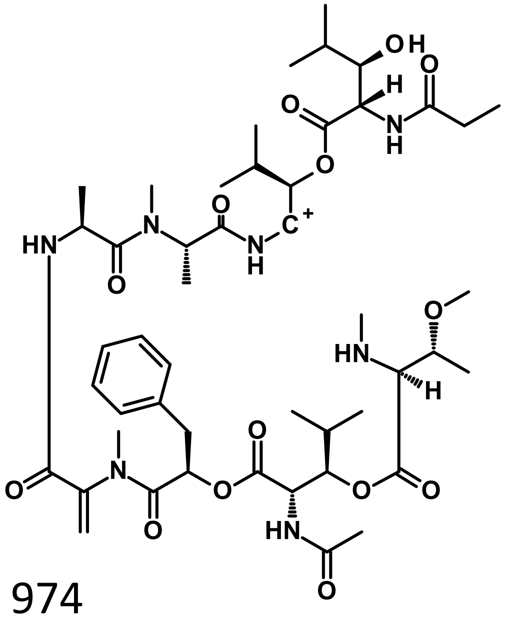

974

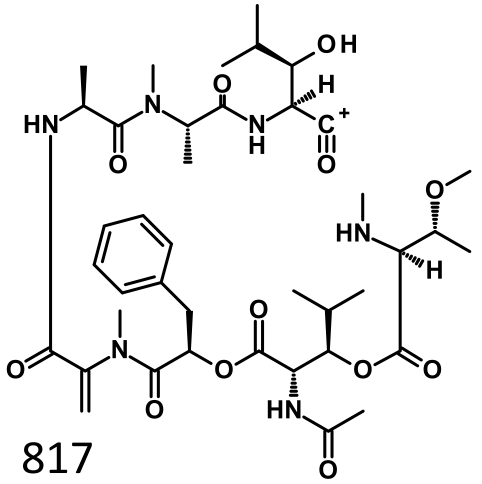

817

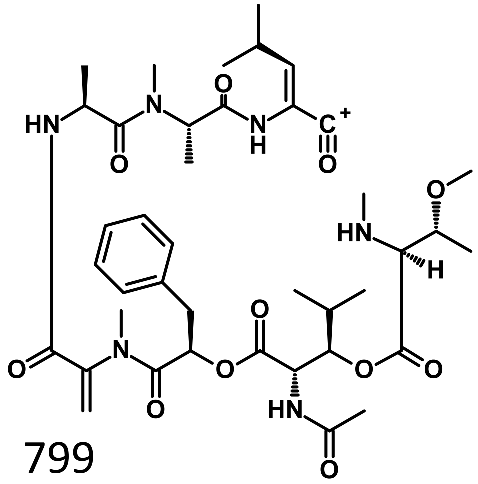

799

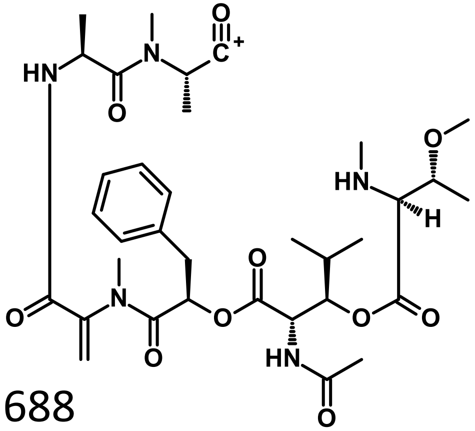

688

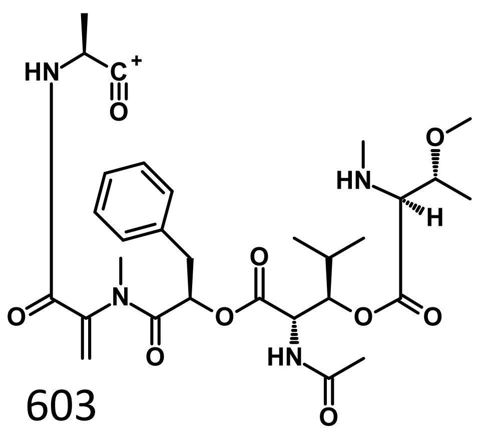

603

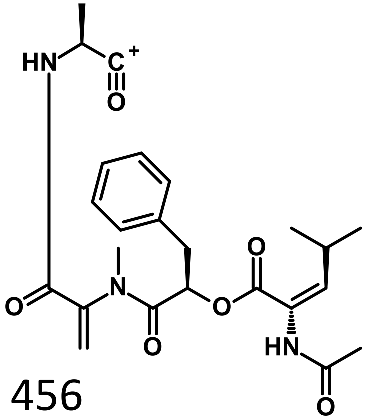

456

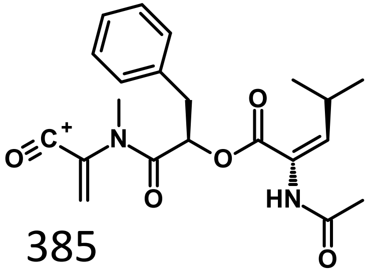

385

AC-1 RT:44.25 min *m/z*:1032.5500

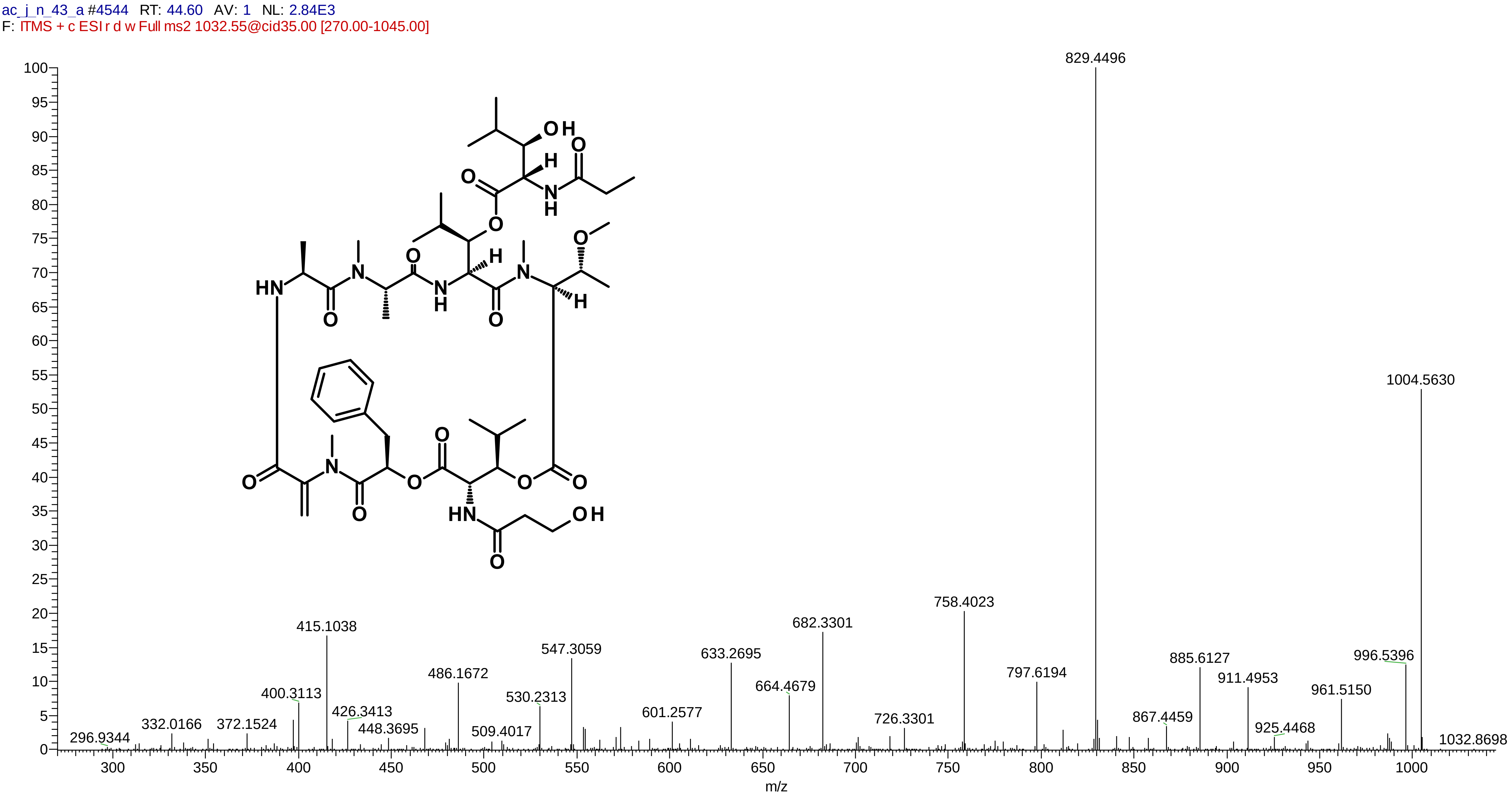

AC-1 RT:44.25 min *m/z*:1032.5500

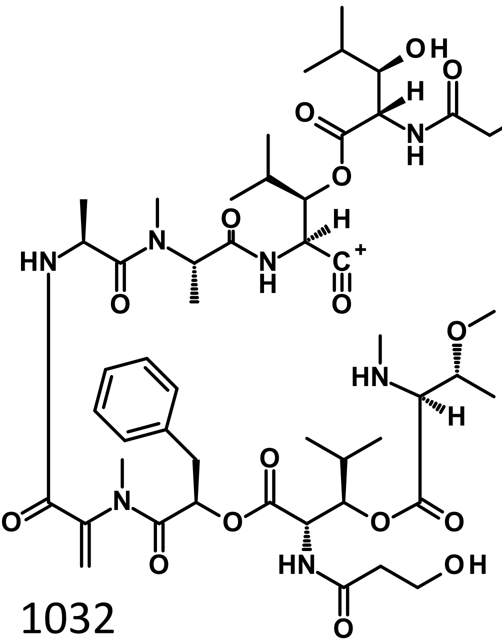

1032

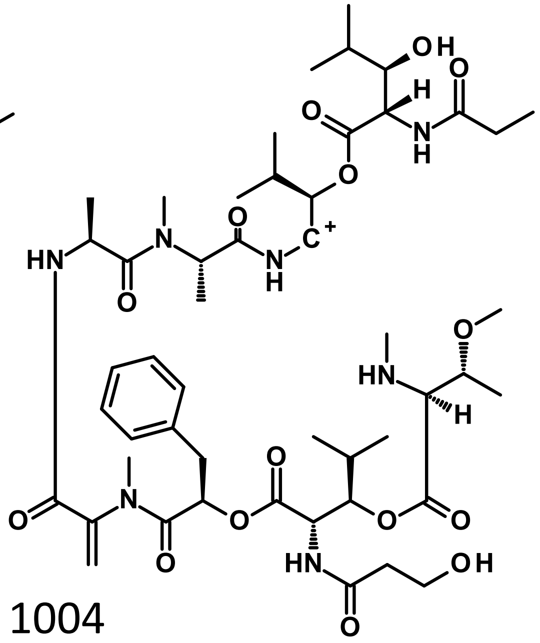

1004

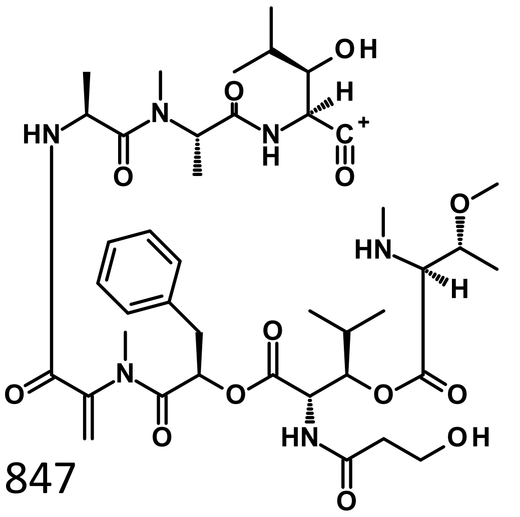

847

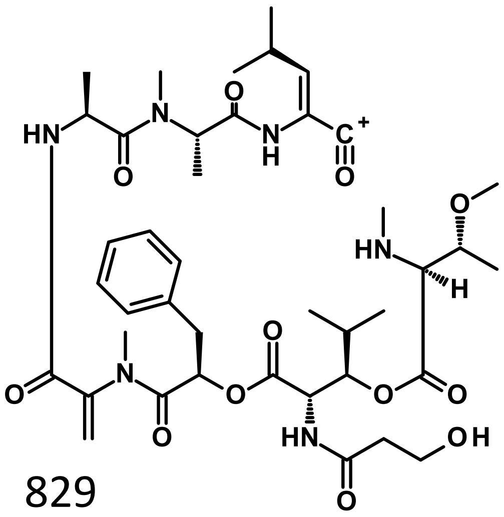

829

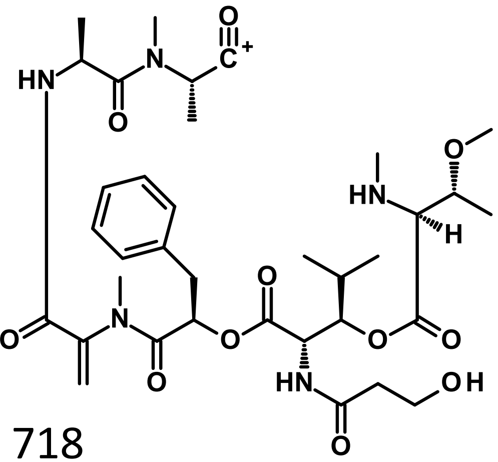

718

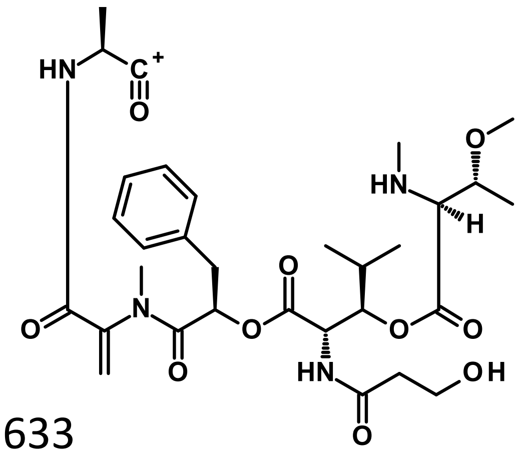

633

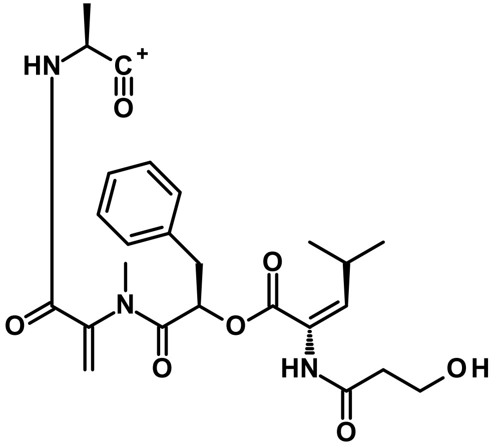

486

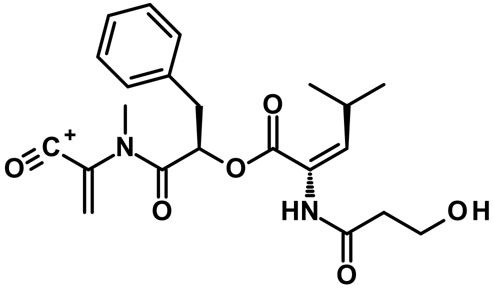

415

AC-SC RT:39.74 min *m/z*:817.4342

ac_s_n_62_a #4133 RT: 39.70 AV: 1 NL: 3.28E4
F: ITMS + c ESI r d w Full ms2 817.52@cid35.00 [215.00-830.00]

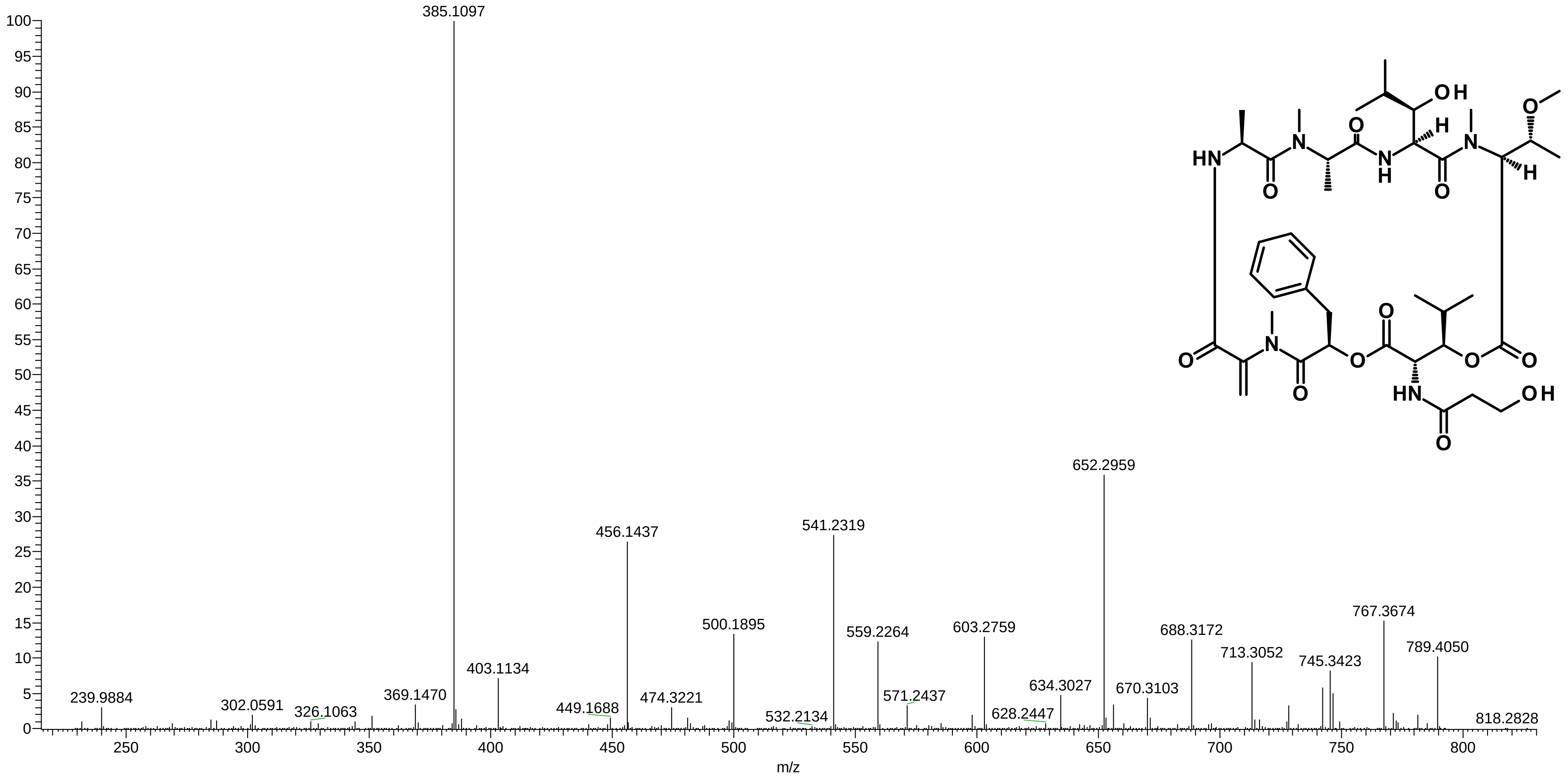

AC-SC RT:39.74 min *m/z*:817.4342

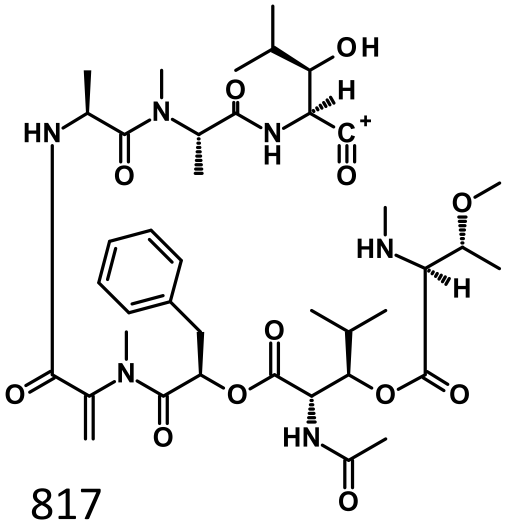

817

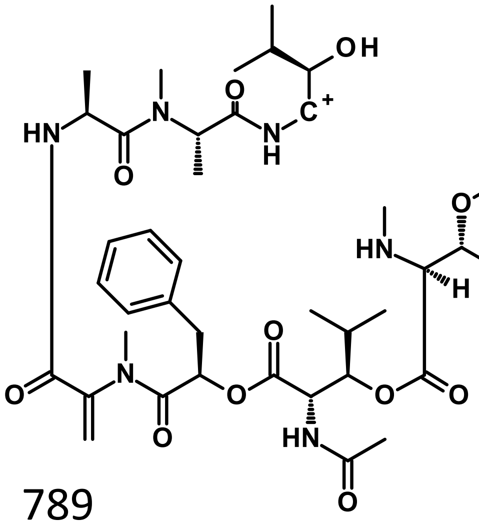

789

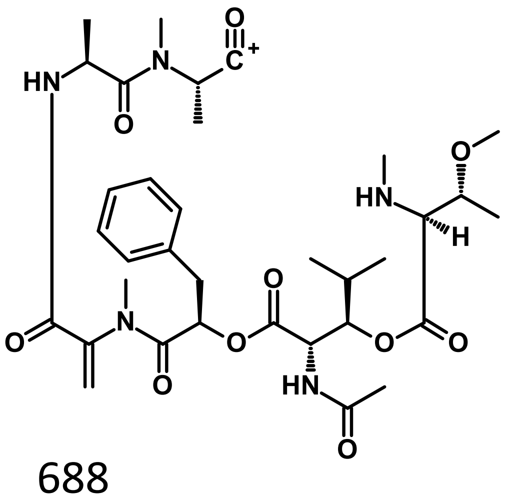

688

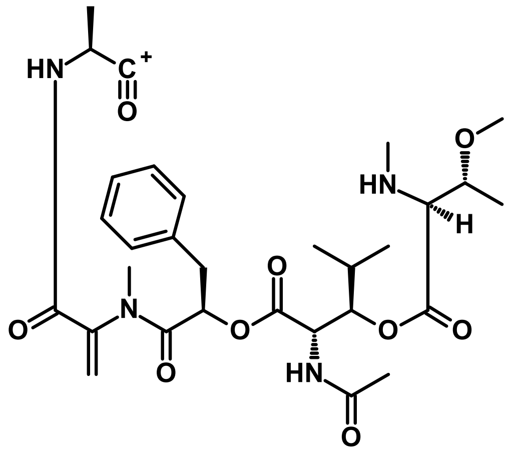

603

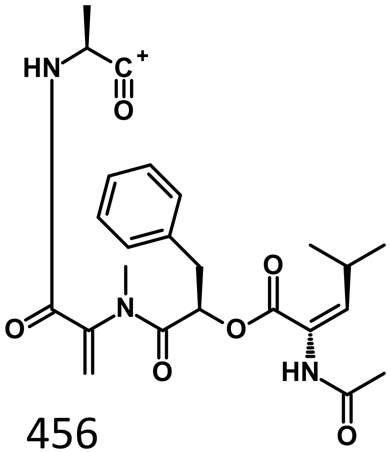

456

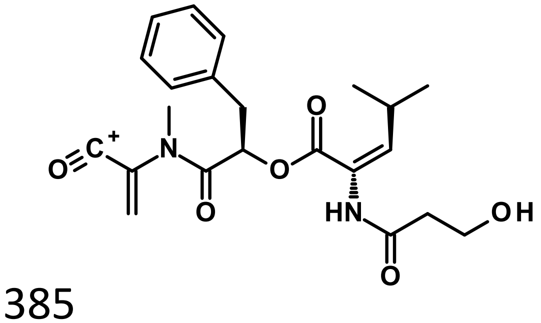

385

Putative cyclic-depsipeptide RT:31.76 min *m/z*:1123.5748

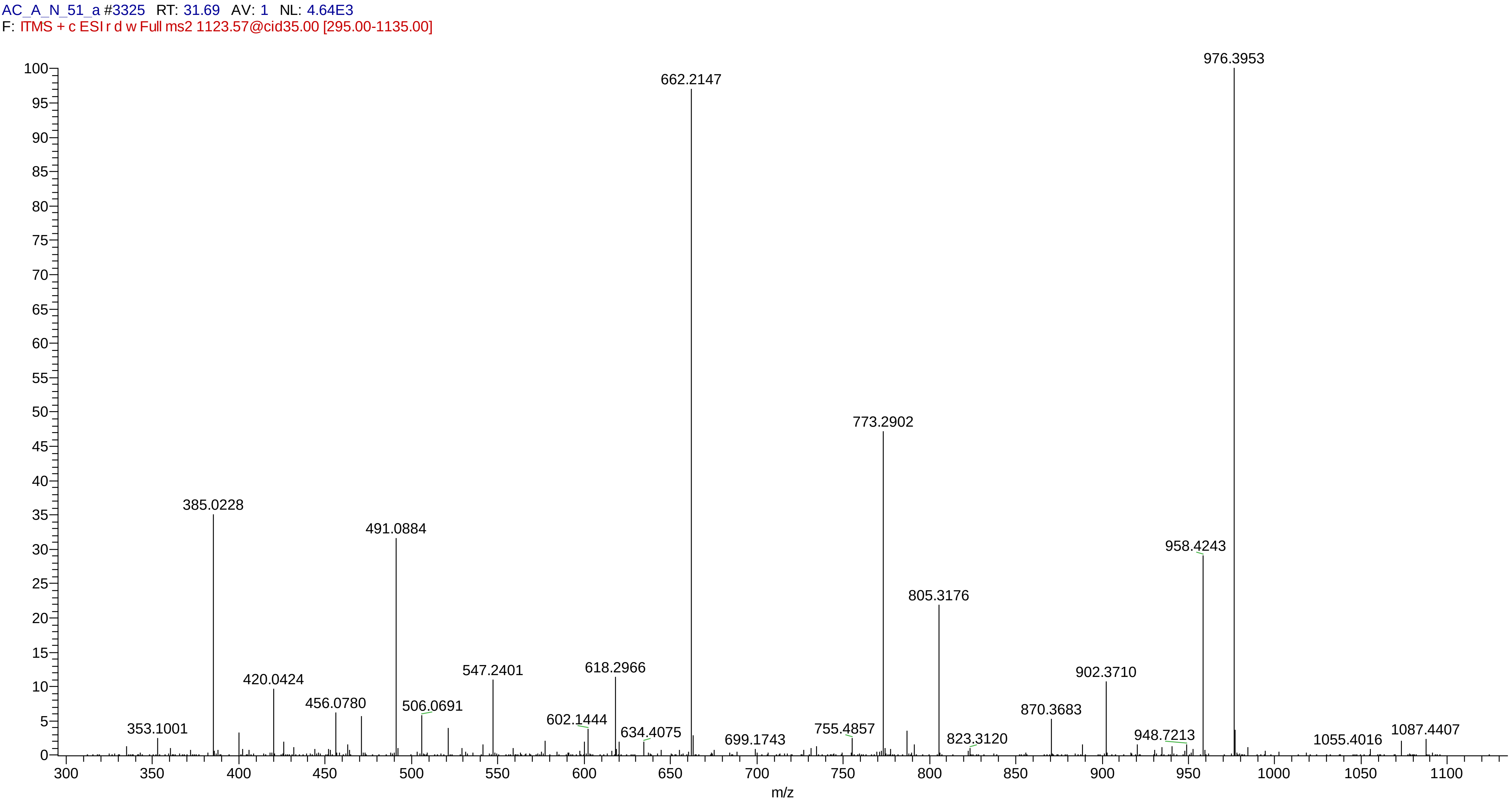

Putative cyclic-depsipeptide RT:31.76 min *m/z*:1123.5748

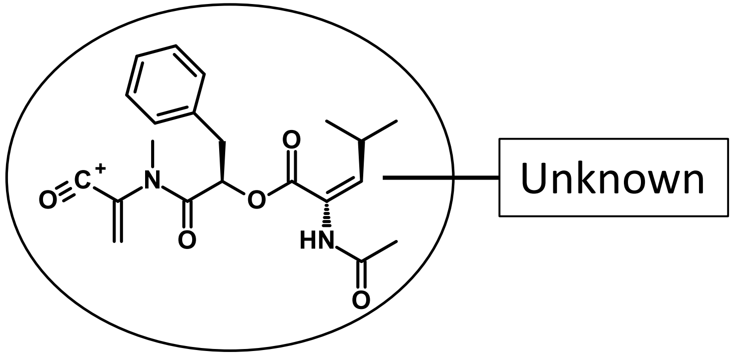

385+121=506

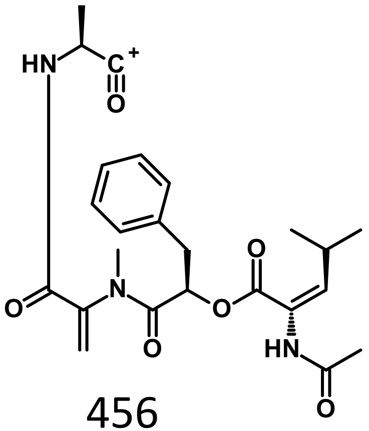

456

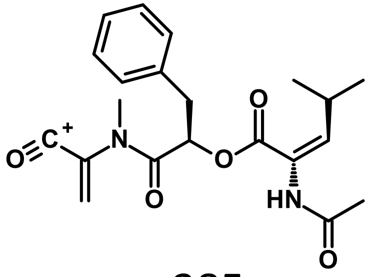

385

Putative cyclic-depsipeptide RT:35.12 min *m/z*:1123.5715

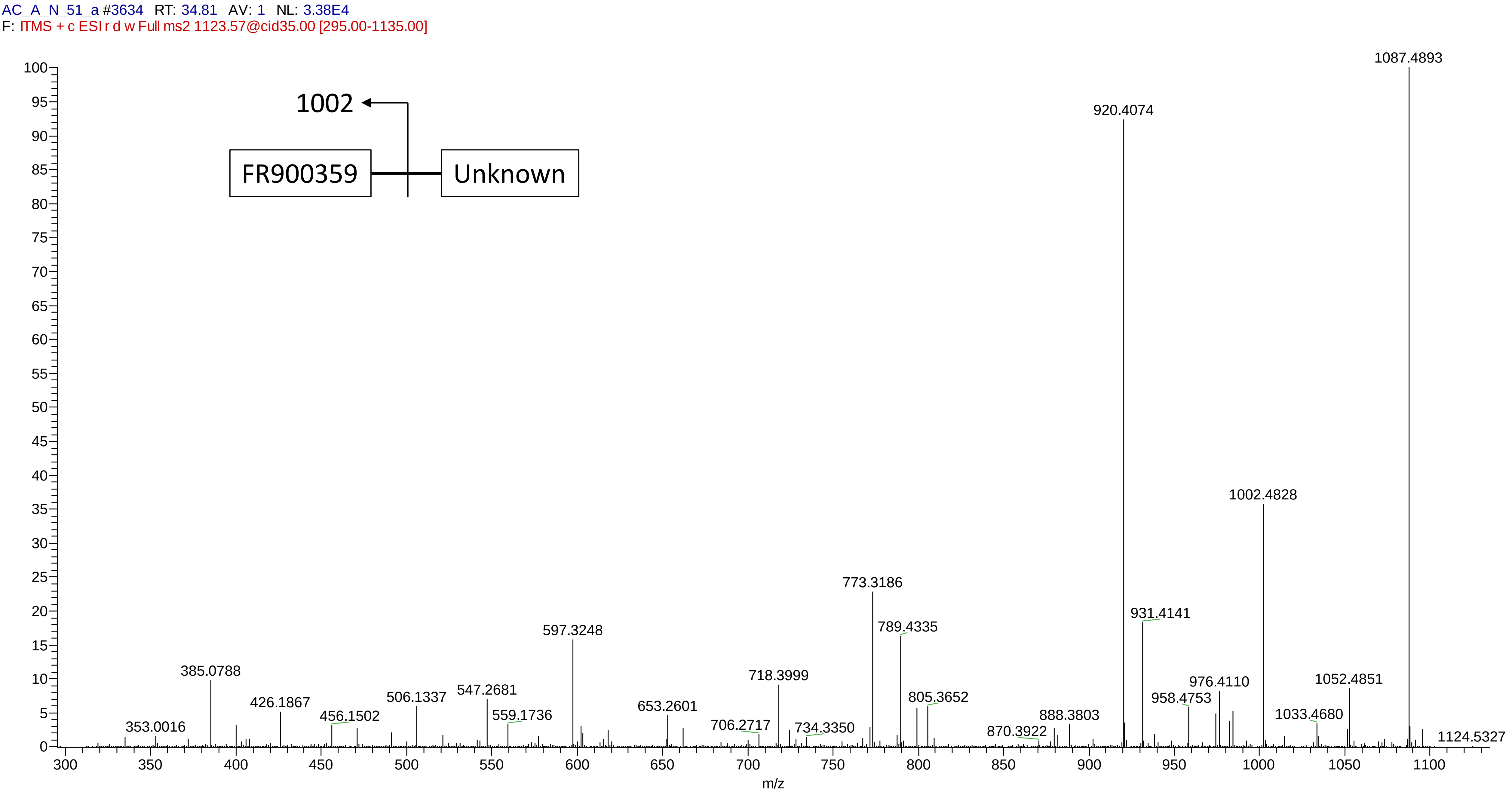

Putative cyclic-depsipeptide RT:35.12 min *m/z*:1123.5715

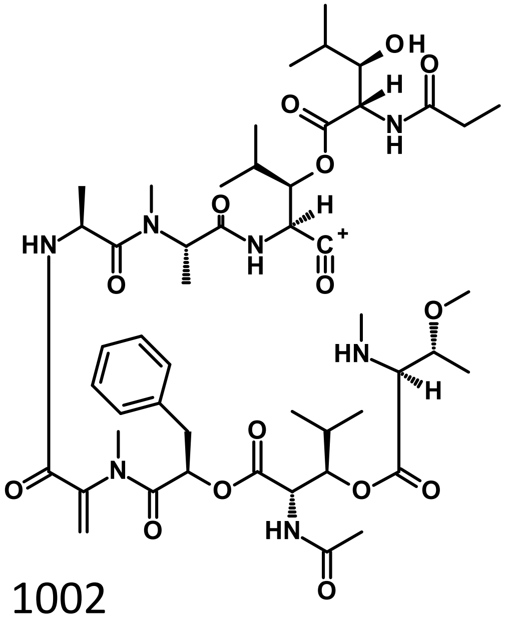

1002

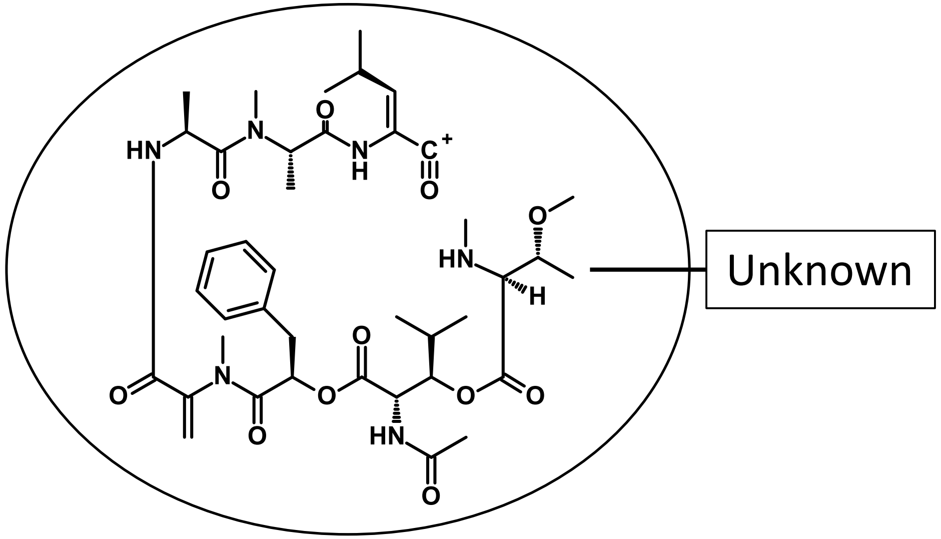

799+121=920

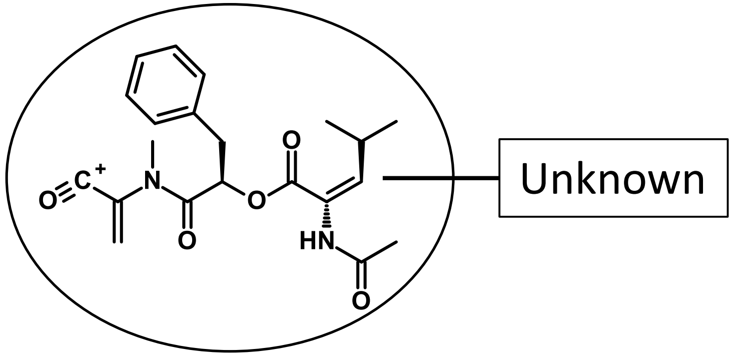

385+121=506

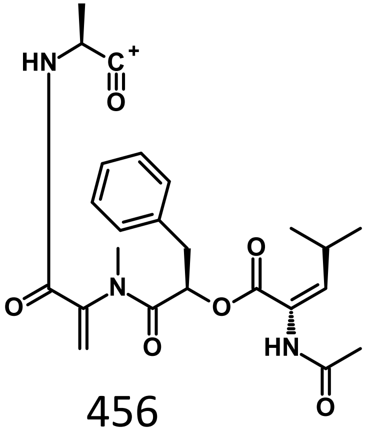

456

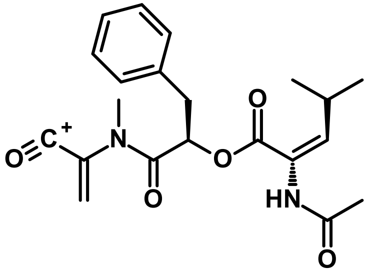

385

Putative cyclic-depsipeptide RT:35.12 min *m/z*:1123.5715 MS3

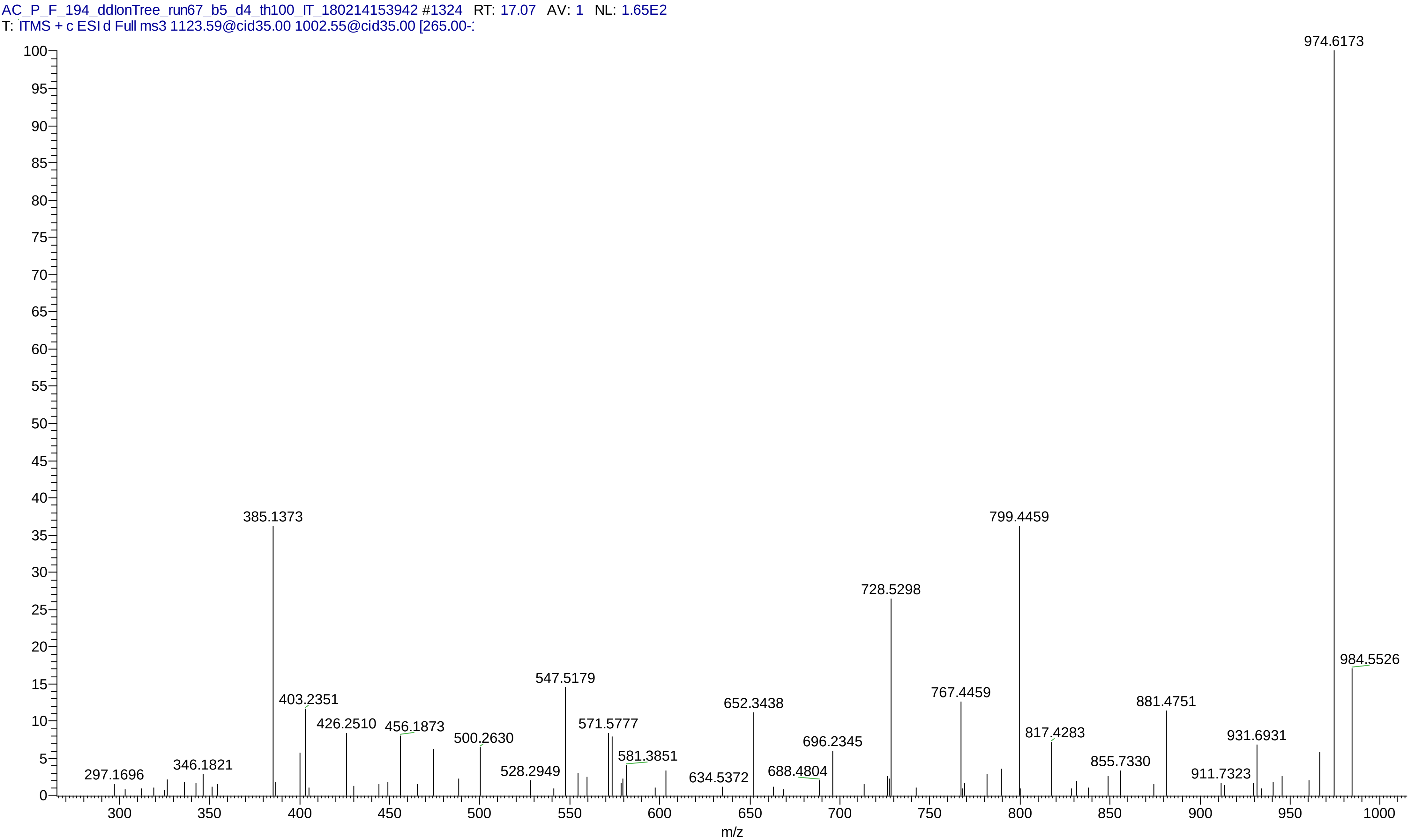

FR900359 RT:48.38 min *m/z*:1002.5394

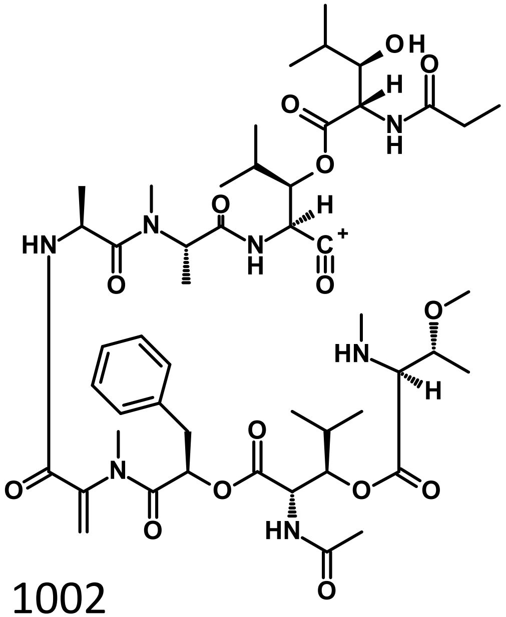

1002

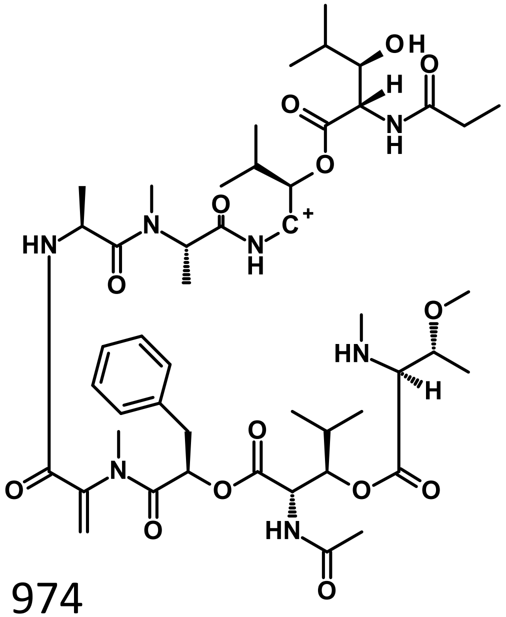

974

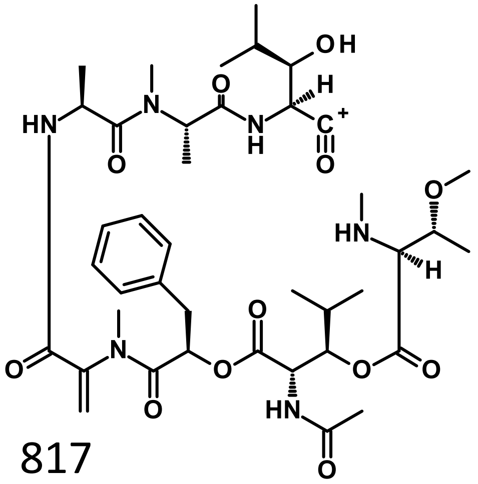

817

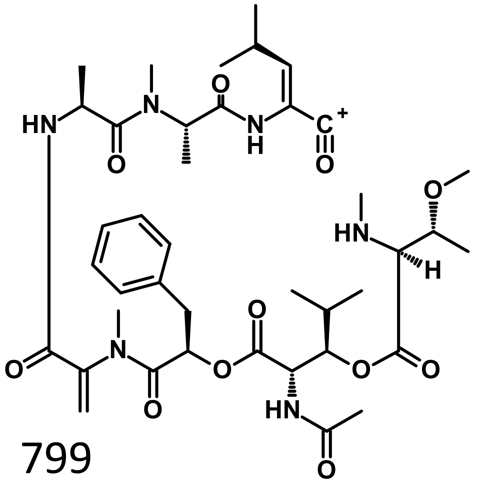

799

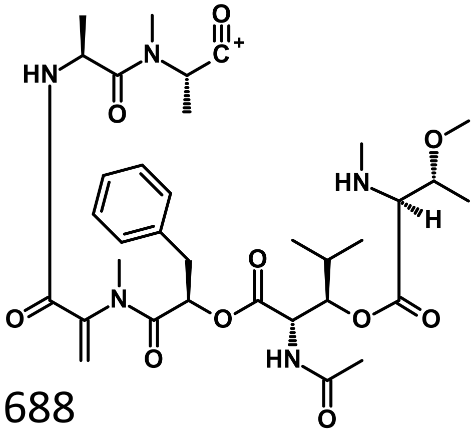

688

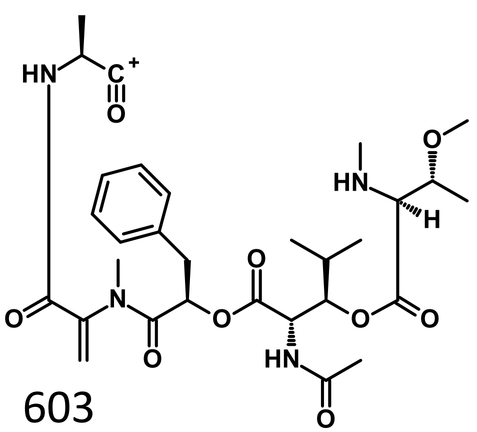

603

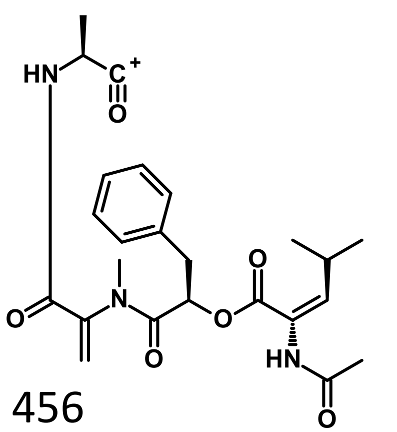

456

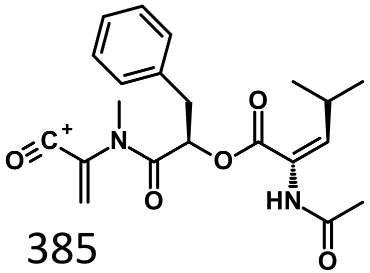

385

Putative cyclic-depsipeptide RT:37.36 min *m/z*:1123.5723

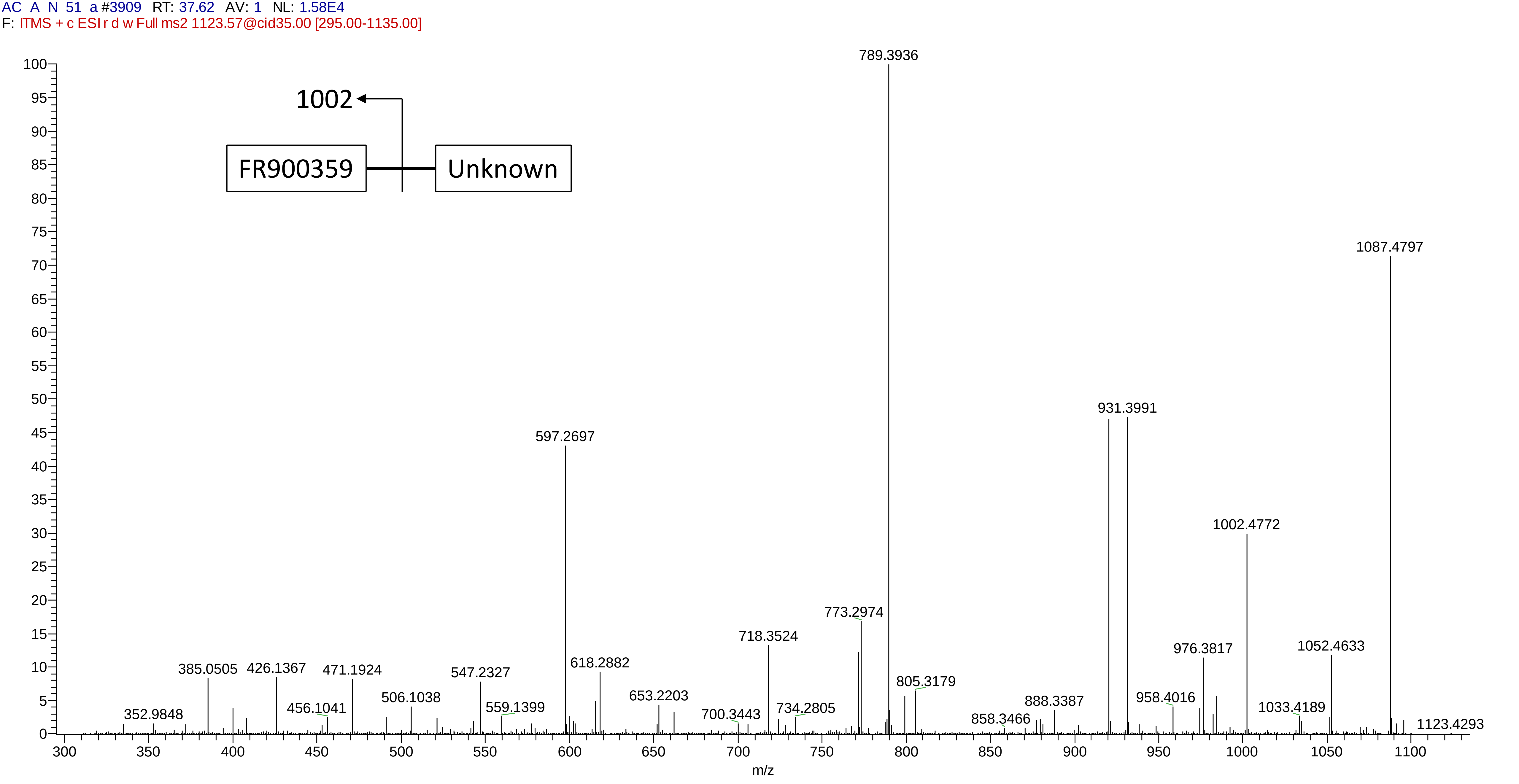

Putative cyclic-depsipeptide RT:37.36 min *m/z*:1123.5723

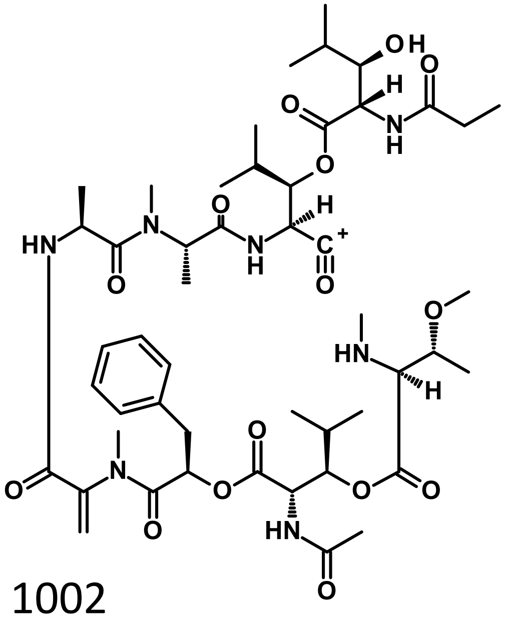

1002

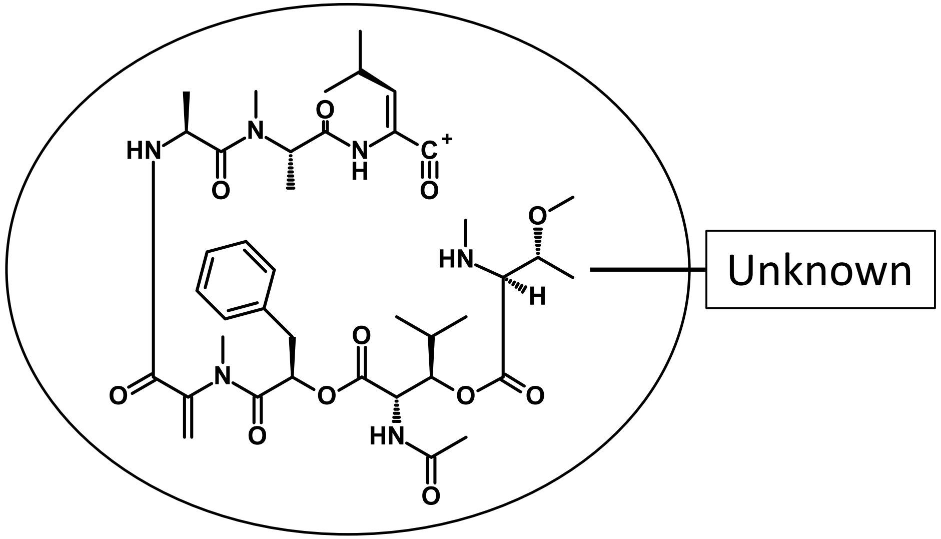

799+121=920

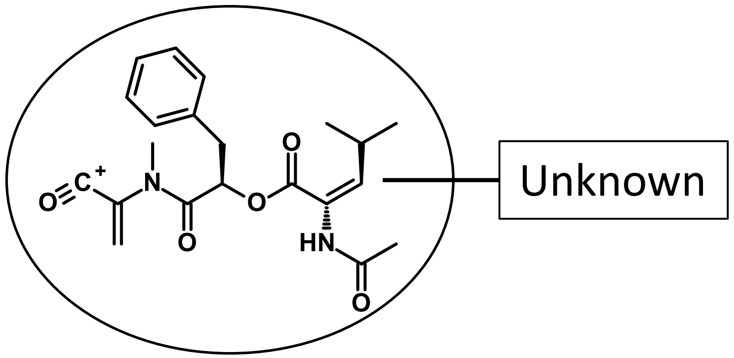

385+121=506

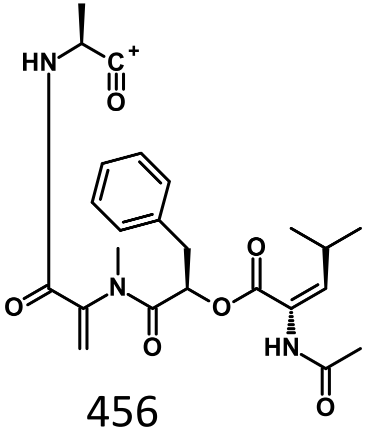

456

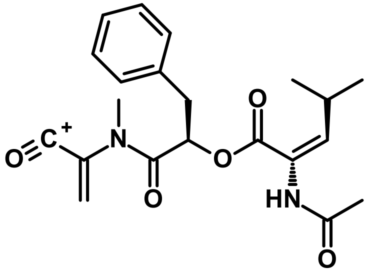

385

Putative cyclic-depsipeptide RT:42.33 min *m/z*:988.5237

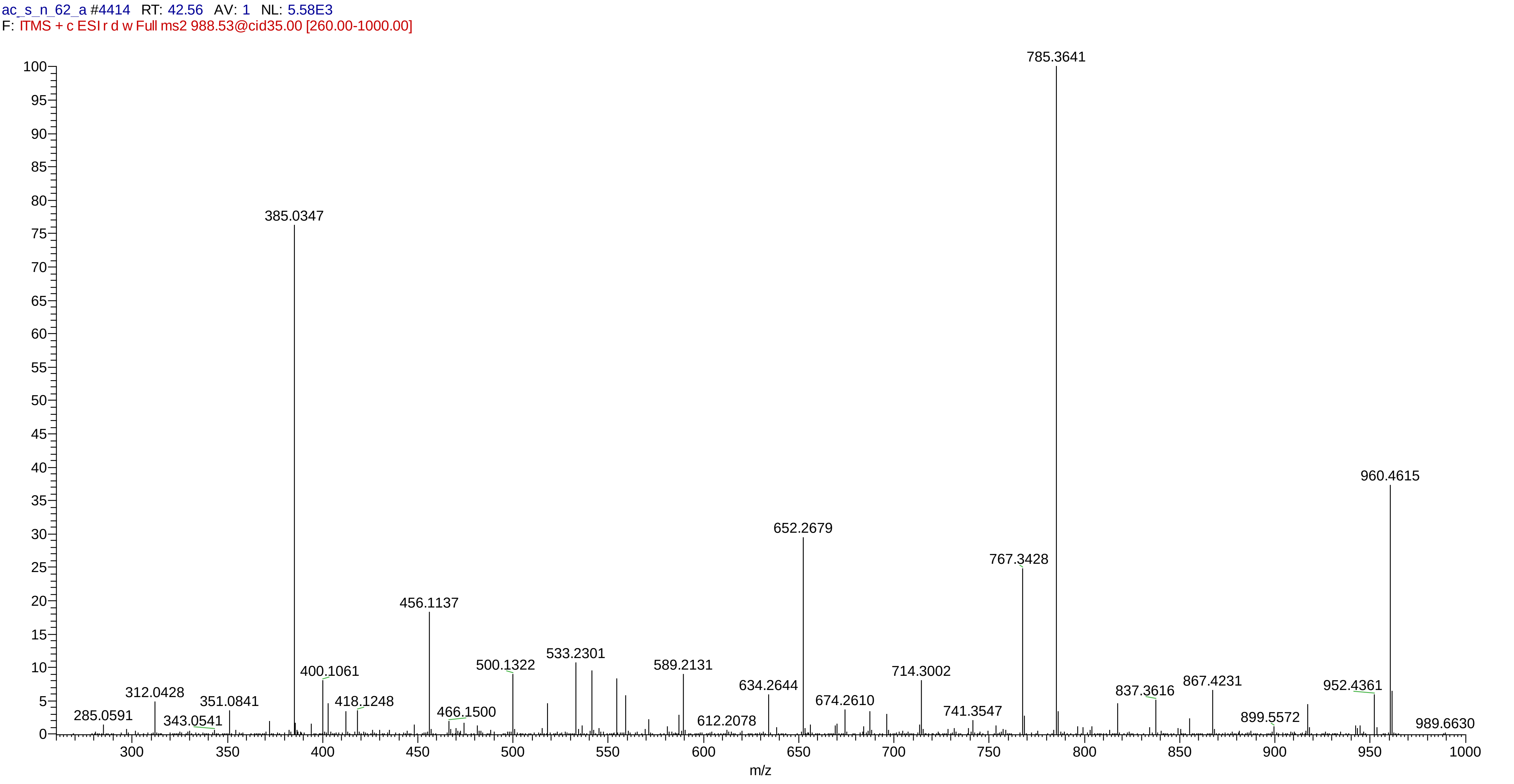

Putative cyclic-depsipeptide RT:42.33 min *m/z*:988.5237

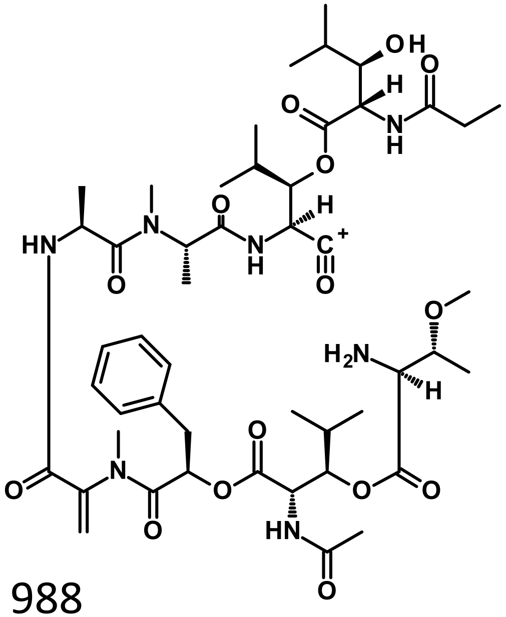

988

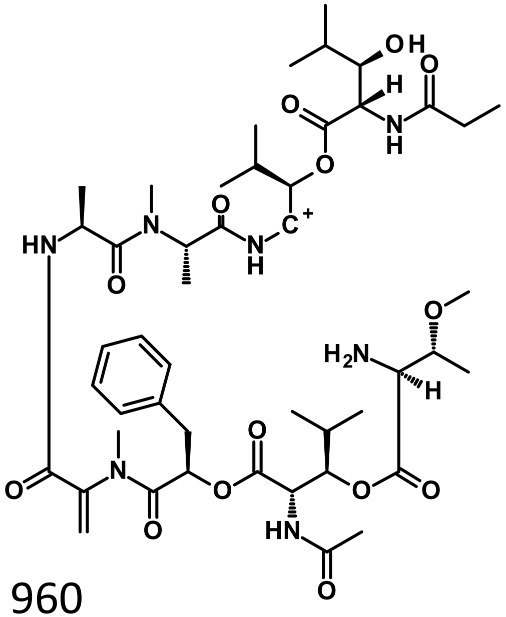

960

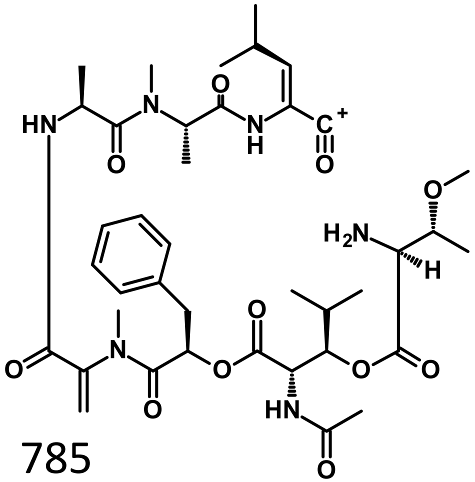

785

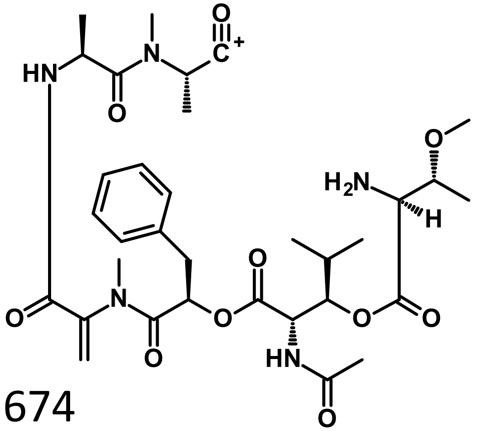

674

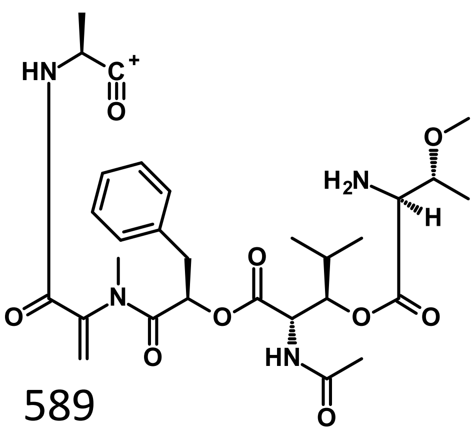

589

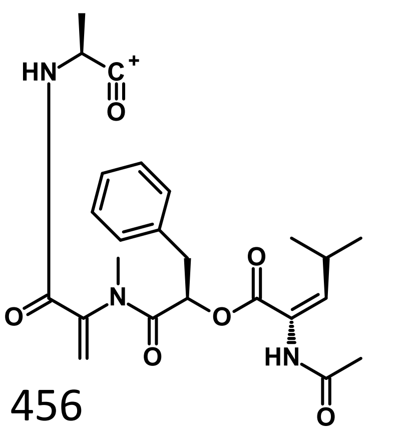

456

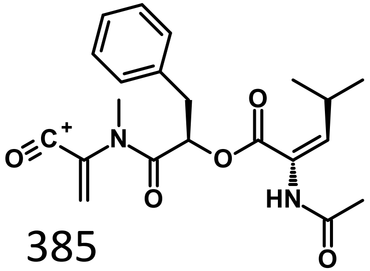

385

Putative cyclic-depsipeptide RT:45.46 min *m/z*:988.5237

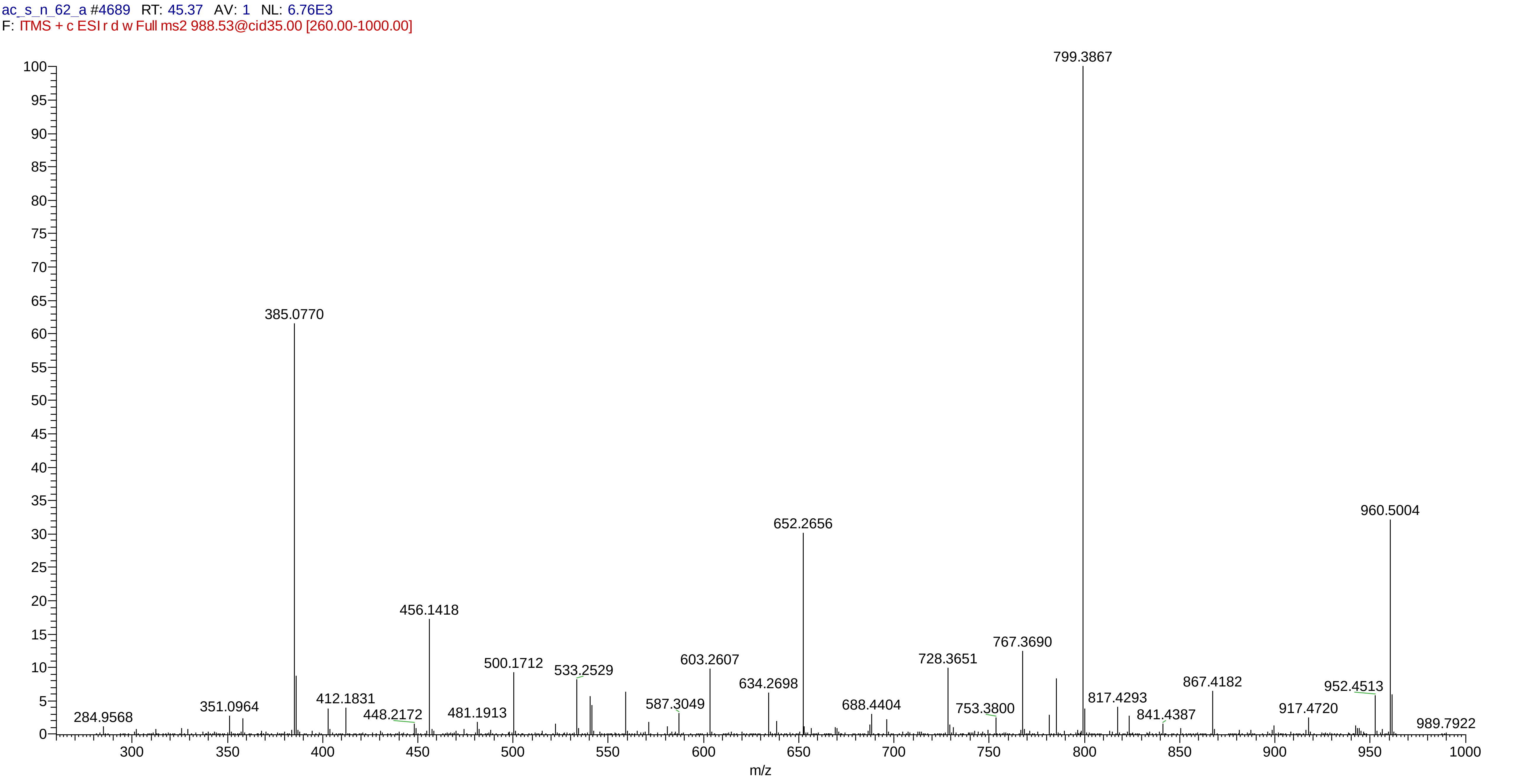

Putative cyclic-depsipeptide RT:45.46 min *m/z*:988.5237

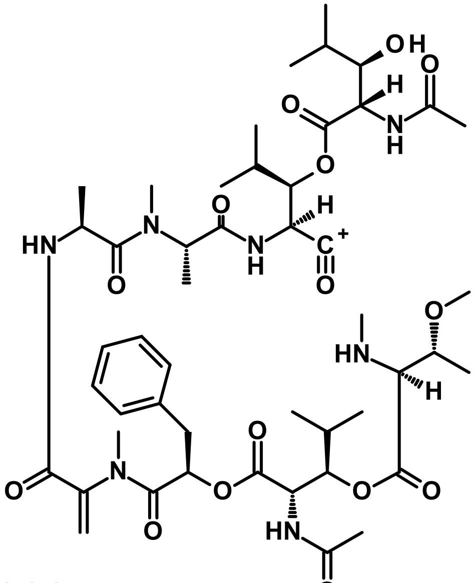

988

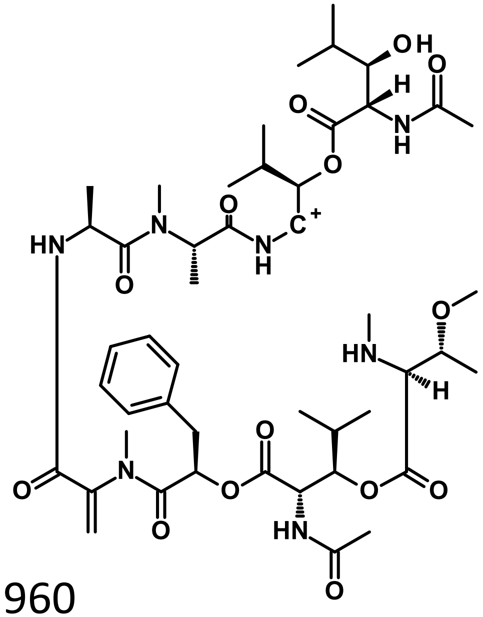

960

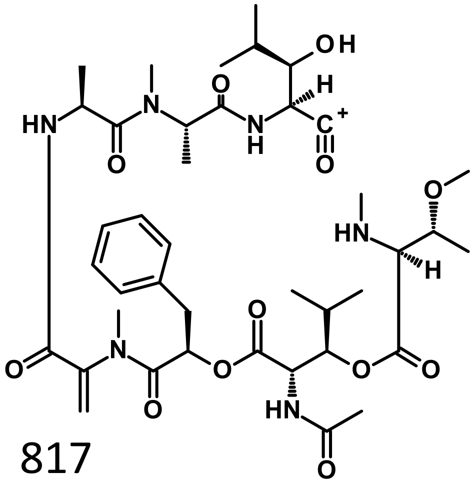

817

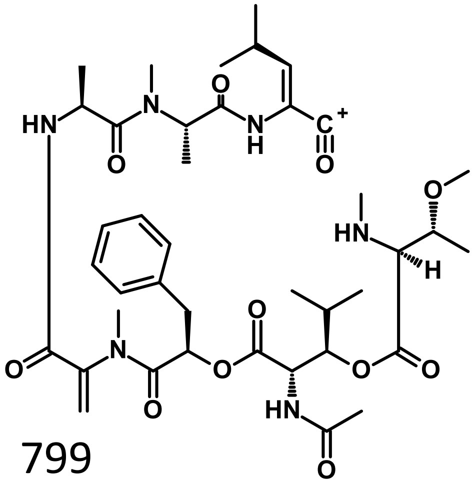

799

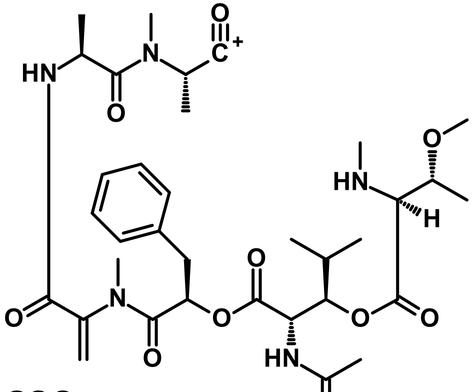

688

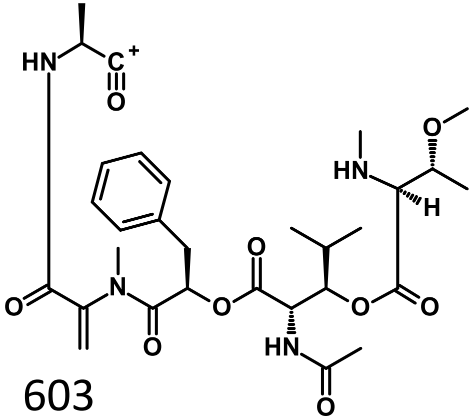

603

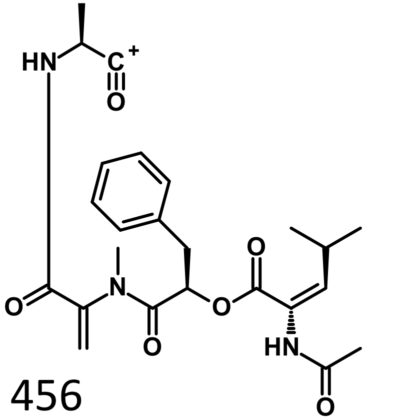

456

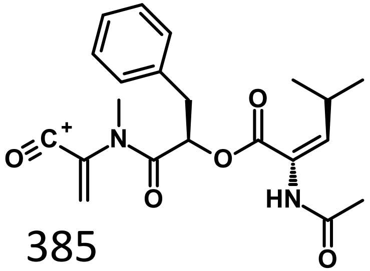

385
