# Supplementary material for: Dissecting Metabolism of Leaf Nodules in Ardisia crenata and Psychotria punctata
Source: Front Mol Biosci. 2021 Jul 30;8:683671. doi: 10.3389/fmolb.2021.683671 (PMC8362603; doi:10.3389/fmolb.2021.683671)
Supplement: Supplementary file 9 [file Table4.docx]

NMR:

Epicatechin 3'*O*-3-hydroxy-2-methyl-propanoate (**1**): white solid: **^1^H-NMR (600.13 MHz, CD_3_OD)** δ = 6.96 (s *apparent pattern*, 1H, H-2''), 6.79 (m, 1H, H-6''), 6.78 (m, 1H, H-5''), 5.97 (d, *J* = 1.6 Hz, 1H, H-6'), 5.95 (d, *J* = 1.6 Hz, 1H, H-8'), 5.42 (t *apparent pattern*, *J* = 1.6 Hz, 1H, H-3'), 5.01 (s *apparent pattern*, 1H, H-2'), 3.60 (dd, *J* = 10.8, 7.5 Hz, 1H, H-3a), 3.43 (dd, *J* = 10.8, 6.0 Hz, 1H, H-3b), 2.97 (dd, *J* = 17.4, 4.6 Hz, 1H, H-4'a), 2.84 (d *apparent pattern*, *J* = 17.4 Hz, 1H, H-4'b), 2.53 (tq, *J* = 7.4, 6.8 Hz, 1H, H-2), 0.94 (d, *J* = 7.1 Hz, 3H, H-4); **^13^C-NMR (150.61 MHz, CD_3_OD)** δ = 176.1 (C-1), 157.9 (C-5'), 157.9 (C-7'), 157.1 (C-9'), 146.0 (C-3''), 146.0 (C-4''), 131.3 (C-1''), 119.0 (C-6''), 115.9 (C-2''), 115.0 (C-5''), 99.2 (C-10), 96.5 (C-6'), 95.7 (C-8'), 78.2 (C-2'), 69.9 (C-3'), 65.0 (C-3), 43.6 (C-2), 26.7 (C-4'), 13.9 (C-4); **HRMS (ESI)** calculated for C_19_H_21_O_8_^+^: [M+H]^+^ 377.1231; found [M+H ]^+^ 377.1211.

Catechin (**2**): white solid: **^1^H-NMR (600.13 MHz, CD_3_OD)** δ = 6.87 (d, *J* = 1.8 Hz, 1H, H-2'), 6.76 (m, 1H, H-5'), 6.72 (m, 1H, H-6'), 5.96 (d, *J* = 2.3 Hz, 1H, H-6), 5.88 (d, *J* = 2.3 Hz, 1H, H-8), 4.59 (d, *J* = 7.6 Hz, 1H, H-2), 4.00 (ddd, *J* = 13.2, 7.9, 5.4 Hz, 1H, H-3), 2.84 (m, 1H, H-4a), 2.53 (dd, *J* = 16.1, 8.1 Hz, 1H, H-4b); **^13^C-NMR (150.61 MHz, CD_3_OD)** δ = 157.9 (C-7), 157.6 (C-5), 156.9 (C-9), 146.3 (C-4'), 146.2 (C-3'), 132.2 (C-1'), 120.0 (C-6'), 116.1 (C-5'), 115.2 (C-2'), 100.8 (C-10), 96.3 (C-6), 95.6 (C-8), 82.9 (C-2), 68.8 (C-3), 28.5 (C-4); **HRMS (ESI)** calculated for C_15_H_15_O_6_^+^: [M+H]^+^ 291.0863; found [M+H]^+^ 291.0856.

Epicatechin (**3**): white solid: **^1^H-NMR (600.13 MHz, CD_3_OD)** δ = 7.00 (d, *J* = 1.9 Hz, 1H, H-2'), 6.79 (m, 1H, H_6'), 6.76 (m, 1H, H-5'), 5.97 (d, *J* = 2.4 Hz, 1H, H-6), 5.95 (d, *J* = 2.4 Hz, 1H, H-8), 4.85 (s *apparent pattern*, 1H, H-2), 4.21 (m, 1H, H-3), 2.86 (m, 1H, H-4a), 2.77 (dd, *J* = 16.7, 2.8 Hz, 1H, H-4b); **^13^C-NMR (150.61 MHz, CD_3_OD)** δ = 158.0 (C-5), 157.7 (C-7), 157.3 (C-9), 146.0 (C-3'), 145.8 (C-4'), 132.3 (C-1'), 119.4 (C-6'), 115.9 (C-5'), 115.3 (C-2'), 100.1 (C-10), 96.4 (C-6), 95.9 (C-8), 79.9 (C-2), 67.5 (C-3), 29.3 (C-4); **HRMS (ESI)** calculated for C_15_H_15_O_6_^+^: [M+H]^+^ 291.0863; found [M+H]^+^ 291.0858.
